# Predicting anti-RhD titers in donors: Boostering response and decline rates are personal

**DOI:** 10.1371/journal.pone.0196382

**Published:** 2018-04-26

**Authors:** Anneke S. de Vos, Ellen C. E. van der Schoot, Dimitris Rizopoulos, Mart P. Janssen

**Affiliations:** 1 Transfusion Technology Assessment Department, Sanquin Research, Amsterdam, the Netherlands; 2 Department of Experimental Immunohematology, Sanquin Research, Amsterdam, the Netherlands; 3 Department of Biostatistics, Erasmus University Medical Center, Rotterdam, the Netherlands; Australian Red Cross Blood Service, AUSTRALIA

## Abstract

**Background:**

Anti-RhD immunised donors provide anti-RhD immunoglobulins used for the prevention of rhesus disease. These donors are periodically hyper-immunised (boostered) to retain a high titer level of anti-RhD.

**Study design and methods:**

We analysed anti-RhD donor records from 1998 to 2016, consisting of 30,116 anti-RhD titers from 755 donors, encompassing 3,372 booster events. Various models were fit to these data to allow describing the anti-RhD titers over time.

**Results:**

A random effects model with a log-linear anti-RhD titer decline over time and a saturating titer response to boostering is shown to fit the data well. This model contains two general model parameters, relating timing and maximum of the booster effect, as well as two parameters characterizing the individual donor, namely how fast the booster effect saturates with current titer and the anti-RhD decline rate. The average individual log_2_ decline is 0.55 per year, i.e. a 32% decline in absolute titer, with half of the donors declining between 13% and 41% per year. Their anti-RhD titer peaks around 26 days following a booster event. Boostering response reduces with higher titers at boostering; at median titer (log_2_ 11) the mean increase per booster is log_2_ 0.38, that is from an absolute titer of 2048 to 2665 (+30%), with half of all donors increasing between 16% and 65% in their titer.

**Conclusion:**

The model describes anti-RhD titer change per individual with only four parameters, two of which are donor specific. This information can be used to enhance the blood bank’s immunisation programme, by deriving individualized immunization policies in which boostering is adjusted to the anticipated anti-RhD decline, effectiveness of boostering and titer levels required.

## Introduction

Anti-RhD immunised donors provide anti-RhD immunoglobulins, which is the main constituent in the prophylaxis used for the prevention of foetal rhesus disease [1]. During pregnancy or birth of an RhD-positive child, RhD-negative women may become immunized against RhD. Their antibodies can cross the placenta, destroying the red blood cells of a (subsequent) RhD-positive foetus, the cause of foetal rhesus disease. Timely administration of anti-RhD immunoprophylaxis prevents such immunisation and thereby this disease [2].

Administration of anti-RhD immunoprophylaxis has been applied since 1969 in the Netherlands. With added antenatal administration improving performance further since 1998, this practice has lowered the number of intrauterine foetal deaths by a hundredfold [1].

The success of this practice however presents a difficulty to its own continuation: natural immunisation has become rare, which has led to a decline in the availability of anti-RhD donors [3]. To uphold the donor population in the Netherlands, woman over reproductive age and man as well have been purposely immunized. As anti-RhD titers normally decline over time without exposure, donors are also periodically hyper-immunised (boostered) in order to stimulate continued production of anti-RhD immunoglobulins. Comparable to vaccination however, such booster events are a notable burden on donors.

In this manuscript we describe an analysis of historical data of anti-RhD donors in the Netherlands. Our aim was to quantify the effect of booster events, as dependent on known variables. A model which allows prediction of the anti-RhD titer response in donors can be used to rationalize and optimize boostering strategies for donors, minimizing the burden on donors whilst maintaining anti-RhD production levels required.

## Materials and methods

### Description of the data-set

We analysed anti-RhD donor records of the Dutch blood-bank Sanquin from 1998 to 2016 [3]. Data on anti-RhD titers from donations were available from September 1994 onwards. However, we noted a large number of substantial anti-RhD titer increases unexplained by records of preceding booster events. As such observations indicating lower data validity occur almost exclusively in the records before 1998, these earlier data were omitted from our analyses.

Donors are allowed to donate every two weeks, and their anti-RhD titer is measured at each donation on discrete log_2_ scale. Policy is for new donors to be boostered at their first three to five donations to bring their anti-RhD titer up to a high level, and subsequently for boosters to be administered when anti-RhD titer lowers. In total the analysed data-set comprises 30,116 anti-RhD titers from 755 repeat donors, encompassing 3,372 booster events (mean records per donor 39.9, median 20 and maximum 277, mean booster events per donor 4.5, median 4 and maximum 22). For 90% of only the records from 2005–2016 the precise donation date is known. When the donation date was not recorded, as a proxy the date logged for the availability of the anti-RhD test results was used. On average test results were available 2.6 days after a donation.

All donors included in our study have provided consent for their donations and anonymized data to be used for scientific research. A dataset with titers and boostering events is provided as supplementary material to this paper (S1 Dataset). To protect the privacy of the donors, all information related to the timing of donations, as well as donor age and gender were removed from the original dataset. Requests to use the original dataset for further research can be directed to the corresponding author and/or the research director of the Sanquin Blood Supply Foundation. It is at the discretion of Sanquin to grant such requests.

### Analyses

Due to the complex nature of the data, which includes multiple booster responses as well as natural titer change over time, we have performed our analysis in several steps, where the final step (Step 3) fits a model on the full data-set. In smaller initial steps we first examined how anti-RhD titer changes in the absence of boostering, Step 1, and next the effect of a single booster event, Step 2. While in Step 2A we focus on the timing of the boostering effect, in Step 2B we focus on the magnitude of the induced anti-RhD titer increase. For Steps 1 and 2A we used only the data with exact known donation dates, while for Steps 2B and 3 we used the full data-set. The steps are explained in detail below. All analyses were performed in the *R* language and environment for statistical computing (version 3.1.2) [4].

#### Step 1: Natural anti-RhD decline

We first fit anti-RhD titer change over time in the absence of boostering. To eliminate the impact of boostering we considered only donations 100 days or more since any last booster event. Note that the 100 day period was derived from the results obtained from Step 2A. Each first compliant titer measurement was set as time 0, i.e. follow-up time restarted with an intervening booster event for a donor. The dependent model variable was the change in anti-RhD titer from measurement at time 0, the explanatory variable time. This data-subset comprised of 9,687 anti-RhD titer differences from 323 individuals. Using maximum likelihood assessment, the lowest BIC score was obtained for a model with random effects and log-linear decline over time; i.e. *T*_*i*_(*t*) − *T*_*i*_(*t* = 0) = *D*_*i*_*t* + ∈. Here *T*_*i*_(*t*) represents the log_2_ titer measurement at time *t* of donor *i*. By $D_{i}∼Normal(\mu_{D}=D,\sigma_{D}^{2})$ the natural log-linear rate of change for donor *i* is given, and the measuring error is $\in ∼Normal(\mu_{\in }=0,\;\sigma_{\in }^{2})$.

#### Step 2A: Timing of the boostering effect

Next we examined the effect of boostering, first focussing on the time envelope of the boostering effect. The model was fit up to 200 days following boostering or until a next booster event. To isolate the individual boostering effect we also excluded boosters less than 100 days from a previous booster event. The remaining data-subset comprises of 3,137 changes in anti-RhD titer following 709 booster events of 250 donors. We fitted the boostering effect on top of the population average log-linear decline rate *D* established in Step 1. Using nonlinear least squares regression, the lowest normalised squared errors score was obtained with logistic saturating increase from the time of boostering: the fit formula was $T_{i}(t)-T_{i}(t=0)=2B\;\left(\frac{1}{1+e^{-Ht}}-\frac{1}{2}\right)+Dt$. In this formula *B* is the maximum increase in anti-RhD titer from boostering, and *H* scales the speed of the increase after boostering. The maximum anti-RhD titer difference was estimated at 26 days from boostering. This number was used in the next step of the analysis.

#### Step 2B: Magnitude of the boostering effect

The magnitude of the boostering effect was found to be strongly correlated with a donor’s anti-RhD titer at boostering. We fitted the change in log_2_ titer at its peak value with log_2_ starting titer as the explanatory variable. Peak value was defined as the measurement at the first time point per donor per booster event that was from 26 to 50 days subsequent to boostering. We excluded donations with a previous booster event occurring less than 50 days before the booster event of interest, but in this analysis did not exclude subsequent boosters; this would have resulted in too little observations especially in the low starting titer region, since new donors with low starting titer are boostered several times. In effect this analysis establishes a maximum in single boostering effect. The data-set for this analysis contained 1,992 titer changes after boostering of 550 donors. Using nonlinear least squares regression, a logistic decline in boostering effect was found to fit the data well, i.e. $T_{i}(t)-T_{i}(t=0)=2B\;\left(1-\frac{1}{1+e^{-LT_{i}(t=0)}}\right)+tD$, where *B* again represents the maximum boostering effect, and *L* scales the speed of the decline of the boostering effect.

#### Step 3: The integral model

Finally, putting together our findings from the previous steps, we fitted a non-linear random effects model to the full dataset using maximum-likelihood estimation. We assumed each individual booster event to have an independent effect on anti-RhD titer. The integral model for change *C* in log_2_ titer can then be formulated as:

$$C(t)=T_{i}(t)-T_{i}(t=0)=D_{i}t+\sum_{j=1}^{j=m_{i}}\left(x_{i,j}>t)4B\;(\frac{1}{1+e^{-H(t-x_{i,j})}}-\frac{1}{2})(1-\frac{1}{1+e^{-L_{i}T_{i}(t=x_{i,j})}}\right)+\in$$

Here *x*_*i*,*j*_ represents the *j*^th^ boostering of donor *i*, who received *m*_*i*_ boosters in total. We refitted the earlier individualised constant log-linear decline rate *D*_*i*_ in this random effects model. Since timing of the boostering effect *H* has very little impact on the integral model fit (resulting in flat likelihood surface), it was infeasible to re-fit this model parameter. The parameter value was therefore taken from results of Step 2A. The magnitude of the boostering effect *B* was re-fit, as was the logistic saturation in the effect *L*_*i*_, now as an individualised random effects parameter.

The model was run initially using the titers at the time of boostering *T*_*i*_(*t* = *x*_*i*,*j*_) as measured. To have the model fully predictive, starting titers at the time of boostering were derived from the model fit and the model parameters re-estimated. This process was iterated until the fitted parameters reached steady state values (in the first seven digits) after 30 loops. In this procedure titer estimates for *T*_*i*_(*t* = *x*_*i*,*j*_) below 0 were rounded up to 0. Starting parameter values for the optimisation procedure were based on results from Steps 1 and 2.

#### Model check and validation

The average absolute and squared residuals were calculated per donor to check the model fit, with model titer predictions less than zero rounded up to 0. We validated the model by refitting the model on the records of a random subset of 500 of the 755 donors (through Steps 1, 2A and 3). Next, using the resulting random effects distributional constraints we refitted *D*_*i*_ and *L*_*i*_ for the 255 donors outside the main fit, by maximising the likelihood:

$$L_{i}=L(D_{i,}{,L}_{i}|D,\sigma_{D}^{2},L,\sigma_{L}^{2},\sigma_{D,L})\prod_{k=1}^{k=w_{i}}C_{i}(t_{k}|{D_{i},L}_{i})L(\in |\sigma_{\in }^{2})$$

Here *C*_*i*_(*t*_*k*_) is the *k*^th^ difference in measurements, and *w*_*i*_ the total number of measurement differences for donor *i*. $L(\in |\sigma_{\in }^{2})$ is the probability density function of the normal distribution, and $L(D_{i,}{,L}_{i}|D,\sigma_{D}^{2},L,\sigma_{L}^{2},\sigma_{D,L})$ is the probability density function of the bivariate normal distribution [5]. The optimum in likelihood per donor was determined by maximizing the negative of the log of $L_{i}$. Again this fitting procedure is iterated, updating titers at boostering in each fit from the previous fit (the initial fit using measurements, 50 iterations per donor).

## Results

### Step 1: Natural anti-RhD decline

**Fig 1:**
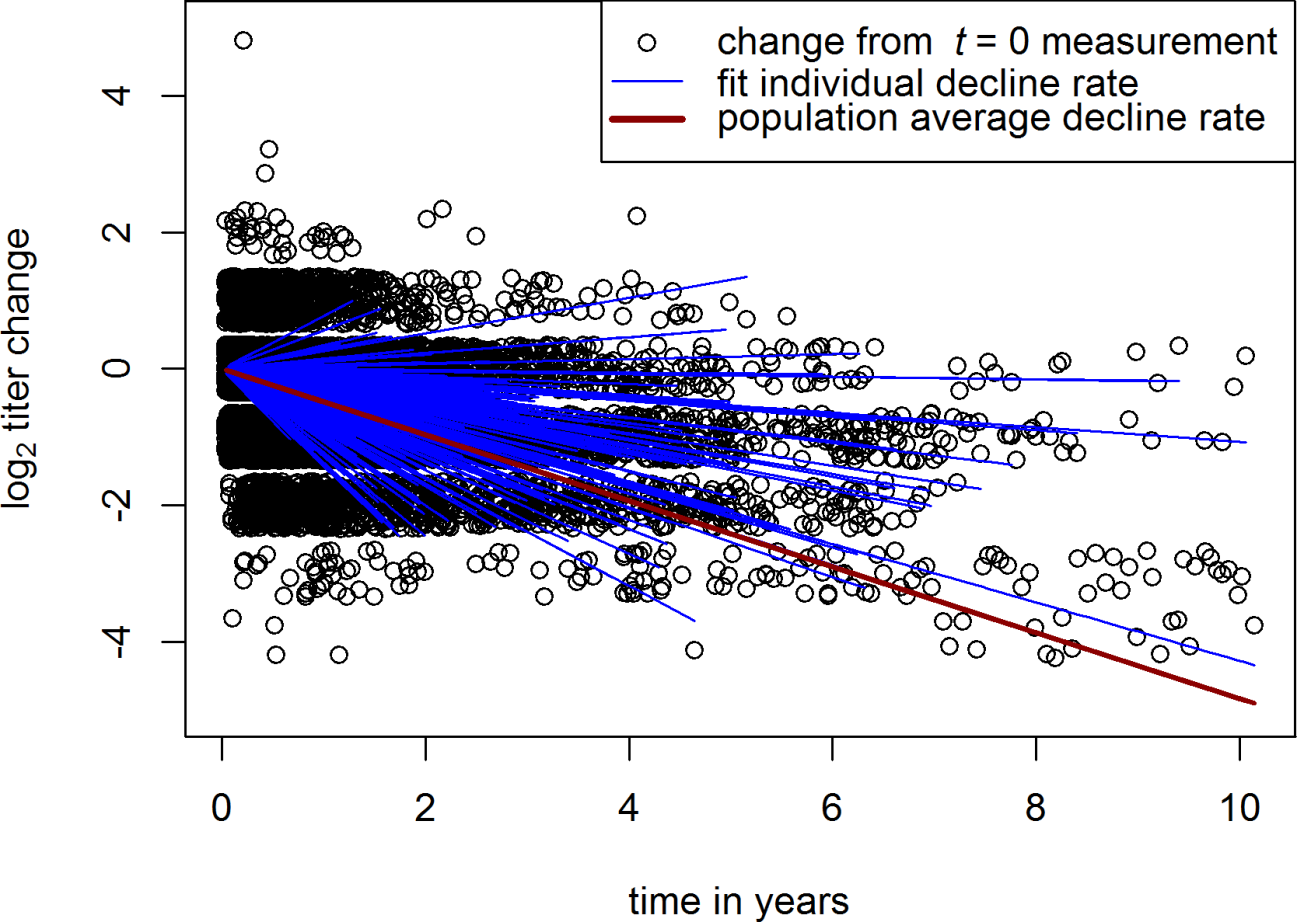
Random effects model fit to the natural anti-RhD titer decline of donors in the absence of boostering. A jitter of 1.75 was applied to the titer change measurements to enhance interpretability of the data. For individual fits, the line ends at a donor’s maximum time of measurement. Note that for 5% of donors a small positive annual change is modelled due to measurement errors and since it is not possible to restrict the model to negative values.

The best fit to the data-set from which we excluded booster events was obtained with a random-effects model, with a log-linear decline rate in anti-RhD titer (see Fig 1). In a random effects model the random effects parameters are fit per individual, where the individual estimates combined are restrained to form a normal distribution surrounding a population mean [6]. Those donors who decline slowly remain unboostered longer and will therefore provide a relatively large number of high titers at later time points. This would lead to biased estimates of the overall decline rate in an analysis that would not group observations per individual (see also S1 Fig). However, since the random effects model fits the data per individual, it is unaffected by this form of bias.

### Step 2A: Timing of the boostering effect

**Fig 2:**
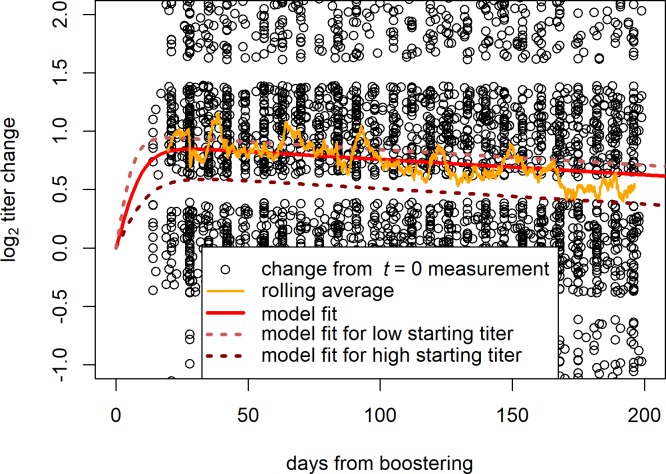
Change in anti-RhD titer after boostering. A jitter of 1.95 was applied to the titer change measurements to enhance interpretability of the data. The rolling average is calculated per 100 data points; the data was sorted by days from boostering but otherwise randomly ordered, per each consecutive 100 points both the average in titer change and the average in days from boostering was calculated. The population average change in log_2_ titer is fit with a model of logistic increase on top of the population average decline determined in Step 1. The upper and lower dotted lines show the model fit for all booster events where the starting log_2_ anti-RhD titer was ≤ 11 and ≥ 11 respectively (log_2_ 11 being the median starting titer). A peak in anti-RhD titer is reached quickly, at around 26 days for all estimates. Note however the paucity of titer measurements following early after boostering, as donations are at least 14 days apart. For a longer time scale and full data visualisation, see S1 Fig.

Boostering usually leads to an increase in anti-RhD titer (see Fig 2). To analyse the timing of this effect, we fitted a model to the average titer-change of all donors over time since boostering. A peak in anti-RhD titer appears to be reached quickly, at around 26 days from boostering. After 26 days the average anti-RhD titer declines again log-linearly, at the rate determined in Step 1.

### Step 2B: Magnitude of the boostering effect

**Fig 3:**
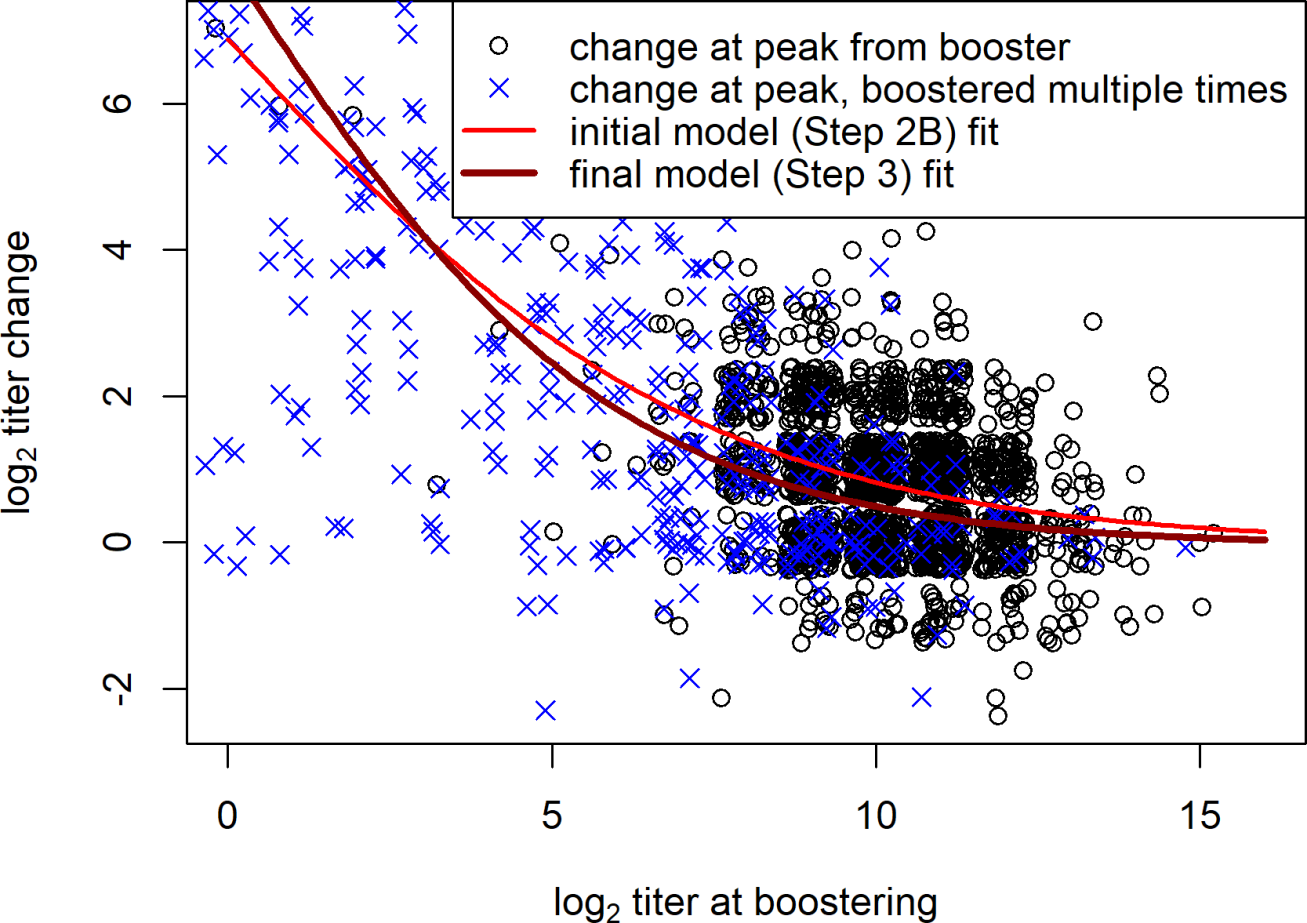
Peak in anti-RhD titer gain after boostering. A jitter of 1.95 was applied on the measurements of both titer change and titer at boostering to enhance interpretability of the data. Individuals were not boostered in the 50 days preceding the booster of interest. Black circles denote the approximate peak in increase assumed at 26–50 days from boostering (see Step 2A results), when a donor was boostered only once. Blue crosses denote this increase when one or more boosters were given subsequent to the booster of interest. Our best model fit to all these data-points together (shown for *t* = 26) hence represents an overestimate of the titer gain from boostering once. Also shown is the fit dependence from our final model (see Step 3 in the main text, shown for an average individual and also for 26 days from boostering).

From Fig 2 it also becomes clear that the increase in anti-RhD titer from boostering is correlated with the donor’s titer at the time of boostering: the increase is substantially lower when the starting titer is high. In Fig 3 the peak increase in titer, (assumed at 26 to 50 days from boostering, see Step 2A), is plotted against the titer at the time of boostering. The best fit for this relationship was found modelling the log anti-RhD titer increase as logistically saturating with the log anti-RhD starting titer. Note that while on the log scale monotonic decrease in the booster effect with starting titer is clear, translated to an absolute scale the magnitude of increase seems to remain constant over a broad range of starting titers (shown in S2 Fig).

### Step 3: The integral model

**Fig 4:**
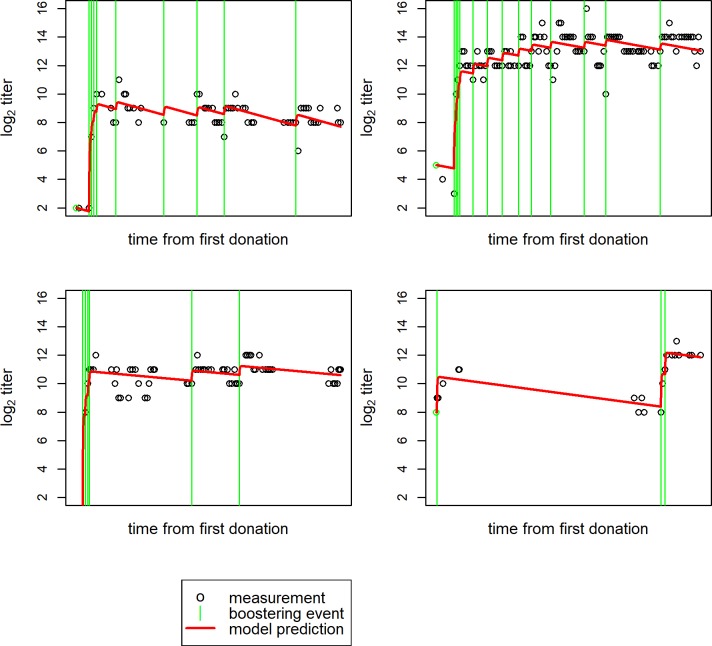
Four examples of model fits to individual donor titers observed over time. **(**See S5 Fig for the model fits for all individual donors).

By combining the findings from the earlier steps, we obtain a model which describes the full data-set. With the assumption that individual booster events affect anti-RhD titer independently, our integral model of anti-RhD titer change in donors includes only four parameters. Two of these were fit as donor level parameters, namely the natural decline rate, and the speed of saturation in titer response with the starting titer at boostering. The two general model parameters determine the maximum boostering effect (at a starting titer log_2_ 0), and the time to titer increase after boostering. Using their first anti-RhD titer measurement, the dates of booster events and their two fitted individual parameters, the model allows predicting all further titer measurements of a donor (see Fig 4).

The average donor experiences an annual decline in log_2_ anti-RhD titer of 0.55 (see Table 1). This translates to a decline of 32% of a donor’s absolute titer every year. Of all donors 11% are fit with a (small) positive annual change, which is biologically implausible and likely an artefact of the short follow-up time for some donors and the rather large error in measurements; the model estimates that in one third of records the actual titer is at least log_2_ 0.60 above or under the measured titer. To exclude the implausible values by these causes, we restrict our model summary to the 50% limits of the fit donor distribution. We see that individual donors vary greatly, with the mid half of donors estimated to decline from 0.20 to 0.77 log_2_ per year (-13% to -41% in absolute titer).

**Table 1:** Model fit.

Parameter fit	Annual log_2_ change in anti-RhD titer	Boostering effect in log_2_ titer at starting titer log_2_ 3	Boostering effect in log_2_ titer at starting titer log_2_ 9	Boostering effect in log_2_ titer at starting titer log_2_ 11	Boostering effect in log_2_ titer at starting titer log_2_ 13	Mean eventual log_2_ titer when boostering 0.5 times yearly	Mean eventual log_2_ titer when boostering once yearly	Mean eventual log_2_ titer when boostering 1.5 times yearly
Population average^a^	- 0.55	+ 4.26	+ 0.73	+ 0.38	+ 0.20	8.28	10.16	11.31
Upper 25^th^ percentile of fit donors^b^	- 0.20	+ 4.84	+ 1.21	+ 0.72	+ 0.42	12.18	14.64	16.04
Median value of the fit donors^b^	- 0.42	+ 4.33	+ 0.78	+ 0.41	+ 0.22	9.47	11.53	12.75
Lower 25^th^ percentile of fit donors^b^	- 0.77	+ 3.76	+ 0.45	+ 0.21	+ 0.10	7.37	9.09	10.11

^a^ Population average values directly from the model fit.

^b^ As a fraction of the population of anti-RhD donors that were fit with the model (first column N = 755 donors, remaining columns N = 590, excluding 165 donors who were never boostered).

While the maximum recorded titer measurement is log_2_ 18, only 2% of all measurements were equal or greater than log_2_ 14, the median measurement was log_2_ 11.

Starting at a population median titer of log_2_ 11 the average donor’s increase in anti-RhD titer due to boostering is log_2_ 0.38 (+30% in absolute titer value). Starting from log_2_ 3, 9 or 13 this increase is fit respectively as log_2_ 4.26, 0.73 and 0.20 (+1816%, +66% and +15% on absolute scale). Relative variation between donors is especially large at high titer; from titer at boostering of log_2_ 13 the lower 25^th^ percentile of donor’s goes up at most log_2_ 0.10 per booster event, while the top 25^th^ percentile is still expected to go up log_2_ 0.42 or more (7% and 34% absolute increase respectively).

With the fitted model we can analyse the anticipated effect of different boostering regimes. If boostering would occur at a fixed interval, a donors’ titer is expected to reach a dynamic plateau where the increase in titer by boostering equals the decline in titer over the interval. When boostered annually the average donor’s titer is expected to fluctuate between log_2_ 9.90 just before boostering and 10.40 at 26 days after boostering. Average titer over the year between booster events would be log_2_ 10.16.

The average donor’s plateau titer would decrease to log_2_ 8.28 when boostering would occur only once per two years. As boostering has more effect at lower starting titers, increasing the boostering frequency has less effect on the plateau log_2_ titer: it increases to 11.31 at a boostering frequency of thrice per two years. The relative gain in titer with boostering is greater for those most sensitive to boostering: for those at the highest 25^th^ percentile, changing frequency from 0.5 to 1.5 times per year increases the plateau log_2_ titer from 12.18 to 16.04.

76 of the 755 donors (10%) in our data-set are male, their median estimated yearly decline was log_2_ 0.36 versus log_2_ 0.42 for the females. Starting at log_2_ 11, median increase from boostering was 0.50 for men versus 0.41 for women. Neither gender difference was statistically significant; t-test *p* = 0.61 and *p* = 0.48 respectively testing for difference in the mean of the parameters *D*_*i*_ and *L*_*i*_ by gender.

We did find a significant correlation between age at first donation of a donor and the fitted decline rate *D*_*i*_, at −0.119 (Pearson’s correlation, *p* = 0.00) (see S3 Fig).We found no indication for influence of age on the boostering effect; the correlation between age at first recorded booster event and *L*_*i*_ was +0.009 (*p* = 0.827).

### Model check and validation

On average, the absolute error in prediction per measurement is log_2_ 0.63 (the mean squared error is log_2_ 0.75). For 94 of the 755 donors (12%) the mean absolute error is more than log_2_ 1, for 16 donors (2%) this error is more than log_2_ 2 (see S2 which contains graphs for the individual model fits for all donors, ordered by their mean absolute error). Among those with larger fit errors we see 7 donors who despite being boostered–some up to 4 or even 5 times–never reached a titer higher than log_2_ 5; they seem unresponsive to boostering (see S5 Fig for donors 724, 729, 735, 747, 752, 753 and 755).

When refitting the model over the records of a random subset of 500 of all 755 donors, model parameters changed only slightly (see S4 Fig). For the donor records included in the refit of the model the average absolute model fit error was 0.63, which is identical to the mean error in the main fit over all donors. The mean average error is only slightly higher (0.65) for the records of the remaining 255 donors. Their measurements were approximated by the model fitting only parameters *D*_*i*_ and *L*_*i*_, as restrained by the refit random-effects model (see Methods).

## Discussion

The presented model is a simplification of biological reality. The processes underlying the dynamics of anti-RhD titer change are actually complex, involving many immune cell types [7]. Yet, our model does capture what is most important: the fact that titers in donors gradually decline over time unless boostered, that the impact of boostering saturates with increasing titer, and that of these effects the magnitudes are personal. This allows our model to describe how anti-RhD titer changes over time in individual donors.

When a donor is boostered a higher initial titer results in lower immune response on log scale. Although the absolute response in anti-RhD production may, as long as it is not too high, be independent of the starting titer, clearly most if not all anti-RhD production is suppressed above some individual maximum titer level. We can only speculate as to this apparent antibody-mediated immunosuppression; understanding the phenomenon mechanistically would require further empirical research.

It has long been known that individuals differ substantially in the maximum of their anti-RhD titer: famously, one man in Australia with an exceptionally high titer was solely responsible for a considerable fraction of all immunoprophylaxis produced within this country [8]. Yet proper quantification of such individual differences is needed to allow optimisation of the anti-RhD yield whilst minimizing donor exposure to RhD-positive blood.

The titer response data analysed is unique in extent: we know of no other published information on anti-RhD titer response over such long follow-up time. Only the availability of measurements on such a large number of donors exposed to multiple booster events allowed modelling of individual responses. Yet limitations of our results are due to the observational and historical nature of the data.

For instance, unfortunately it was not recorded which donors were newly immunised and which immunised naturally by pregnancy with an RhD-positive foetus. Therefore we could not address whether processes may be different between natural versus induced immunisation, and when first becoming immunised compared to when hyper-immunised. Unless infertile, woman under 45 did not receive any boosters, recruitment differed between sexes (with most woman being naturally immunised), and donor protocols and recruitment strategies changed over time. These facts may have induced or obscured associations between observed outcomes.

We found no effect of gender on the fitted individual parameters, but we did find age to be associated with the individual decline rate. Such an effect of age on antibody production, including menopause induced for woman, and senescence effects, has been reported for different antigens [9,10, 11]. Although our model might potentially be improved by incorporating age, a changing decline rate would be challenging to implement. In addition, the impact of such an extension is expected to be limited, as the observed age effect is only small when compared to the overall intra-individual variation.

Differential drop-out of individuals does not lead to bias for factors modelled as random effects at donor level. However, it is likely that donors with low anti-RhD titers stopped donating more frequently before the start of the data inclusion period (1998). Also, woman immunised by pregnancy showing high anti-RhD titers are more likely to have been identified and recruited. Therefore, the donor population analysed is likely characterised by relatively high titers and is not expected to reflect the general Dutch population if immunised.

Much of the titer increase is already achieved at 14 days from boostering (the minimum time between donations). Ideally, in order to obtain a better estimate for the timing of boostering effect, in directed research multiple titer measurements would follow more closely upon boostering. Further research of importance for boostering strategies would address the burden of boostering as experienced by donors, and how this relates to the boostering frequency and titer levels.

Our model validation shows that the model is relatively insensitive to outliers. Hence for the prediction of future titers, for new donors only their two individual model parameters need to be determined. However, defining what data is needed for a sufficiently accurate prediction is complicated, as the precision in the decline rate depends on unboostered follow-up time whereas the precision in boostering effect depends on the number of boosters and the range of titers at boostering. Further research is required in order to adequately address this problem.

Despite careful checking and excluding years of lower data quality, some documentation errors or missing booster events likely remain in the data set, which may explain some of the bad individual fits.

A relatively large error was apparent in the titer measurements, especially when translated to absolute scale on which the antibody yield ultimately depends. We expect that predictions might be improved substantially if more accurate titer measurements would be available. The fit distributions of the individual parameters would be narrower; fewer donors would be fit with a spurious positive natural titer change. Enhanced prediction power given different measurement scenarios could be assessed by simulations.

Model utility may also be improved if one could link the individual decline rate and sensitivity to boostering to biological donor characteristics [12, 13]. If such associations could be established, one could predict individual (new) donor responses on basis of for example donor allotyping.

If certain biological factors would have a great impact on anti-RhD titer, we would expect to see different groups of donors, rather than a continuum in the individual characteristics [14]. However, we did not find any evidence for such sub-groups of donors. For example, we observe a continuum in the mean titer over the donation career of donors (excluding their first donation year) (results not shown). We expect however that individuals not responding or hardly responding to boostering may form a sub-group of the population. A few of these in the data-set strongly inflated the fitted variance in the decline rate (see S4 Fig). However, since it was not recorded who were newly immunised, this data-set is not suitable for quantifying a proportion of “non-responders” in the Dutch donor population.

Despite the fact that anti-RhD donors are an exceptionally motivated group of altruists, burdening them by booster events should be avoided as much as possible. Current protocol calls for boostering of donors when their titer declines by a factor of four (log_2_ 2) below their plateau value, or when their titer declines to below log_2_ 9, but in principle not with a frequency greater than once per year. Especially in light of the large measurement error obscuring actual decline in titer, and the fact that apparent titer plateau is determined by past boostering frequency, this protocol is not straightforward to follow. Measurement error may lead to delays in boostering or unnecessary booster events. The latter could be the reason why we did not observe any increase in titer after many of the given boosters (see Fig 3).

The model derived in this paper can be used to predict change in anti-RhD titers in donors over time. This can be used to derive an enhanced immunization policy in which boostering is adjusted to the anticipated anti-RhD decline, the effectiveness of boostering and the anti-RhD titer required.

## Supporting information

S1 Dataset Subsequent donor titers at time of donation and indication of boostering of all 755 donors in our study. (CSV)

S1 Fig Change in anti-RhD titer after boostering. Same data are presented as in Fig 2 in the main text, with additionally the measurements ≥200 days from boostering (excluding from view one log_2_ titer change measurement at -10).
(JPG)

S2 Fig Peak in anti-RhD titer gain after boostering. Same data are presented as in Fig 3 in the main text; here the titer change is plotted on an absolute scale.
(JPG)

S3 Fig Relationship between the individually fitted titer decline parameter *D*_*i*_ and an individual’s age at first donation in the data-set. Pearson’s correlation coefficient is −0.119 (*p* = 0.001).
(JPG)

S4 Fig Histograms of the individual model parameter values for the 755 donors, overlaid with the normal distributions of the random effects model fit. Top: the log decline rate per day *D*_*i*_. Bottom: the saturation speed in decline of boostering effect with log_2_ starting titer, *L*_*i*_ (excluding the 165 donors who were never boostered).
(JPG)

S5 Fig Model fit per donor to observed titers and boostering events over time. (PDF)
