# Correction: Predicting anti-RhD titers in donors: Boostering response and decline rates are personal

**DOI:** 10.1371/journal.pone.0198381

**Published:** 2018-05-24

**Authors:** Anneke S. de Vos, C. Ellen van der Schoot, Dimitris Rizopoulos, Mart P. Janssen

The second author’s name is spelled incorrectly. The correct name is: C. Ellen van der Schoot. The correct citation is: de Vos AS, van der Schoot CE, Rizopoulos D, Janssen MP (2018) Predicting anti-RhD titers in donors: Boostering response and decline rates are personal. PLoS ONE 13(4): e0196382. https://doi.org/10.1371/journal.pone.0196382
