# Supplementary figures and images for: Predicting anti-RhD titers in donors: Boostering response and decline rates are personal
Source: PLoS One. 2018 Apr 26;13(4):e0196382. doi: 10.1371/journal.pone.0196382 (PMC5919536; doi:10.1371/journal.pone.0196382)

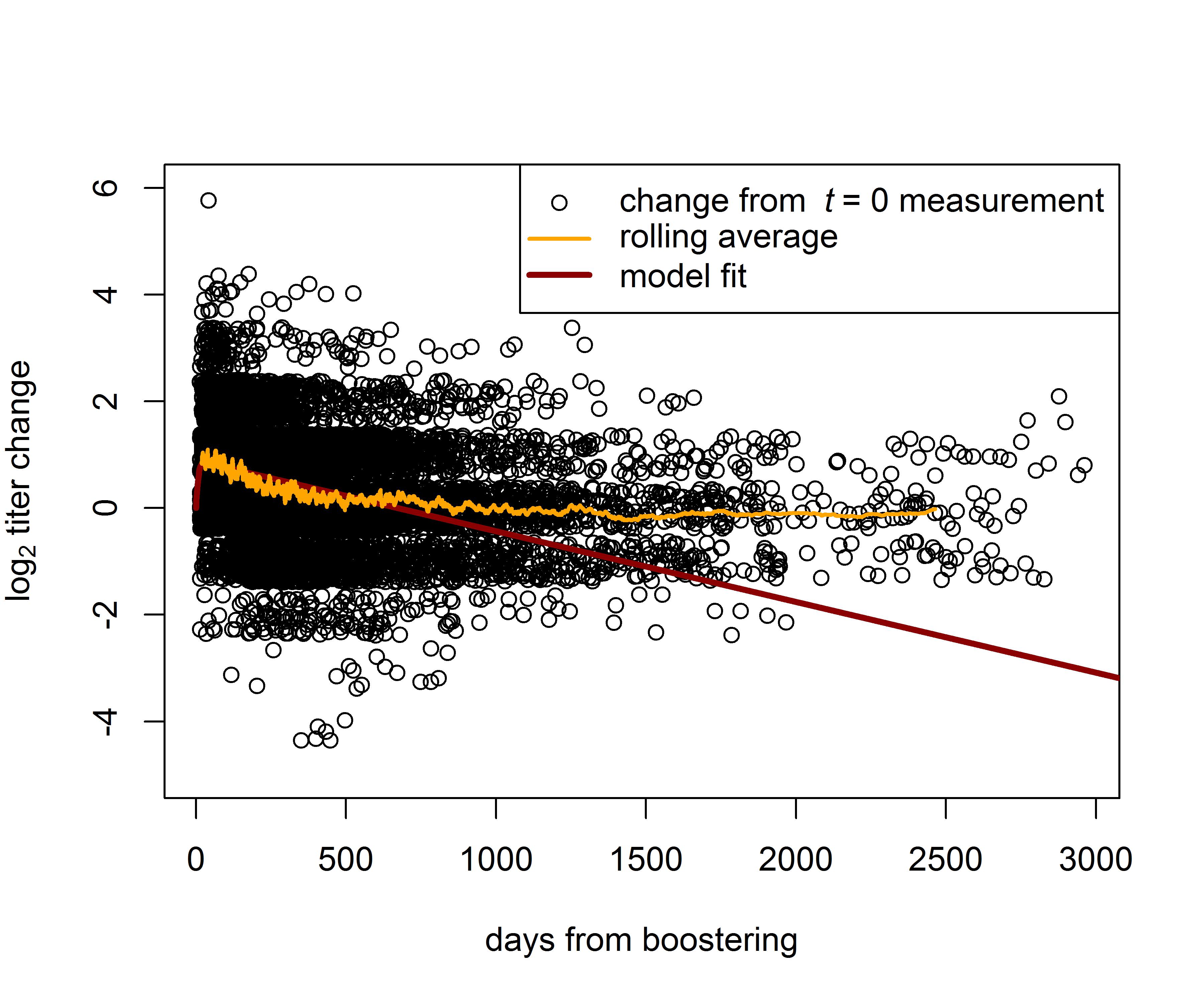

Supplement: S1 Fig — Same data are presented as in Fig 2 in the main text, with additionally the measurements ≥200 days from boostering (excluding from view one log2 titer change measurement at -10). (JPG) [file pone.0196382.s002.jpg]

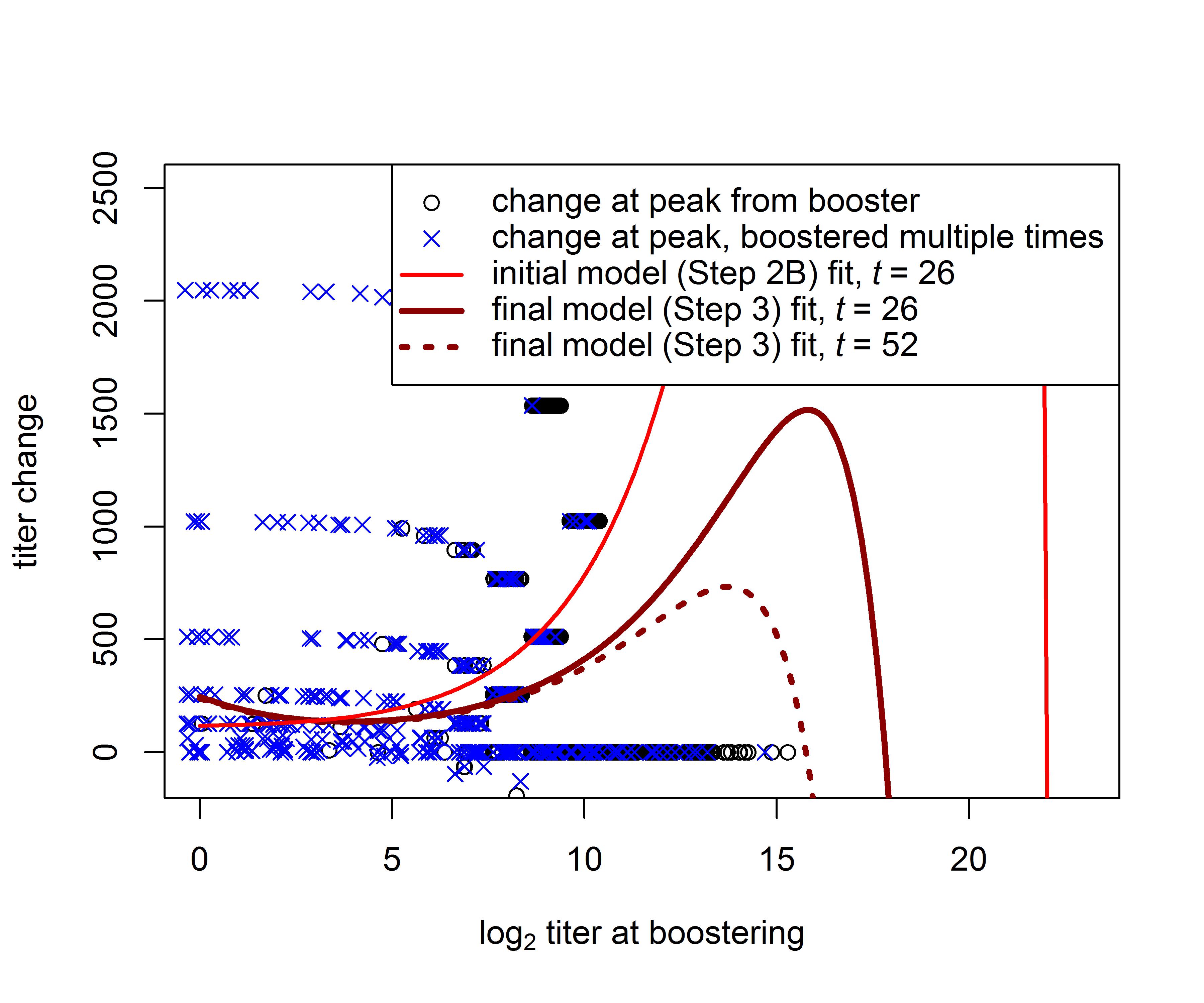

Supplement: S2 Fig — Same data are presented as in Fig 3 in the main text; here the titer change is plotted on an absolute scale. (JPG) [file pone.0196382.s003.jpg]

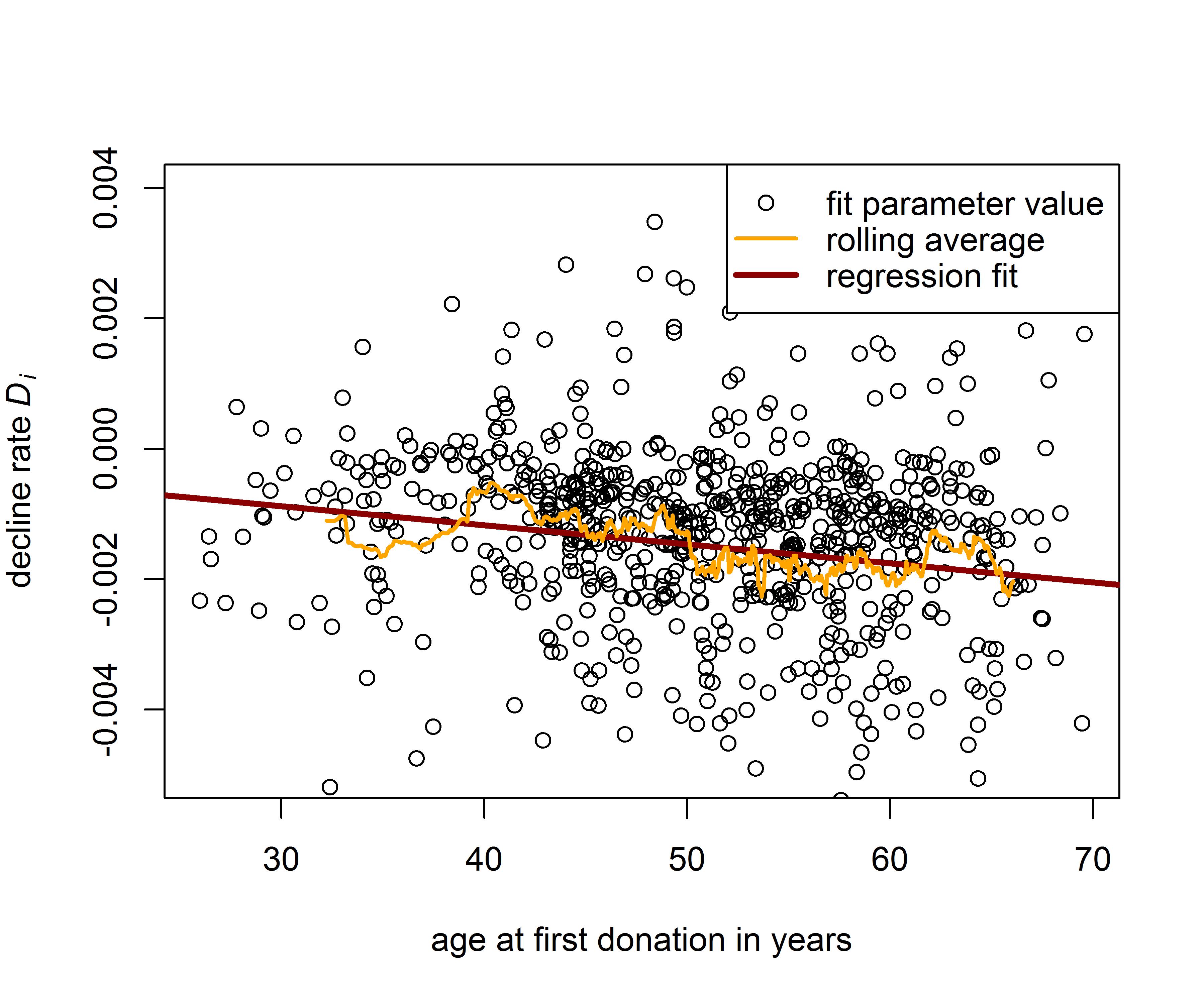

Supplement: S3 Fig — Pearson’s correlation coefficient is −0.119 (p = 0.001). (JPG) [file pone.0196382.s004.jpg]

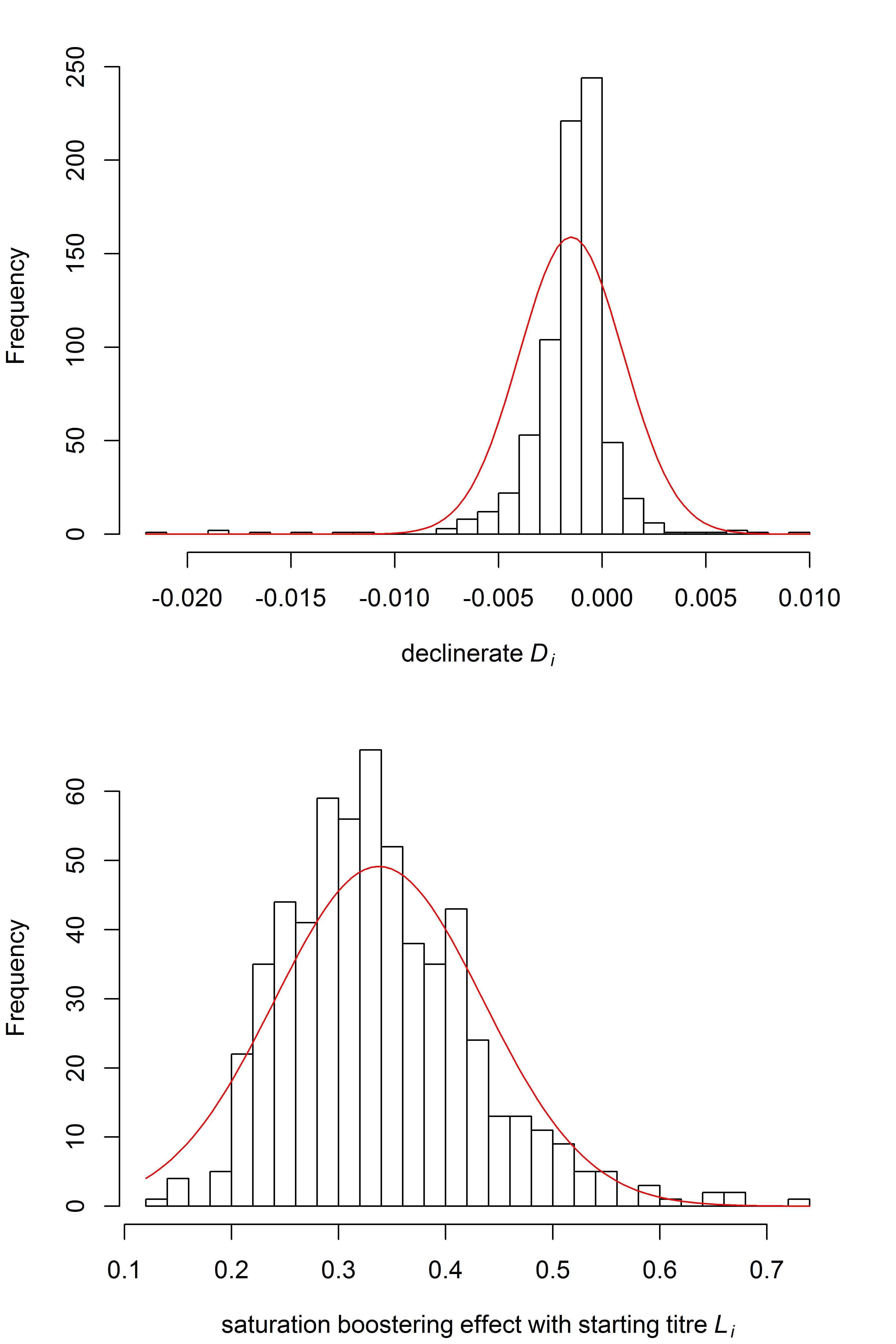

Supplement: S4 Fig — Top: the log decline rate per day Di. Bottom: the saturation speed in decline of boostering effect with log2 starting titer, Li (excluding the 165 donors who were never boostered). (JPG) [file pone.0196382.s005.jpg]
