# Supplementary material for: Predicting anti-RhD titers in donors: Boostering response and decline rates are personal
Source: PLoS One. 2018 Apr 26;13(4):e0196382. doi: 10.1371/journal.pone.0196382 (PMC5919536; doi:10.1371/journal.pone.0196382)
Supplement: S5 Fig — (PDF) [file pone.0196382.s006.pdf]

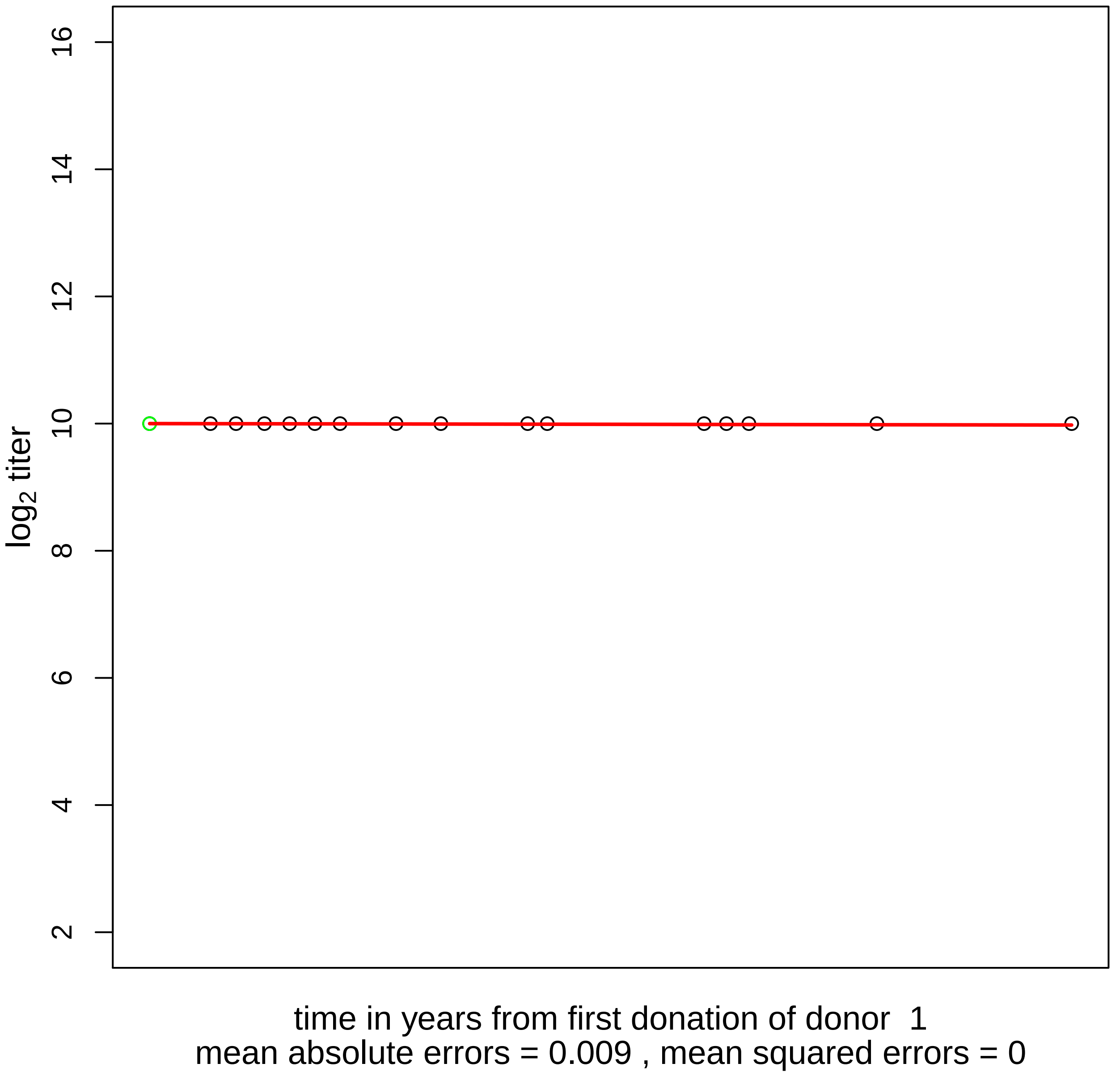

log2 titer
2
4
6
8
10
12
14
16
time in years from first donation of donor 1
mean absolute errors = 0.009 , mean squared errors = 0

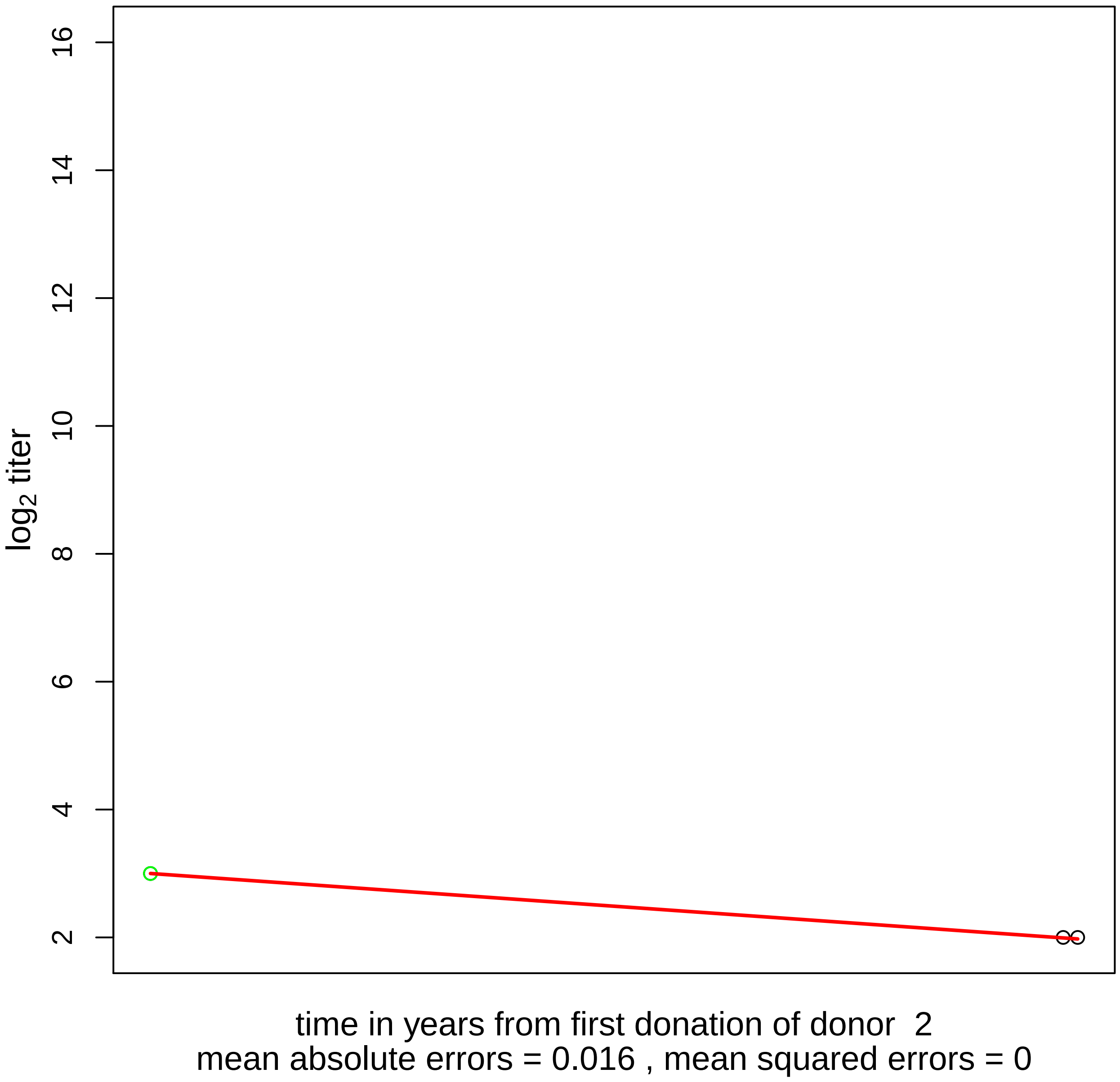
16
14
12
10
8
6
4
2
log2 titer
time in years from first donation of donor 2
mean absolute errors = 0.016 , mean squared errors = 0

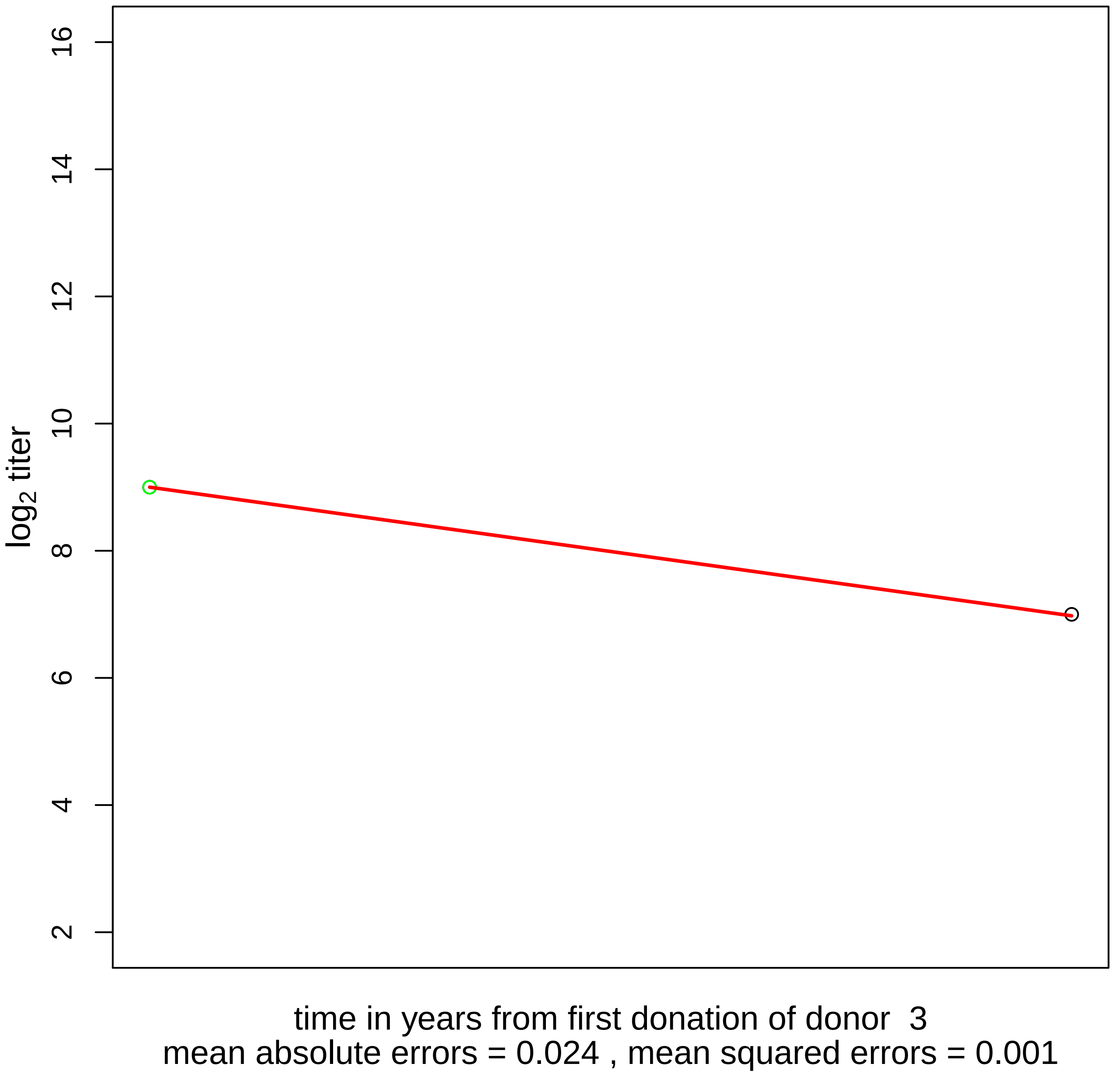
log2 titer
16
14
12
10
8
6
4
2
time in years from first donation of donor 3
mean absolute errors = 0.024 , mean squared errors = 0.001

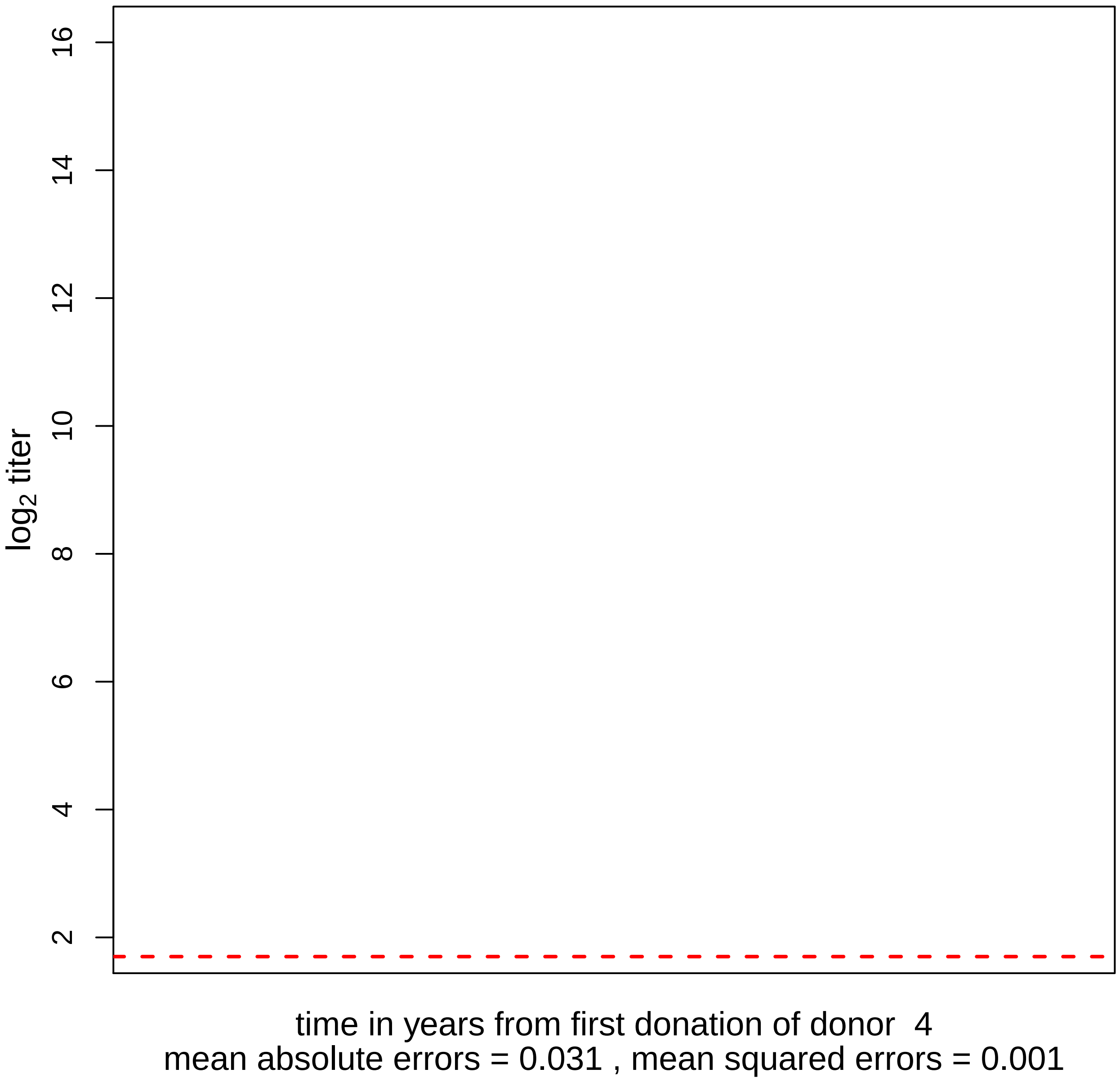

16
14
12
10
8
6
4
2
log2 titer
time in years from first donation of donor 4
mean absolute errors = 0.031 , mean squared errors = 0.001

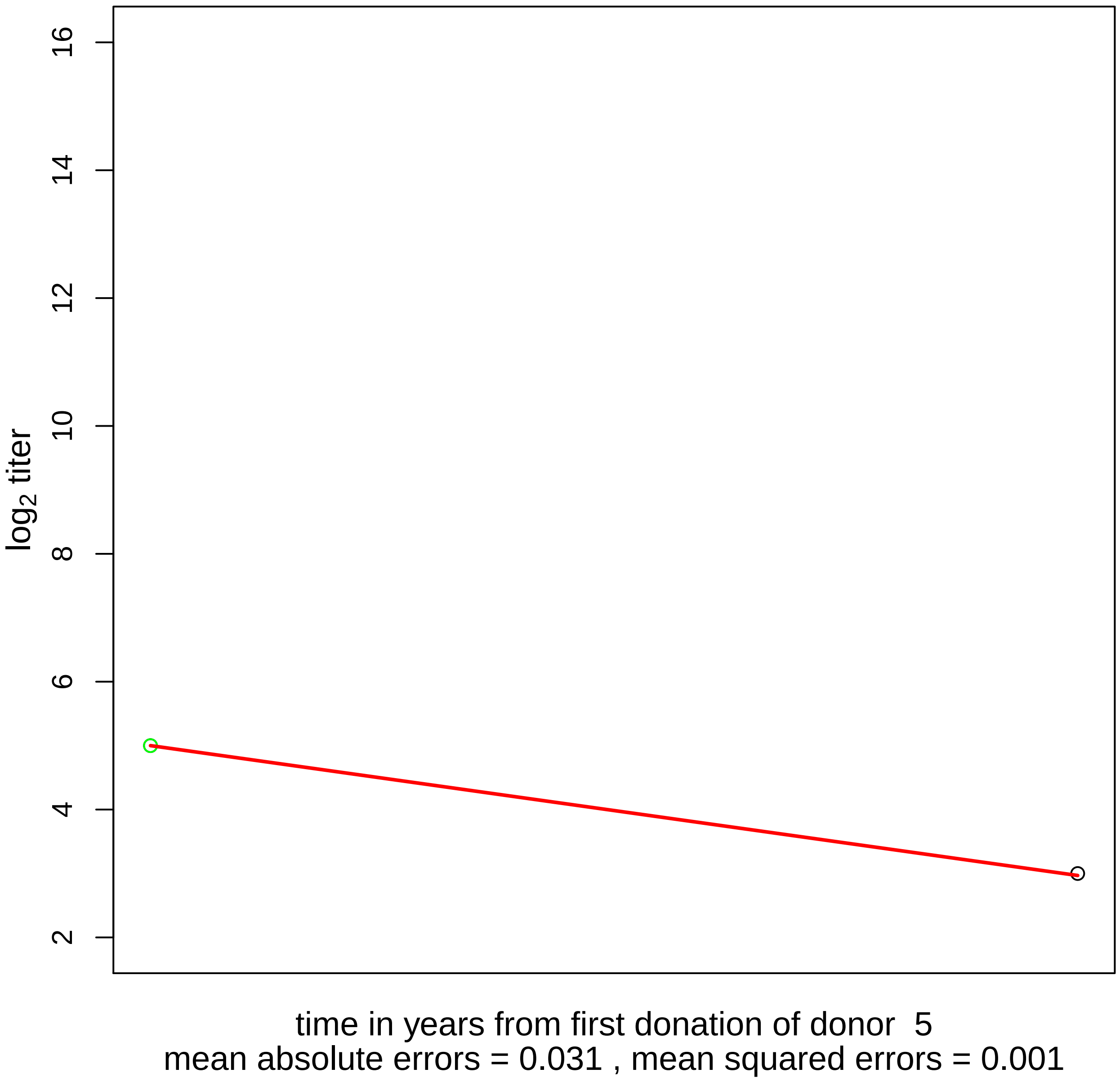
16
14
12
10
8
6
4
2
log2 titer
time in years from first donation of donor 5
mean absolute errors = 0.031 , mean squared errors = 0.001

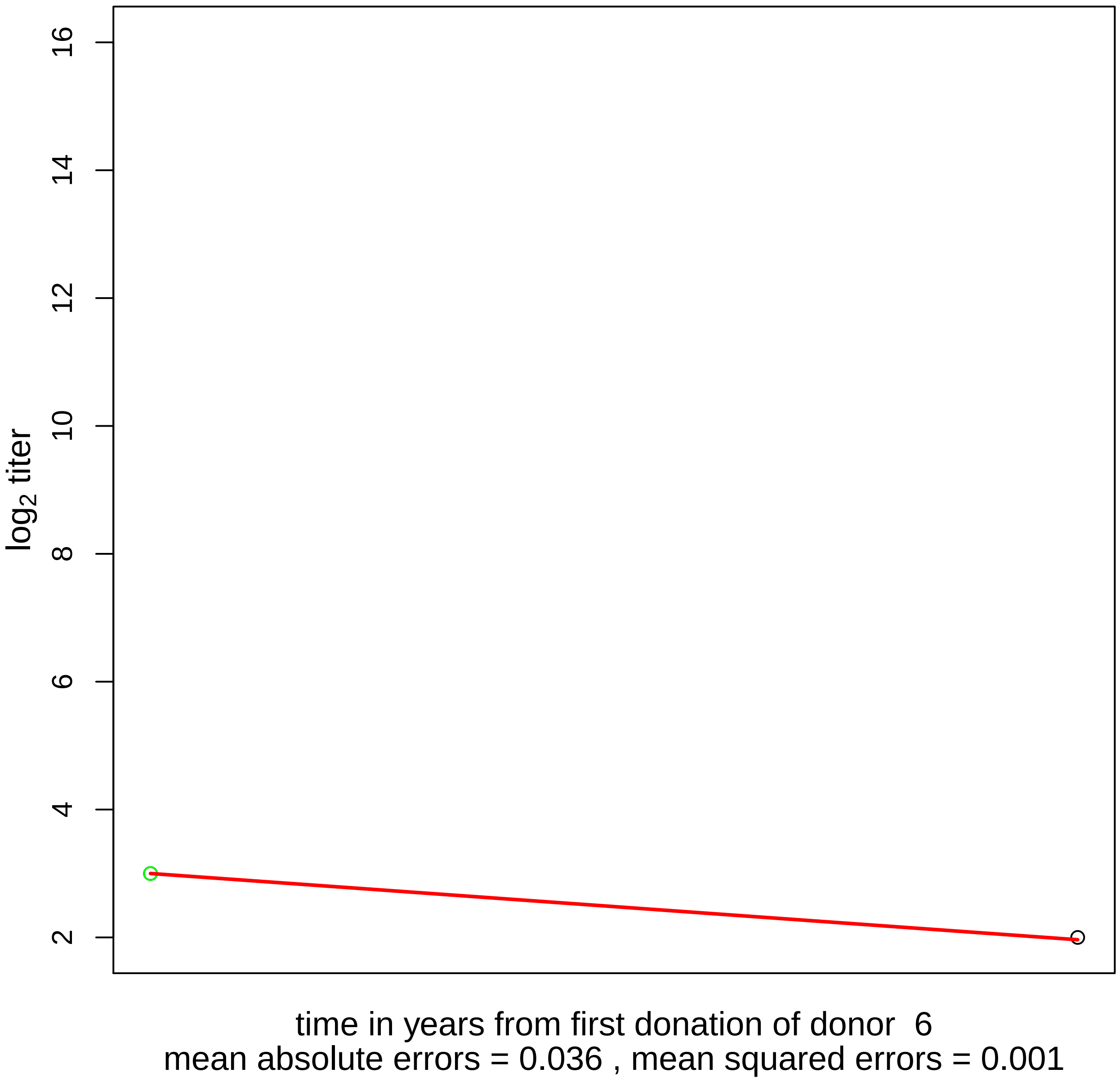

log2 titer
16
14
12
10
8
6
4
2
time in years from first donation of donor 6
mean absolute errors = 0.036 , mean squared errors = 0.001

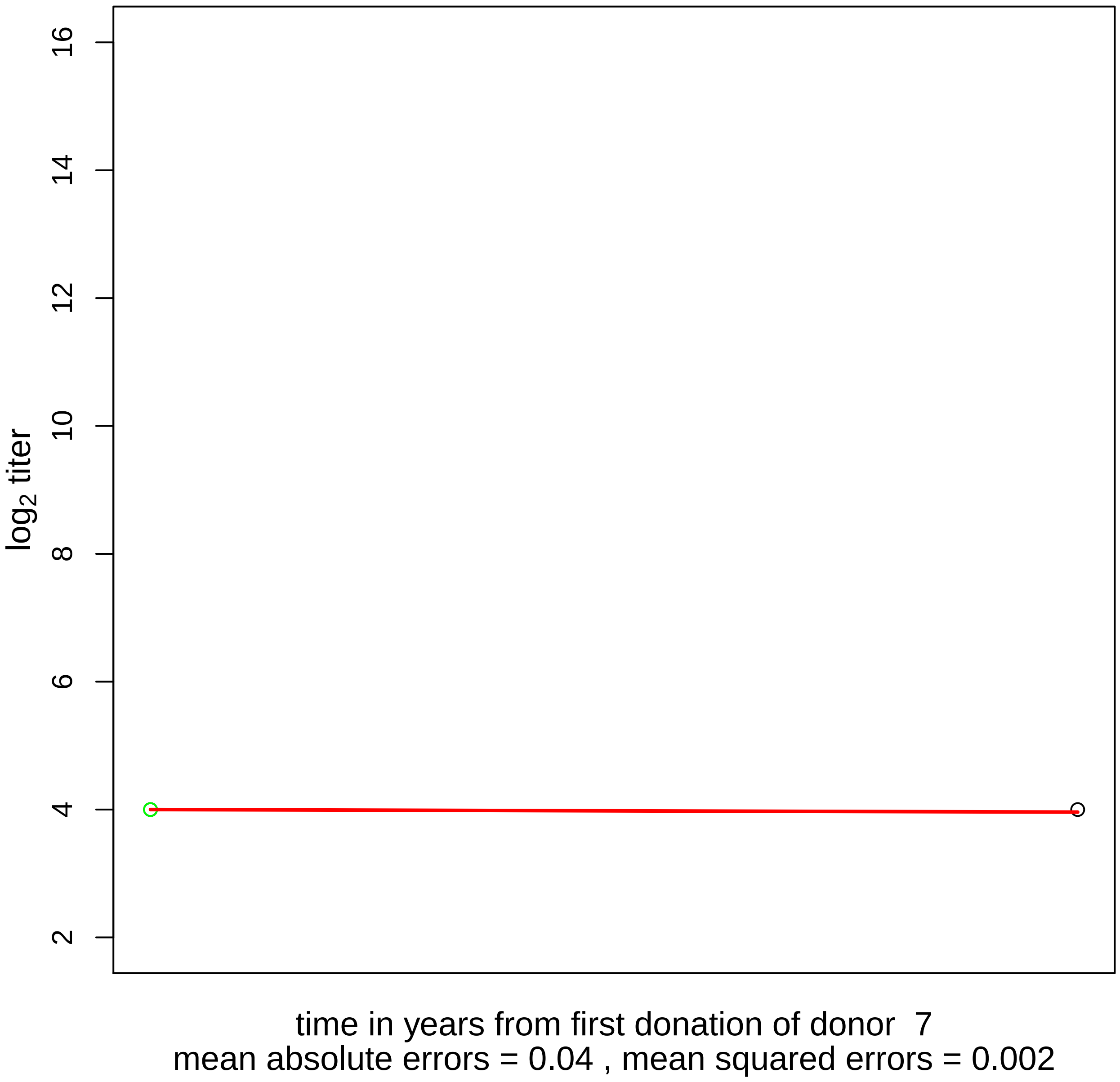

16
14
12
10
8
6
4
2
log2 titer
time in years from first donation of donor 7
mean absolute errors = 0.04 , mean squared errors = 0.002

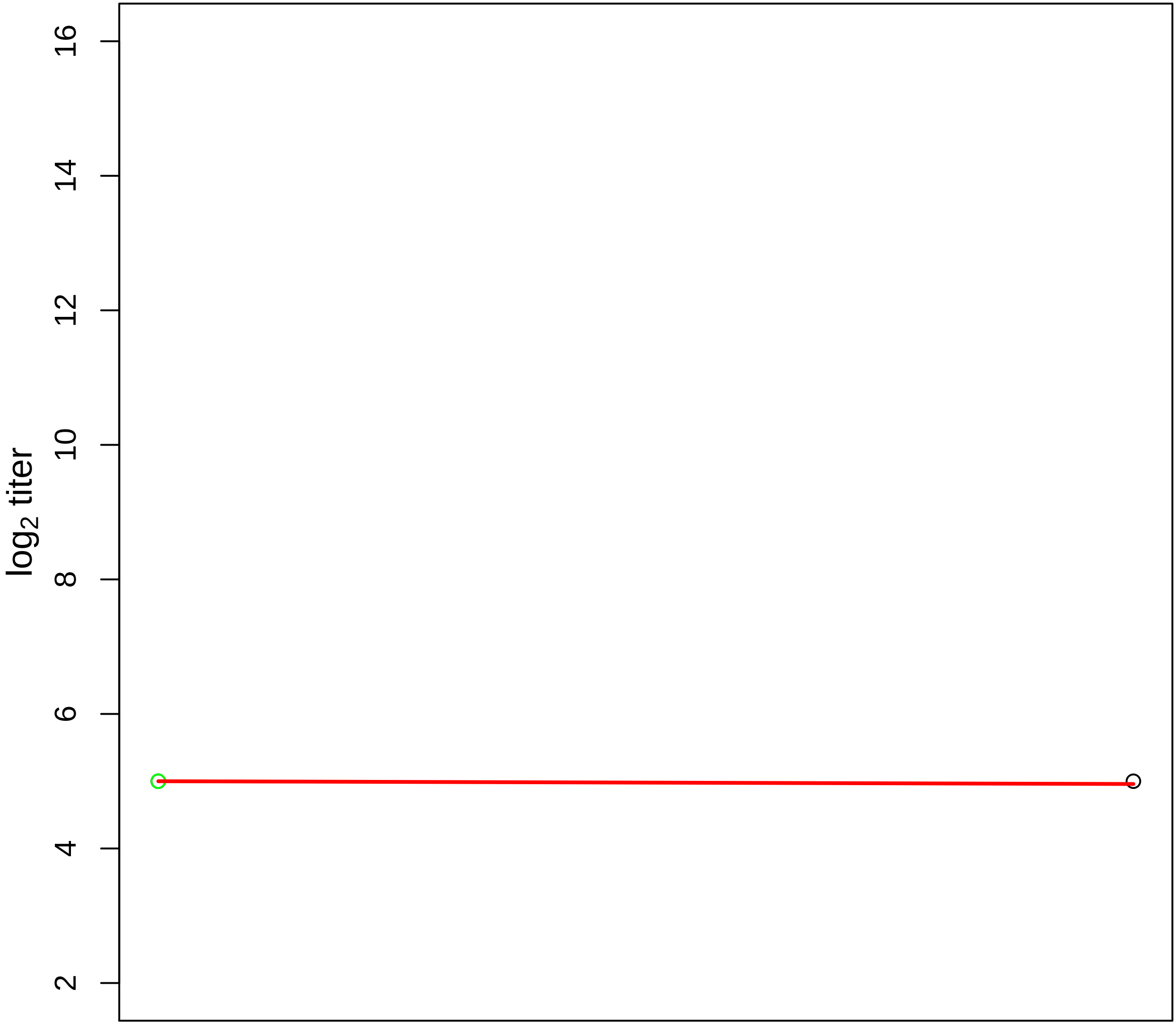

time in years from first donation of donor 8
mean absolute errors = 0.042 , mean squared errors = 0.002

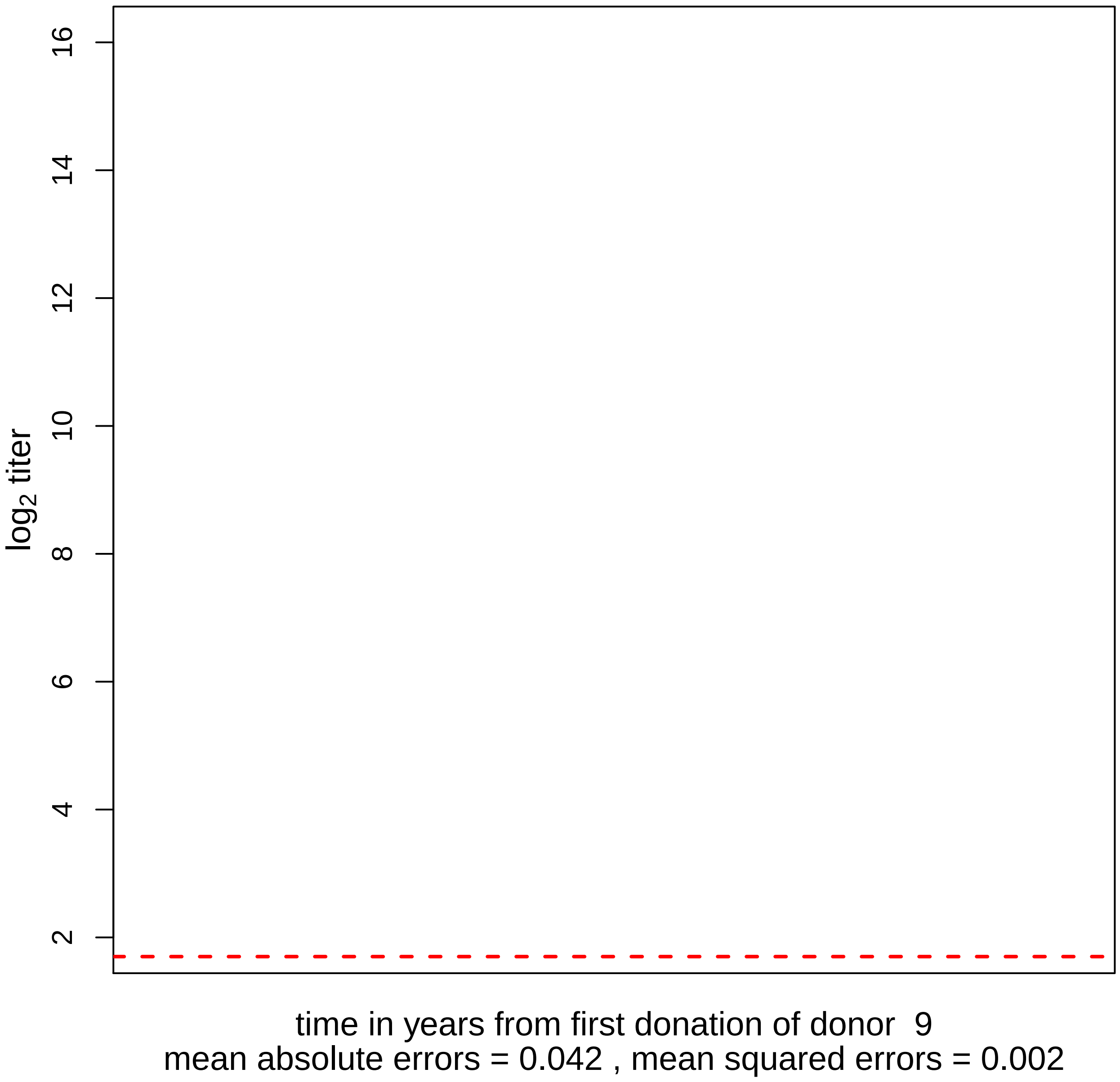

log2 titer
16
14
12
10
8
6
4
2
time in years from first donation of donor 9
mean absolute errors = 0.042 , mean squared errors = 0.002

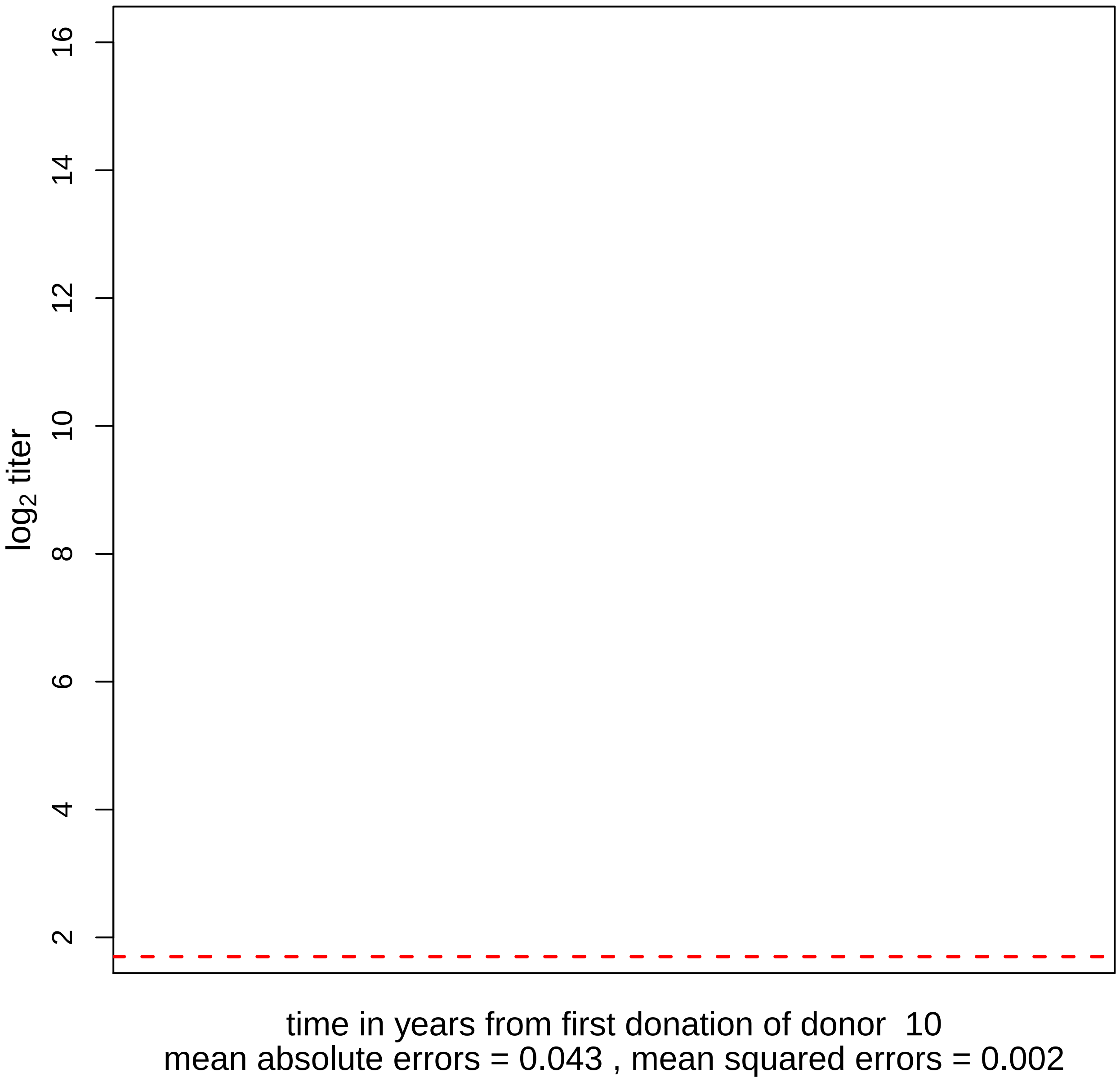
16
14
12
10
8
6
4
2
log2 titer
time in years from first donation of donor 10
mean absolute errors = 0.043 , mean squared errors = 0.002

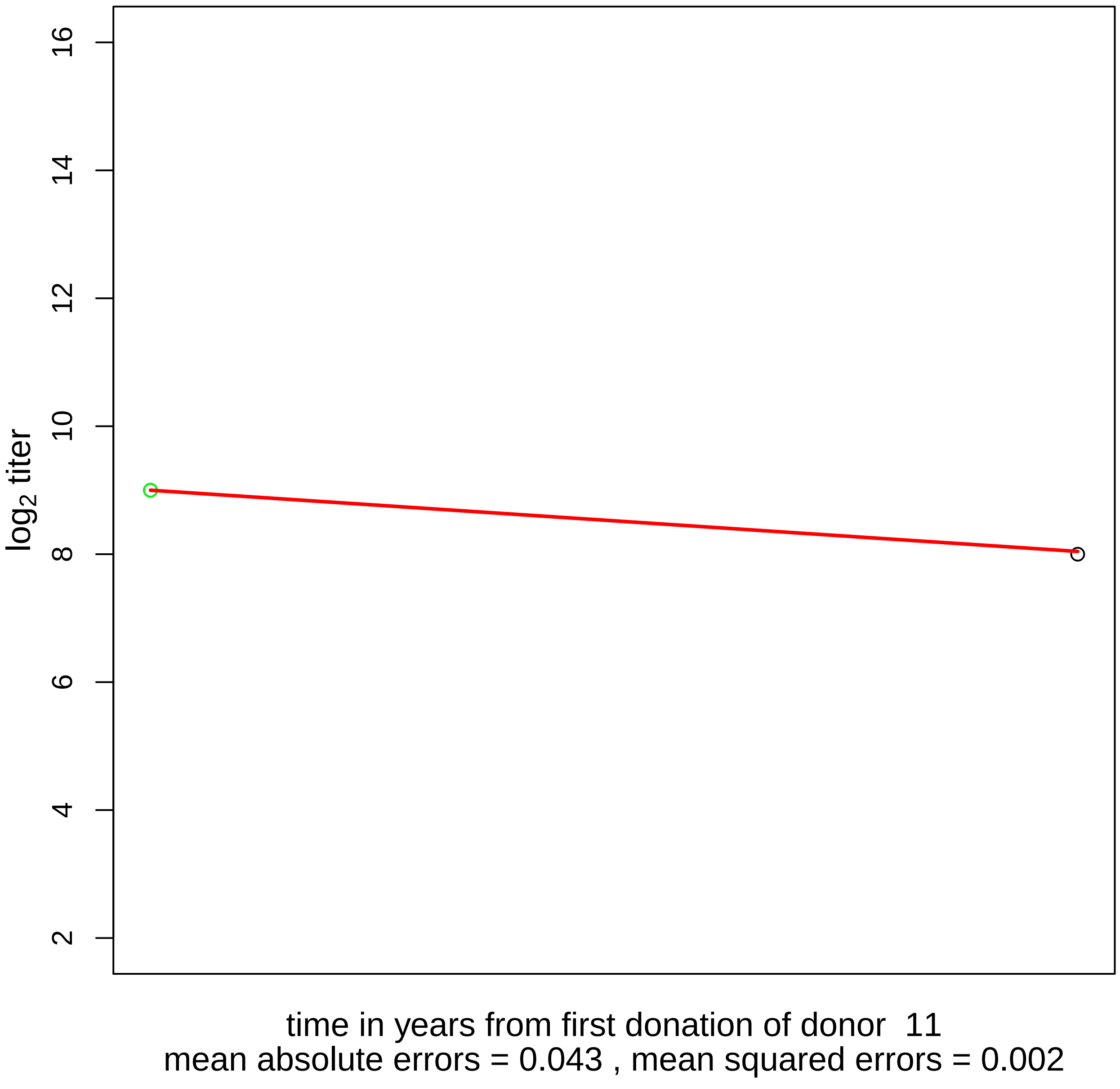

log2 titer
2
4
6
8
10
12
14
16
time in years from first donation of donor 11
mean absolute errors = 0.043 , mean squared errors = 0.002

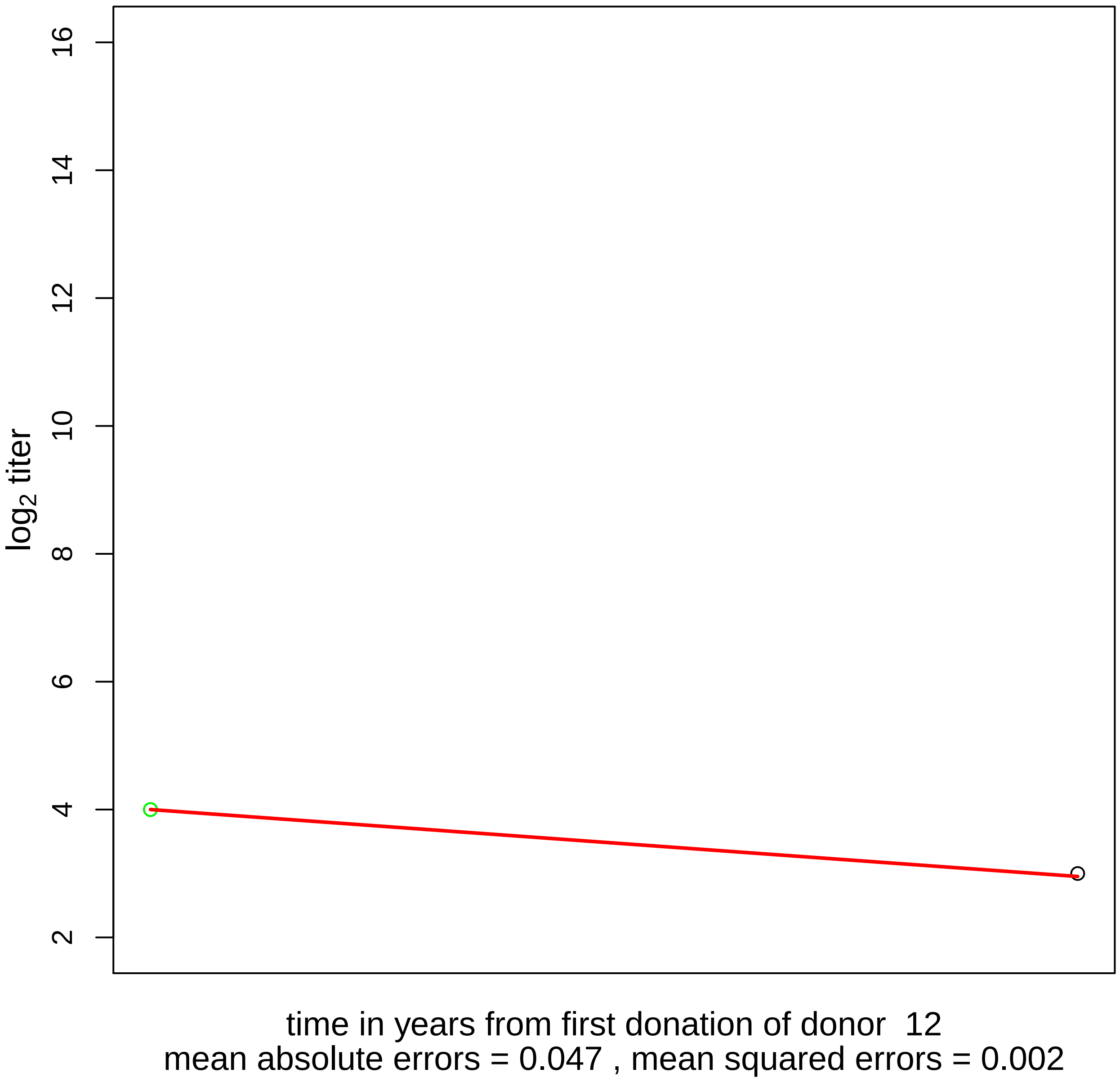

16
14
12
10
8
6
4
2
$\log_2$ titer
time in years from first donation of donor 12
mean absolute errors = 0.047 , mean squared errors = 0.002

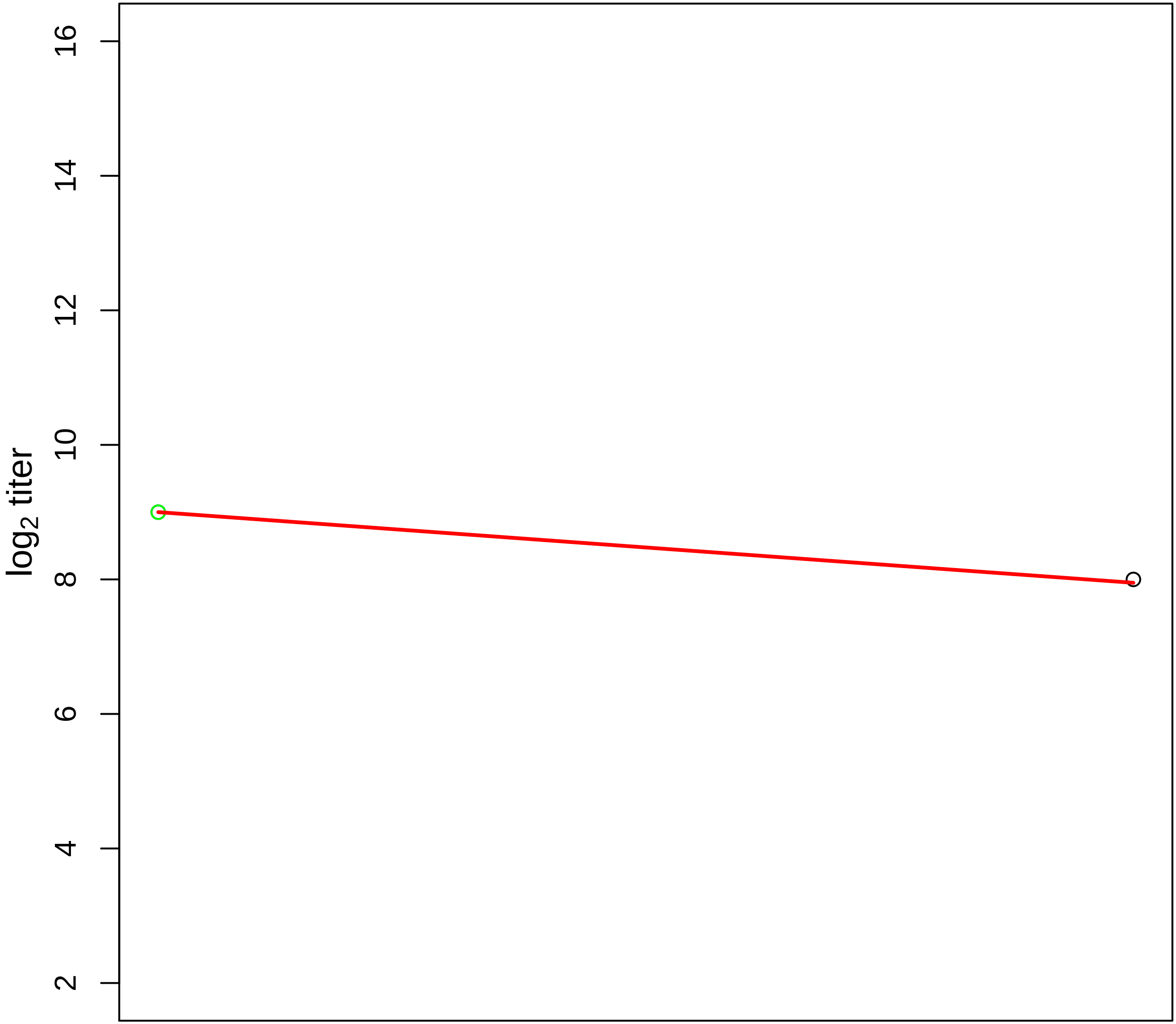

time in years from first donation of donor 13
mean absolute errors = 0.05 , mean squared errors = 0.003

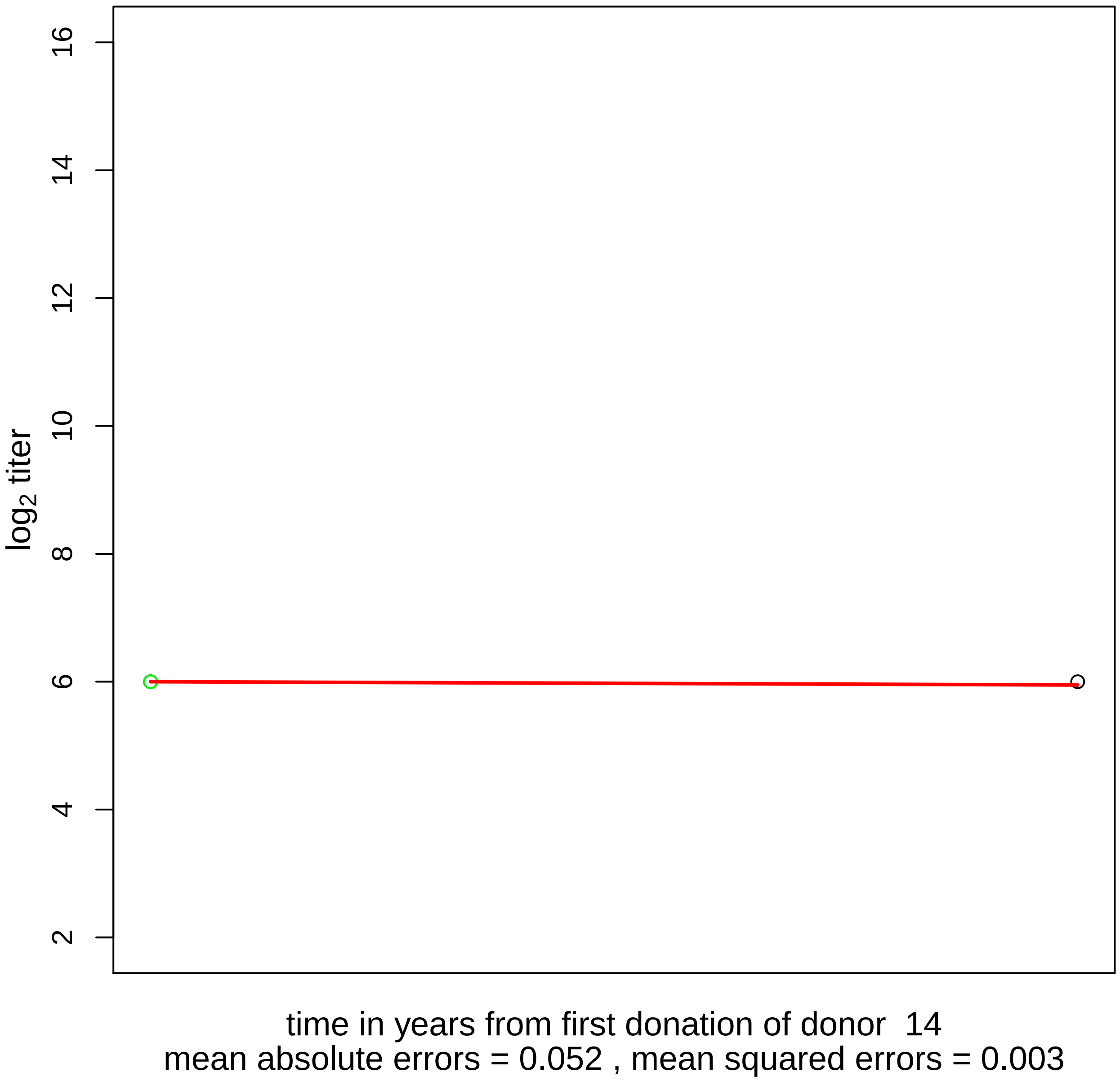
log2 titer
16
14
12
10
8
6
4
2
time in years from first donation of donor 14
mean absolute errors = 0.052 , mean squared errors = 0.003

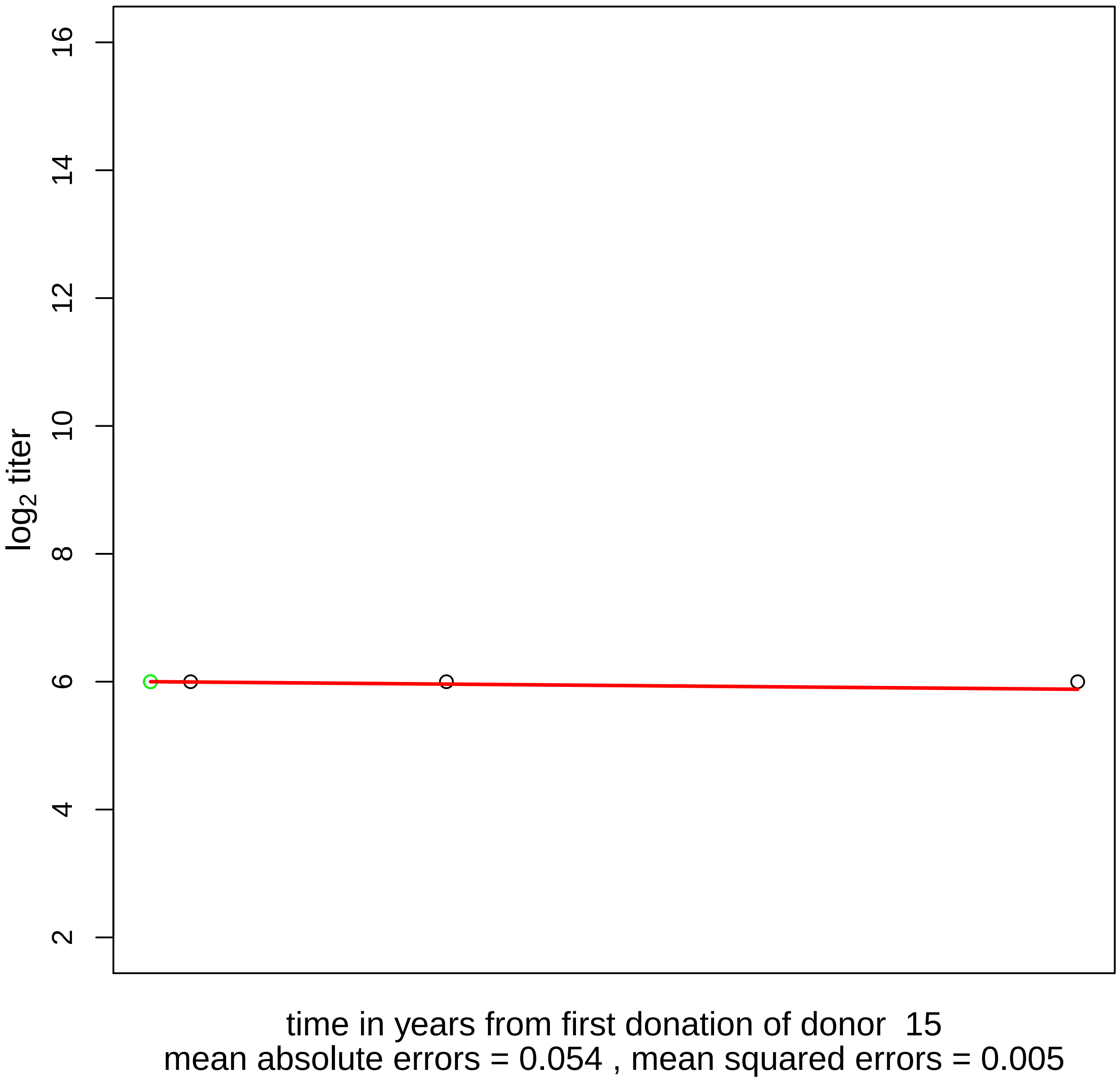

log2 titer
16
14
12
10
8
6
4
2
time in years from first donation of donor 15
mean absolute errors = 0.054 , mean squared errors = 0.005

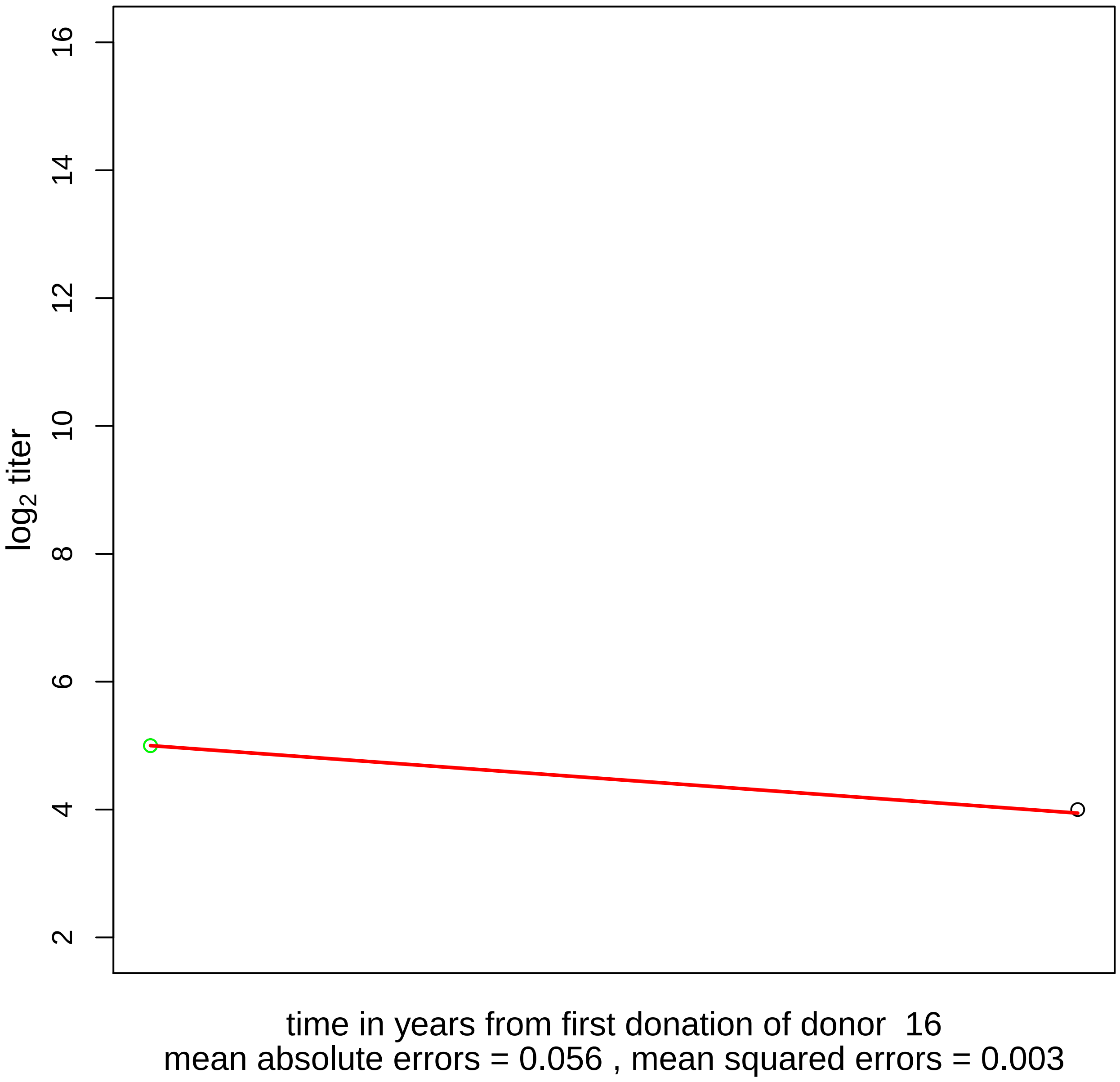
log2 titer
16
14
12
10
8
6
4
2
time in years from first donation of donor 16
mean absolute errors = 0.056 , mean squared errors = 0.003

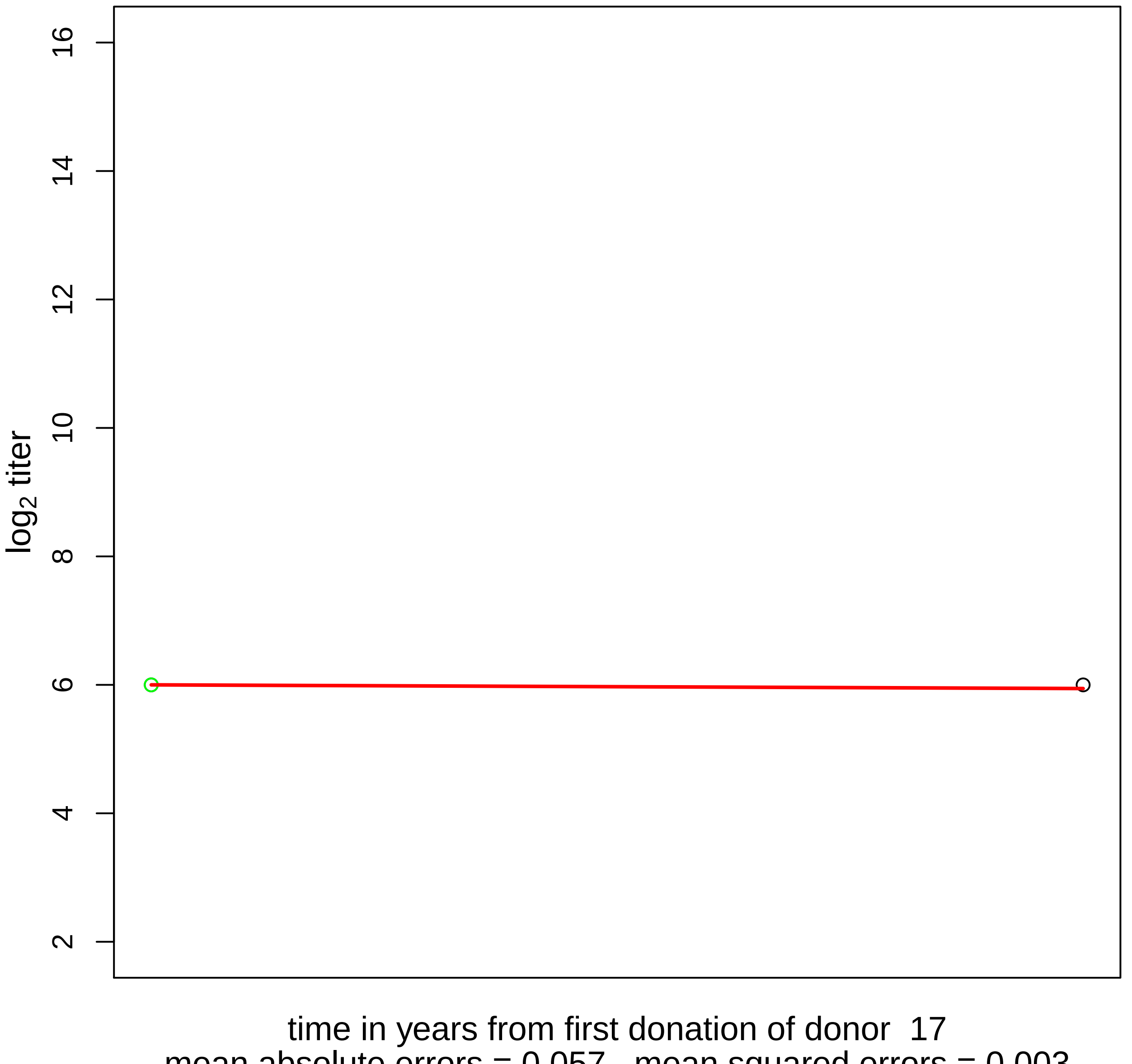
log2 titer
2
4
6
8
10
12
14
16
time in years from first donation of donor 17
mean absolute errors = 0.057 , mean squared errors = 0.003

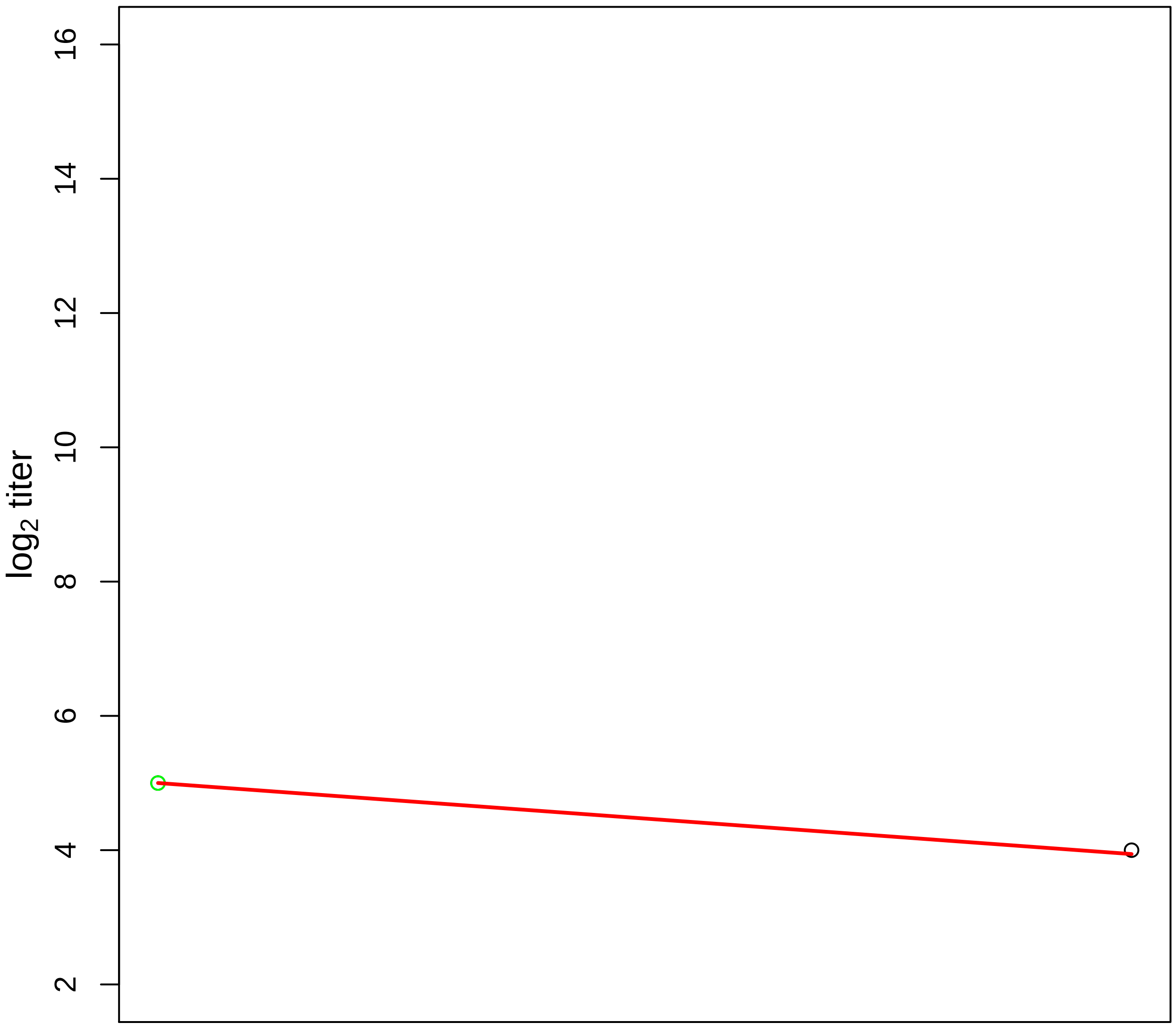

time in years from first donation of donor 18
mean absolute errors = 0.058 , mean squared errors = 0.003

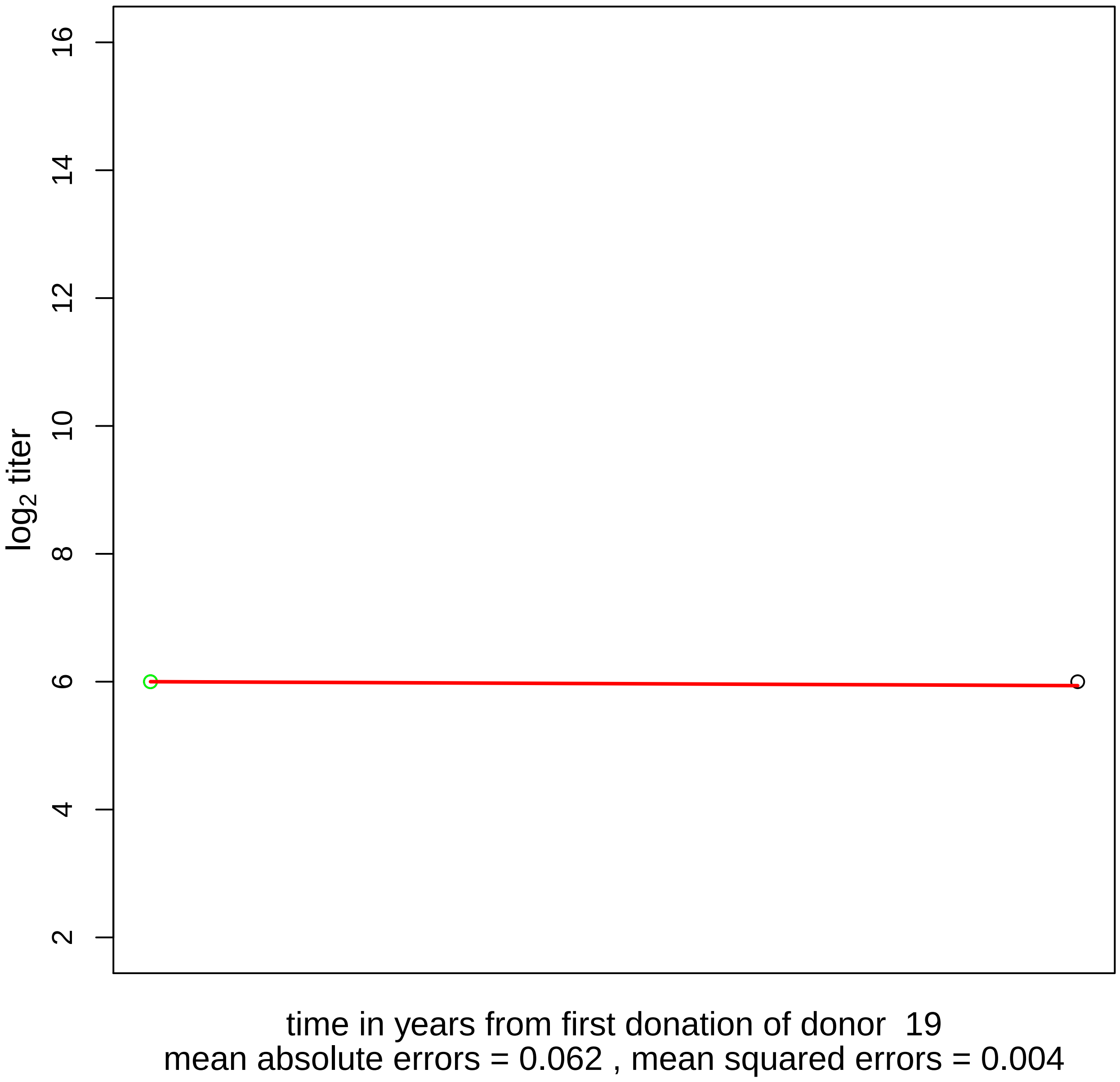
log2 titer
16
14
12
10
8
6
4
2
time in years from first donation of donor 19
mean absolute errors = 0.062 , mean squared errors = 0.004

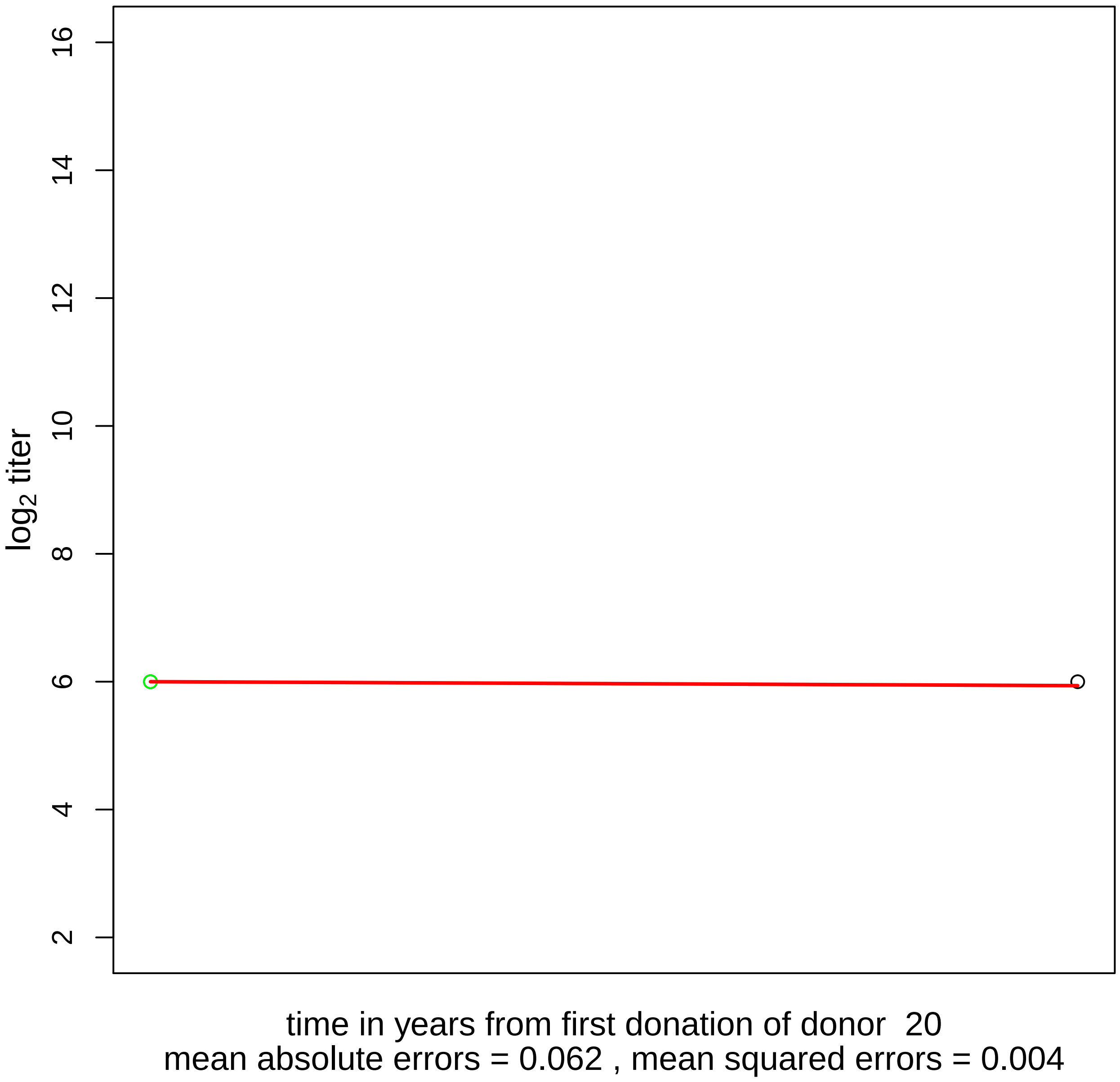

16
14
12
10
8
6
4
2
log2 titer
time in years from first donation of donor 20
mean absolute errors = 0.062 , mean squared errors = 0.004

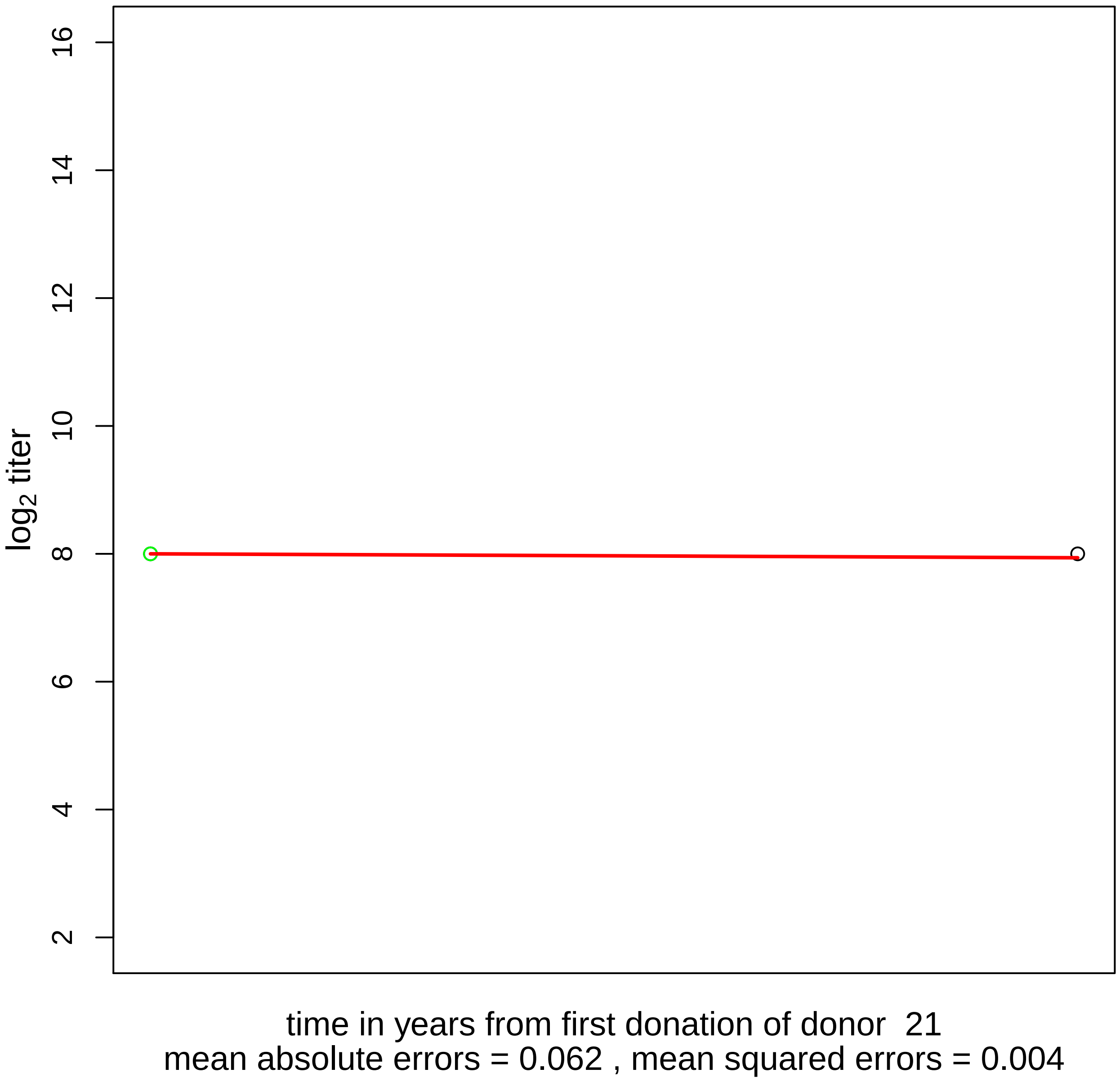

log2 titer
2
4
6
8
10
12
14
16
time in years from first donation of donor 21
mean absolute errors = 0.062 , mean squared errors = 0.004

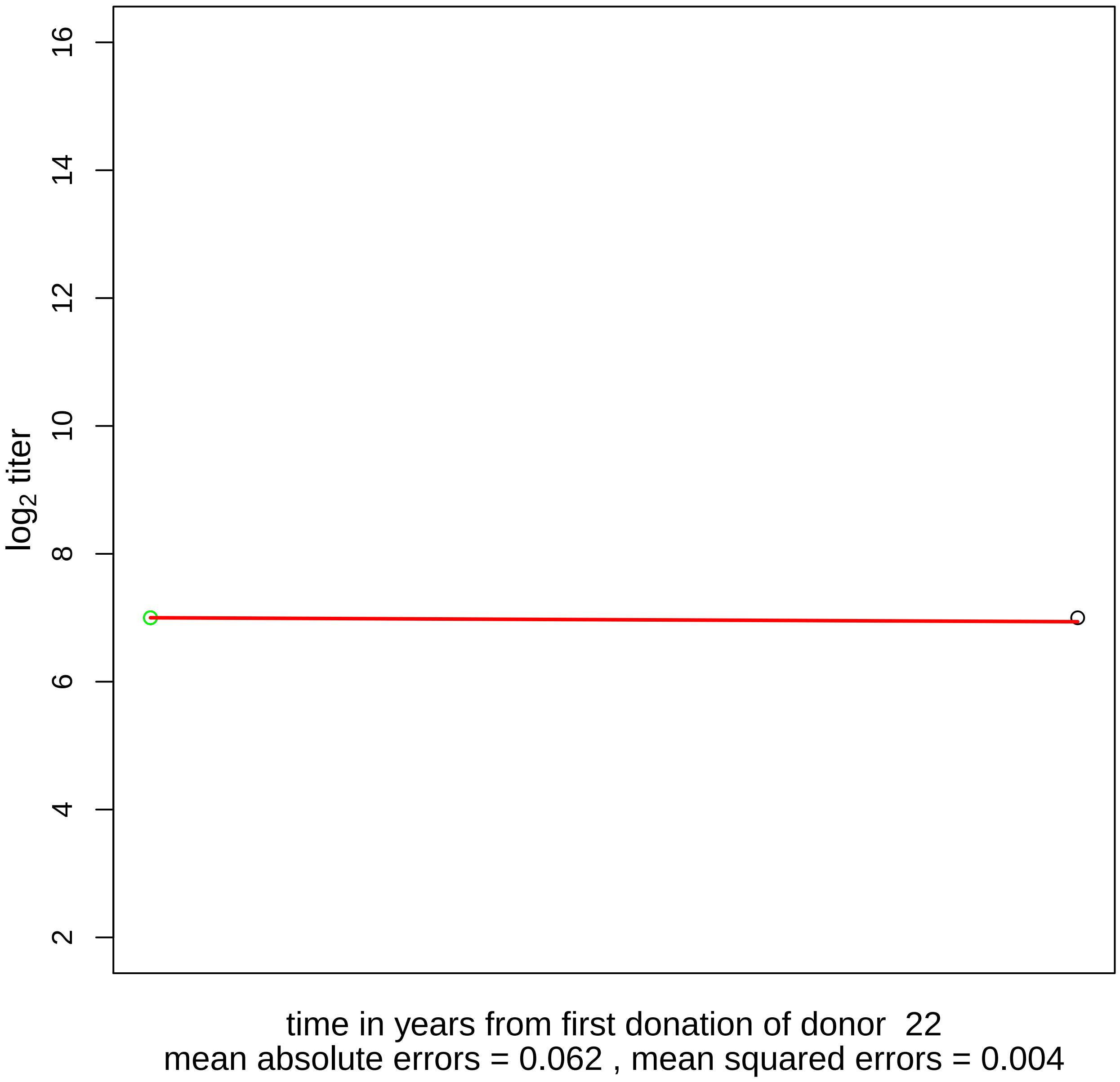

16
14
12
10
8
6
4
2
log2 titer
time in years from first donation of donor 22
mean absolute errors = 0.062 , mean squared errors = 0.004

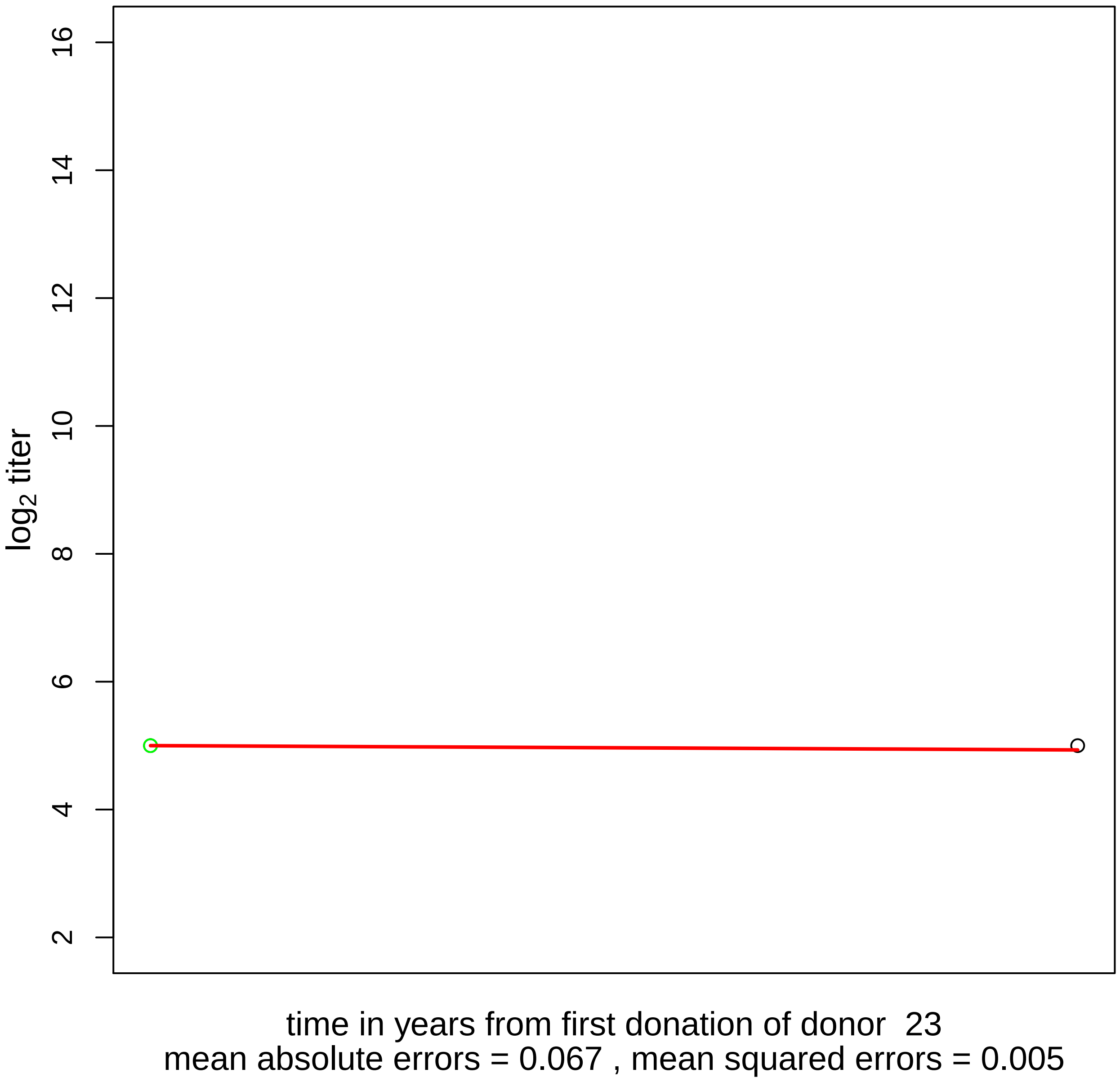
log2 titer
2
4
6
8
10
12
14
16
time in years from first donation of donor 23
mean absolute errors = 0.067 , mean squared errors = 0.005

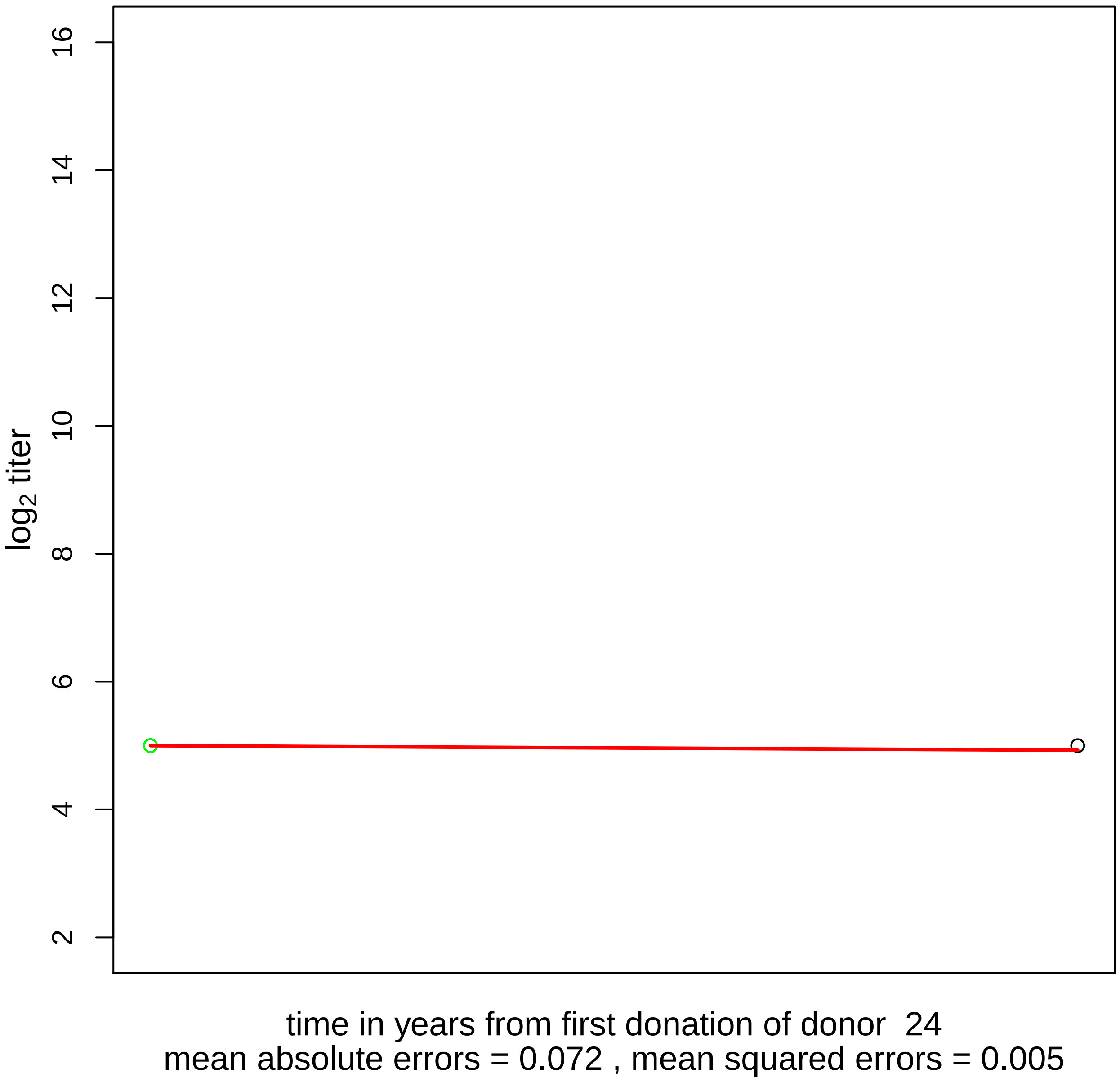

log2 titer
16
14
12
10
8
6
4
2
time in years from first donation of donor 24
mean absolute errors = 0.072 , mean squared errors = 0.005

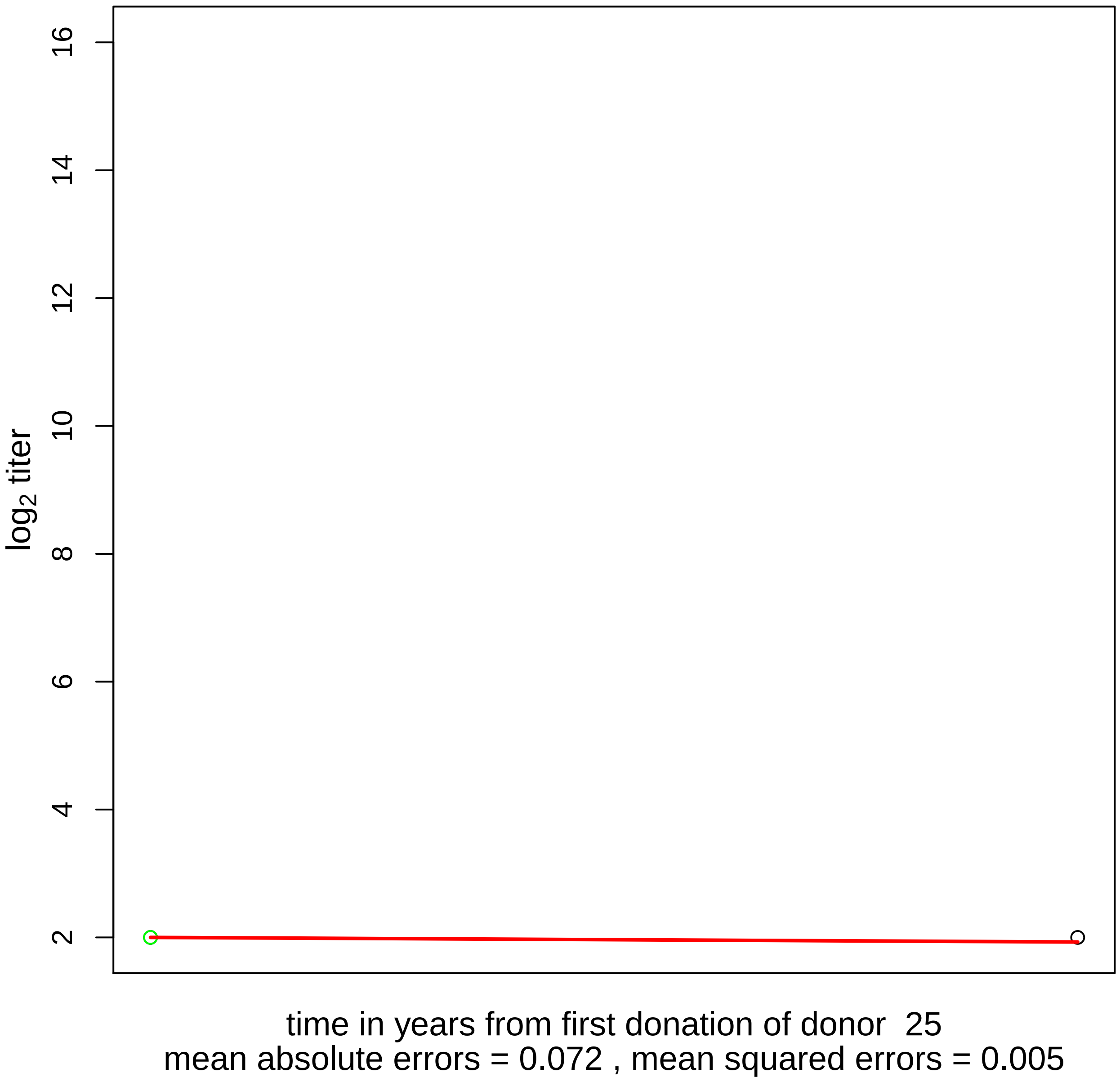

16
14
12
10
8
6
4
2
log2 titer
time in years from first donation of donor 25
mean absolute errors = 0.072 , mean squared errors = 0.005

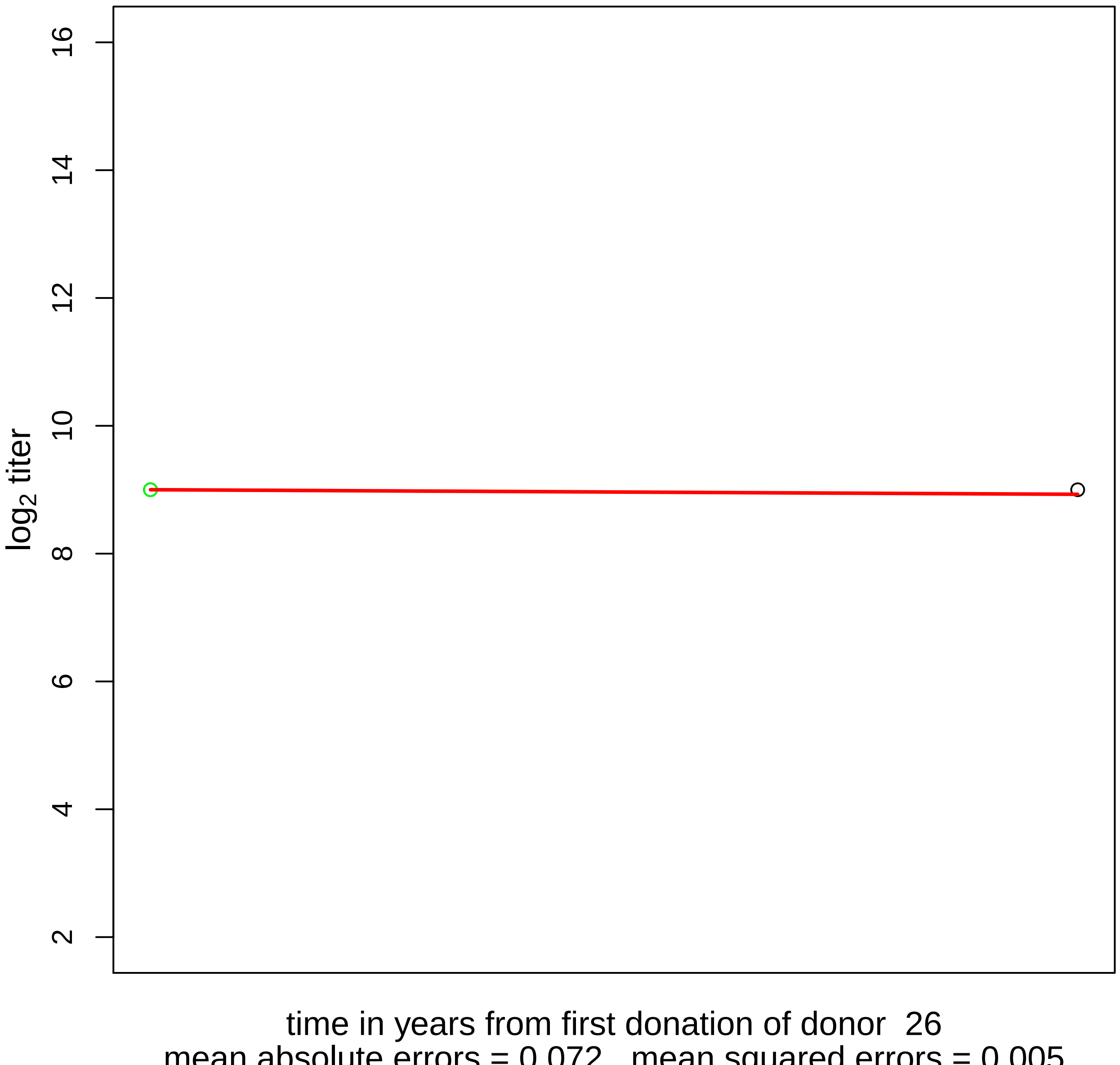

16
14
12
10
8
6
4
2
log2 titer
time in years from first donation of donor 26
mean absolute errors = 0.072 , mean squared errors = 0.005

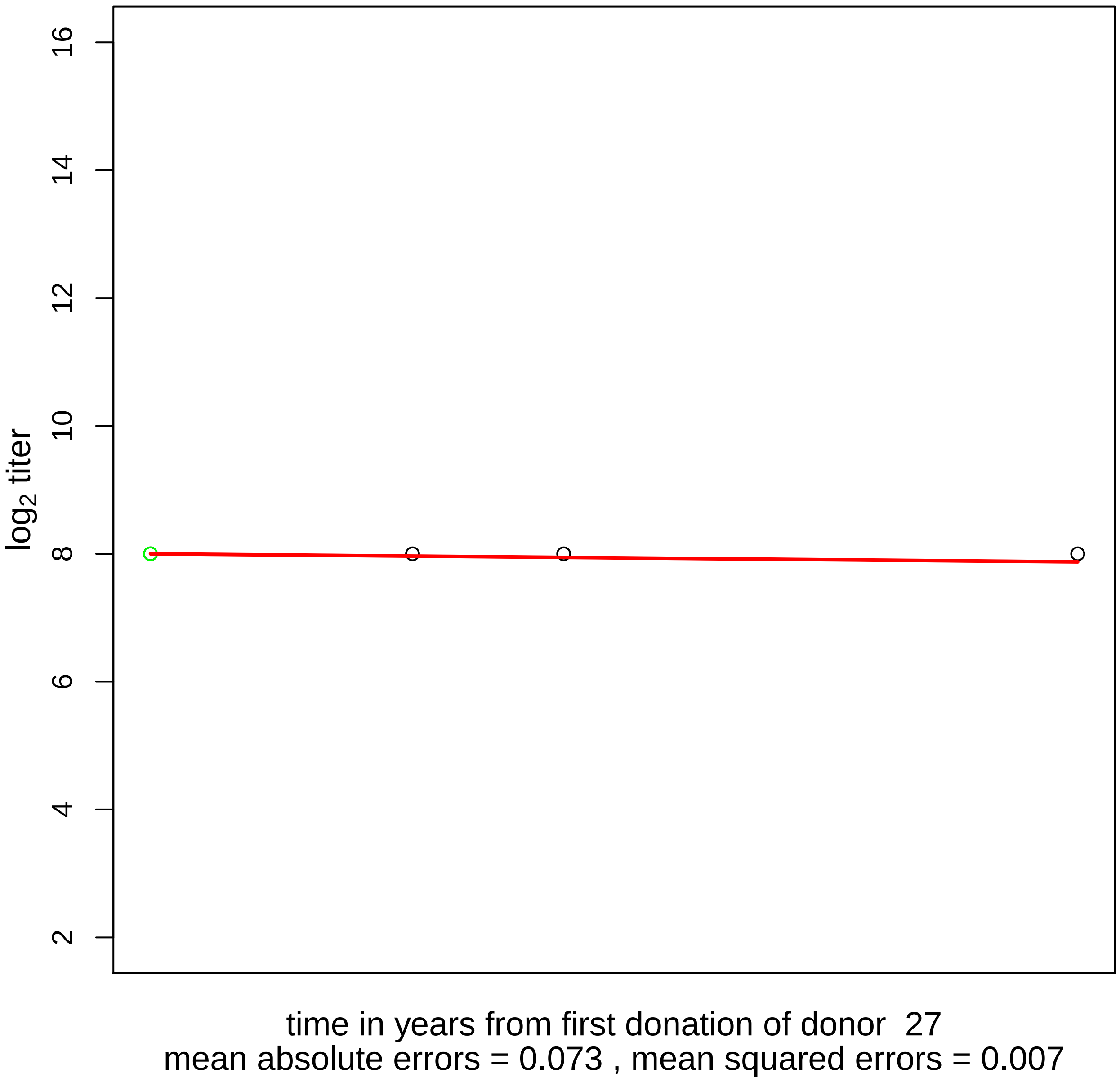

log2 titer
2
4
6
8
10
12
14
16
time in years from first donation of donor 27
mean absolute errors = 0.073 , mean squared errors = 0.007

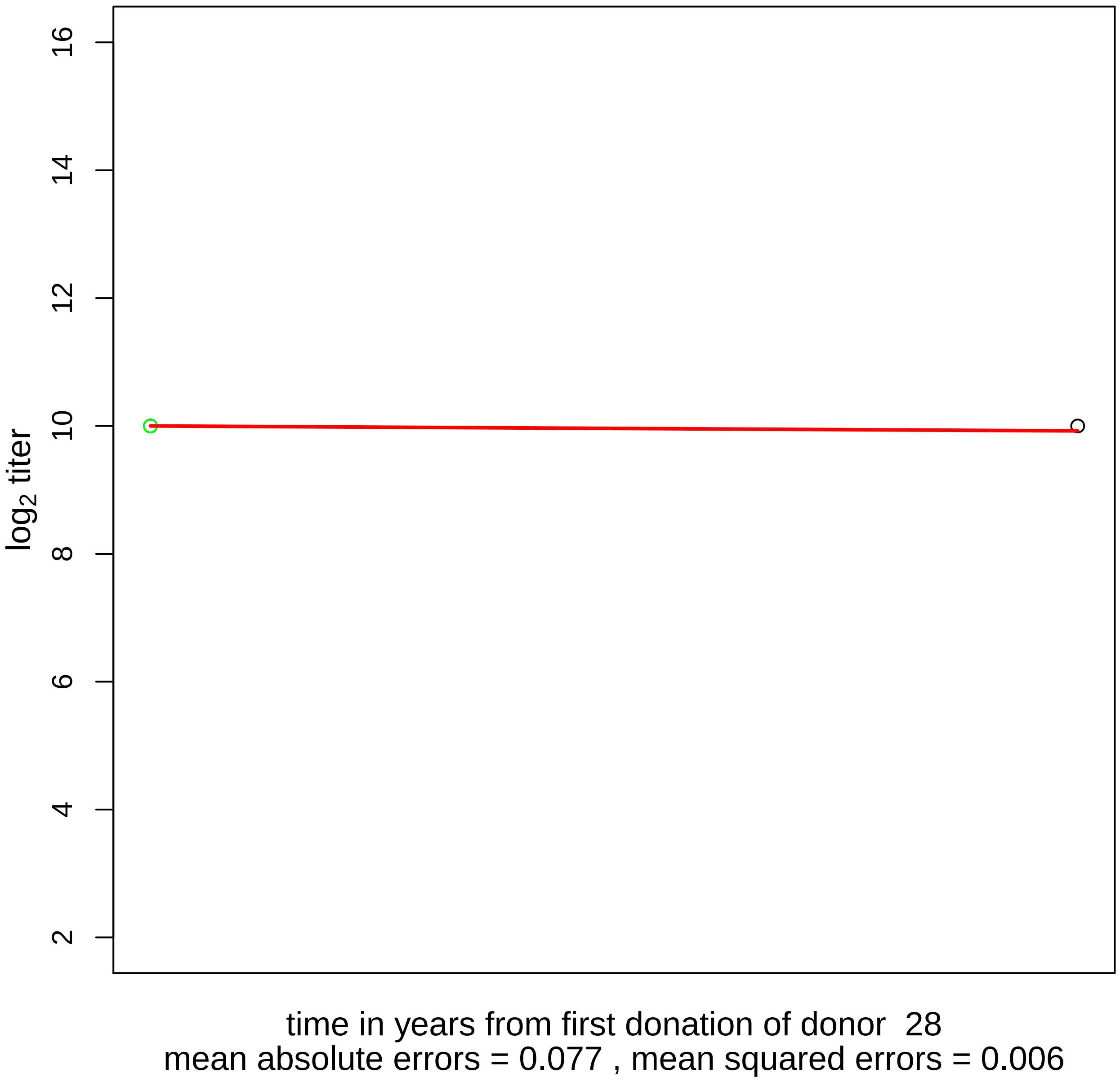

log2 titer
16
14
12
10
8
6
4
2
time in years from first donation of donor 28
mean absolute errors = 0.077 , mean squared errors = 0.006

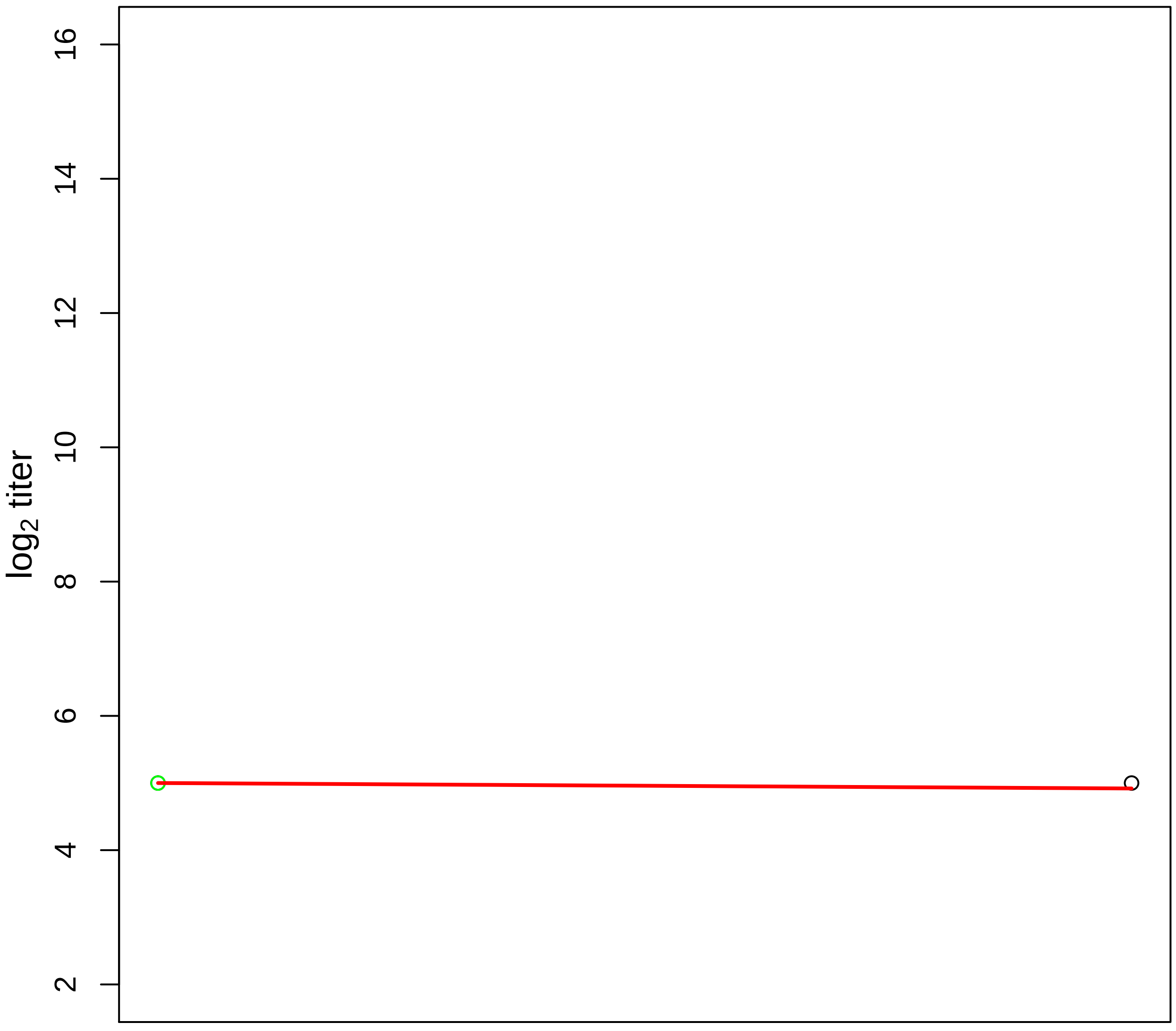

time in years from first donation of donor 29
mean absolute errors = 0.081 , mean squared errors = 0.007

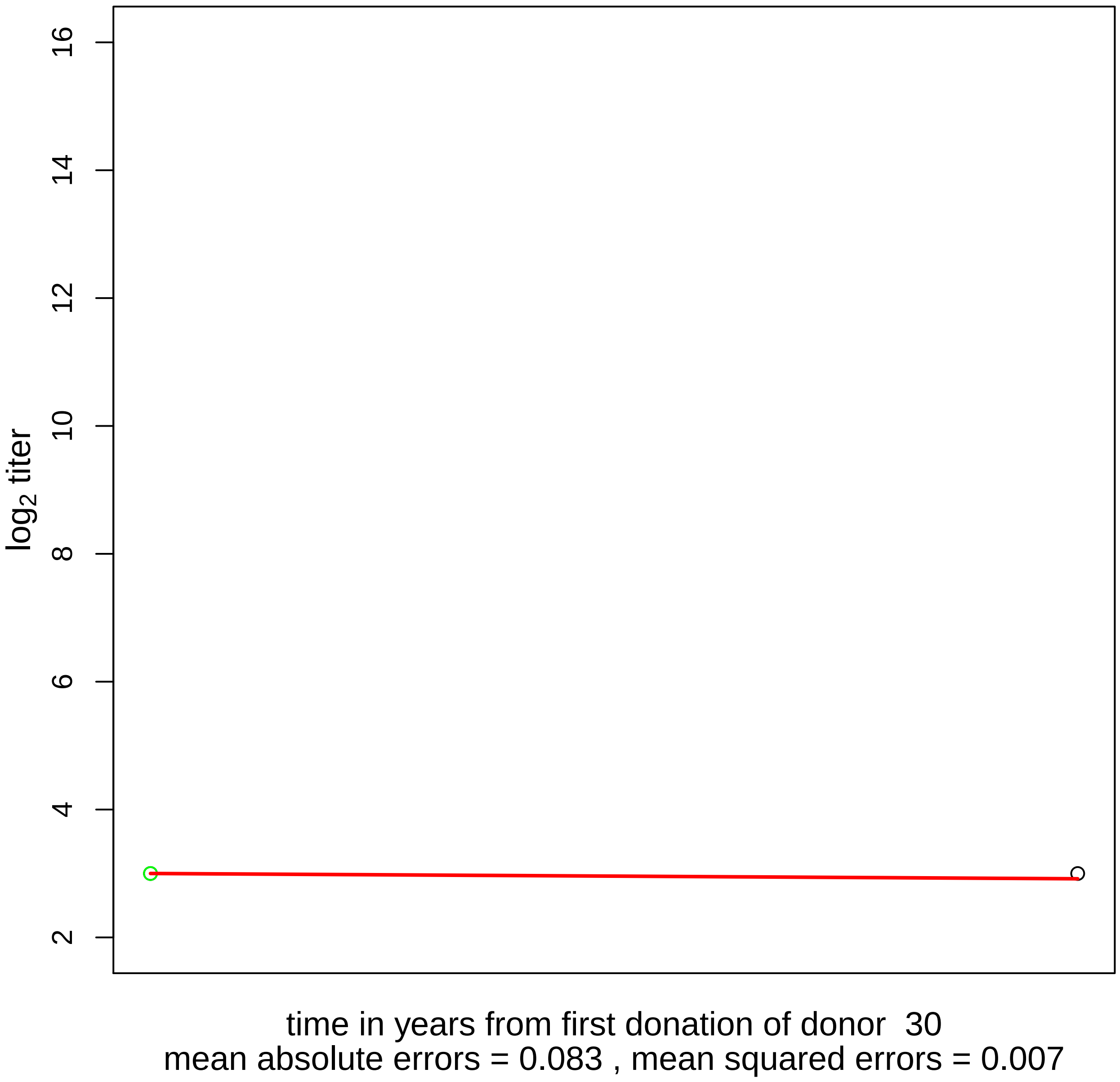

log2 titer
16
14
12
10
8
6
4
2
time in years from first donation of donor 30
mean absolute errors = 0.083 , mean squared errors = 0.007

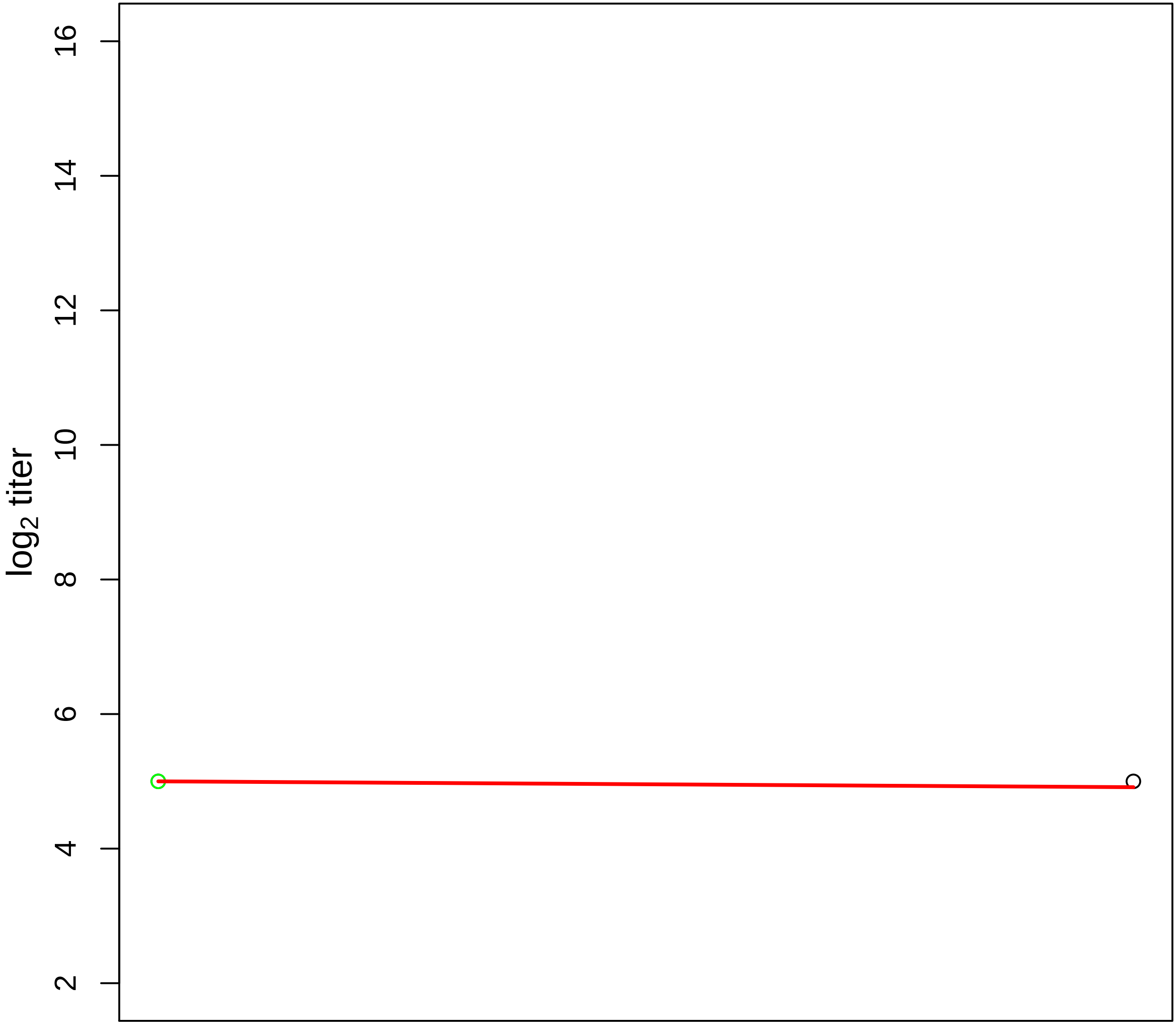

time in years from first donation of donor 31
mean absolute errors = 0.087 , mean squared errors = 0.008

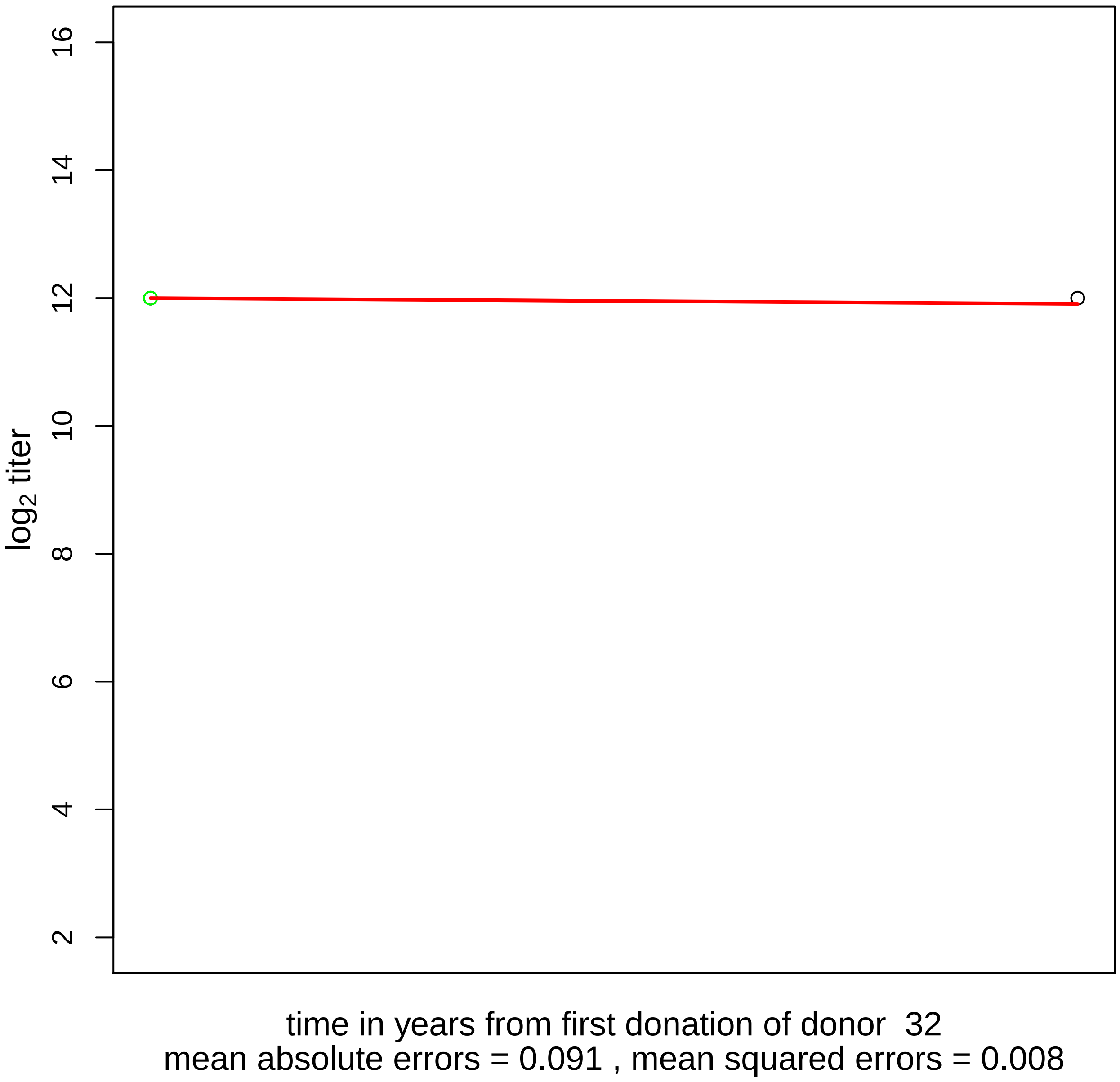

log2 titer
16
14
12
10
8
6
4
2
time in years from first donation of donor 32
mean absolute errors = 0.091 , mean squared errors = 0.008

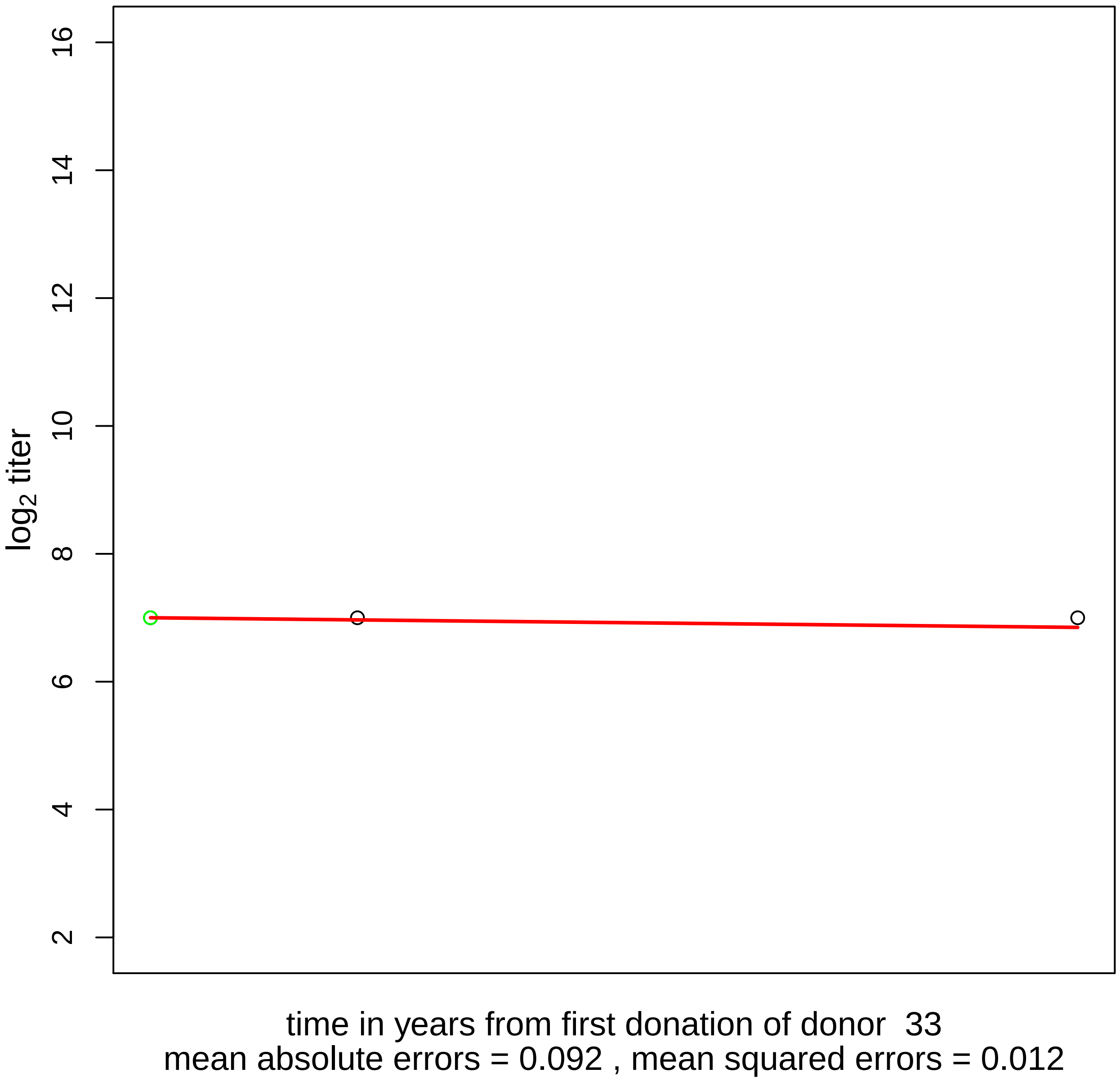

log2 titer
2
4
6
8
10
12
14
16
time in years from first donation of donor 33
mean absolute errors = 0.092 , mean squared errors = 0.012

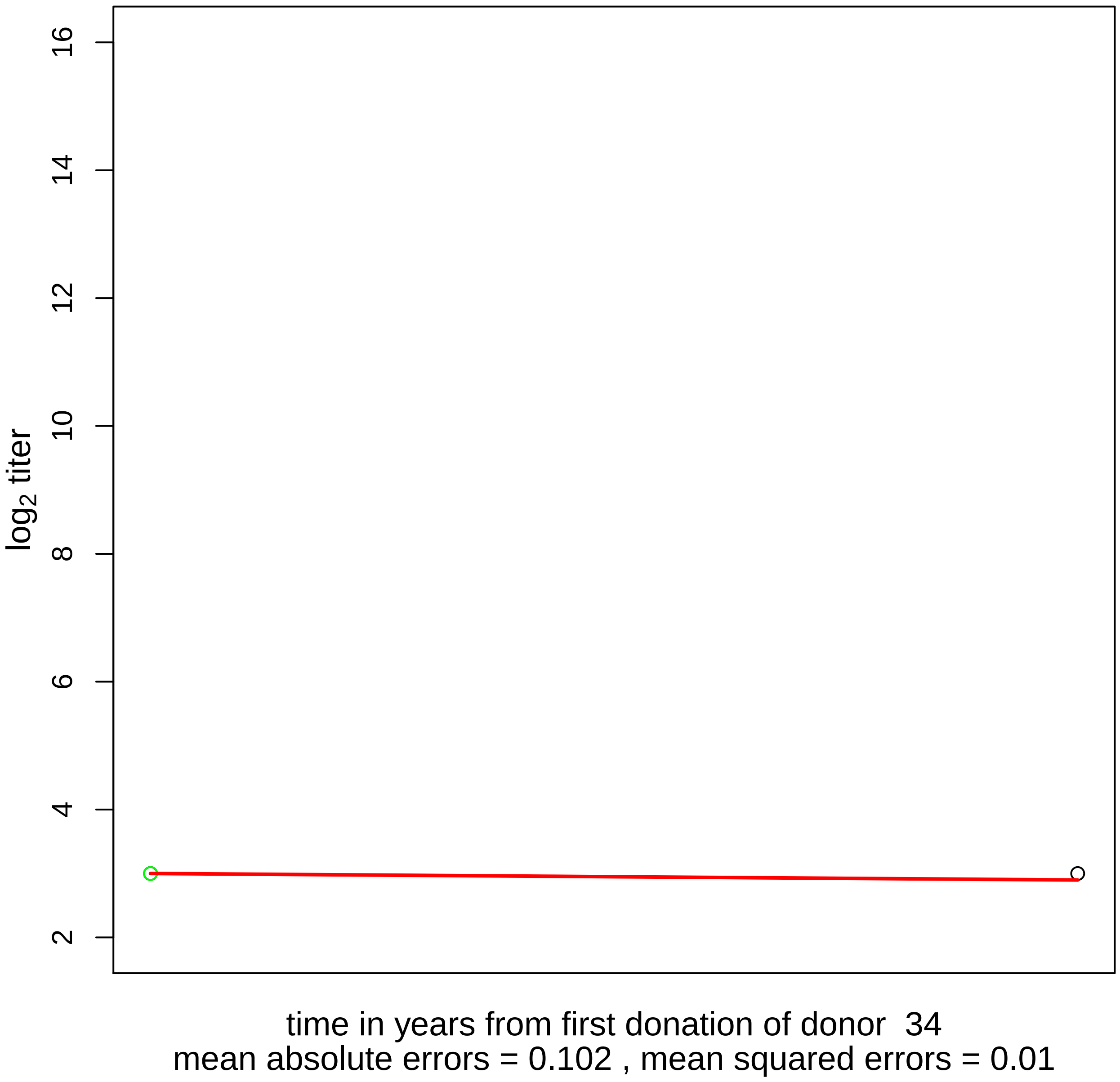
log2 titer
16
14
12
10
8
6
4
2
time in years from first donation of donor 34
mean absolute errors = 0.102 , mean squared errors = 0.01

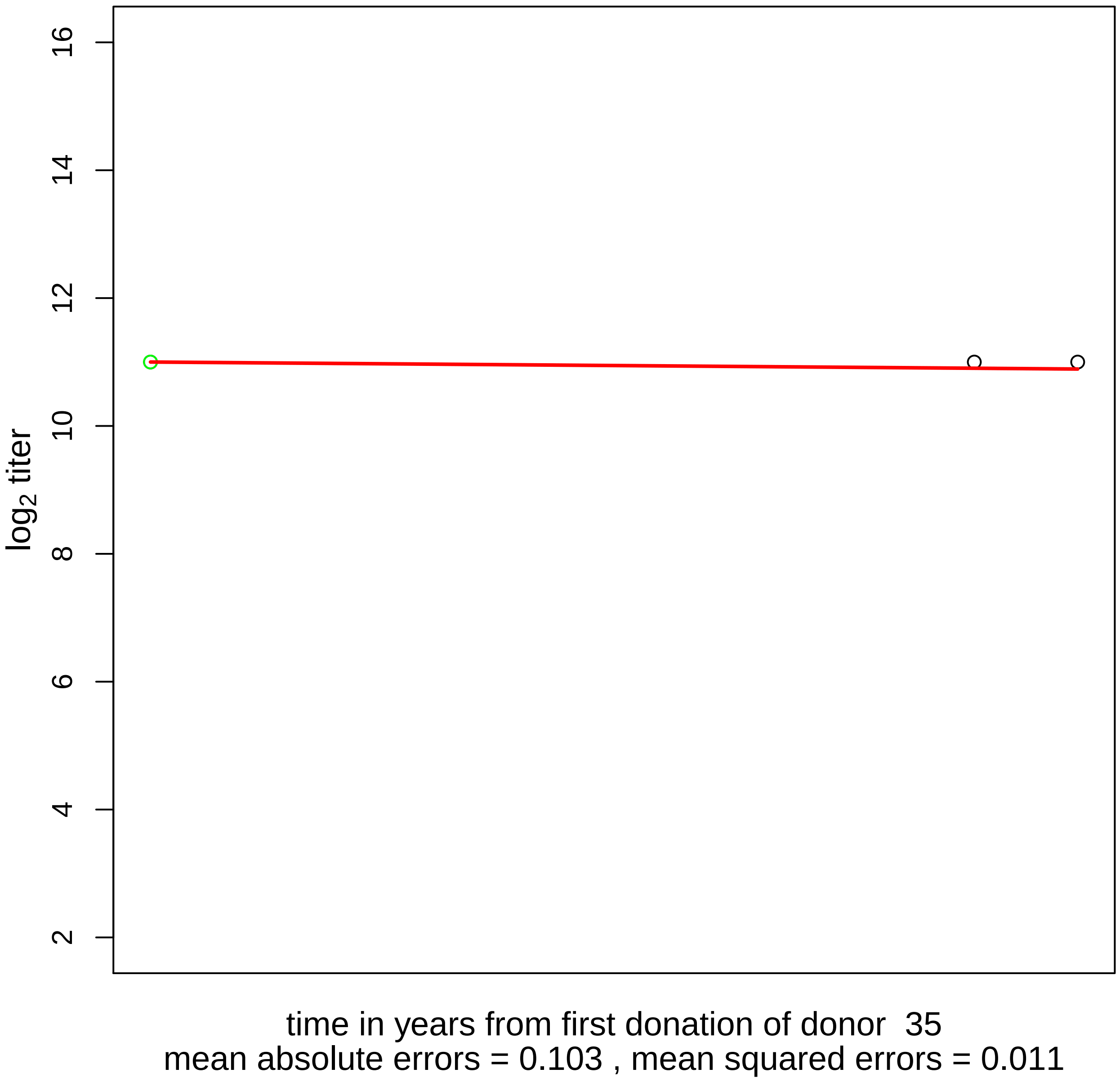

2
4
6
8
10
12
14
16
log2 titer
time in years from first donation of donor 35
mean absolute errors = 0.103 , mean squared errors = 0.011

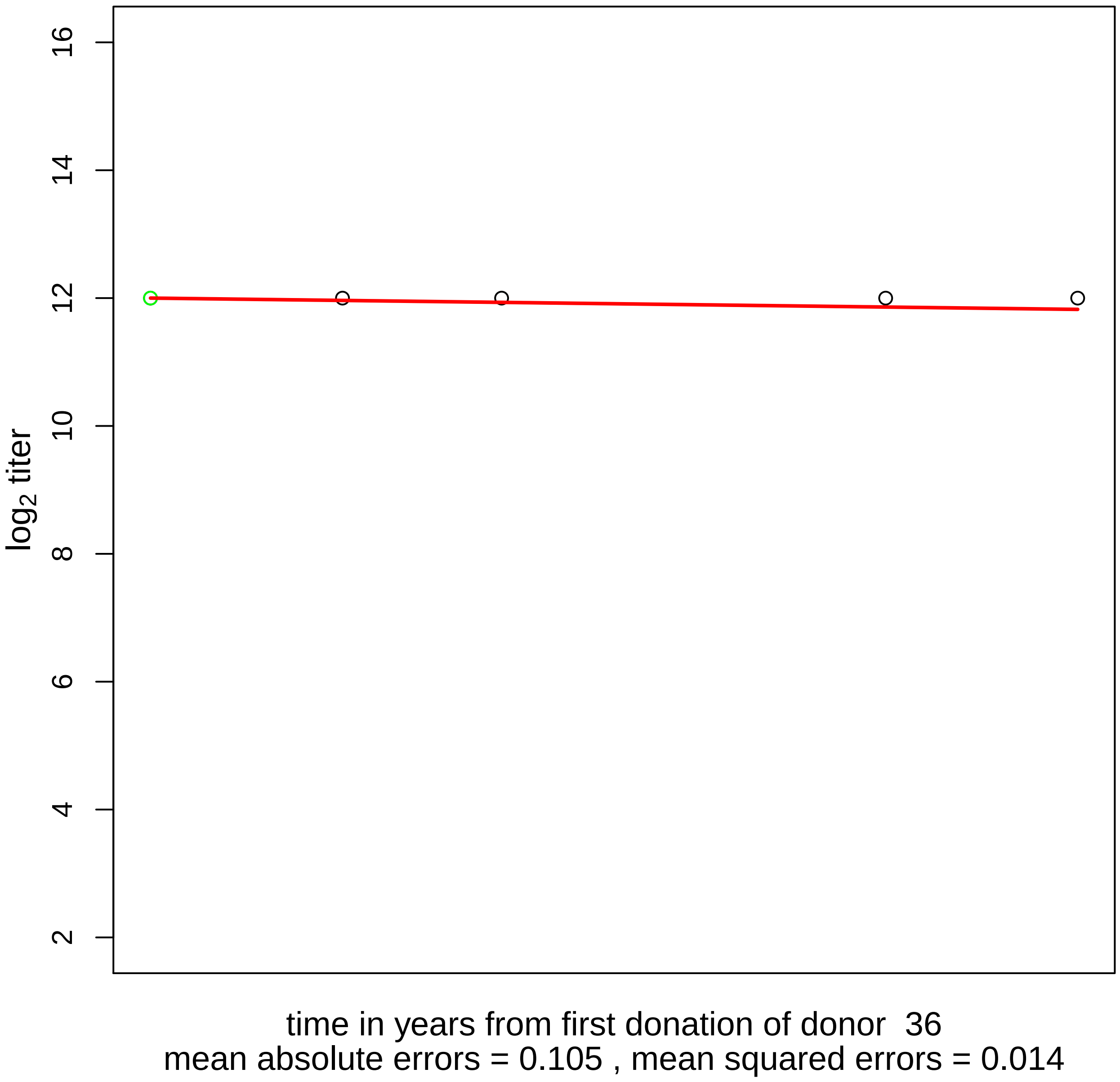

16
14
12
10
8
6
4
2
log2 titer
time in years from first donation of donor 36
mean absolute errors = 0.105 , mean squared errors = 0.014

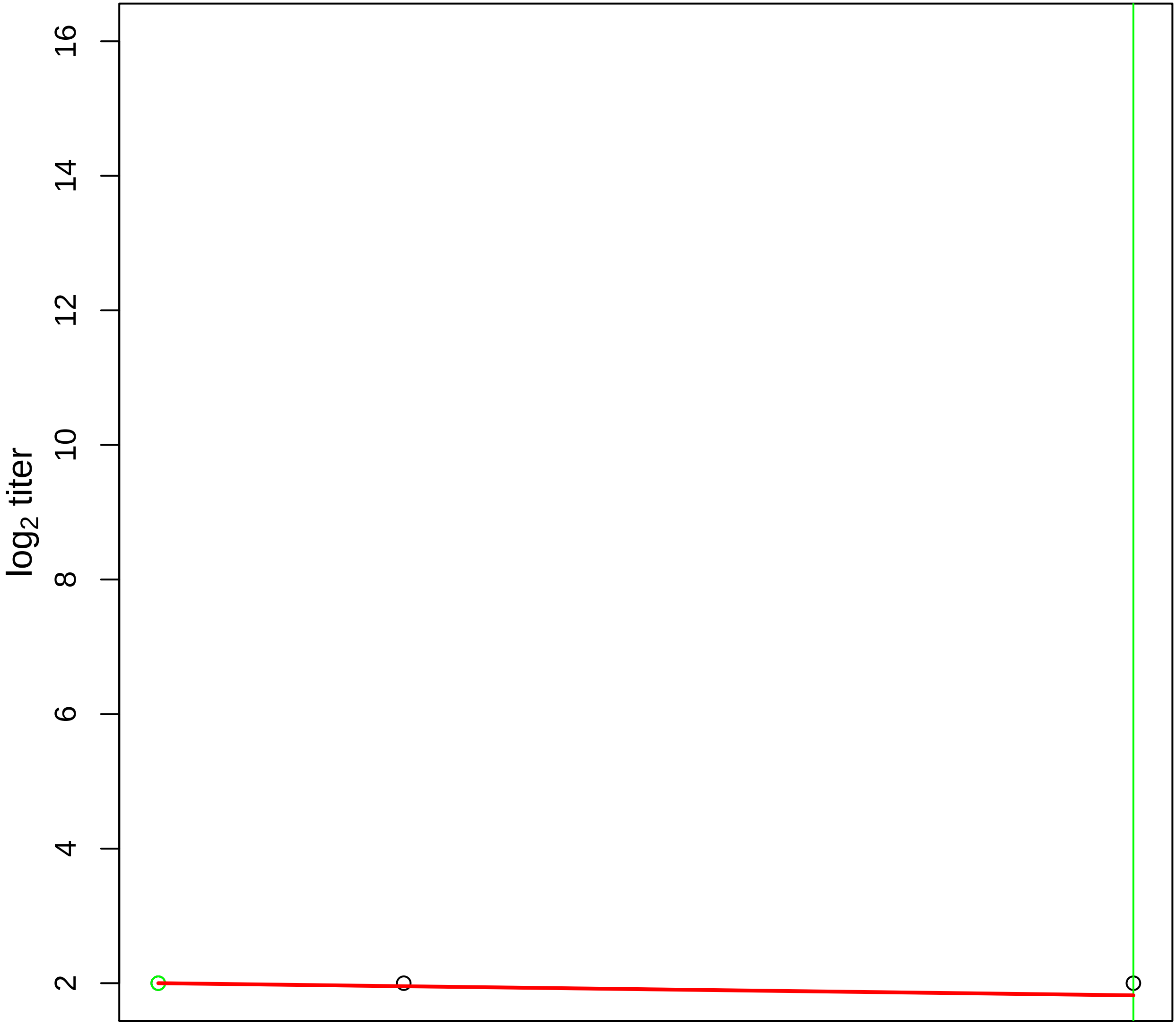

time in years from first donation of donor 37
mean absolute errors = 0.113 , mean squared errors = 0.017

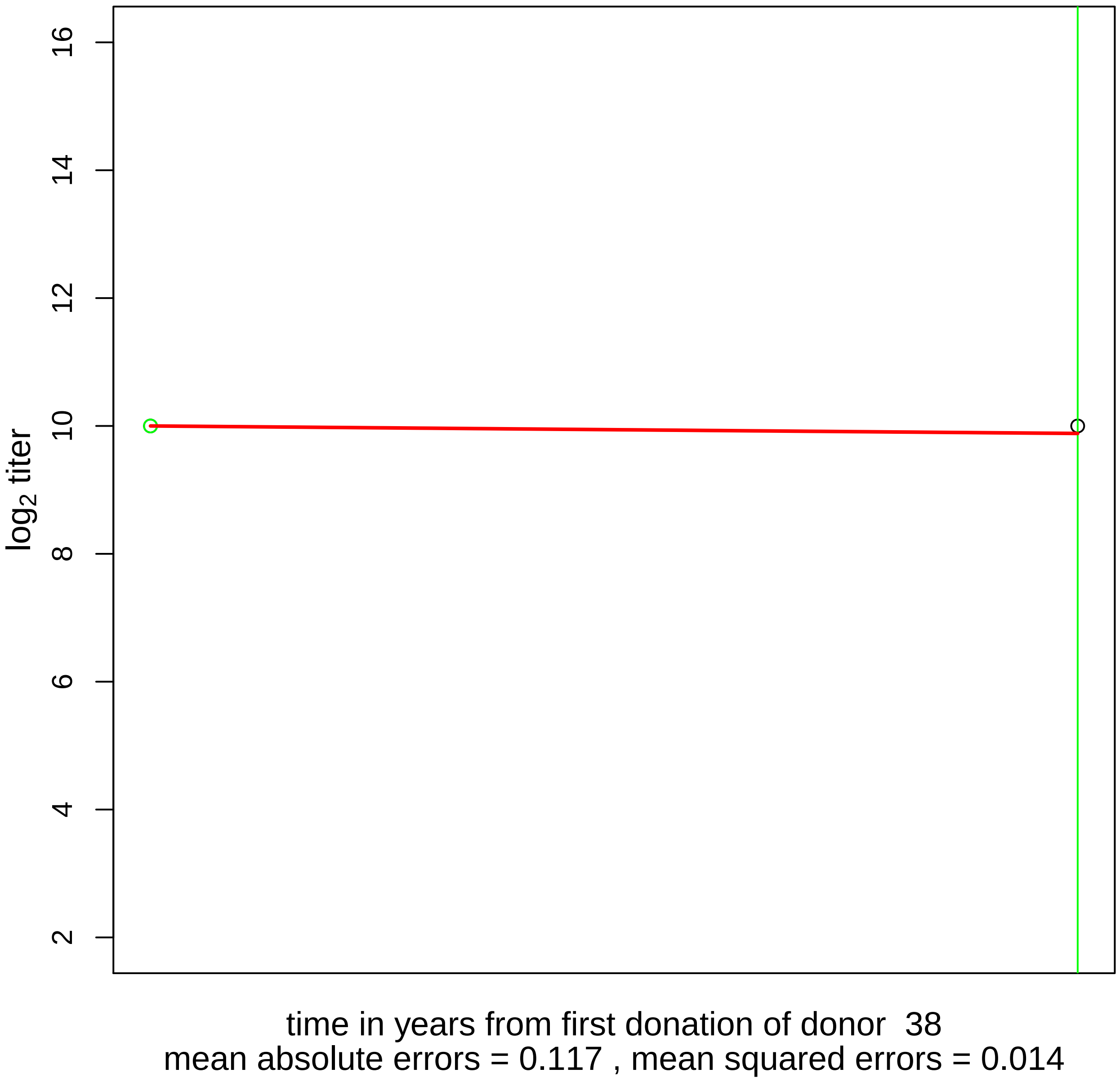

16
14
12
10
8
6
4
2
$\log_2$ titer
time in years from first donation of donor 38
mean absolute errors = 0.117 , mean squared errors = 0.014

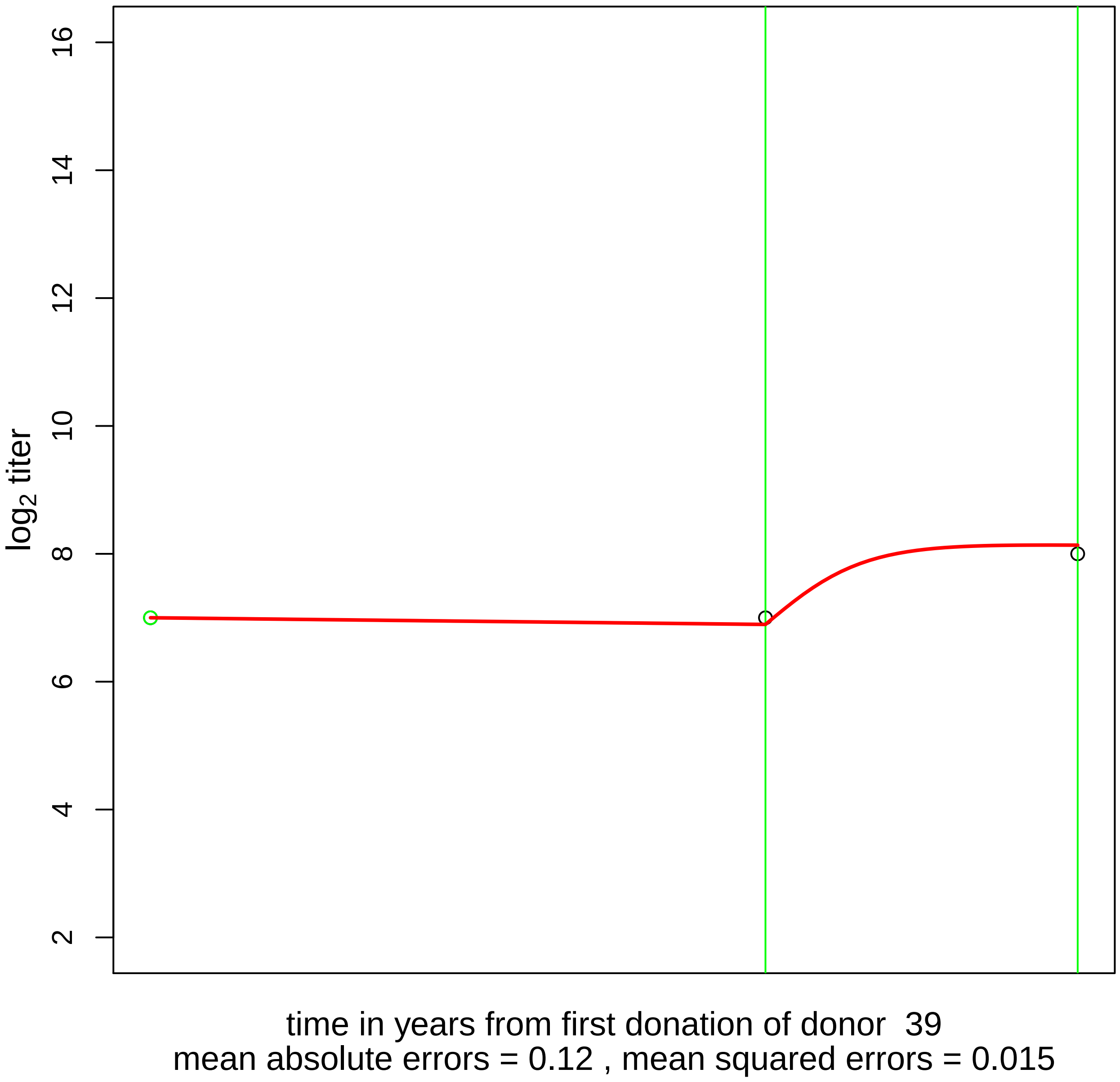
log2 titer
16
14
12
10
8
6
4
2
time in years from first donation of donor 39
mean absolute errors = 0.12 , mean squared errors = 0.015

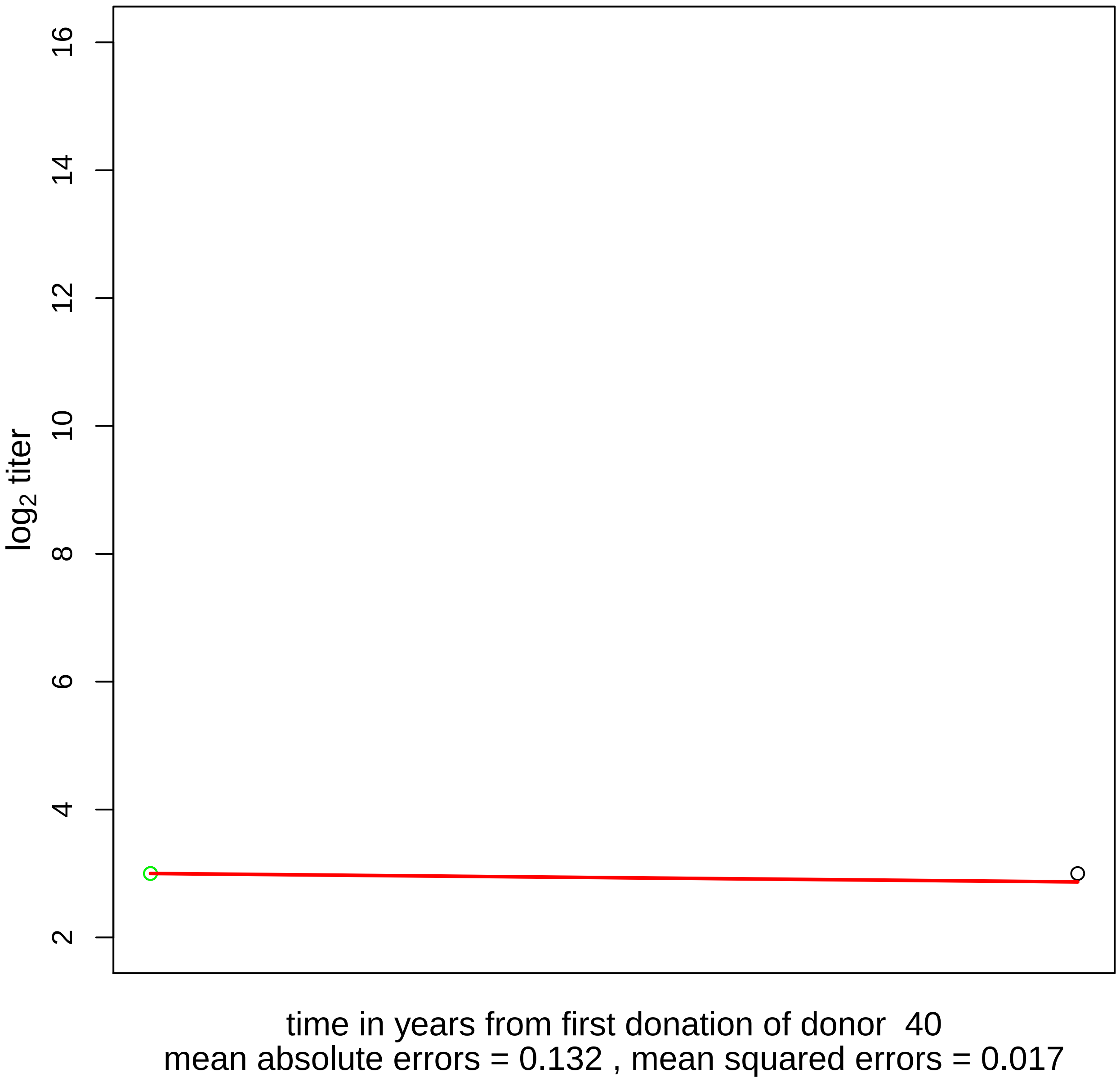
log2 titer
16
14
12
10
8
6
4
2
time in years from first donation of donor 40
mean absolute errors = 0.132 , mean squared errors = 0.017

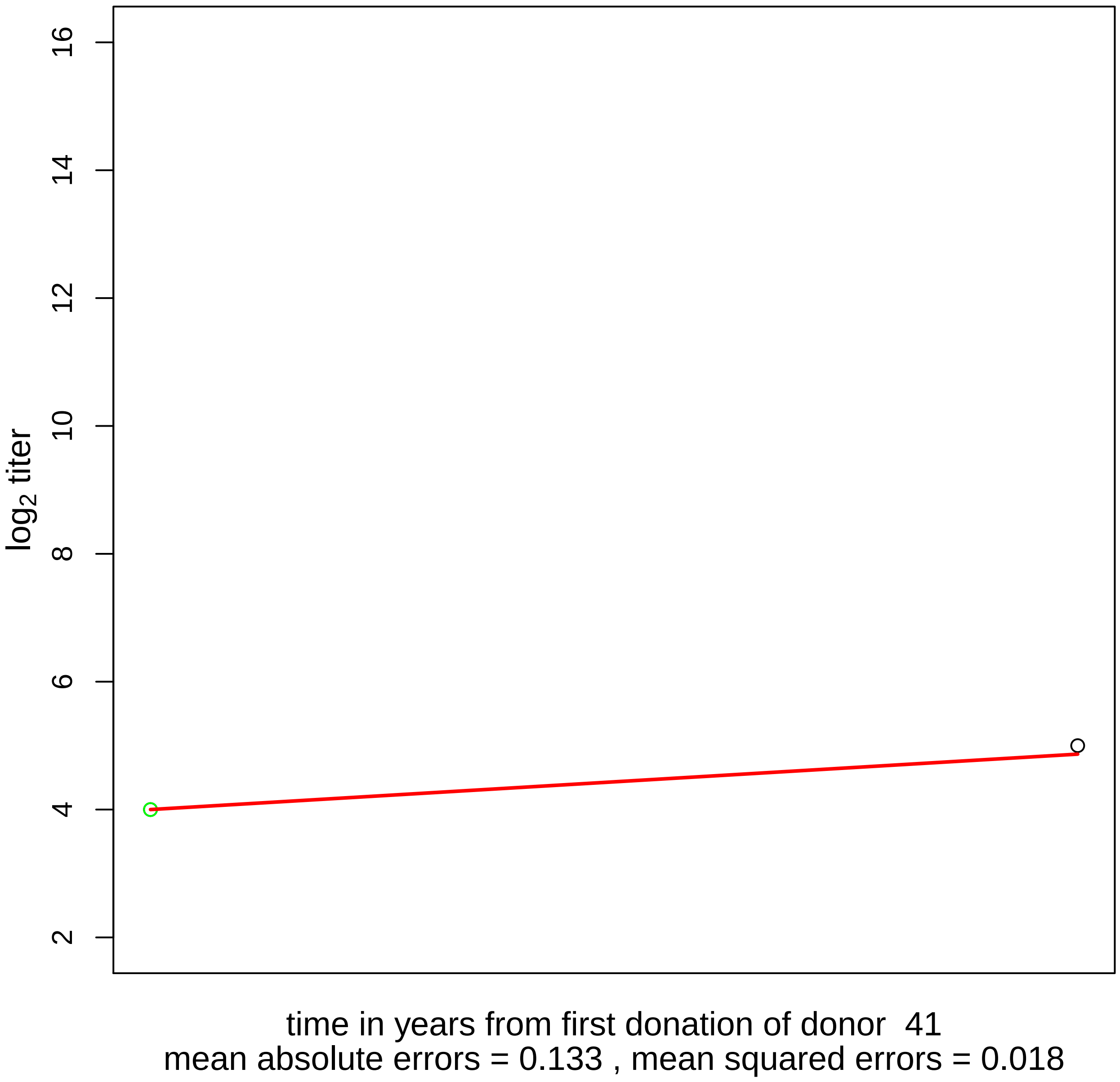
16
14
12
10
8
6
4
2
log2 titer
time in years from first donation of donor 41
mean absolute errors = 0.133 , mean squared errors = 0.018

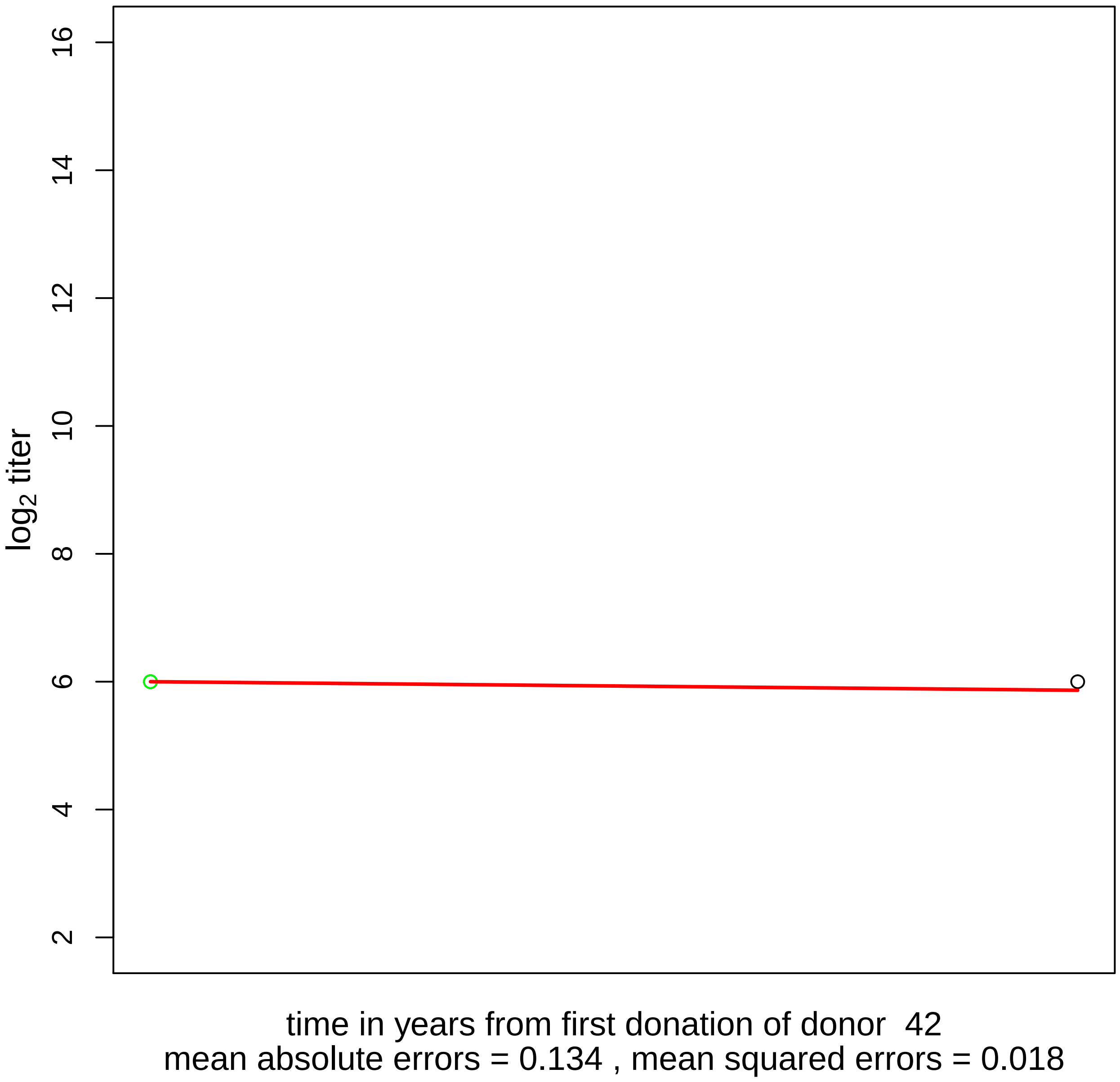

log2 titer
2
4
6
8
10
12
14
16
time in years from first donation of donor 42
mean absolute errors = 0.134 , mean squared errors = 0.018

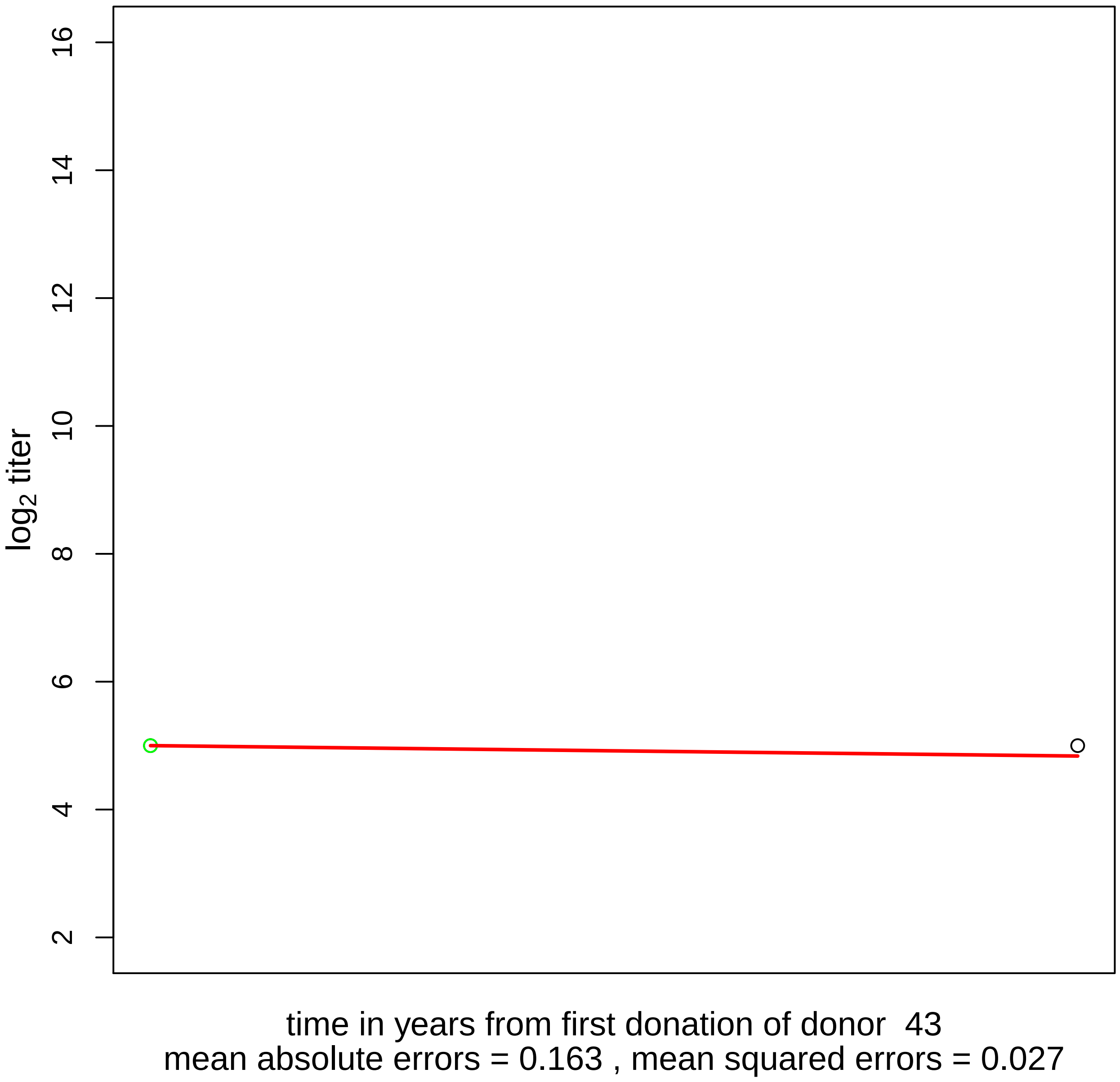

log2 titer
16
14
12
10
8
6
4
2
time in years from first donation of donor 43
mean absolute errors = 0.163 , mean squared errors = 0.027

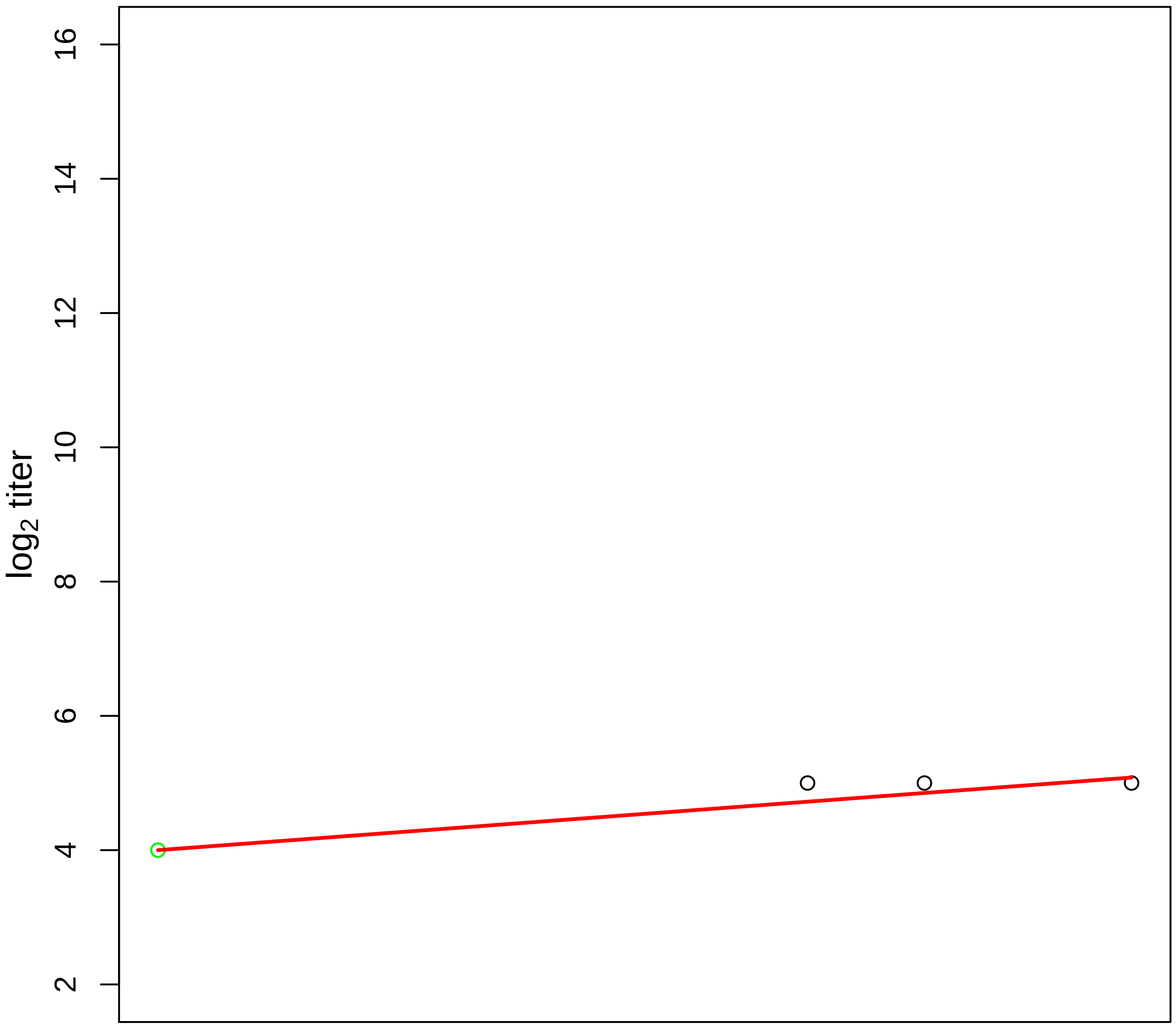

time in years from first donation of donor 44
mean absolute errors = 0.169 , mean squared errors = 0.035

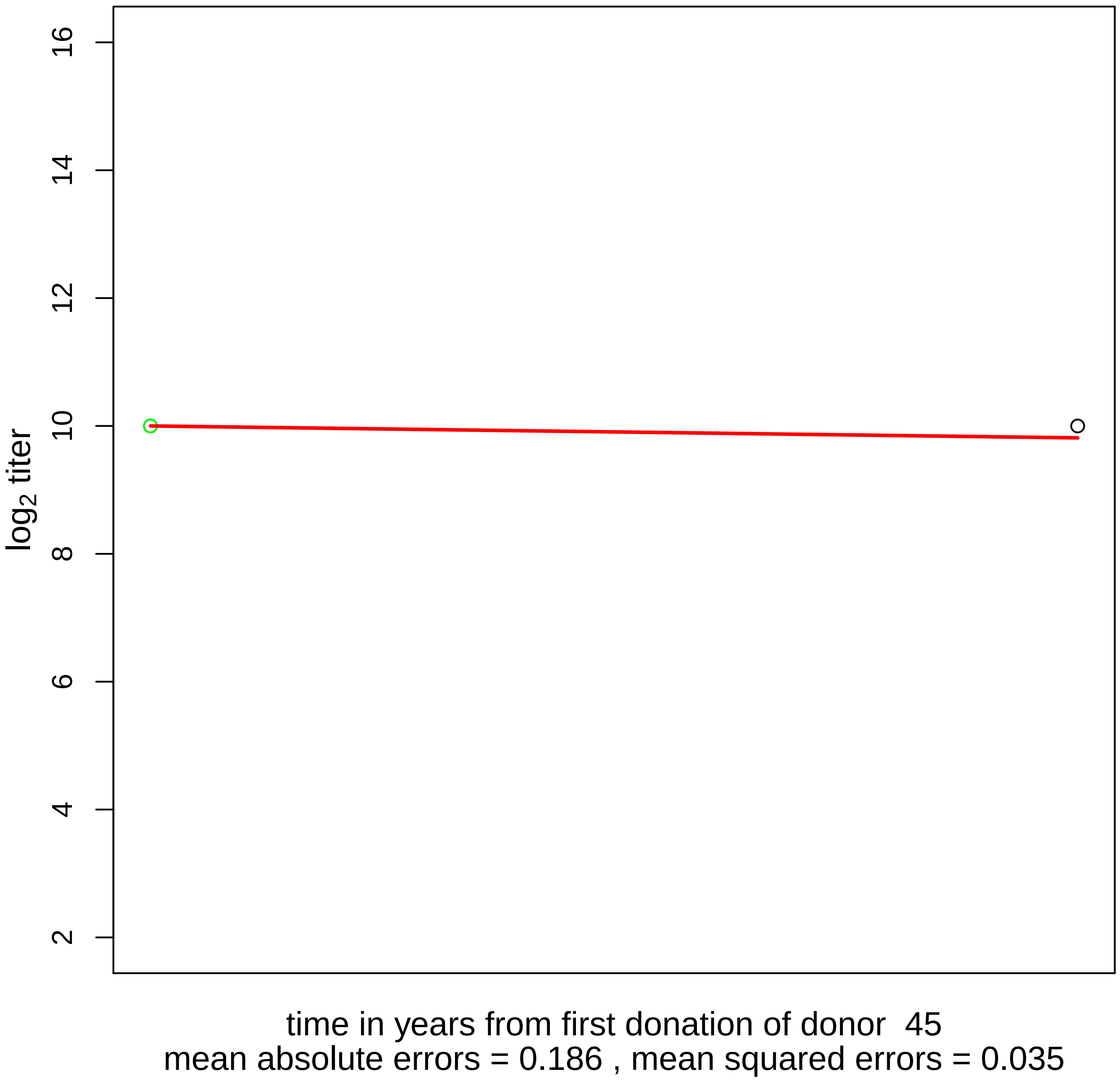

log2 titer
2
4
6
8
10
12
14
16
time in years from first donation of donor 45
mean absolute errors = 0.186 , mean squared errors = 0.035

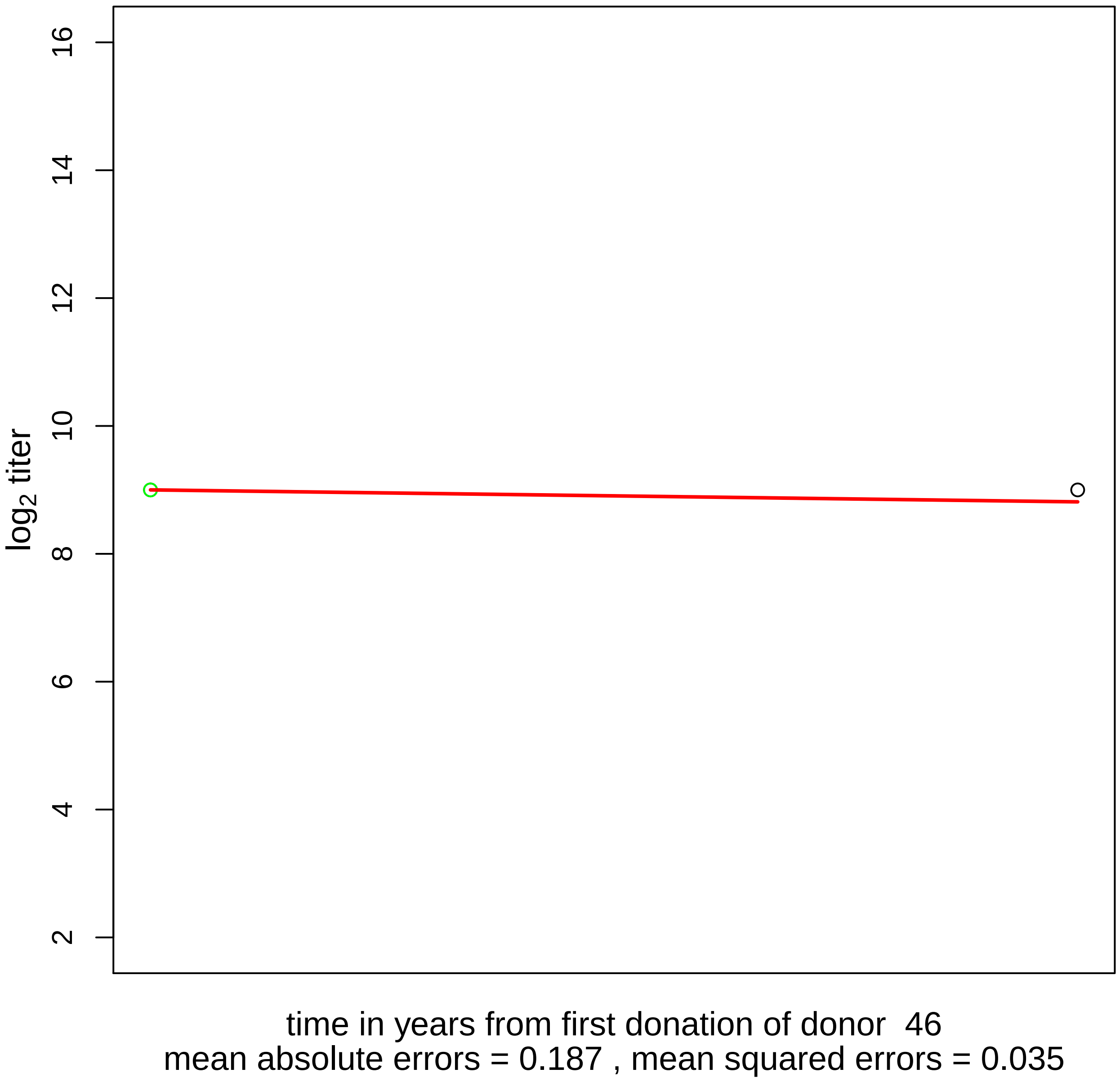
16
14
12
10
8
6
4
2
log_2 titer
time in years from first donation of donor 46
mean absolute errors = 0.187 , mean squared errors = 0.035

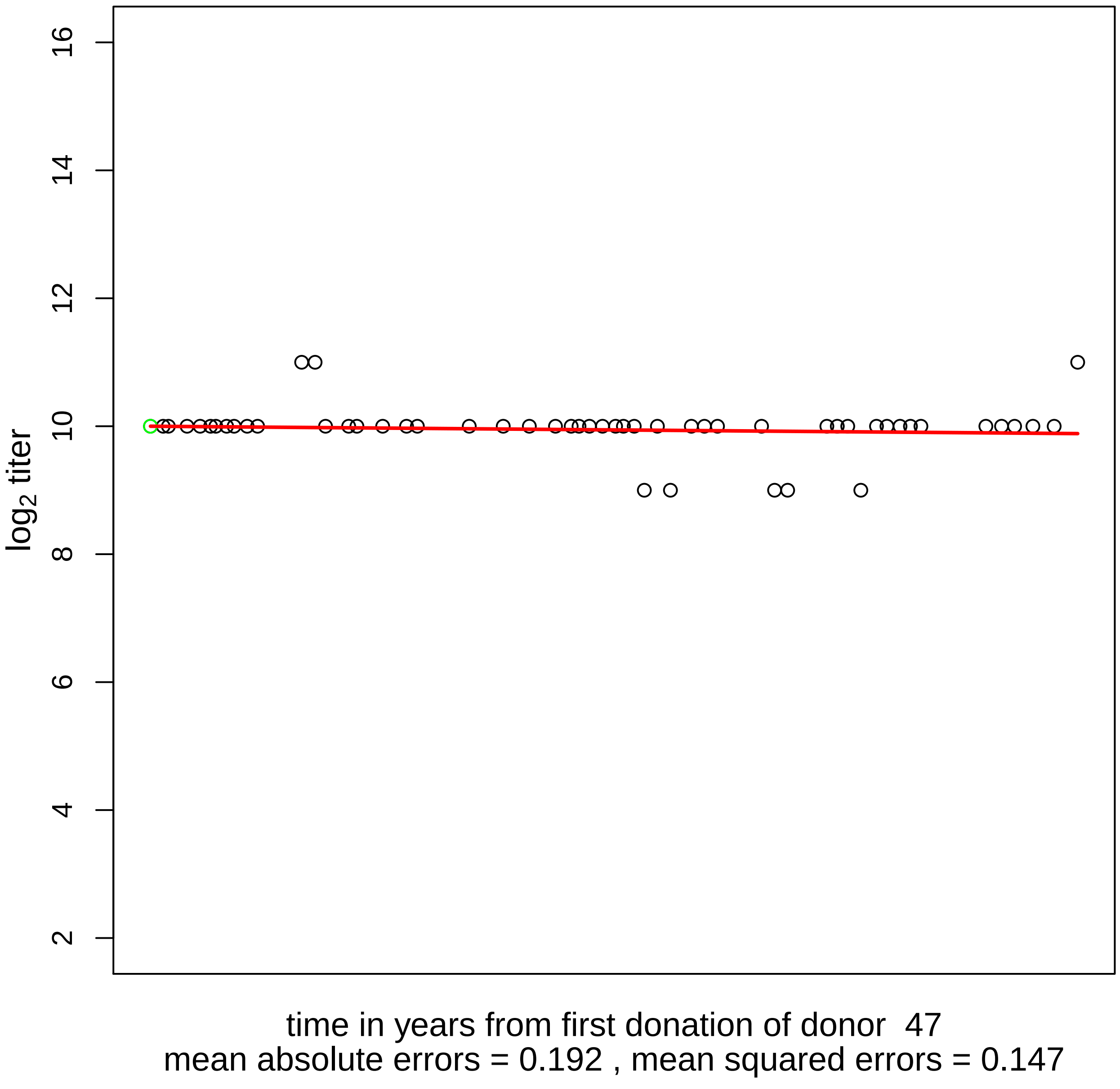

log2 titer
2
4
6
8
10
12
14
16
time in years from first donation of donor 47
mean absolute errors = 0.192 , mean squared errors = 0.147

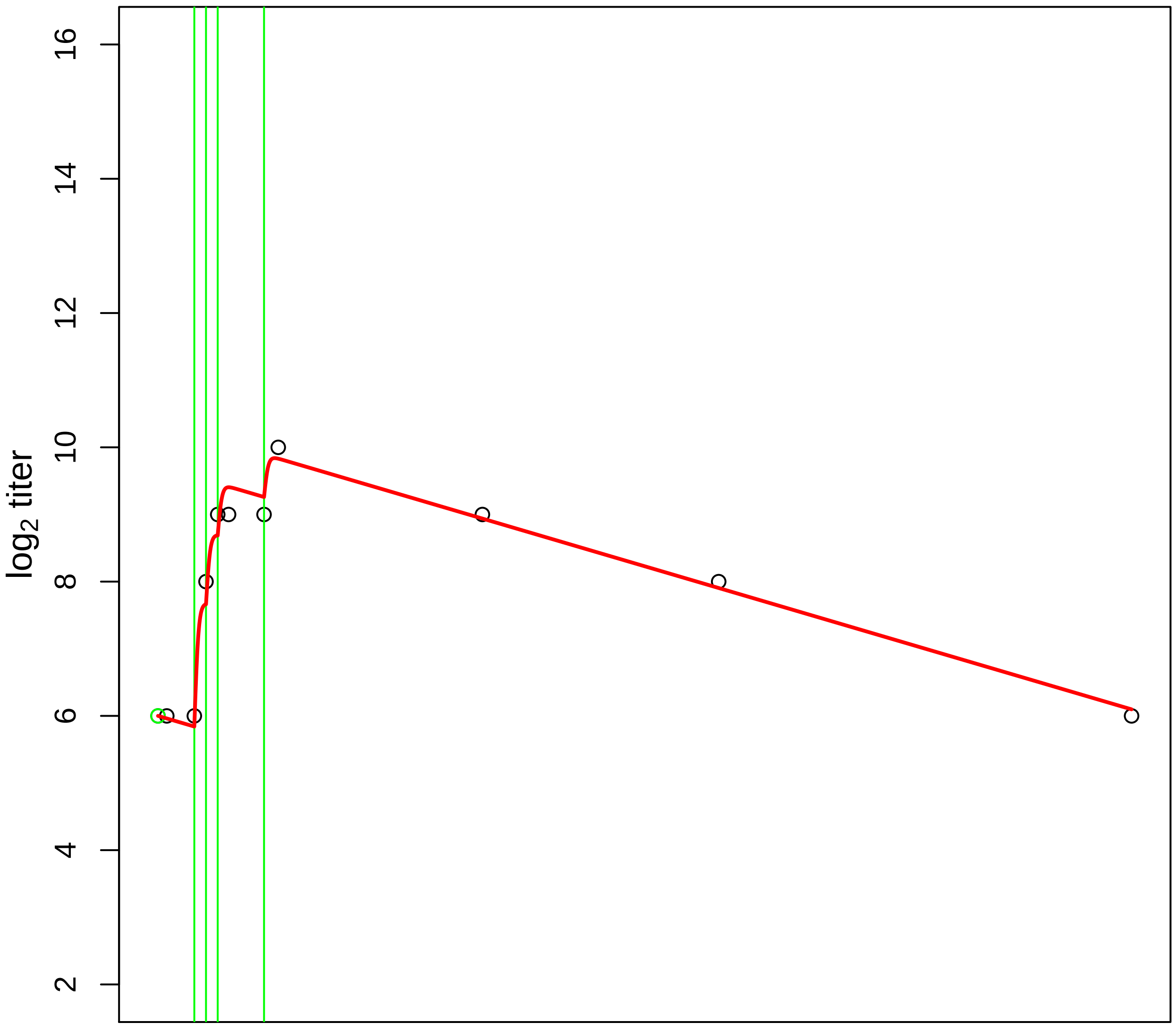

time in years from first donation of donor 48
mean absolute errors = 0.194 , mean squared errors = 0.052

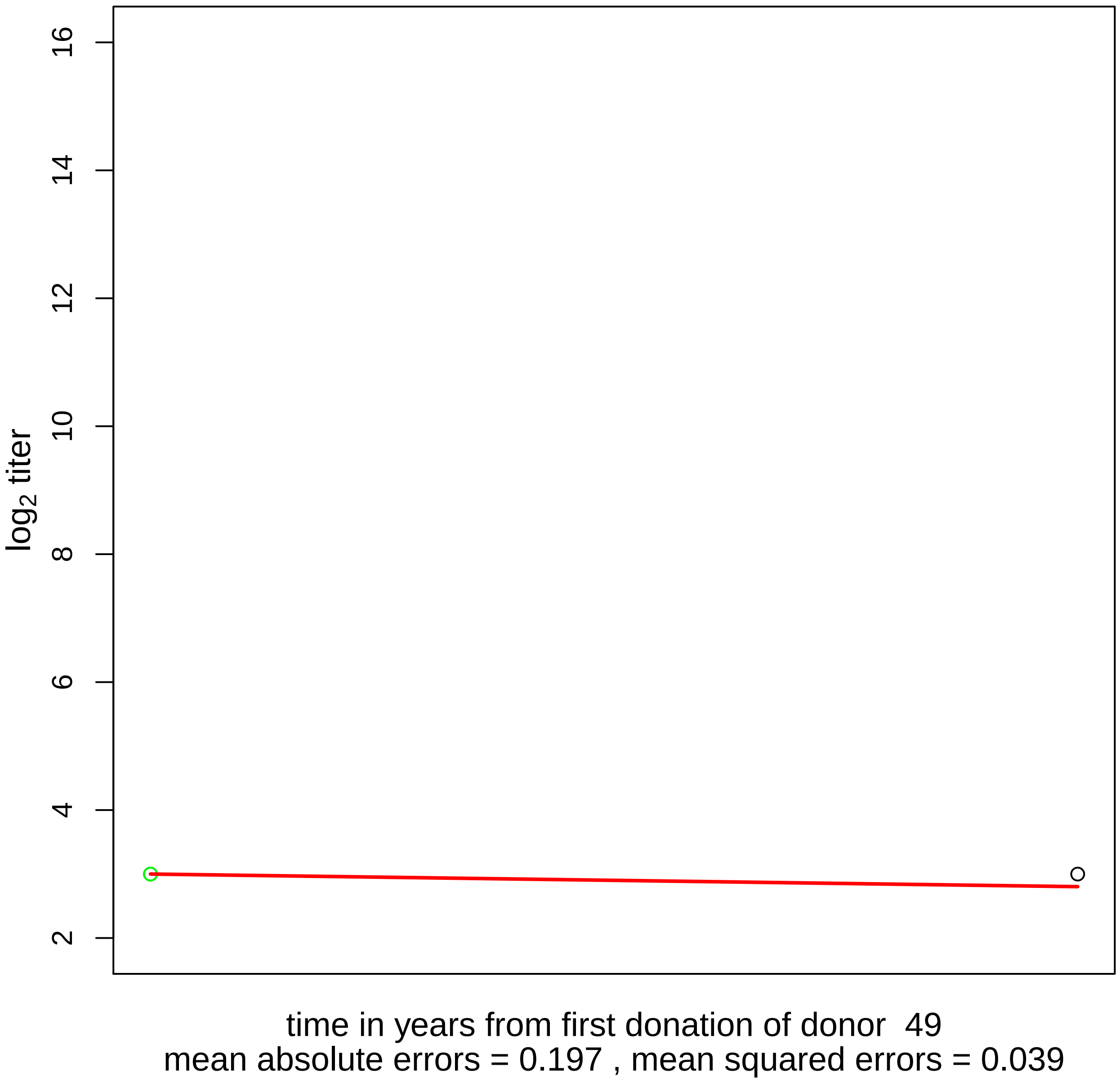

16
14
12
10
8
6
4
2
log2 titer
time in years from first donation of donor 49
mean absolute errors = 0.197 , mean squared errors = 0.039

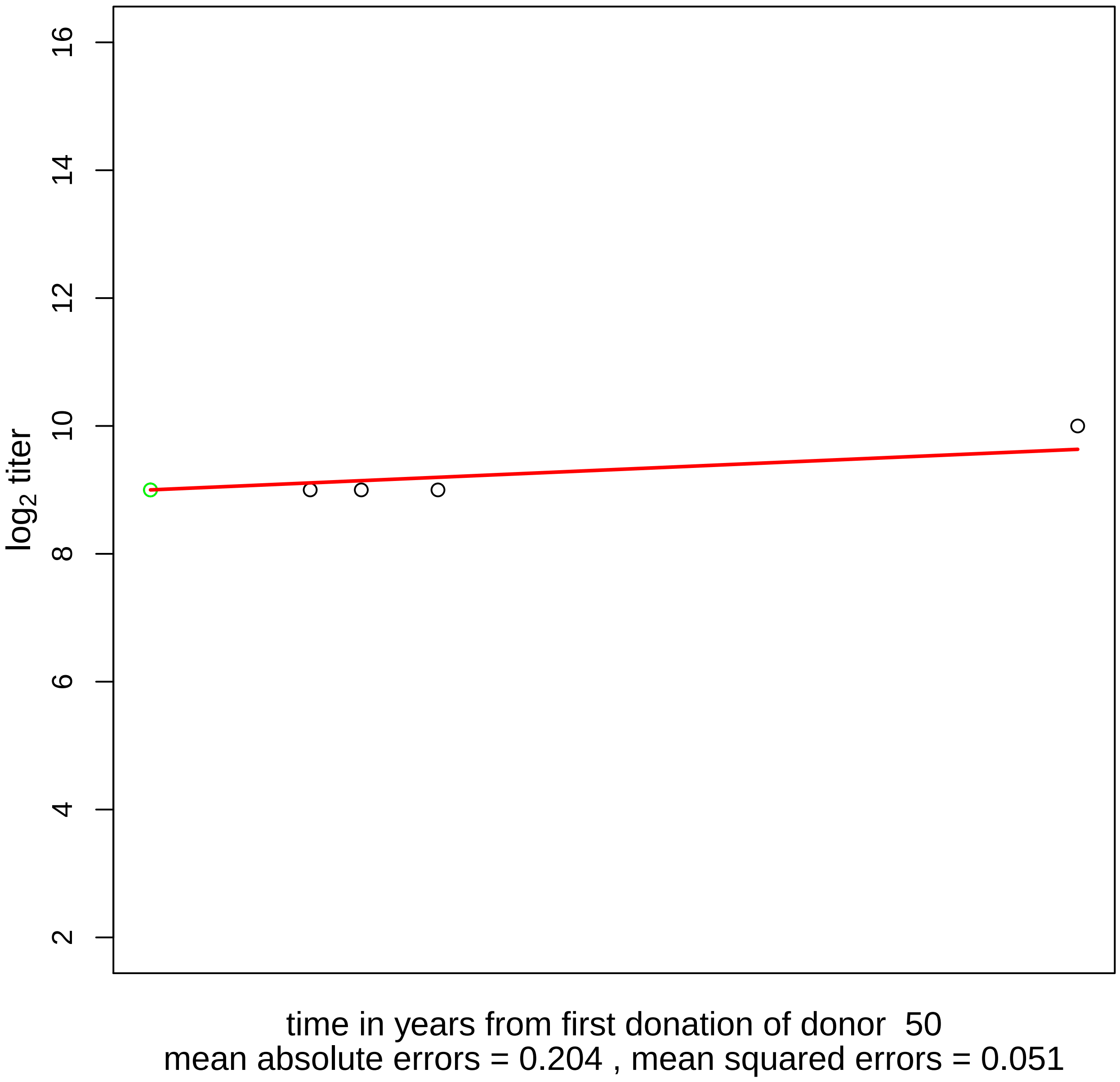

16
14
12
10
8
6
4
2
$\log_2$ titer
time in years from first donation of donor 50
mean absolute errors = 0.204 , mean squared errors = 0.051

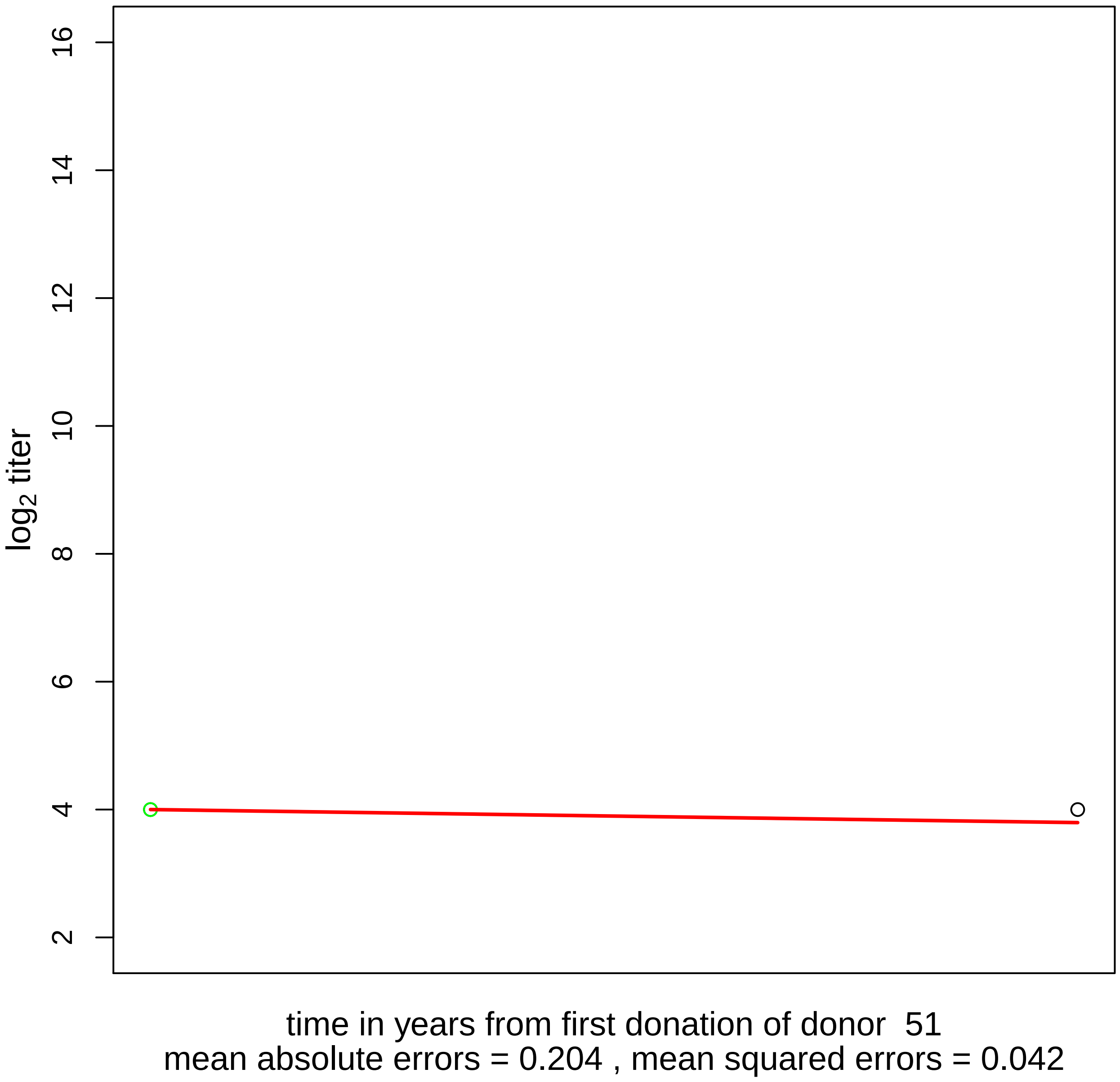
16
14
12
10
8
6
4
2
log2 titer
time in years from first donation of donor 51
mean absolute errors = 0.204 , mean squared errors = 0.042

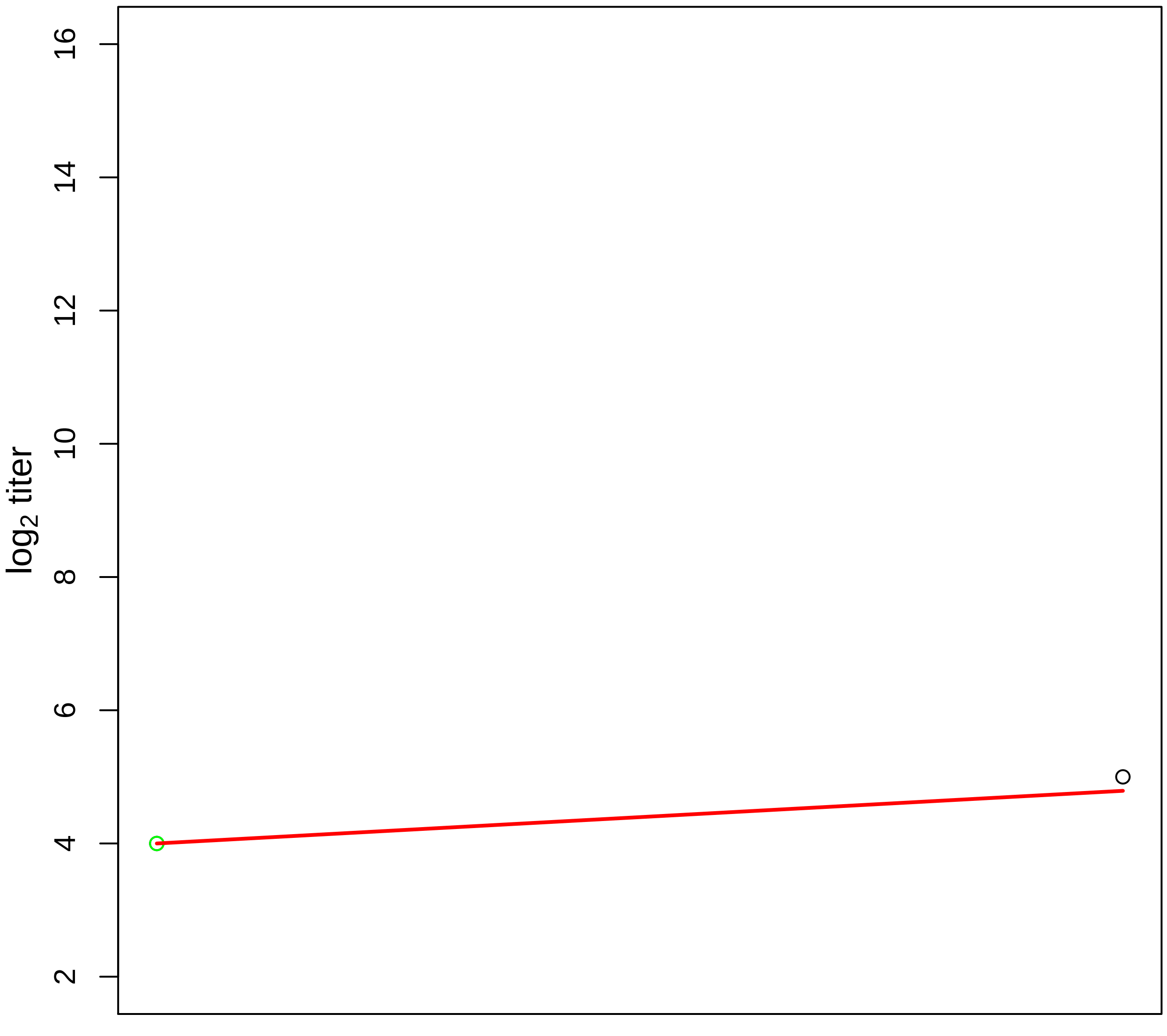

time in years from first donation of donor 52
mean absolute errors = 0.21 , mean squared errors = 0.044

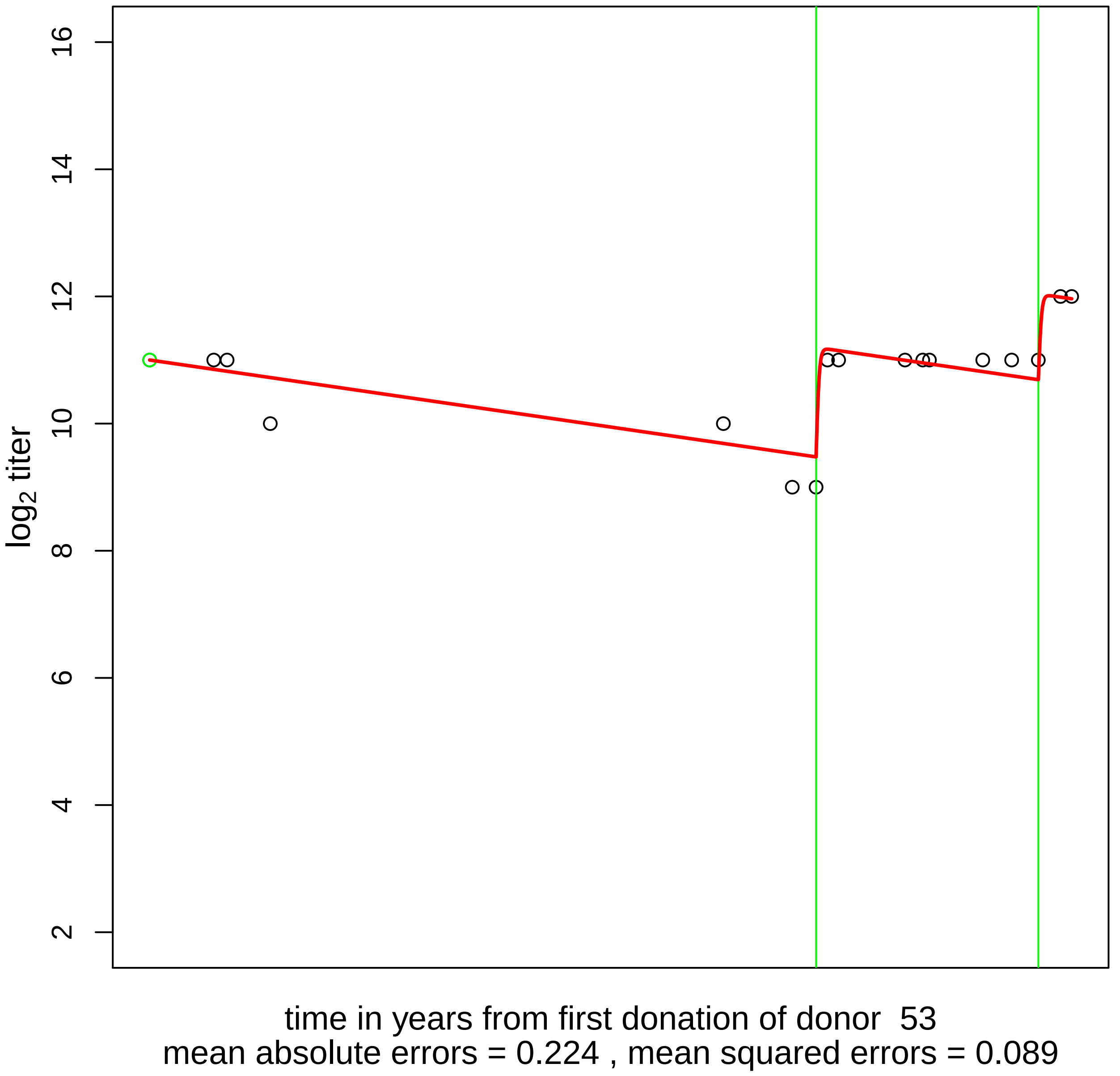

16
14
12
10
8
6
4
2
log2 titer
time in years from first donation of donor 53
mean absolute errors = 0.224 , mean squared errors = 0.089

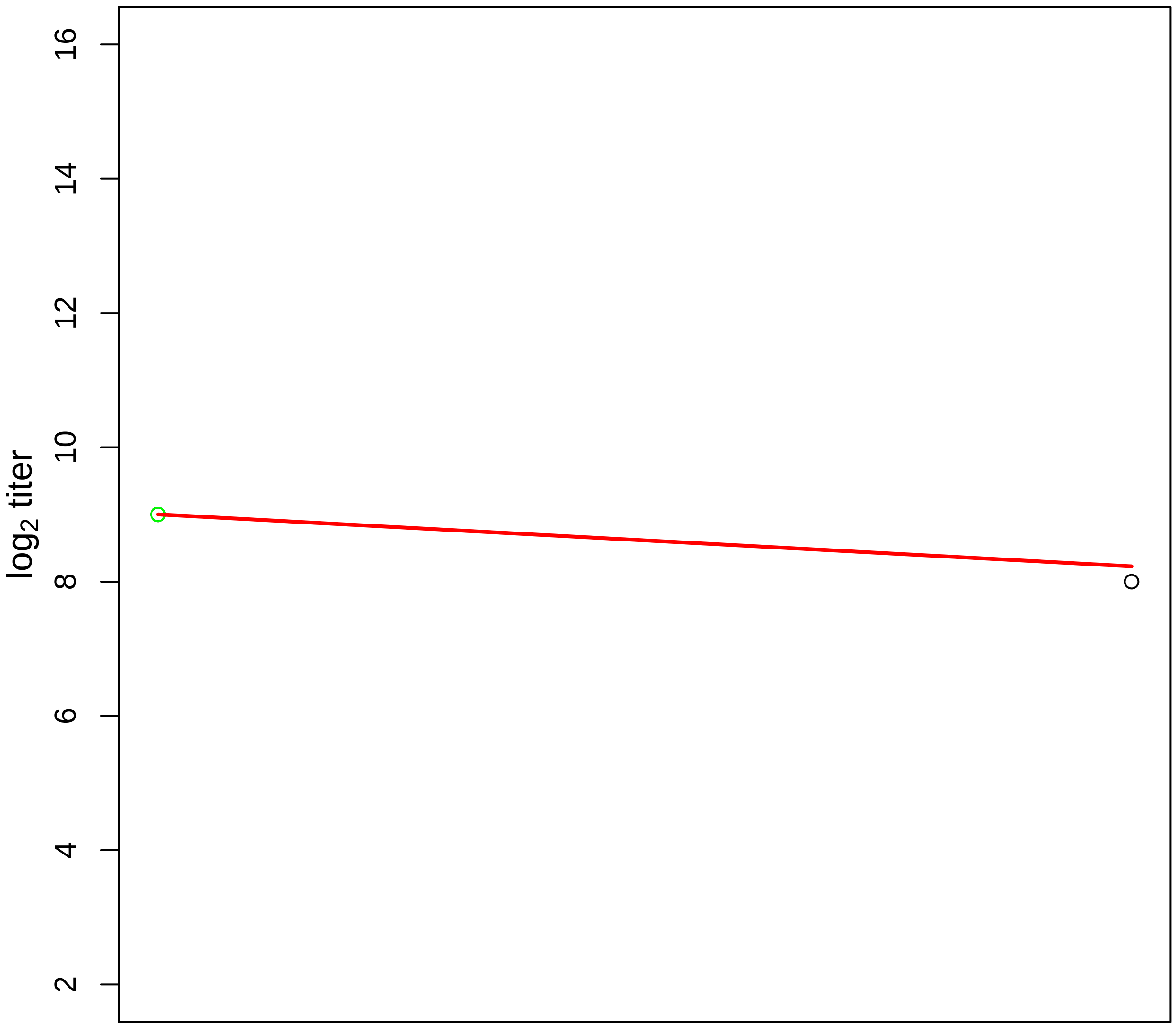

time in years from first donation of donor 54
mean absolute errors = 0.228 , mean squared errors = 0.052

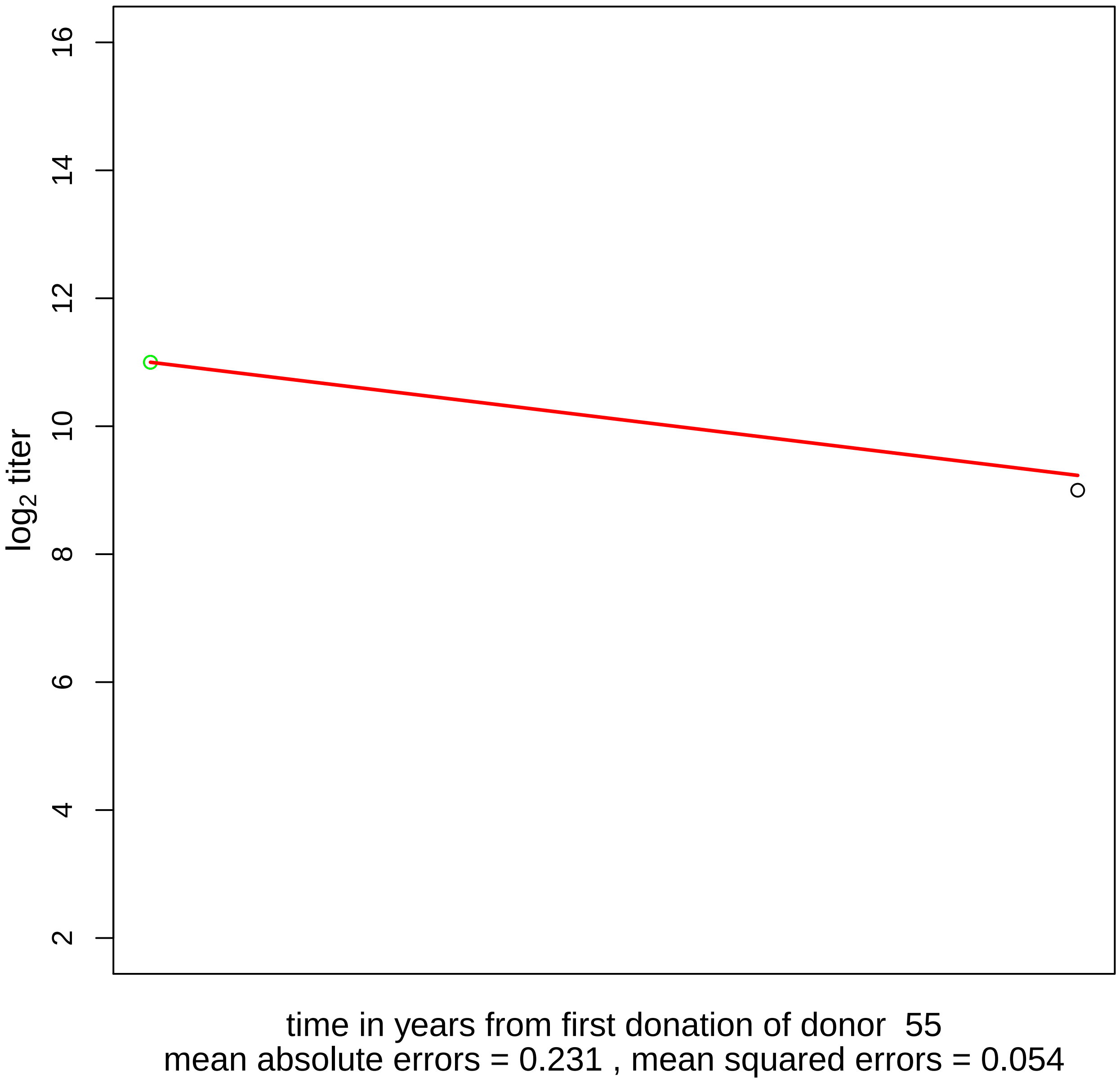

16
14
12
10
8
6
4
2
log2 titer
time in years from first donation of donor 55
mean absolute errors = 0.231 , mean squared errors = 0.054

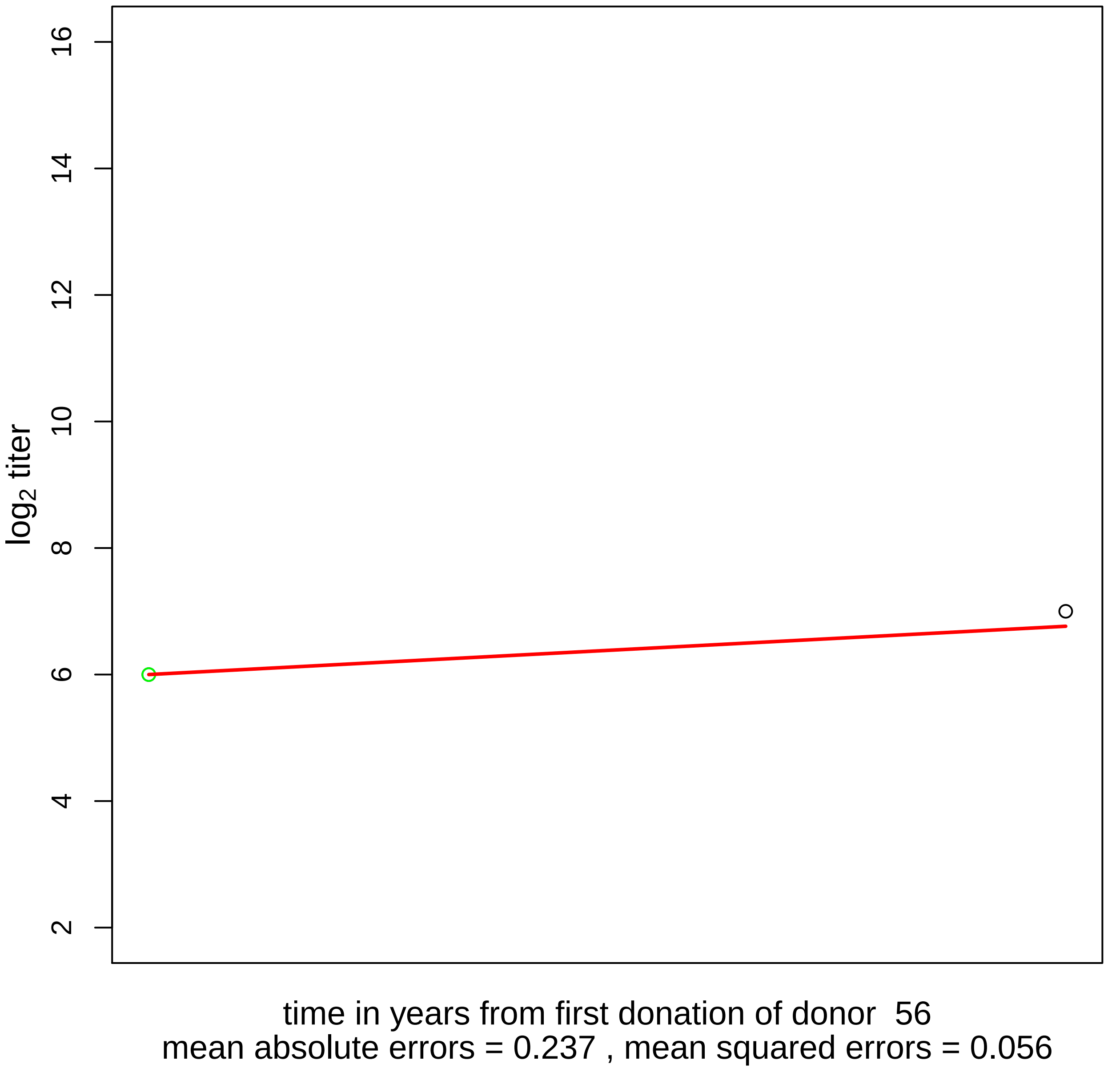

16
14
12
10
8
6
4
2
log2 titer
time in years from first donation of donor 56
mean absolute errors = 0.237 , mean squared errors = 0.056

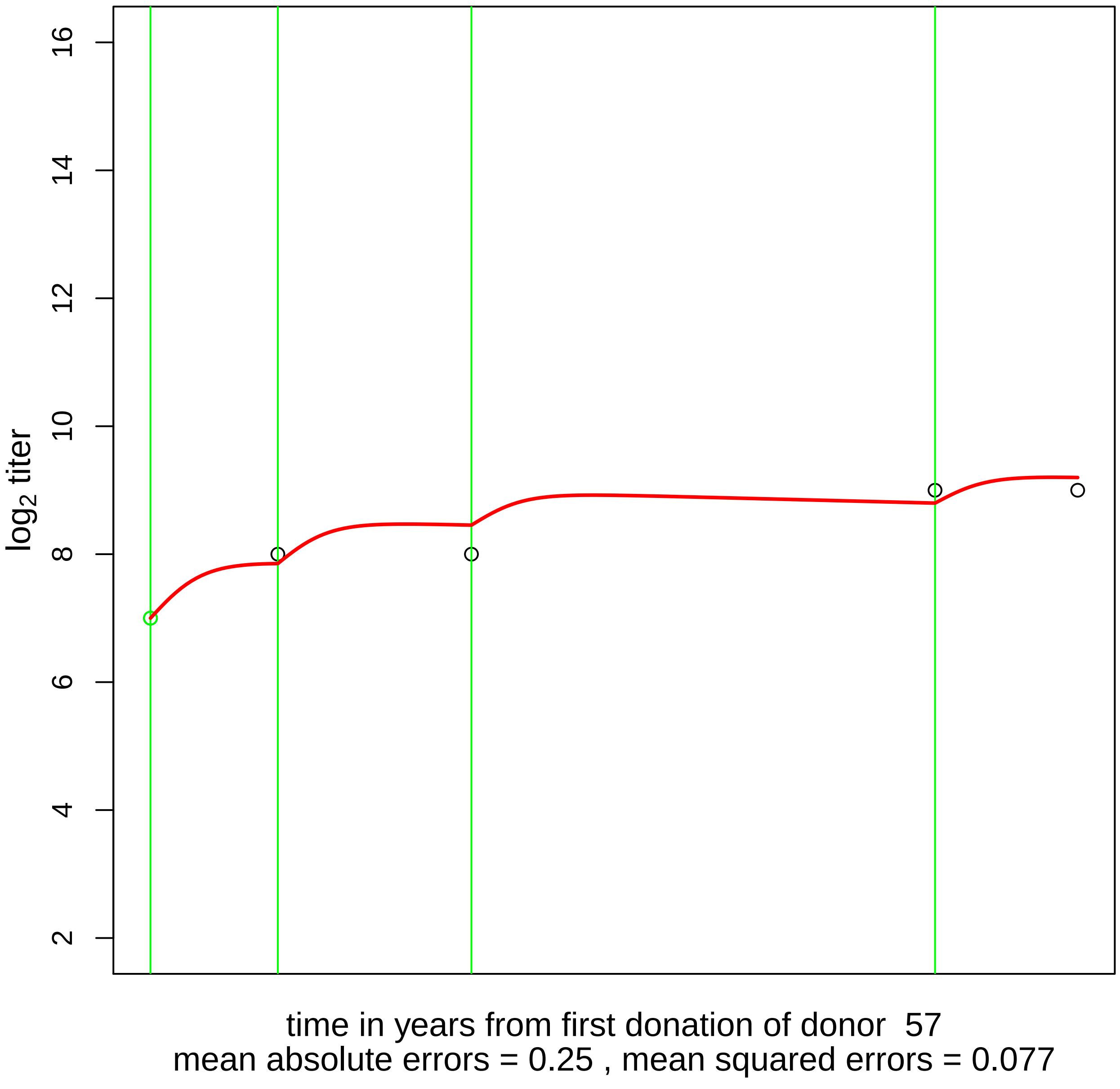

log2 titer
2
4
6
8
10
12
14
16
time in years from first donation of donor 57
mean absolute errors = 0.25 , mean squared errors = 0.077

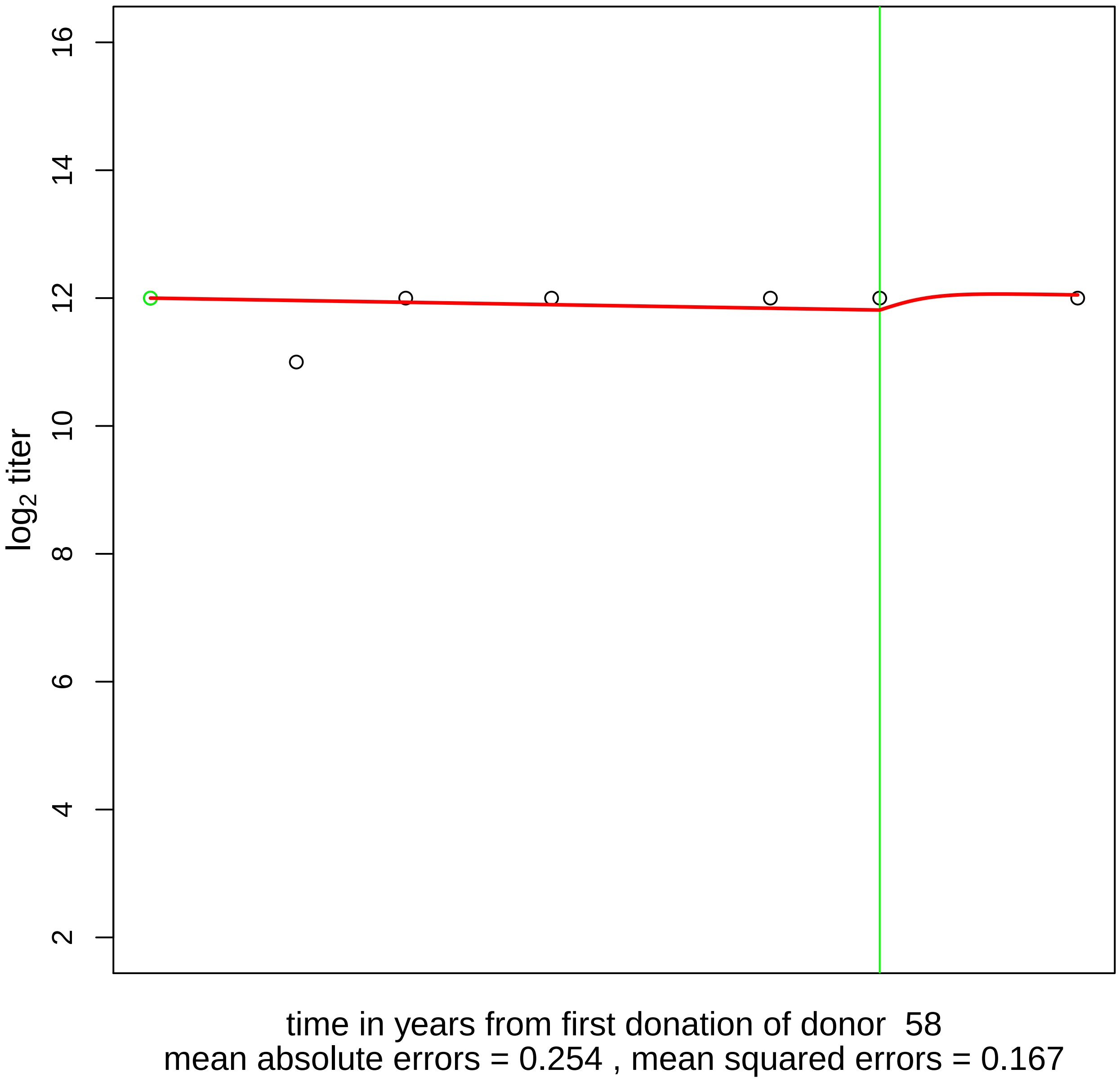

log2 titer
16
14
12
10
8
6
4
2
time in years from first donation of donor 58
mean absolute errors = 0.254 , mean squared errors = 0.167

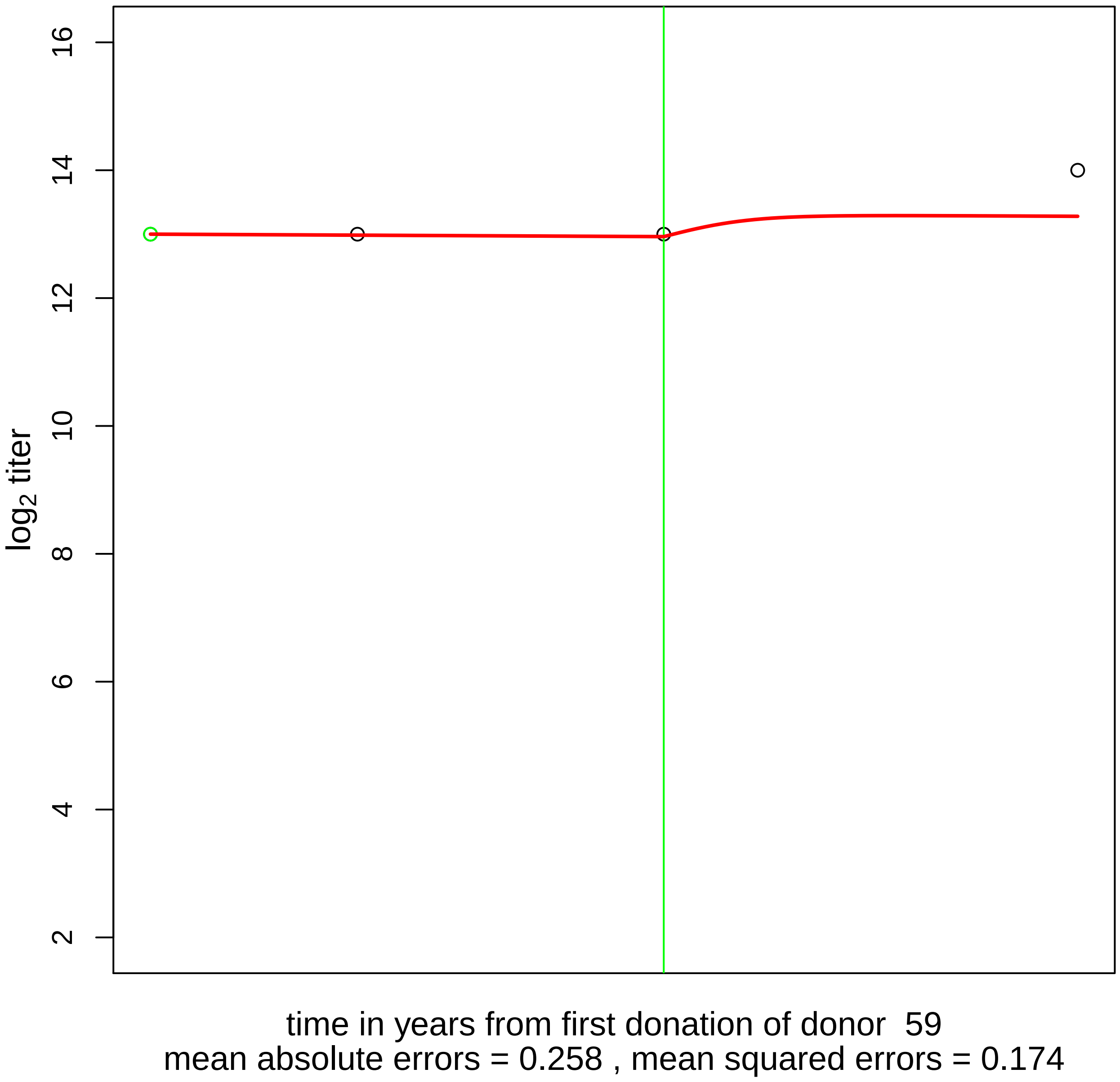

16
14
12
10
8
6
4
2
log2 titer
time in years from first donation of donor 59
mean absolute errors = 0.258 , mean squared errors = 0.174

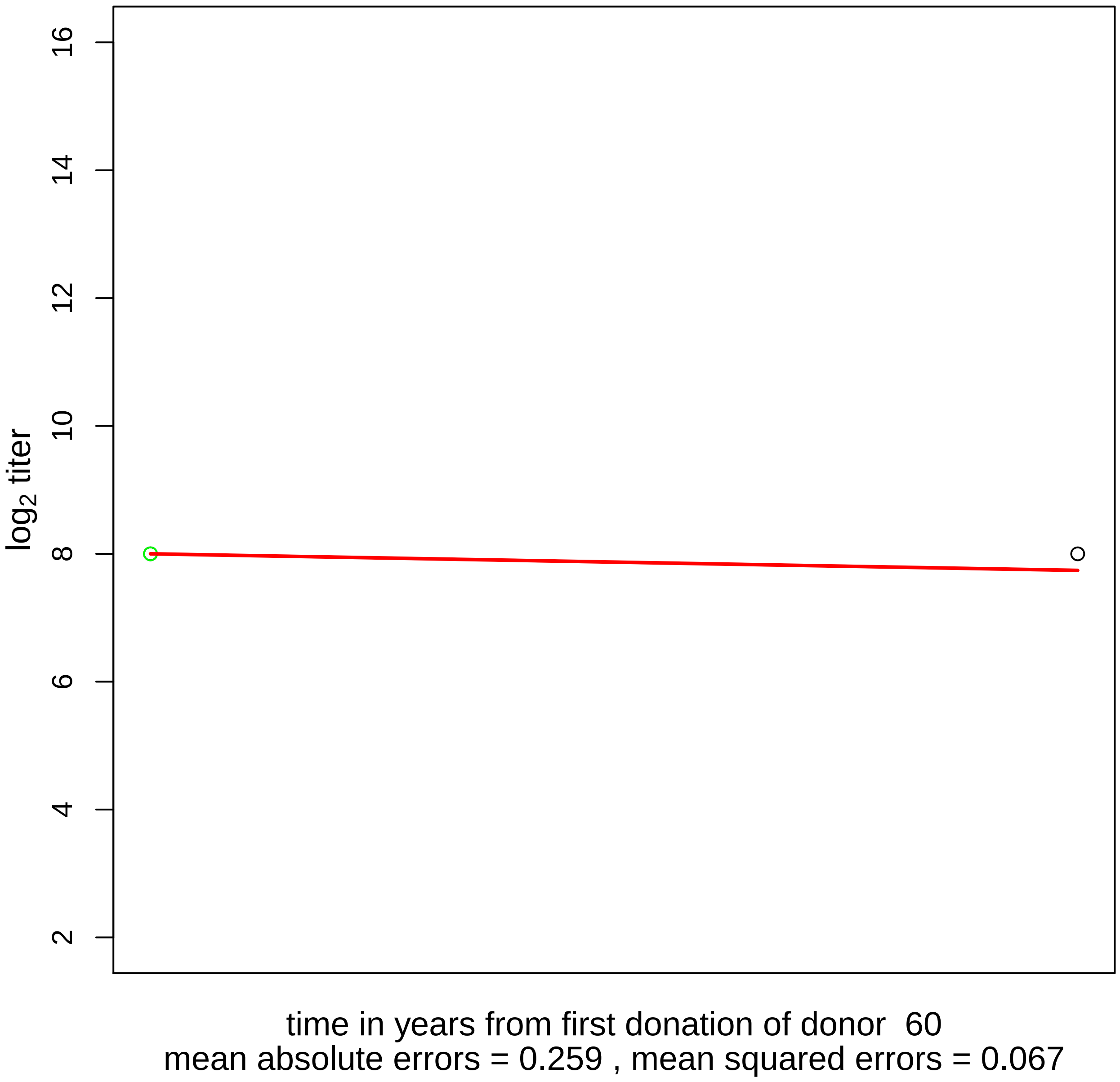
log2 titer
16
14
12
10
8
6
4
2
time in years from first donation of donor 60
mean absolute errors = 0.259 , mean squared errors = 0.067

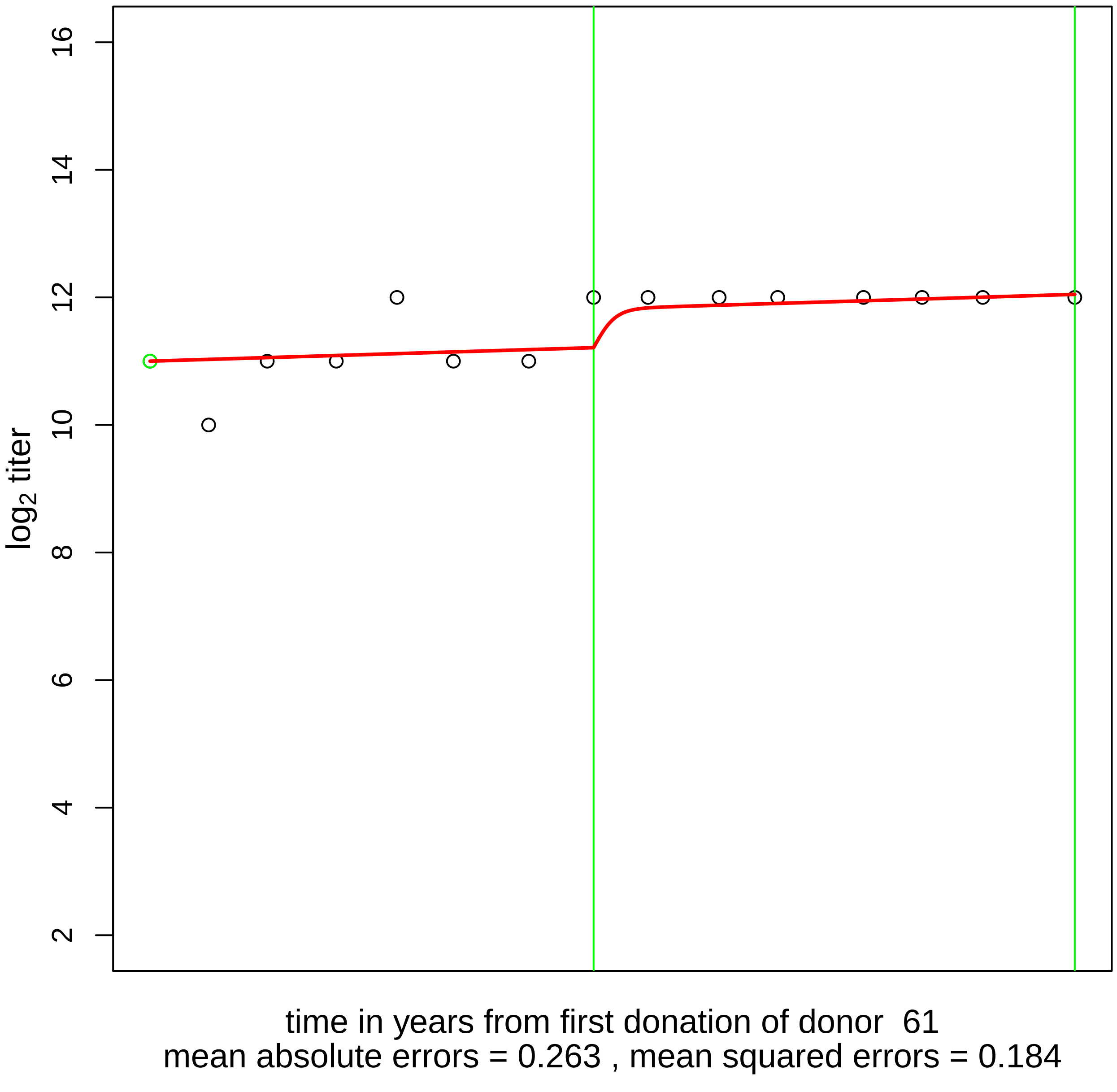

2
4
6
8
10
12
14
16
log2 titer
time in years from first donation of donor 61
mean absolute errors = 0.263 , mean squared errors = 0.184

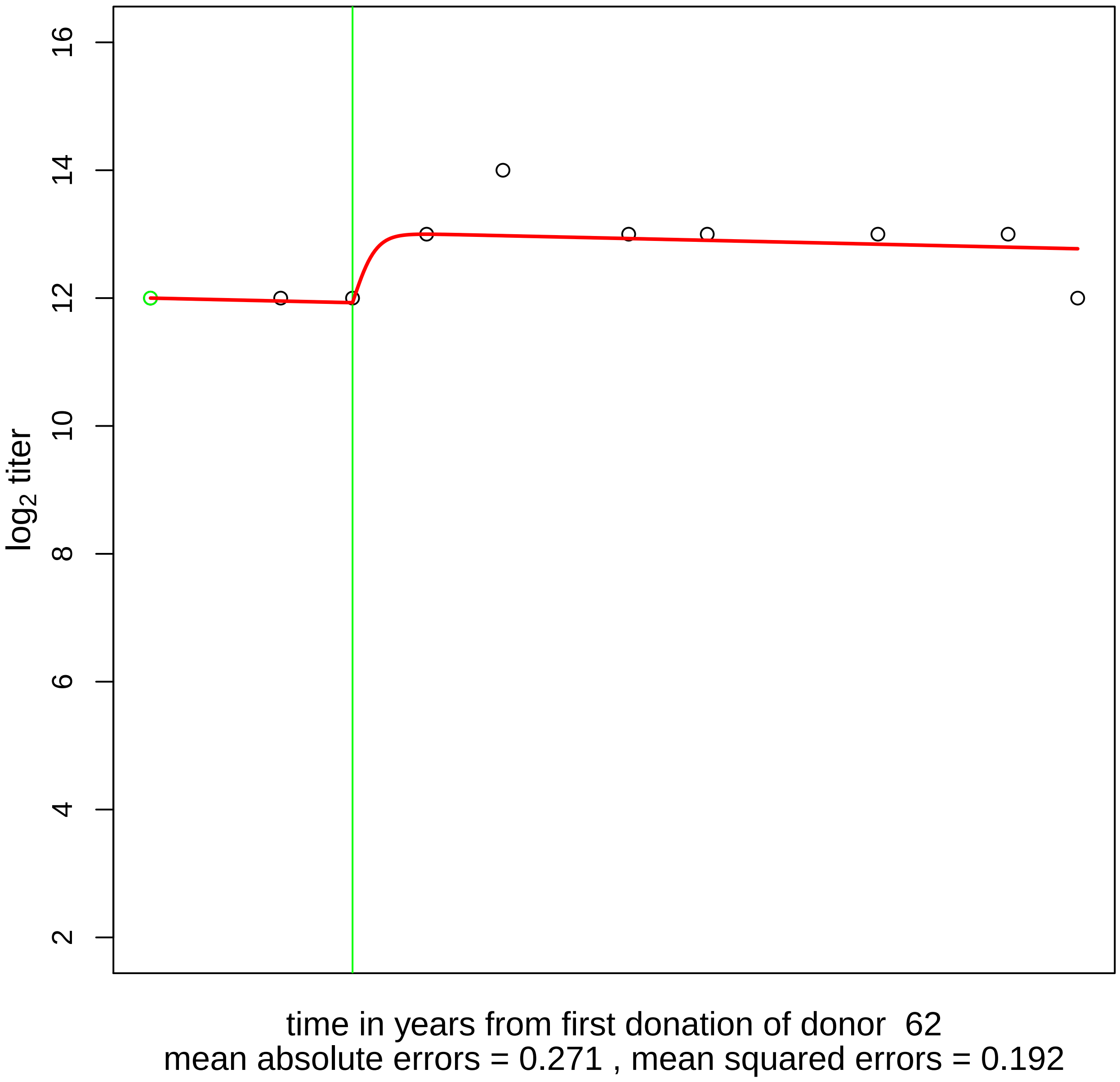

16
14
12
10
8
6
4
2
log2 titer
time in years from first donation of donor 62
mean absolute errors = 0.271 , mean squared errors = 0.192

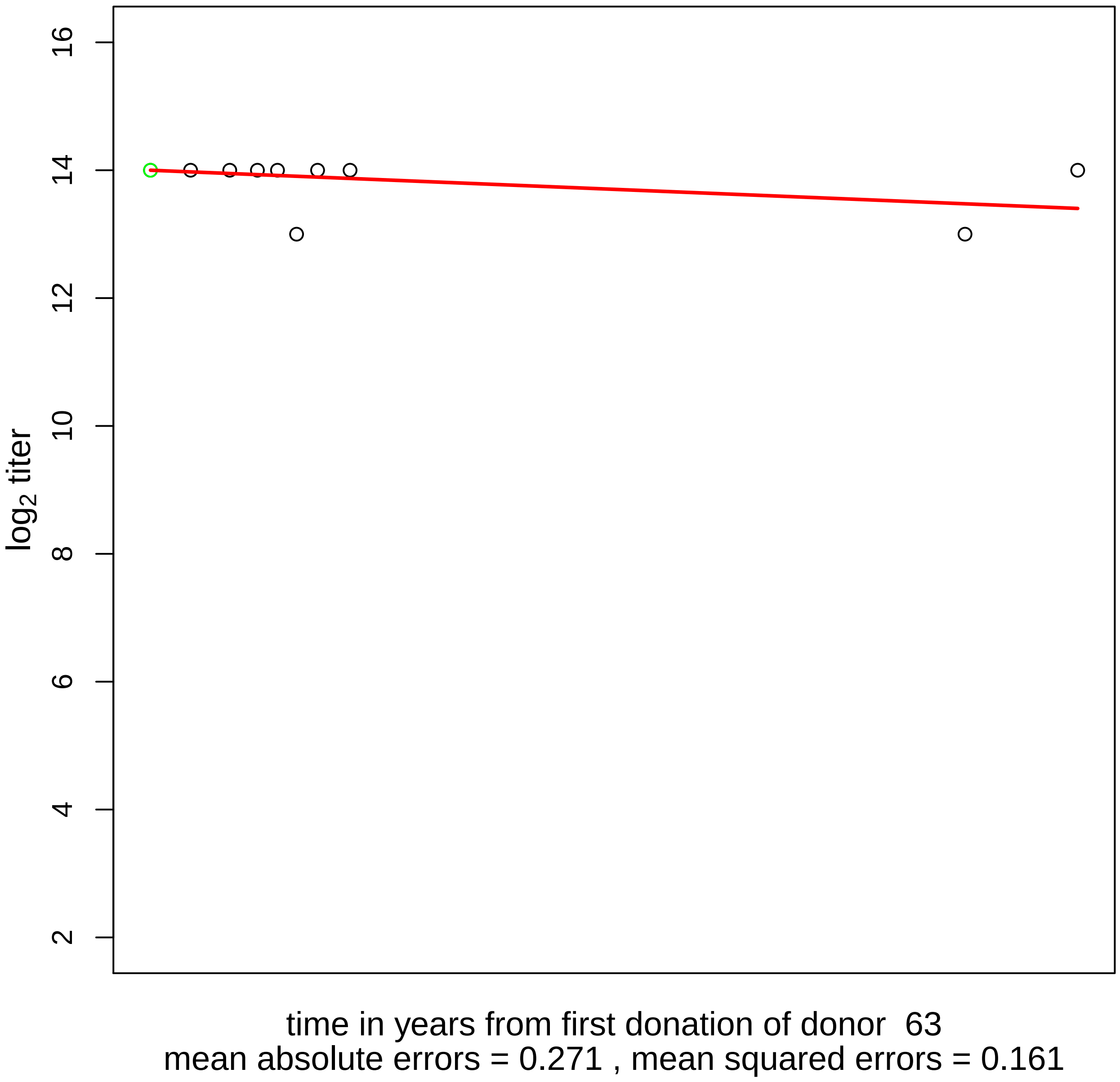

log2 titer
16
14
12
10
8
6
4
2
time in years from first donation of donor 63
mean absolute errors = 0.271 , mean squared errors = 0.161

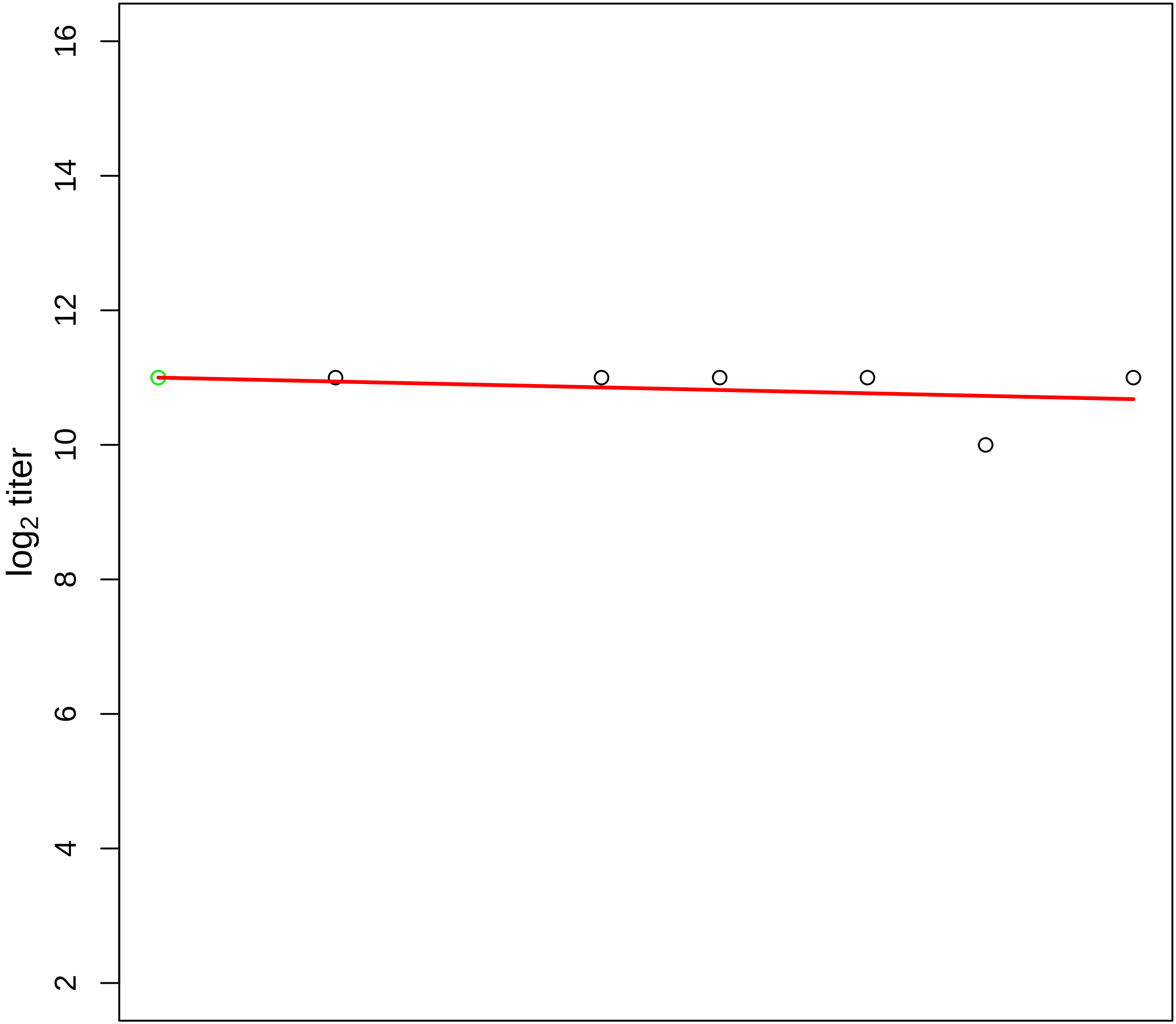

time in years from first donation of donor 64
mean absolute errors = 0.278 , mean squared errors = 0.124

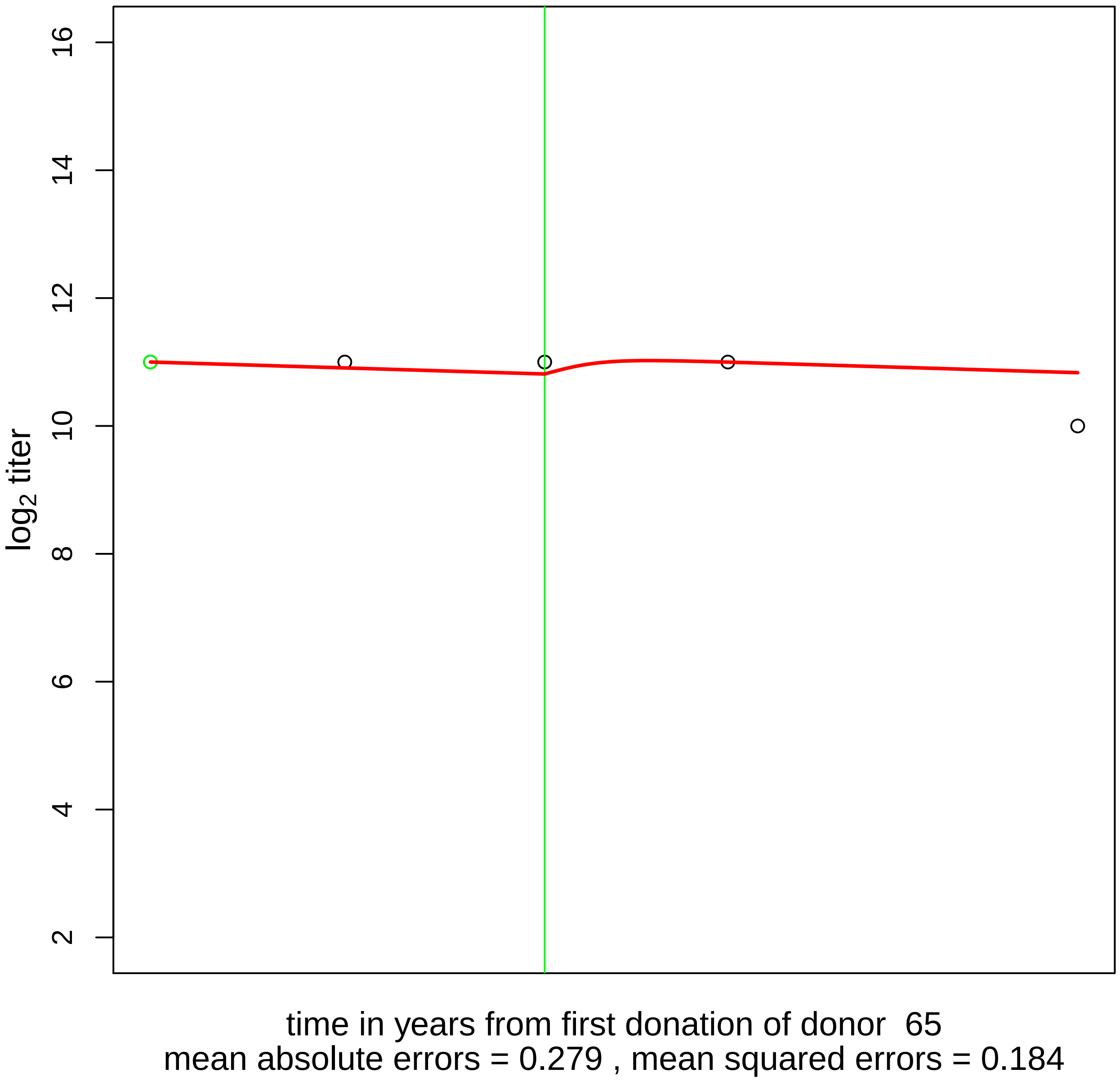
16
14
12
10
8
6
4
2
log2 titer
time in years from first donation of donor 65
mean absolute errors = 0.279 , mean squared errors = 0.184

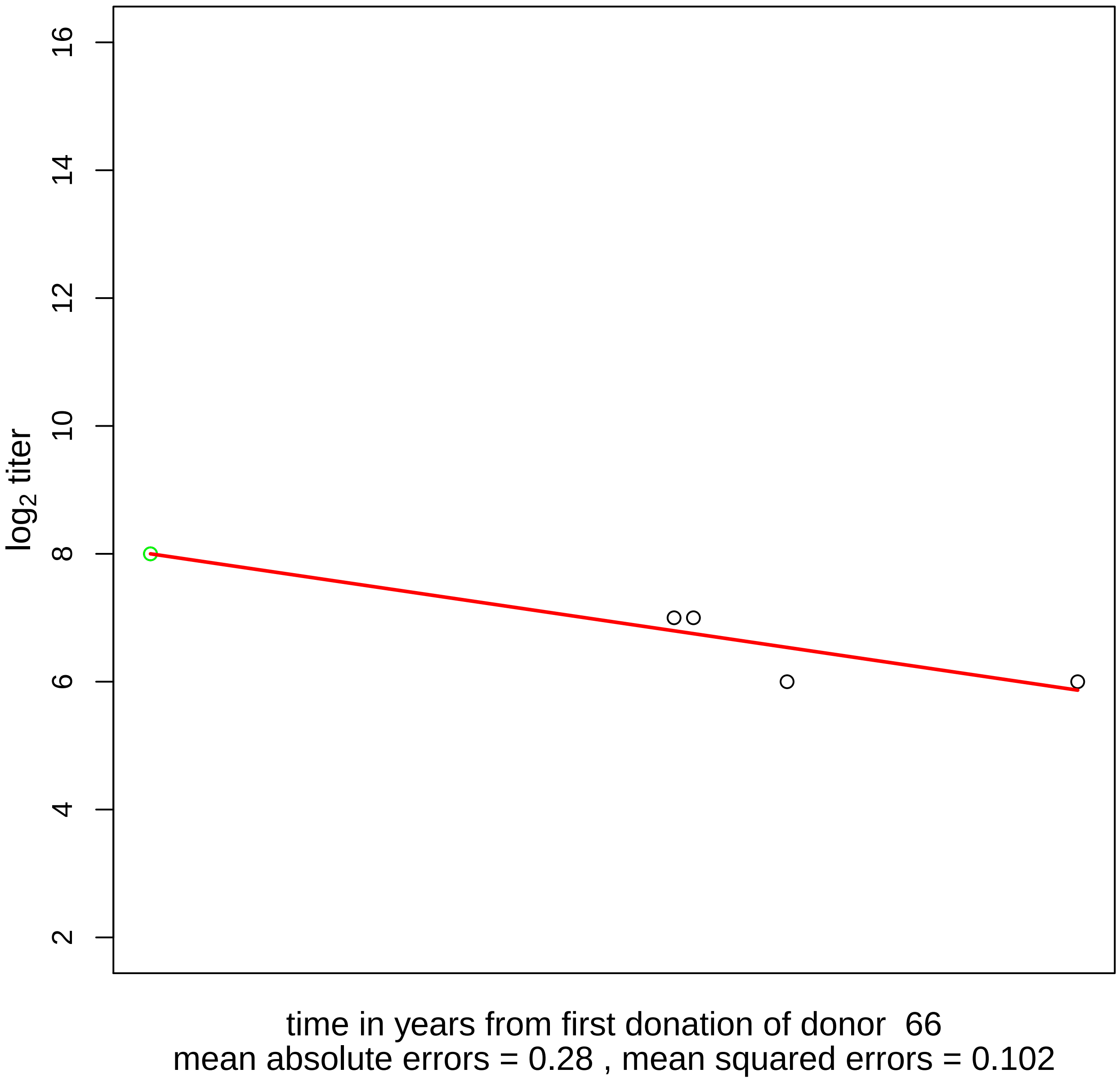

16
14
12
10
8
6
4
2
log2 titer
time in years from first donation of donor 66
mean absolute errors = 0.28 , mean squared errors = 0.102

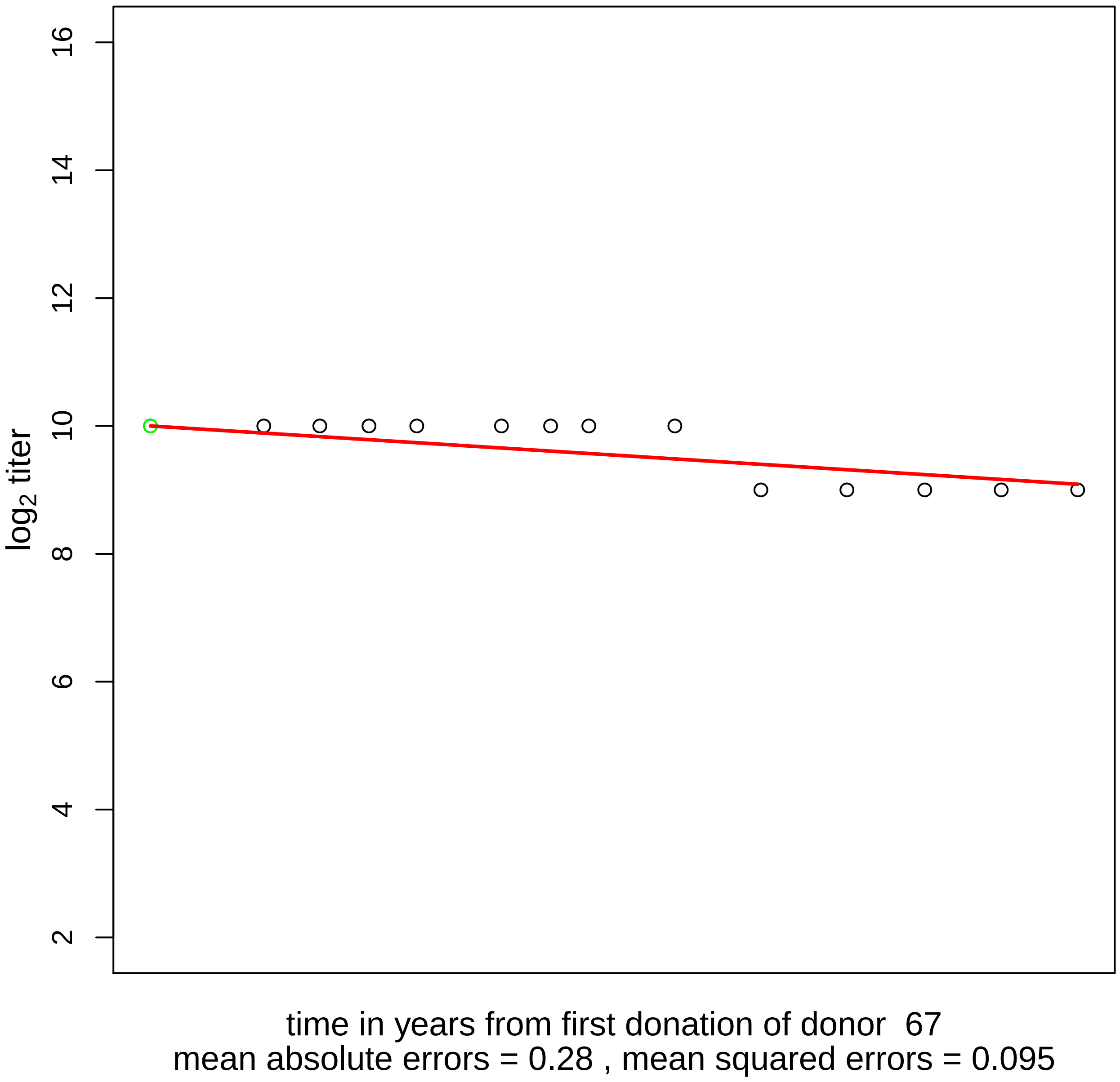
16
14
12
10
8
6
4
2
log2 titer
time in years from first donation of donor 67
mean absolute errors = 0.28 , mean squared errors = 0.095

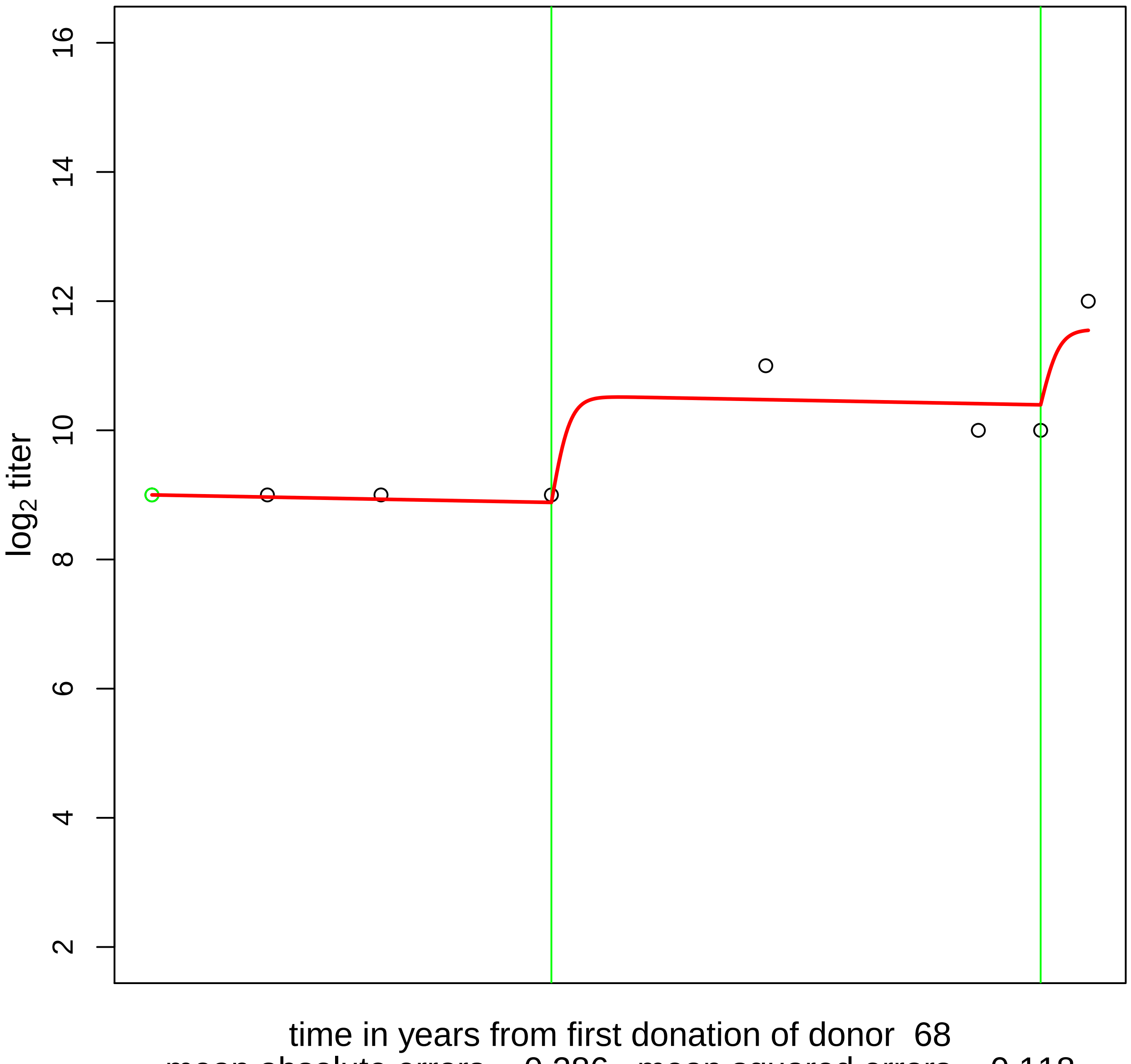

log2 titer
2
4
6
8
10
12
14
16
time in years from first donation of donor 68
mean absolute errors = 0.286 , mean squared errors = 0.118

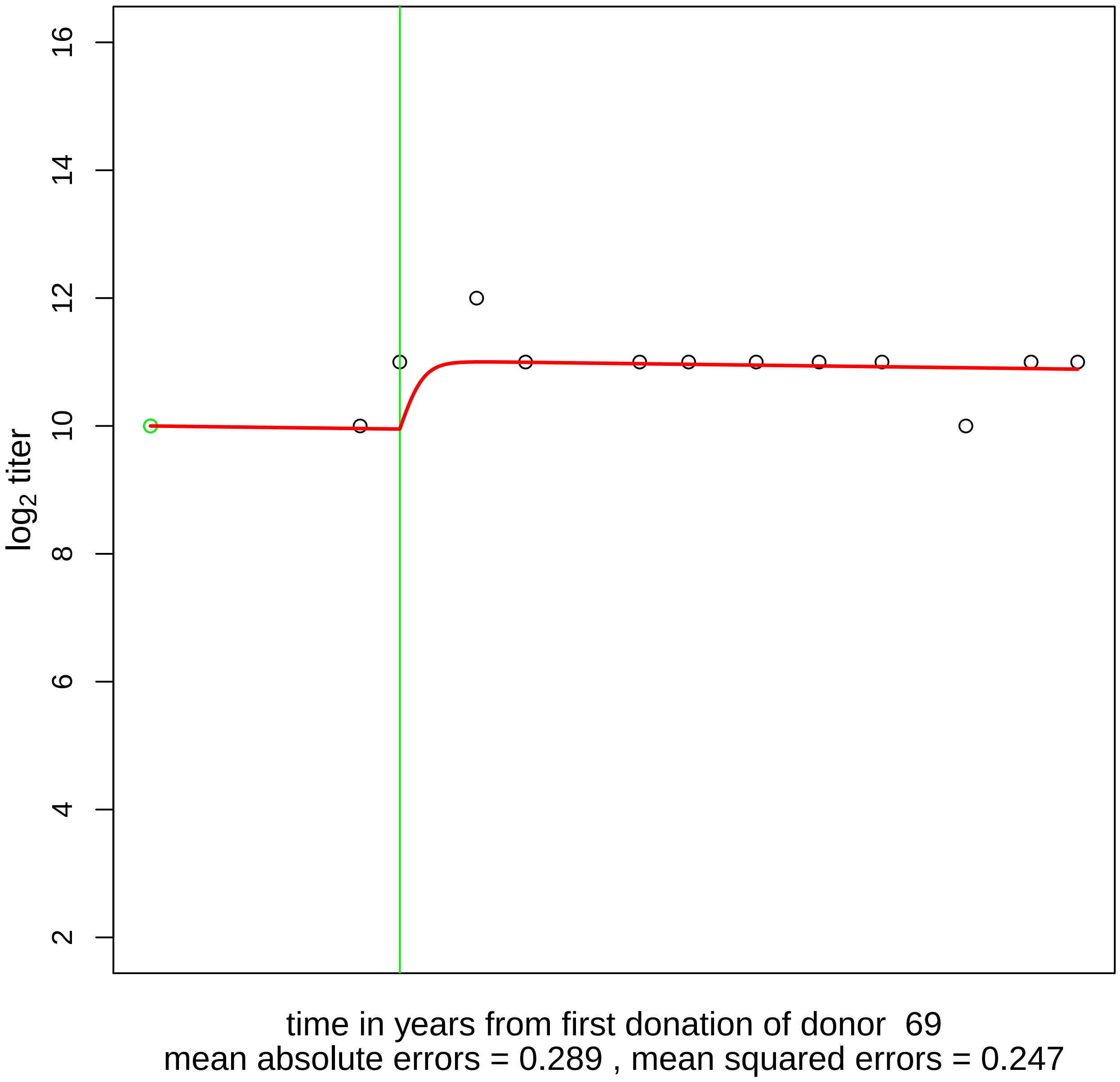

2
4
6
8
10
12
14
16
log2 titer
time in years from first donation of donor 69
mean absolute errors = 0.289 , mean squared errors = 0.247

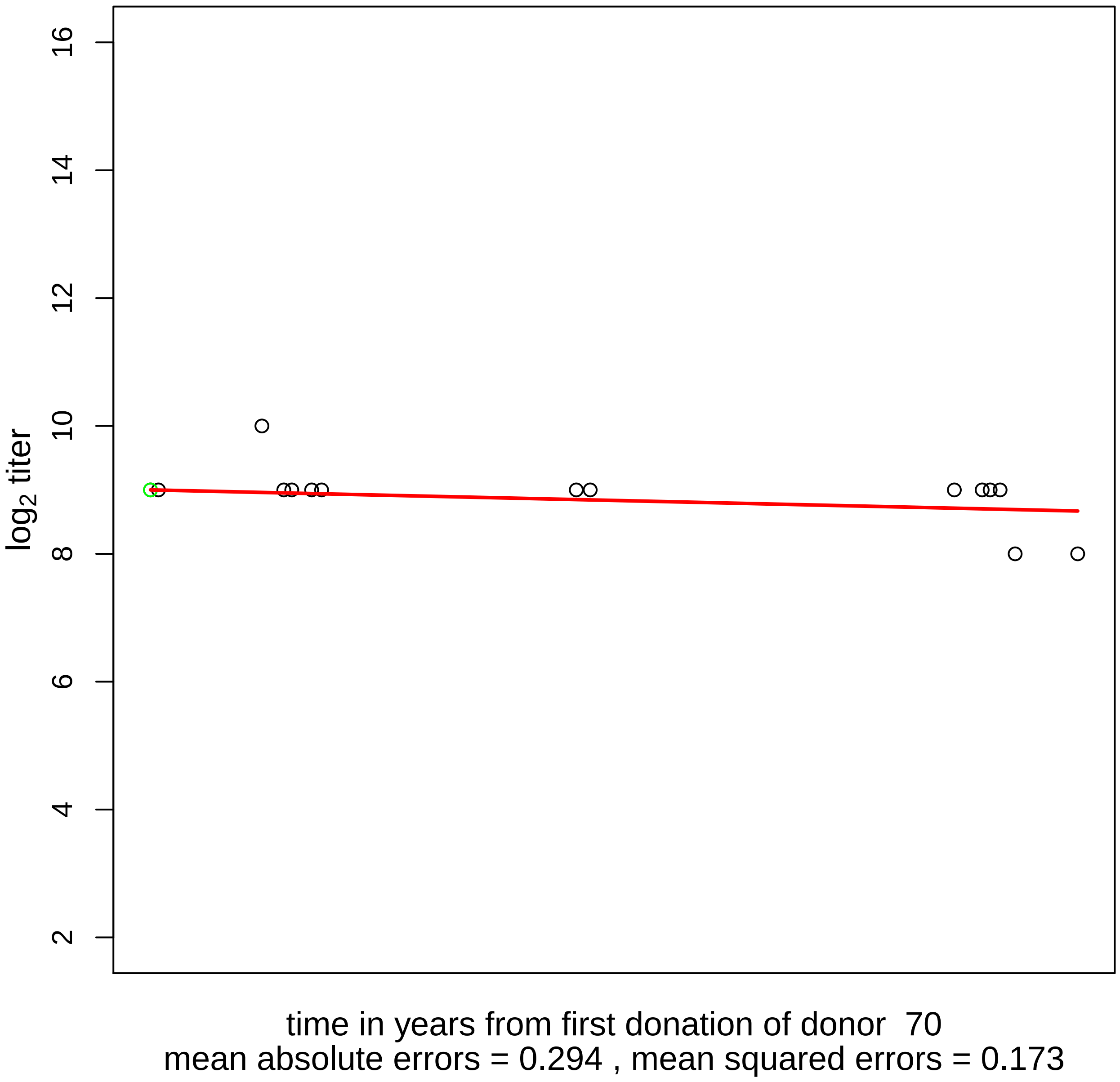

2
4
6
8
10
12
14
16
$\log_2$ titer
time in years from first donation of donor 70
mean absolute errors = 0.294 , mean squared errors = 0.173

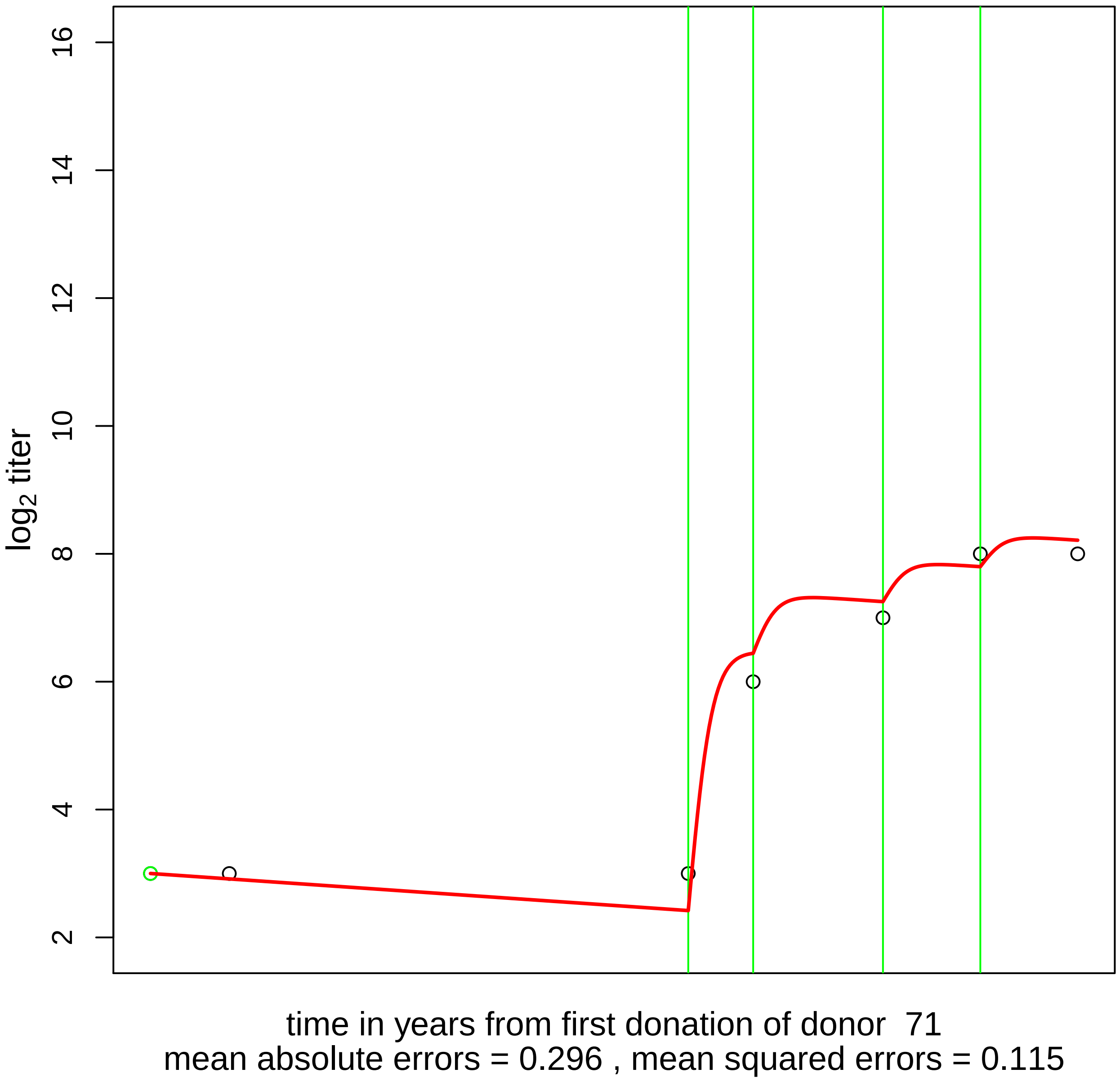

2
4
6
8
10
12
14
16
log2 titer
time in years from first donation of donor 71
mean absolute errors = 0.296 , mean squared errors = 0.115

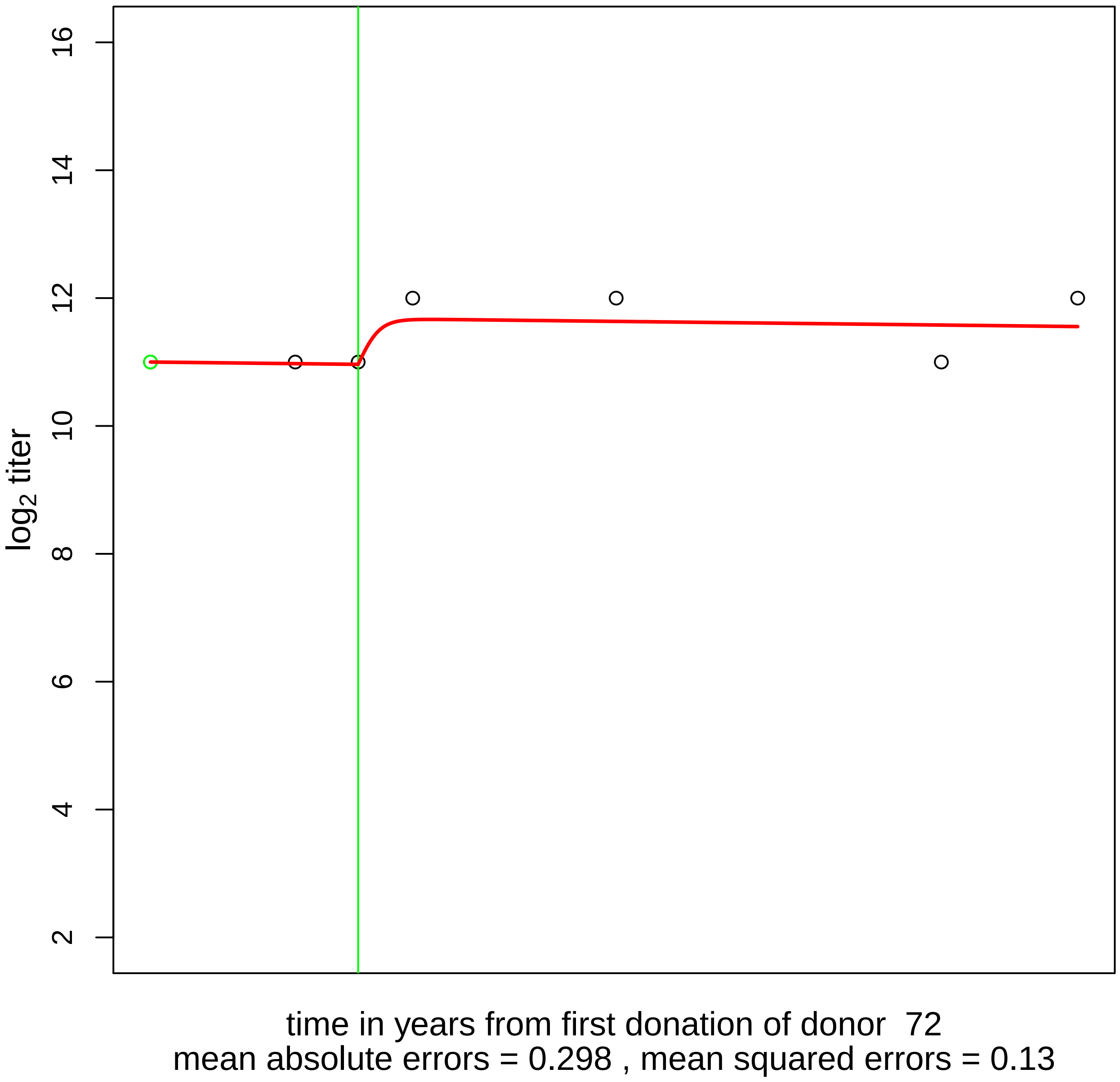

16
14
12
10
8
6
4
2
$\log_2$ titer
time in years from first donation of donor 72
mean absolute errors = 0.298 , mean squared errors = 0.13

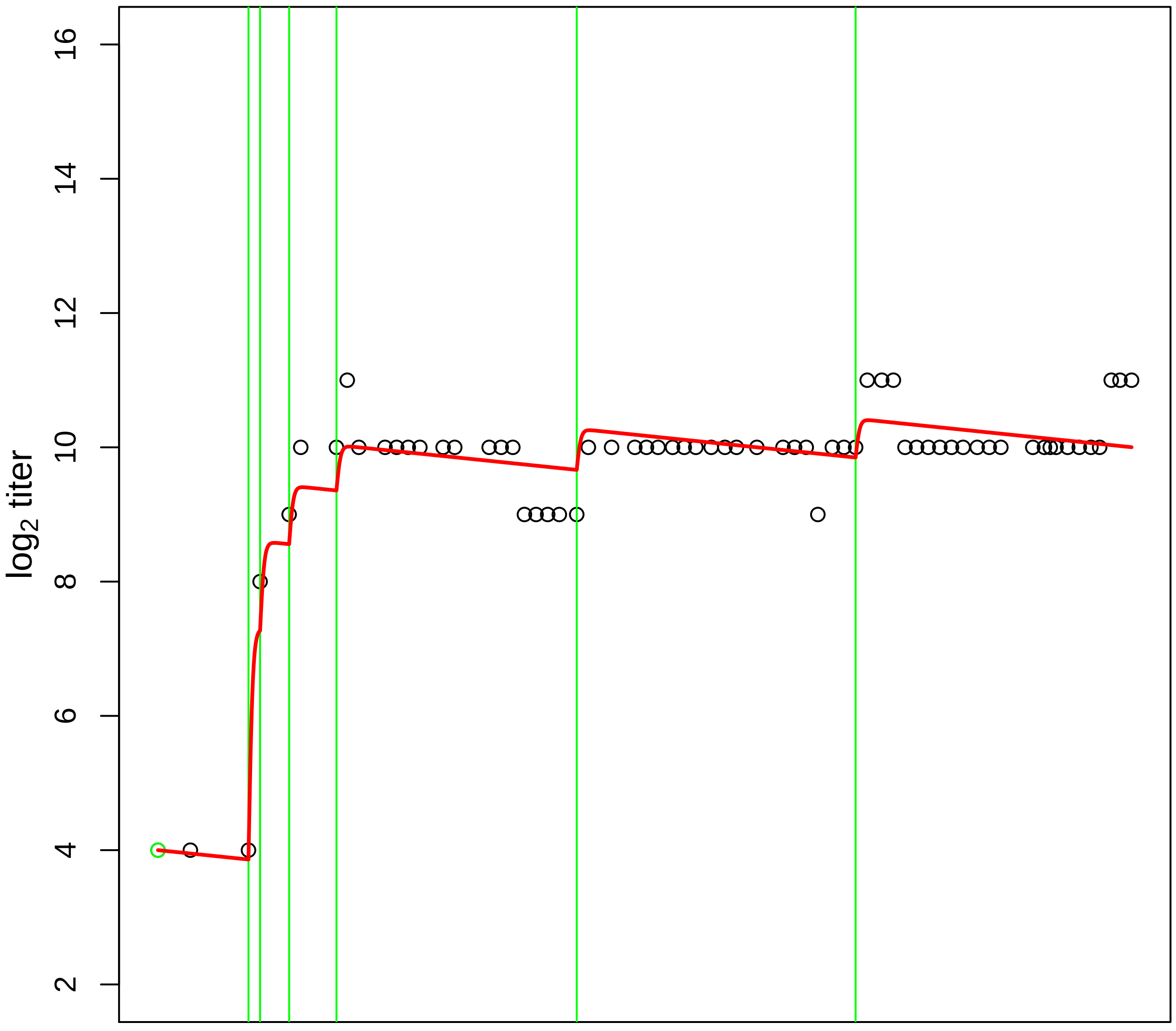

time in years from first donation of donor 73
mean absolute errors = 0.303 , mean squared errors = 0.175

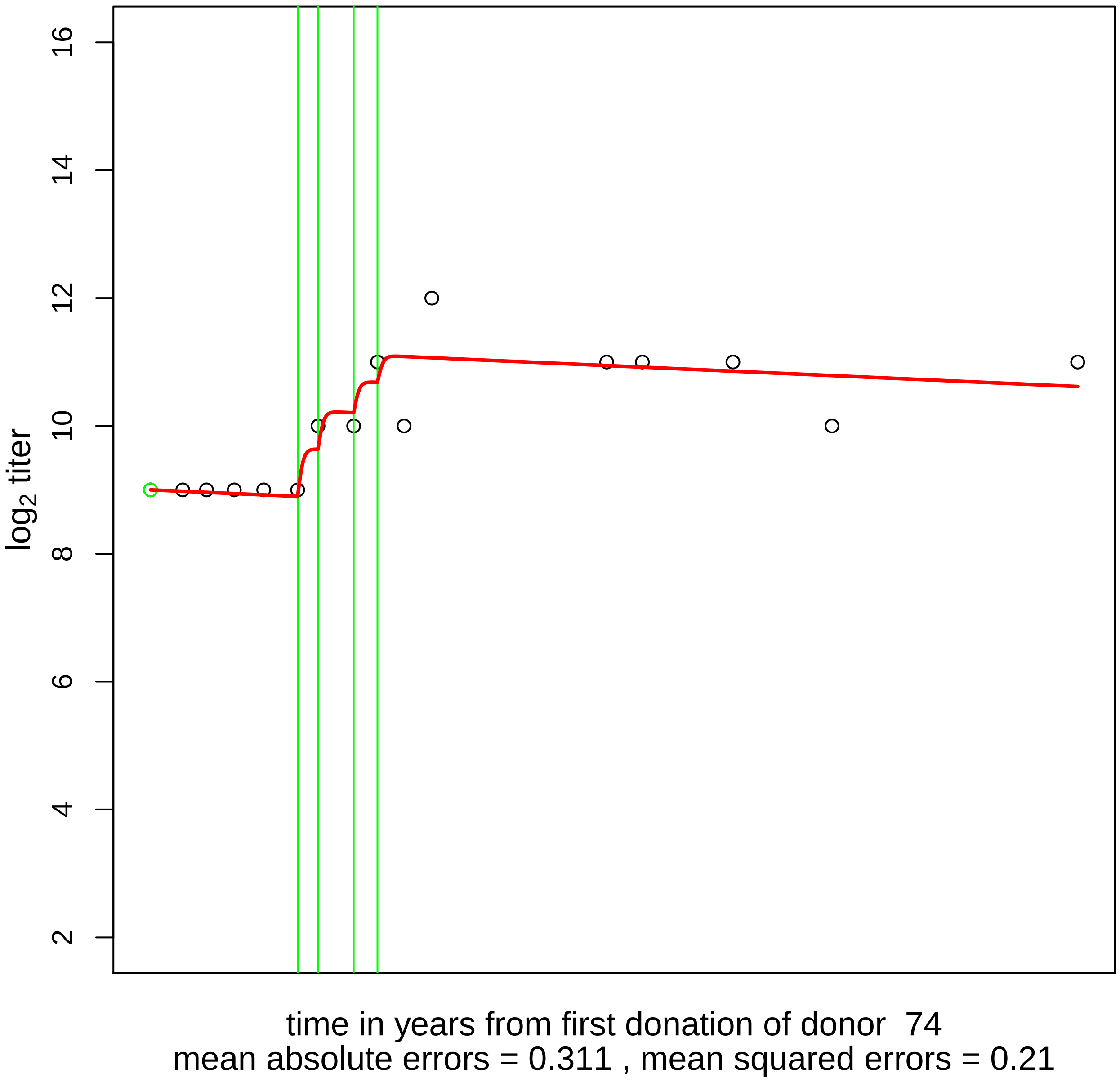

log2 titer
2
4
6
8
10
12
14
16
time in years from first donation of donor 74
mean absolute errors = 0.311 , mean squared errors = 0.21

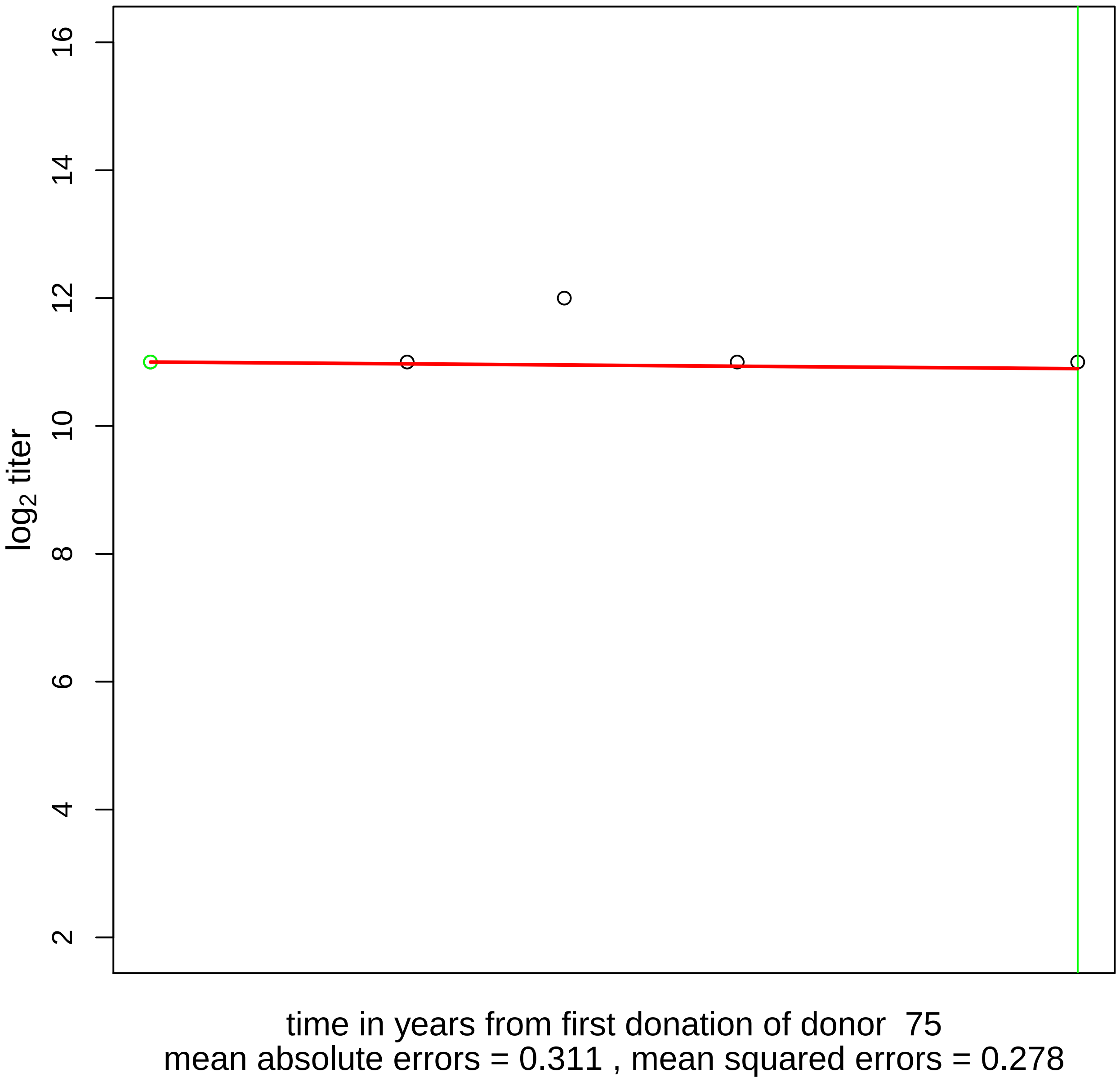

16
14
12
10
8
6
4
2
$\log_2$ titer
time in years from first donation of donor 75
mean absolute errors = 0.311 , mean squared errors = 0.278

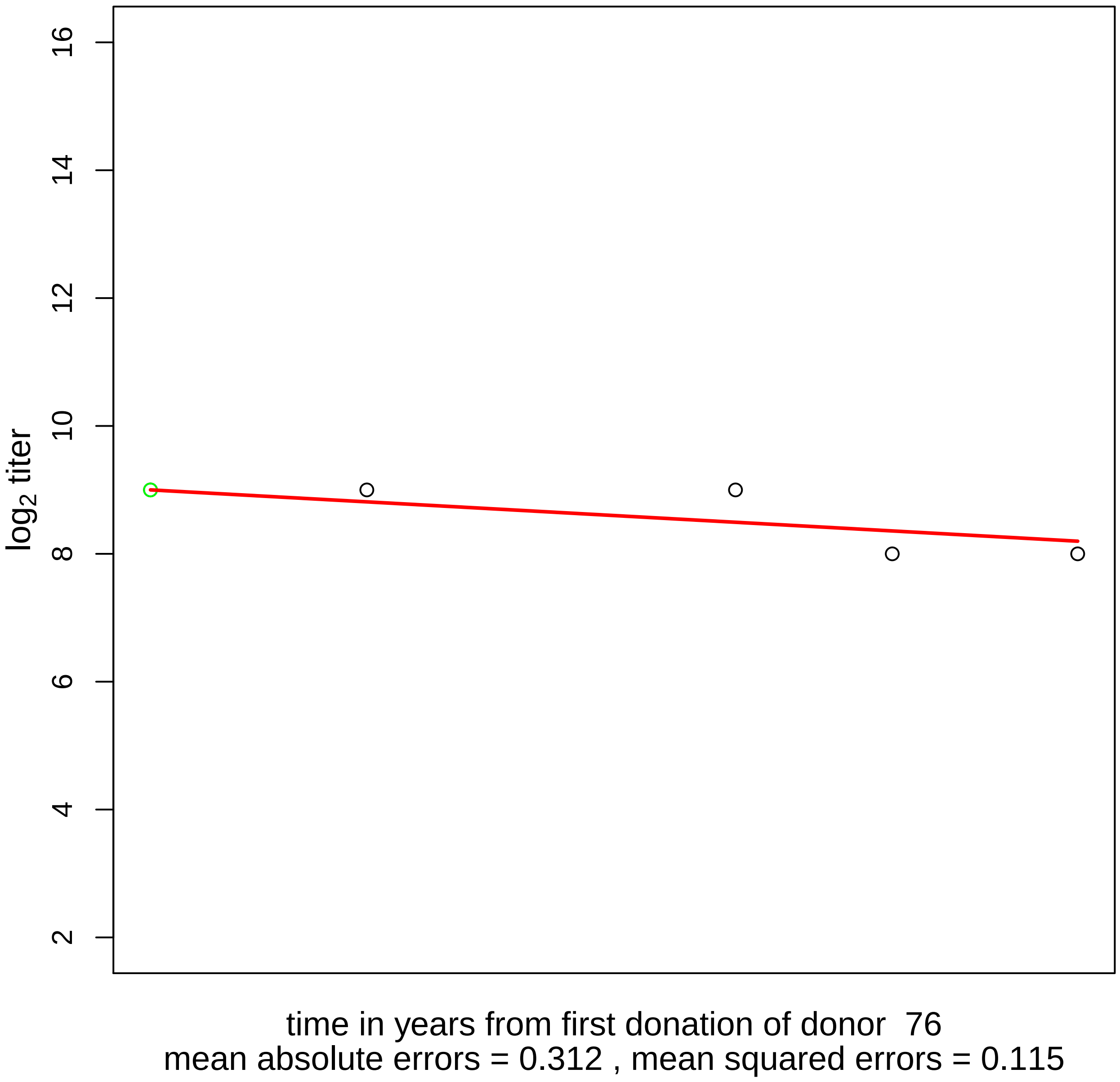

log2 titer
2
4
6
8
10
12
14
16
time in years from first donation of donor 76
mean absolute errors = 0.312 , mean squared errors = 0.115

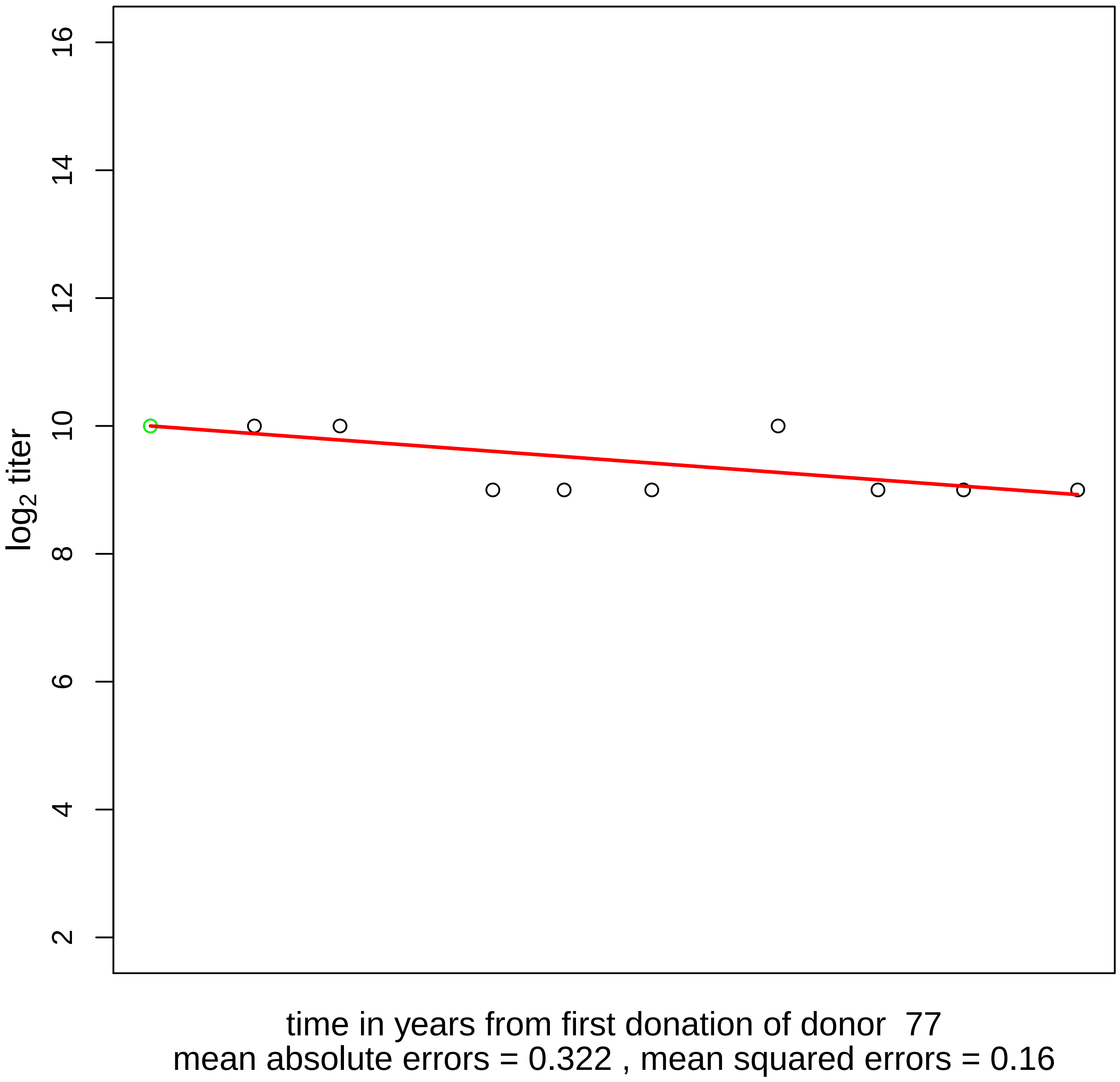

log2 titer
16
14
12
10
8
6
4
2
time in years from first donation of donor 77
mean absolute errors = 0.322 , mean squared errors = 0.16

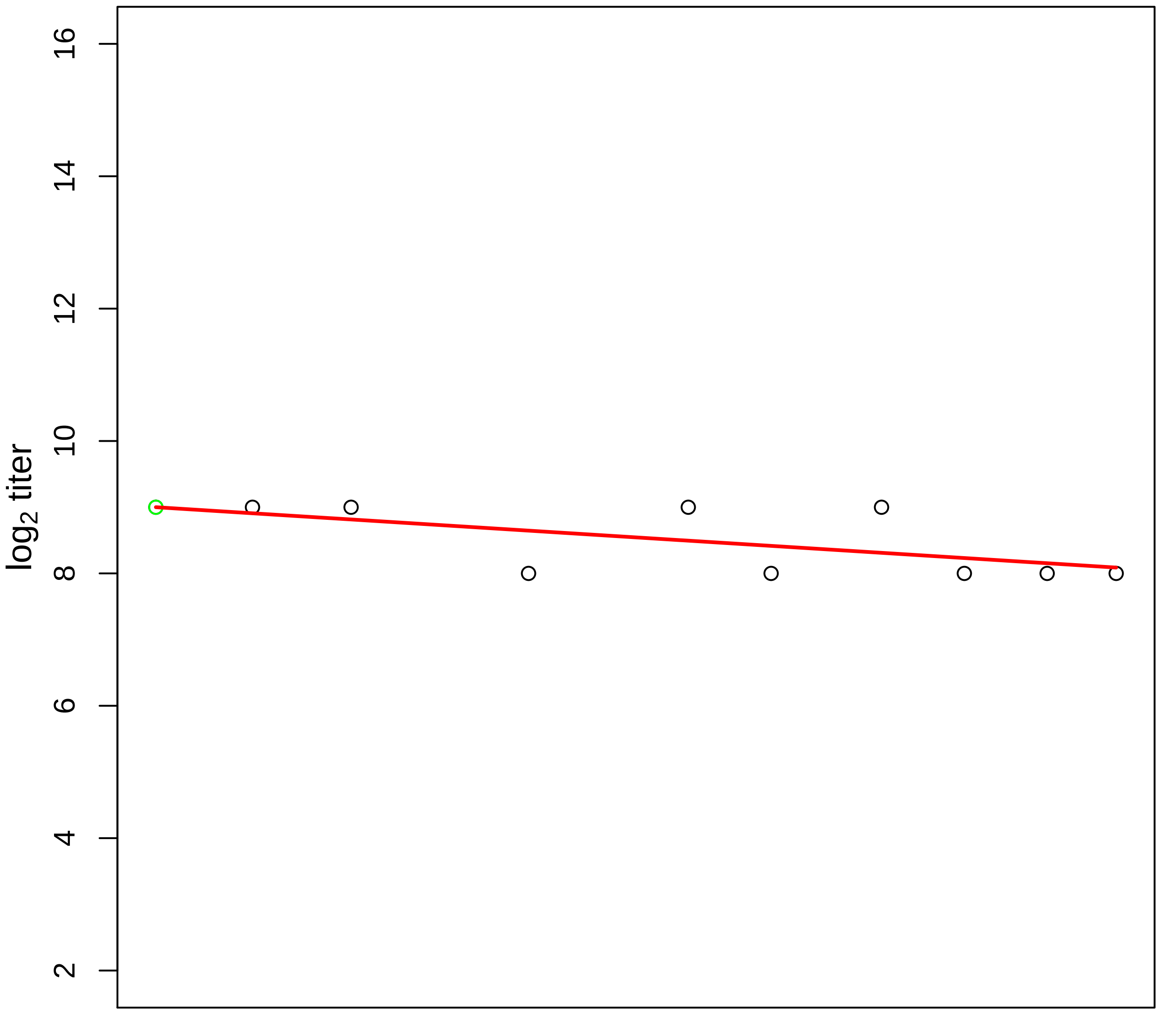

time in years from first donation of donor 78
mean absolute errors = 0.334 , mean squared errors = 0.161

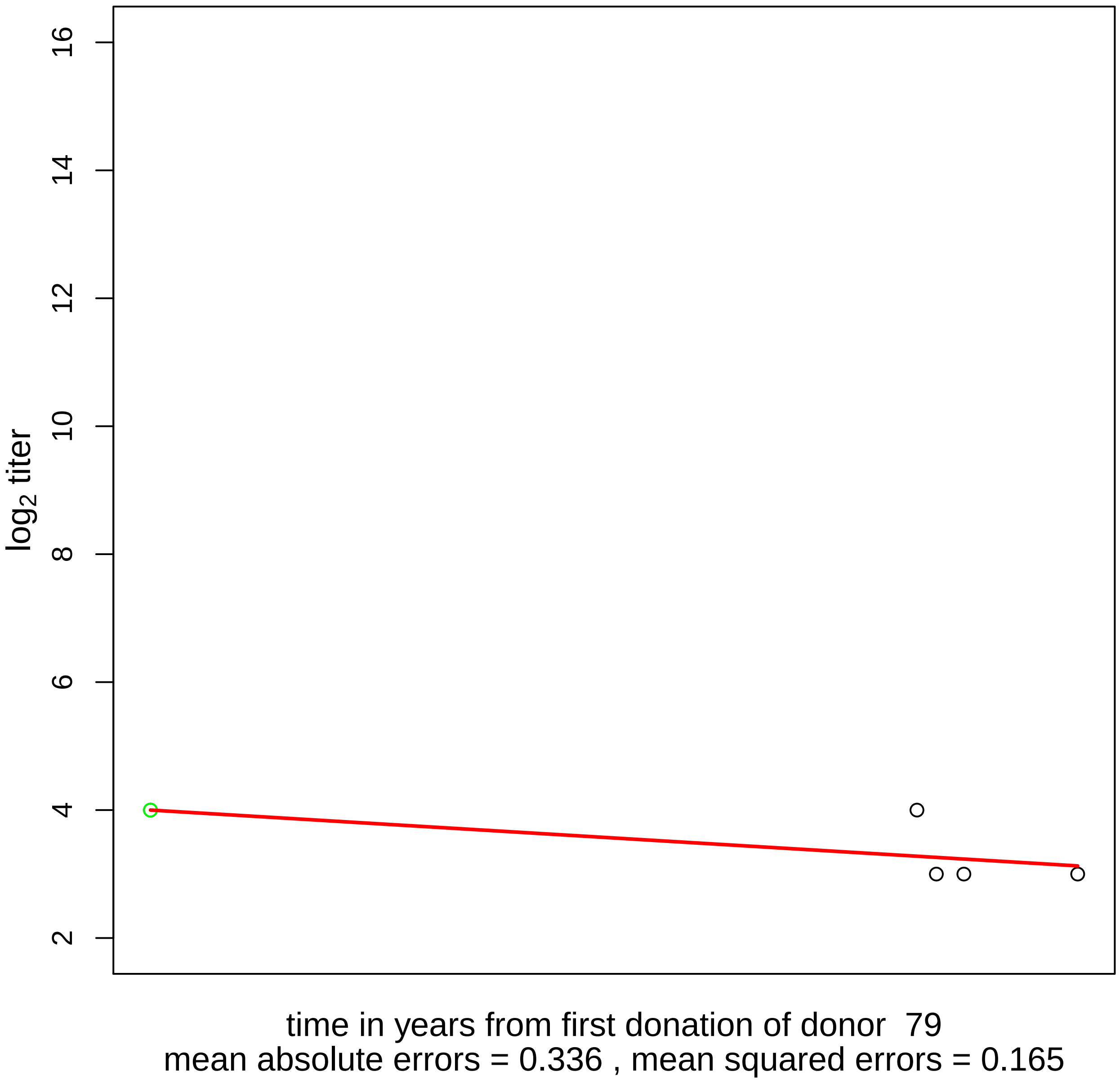
log2 titer
16
14
12
10
8
6
4
2
time in years from first donation of donor 79
mean absolute errors = 0.336 , mean squared errors = 0.165

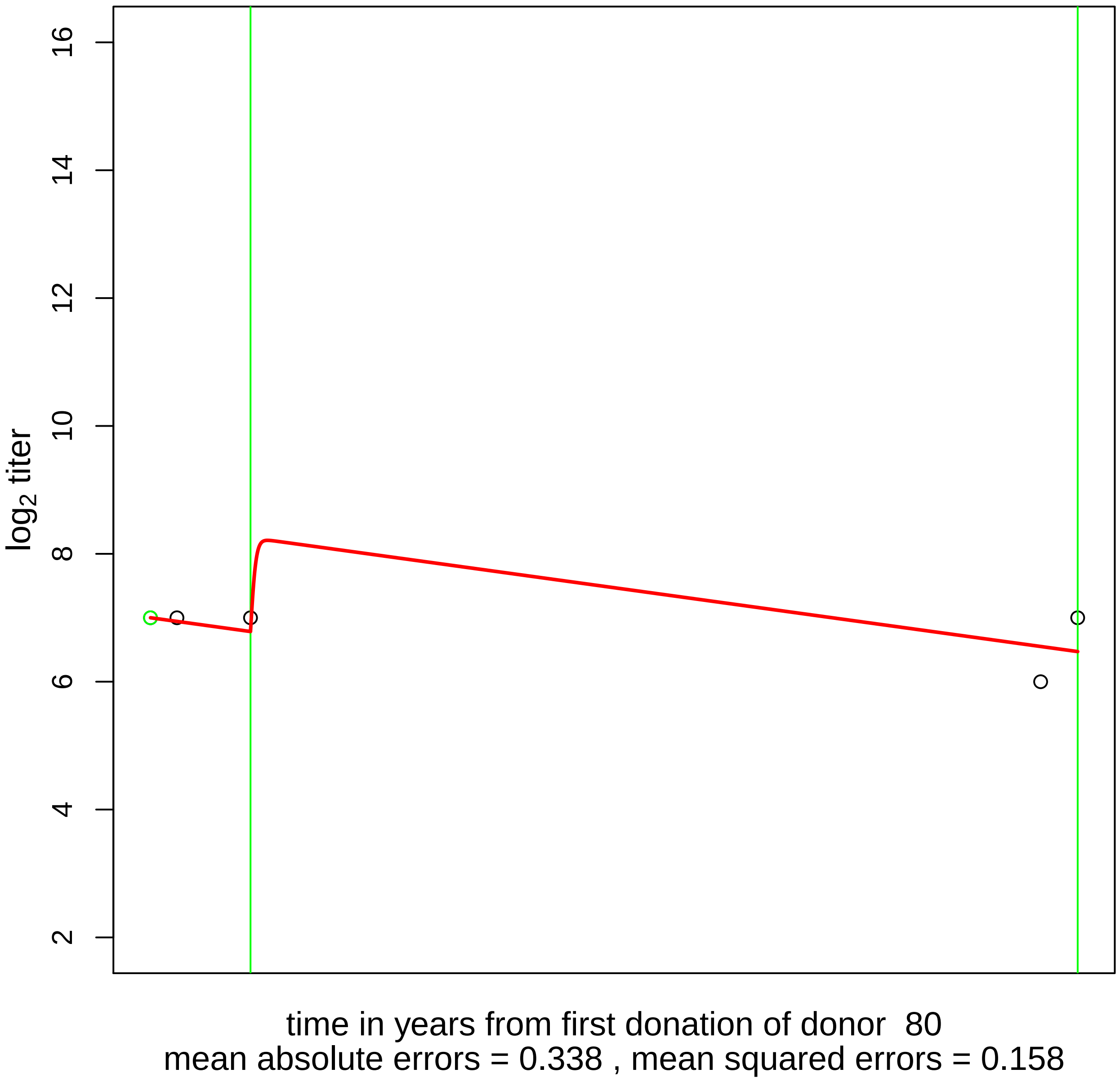

log2 titer
2
4
6
8
10
12
14
16
time in years from first donation of donor 80
mean absolute errors = 0.338 , mean squared errors = 0.158

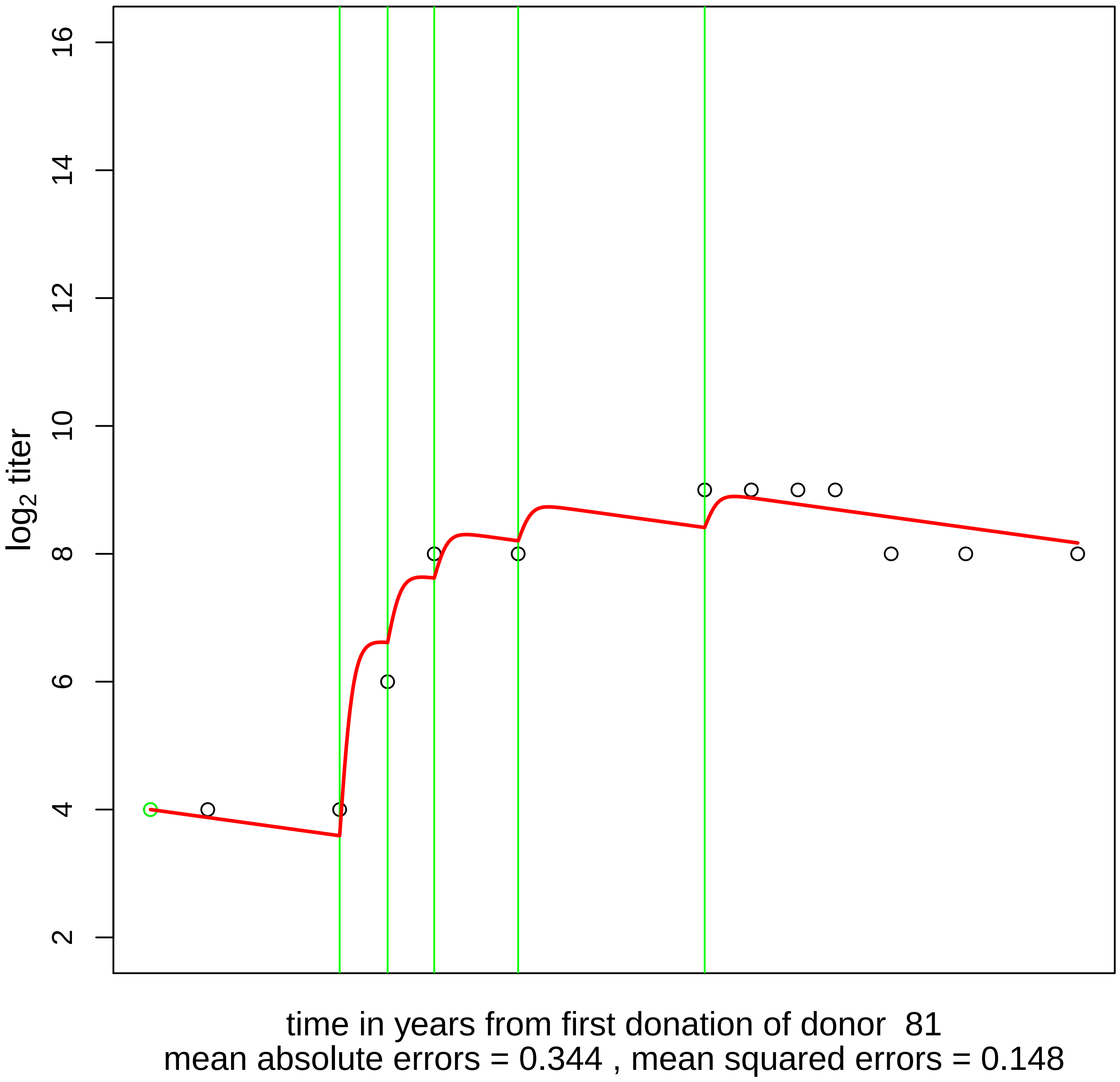
log2 titer
16
14
12
10
8
6
4
2
time in years from first donation of donor 81
mean absolute errors = 0.344 , mean squared errors = 0.148

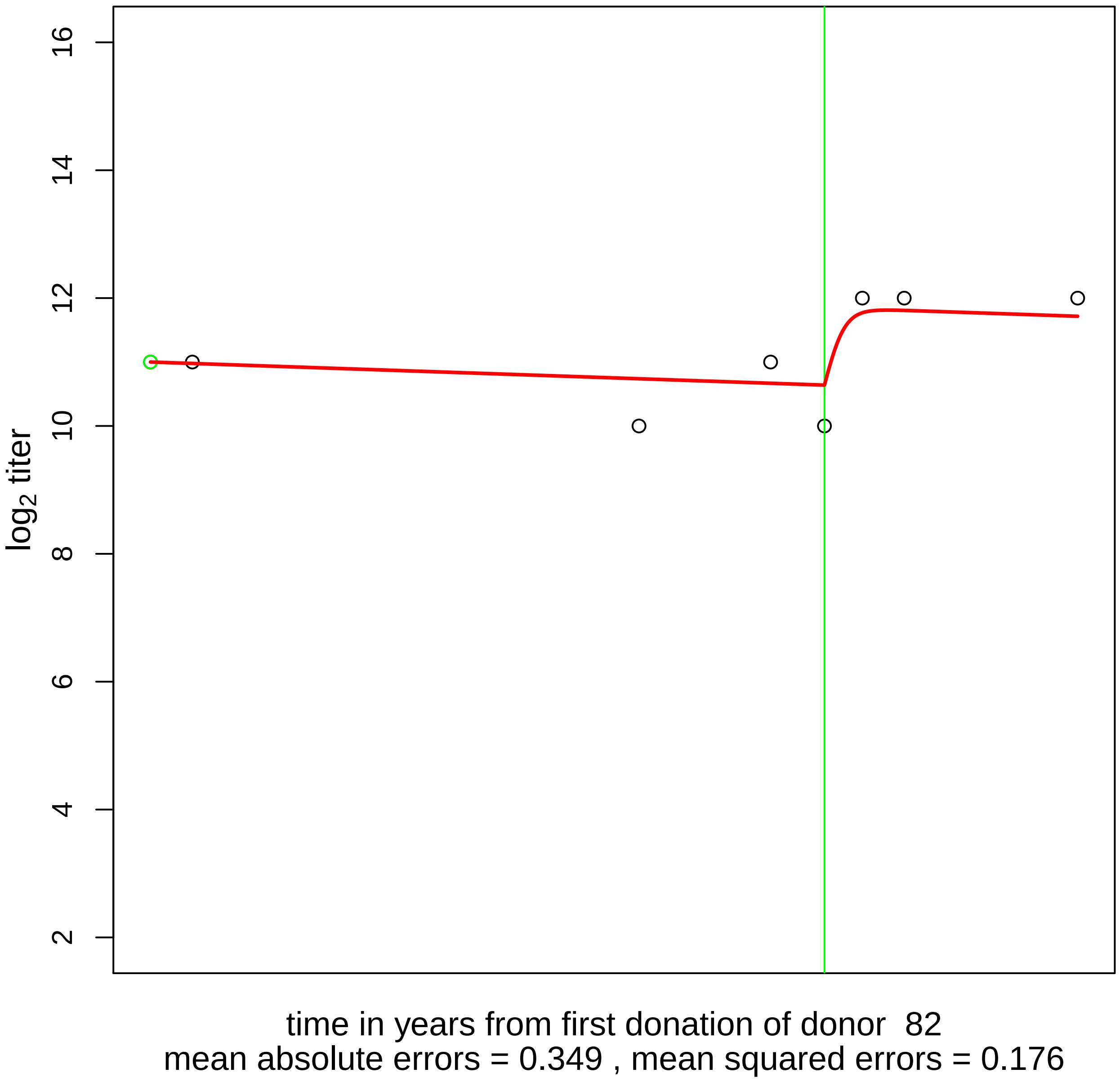

16
14
12
10
8
6
4
2
log2 titer
time in years from first donation of donor 82
mean absolute errors = 0.349 , mean squared errors = 0.176

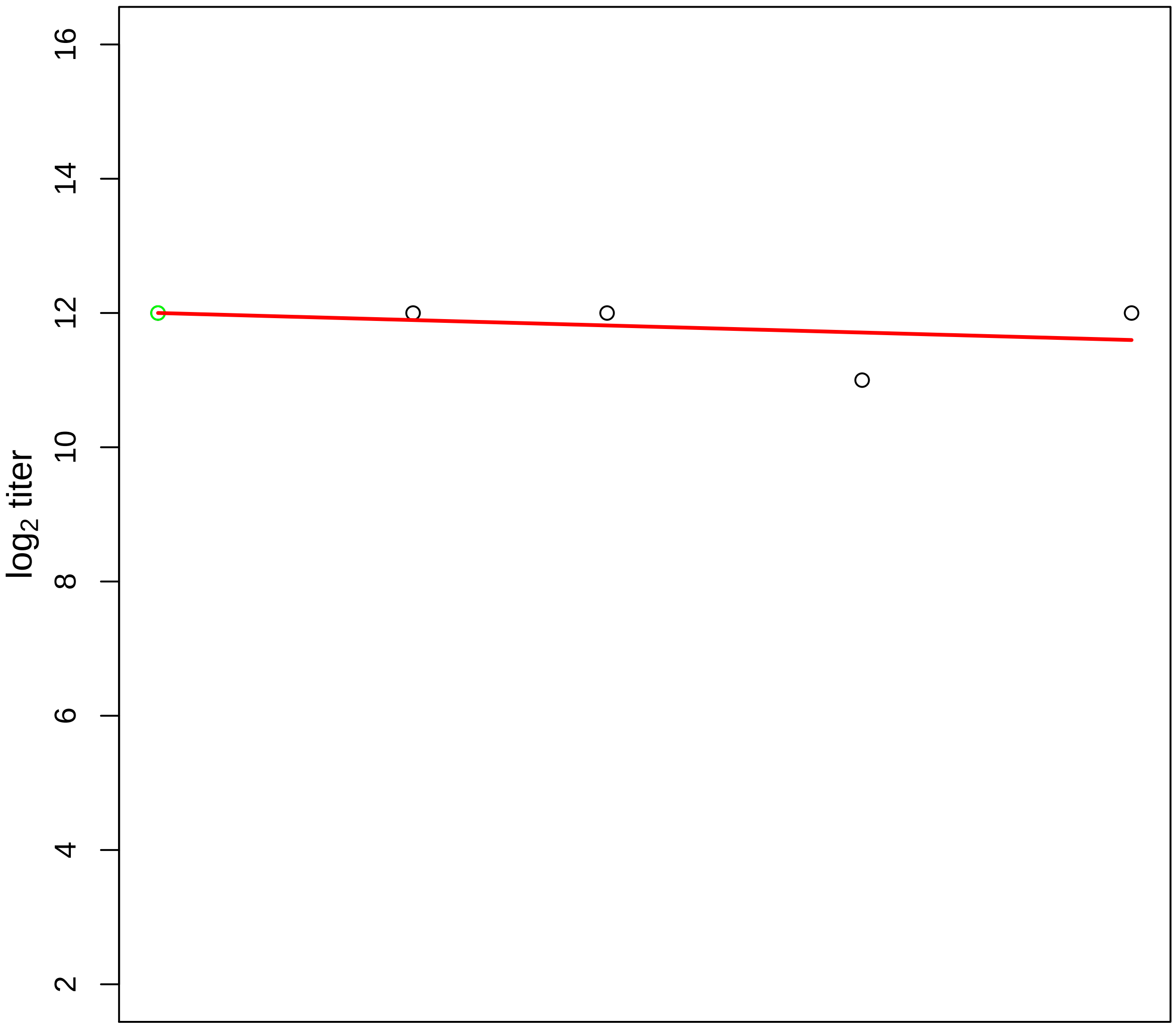

time in years from first donation of donor 83
mean absolute errors = 0.351 , mean squared errors = 0.178

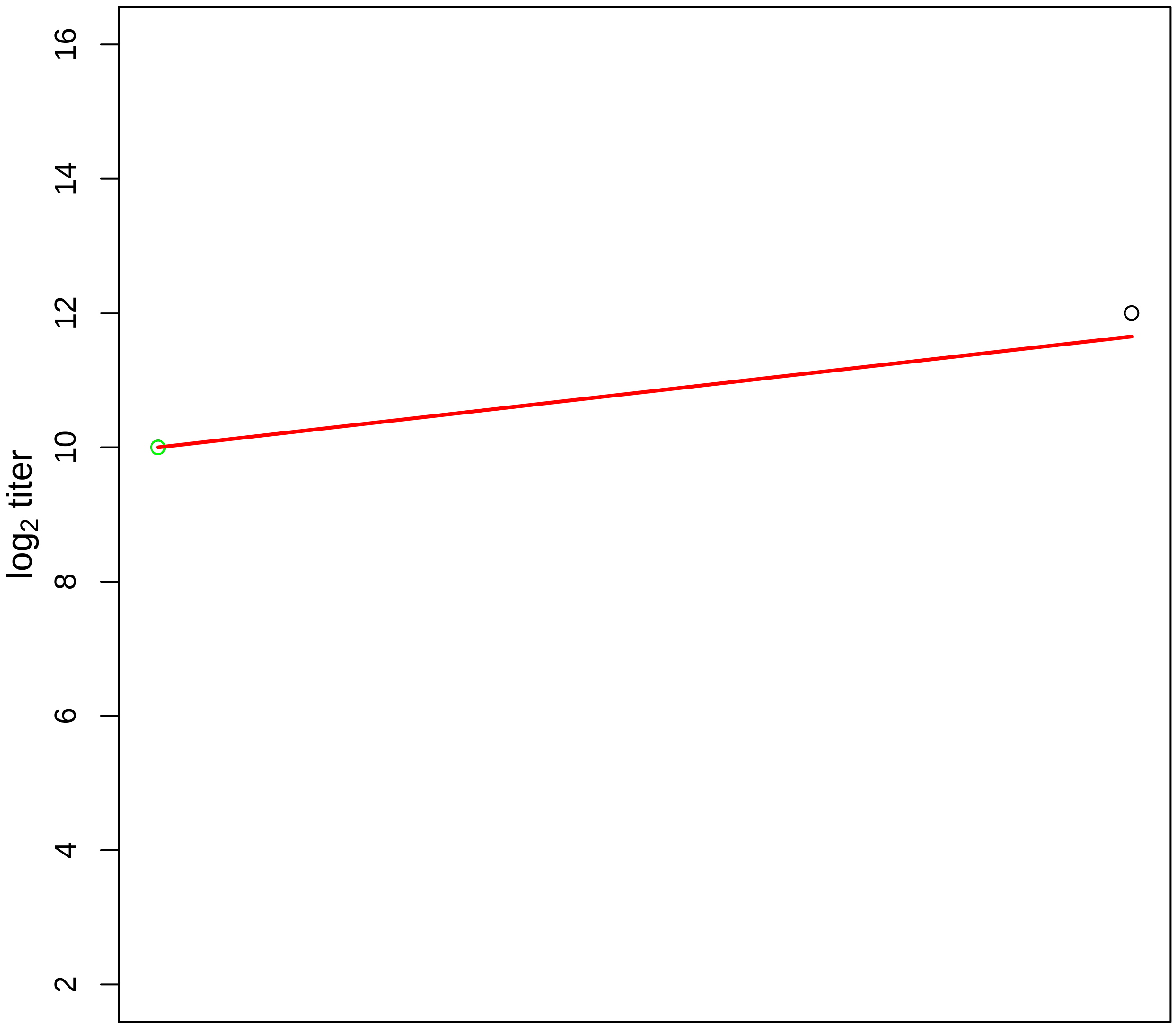

time in years from first donation of donor 84
mean absolute errors = 0.351 , mean squared errors = 0.123

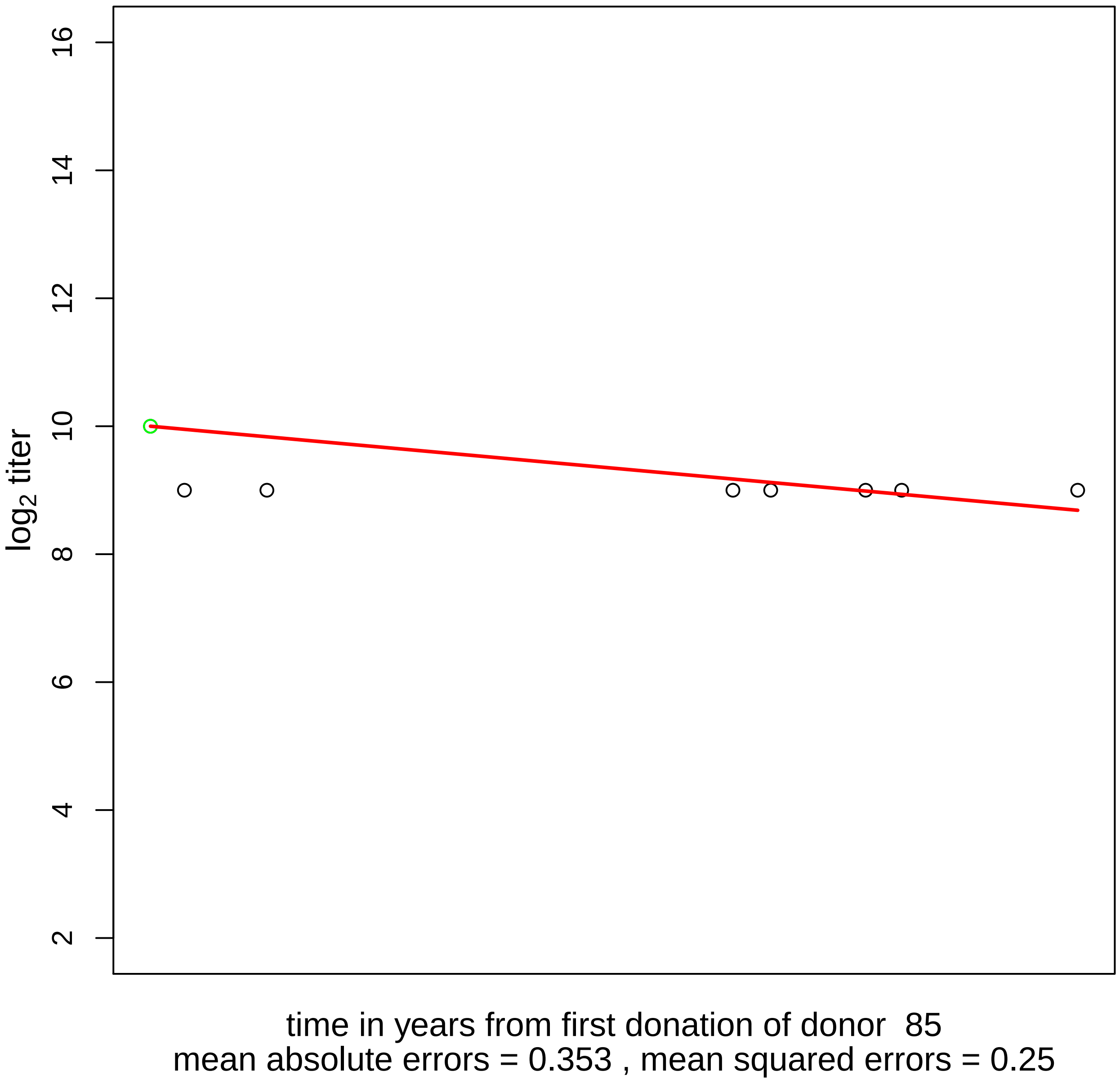
16
14
12
10
8
6
4
2
$\log_2$ titer
time in years from first donation of donor 85
mean absolute errors = 0.353 , mean squared errors = 0.25

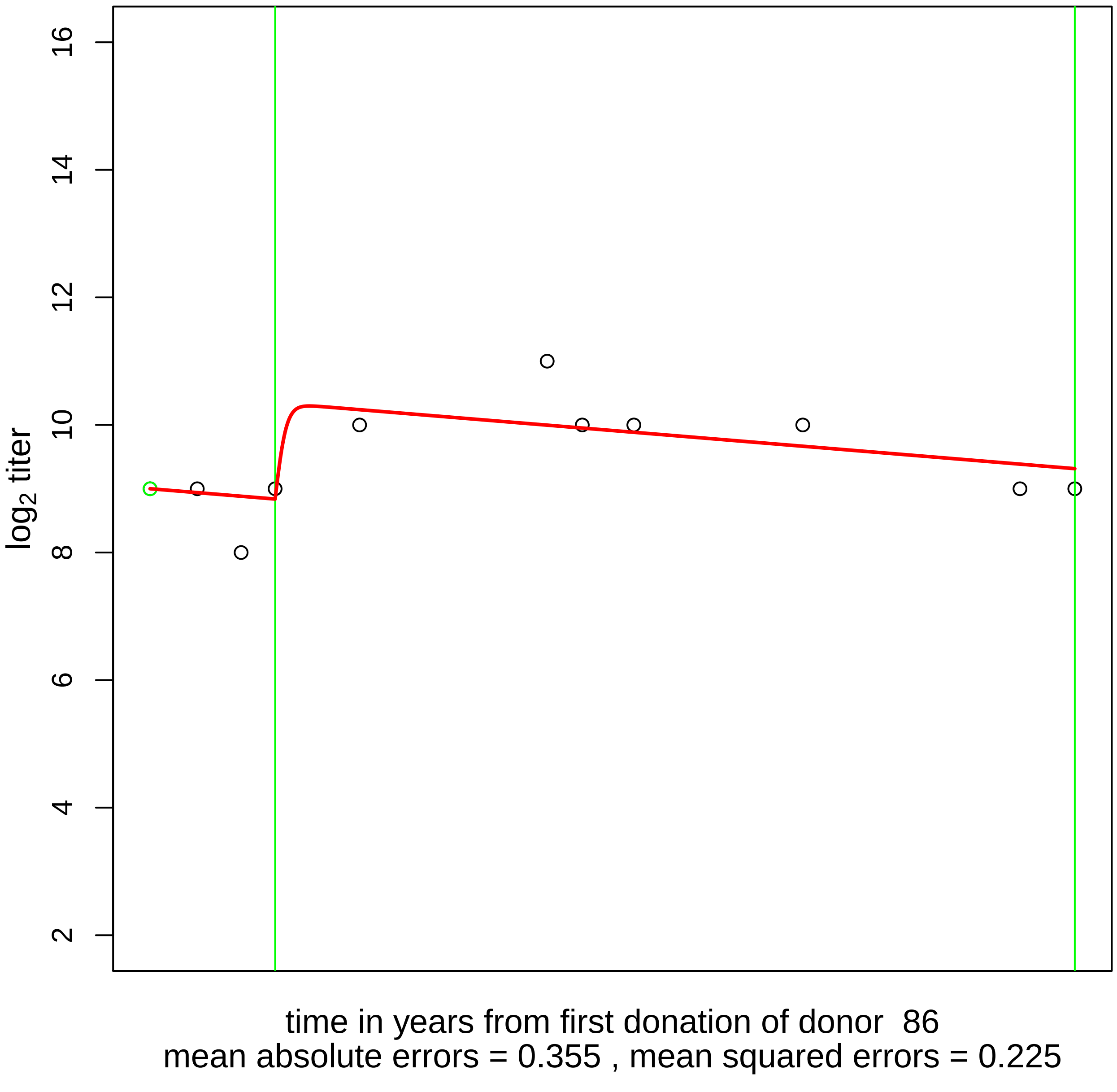

16
14
12
10
8
6
4
2
$\log_2$ titer
time in years from first donation of donor 86
mean absolute errors = 0.355 , mean squared errors = 0.225

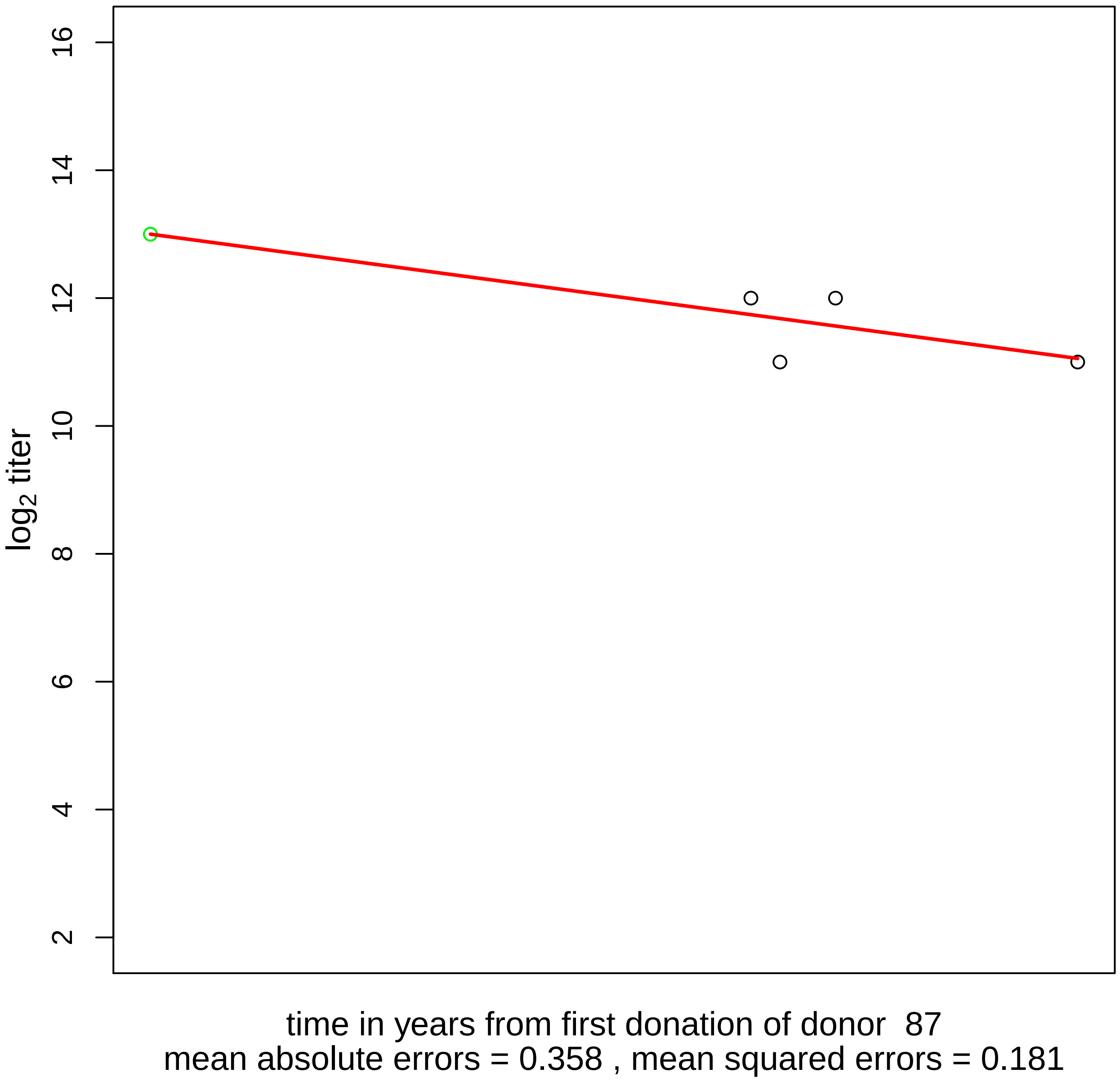

16
14
12
10
8
6
4
2
log2 titer
time in years from first donation of donor 87
mean absolute errors = 0.358 , mean squared errors = 0.181

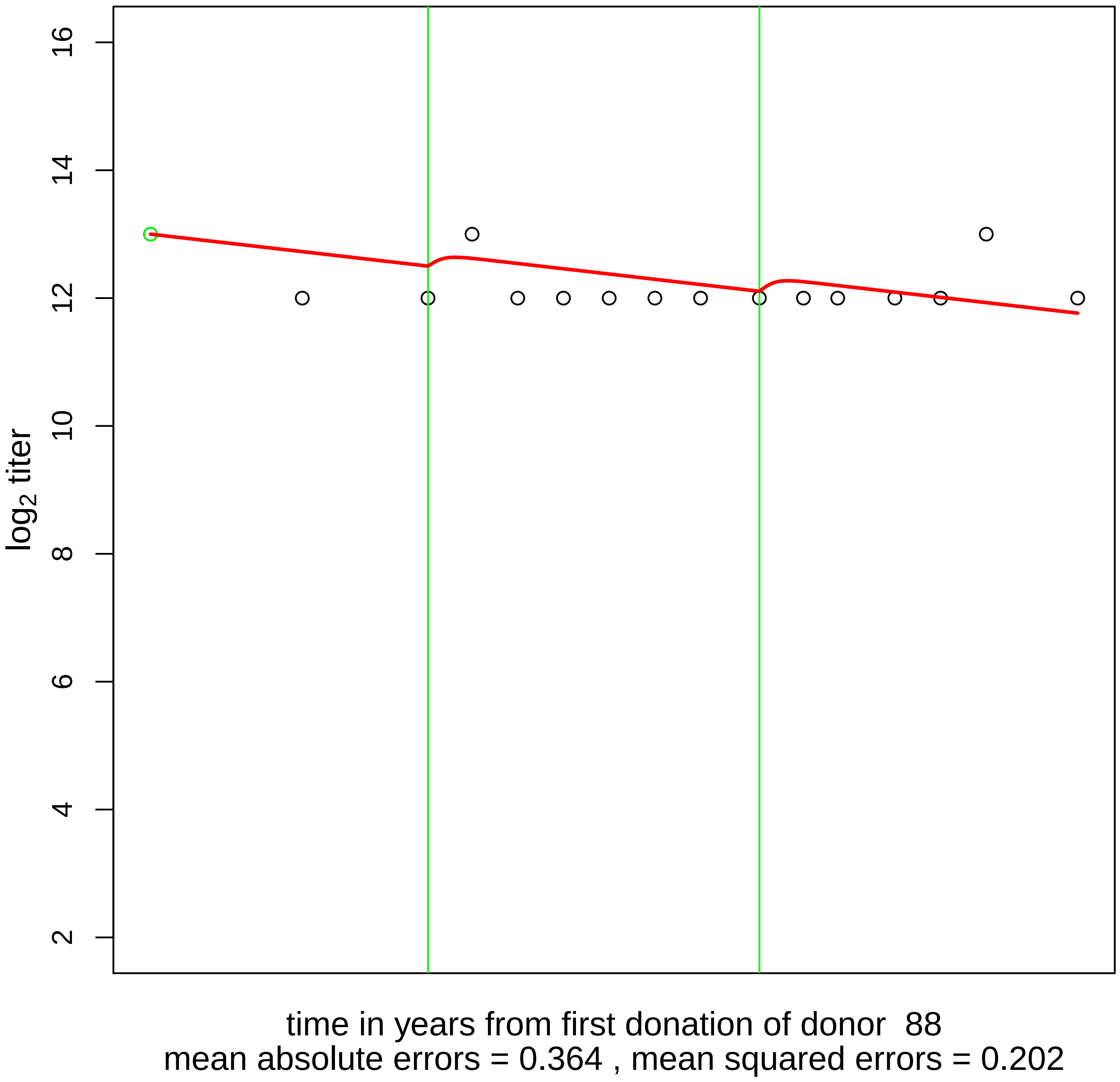

16
14
12
10
8
6
4
2
log2 titer
time in years from first donation of donor 88
mean absolute errors = 0.364 , mean squared errors = 0.202

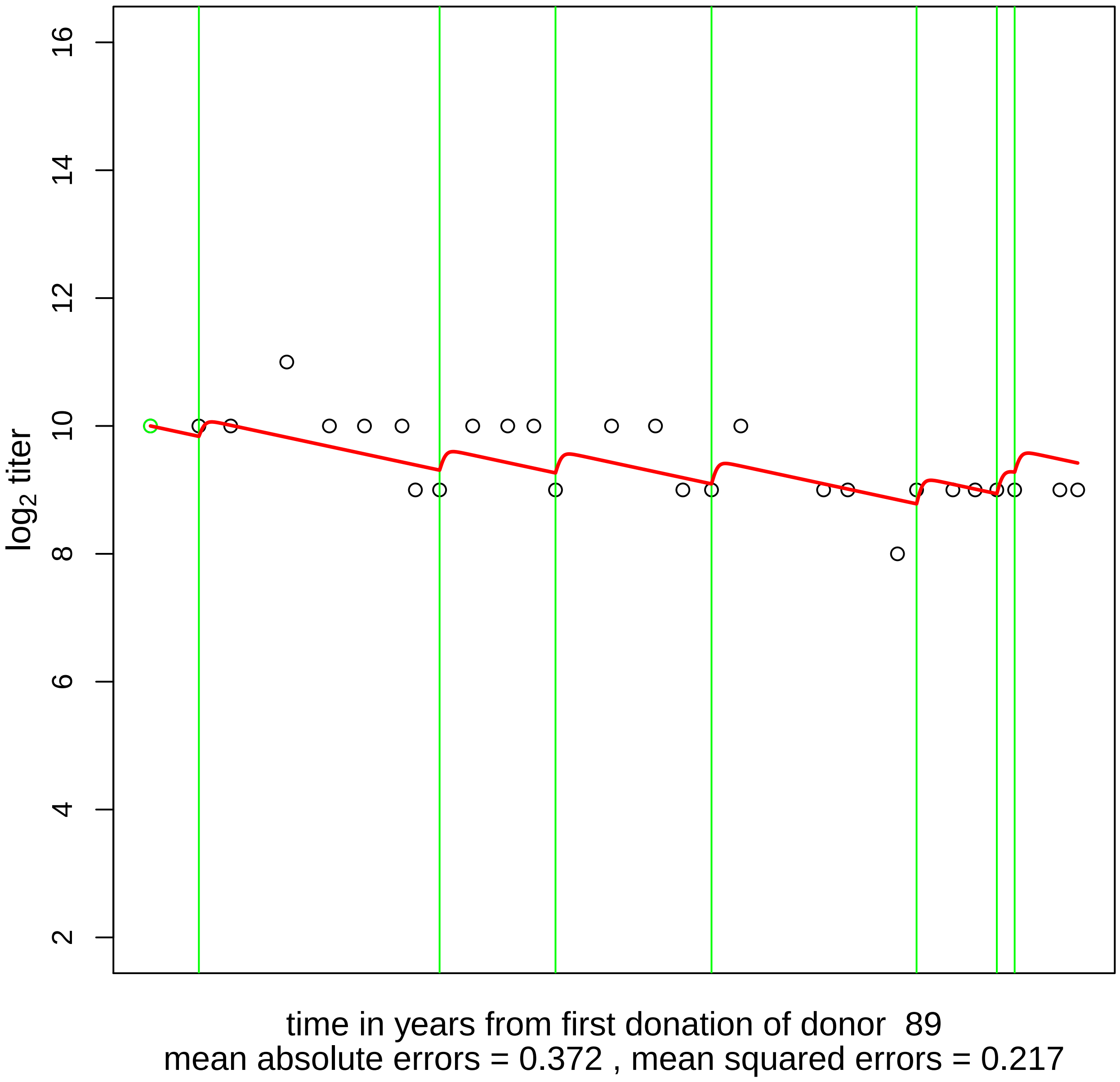

log2 titer
2
4
6
8
10
12
14
16
time in years from first donation of donor 89
mean absolute errors = 0.372 , mean squared errors = 0.217

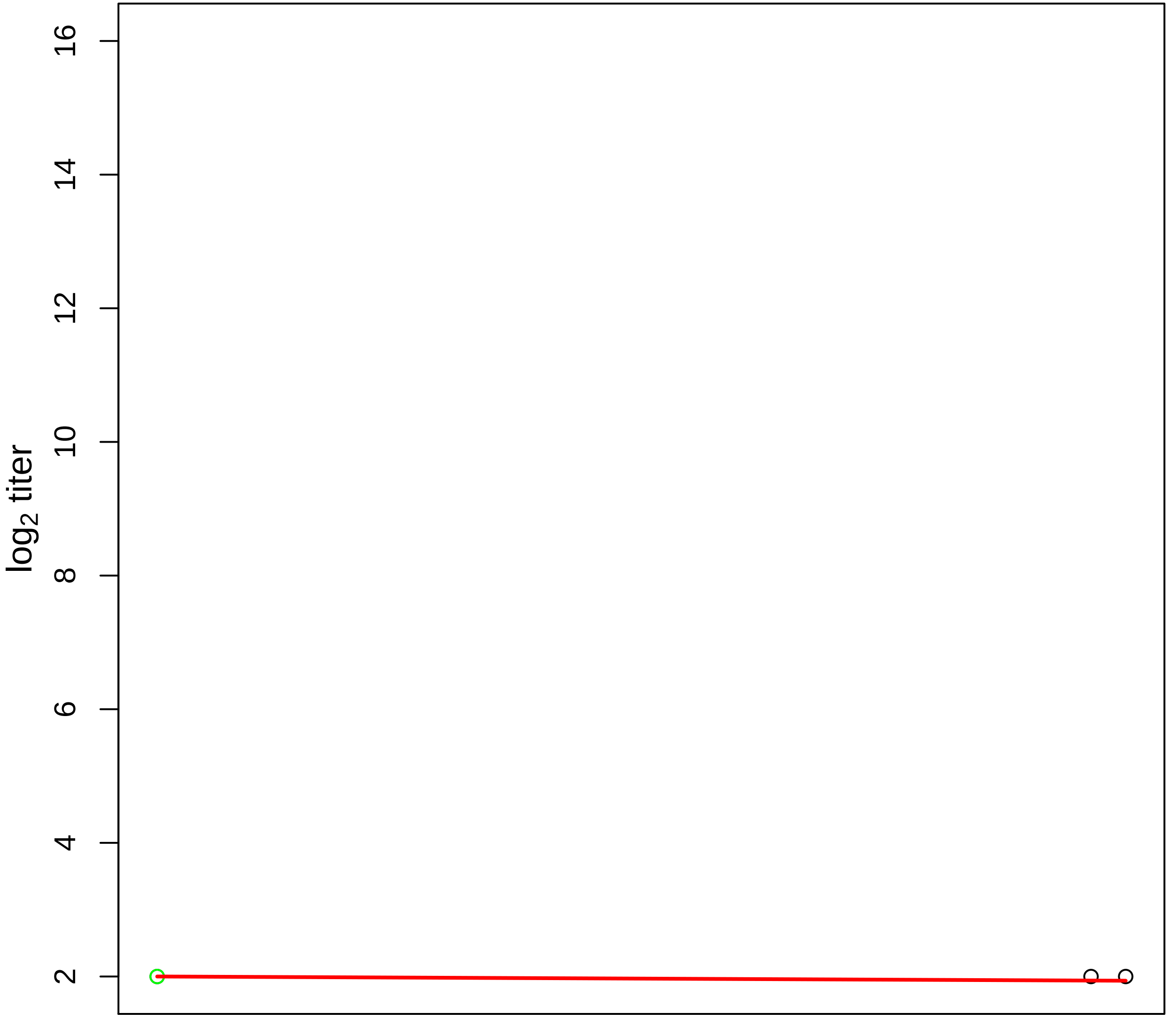

time in years from first donation of donor 90
mean absolute errors = 0.374 , mean squared errors = 0.335

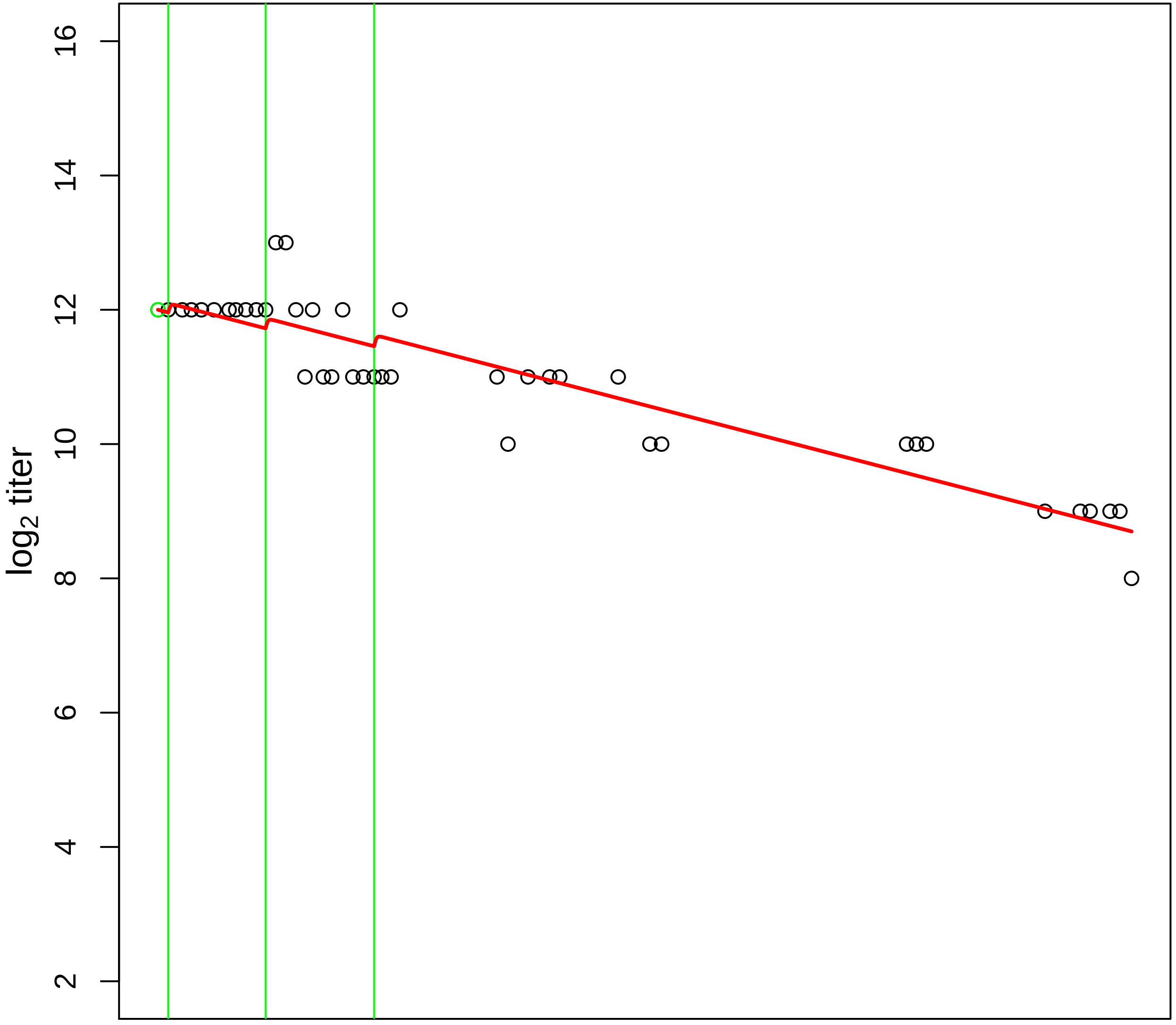

time in years from first donation of donor 91
mean absolute errors = 0.375 , mean squared errors = 0.234

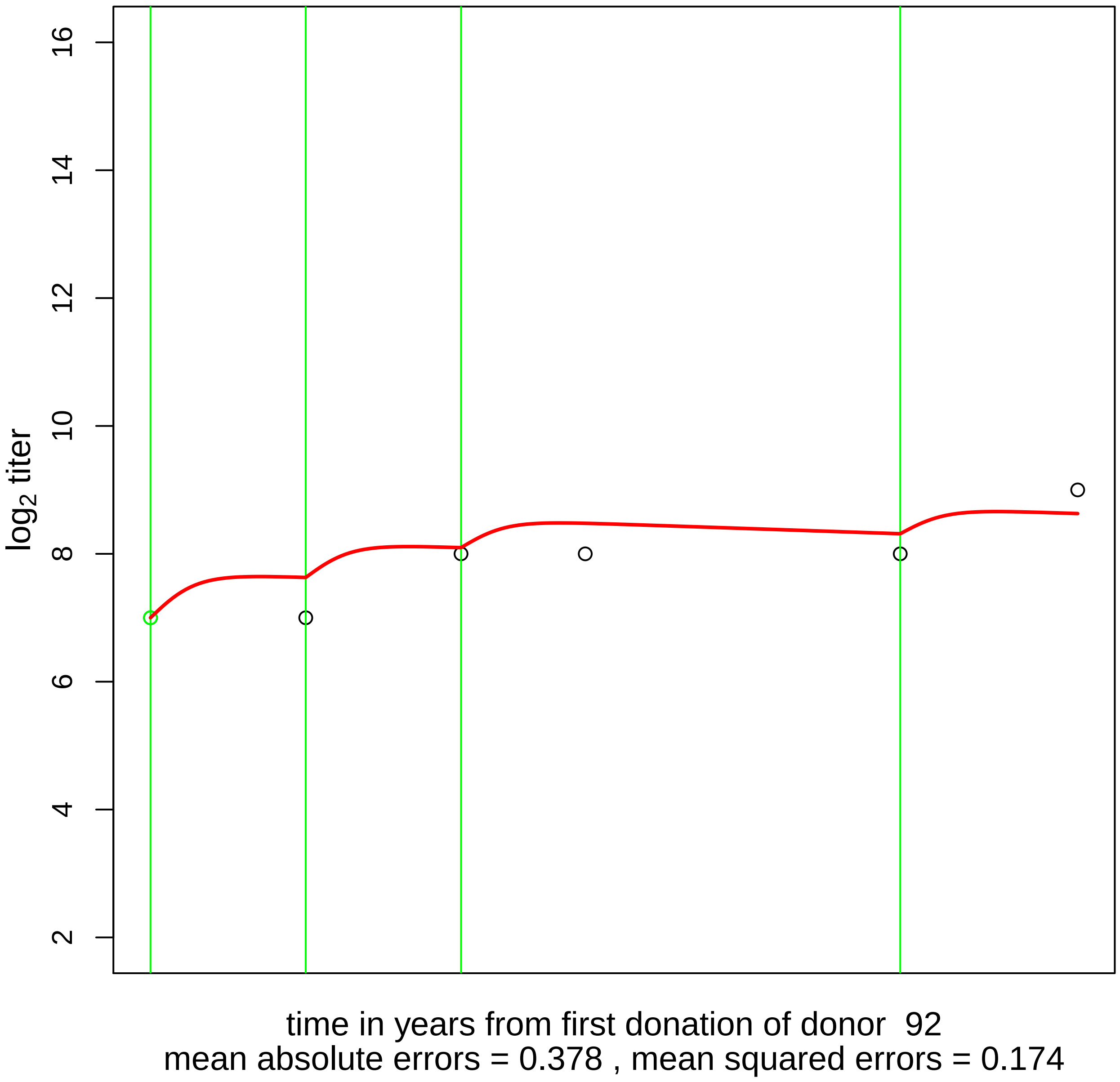

log2 titer
16
14
12
10
8
6
4
2
time in years from first donation of donor 92
mean absolute errors = 0.378 , mean squared errors = 0.174

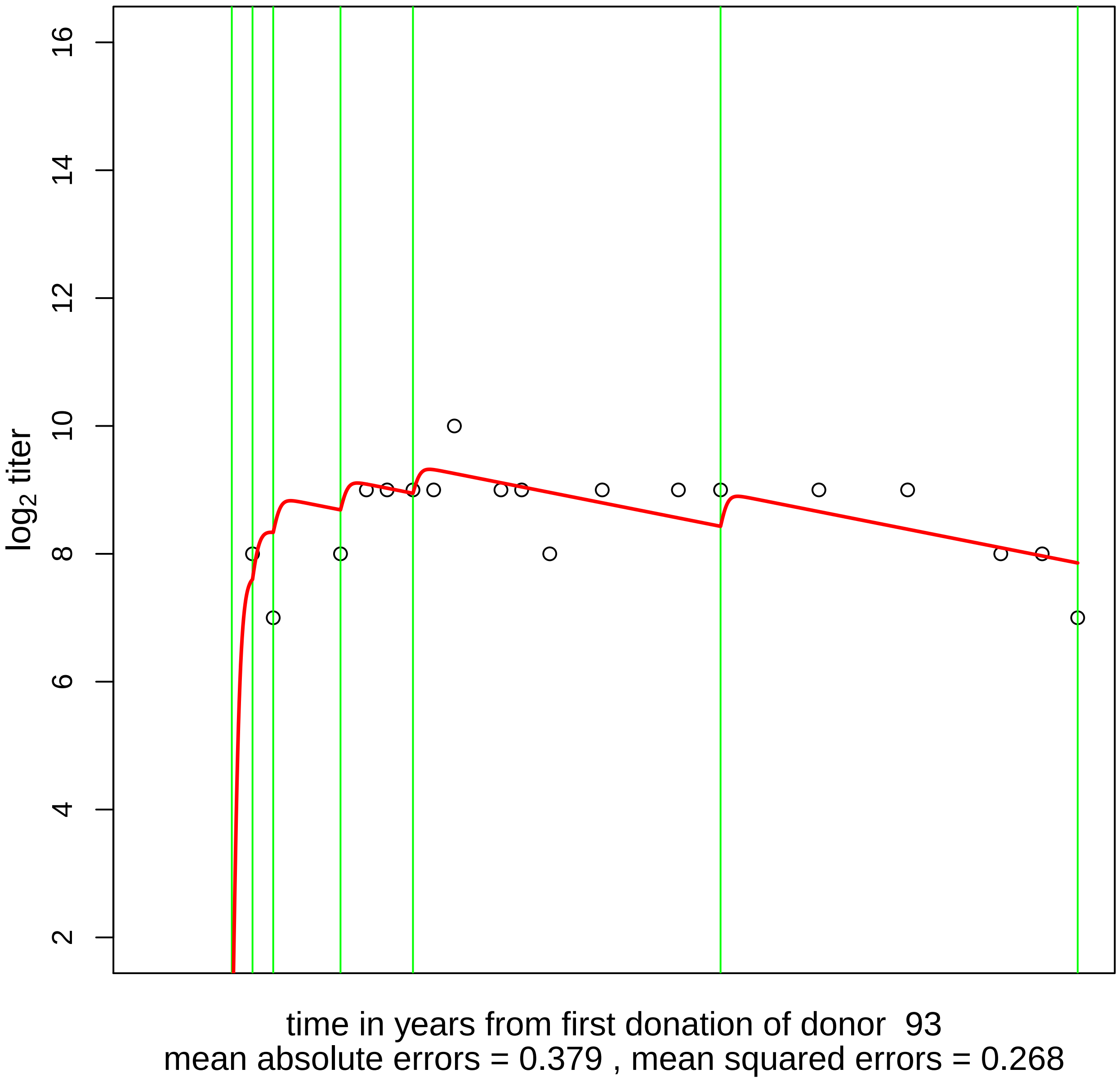

log2 titer
16
14
12
10
8
6
4
2
time in years from first donation of donor 93
mean absolute errors = 0.379 , mean squared errors = 0.268

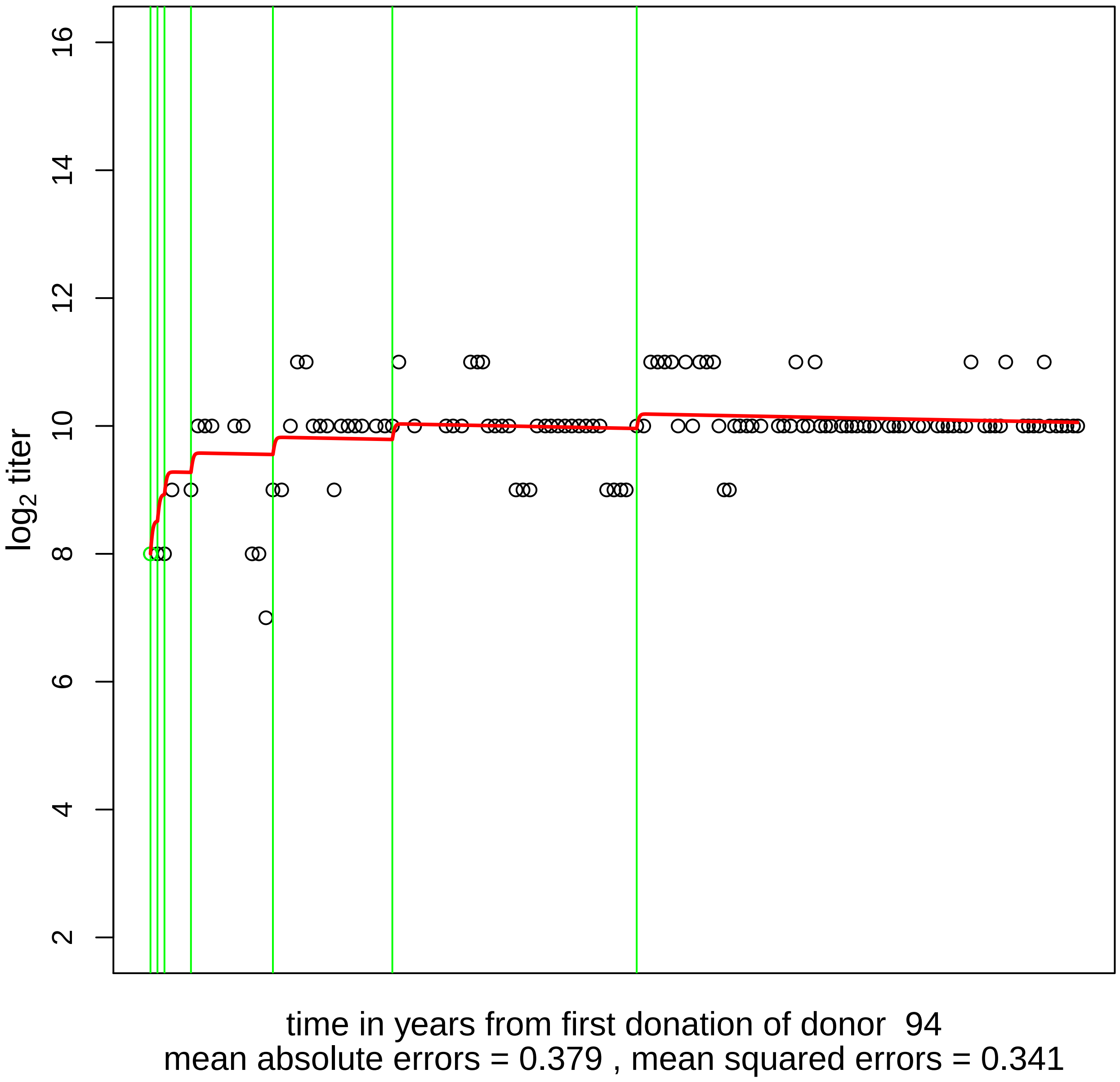

2
4
6
8
10
12
14
16
$\log_2$ titer
time in years from first donation of donor 94
mean absolute errors = 0.379 , mean squared errors = 0.341

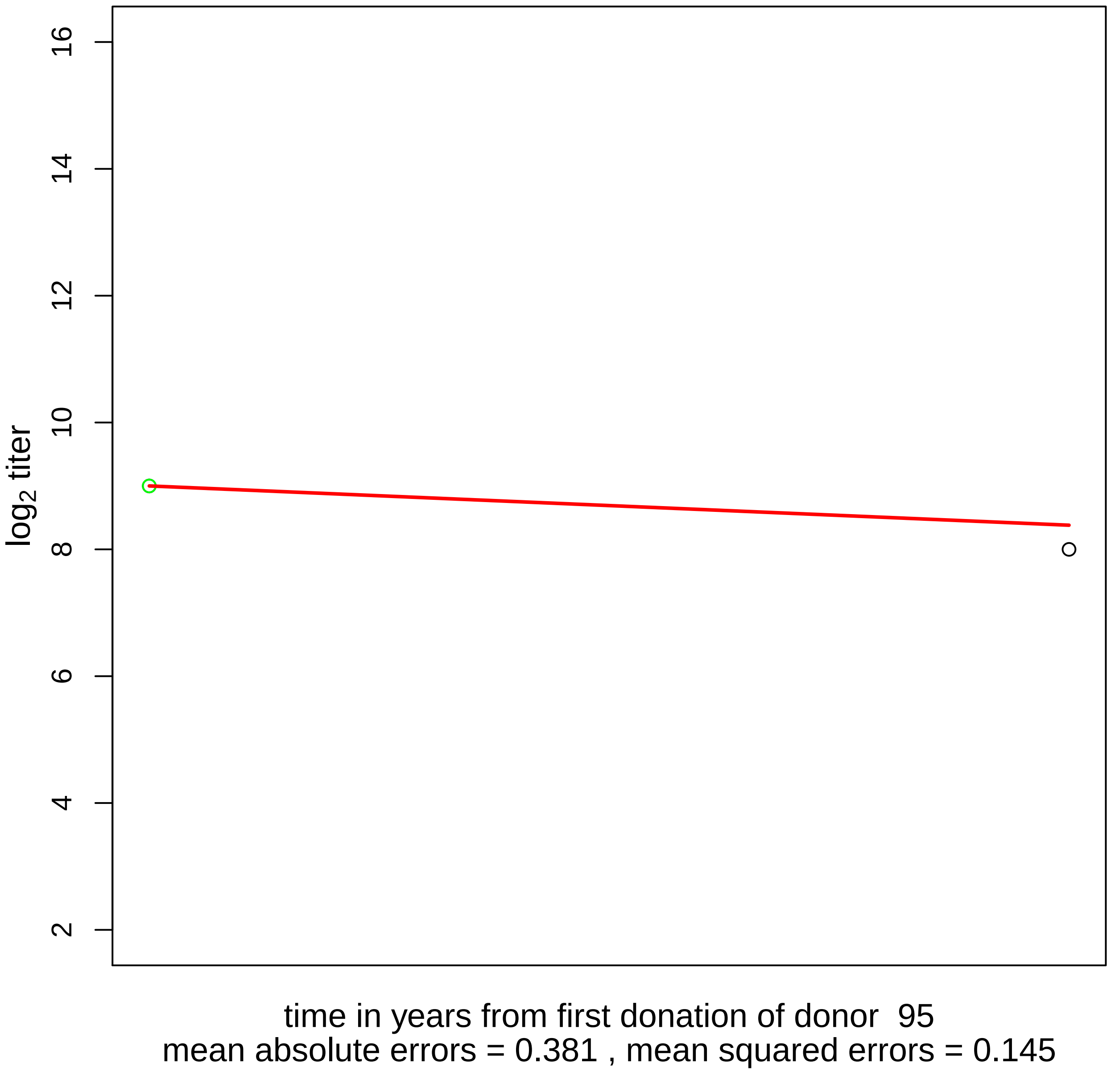

16
14
12
10
8
6
4
2
$\log_2$ titer
time in years from first donation of donor 95
mean absolute errors = 0.381 , mean squared errors = 0.145

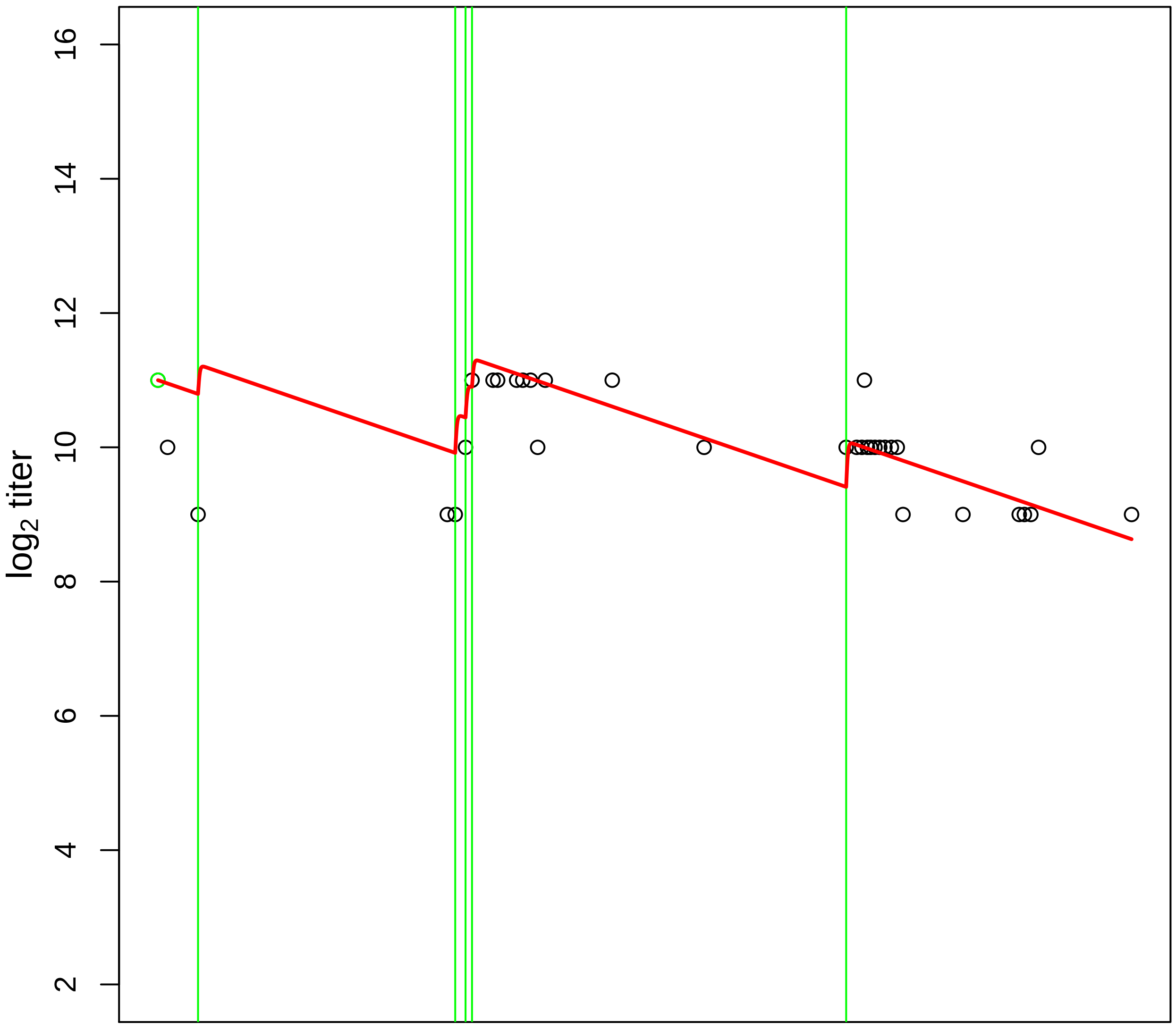

time in years from first donation of donor 96
mean absolute errors = 0.384 , mean squared errors = 0.324

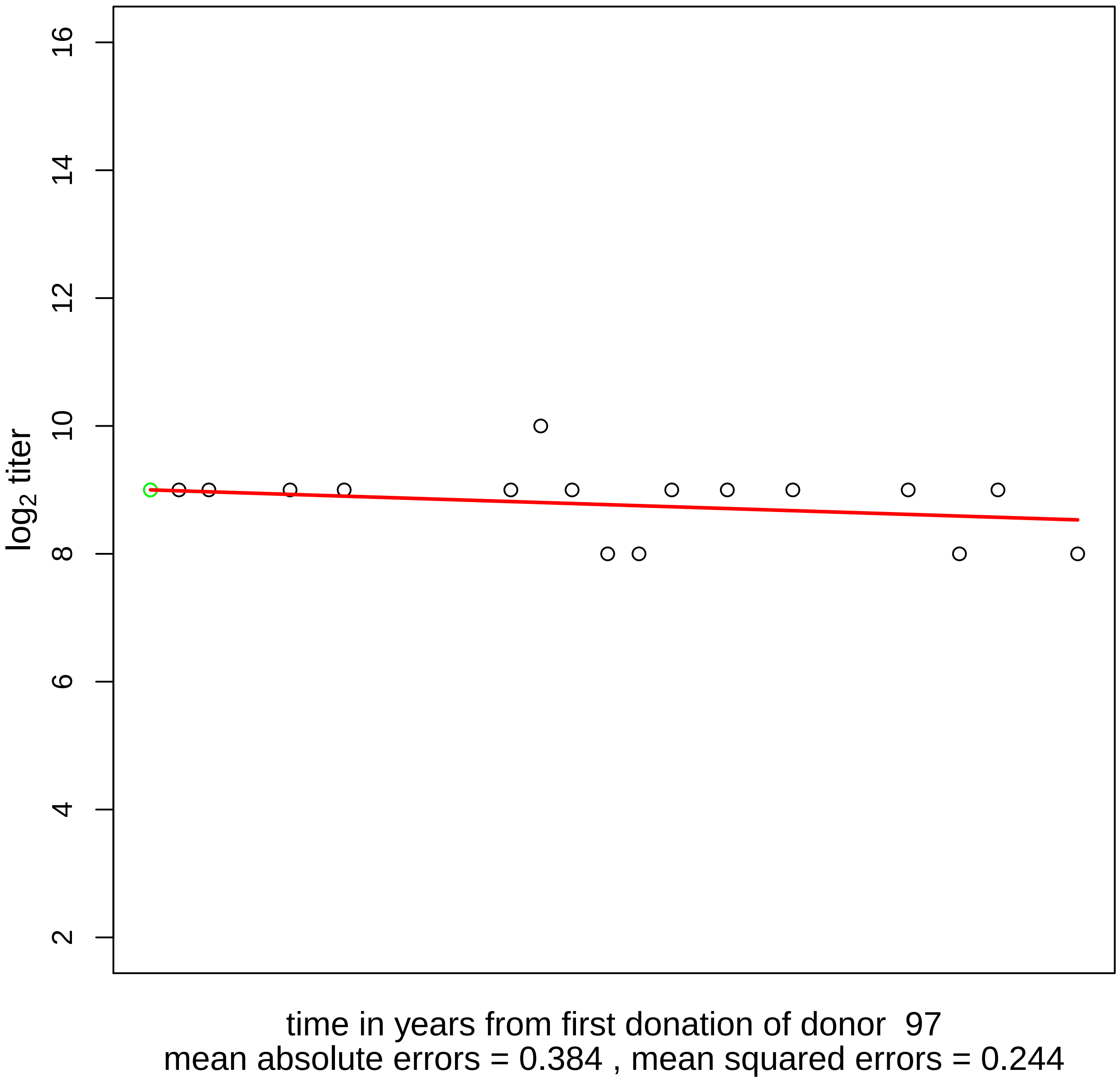
16
14
12
10
8
6
4
2
log2 titer
time in years from first donation of donor 97
mean absolute errors = 0.384 , mean squared errors = 0.244

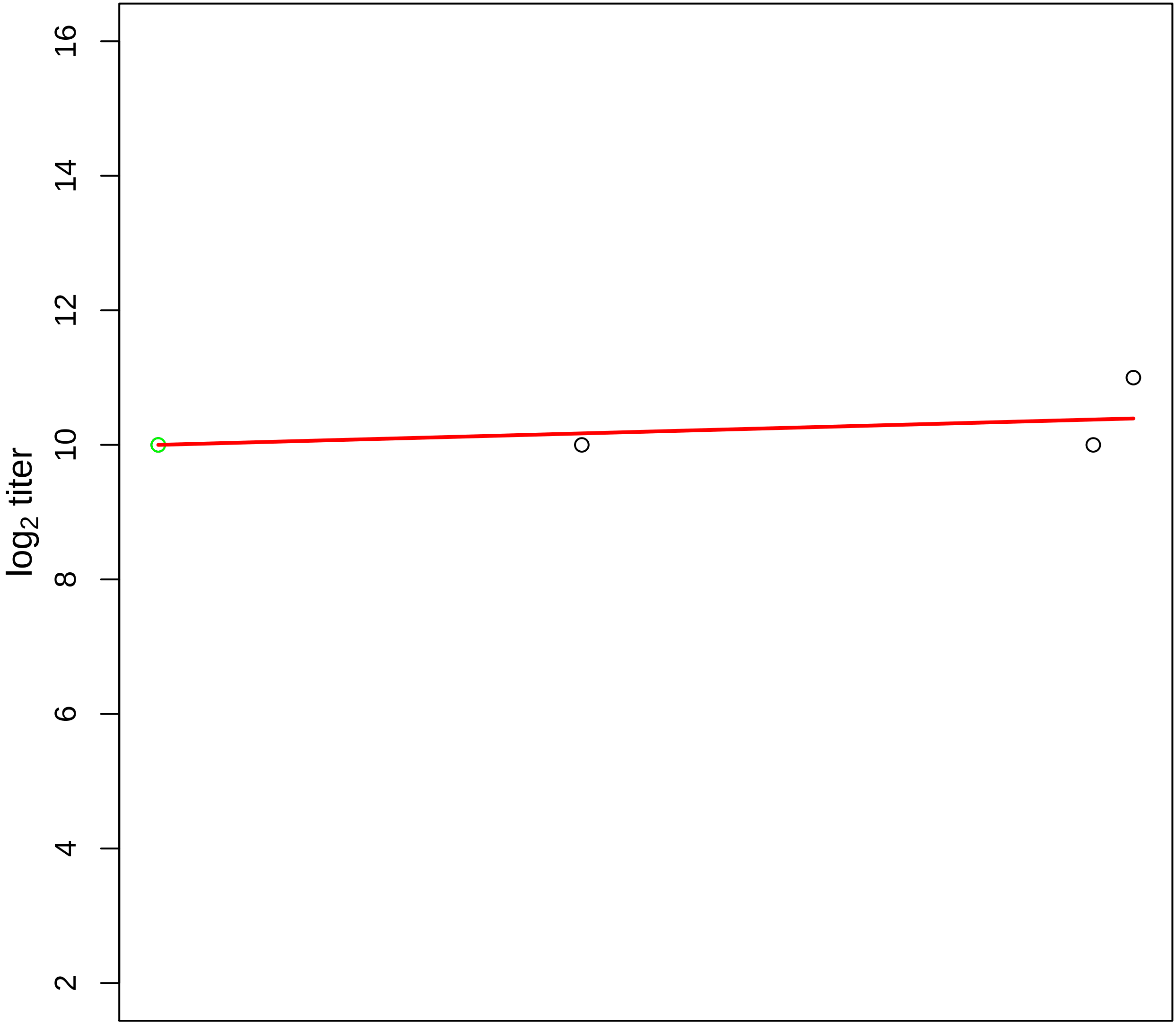

time in years from first donation of donor 98
mean absolute errors = 0.385 , mean squared errors = 0.18

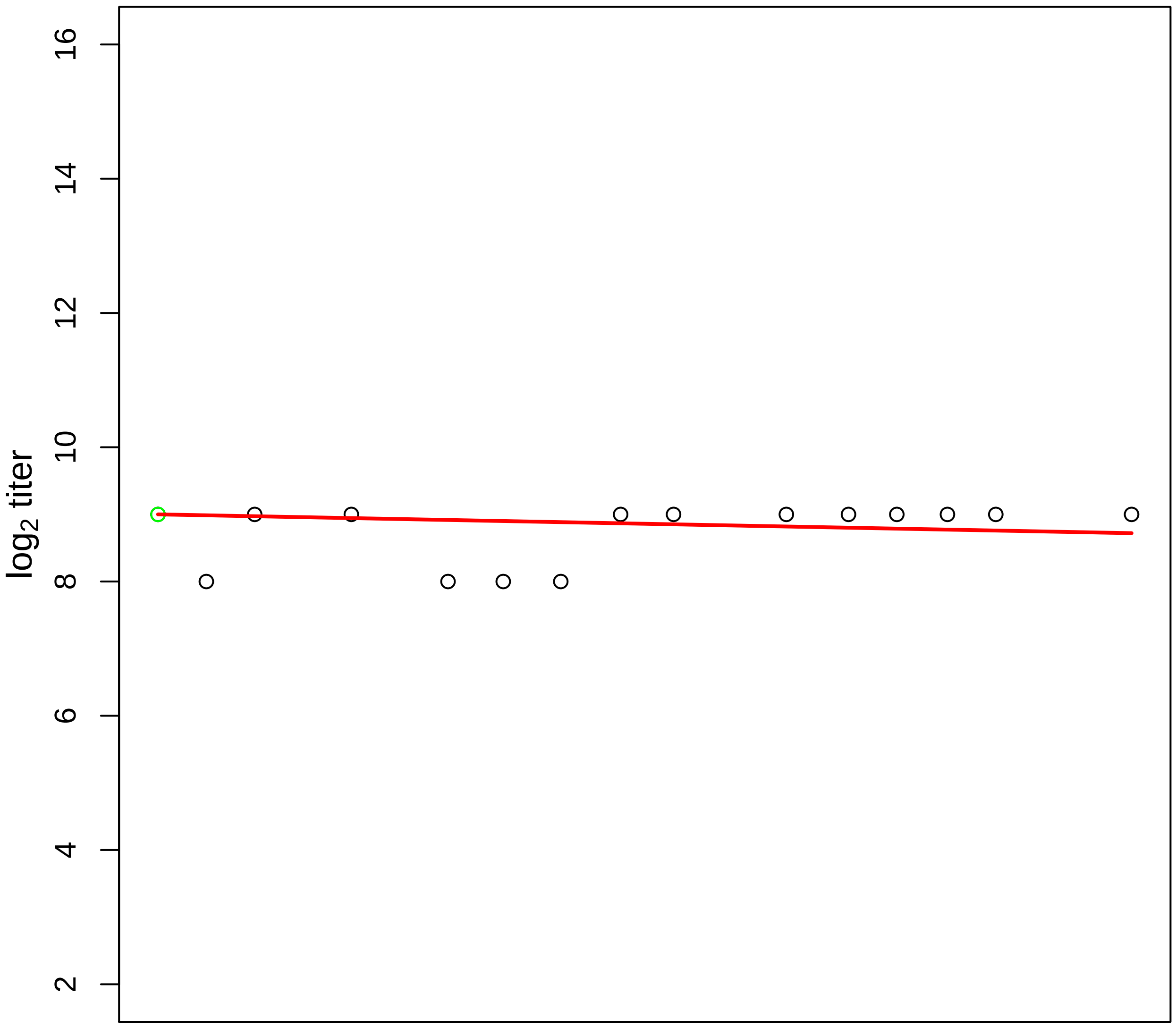

time in years from first donation of donor 99
mean absolute errors = 0.385 , mean squared errors = 0.268

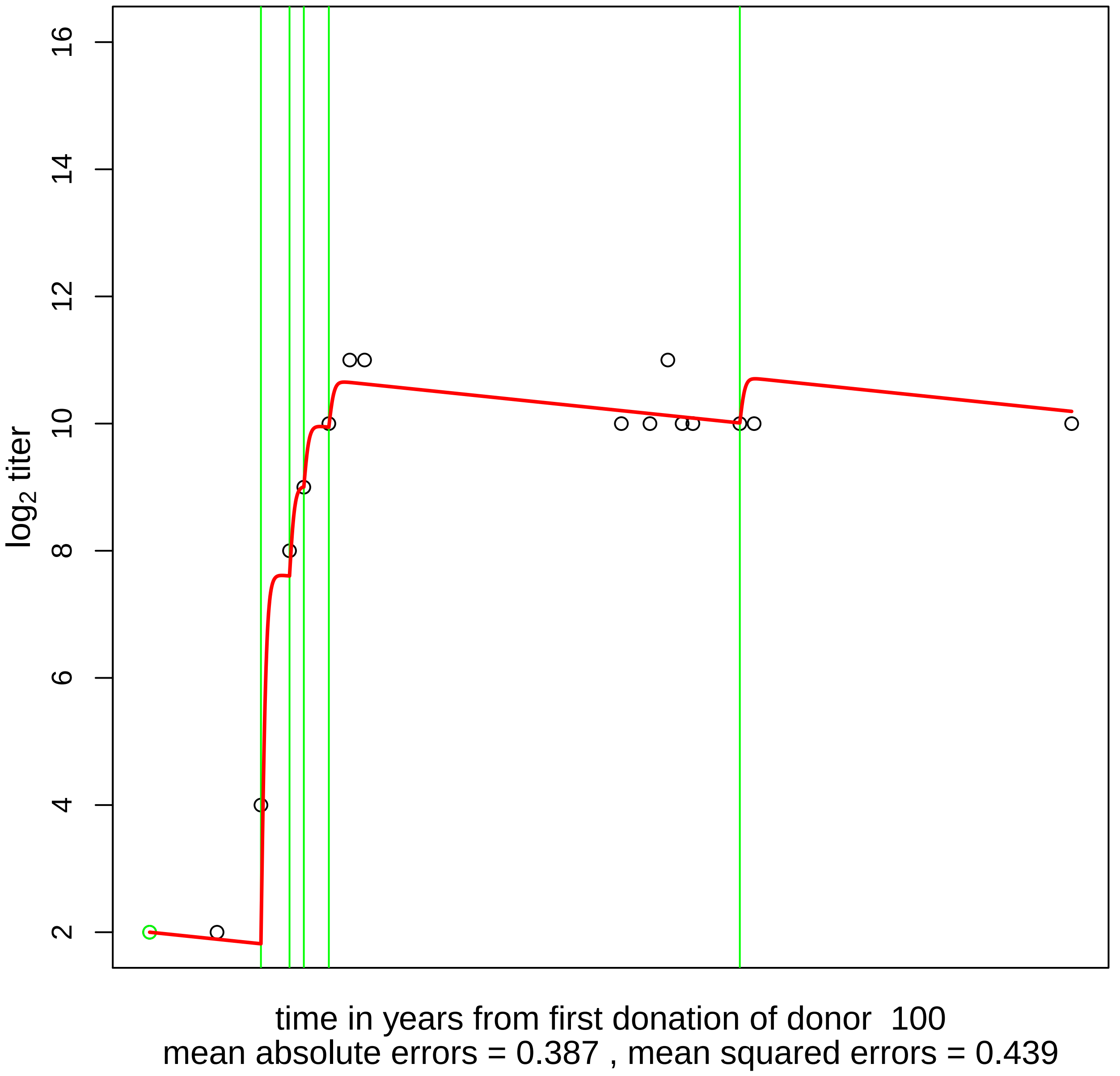

log2 titer
16
14
12
10
8
6
4
2
time in years from first donation of donor 100
mean absolute errors = 0.387 , mean squared errors = 0.439

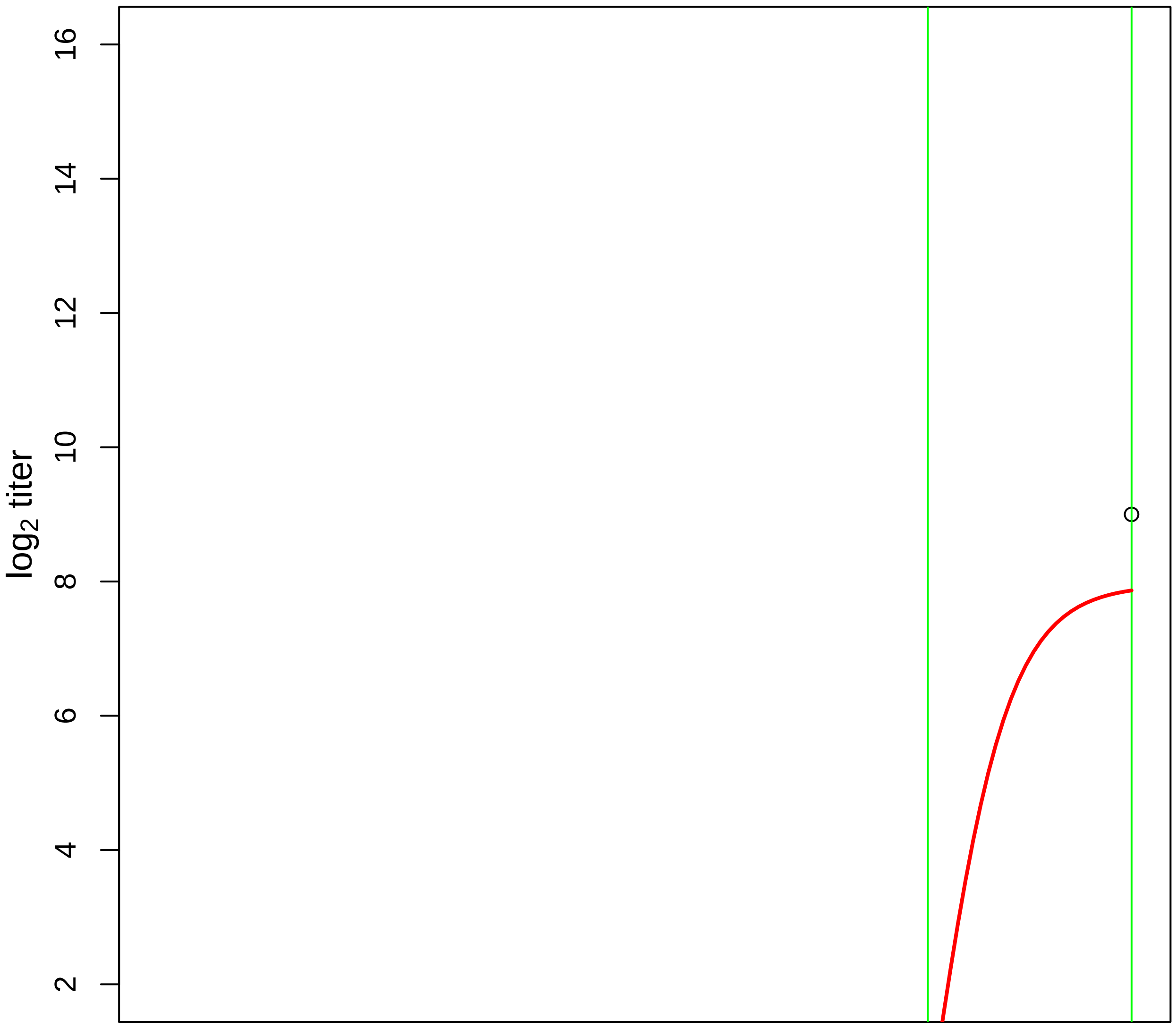

time in years from first donation of donor 101
mean absolute errors = 0.39 , mean squared errors = 0.428

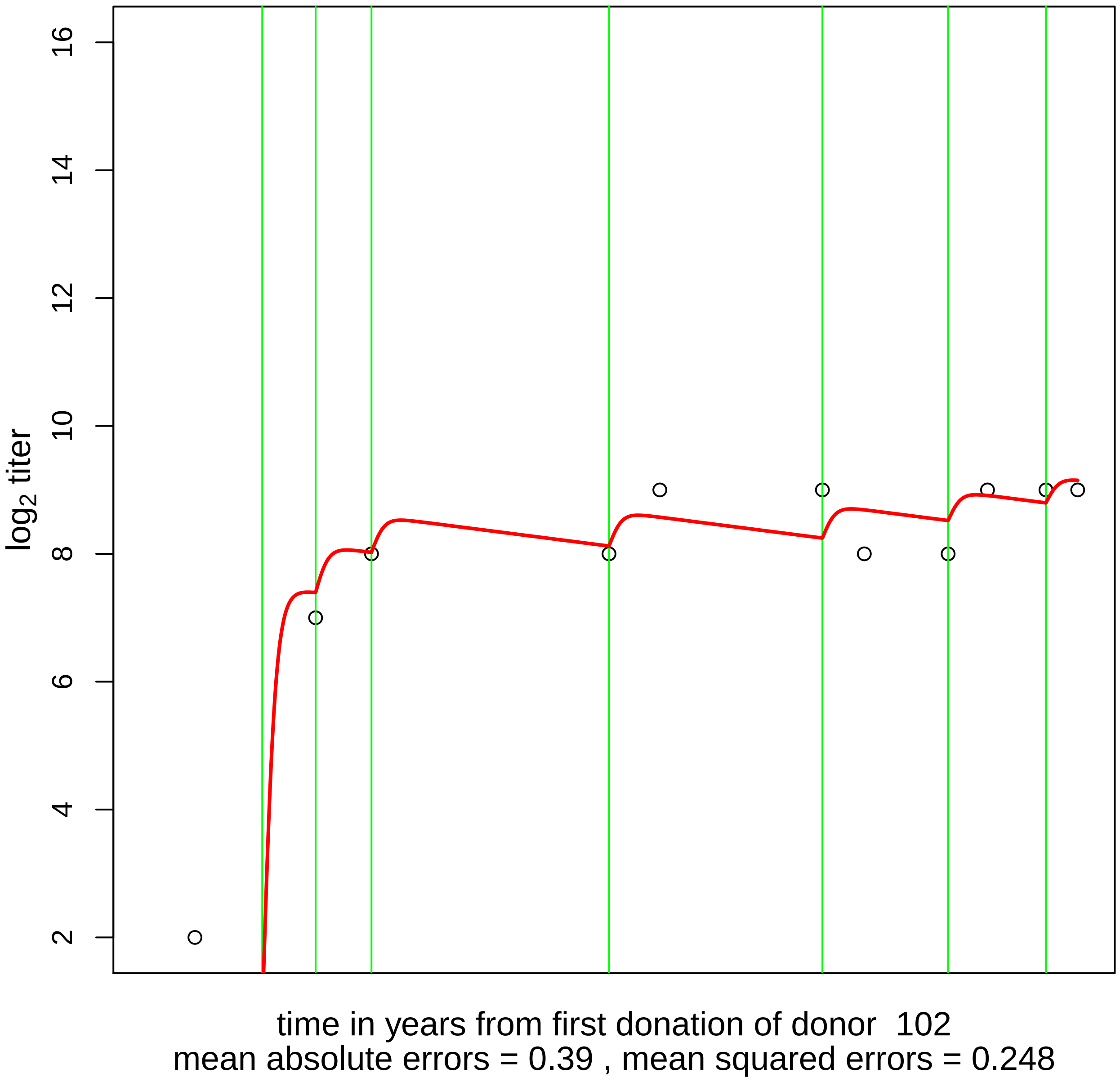

16
14
12
10
8
6
4
2
log2 titer
time in years from first donation of donor 102
mean absolute errors = 0.39 , mean squared errors = 0.248

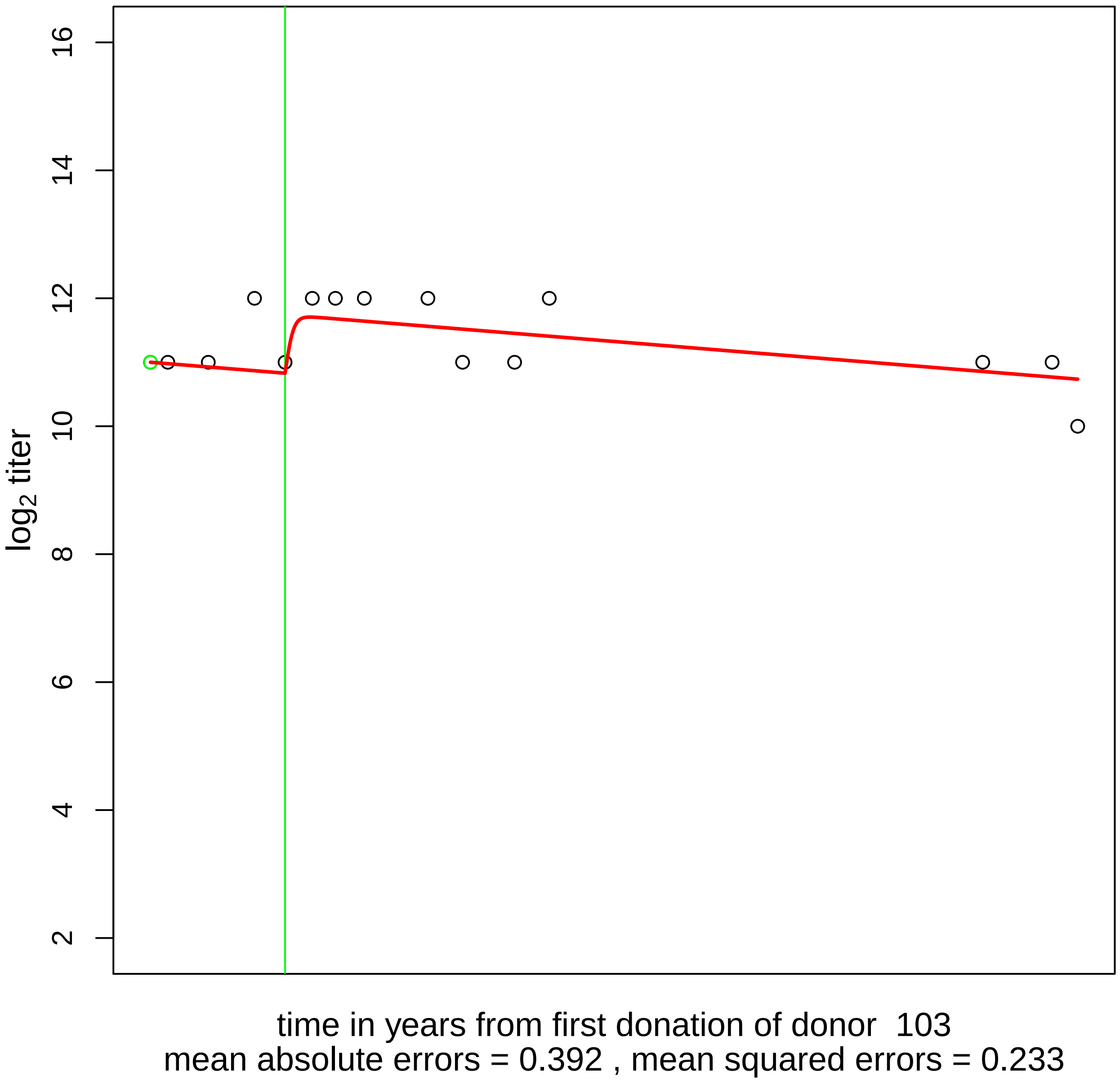

16
14
12
10
8
6
4
2
$\log_2$ titer
time in years from first donation of donor 103
mean absolute errors = 0.392 , mean squared errors = 0.233

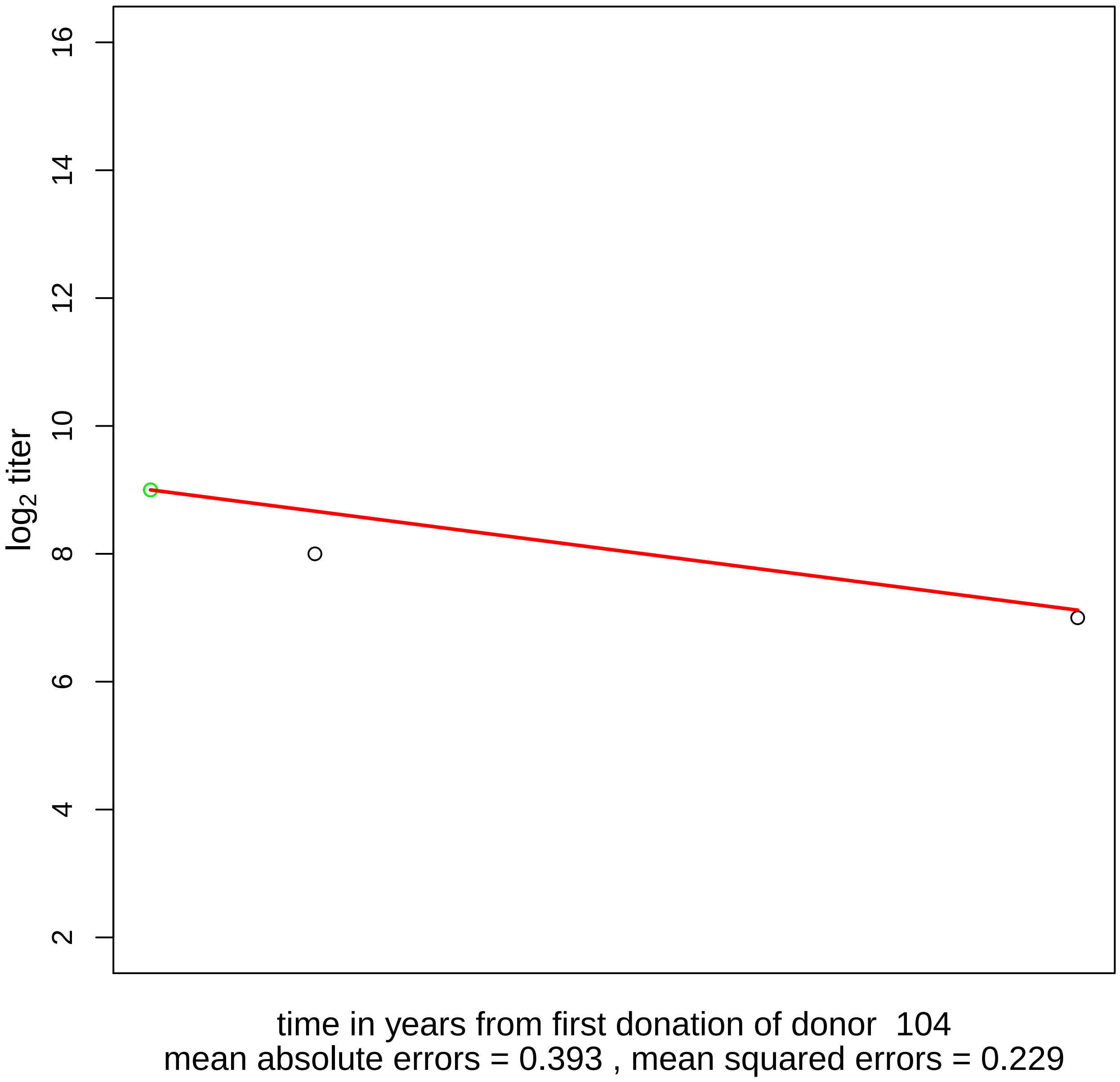

log2 titer
2
4
6
8
10
12
14
16
time in years from first donation of donor 104
mean absolute errors = 0.393 , mean squared errors = 0.229

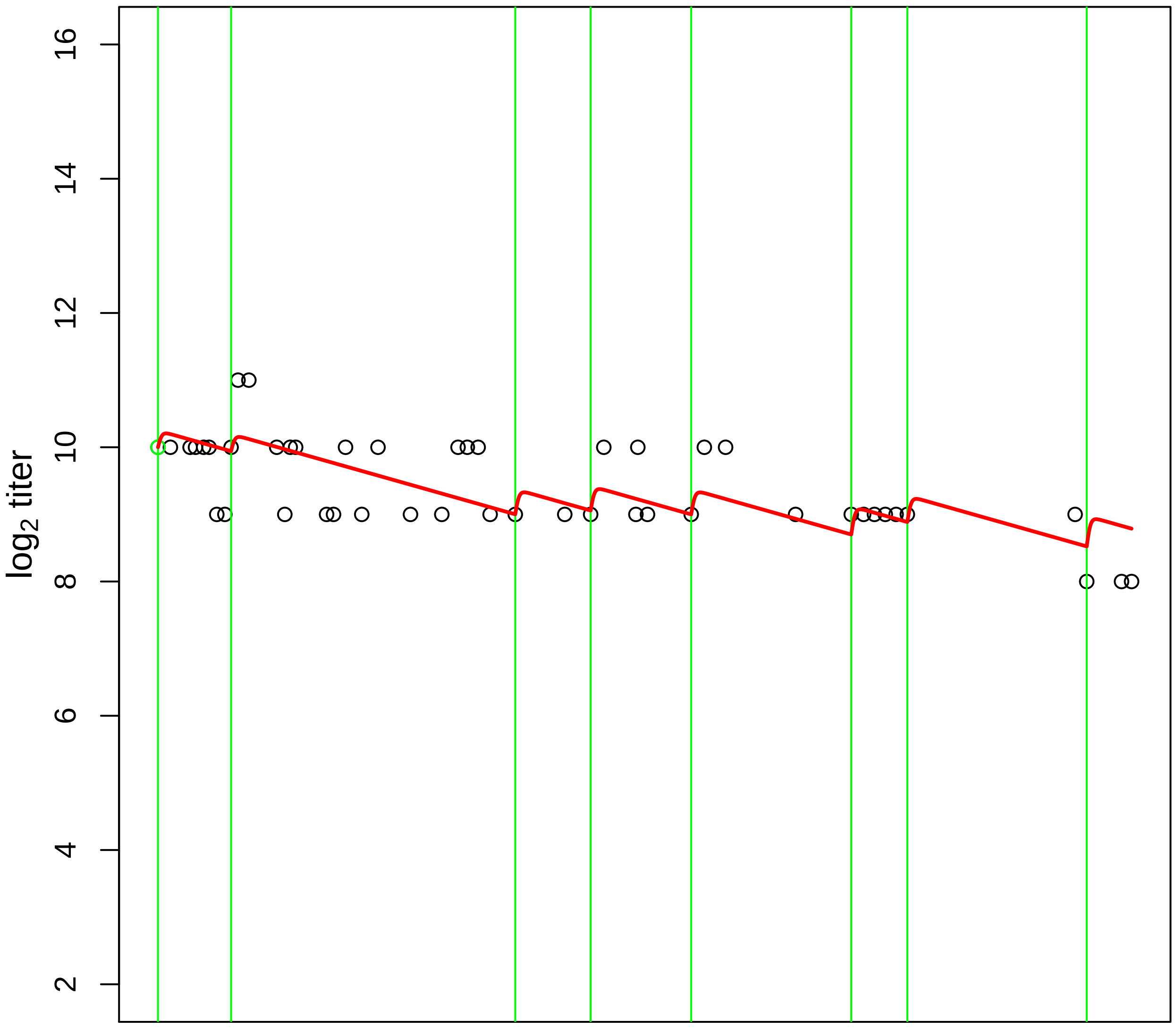

time in years from first donation of donor 105
mean absolute errors = 0.397 , mean squared errors = 0.275

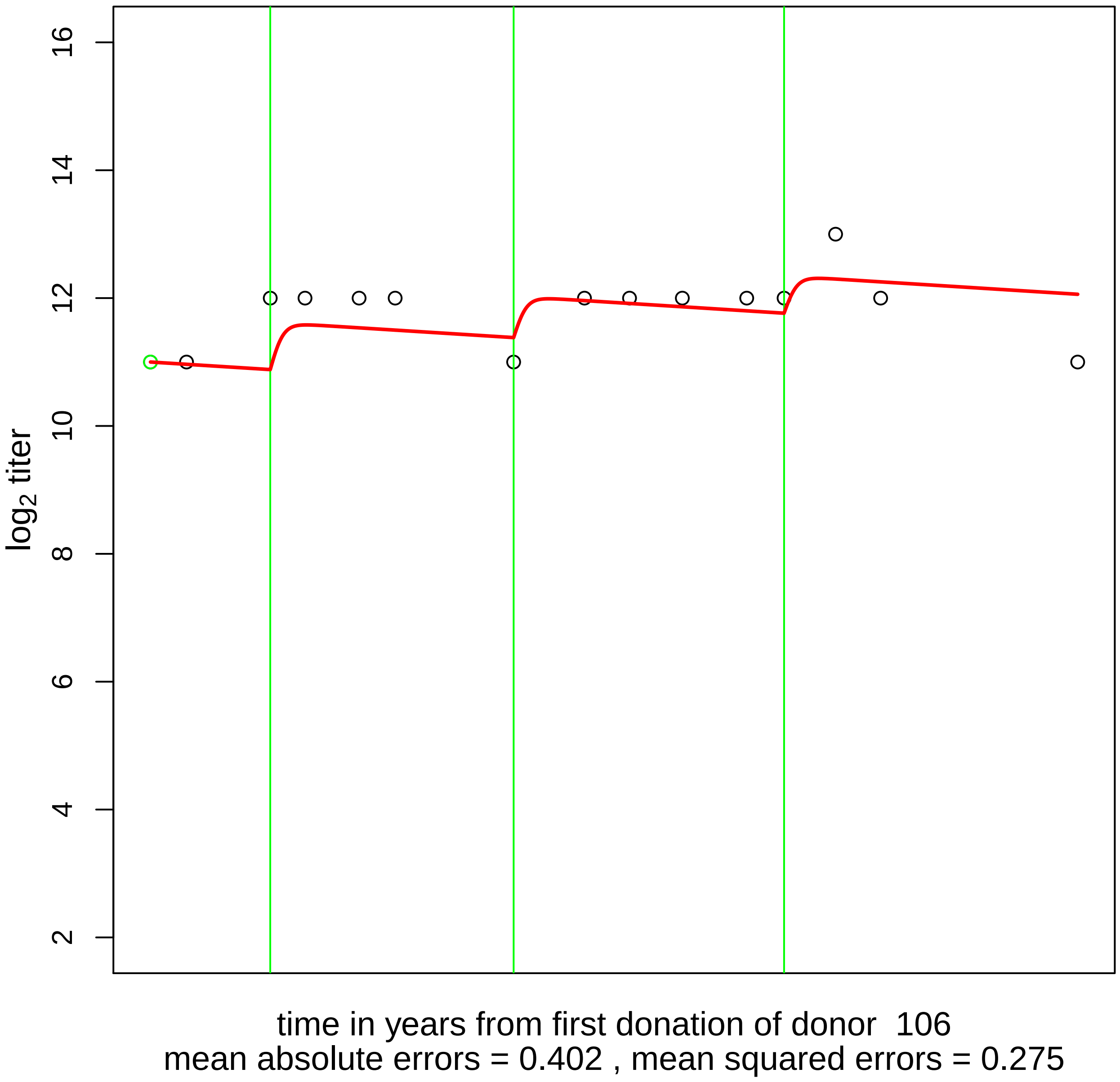

16
14
12
10
8
6
4
2
log2 titer
time in years from first donation of donor 106
mean absolute errors = 0.402 , mean squared errors = 0.275

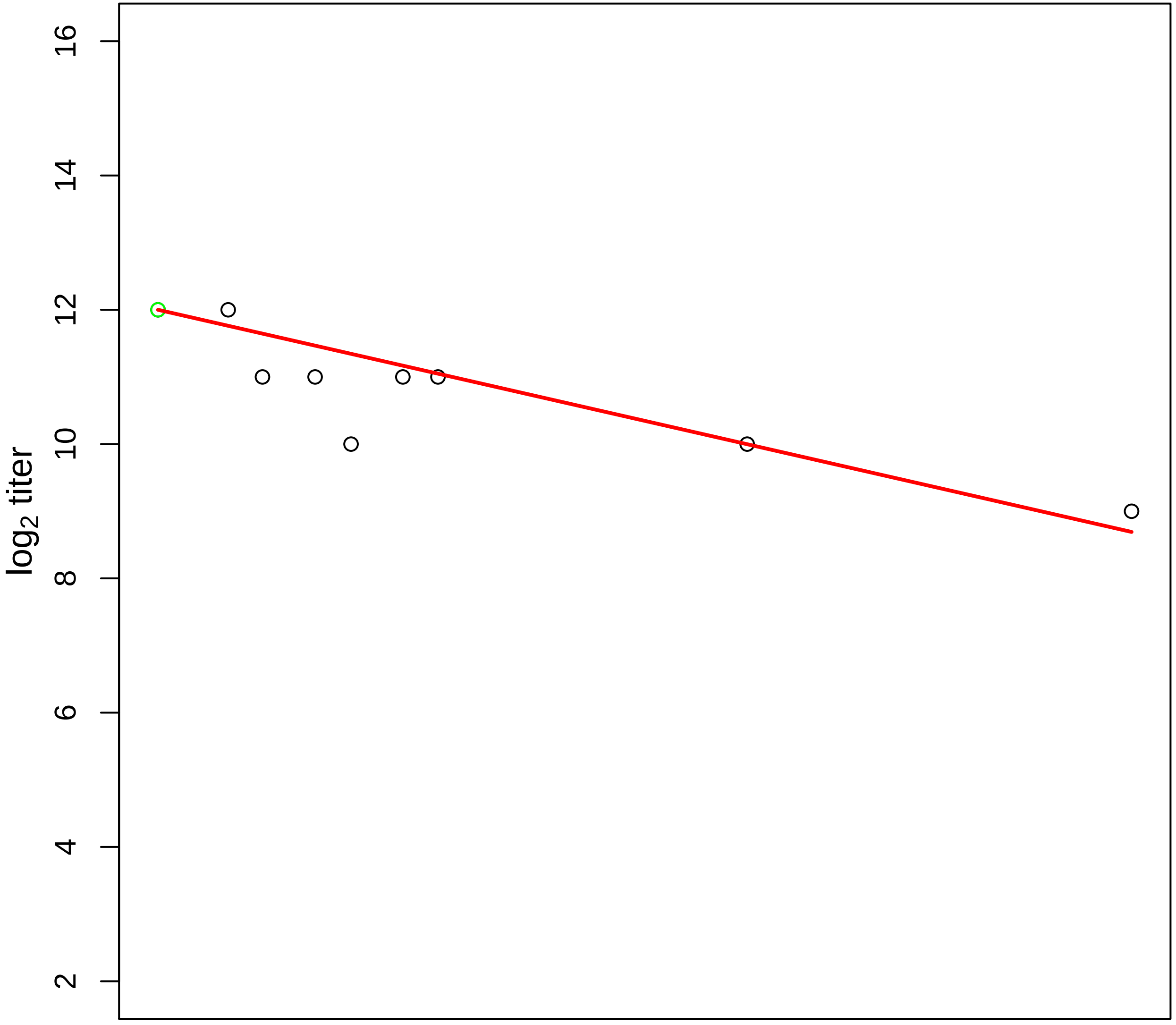

time in years from first donation of donor 107
mean absolute errors = 0.403 , mean squared errors = 0.328

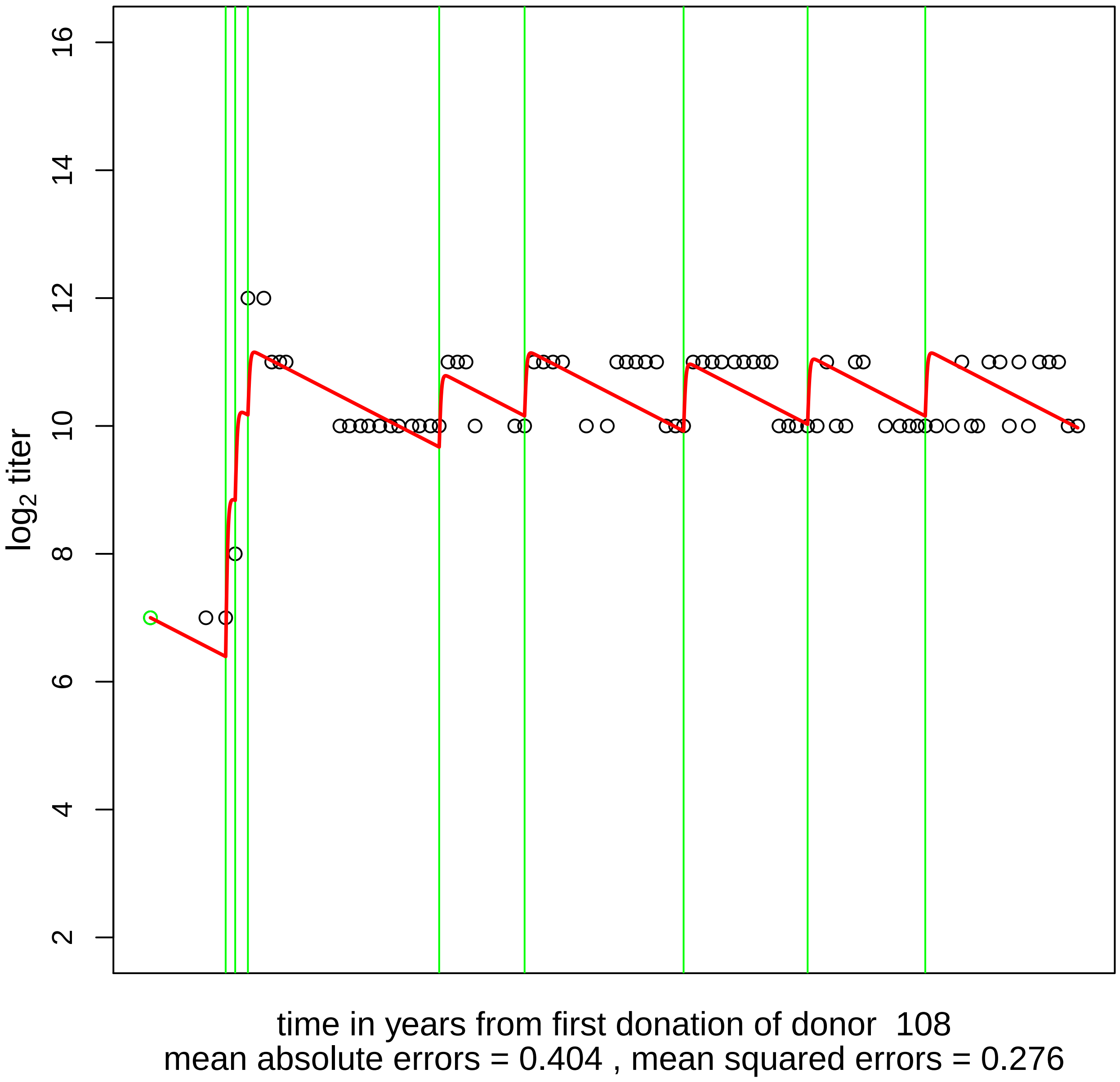

16
14
12
10
8
6
4
2
log2 titer
time in years from first donation of donor 108
mean absolute errors = 0.404 , mean squared errors = 0.276

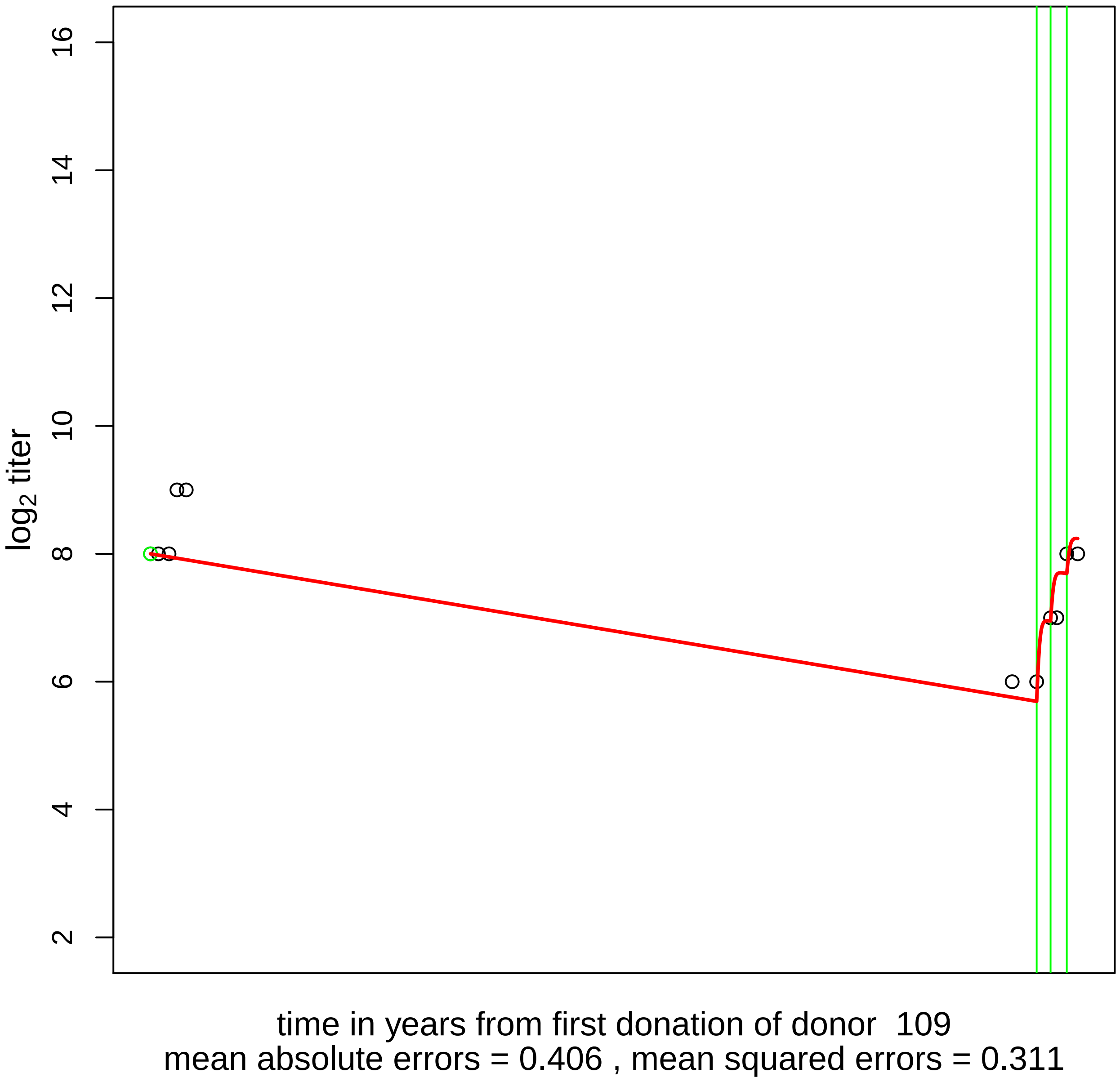
log2 titer
16
14
12
10
8
6
4
2
time in years from first donation of donor 109
mean absolute errors = 0.406 , mean squared errors = 0.311

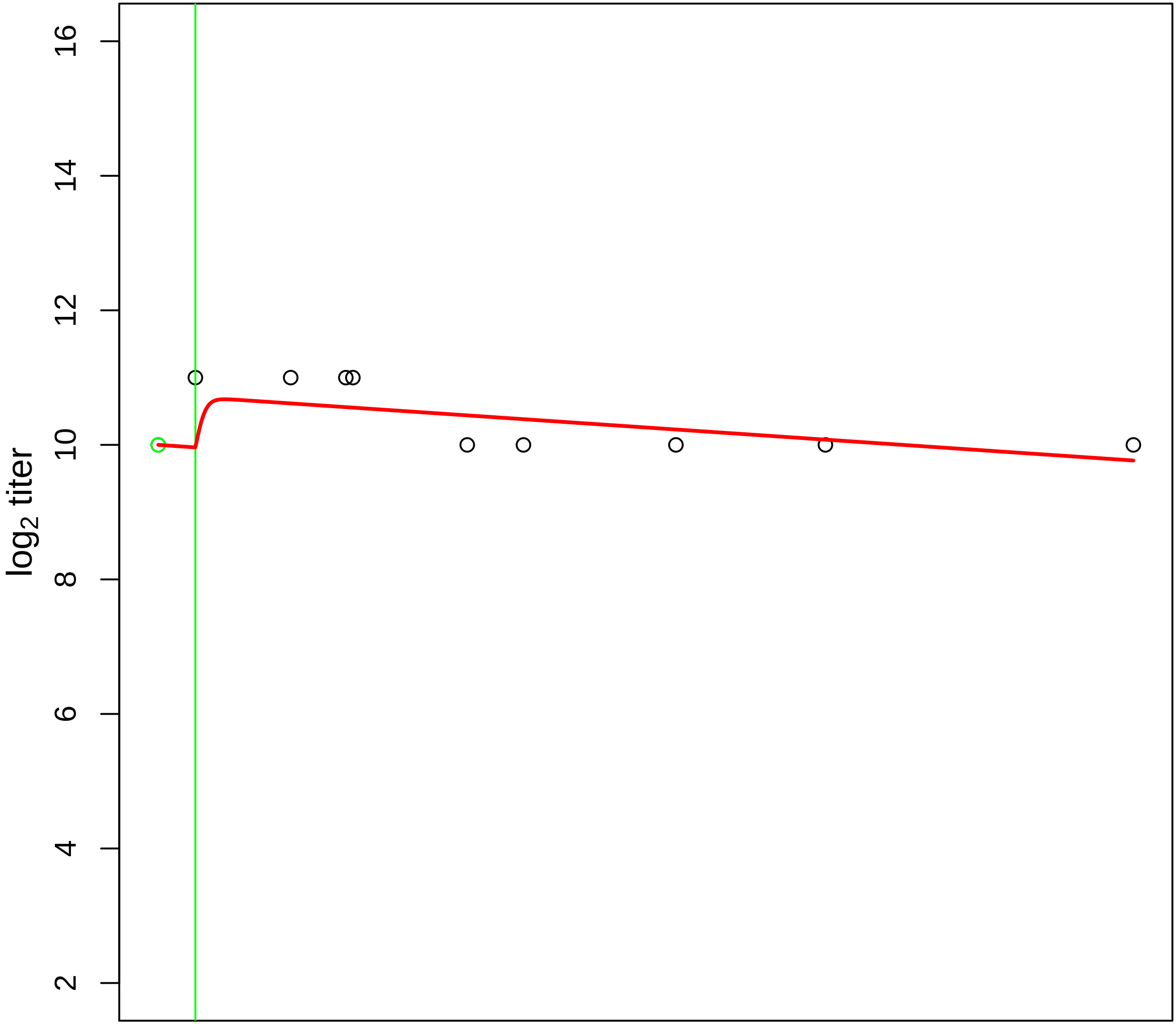

time in years from first donation of donor 110
mean absolute errors = 0.407 , mean squared errors = 0.23

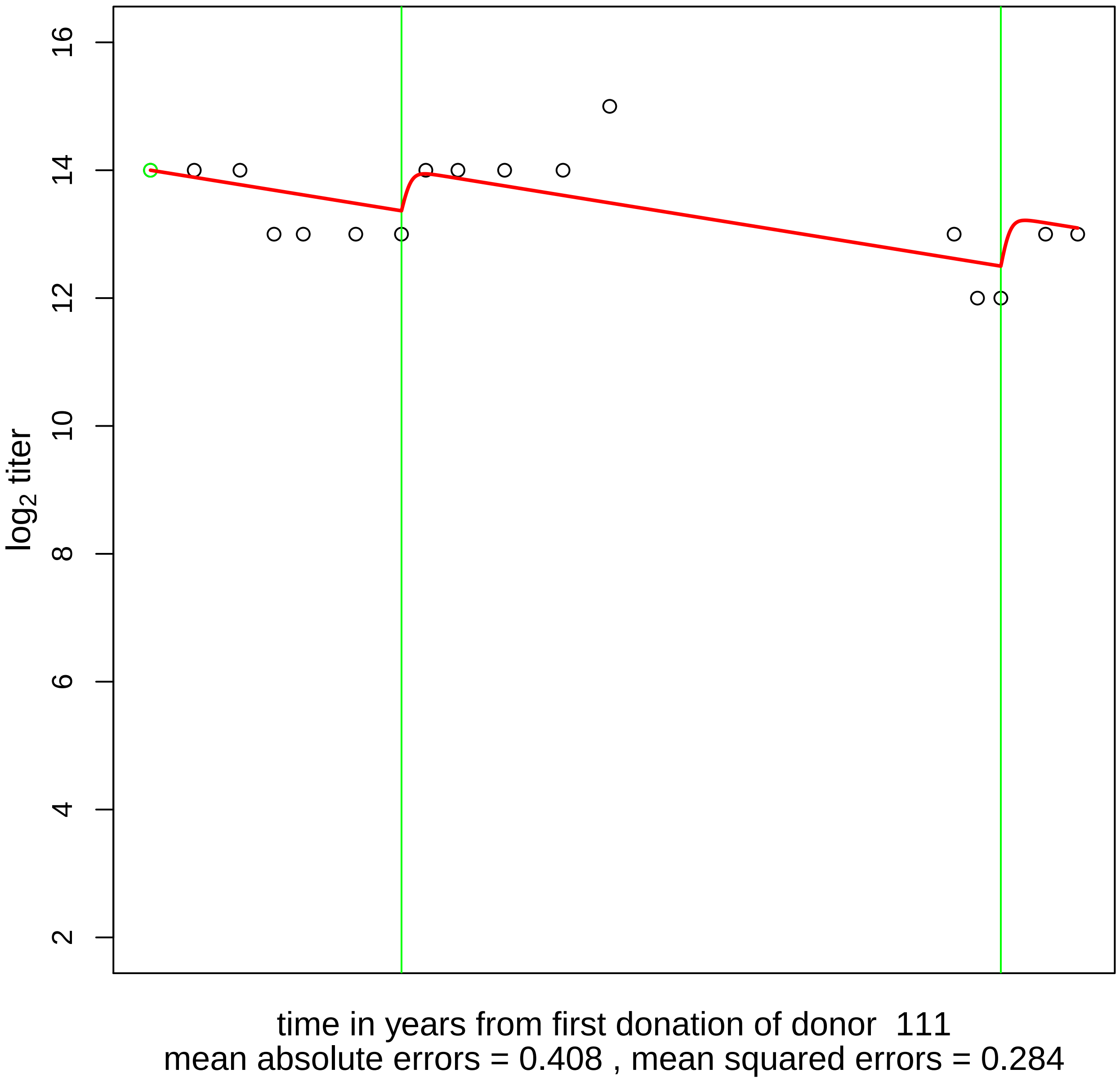

16
14
12
10
8
6
4
2
log2 titer
time in years from first donation of donor 111
mean absolute errors = 0.408 , mean squared errors = 0.284

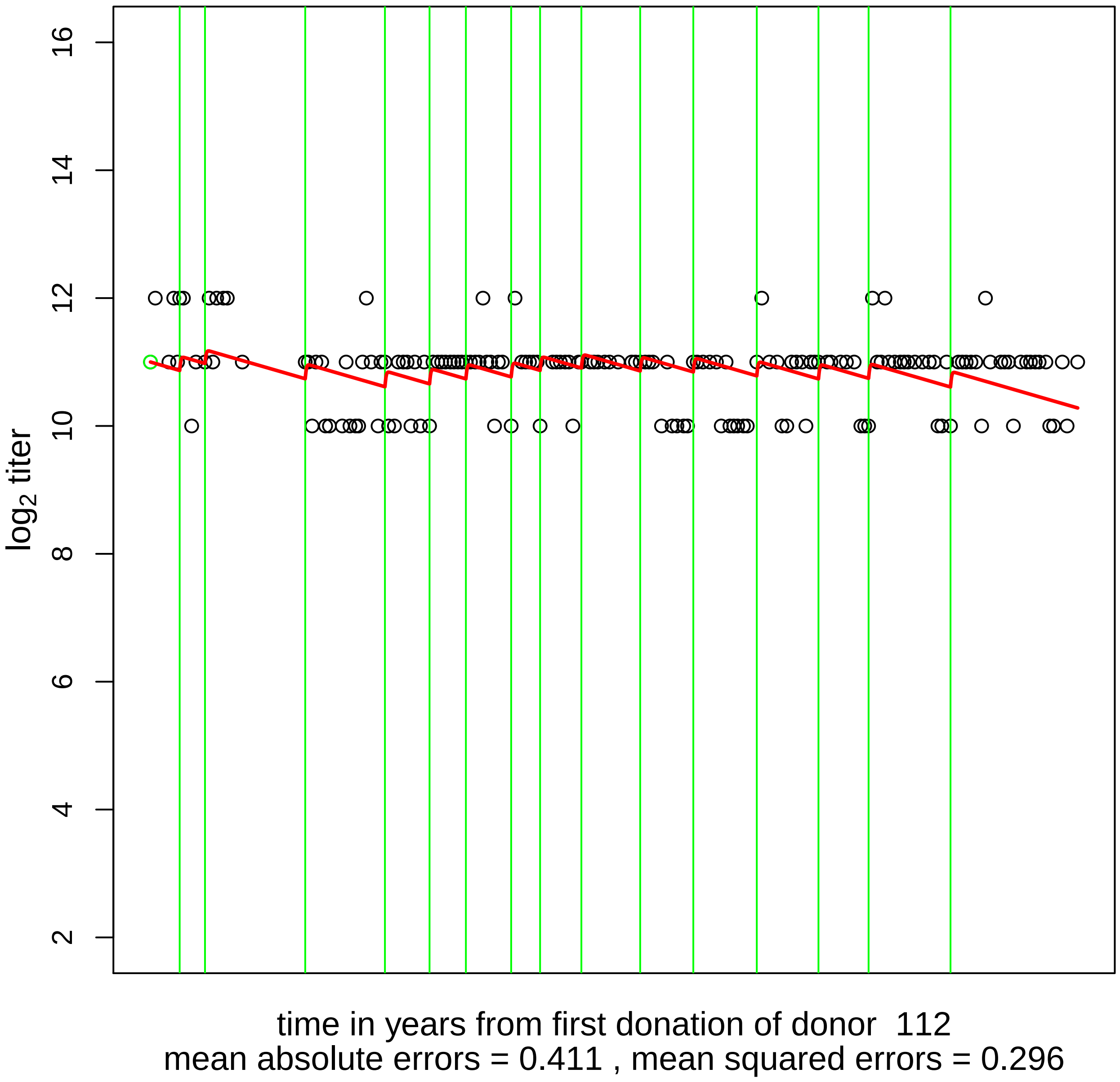

16
14
12
10
8
6
4
2
$\log_2$ titer
time in years from first donation of donor 112
mean absolute errors = 0.411 , mean squared errors = 0.296

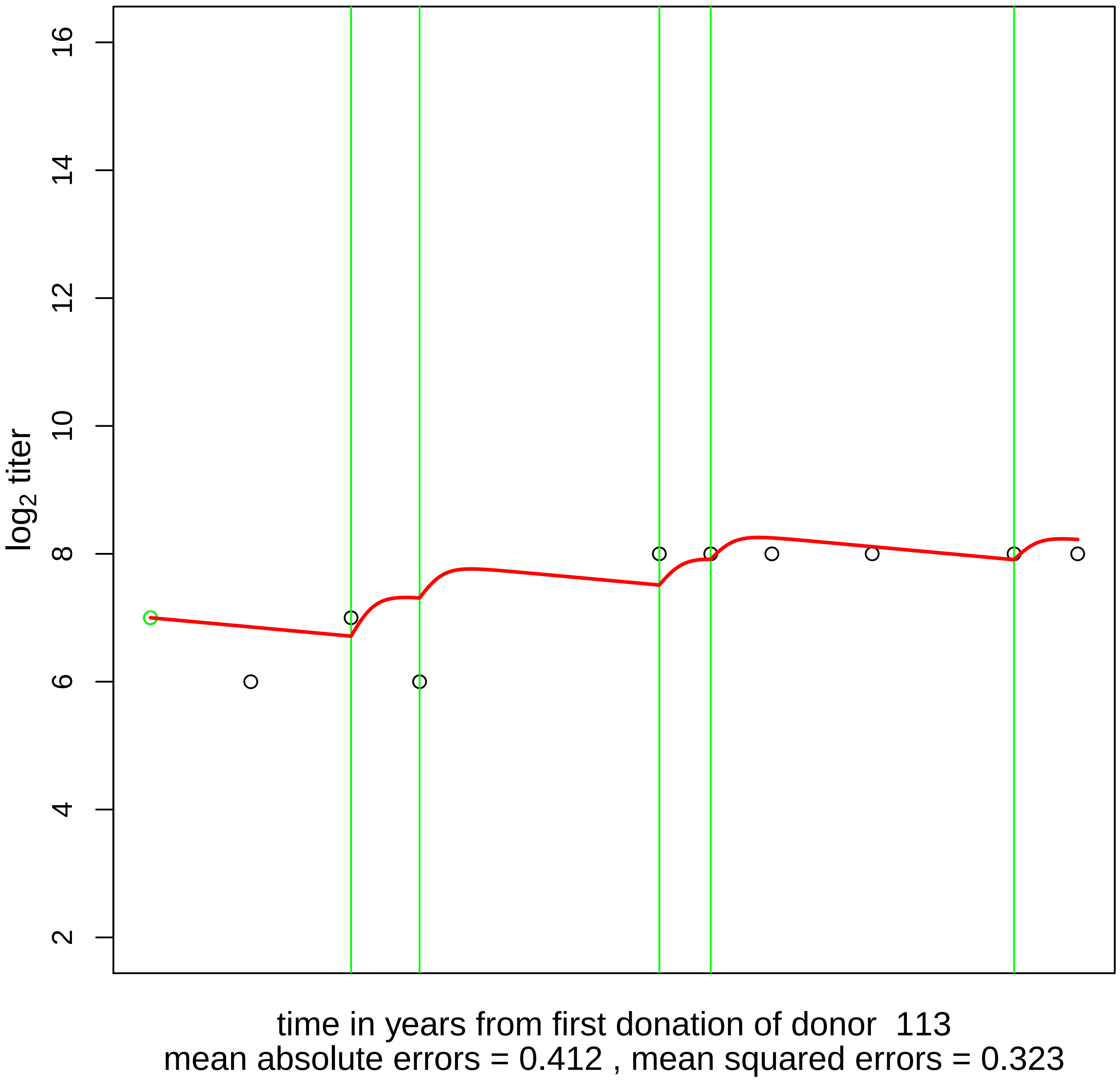

16
14
12
10
8
6
4
2
$\log_2$ titer
time in years from first donation of donor 113
mean absolute errors = 0.412 , mean squared errors = 0.323

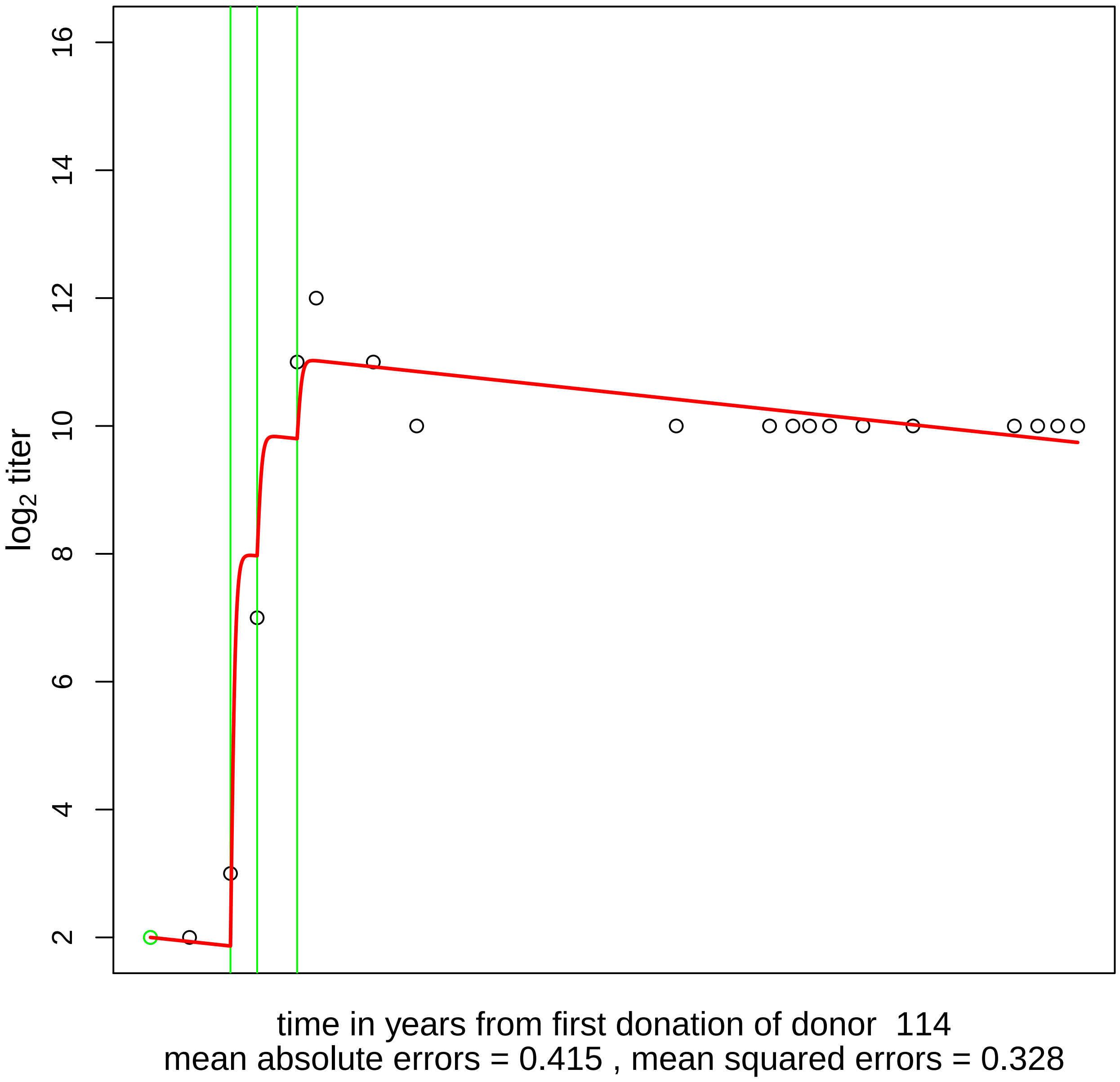
16
14
12
10
8
6
4
2
log2 titer
time in years from first donation of donor 114
mean absolute errors = 0.415 , mean squared errors = 0.328

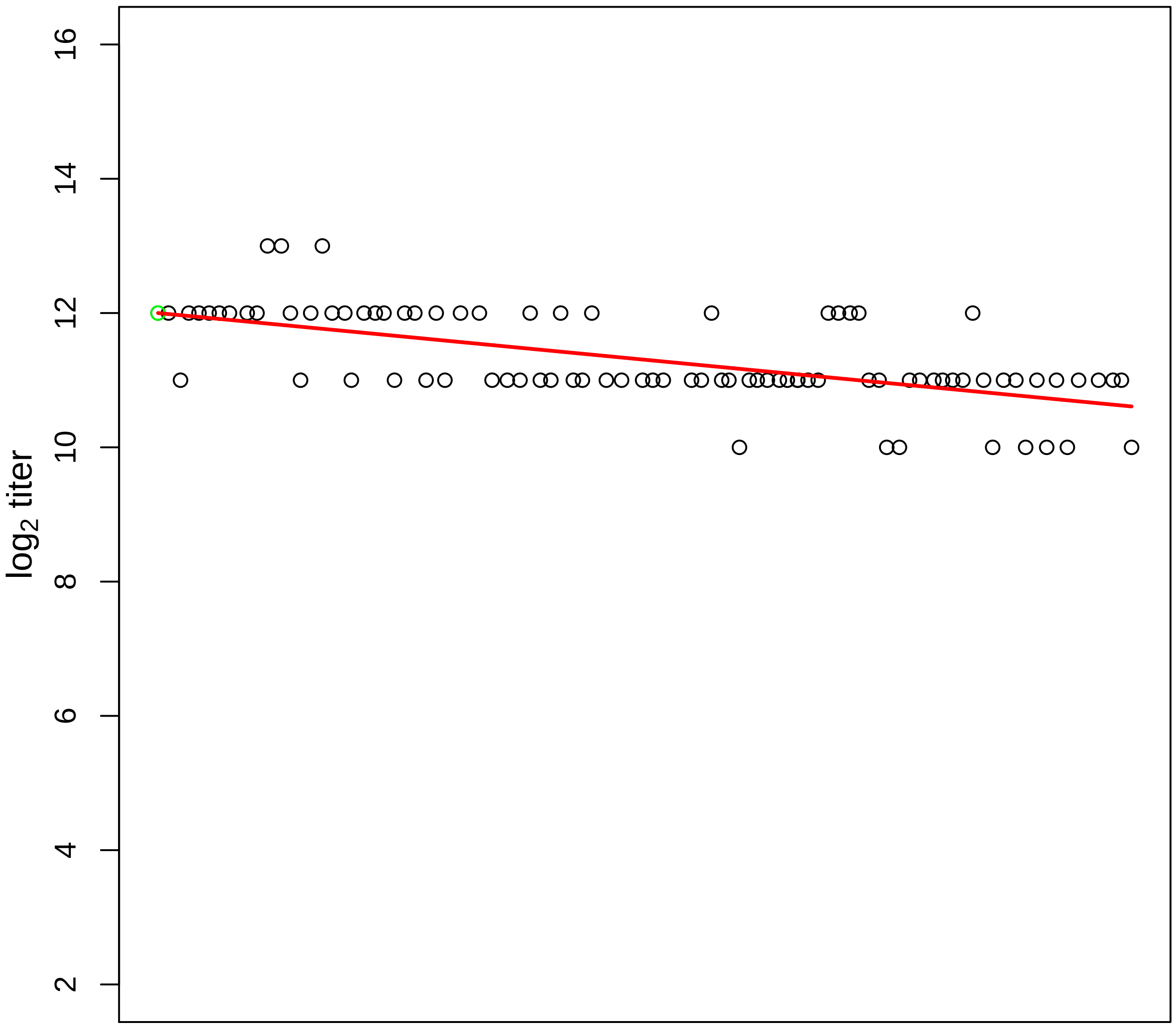

time in years from first donation of donor 115
mean absolute errors = 0.415 , mean squared errors = 0.28

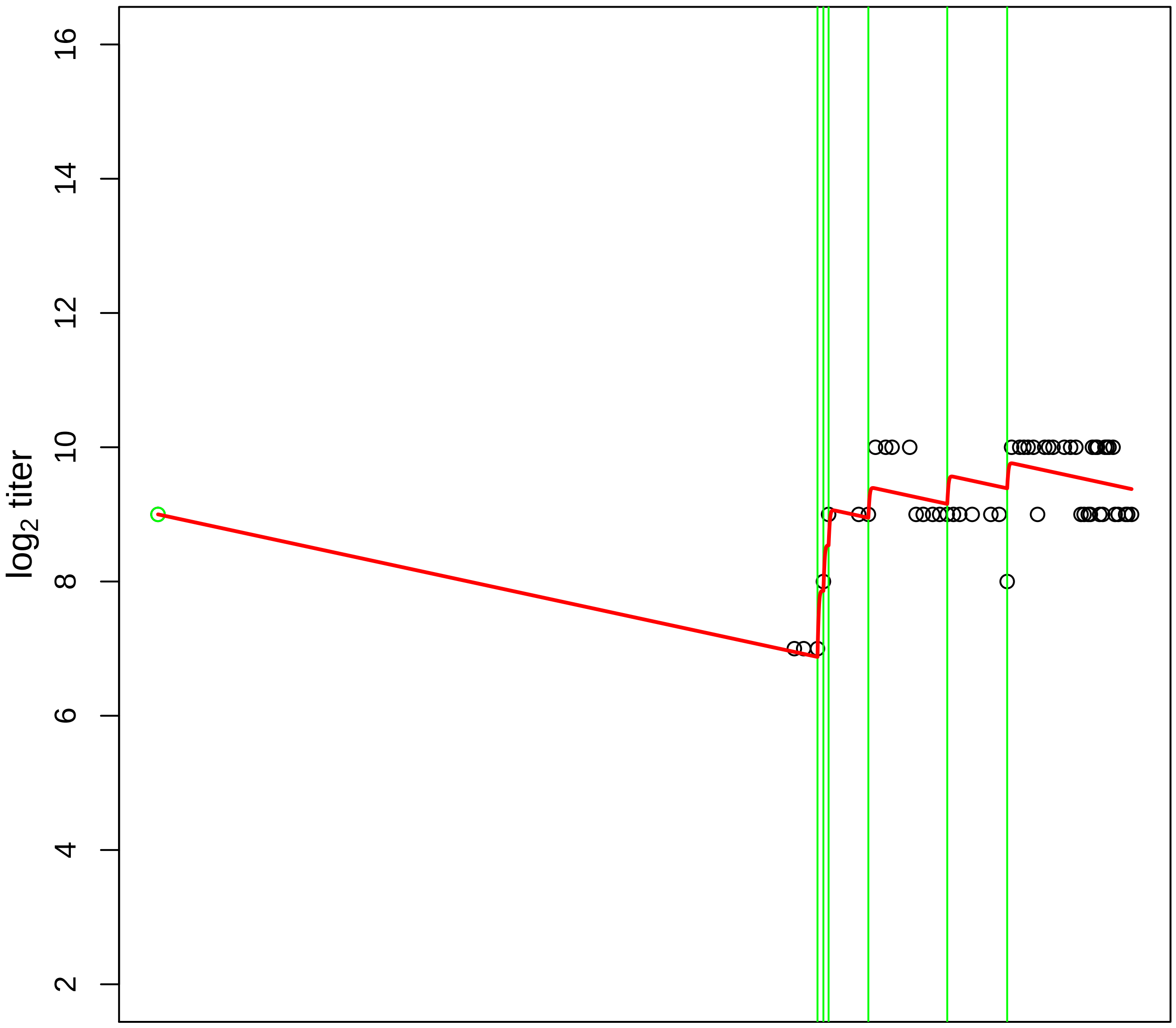

time in years from first donation of donor 116
mean absolute errors = 0.416 , mean squared errors = 0.221

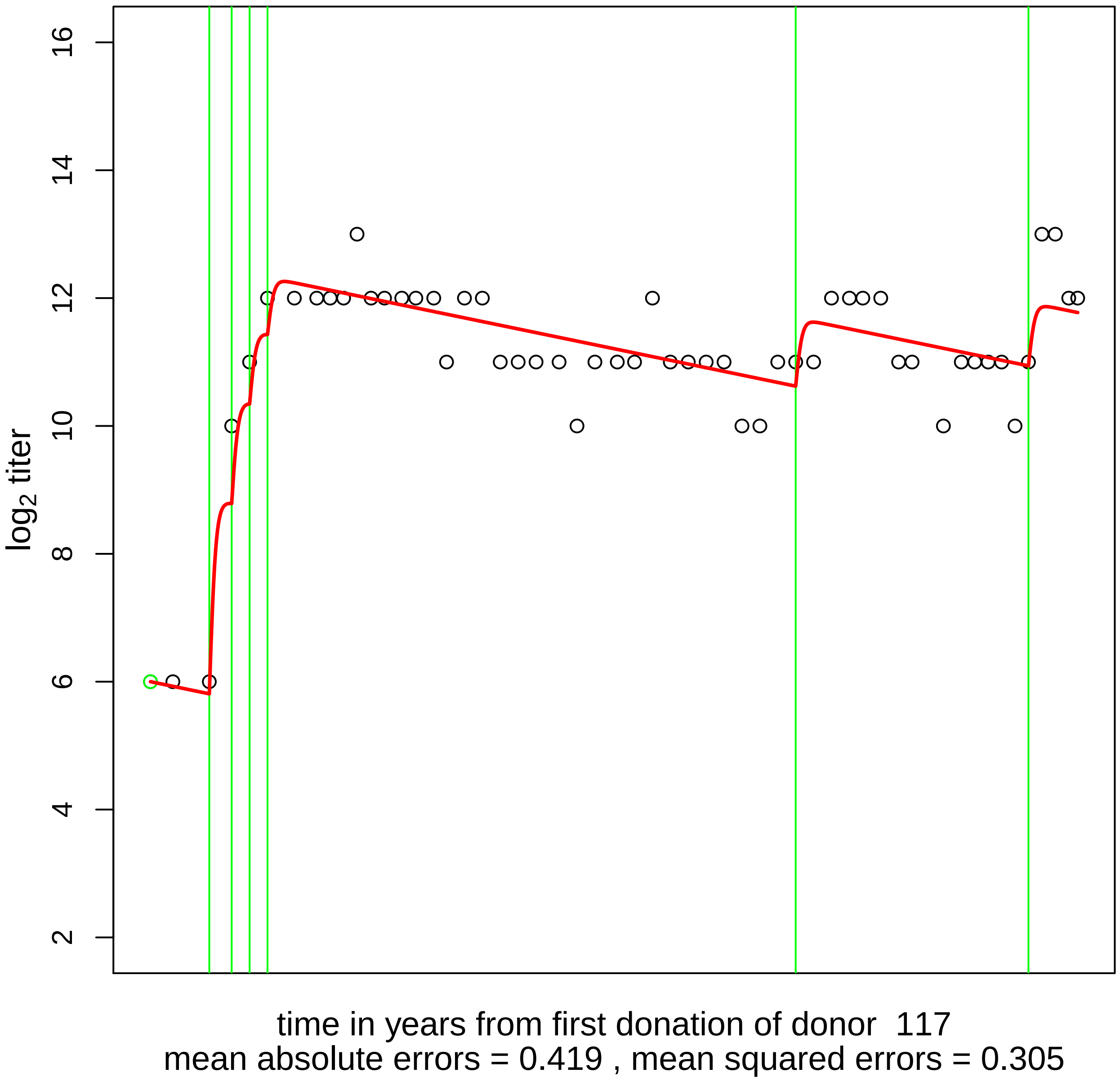

log2 titer
2
4
6
8
10
12
14
16
time in years from first donation of donor 117
mean absolute errors = 0.419 , mean squared errors = 0.305

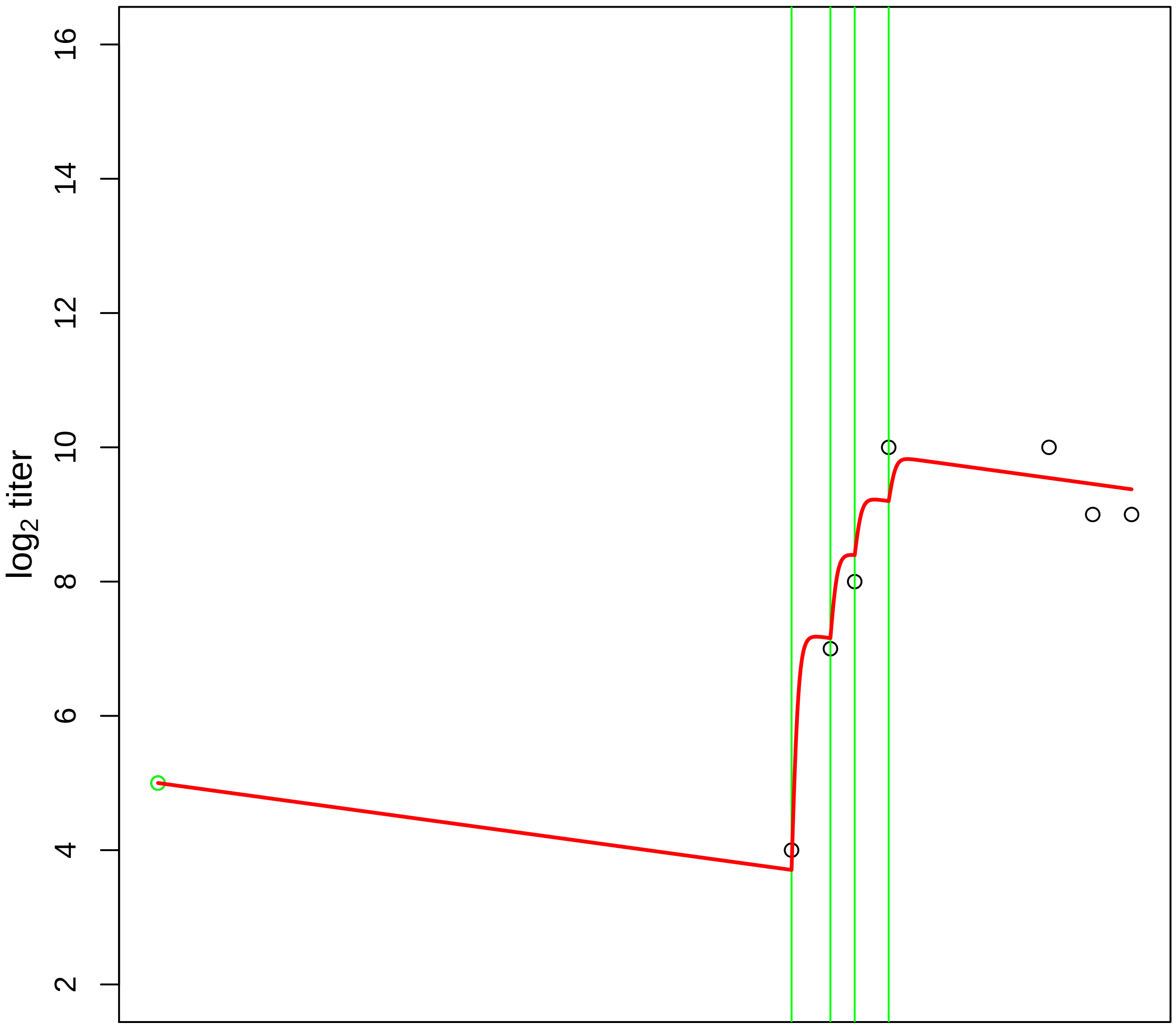

time in years from first donation of donor 118
mean absolute errors = 0.419 , mean squared errors = 0.209

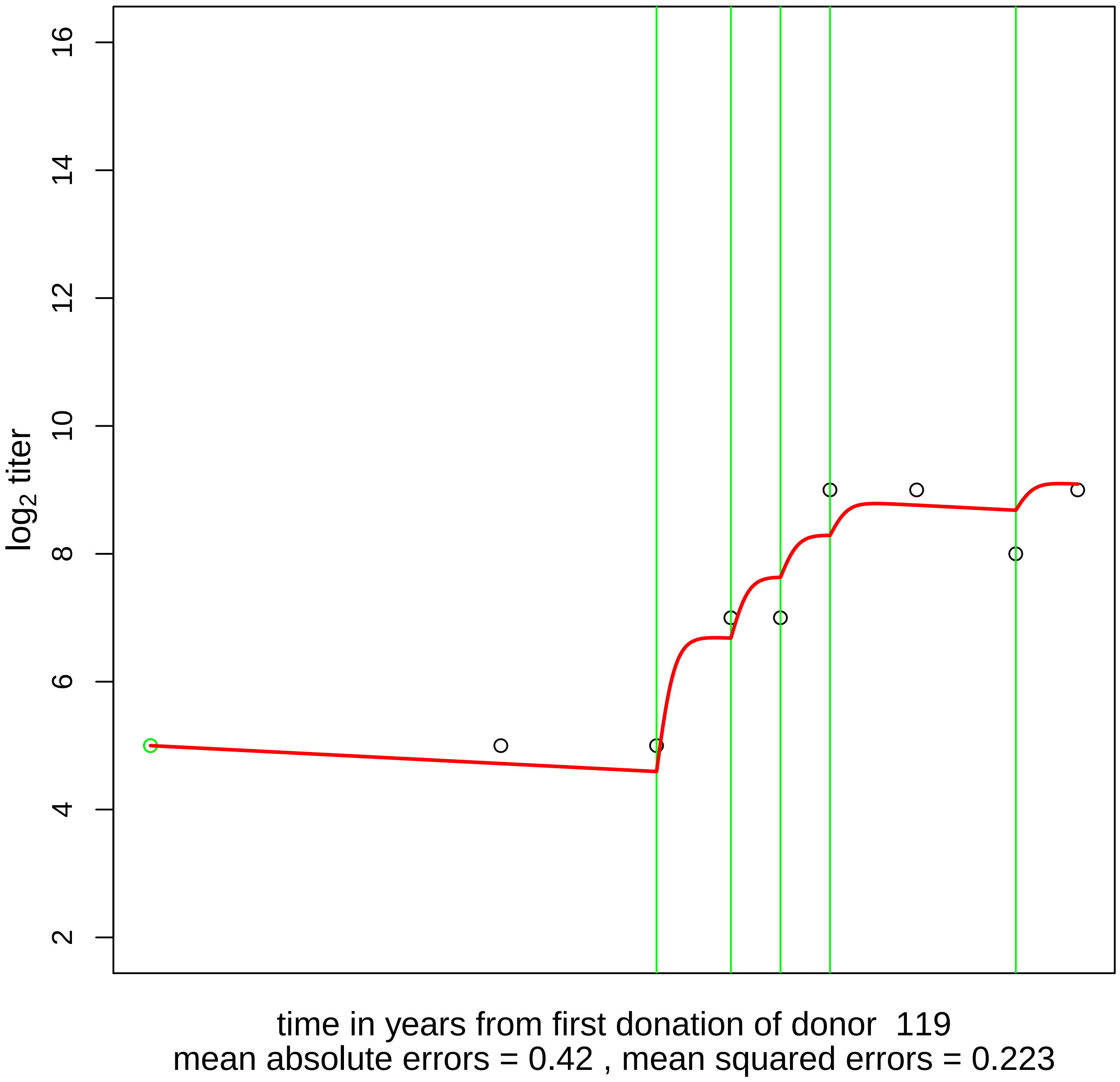

log2 titer
16
14
12
10
8
6
4
2
time in years from first donation of donor 119
mean absolute errors = 0.42 , mean squared errors = 0.223

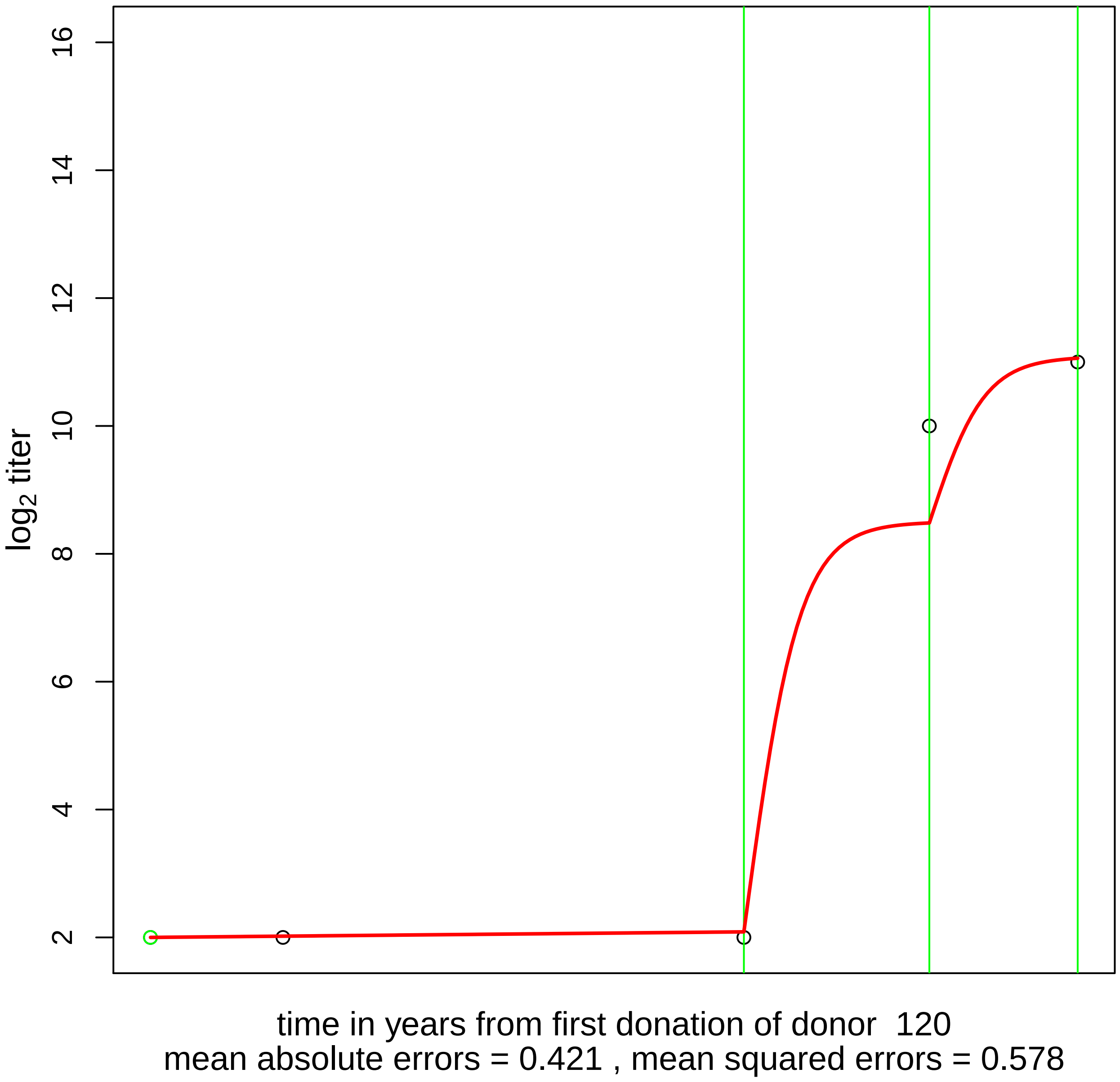

log2 titer
16
14
12
10
8
6
4
2
time in years from first donation of donor 120
mean absolute errors = 0.421 , mean squared errors = 0.578

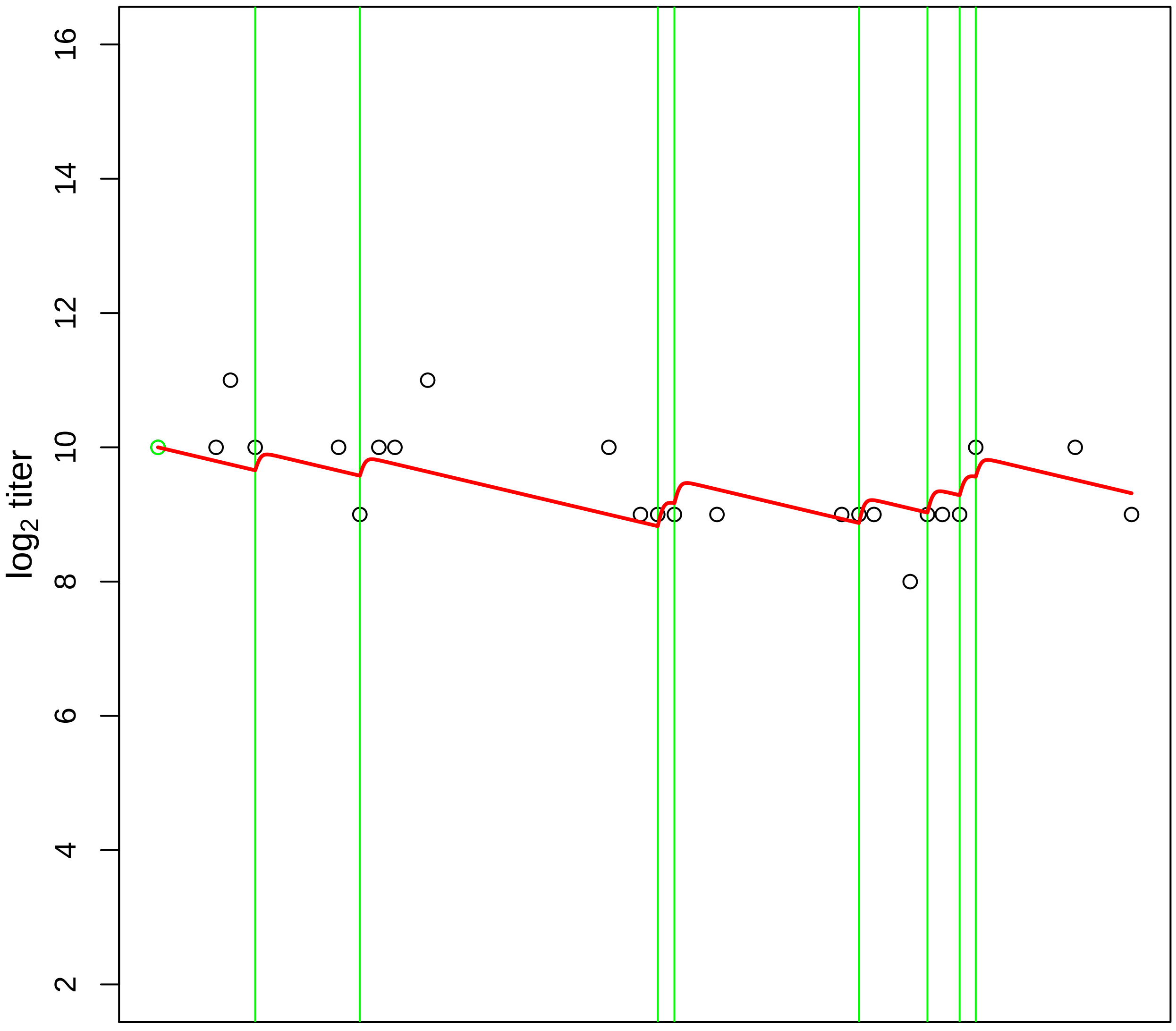

time in years from first donation of donor 121
mean absolute errors = 0.424 , mean squared errors = 0.319

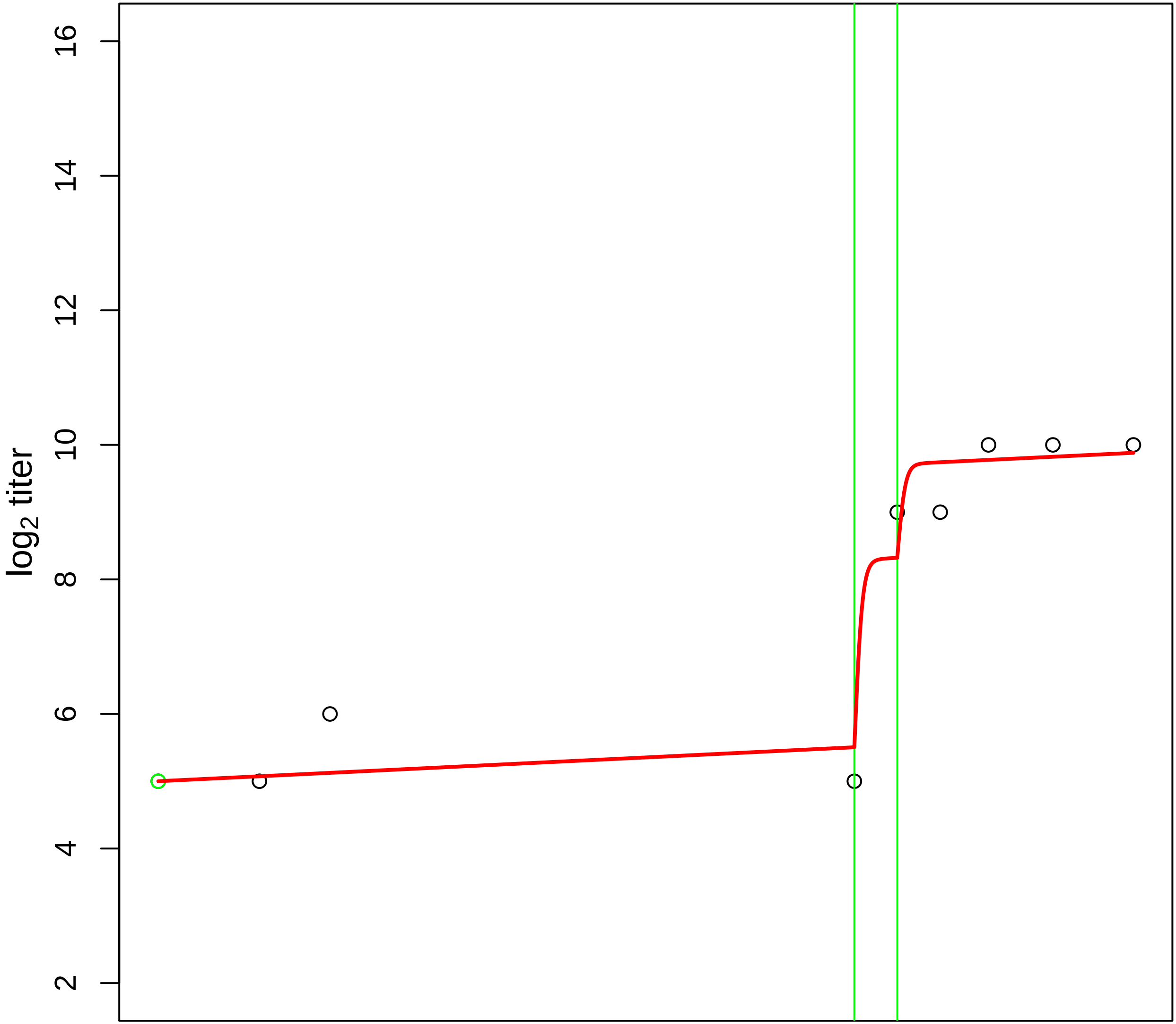

time in years from first donation of donor 122
mean absolute errors = 0.424 , mean squared errors = 0.267

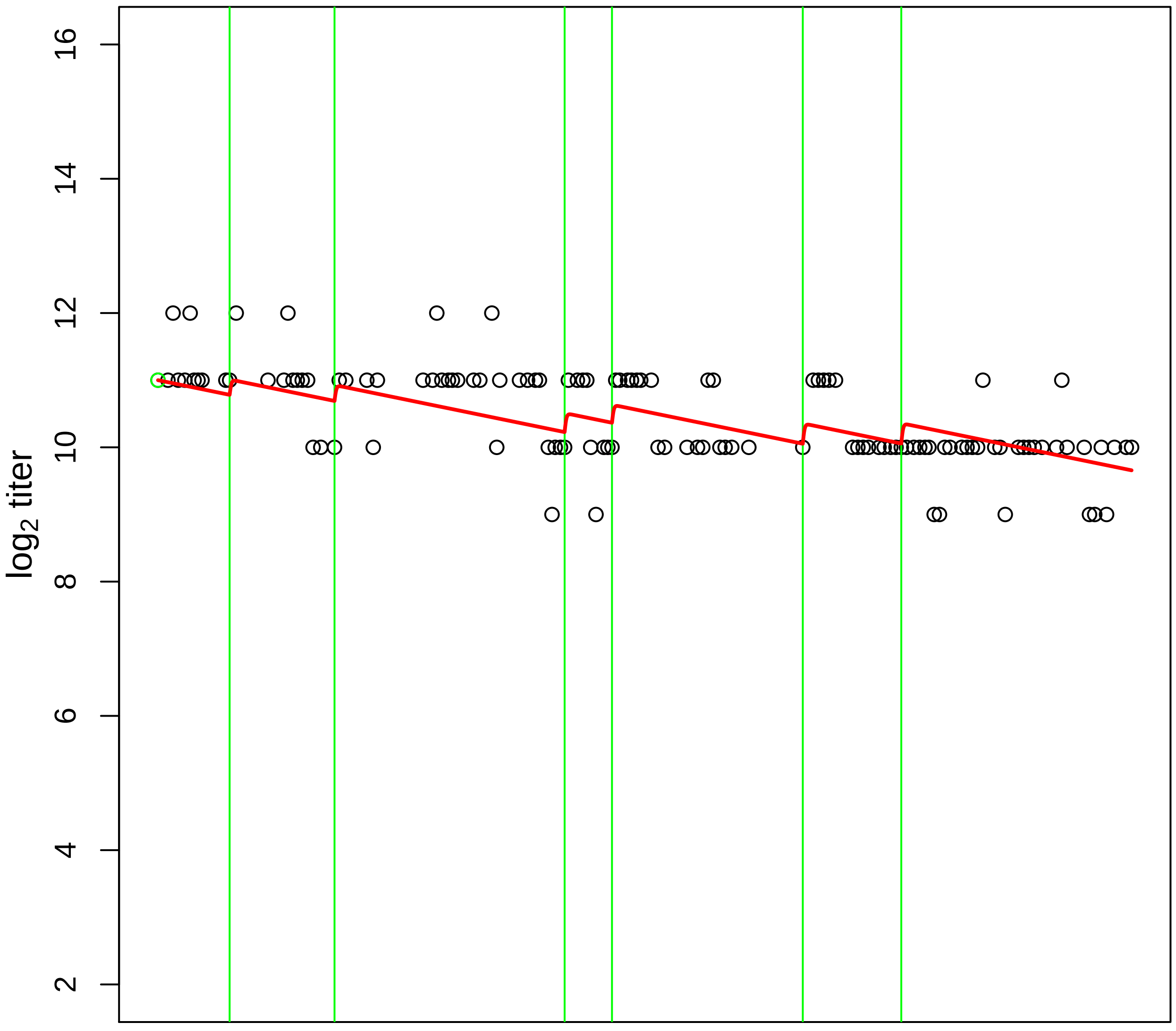

time in years from first donation of donor 123
mean absolute errors = 0.426 , mean squared errors = 0.298

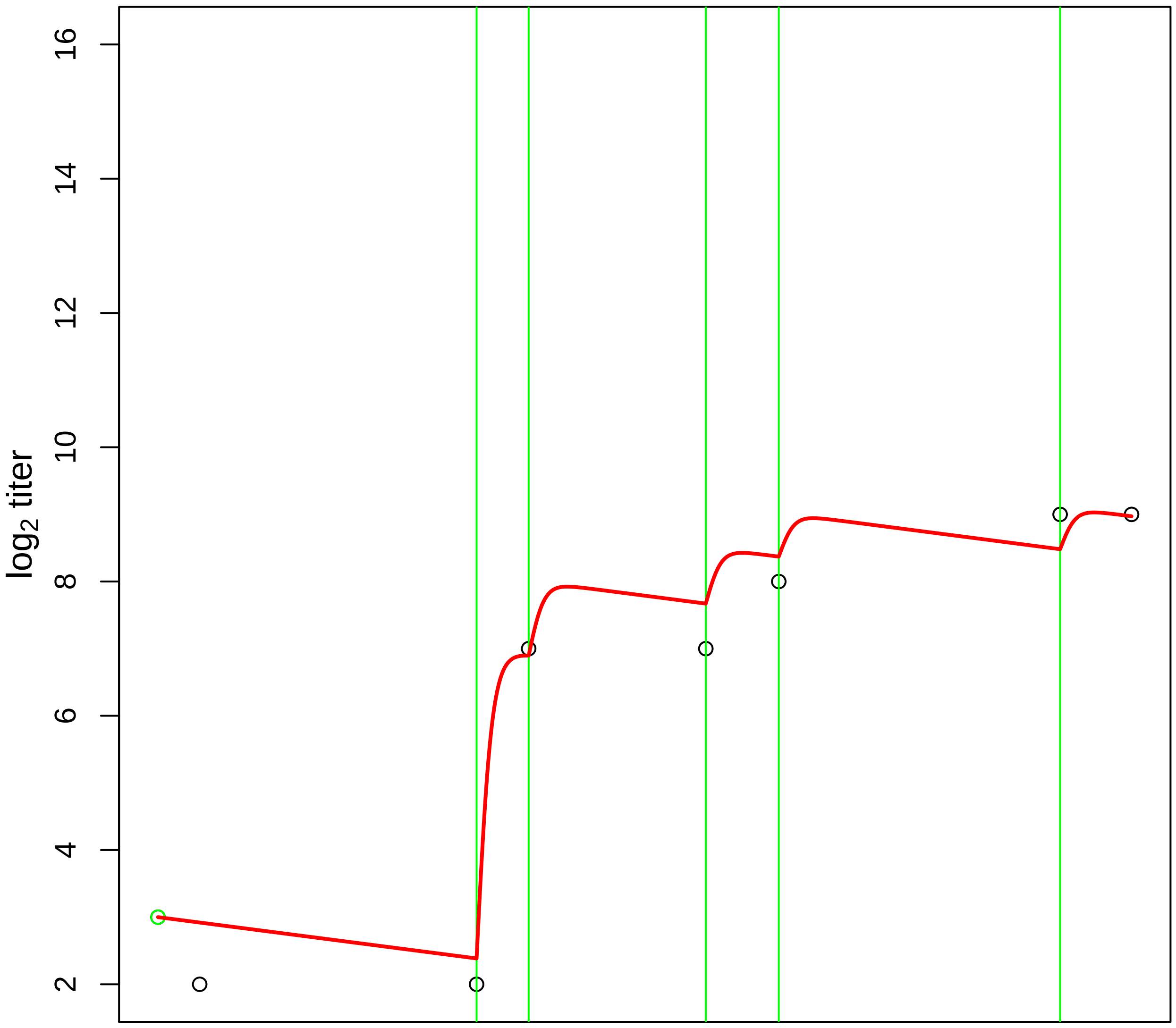

time in years from first donation of donor 124
mean absolute errors = 0.428 , mean squared errors = 0.266

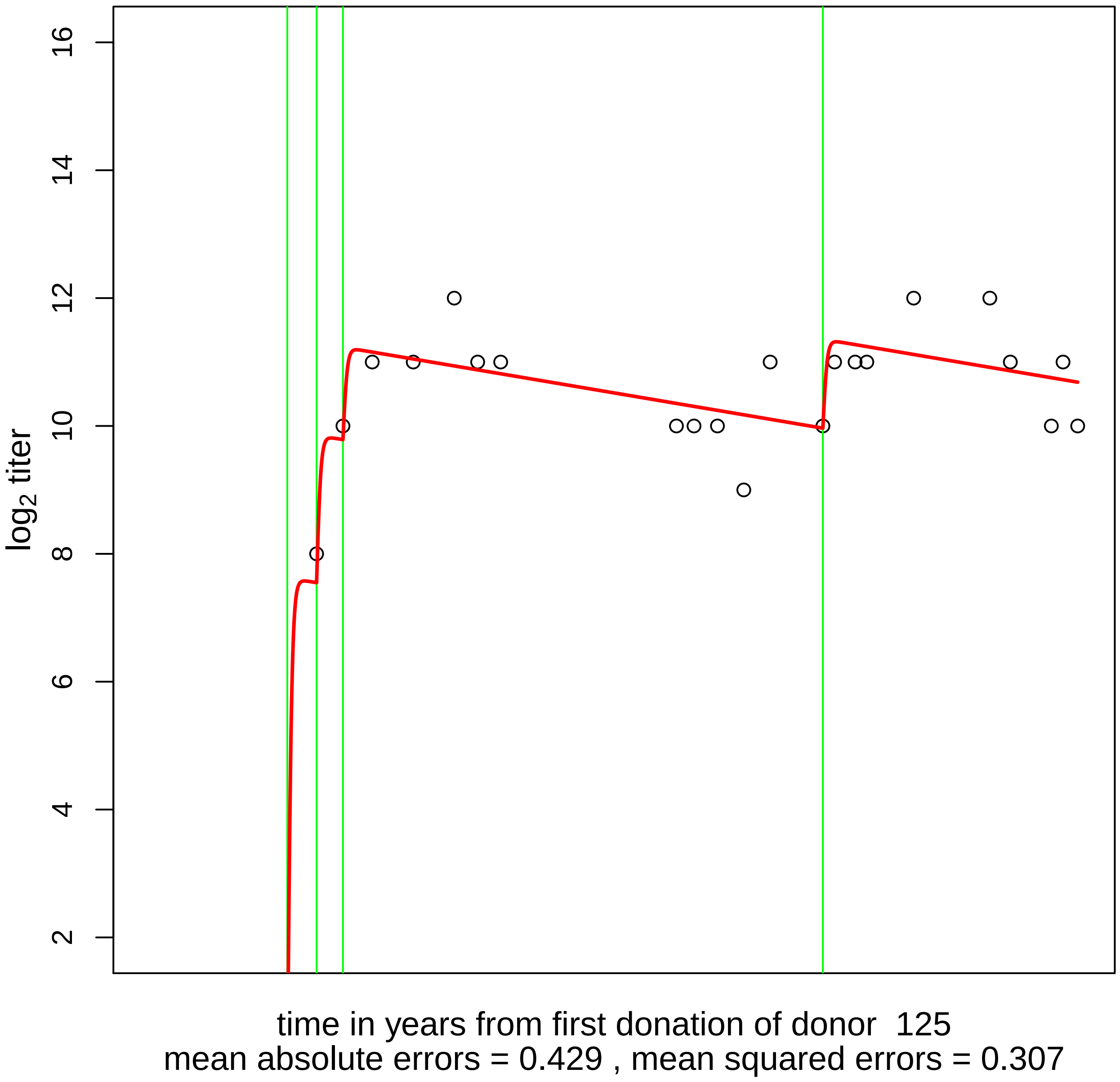

log₂ titer
2
4
6
8
10
12
14
16
time in years from first donation of donor 125
mean absolute errors = 0.429 , mean squared errors = 0.307

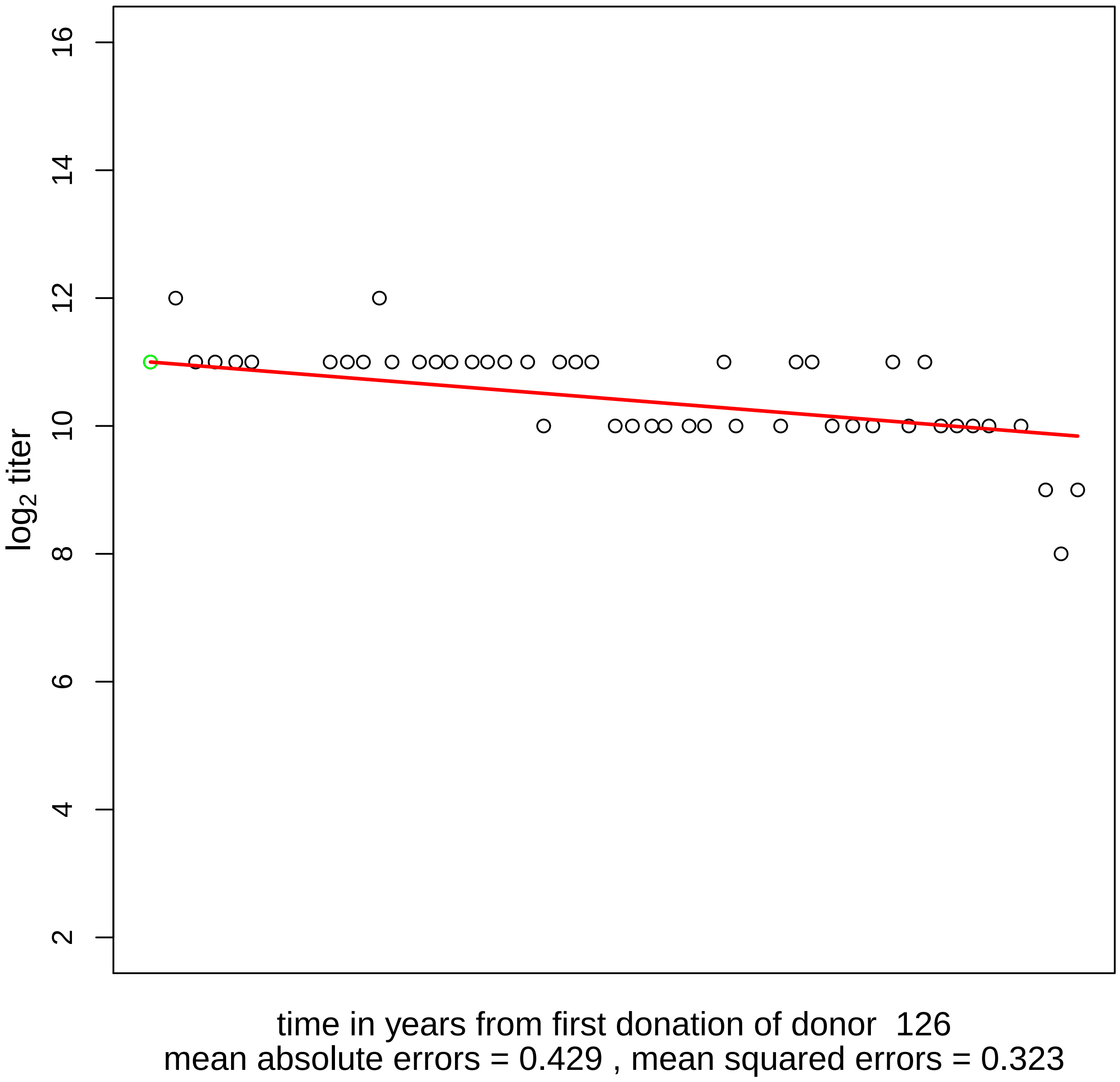

2
4
6
8
10
12
14
16
$\log_2$ titer
time in years from first donation of donor 126
mean absolute errors = 0.429 , mean squared errors = 0.323

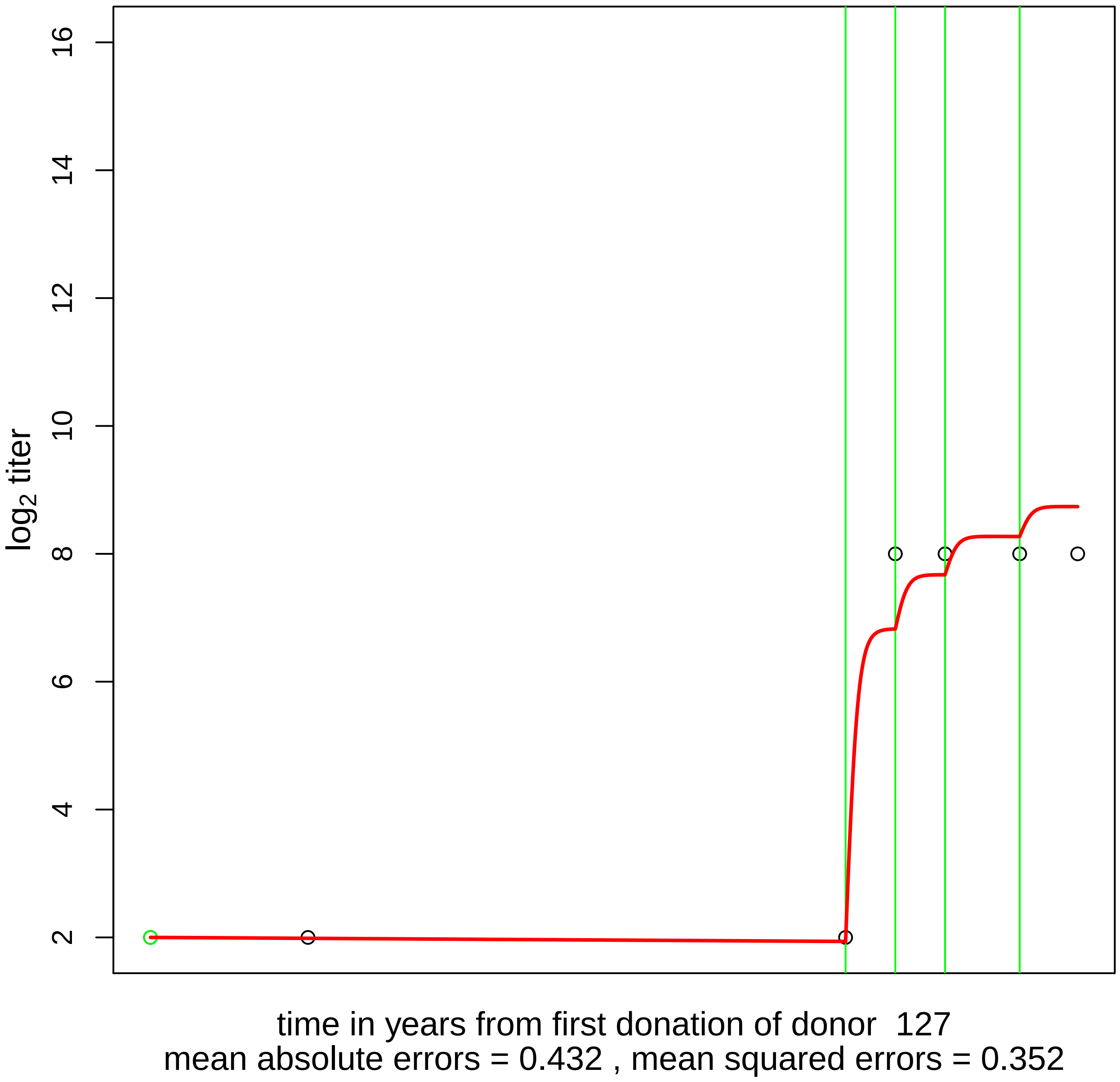

16
14
12
10
8
6
4
2
$\log_2$ titer
time in years from first donation of donor 127
mean absolute errors = 0.432 , mean squared errors = 0.352

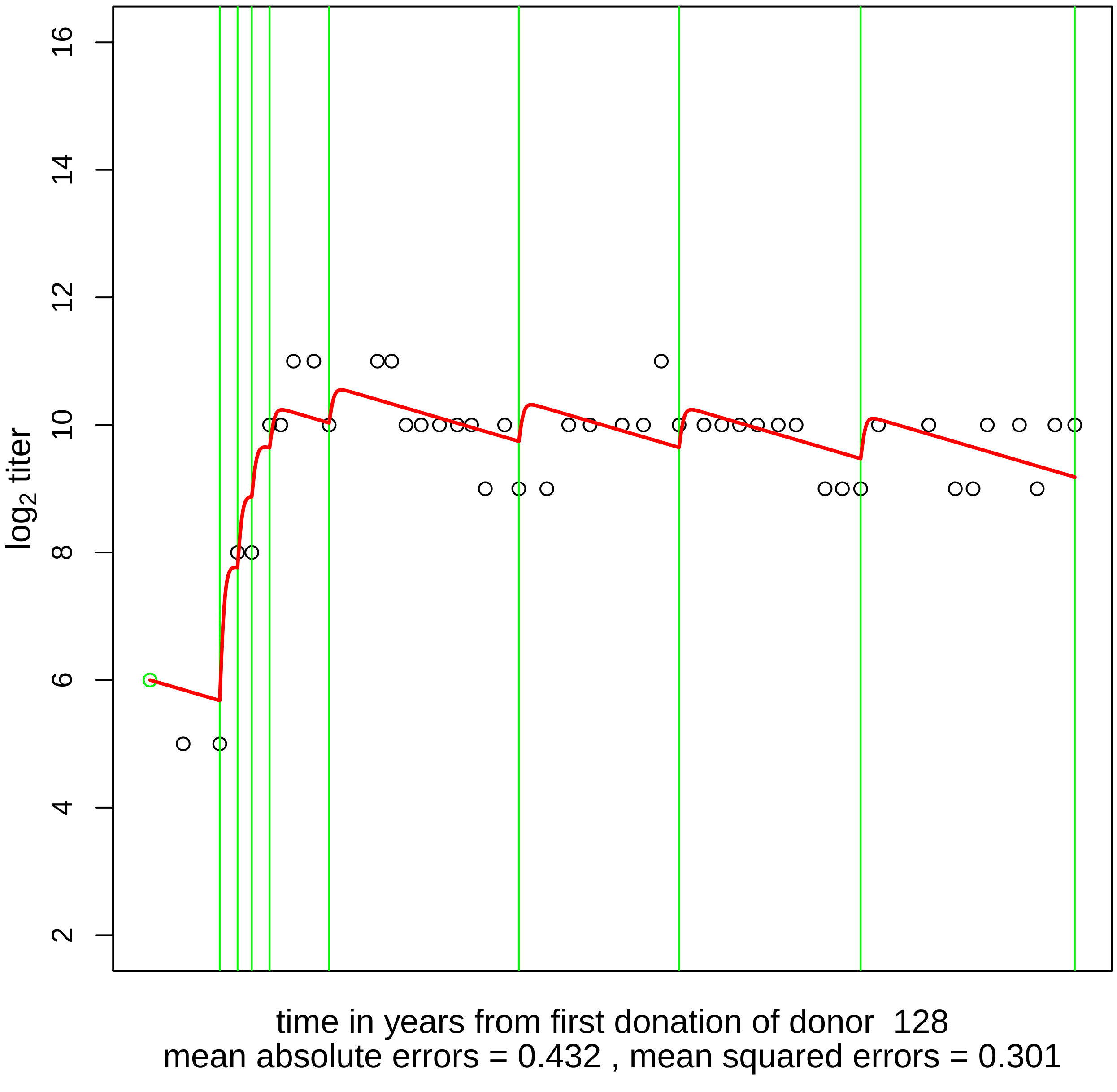

$\log_2$ titer
2
4
6
8
10
12
14
16
time in years from first donation of donor 128
mean absolute errors = 0.432 , mean squared errors = 0.301

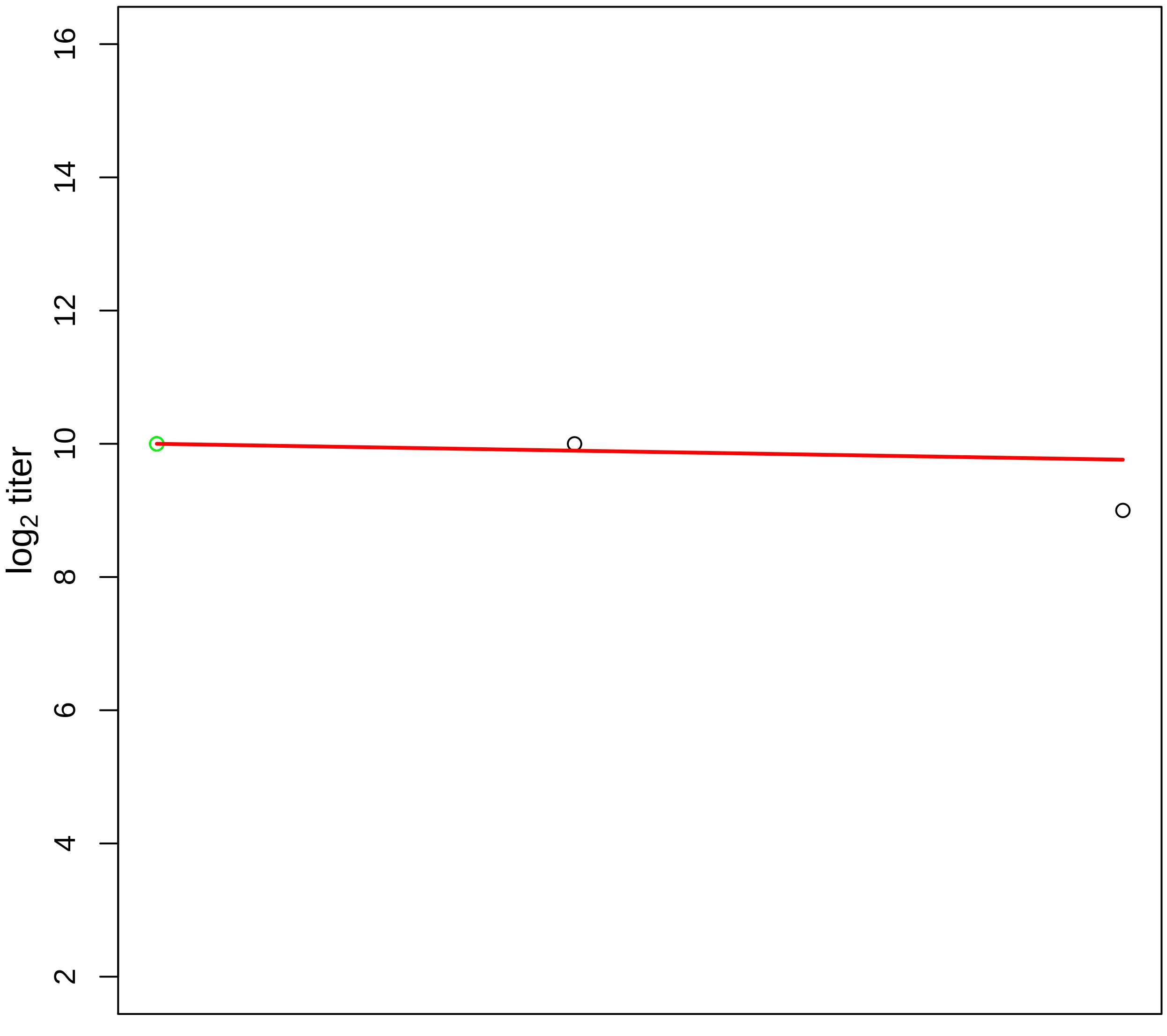

time in years from first donation of donor 129
mean absolute errors = 0.432 , mean squared errors = 0.295

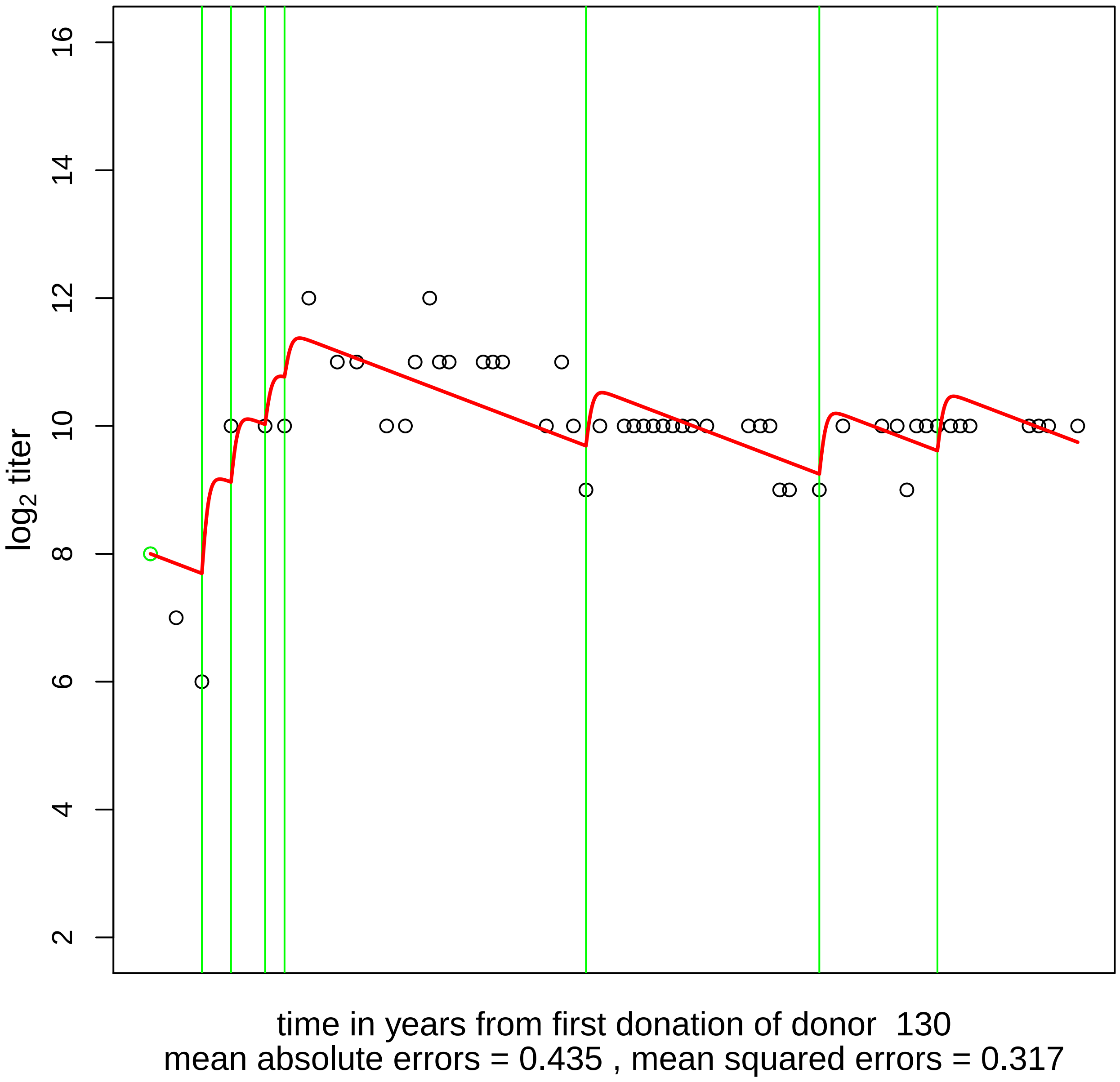

16
14
12
10
8
6
4
2
$\log_2$ titer
time in years from first donation of donor 130
mean absolute errors = 0.435 , mean squared errors = 0.317

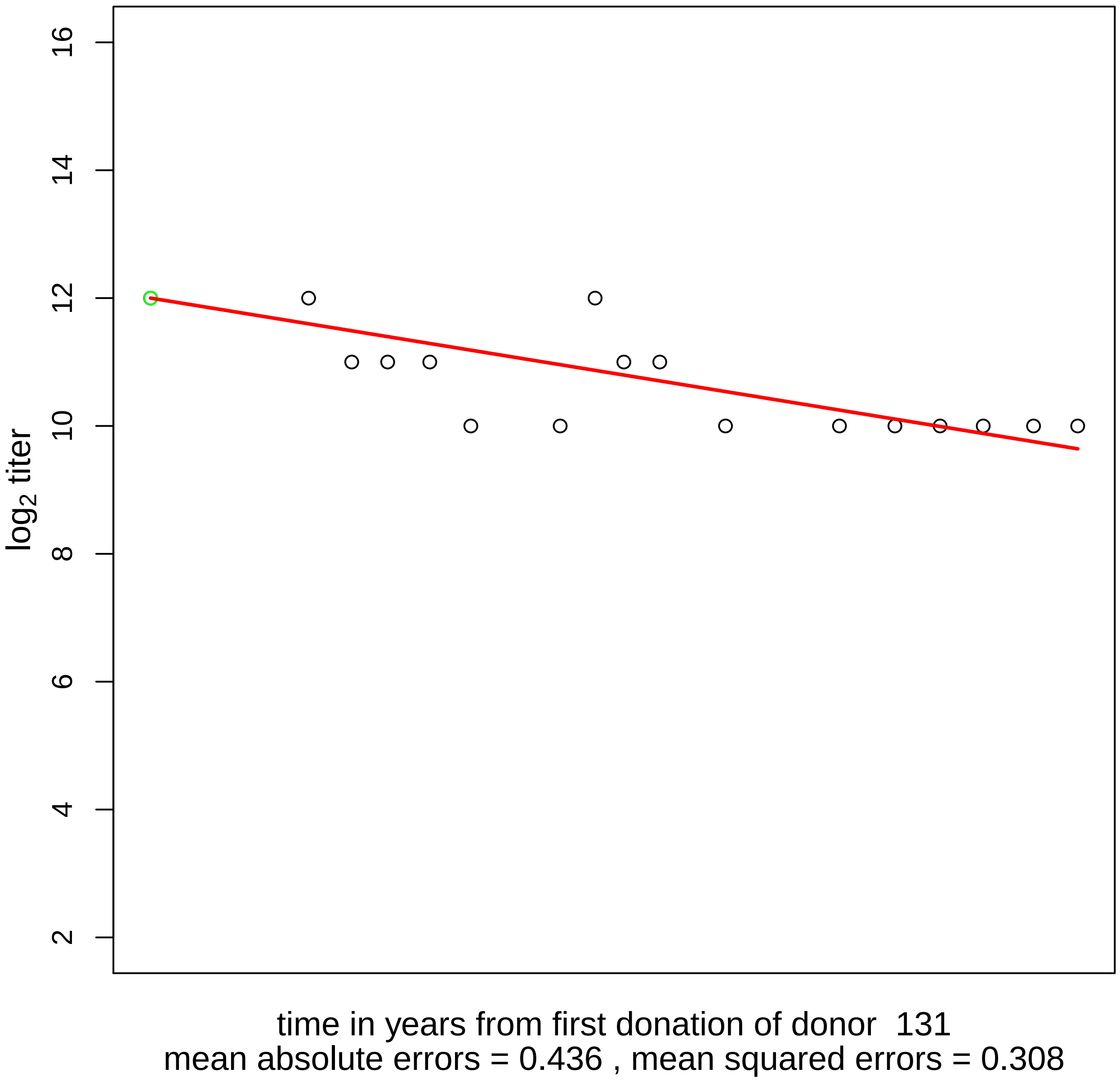

16
14
12
10
8
6
4
2
log2 titer
time in years from first donation of donor 131
mean absolute errors = 0.436 , mean squared errors = 0.308

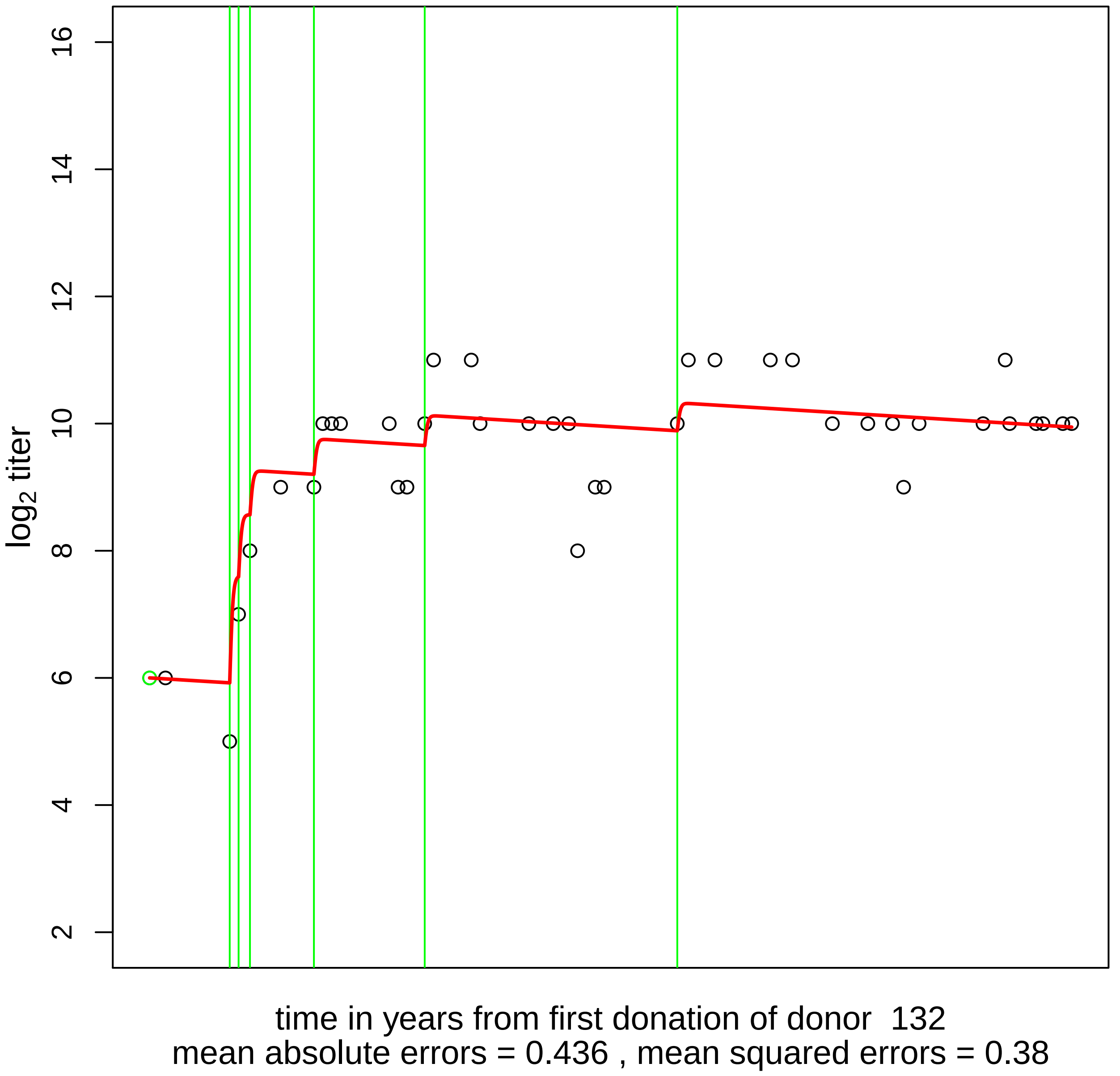

$\log_2$ titer
2
4
6
8
10
12
14
16
time in years from first donation of donor 132
mean absolute errors = 0.436 , mean squared errors = 0.38

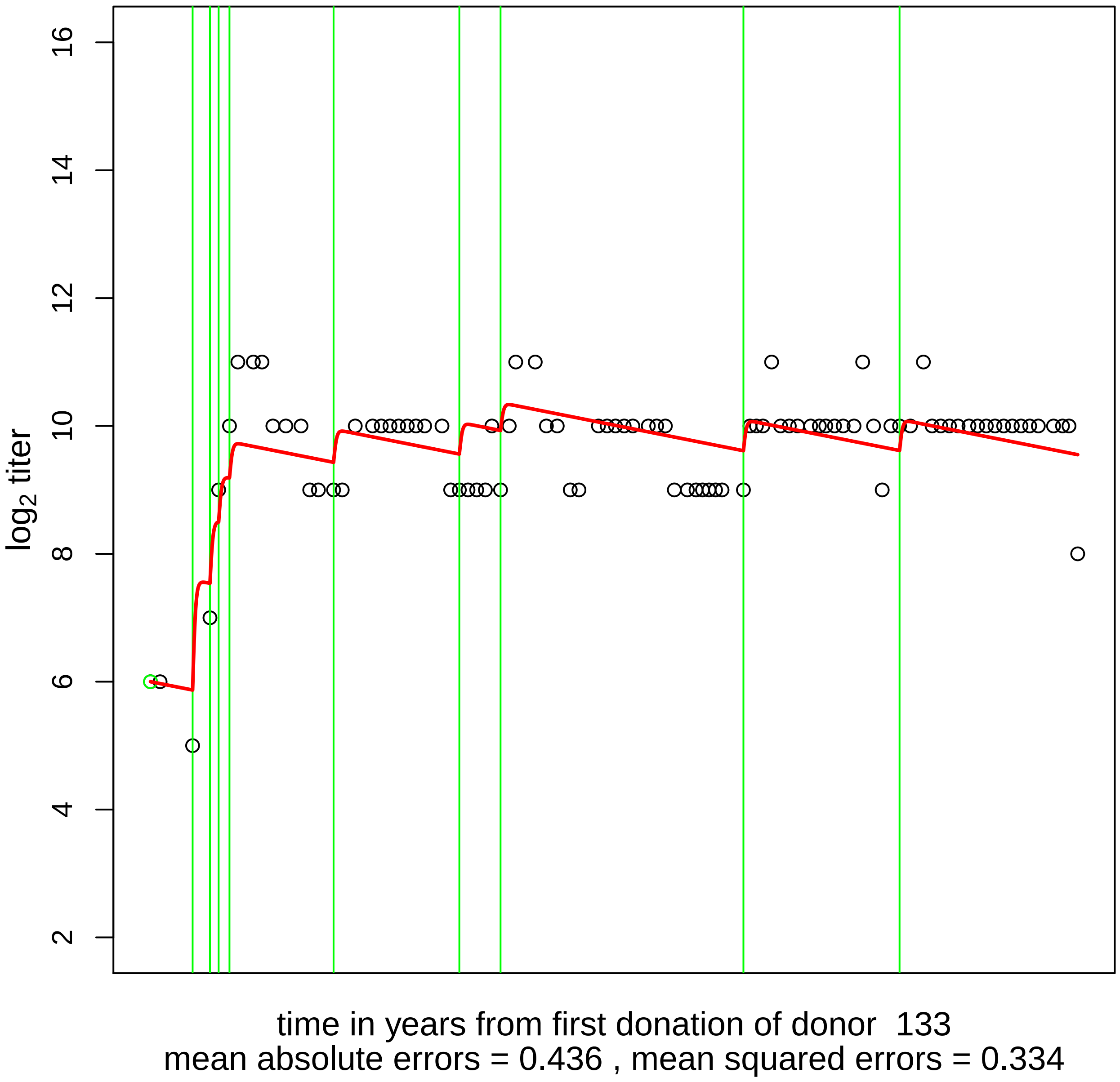

16
14
12
10
8
6
4
2
$\log_2$ titer
time in years from first donation of donor 133
mean absolute errors = 0.436 , mean squared errors = 0.334

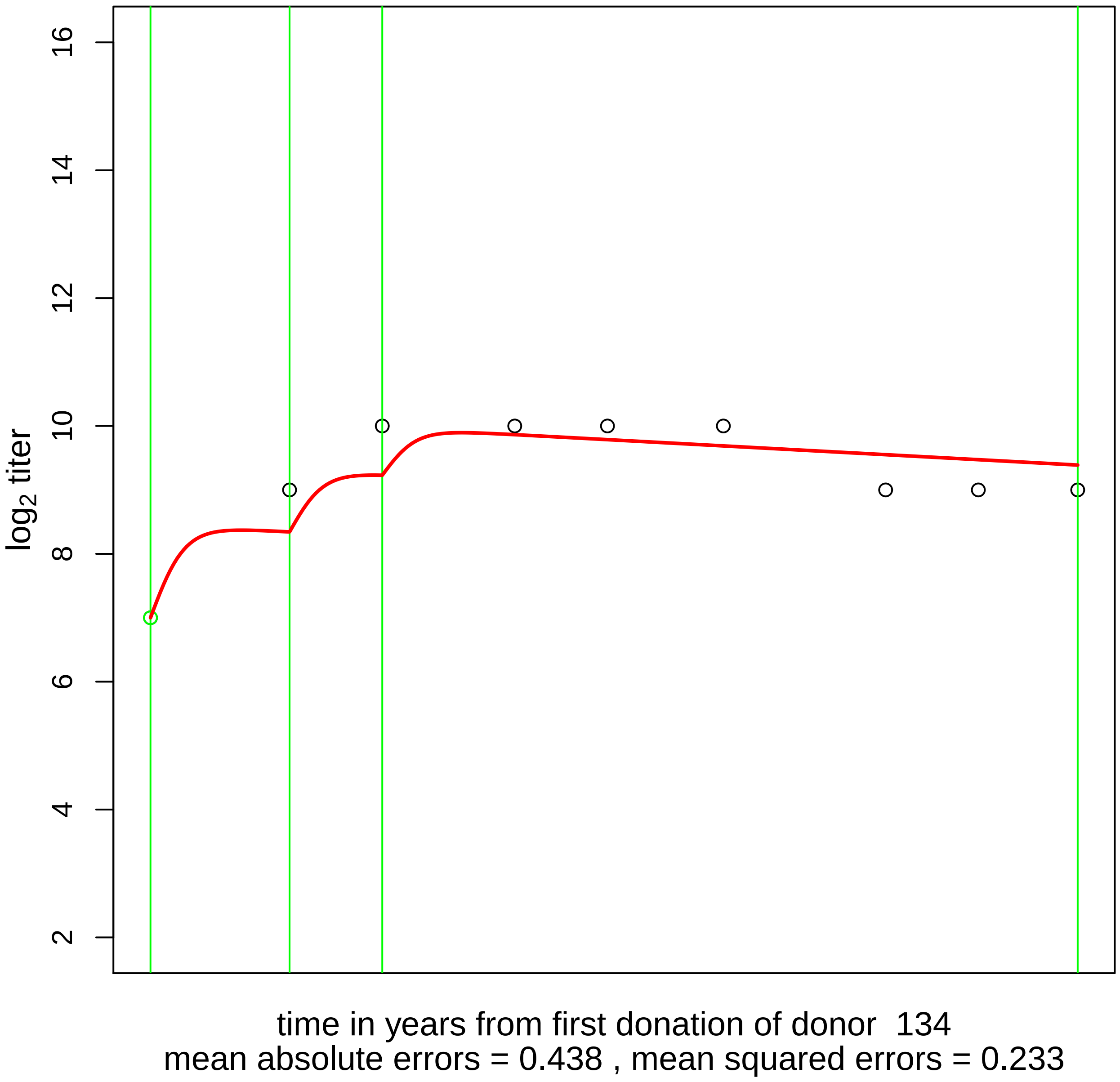
log2 titer
16
14
12
10
8
6
4
2
time in years from first donation of donor 134
mean absolute errors = 0.438 , mean squared errors = 0.233

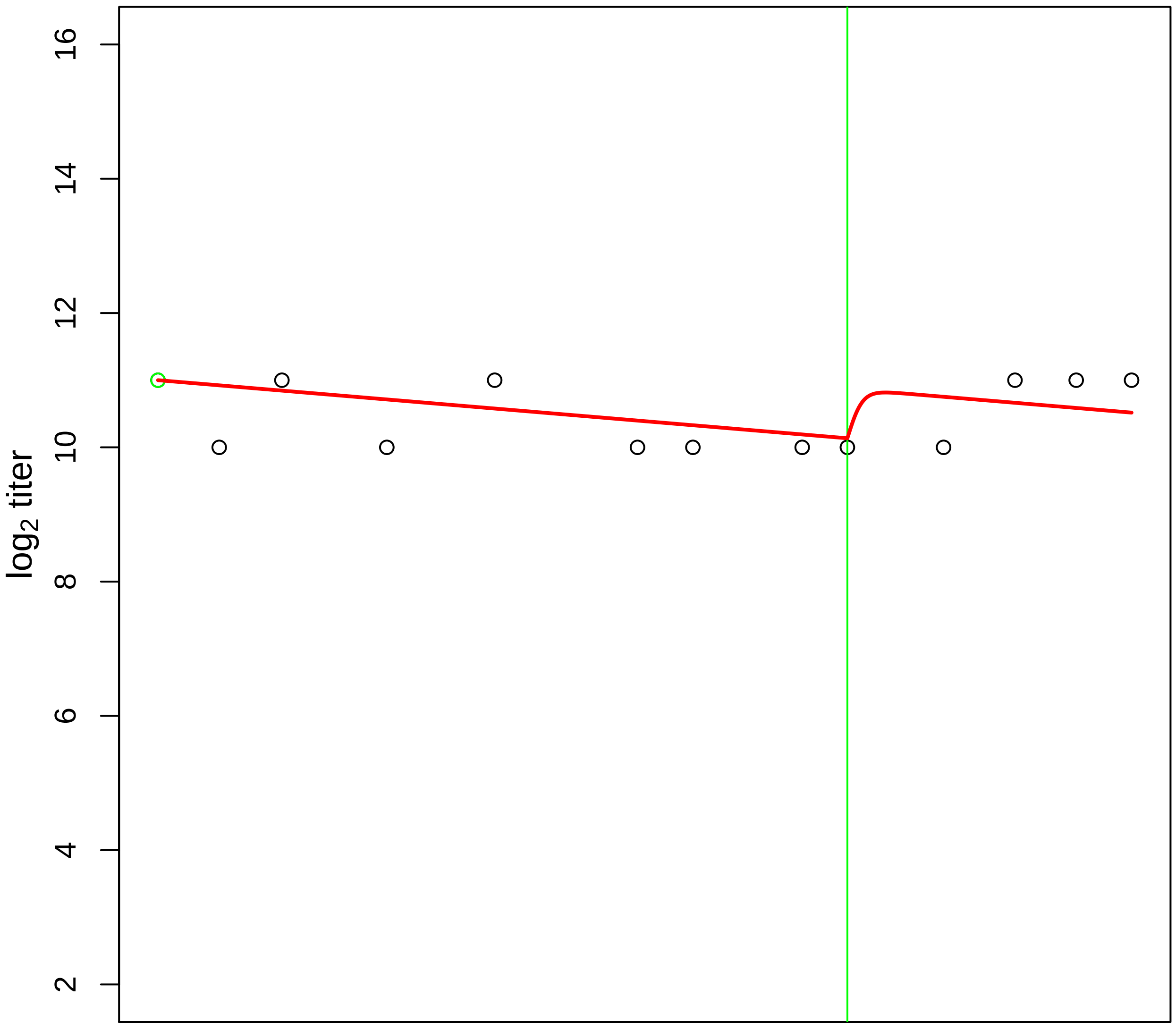

time in years from first donation of donor 135
mean absolute errors = 0.438 , mean squared errors = 0.247

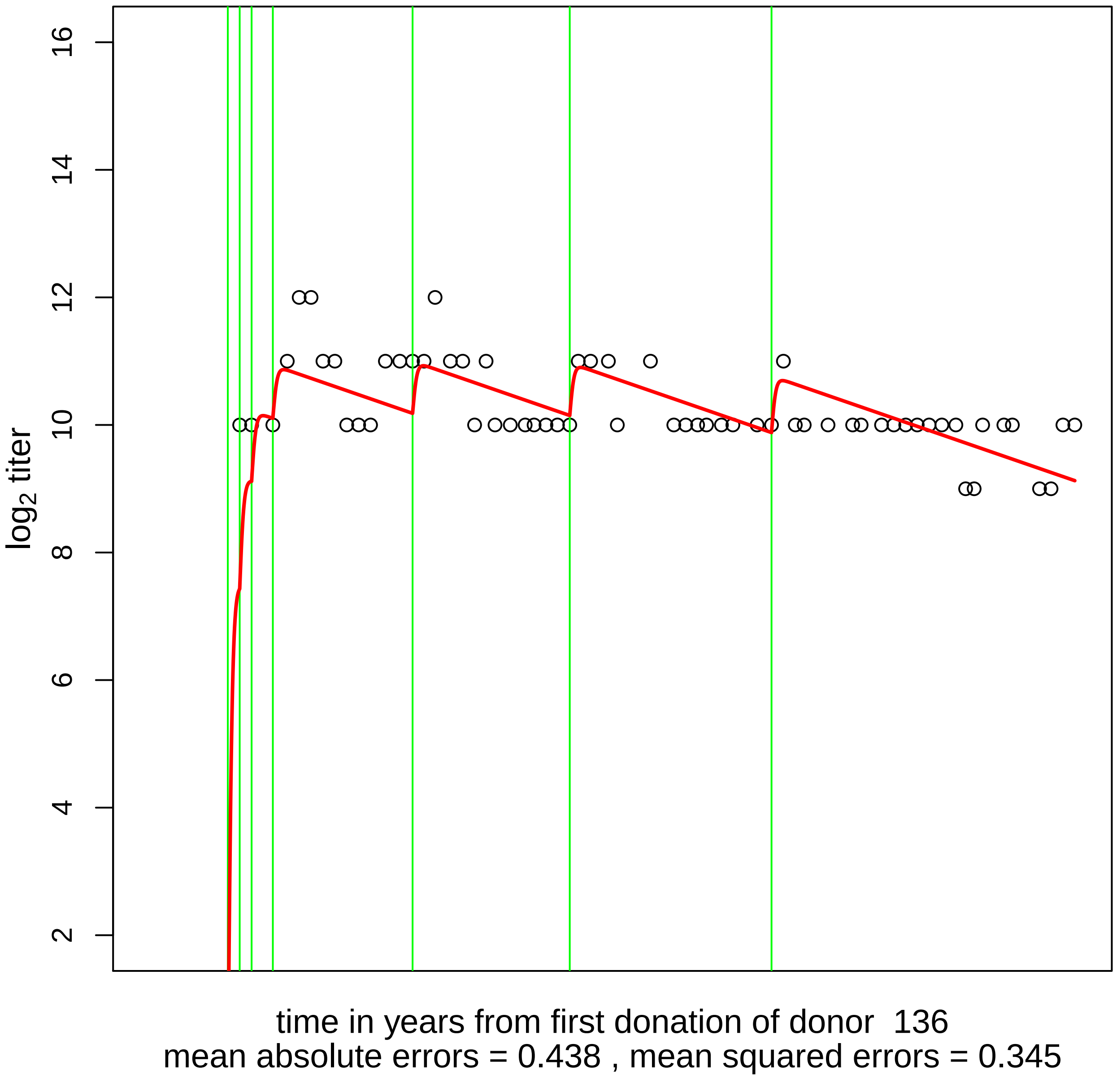
16
14
12
10
8
6
4
2
log2 titer
time in years from first donation of donor 136
mean absolute errors = 0.438 , mean squared errors = 0.345

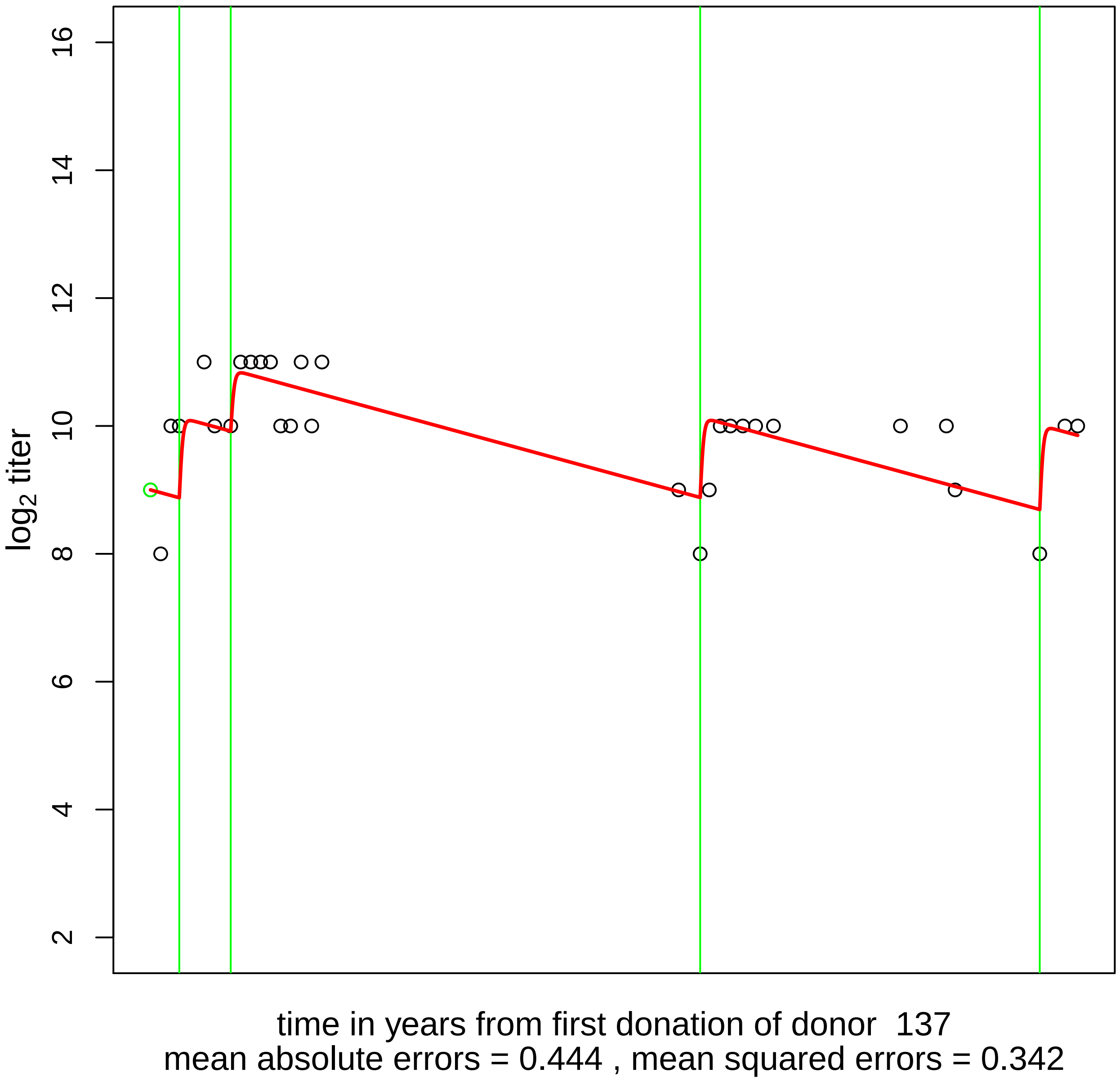

16
14
12
10
8
6
4
2
log2 titer
time in years from first donation of donor 137
mean absolute errors = 0.444 , mean squared errors = 0.342

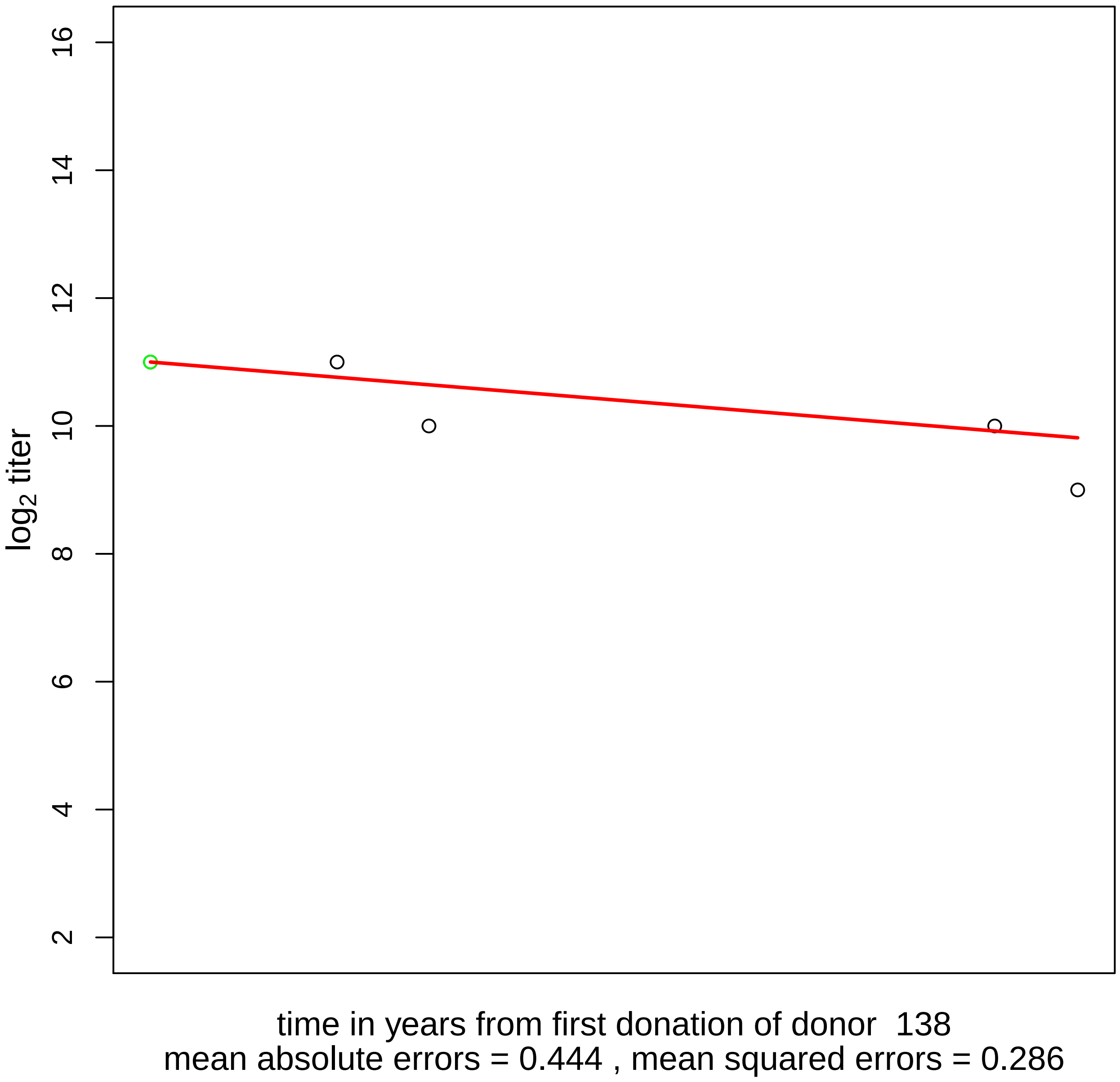

16
14
12
10
8
6
4
2
log2 titer
time in years from first donation of donor 138
mean absolute errors = 0.444 , mean squared errors = 0.286

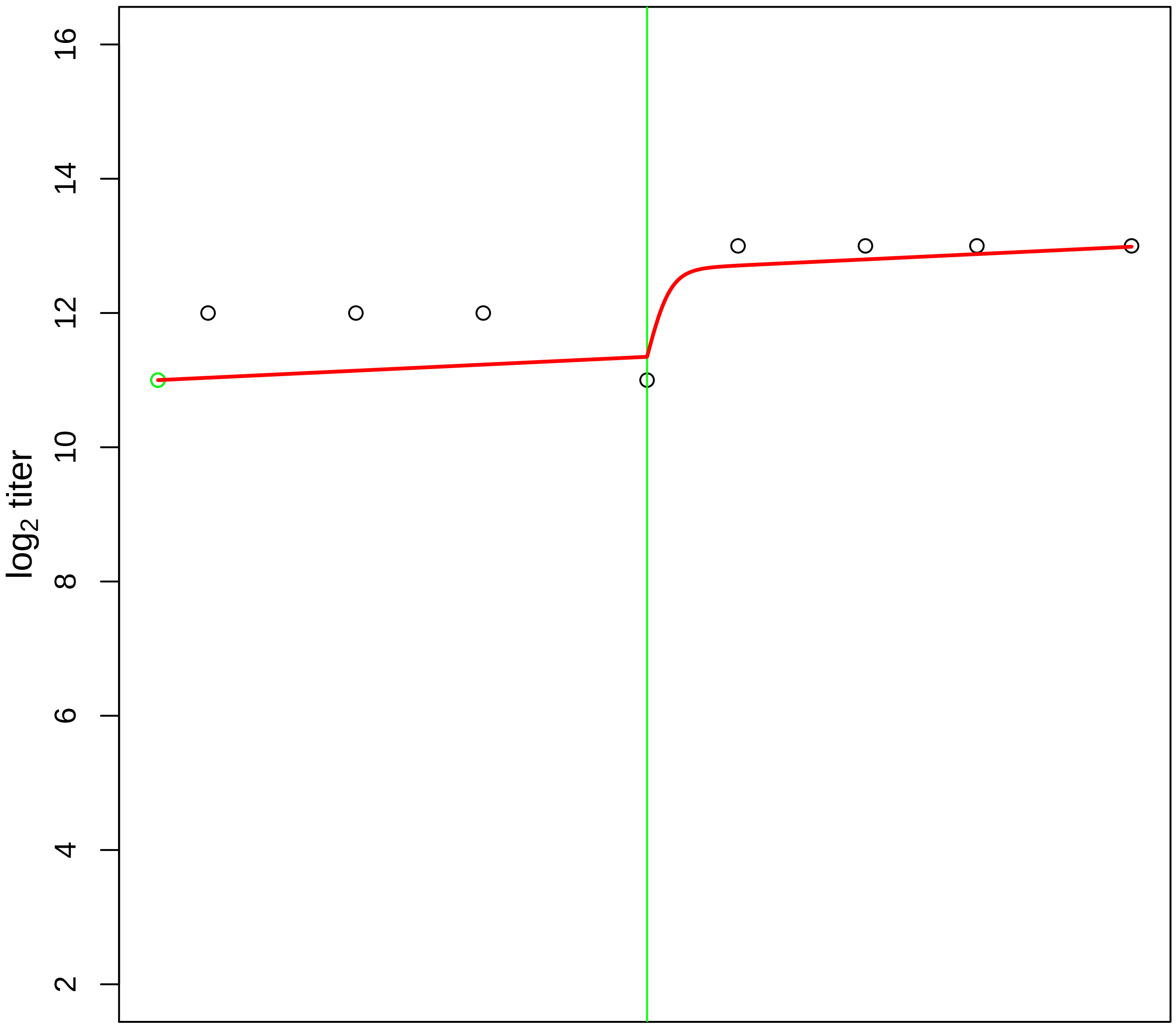

time in years from first donation of donor 139
mean absolute errors = 0.446 , mean squared errors = 0.315

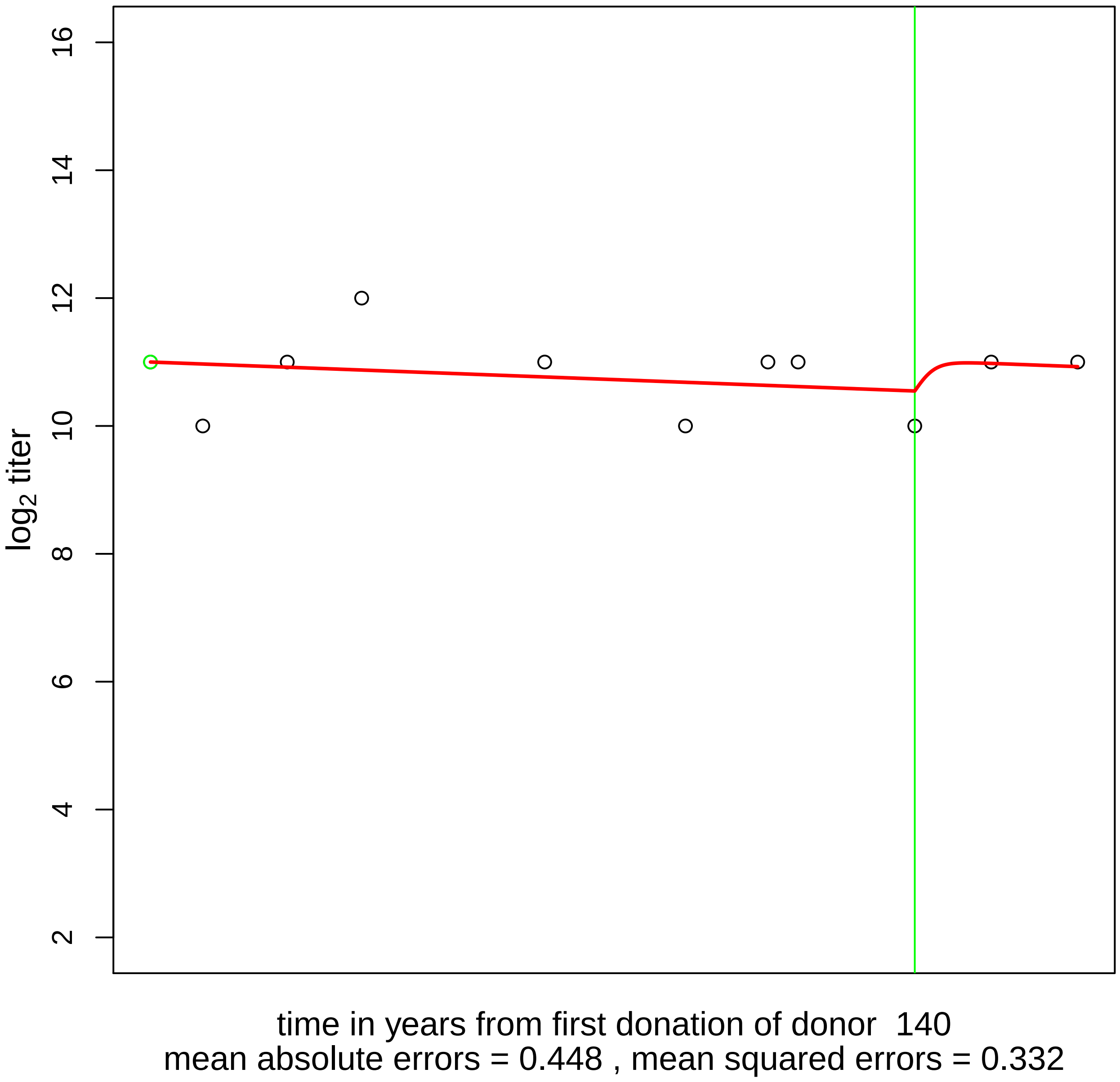

2
4
6
8
10
12
14
16
log2 titer
time in years from first donation of donor 140
mean absolute errors = 0.448 , mean squared errors = 0.332

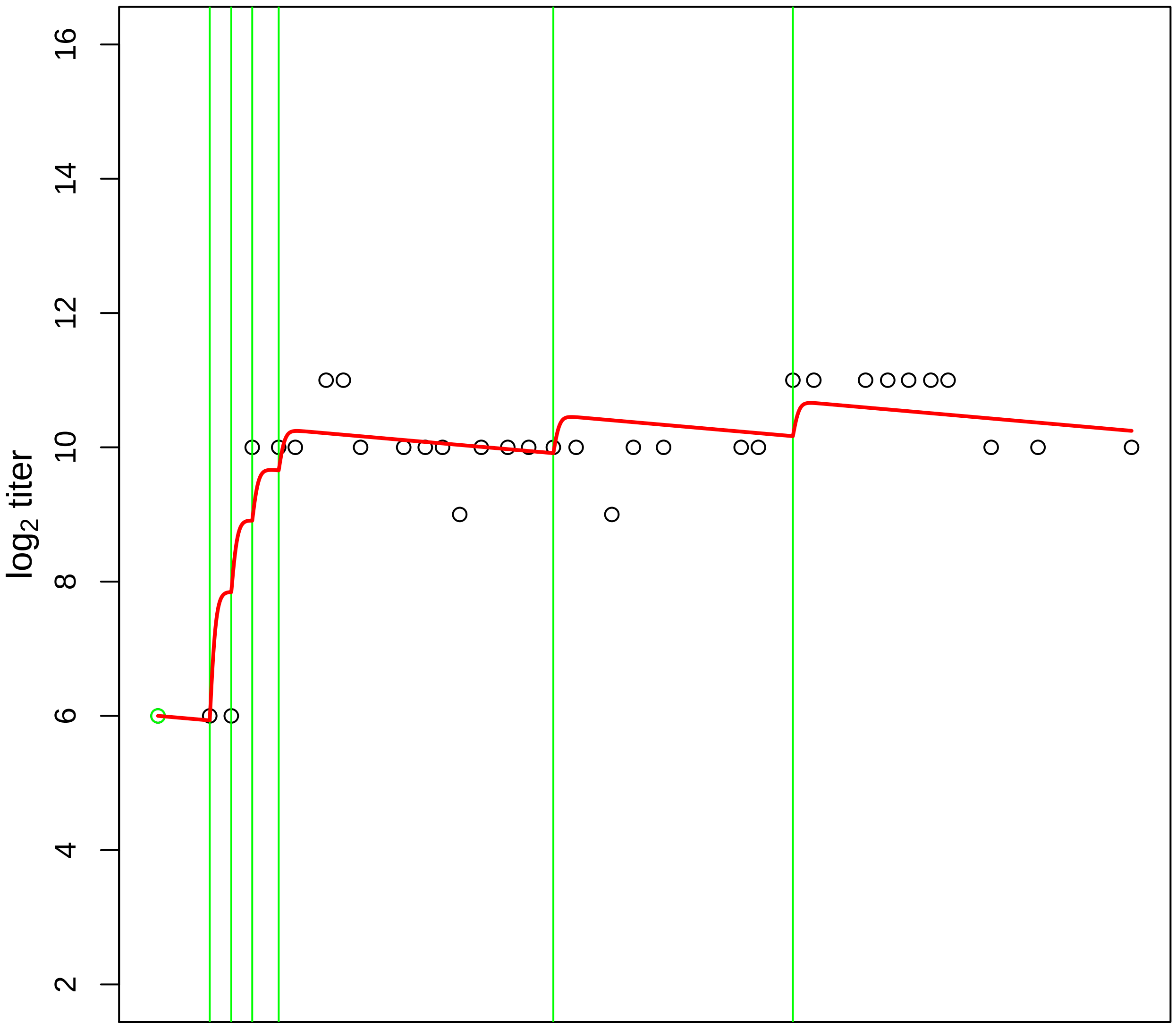

time in years from first donation of donor 141
mean absolute errors = 0.448 , mean squared errors = 0.374

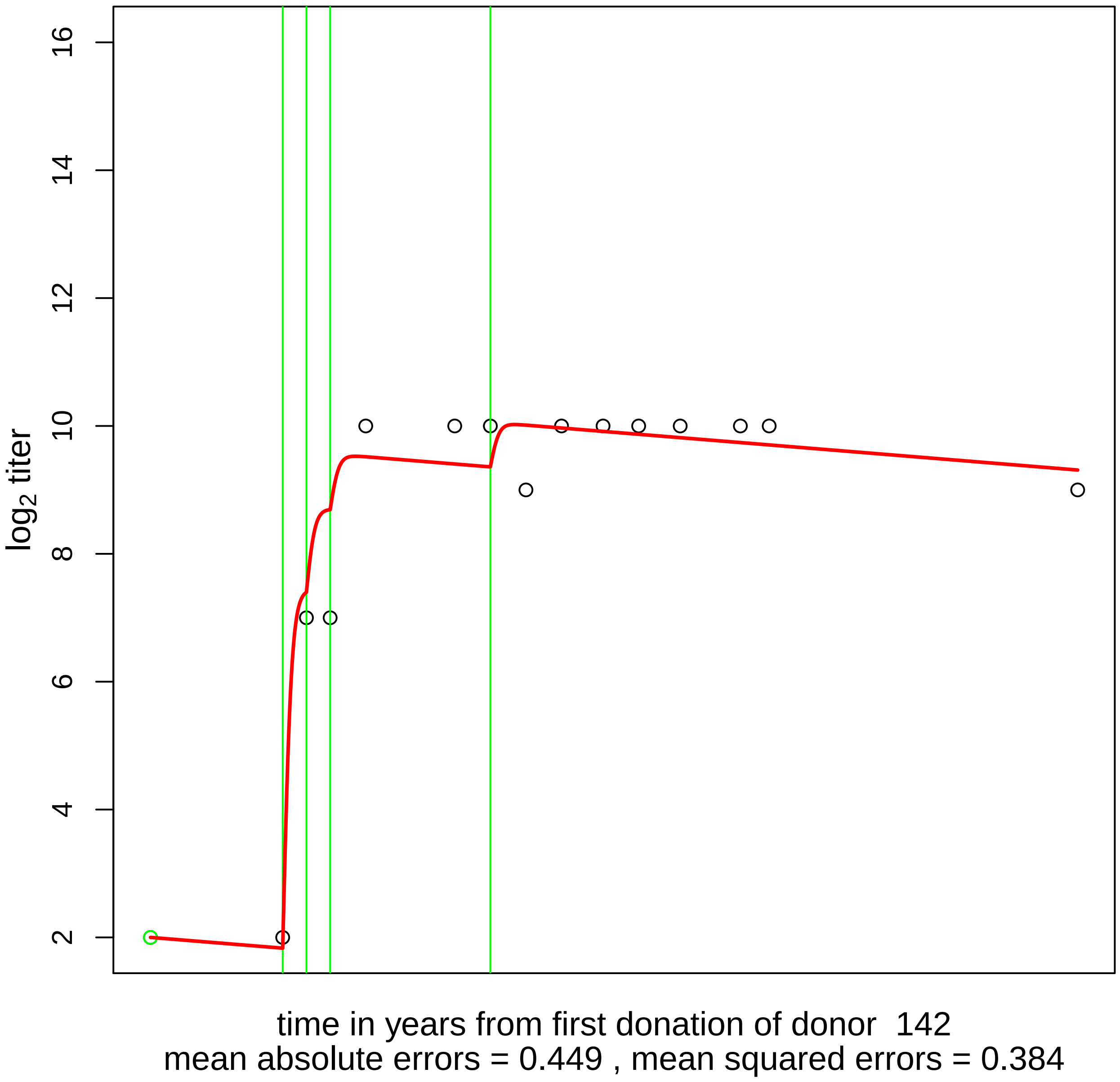
log2 titer
2
4
6
8
10
12
14
16
time in years from first donation of donor 142
mean absolute errors = 0.449 , mean squared errors = 0.384

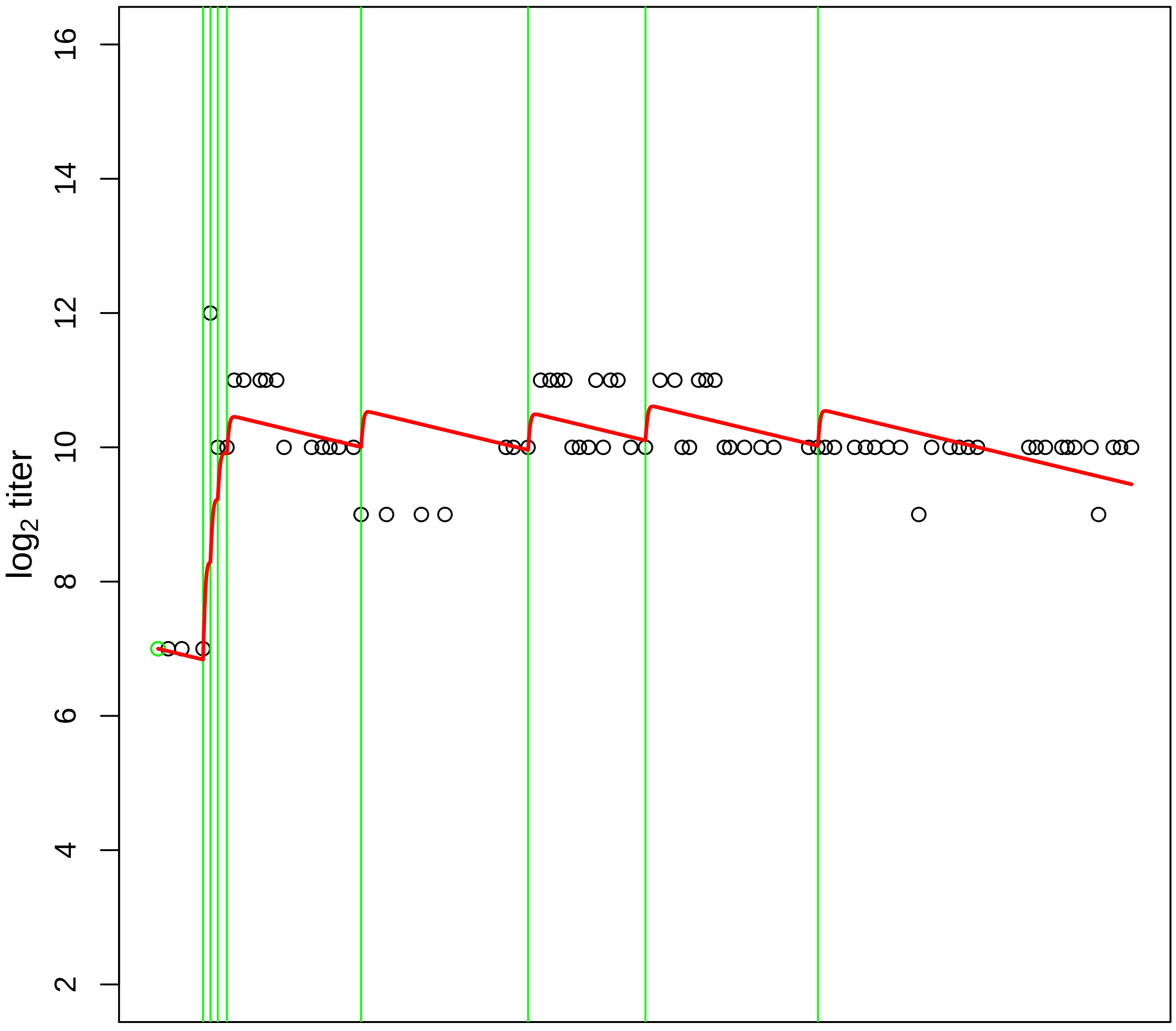

time in years from first donation of donor 143
mean absolute errors = 0.449 , mean squared errors = 0.444

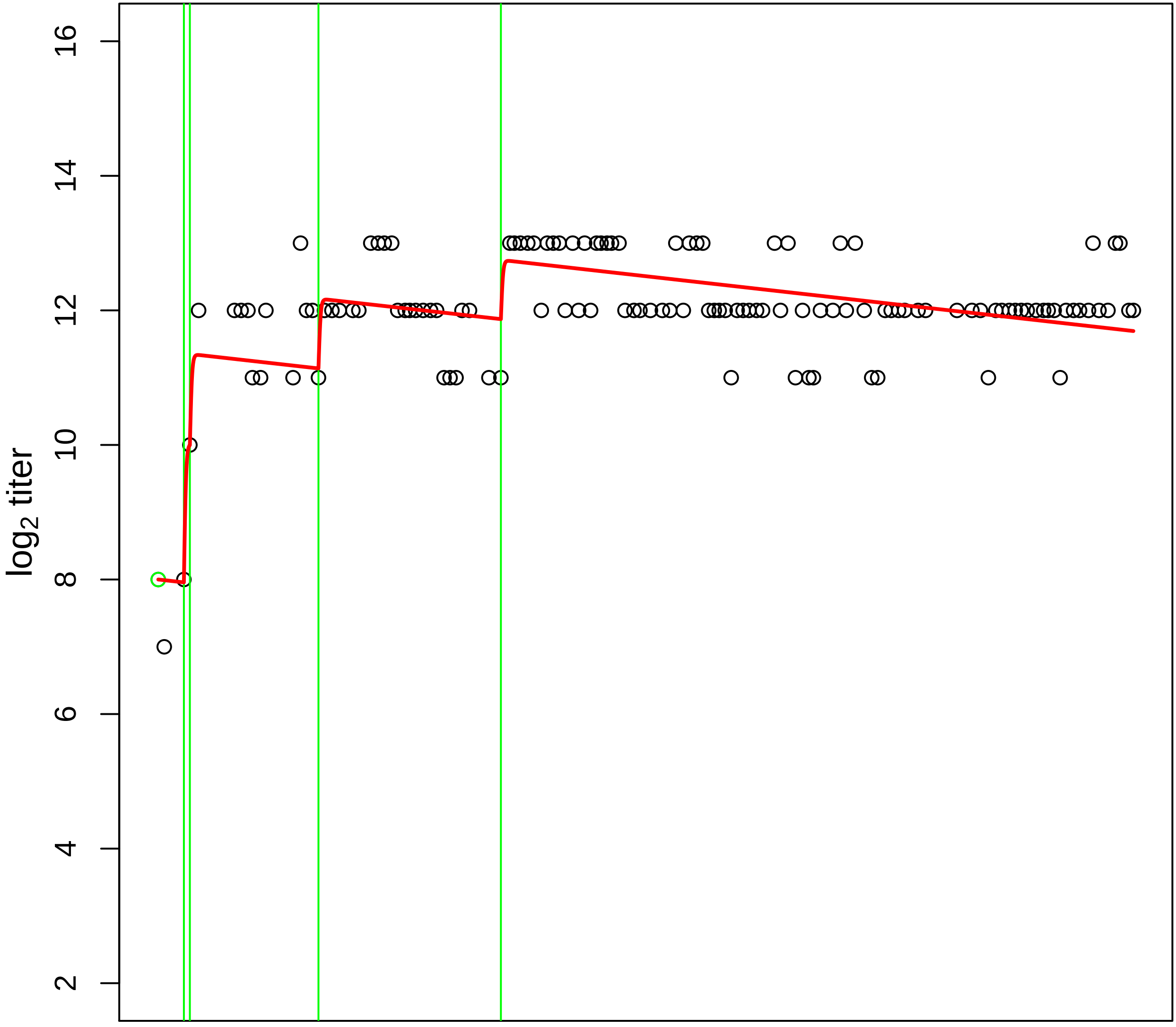

time in years from first donation of donor 144
mean absolute errors = 0.45 , mean squared errors = 0.344

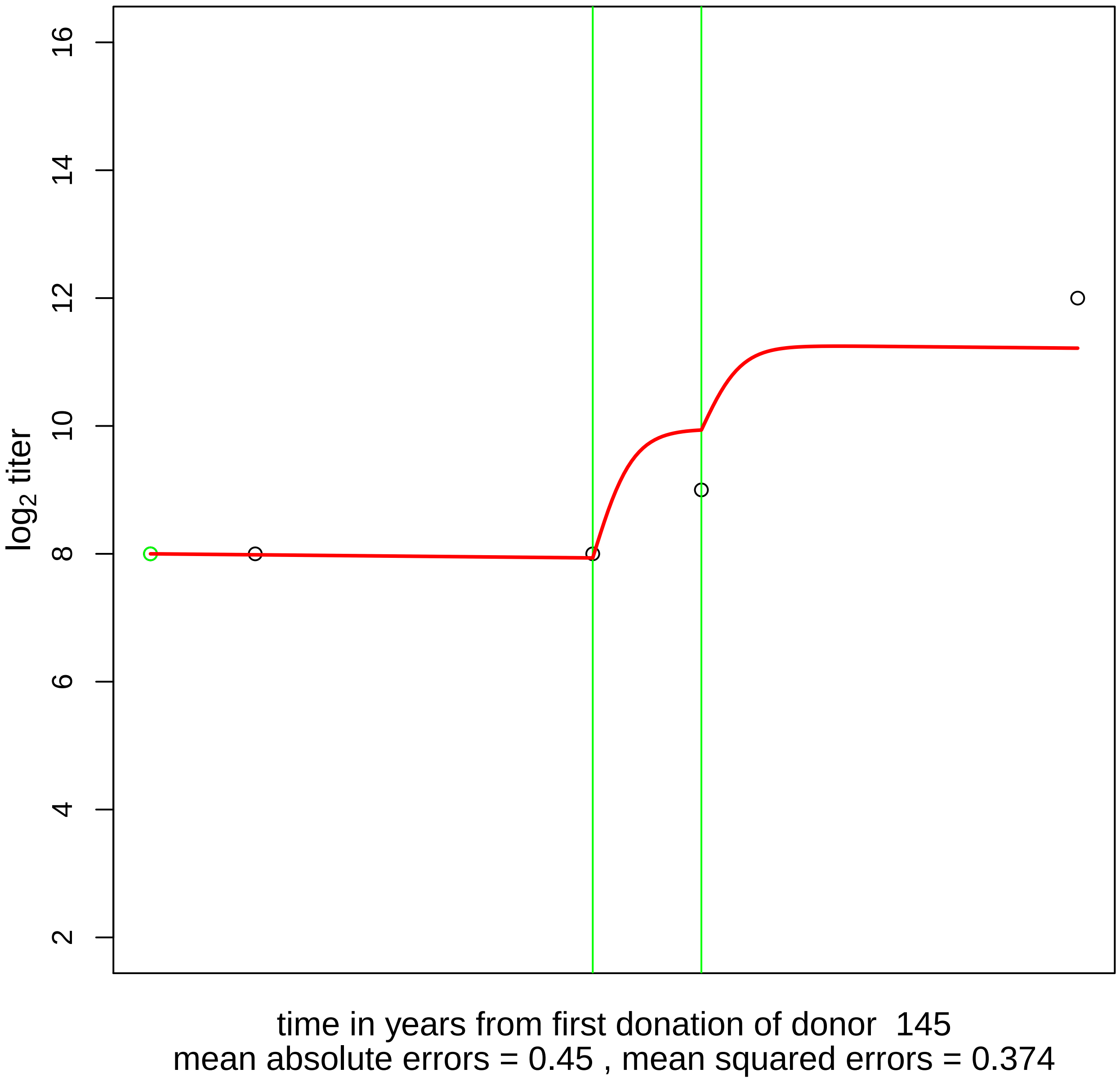

log2 titer
2
4
6
8
10
12
14
16
time in years from first donation of donor 145
mean absolute errors = 0.45 , mean squared errors = 0.374

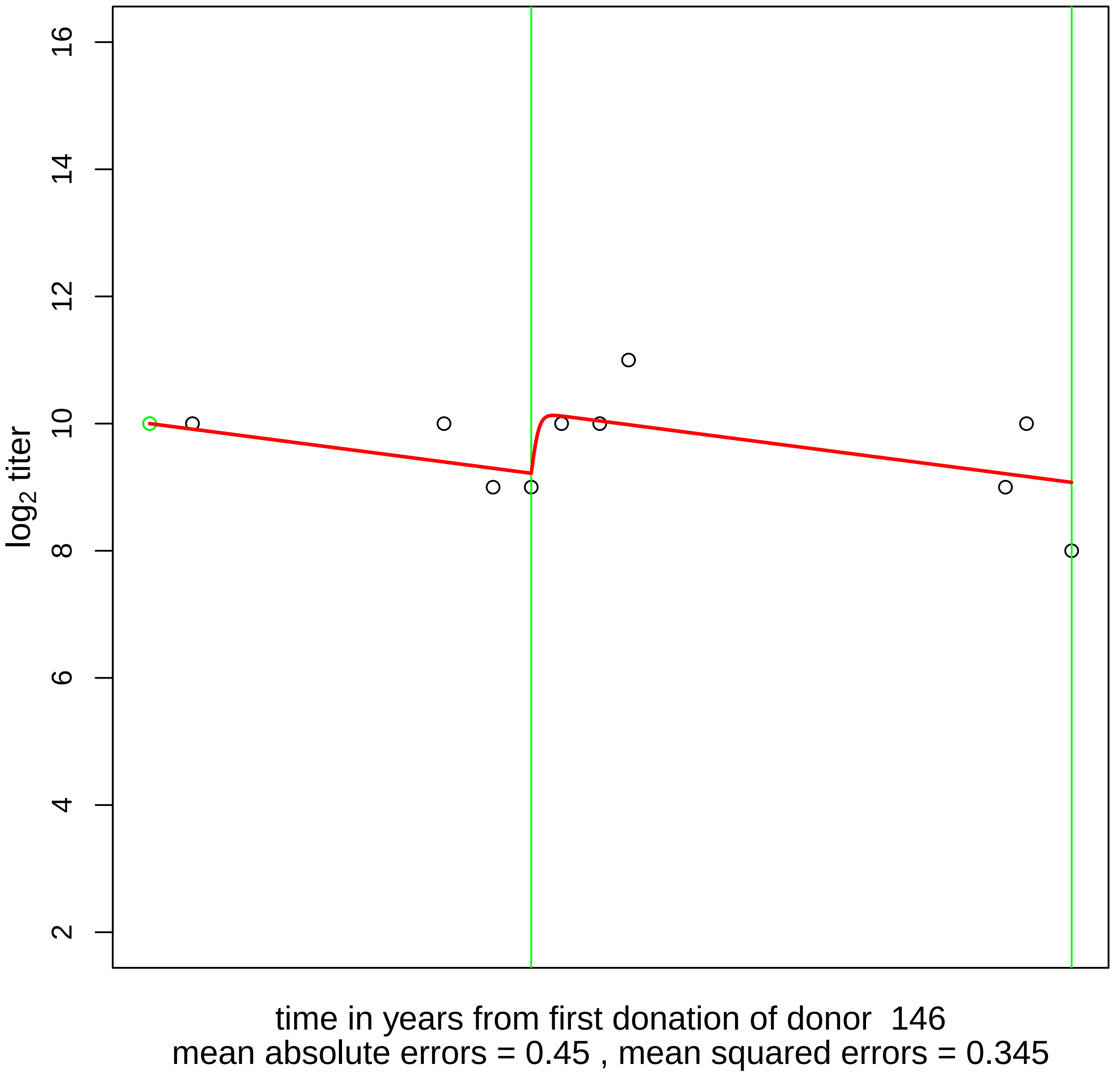

log2 titer
2
4
6
8
10
12
14
16
time in years from first donation of donor 146
mean absolute errors = 0.45 , mean squared errors = 0.345

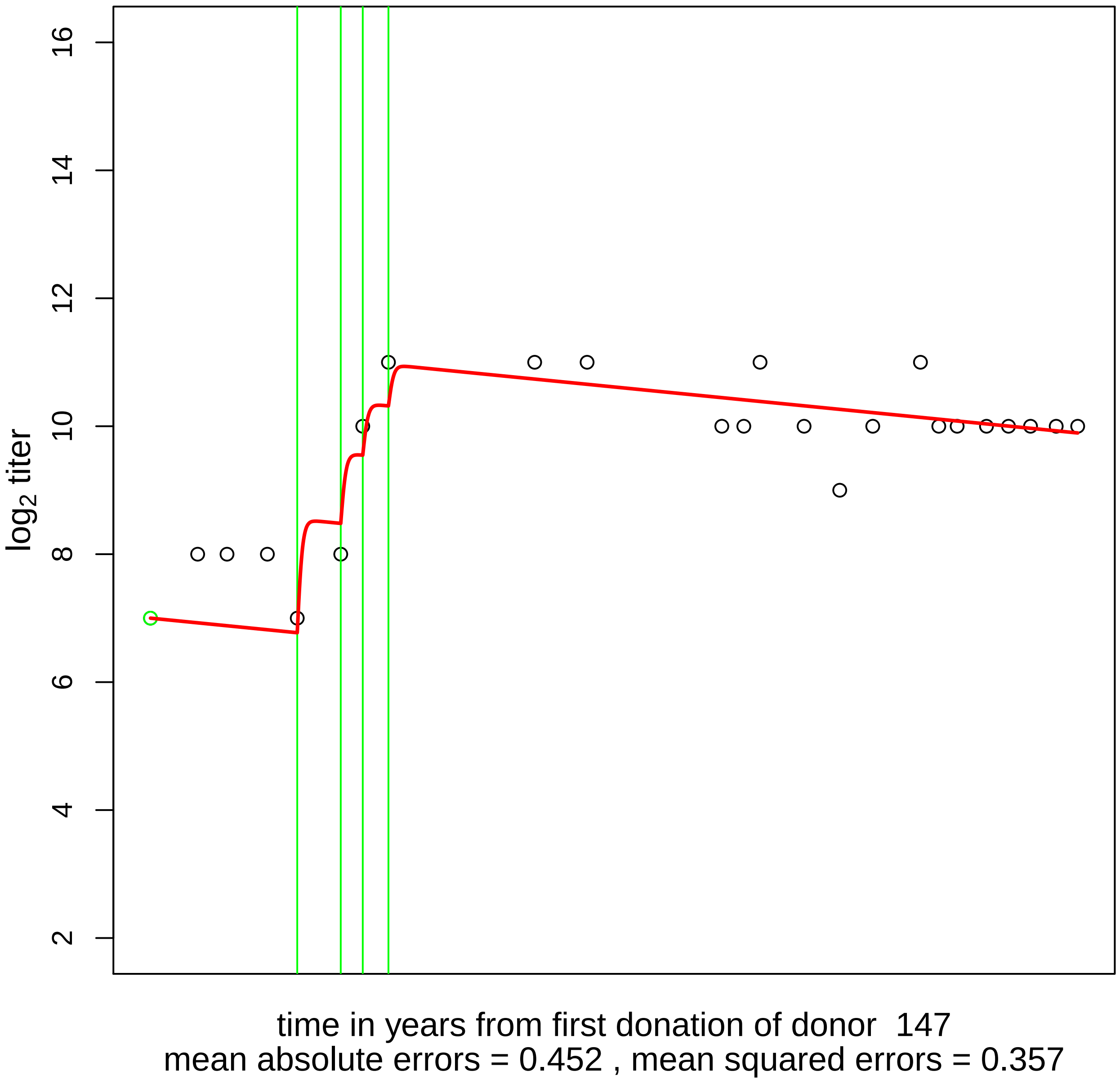

16
14
12
10
8
6
4
2
log2 titer
time in years from first donation of donor 147
mean absolute errors = 0.452 , mean squared errors = 0.357

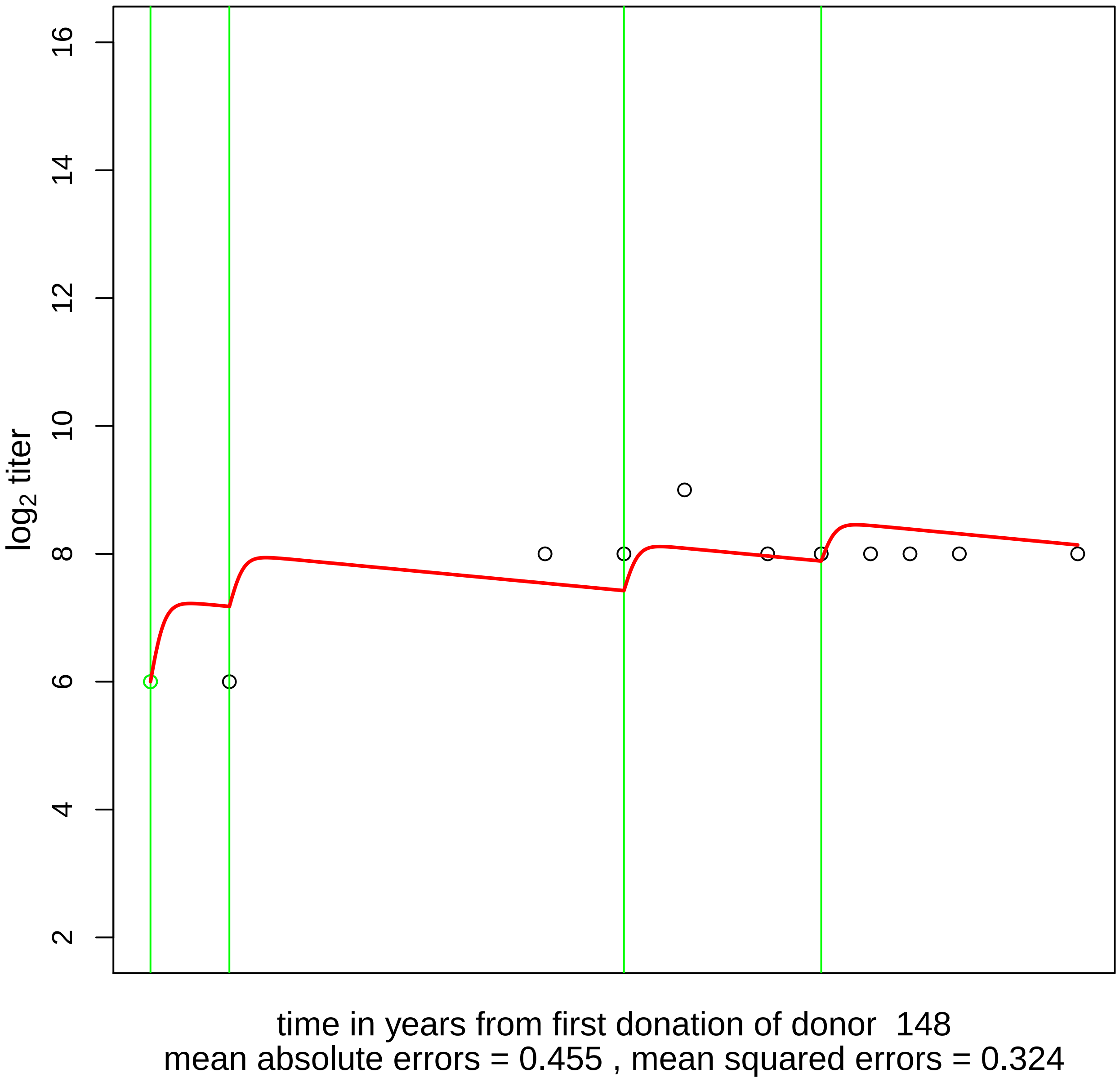

16
14
12
10
8
6
4
2
log_2 titer
time in years from first donation of donor 148
mean absolute errors = 0.455 , mean squared errors = 0.324

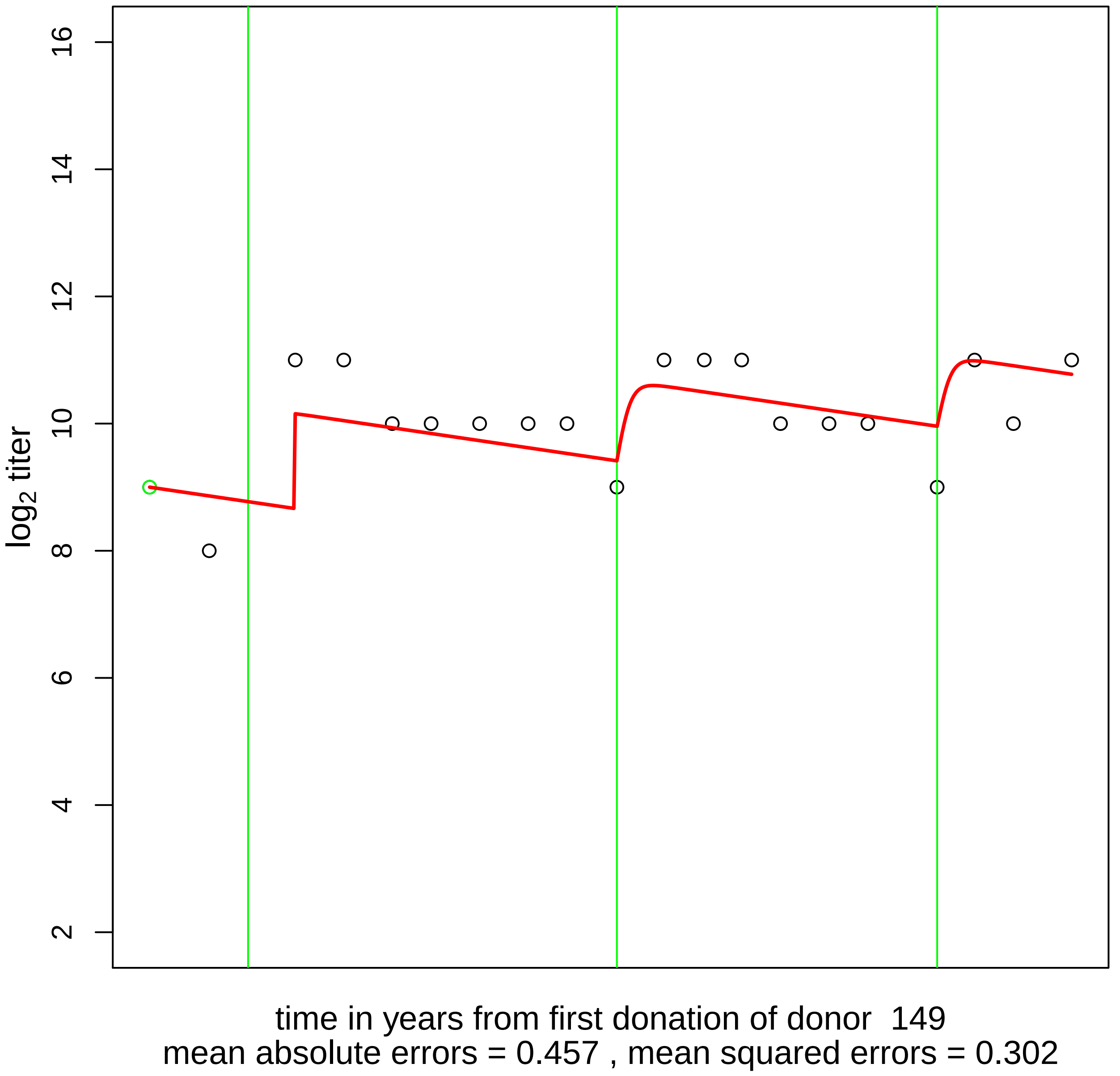

16
14
12
10
8
6
4
2
$\log_2$ titer
time in years from first donation of donor 149
mean absolute errors = 0.457 , mean squared errors = 0.302

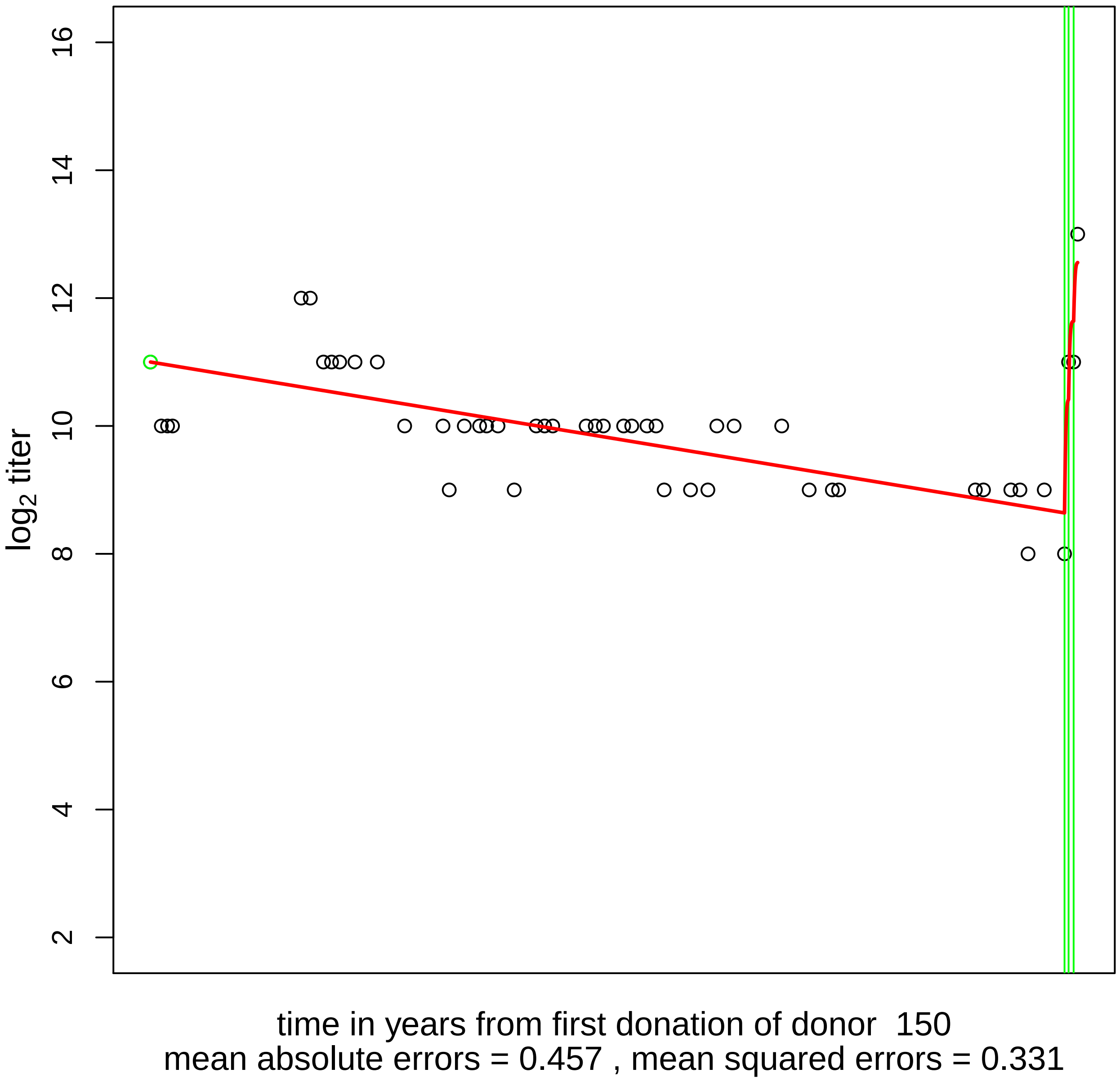

log2 titer
2
4
6
8
10
12
14
16
time in years from first donation of donor 150
mean absolute errors = 0.457 , mean squared errors = 0.331

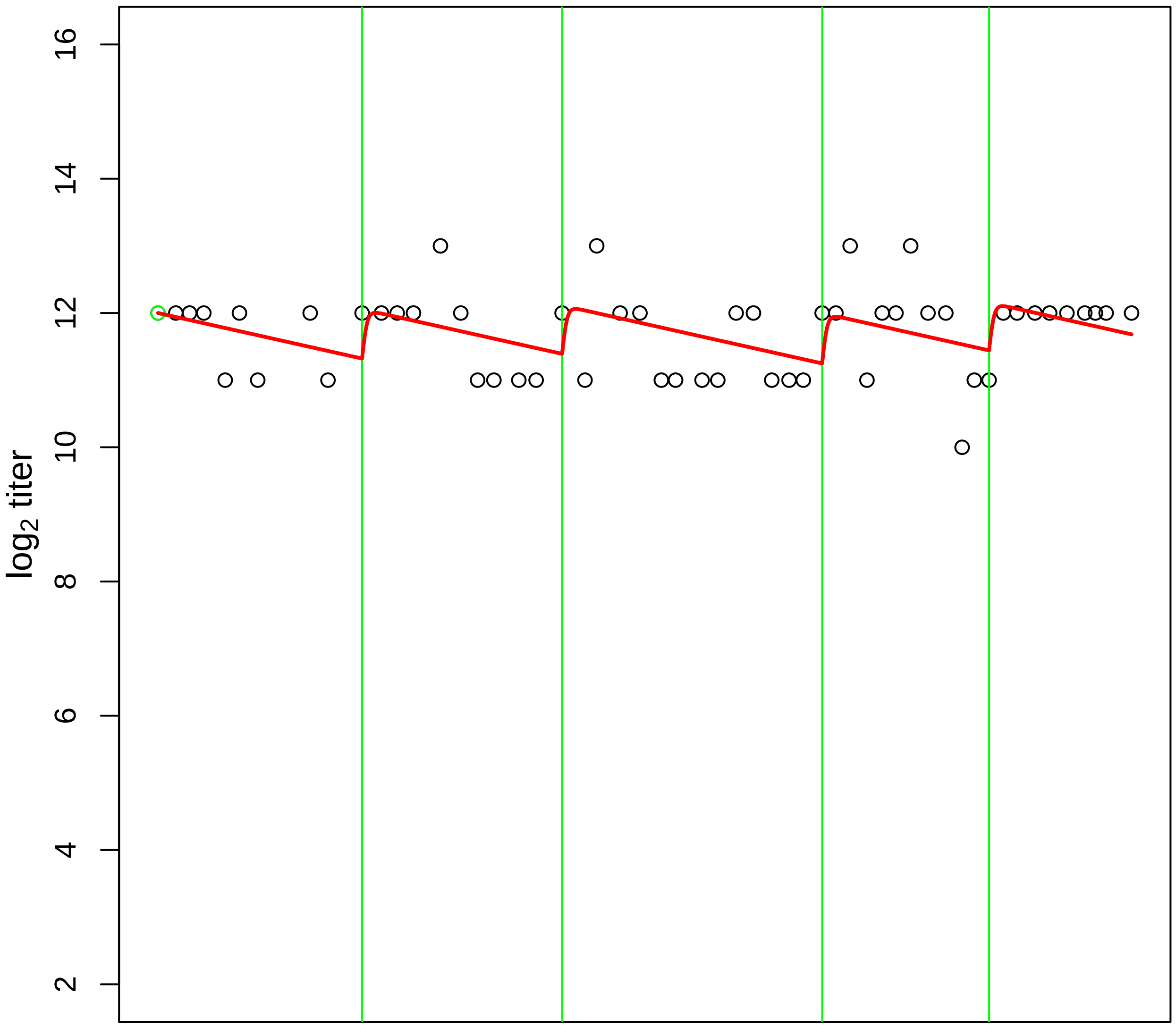

time in years from first donation of donor 151
mean absolute errors = 0.458 , mean squared errors = 0.337

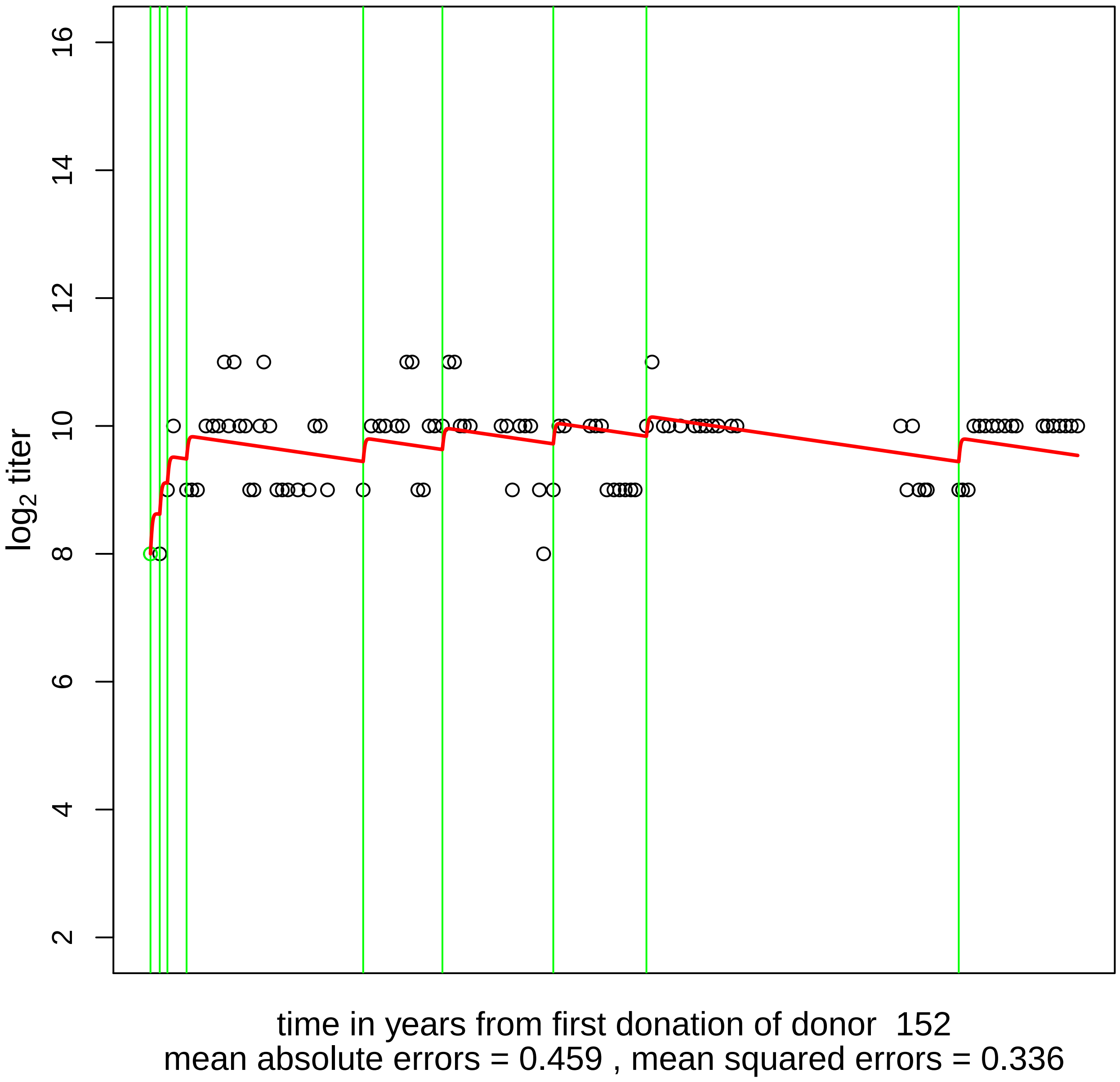
16
14
12
10
8
6
4
2
$\log_2$ titer
time in years from first donation of donor 152
mean absolute errors = 0.459 , mean squared errors = 0.336

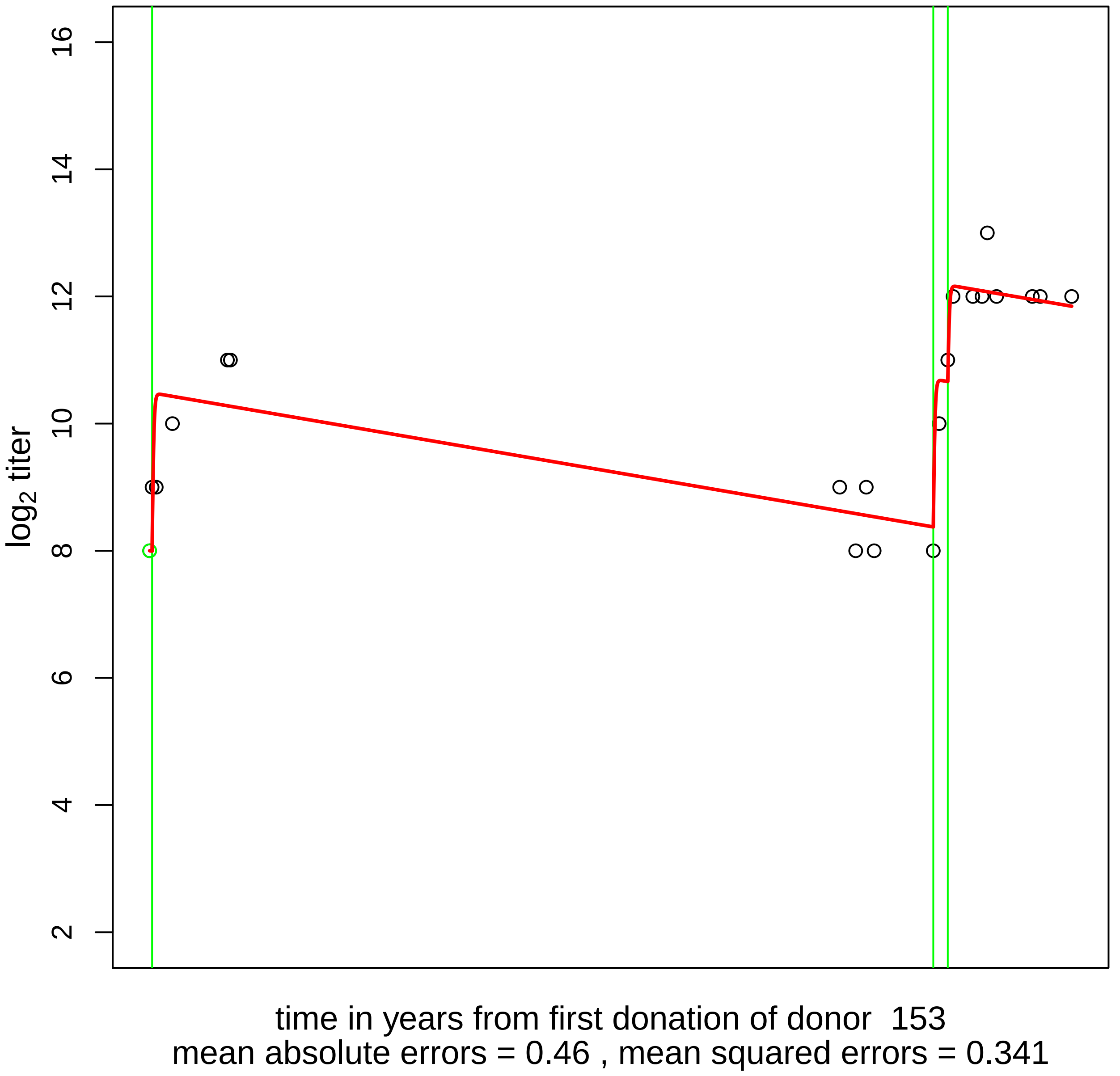

log2 titer
16
14
12
10
8
6
4
2
time in years from first donation of donor 153
mean absolute errors = 0.46 , mean squared errors = 0.341

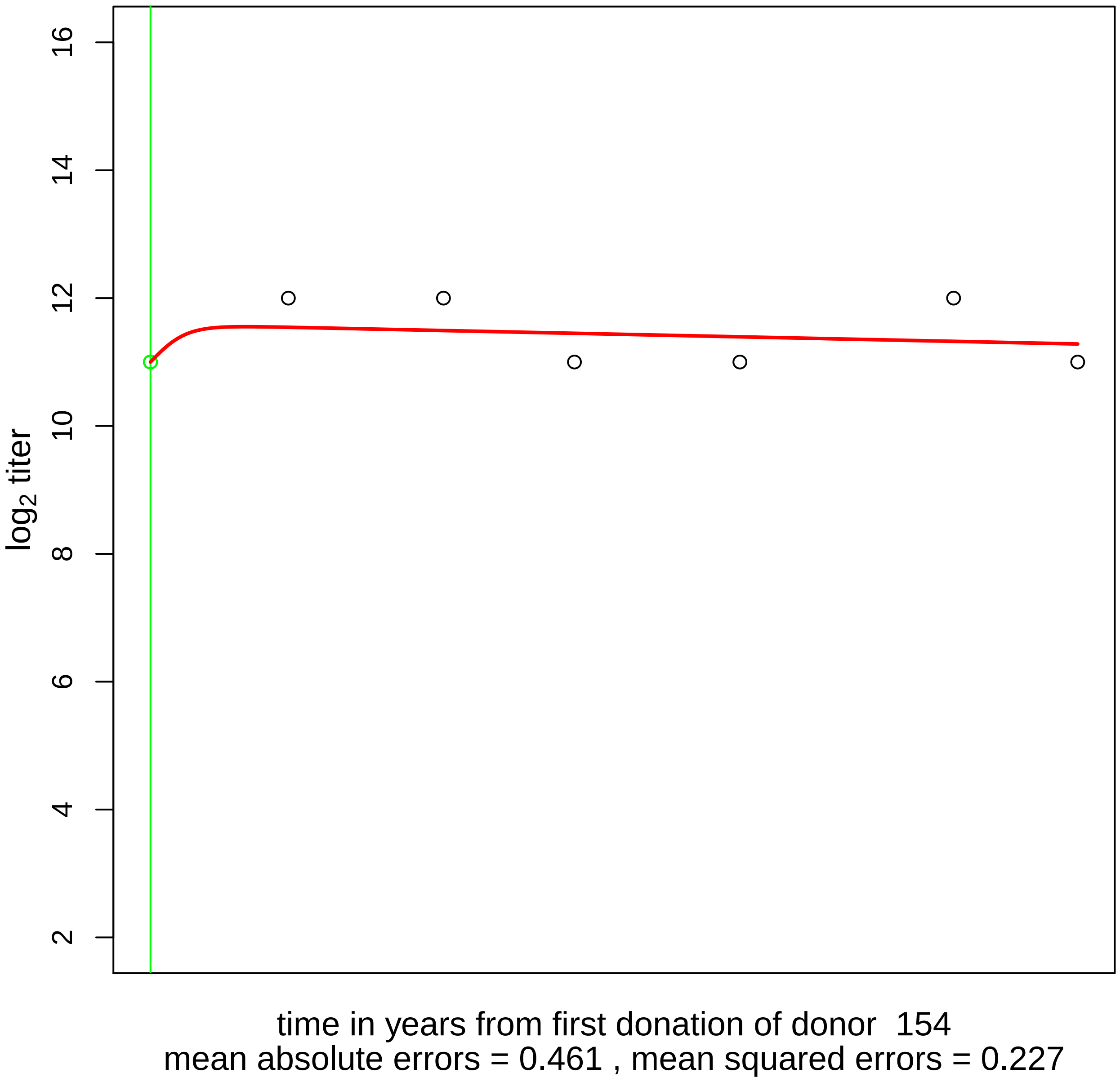

log2 titer
16
14
12
10
8
6
4
2
time in years from first donation of donor 154
mean absolute errors = 0.461 , mean squared errors = 0.227

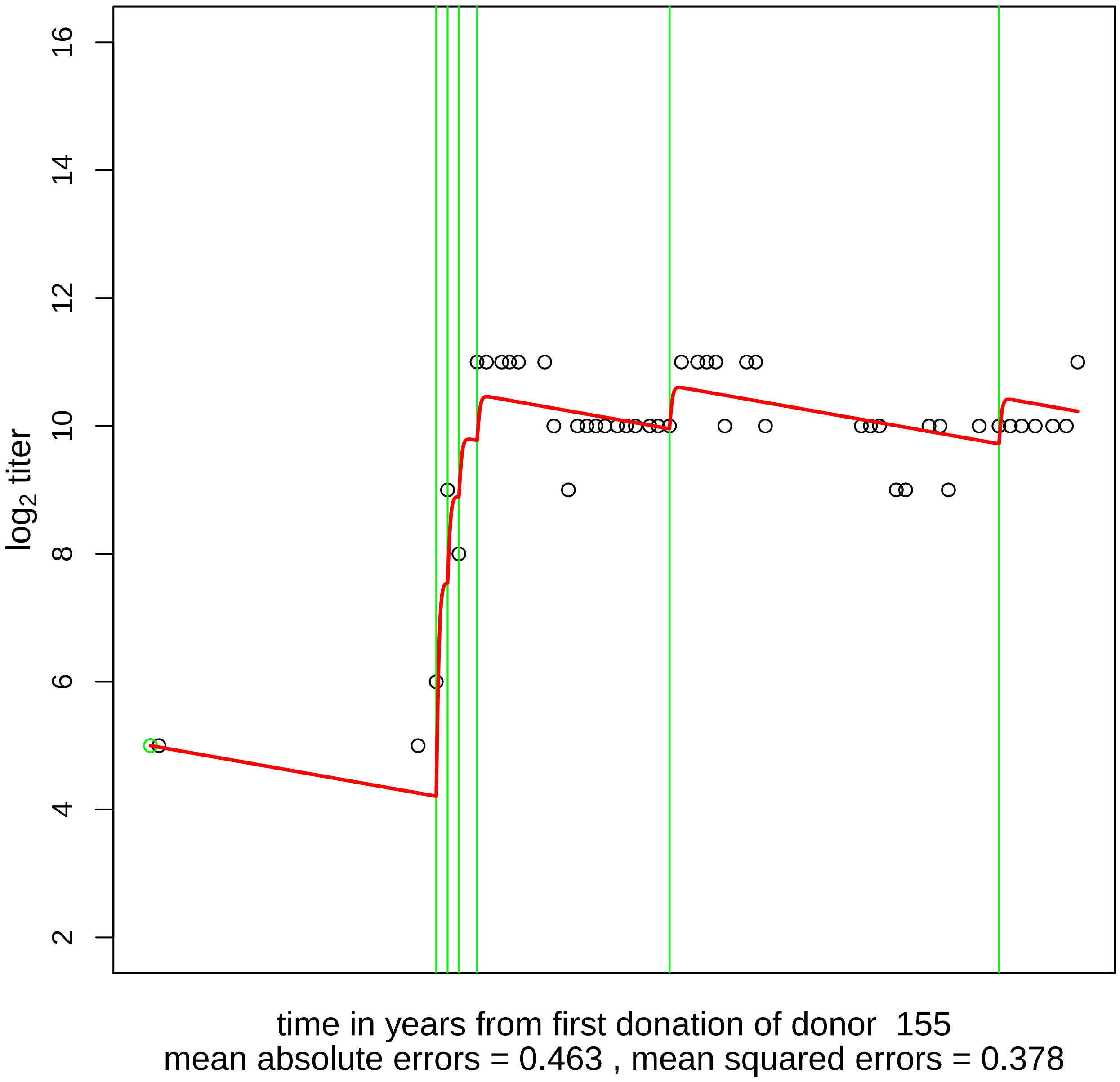

16
14
12
10
8
6
4
2
$\log_2$ titer
time in years from first donation of donor 155
mean absolute errors = 0.463 , mean squared errors = 0.378

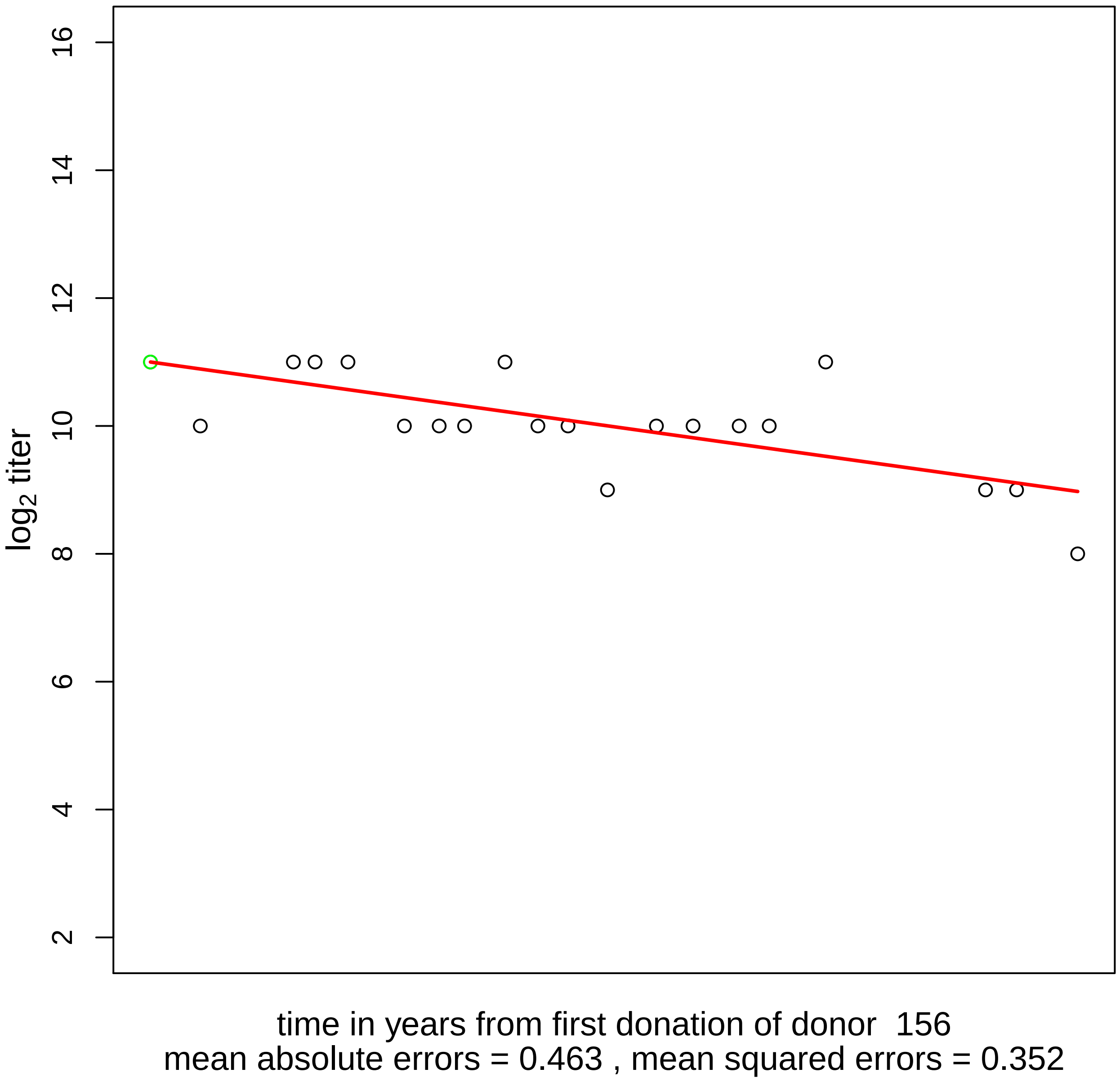

log2 titer
2
4
6
8
10
12
14
16
time in years from first donation of donor 156
mean absolute errors = 0.463 , mean squared errors = 0.352

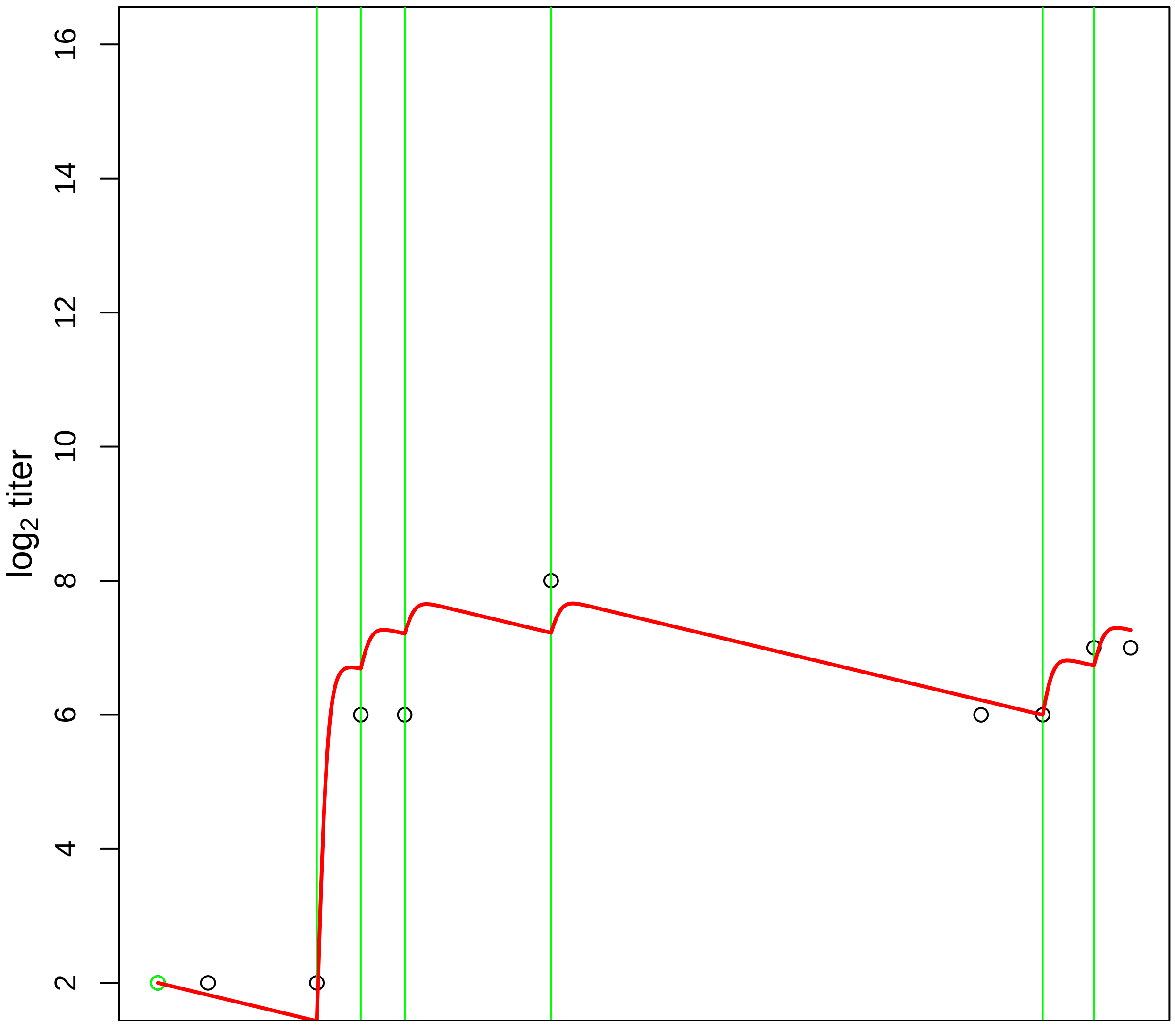

time in years from first donation of donor 157
mean absolute errors = 0.464 , mean squared errors = 0.343

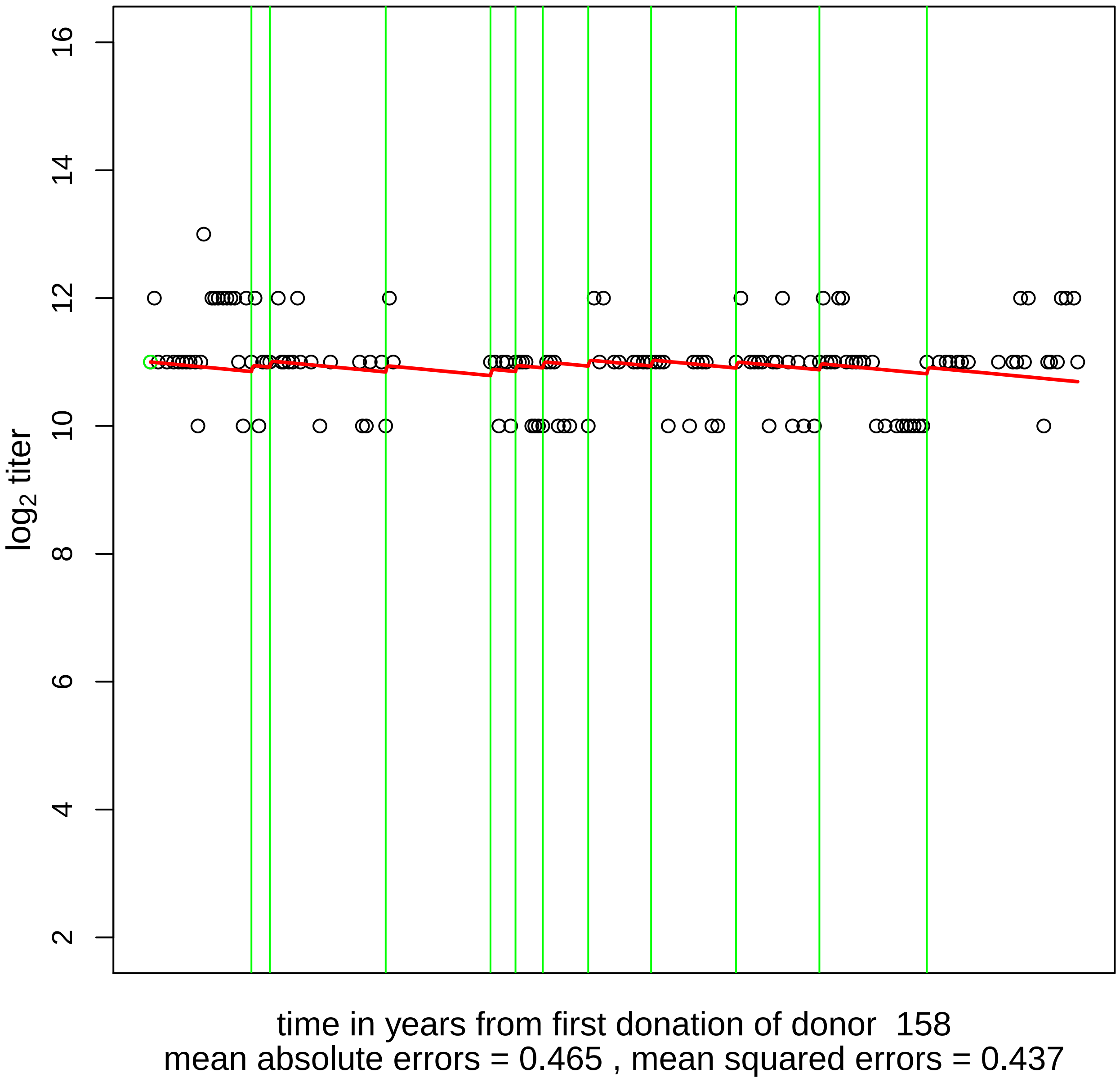

log2 titer
16
14
12
10
8
6
4
2
time in years from first donation of donor 158
mean absolute errors = 0.465 , mean squared errors = 0.437

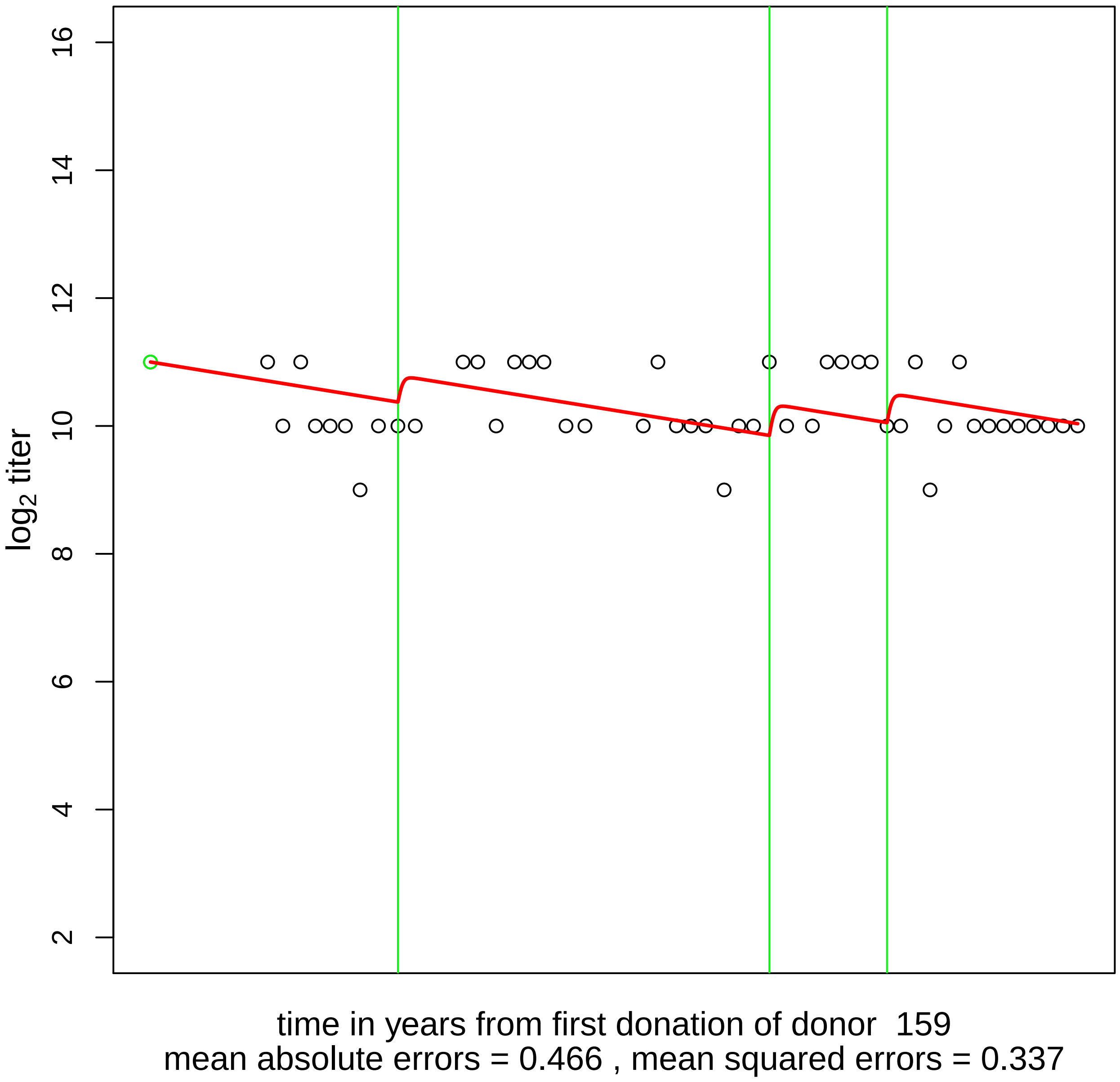

16
14
12
10
8
6
4
2
log2 titer
time in years from first donation of donor 159
mean absolute errors = 0.466 , mean squared errors = 0.337

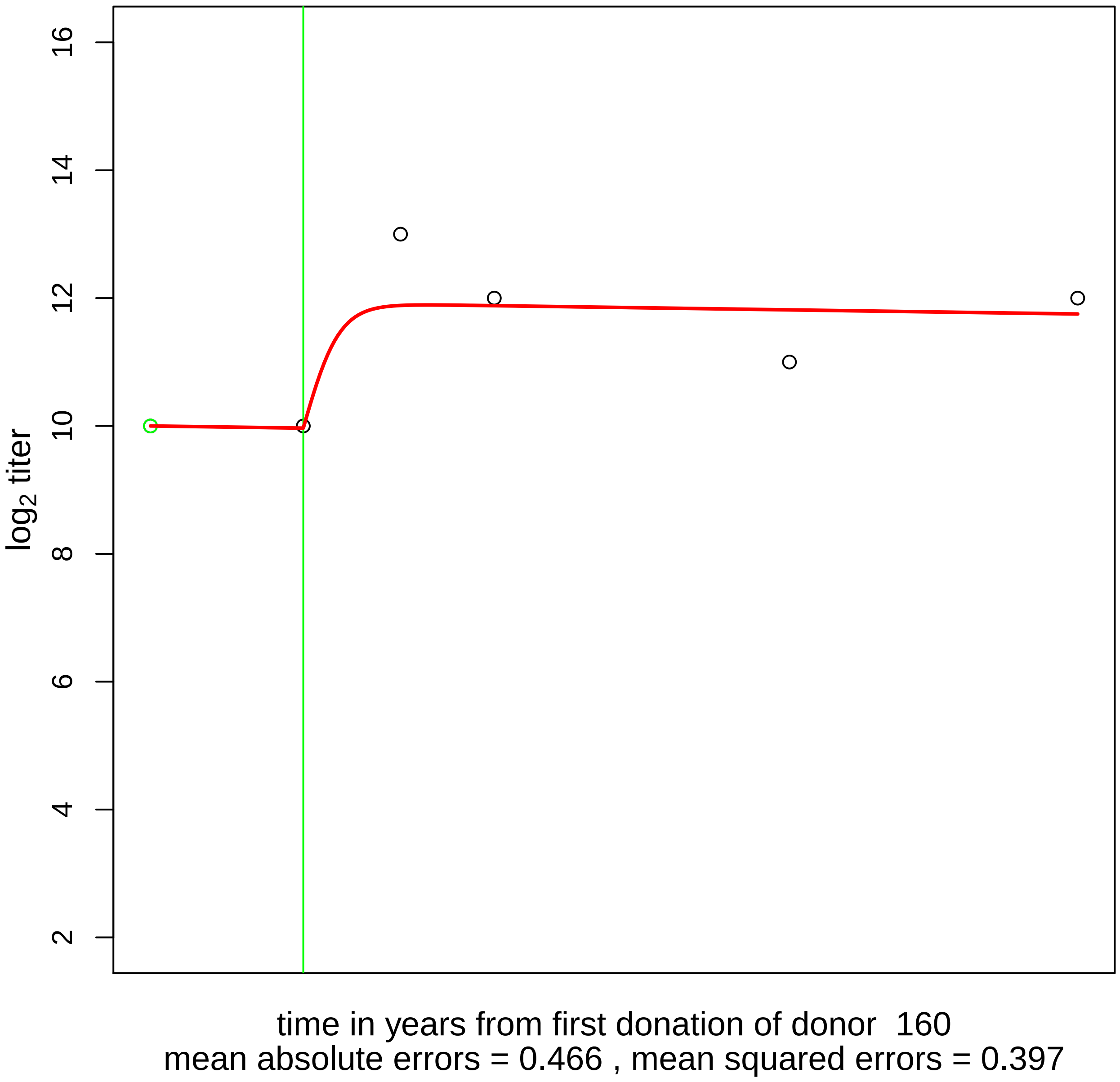

16
14
12
10
8
6
4
2
$\log_2$ titer
time in years from first donation of donor 160
mean absolute errors = 0.466 , mean squared errors = 0.397

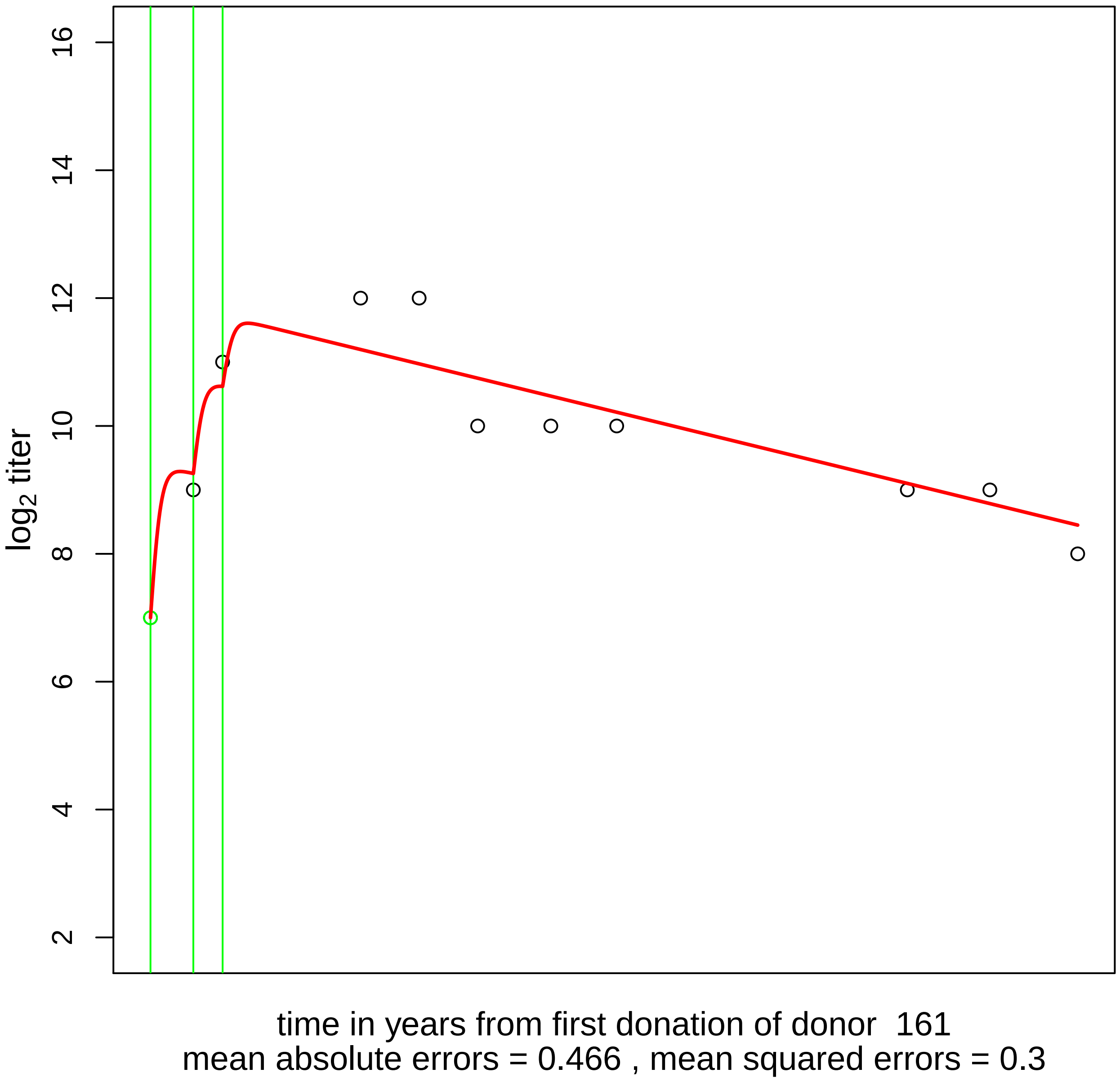

16
14
12
10
8
6
4
2
log2 titer
time in years from first donation of donor 161
mean absolute errors = 0.466 , mean squared errors = 0.3

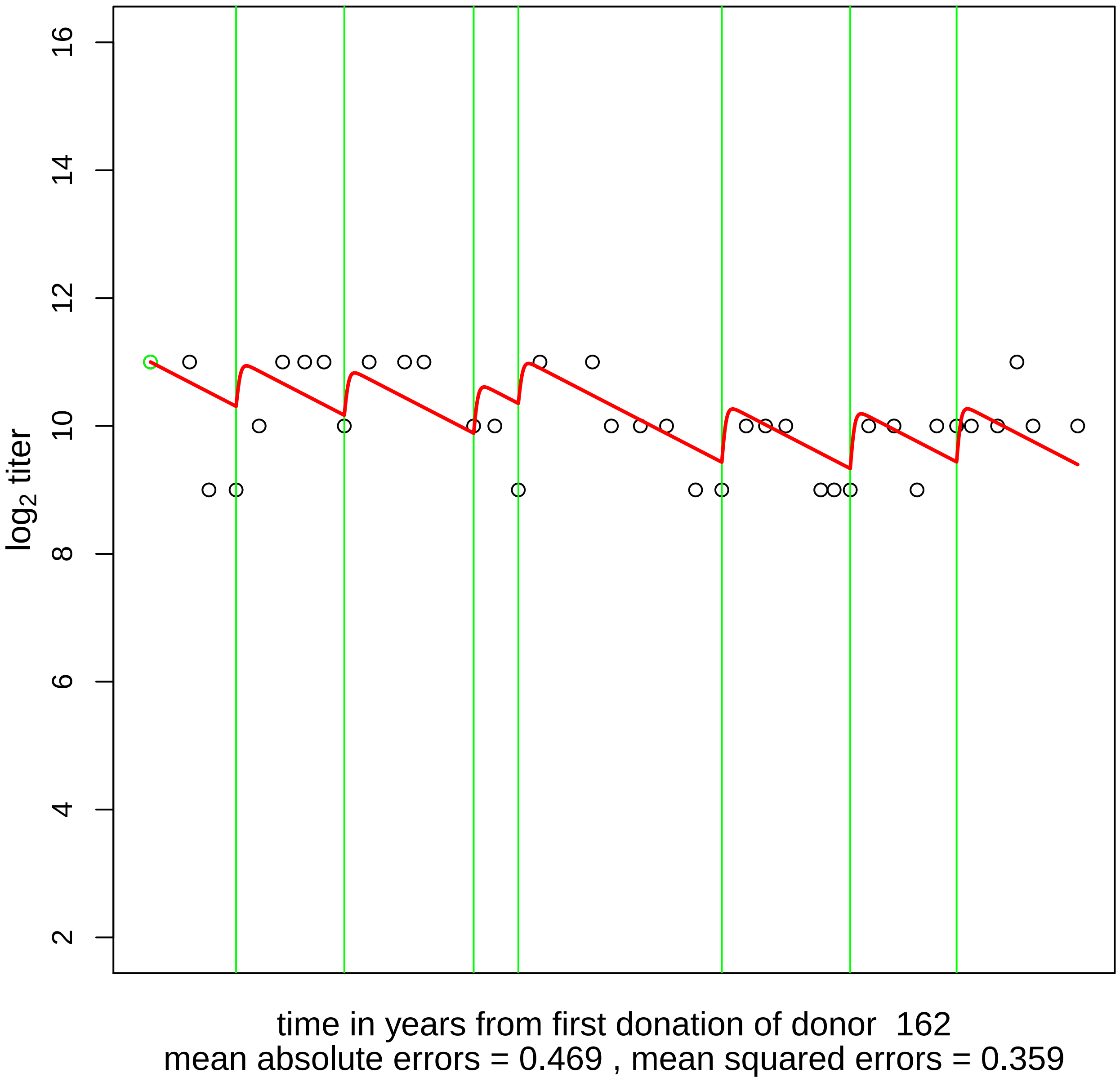

log₂ titer
2
4
6
8
10
12
14
16
time in years from first donation of donor 162
mean absolute errors = 0.469 , mean squared errors = 0.359

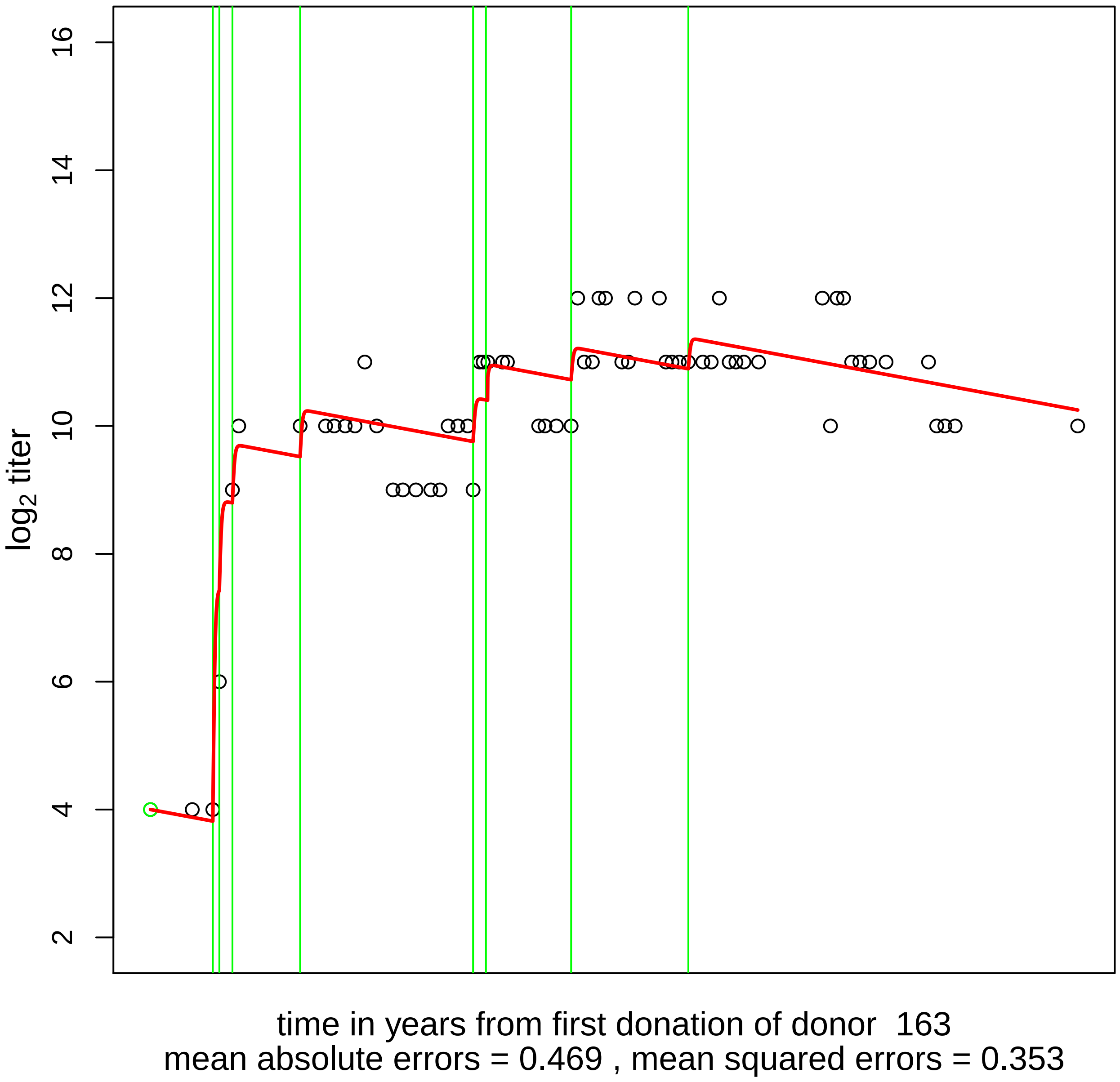

16
14
12
10
8
6
4
2
$\log_2$ titer
time in years from first donation of donor 163
mean absolute errors = 0.469 , mean squared errors = 0.353

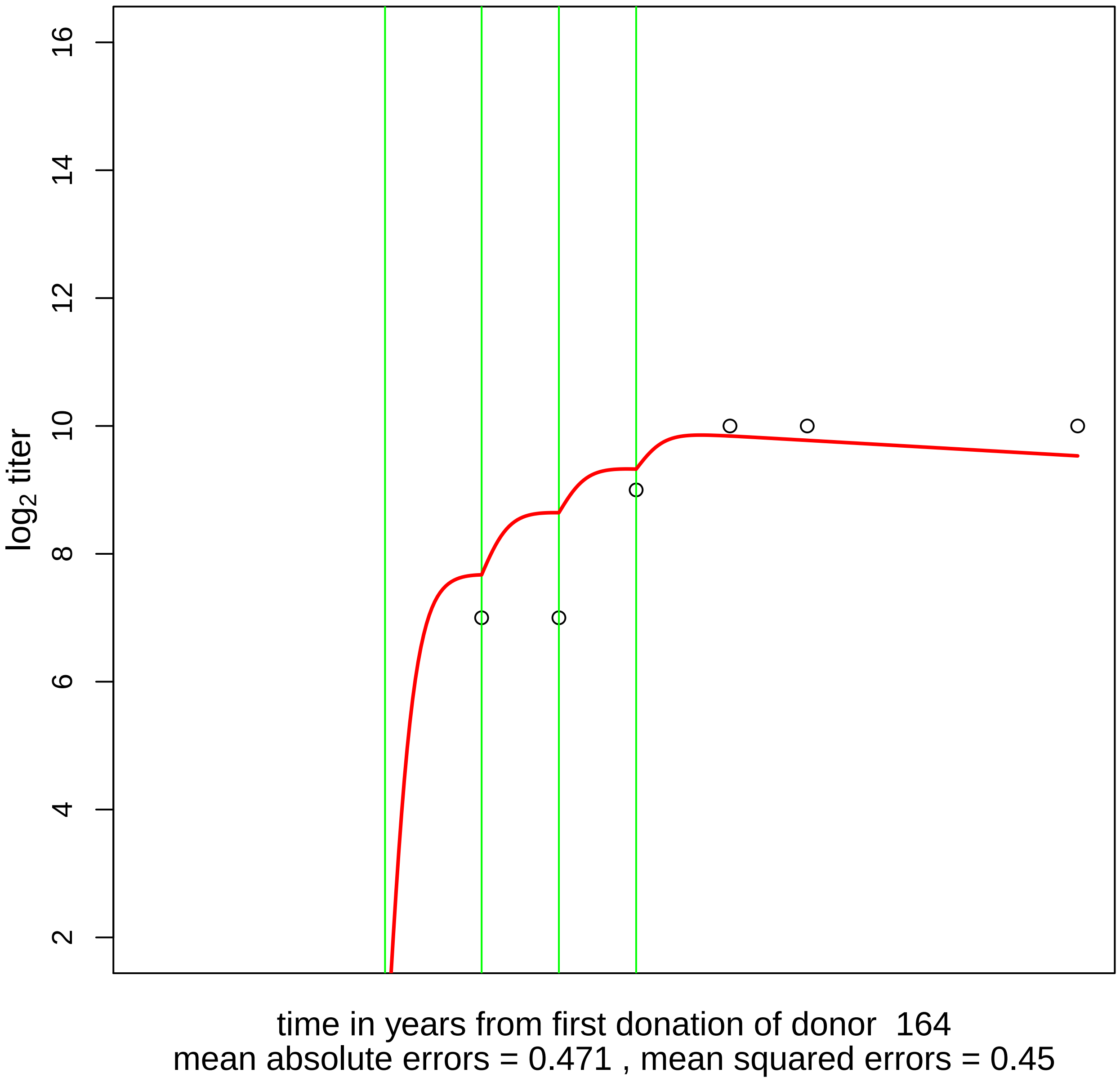

16
14
12
10
8
6
4
2
log2 titer
time in years from first donation of donor 164
mean absolute errors = 0.471 , mean squared errors = 0.45

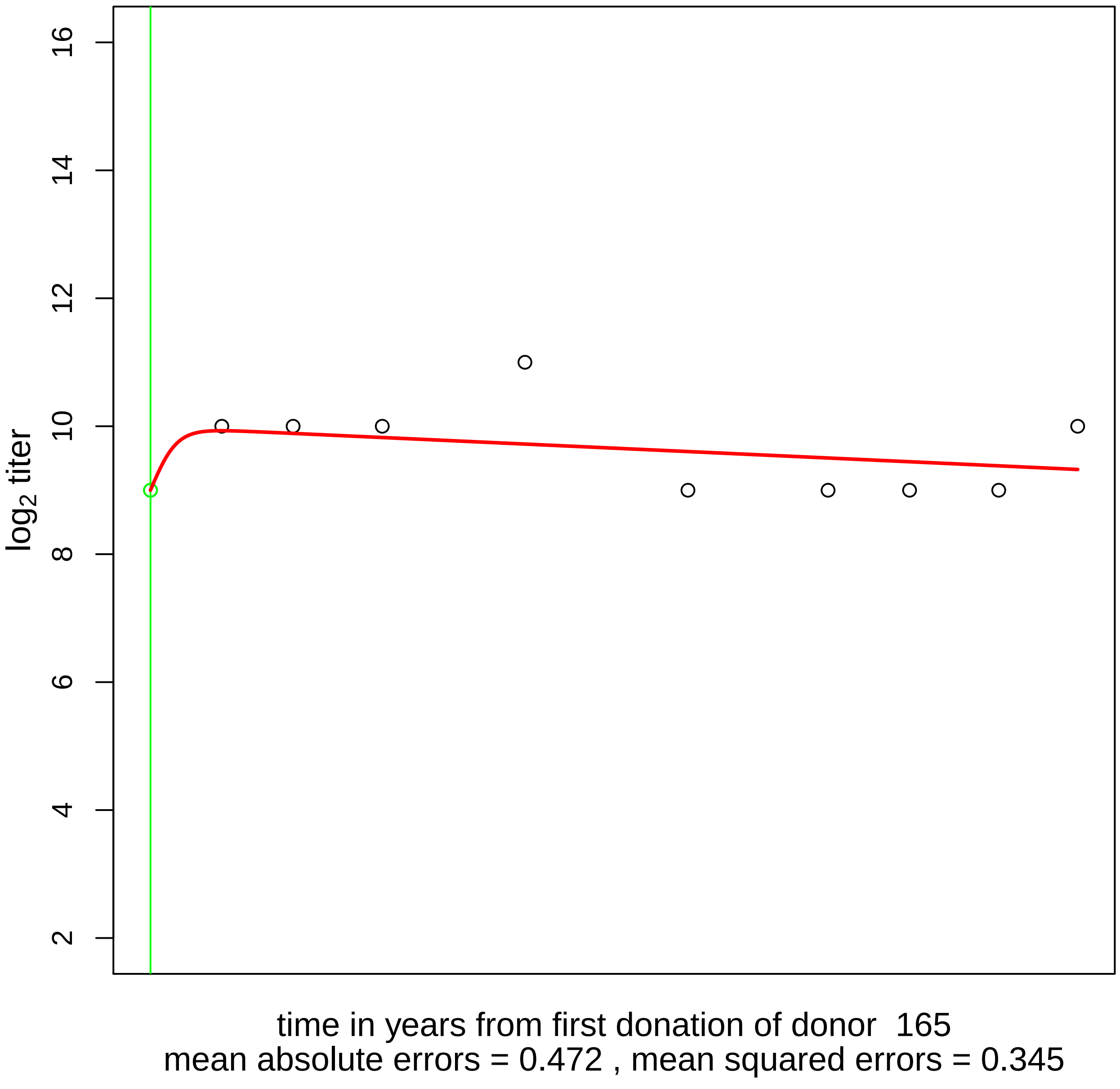

16
14
12
10
8
6
4
2
log2 titer
time in years from first donation of donor 165
mean absolute errors = 0.472 , mean squared errors = 0.345

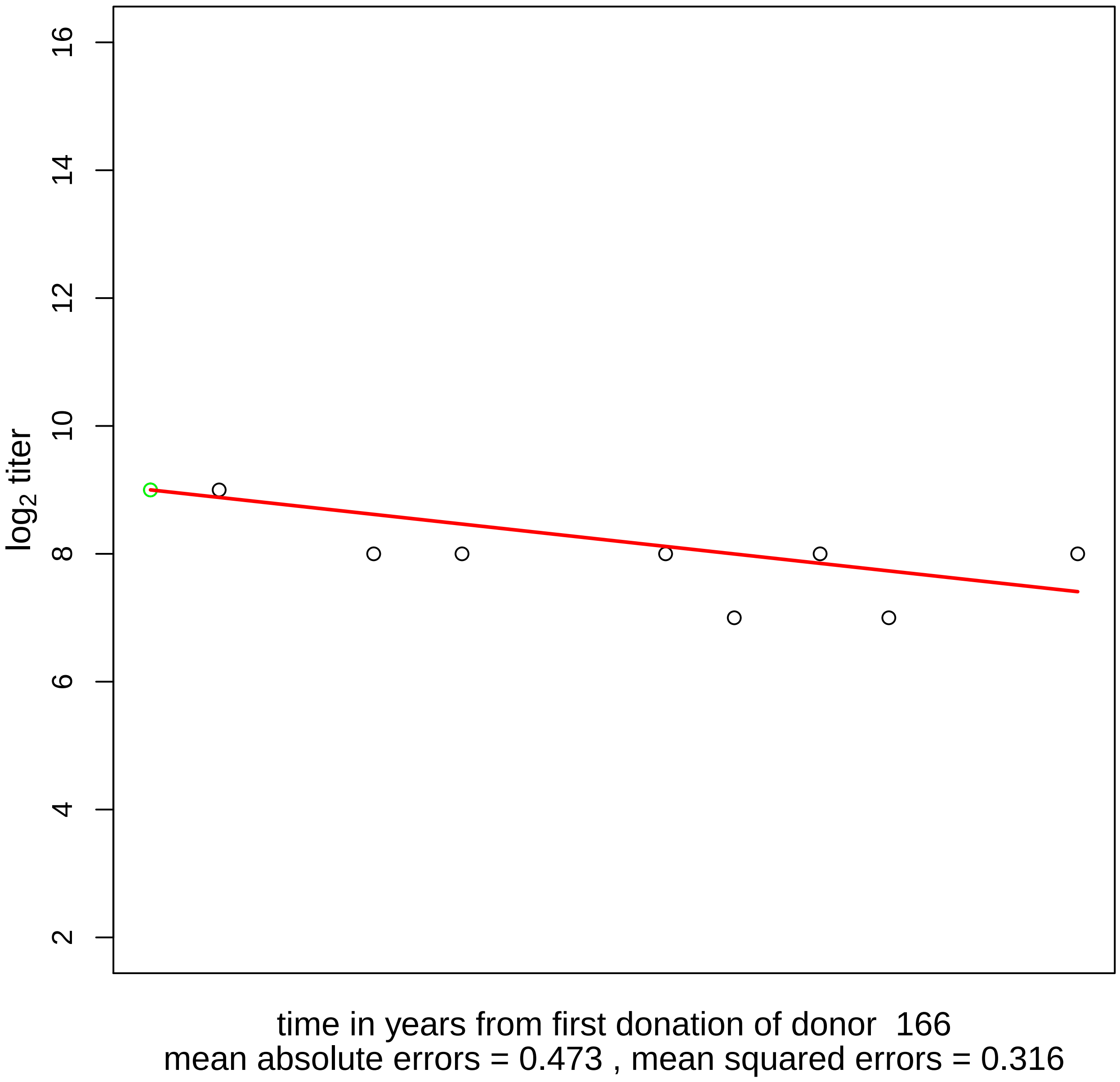

16
14
12
10
8
6
4
2
$\log_2$ titer
time in years from first donation of donor 166
mean absolute errors = 0.473 , mean squared errors = 0.316

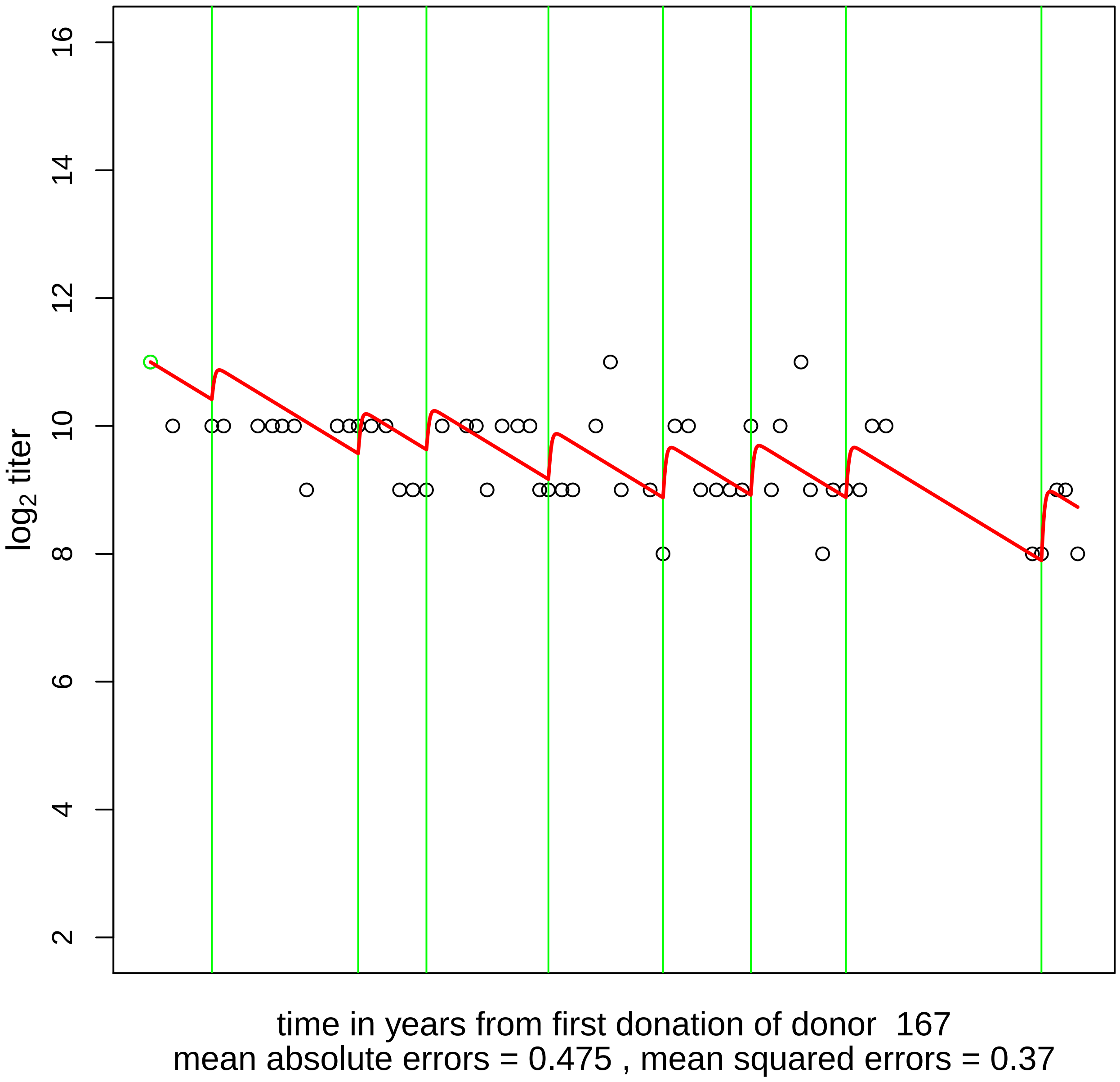
16
14
12
10
8
6
4
2
$\log_2$ titer
time in years from first donation of donor 167
mean absolute errors = 0.475 , mean squared errors = 0.37

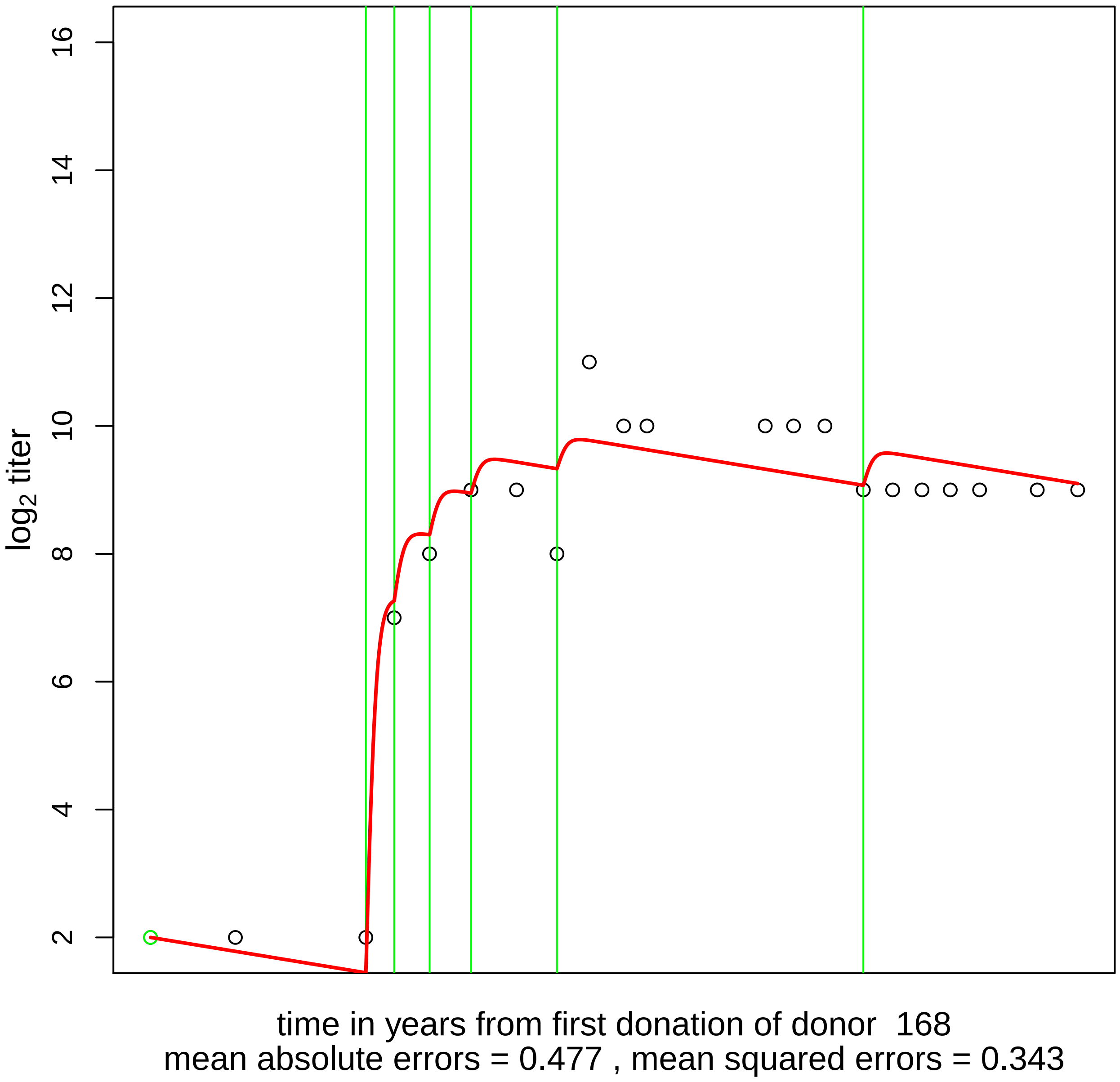

log2 titer
2
4
6
8
10
12
14
16
time in years from first donation of donor 168
mean absolute errors = 0.477 , mean squared errors = 0.343

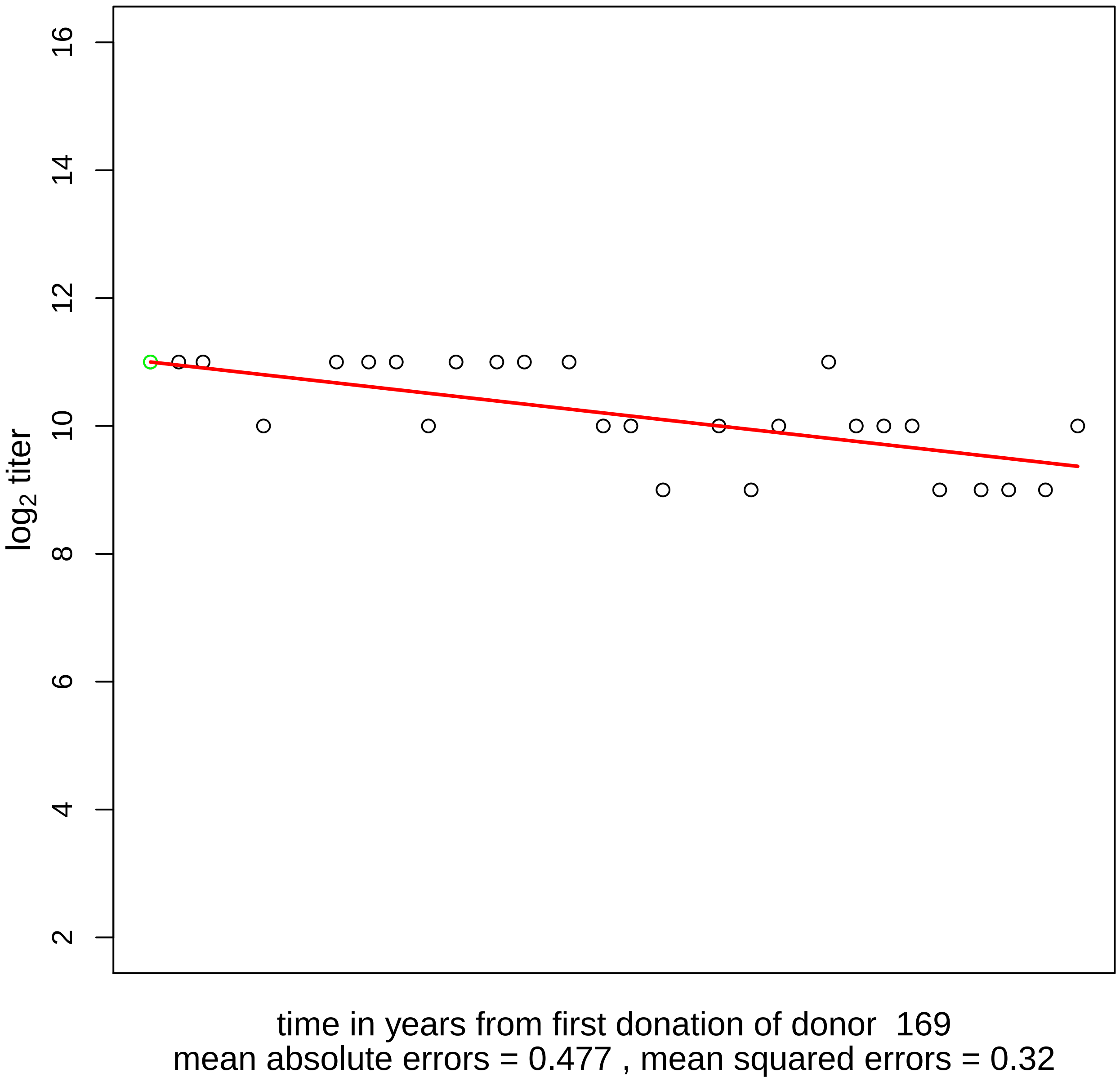

16
14
12
10
8
6
4
2
$\log_2$ titer
time in years from first donation of donor 169
mean absolute errors = 0.477 , mean squared errors = 0.32

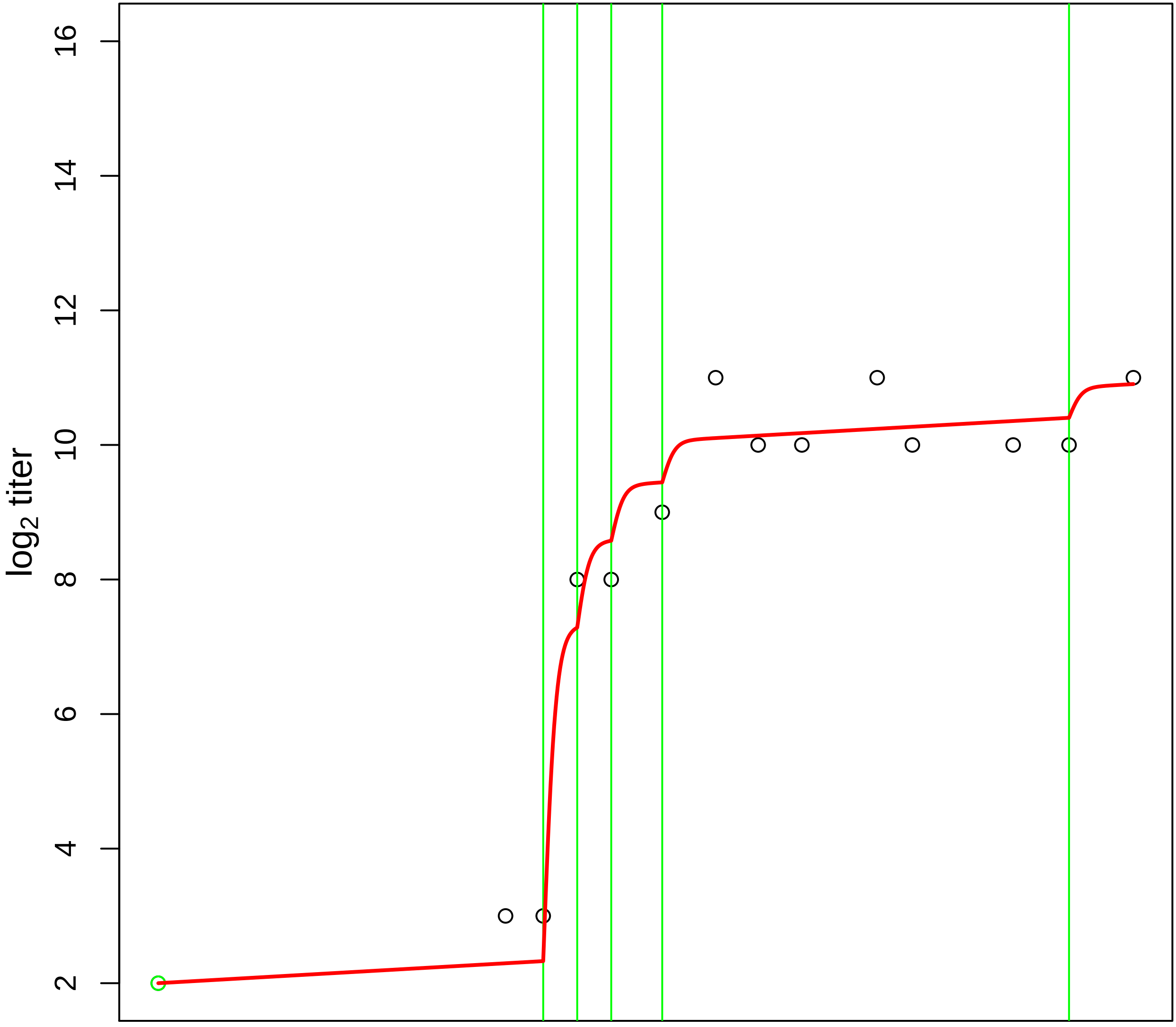

time in years from first donation of donor 170
mean absolute errors = 0.478 , mean squared errors = 0.292

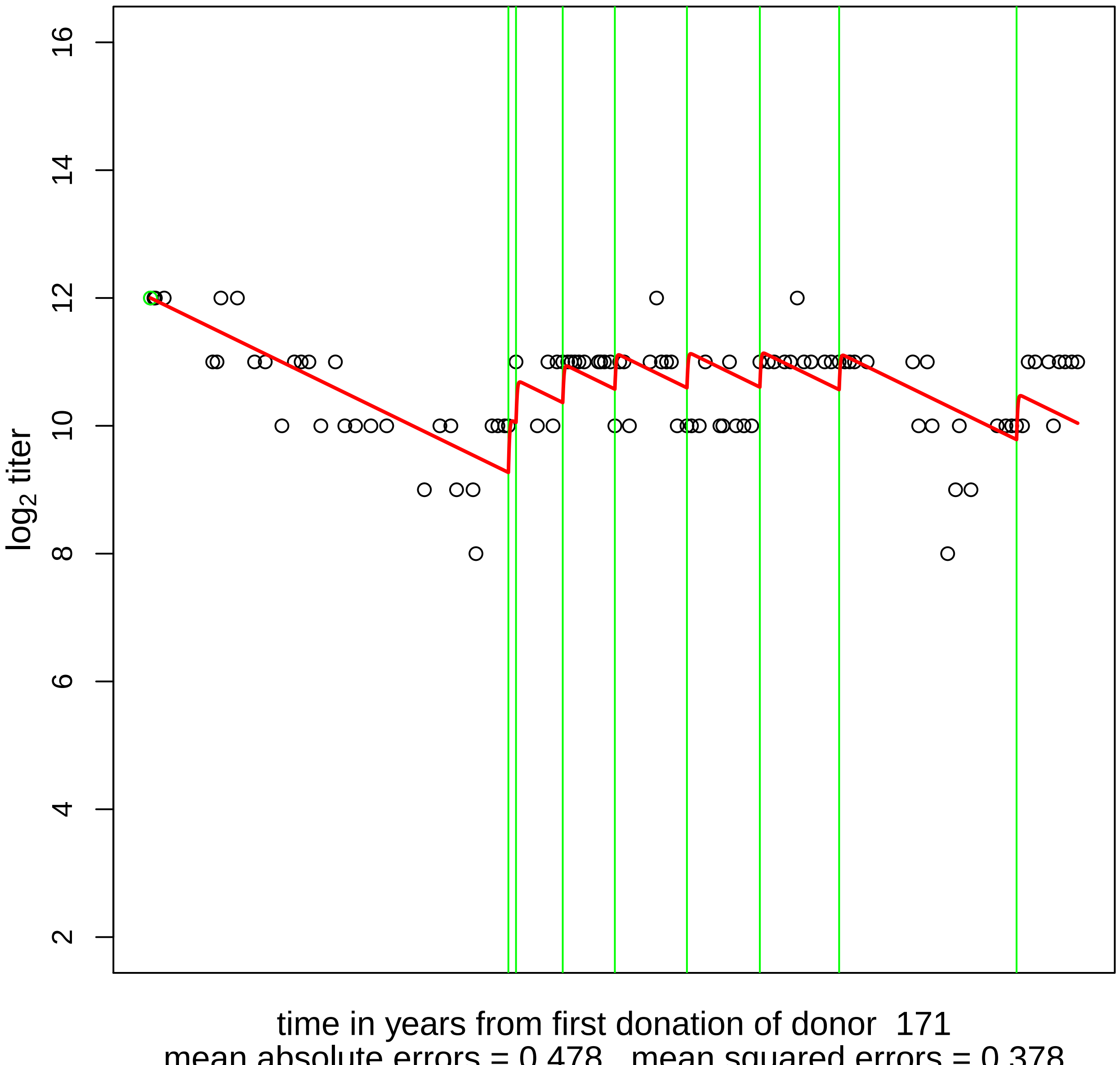

2
4
6
8
10
12
14
16
log2 titer
time in years from first donation of donor 171
mean absolute errors = 0.478 , mean squared errors = 0.378

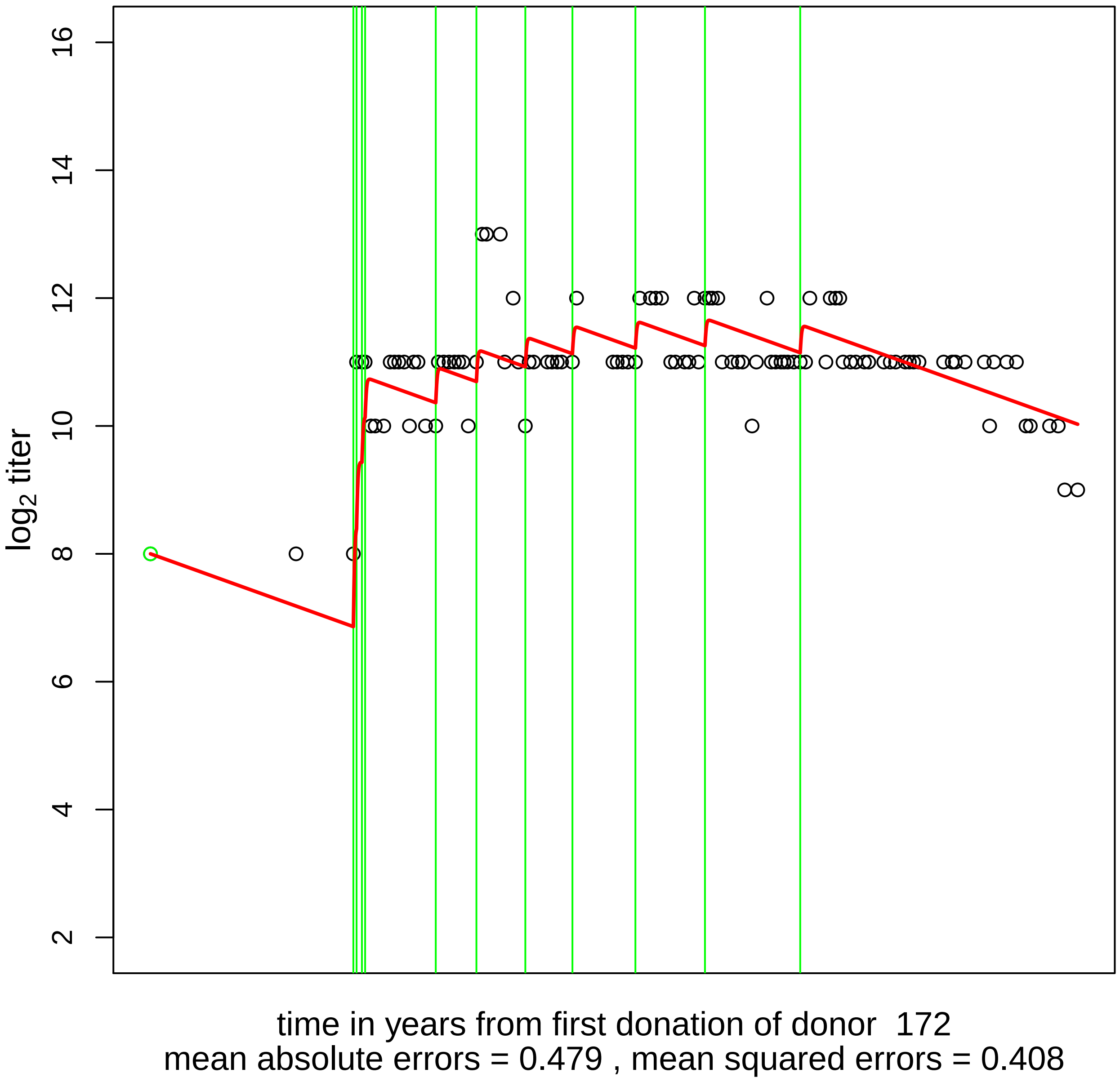

16
14
12
10
8
6
4
2
$\log_2$ titer
time in years from first donation of donor 172
mean absolute errors = 0.479 , mean squared errors = 0.408

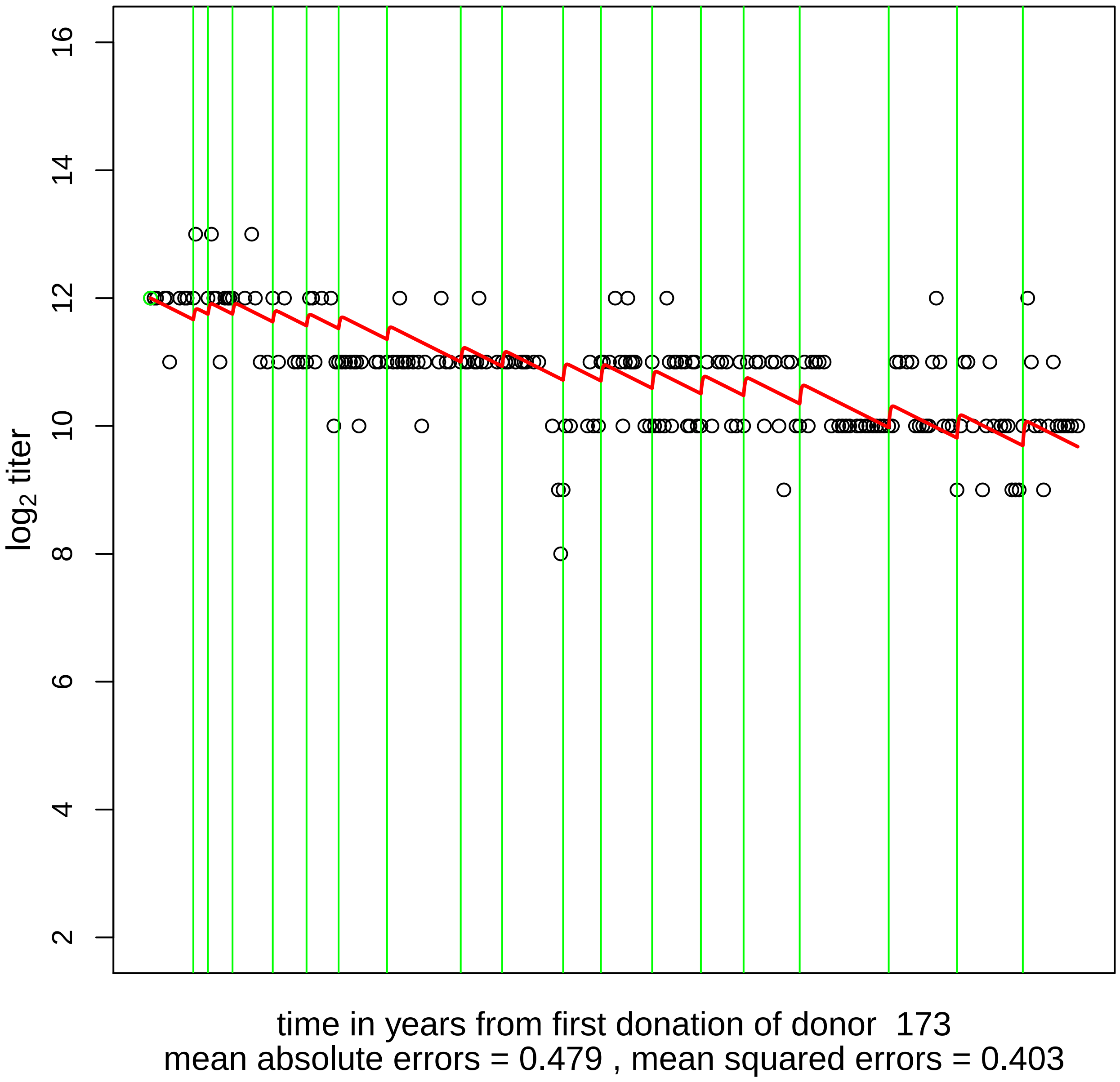
16
14
12
10
8
6
4
2
log2 titer
time in years from first donation of donor 173
mean absolute errors = 0.479 , mean squared errors = 0.403

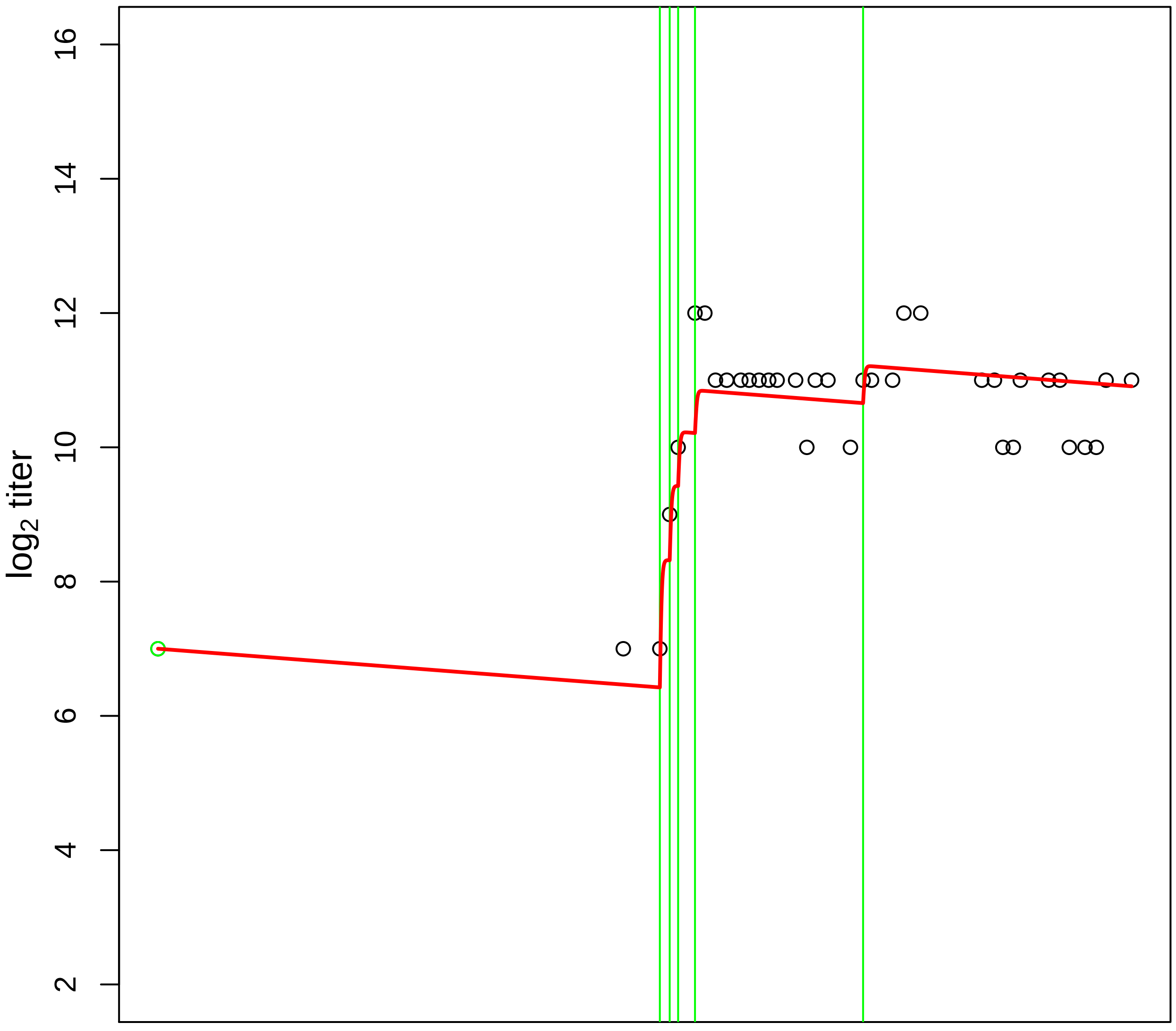

time in years from first donation of donor 174
mean absolute errors = 0.48 , mean squared errors = 0.403

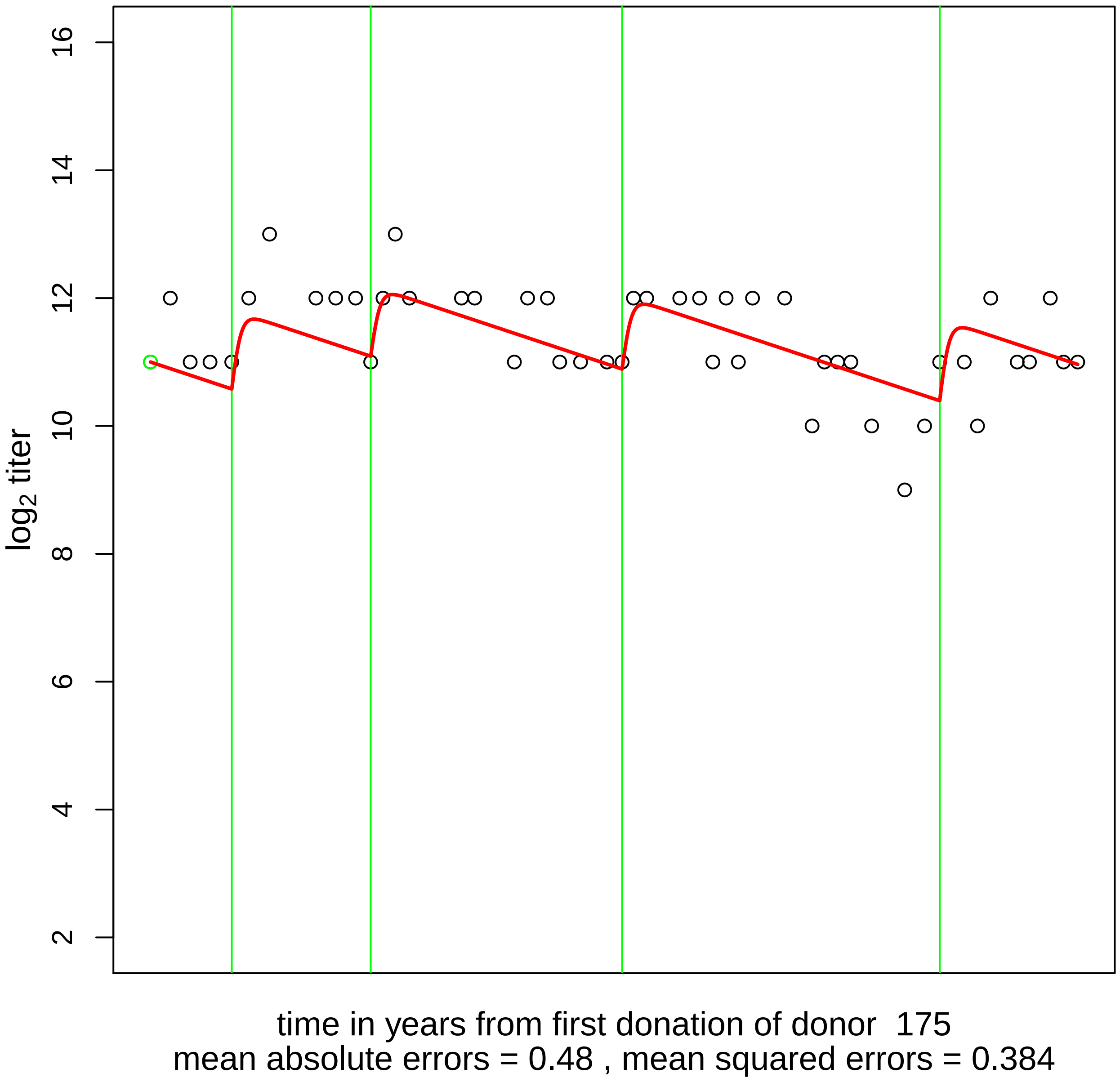

log2 titer
16
14
12
10
8
6
4
2
time in years from first donation of donor 175
mean absolute errors = 0.48 , mean squared errors = 0.384

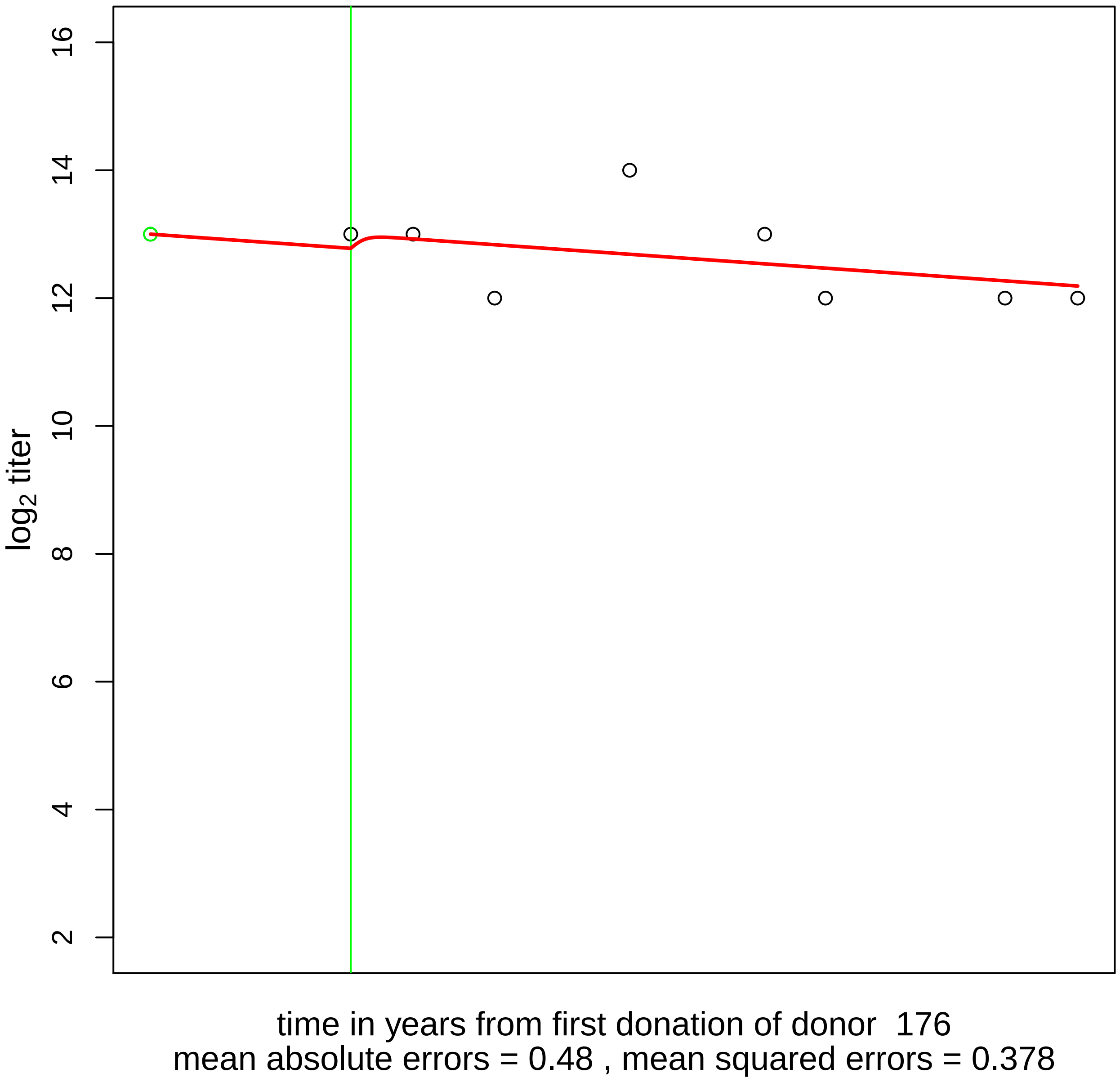

16
14
12
10
8
6
4
2
log2 titer
time in years from first donation of donor 176
mean absolute errors = 0.48 , mean squared errors = 0.378

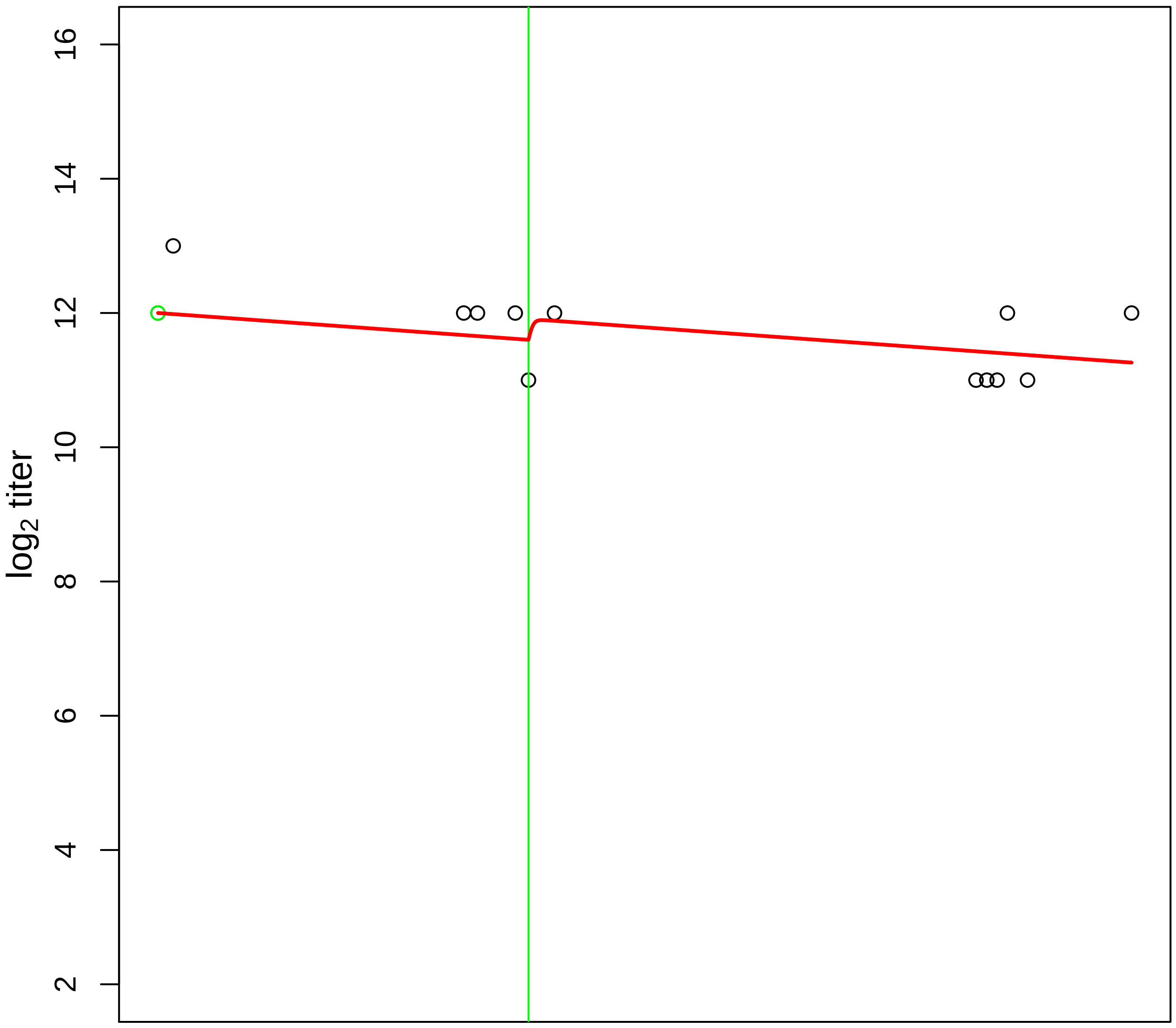

time in years from first donation of donor 177
mean absolute errors = 0.48 , mean squared errors = 0.28

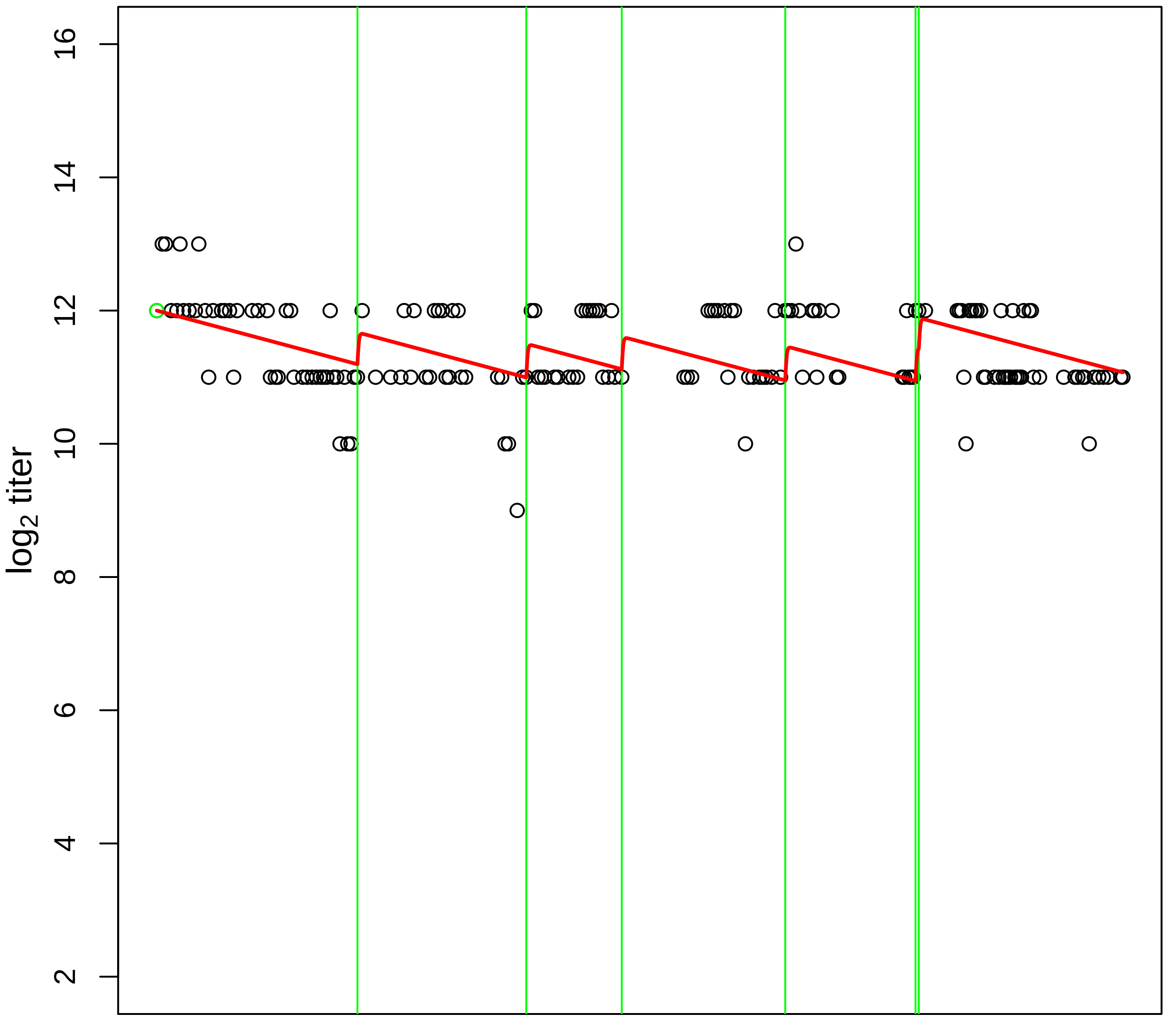

time in years from first donation of donor 178
mean absolute errors = 0.481 , mean squared errors = 0.348

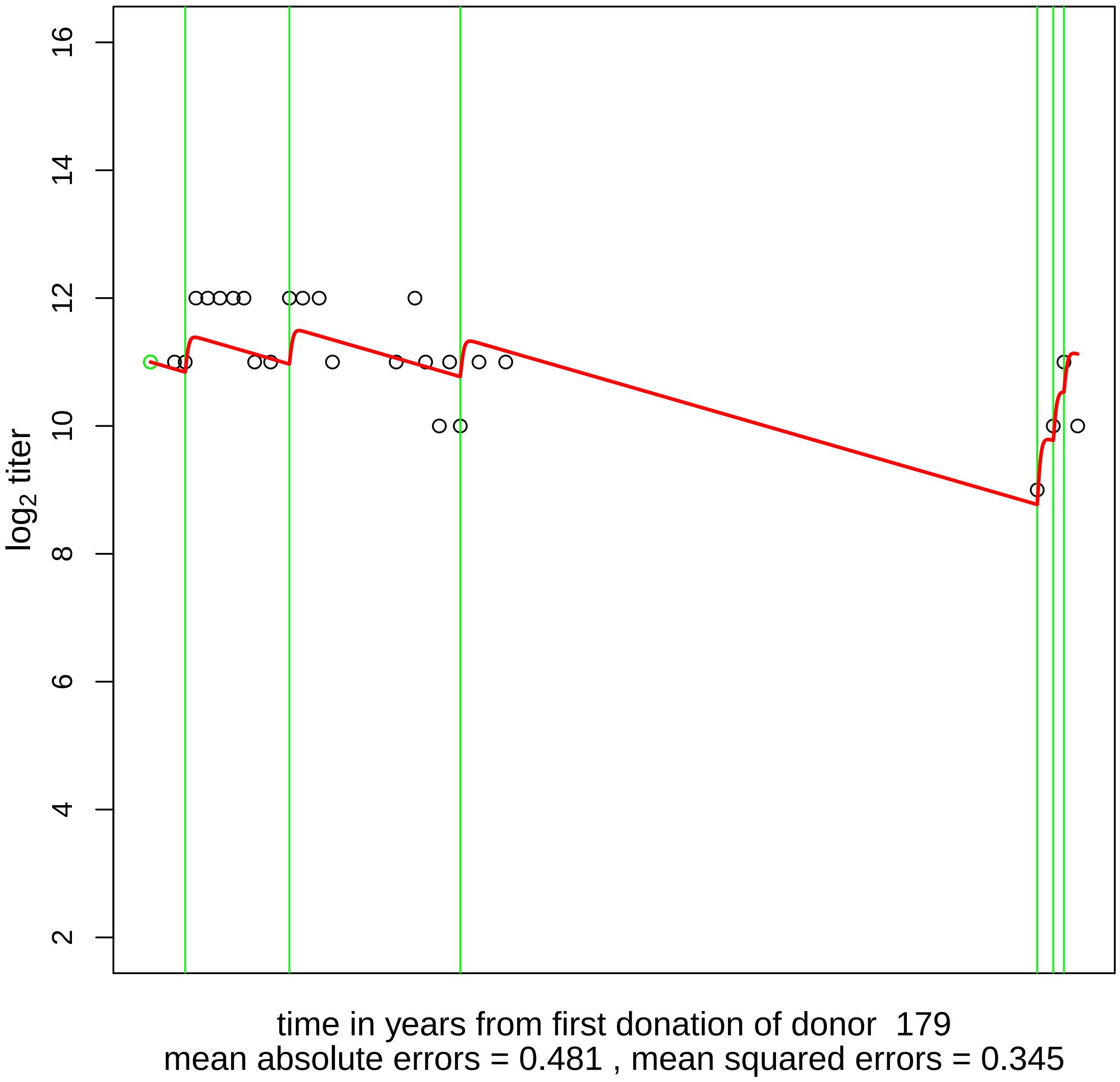

log2 titer
16
14
12
10
8
6
4
2
time in years from first donation of donor 179
mean absolute errors = 0.481 , mean squared errors = 0.345

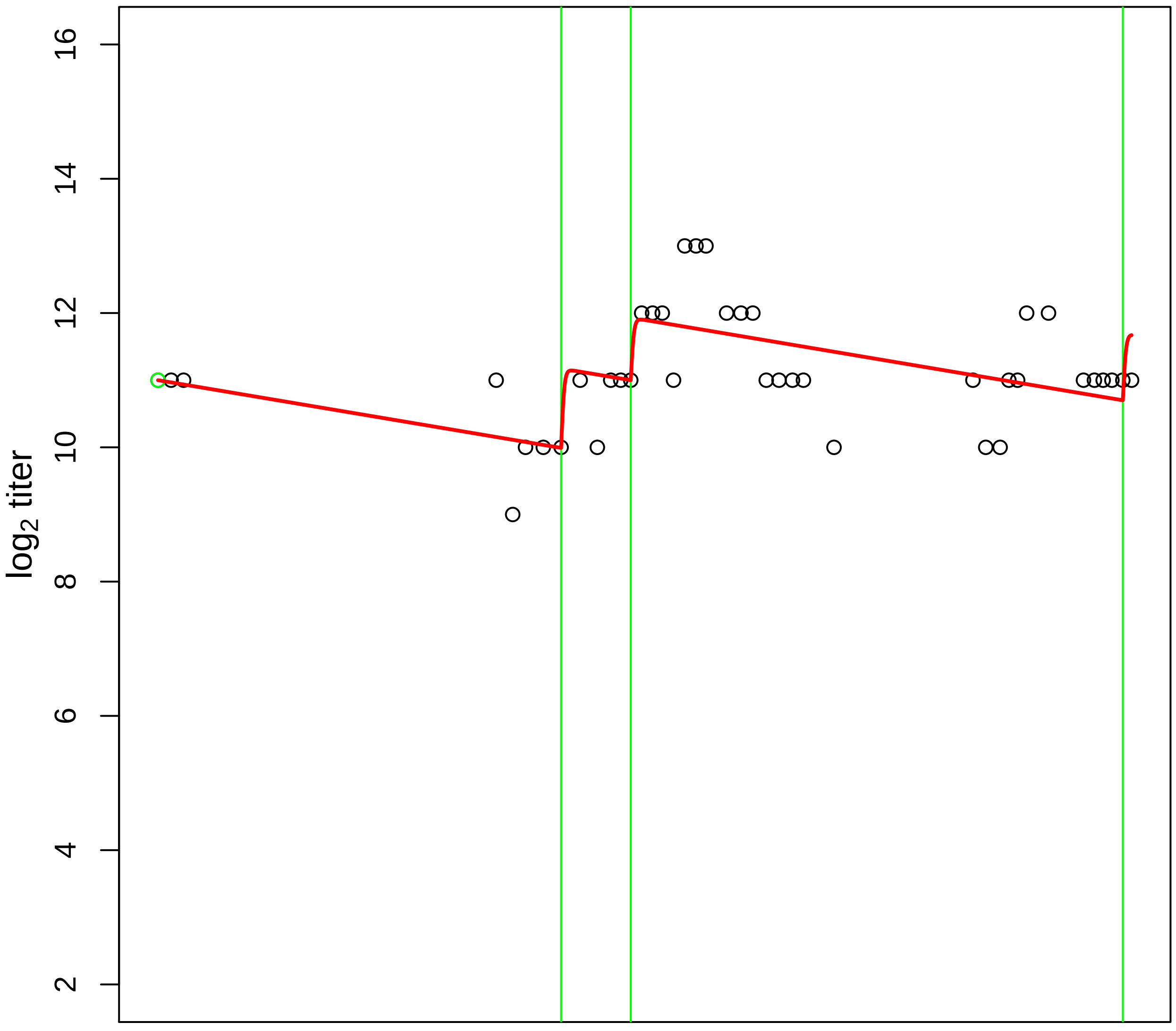

time in years from first donation of donor 180
mean absolute errors = 0.481 , mean squared errors = 0.431

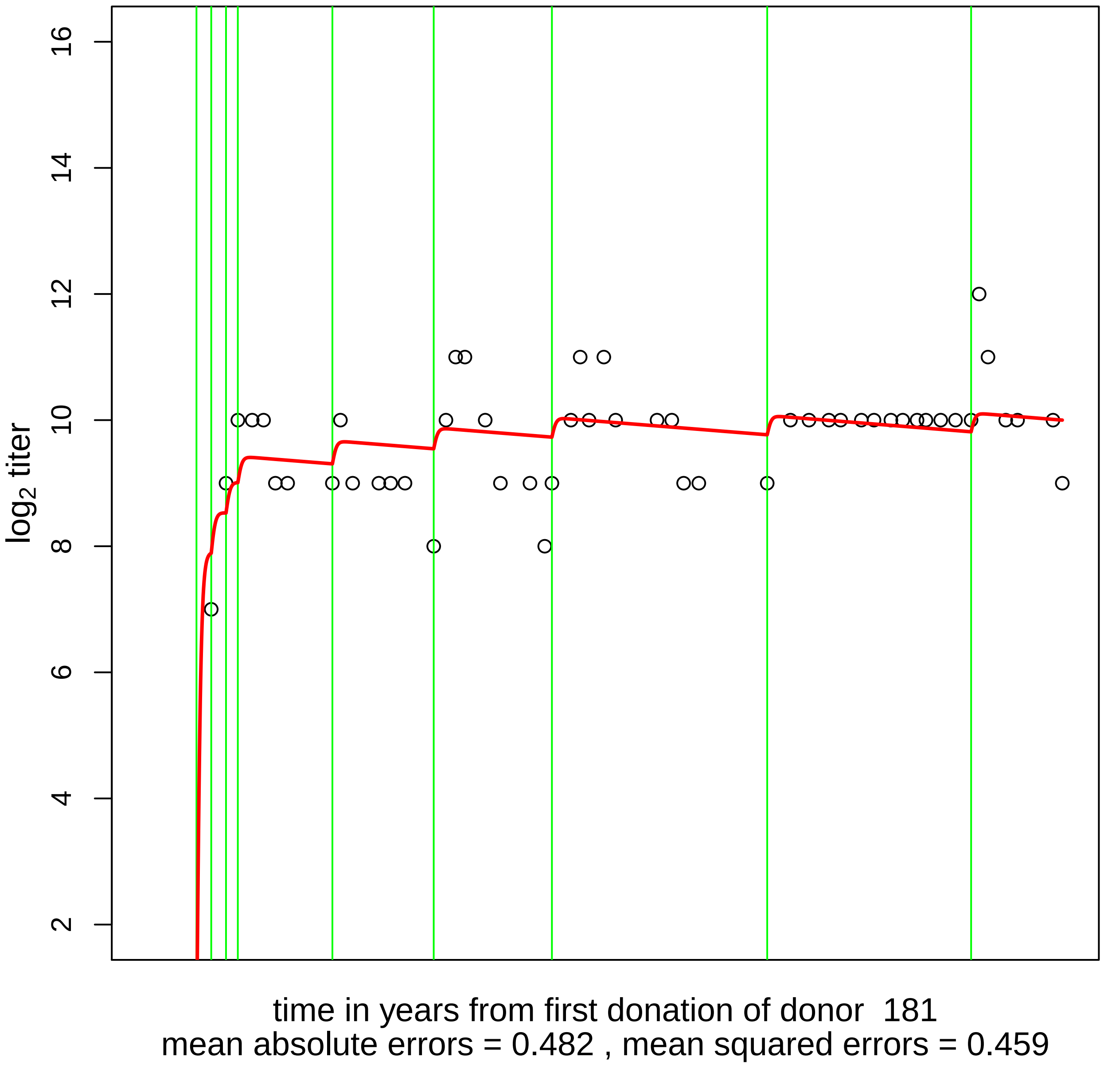

log2 titer
2
4
6
8
10
12
14
16
time in years from first donation of donor 181
mean absolute errors = 0.482 , mean squared errors = 0.459

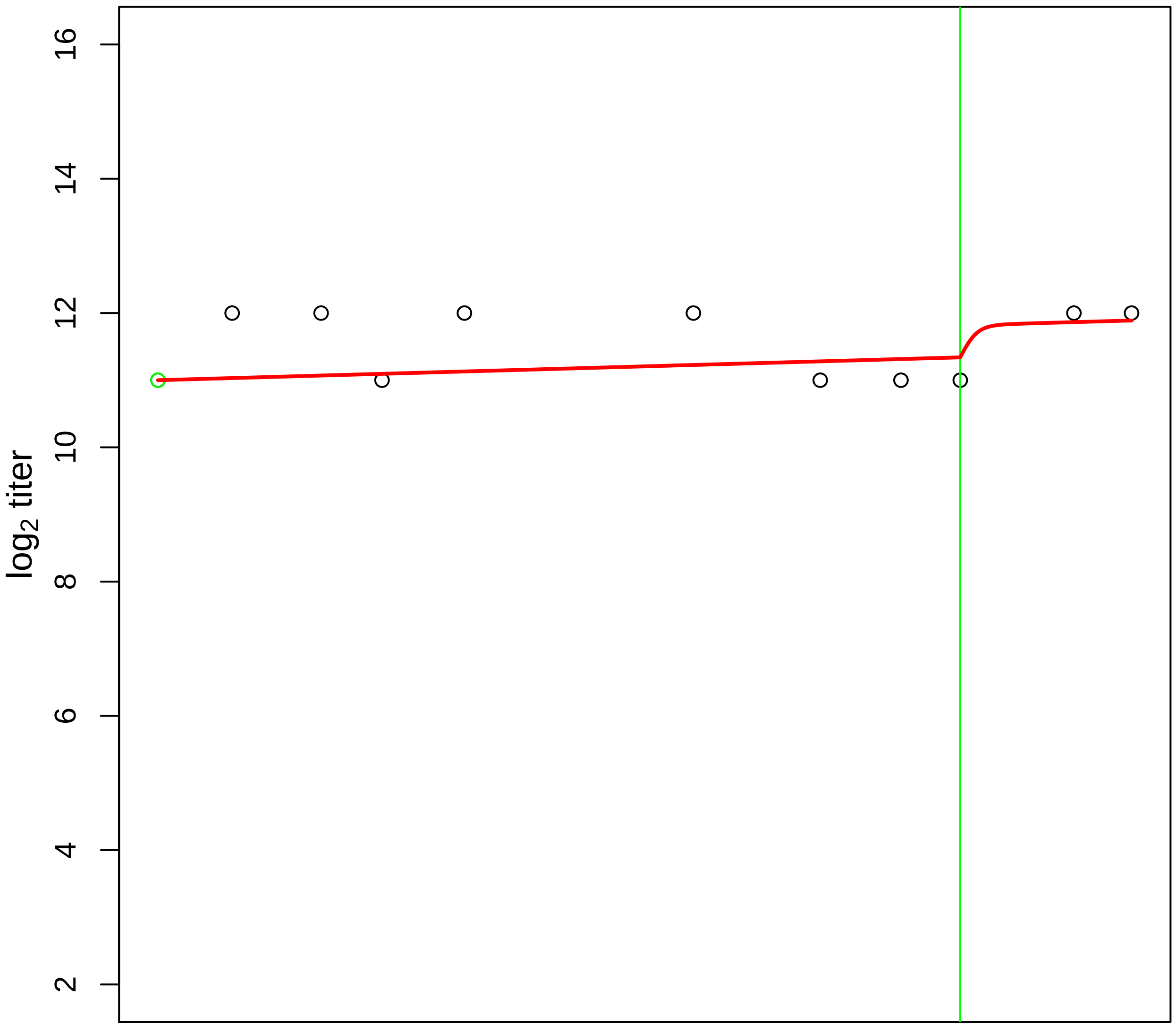

time in years from first donation of donor 182
mean absolute errors = 0.482 , mean squared errors = 0.349

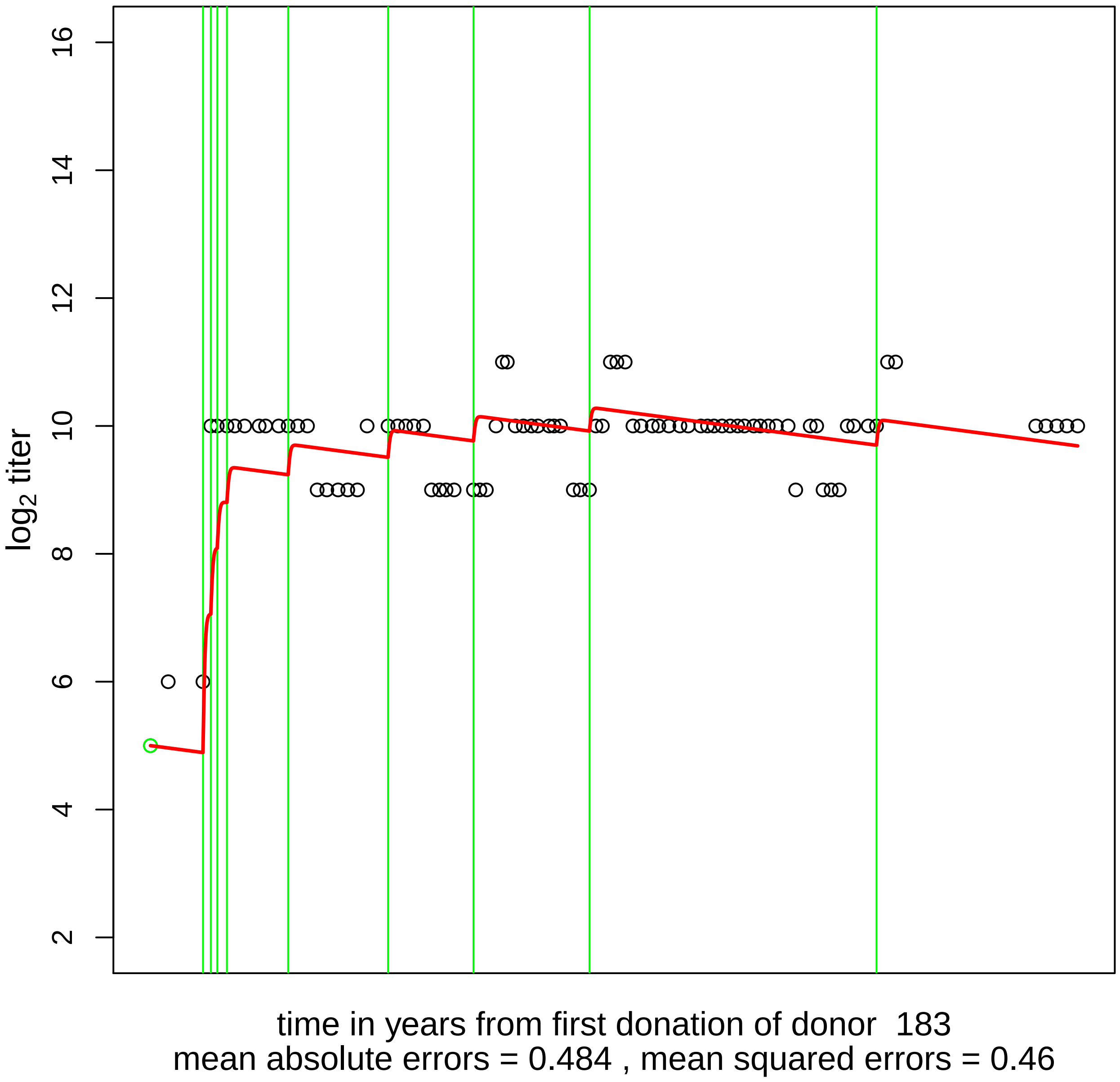

$\log_2$ titer
16
14
12
10
8
6
4
2
time in years from first donation of donor 183
mean absolute errors = 0.484 , mean squared errors = 0.46

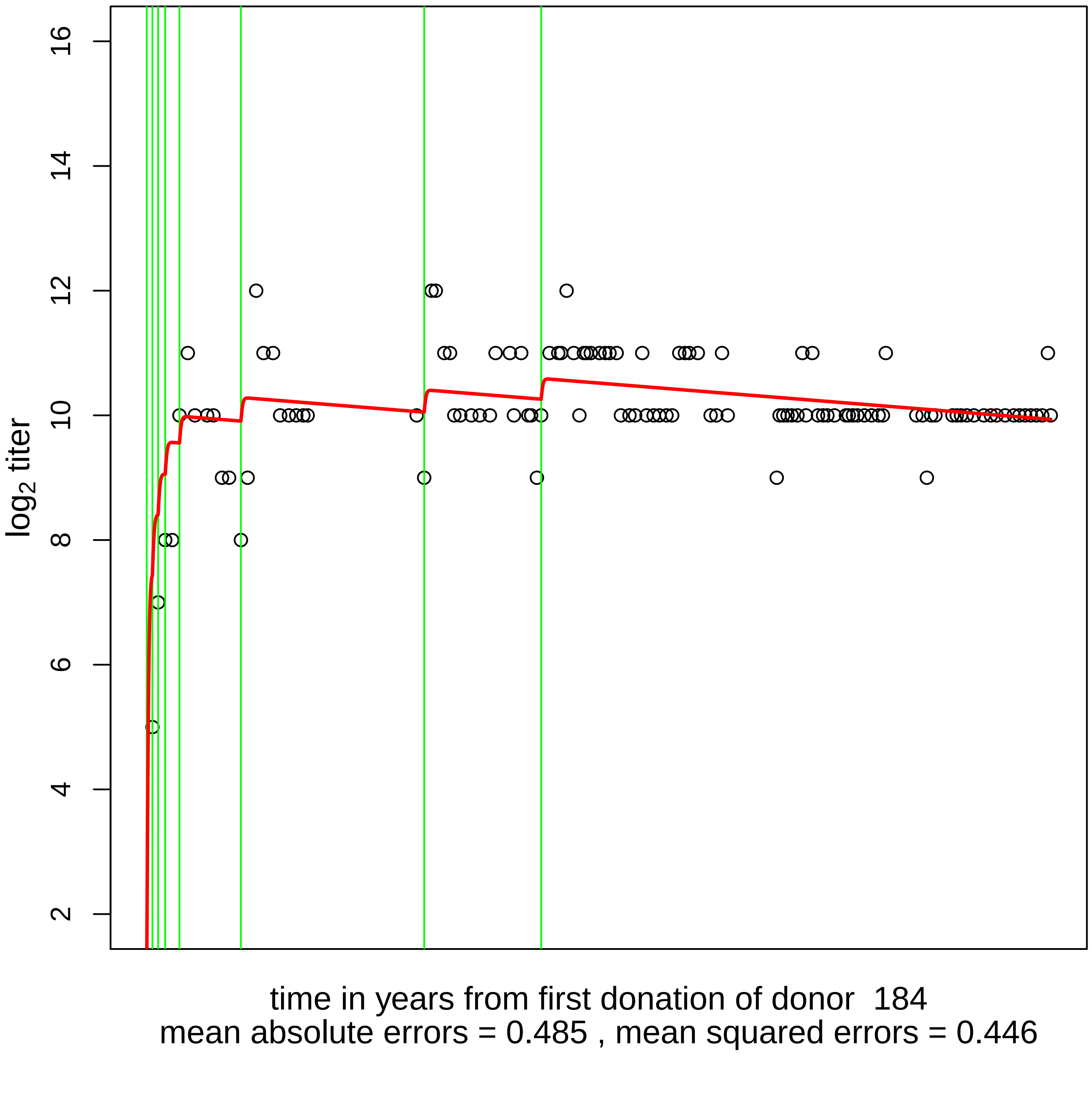

16
14
12
10
8
6
4
2
log2 titer
time in years from first donation of donor 184
mean absolute errors = 0.485 , mean squared errors = 0.446

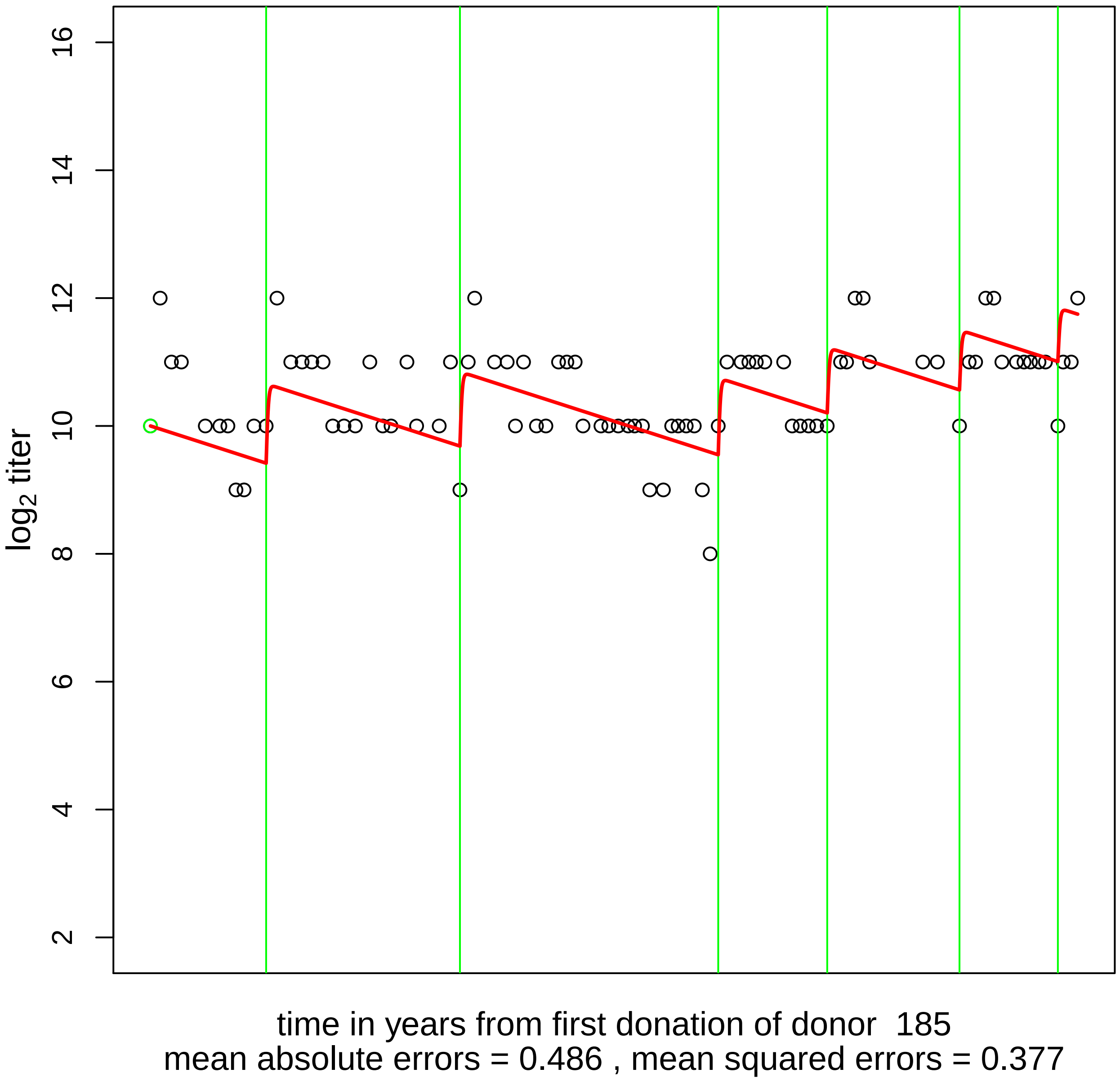

$\log_2$ titer
2
4
6
8
10
12
14
16
time in years from first donation of donor 185
mean absolute errors = 0.486 , mean squared errors = 0.377

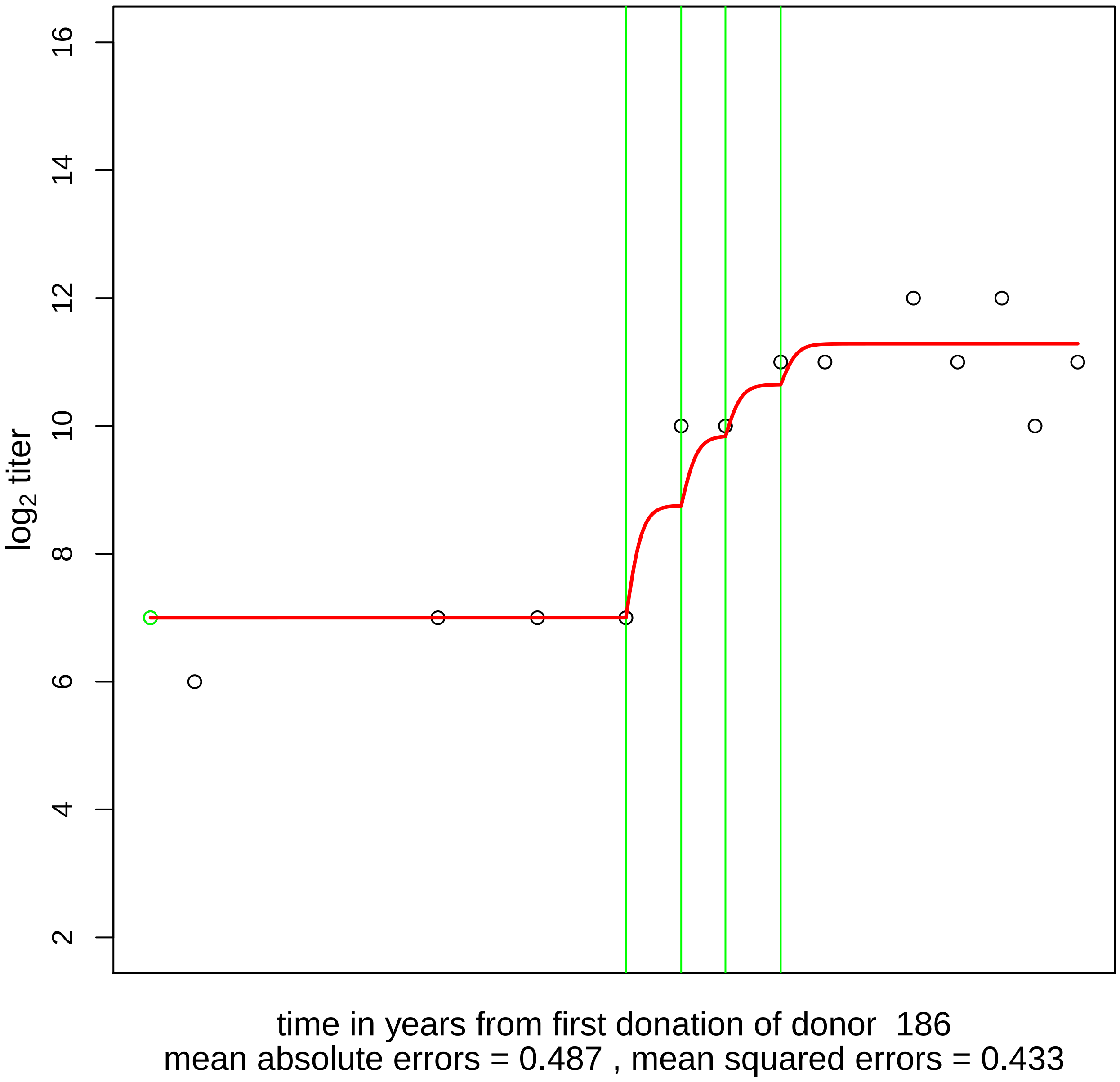

log2 titer
2
4
6
8
10
12
14
16
time in years from first donation of donor 186
mean absolute errors = 0.487 , mean squared errors = 0.433

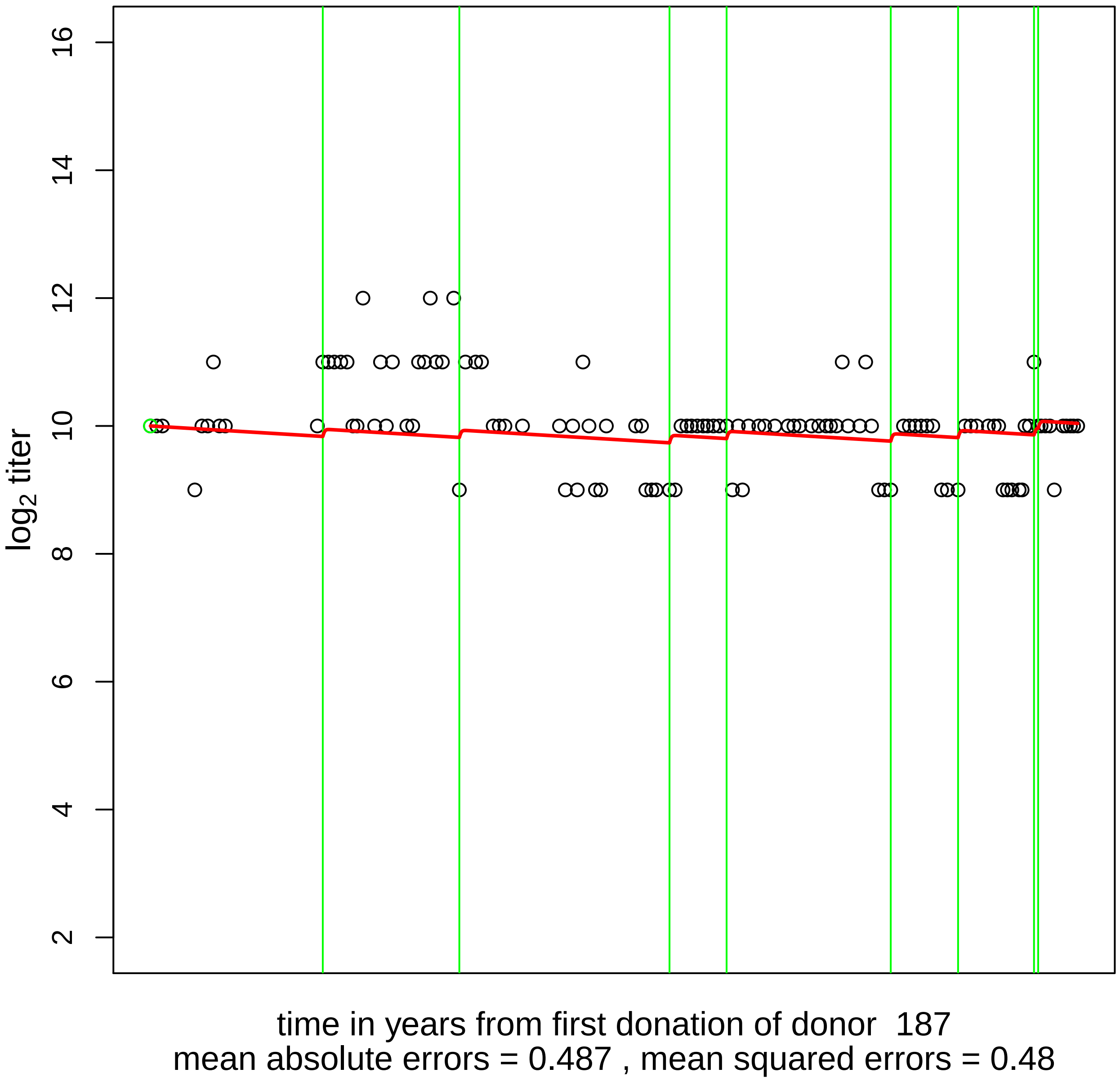

2
4
6
8
10
12
14
16
$\log_2$ titer
time in years from first donation of donor 187
mean absolute errors = 0.487 , mean squared errors = 0.48

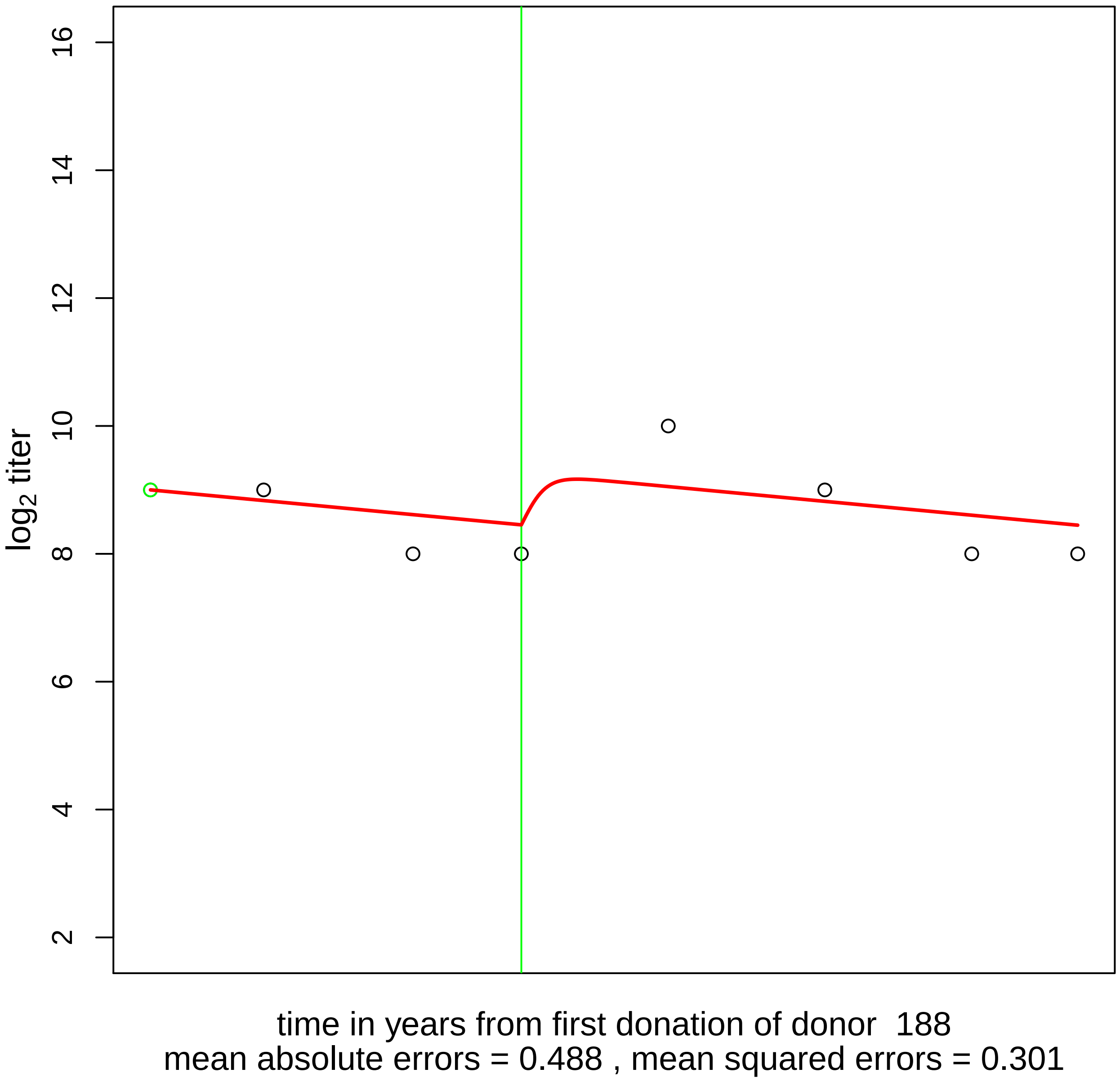

16
14
12
10
8
6
4
2
log2 titer
time in years from first donation of donor 188
mean absolute errors = 0.488 , mean squared errors = 0.301

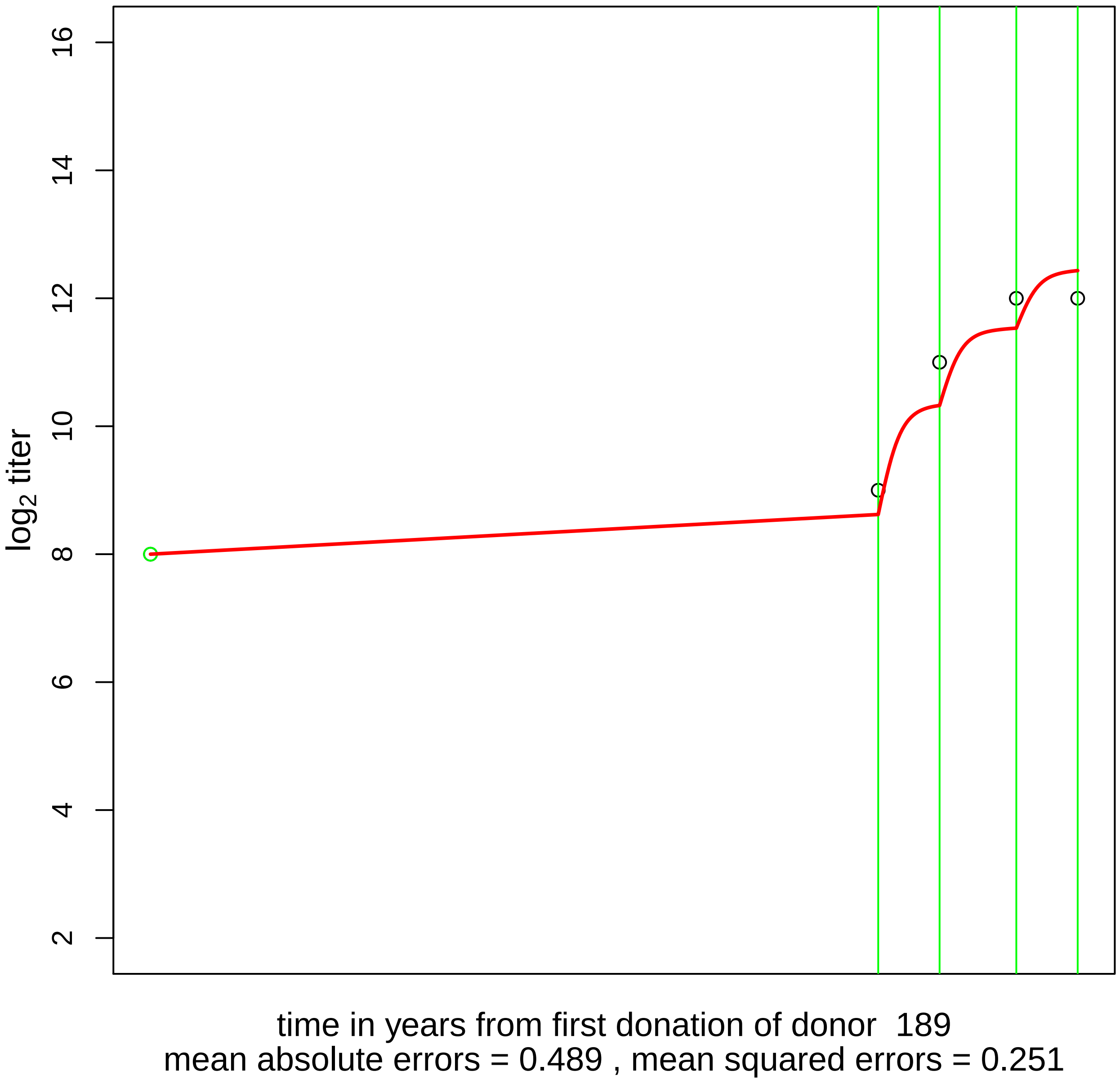

log2 titer
16
14
12
10
8
6
4
2
time in years from first donation of donor 189
mean absolute errors = 0.489 , mean squared errors = 0.251

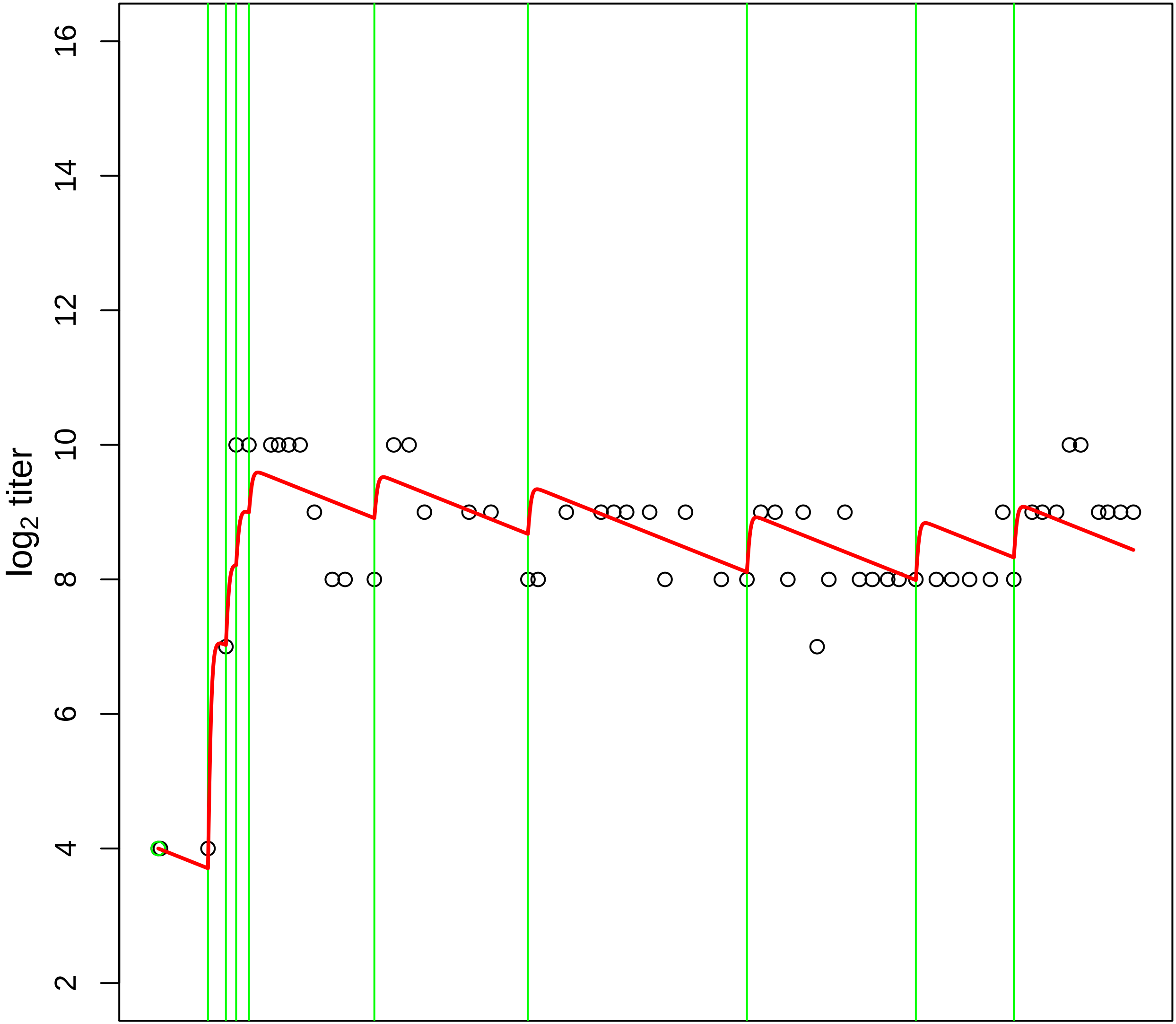

time in years from first donation of donor 190
mean absolute errors = 0.489 , mean squared errors = 0.408

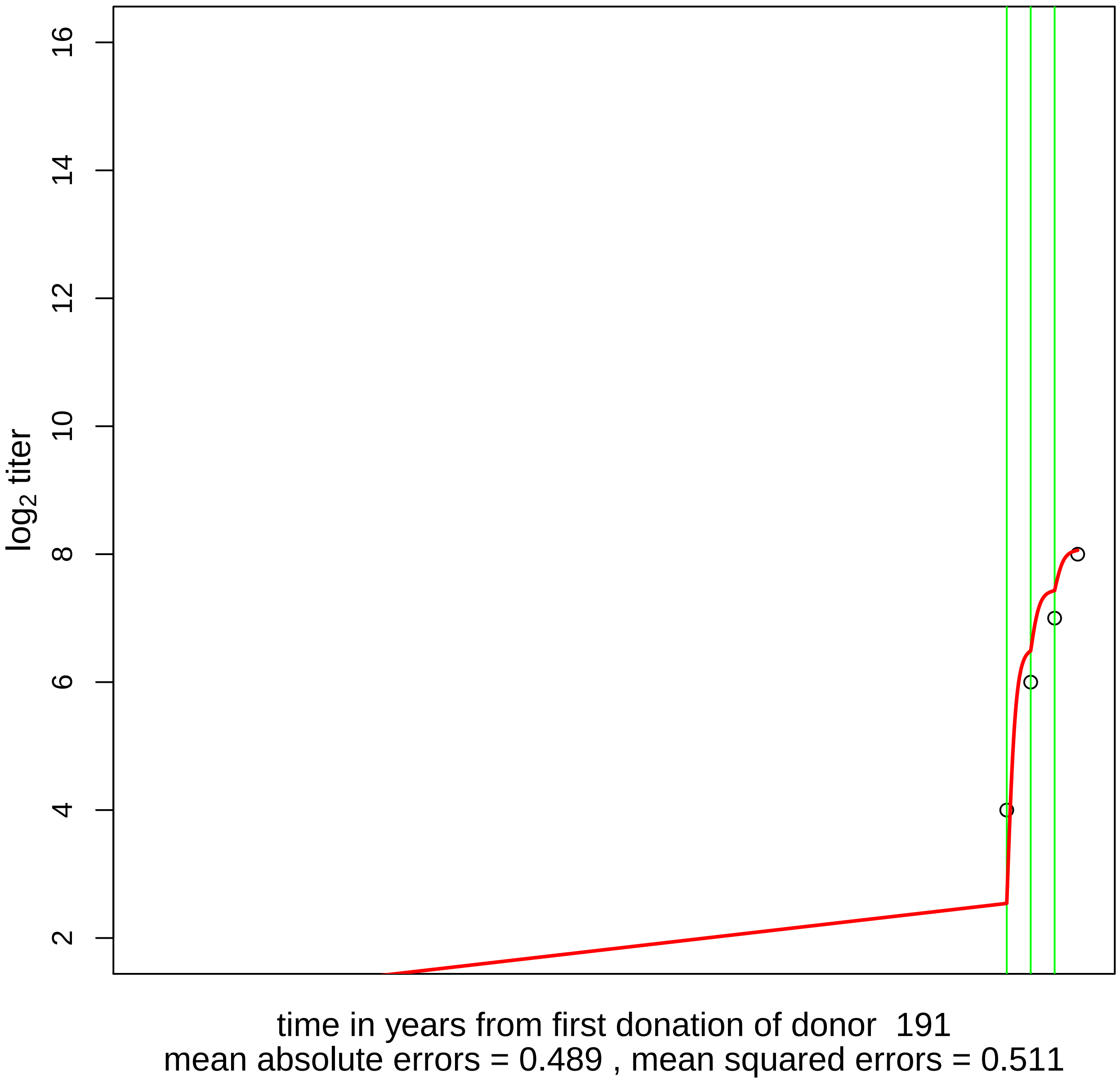
16
14
12
10
8
6
4
2
$\log_2$ titer
time in years from first donation of donor 191
mean absolute errors = 0.489 , mean squared errors = 0.511

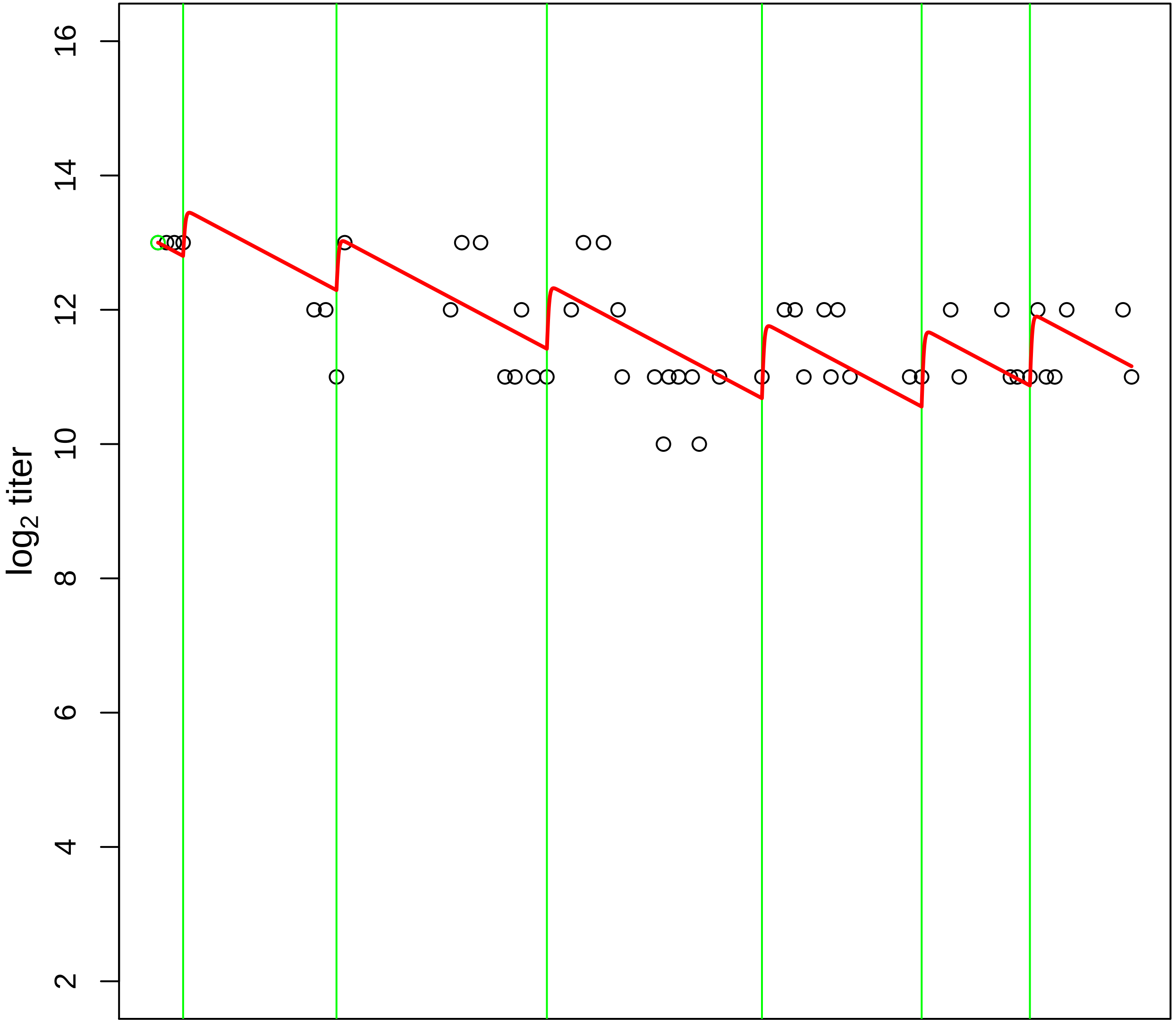

time in years from first donation of donor 192
mean absolute errors = 0.489 , mean squared errors = 0.365

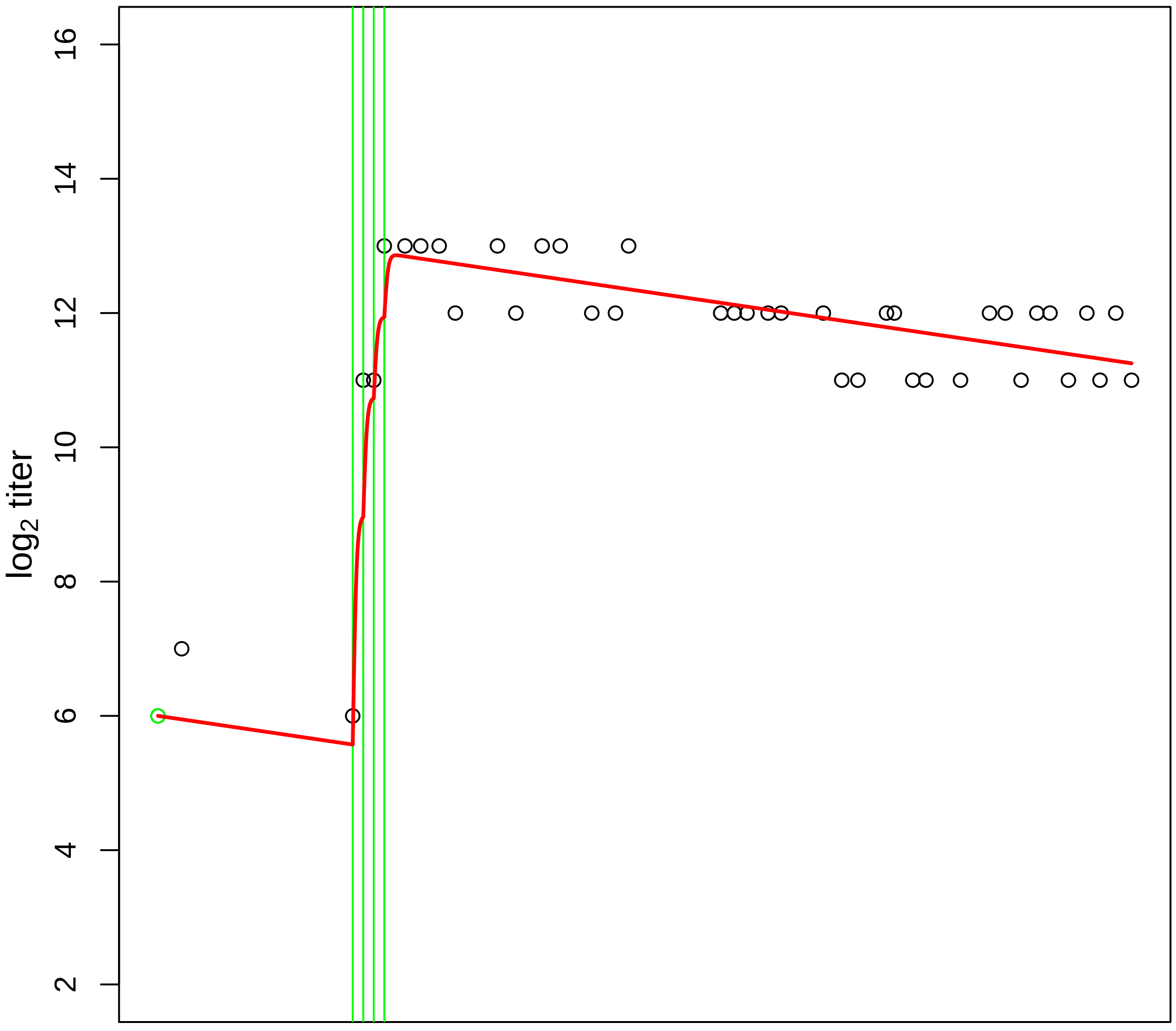

time in years from first donation of donor 193
mean absolute errors = 0.49 , mean squared errors = 0.375

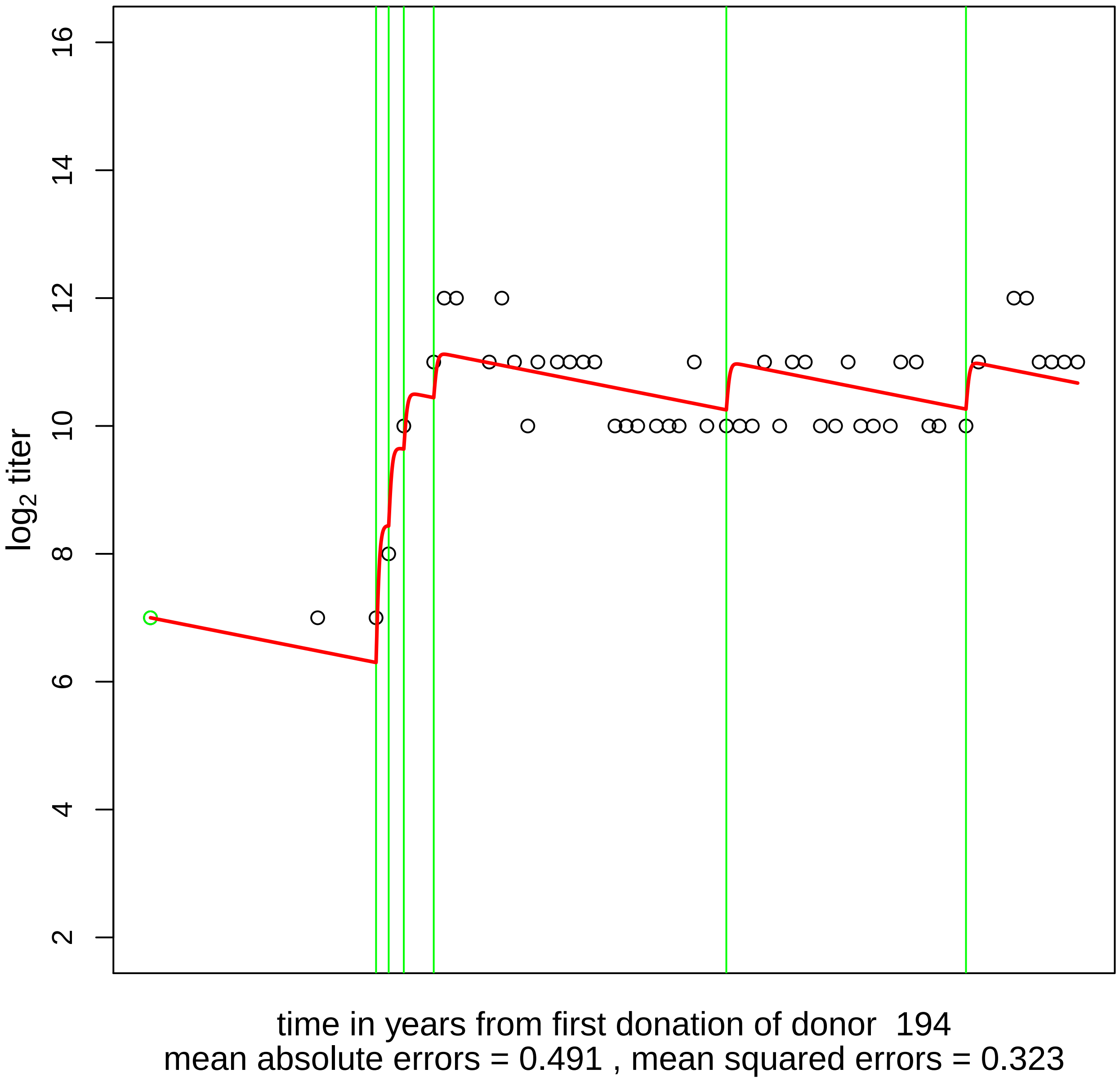

log2 titer
2
4
6
8
10
12
14
16
time in years from first donation of donor 194
mean absolute errors = 0.491 , mean squared errors = 0.323

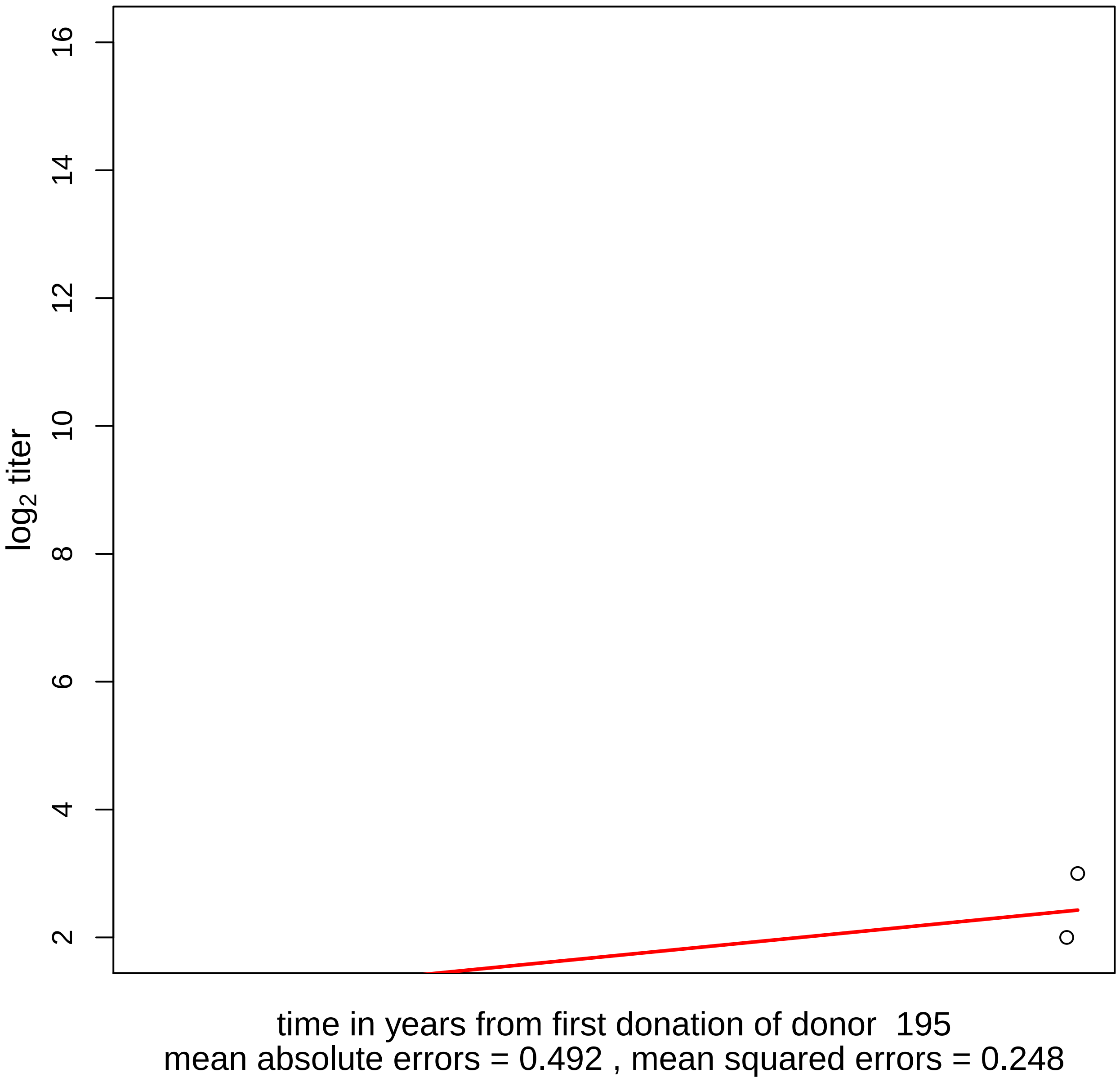

log2 titer
16
14
12
10
8
6
4
2
time in years from first donation of donor 195
mean absolute errors = 0.492 , mean squared errors = 0.248

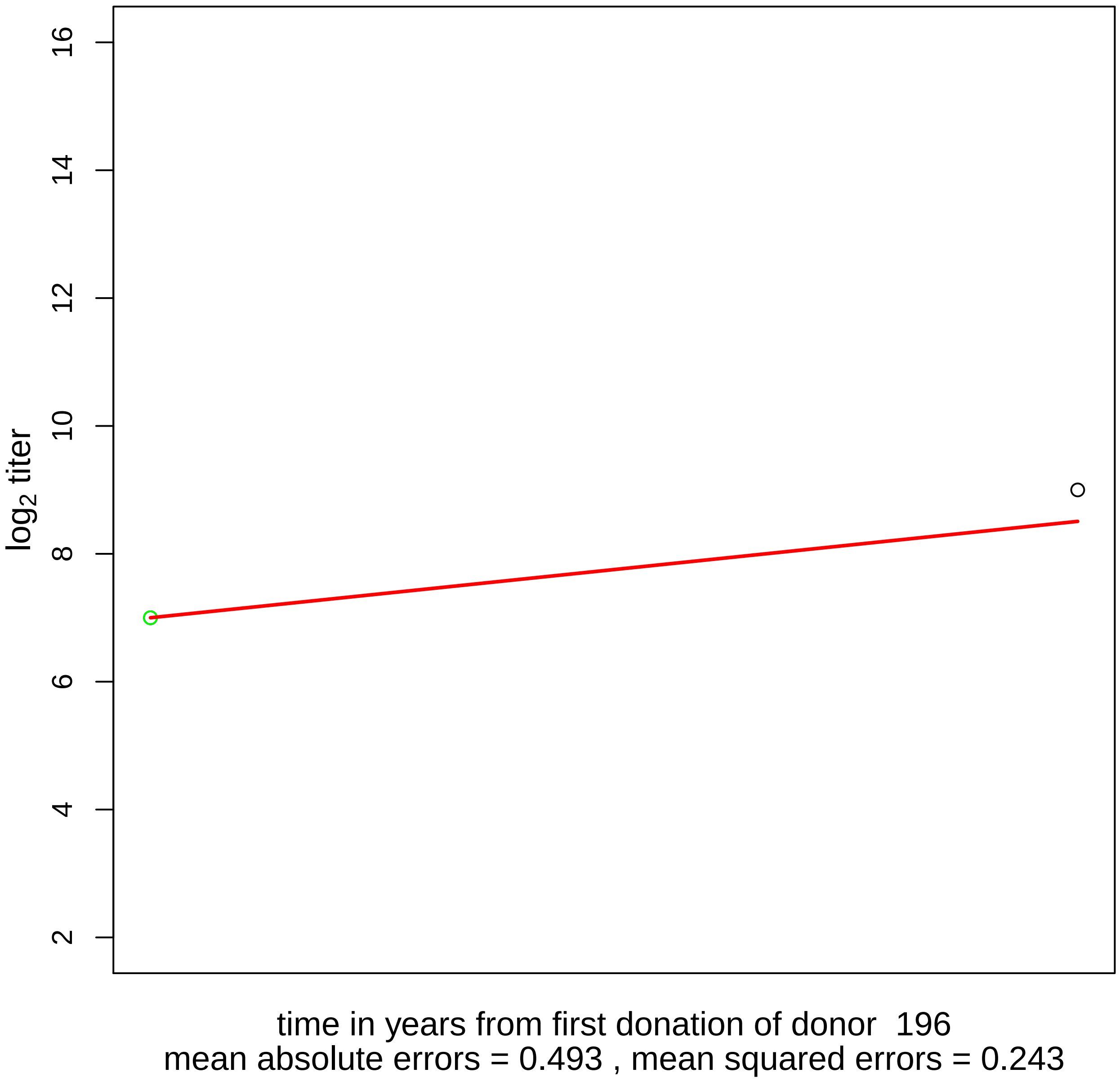

log2 titer
16
14
12
10
8
6
4
2
time in years from first donation of donor 196
mean absolute errors = 0.493 , mean squared errors = 0.243

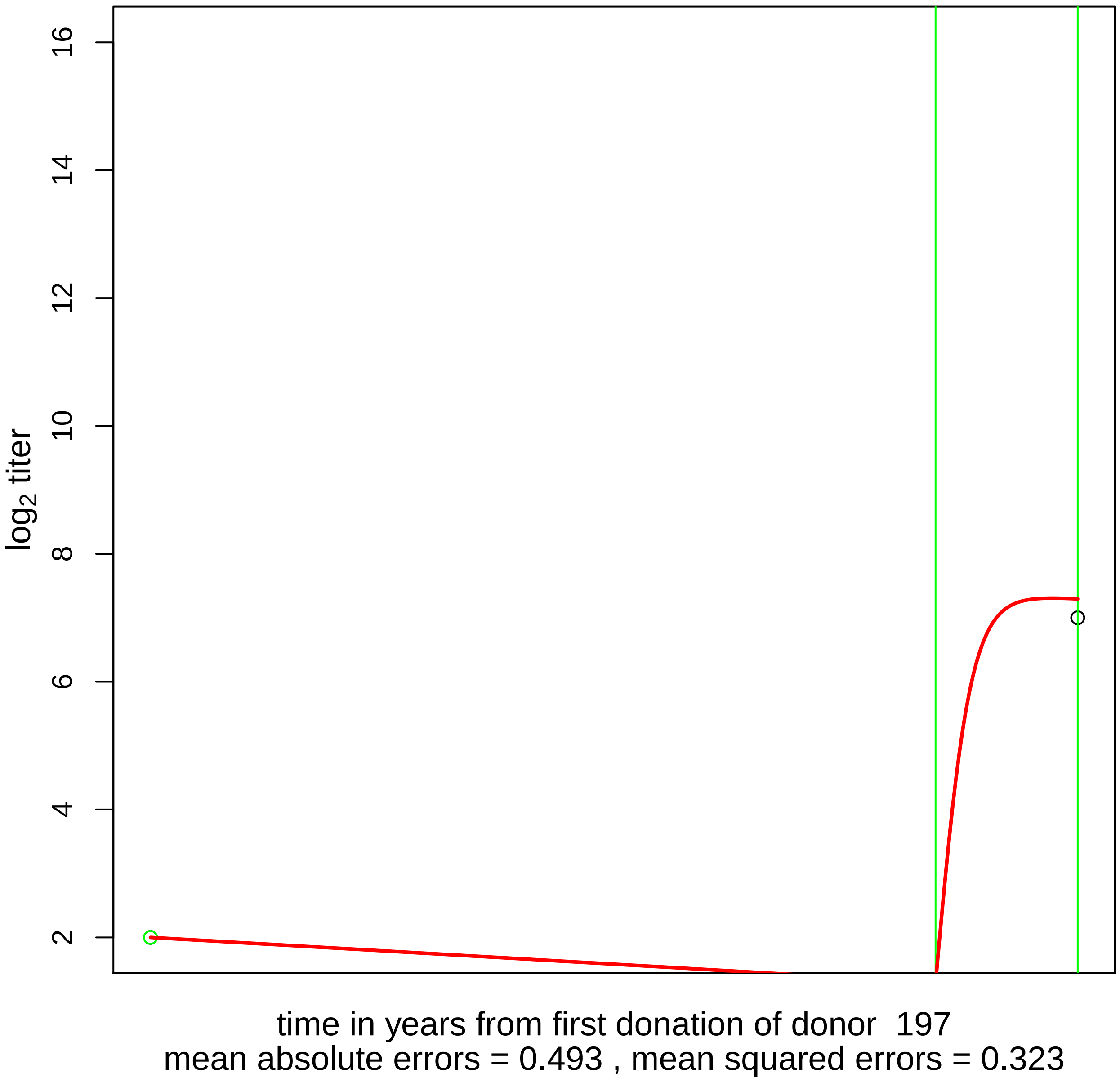

log2 titer
16
14
12
10
8
6
4
2
time in years from first donation of donor 197
mean absolute errors = 0.493 , mean squared errors = 0.323

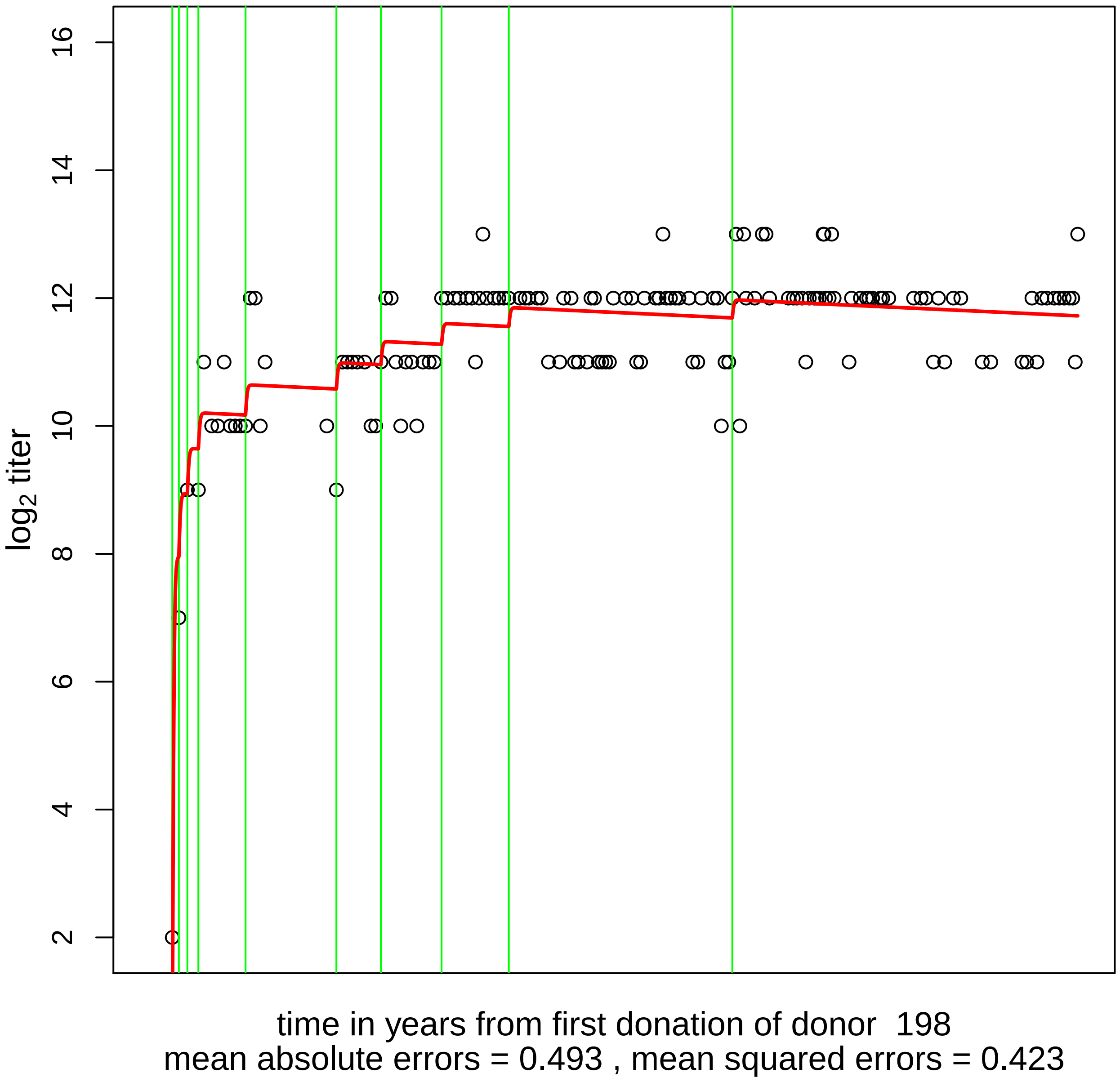

log_2 titer
2
4
6
8
10
12
14
16
time in years from first donation of donor 198
mean absolute errors = 0.493 , mean squared errors = 0.423

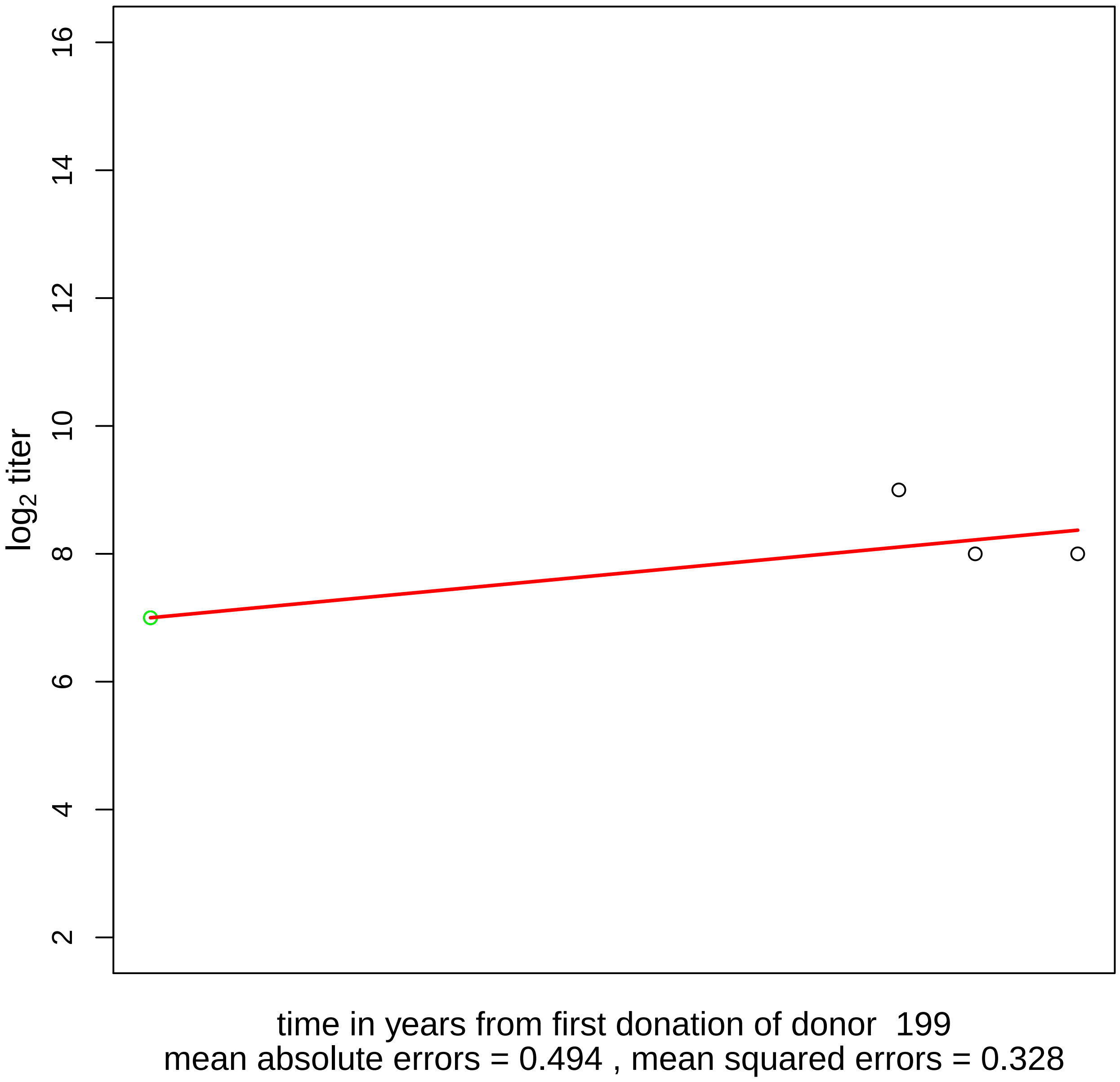
log2 titer
16
14
12
10
8
6
4
2
time in years from first donation of donor 199
mean absolute errors = 0.494 , mean squared errors = 0.328

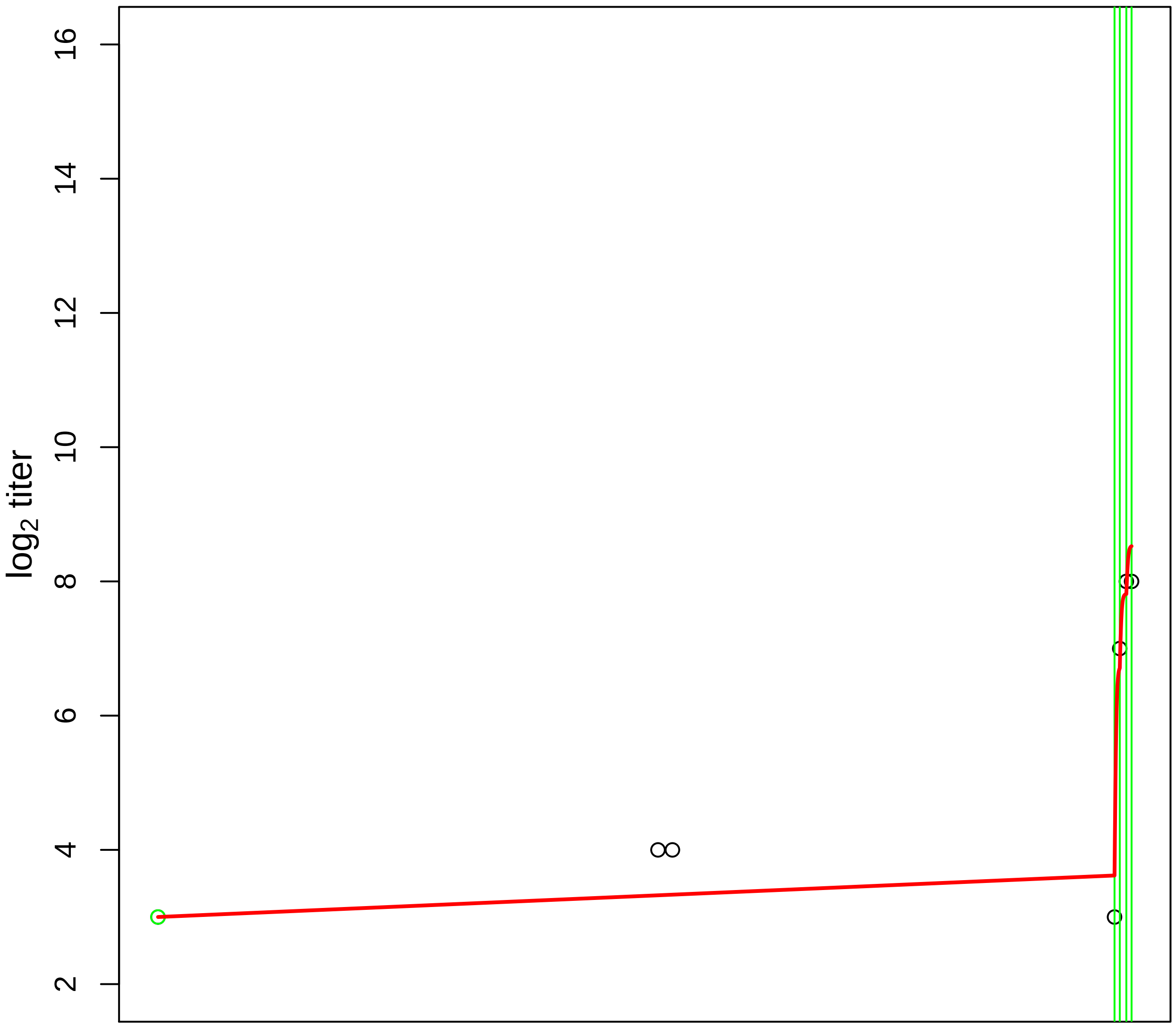

time in years from first donation of donor 200
mean absolute errors = 0.495 , mean squared errors = 0.28

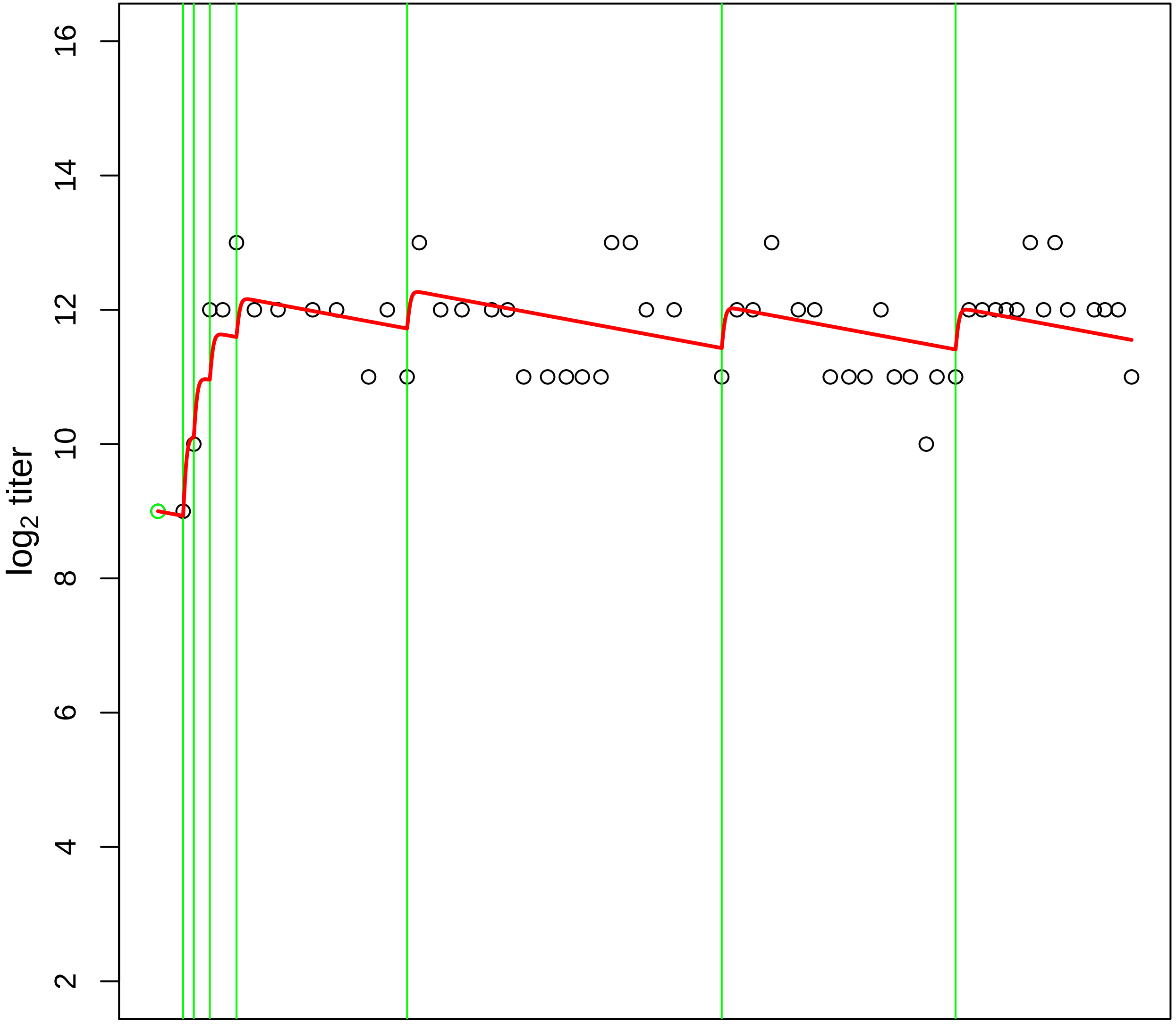

time in years from first donation of donor 201
mean absolute errors = 0.496 , mean squared errors = 0.419

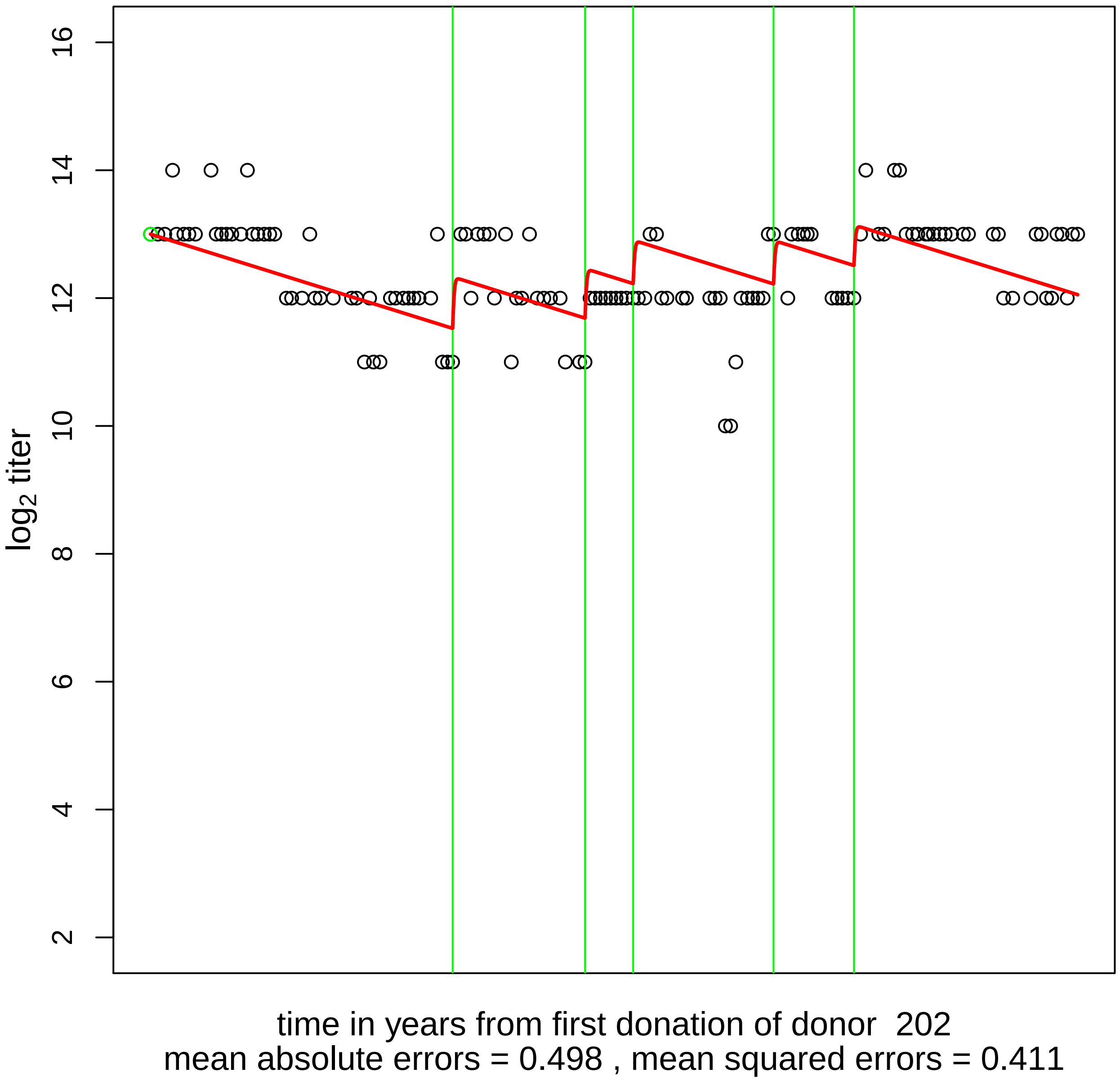
16
14
12
10
8
6
4
2
$\log_2$ titer
time in years from first donation of donor 202
mean absolute errors = 0.498 , mean squared errors = 0.411

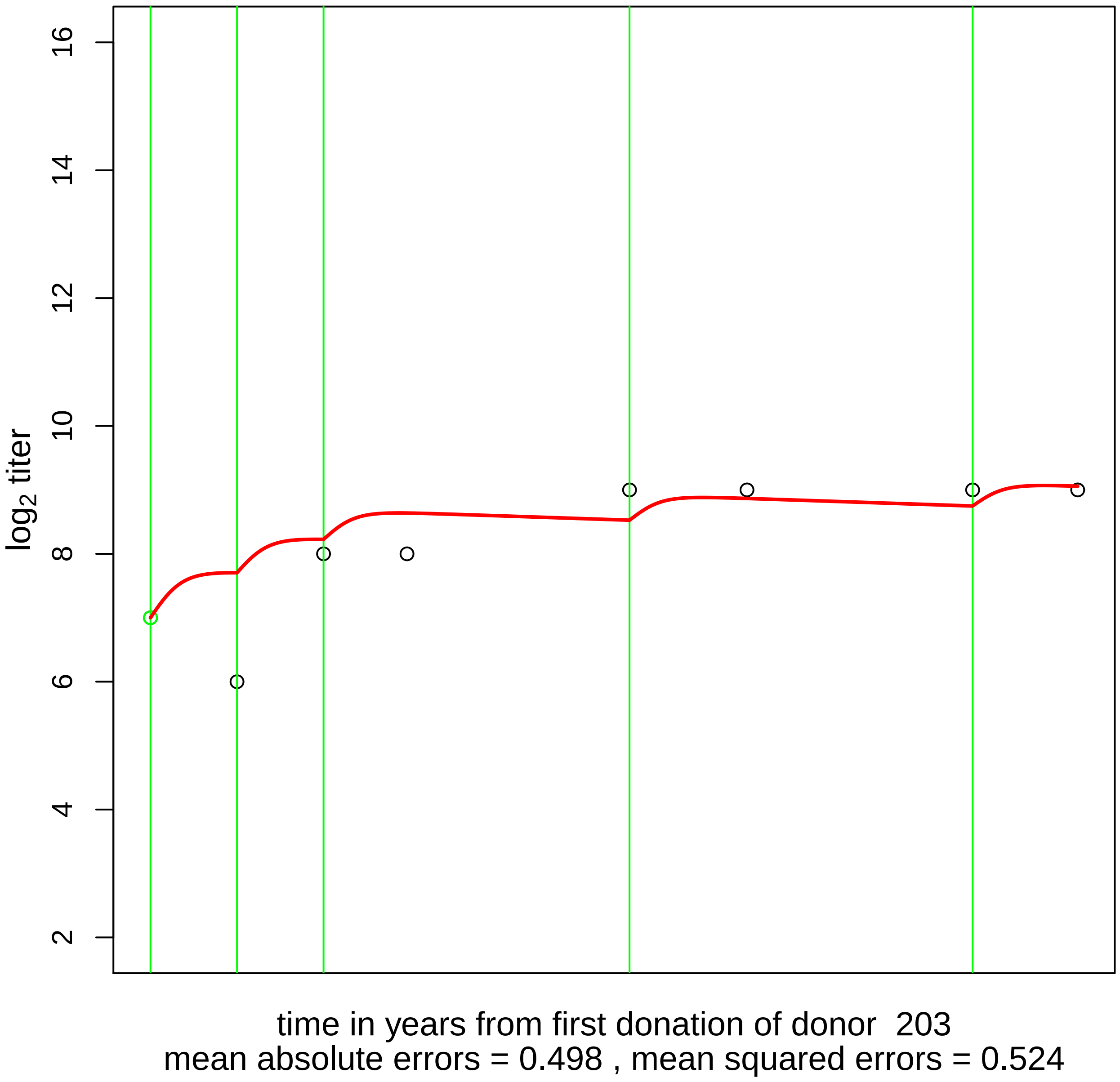

log2 titer
2
4
6
8
10
12
14
16
time in years from first donation of donor 203
mean absolute errors = 0.498 , mean squared errors = 0.524

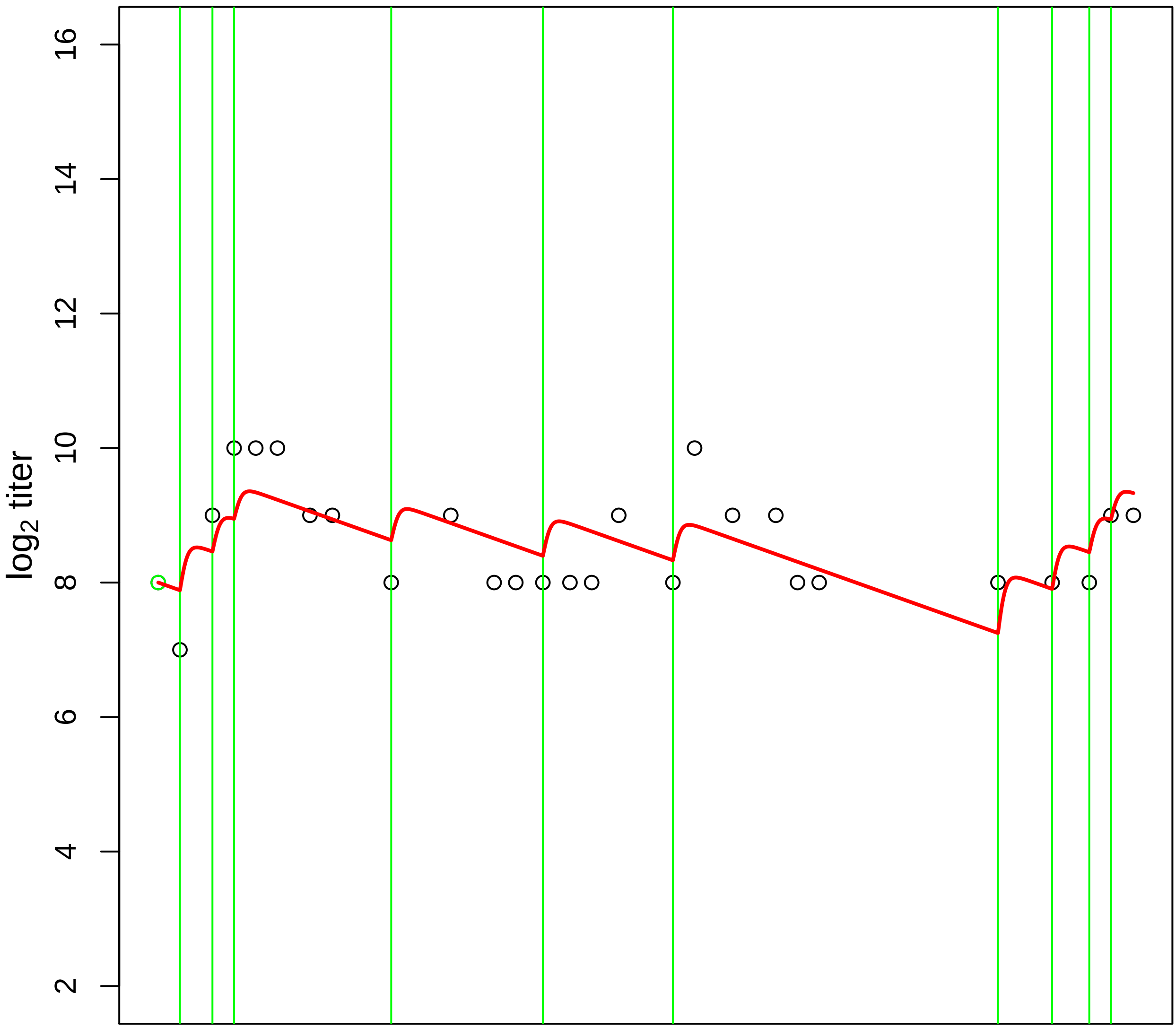

time in years from first donation of donor 204
mean absolute errors = 0.499 , mean squared errors = 0.343

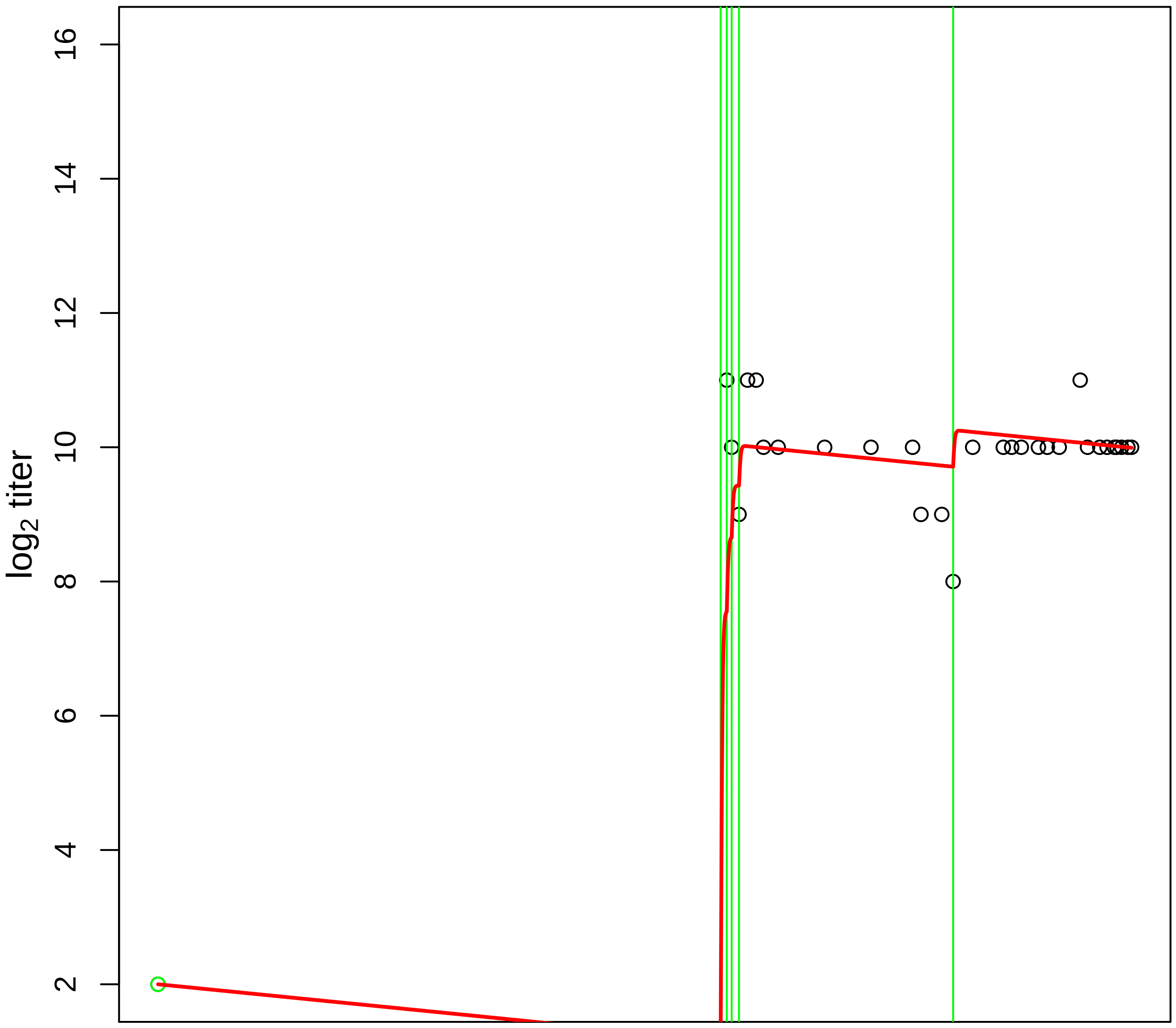

time in years from first donation of donor 205
mean absolute errors = 0.499 , mean squared errors = 0.769

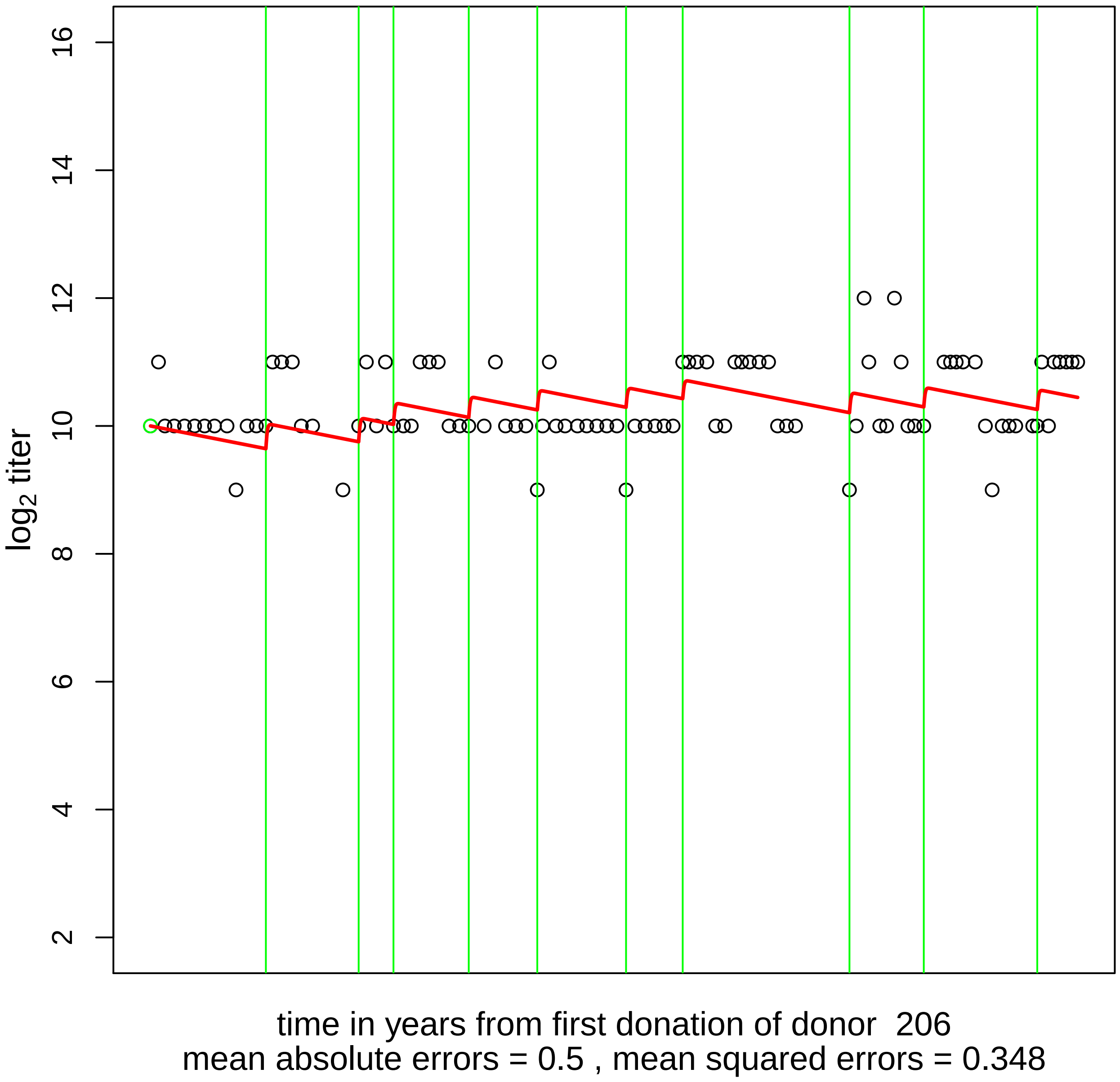

16
14
12
10
8
6
4
2
$\log_2$ titer
time in years from first donation of donor 206
mean absolute errors = 0.5 , mean squared errors = 0.348

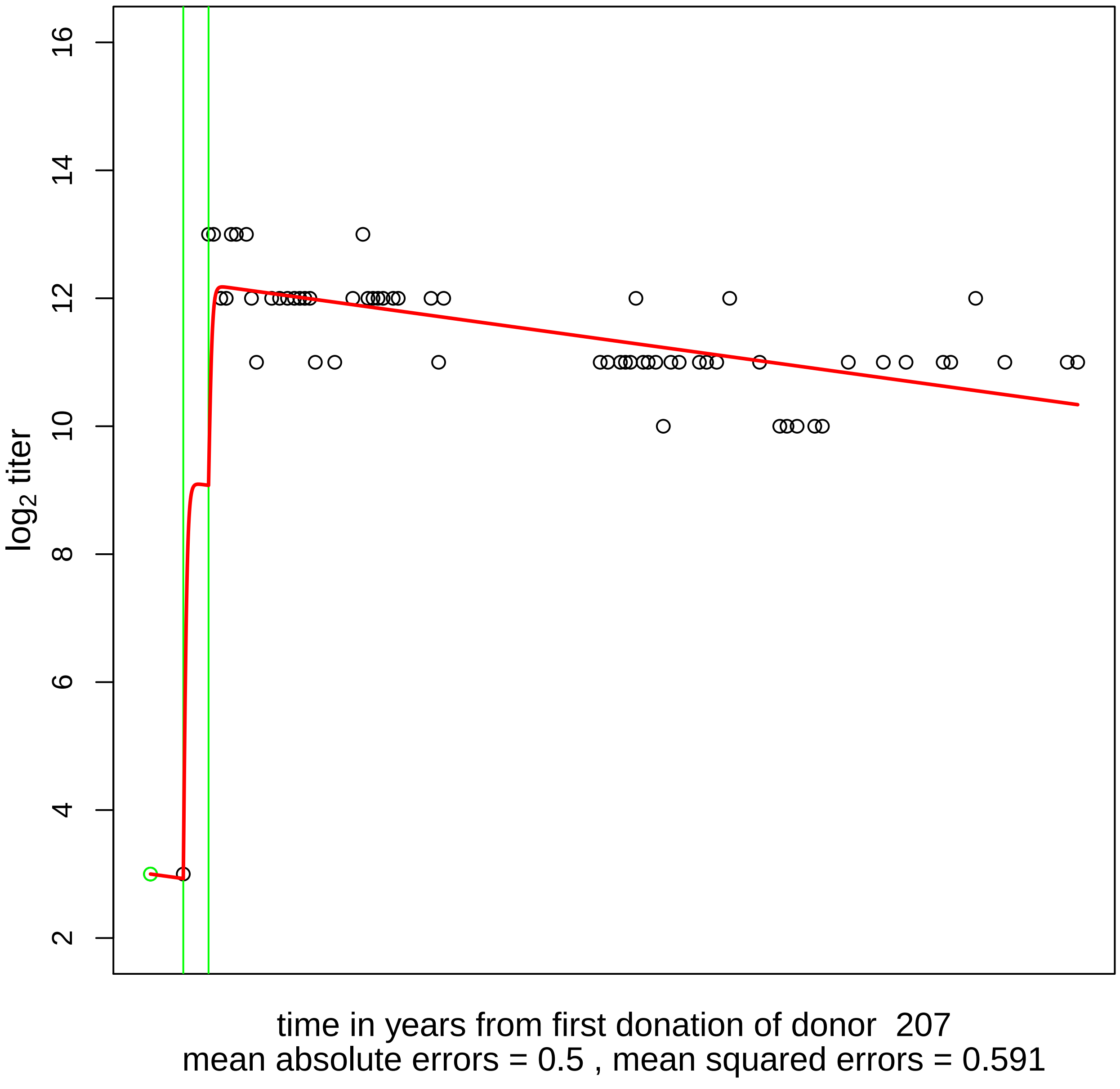

log2 titer
16
14
12
10
8
6
4
2
time in years from first donation of donor 207
mean absolute errors = 0.5 , mean squared errors = 0.591

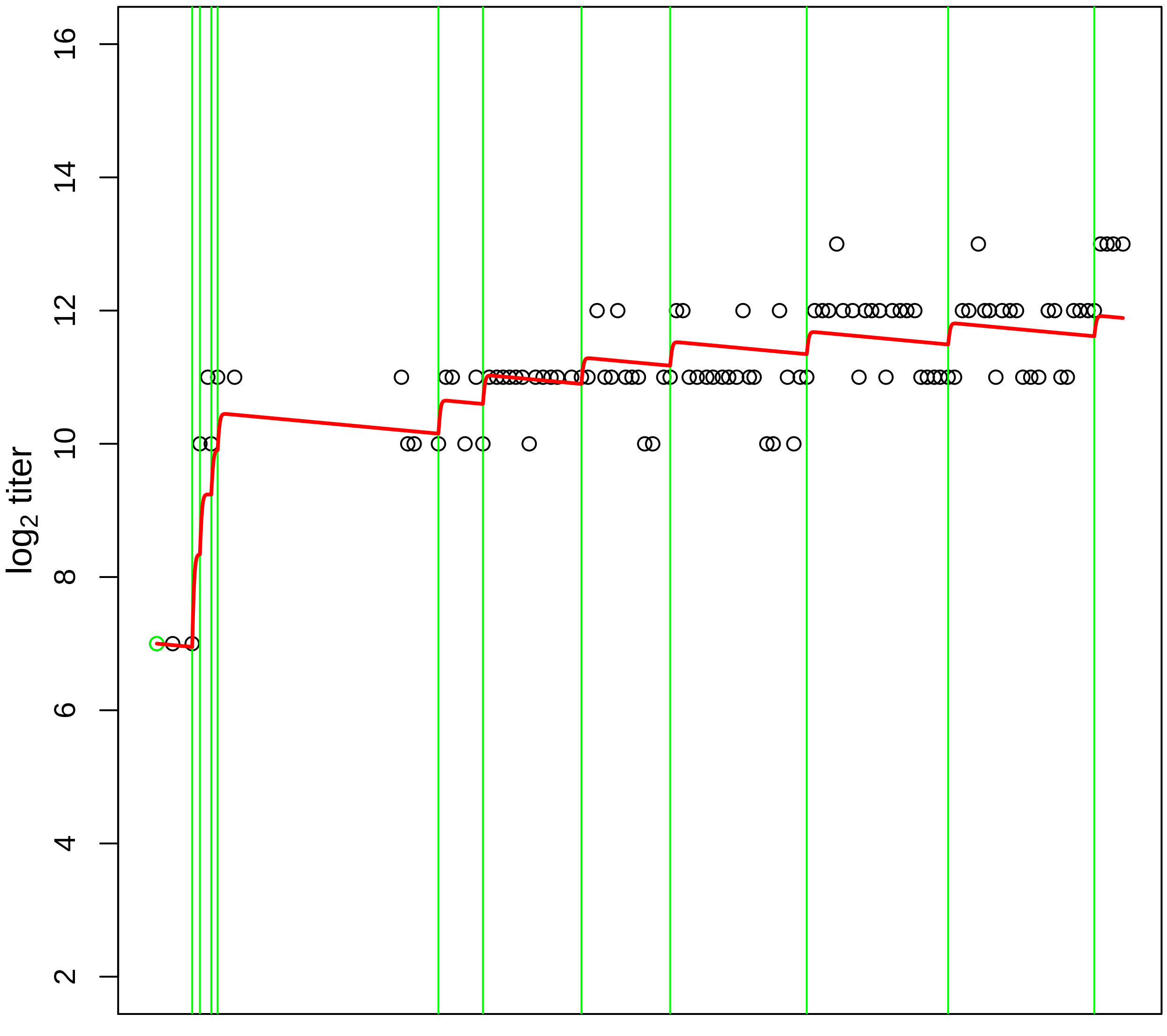

time in years from first donation of donor 208
mean absolute errors = 0.5 , mean squared errors = 0.393

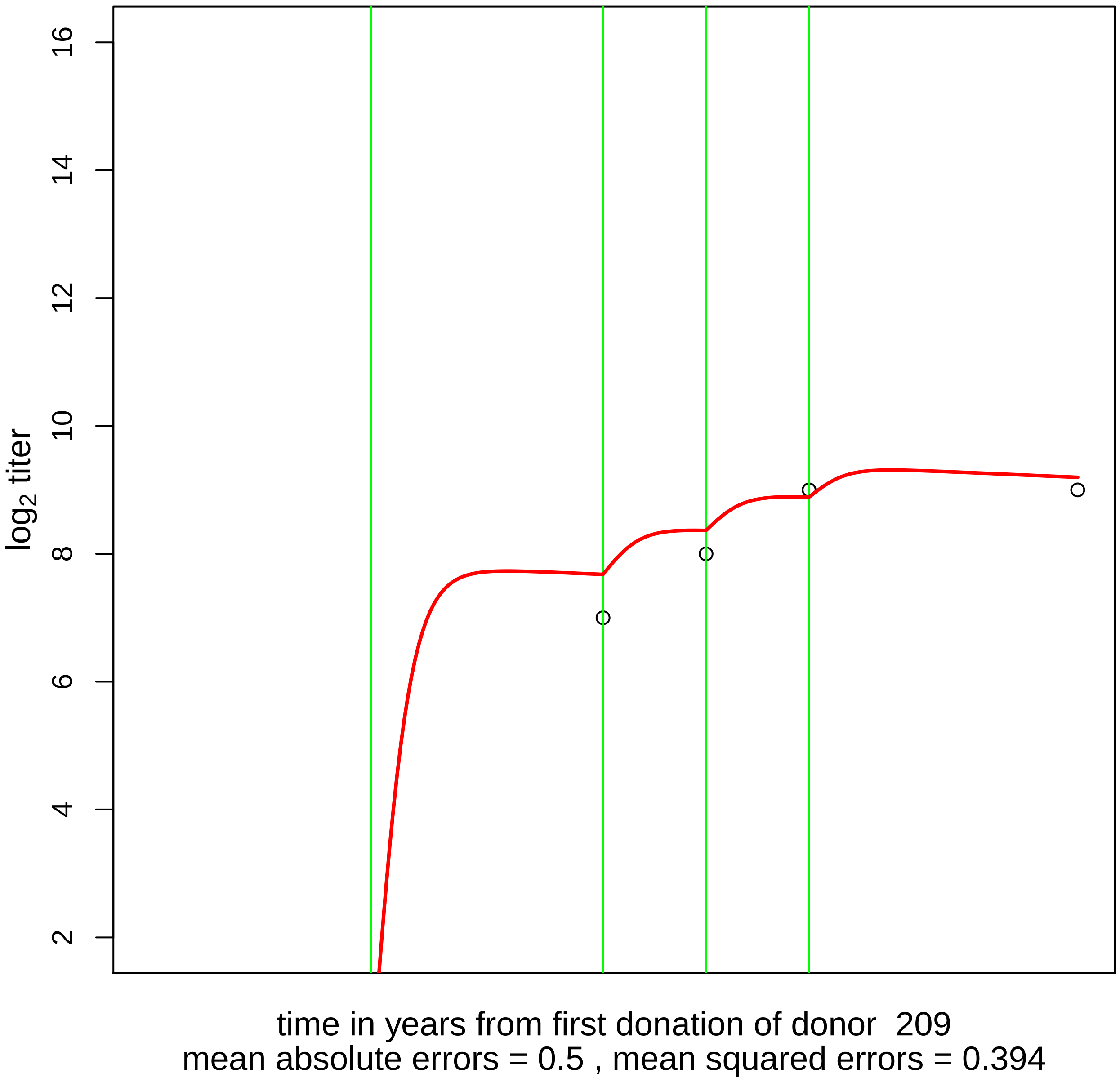

16
14
12
10
8
6
4
2
log2 titer
time in years from first donation of donor 209
mean absolute errors = 0.5 , mean squared errors = 0.394

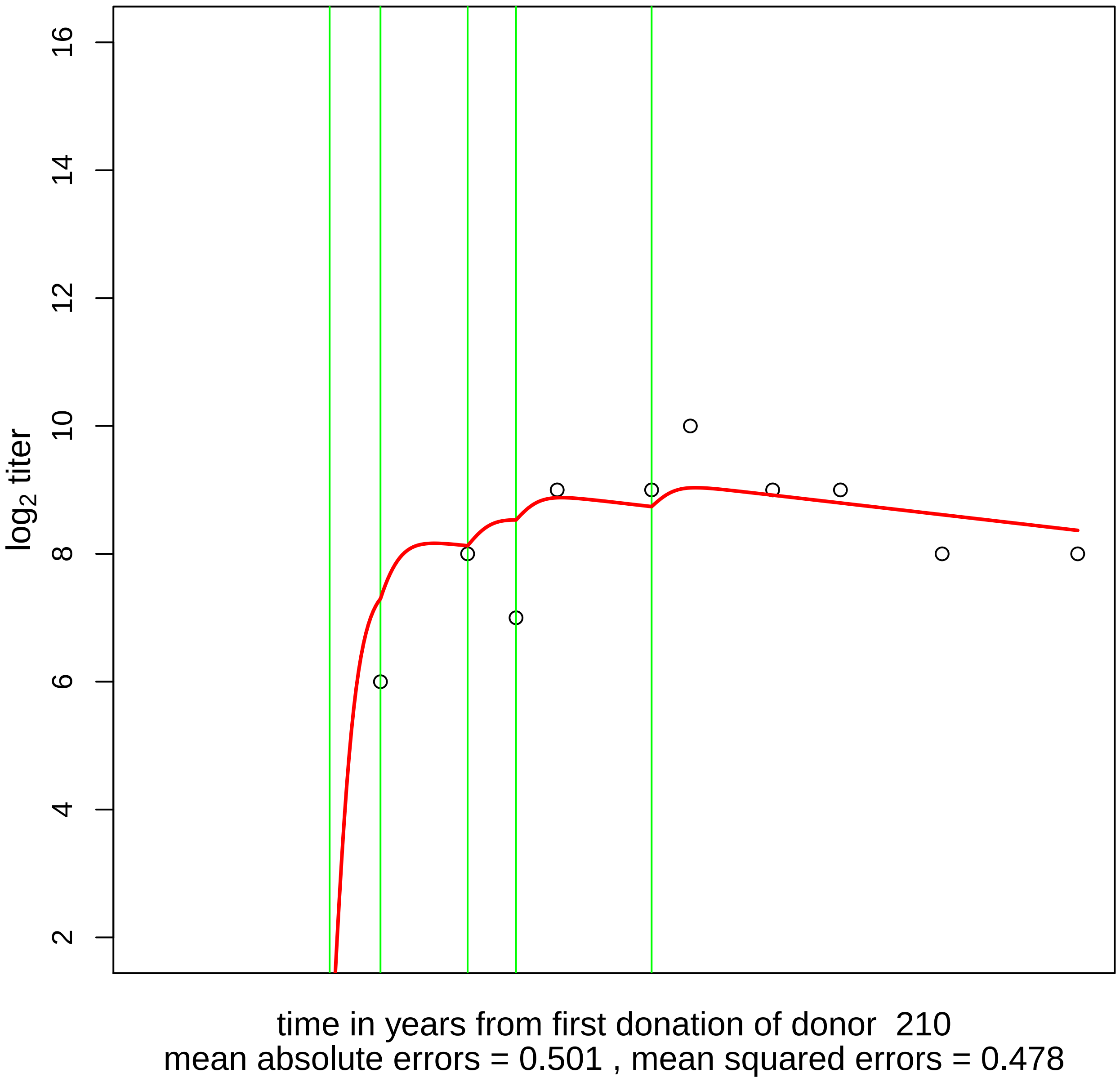

16
14
12
10
8
6
4
2
log2 titer
time in years from first donation of donor 210
mean absolute errors = 0.501 , mean squared errors = 0.478

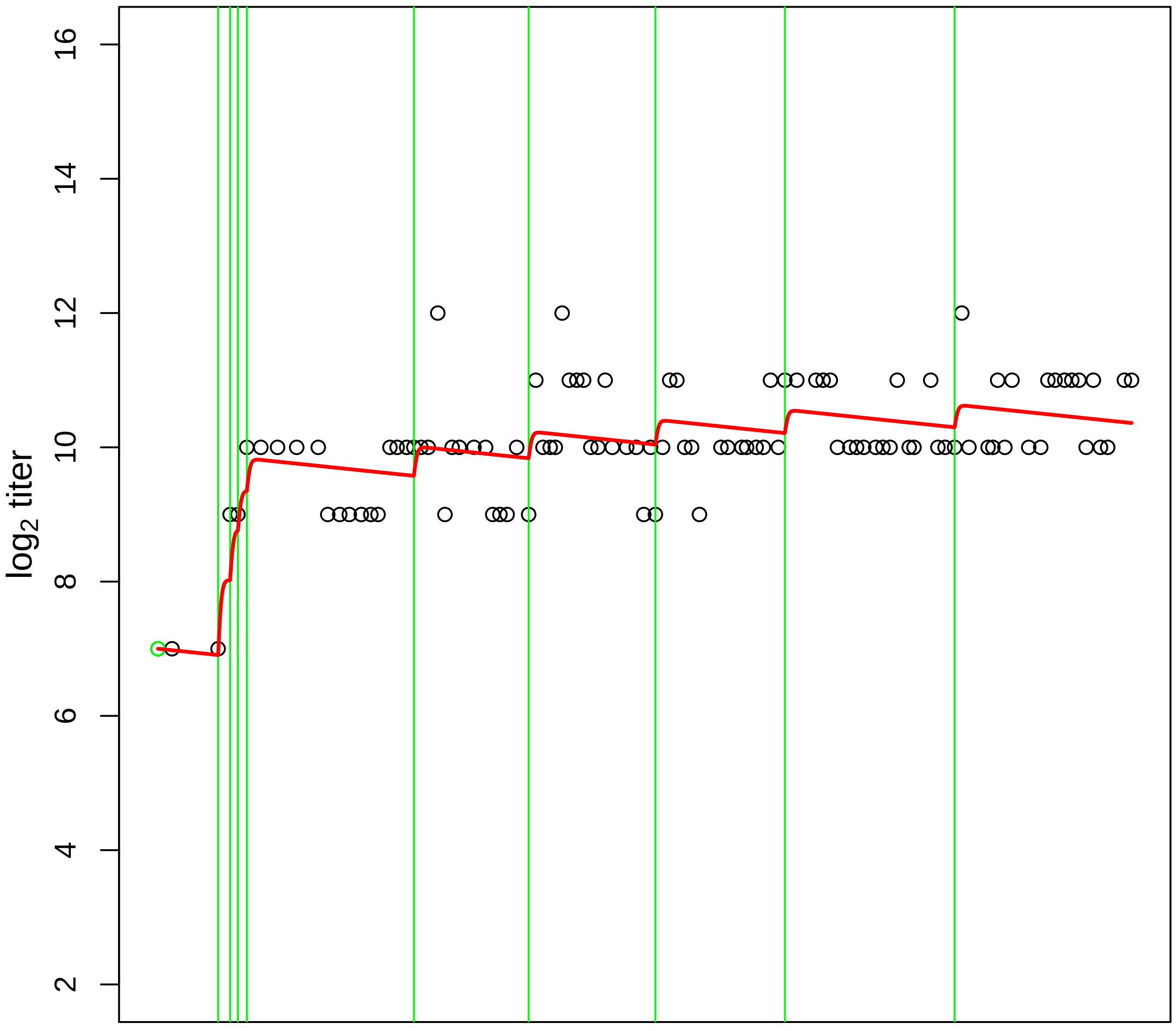

time in years from first donation of donor 211
mean absolute errors = 0.502 , mean squared errors = 0.372

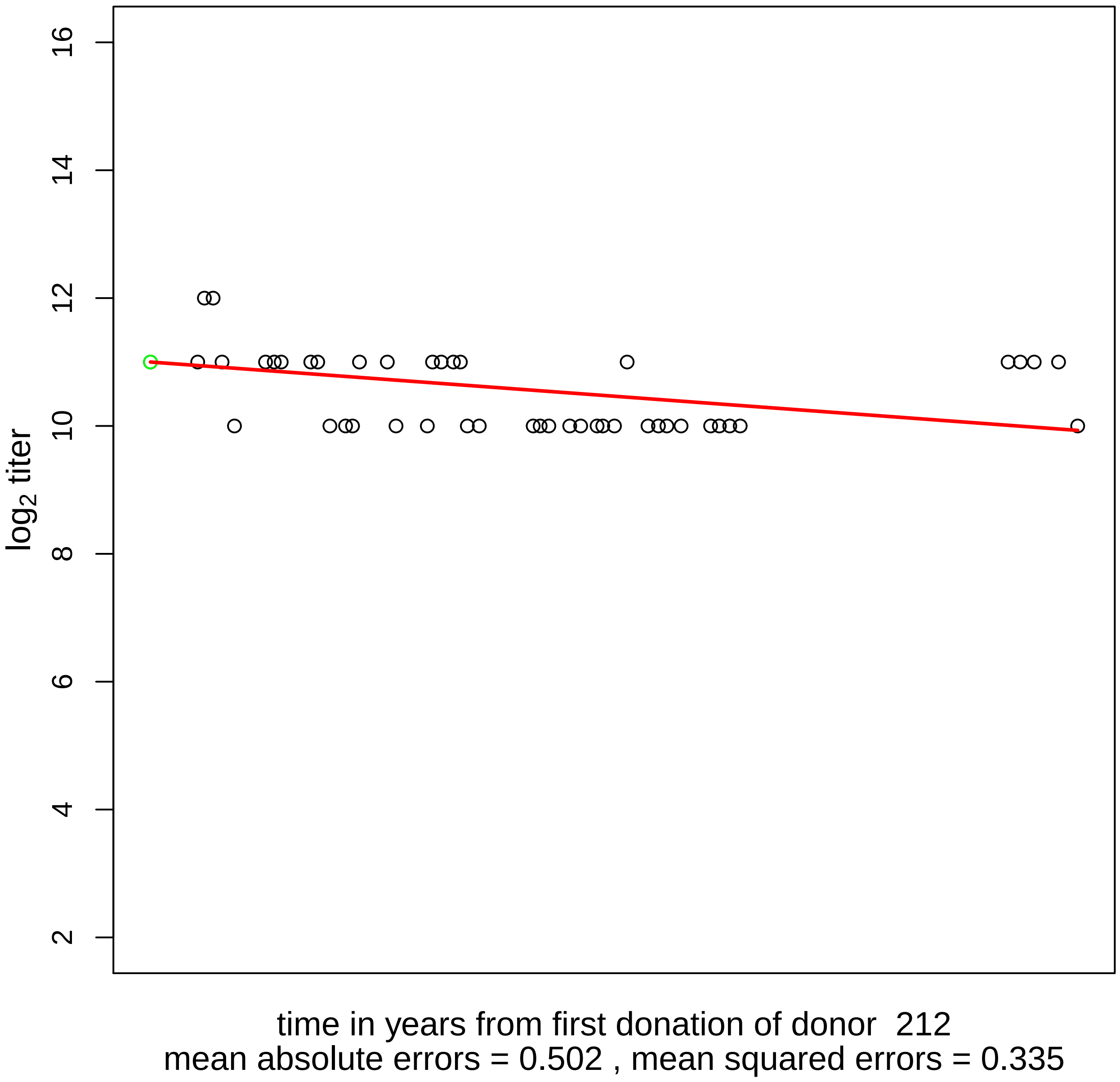

2
4
6
8
10
12
14
16
log2 titer
time in years from first donation of donor 212
mean absolute errors = 0.502 , mean squared errors = 0.335

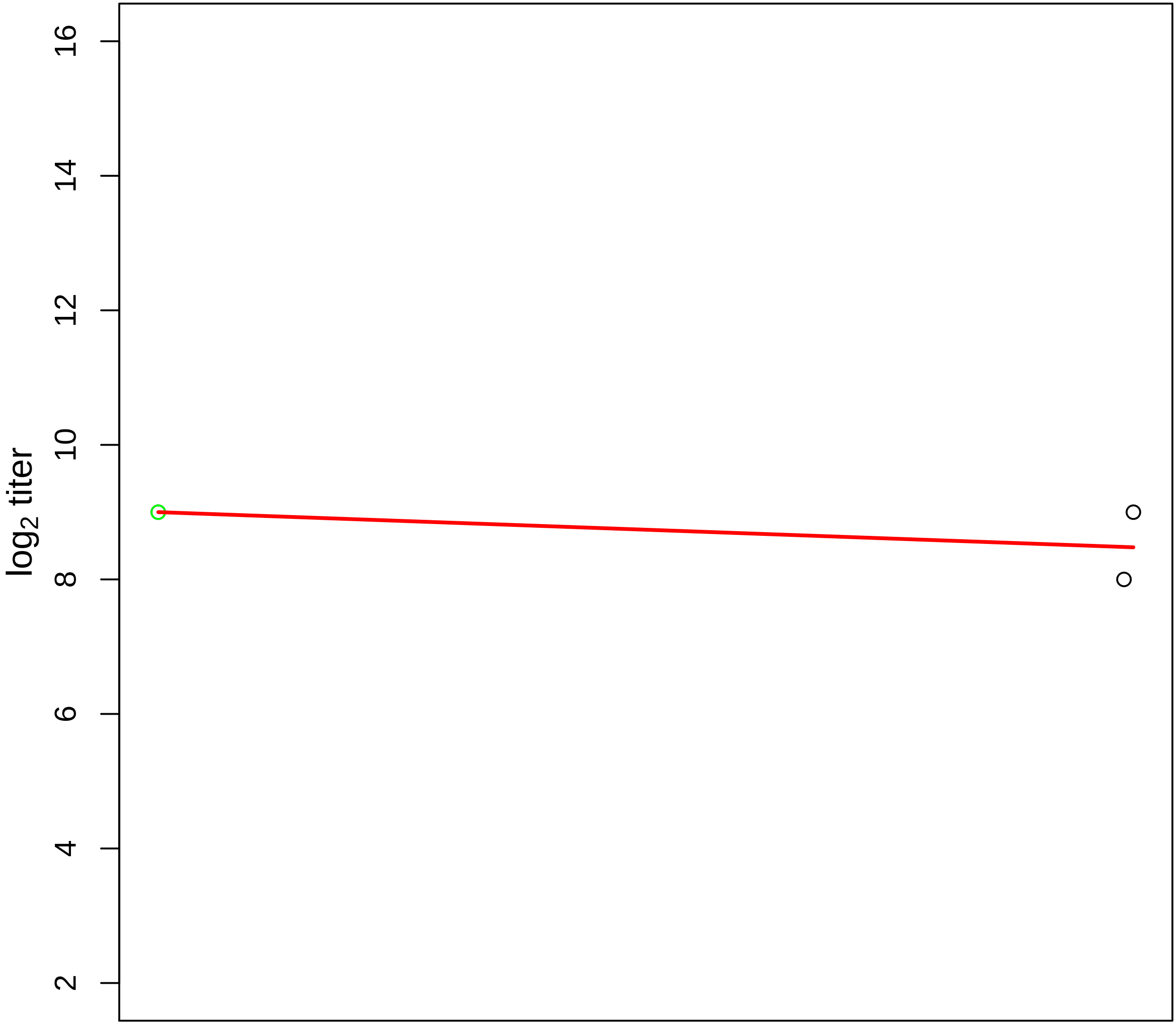

time in years from first donation of donor 213
mean absolute errors = 0.503 , mean squared errors = 0.253

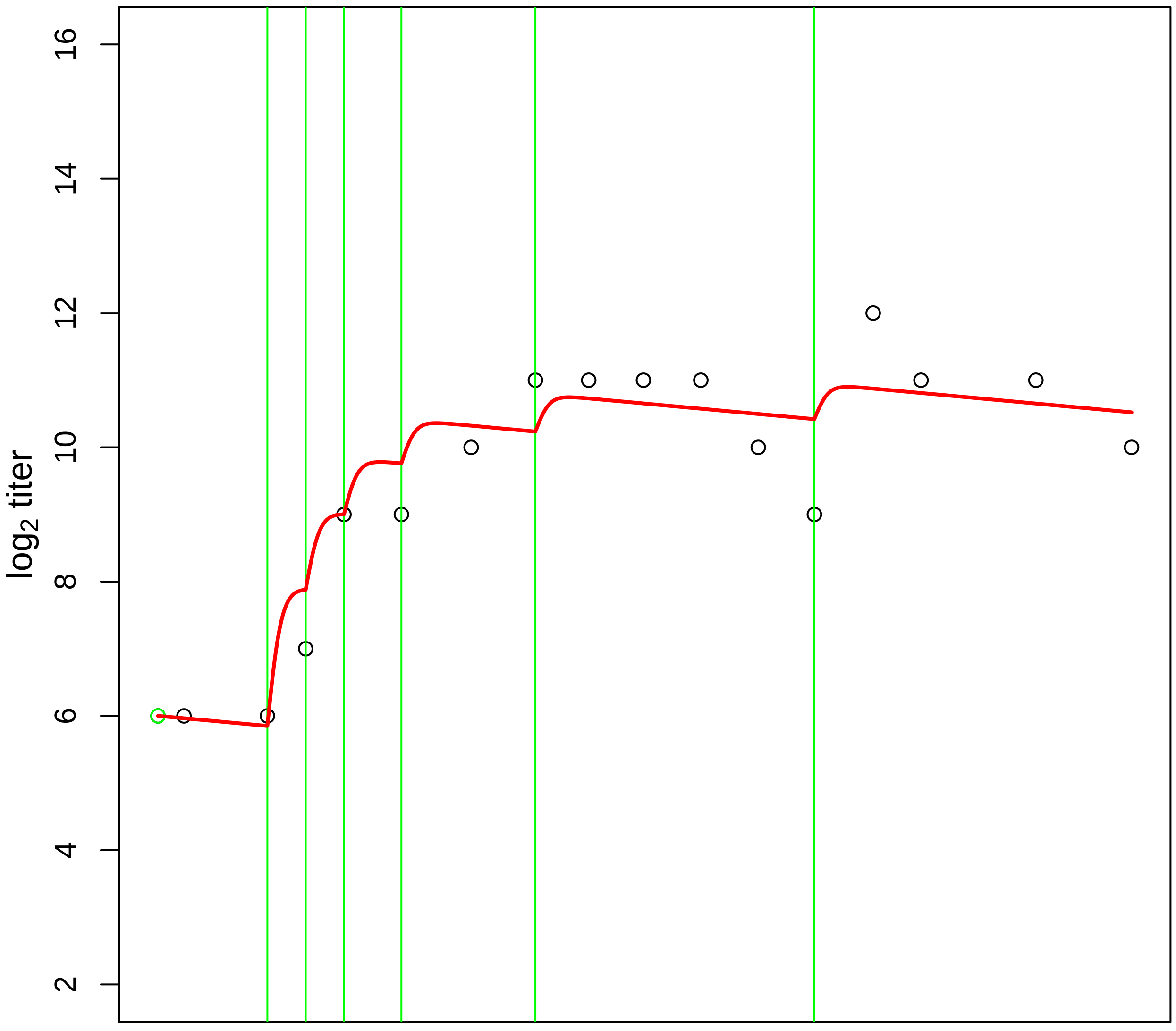

time in years from first donation of donor 214
mean absolute errors = 0.504 , mean squared errors = 0.4

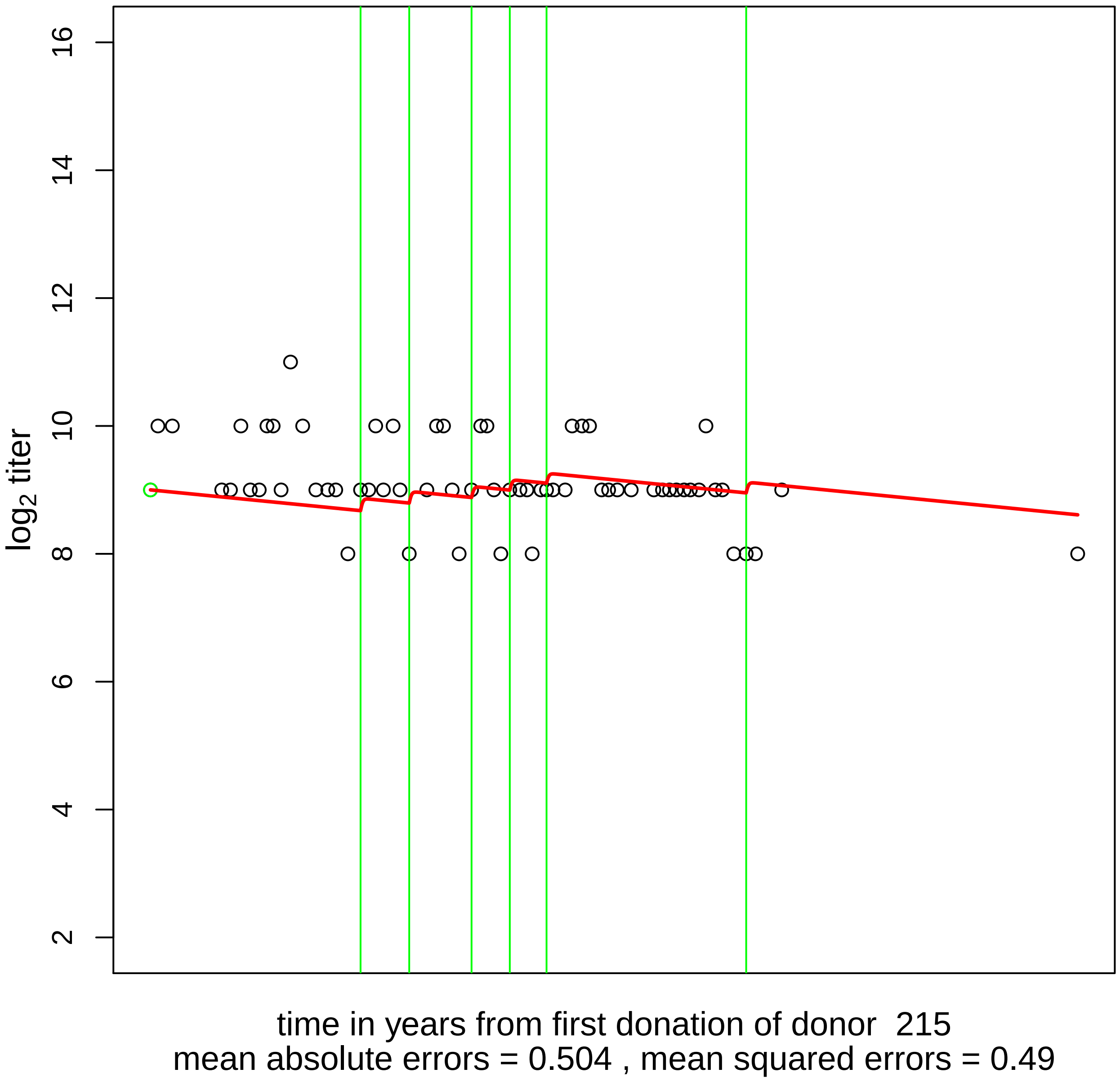

16
14
12
10
8
6
4
2
$\log_2$ titer
time in years from first donation of donor 215
mean absolute errors = 0.504 , mean squared errors = 0.49

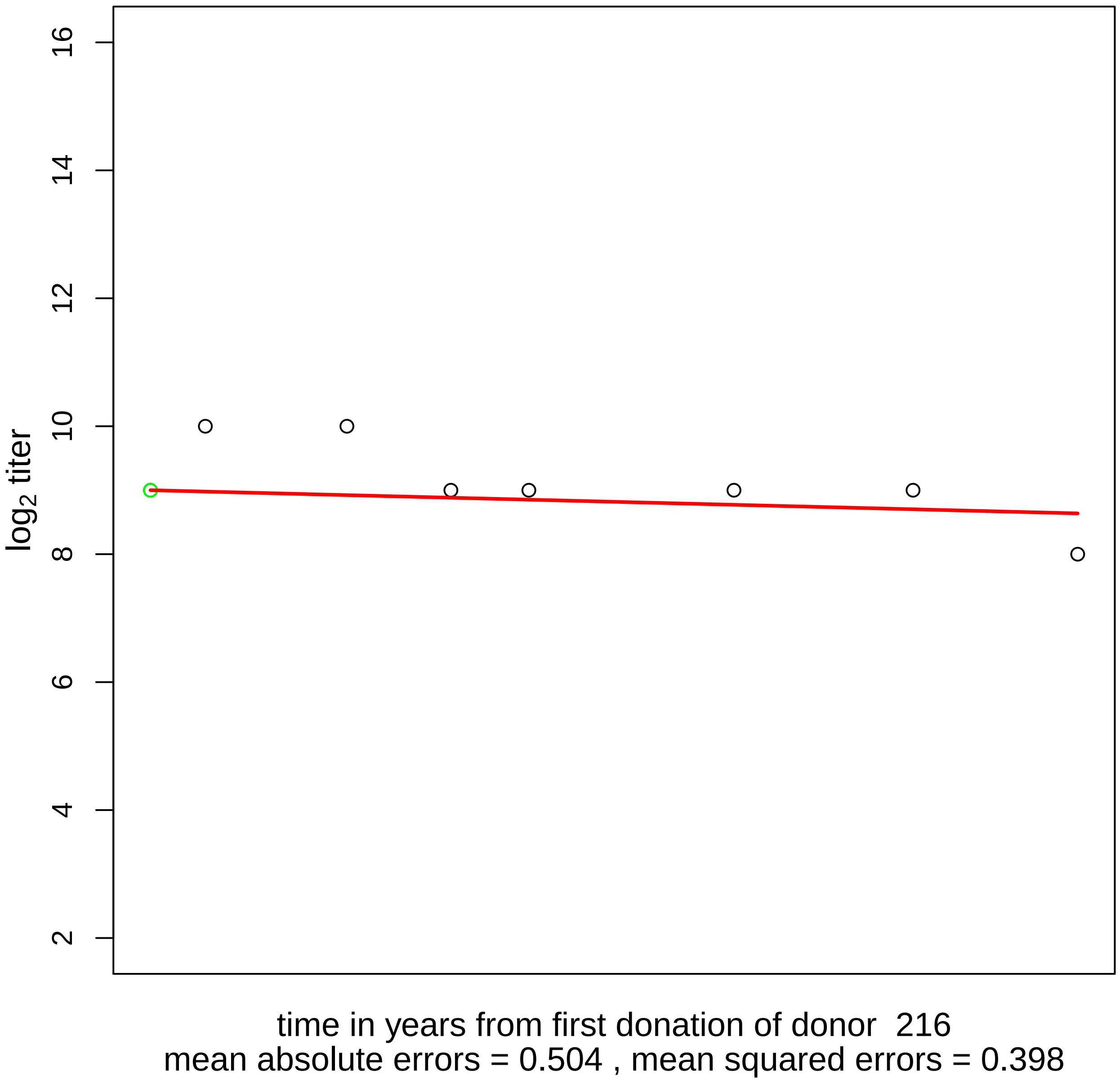

16
14
12
10
8
6
4
2
log_2 titer
time in years from first donation of donor 216
mean absolute errors = 0.504 , mean squared errors = 0.398

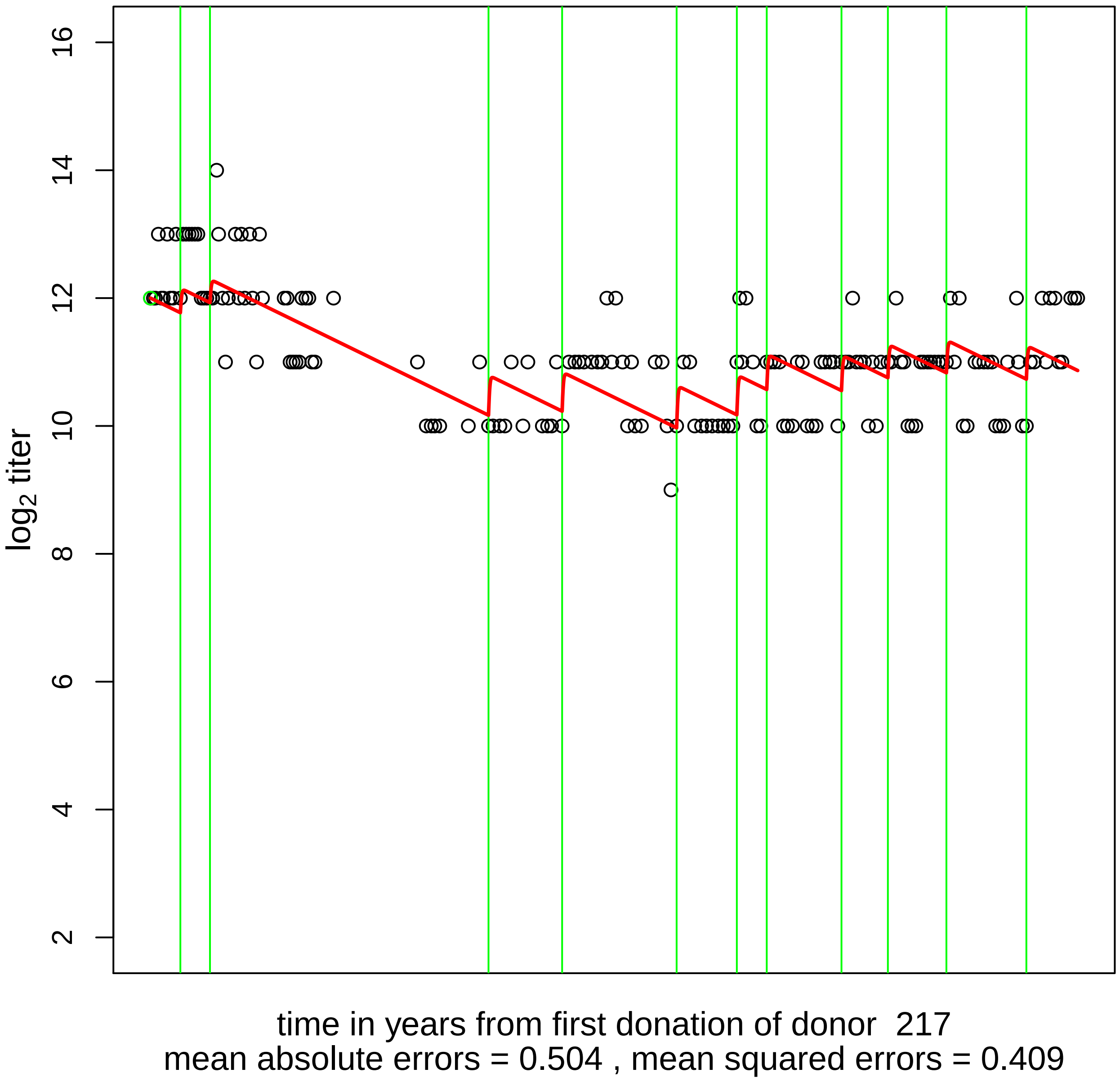

16
14
12
10
8
6
4
2
log_2 titer
time in years from first donation of donor 217
mean absolute errors = 0.504 , mean squared errors = 0.409

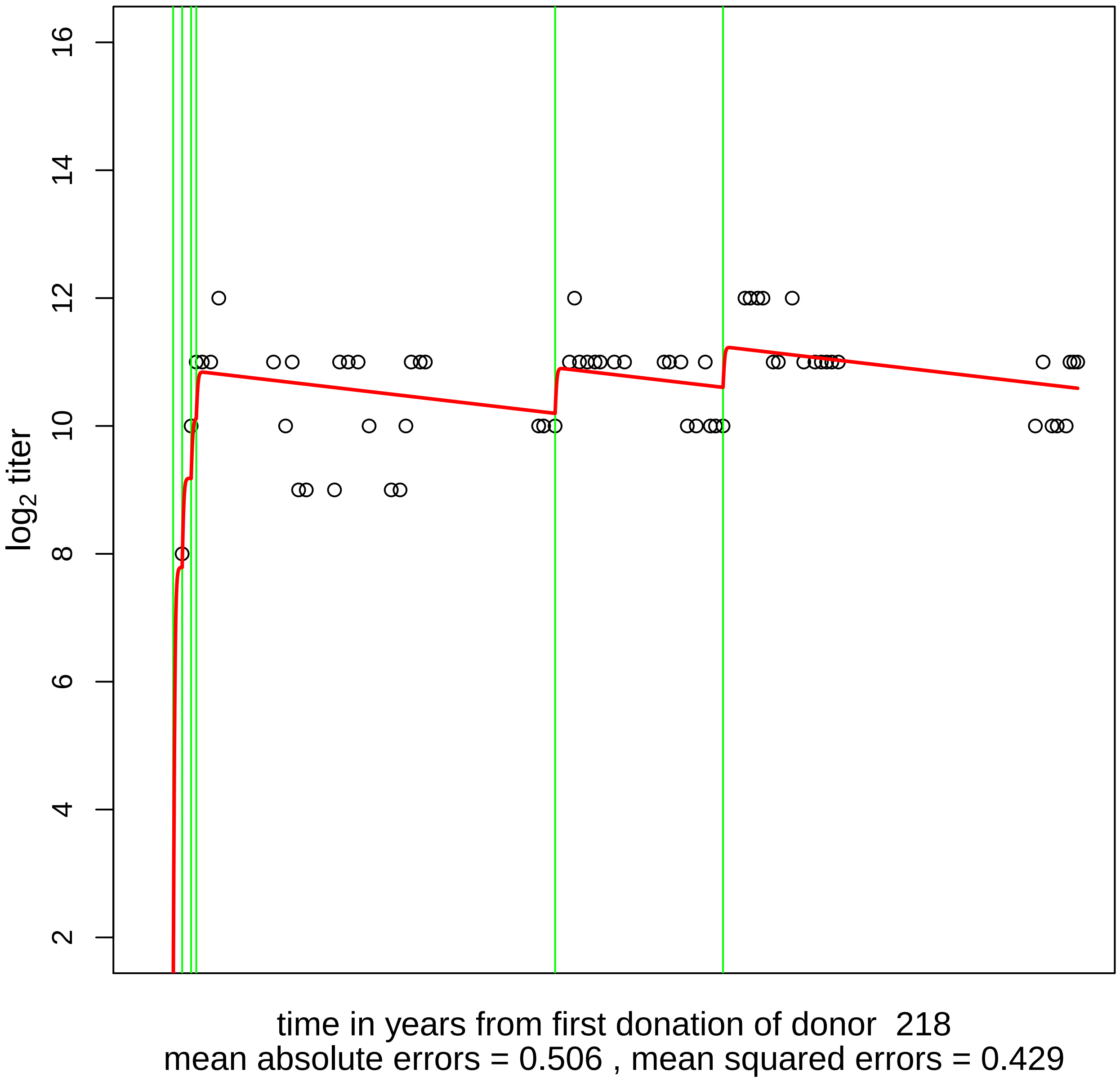
16
14
12
10
8
6
4
2
$\log_2$ titer
time in years from first donation of donor 218
mean absolute errors = 0.506 , mean squared errors = 0.429

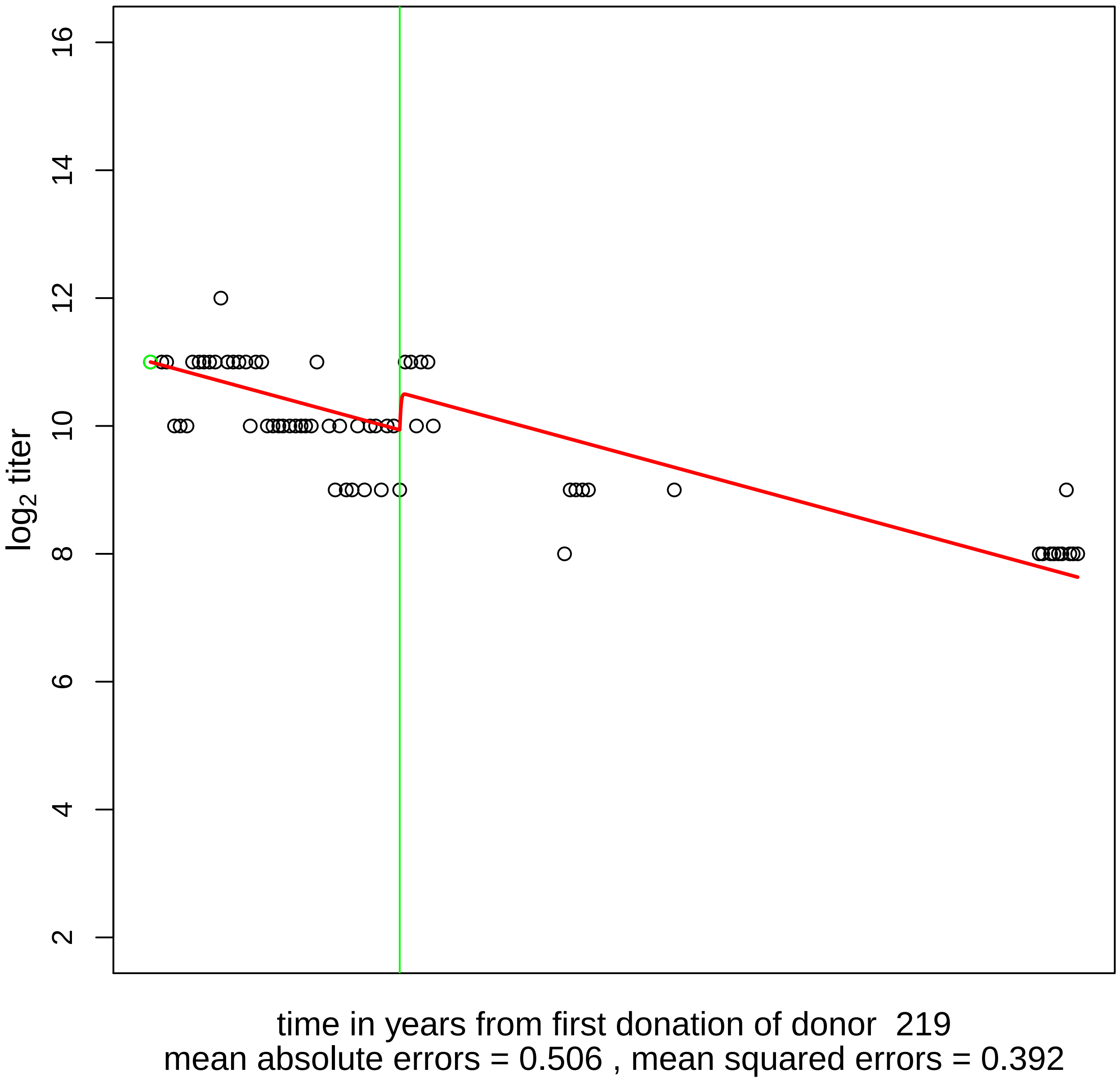

16
14
12
10
8
6
4
2
log2 titer
time in years from first donation of donor 219
mean absolute errors = 0.506 , mean squared errors = 0.392

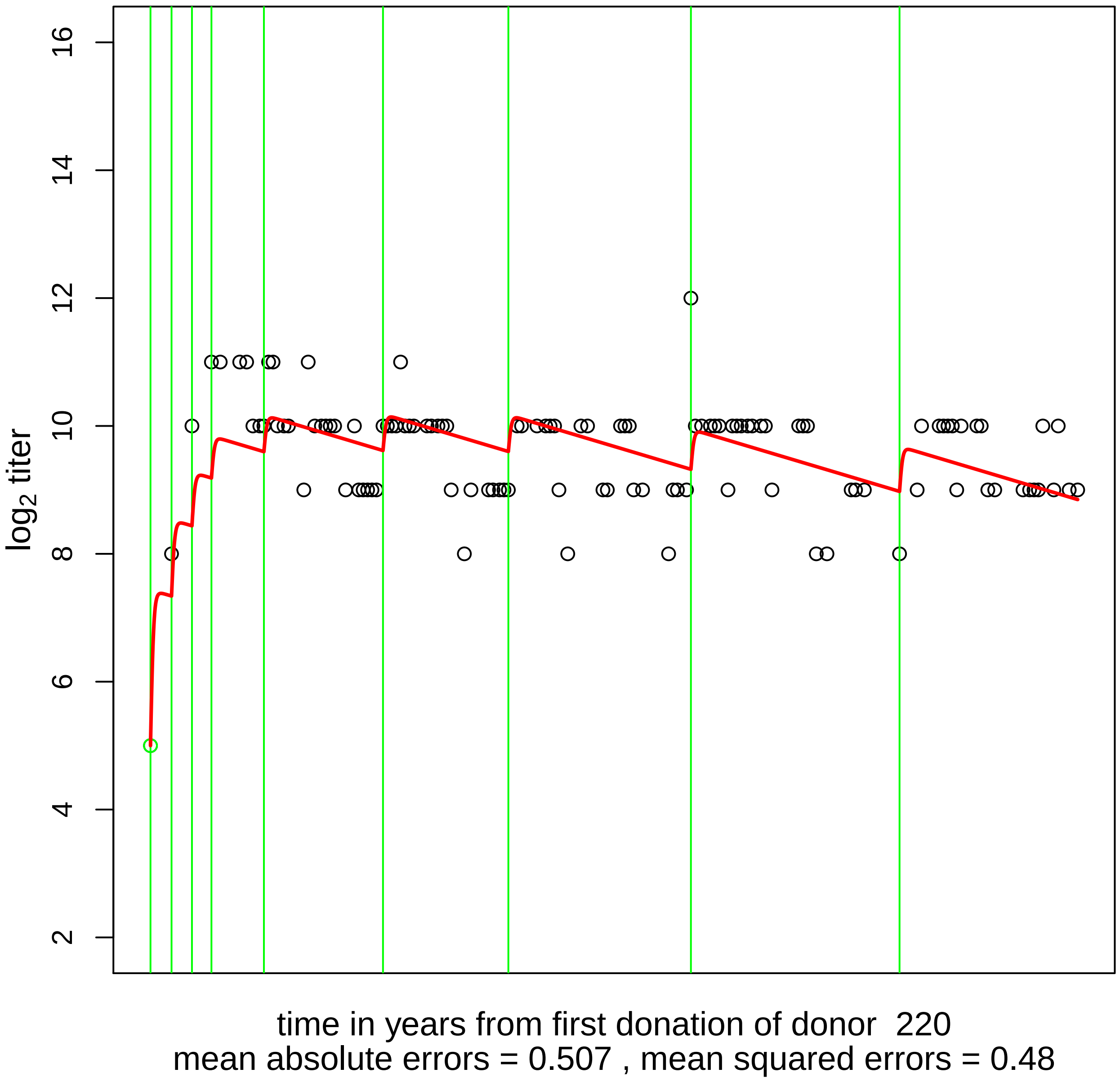

16
14
12
10
8
6
4
2
log2 titer
time in years from first donation of donor 220
mean absolute errors = 0.507 , mean squared errors = 0.48

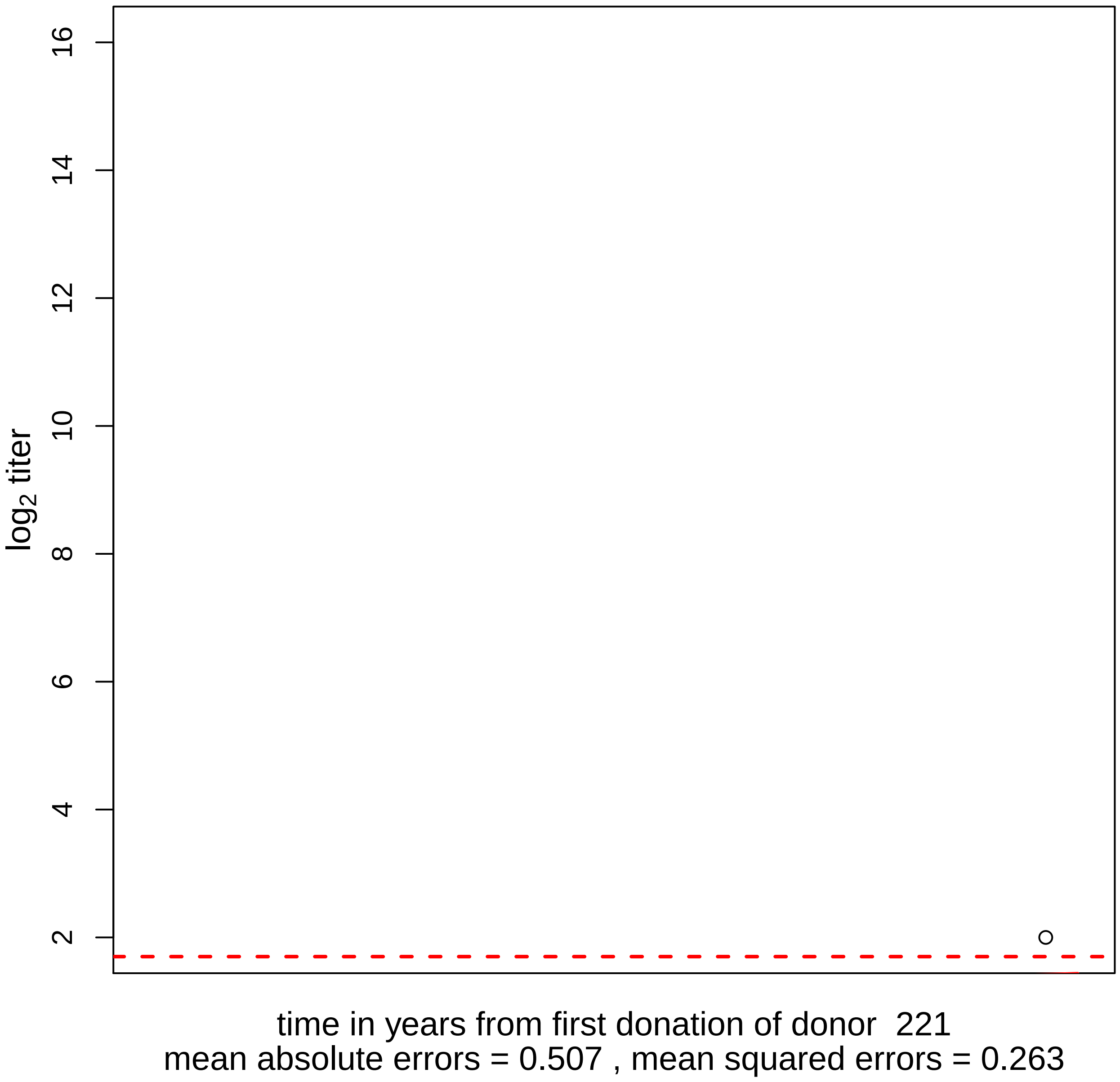

16
14
12
10
8
6
4
2
log2 titer
time in years from first donation of donor 221
mean absolute errors = 0.507 , mean squared errors = 0.263

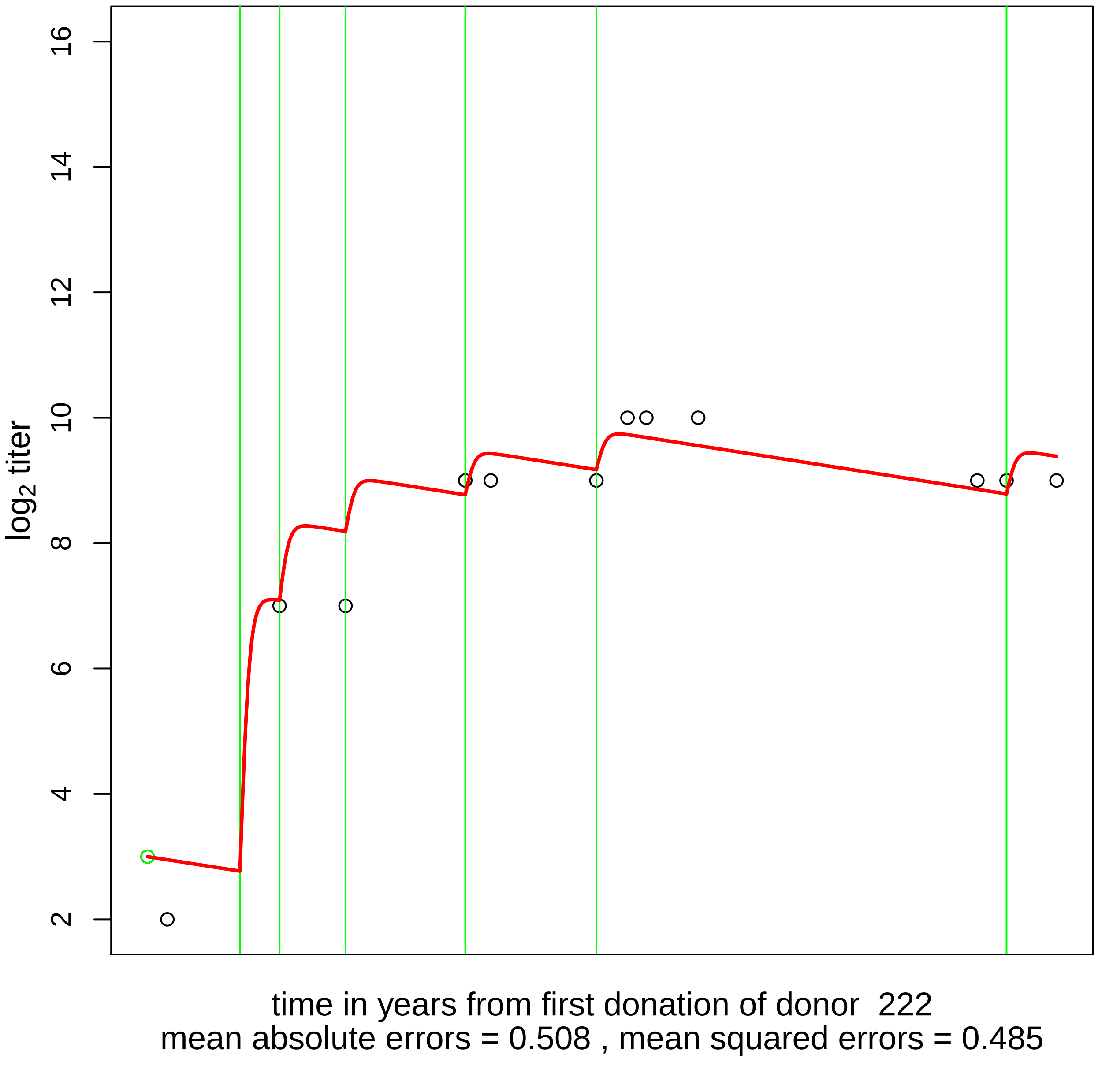

16
14
12
10
8
6
4
2
log2 titer
time in years from first donation of donor 222
mean absolute errors = 0.508 , mean squared errors = 0.485

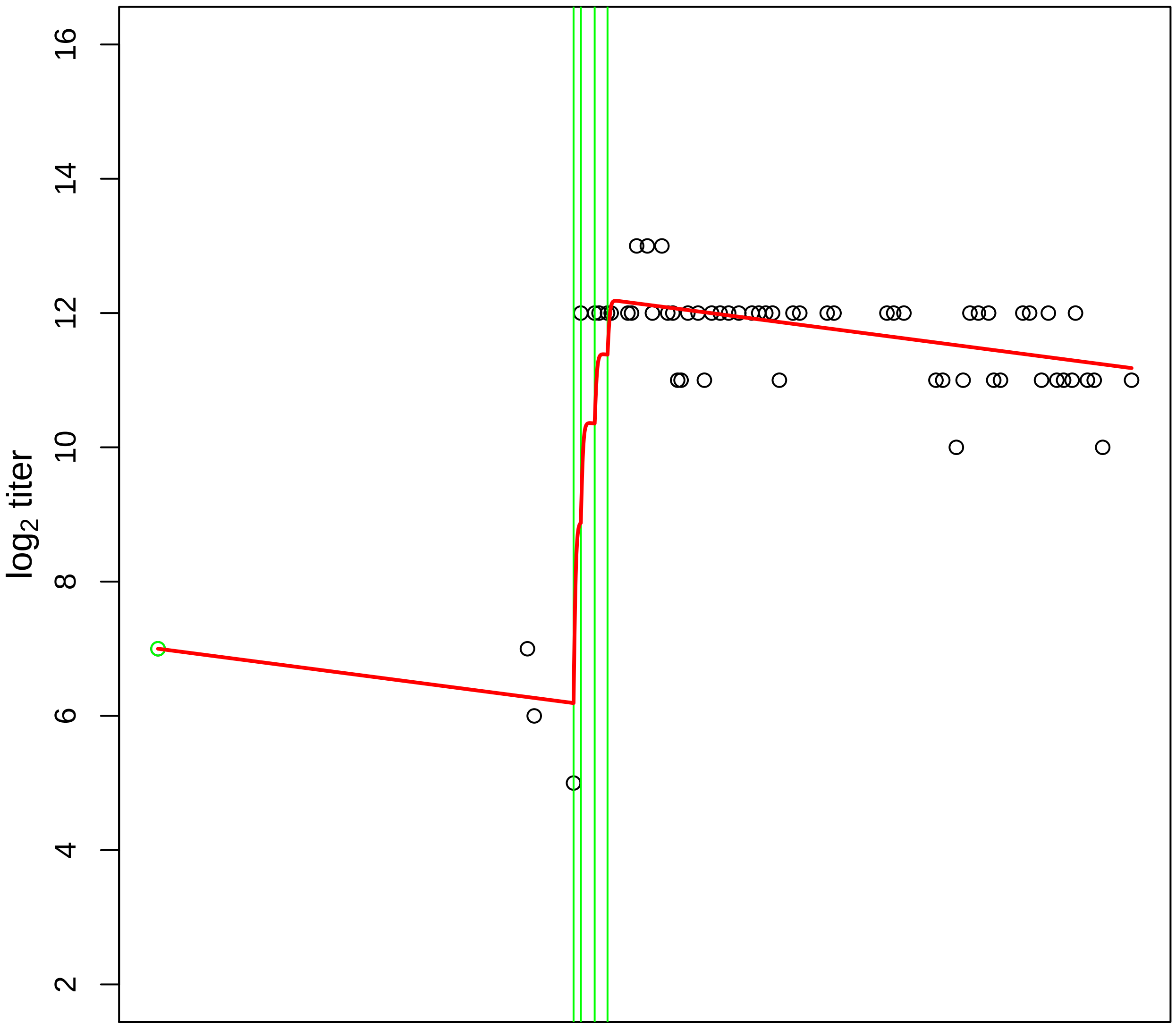

time in years from first donation of donor 223
mean absolute errors = 0.508 , mean squared errors = 0.527

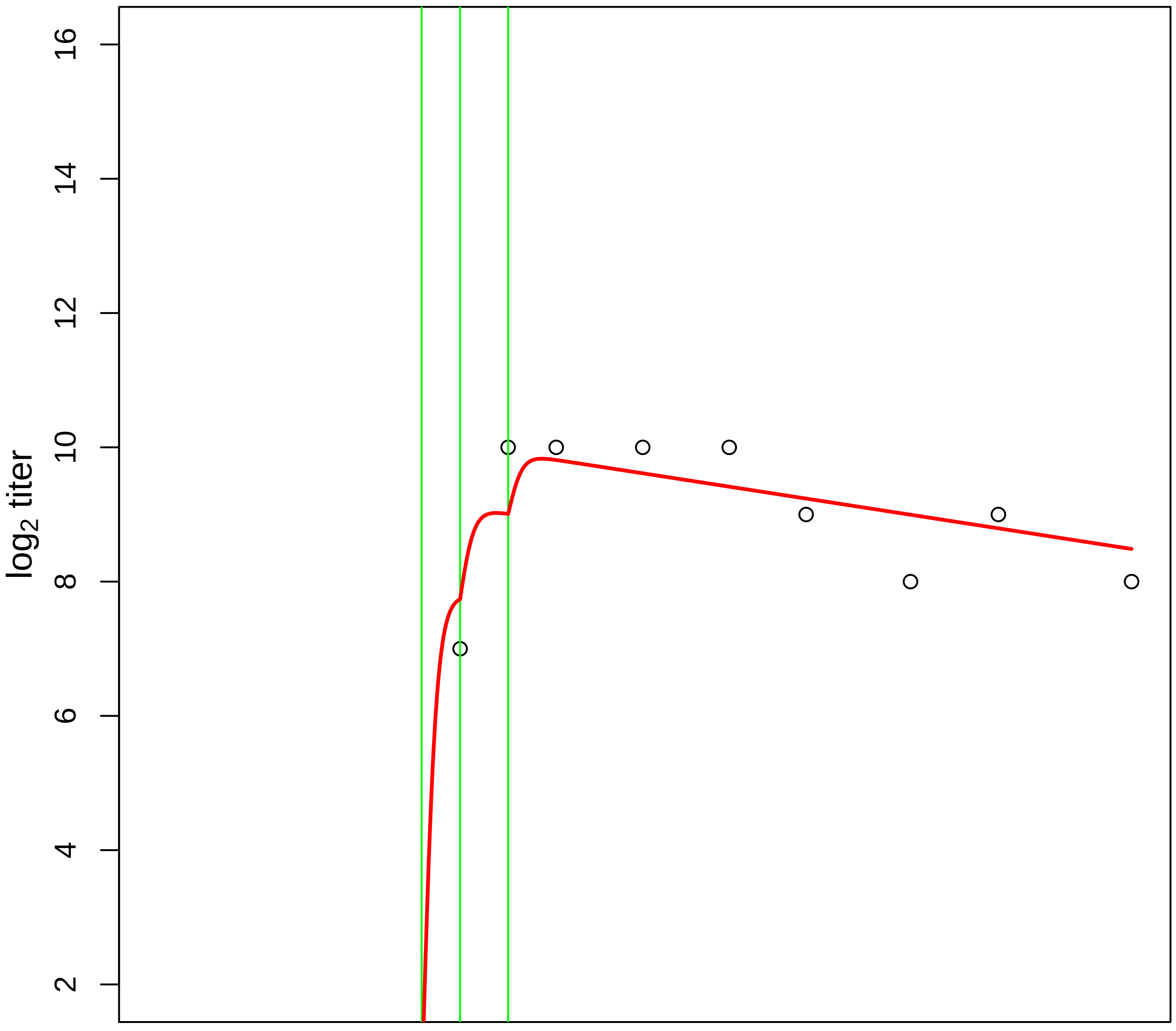

time in years from first donation of donor 224
mean absolute errors = 0.508 , mean squared errors = 0.343

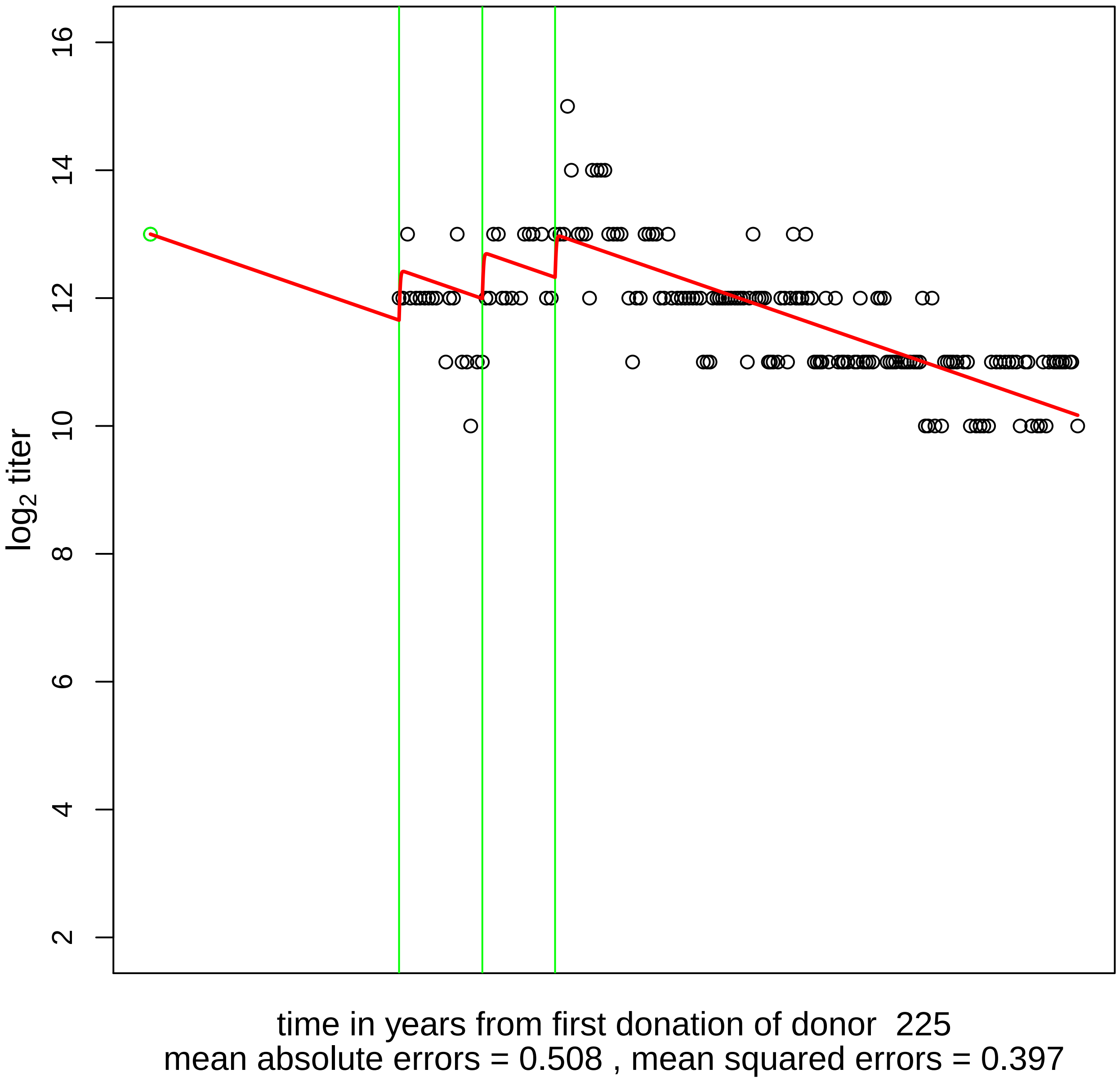

16
14
12
10
8
6
4
2
log2 titer
time in years from first donation of donor 225
mean absolute errors = 0.508 , mean squared errors = 0.397

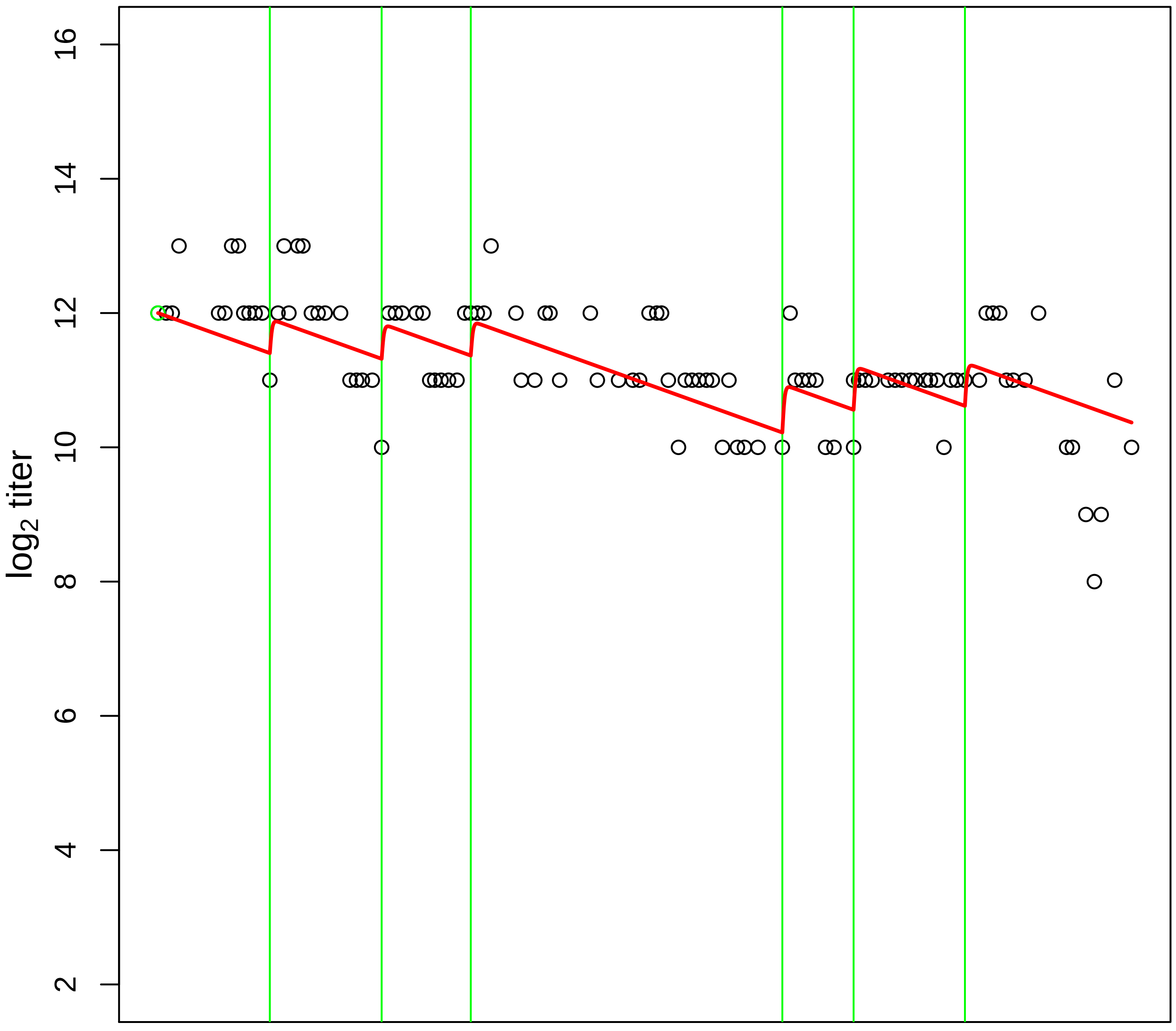

time in years from first donation of donor 226
mean absolute errors = 0.508 , mean squared errors = 0.439

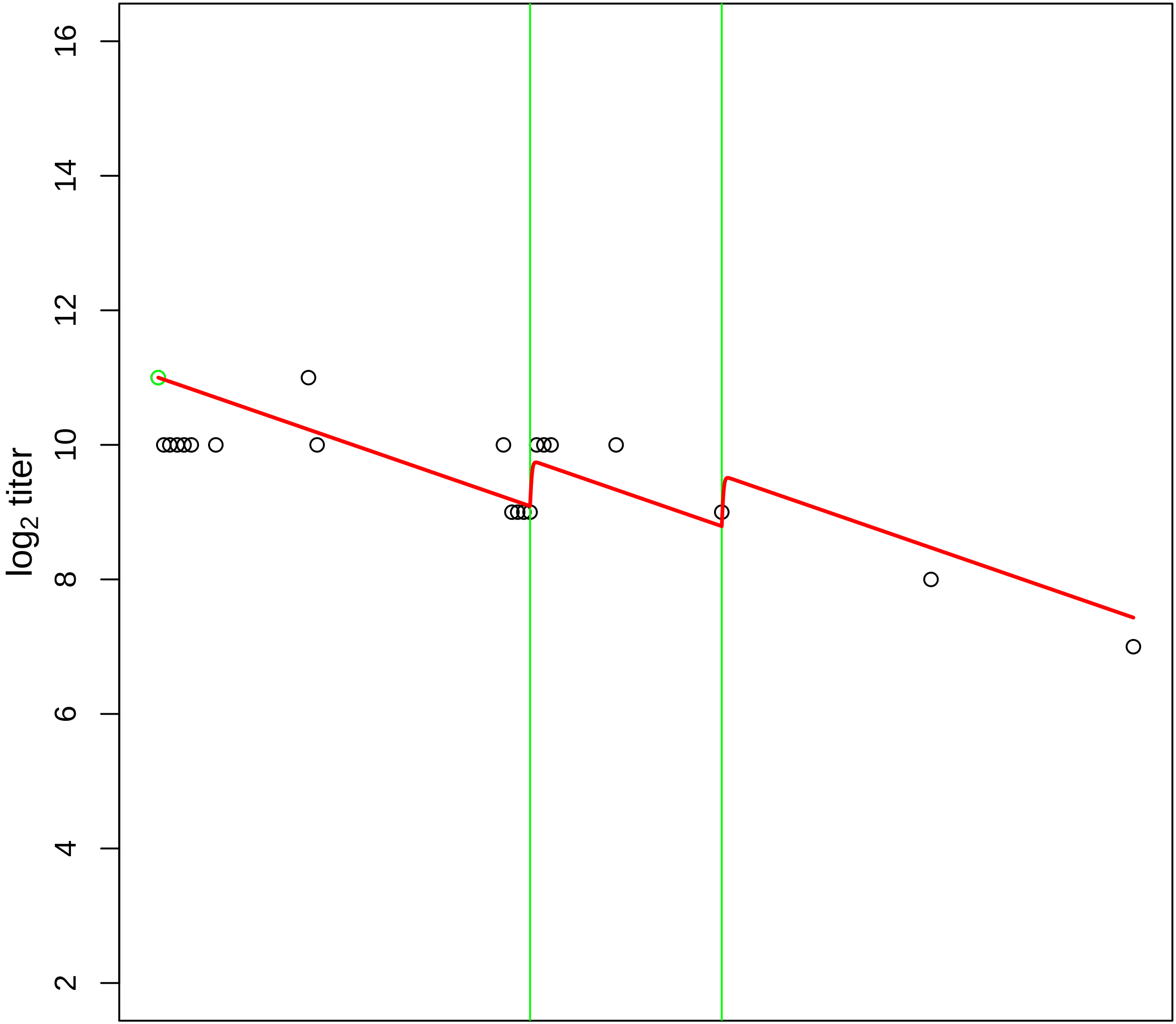

time in years from first donation of donor 227
mean absolute errors = 0.508 , mean squared errors = 0.353

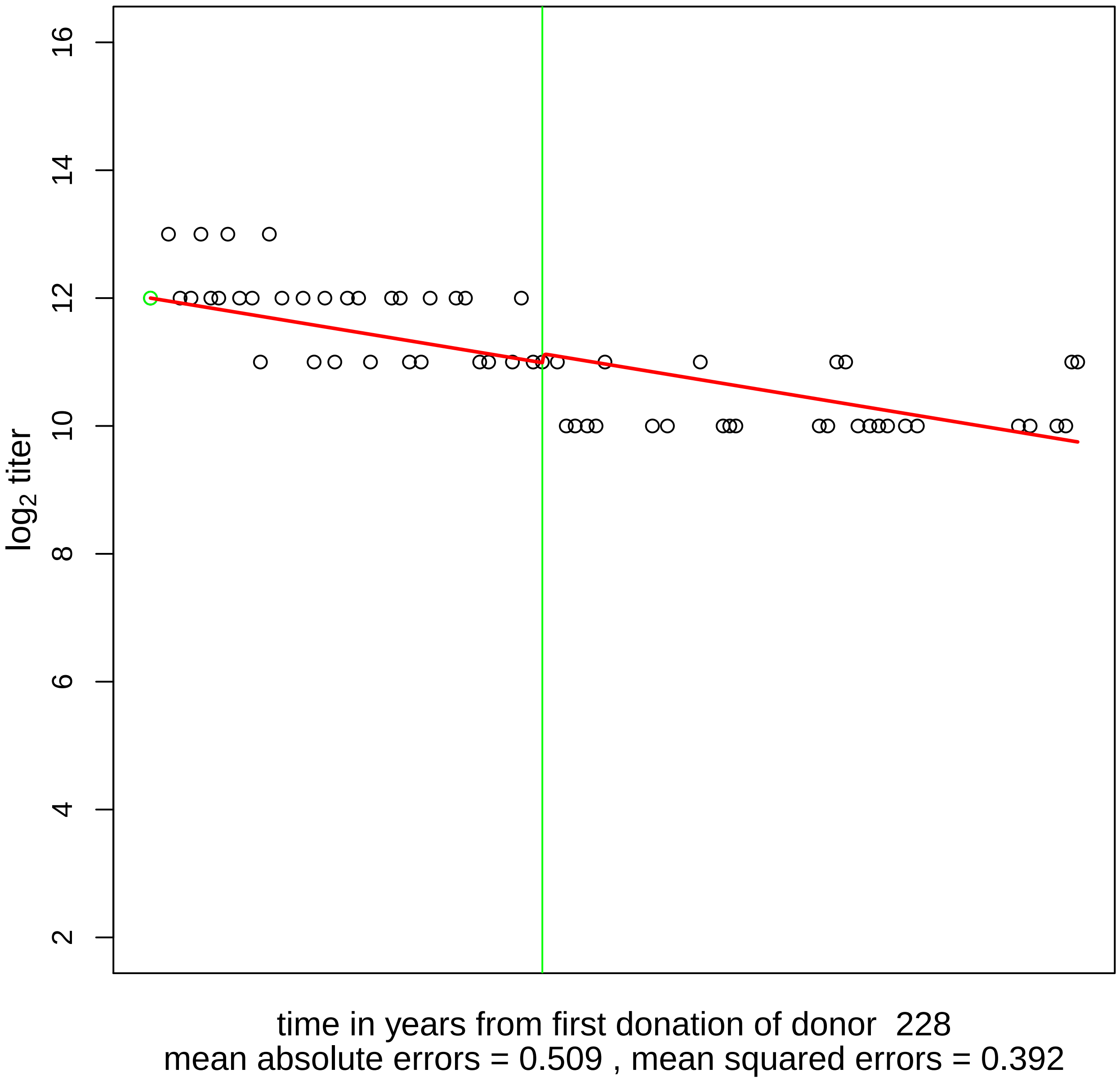

16
14
12
10
8
6
4
2
log2 titer
time in years from first donation of donor 228
mean absolute errors = 0.509 , mean squared errors = 0.392

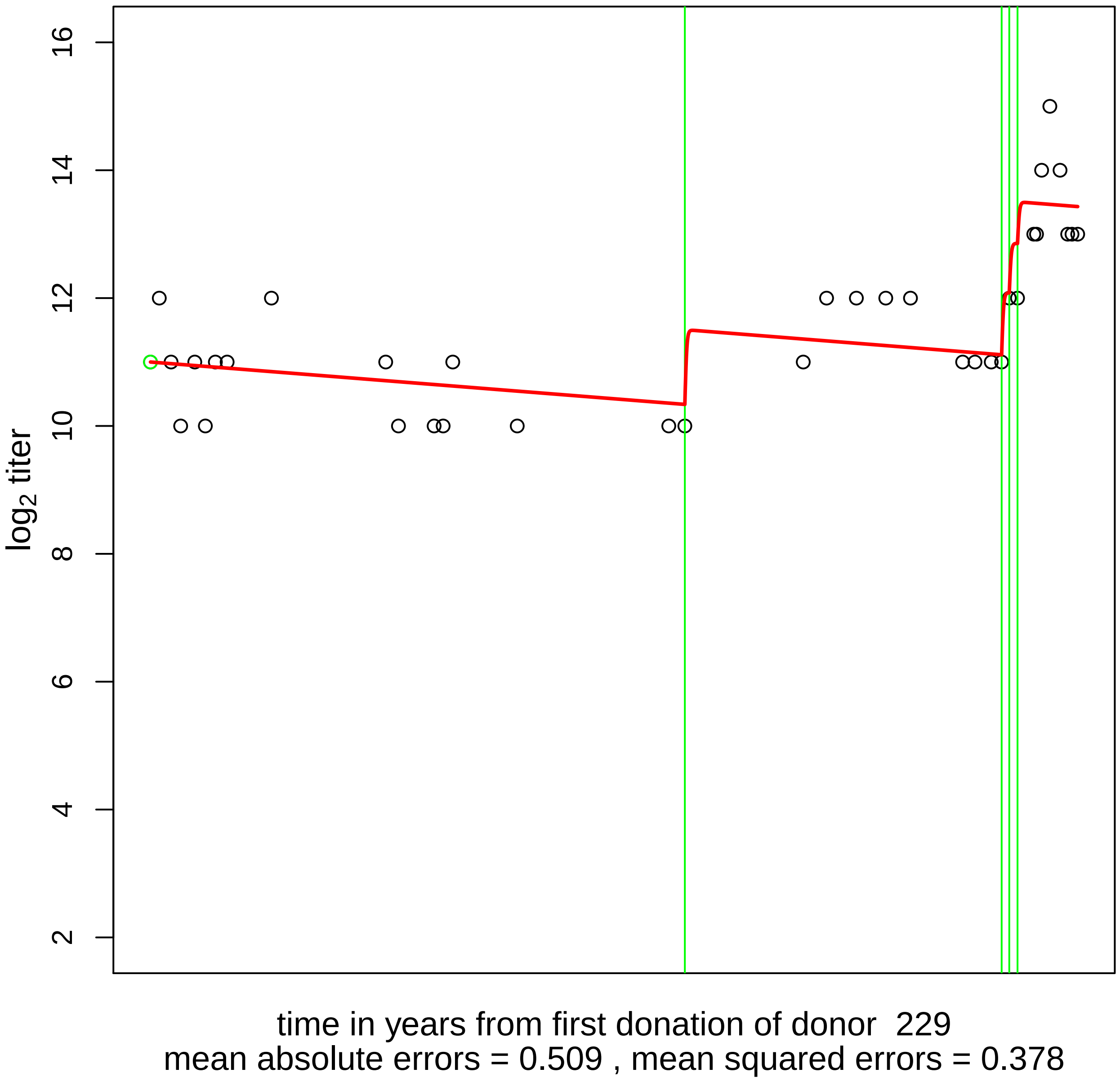

16
14
12
10
8
6
4
2
log2 titer
time in years from first donation of donor 229
mean absolute errors = 0.509 , mean squared errors = 0.378

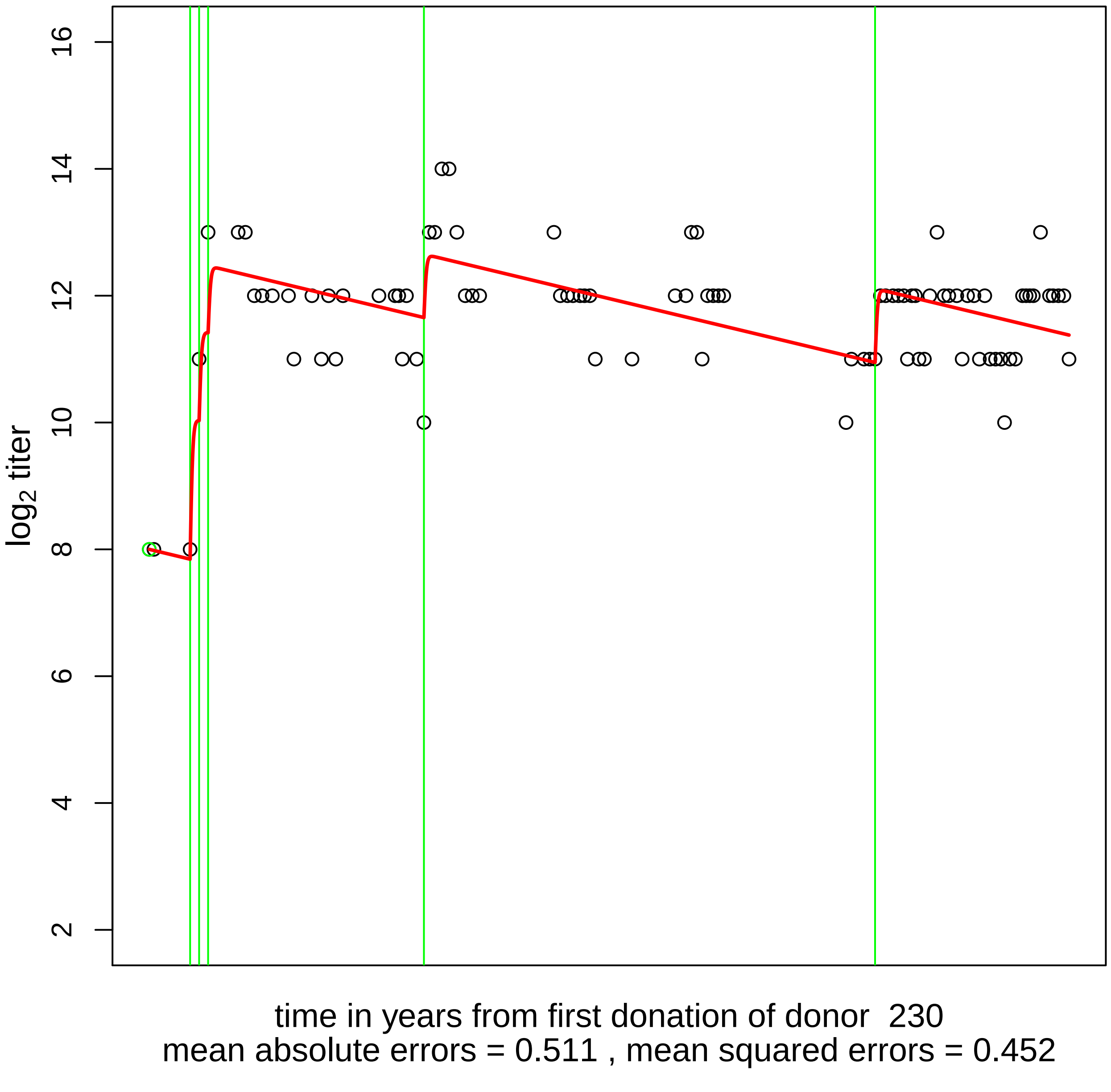

16
14
12
10
8
6
4
2
log2 titer
time in years from first donation of donor 230
mean absolute errors = 0.511 , mean squared errors = 0.452

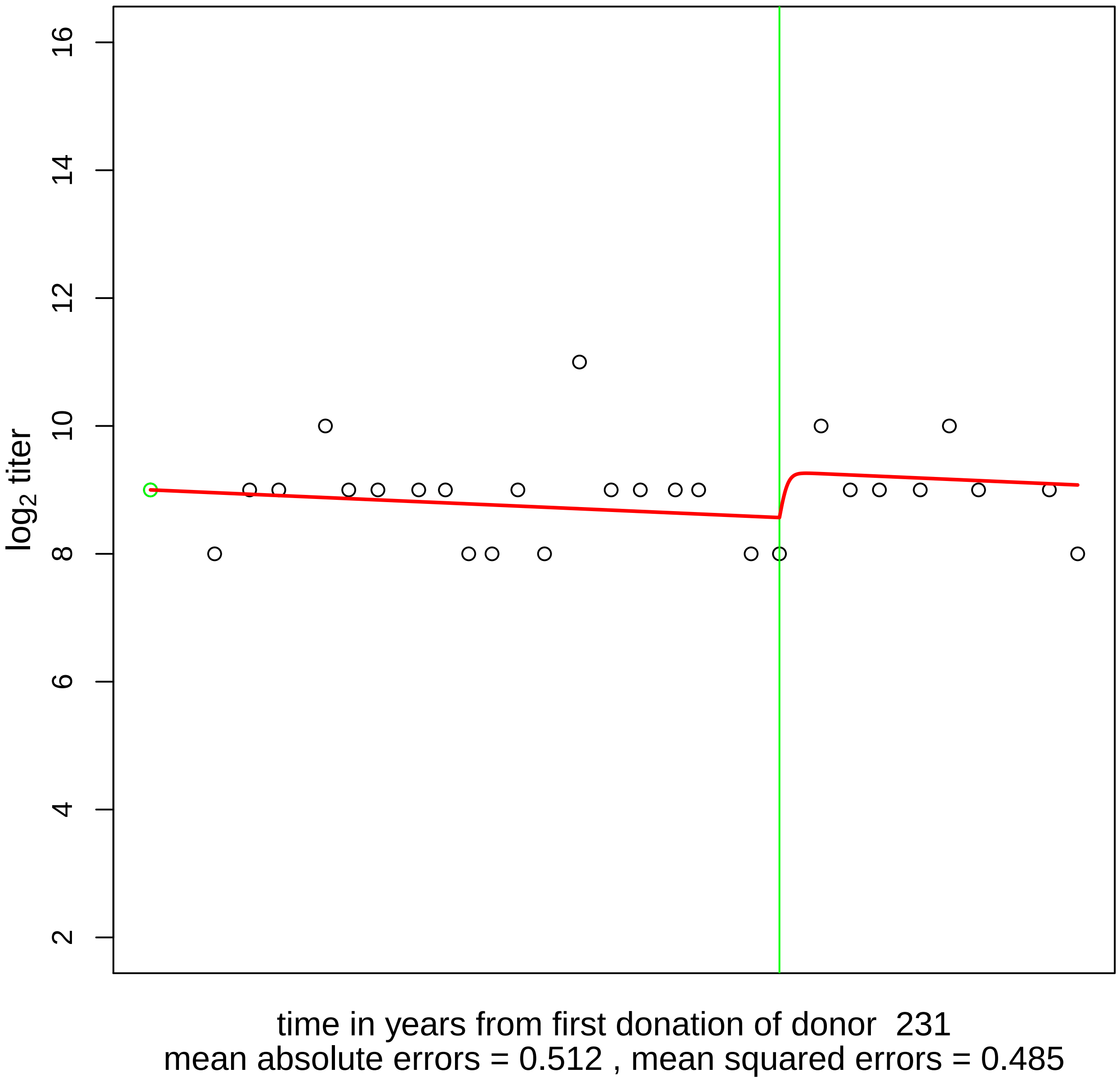

16
14
12
10
8
6
4
2
log2 titer
time in years from first donation of donor 231
mean absolute errors = 0.512 , mean squared errors = 0.485

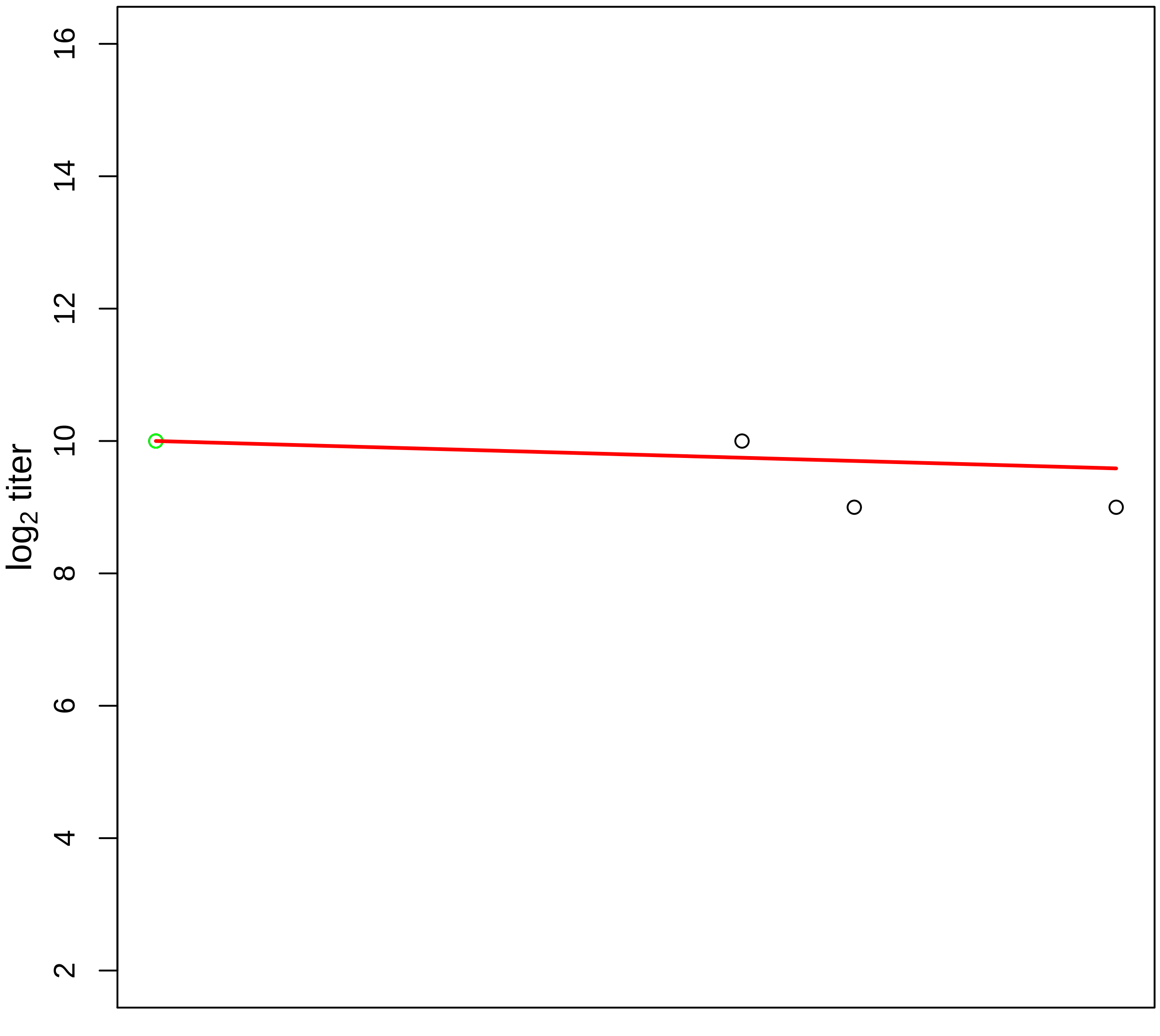

time in years from first donation of donor 232
mean absolute errors = 0.513 , mean squared errors = 0.299

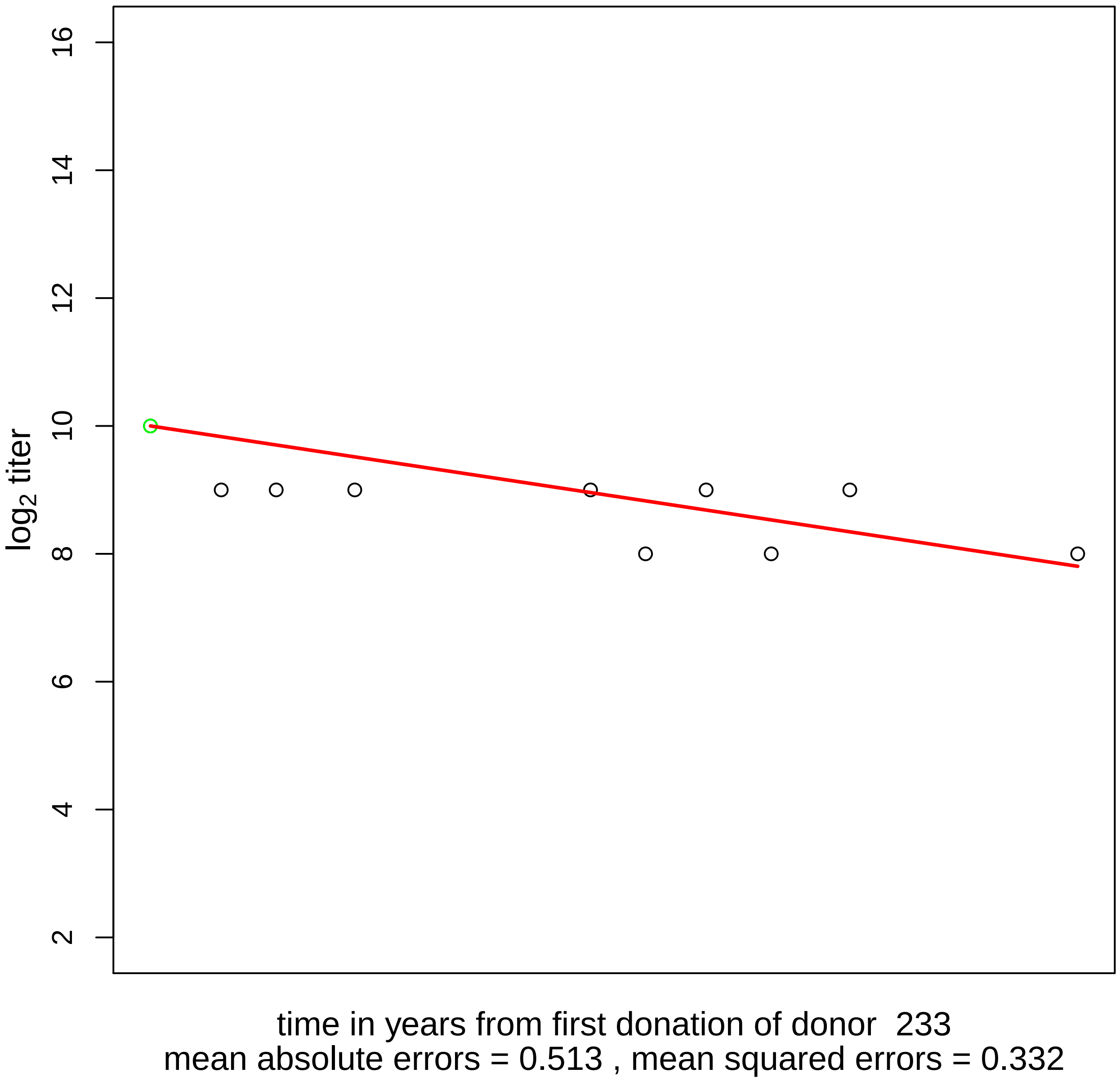

16
14
12
10
8
6
4
2
log2 titer
time in years from first donation of donor 233
mean absolute errors = 0.513 , mean squared errors = 0.332

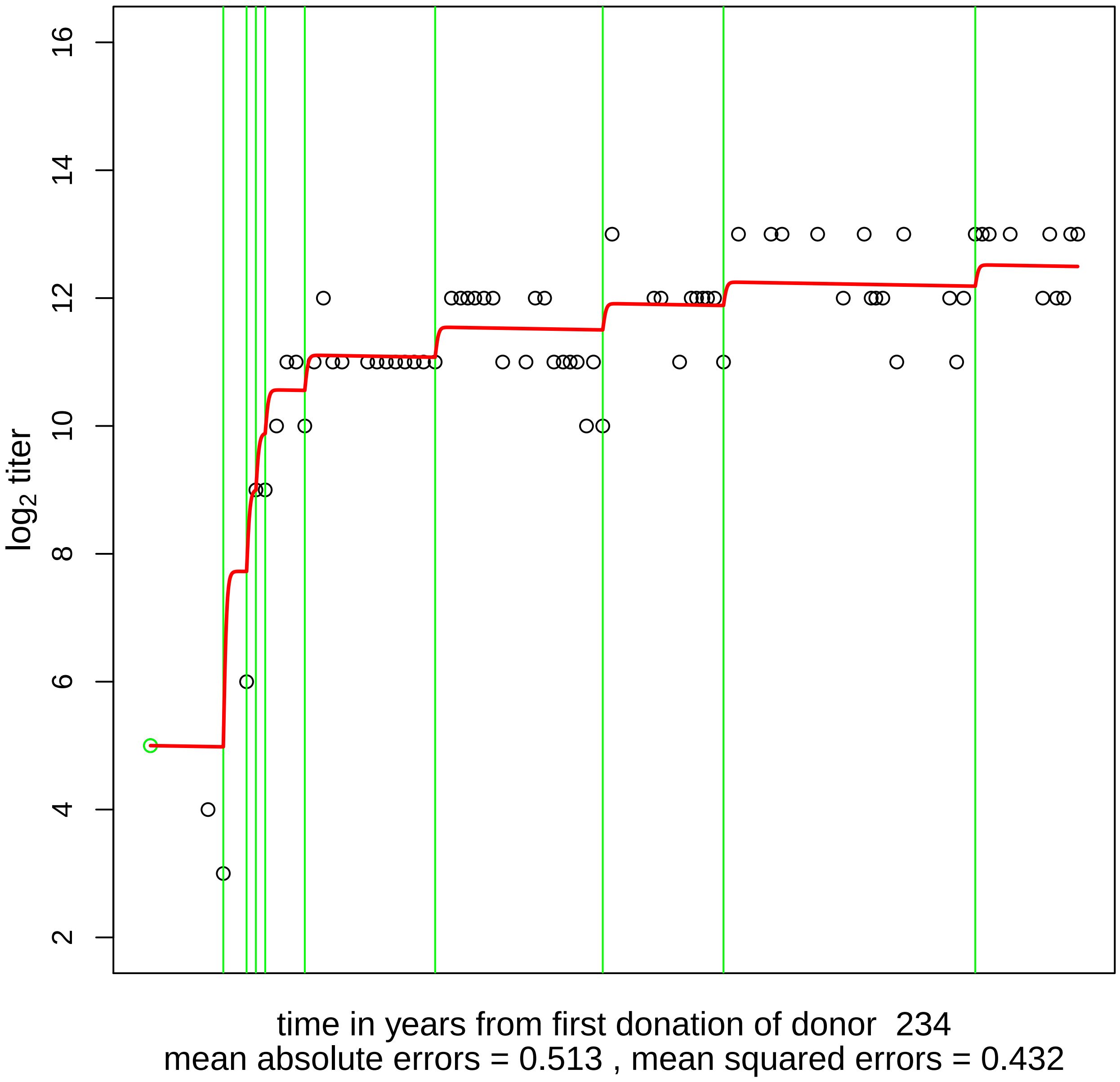

$\log_2$ titer
2
4
6
8
10
12
14
16
time in years from first donation of donor 234
mean absolute errors = 0.513 , mean squared errors = 0.432

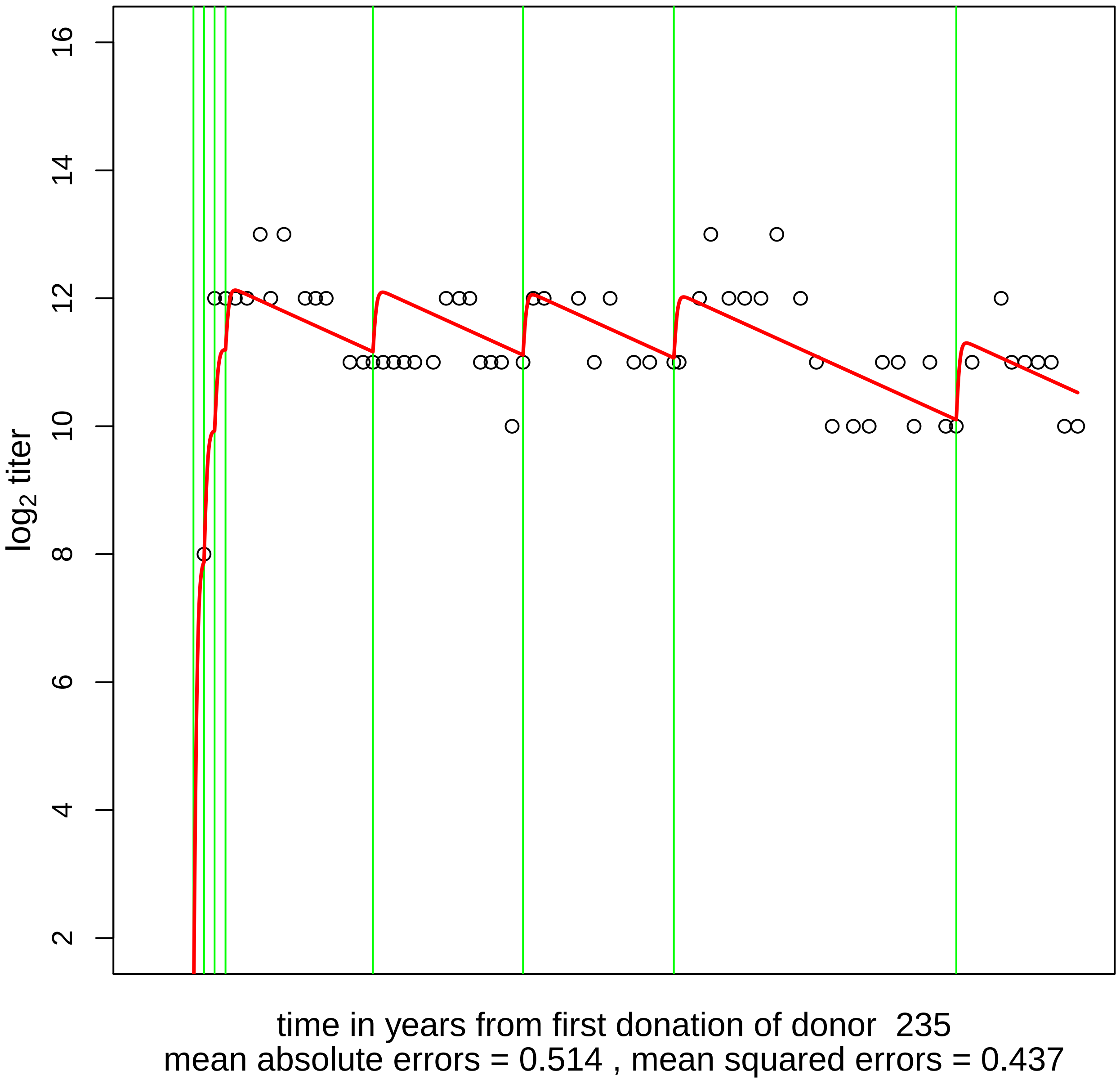

16
14
12
10
8
6
4
2
log2 titer
time in years from first donation of donor 235
mean absolute errors = 0.514 , mean squared errors = 0.437

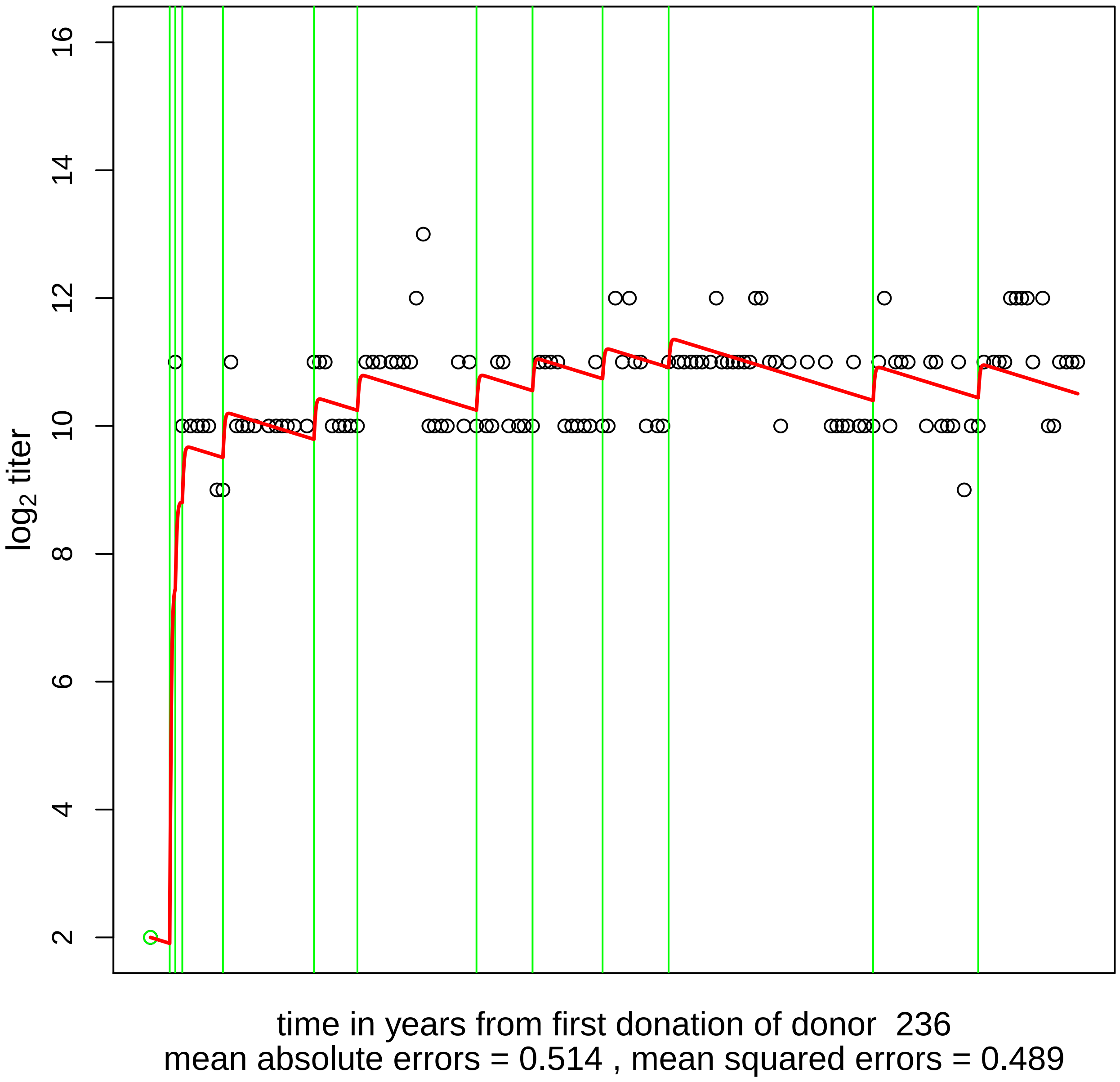

16
14
12
10
8
6
4
2
log2 titer
time in years from first donation of donor 236
mean absolute errors = 0.514 , mean squared errors = 0.489

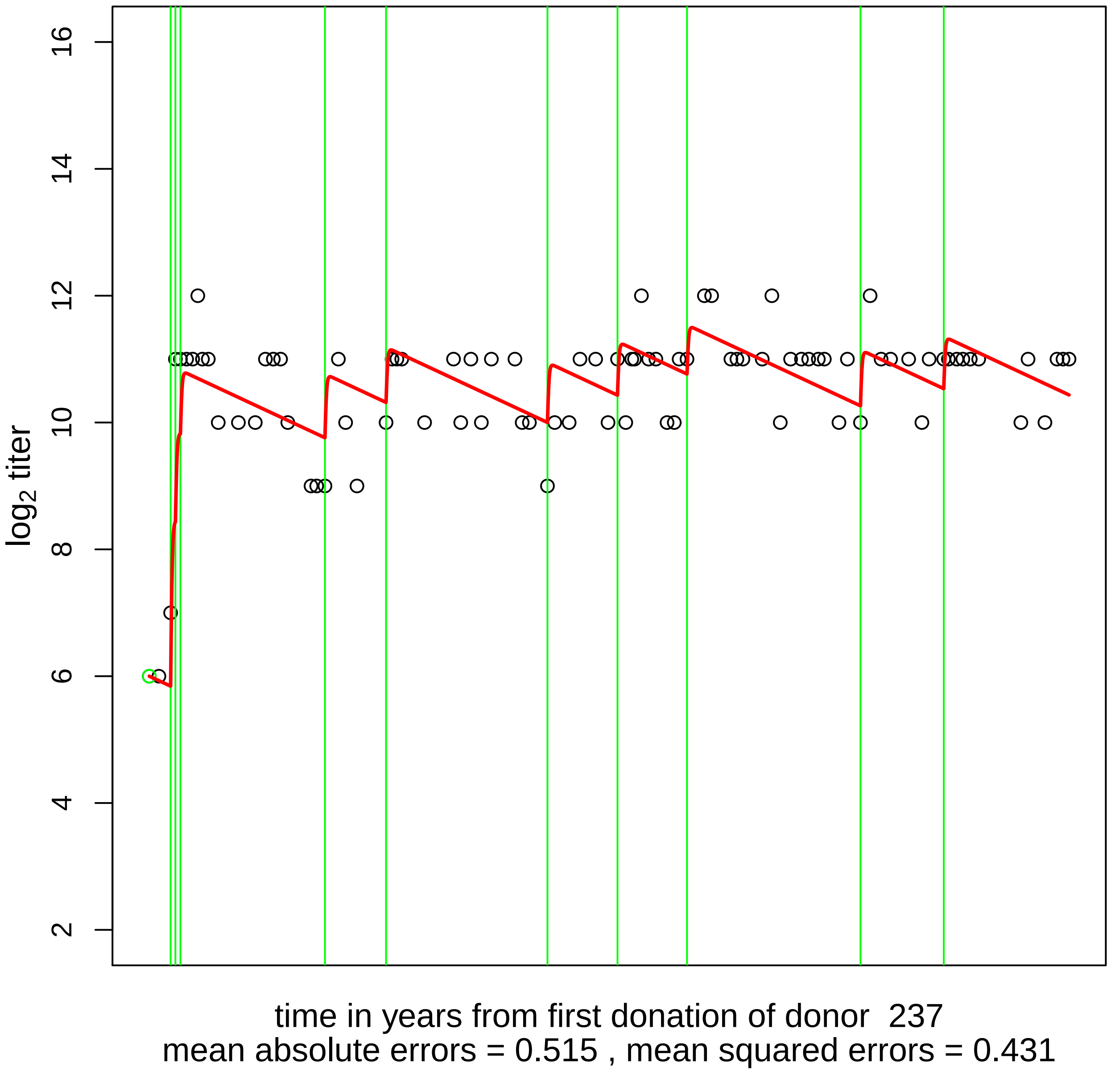

16
14
12
10
8
6
4
2
$\log_2$ titer
time in years from first donation of donor 237
mean absolute errors = 0.515 , mean squared errors = 0.431

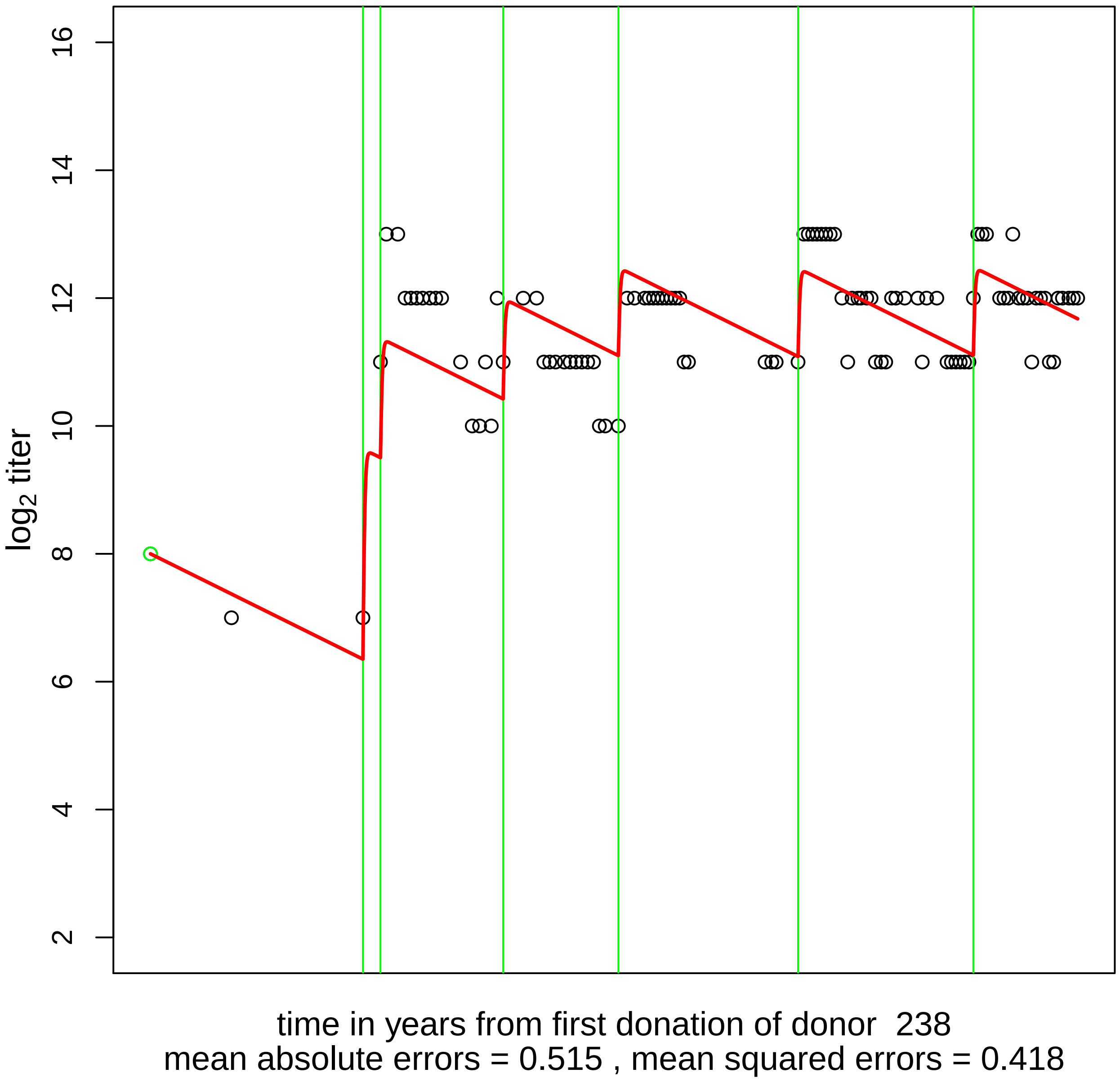

16
14
12
10
8
6
4
2
$\log_2$ titer
time in years from first donation of donor 238
mean absolute errors = 0.515 , mean squared errors = 0.418

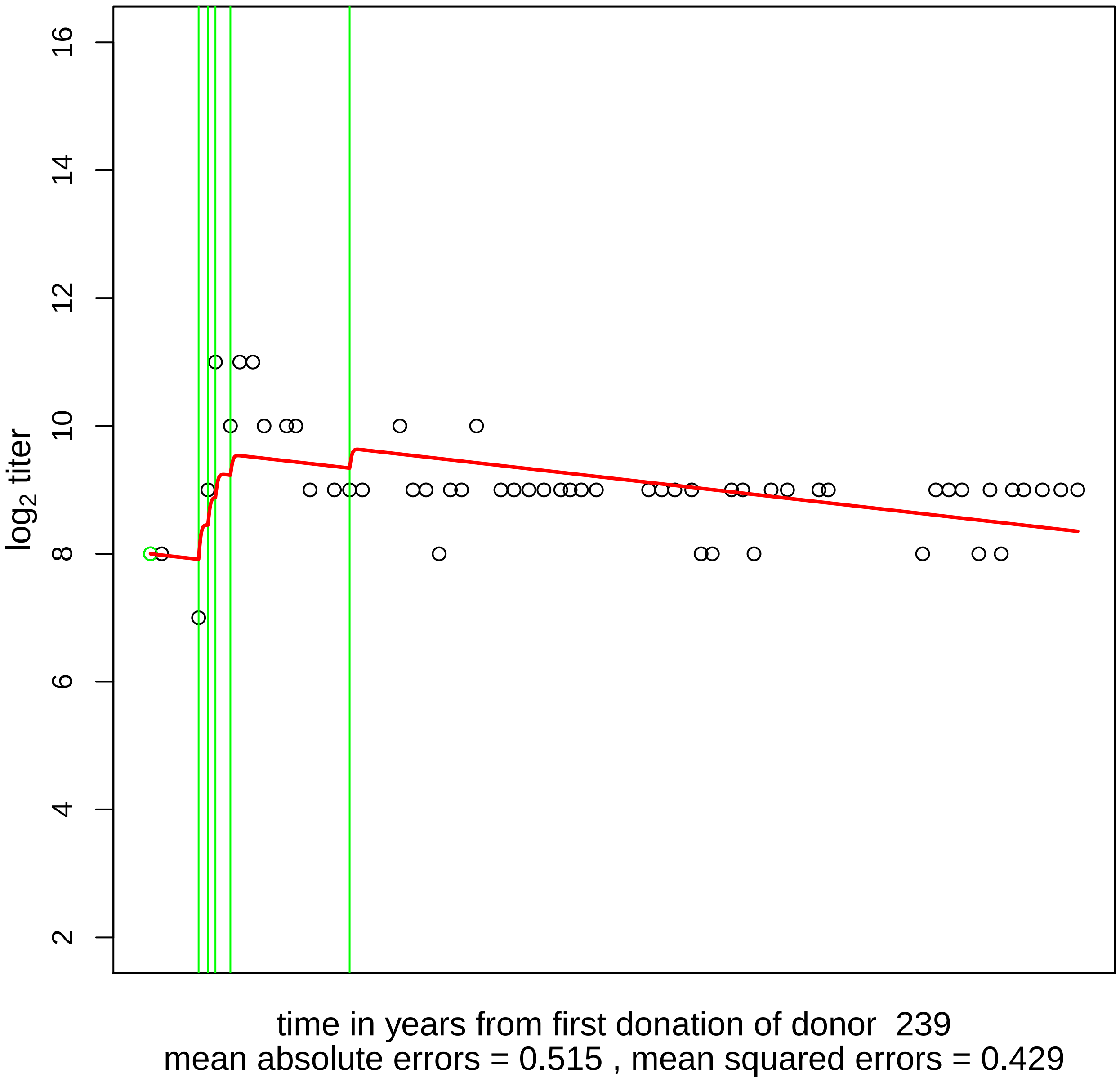

log2 titer
2
4
6
8
10
12
14
16
time in years from first donation of donor 239
mean absolute errors = 0.515 , mean squared errors = 0.429

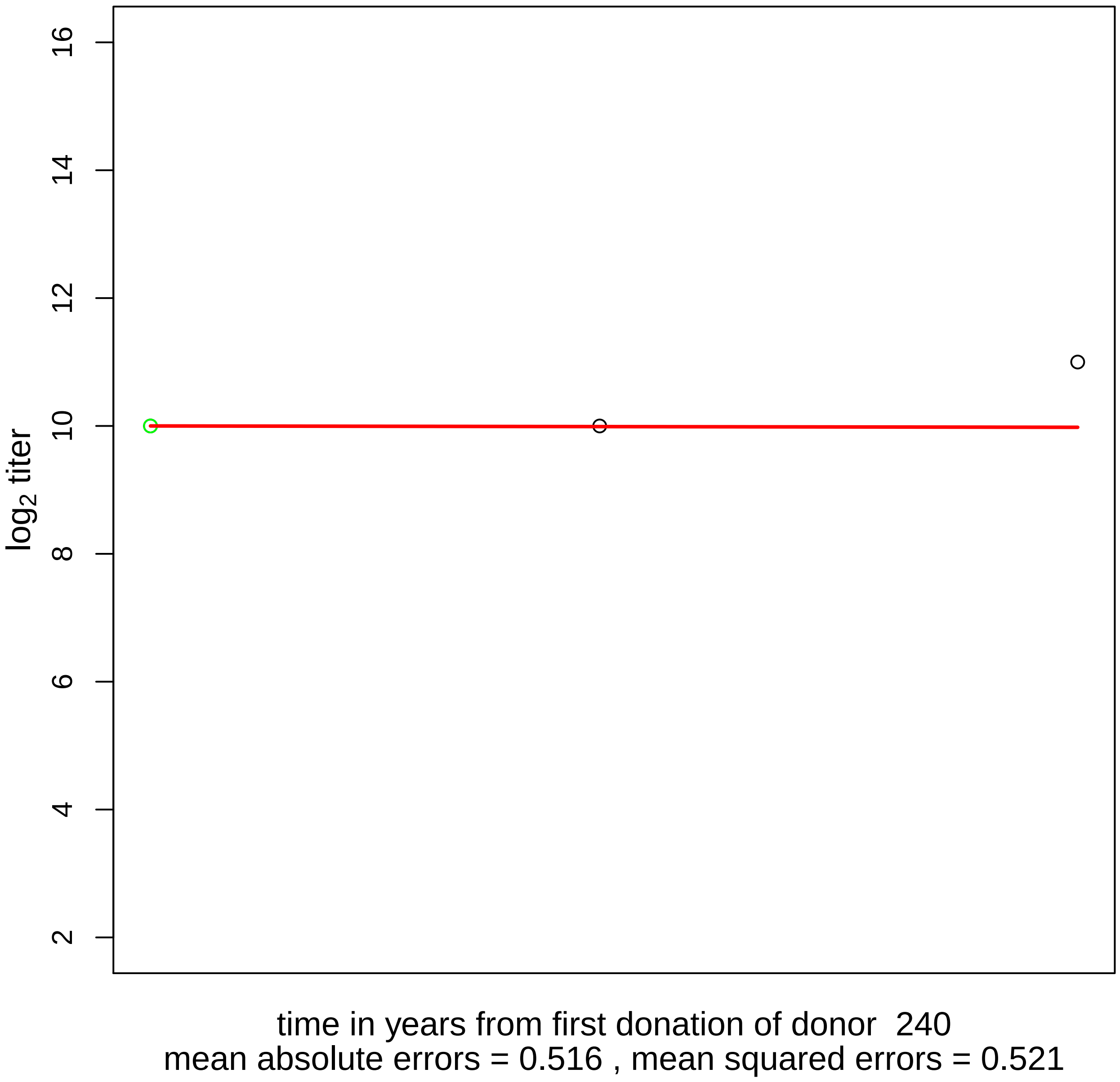

log2 titer
2
4
6
8
10
12
14
16
time in years from first donation of donor 240
mean absolute errors = 0.516 , mean squared errors = 0.521

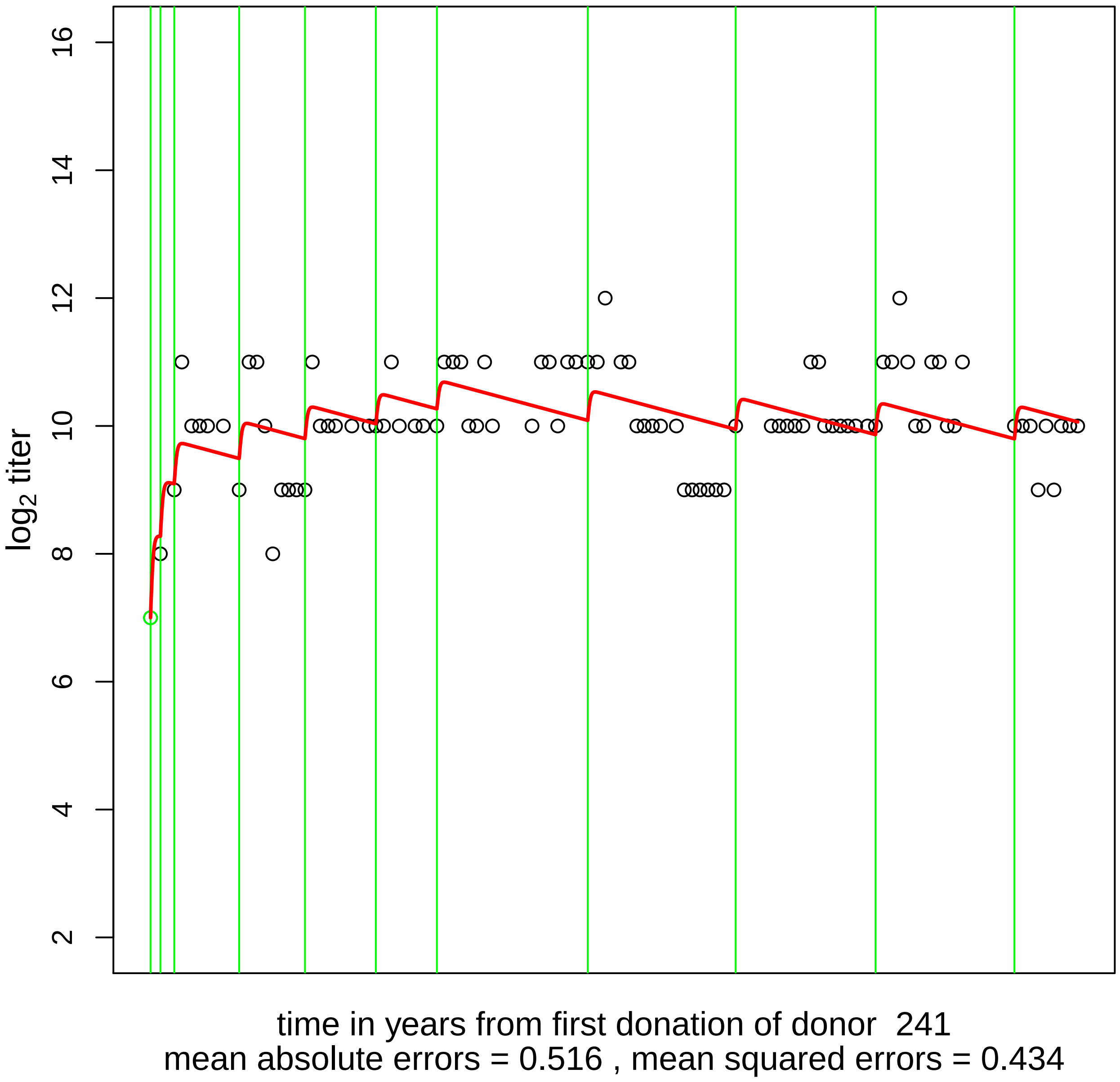

16
14
12
10
8
6
4
2
log2 titer
time in years from first donation of donor 241
mean absolute errors = 0.516 , mean squared errors = 0.434

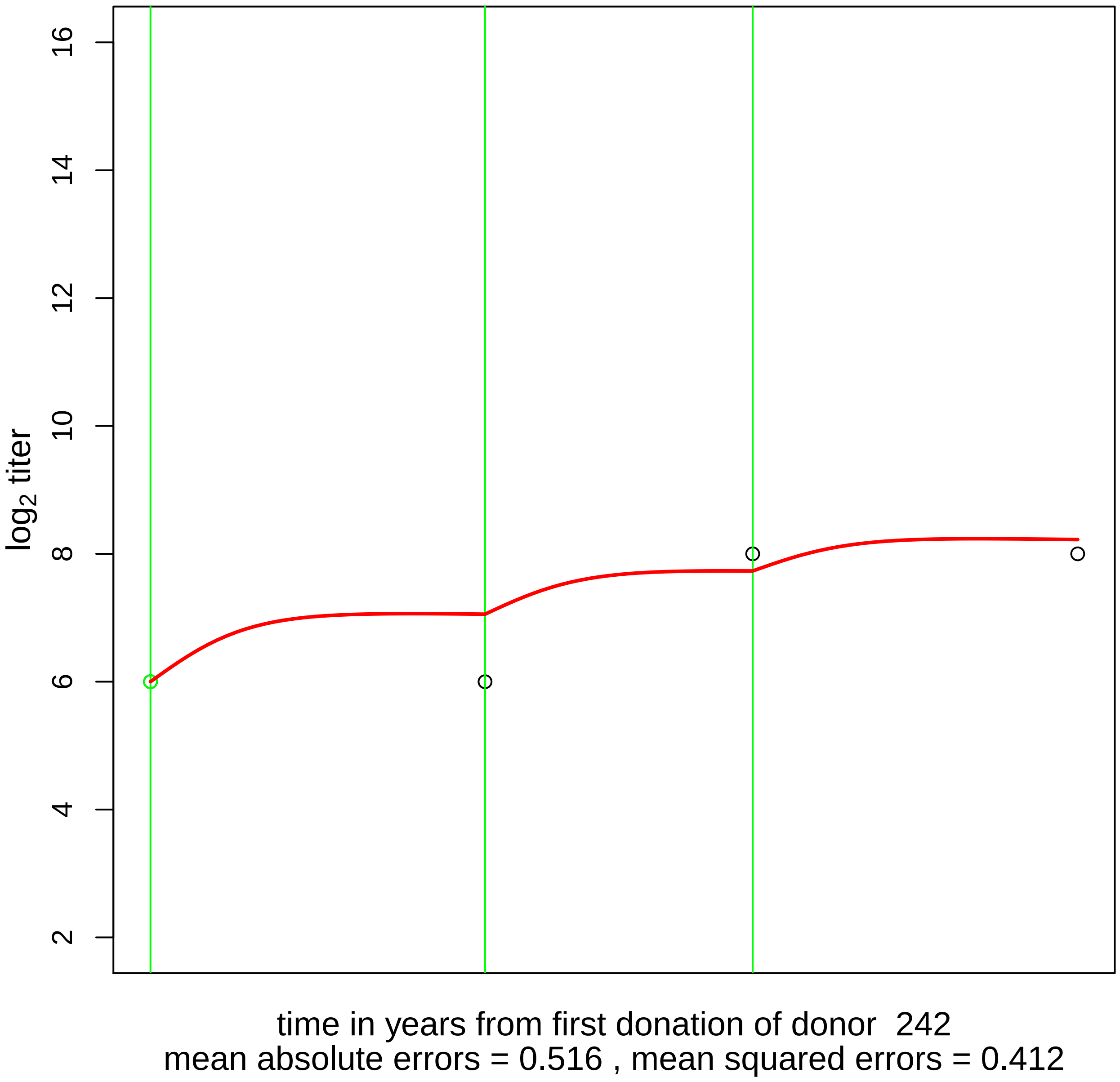

16
14
12
10
8
6
4
2
log2 titer
time in years from first donation of donor 242
mean absolute errors = 0.516 , mean squared errors = 0.412

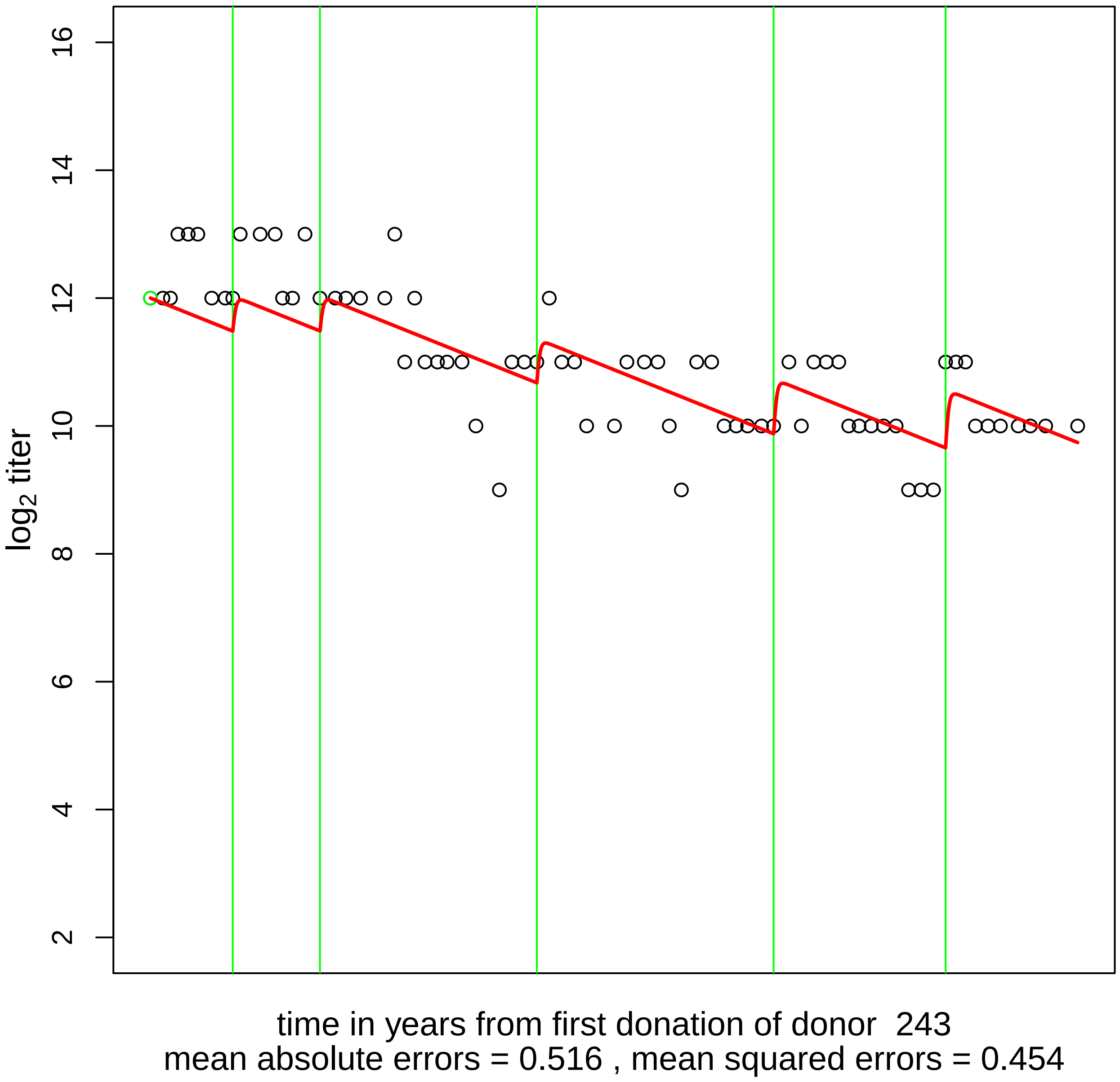

2
4
6
8
10
12
14
16
log2 titer
time in years from first donation of donor 243
mean absolute errors = 0.516 , mean squared errors = 0.454

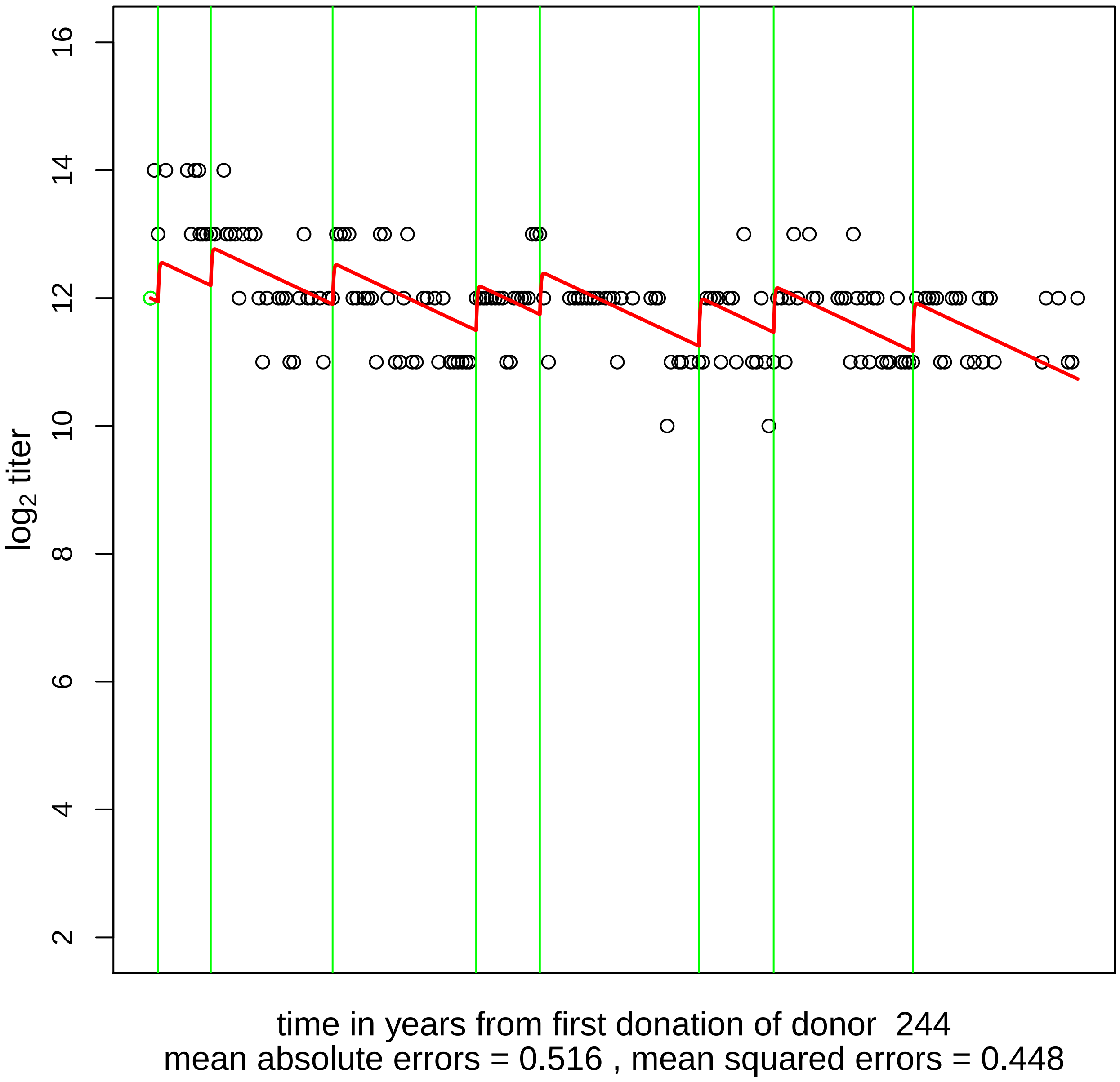

16
14
12
10
8
6
4
2
$\log_2$ titer
time in years from first donation of donor 244
mean absolute errors = 0.516 , mean squared errors = 0.448

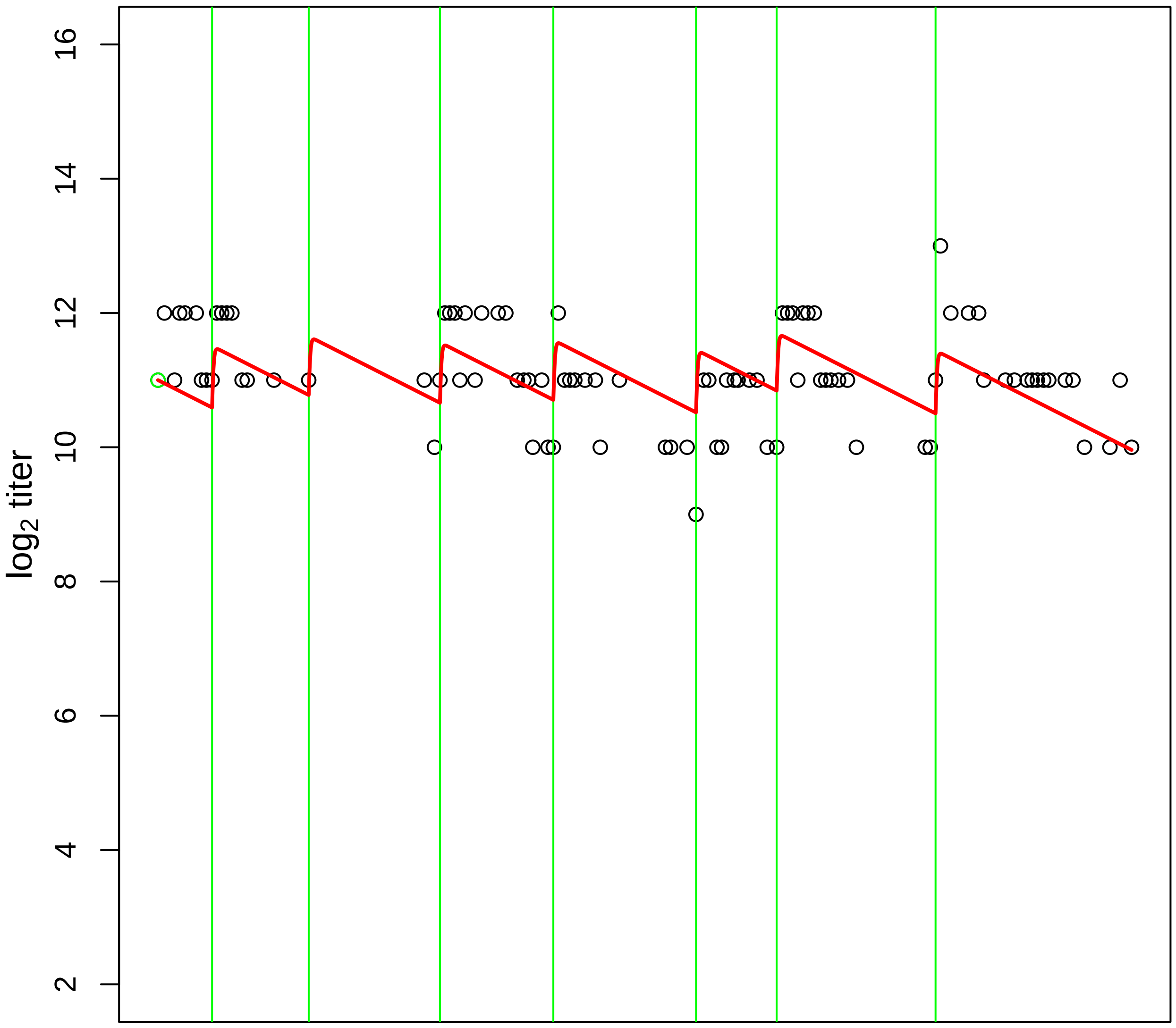

time in years from first donation of donor 245
mean absolute errors = 0.516 , mean squared errors = 0.393

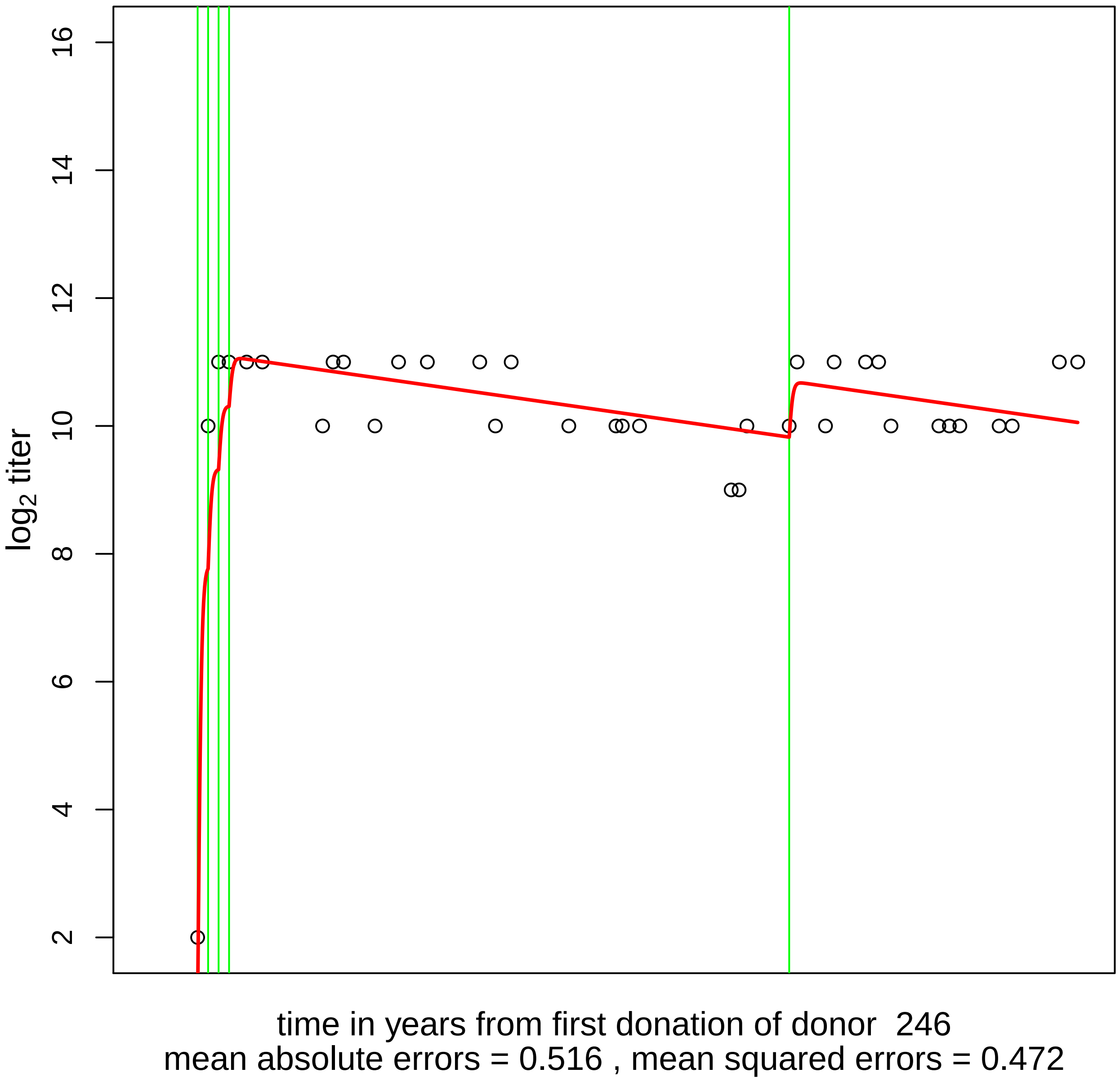

2
4
6
8
10
12
14
16
log2 titer
time in years from first donation of donor 246
mean absolute errors = 0.516 , mean squared errors = 0.472

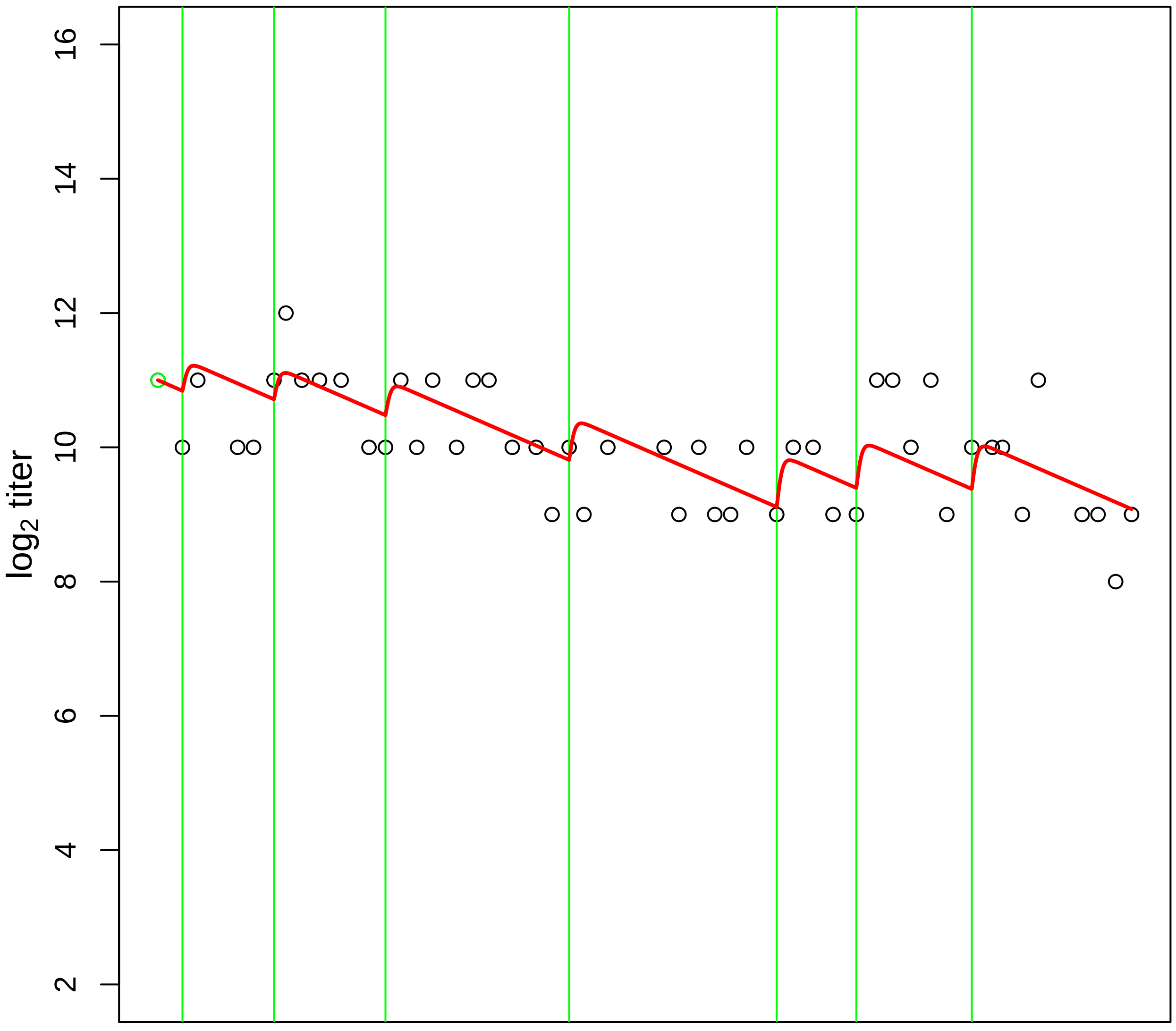

time in years from first donation of donor 247
mean absolute errors = 0.517 , mean squared errors = 0.407

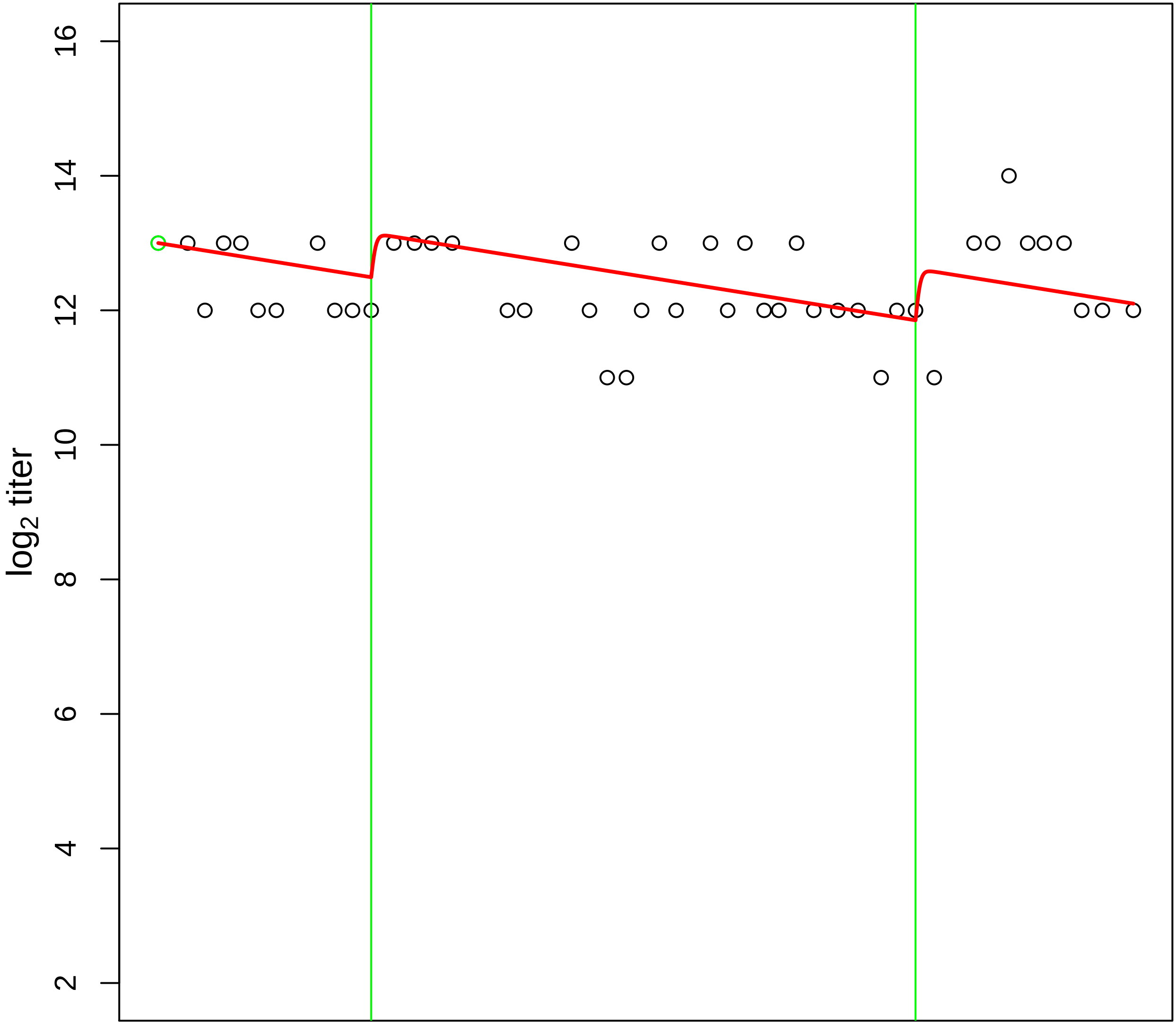

time in years from first donation of donor 248
mean absolute errors = 0.517 , mean squared errors = 0.453

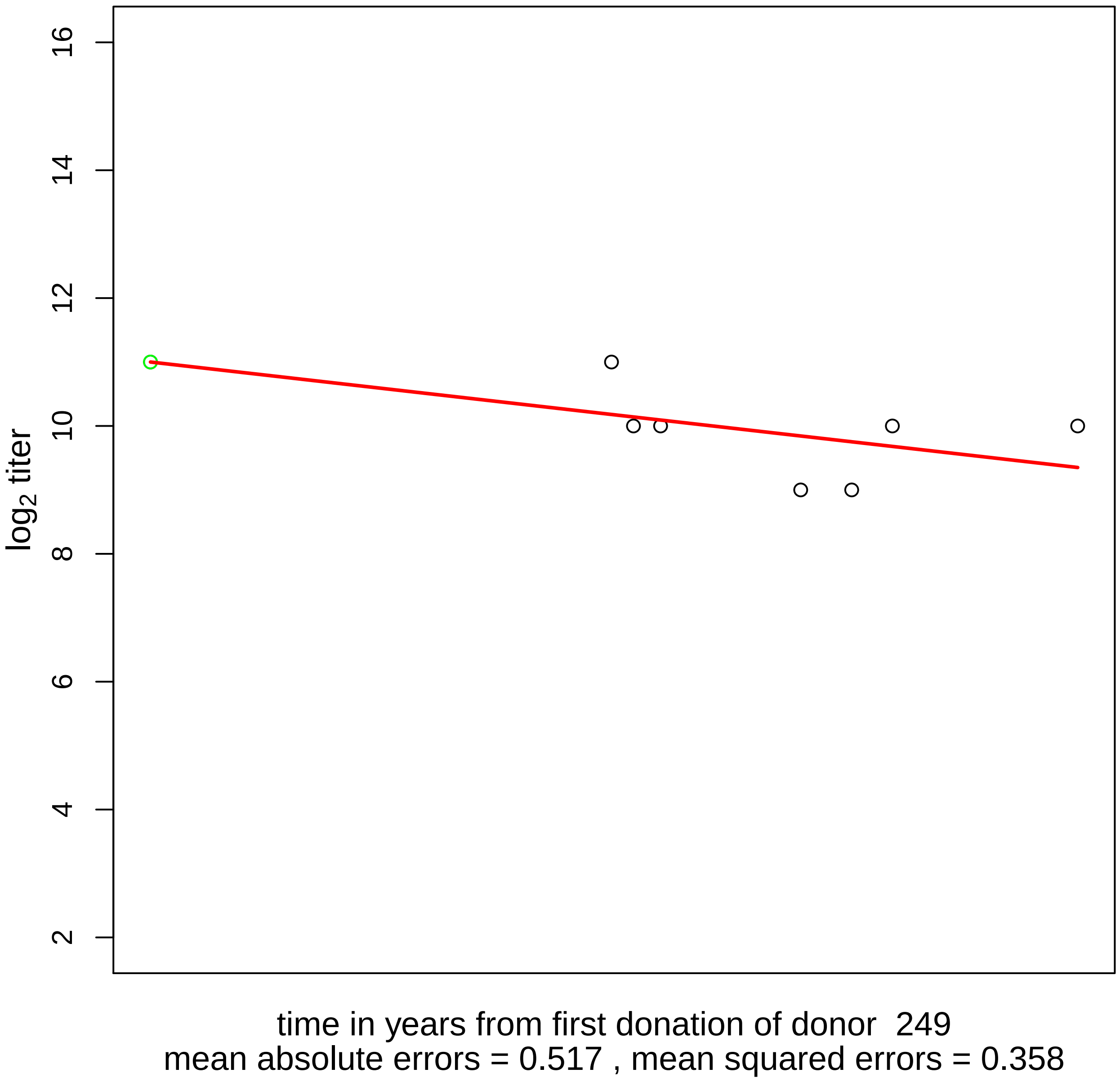

16
14
12
10
8
6
4
2
log2 titer
time in years from first donation of donor 249
mean absolute errors = 0.517 , mean squared errors = 0.358

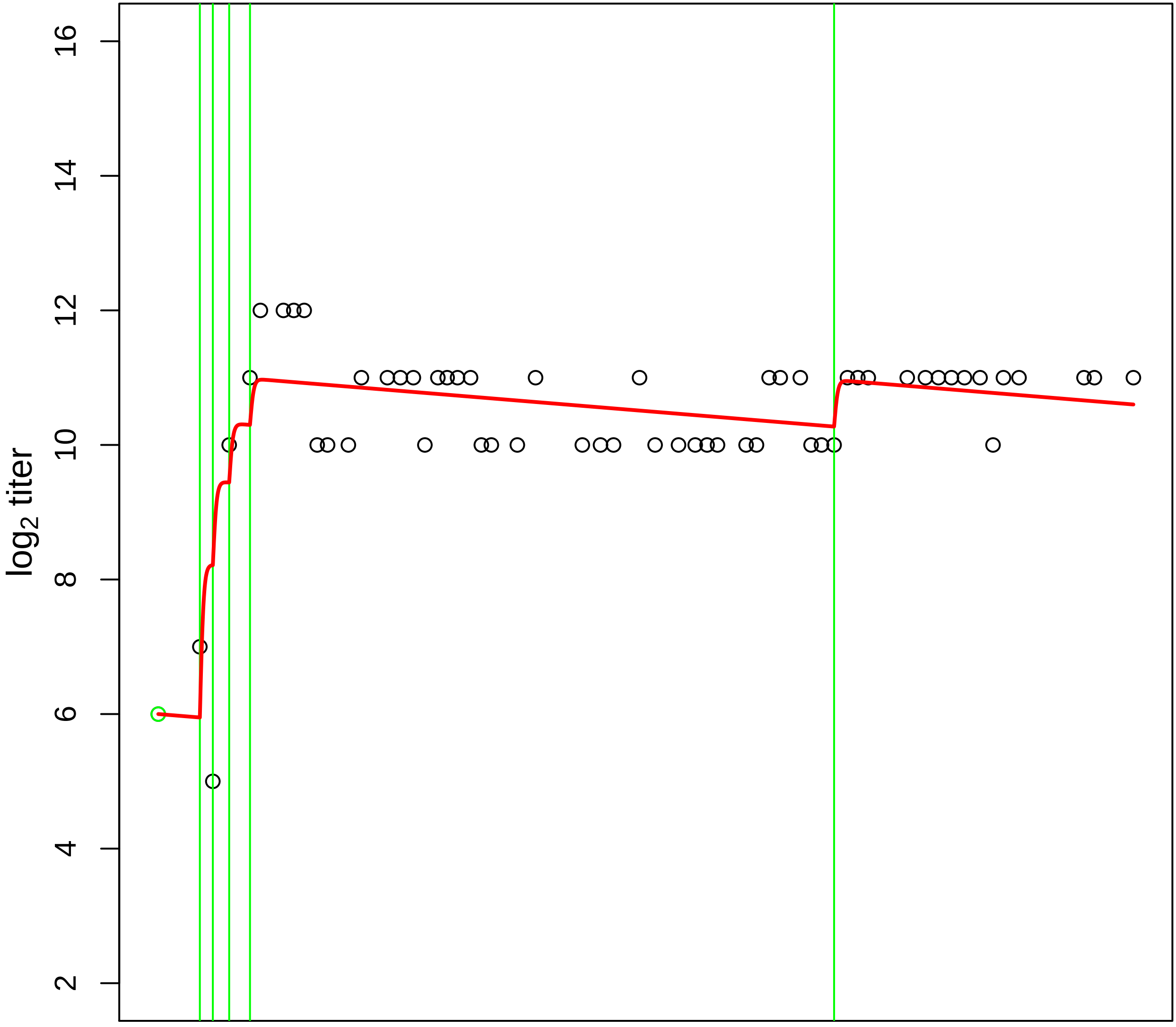

time in years from first donation of donor 250
mean absolute errors = 0.517 , mean squared errors = 0.482

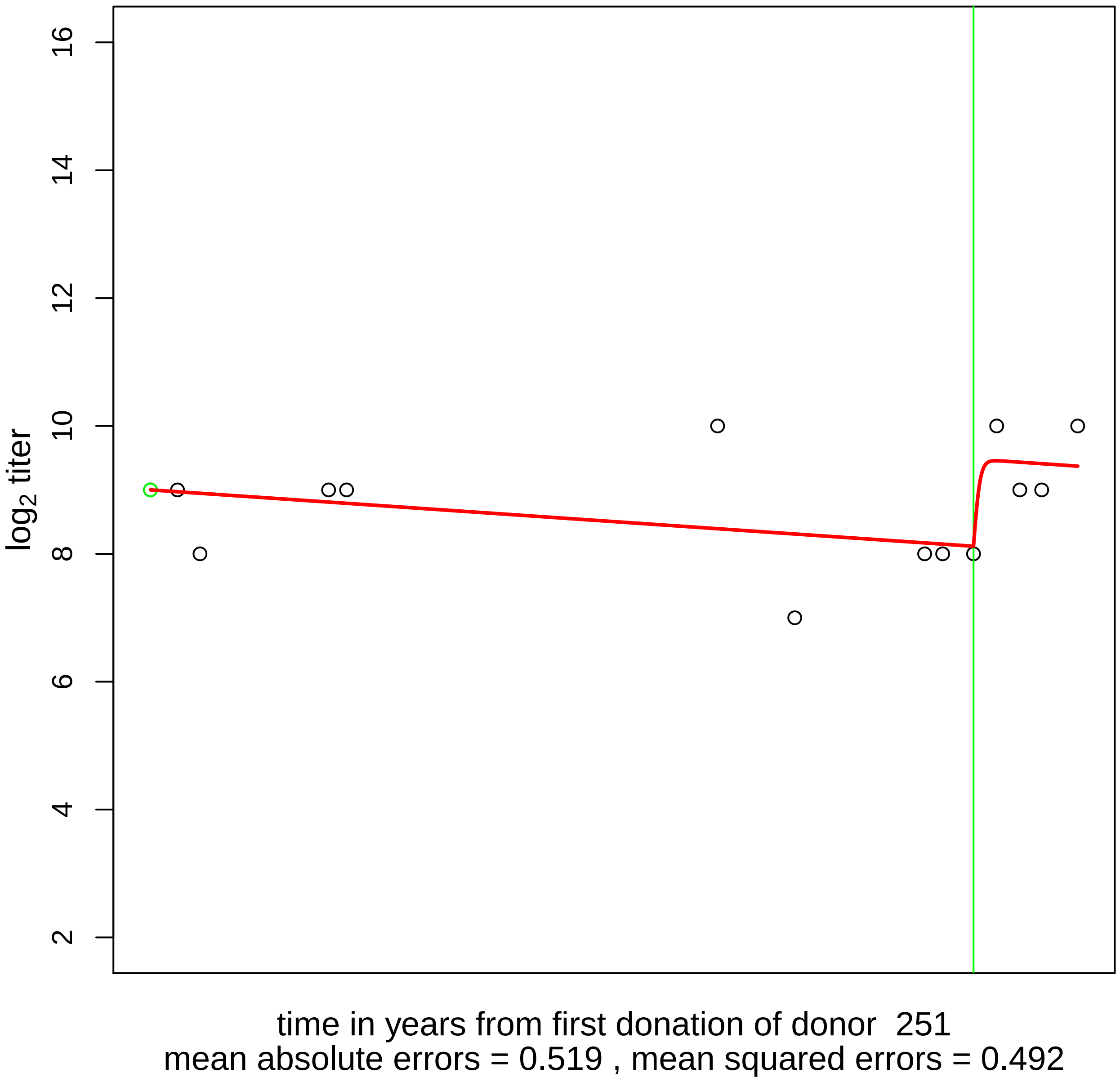

16
14
12
10
8
6
4
2
log2 titer
time in years from first donation of donor 251
mean absolute errors = 0.519 , mean squared errors = 0.492

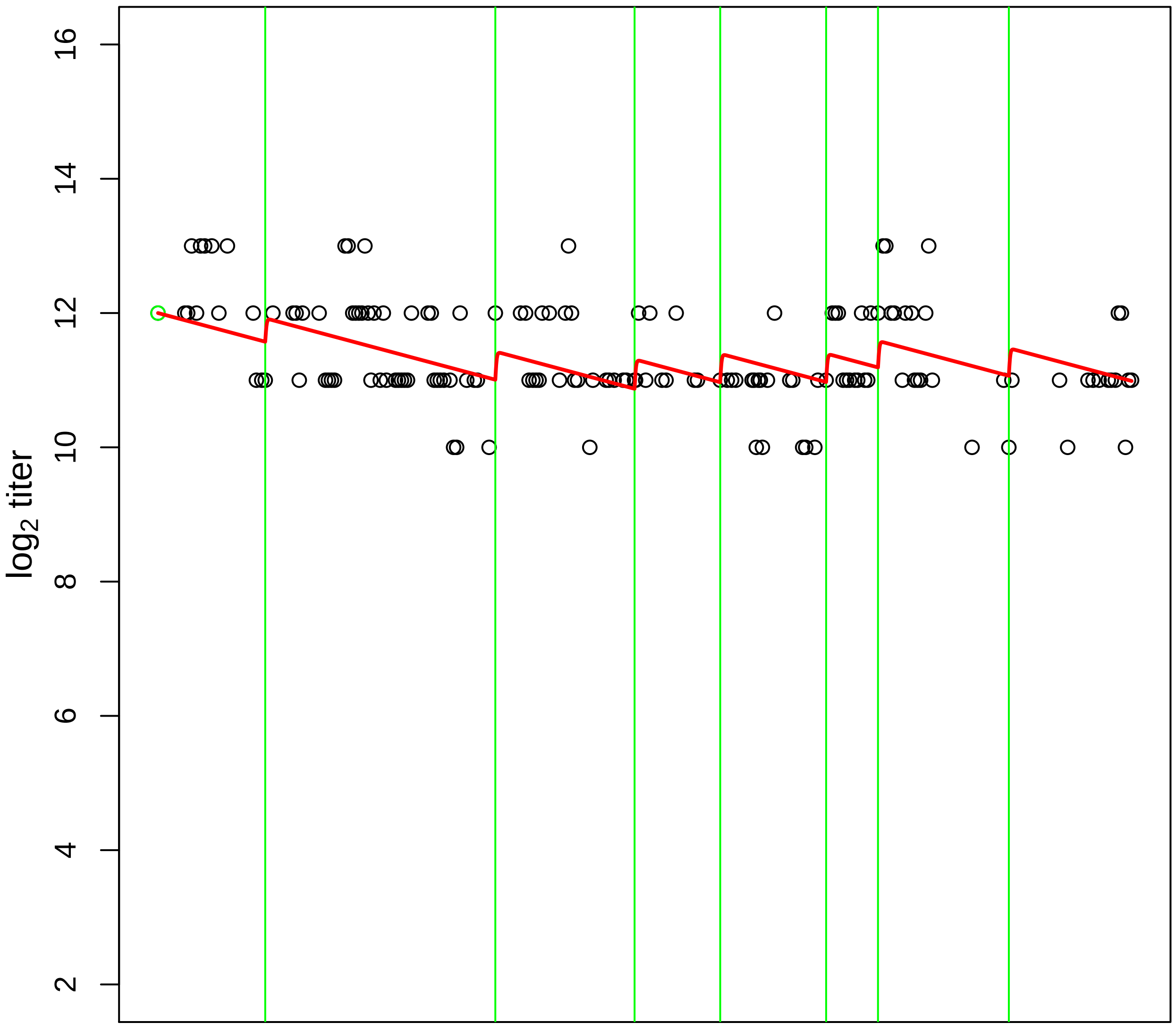

time in years from first donation of donor 252
mean absolute errors = 0.519 , mean squared errors = 0.439

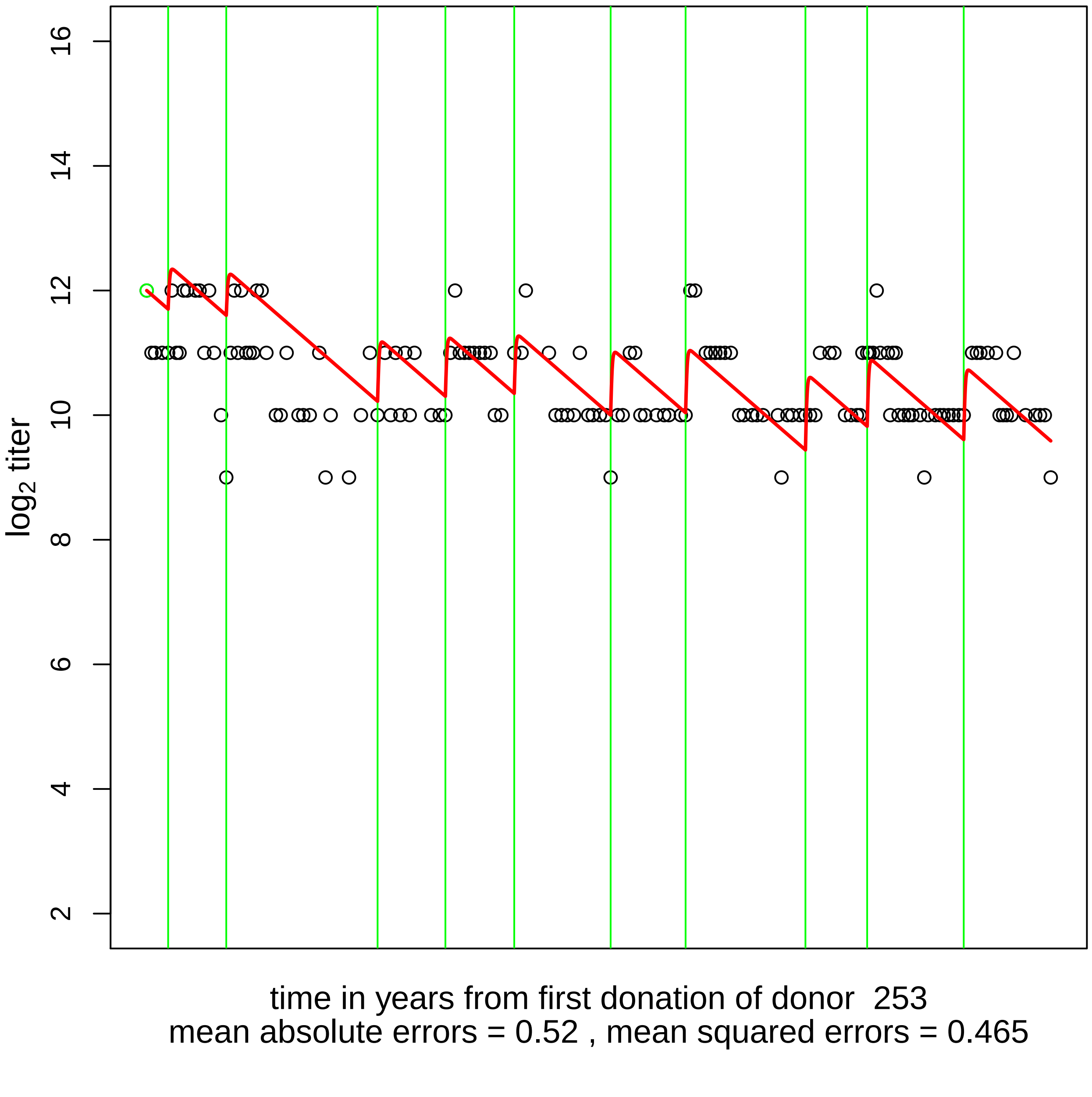
log2 titer
16
14
12
10
8
6
4
2
time in years from first donation of donor 253
mean absolute errors = 0.52 , mean squared errors = 0.465

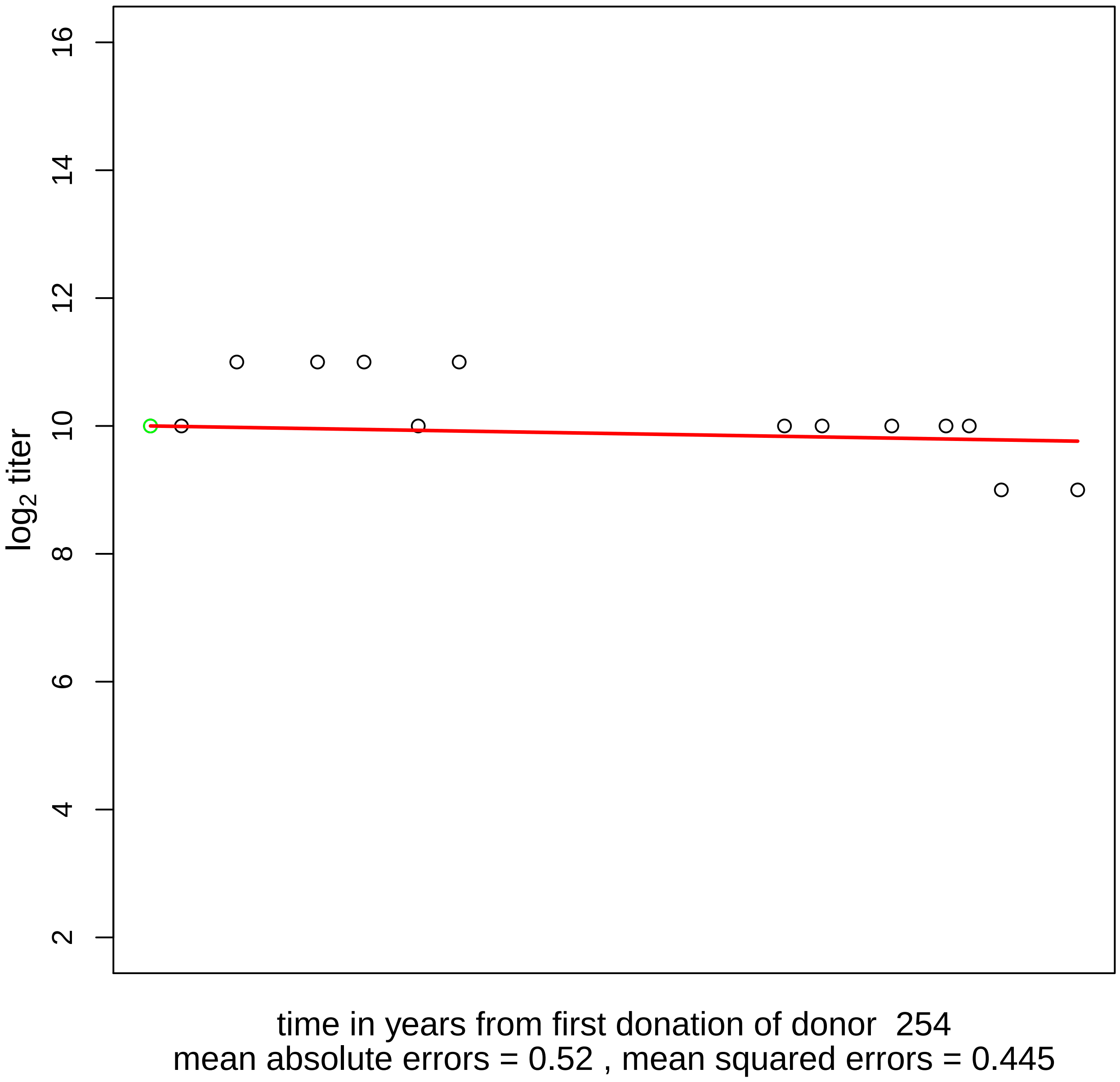

16
14
12
10
8
6
4
2
log_2 titer
time in years from first donation of donor 254
mean absolute errors = 0.52 , mean squared errors = 0.445

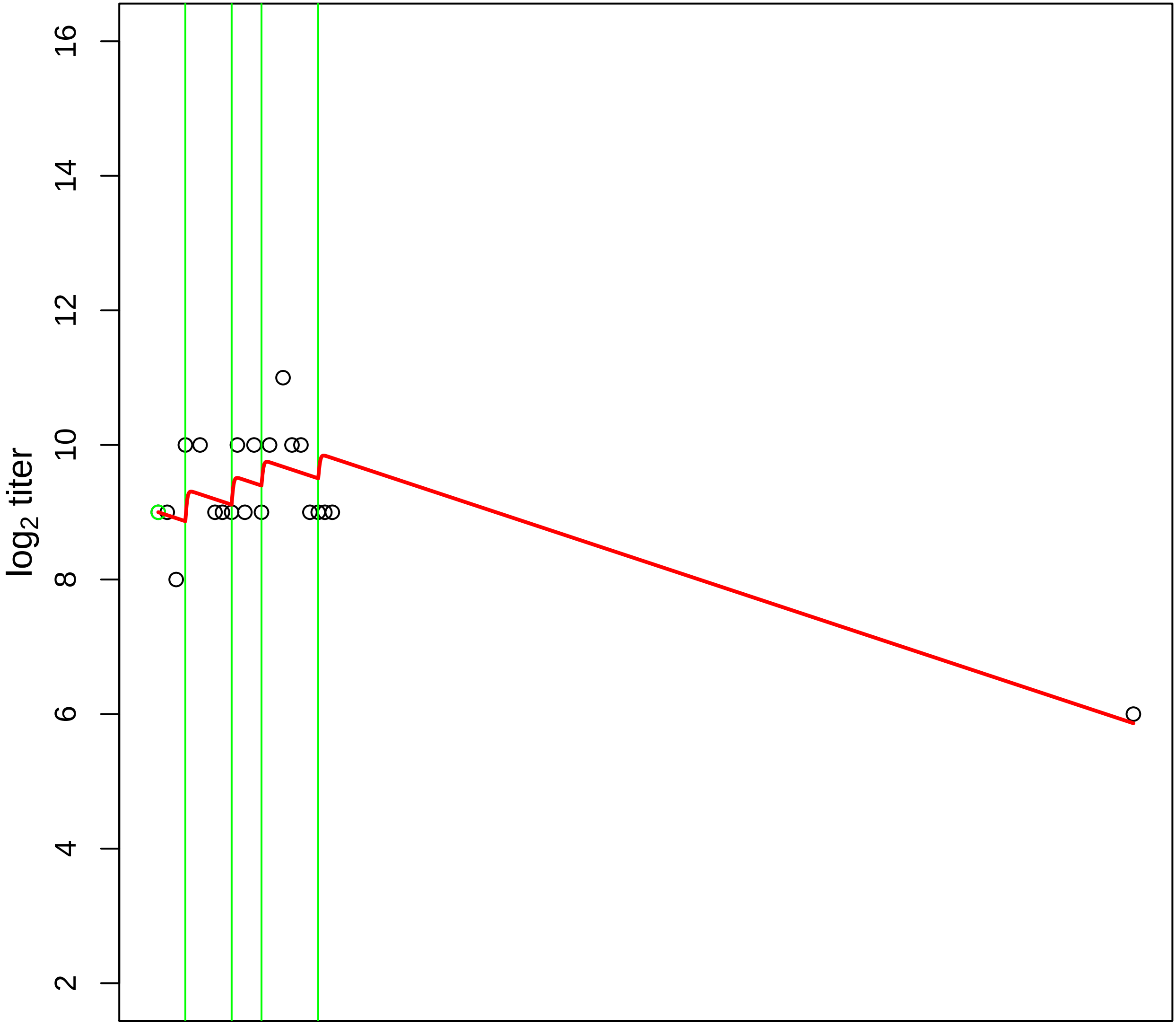

time in years from first donation of donor 255
mean absolute errors = 0.52 , mean squared errors = 0.386

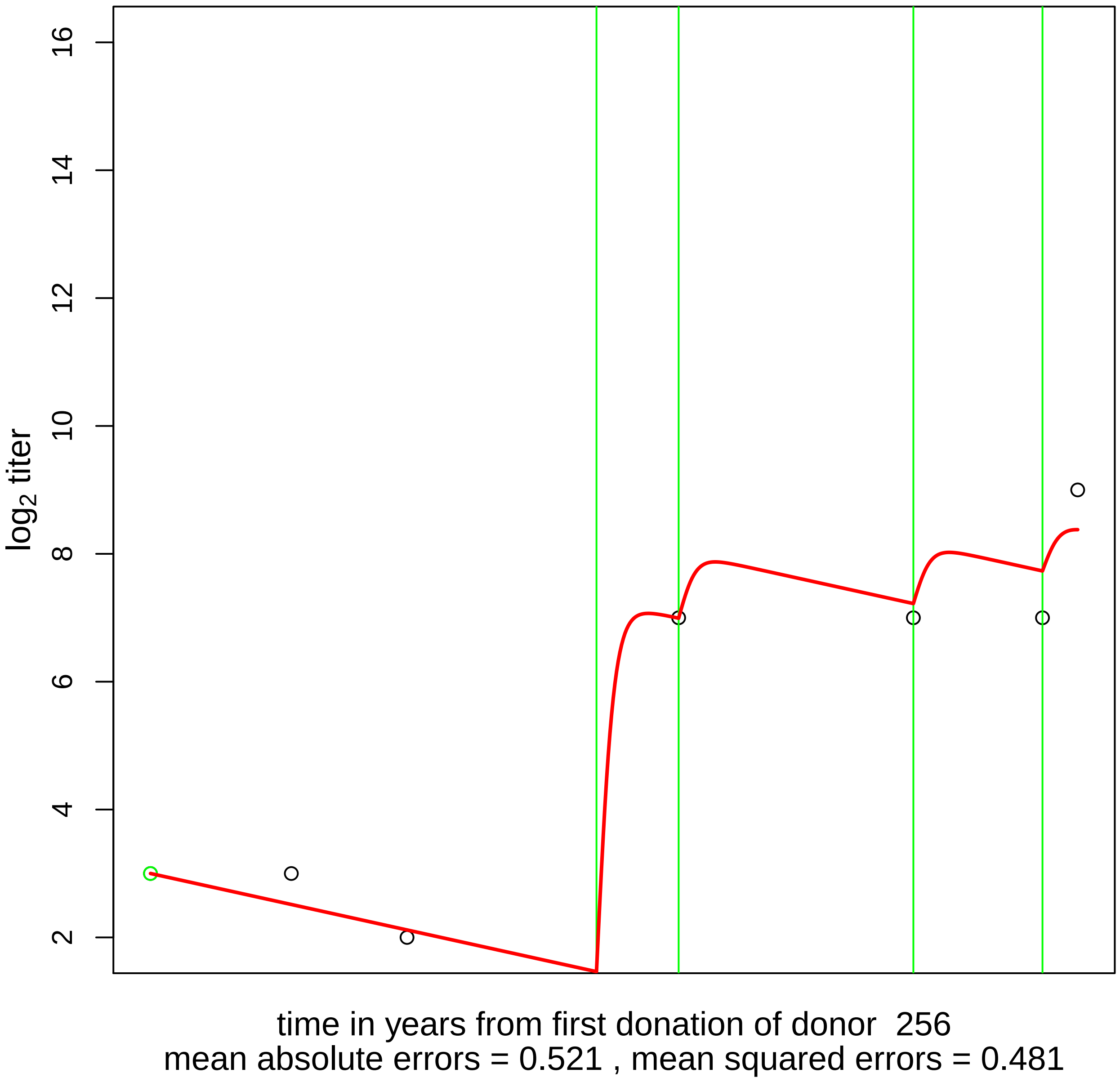

16
14
12
10
8
6
4
2
log2 titer
time in years from first donation of donor 256
mean absolute errors = 0.521 , mean squared errors = 0.481

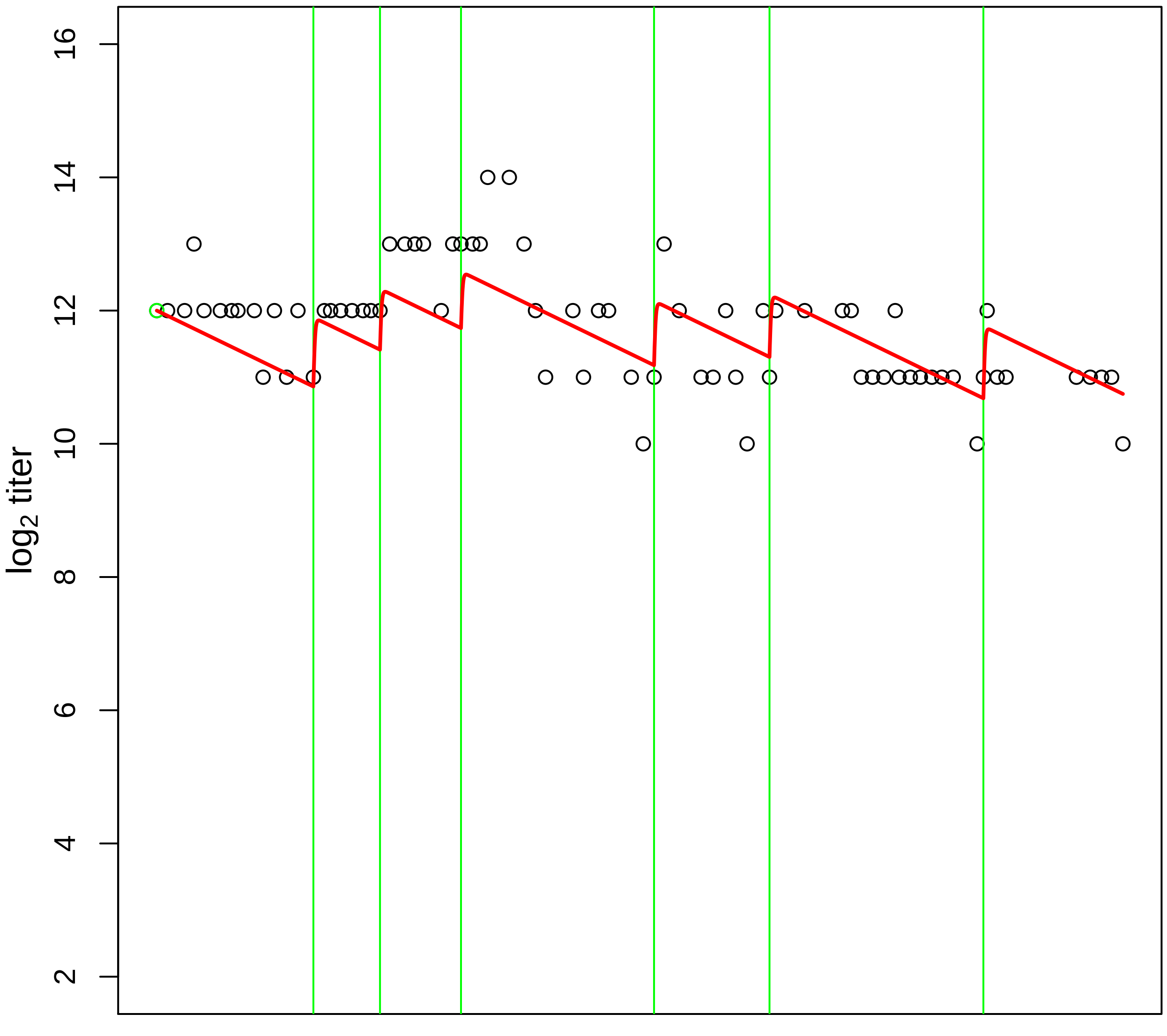

time in years from first donation of donor 257
mean absolute errors = 0.521 , mean squared errors = 0.43

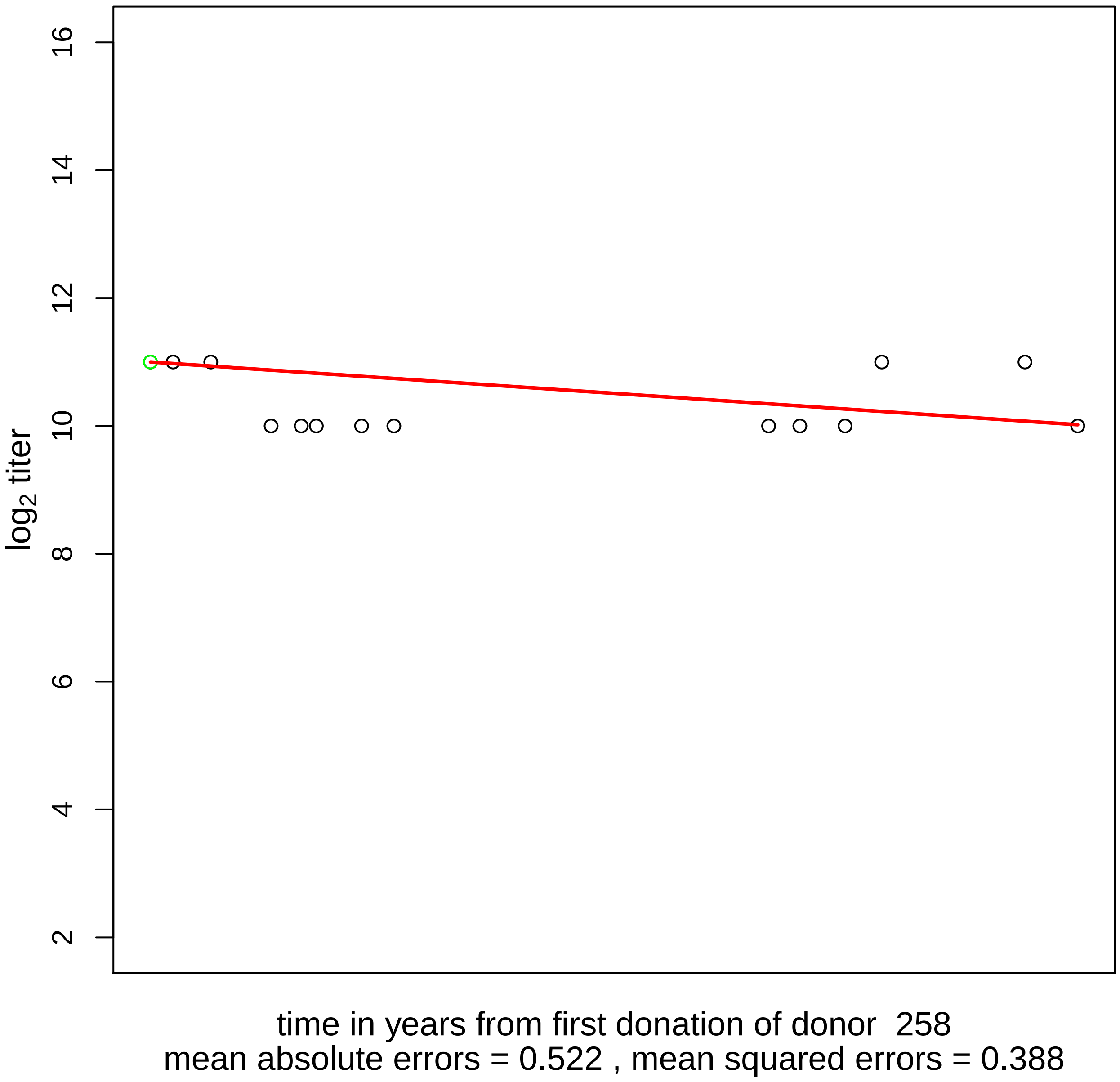

16
14
12
10
8
6
4
2
log2 titer
time in years from first donation of donor 258
mean absolute errors = 0.522 , mean squared errors = 0.388

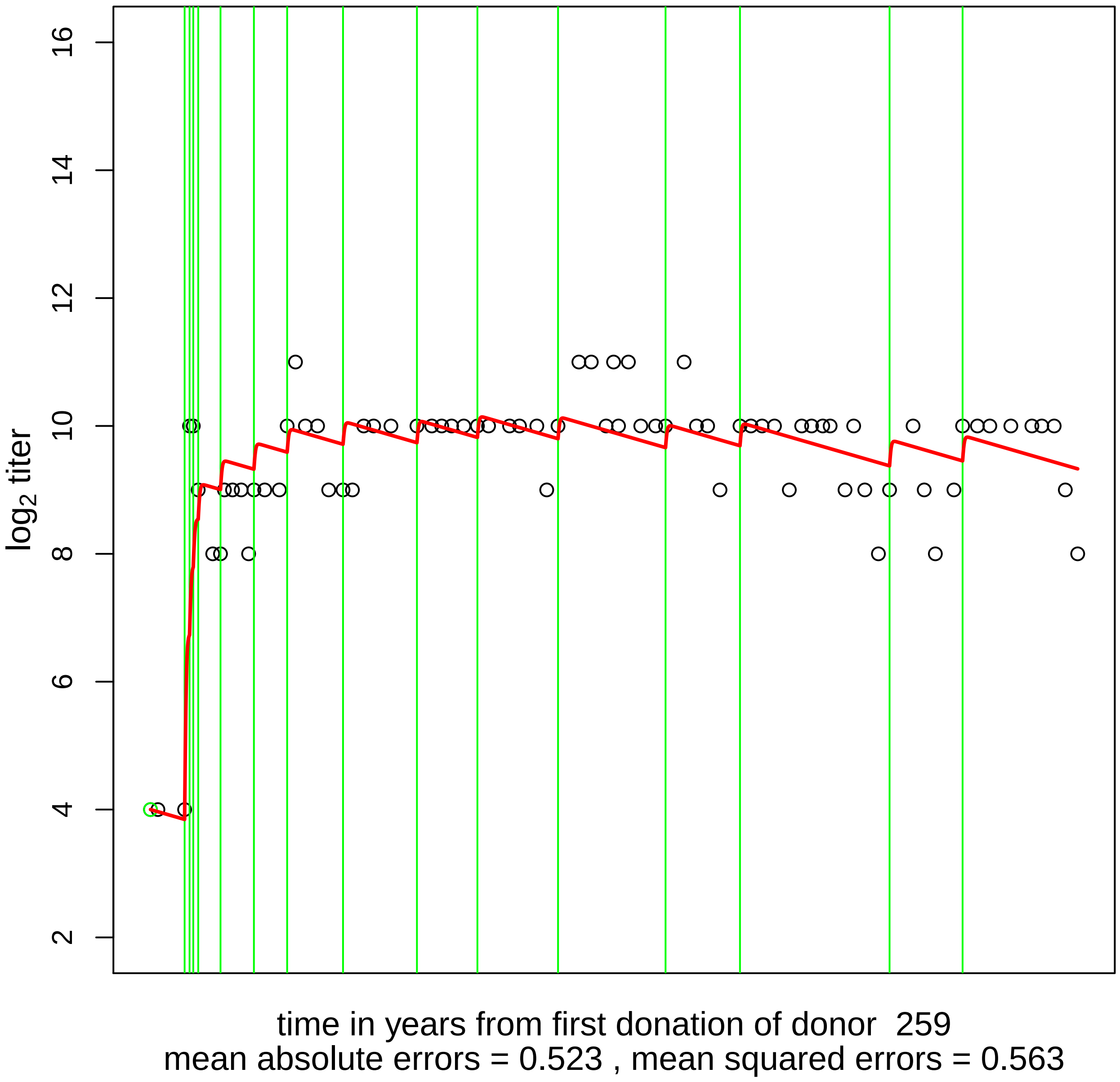

log2 titer
2
4
6
8
10
12
14
16
time in years from first donation of donor 259
mean absolute errors = 0.523 , mean squared errors = 0.563

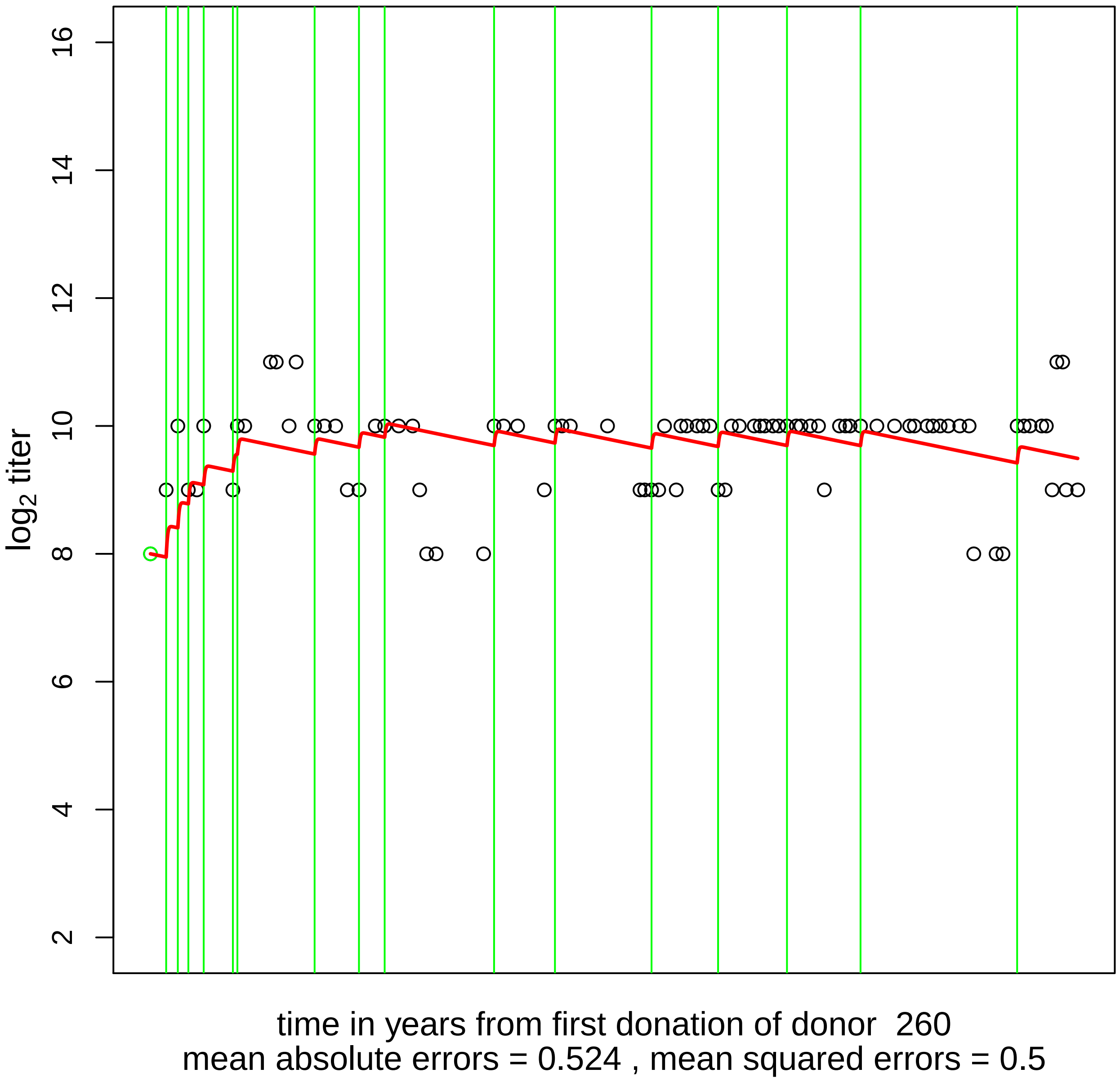

16
14
12
10
8
6
4
2
log2 titer
time in years from first donation of donor 260
mean absolute errors = 0.524 , mean squared errors = 0.5

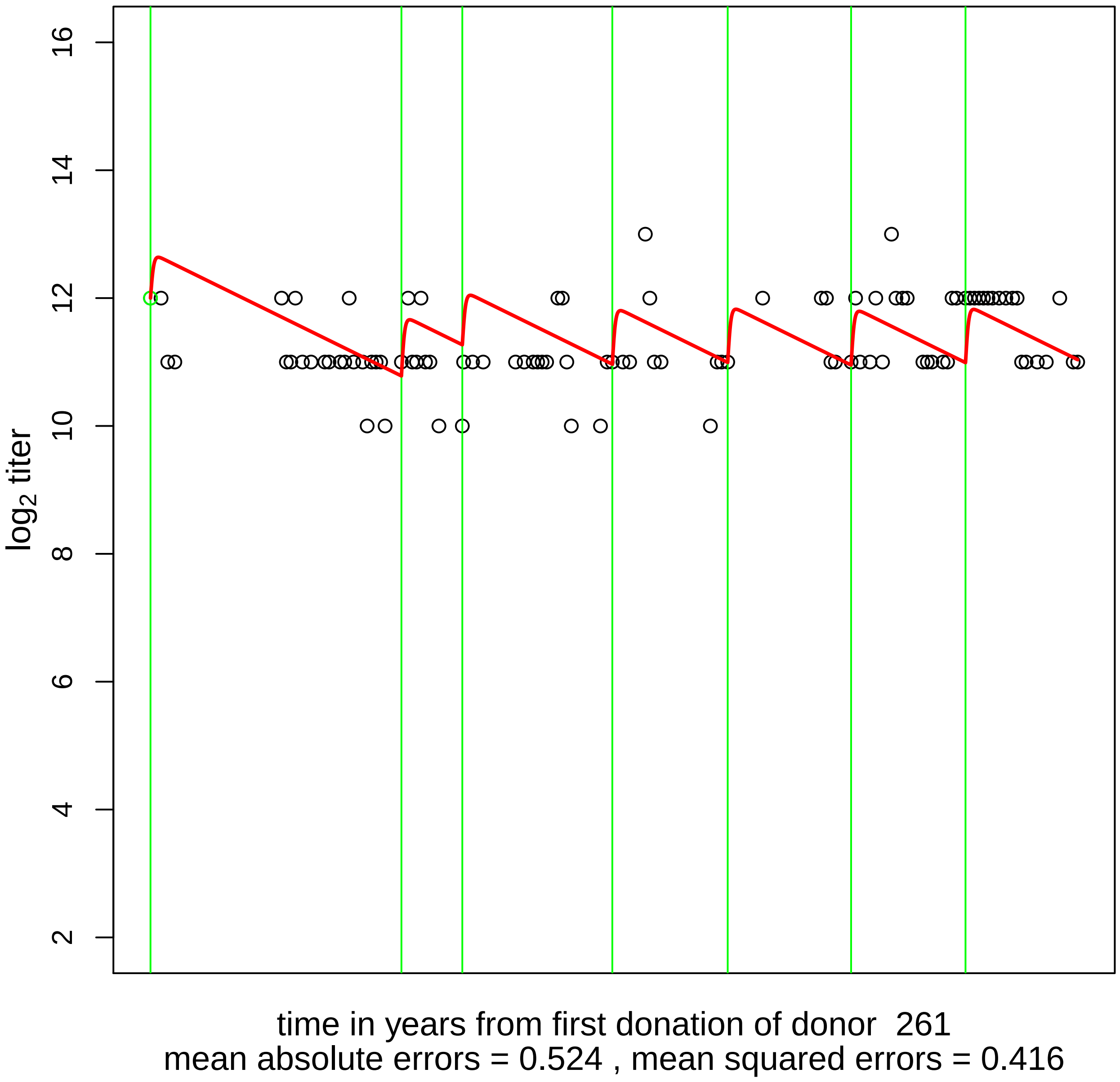
16
14
12
10
8
6
4
2
log2 titer
time in years from first donation of donor 261
mean absolute errors = 0.524 , mean squared errors = 0.416

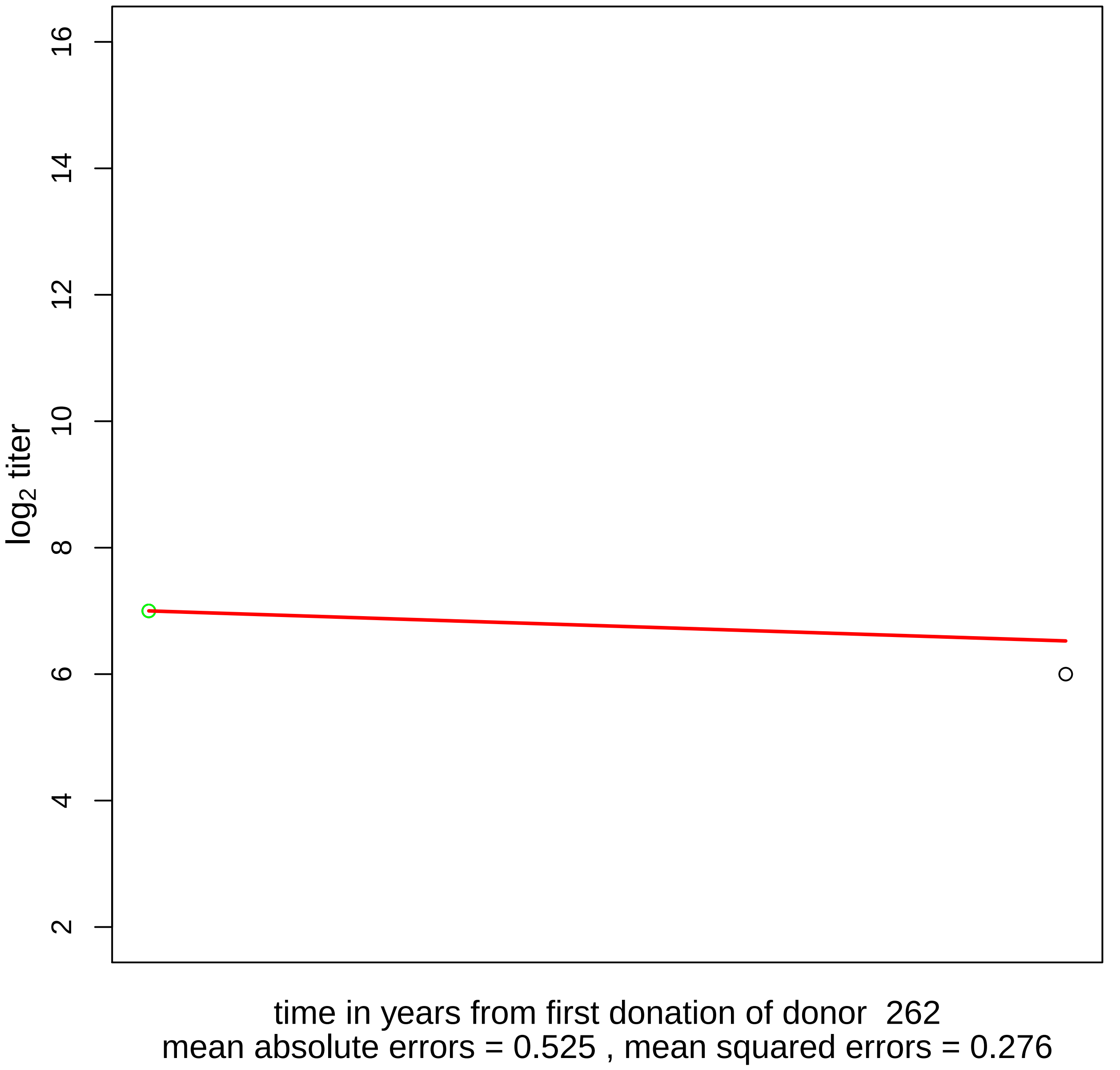

log2 titer
2
4
6
8
10
12
14
16
time in years from first donation of donor 262
mean absolute errors = 0.525 , mean squared errors = 0.276

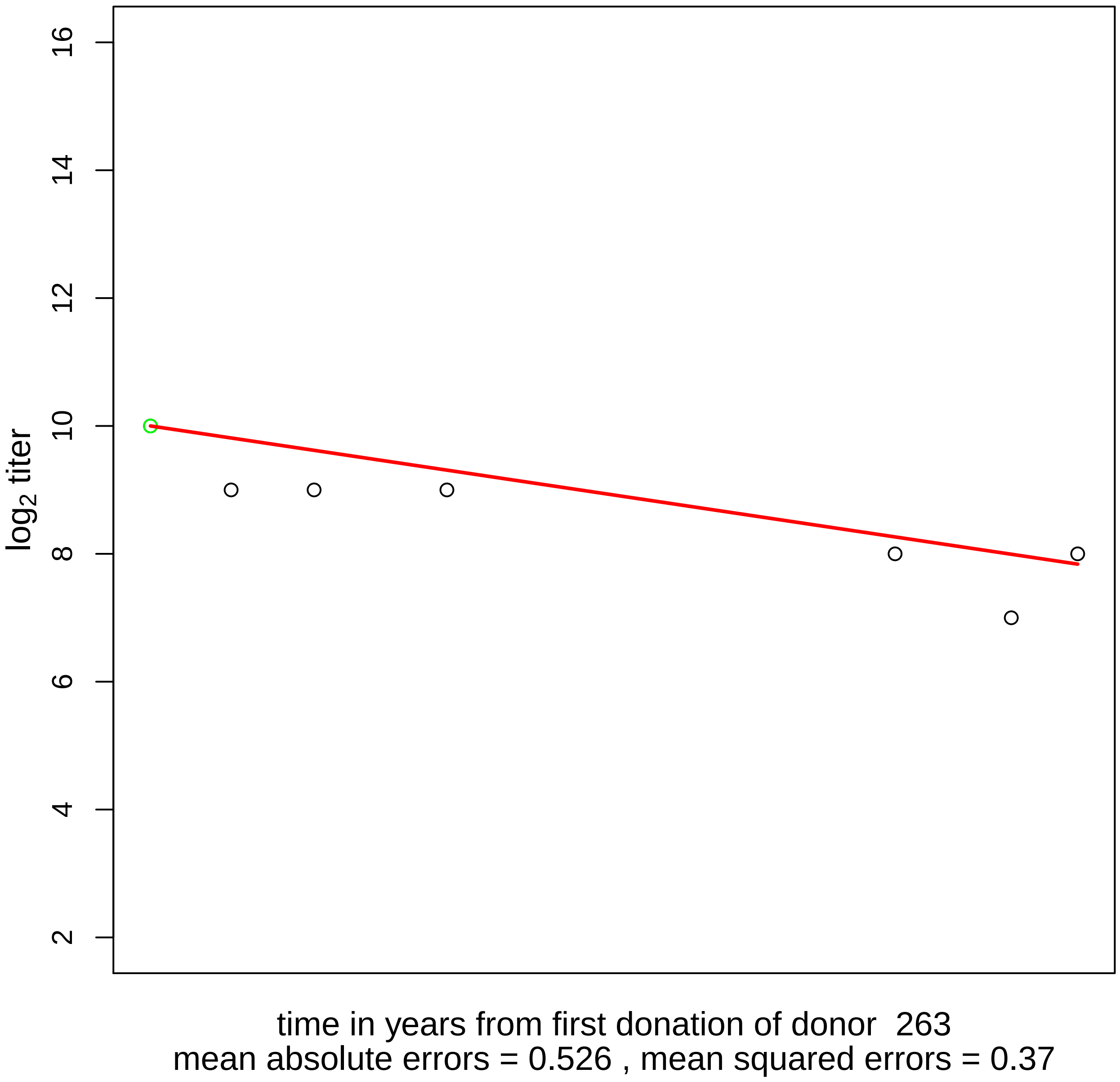

16
14
12
10
8
6
4
2
log2 titer
time in years from first donation of donor 263
mean absolute errors = 0.526 , mean squared errors = 0.37

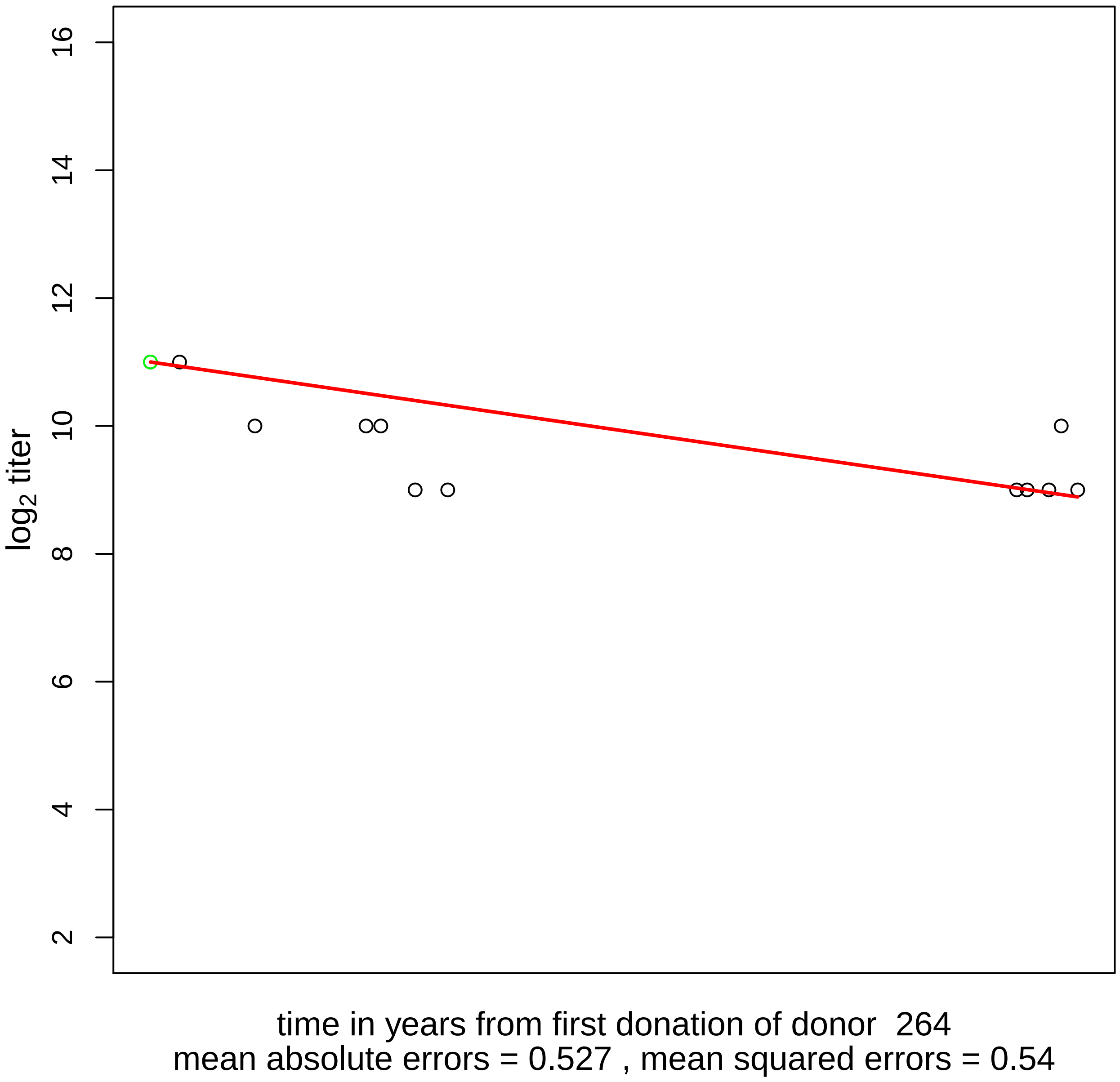

log2 titer
2
4
6
8
10
12
14
16
time in years from first donation of donor 264
mean absolute errors = 0.527 , mean squared errors = 0.54

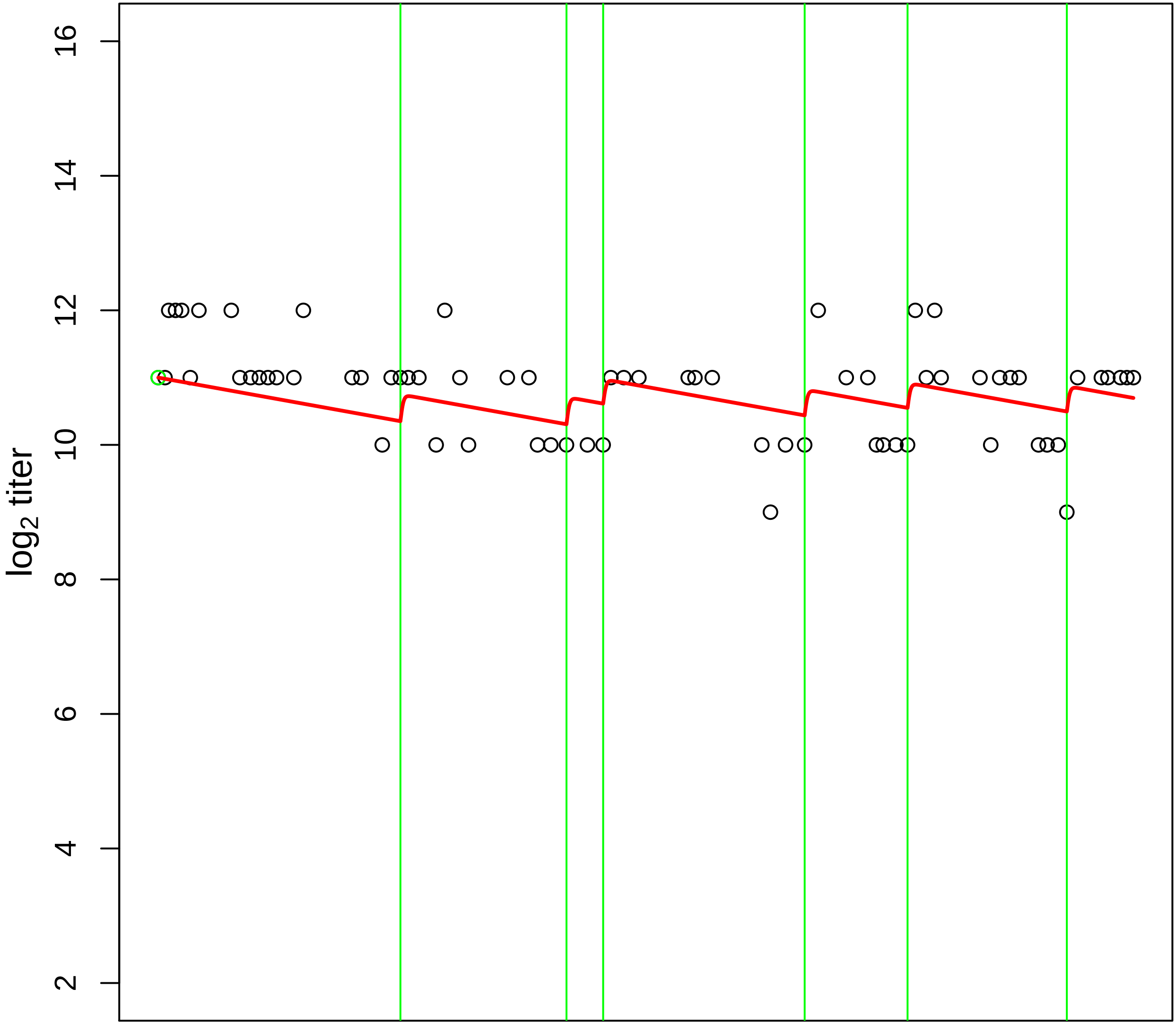

time in years from first donation of donor 265
mean absolute errors = 0.527 , mean squared errors = 0.413

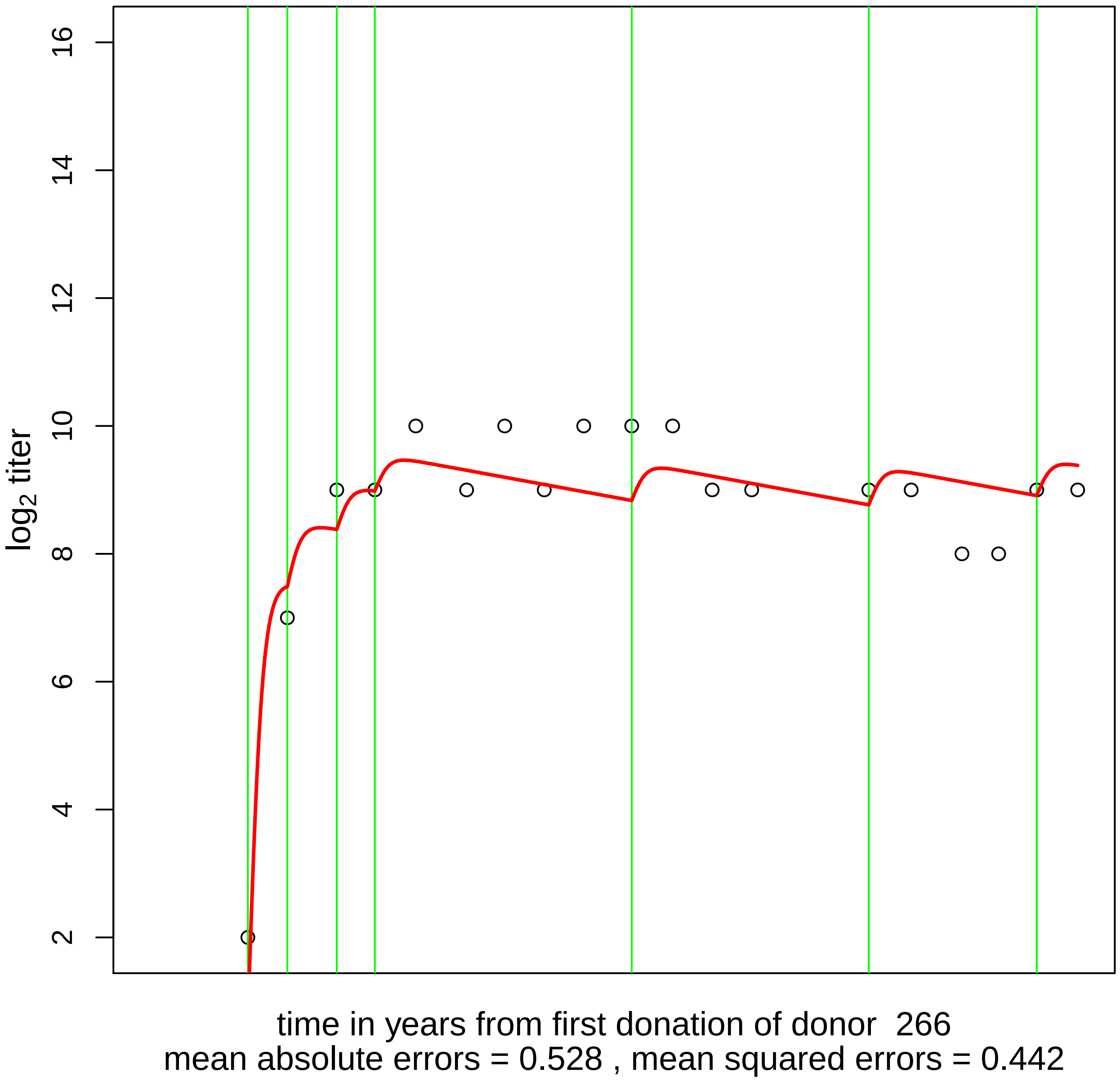

log2 titer
2
4
6
8
10
12
14
16
time in years from first donation of donor 266
mean absolute errors = 0.528 , mean squared errors = 0.442

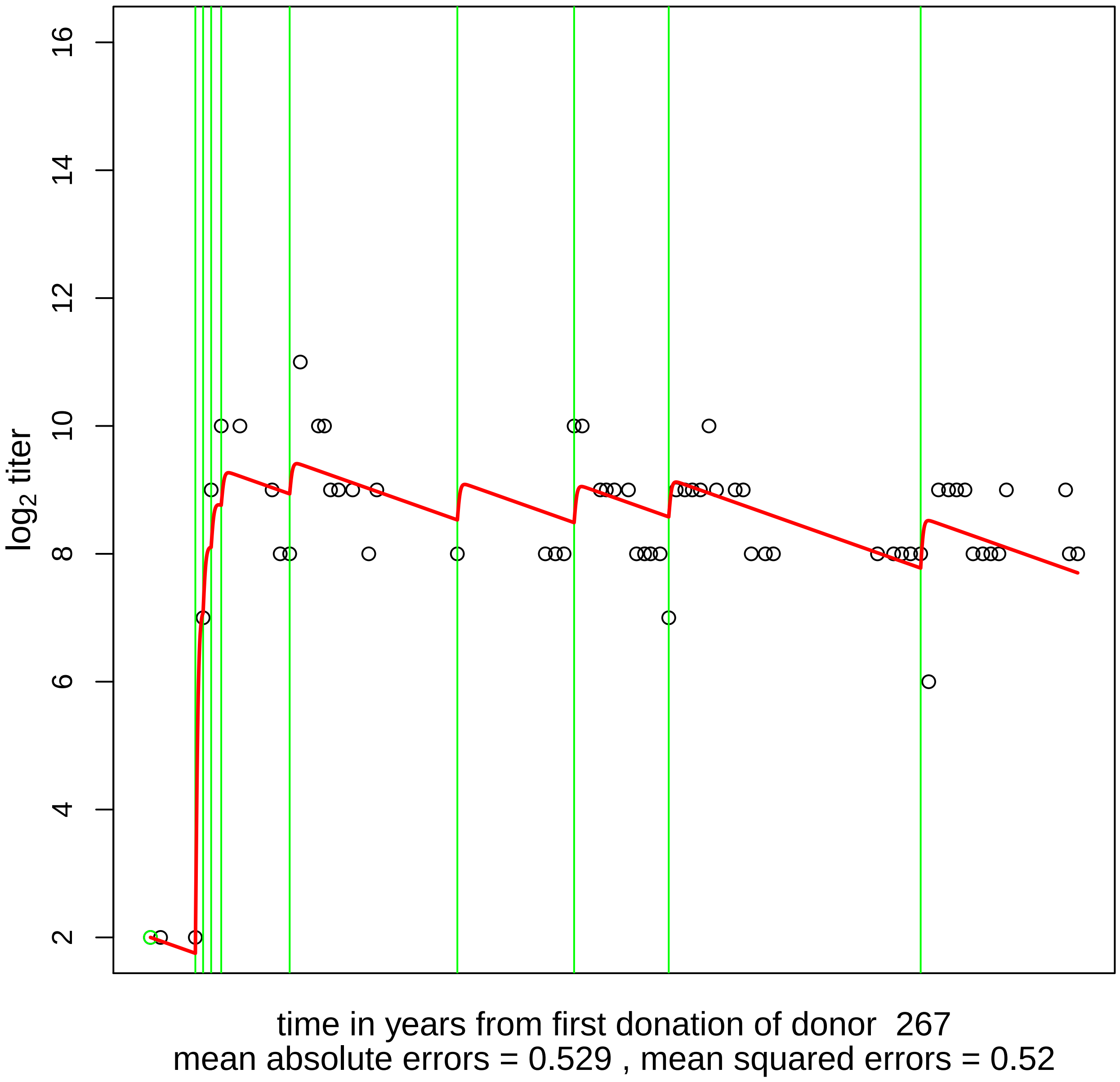

$\log_2$ titer
2
4
6
8
10
12
14
16
time in years from first donation of donor 267
mean absolute errors = 0.529 , mean squared errors = 0.52

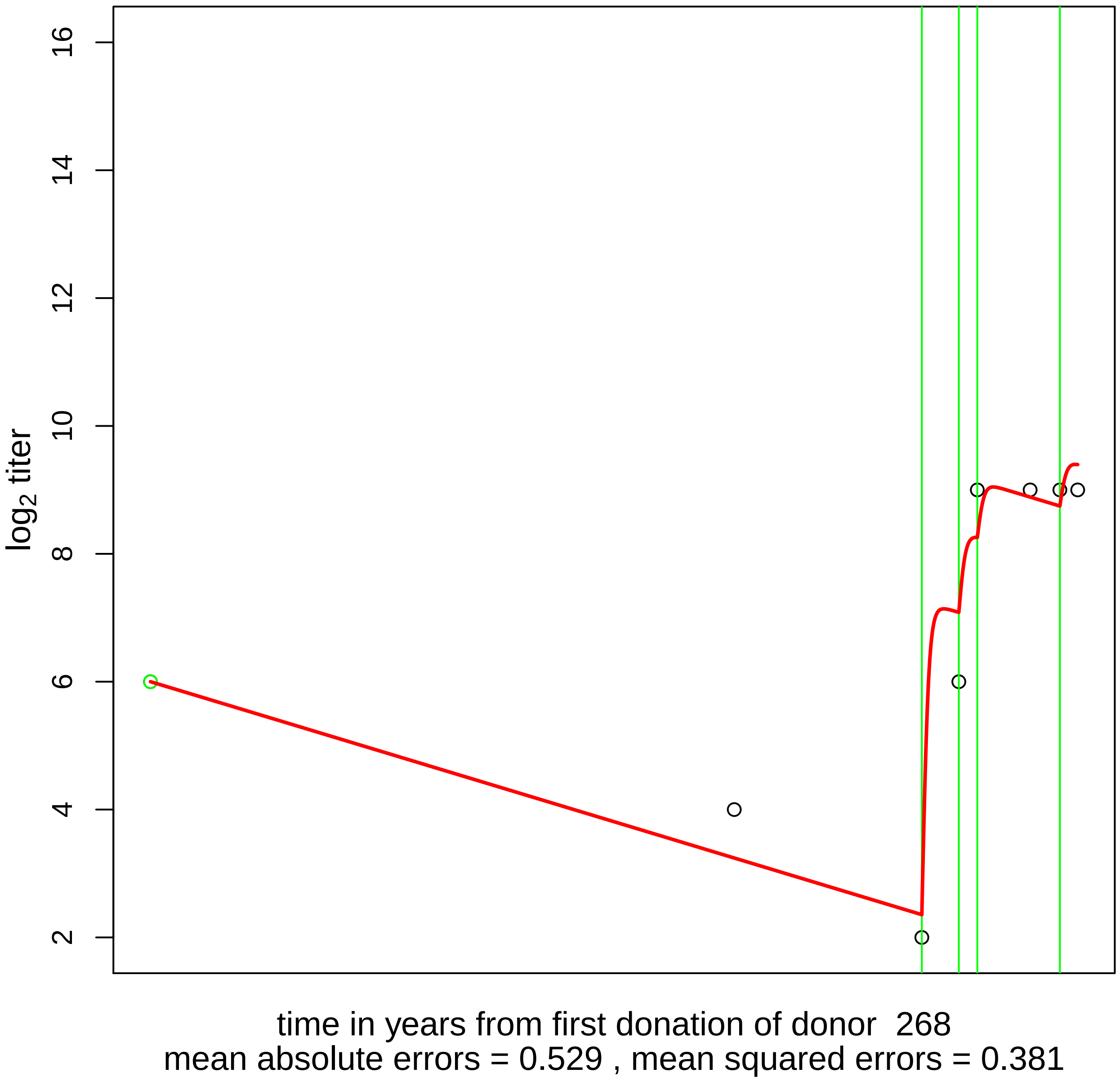
log2 titer
2
4
6
8
10
12
14
16
time in years from first donation of donor 268
mean absolute errors = 0.529 , mean squared errors = 0.381

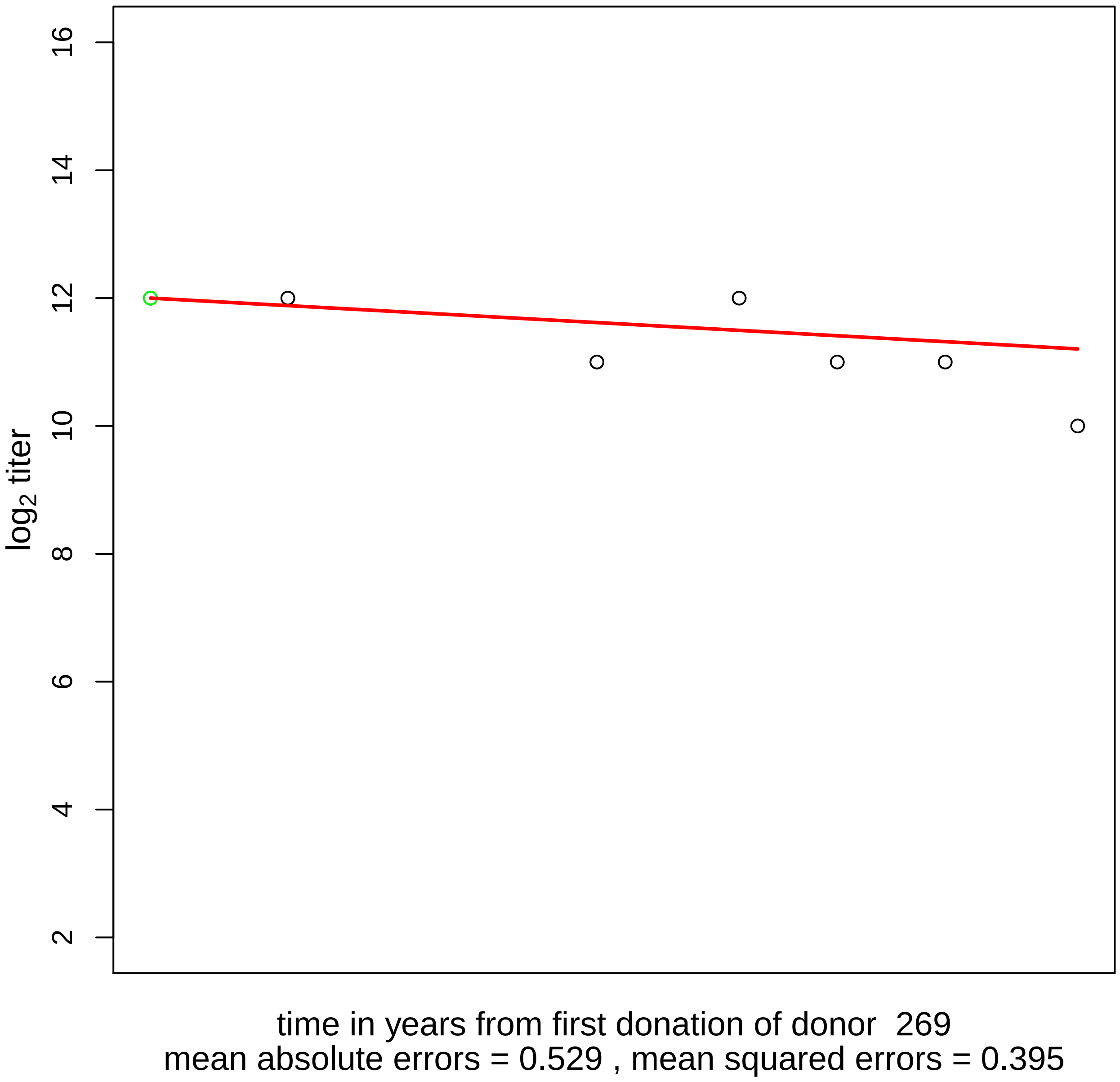
16
14
12
10
8
6
4
2
$\log_2$ titer
time in years from first donation of donor 269
mean absolute errors = 0.529 , mean squared errors = 0.395

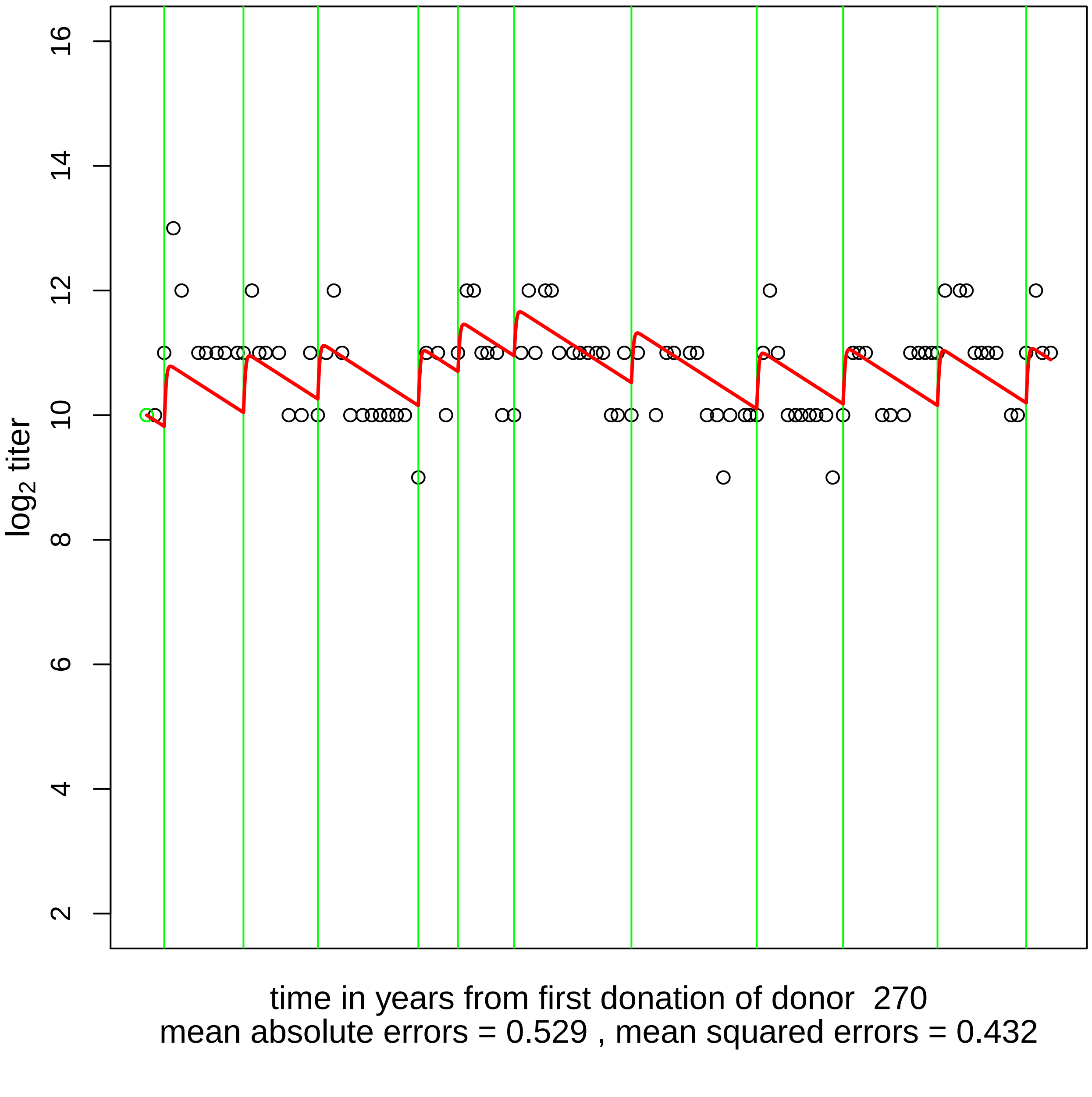

16
14
12
10
8
6
4
2
log2 titer
time in years from first donation of donor 270
mean absolute errors = 0.529 , mean squared errors = 0.432

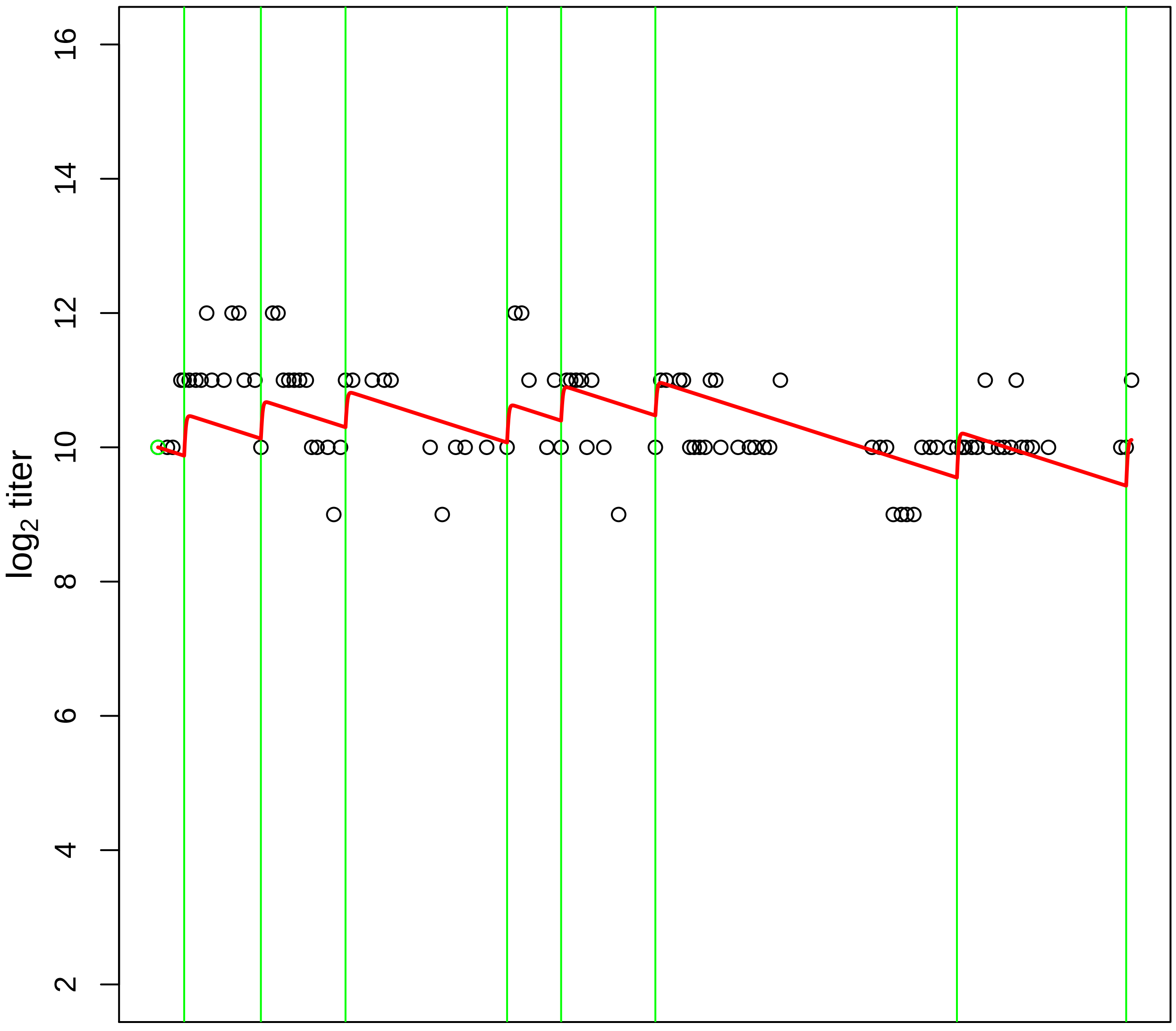

time in years from first donation of donor 271
mean absolute errors = 0.529 , mean squared errors = 0.46

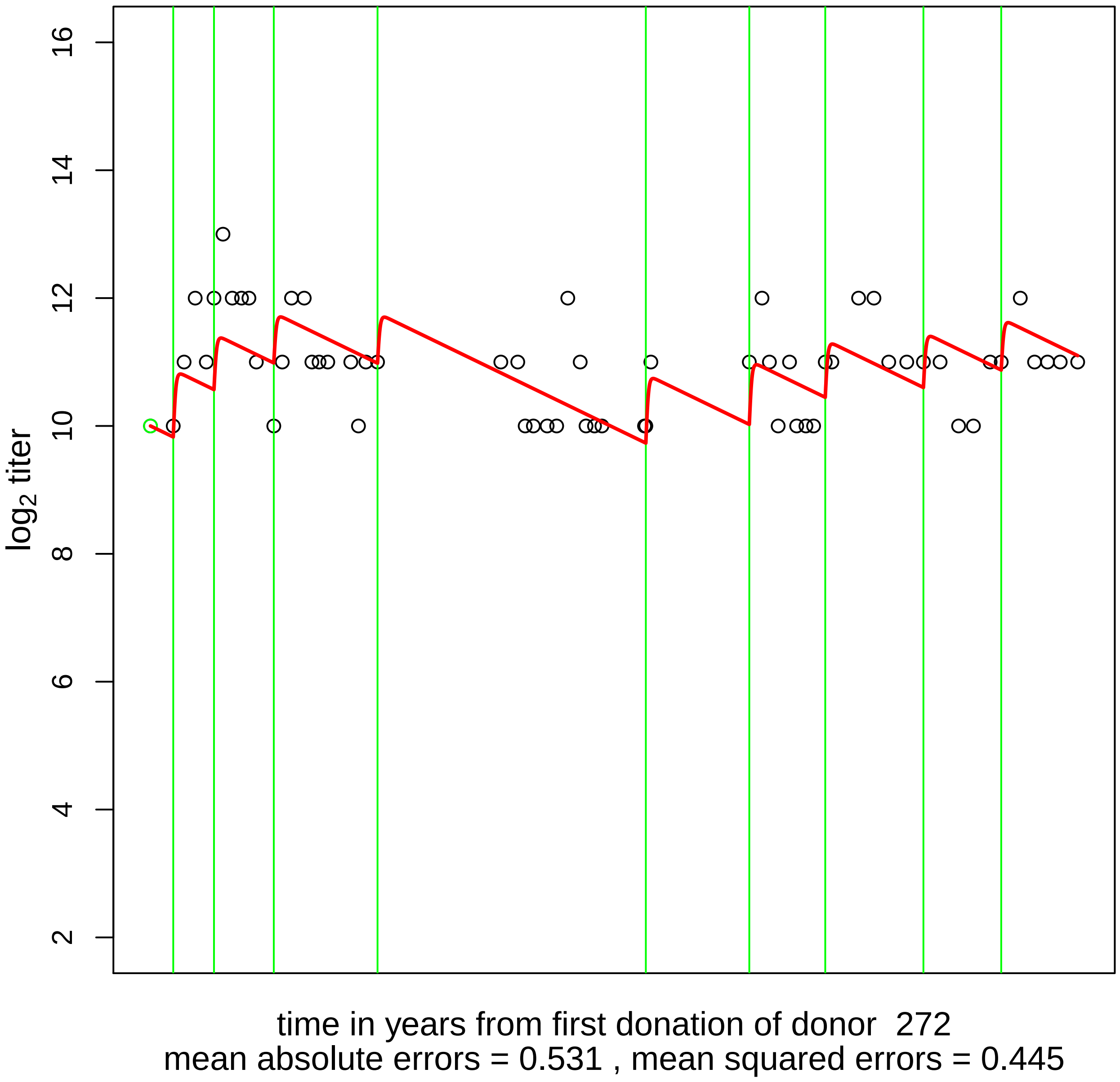

log2 titer
2
4
6
8
10
12
14
16
time in years from first donation of donor 272
mean absolute errors = 0.531 , mean squared errors = 0.445

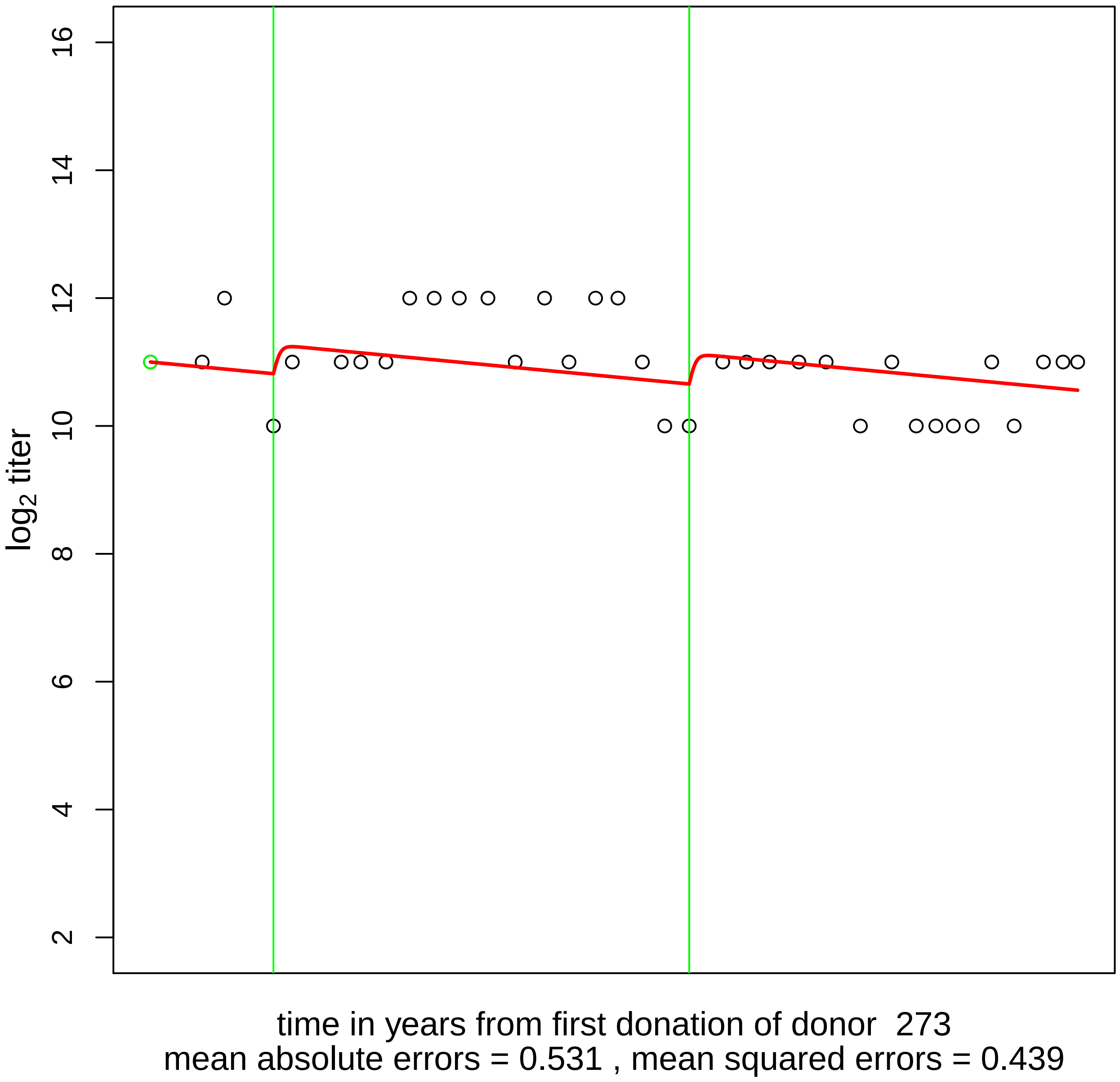

16
14
12
10
8
6
4
2
log2 titer
time in years from first donation of donor 273
mean absolute errors = 0.531 , mean squared errors = 0.439

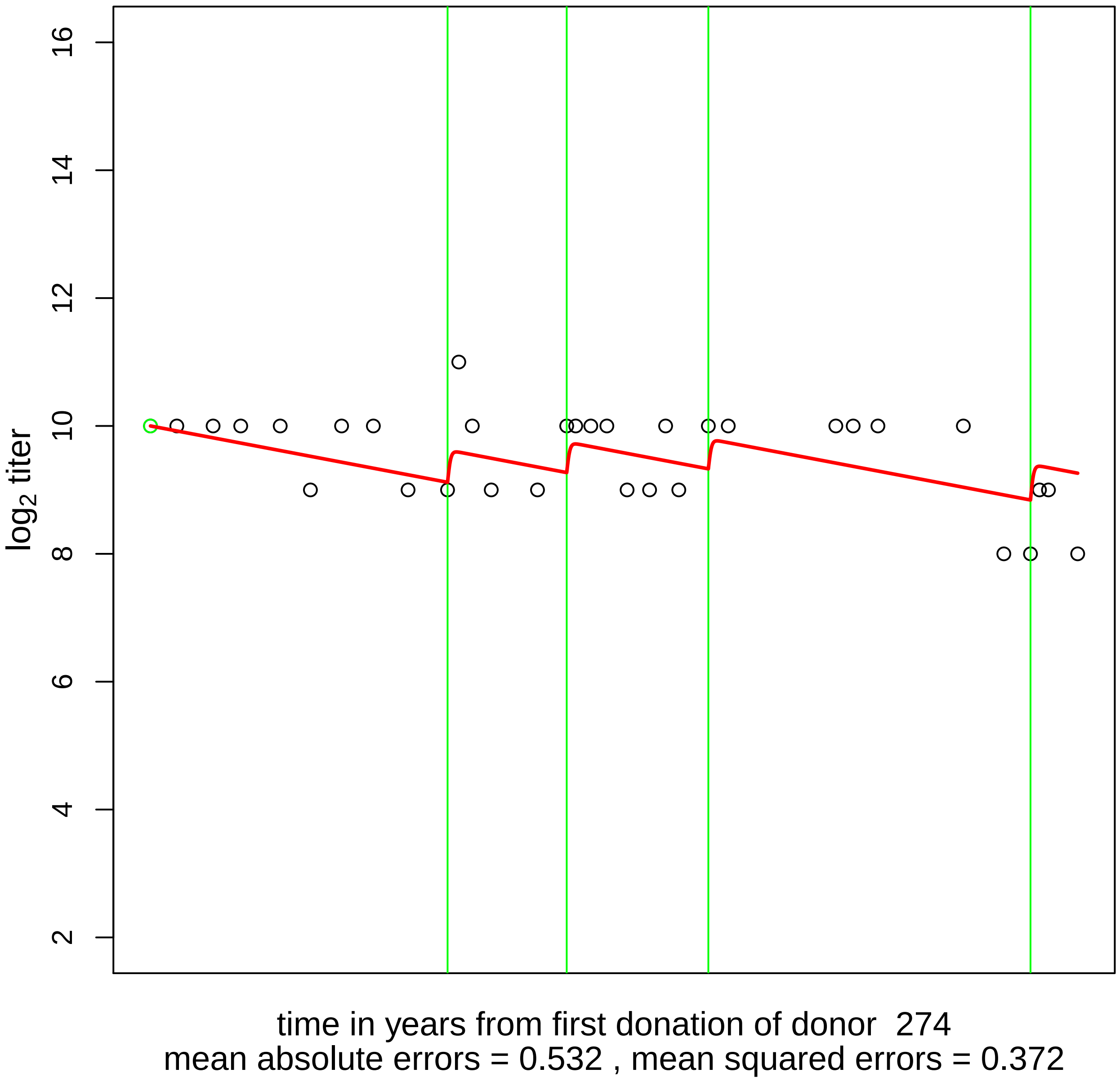

16
14
12
10
8
6
4
2
$\log_2$ titer
time in years from first donation of donor 274
mean absolute errors = 0.532 , mean squared errors = 0.372

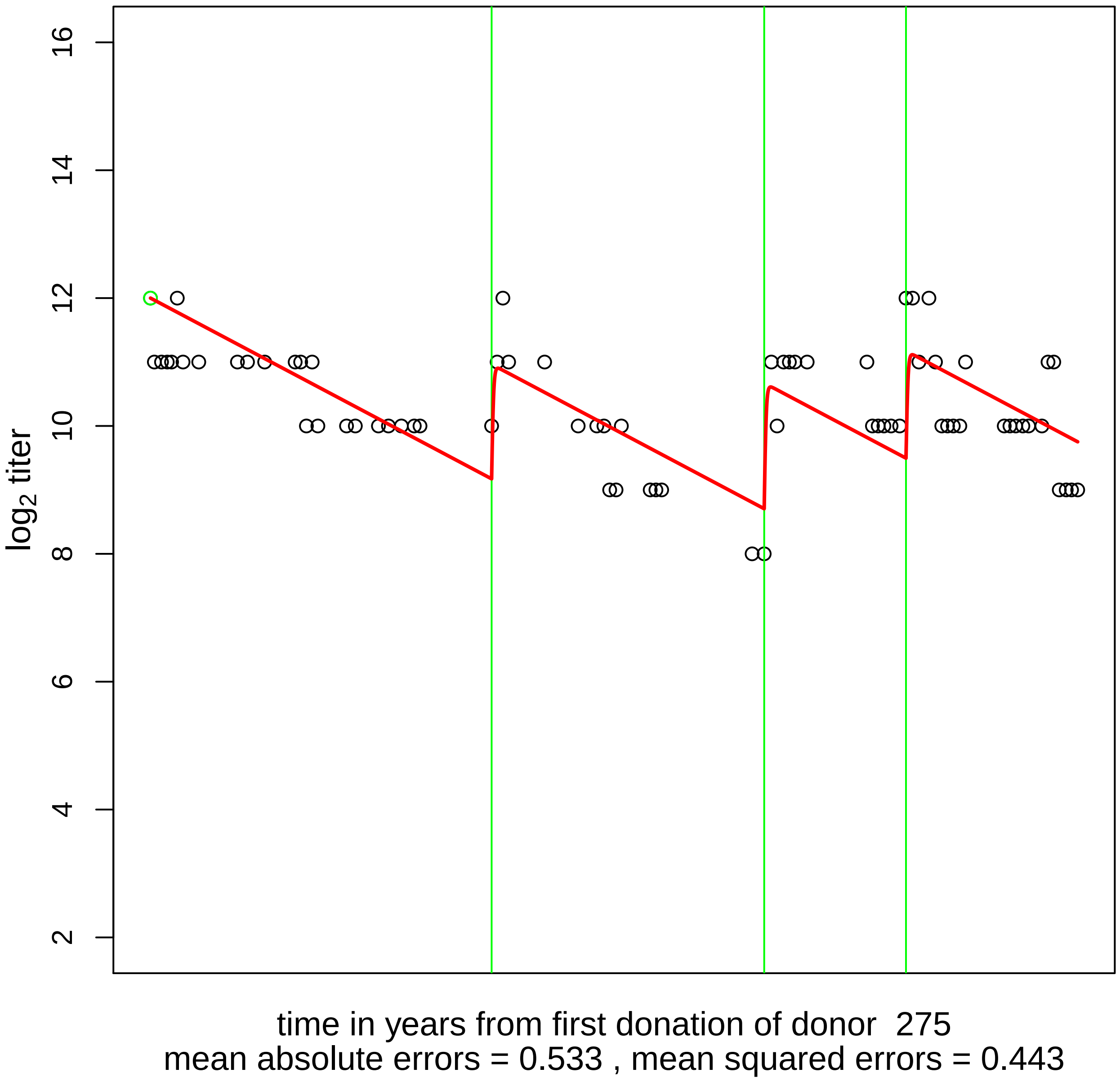

16
14
12
10
8
6
4
2
log2 titer
time in years from first donation of donor 275
mean absolute errors = 0.533 , mean squared errors = 0.443

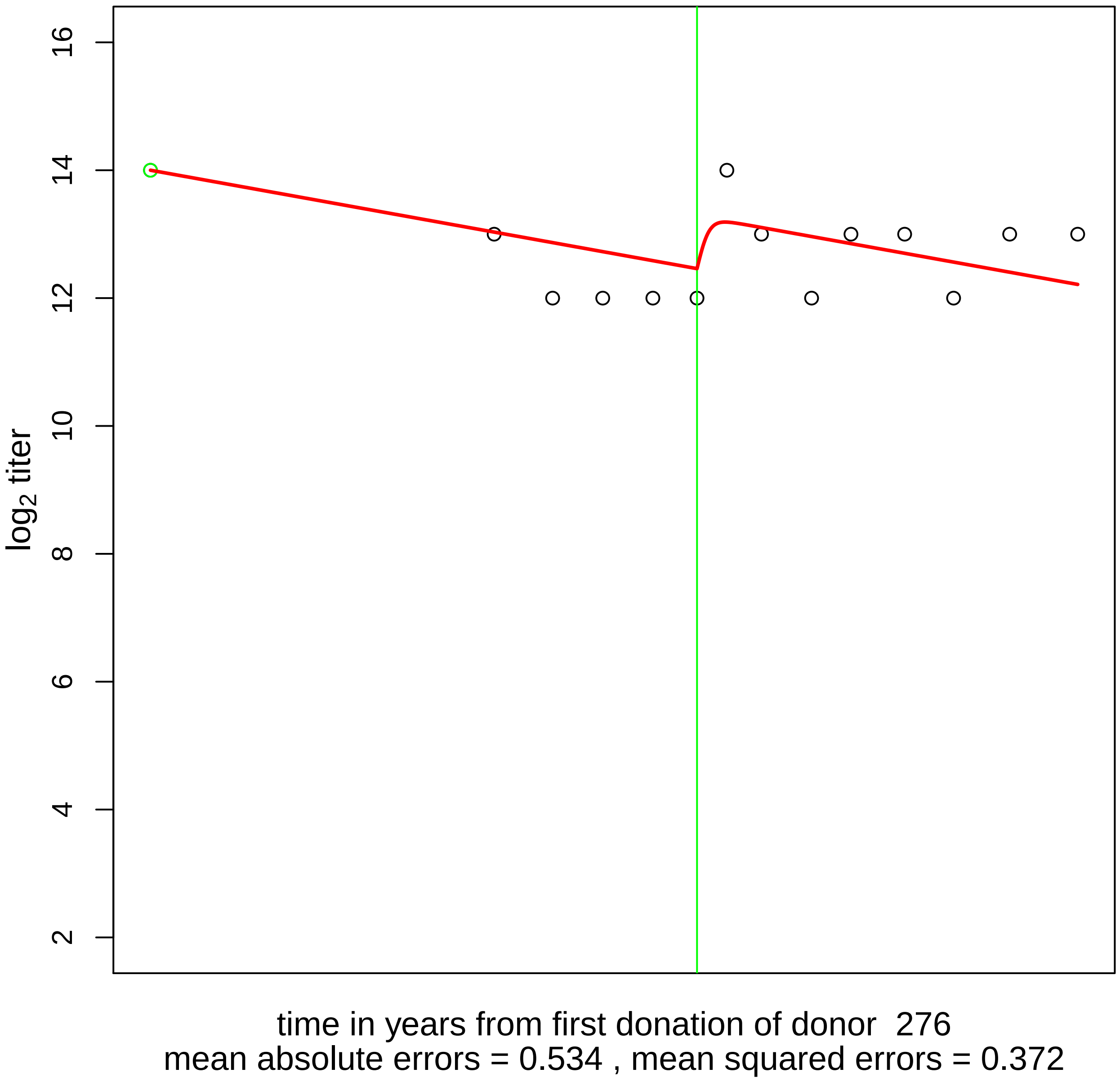
log2 titer
16
14
12
10
8
6
4
2
time in years from first donation of donor 276
mean absolute errors = 0.534 , mean squared errors = 0.372

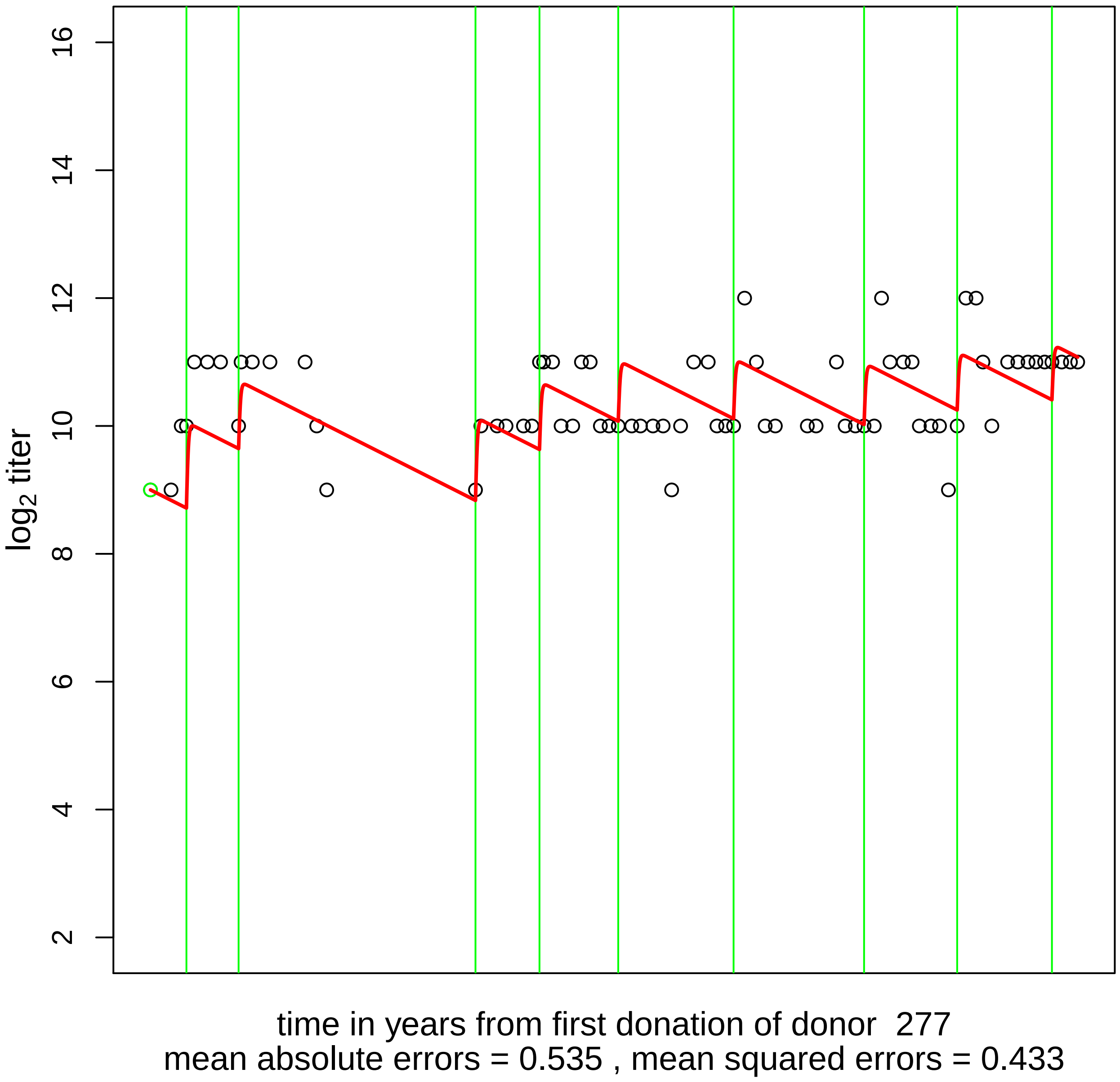

log2 titer
16
14
12
10
8
6
4
2
time in years from first donation of donor 277
mean absolute errors = 0.535 , mean squared errors = 0.433

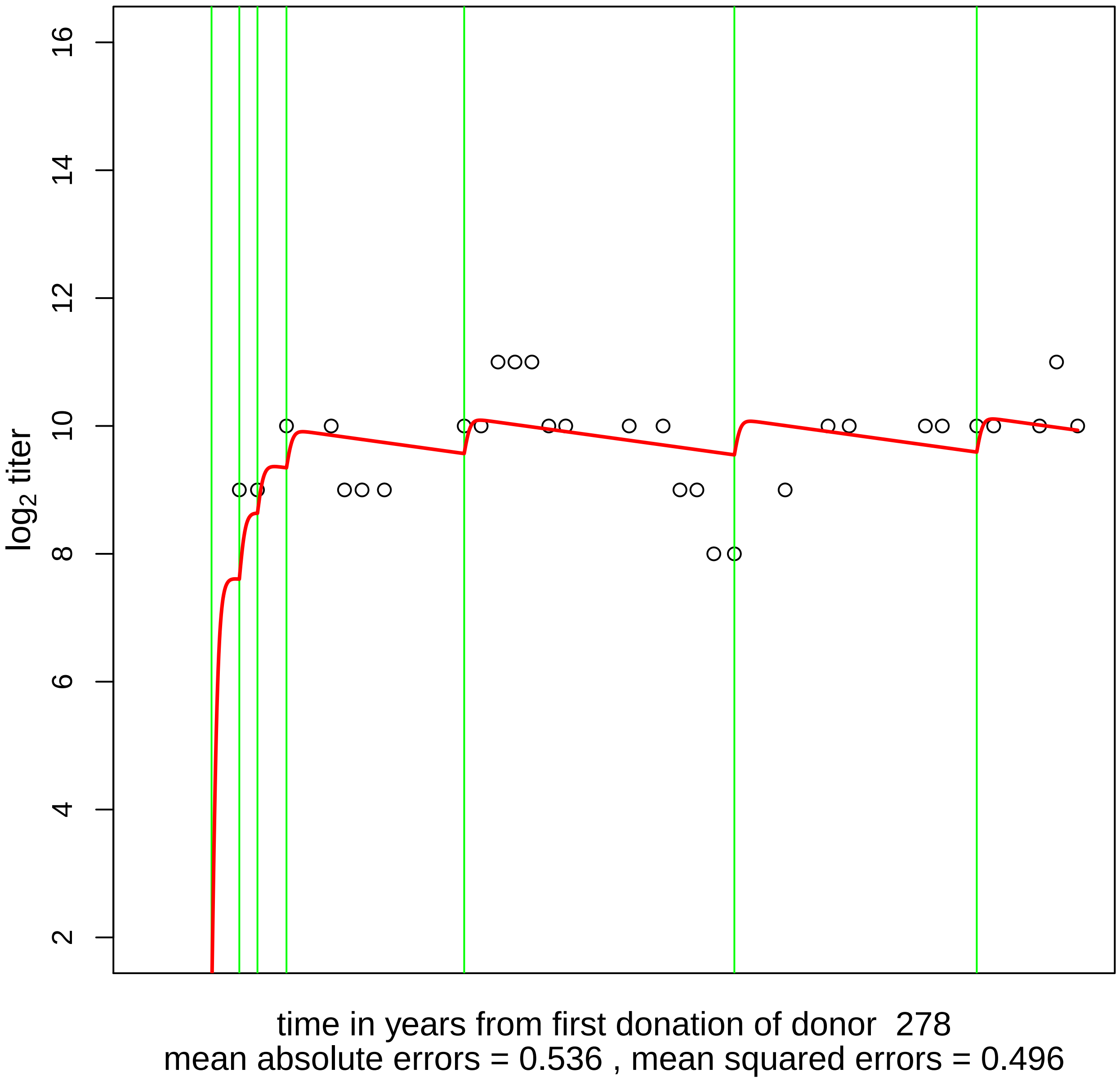

log2 titer
2
4
6
8
10
12
14
16
time in years from first donation of donor 278
mean absolute errors = 0.536 , mean squared errors = 0.496

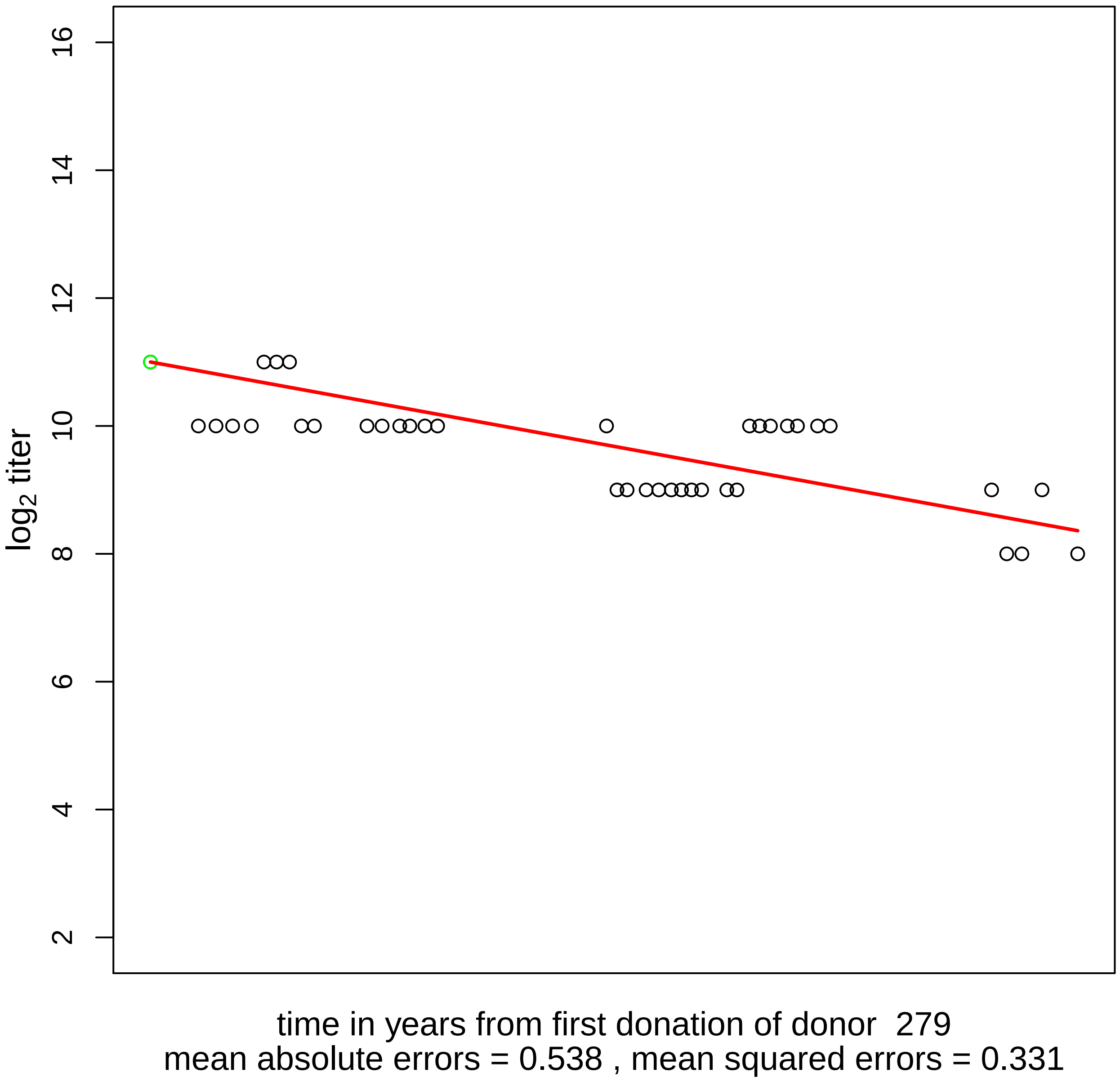

log2 titer
16
14
12
10
8
6
4
2
time in years from first donation of donor 279
mean absolute errors = 0.538 , mean squared errors = 0.331

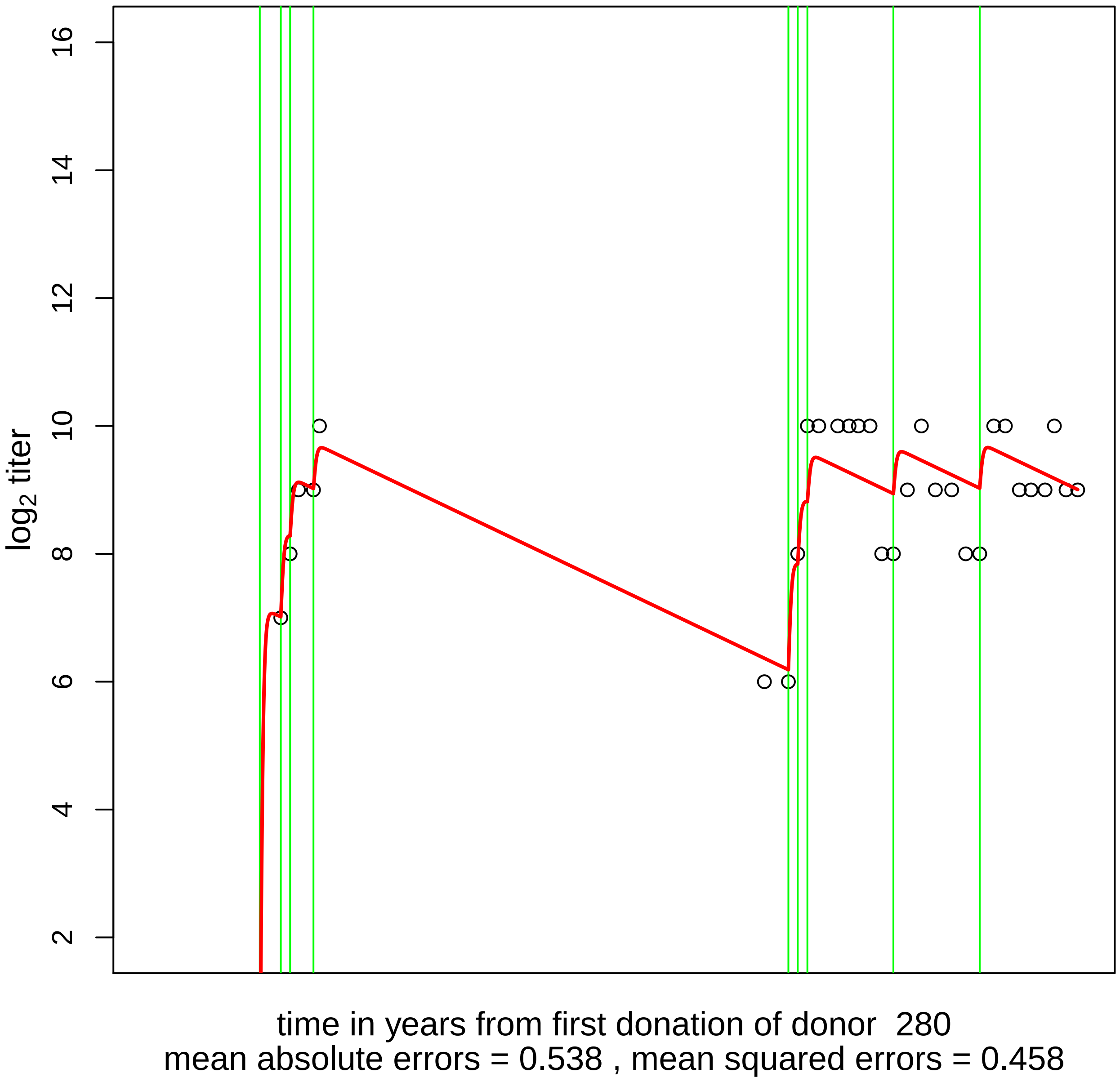

16
14
12
10
8
6
4
2
$\log_2$ titer
time in years from first donation of donor 280
mean absolute errors = 0.538 , mean squared errors = 0.458

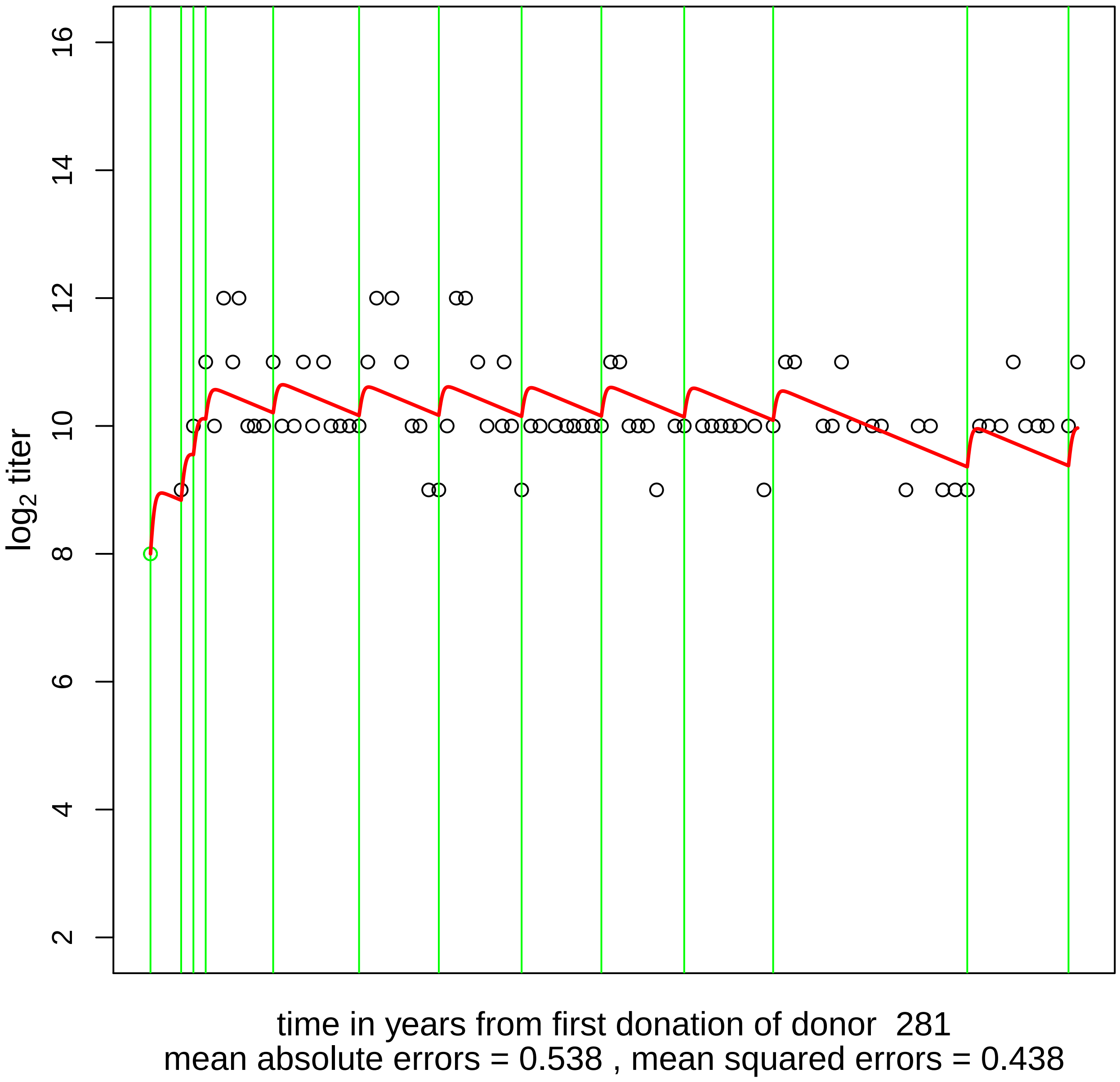

$\log_2$ titer
2
4
6
8
10
12
14
16
time in years from first donation of donor 281
mean absolute errors = 0.538 , mean squared errors = 0.438

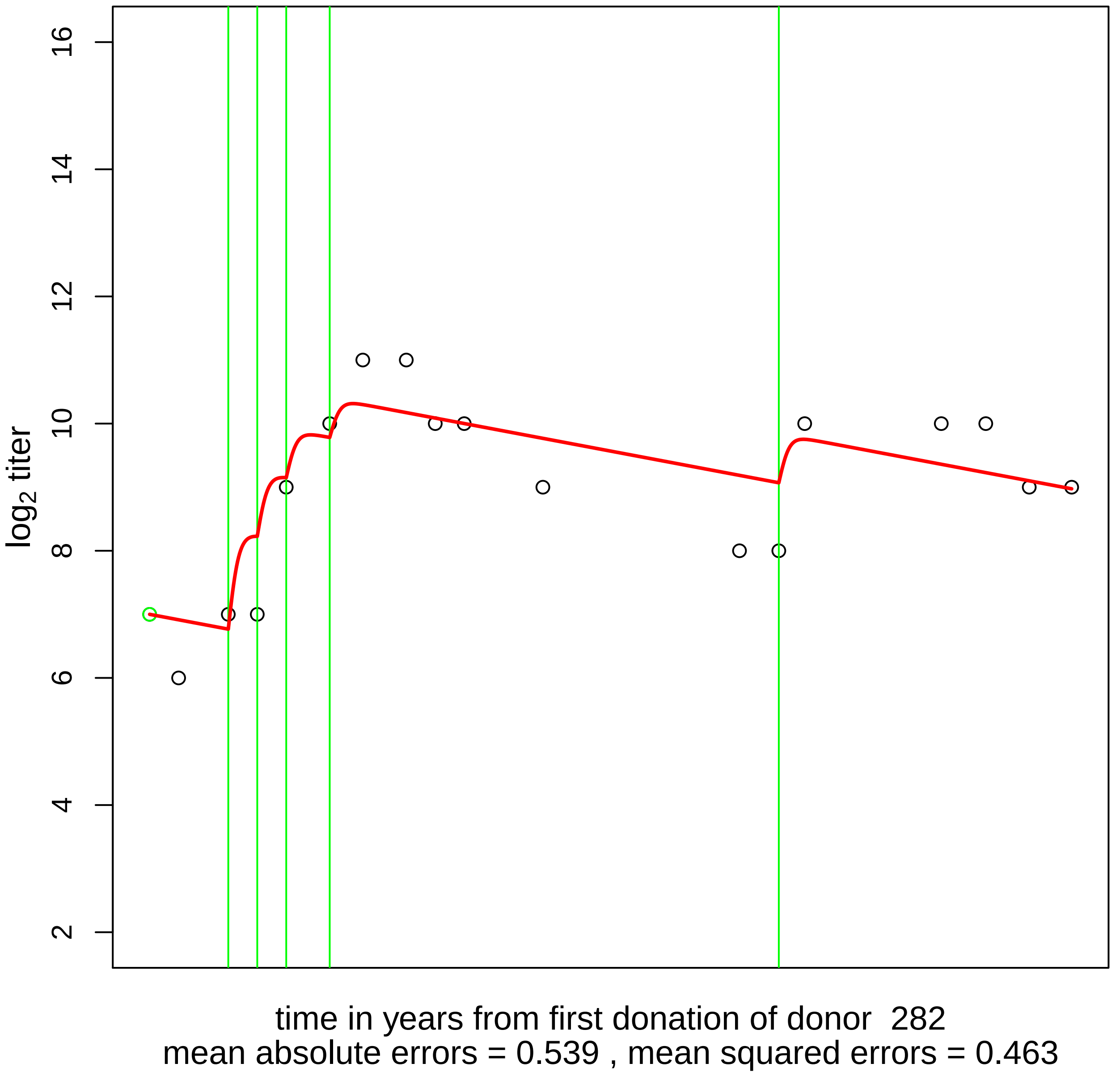

log2 titer
16
14
12
10
8
6
4
2
time in years from first donation of donor 282
mean absolute errors = 0.539 , mean squared errors = 0.463

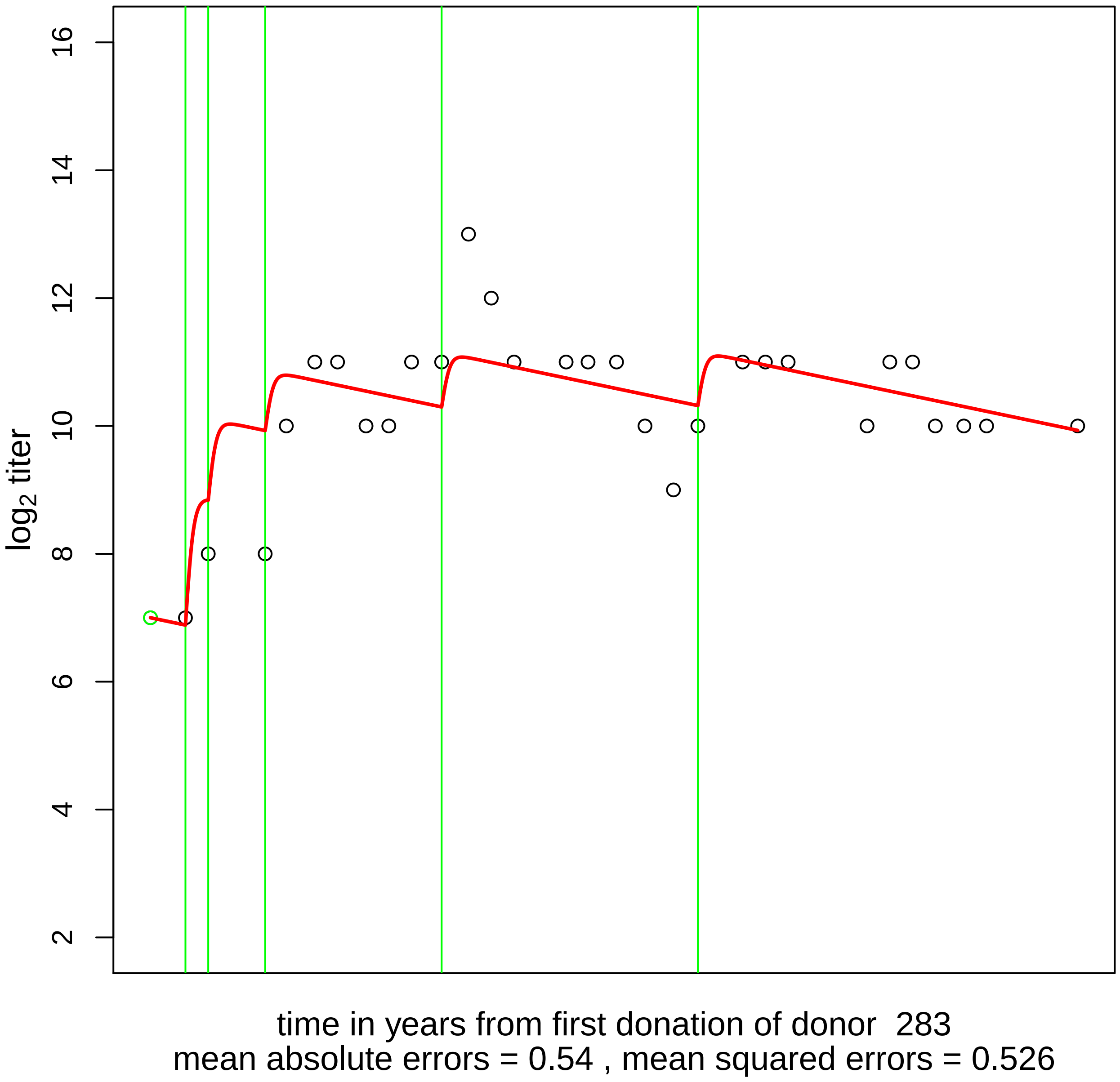

log2 titer
16
14
12
10
8
6
4
2
time in years from first donation of donor 283
mean absolute errors = 0.54 , mean squared errors = 0.526

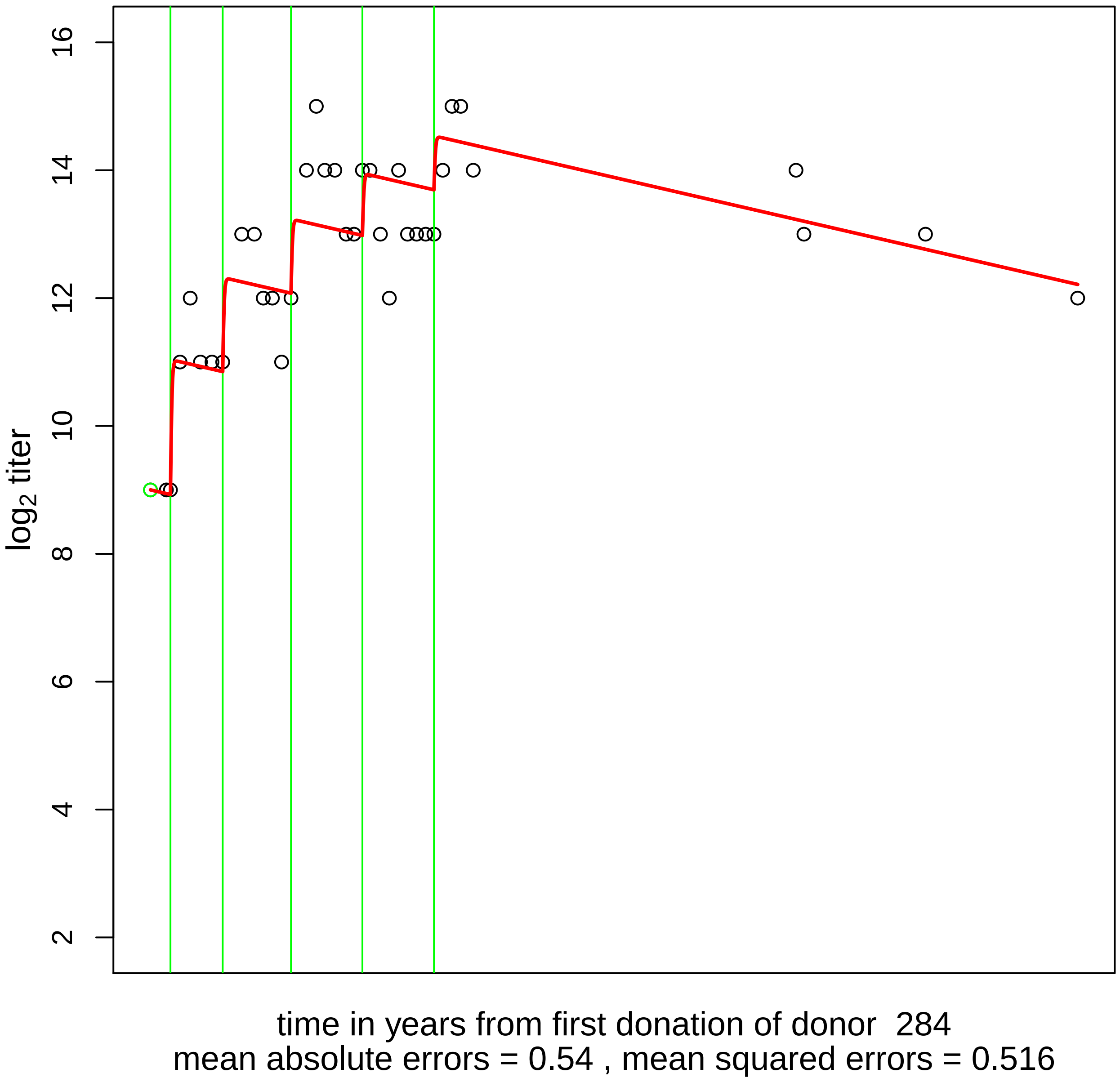

2
4
6
8
10
12
14
16
log2 titer
time in years from first donation of donor 284
mean absolute errors = 0.54 , mean squared errors = 0.516

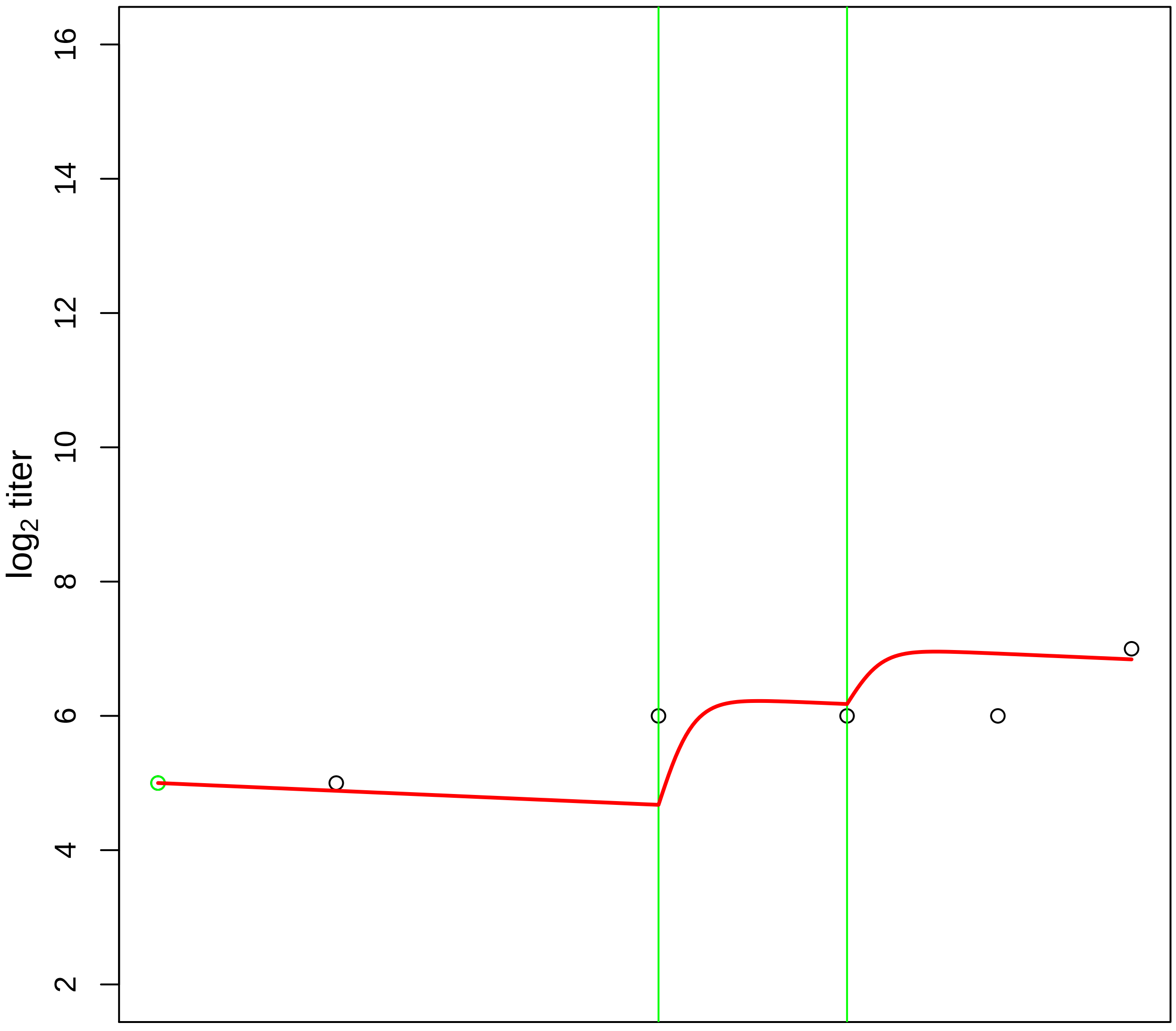

time in years from first donation of donor 285
mean absolute errors = 0.541 , mean squared errors = 0.537

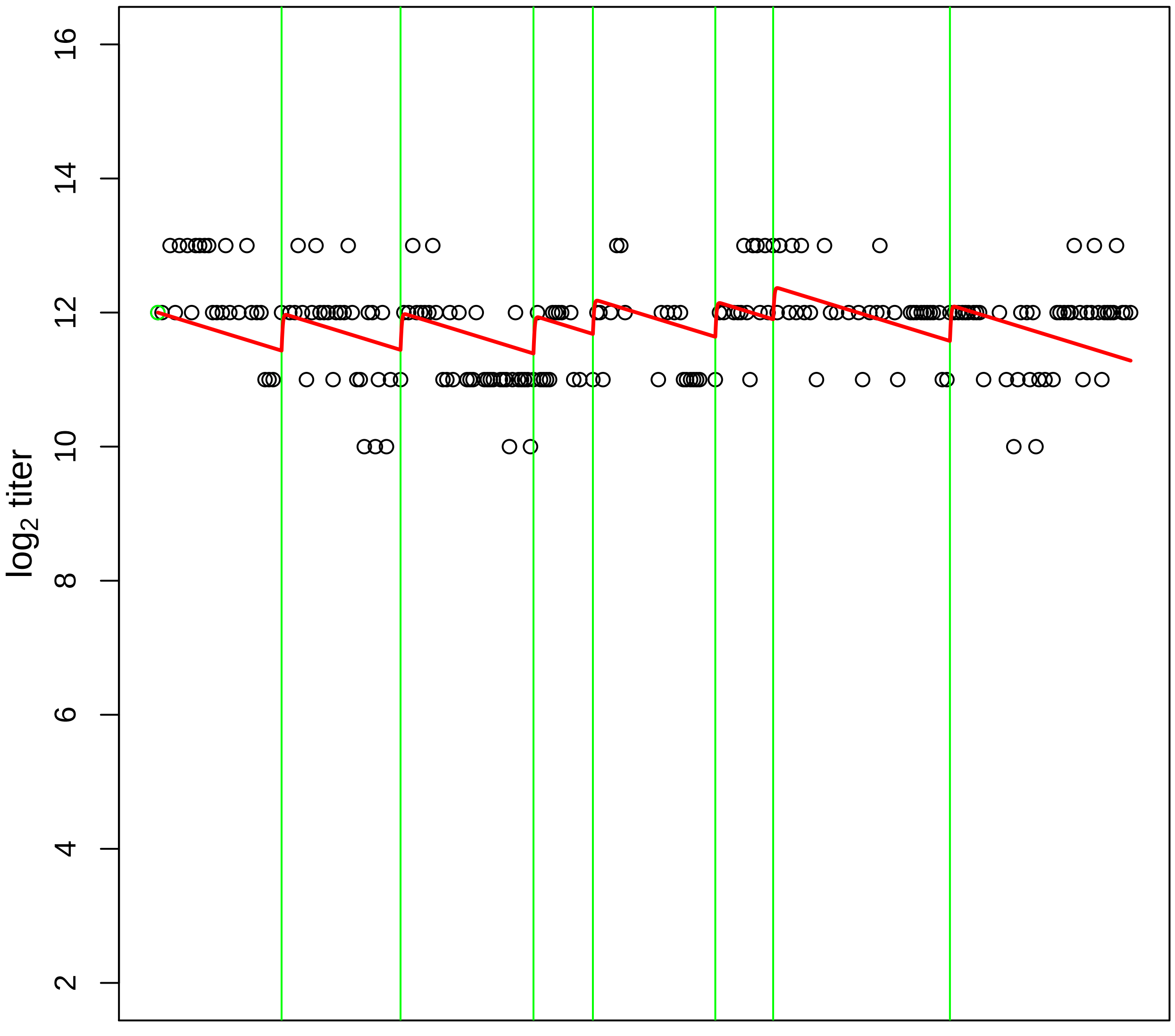

time in years from first donation of donor 286
mean absolute errors = 0.542 , mean squared errors = 0.465

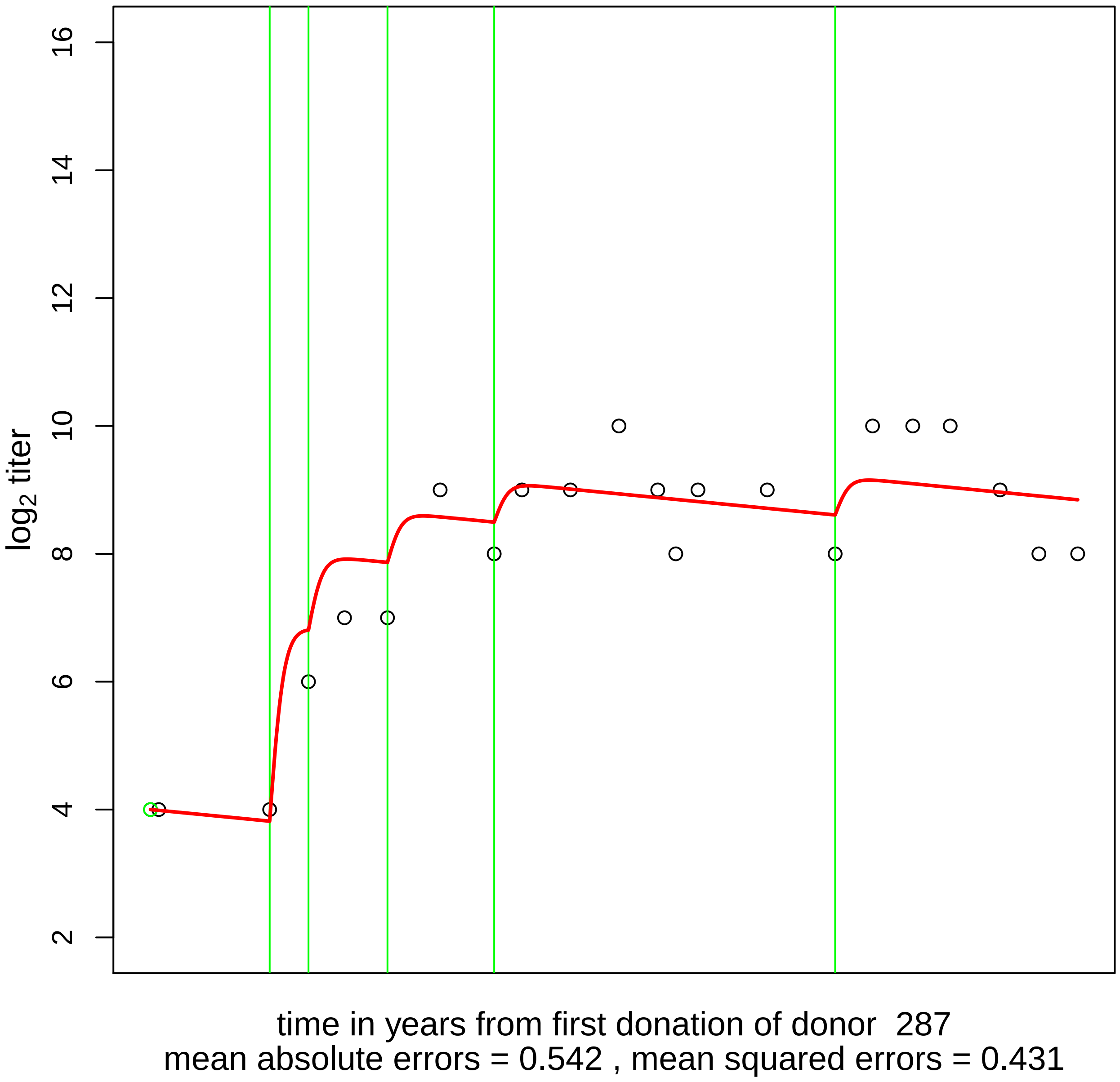

16
14
12
10
8
6
4
2
log2 titer
time in years from first donation of donor 287
mean absolute errors = 0.542 , mean squared errors = 0.431

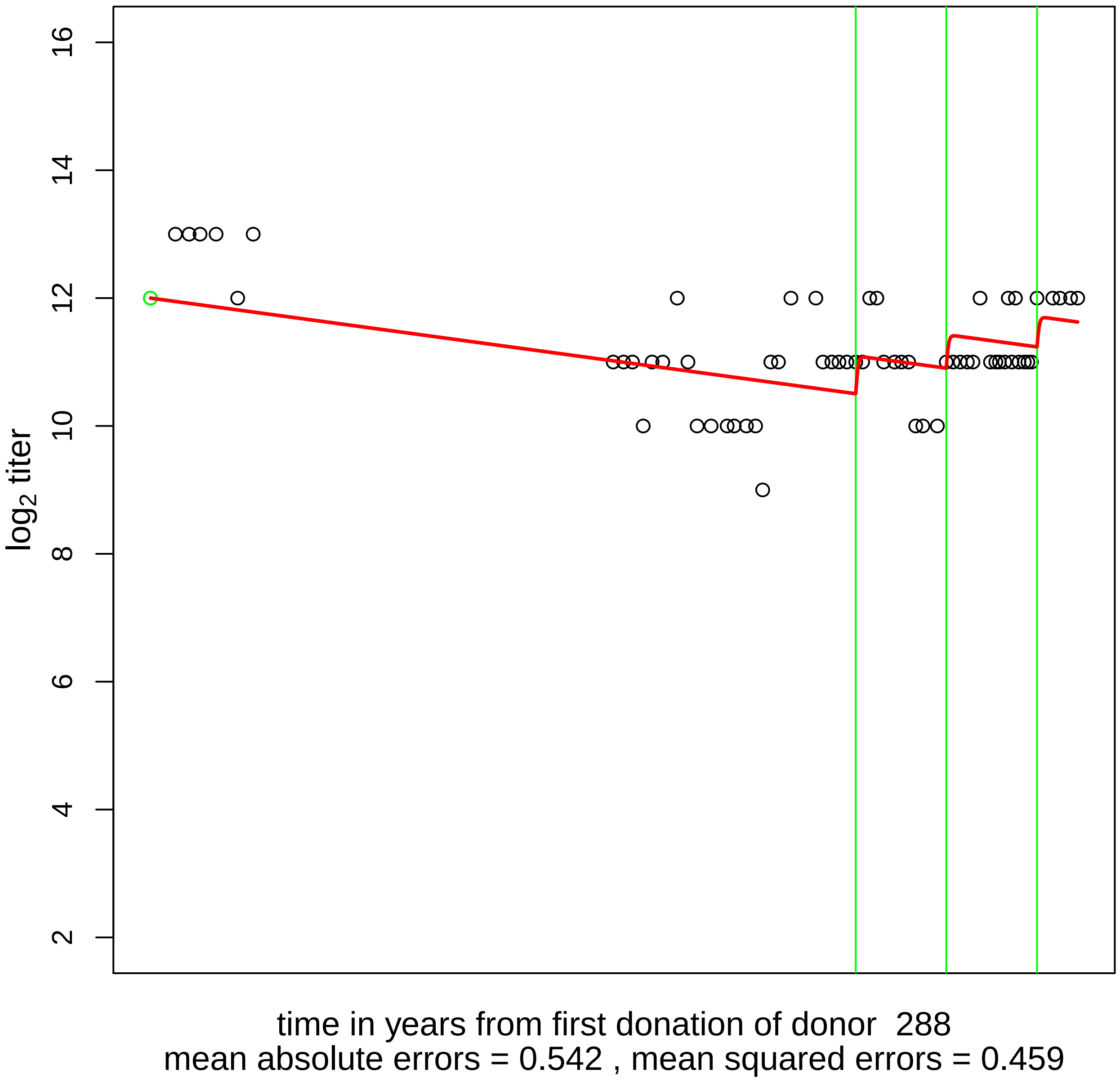

16
14
12
10
8
6
4
2
log2 titer
time in years from first donation of donor 288
mean absolute errors = 0.542 , mean squared errors = 0.459

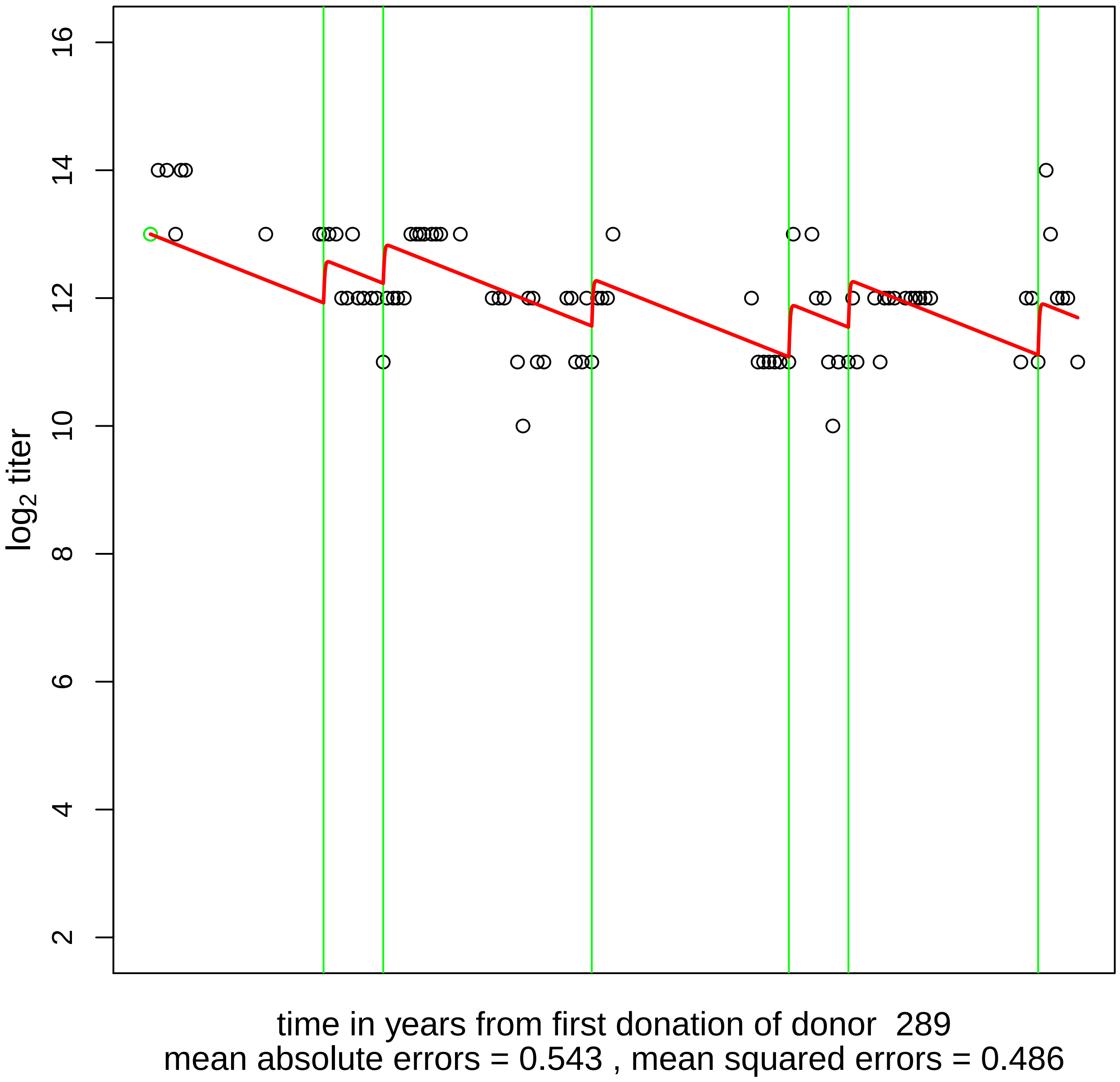

log2 titer
16
14
12
10
8
6
4
2
time in years from first donation of donor 289
mean absolute errors = 0.543 , mean squared errors = 0.486

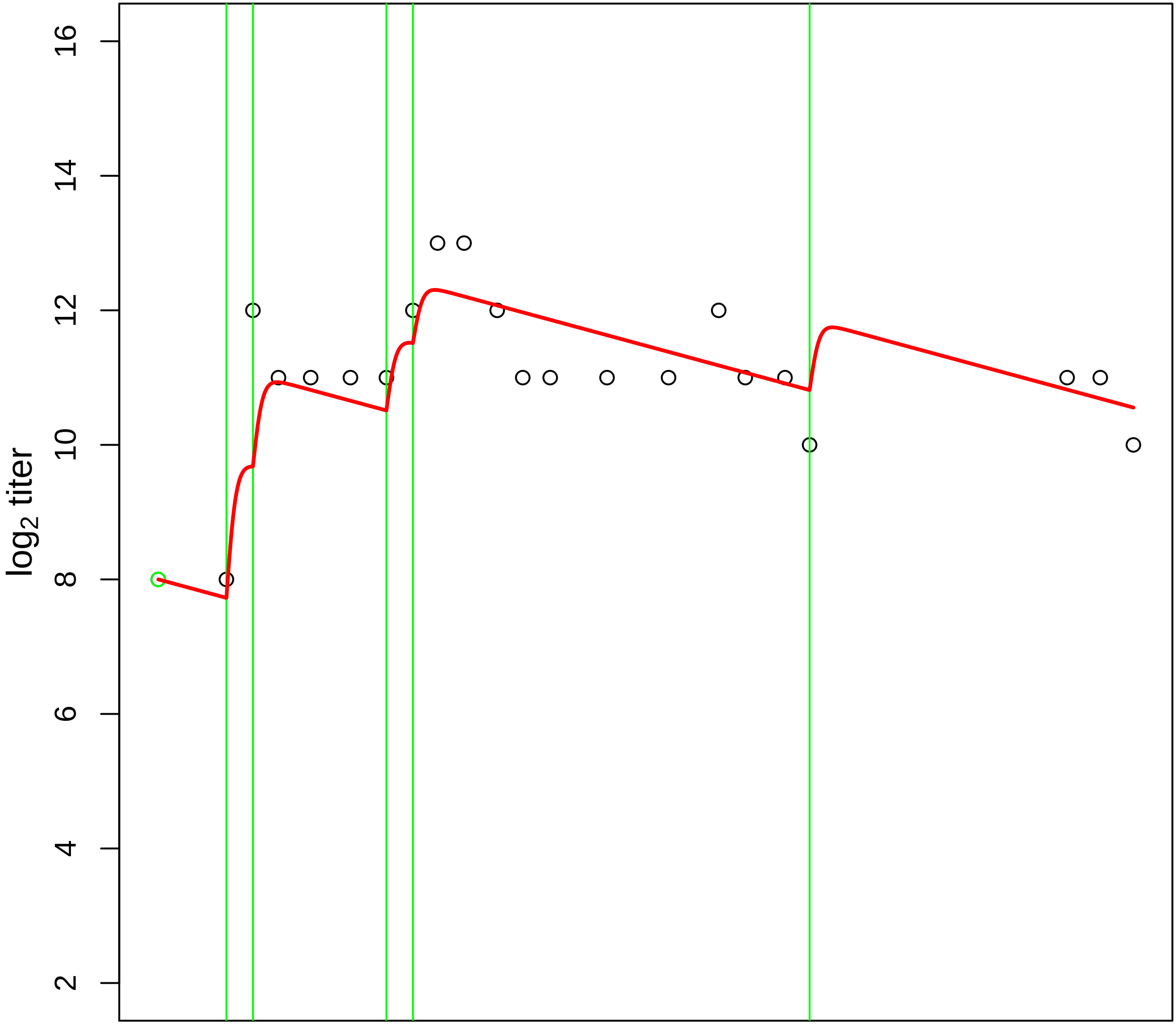

time in years from first donation of donor 290
mean absolute errors = 0.544 , mean squared errors = 0.535

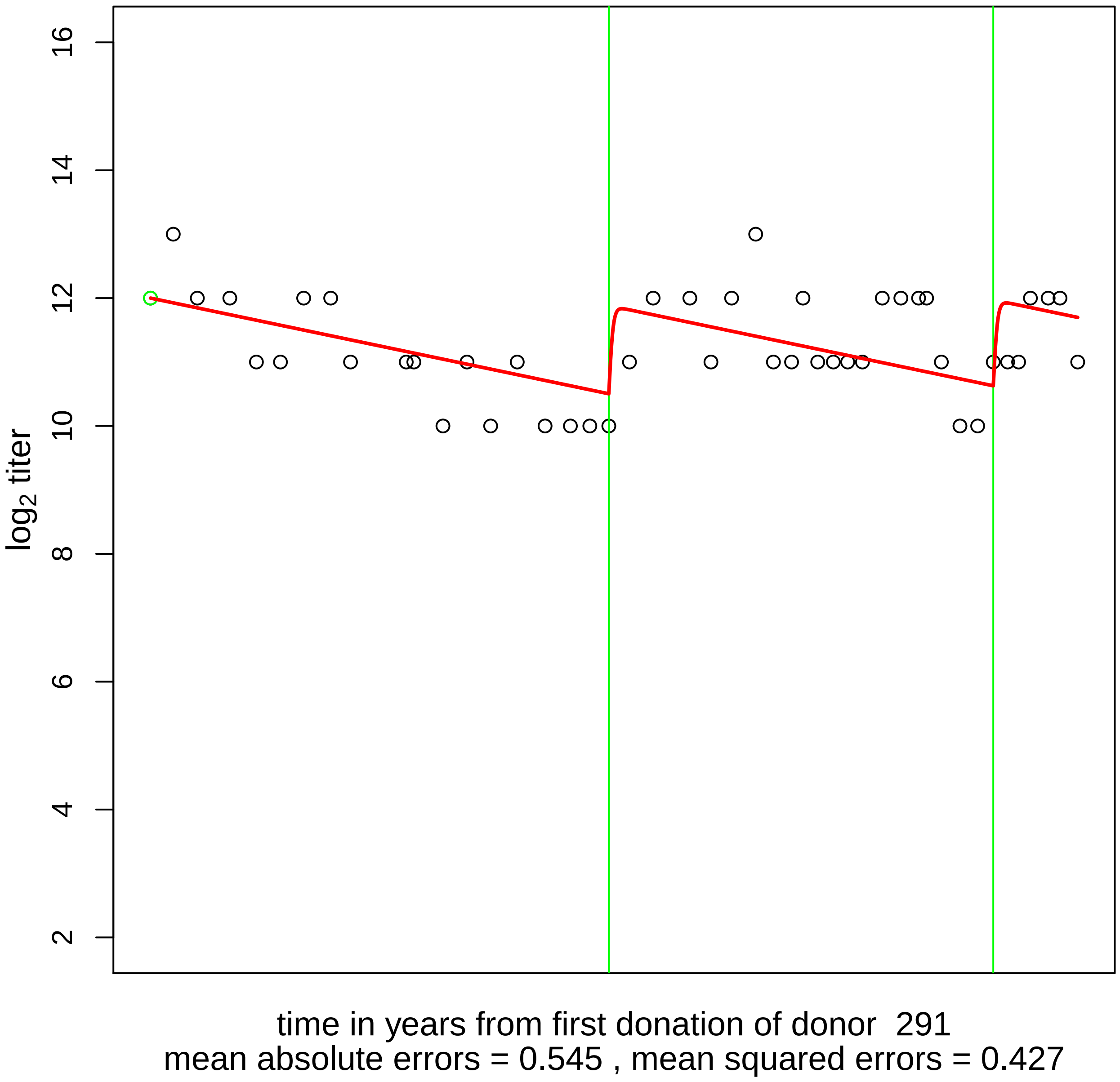

16
14
12
10
8
6
4
2
log2 titer
time in years from first donation of donor 291
mean absolute errors = 0.545 , mean squared errors = 0.427

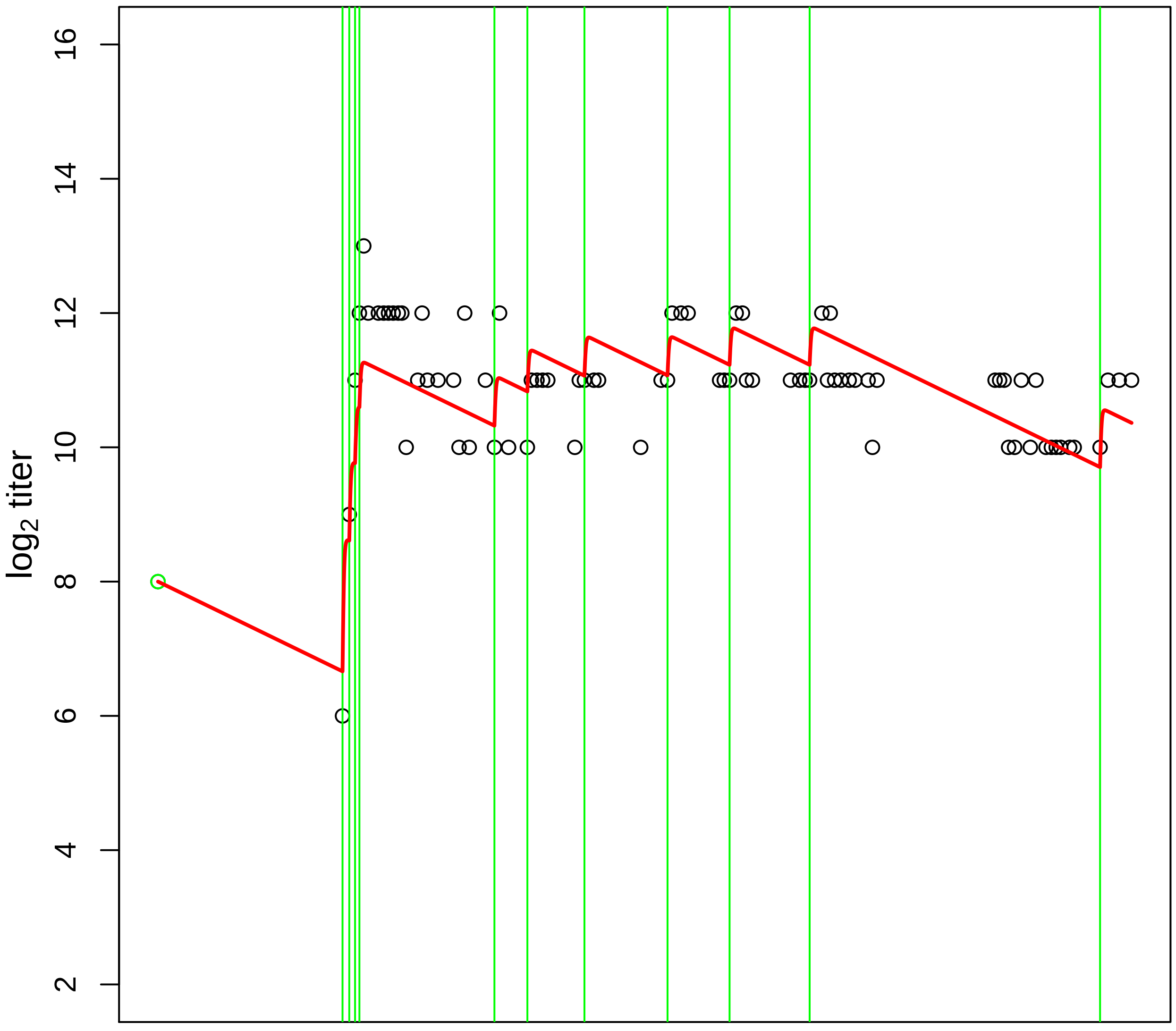

time in years from first donation of donor 292
mean absolute errors = 0.546 , mean squared errors = 0.437

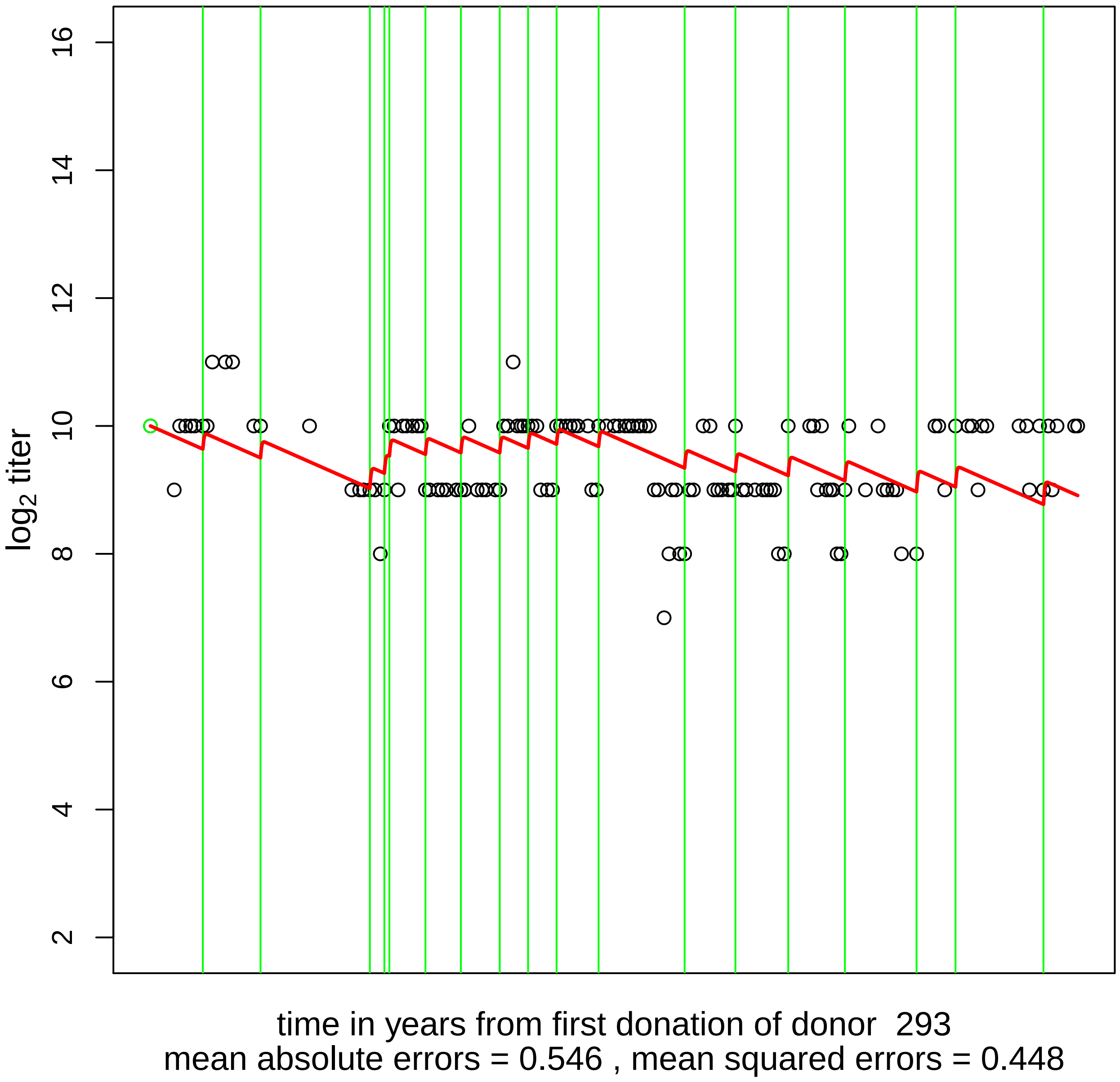

log2 titer
2
4
6
8
10
12
14
16
time in years from first donation of donor 293
mean absolute errors = 0.546 , mean squared errors = 0.448

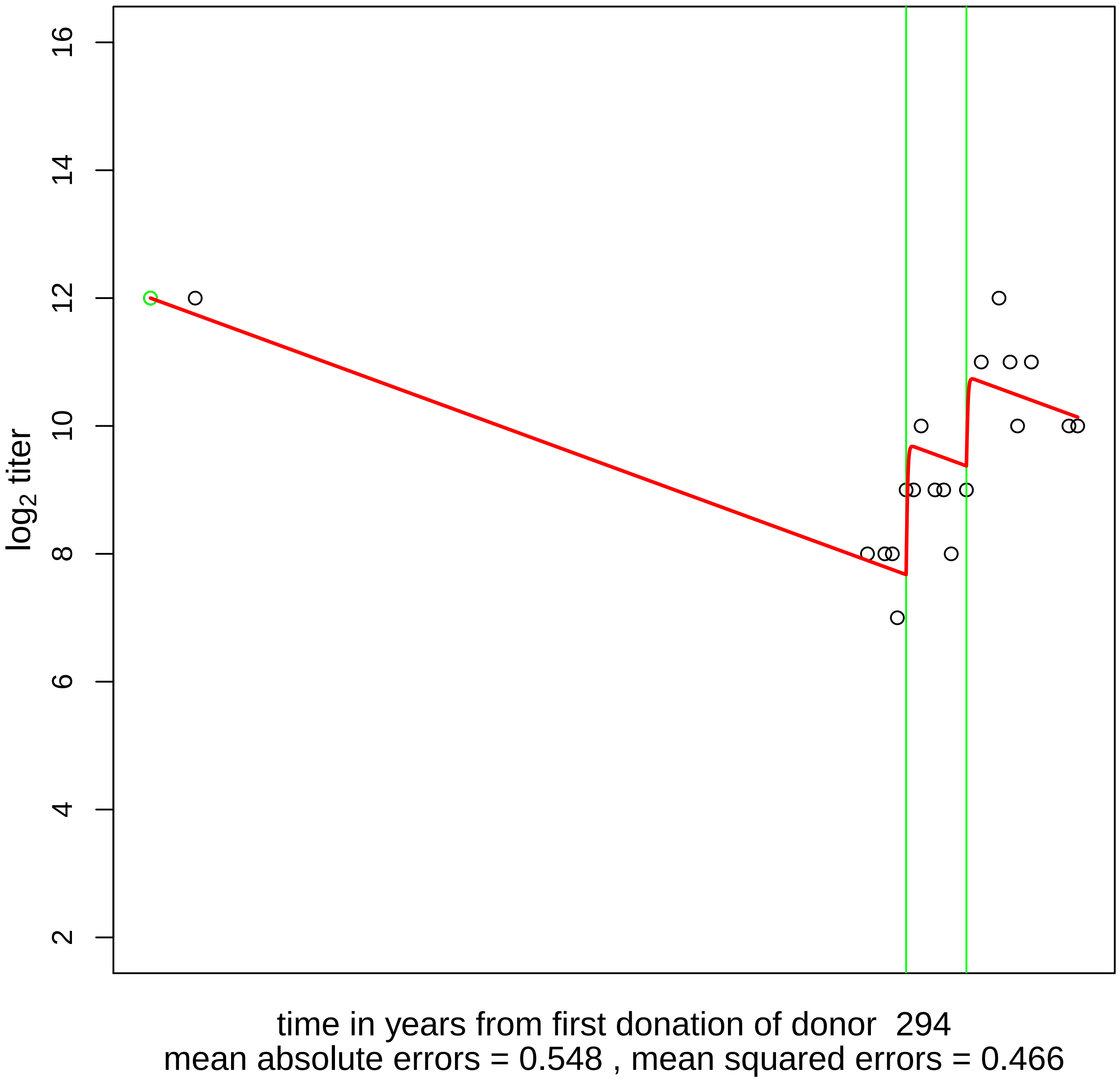
16
14
12
10
8
6
4
2
log2 titer
time in years from first donation of donor 294
mean absolute errors = 0.548 , mean squared errors = 0.466

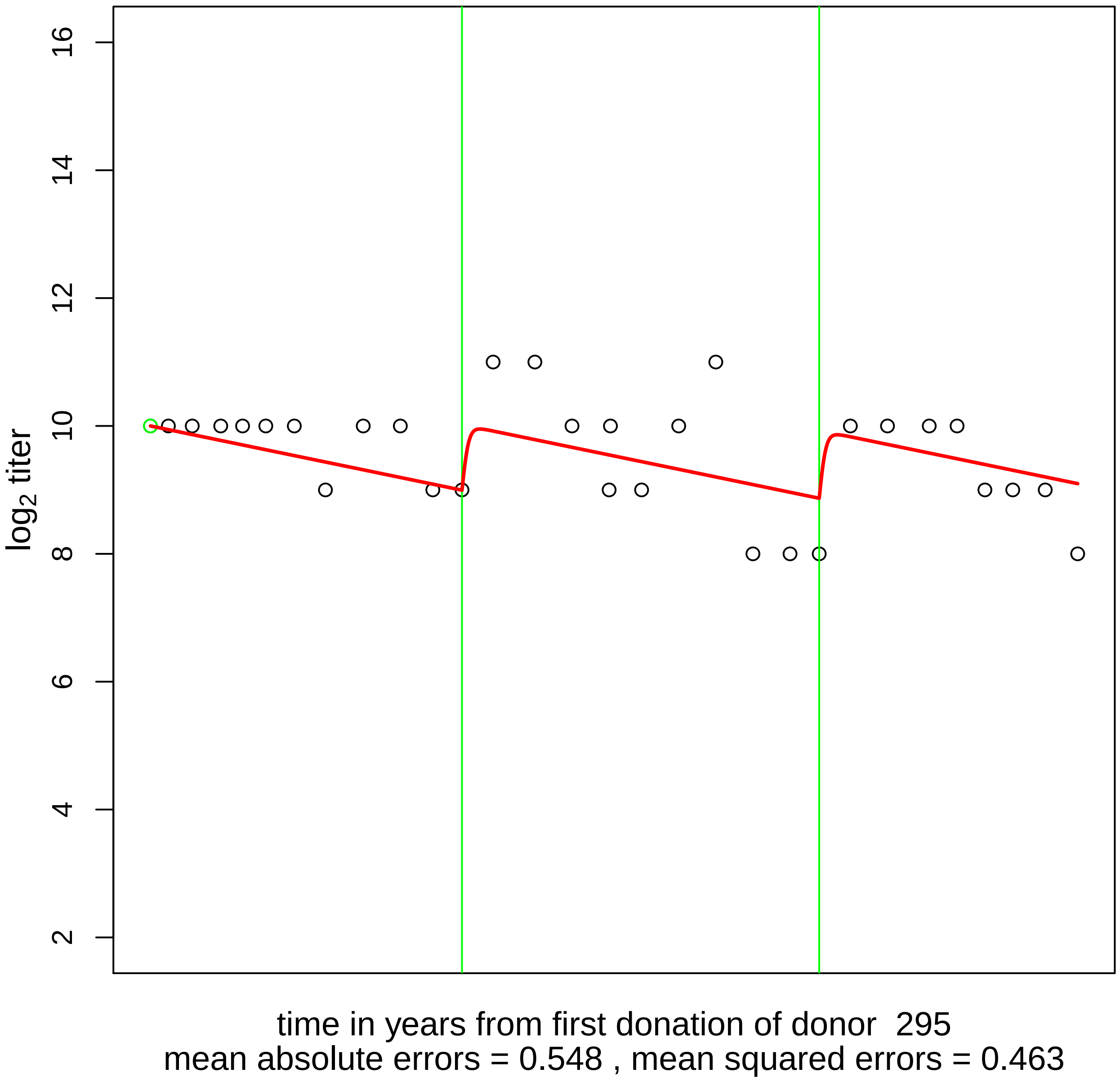
log2 titer
16
14
12
10
8
6
4
2
time in years from first donation of donor 295
mean absolute errors = 0.548 , mean squared errors = 0.463

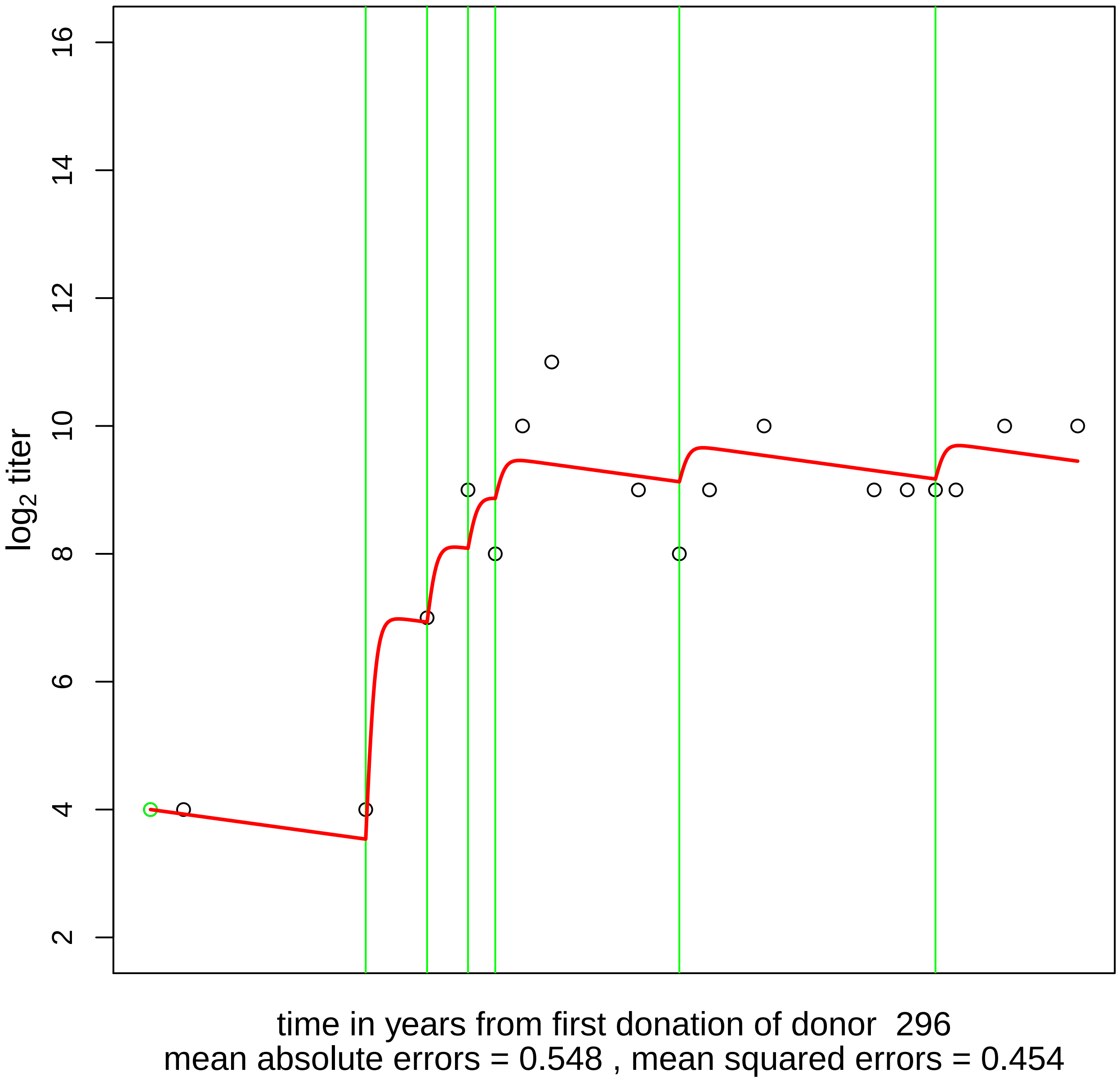

log2 titer
16
14
12
10
8
6
4
2
time in years from first donation of donor 296
mean absolute errors = 0.548 , mean squared errors = 0.454

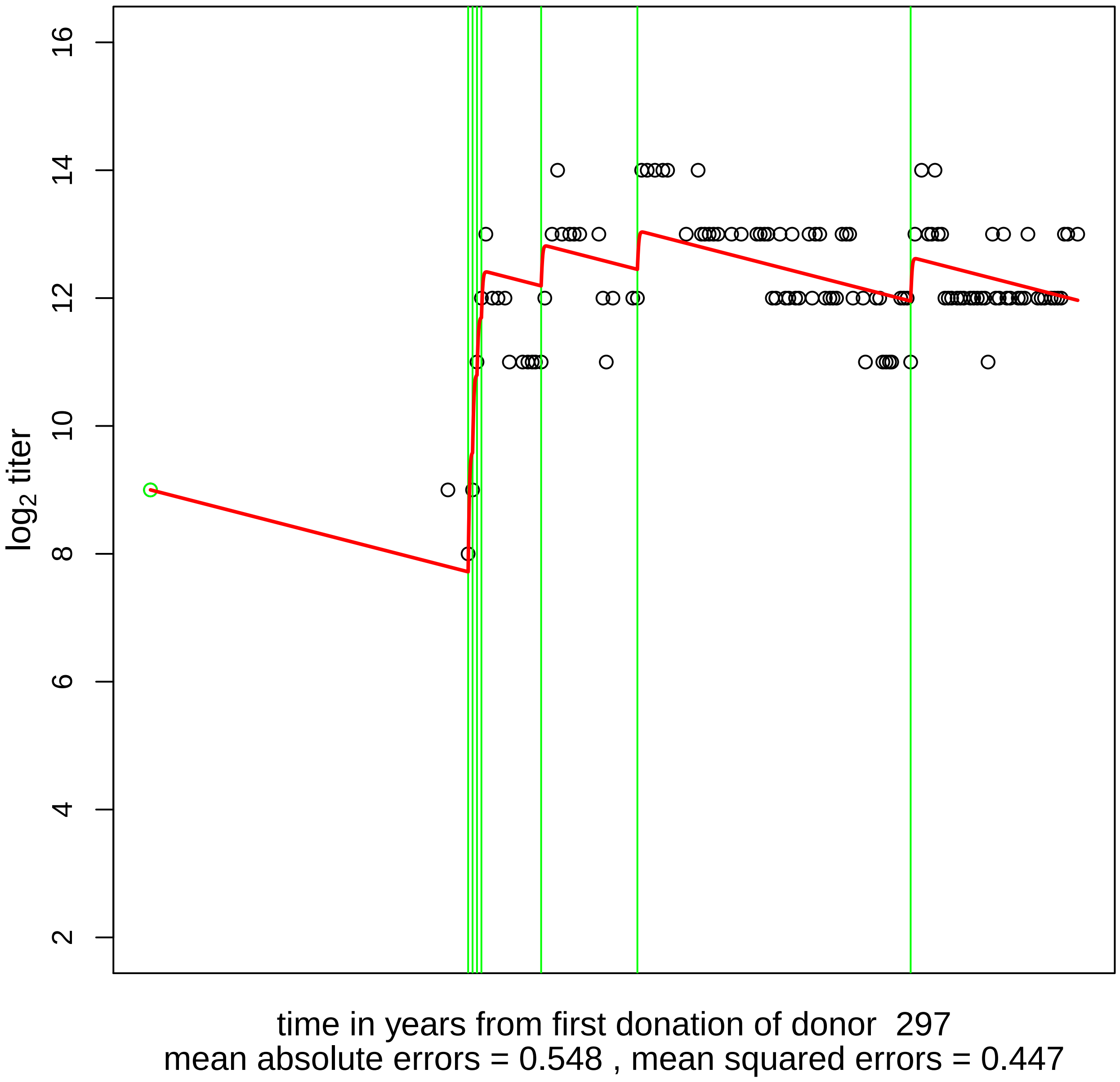

16
14
12
10
8
6
4
2
$\log_2$ titer
time in years from first donation of donor 297
mean absolute errors = 0.548 , mean squared errors = 0.447

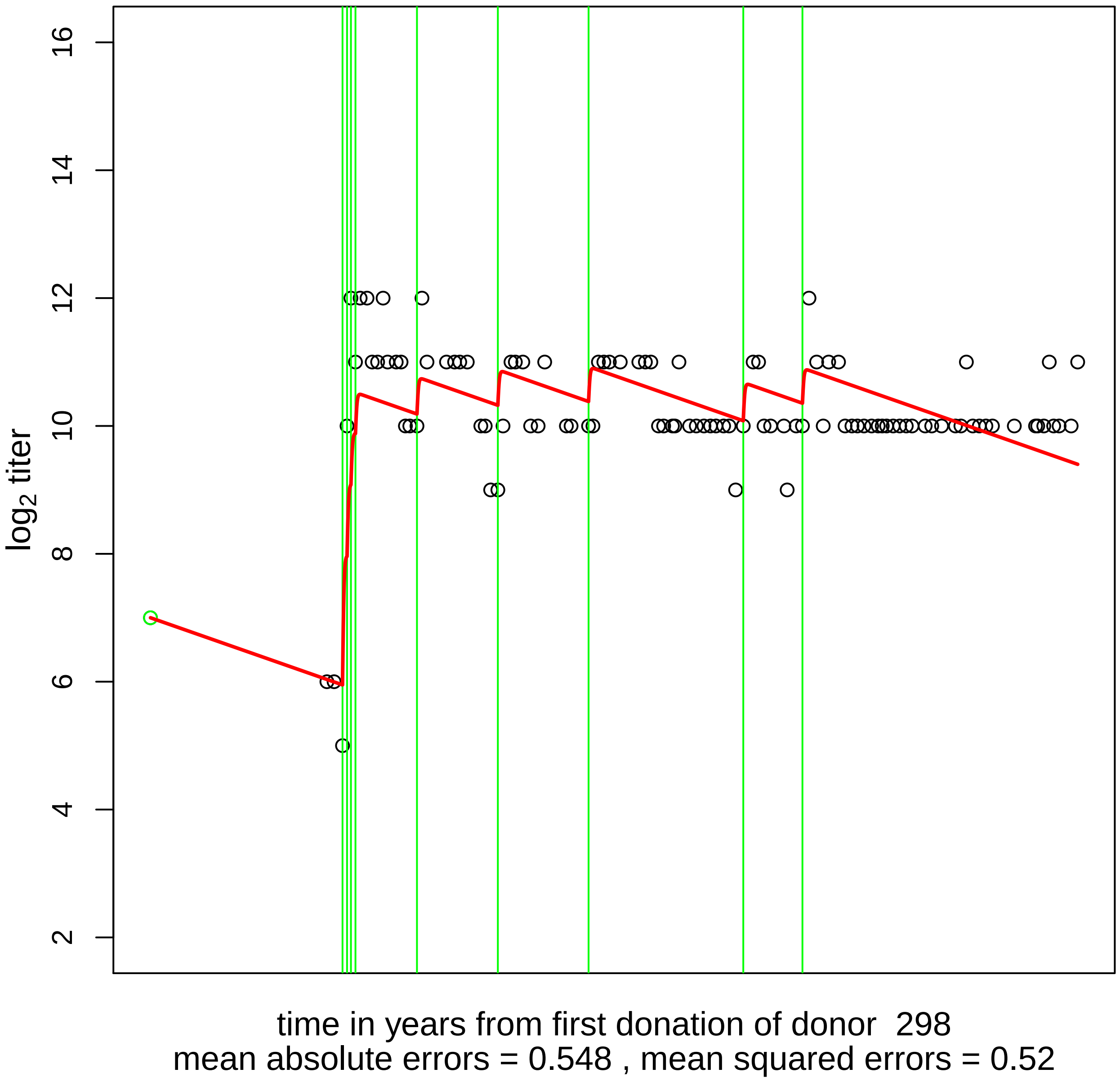

log2 titer
2
4
6
8
10
12
14
16
time in years from first donation of donor 298
mean absolute errors = 0.548 , mean squared errors = 0.52

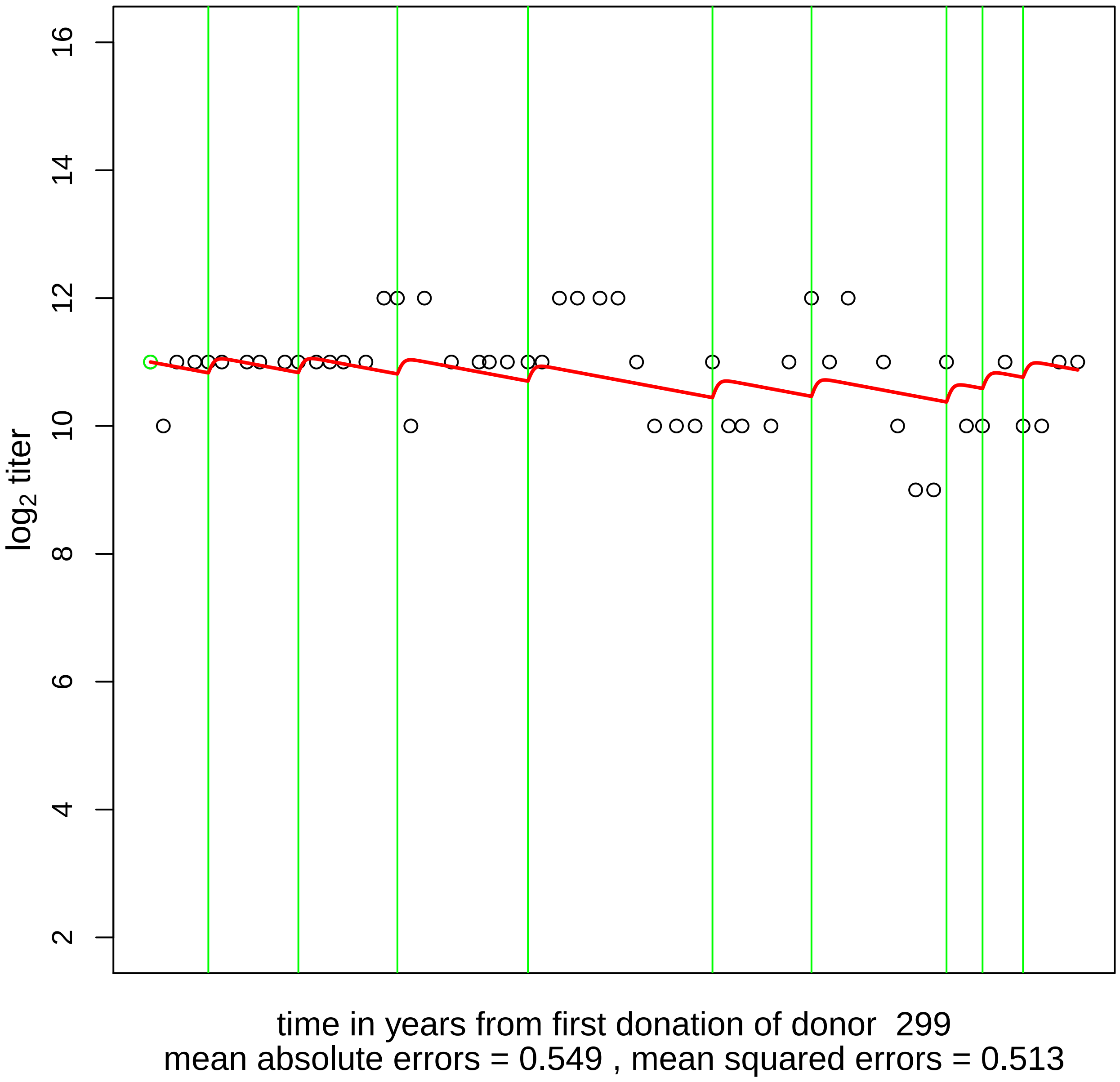
16
14
12
10
8
6
4
2
$\log_2$ titer
time in years from first donation of donor 299
mean absolute errors = 0.549 , mean squared errors = 0.513

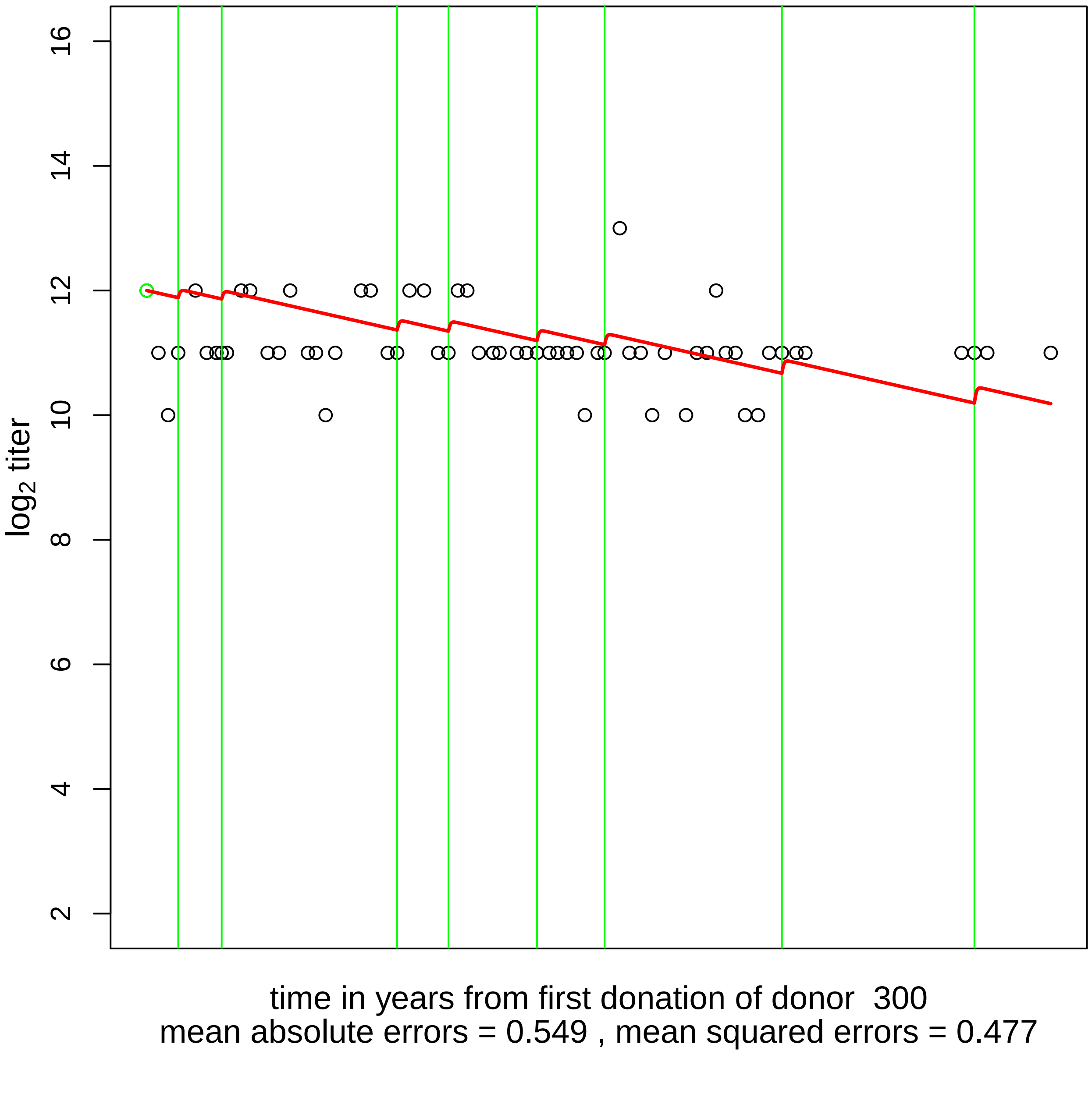

log2 titer
16
14
12
10
8
6
4
2
time in years from first donation of donor 300
mean absolute errors = 0.549 , mean squared errors = 0.477

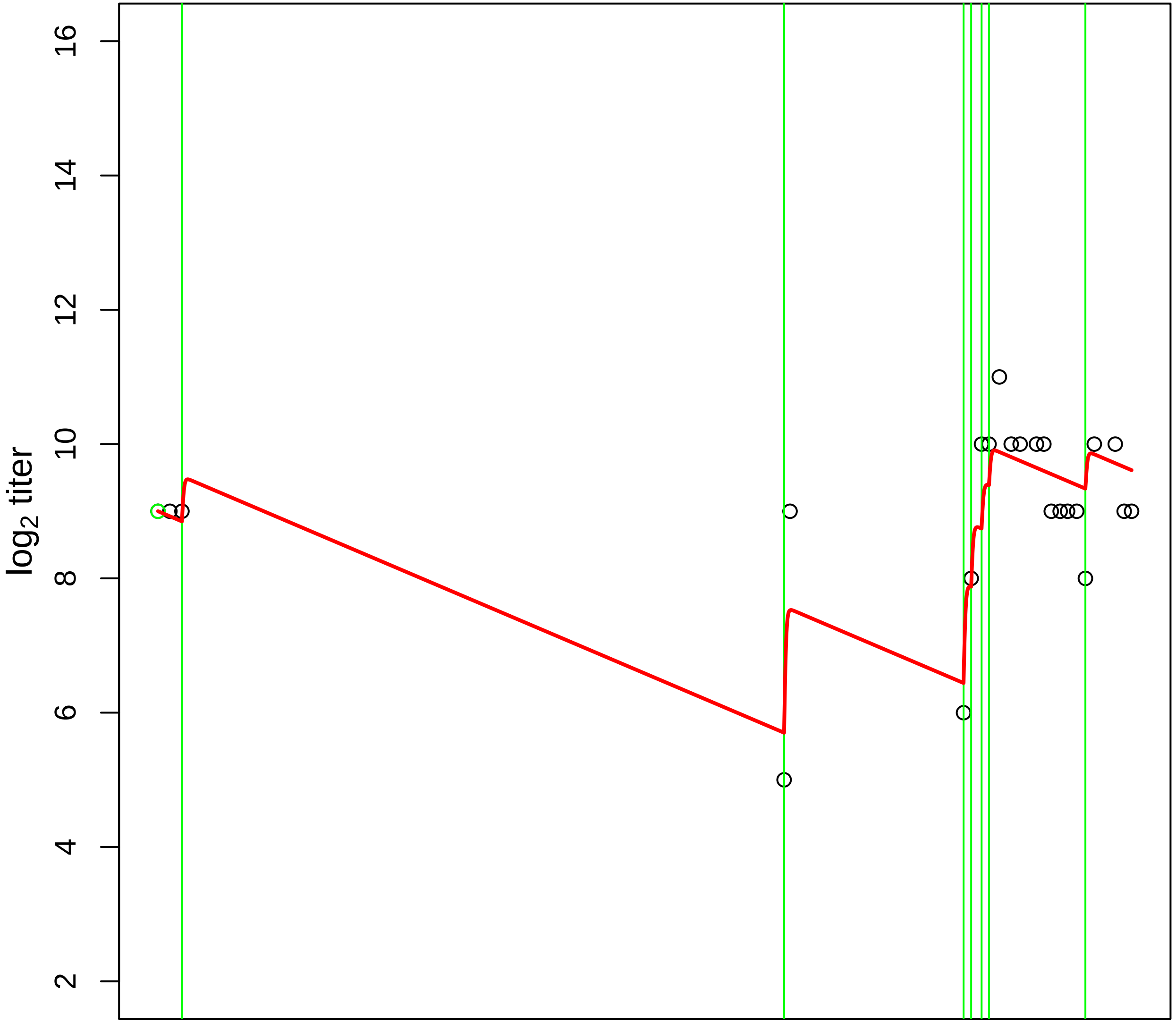

time in years from first donation of donor 301
mean absolute errors = 0.549 , mean squared errors = 0.459

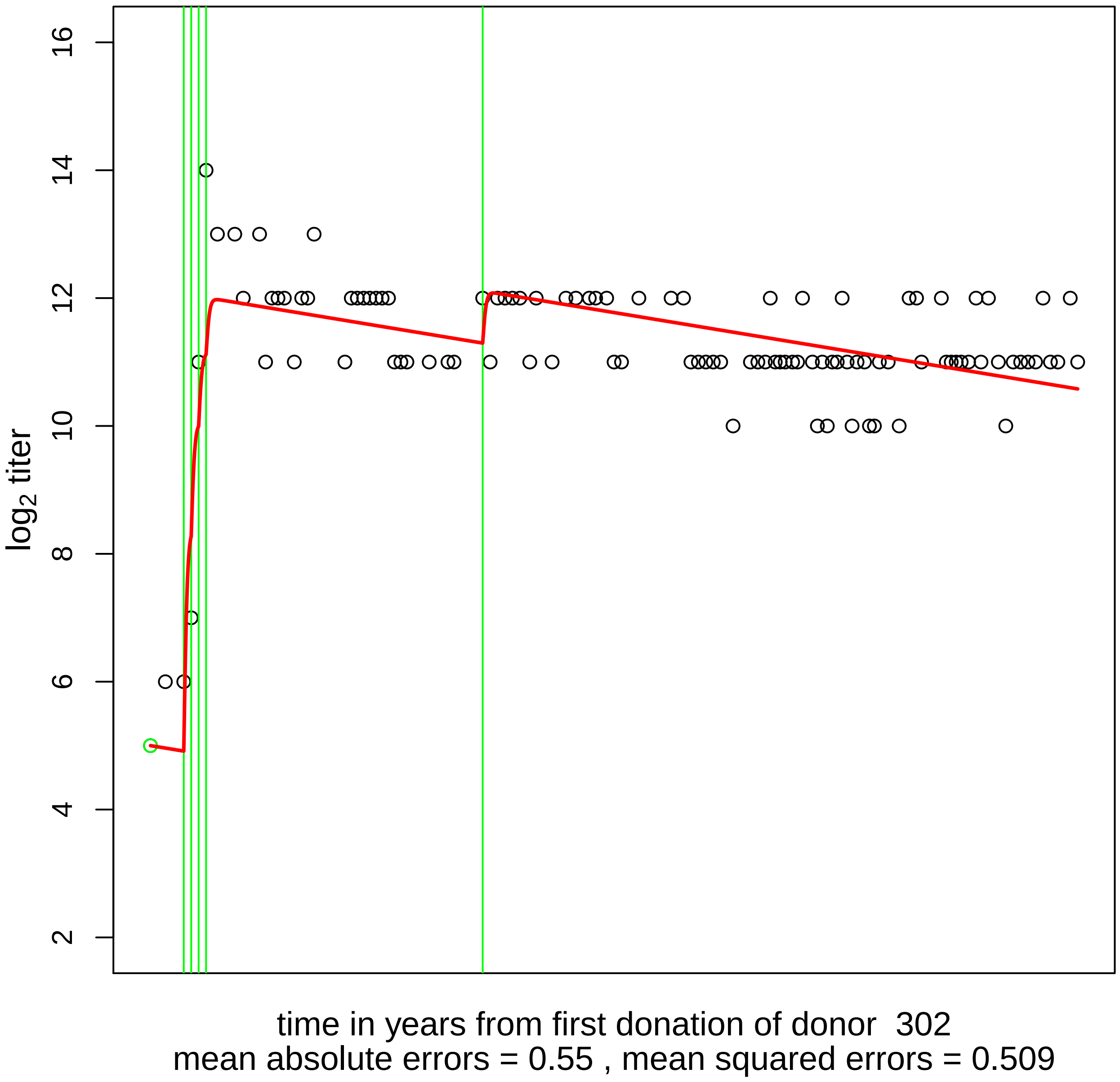

2
4
6
8
10
12
14
16
$\log_2$ titer
time in years from first donation of donor 302
mean absolute errors = 0.55 , mean squared errors = 0.509

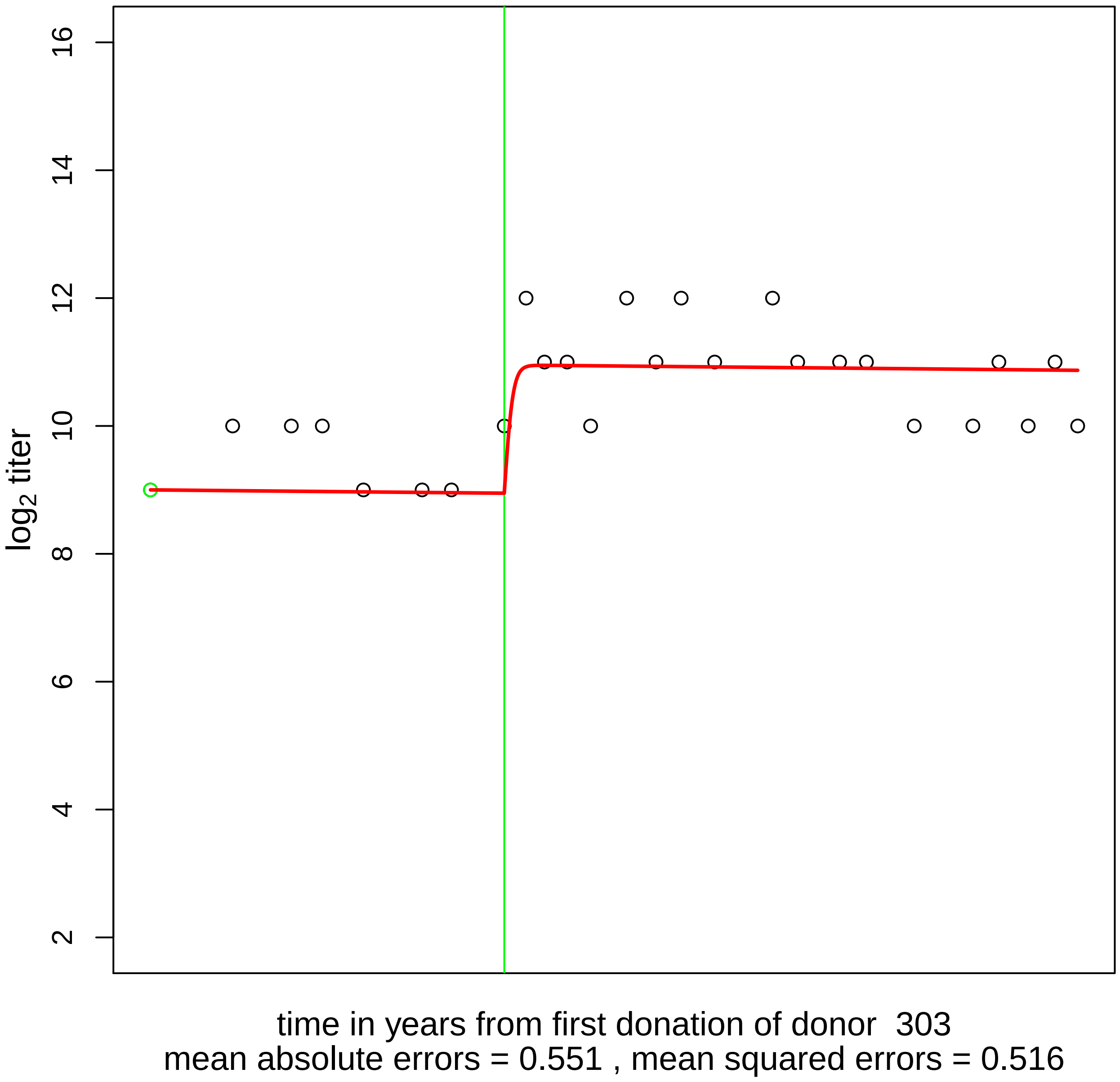
log2 titer
2
4
6
8
10
12
14
16
time in years from first donation of donor 303
mean absolute errors = 0.551 , mean squared errors = 0.516

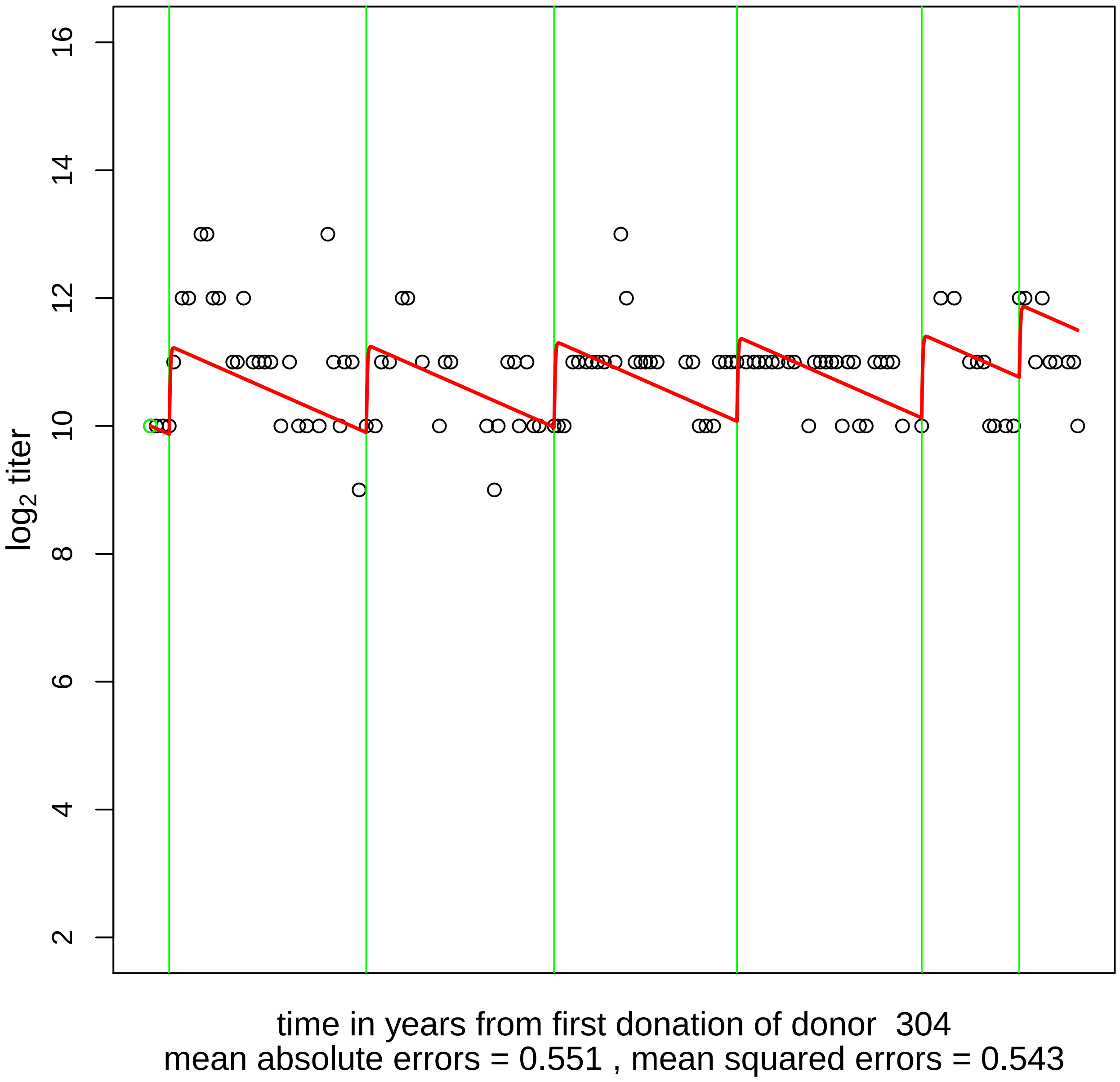
log$_2$ titer
2
4
6
8
10
12
14
16
time in years from first donation of donor 304
mean absolute errors = 0.551 , mean squared errors = 0.543

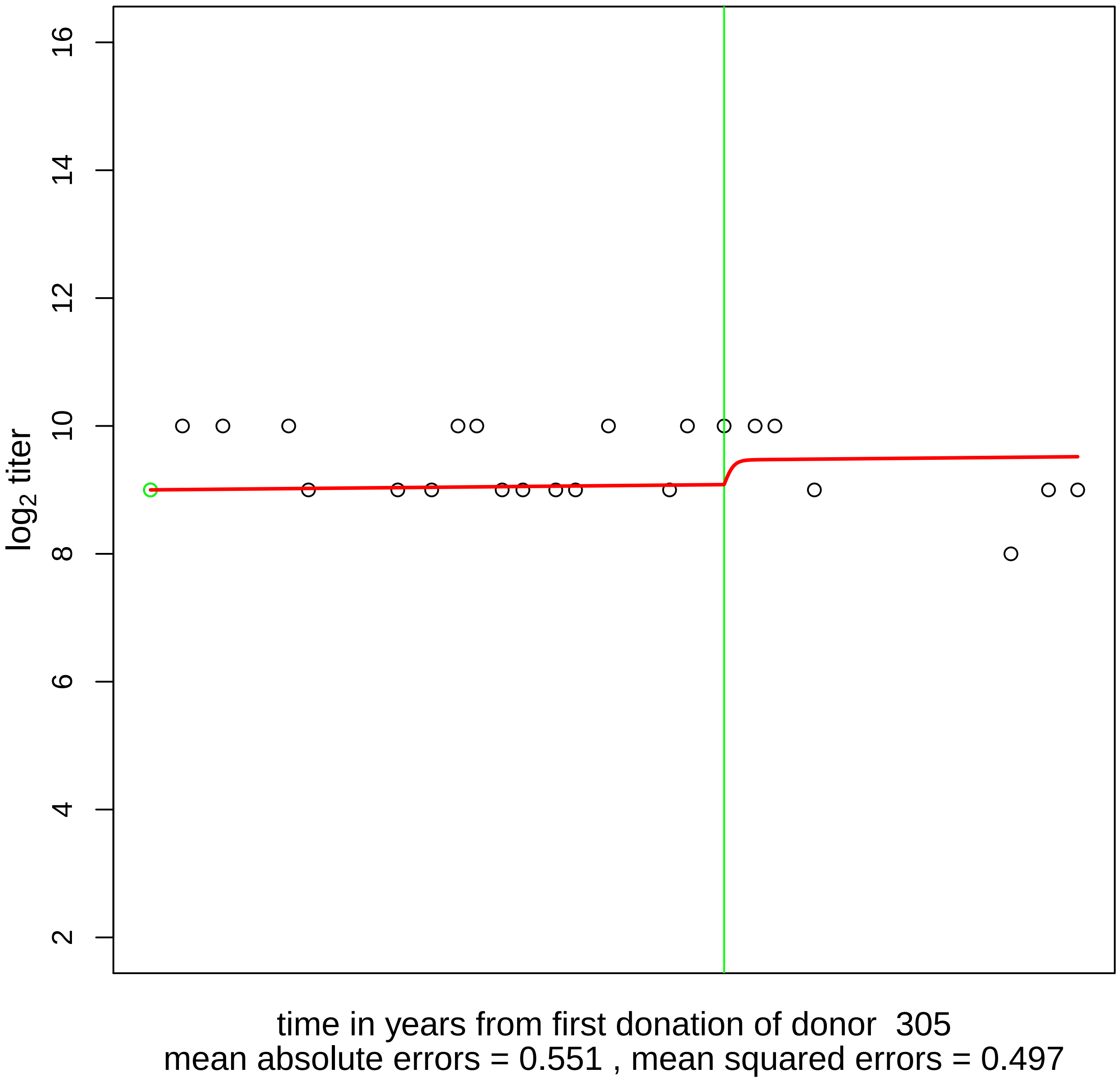

log2 titer
16
14
12
10
8
6
4
2
time in years from first donation of donor 305
mean absolute errors = 0.551 , mean squared errors = 0.497

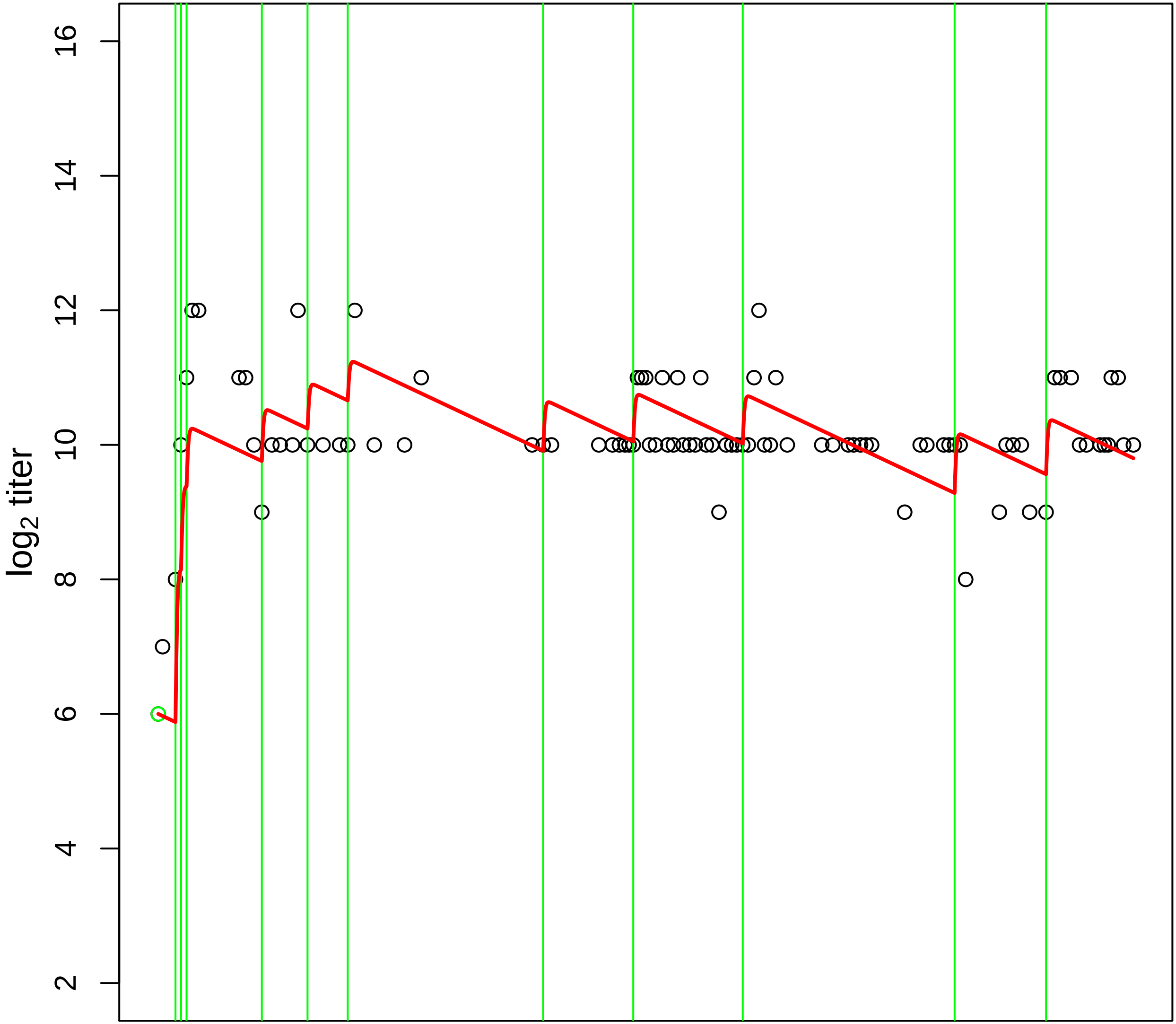

time in years from first donation of donor 306
mean absolute errors = 0.551 , mean squared errors = 0.549

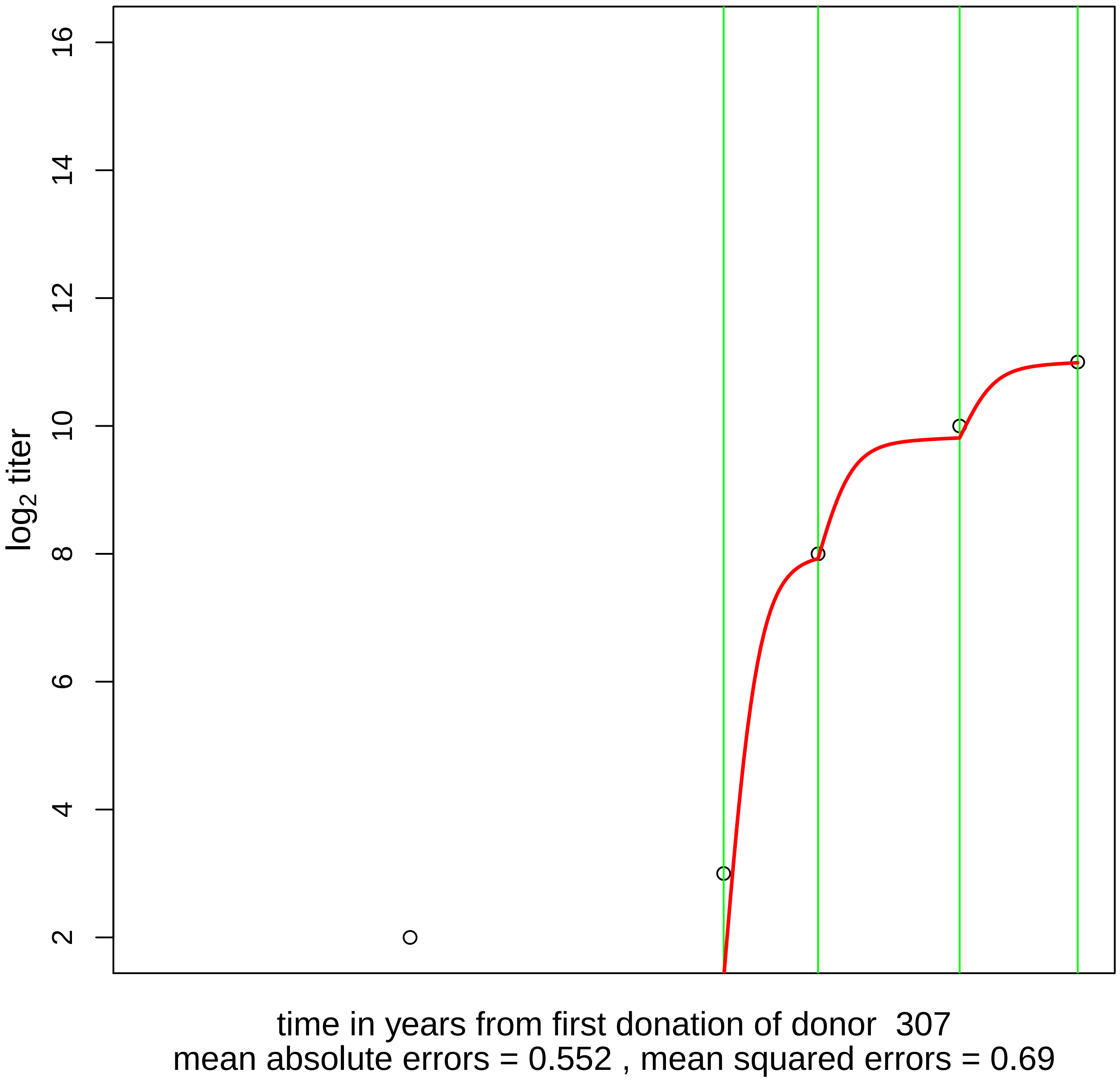

log2 titer
2
4
6
8
10
12
14
16
time in years from first donation of donor 307
mean absolute errors = 0.552 , mean squared errors = 0.69

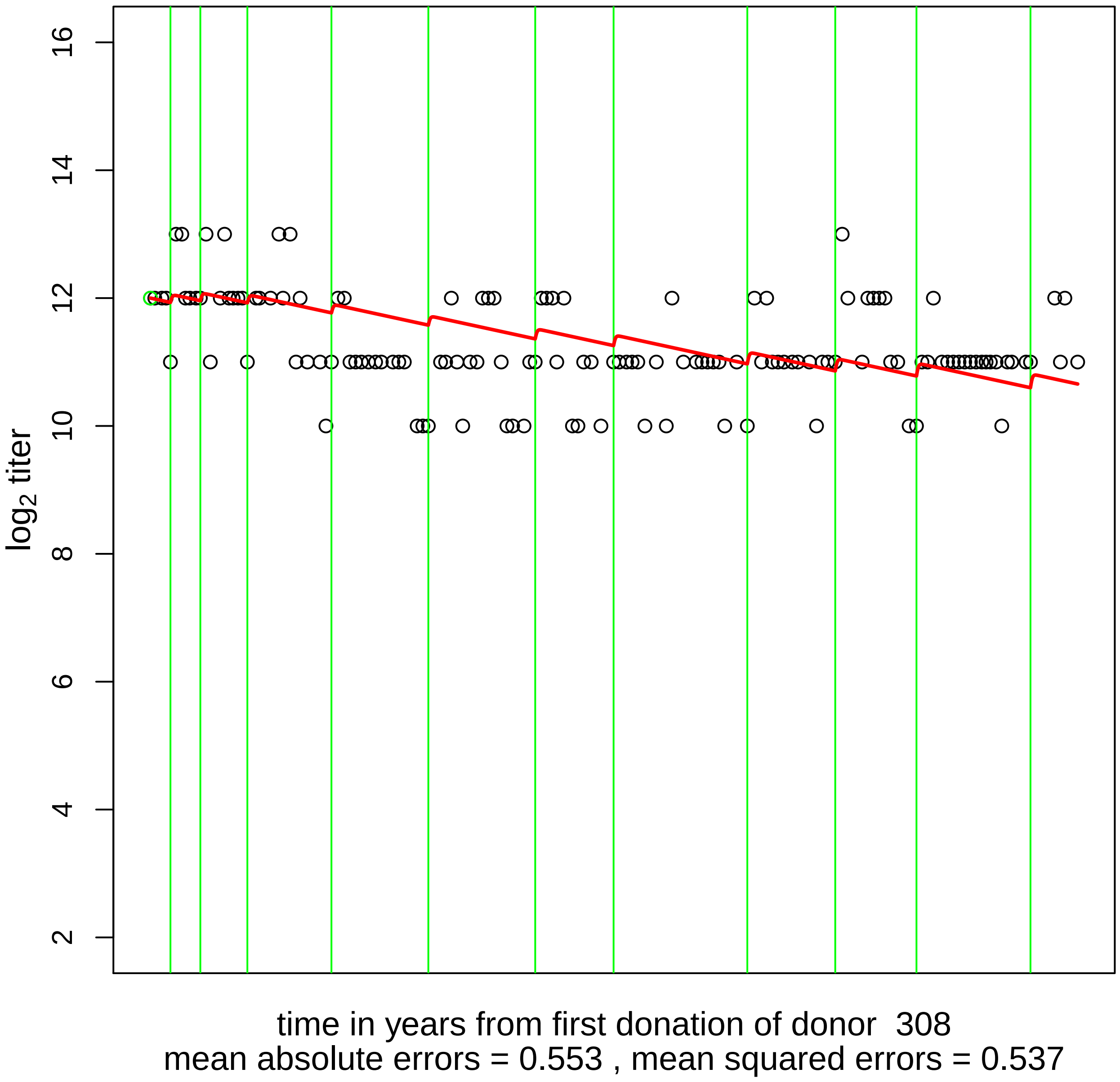

16
14
12
10
8
6
4
2
log2 titer
time in years from first donation of donor 308
mean absolute errors = 0.553 , mean squared errors = 0.537

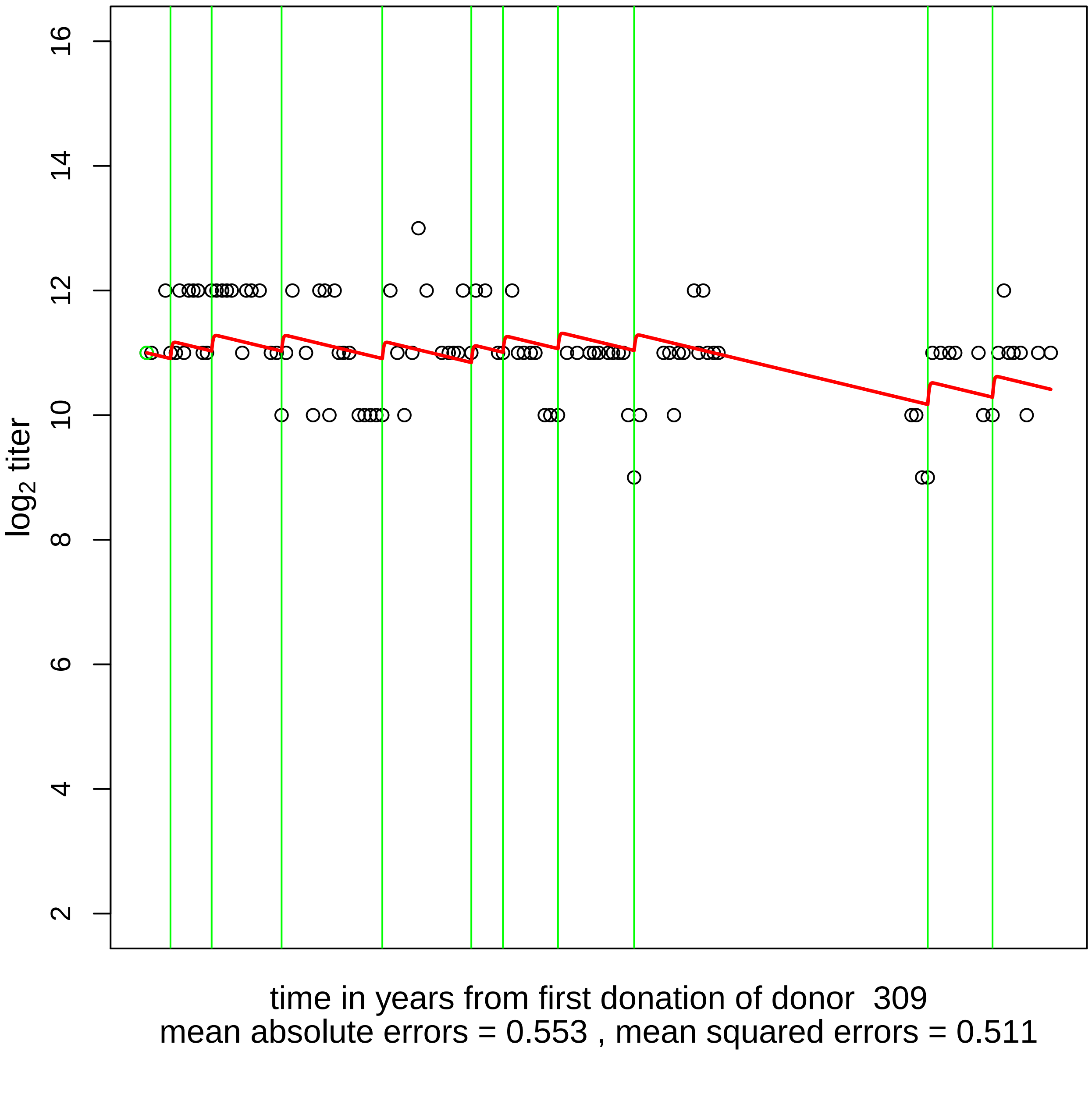

log2 titer
2
4
6
8
10
12
14
16
time in years from first donation of donor 309
mean absolute errors = 0.553 , mean squared errors = 0.511

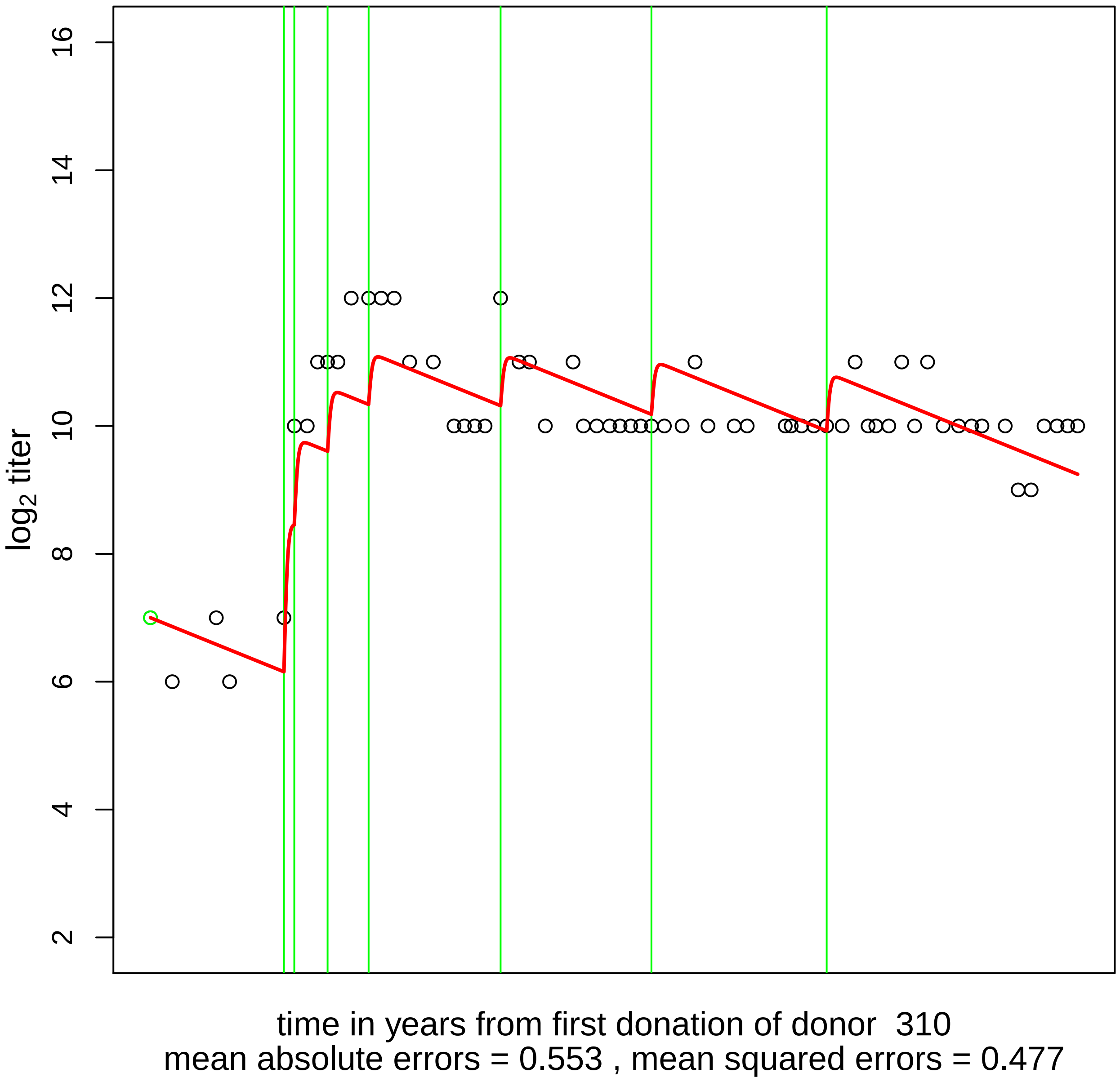

log2 titer
16
14
12
10
8
6
4
2
time in years from first donation of donor 310
mean absolute errors = 0.553 , mean squared errors = 0.477

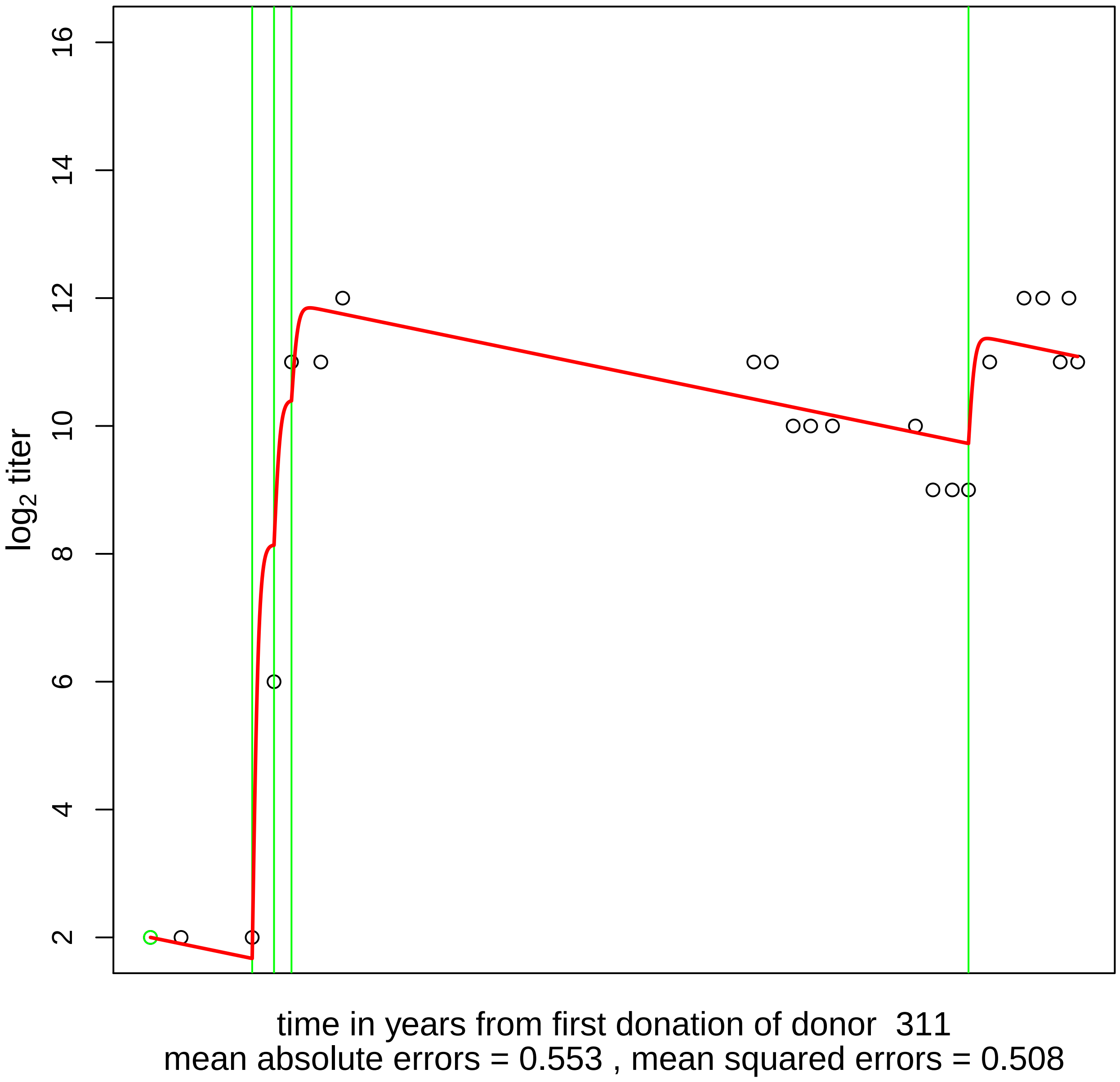

log2 titer
2
4
6
8
10
12
14
16
time in years from first donation of donor 311
mean absolute errors = 0.553 , mean squared errors = 0.508

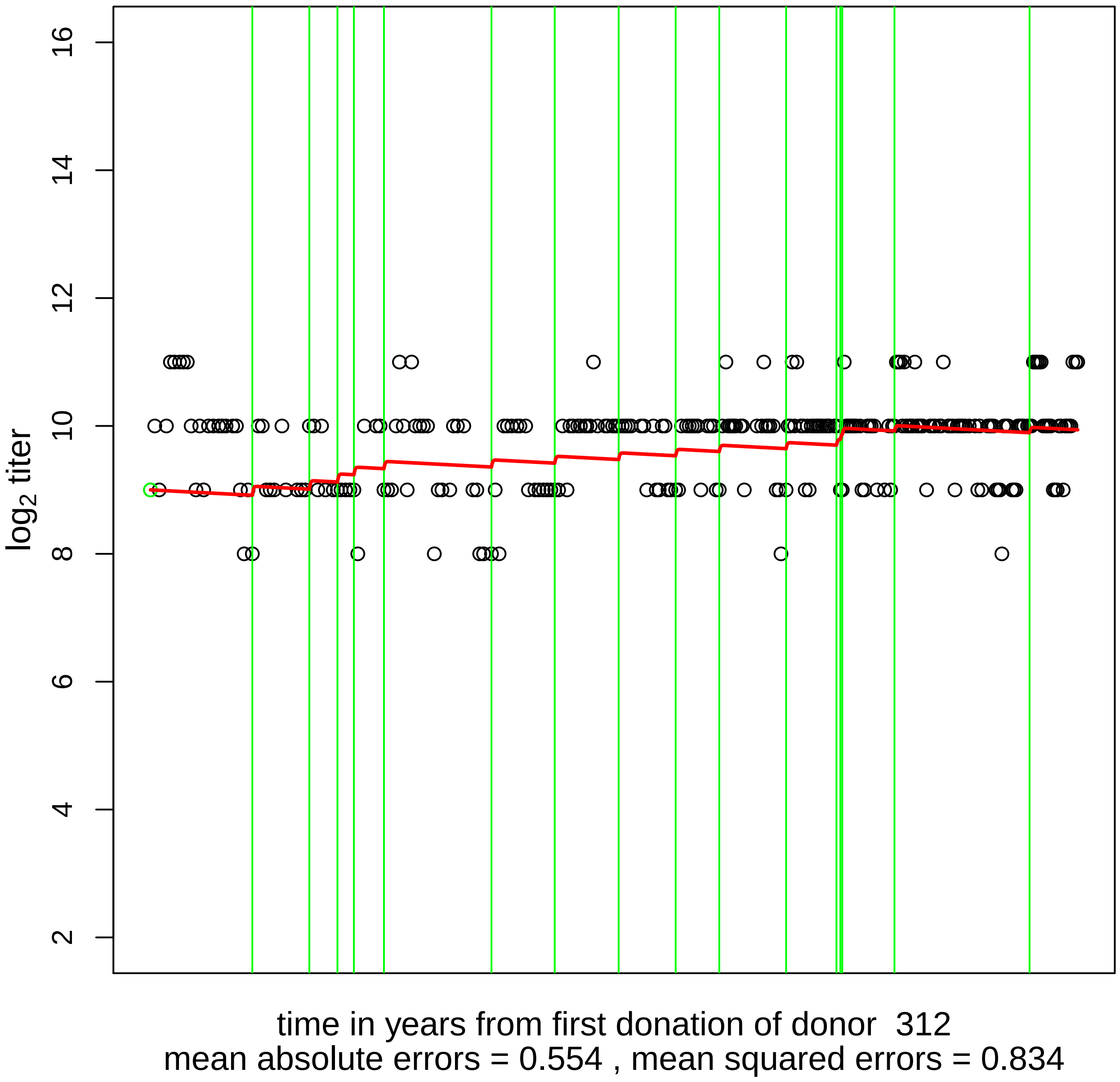

16
14
12
10
8
6
4
2
log2 titer
time in years from first donation of donor 312
mean absolute errors = 0.554 , mean squared errors = 0.834

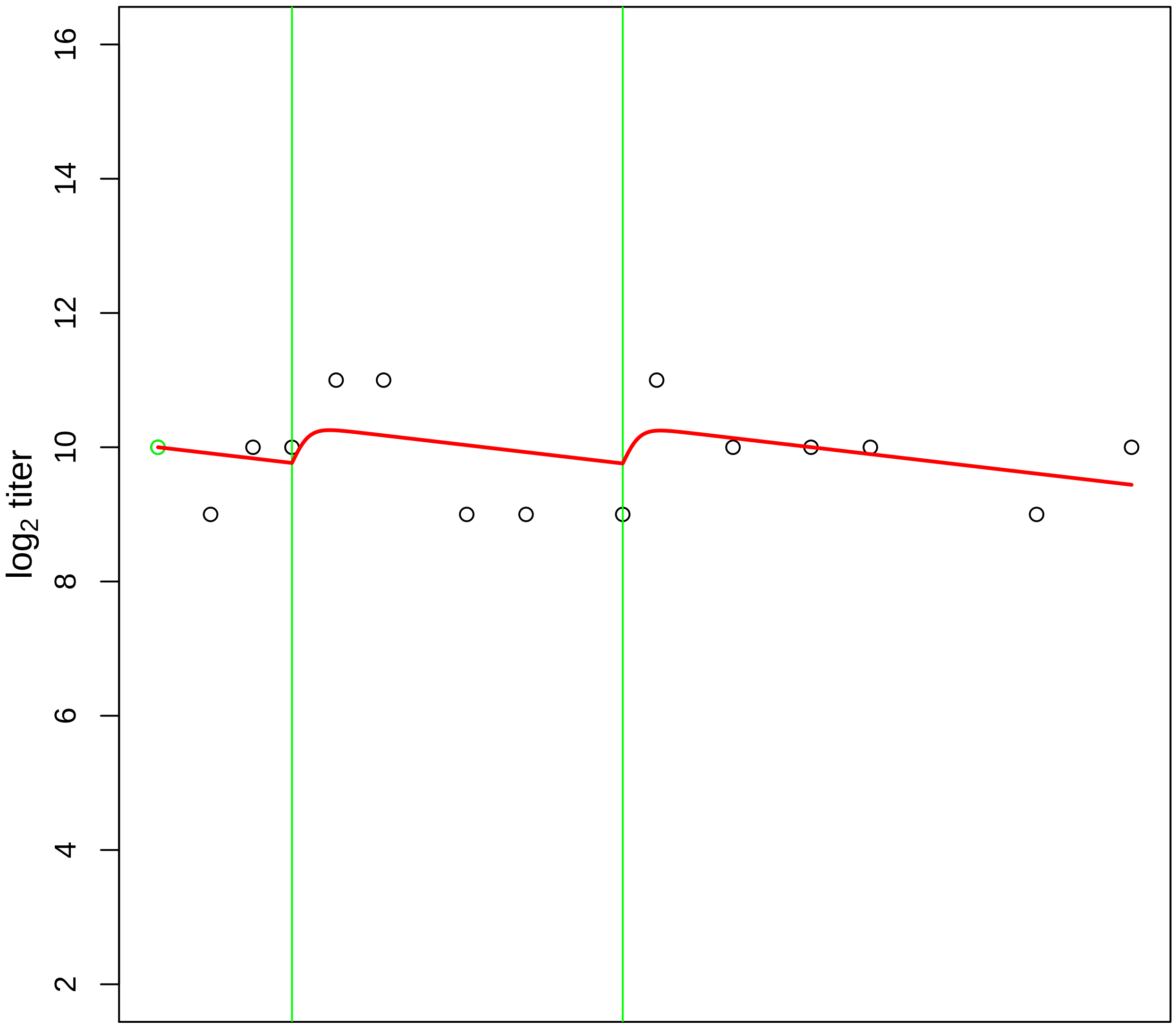

time in years from first donation of donor 313
mean absolute errors = 0.554 , mean squared errors = 0.423

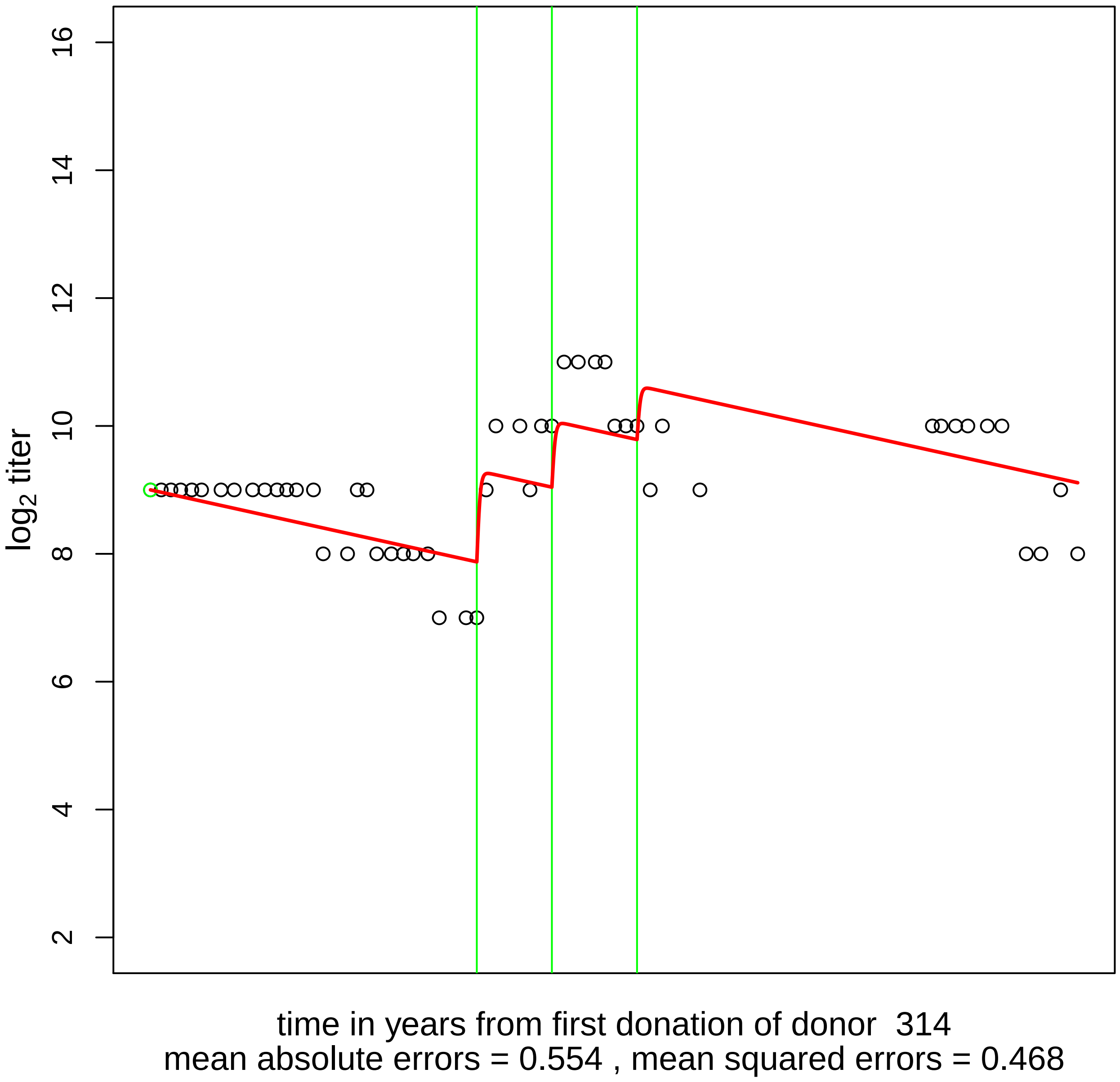

log2 titer
2
4
6
8
10
12
14
16
time in years from first donation of donor 314
mean absolute errors = 0.554 , mean squared errors = 0.468

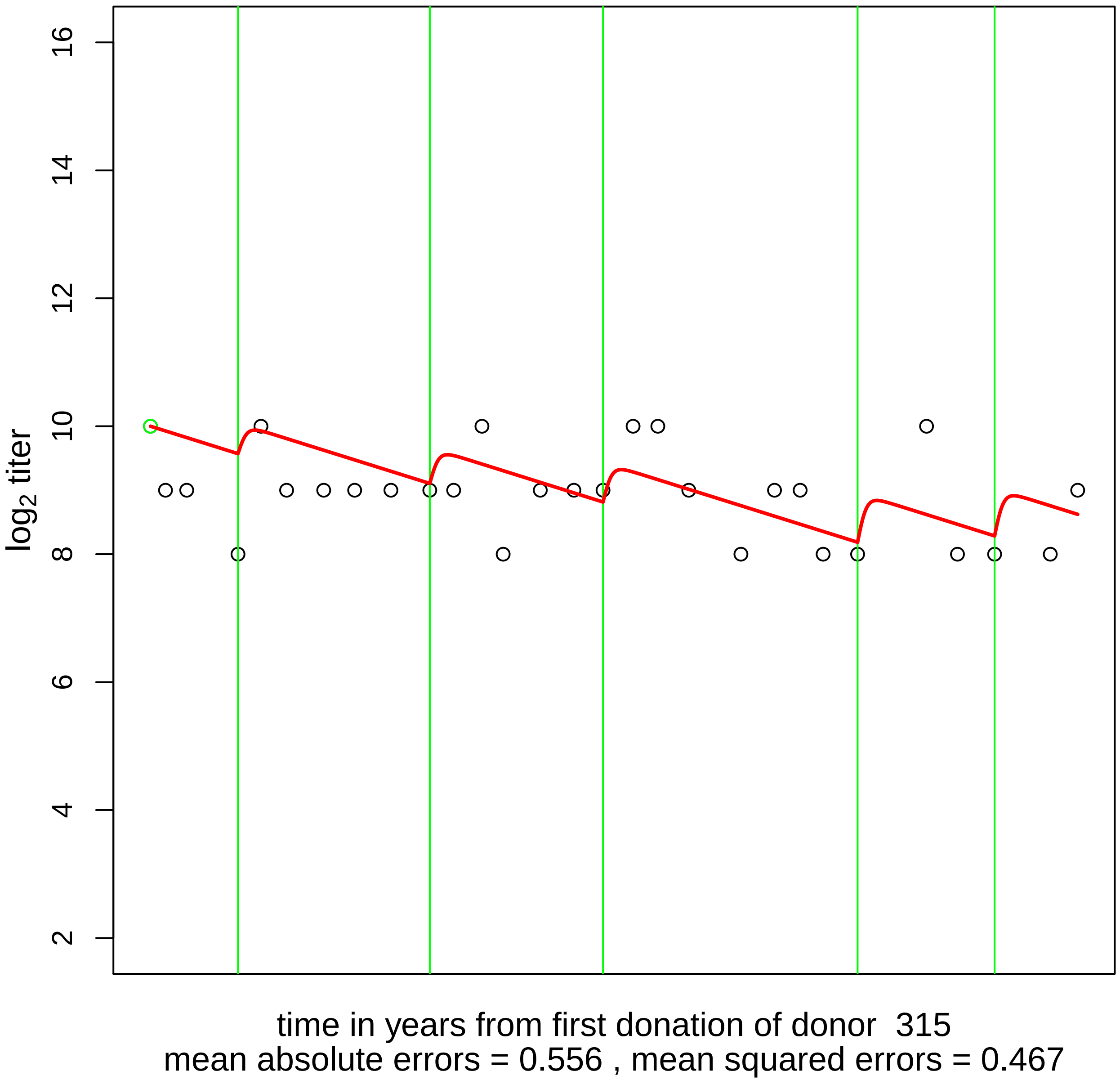

log$_2$ titer
2
4
6
8
10
12
14
16
time in years from first donation of donor 315
mean absolute errors = 0.556 , mean squared errors = 0.467

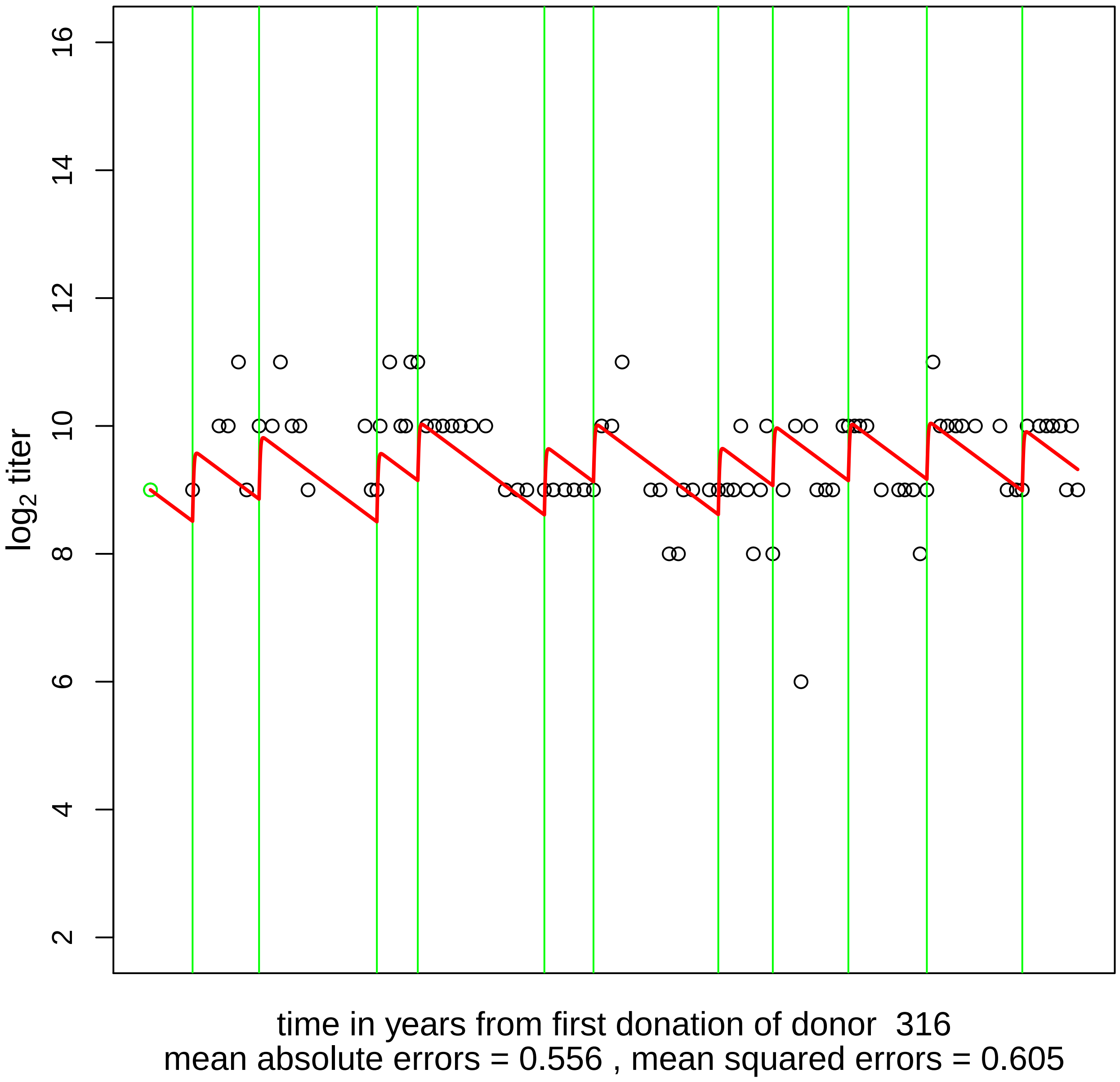

2
4
6
8
10
12
14
16
$\log_2$ titer
time in years from first donation of donor 316
mean absolute errors = 0.556 , mean squared errors = 0.605

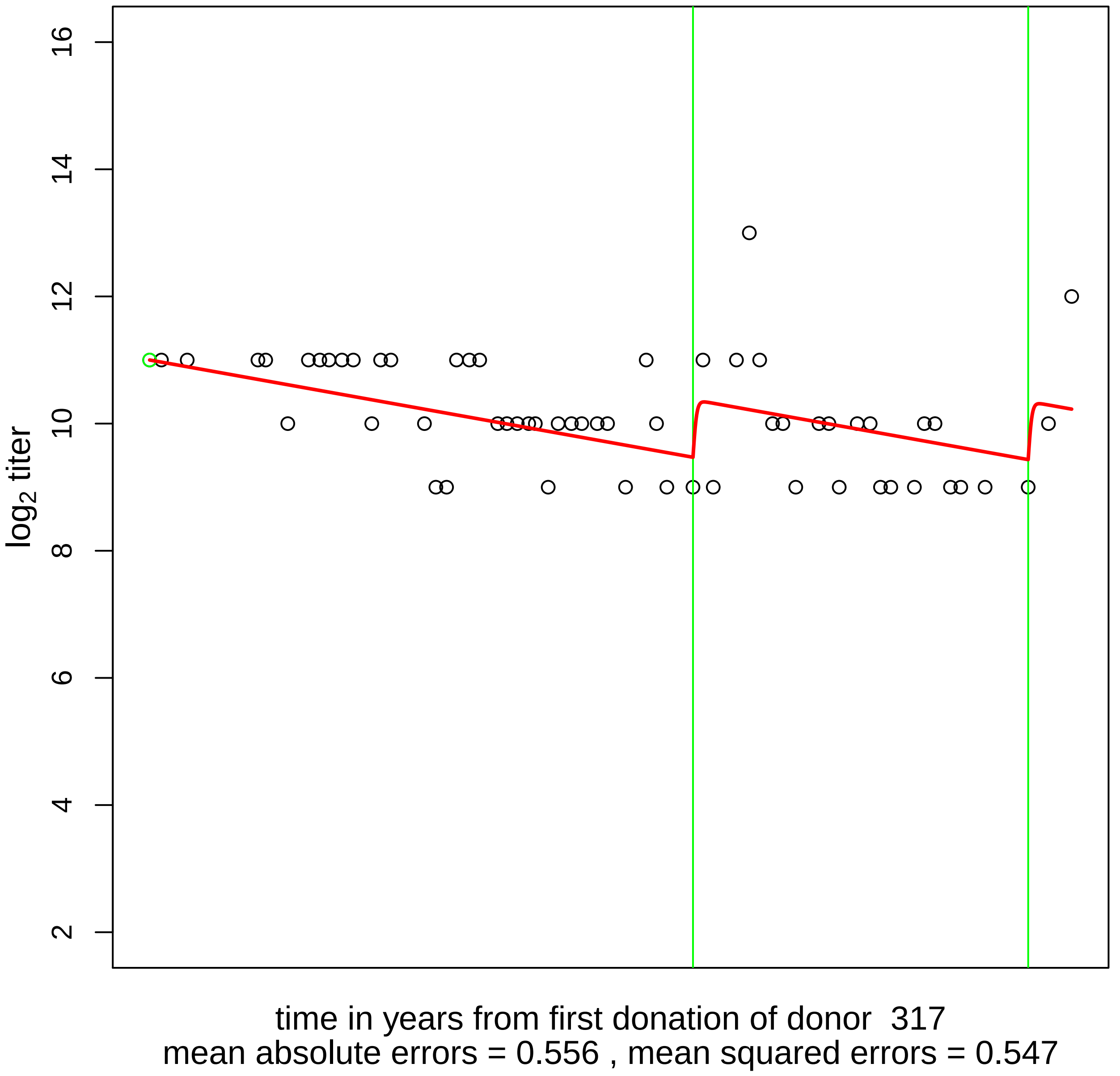

2
4
6
8
10
12
14
16
$\log_2$ titer
time in years from first donation of donor 317
mean absolute errors = 0.556 , mean squared errors = 0.547

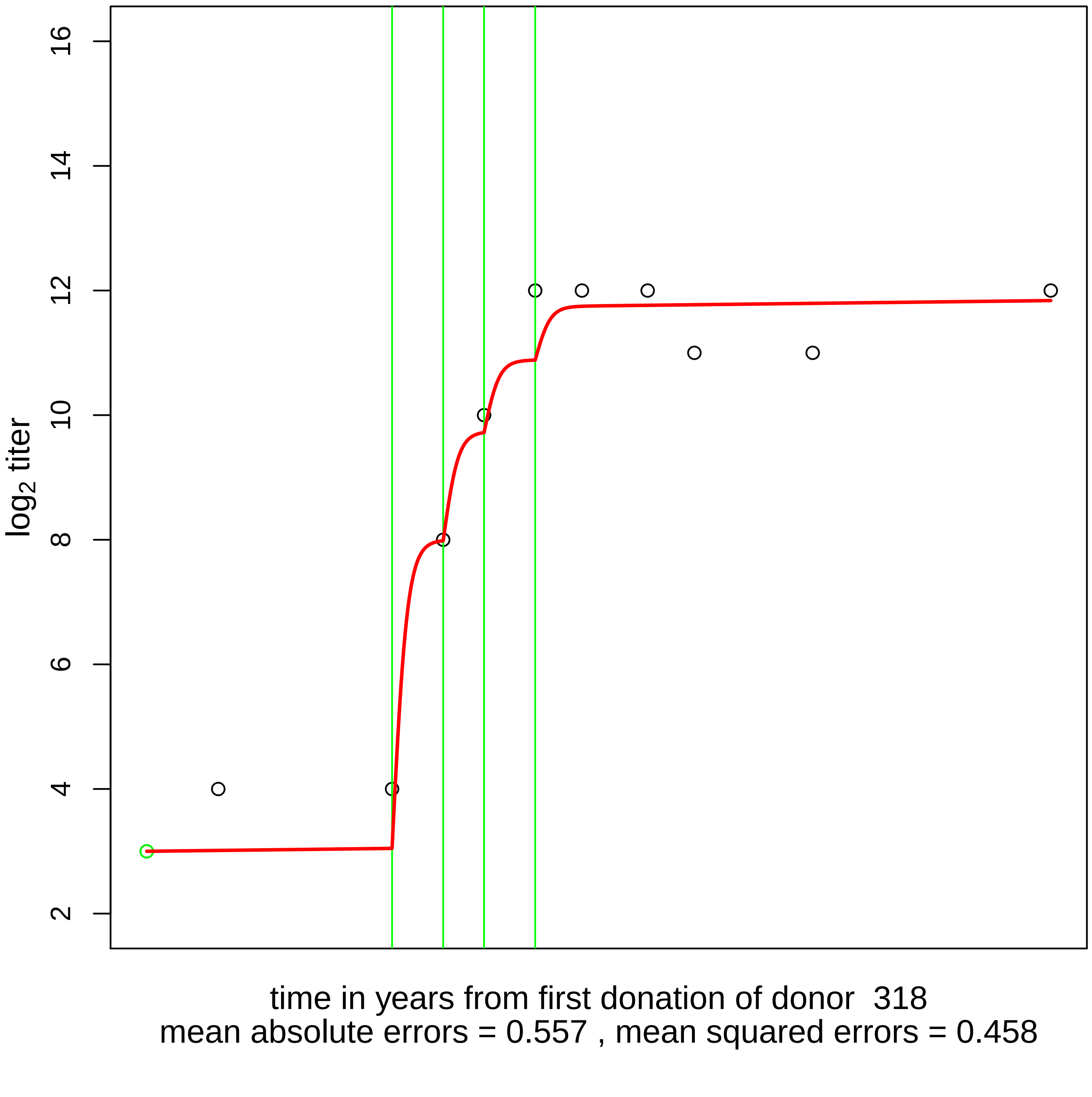

log2 titer
2
4
6
8
10
12
14
16
time in years from first donation of donor 318
mean absolute errors = 0.557 , mean squared errors = 0.458

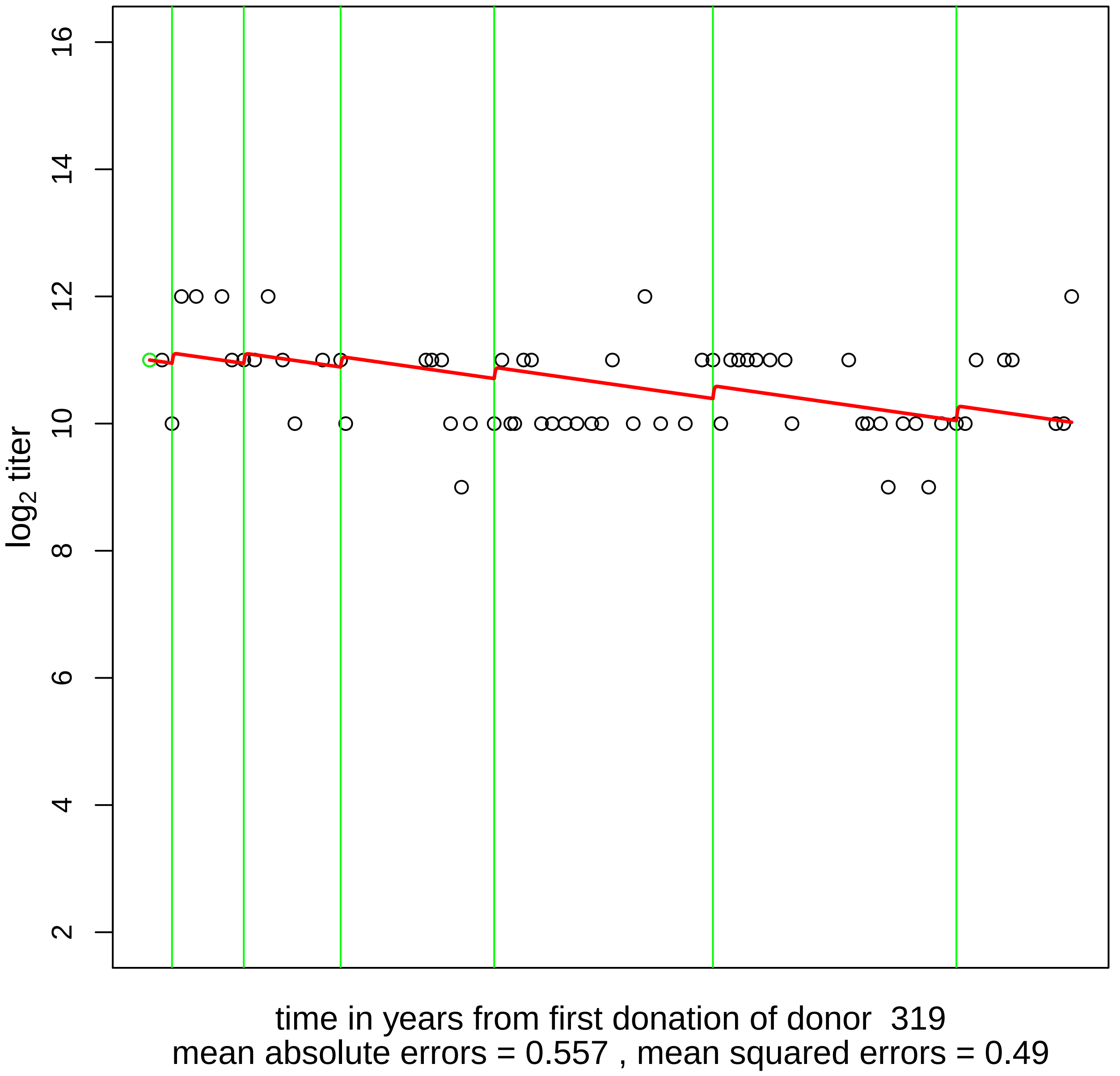

16
14
12
10
8
6
4
2
$\log_2$ titer
time in years from first donation of donor 319
mean absolute errors = 0.557 , mean squared errors = 0.49

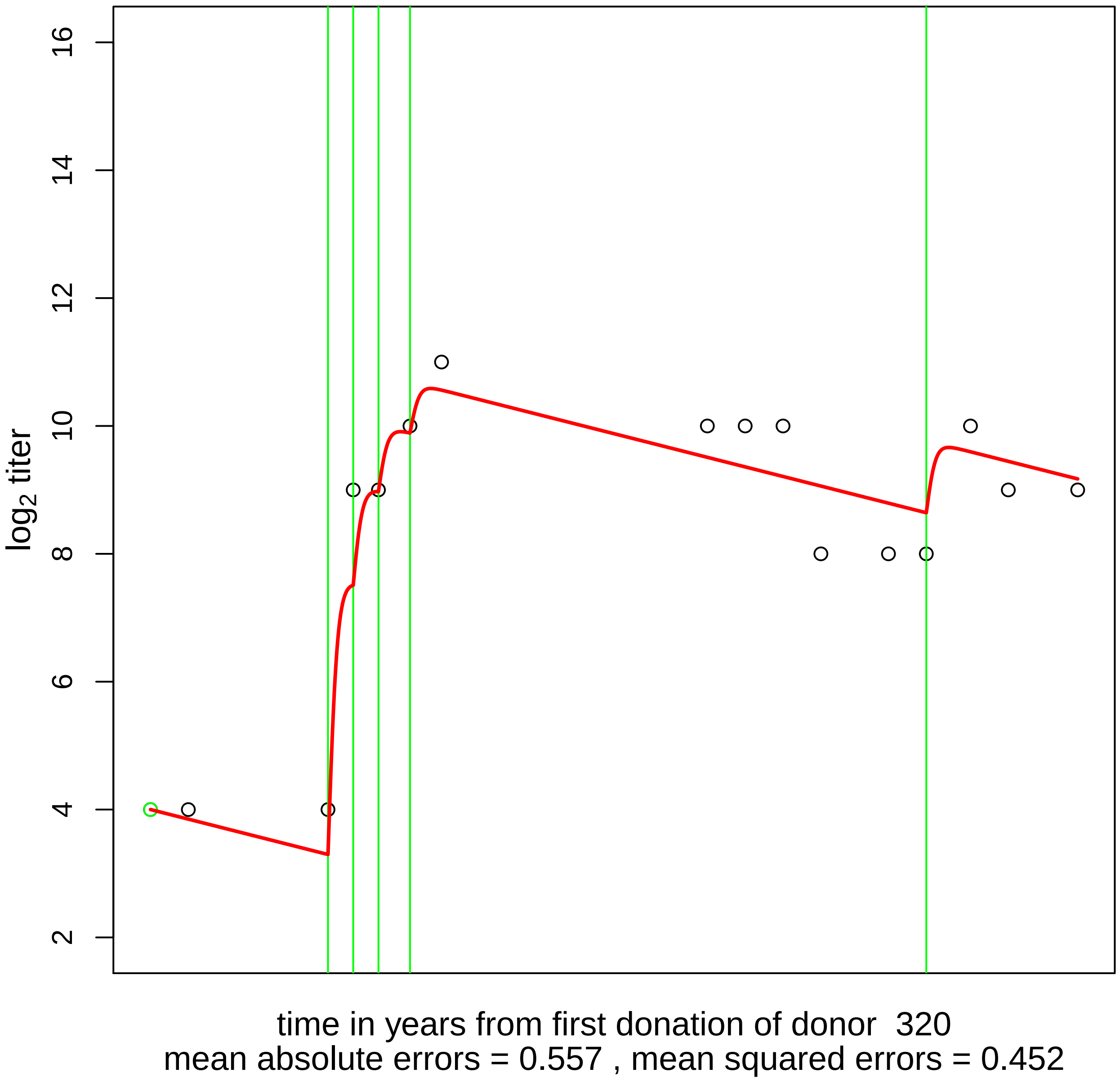

log2 titer
16
14
12
10
8
6
4
2
time in years from first donation of donor 320
mean absolute errors = 0.557 , mean squared errors = 0.452

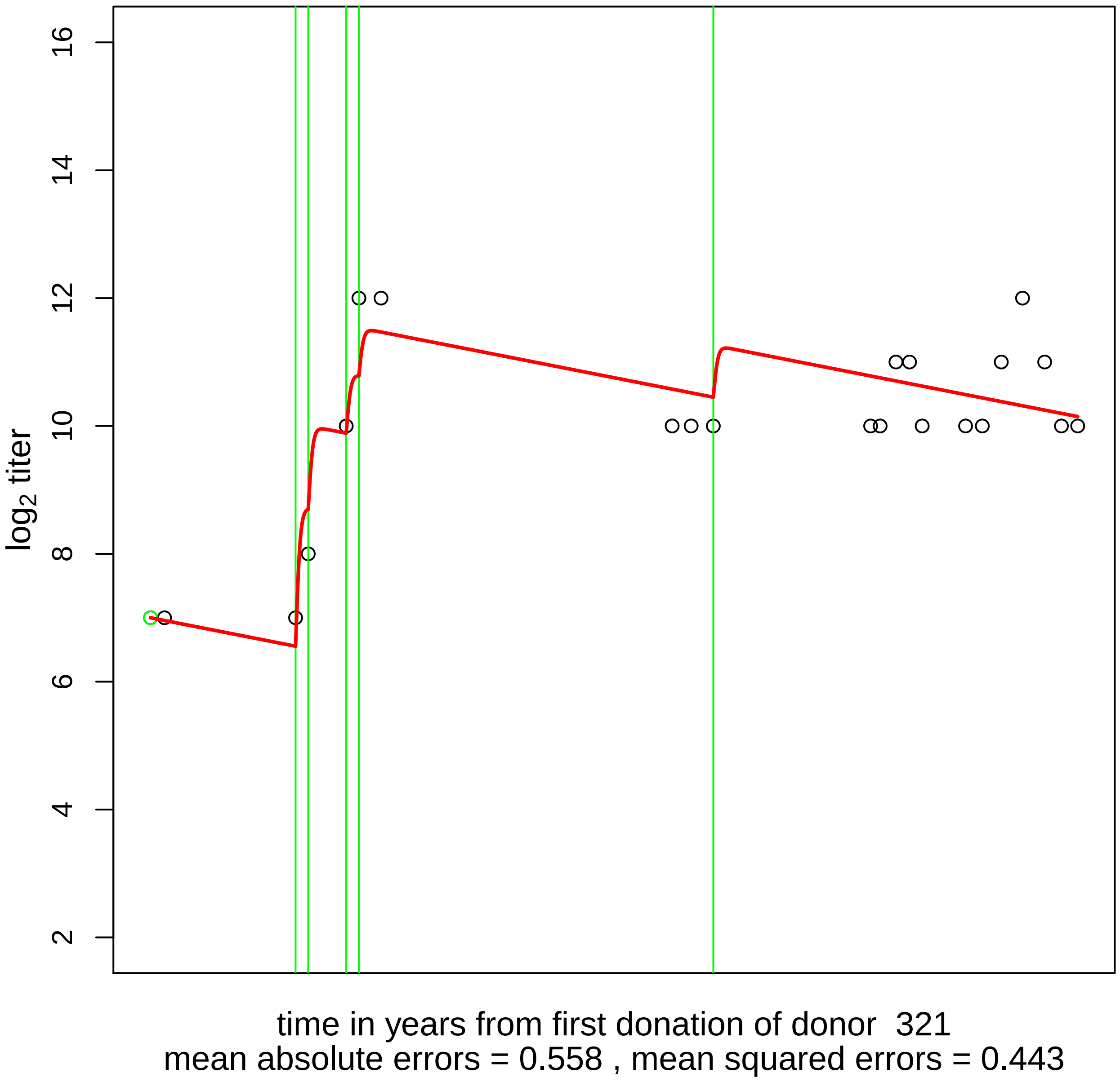

log2 titer
16
14
12
10
8
6
4
2
time in years from first donation of donor 321
mean absolute errors = 0.558 , mean squared errors = 0.443

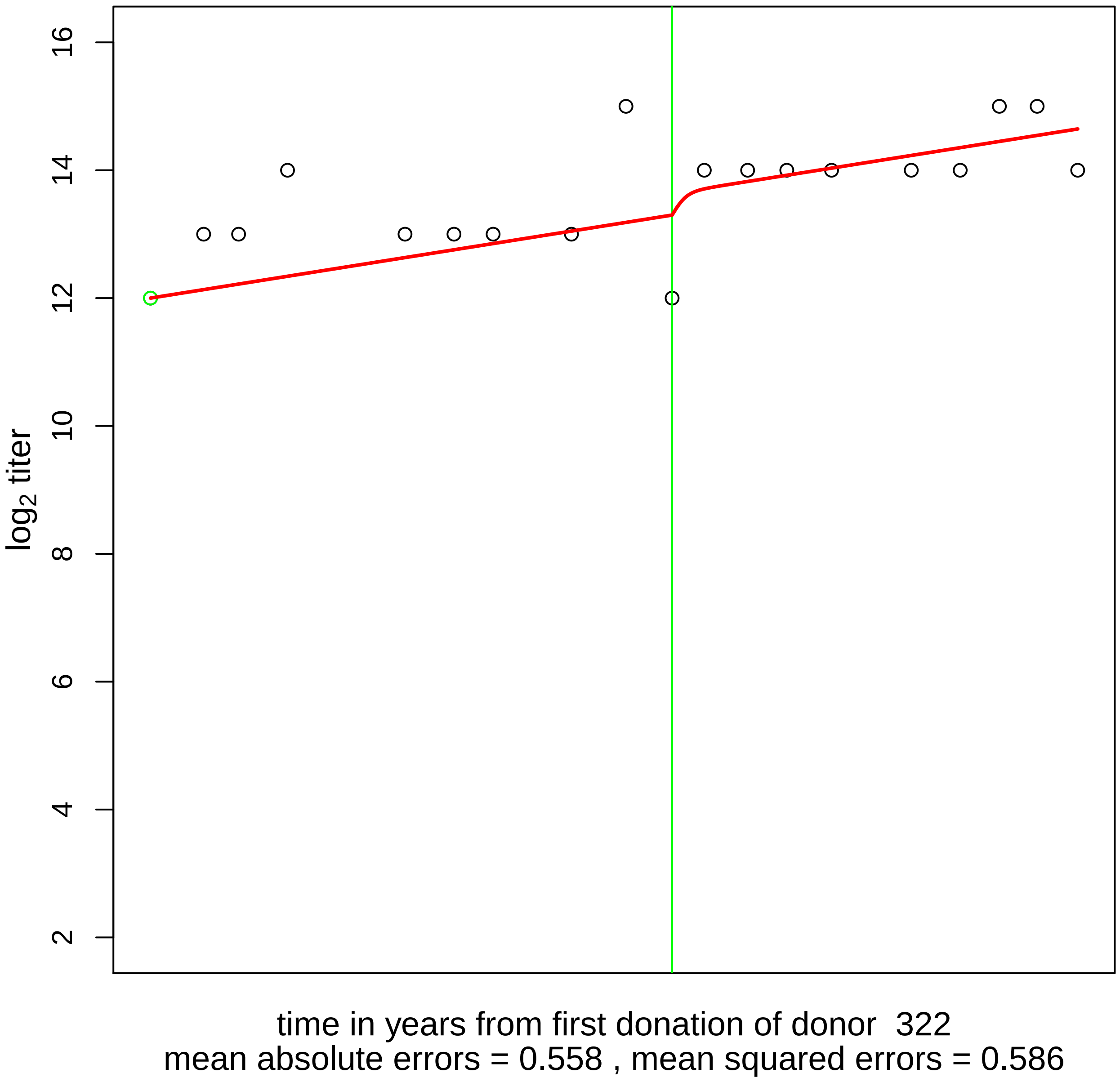
log2 titer
2
4
6
8
10
12
14
16
time in years from first donation of donor 322
mean absolute errors = 0.558 , mean squared errors = 0.586

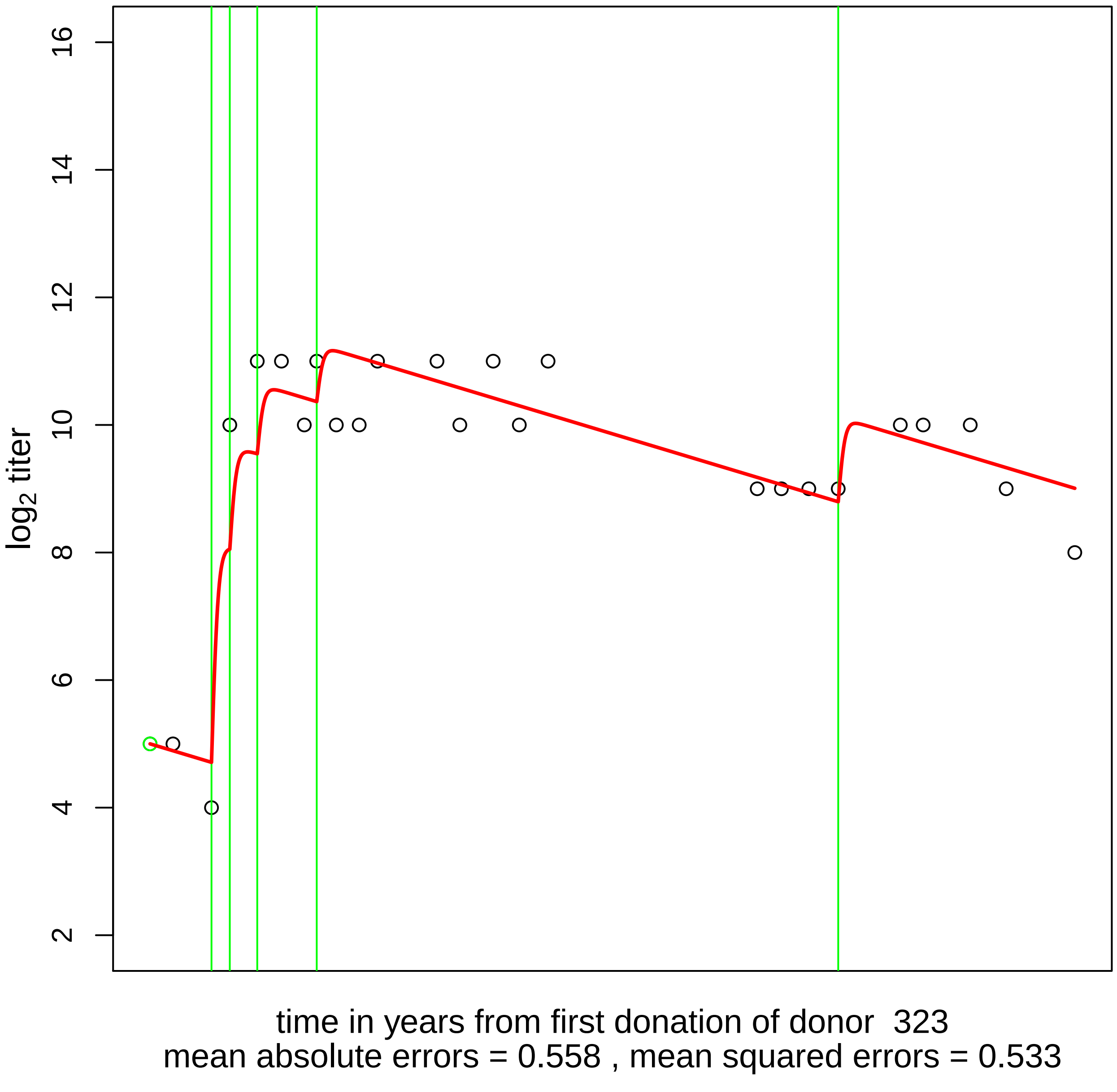

2
4
6
8
10
12
14
16
$\log_2$ titer
time in years from first donation of donor 323
mean absolute errors = 0.558 , mean squared errors = 0.533

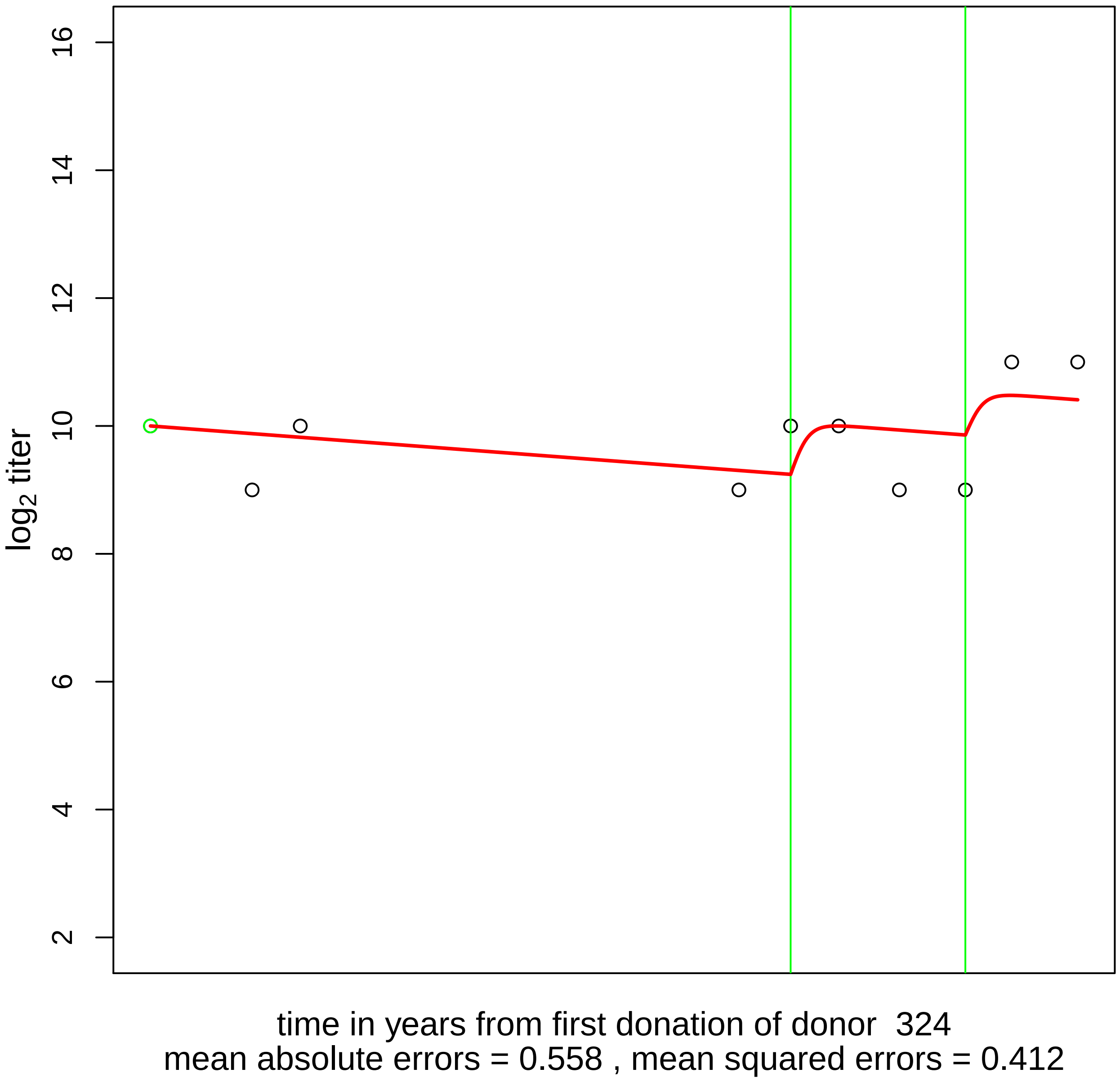

16
14
12
10
8
6
4
2
log2 titer
time in years from first donation of donor 324
mean absolute errors = 0.558 , mean squared errors = 0.412

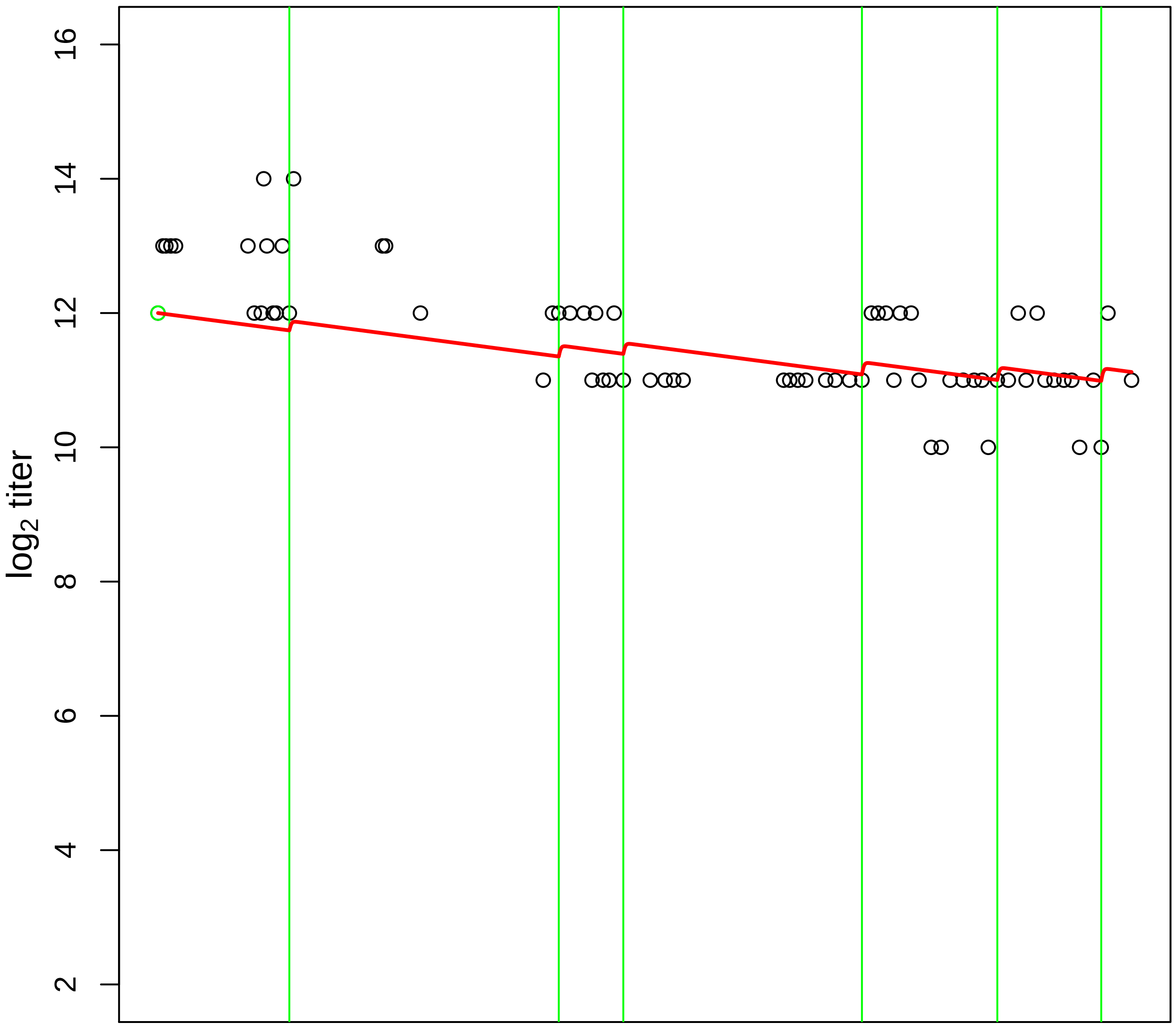

time in years from first donation of donor 325
mean absolute errors = 0.56 , mean squared errors = 0.542

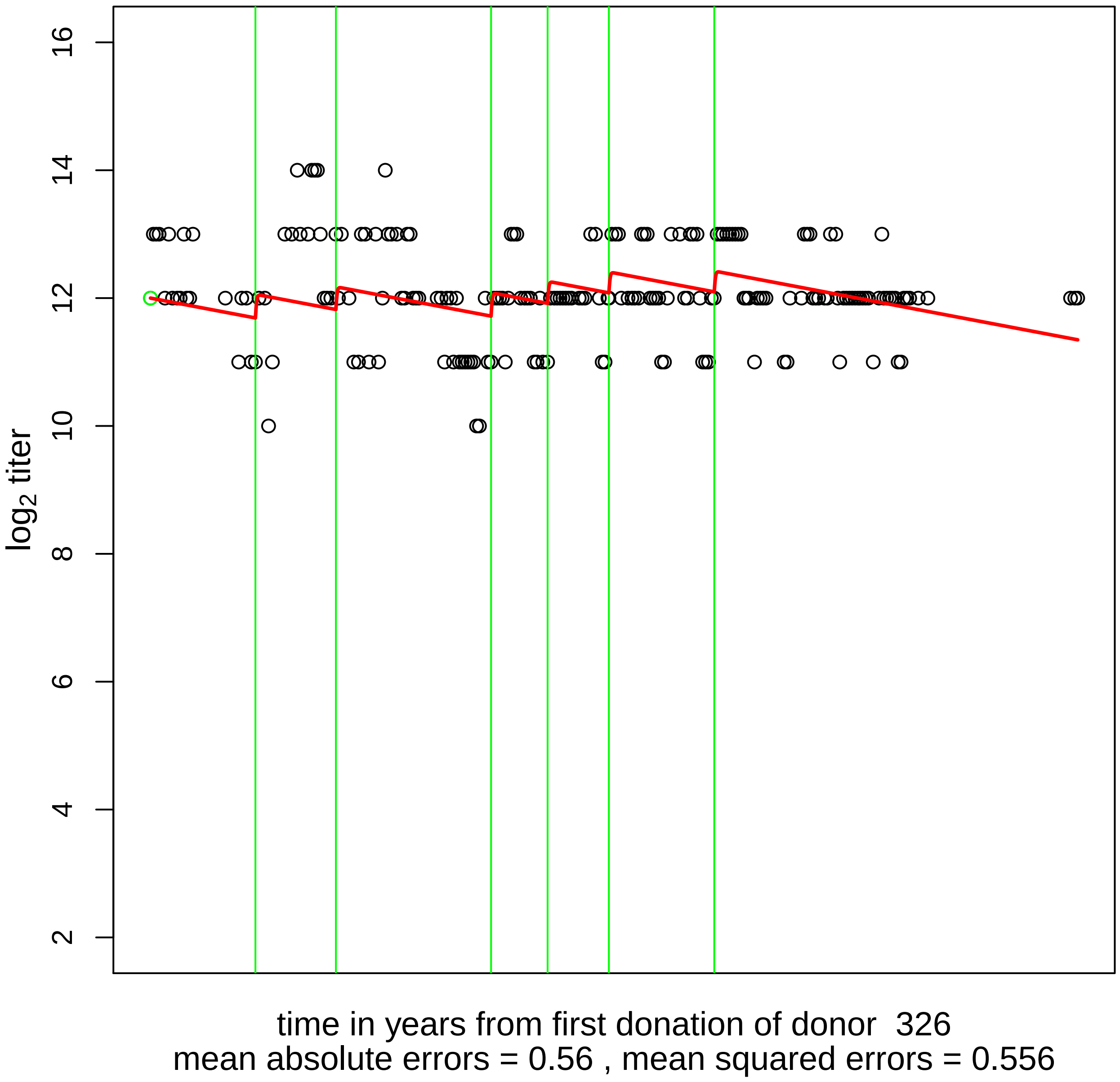
16
14
12
10
8
6
4
2
log2 titer
time in years from first donation of donor 326
mean absolute errors = 0.56 , mean squared errors = 0.556

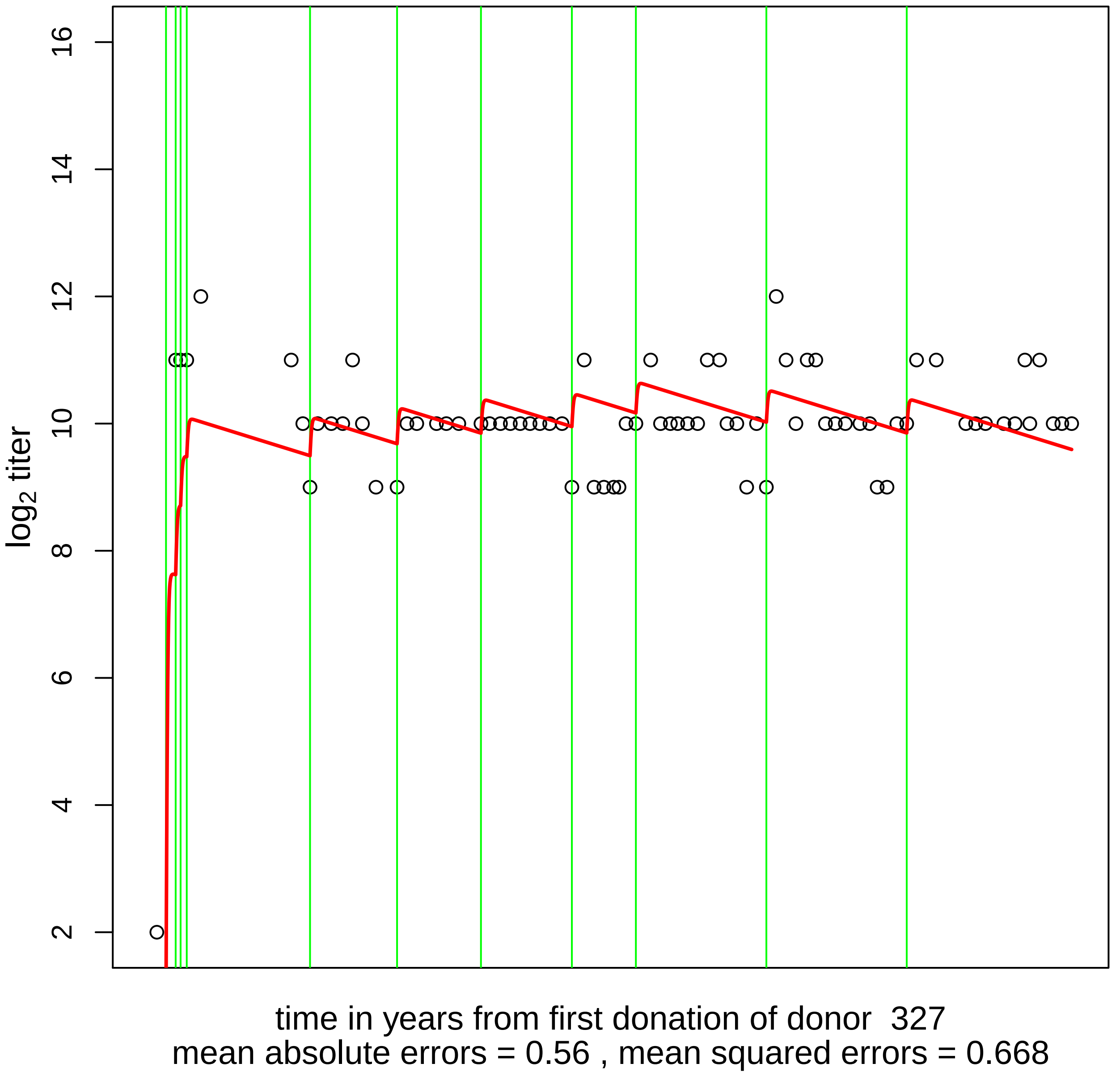

16
14
12
10
8
6
4
2
log2 titer
time in years from first donation of donor 327
mean absolute errors = 0.56 , mean squared errors = 0.668

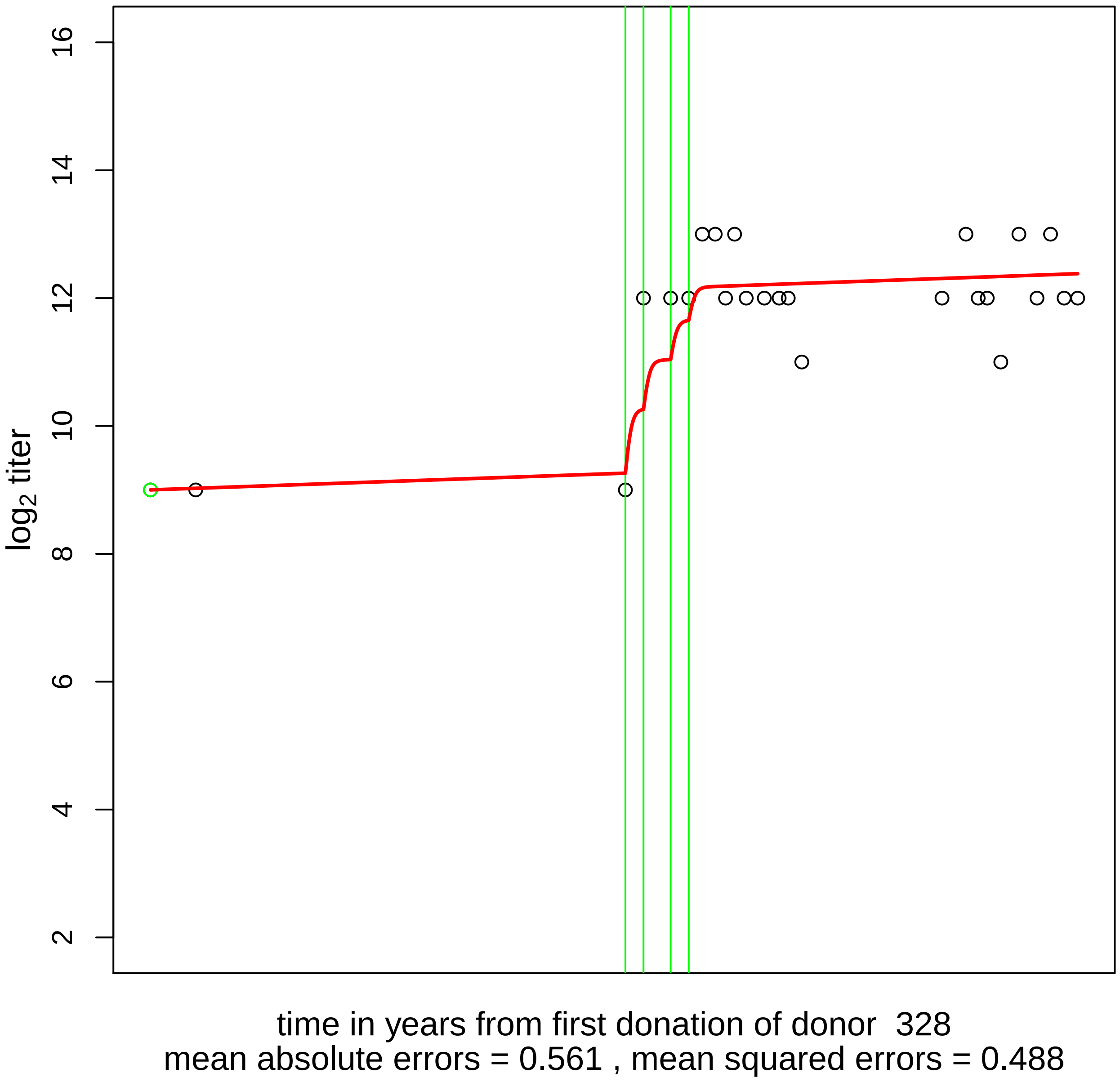

16
14
12
10
8
6
4
2
$\log_2$ titer
time in years from first donation of donor 328
mean absolute errors = 0.561 , mean squared errors = 0.488

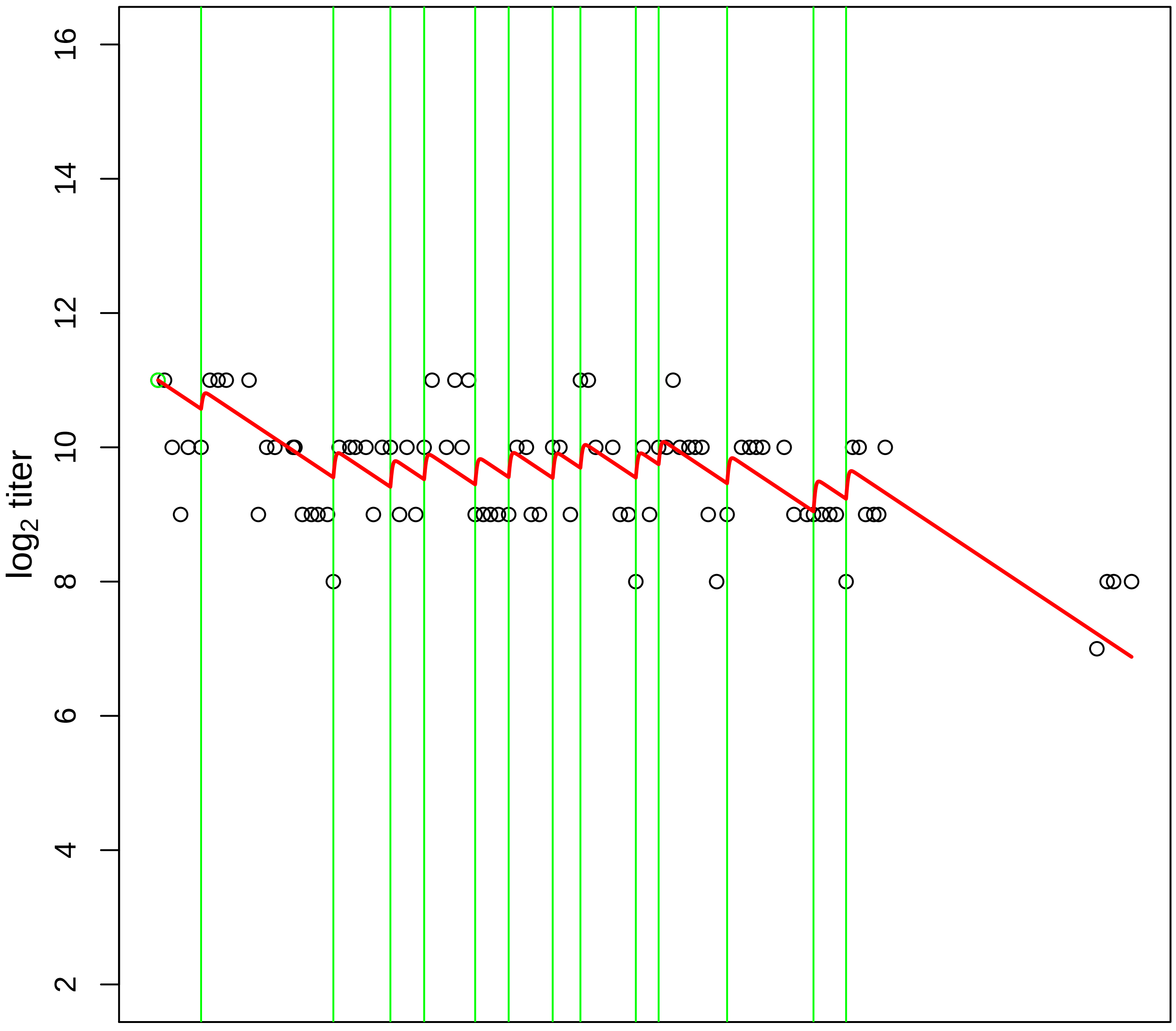

time in years from first donation of donor 329
mean absolute errors = 0.561 , mean squared errors = 0.48

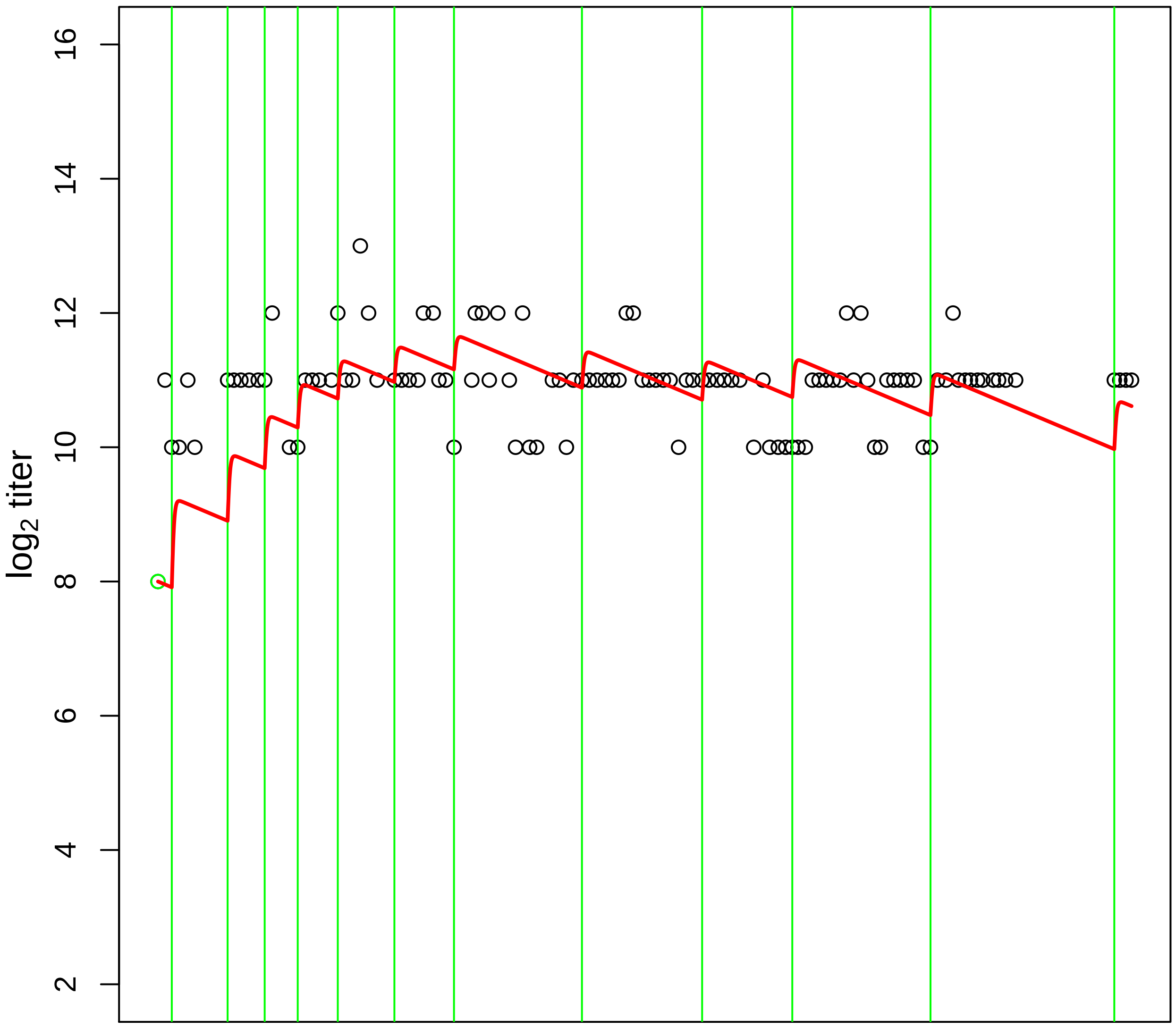

time in years from first donation of donor 330
mean absolute errors = 0.562 , mean squared errors = 0.603

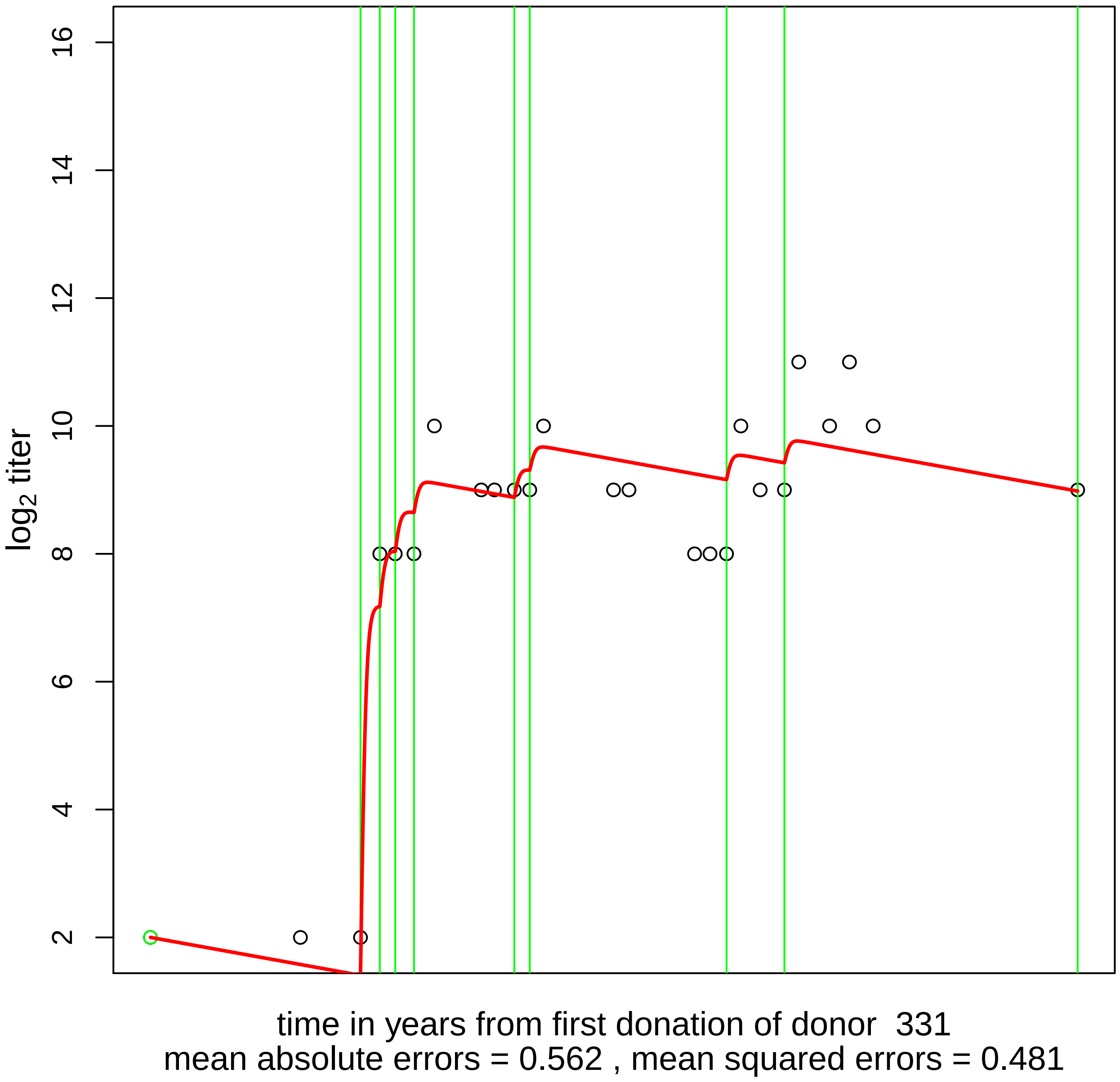

log2 titer
2
4
6
8
10
12
14
16
time in years from first donation of donor 331
mean absolute errors = 0.562 , mean squared errors = 0.481

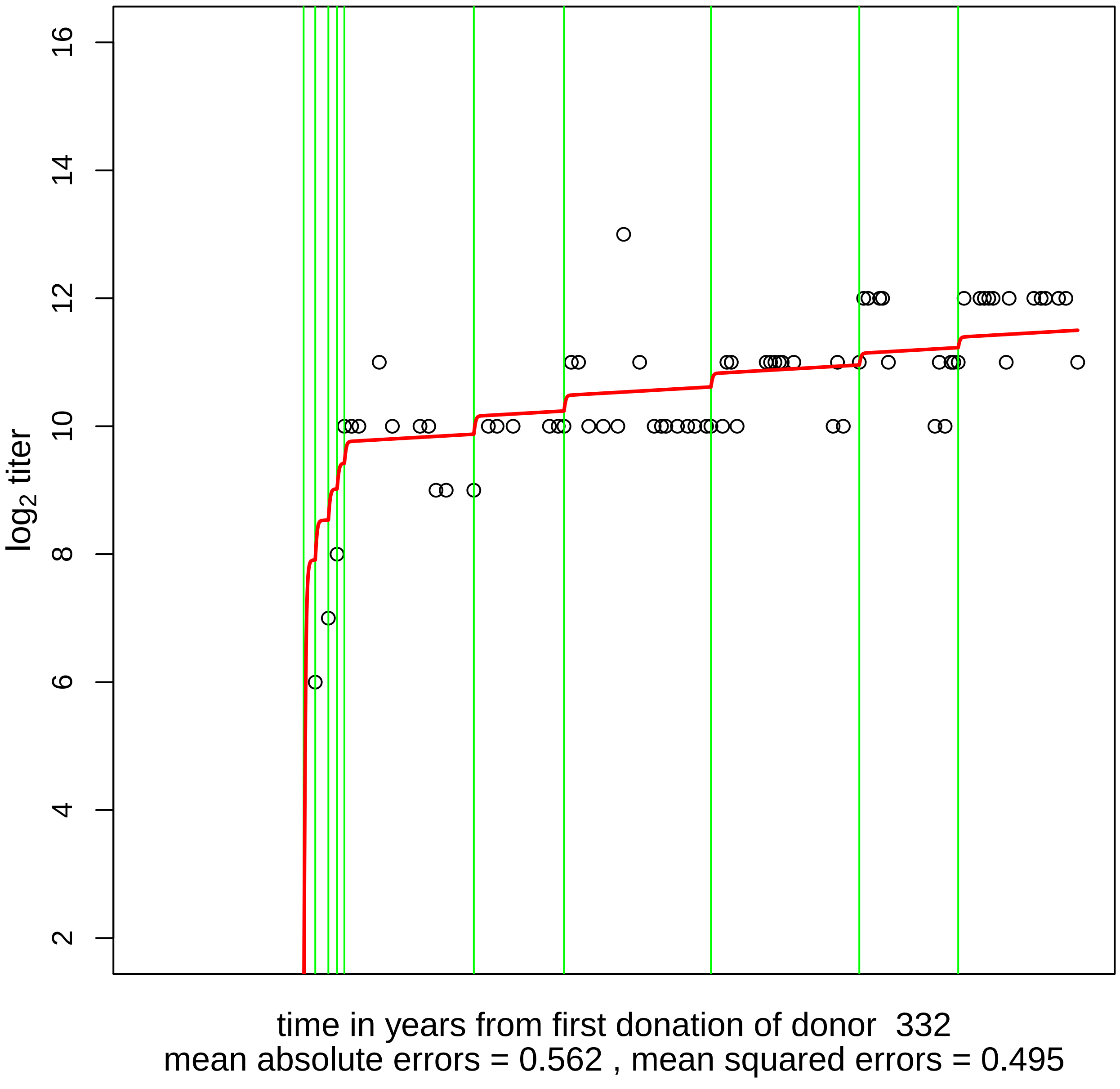

16
14
12
10
8
6
4
2
log2 titer
time in years from first donation of donor 332
mean absolute errors = 0.562 , mean squared errors = 0.495

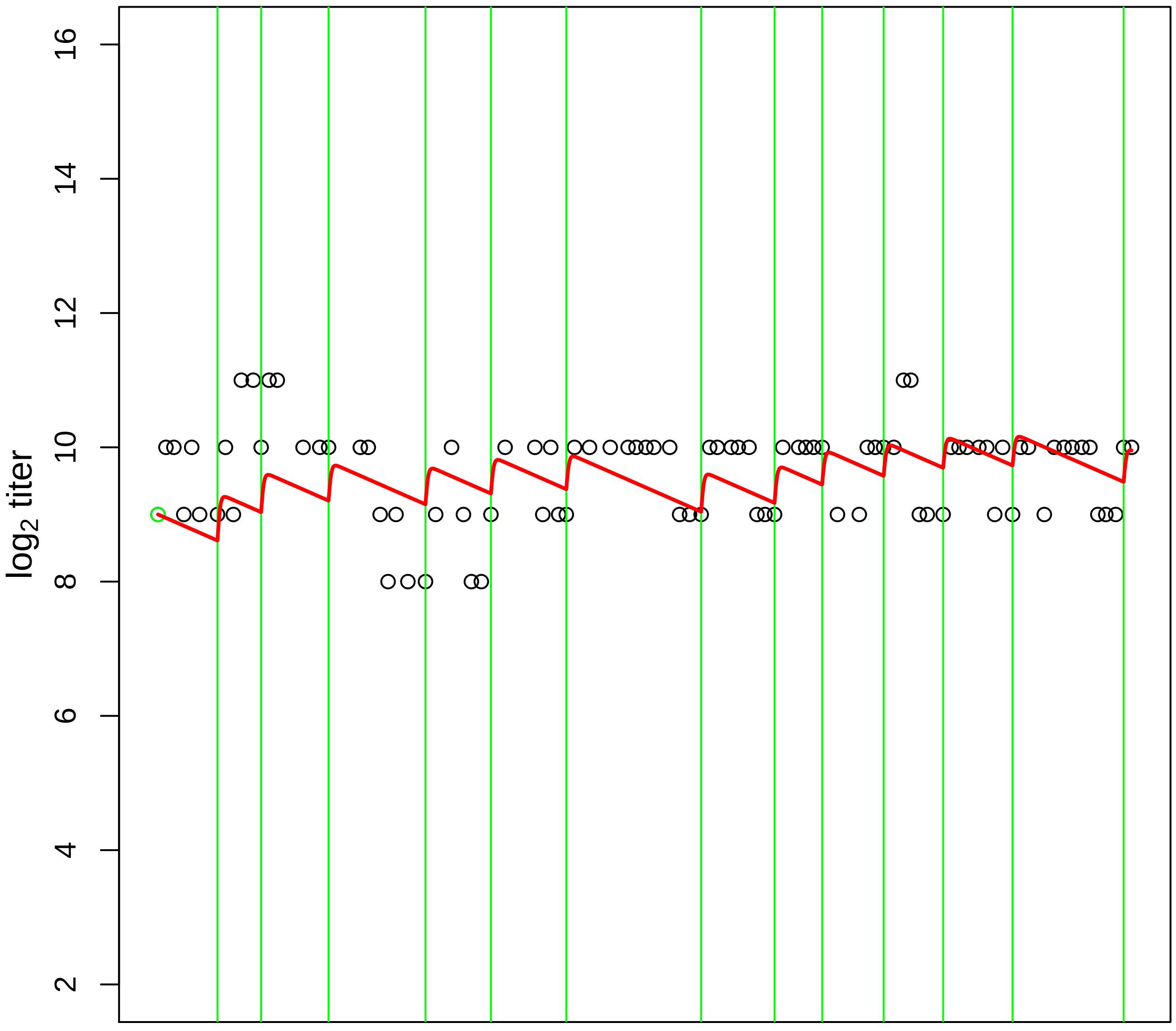

time in years from first donation of donor 333
mean absolute errors = 0.562 , mean squared errors = 0.489

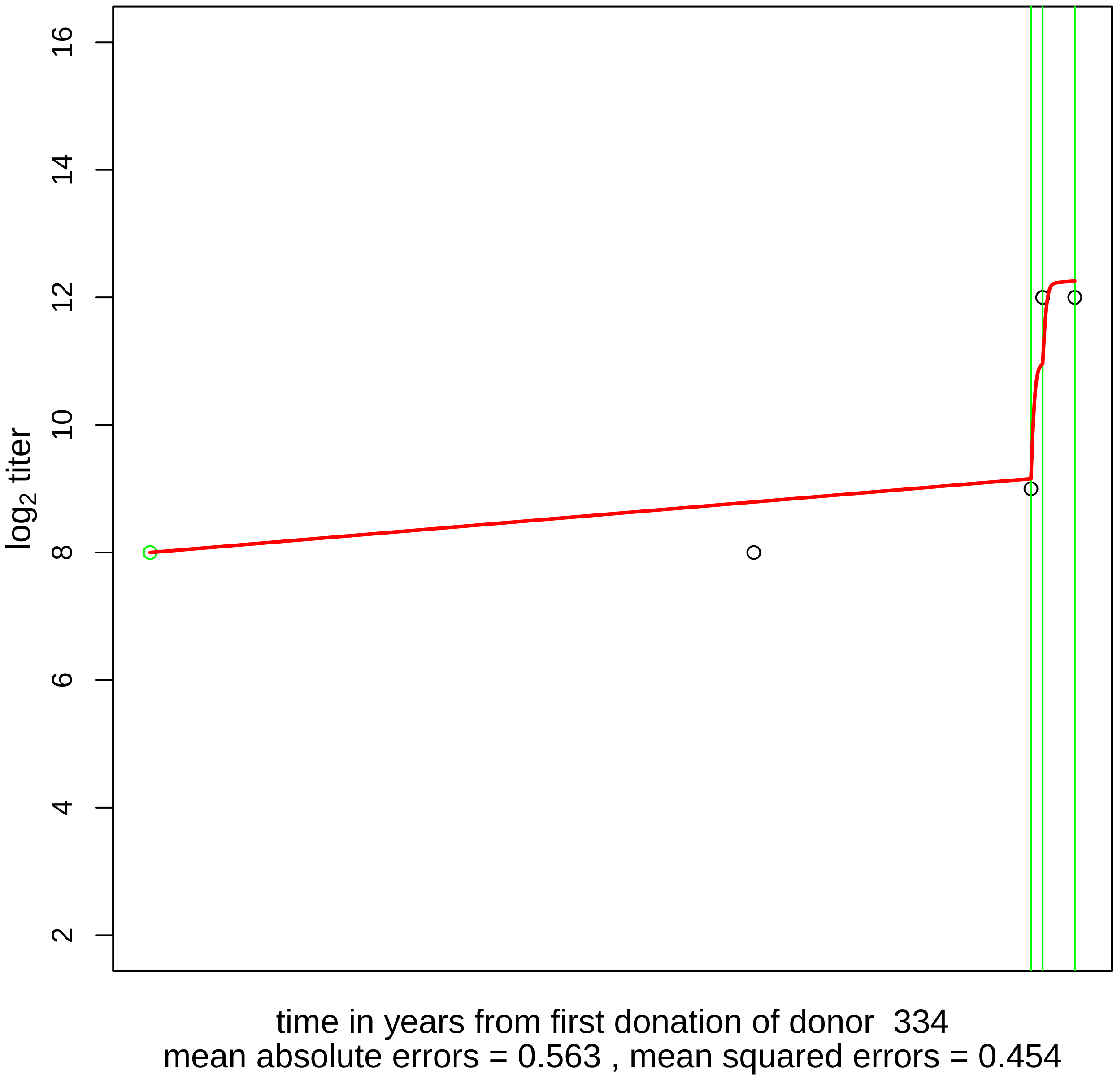

log2 titer
16
14
12
10
8
6
4
2
time in years from first donation of donor 334
mean absolute errors = 0.563 , mean squared errors = 0.454

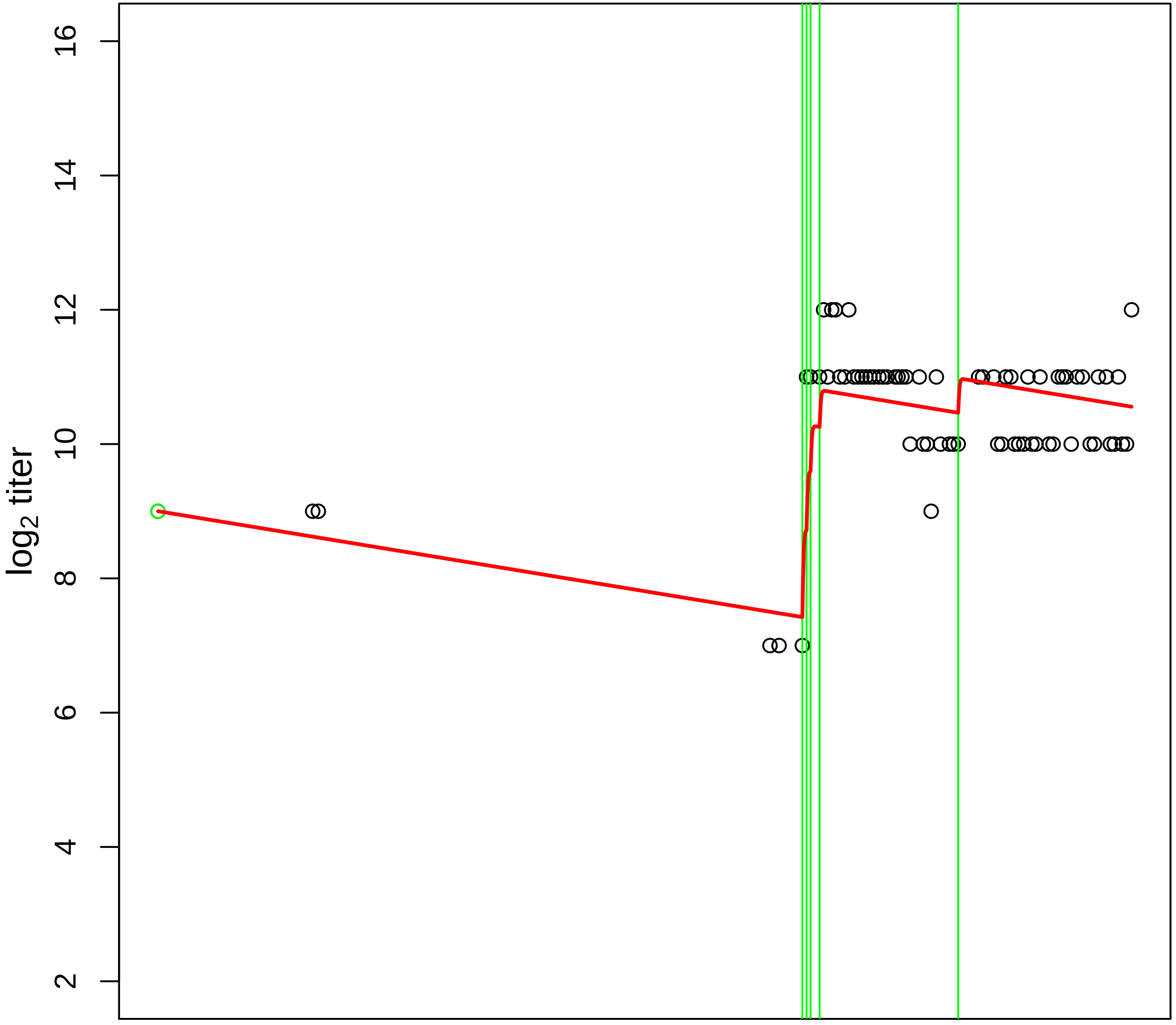

time in years from first donation of donor 335
mean absolute errors = 0.564 , mean squared errors = 0.472

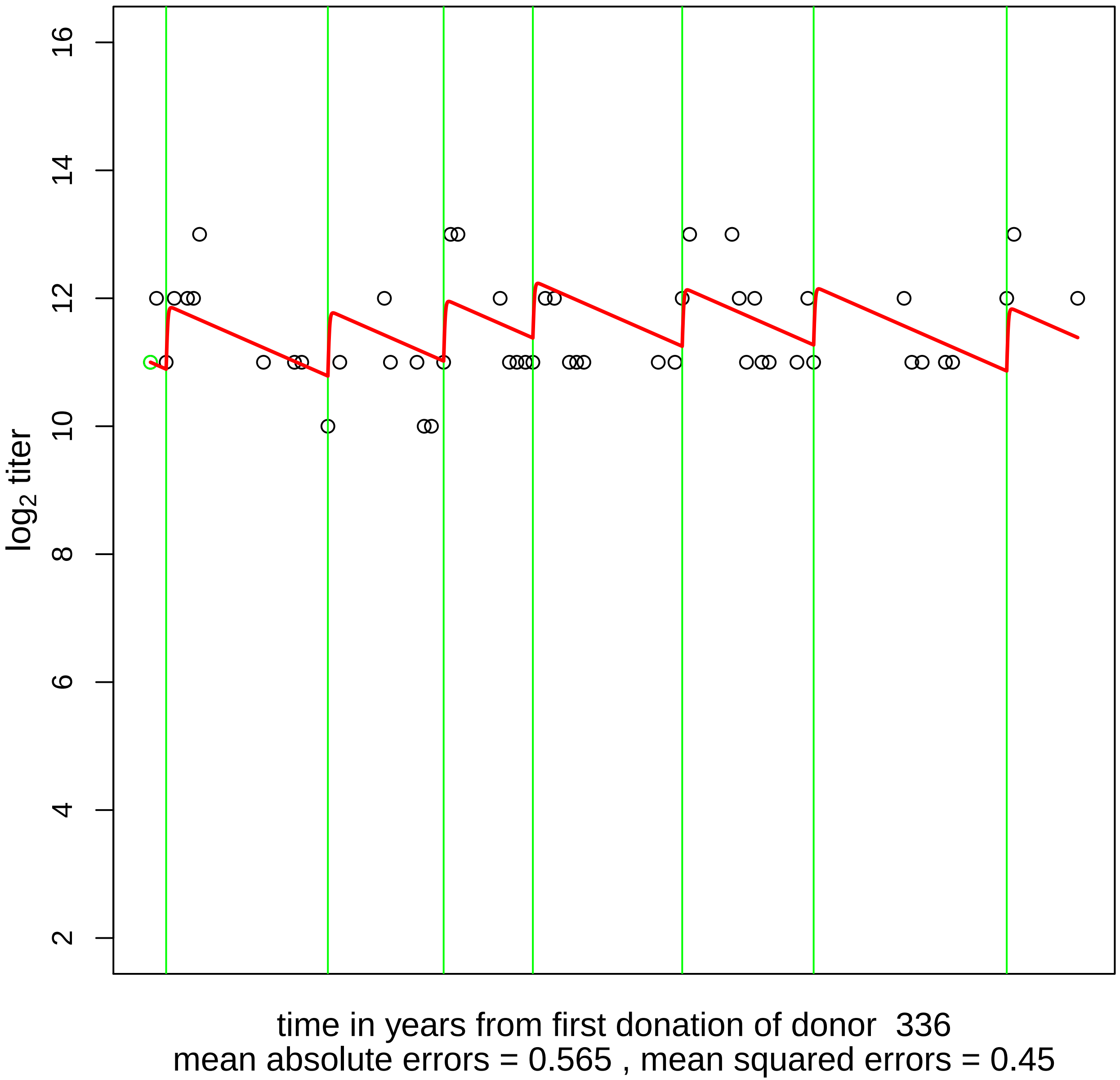

16
14
12
10
8
6
4
2
$\log_2$ titer
time in years from first donation of donor 336
mean absolute errors = 0.565 , mean squared errors = 0.45

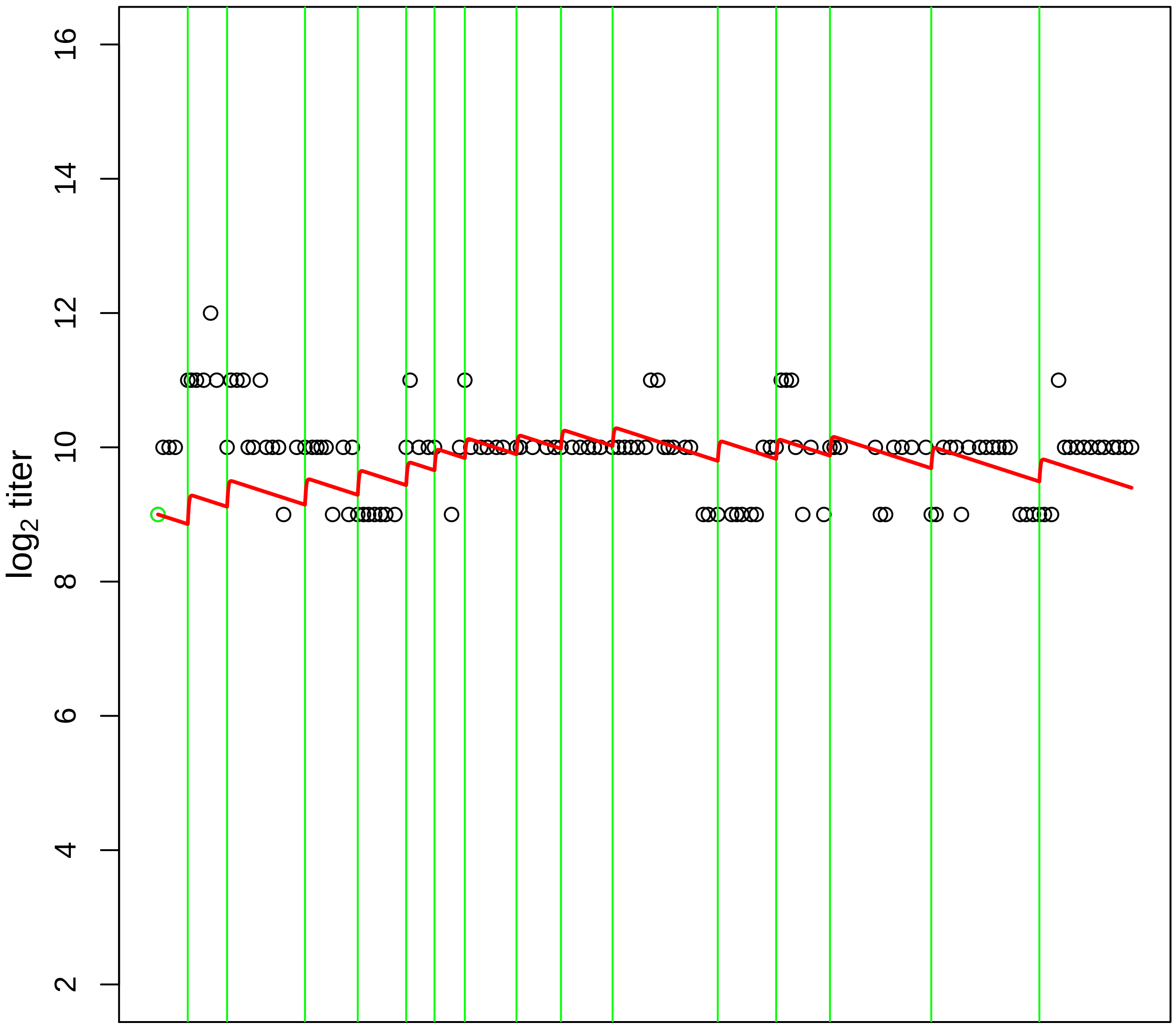

time in years from first donation of donor 337
mean absolute errors = 0.565 , mean squared errors = 0.567

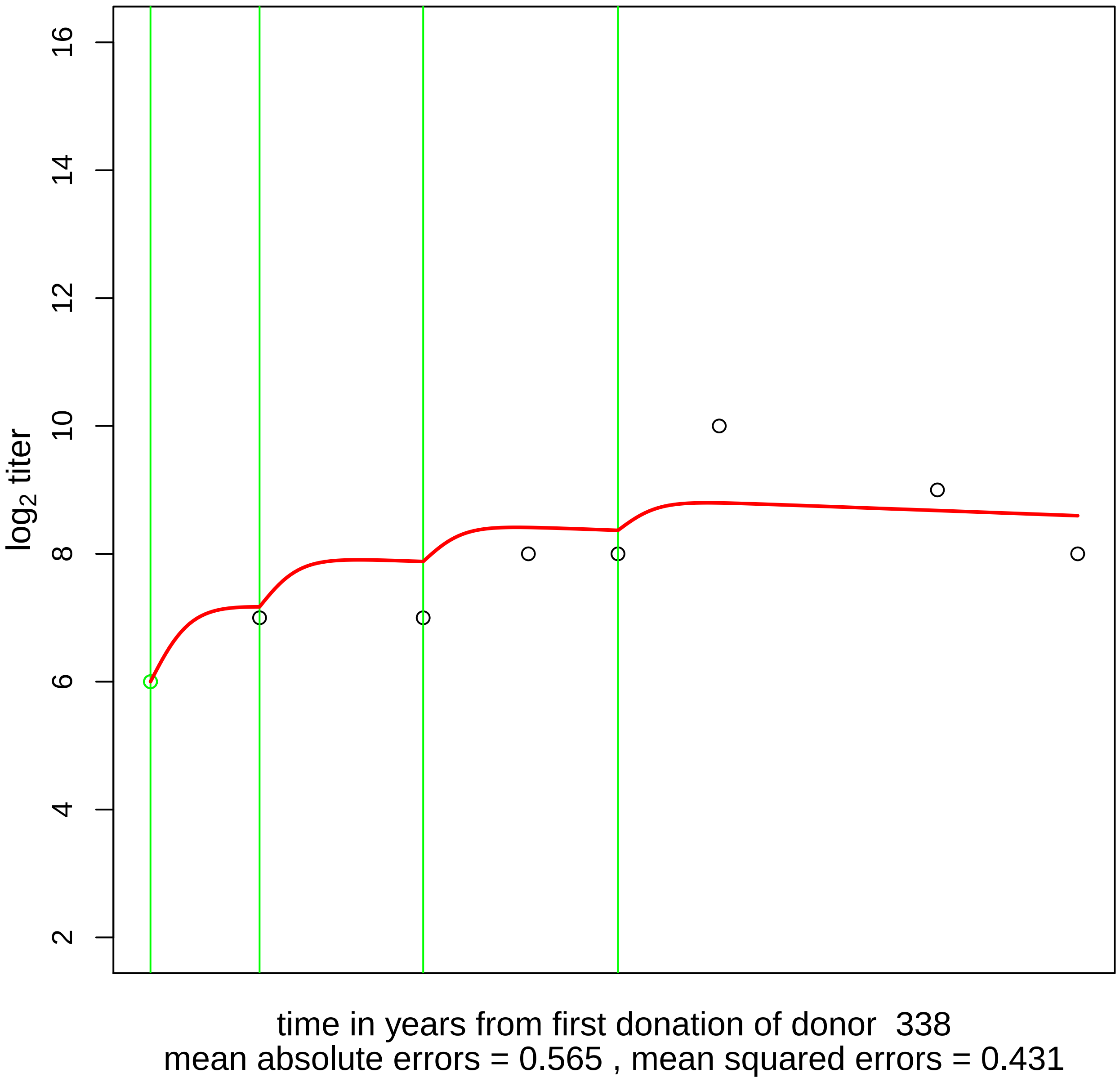

log2 titer
2
4
6
8
10
12
14
16
time in years from first donation of donor 338
mean absolute errors = 0.565 , mean squared errors = 0.431

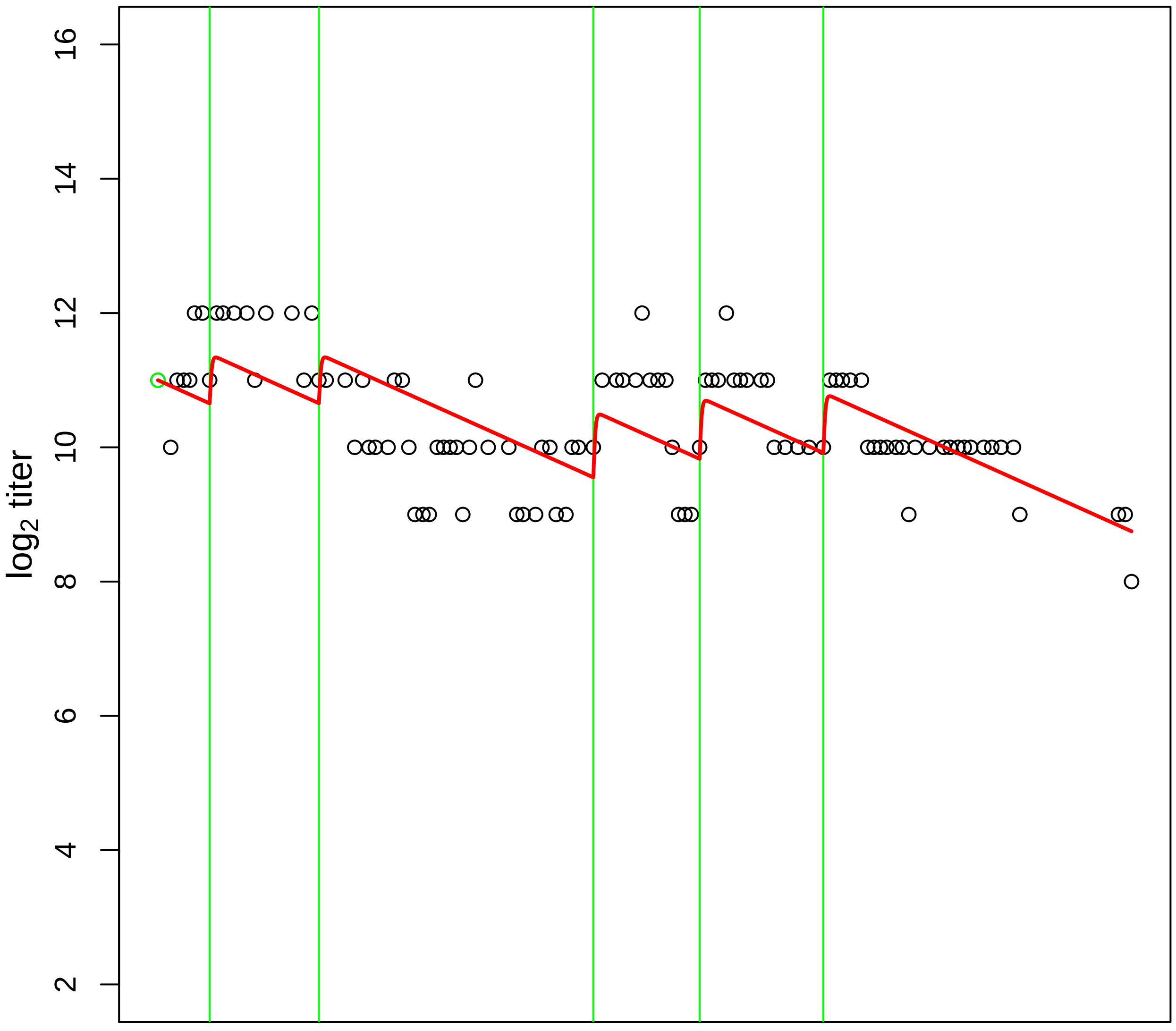

time in years from first donation of donor 339
mean absolute errors = 0.566 , mean squared errors = 0.503

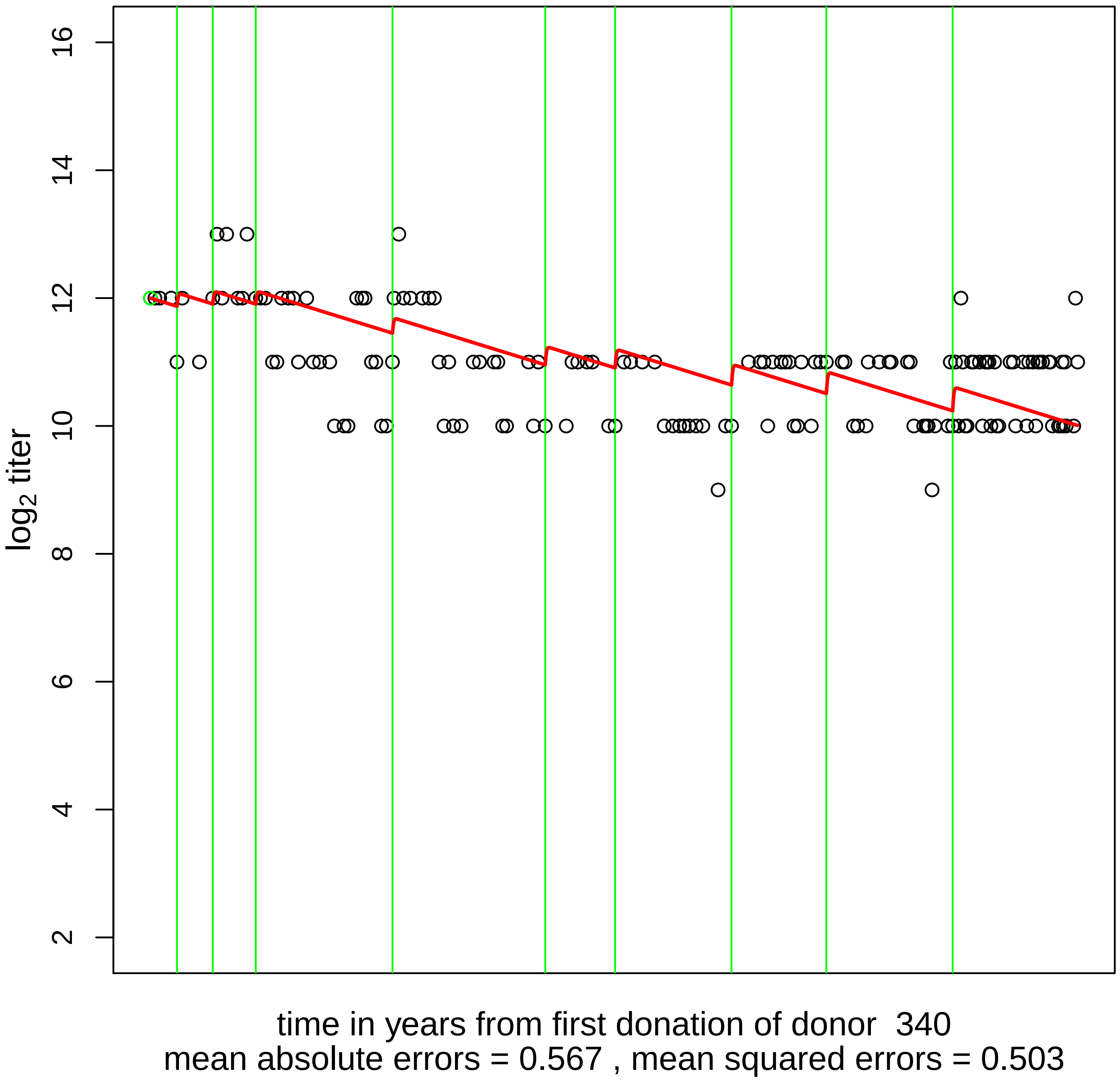

16
14
12
10
8
6
4
2
log_2 titer
time in years from first donation of donor 340
mean absolute errors = 0.567 , mean squared errors = 0.503

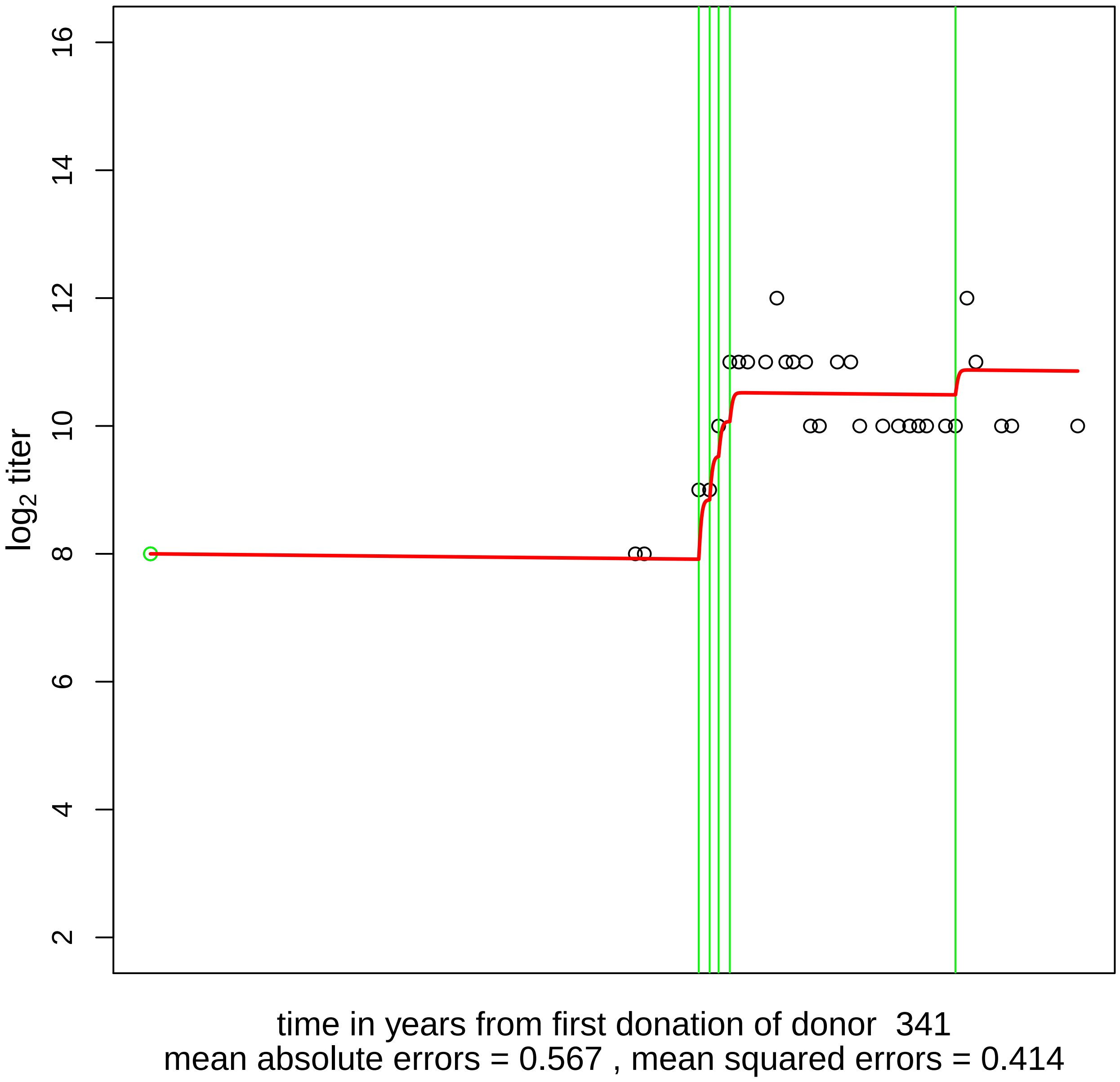

log2 titer
16
14
12
10
8
6
4
2
time in years from first donation of donor 341
mean absolute errors = 0.567 , mean squared errors = 0.414

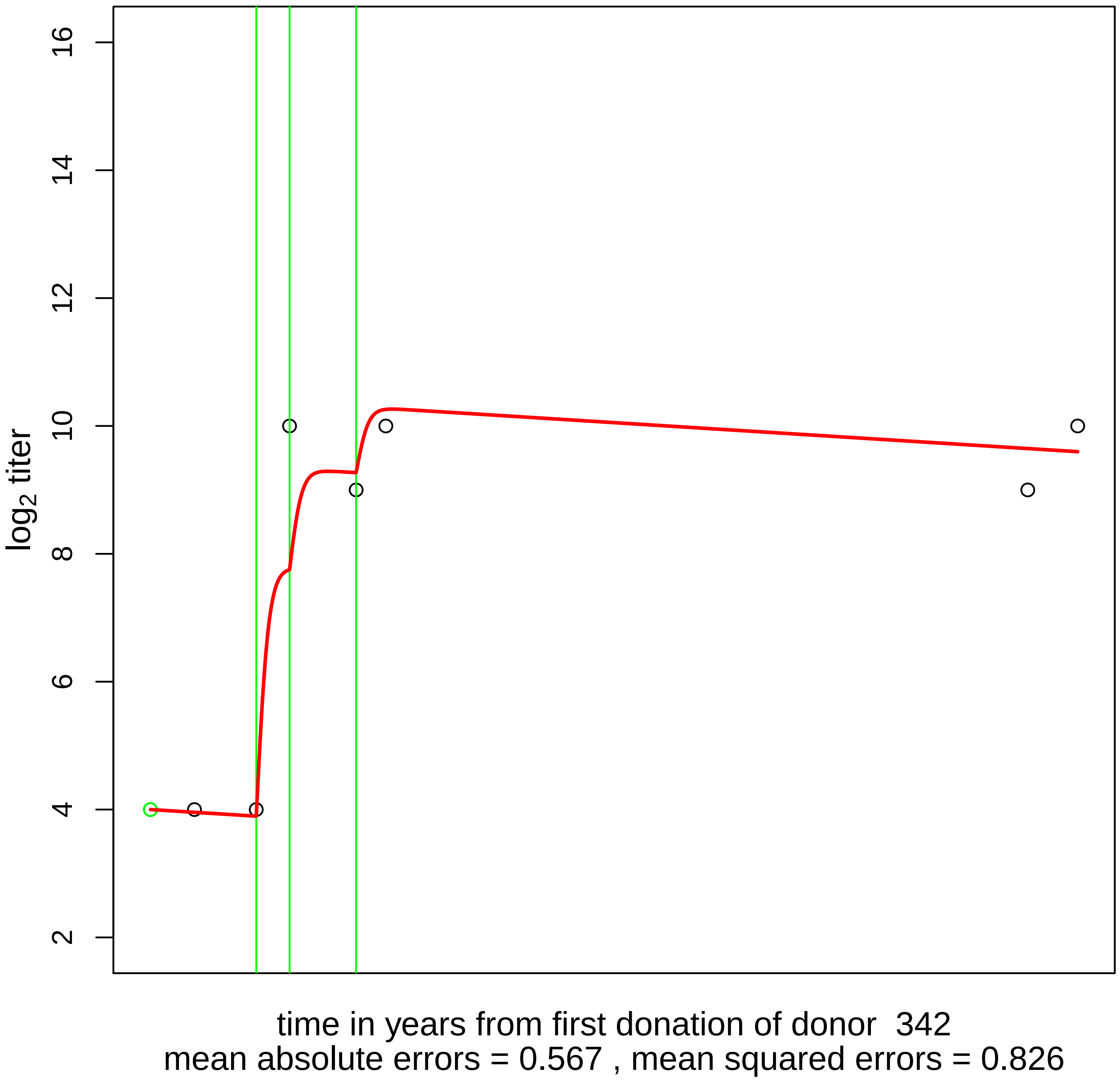

log2 titer
2
4
6
8
10
12
14
16
time in years from first donation of donor 342
mean absolute errors = 0.567 , mean squared errors = 0.826

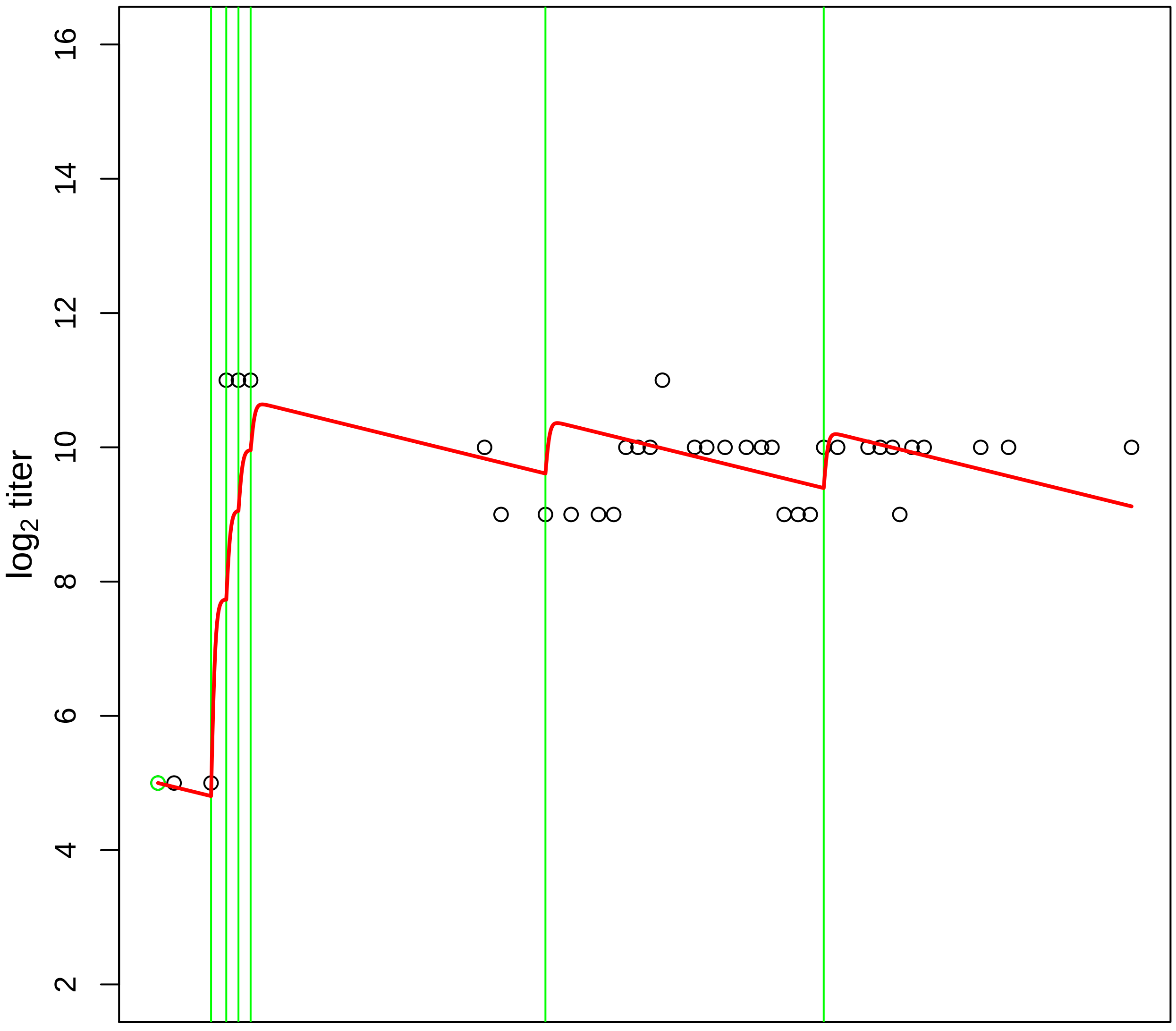

time in years from first donation of donor 343
mean absolute errors = 0.568 , mean squared errors = 0.741

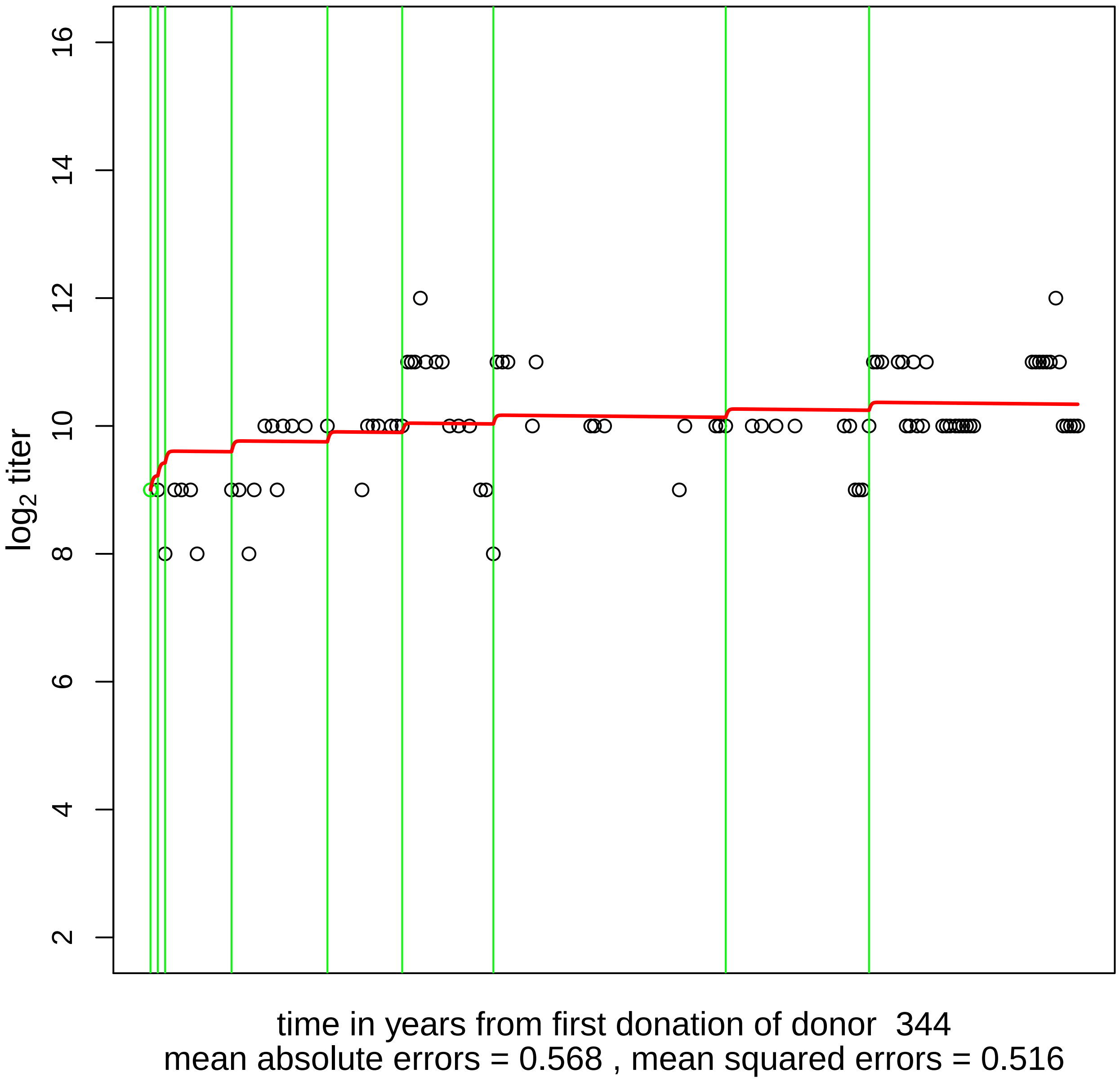

log2 titer
2
4
6
8
10
12
14
16
time in years from first donation of donor 344
mean absolute errors = 0.568 , mean squared errors = 0.516

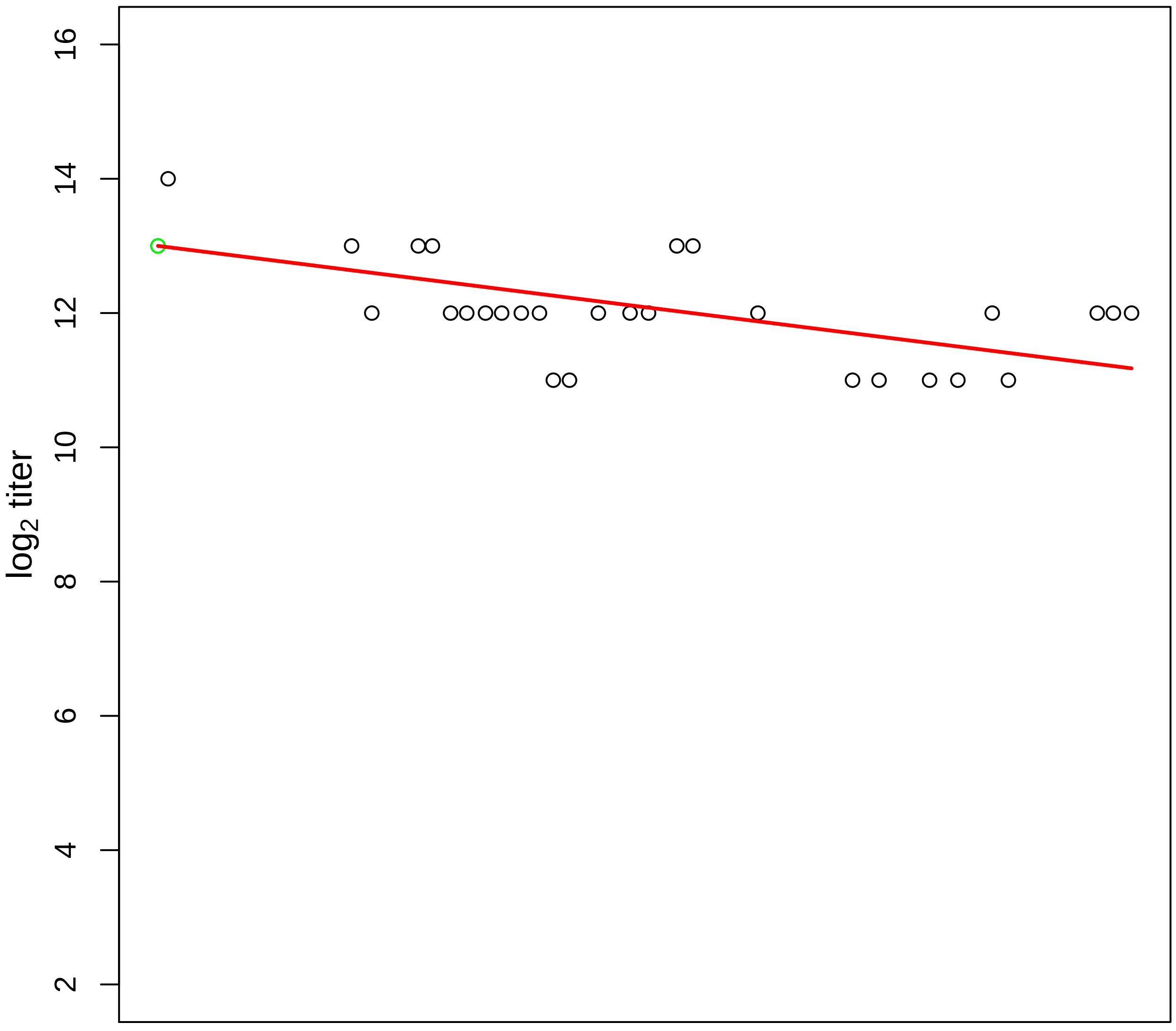

time in years from first donation of donor 345
mean absolute errors = 0.568 , mean squared errors = 0.422

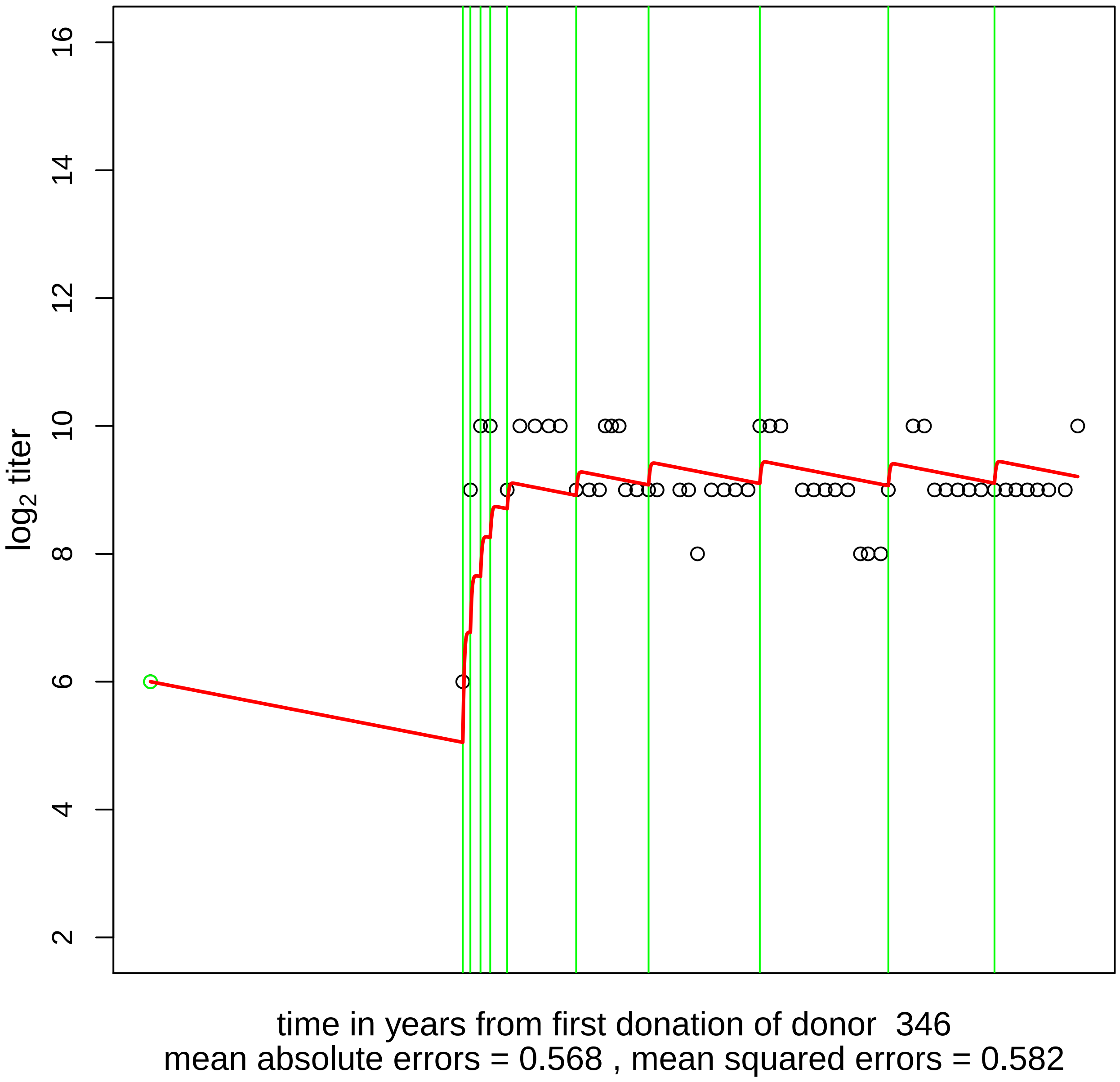
log2 titer
16
14
12
10
8
6
4
2
time in years from first donation of donor 346
mean absolute errors = 0.568 , mean squared errors = 0.582

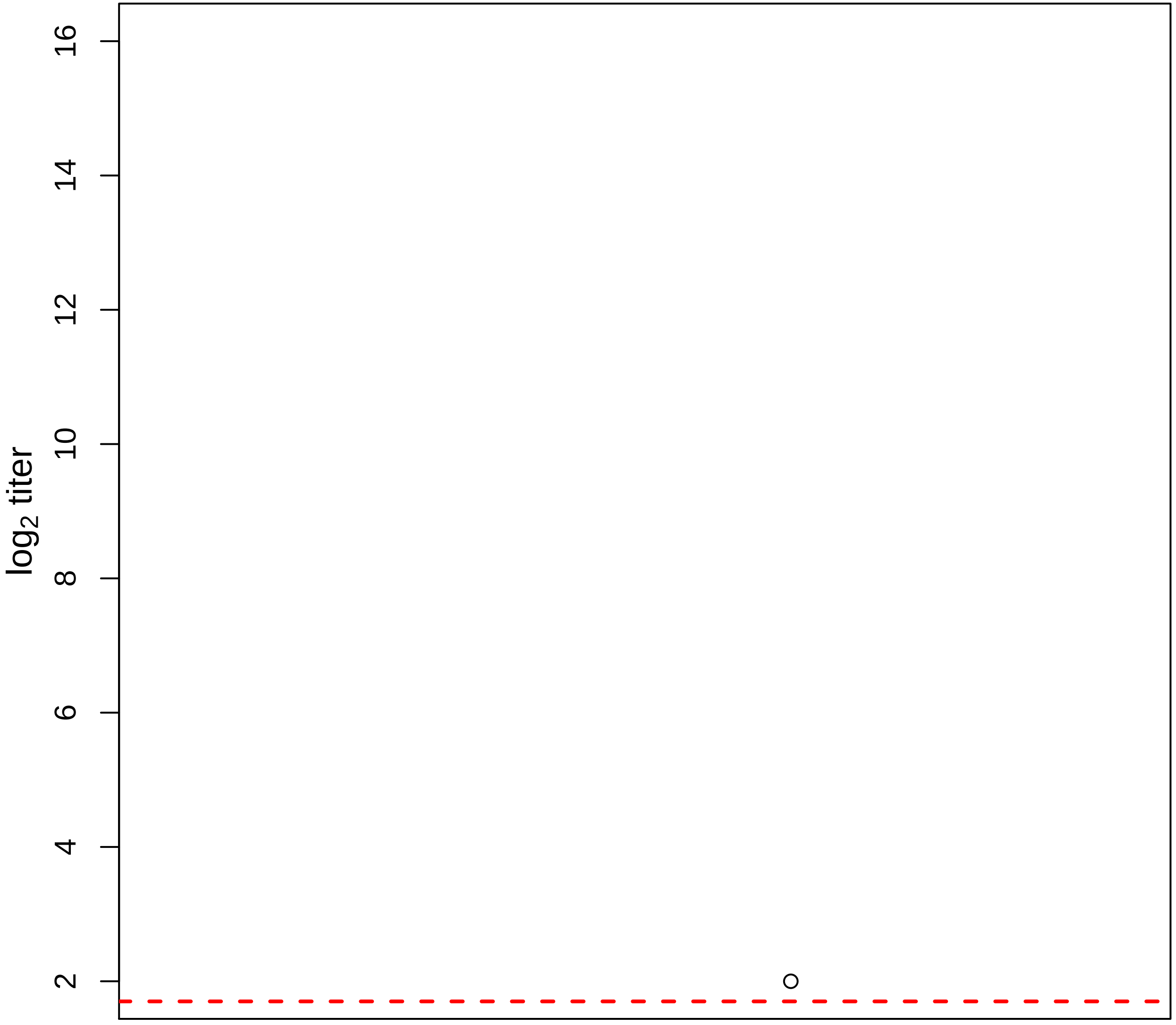

time in years from first donation of donor 347
mean absolute errors = 0.57 , mean squared errors = 0.56

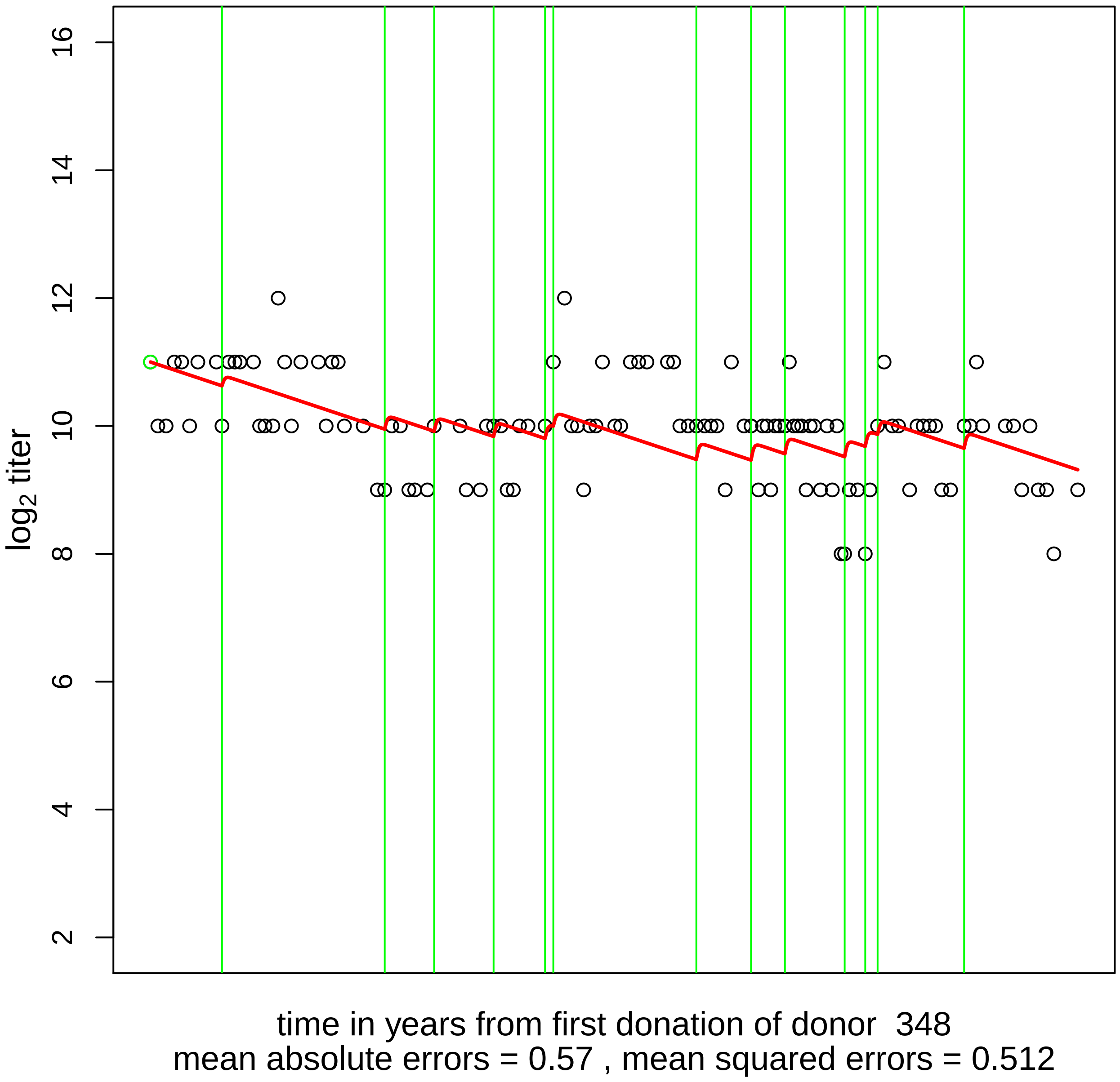

log2 titer
2
4
6
8
10
12
14
16
time in years from first donation of donor 348
mean absolute errors = 0.57 , mean squared errors = 0.512

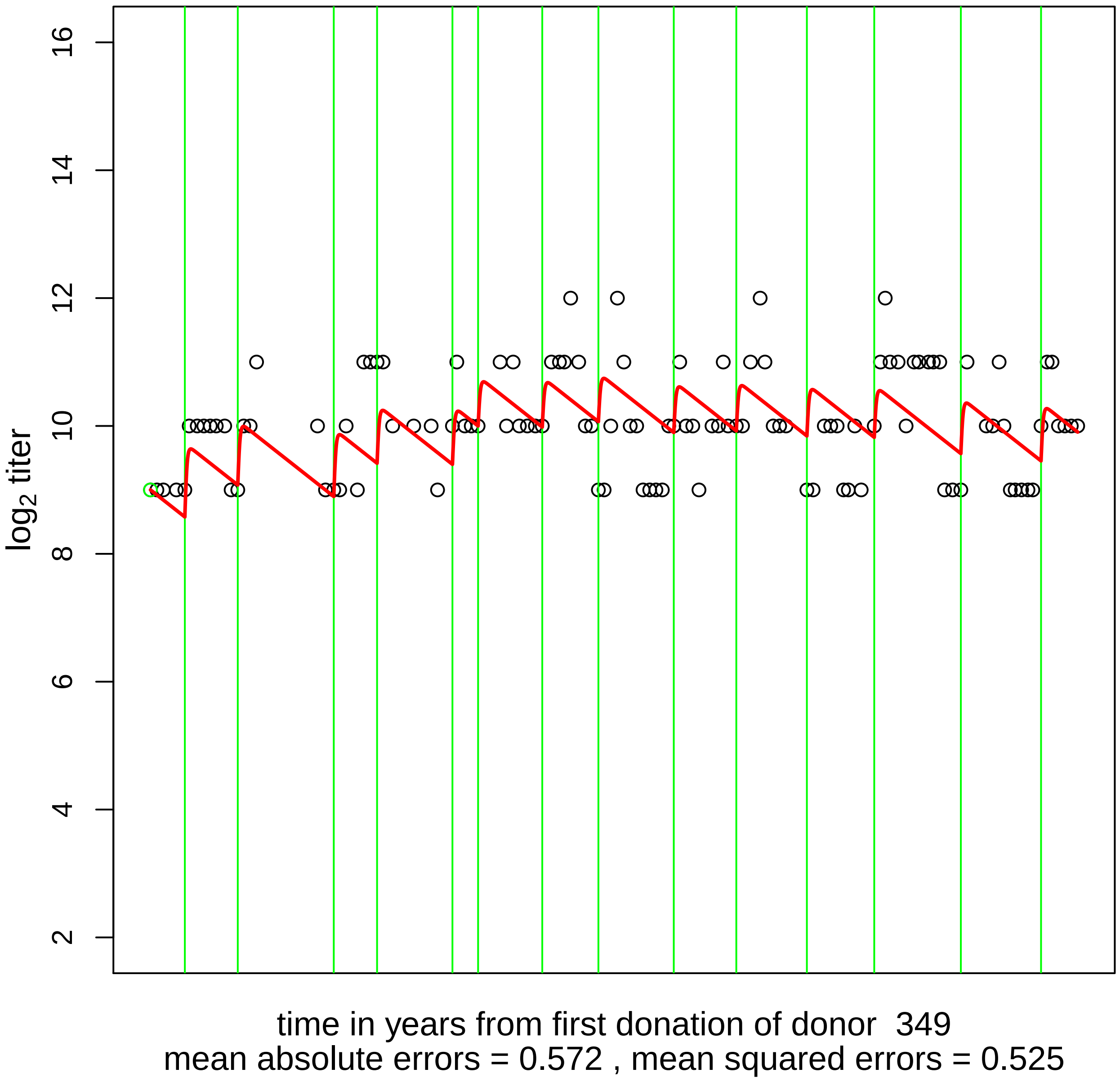

2
4
6
8
10
12
14
16
$\log_2$ titer
time in years from first donation of donor 349
mean absolute errors = 0.572 , mean squared errors = 0.525

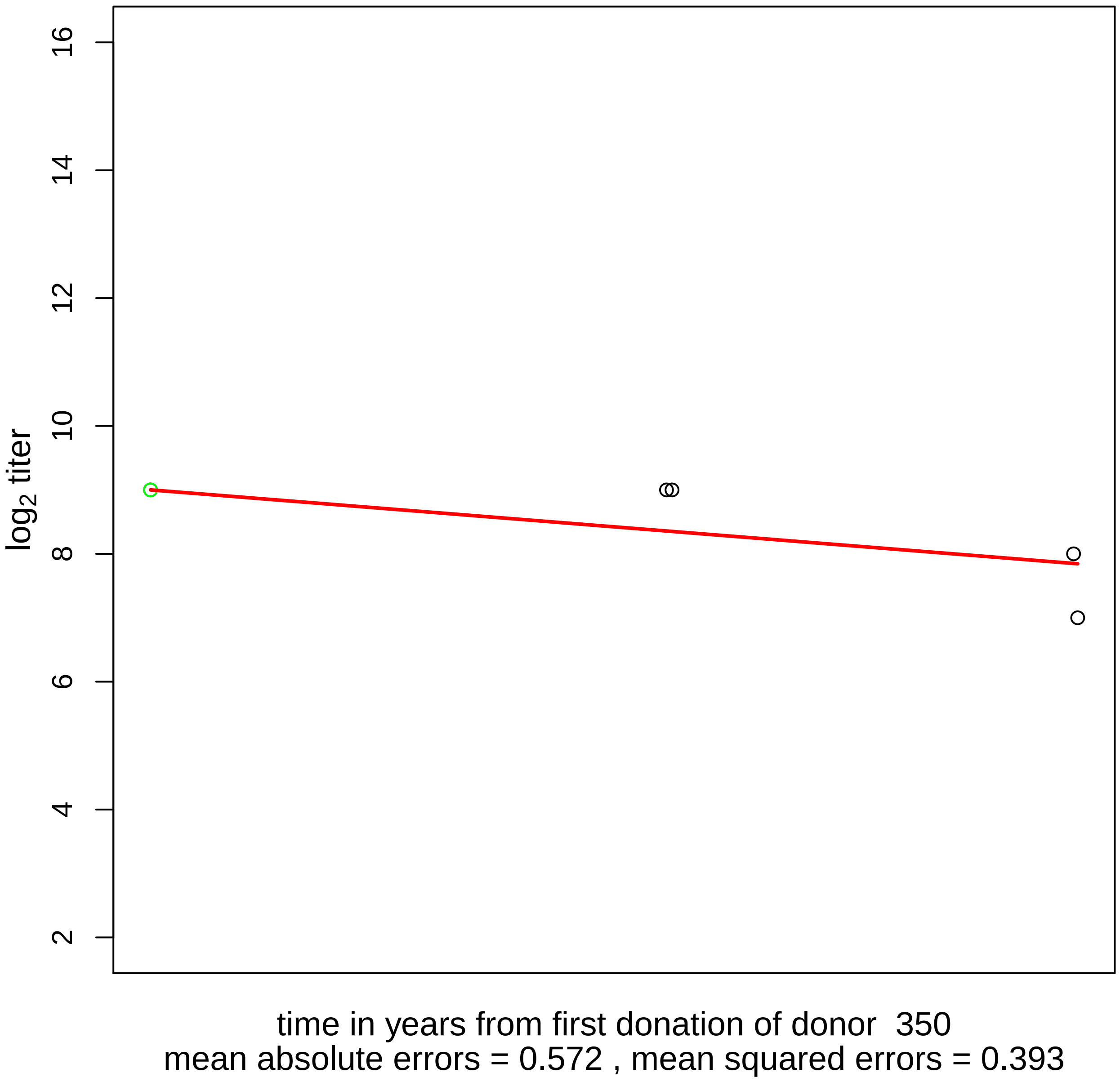

16
14
12
10
8
6
4
2
log2 titer
time in years from first donation of donor 350
mean absolute errors = 0.572 , mean squared errors = 0.393

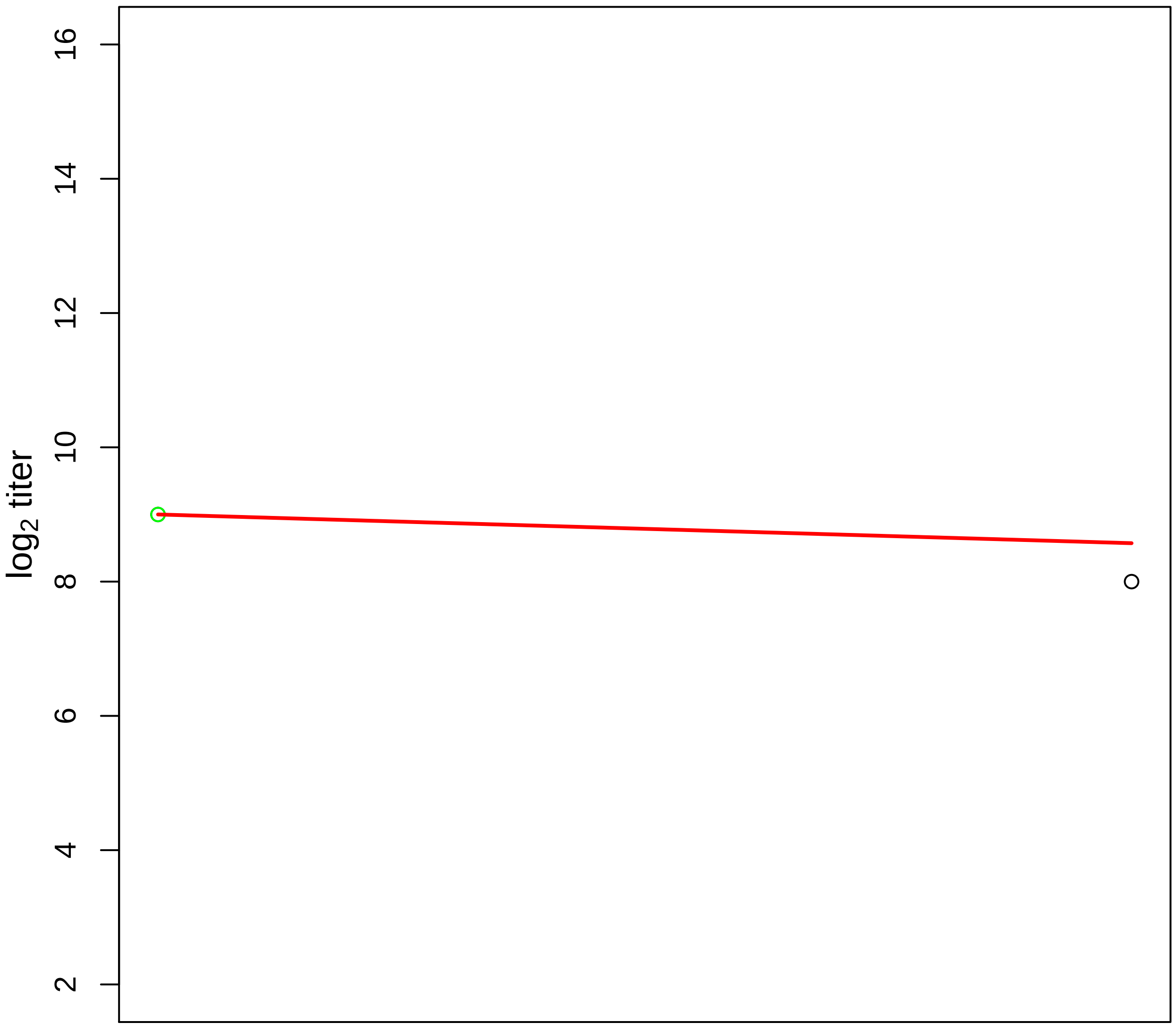

time in years from first donation of donor 351
mean absolute errors = 0.572 , mean squared errors = 0.327

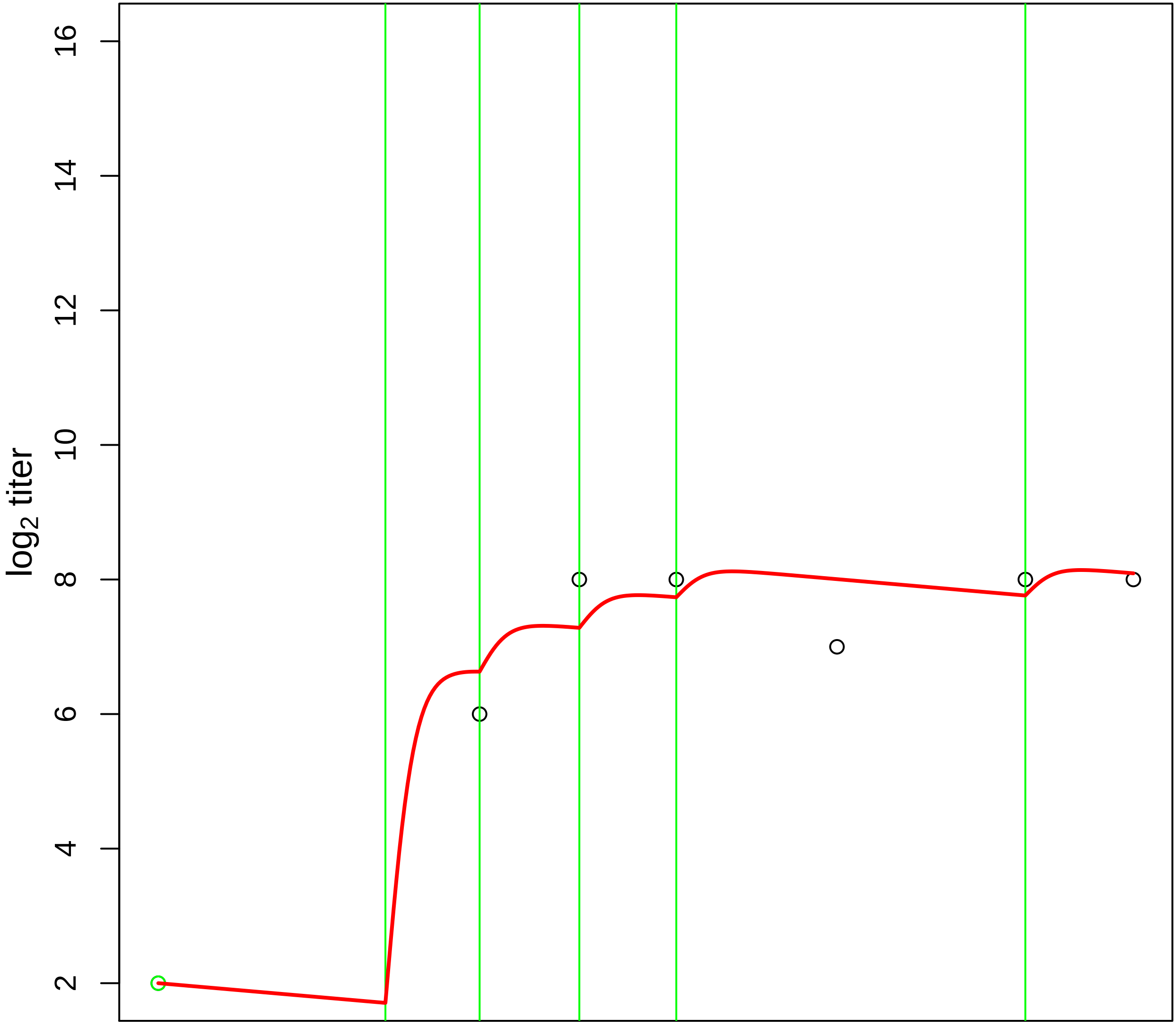

time in years from first donation of donor 352
mean absolute errors = 0.572 , mean squared errors = 0.427

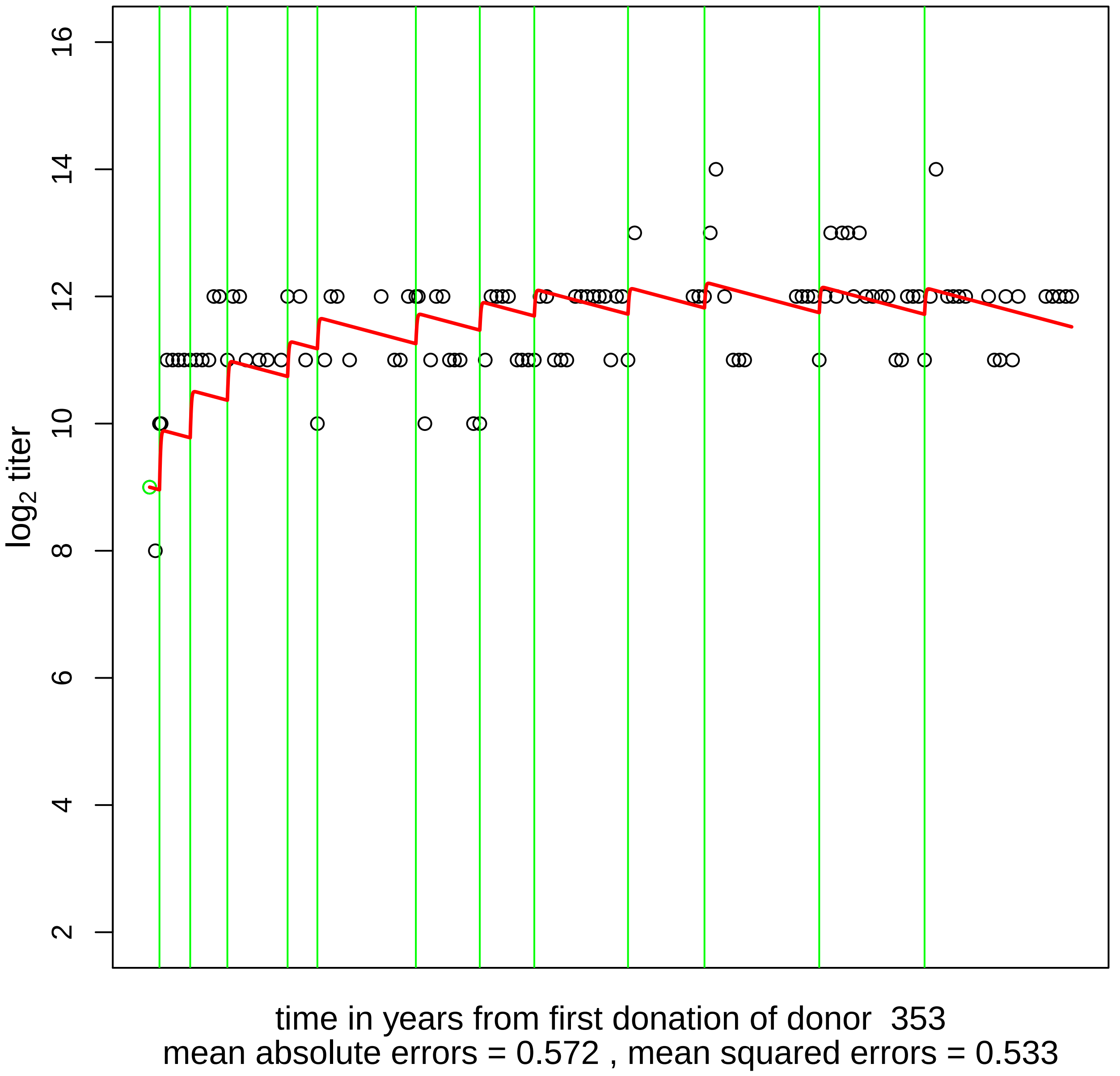

log2 titer
2
4
6
8
10
12
14
16
time in years from first donation of donor 353
mean absolute errors = 0.572 , mean squared errors = 0.533

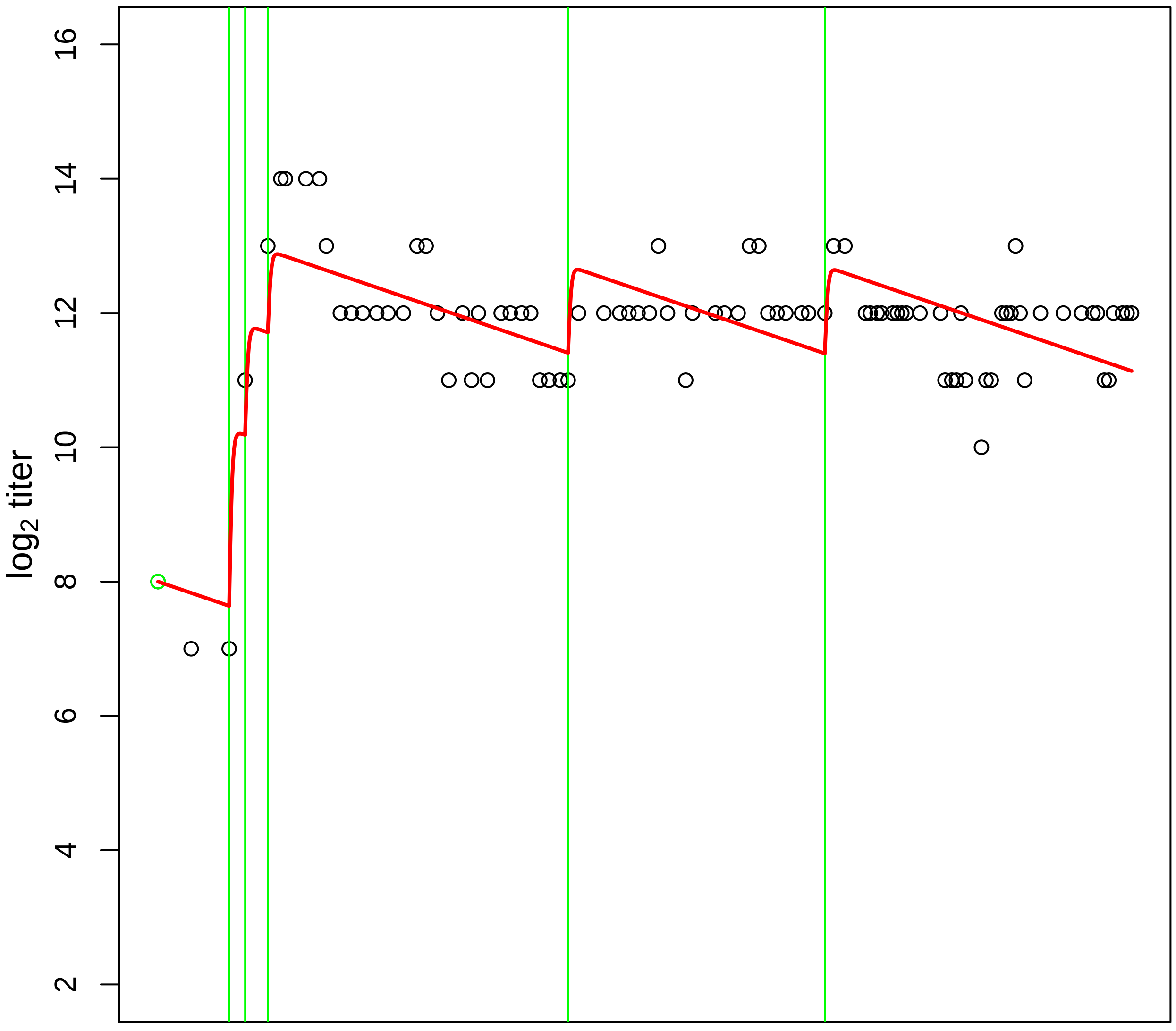

time in years from first donation of donor 354
mean absolute errors = 0.573 , mean squared errors = 0.464

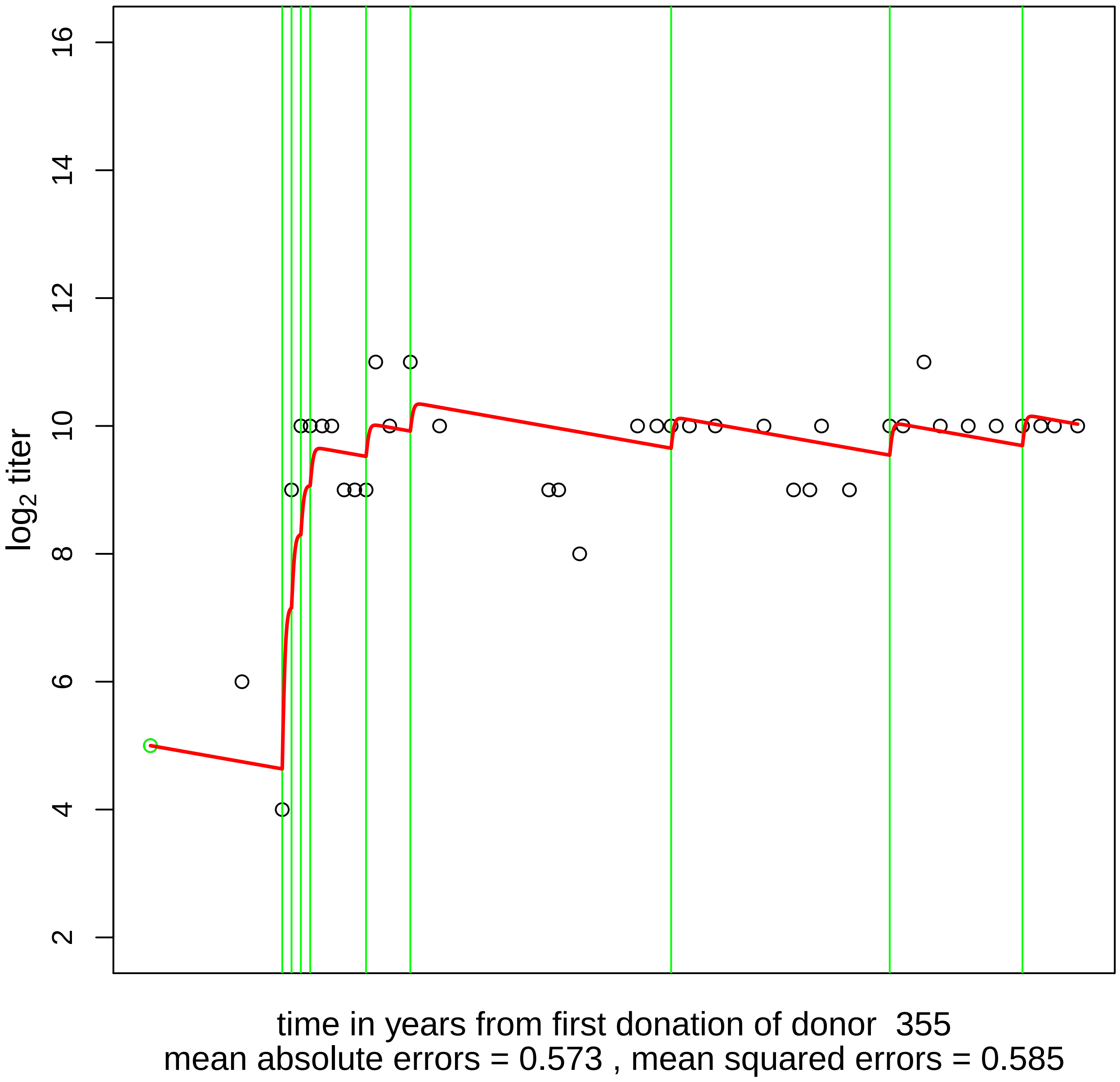

$\log_2$ titer
2
4
6
8
10
12
14
16
time in years from first donation of donor 355
mean absolute errors = 0.573 , mean squared errors = 0.585

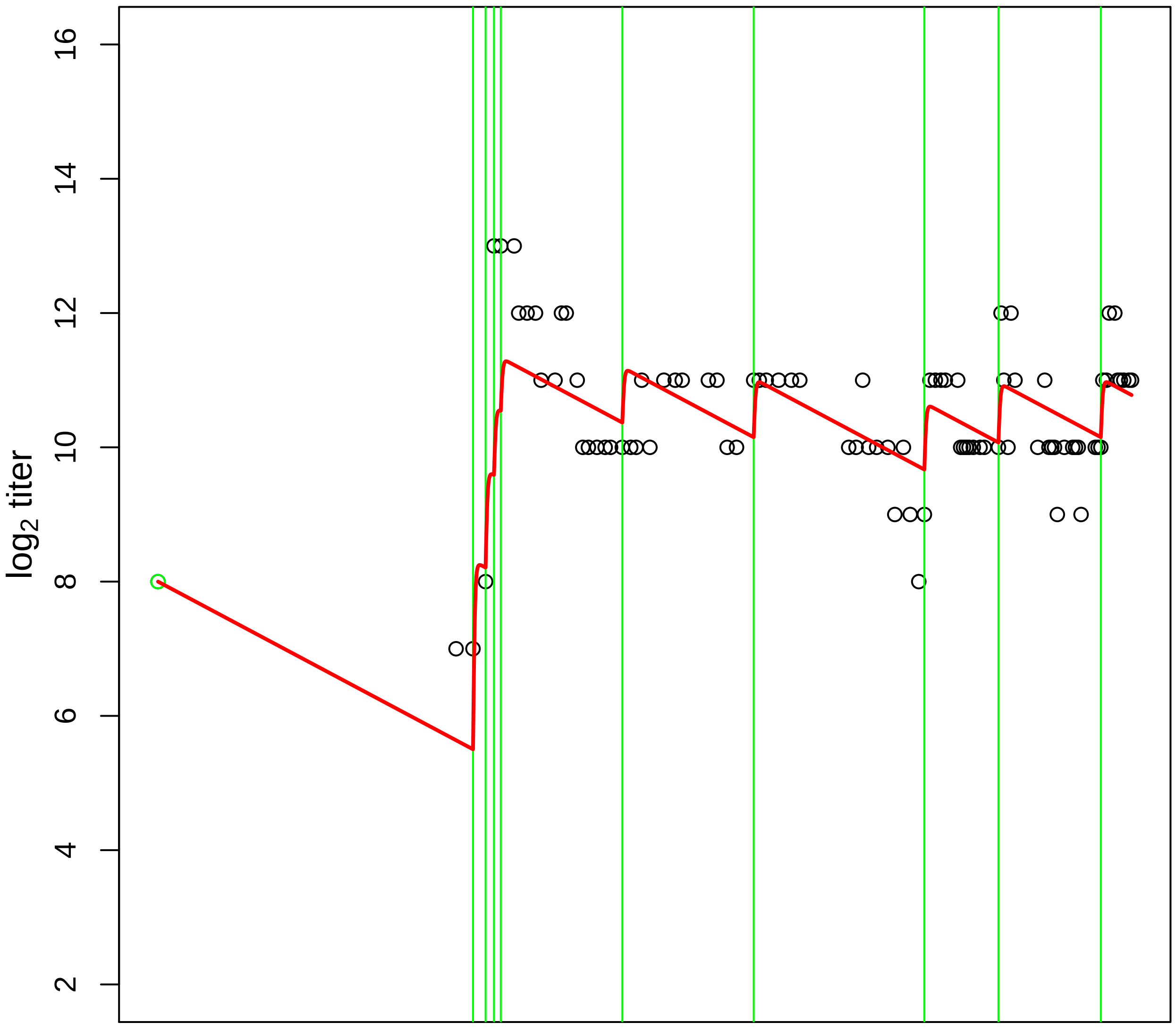

time in years from first donation of donor 356
mean absolute errors = 0.573 , mean squared errors = 0.637

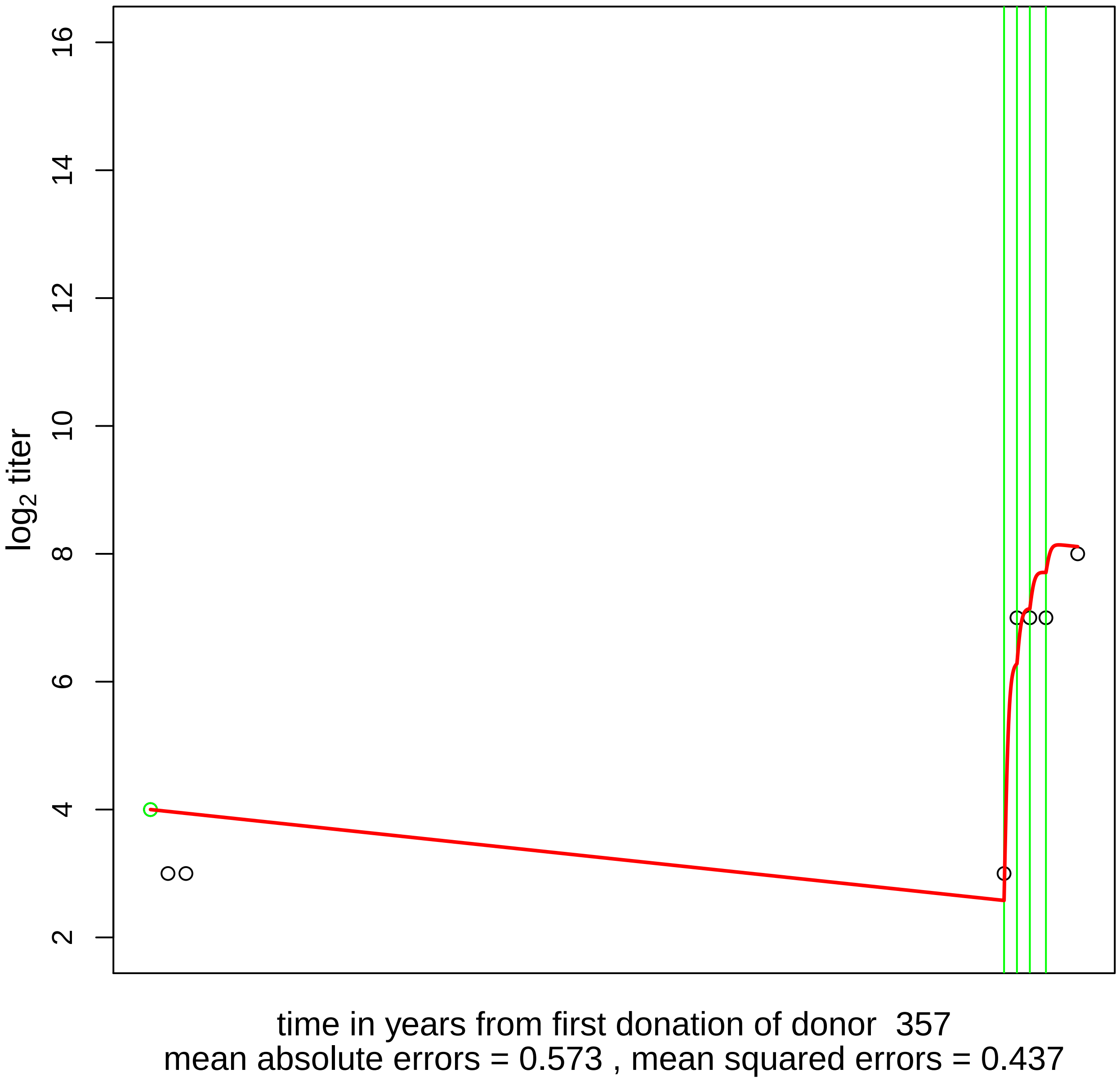

log2 titer
2
4
6
8
10
12
14
16
time in years from first donation of donor 357
mean absolute errors = 0.573 , mean squared errors = 0.437

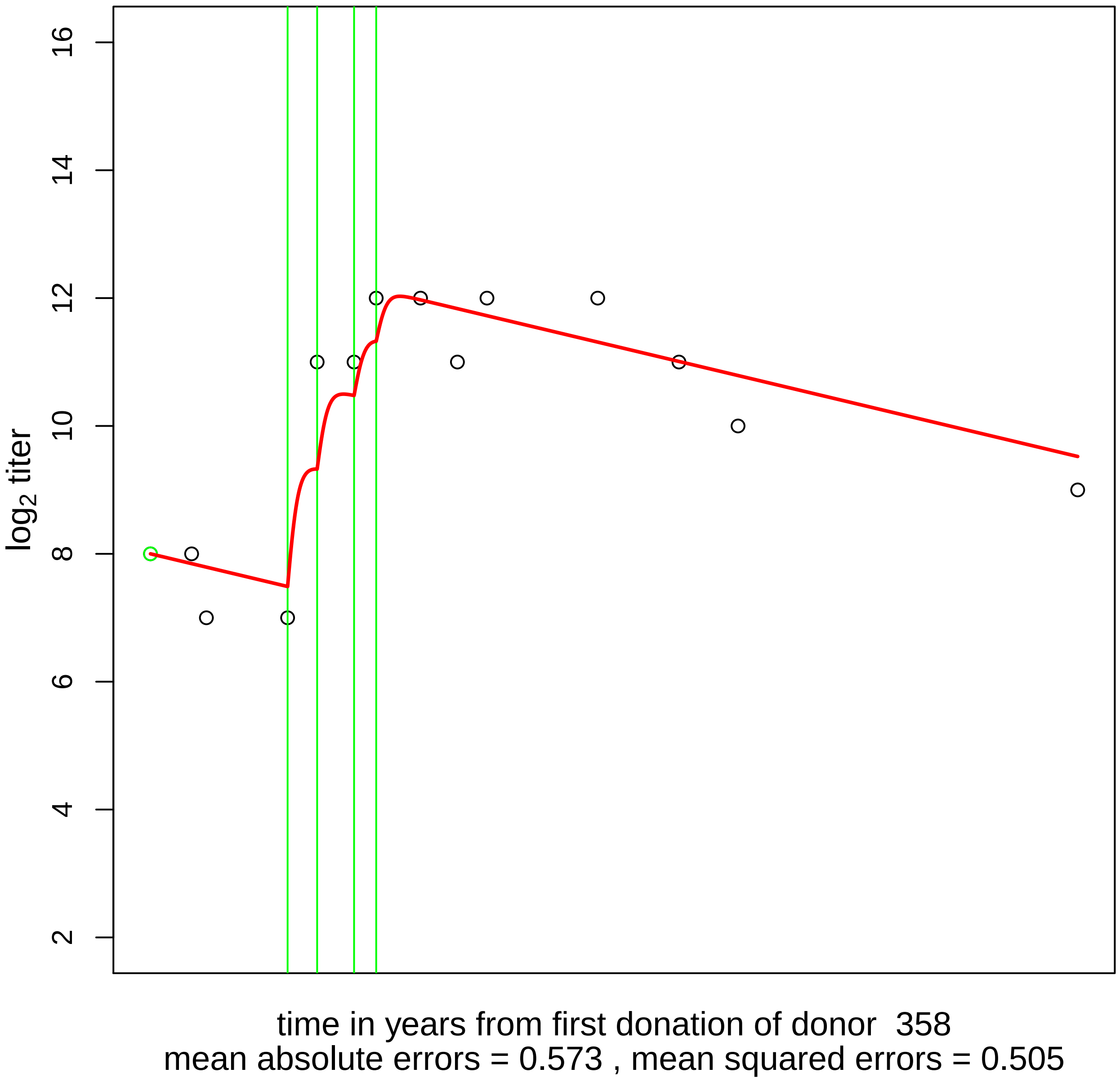

16
14
12
10
8
6
4
2
log2 titer
time in years from first donation of donor 358
mean absolute errors = 0.573 , mean squared errors = 0.505

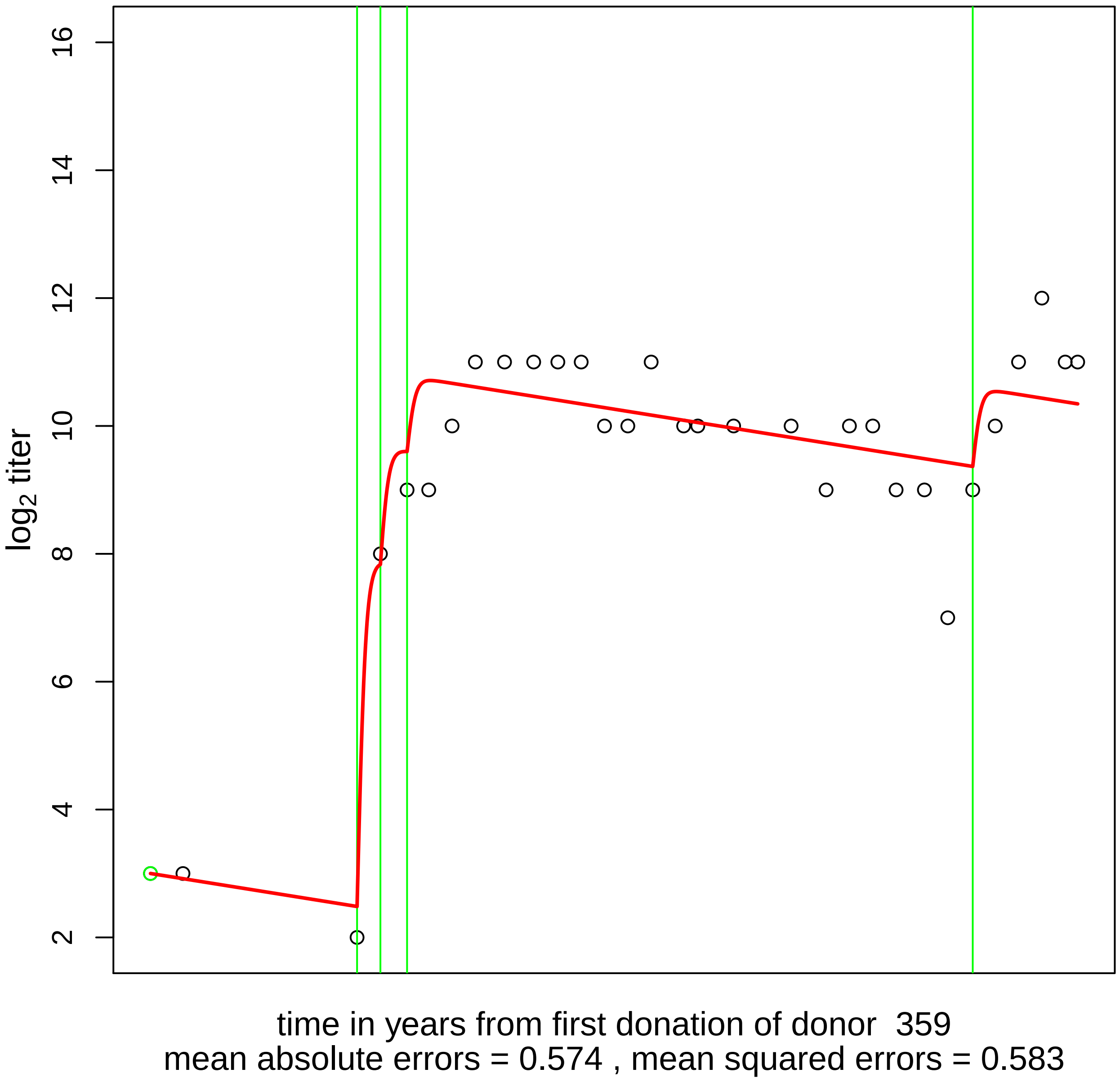

log2 titer
2
4
6
8
10
12
14
16
time in years from first donation of donor 359
mean absolute errors = 0.574 , mean squared errors = 0.583

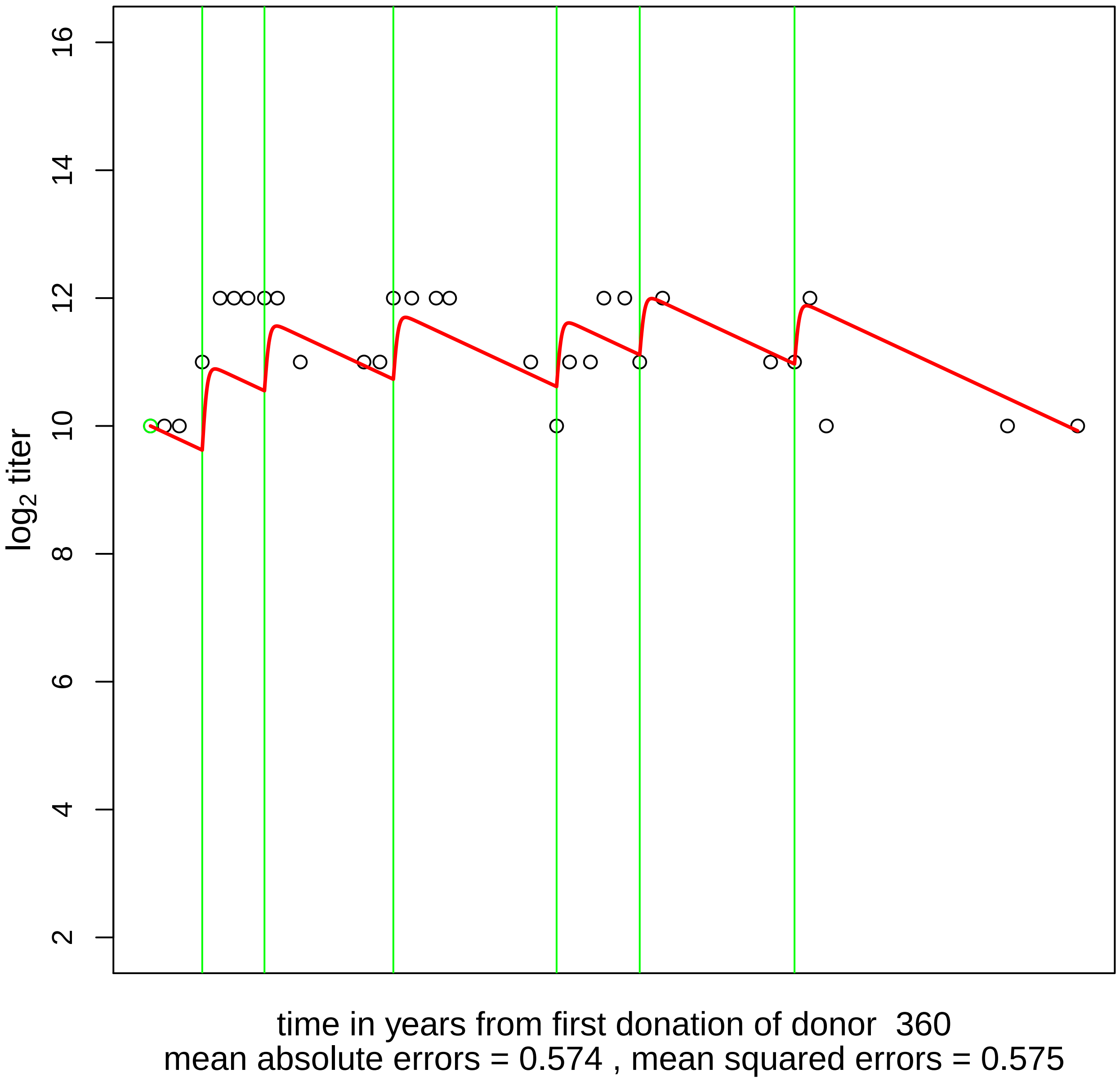

16
14
12
10
8
6
4
2
$\log_2$ titer
time in years from first donation of donor 360
mean absolute errors = 0.574 , mean squared errors = 0.575

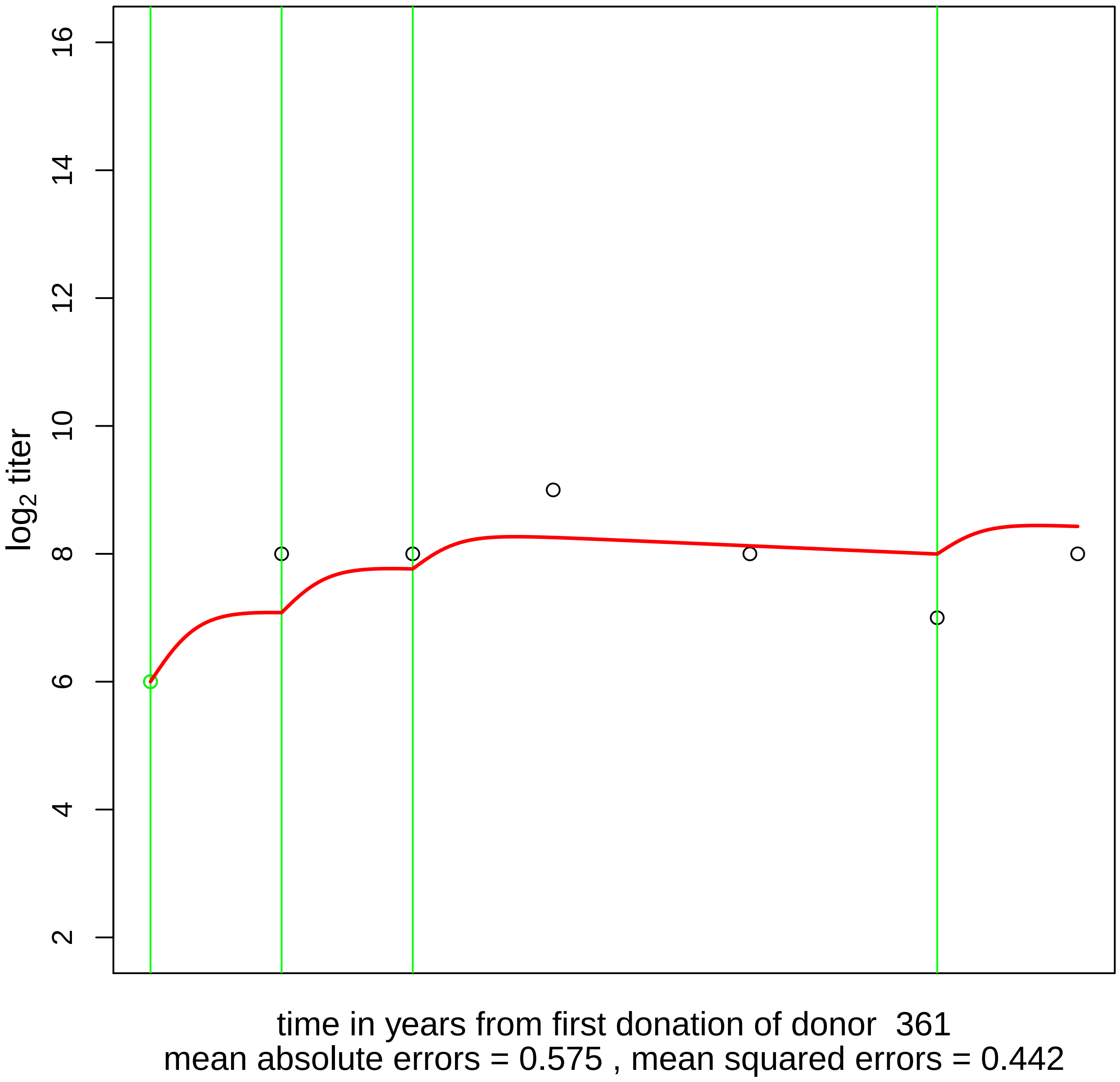

log2 titer
2
4
6
8
10
12
14
16
time in years from first donation of donor 361
mean absolute errors = 0.575 , mean squared errors = 0.442

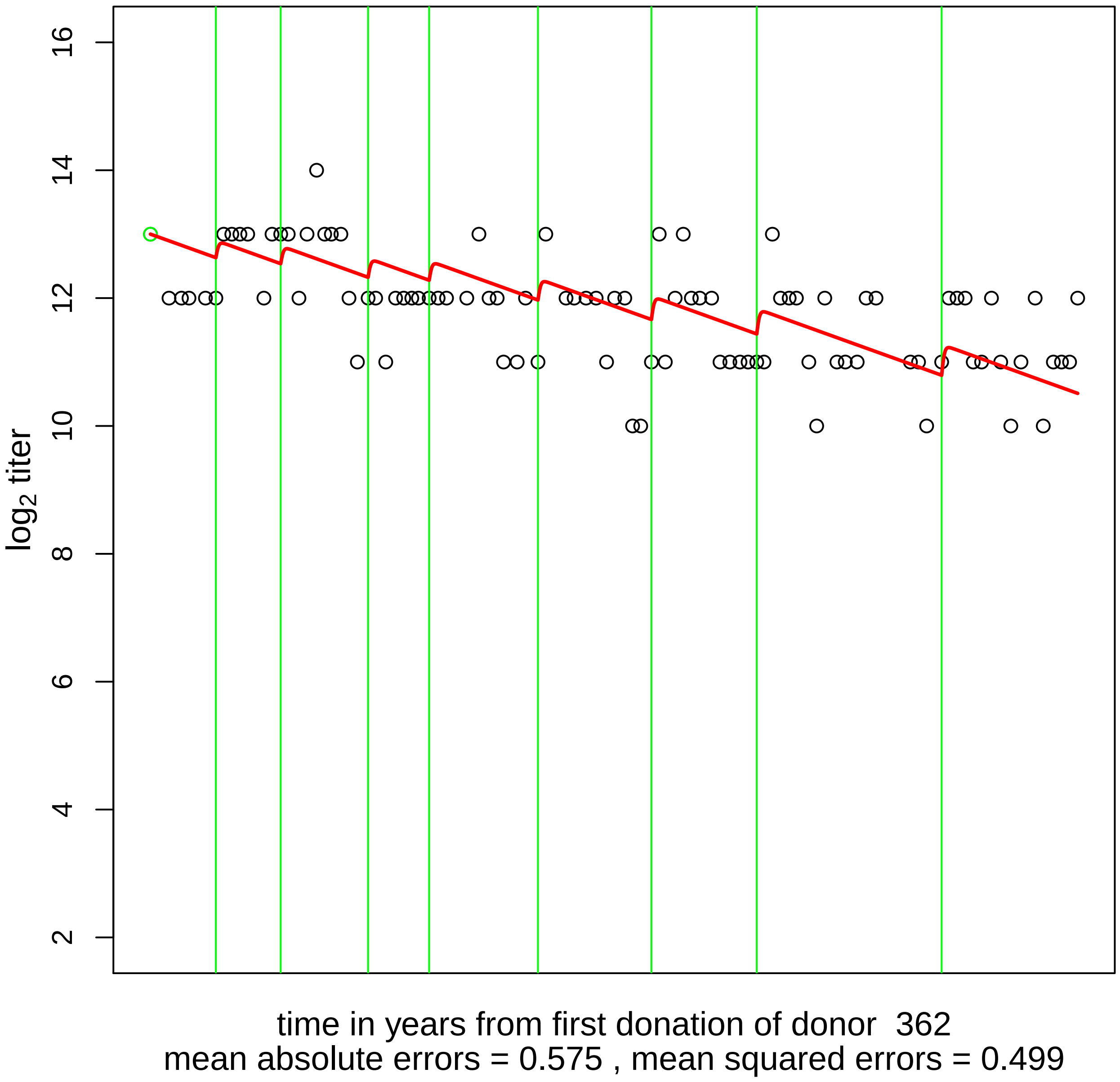

2
4
6
8
10
12
14
16
$\log_2$ titer
time in years from first donation of donor 362
mean absolute errors = 0.575 , mean squared errors = 0.499

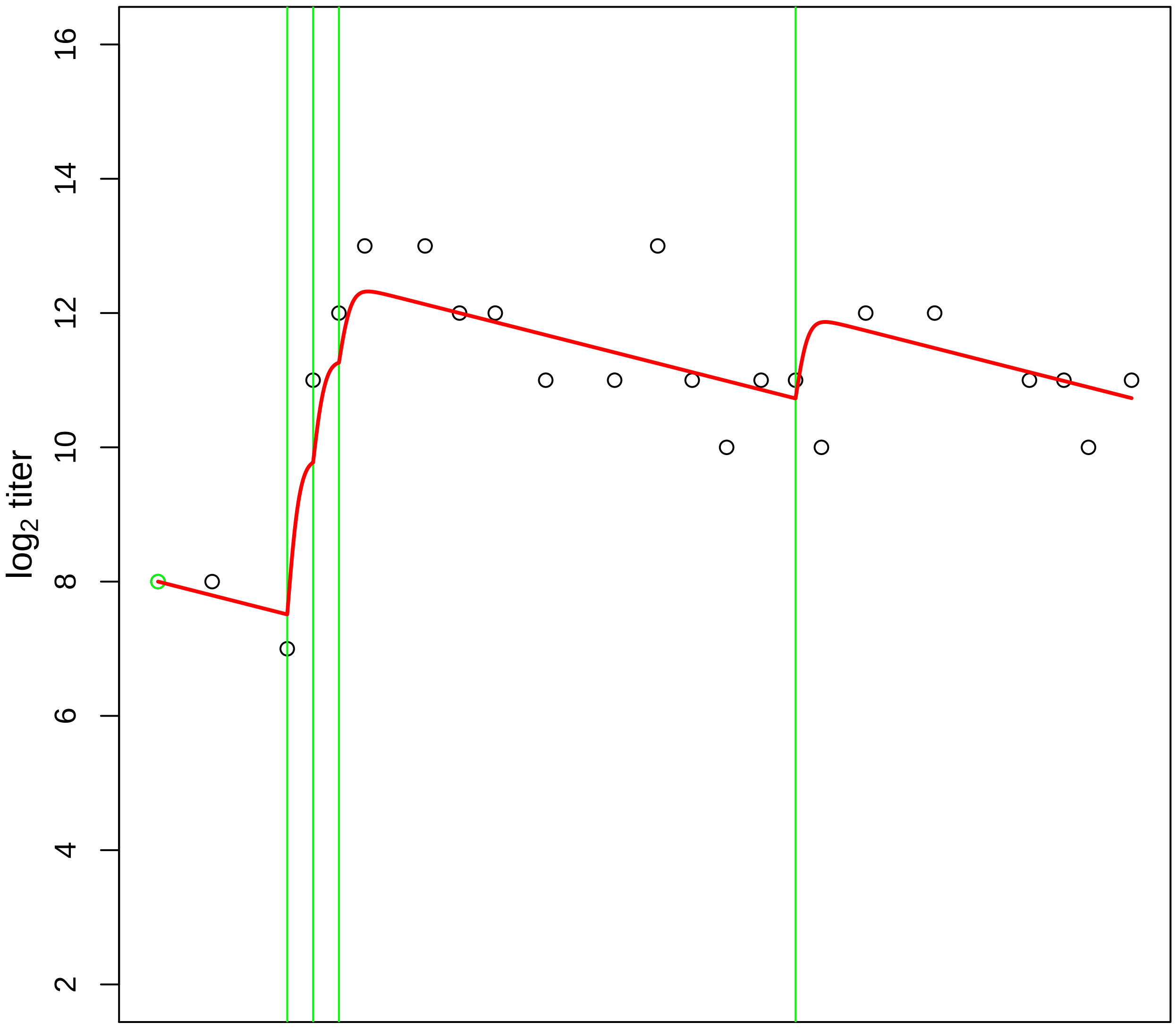

time in years from first donation of donor 363
mean absolute errors = 0.576 , mean squared errors = 0.594

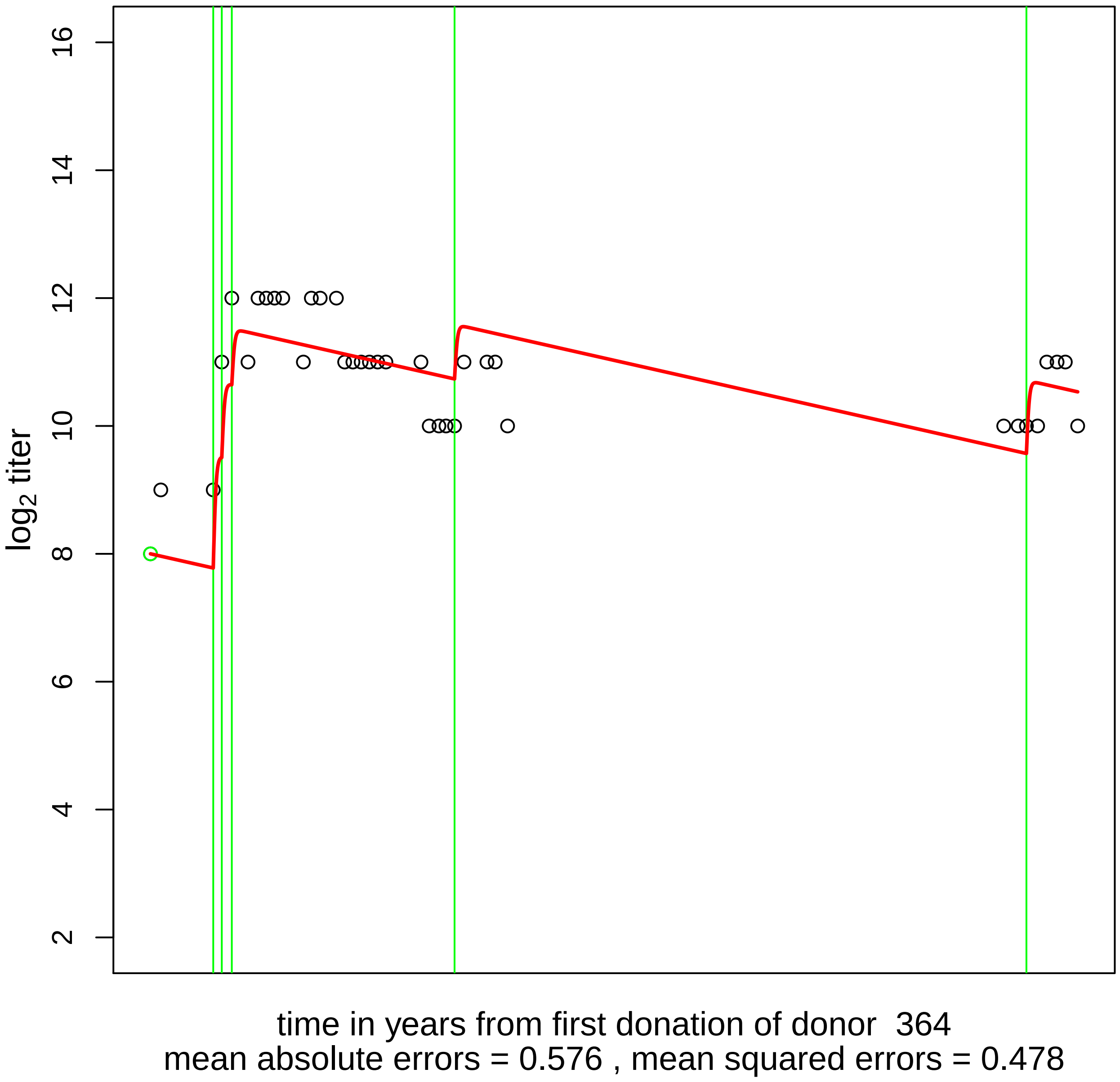
16
14
12
10
8
6
4
2
log2 titer
time in years from first donation of donor 364
mean absolute errors = 0.576 , mean squared errors = 0.478

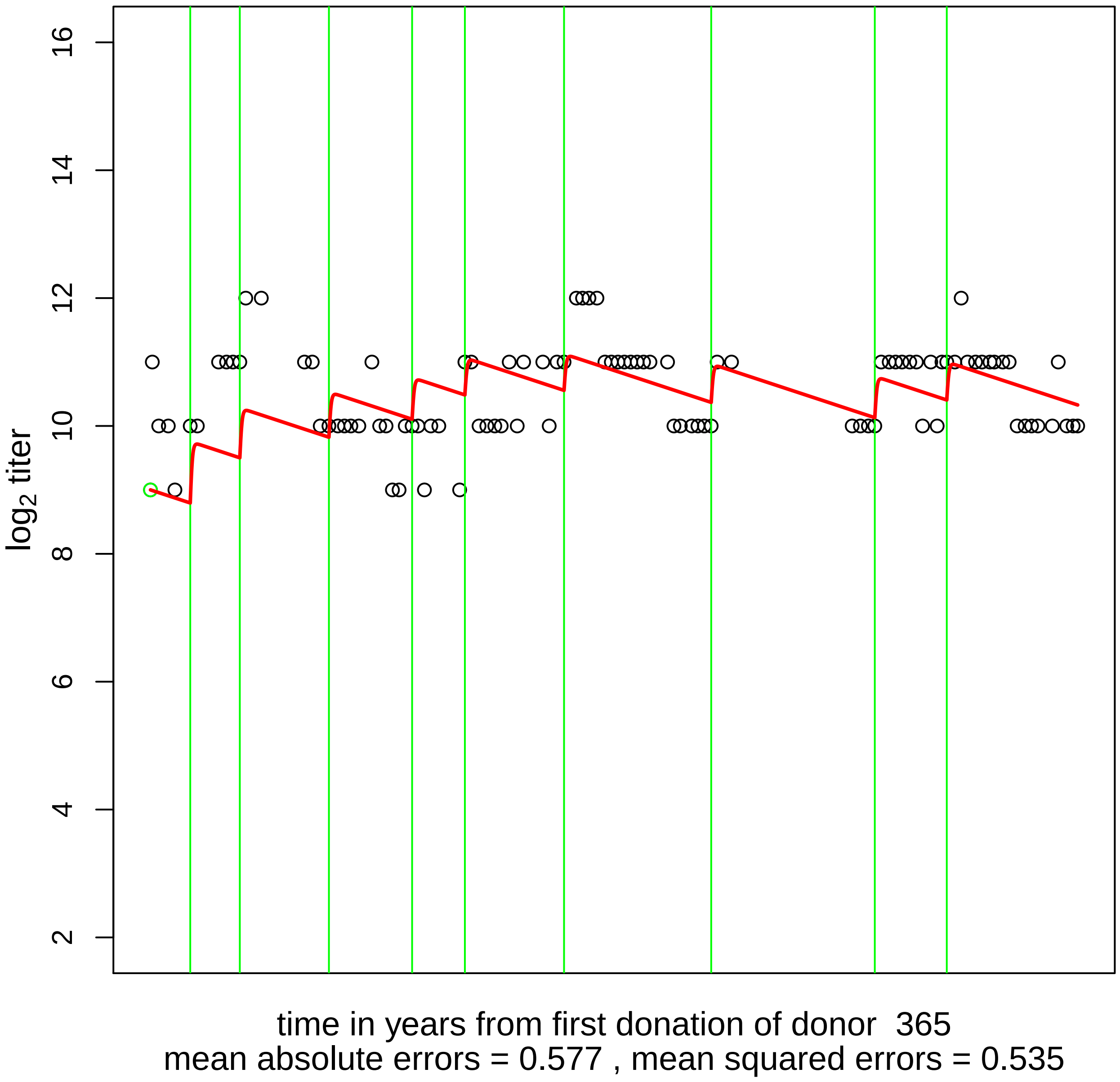
log2 titer
2
4
6
8
10
12
14
16
time in years from first donation of donor 365
mean absolute errors = 0.577 , mean squared errors = 0.535

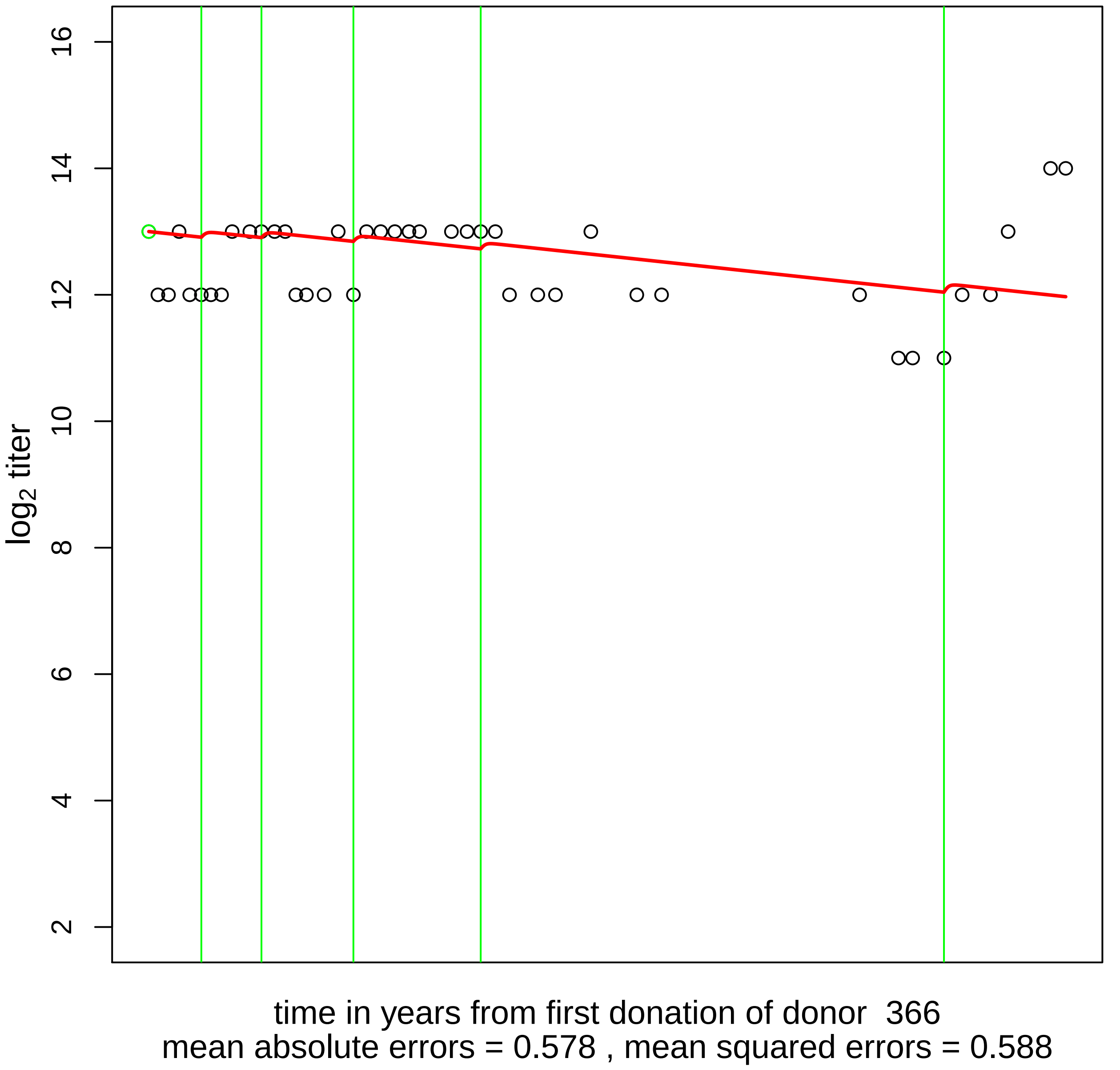

$\log_2$ titer
2
4
6
8
10
12
14
16
time in years from first donation of donor 366
mean absolute errors = 0.578 , mean squared errors = 0.588

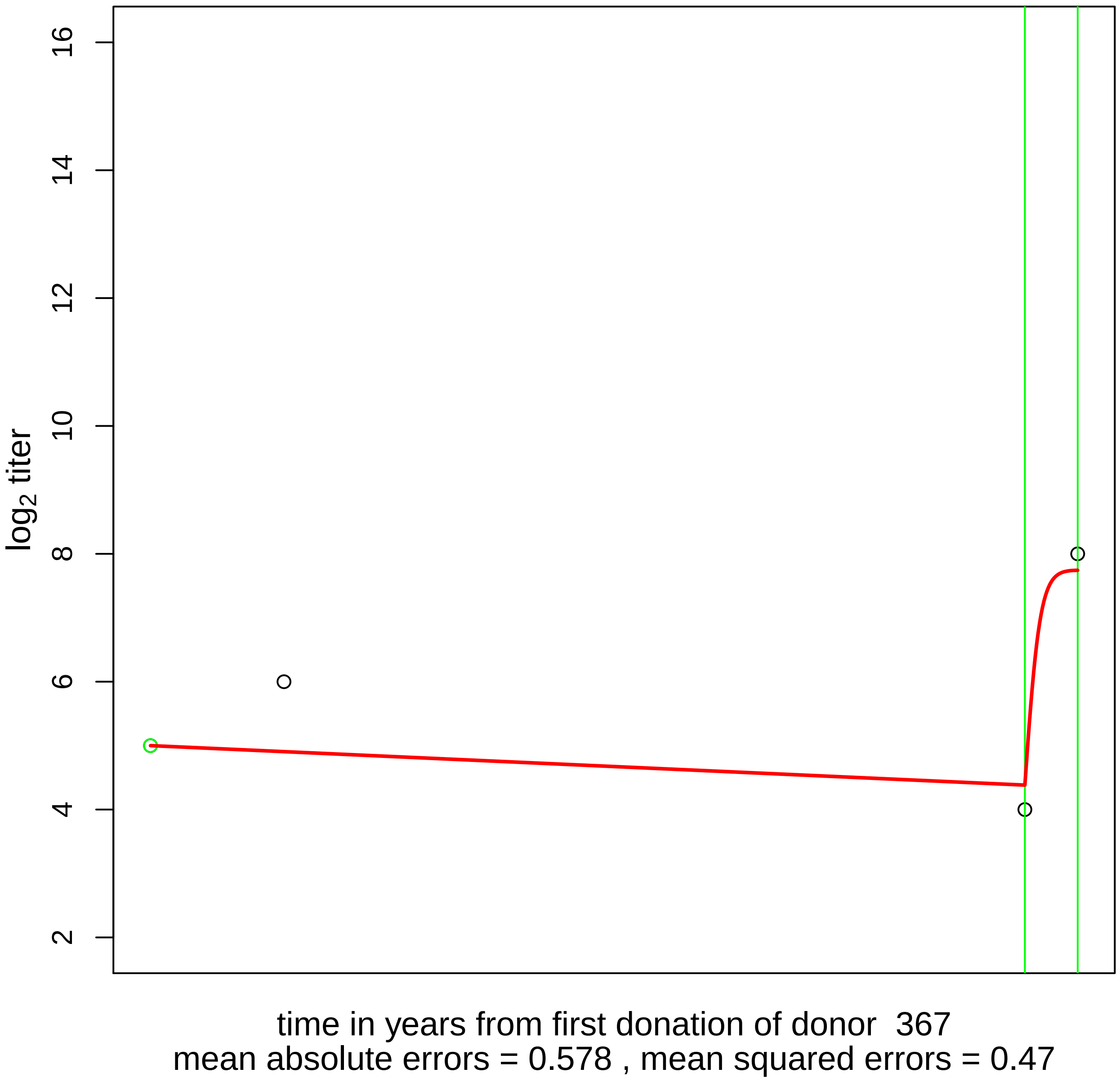
16
14
12
10
8
6
4
2
log2 titer
time in years from first donation of donor 367
mean absolute errors = 0.578 , mean squared errors = 0.47

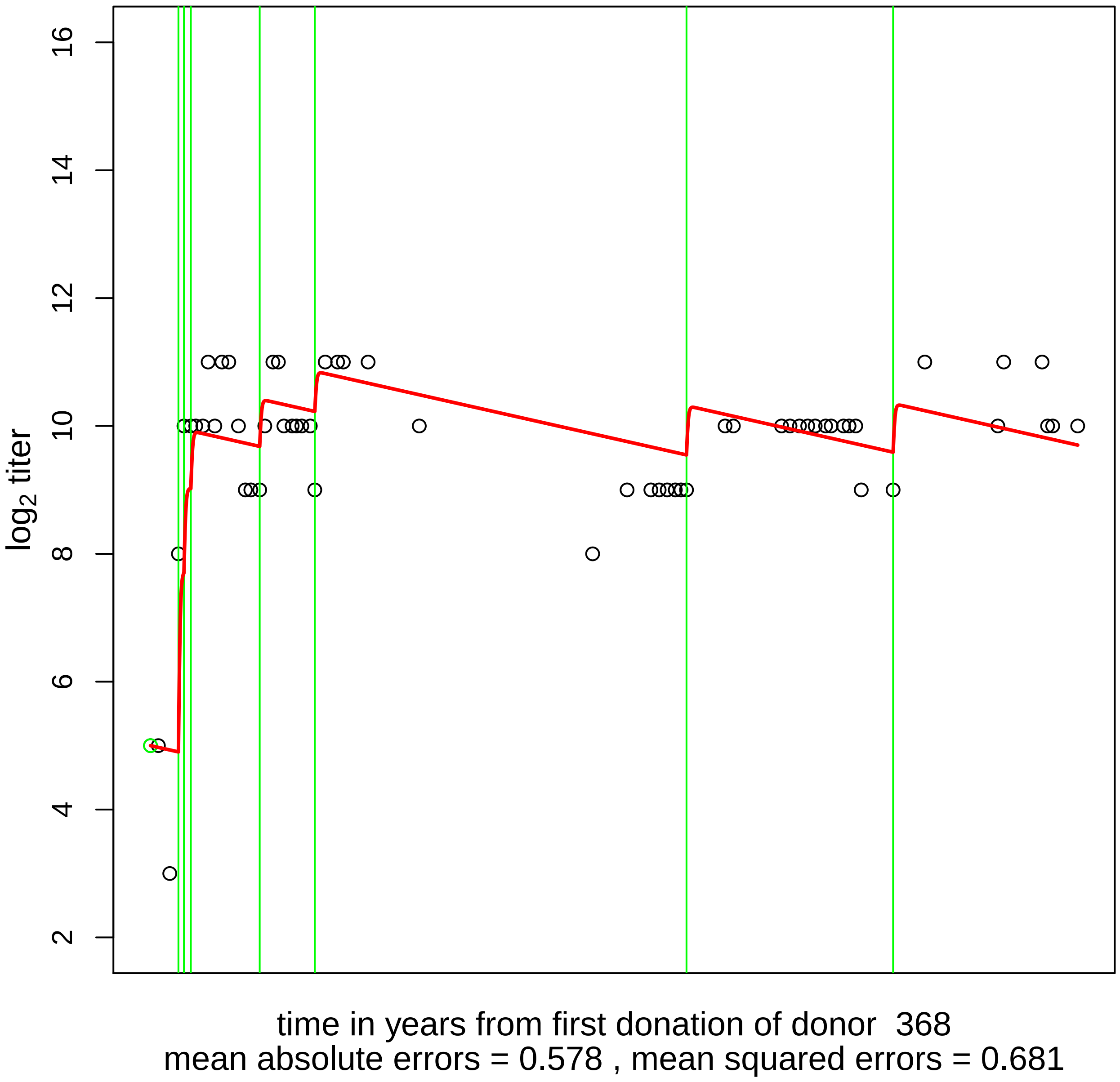

2
4
6
8
10
12
14
16
$\log_2$ titer
time in years from first donation of donor 368
mean absolute errors = 0.578 , mean squared errors = 0.681

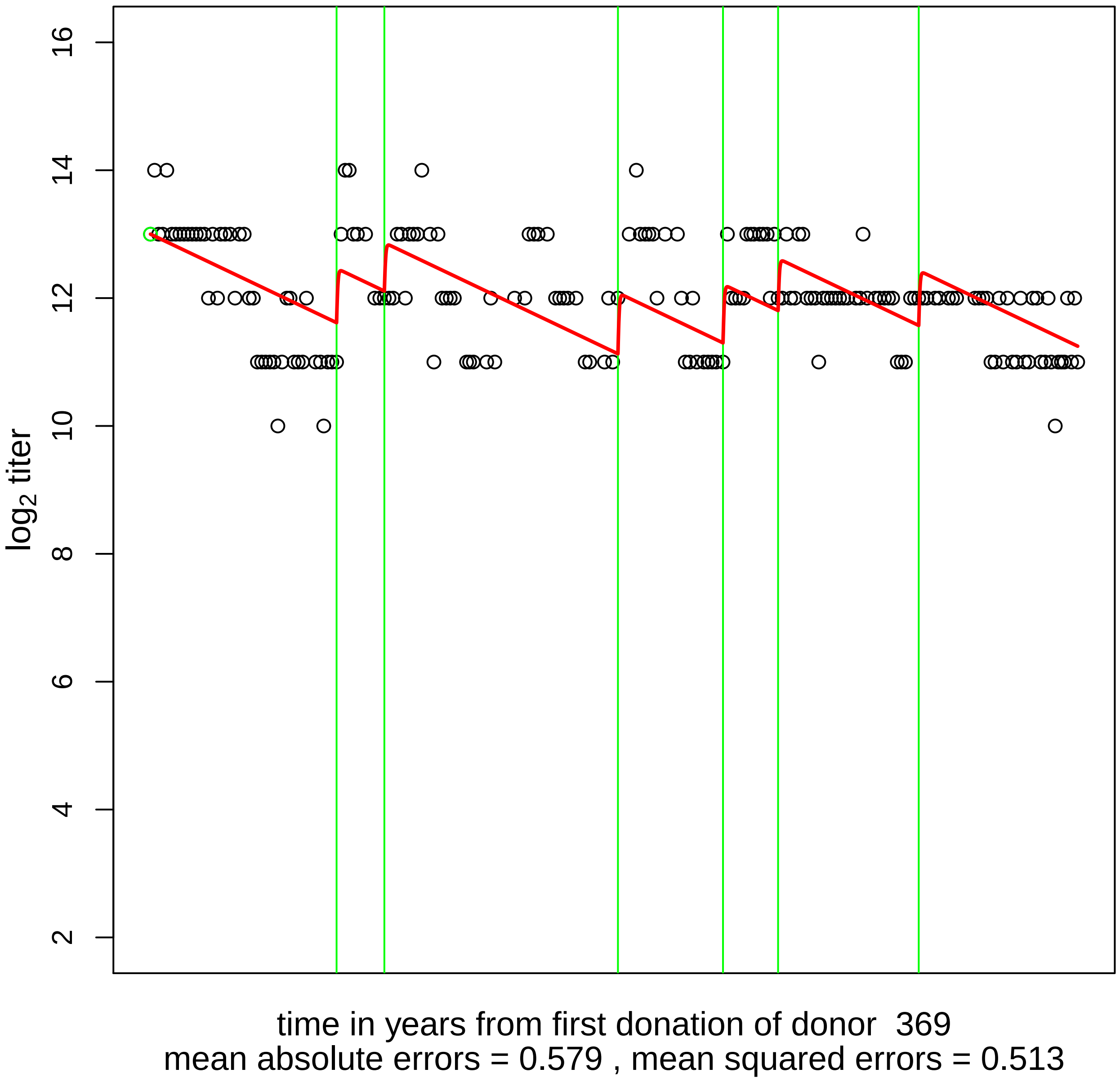

16
14
12
10
8
6
4
2
$\log_2$ titer
time in years from first donation of donor 369
mean absolute errors = 0.579 , mean squared errors = 0.513

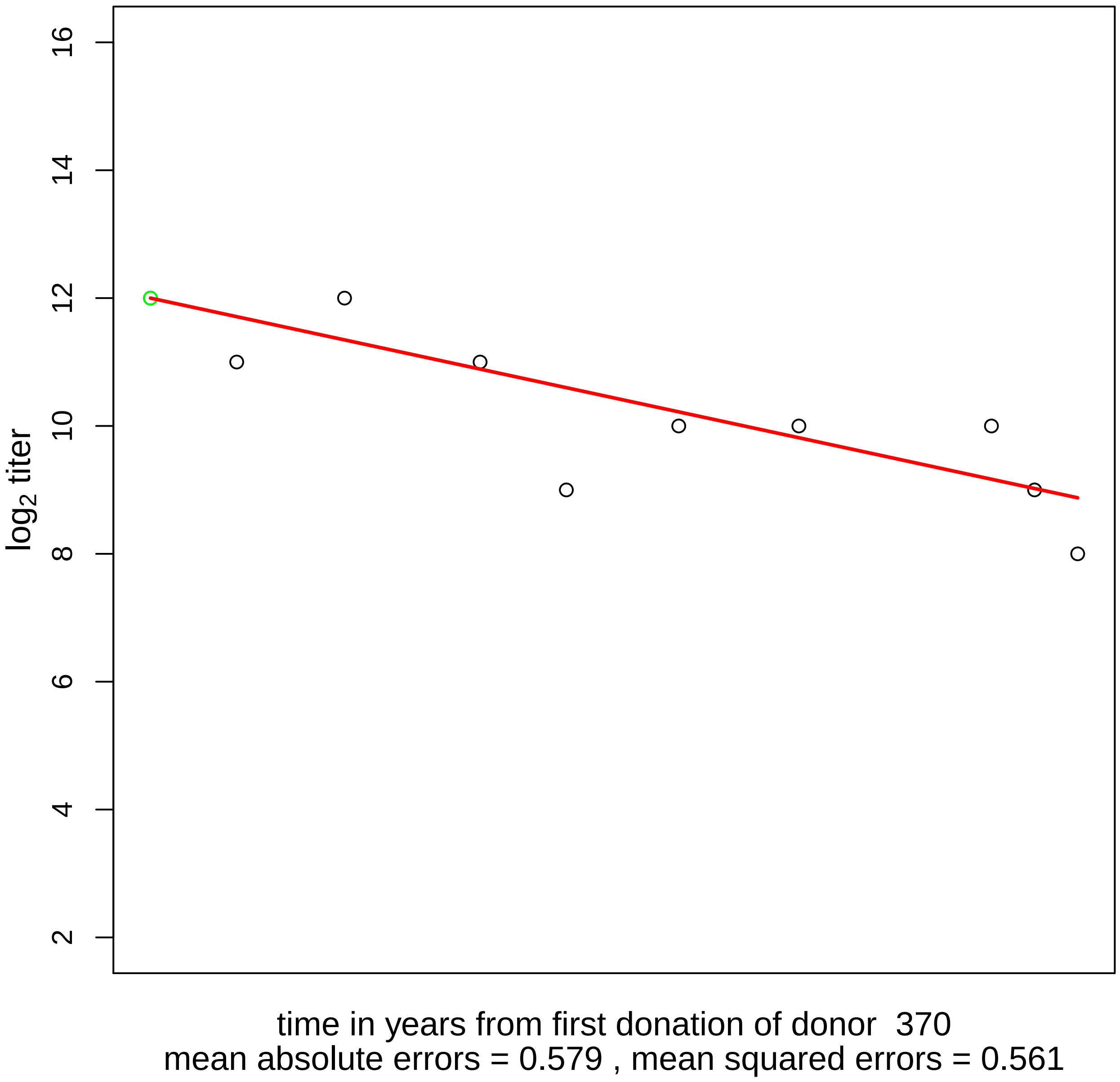

16
14
12
10
8
6
4
2
$\log_2$ titer
time in years from first donation of donor 370
mean absolute errors = 0.579 , mean squared errors = 0.561

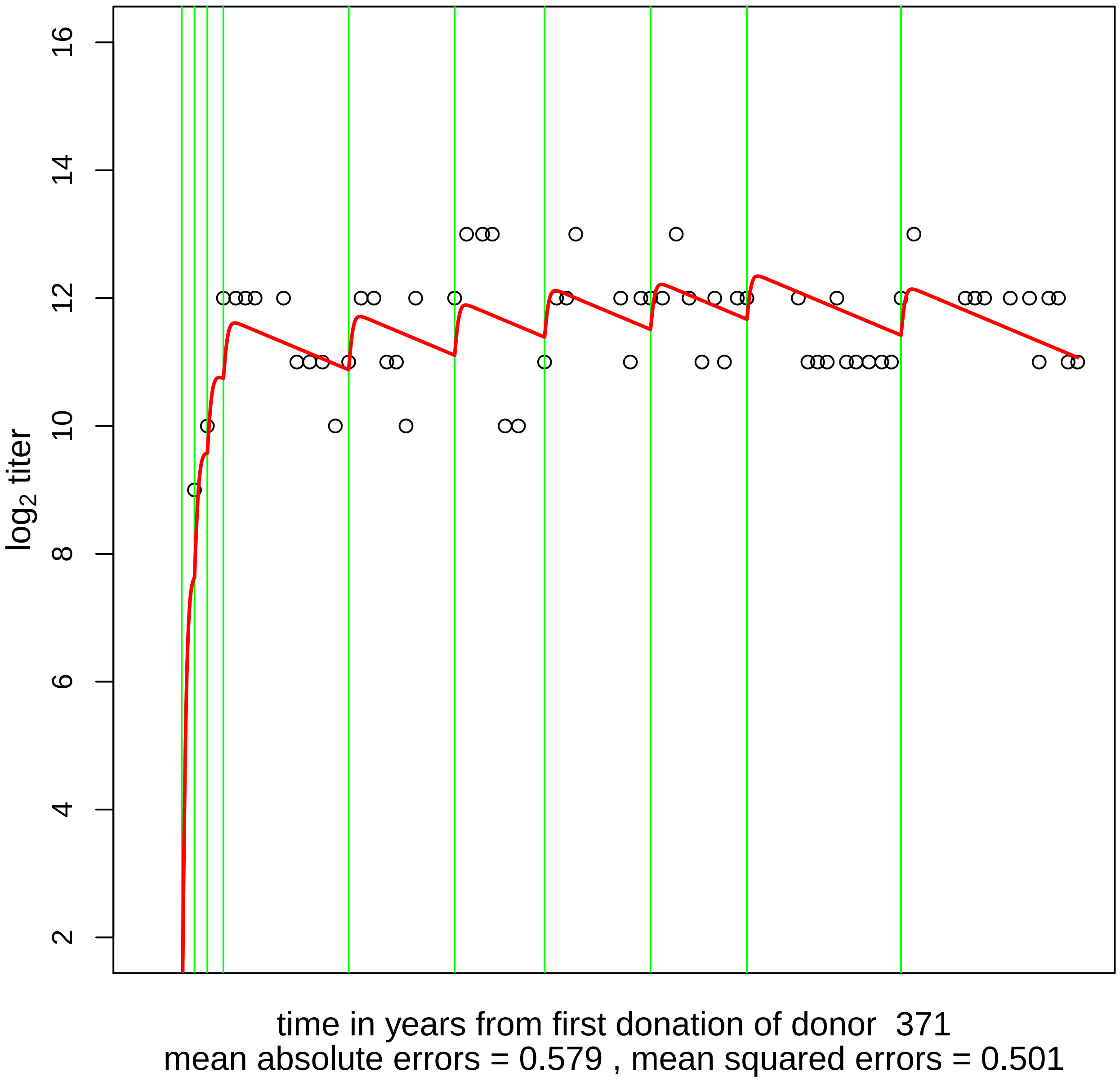

log2 titer
2
4
6
8
10
12
14
16
time in years from first donation of donor 371
mean absolute errors = 0.579 , mean squared errors = 0.501

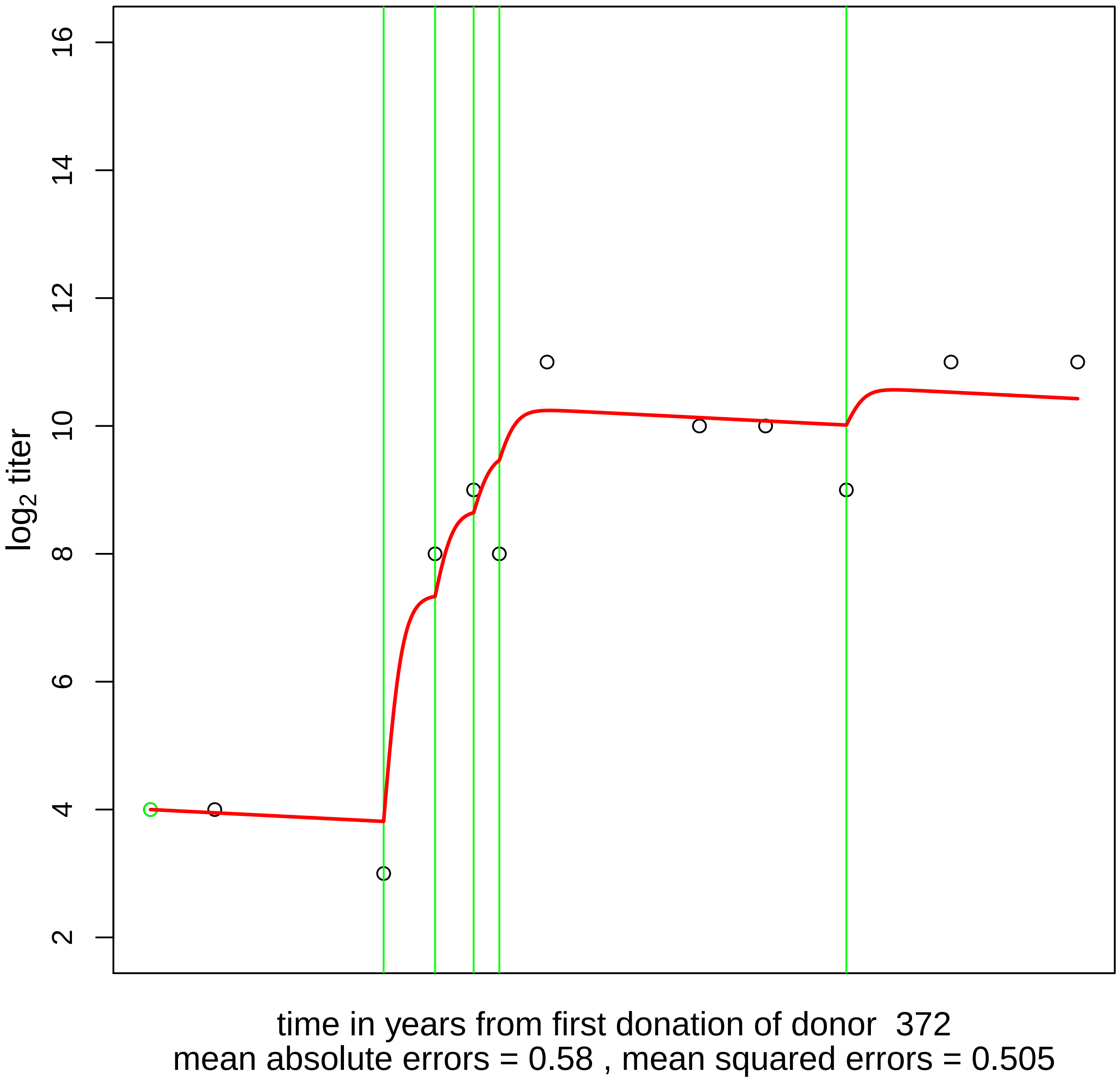
log₂ titer
16
14
12
10
8
6
4
2
time in years from first donation of donor 372
mean absolute errors = 0.58 , mean squared errors = 0.505

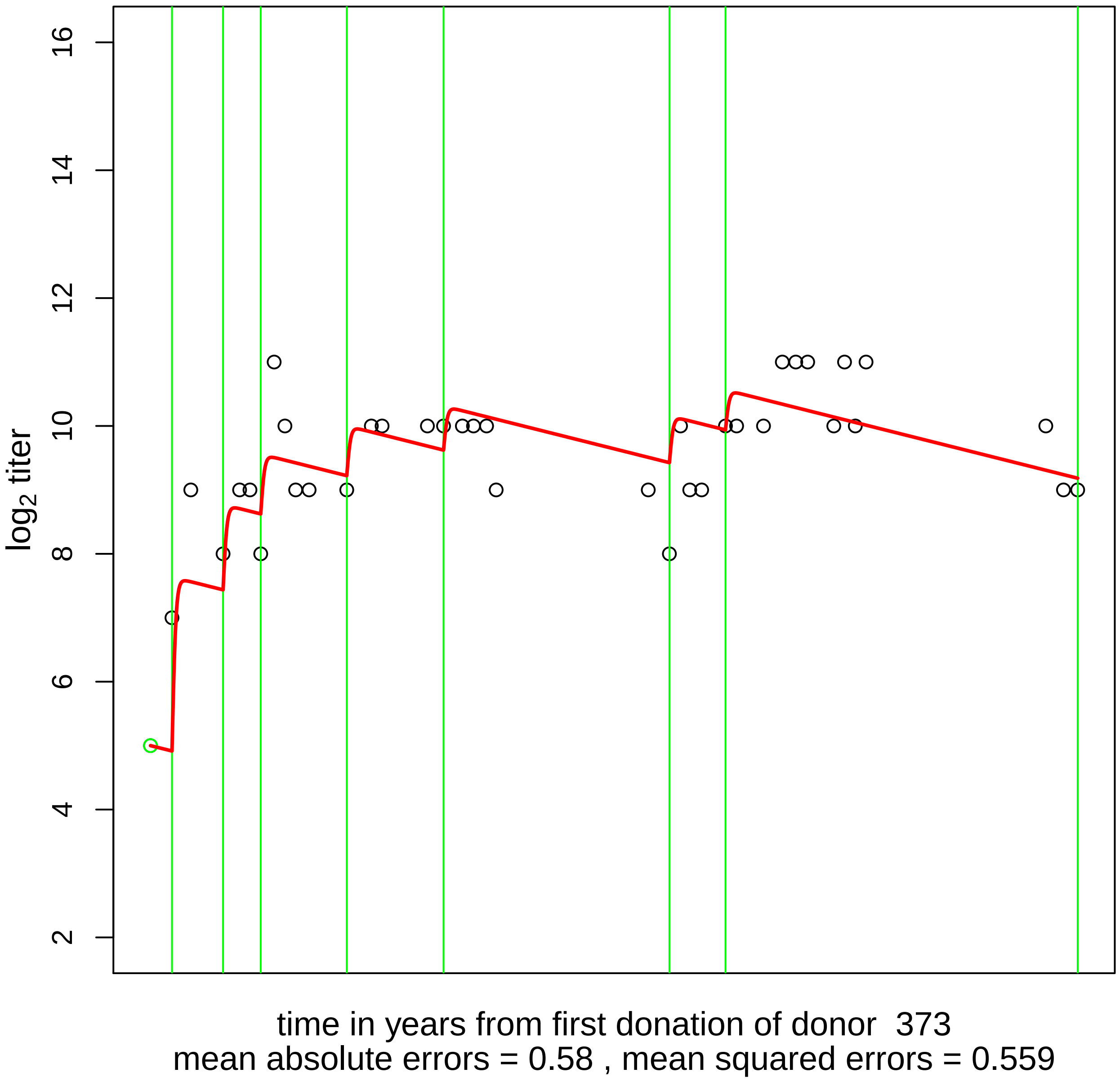

16
14
12
10
8
6
4
2
$\log_2$ titer
time in years from first donation of donor 373
mean absolute errors = 0.58 , mean squared errors = 0.559

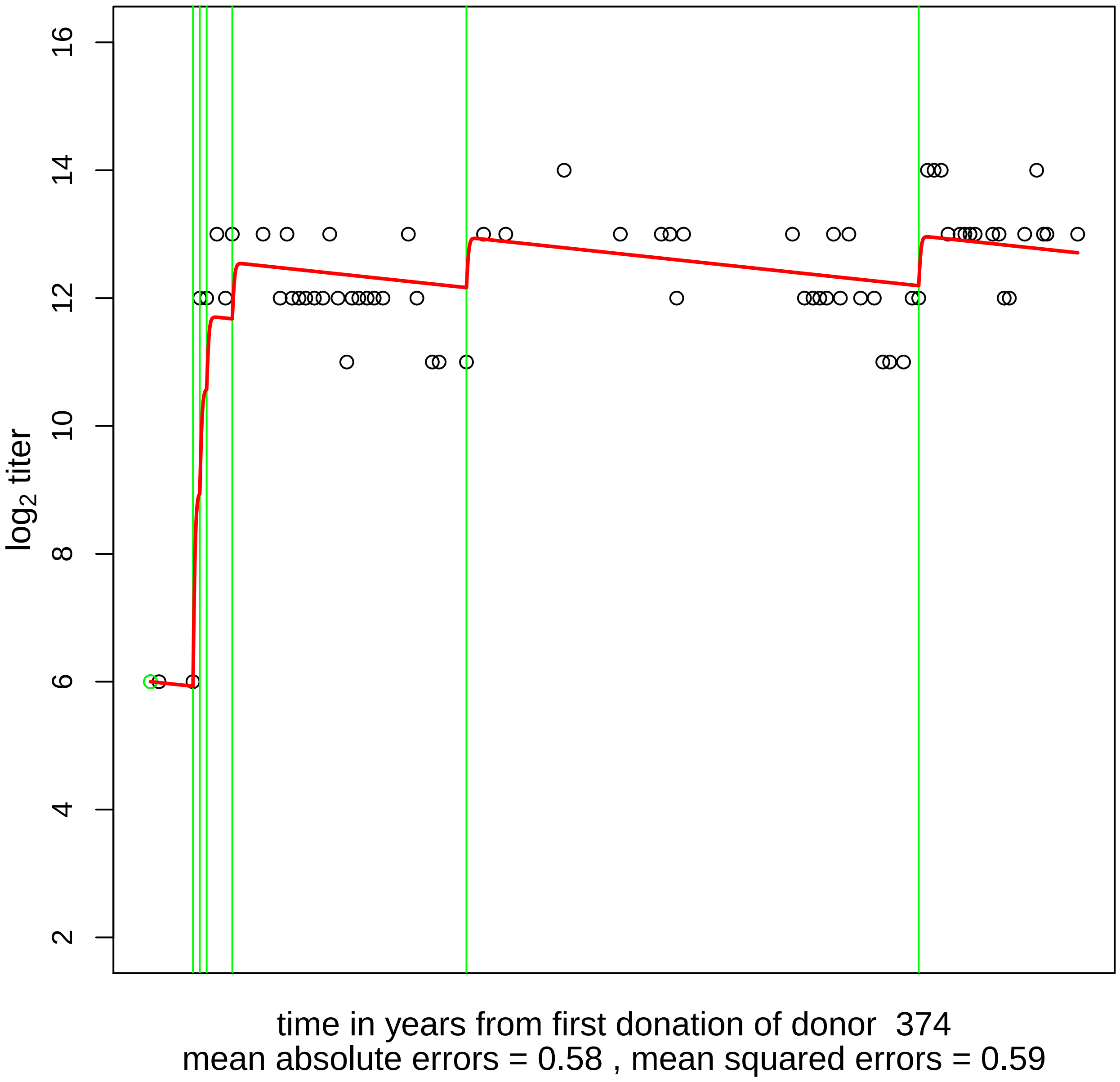
16
14
12
10
8
6
4
2
$\log_2$ titer
time in years from first donation of donor 374
mean absolute errors = 0.58 , mean squared errors = 0.59

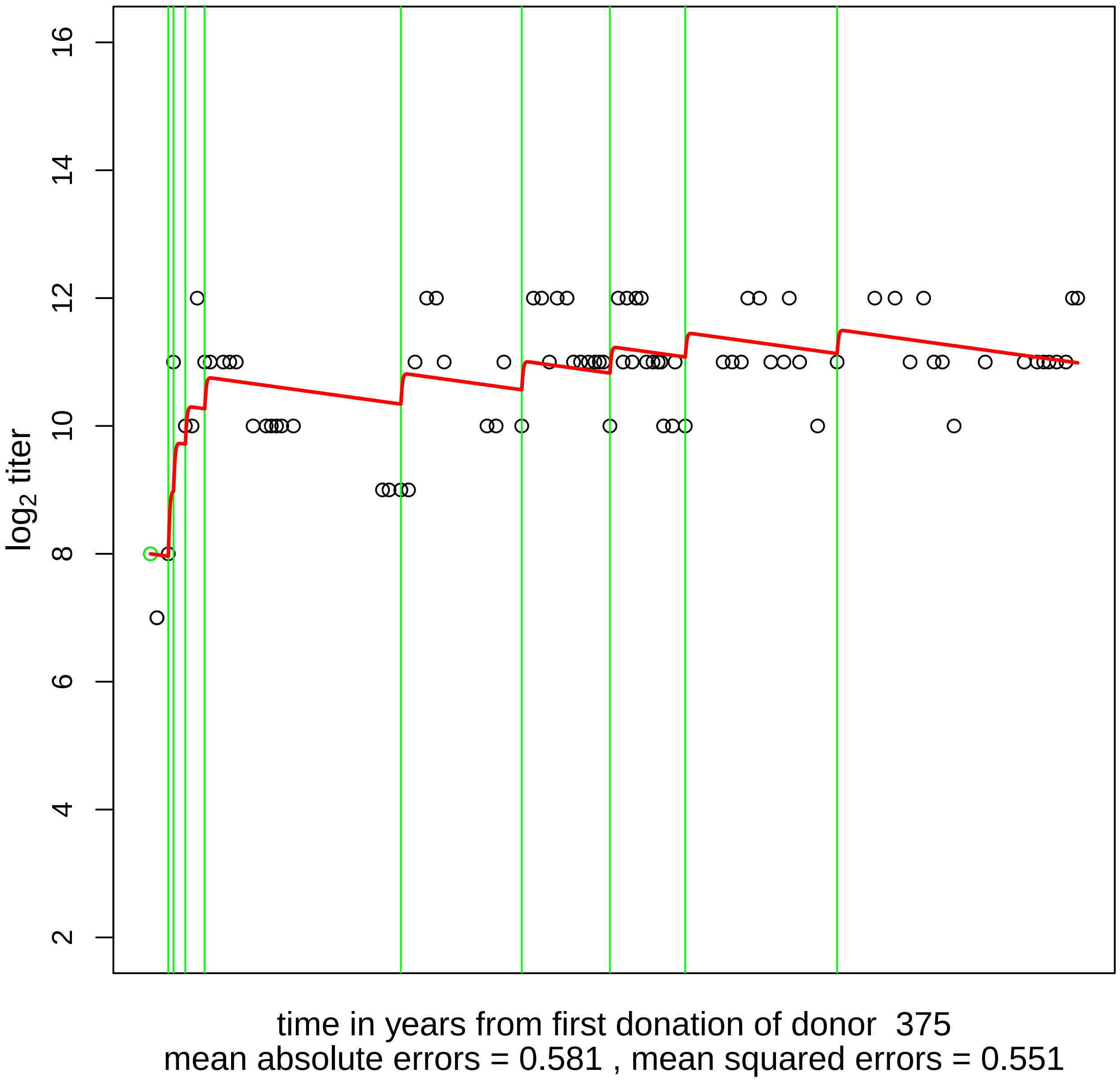

log2 titer
16
14
12
10
8
6
4
2
time in years from first donation of donor 375
mean absolute errors = 0.581 , mean squared errors = 0.551

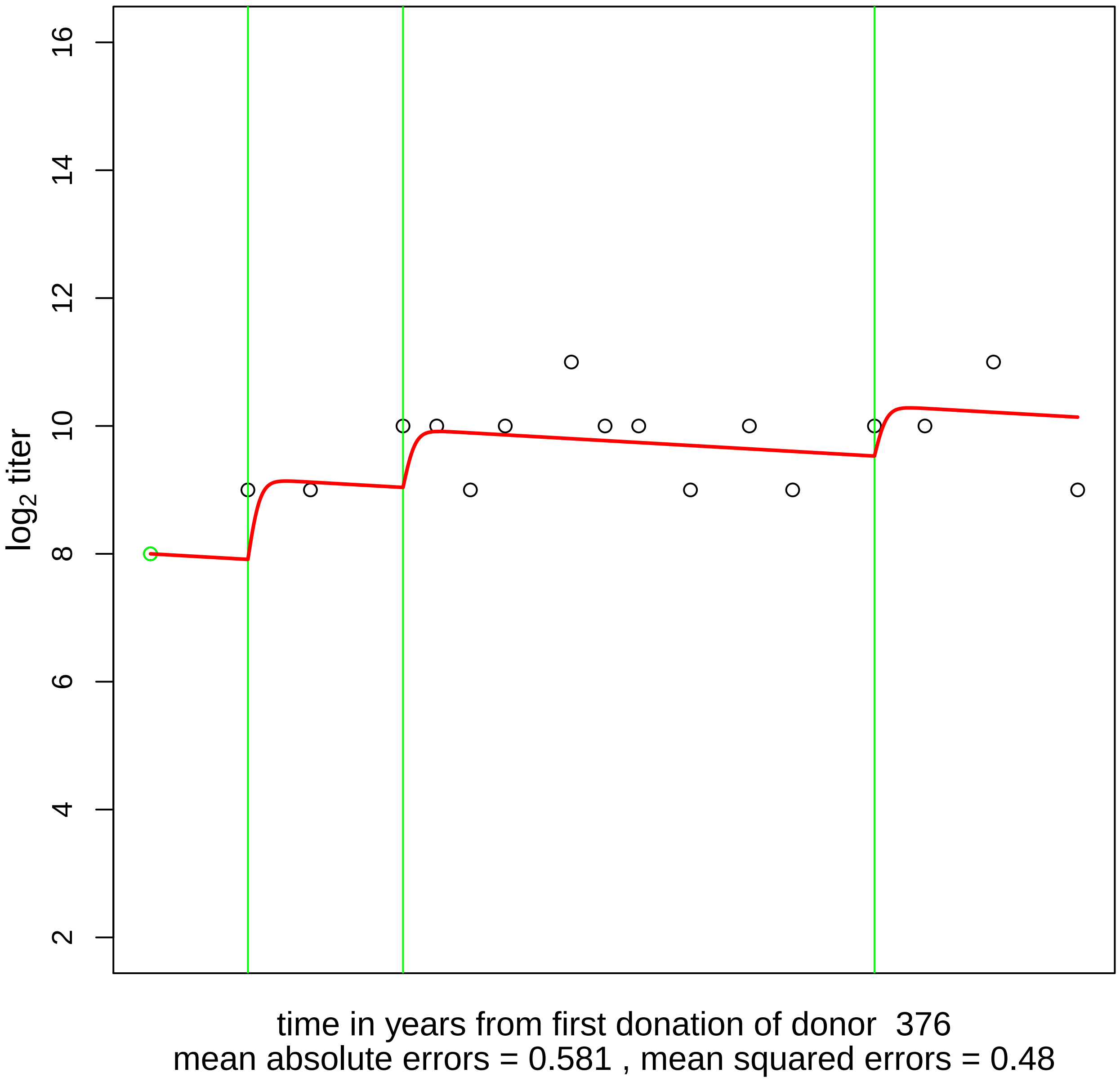

log2 titer
2
4
6
8
10
12
14
16
time in years from first donation of donor 376
mean absolute errors = 0.581 , mean squared errors = 0.48

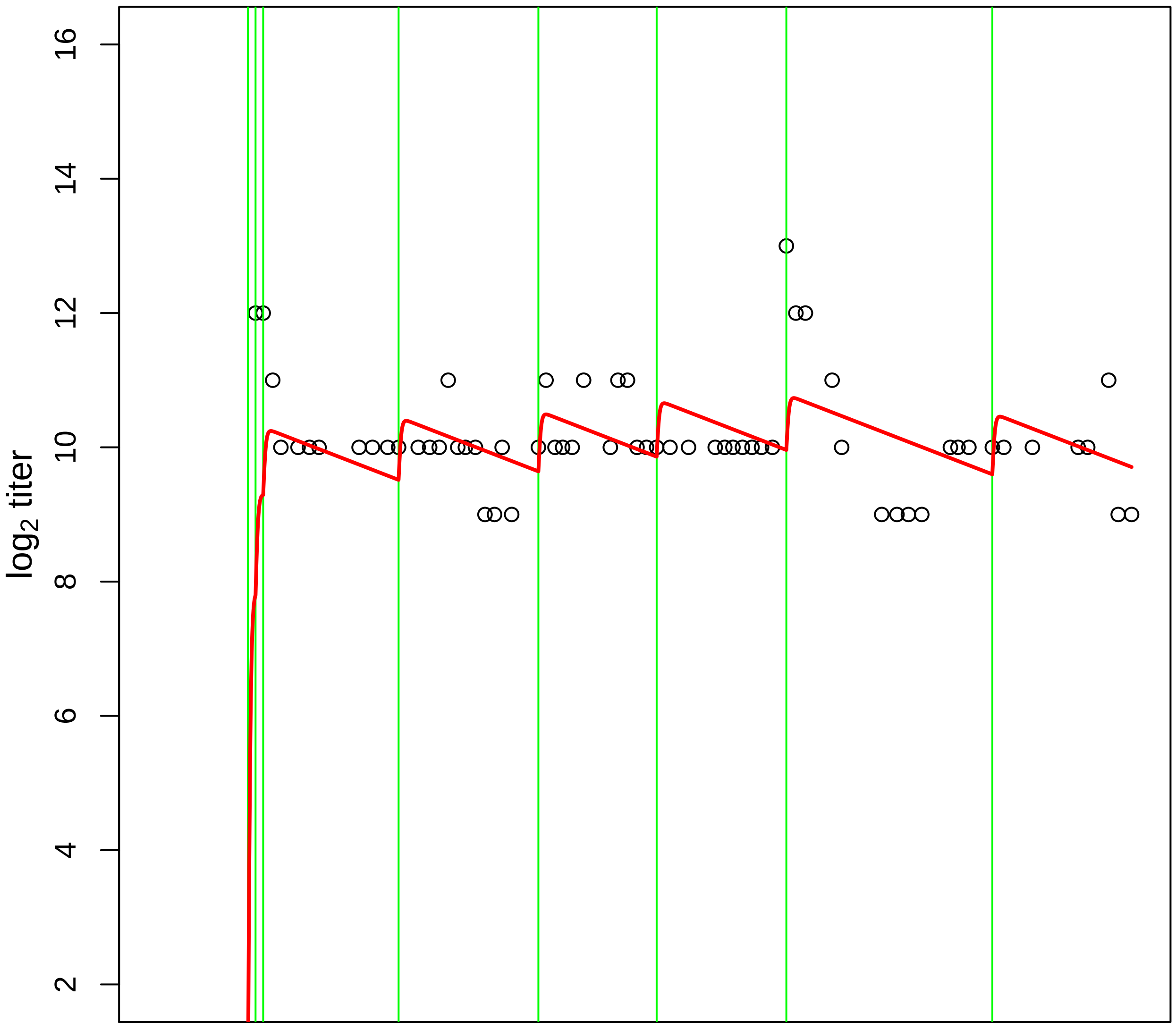

time in years from first donation of donor 377
mean absolute errors = 0.582 , mean squared errors = 0.849

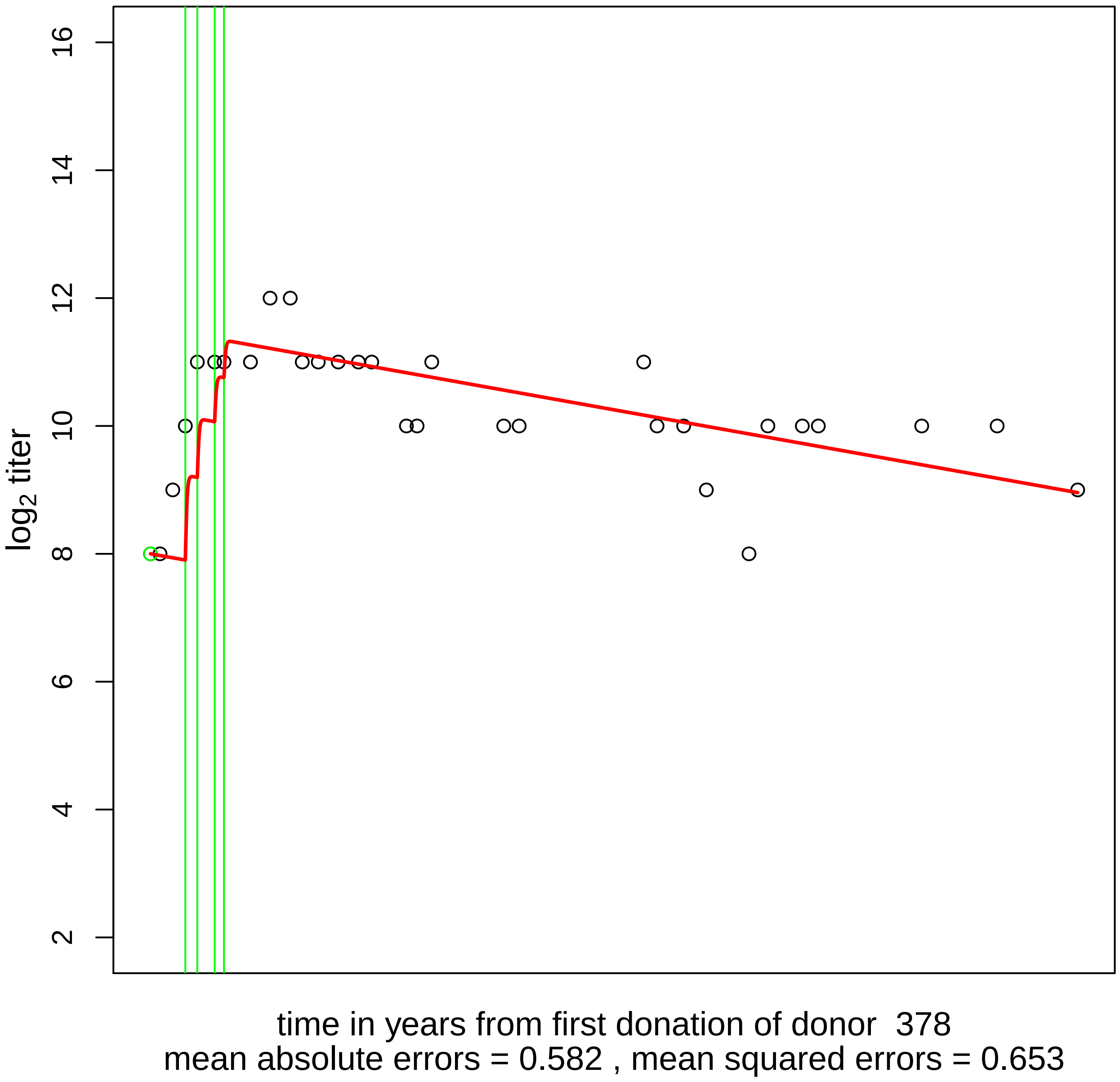

16
14
12
10
8
6
4
2
$\log_2$ titer
time in years from first donation of donor 378
mean absolute errors = 0.582 , mean squared errors = 0.653

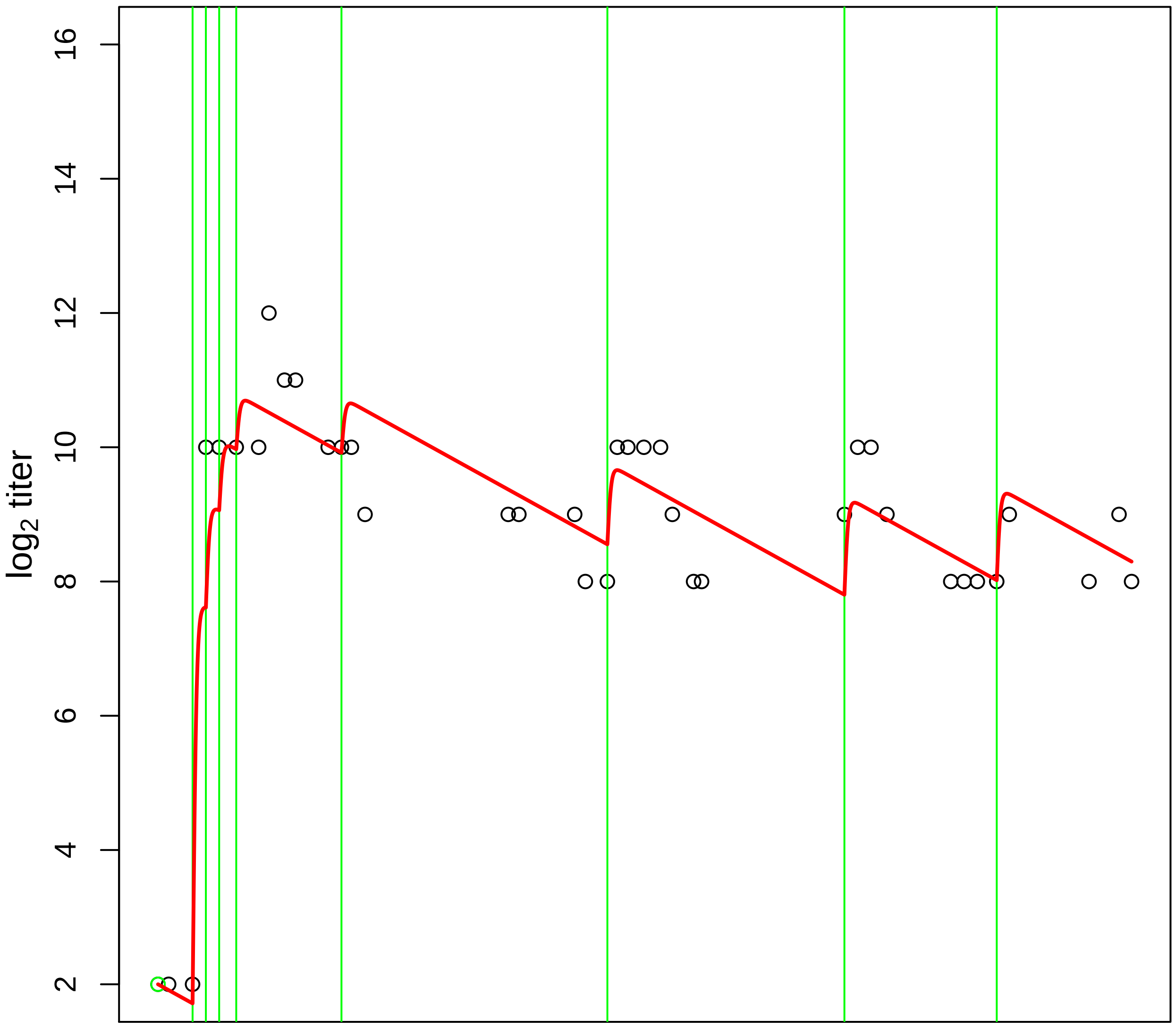

time in years from first donation of donor 379
mean absolute errors = 0.583 , mean squared errors = 0.58

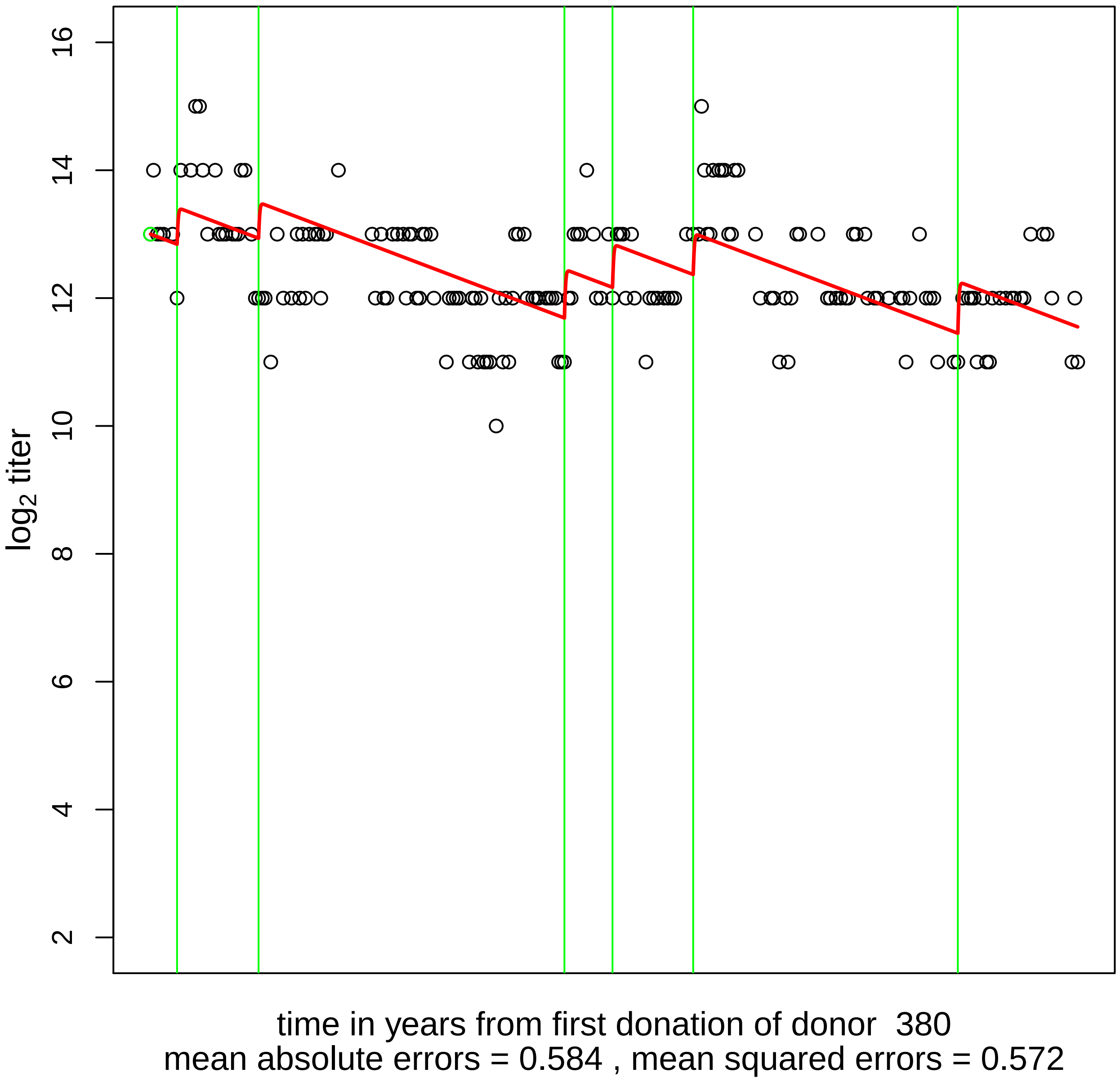
16
14
12
10
8
6
4
2
$\log_2$ titer
time in years from first donation of donor 380
mean absolute errors = 0.584 , mean squared errors = 0.572

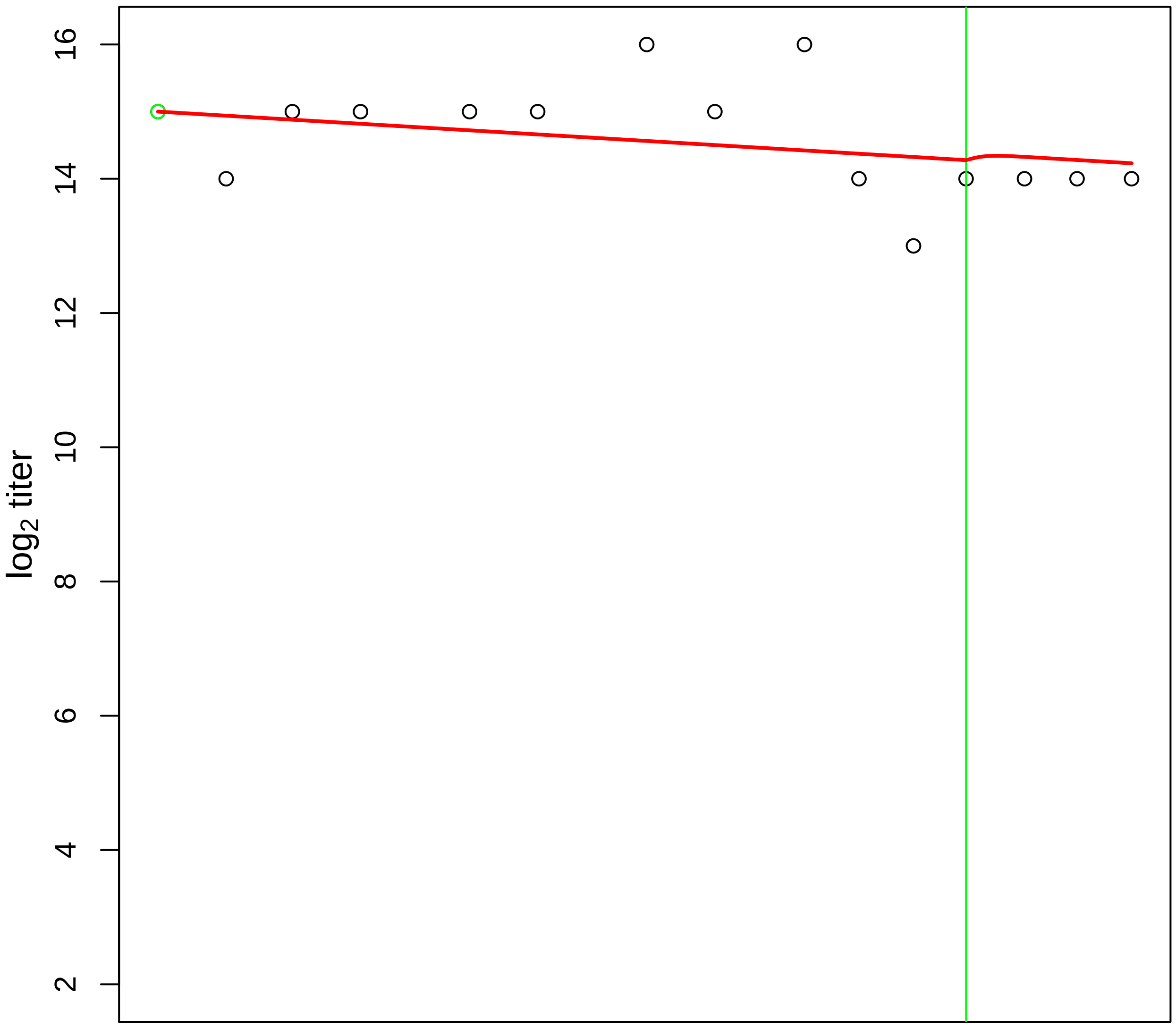

time in years from first donation of donor 381
mean absolute errors = 0.584 , mean squared errors = 0.581

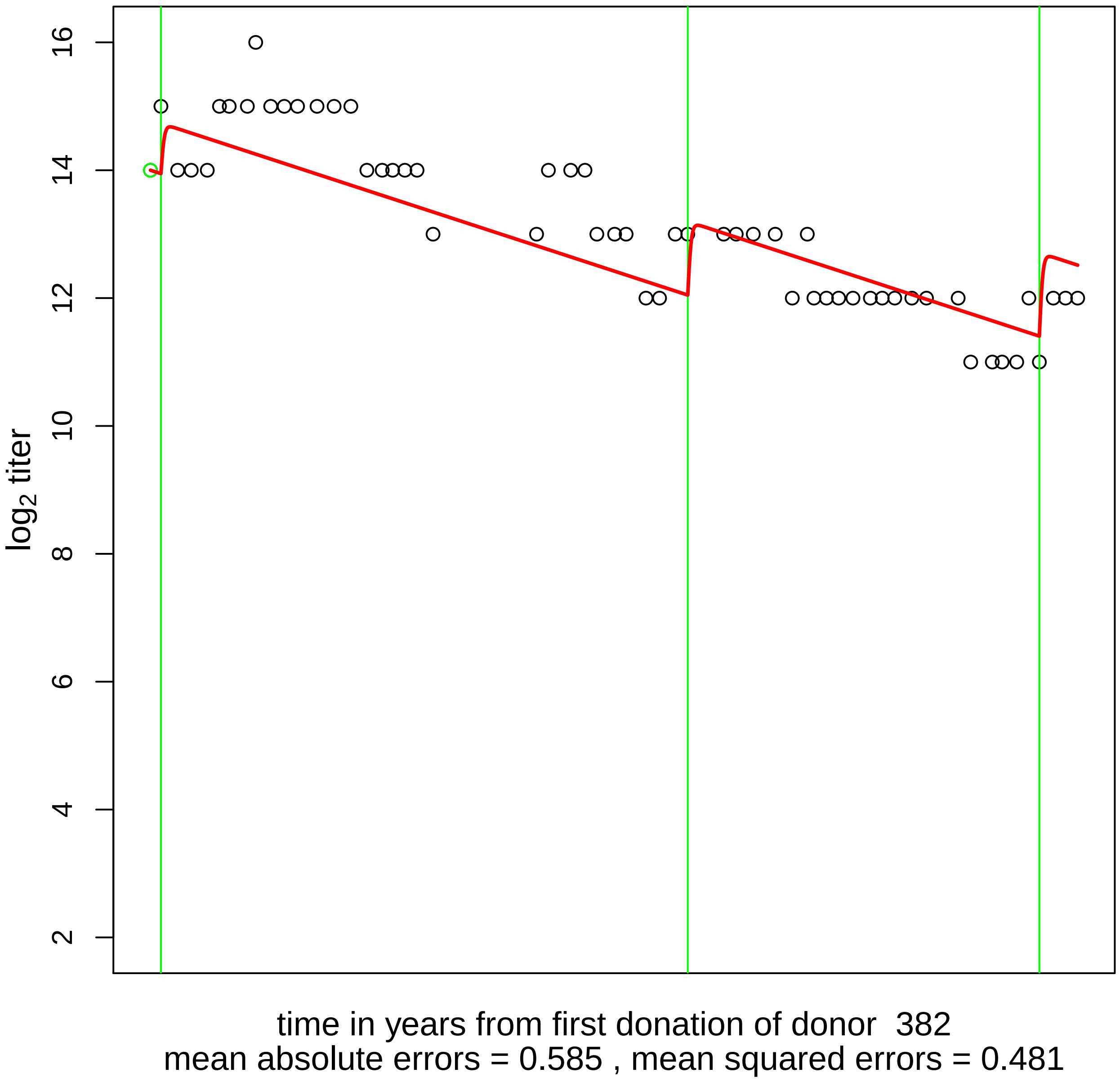
16
14
12
10
8
6
4
2
log2 titer
time in years from first donation of donor 382
mean absolute errors = 0.585 , mean squared errors = 0.481

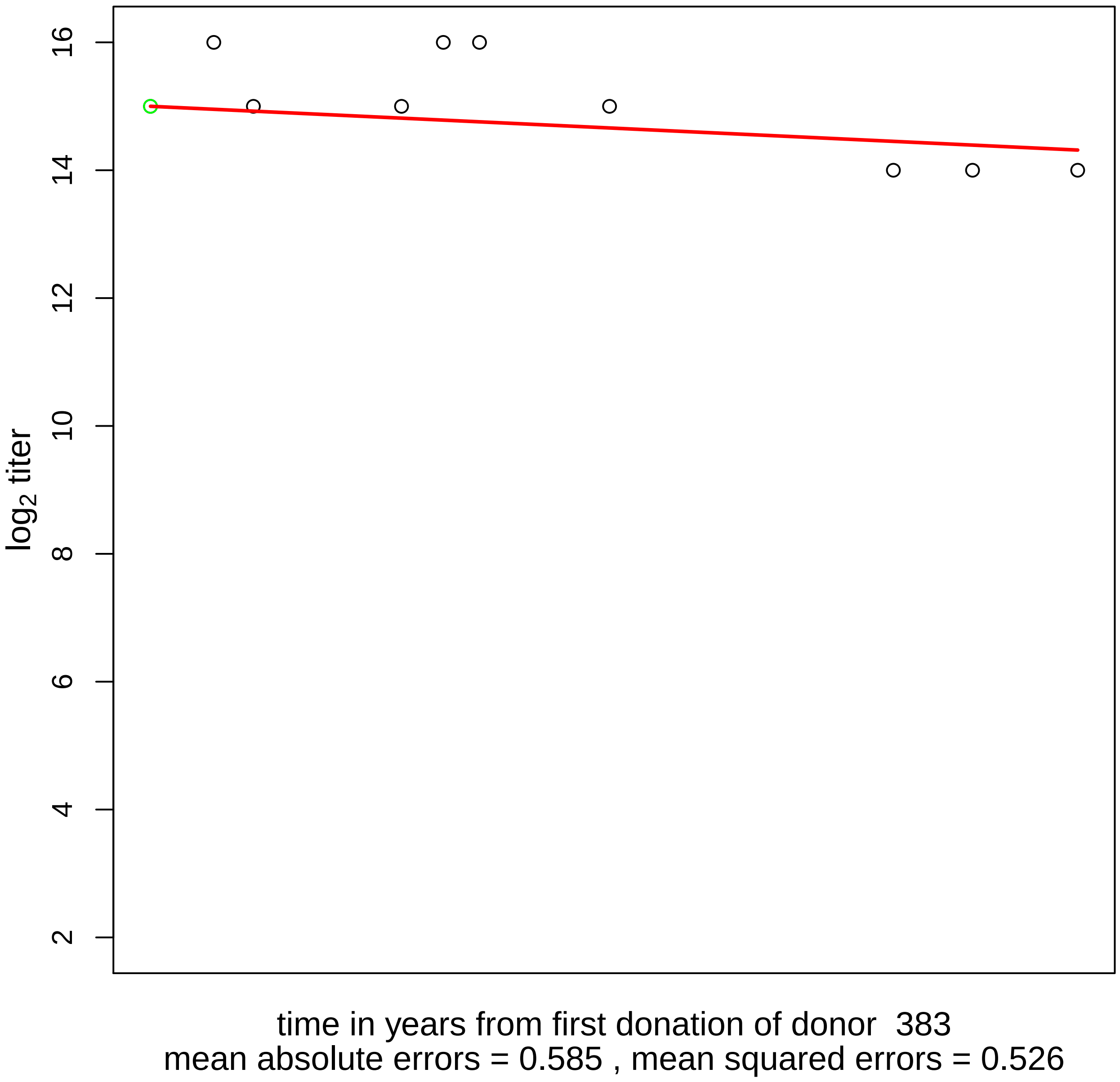

16
14
12
10
8
6
4
2
log2 titer
time in years from first donation of donor 383
mean absolute errors = 0.585 , mean squared errors = 0.526

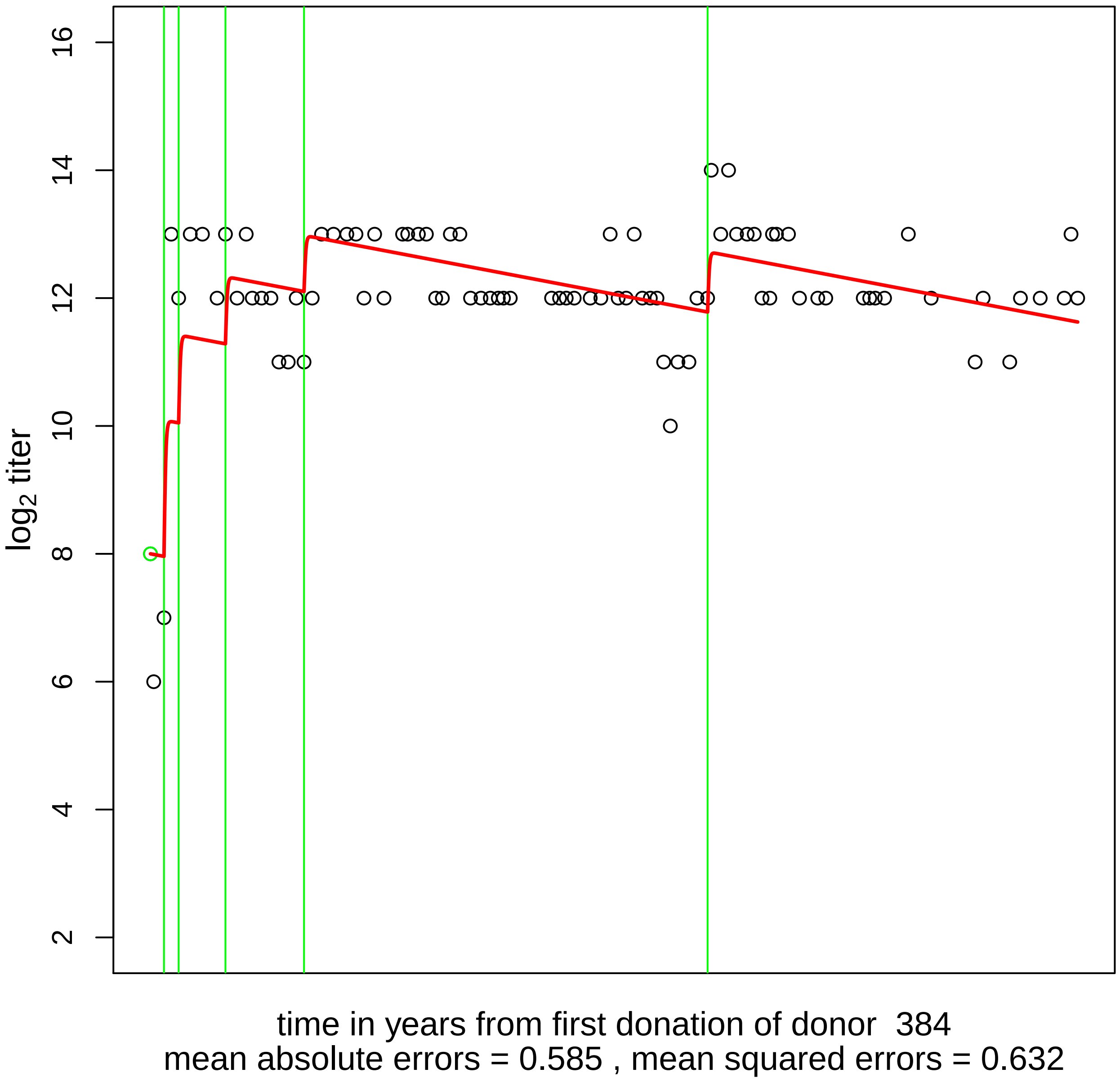

log2 titer
2
4
6
8
10
12
14
16
time in years from first donation of donor 384
mean absolute errors = 0.585 , mean squared errors = 0.632

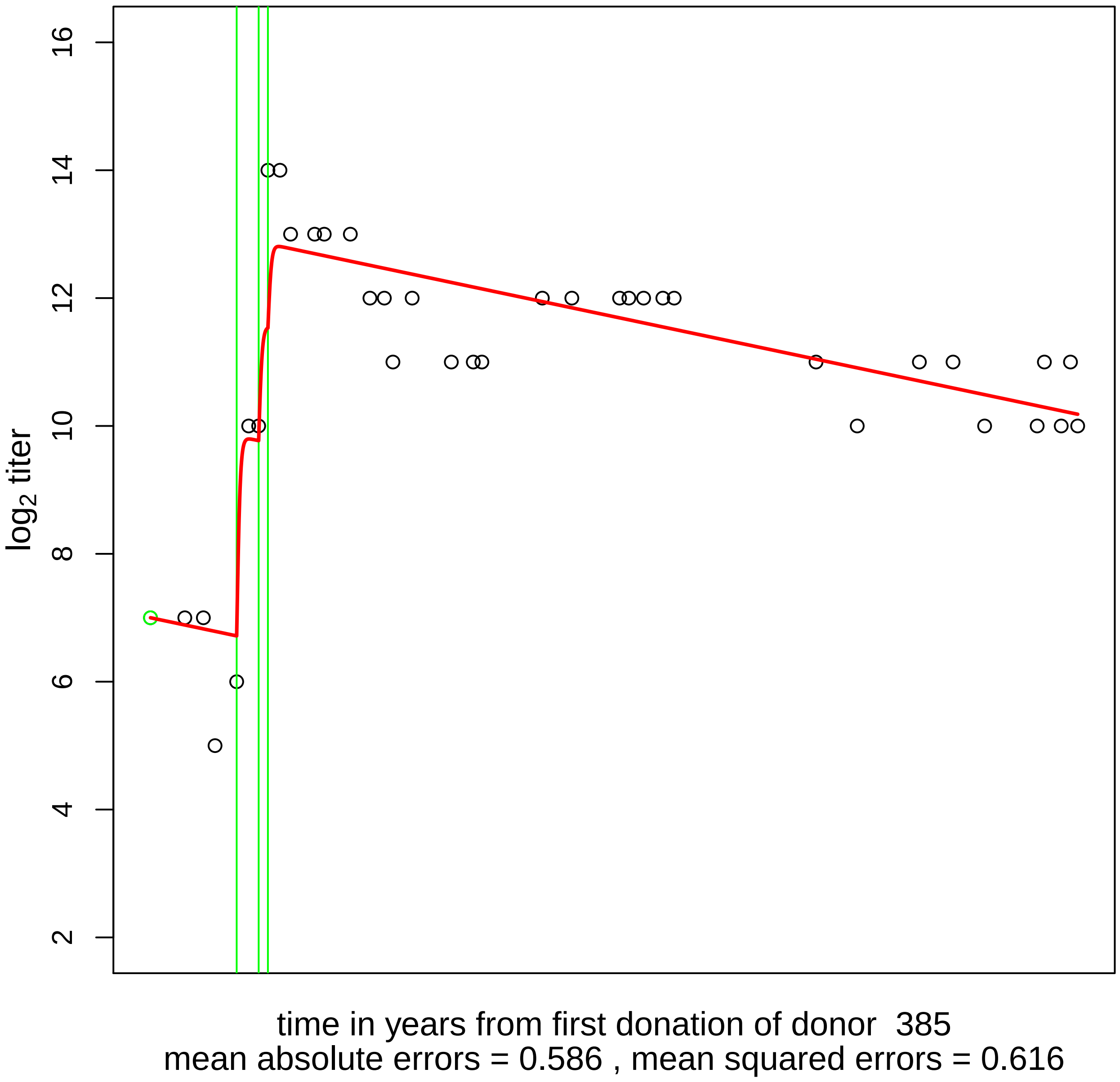

16
14
12
10
8
6
4
2
log2 titer
time in years from first donation of donor 385
mean absolute errors = 0.586 , mean squared errors = 0.616

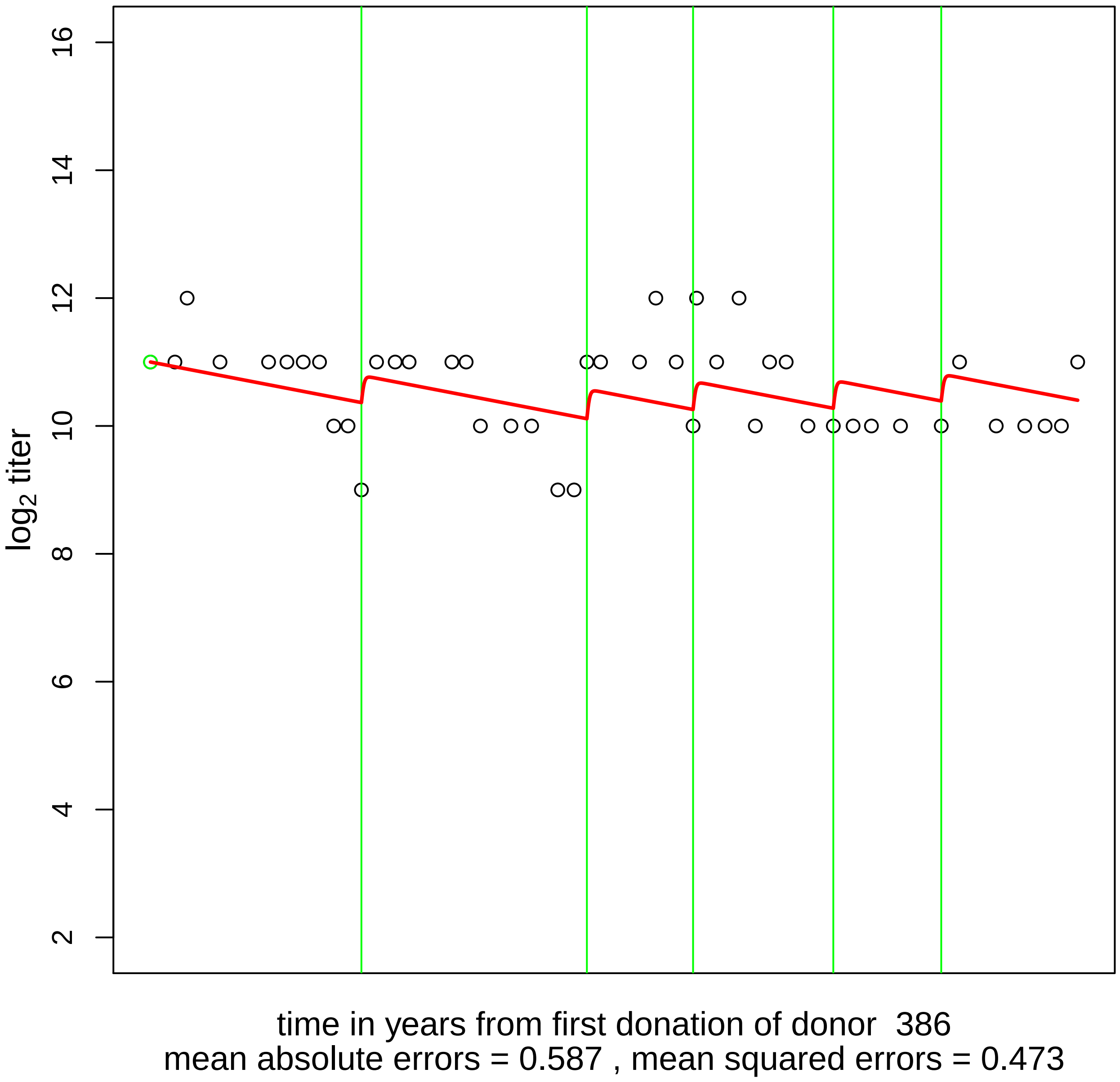

16
14
12
10
8
6
4
2
log2 titer
time in years from first donation of donor 386
mean absolute errors = 0.587 , mean squared errors = 0.473

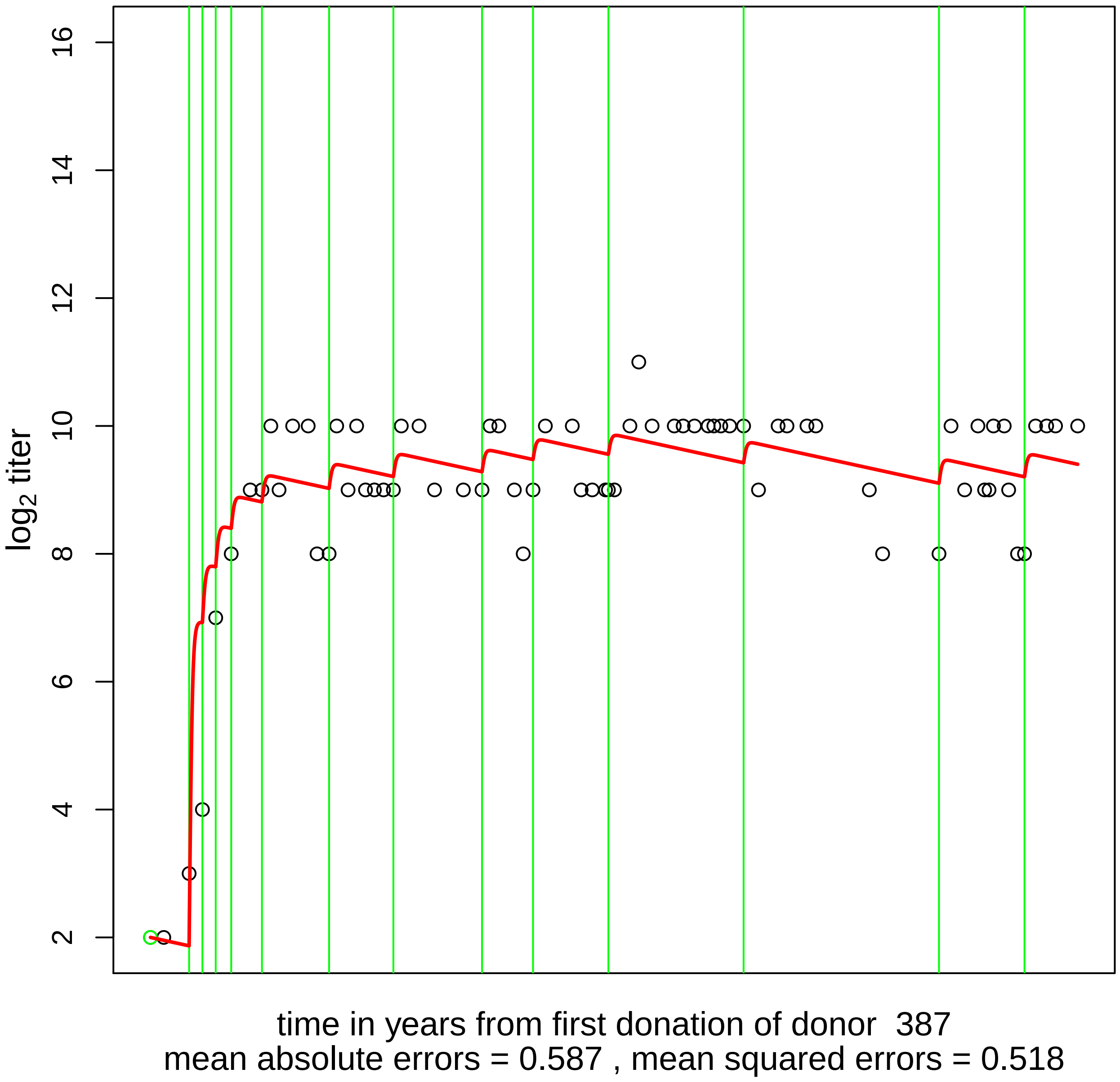

16
14
12
10
8
6
4
2
log2 titer
time in years from first donation of donor 387
mean absolute errors = 0.587 , mean squared errors = 0.518

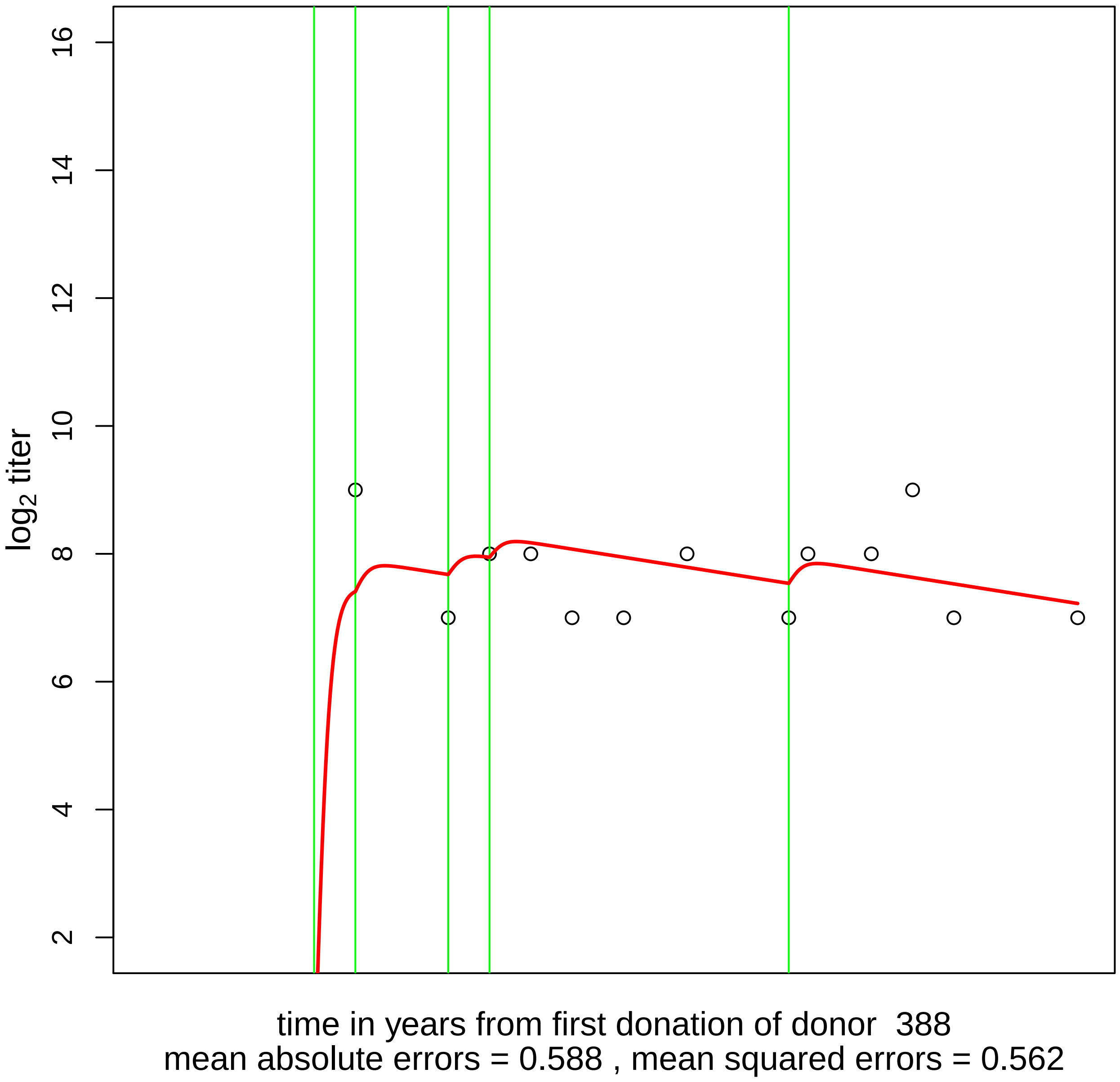

log2 titer
2
4
6
8
10
12
14
16
time in years from first donation of donor 388
mean absolute errors = 0.588 , mean squared errors = 0.562

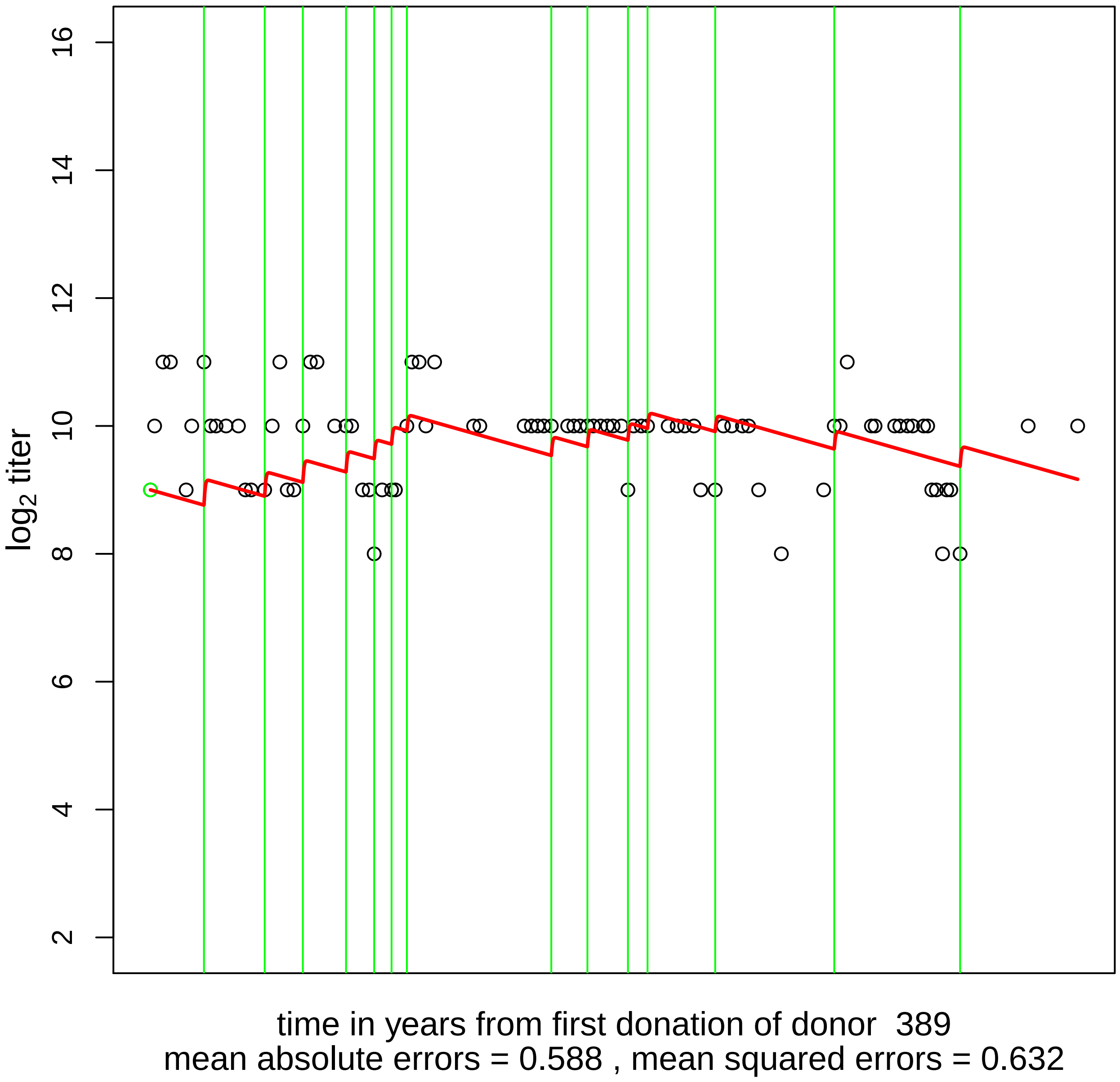

16
14
12
10
8
6
4
2
$\log_2$ titer
time in years from first donation of donor 389
mean absolute errors = 0.588 , mean squared errors = 0.632

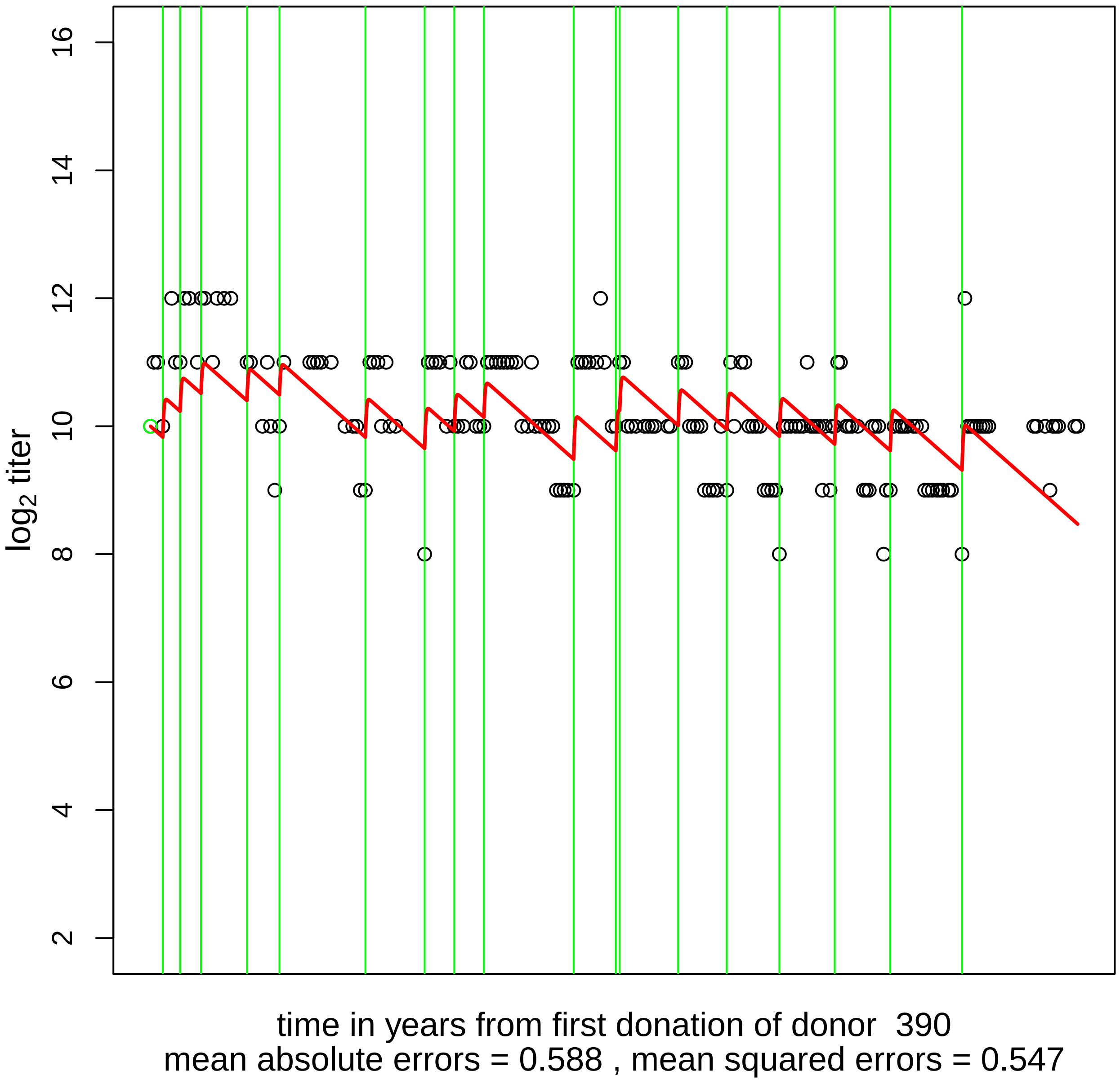

16
14
12
10
8
6
4
2
log2 titer
time in years from first donation of donor 390
mean absolute errors = 0.588 , mean squared errors = 0.547

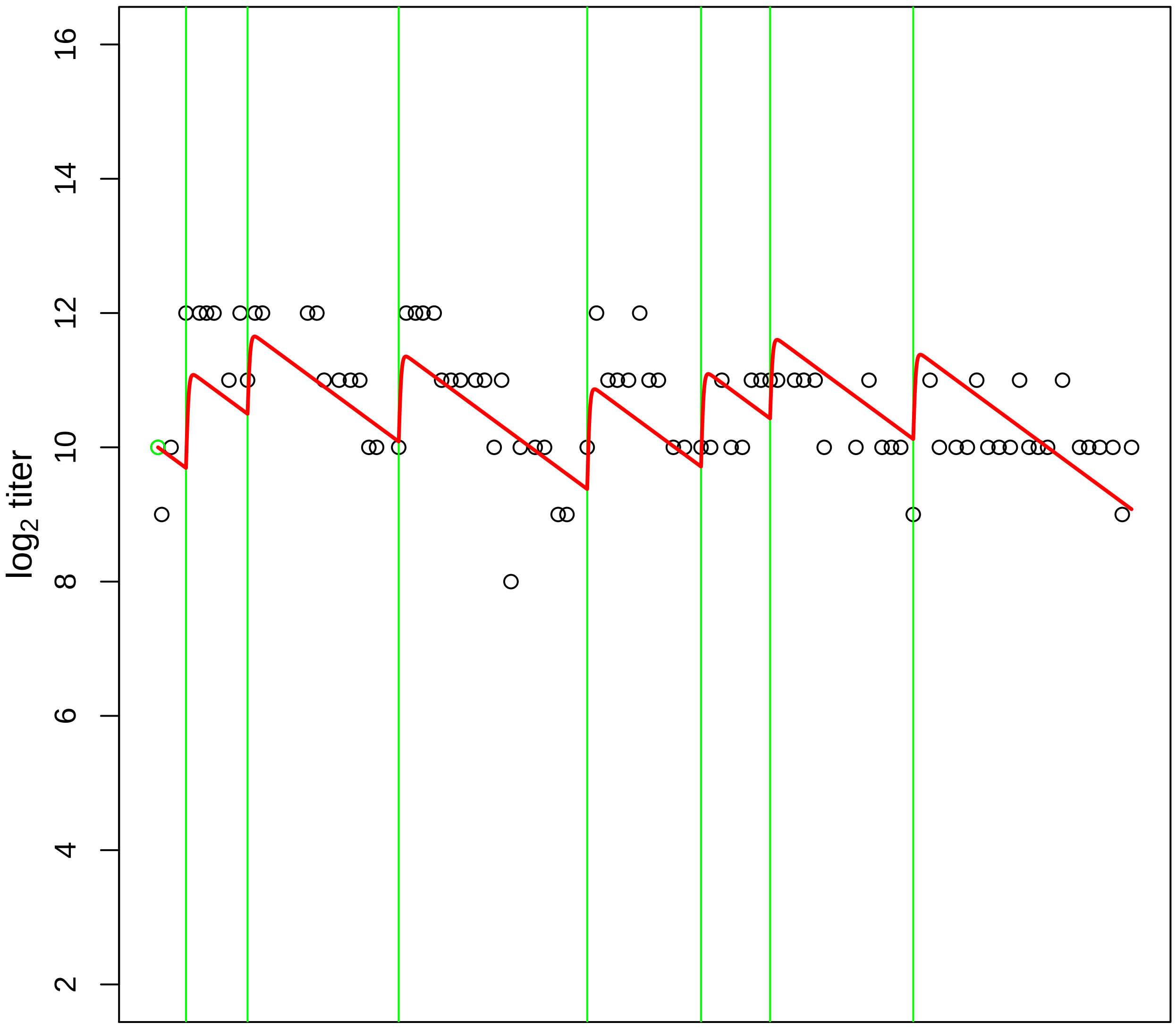

time in years from first donation of donor 391
mean absolute errors = 0.588 , mean squared errors = 0.54

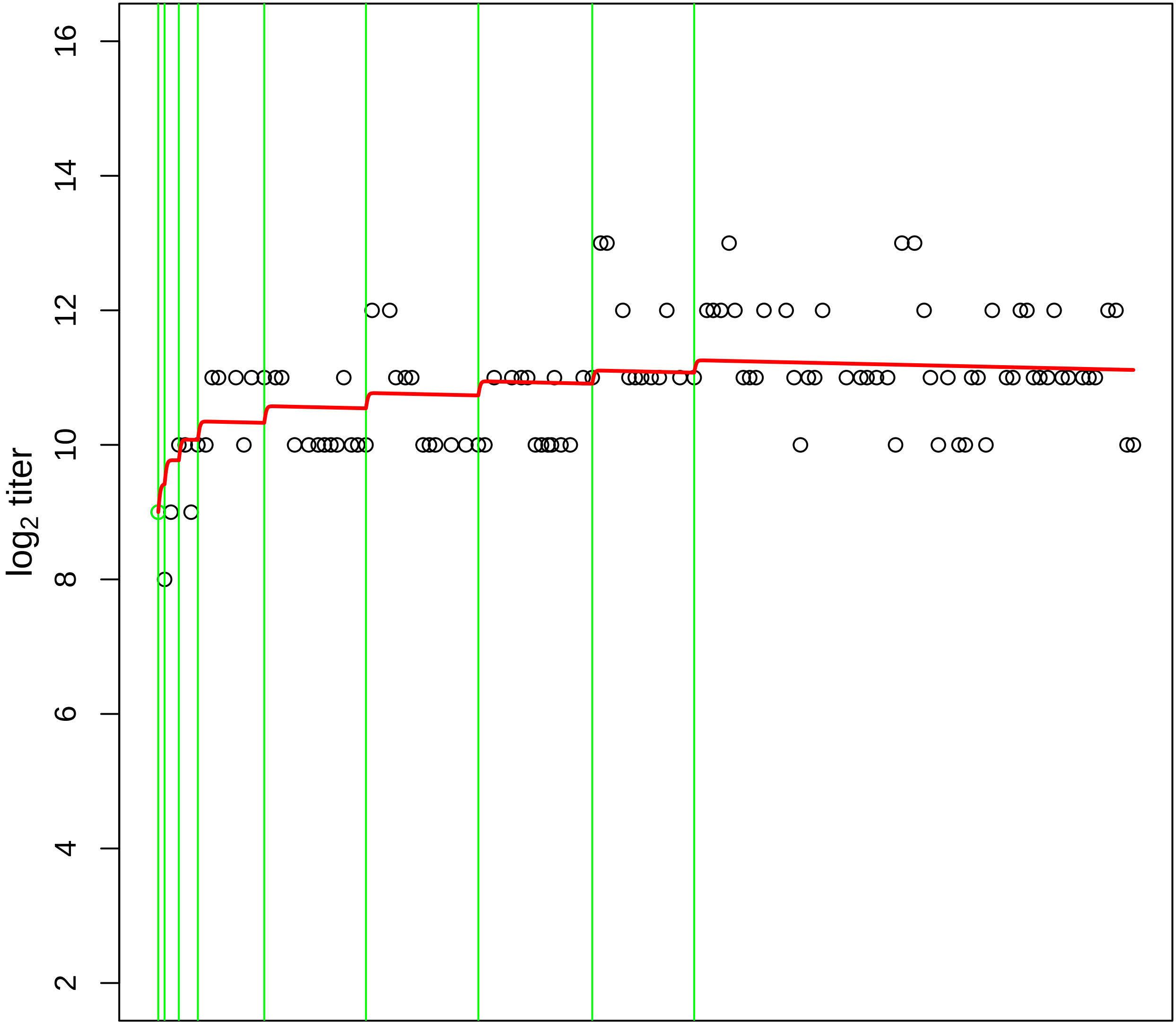

time in years from first donation of donor 392
mean absolute errors = 0.588 , mean squared errors = 0.555

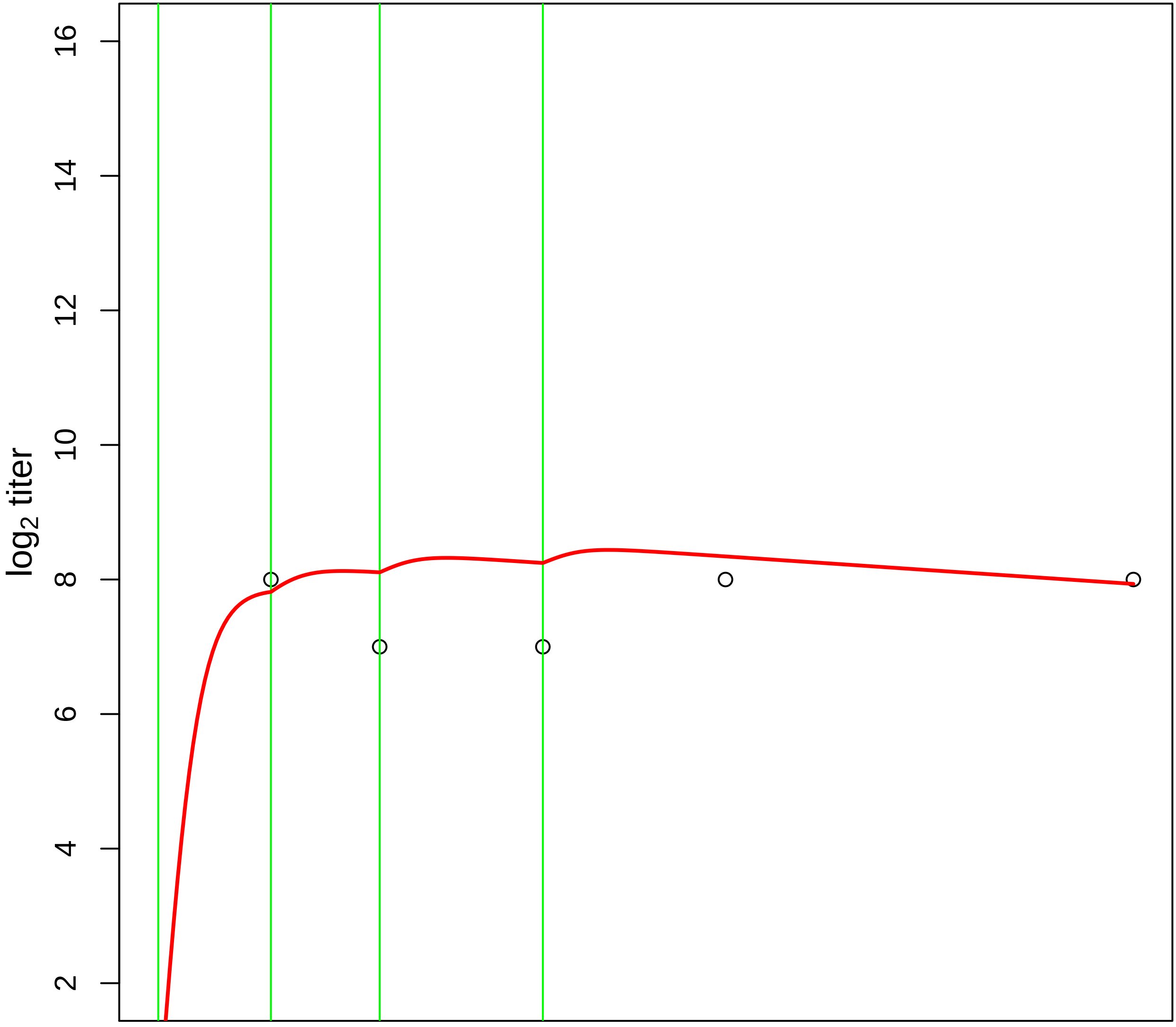

time in years from first donation of donor 393
mean absolute errors = 0.589 , mean squared errors = 0.586

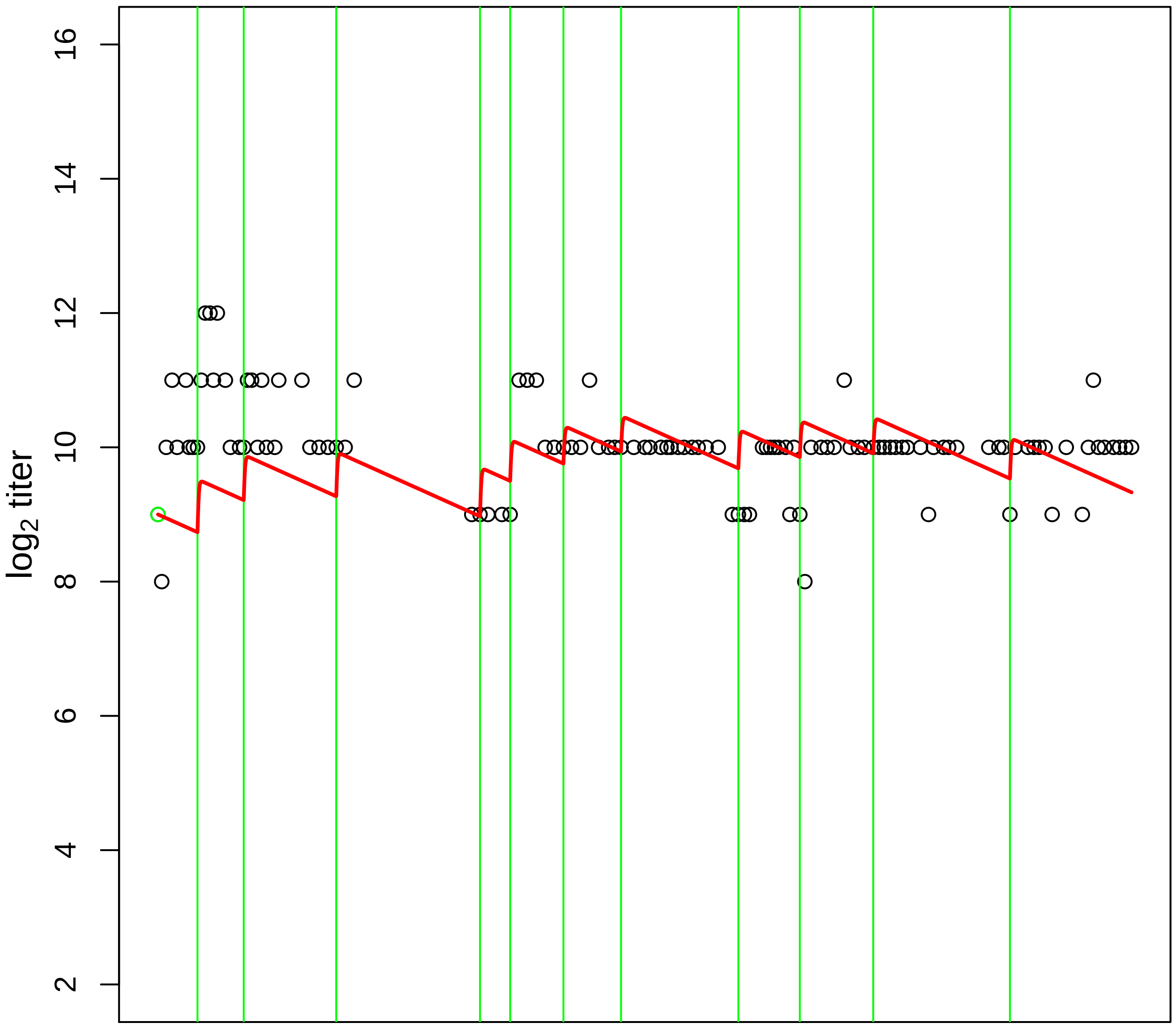

time in years from first donation of donor 394
mean absolute errors = 0.589 , mean squared errors = 0.717

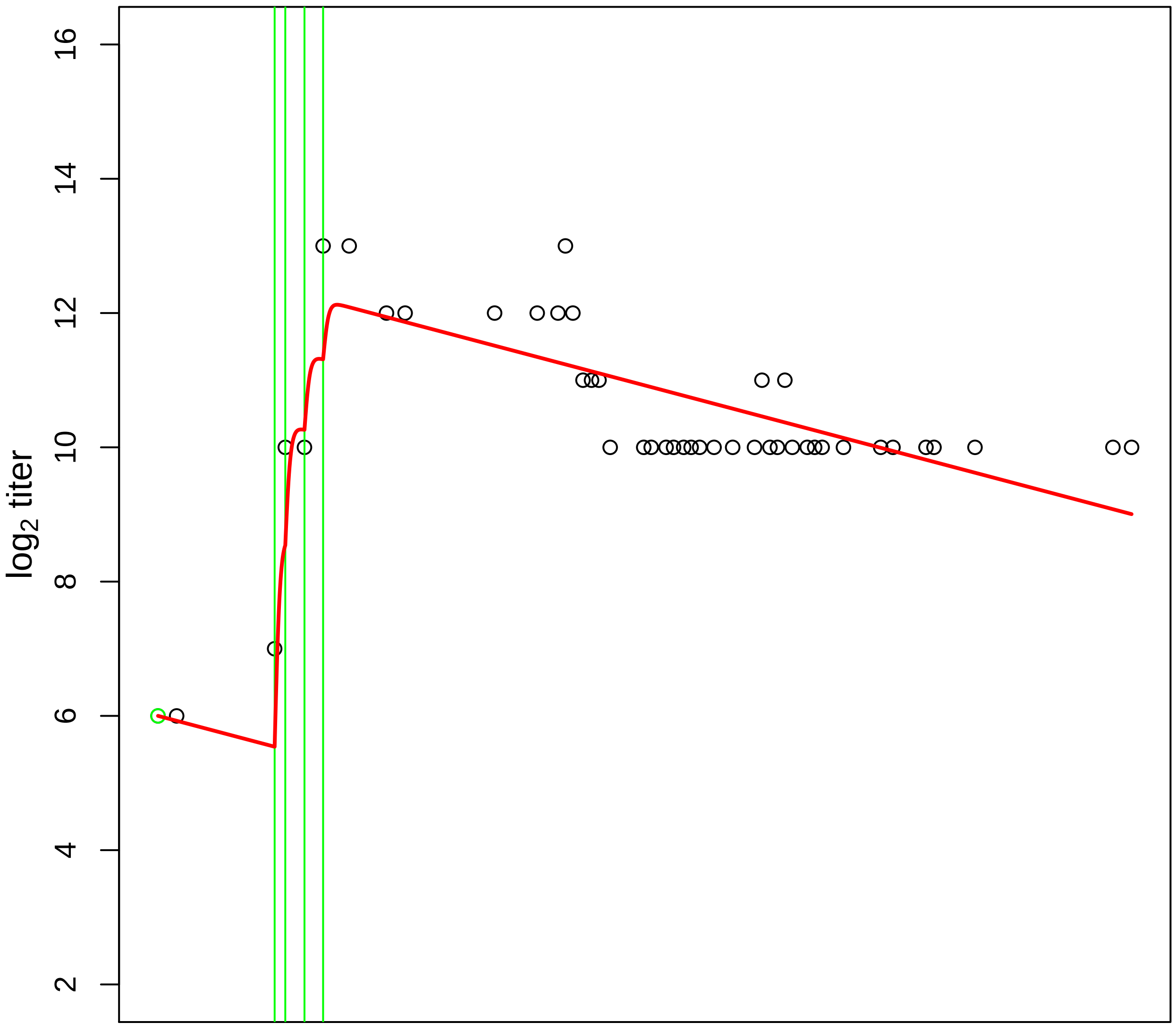

time in years from first donation of donor 395
mean absolute errors = 0.589 , mean squared errors = 0.541

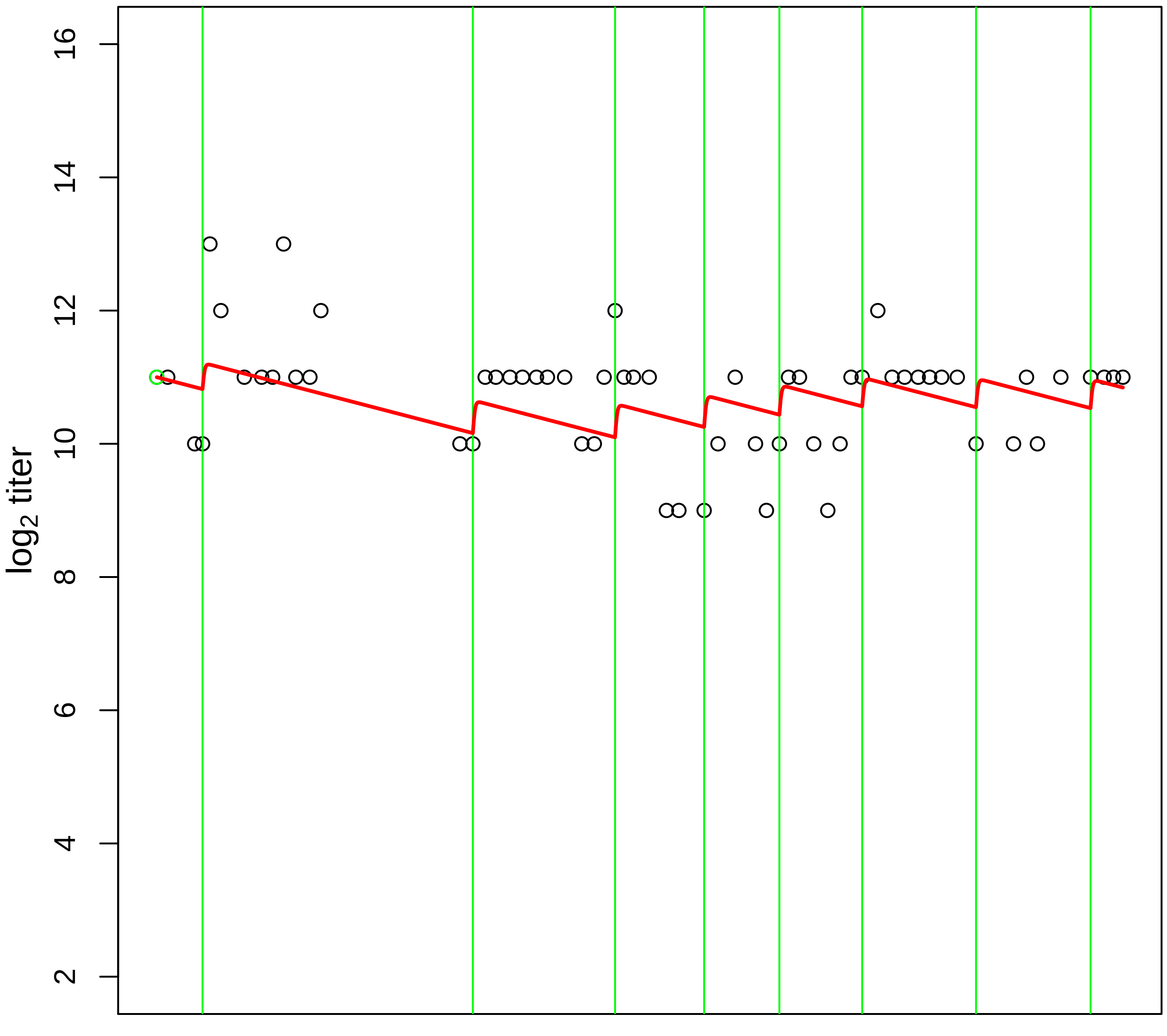

time in years from first donation of donor 396
mean absolute errors = 0.59 , mean squared errors = 0.597

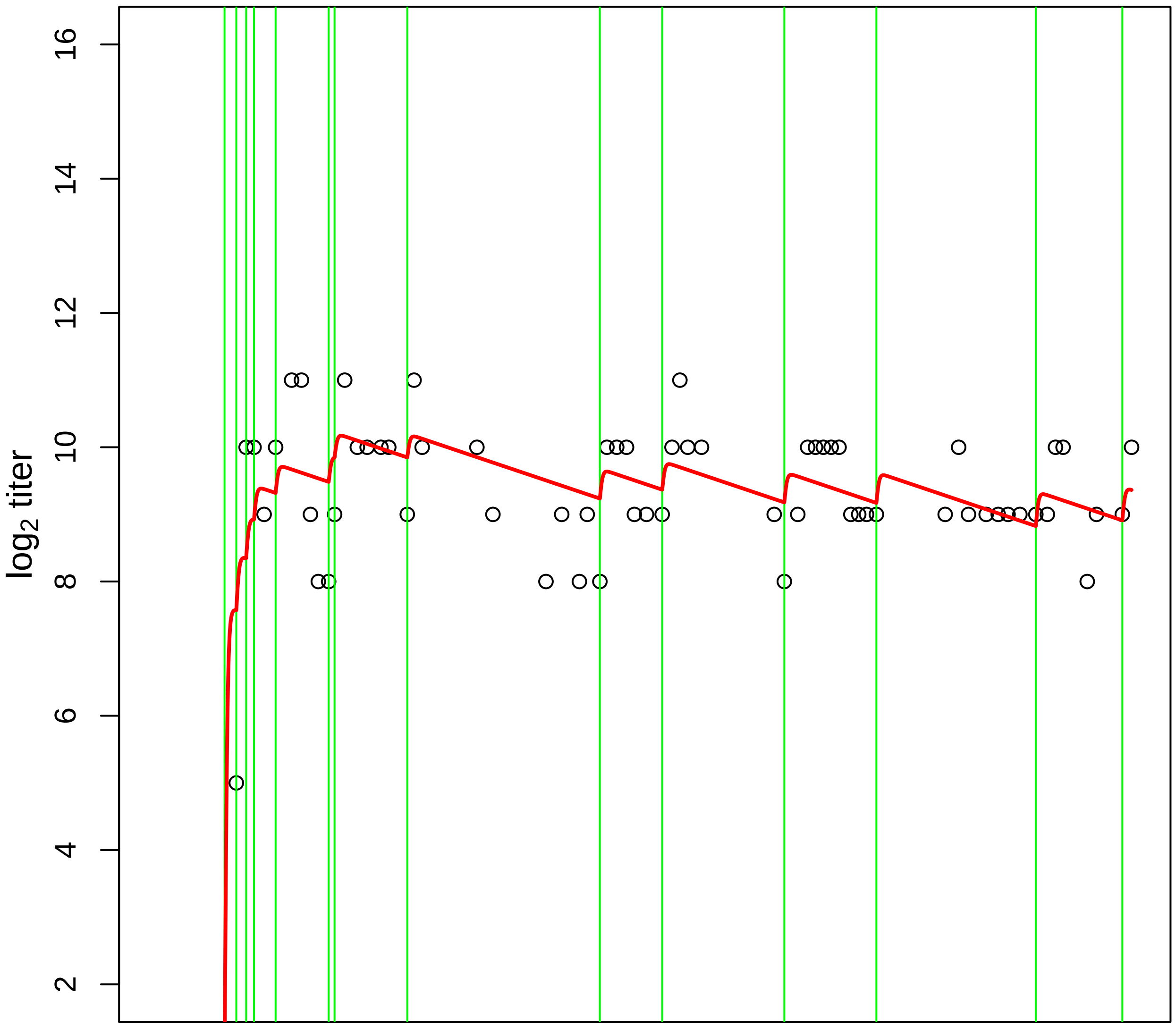

time in years from first donation of donor 397
mean absolute errors = 0.592 , mean squared errors = 0.608

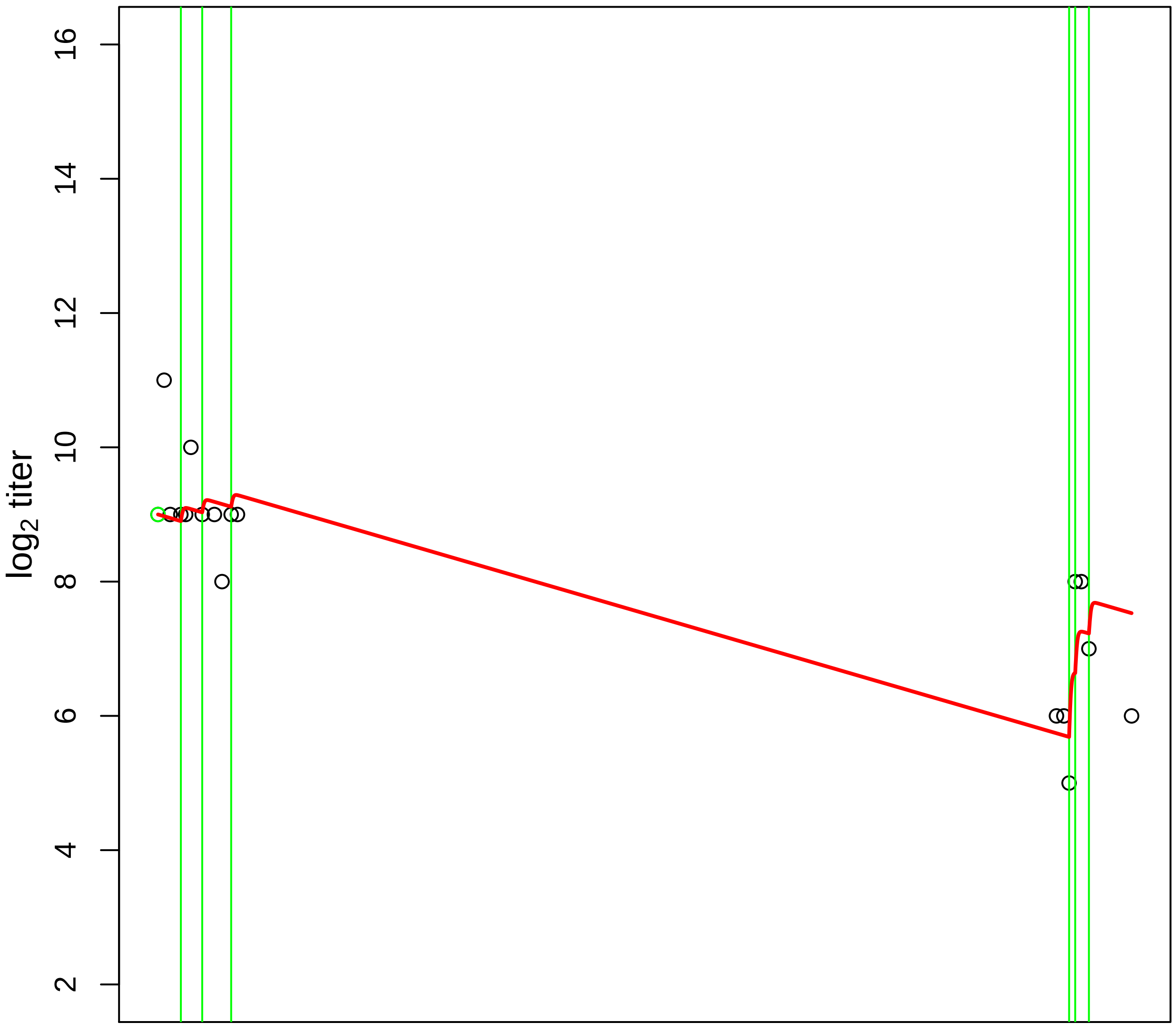

time in years from first donation of donor 398
mean absolute errors = 0.593 , mean squared errors = 0.698

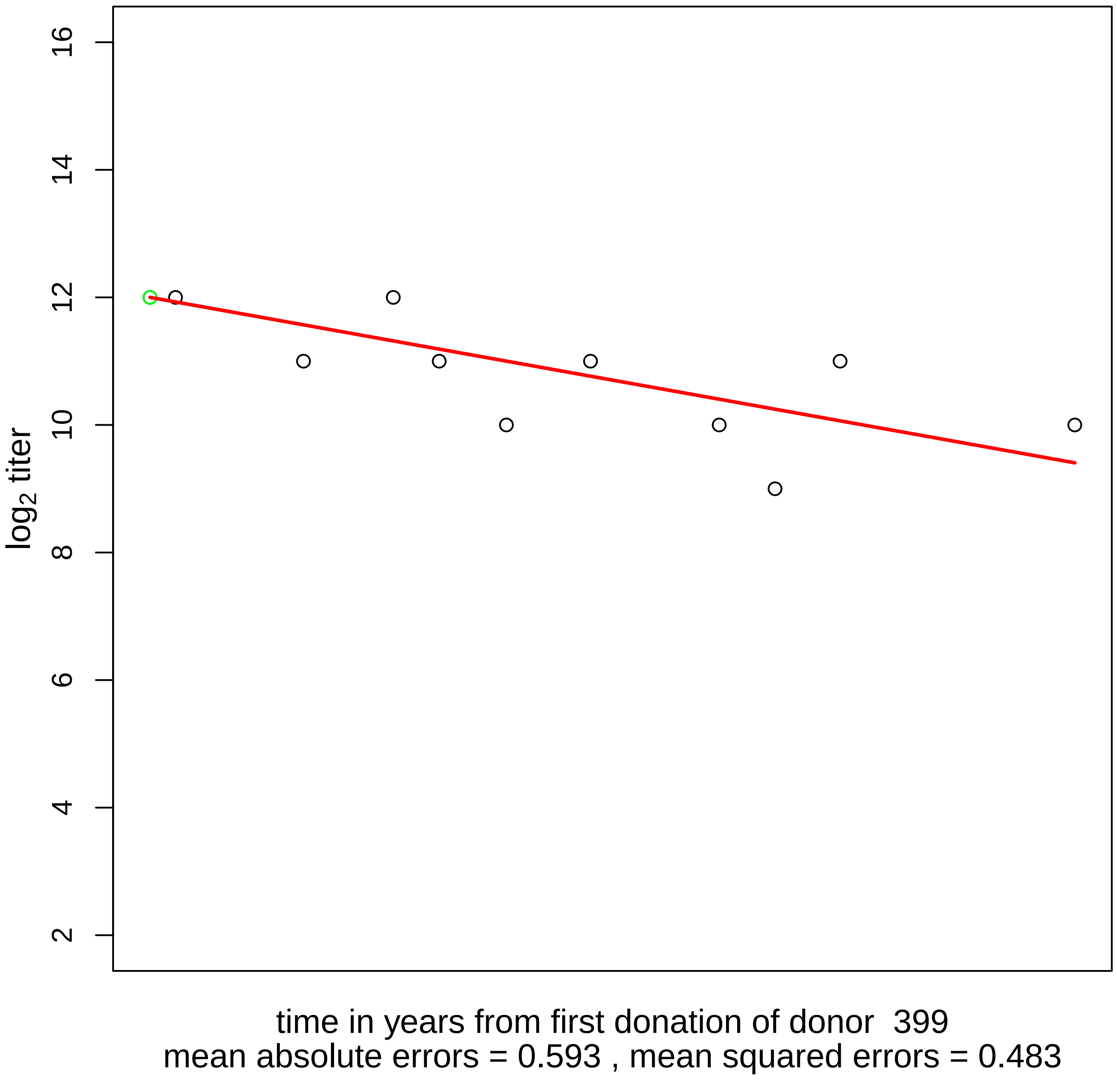

16
14
12
10
8
6
4
2
$\log_2$ titer
time in years from first donation of donor 399
mean absolute errors = 0.593 , mean squared errors = 0.483

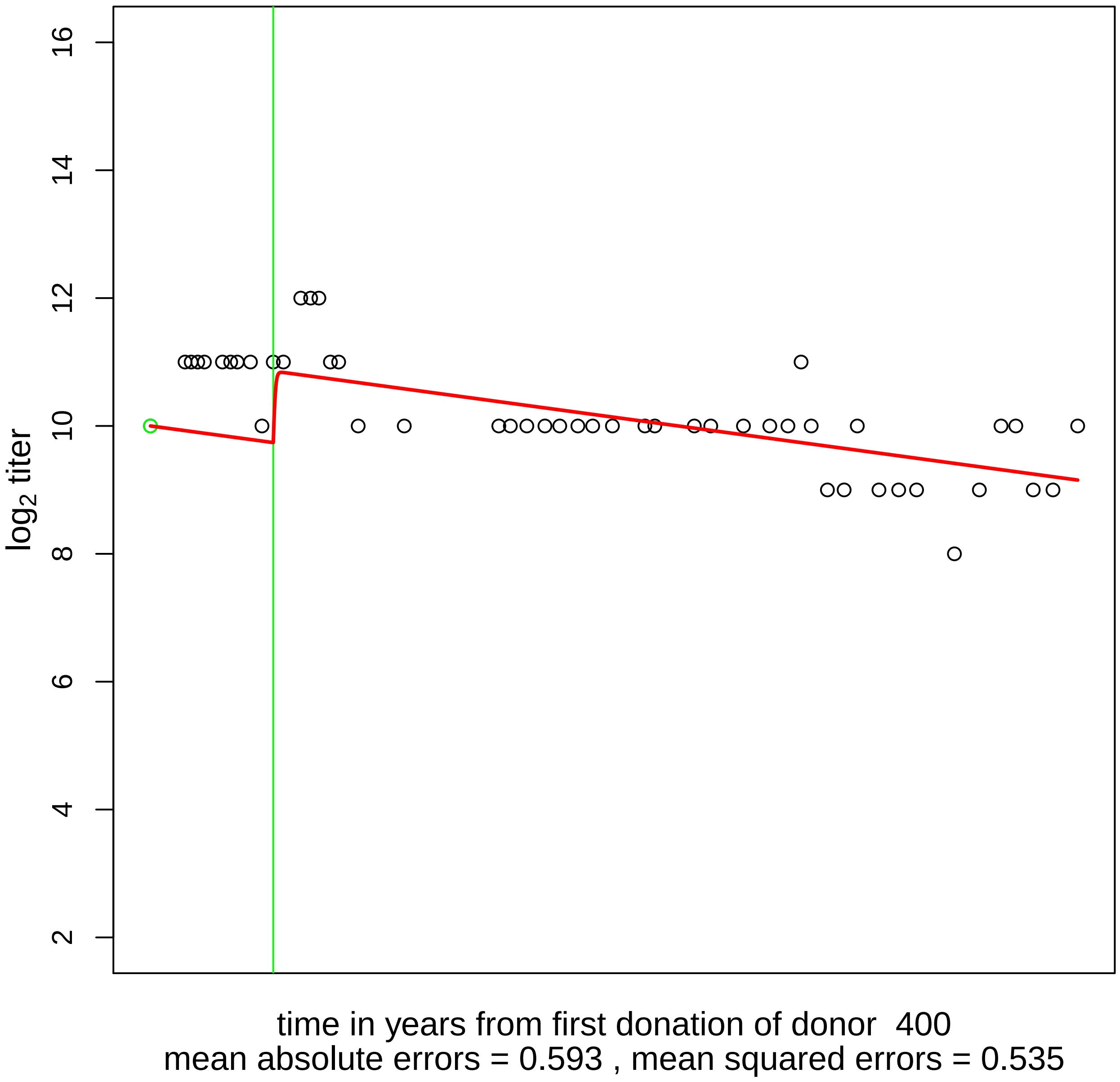

16
14
12
10
8
6
4
2
log2 titer
time in years from first donation of donor 400
mean absolute errors = 0.593 , mean squared errors = 0.535

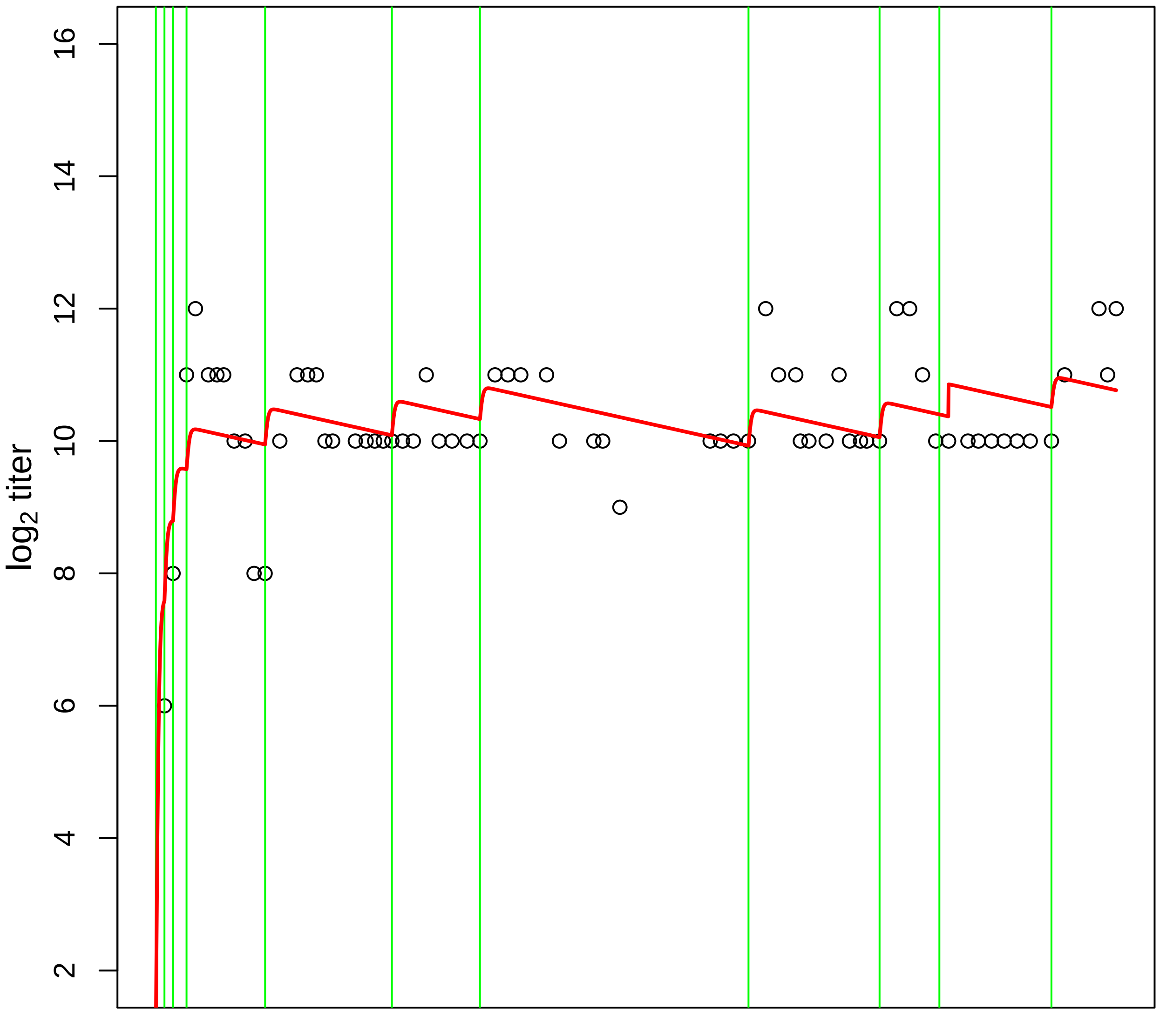

time in years from first donation of donor 401
mean absolute errors = 0.594 , mean squared errors = 0.597

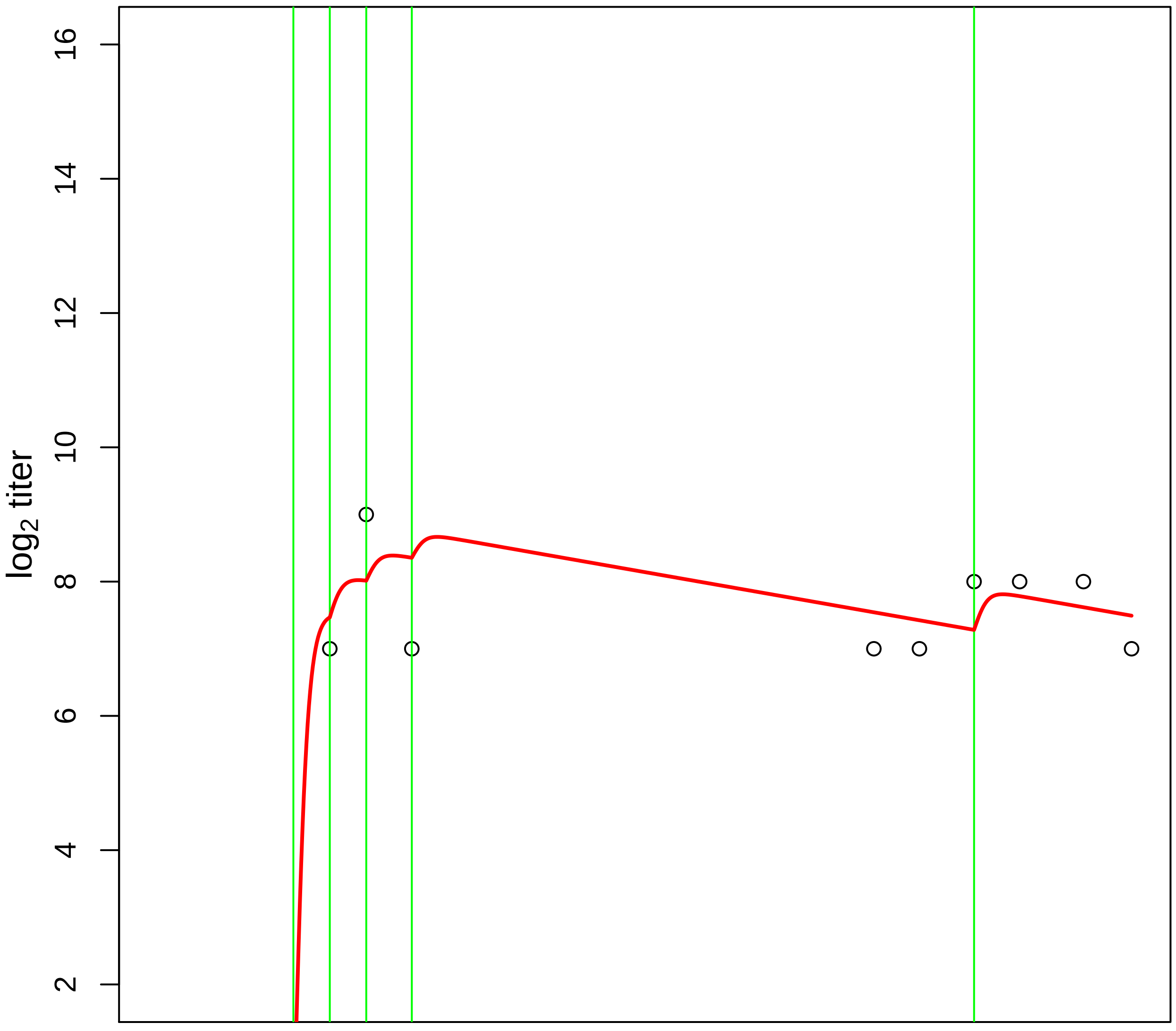

time in years from first donation of donor 402
mean absolute errors = 0.594 , mean squared errors = 0.458

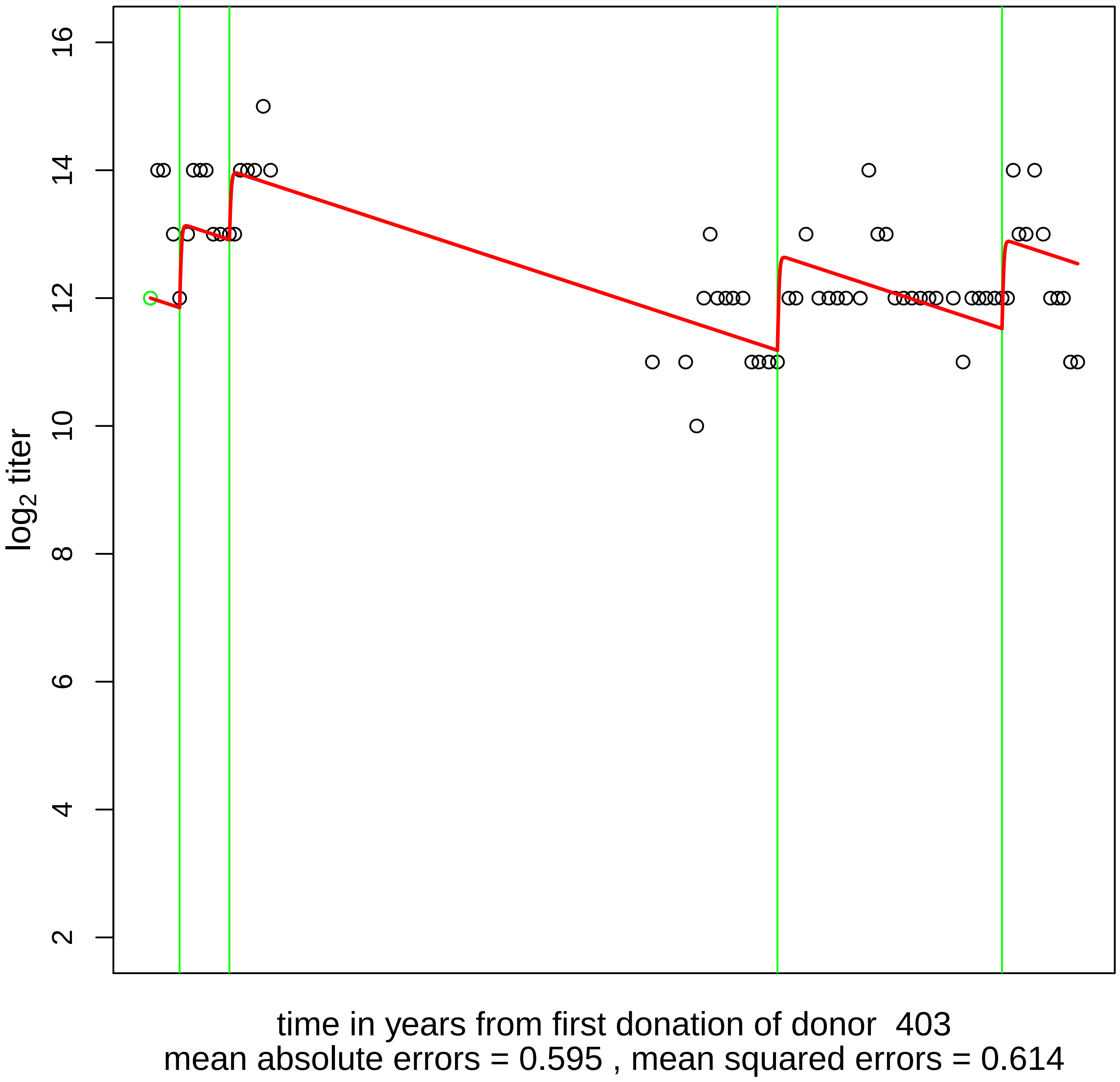

16
14
12
10
8
6
4
2
log2 titer
time in years from first donation of donor 403
mean absolute errors = 0.595 , mean squared errors = 0.614

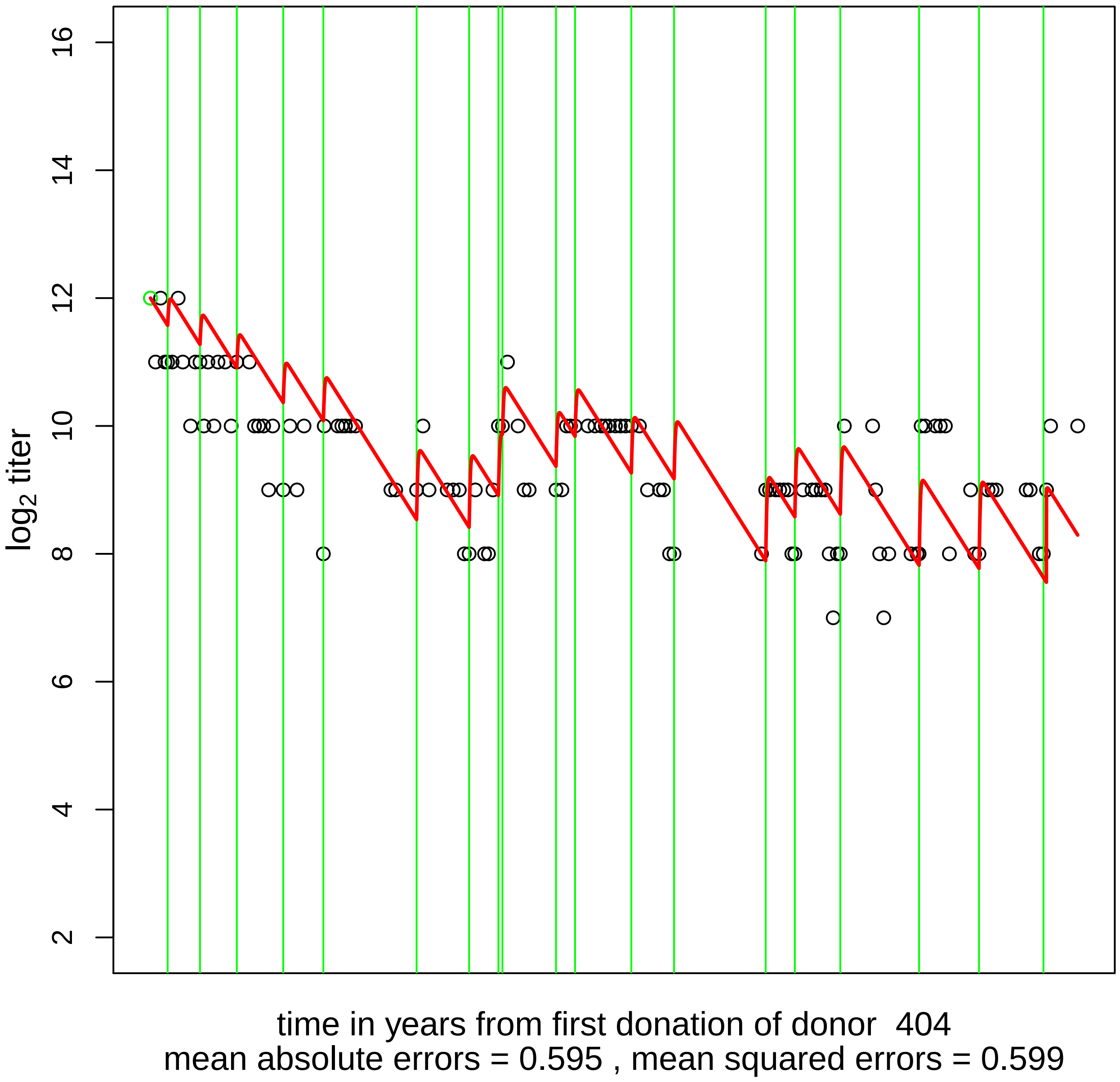

log2 titer
16
14
12
10
8
6
4
2
time in years from first donation of donor 404
mean absolute errors = 0.595 , mean squared errors = 0.599

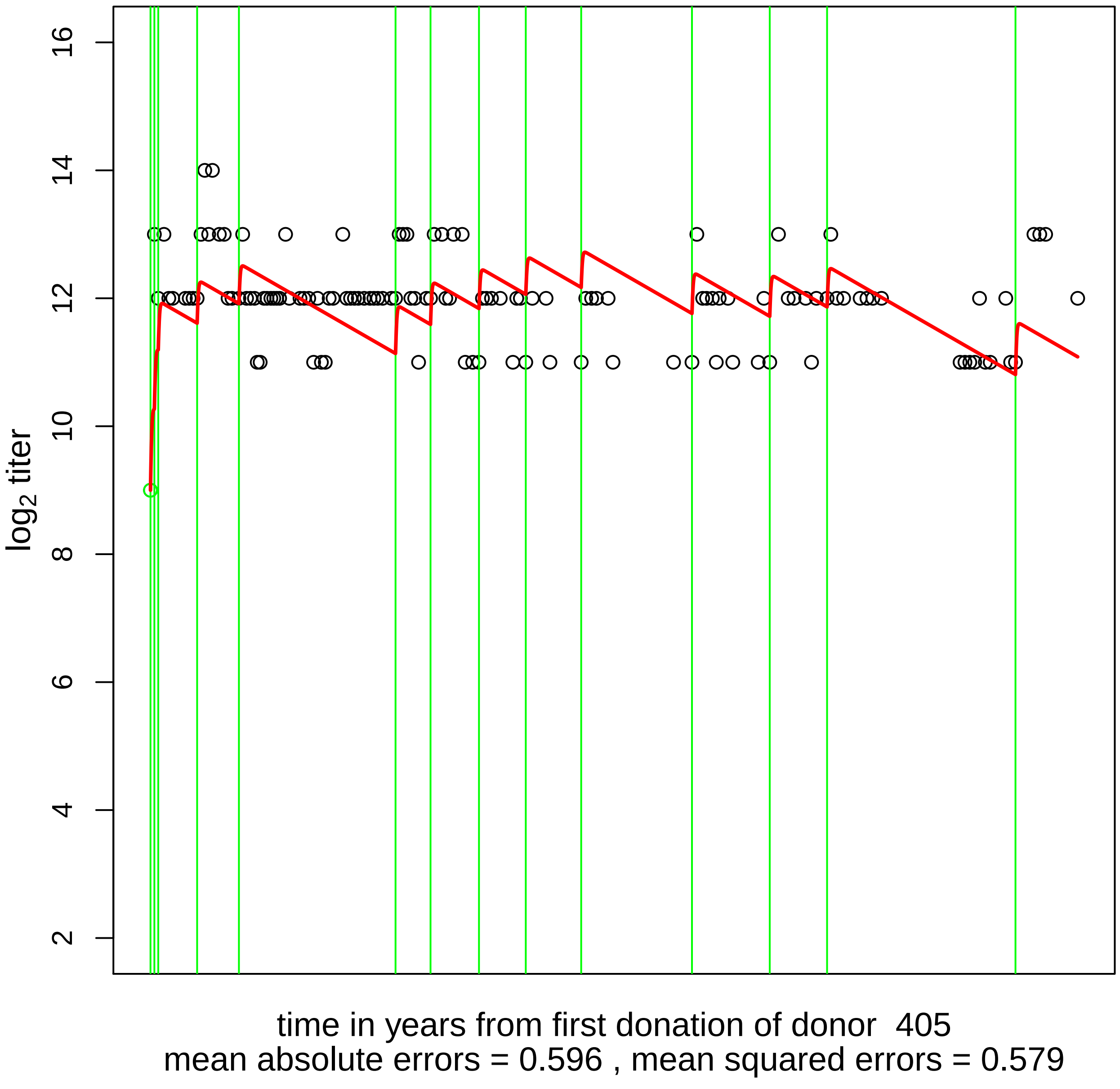

log2 titer
2
4
6
8
10
12
14
16
time in years from first donation of donor 405
mean absolute errors = 0.596 , mean squared errors = 0.579

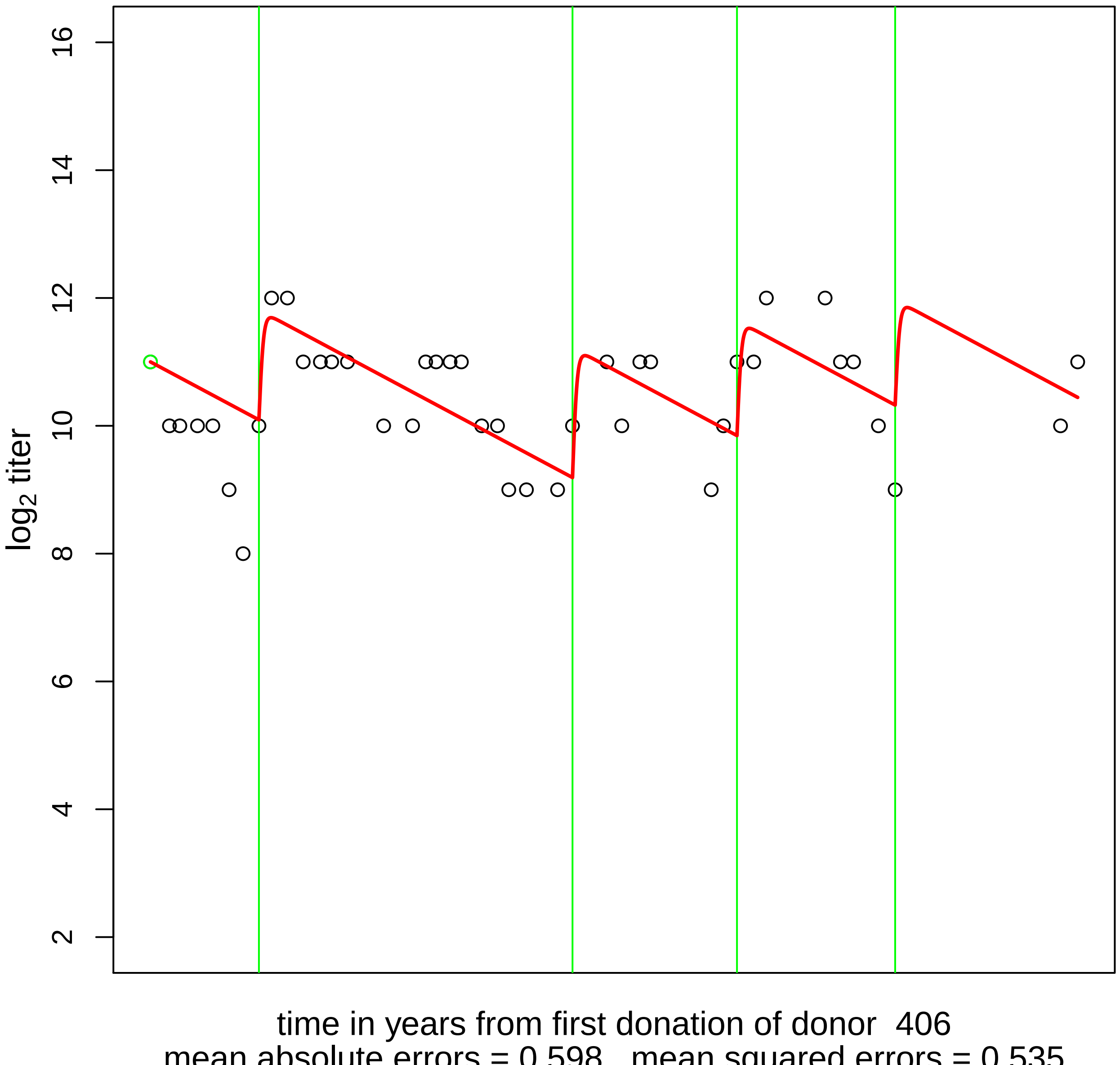

log2 titer
2
4
6
8
10
12
14
16
time in years from first donation of donor 406
mean absolute errors = 0.598 , mean squared errors = 0.535

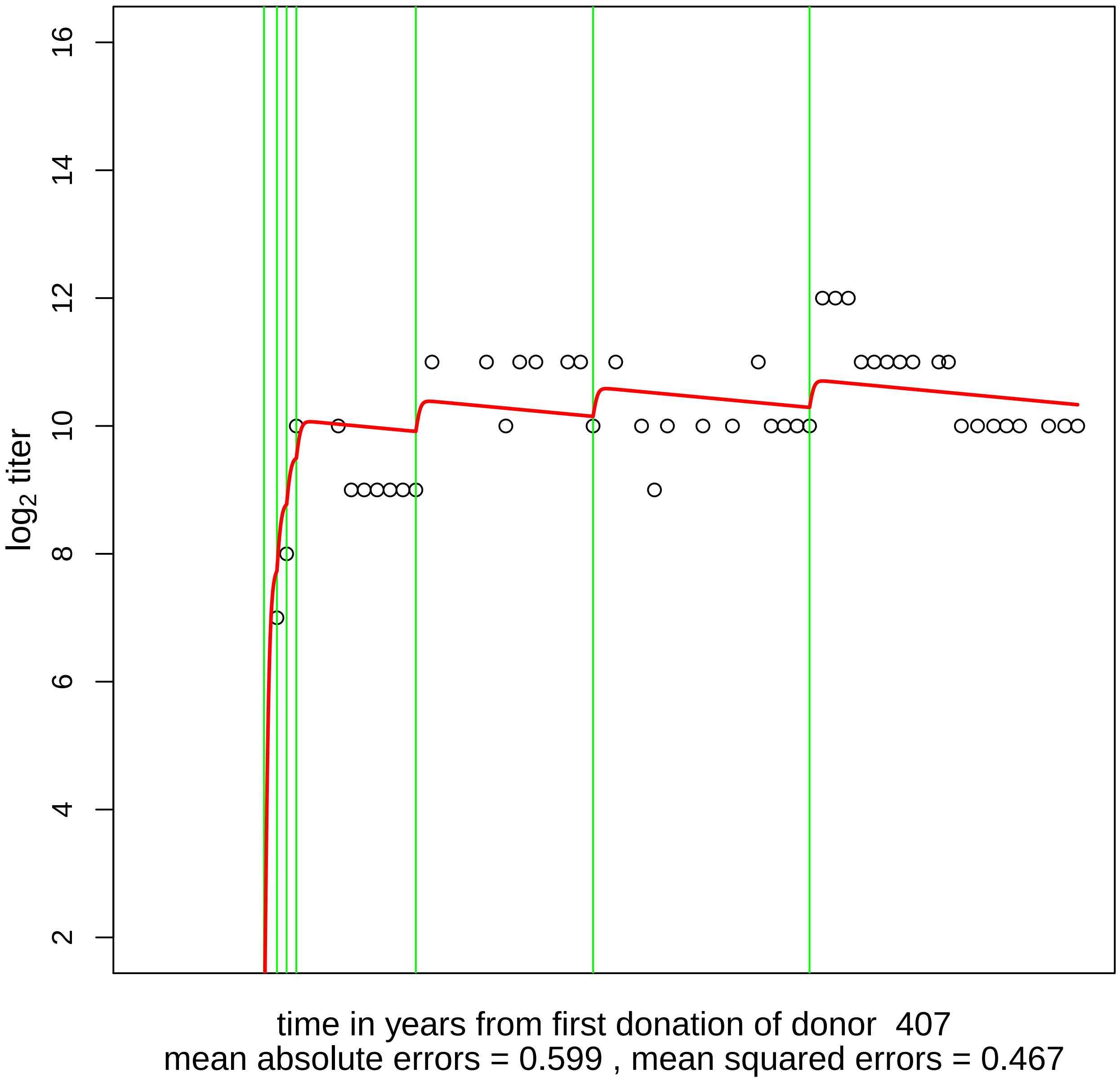

log2 titer
2
4
6
8
10
12
14
16
time in years from first donation of donor 407
mean absolute errors = 0.599 , mean squared errors = 0.467

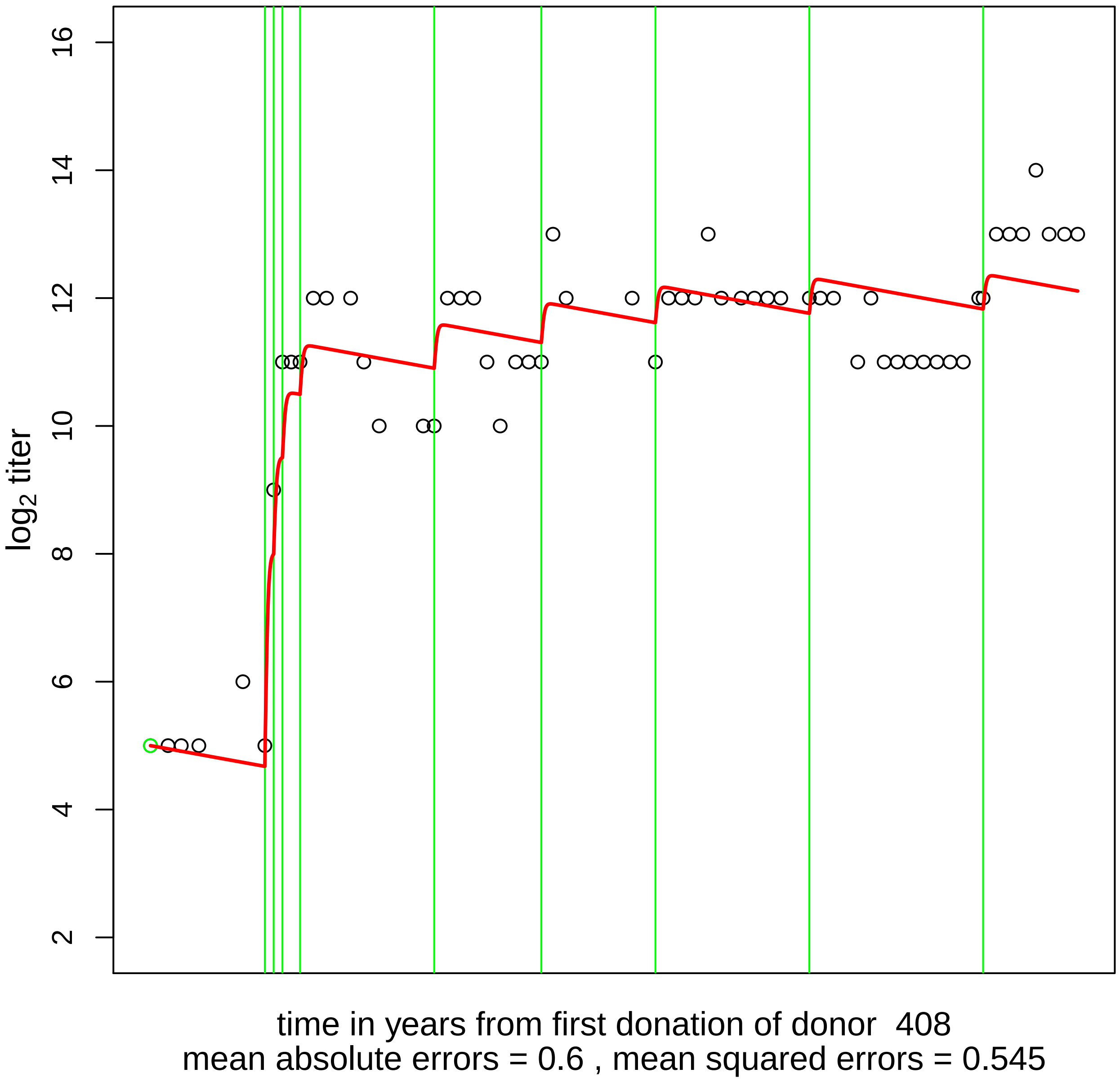

16
14
12
10
8
6
4
2
$\log_2$ titer
time in years from first donation of donor 408
mean absolute errors = 0.6 , mean squared errors = 0.545

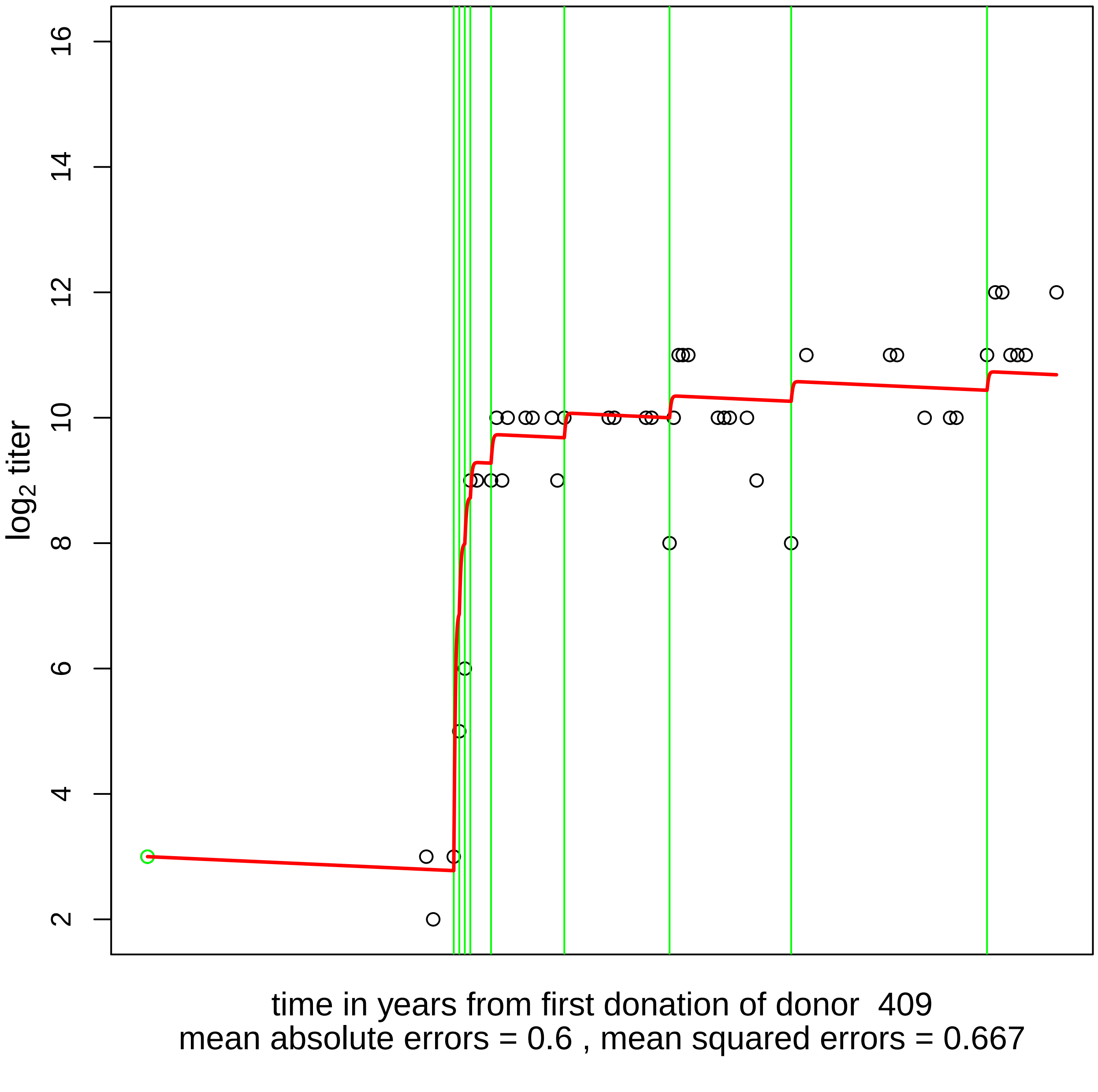

log2 titer
16
14
12
10
8
6
4
2
time in years from first donation of donor 409
mean absolute errors = 0.6 , mean squared errors = 0.667

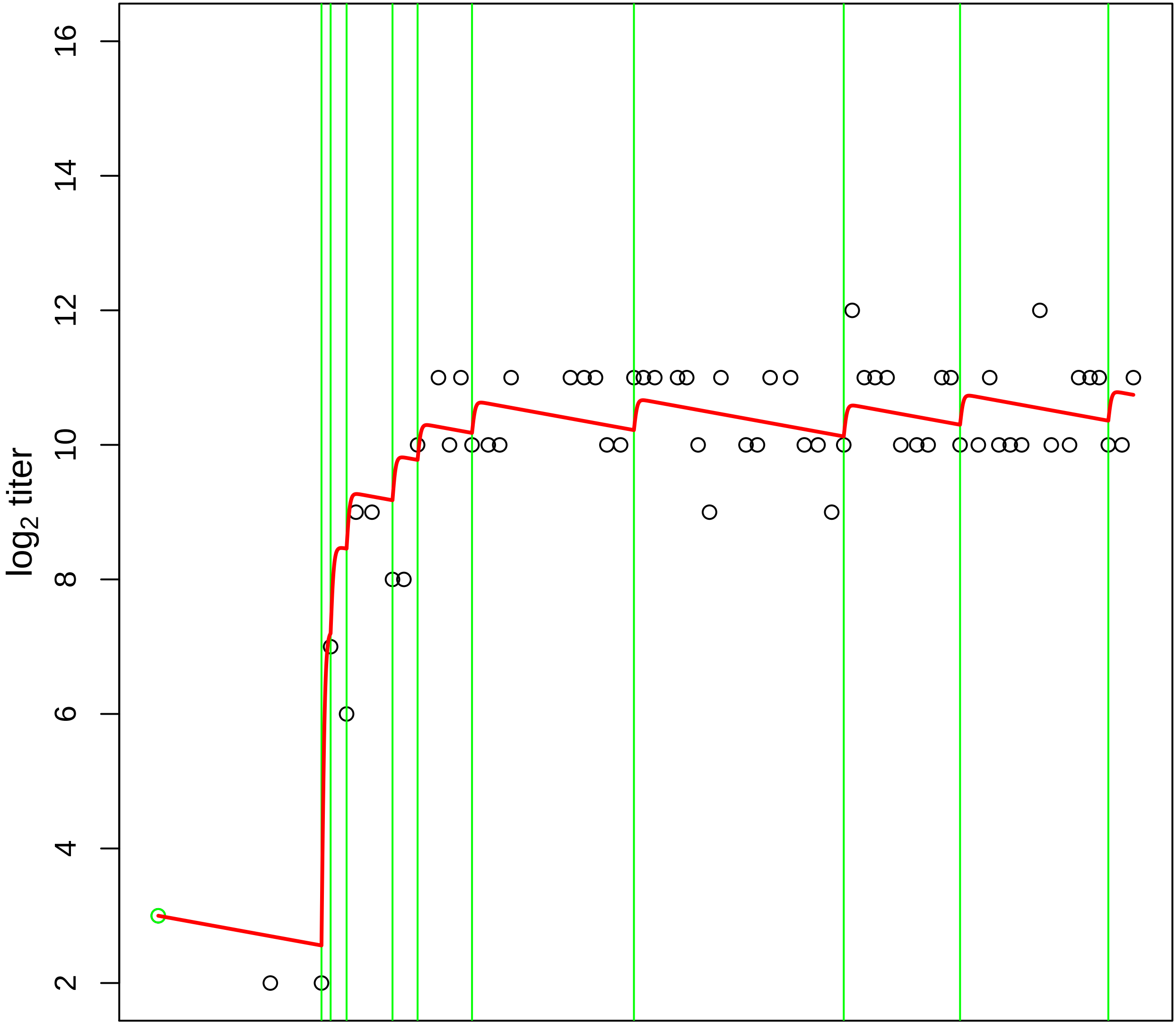

time in years from first donation of donor 410
mean absolute errors = 0.601 , mean squared errors = 0.531

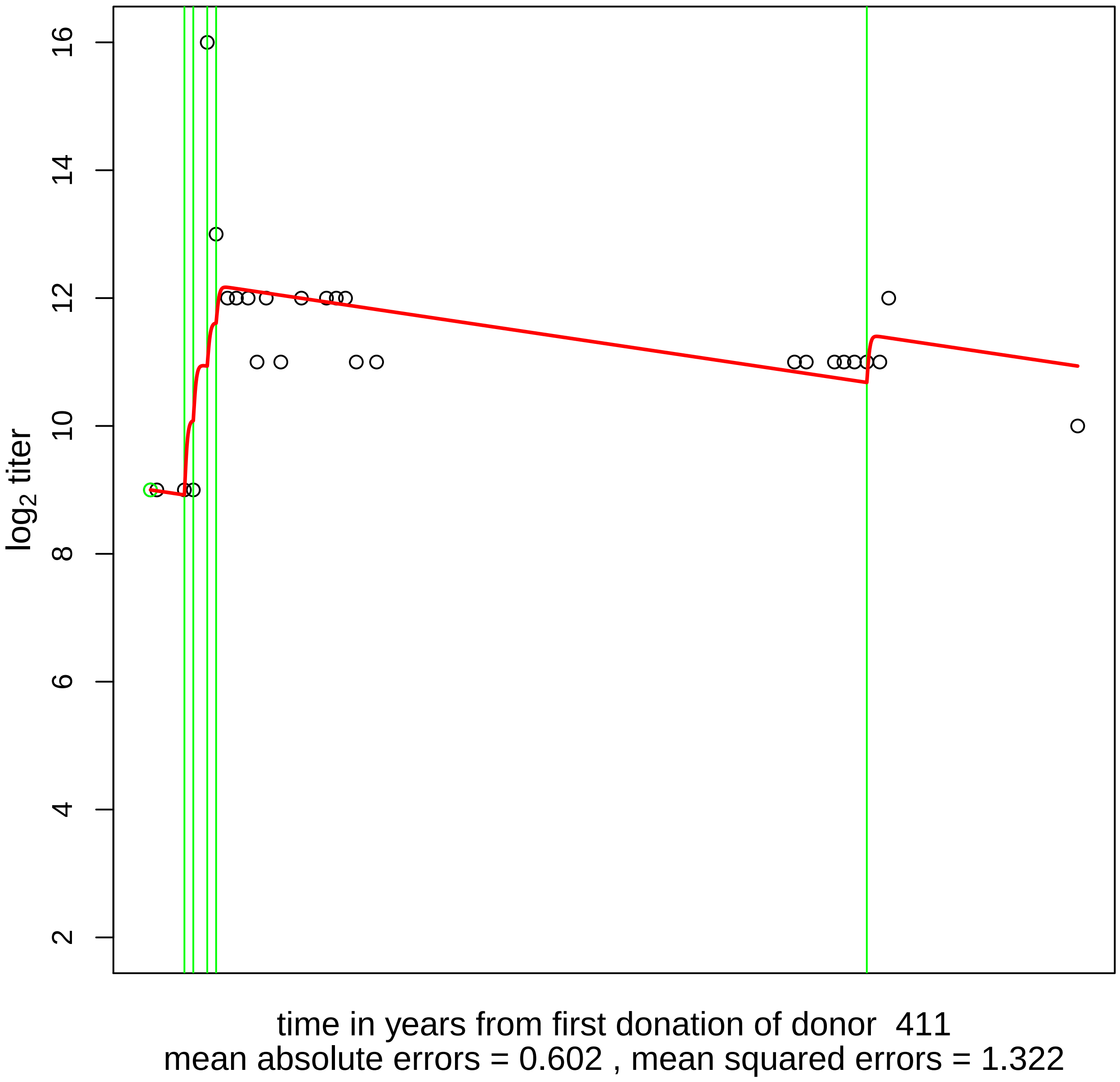

16
14
12
10
8
6
4
2
log2 titer
time in years from first donation of donor 411
mean absolute errors = 0.602 , mean squared errors = 1.322

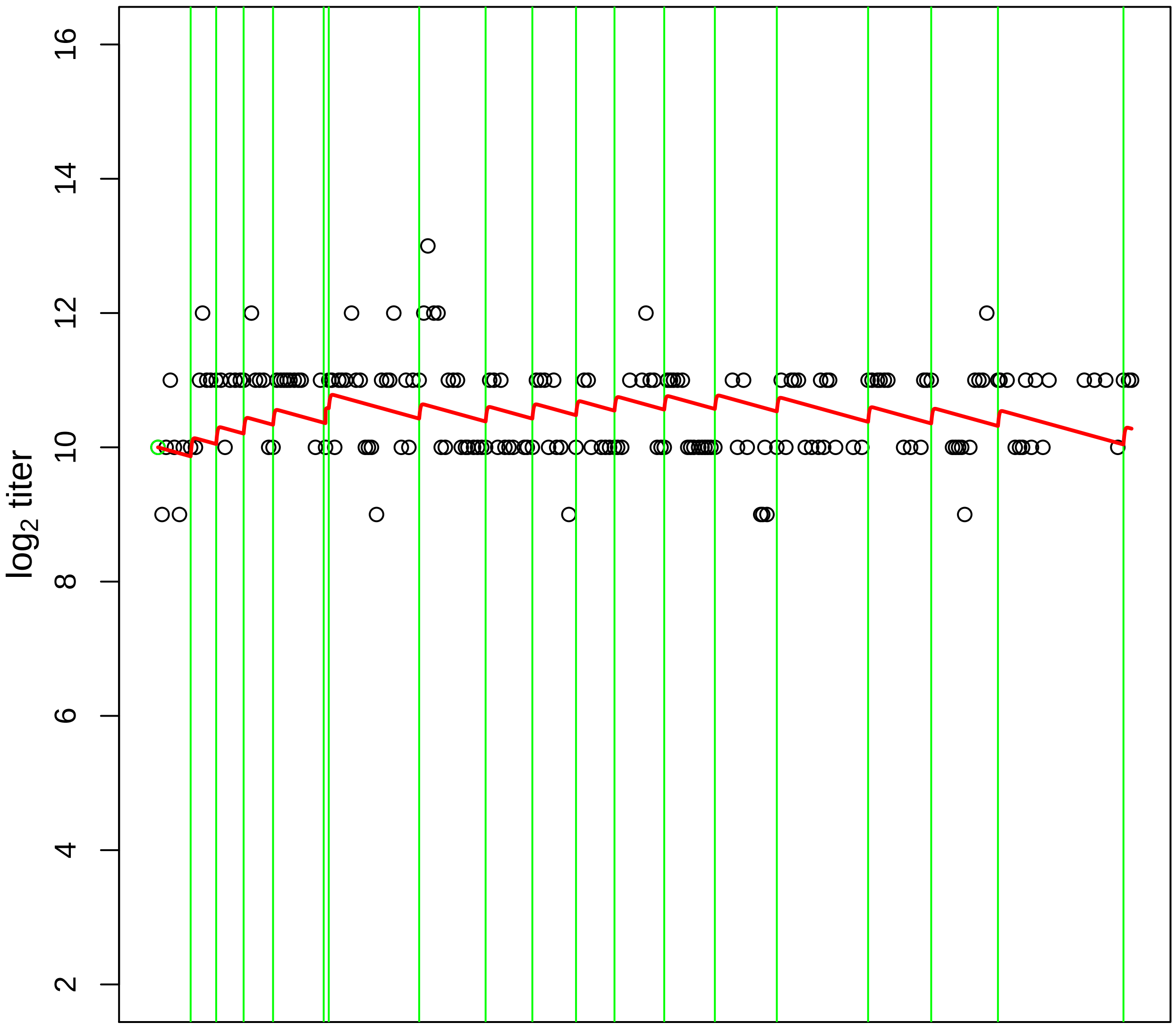

time in years from first donation of donor 412
mean absolute errors = 0.602 , mean squared errors = 0.485

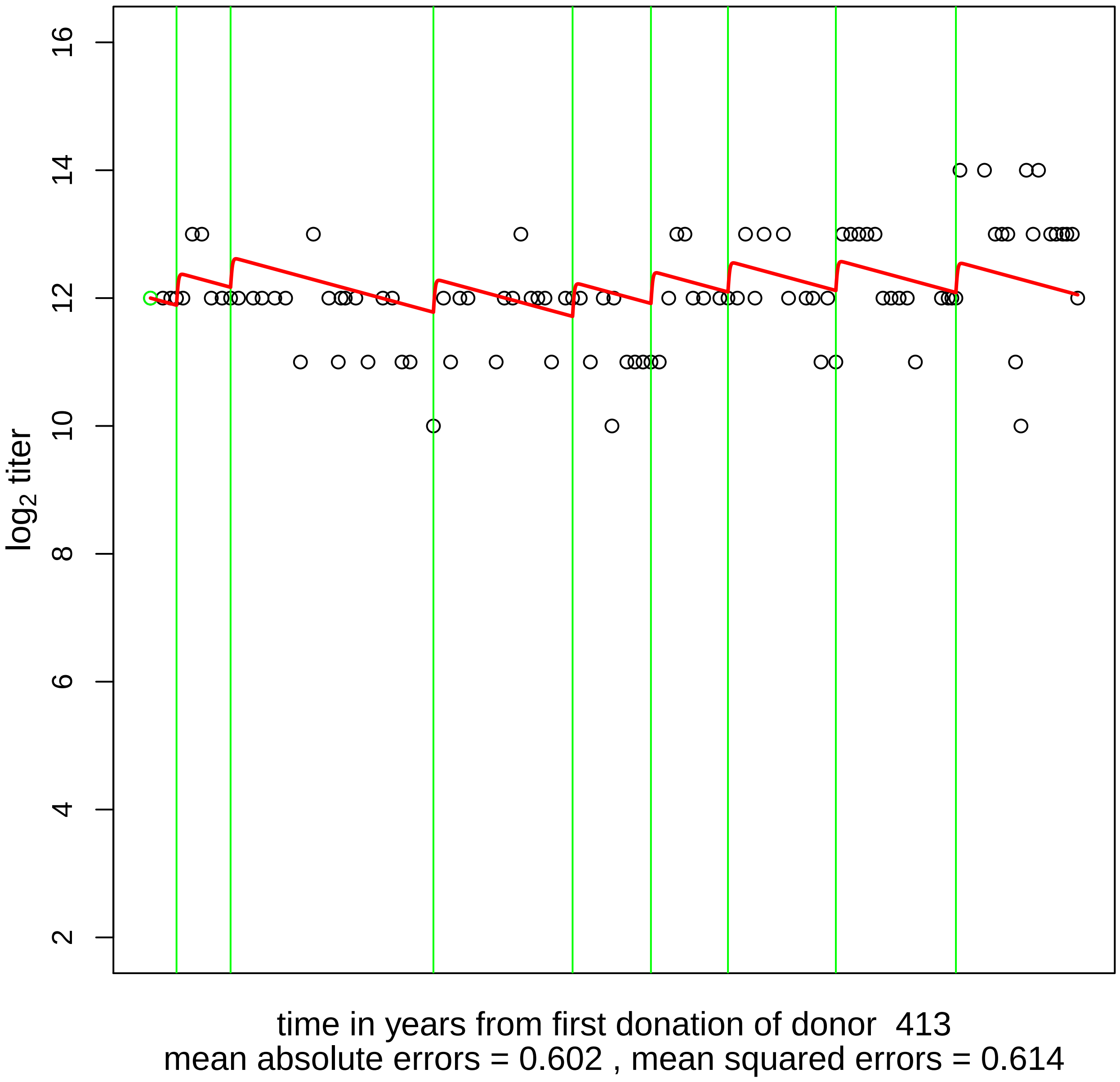

16
14
12
10
8
6
4
2
$\log_2$ titer
time in years from first donation of donor 413
mean absolute errors = 0.602 , mean squared errors = 0.614

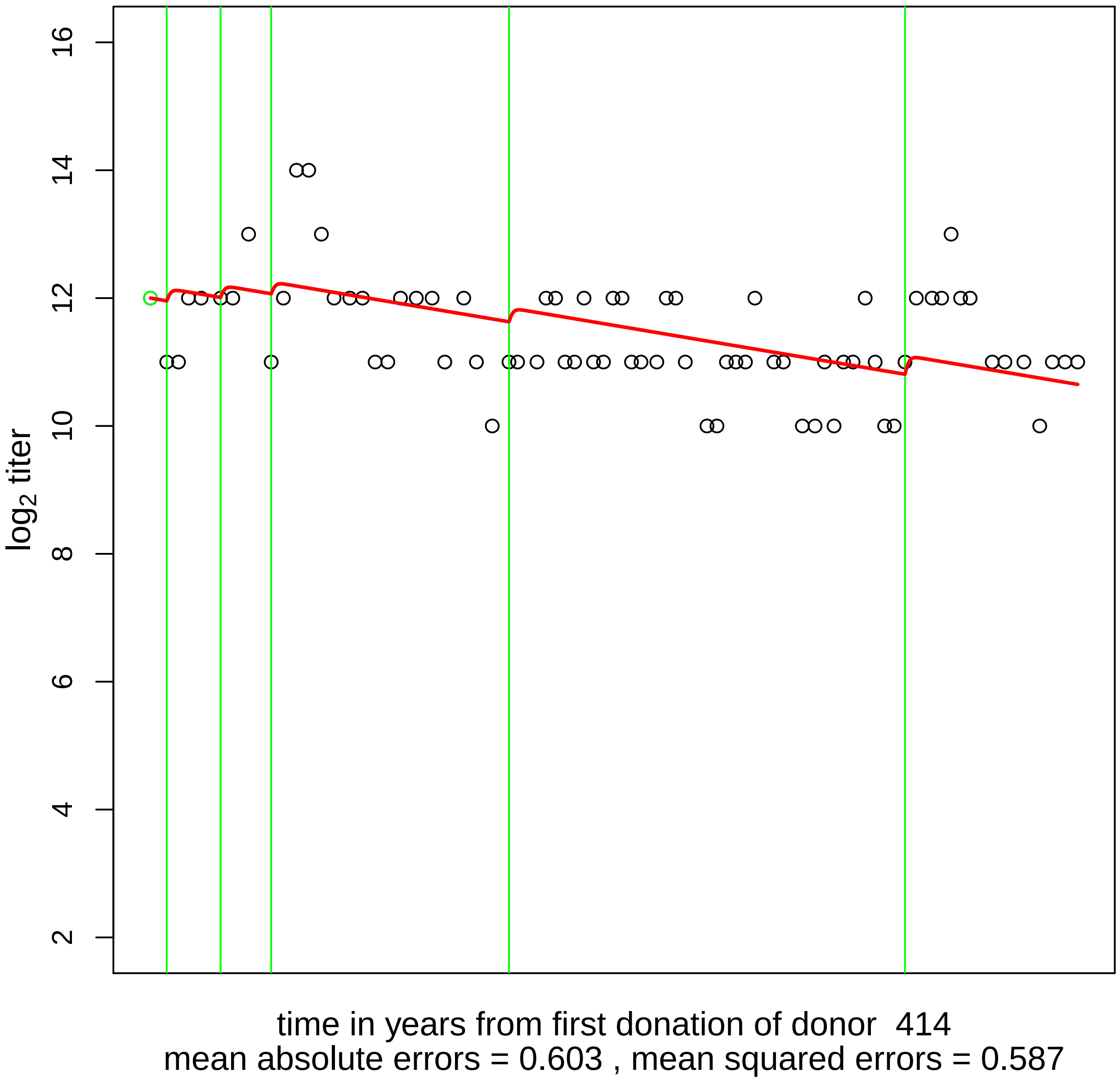

16
14
12
10
8
6
4
2
$\log_2$ titer
time in years from first donation of donor 414
mean absolute errors = 0.603 , mean squared errors = 0.587

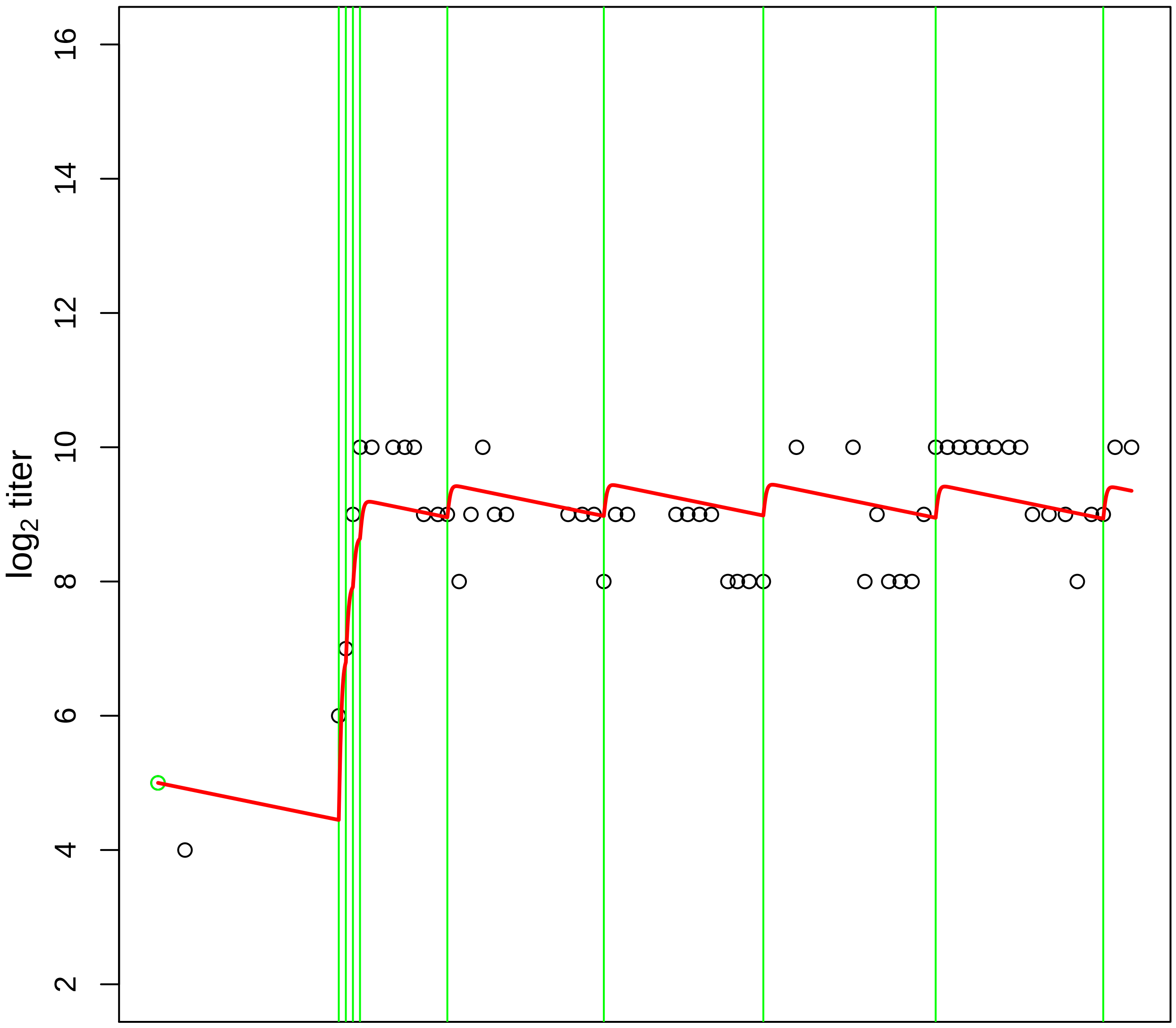

time in years from first donation of donor 415
mean absolute errors = 0.603 , mean squared errors = 0.549

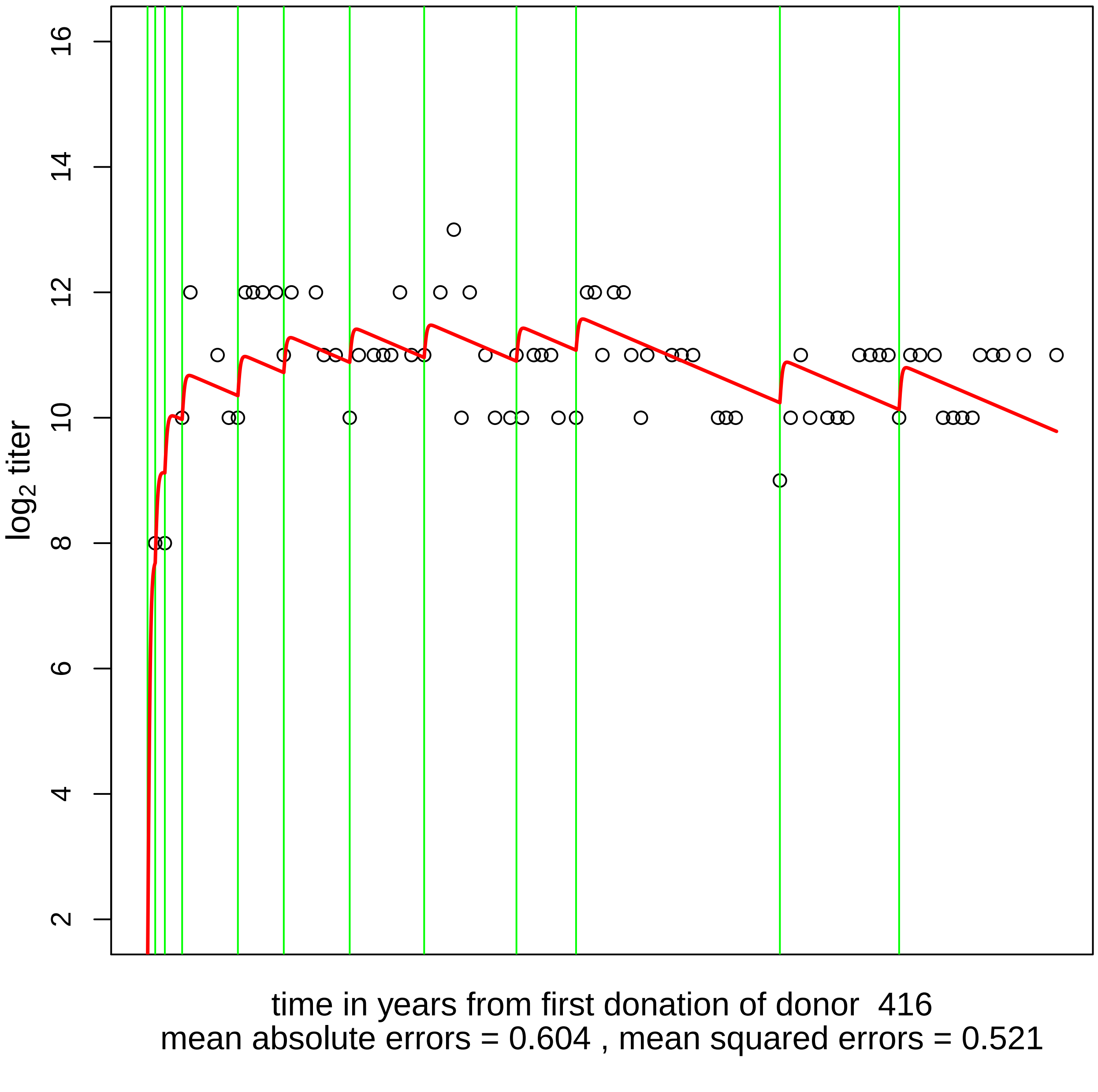

log₂ titer
2
4
6
8
10
12
14
16
time in years from first donation of donor 416
mean absolute errors = 0.604 , mean squared errors = 0.521

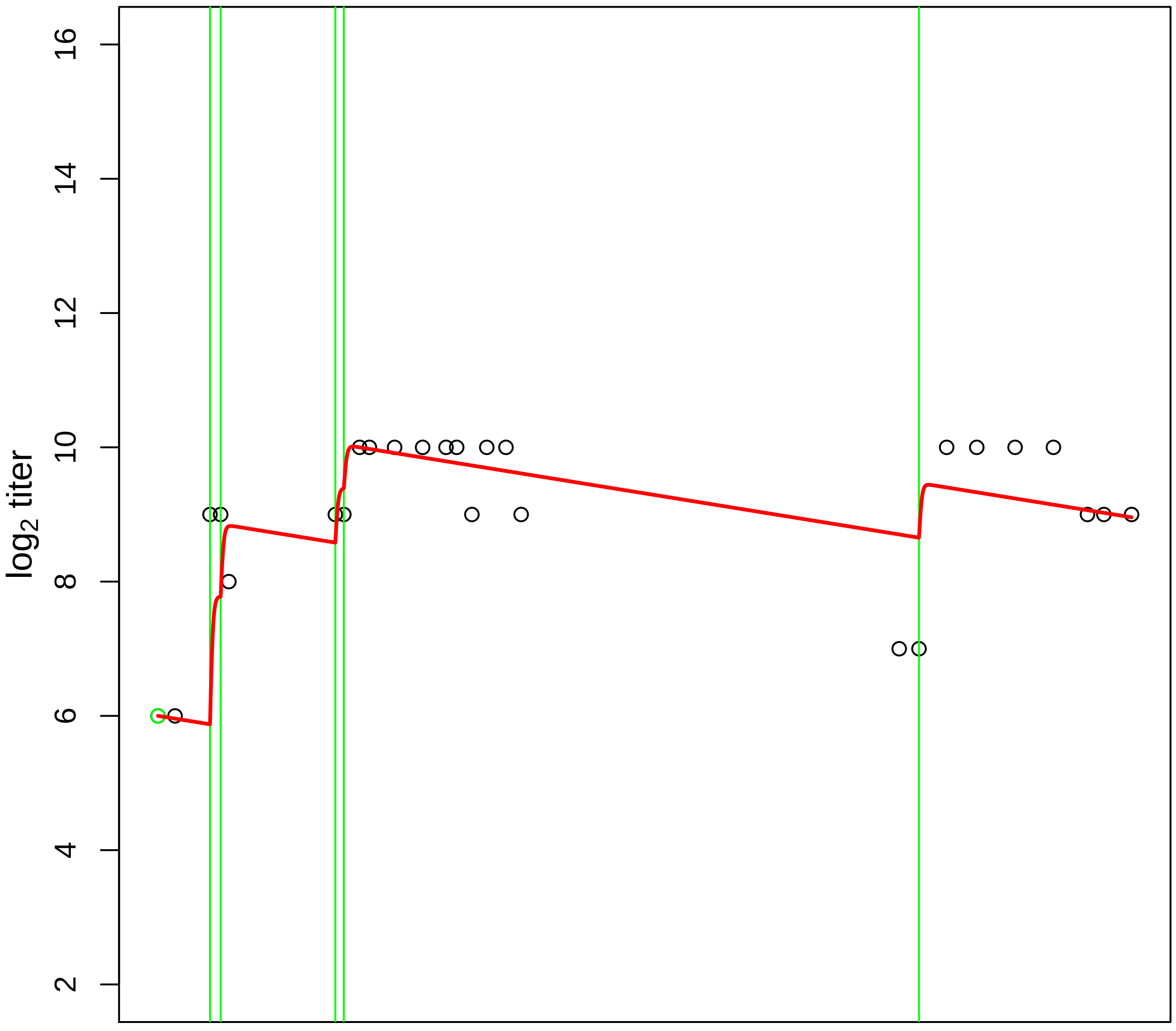

time in years from first donation of donor 417
mean absolute errors = 0.604 , mean squared errors = 0.851

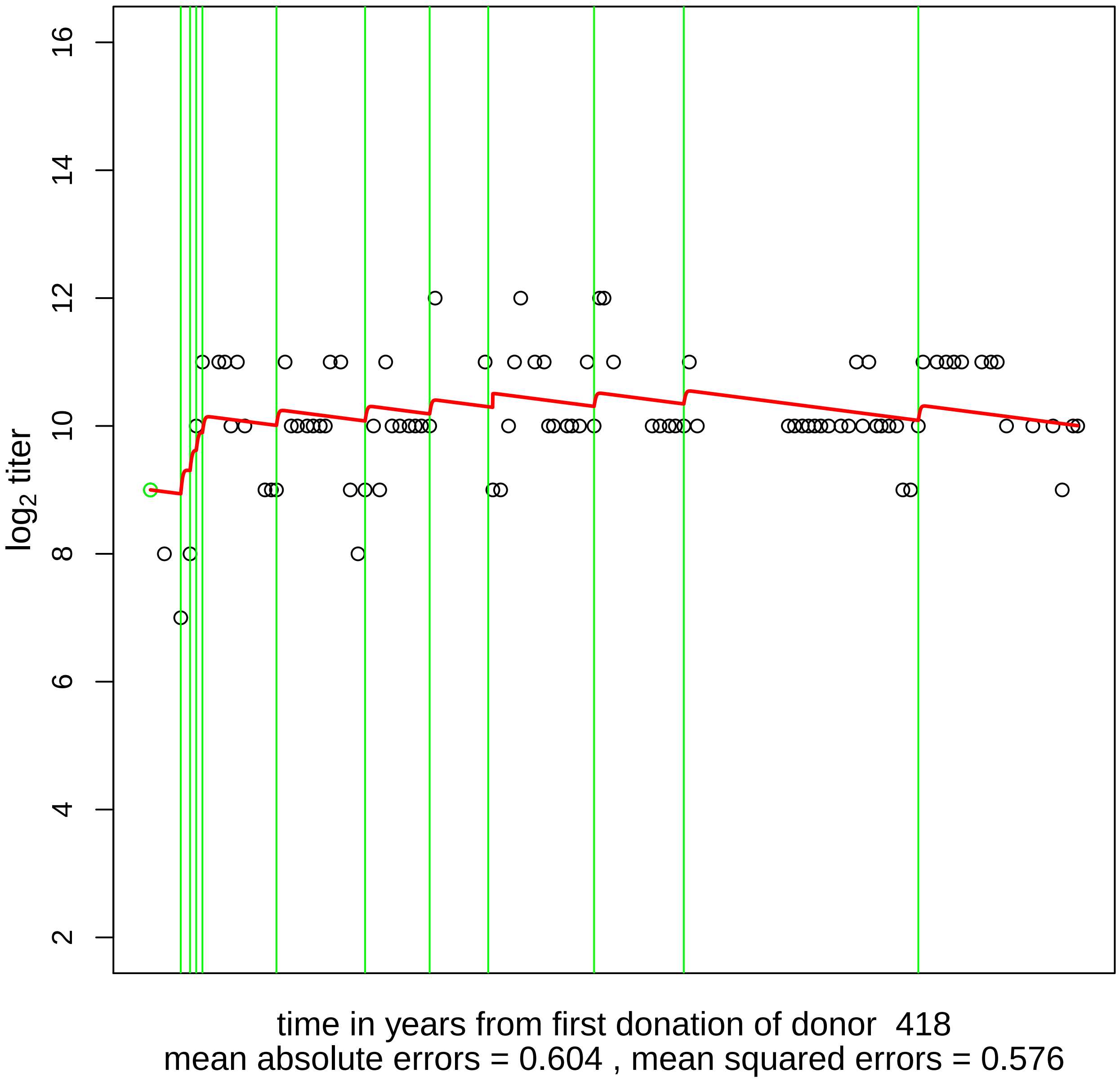

16
14
12
10
8
6
4
2
log2 titer
time in years from first donation of donor 418
mean absolute errors = 0.604 , mean squared errors = 0.576

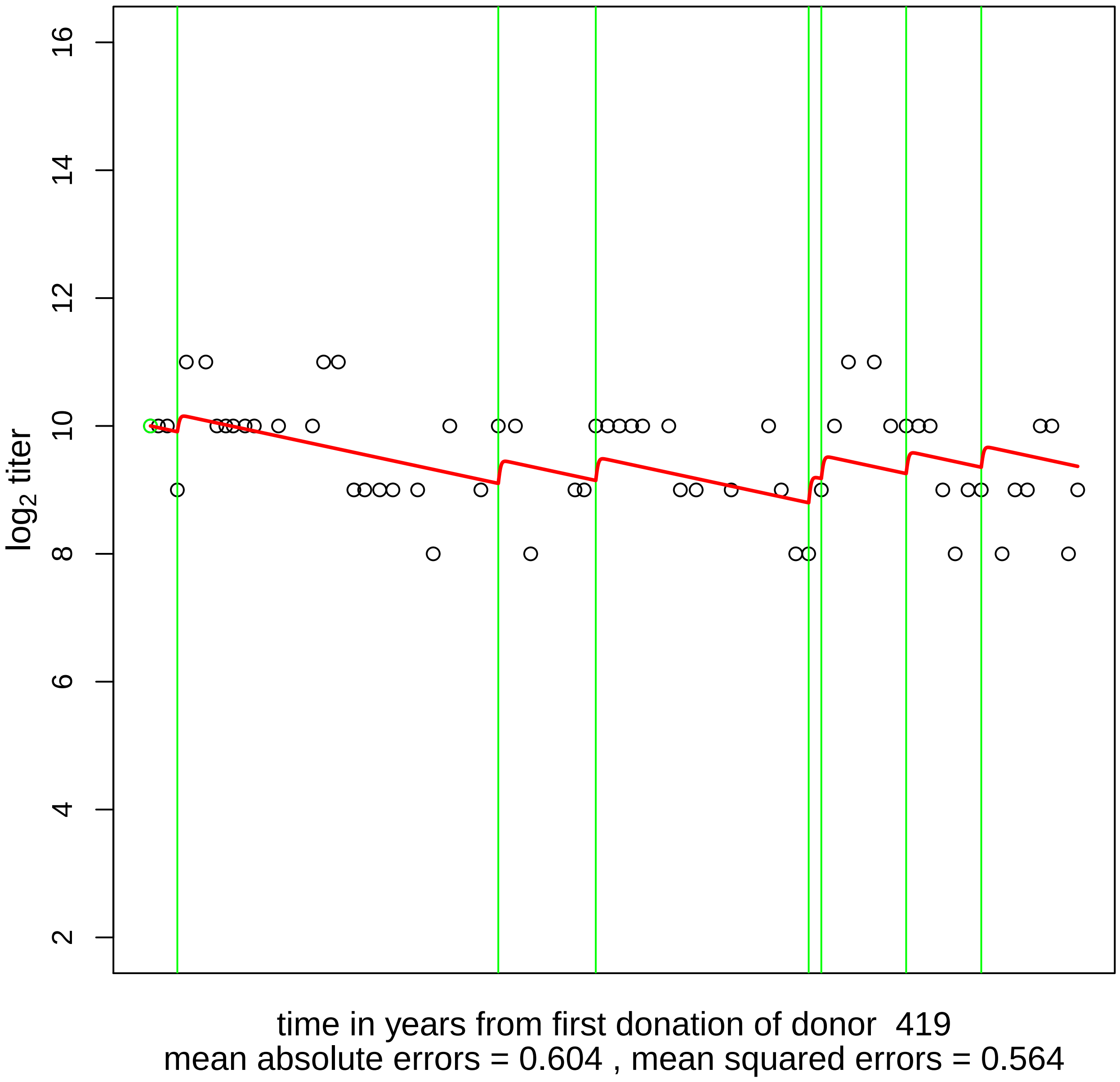

16
14
12
10
8
6
4
2
$\log_2$ titer
time in years from first donation of donor 419
mean absolute errors = 0.604 , mean squared errors = 0.564

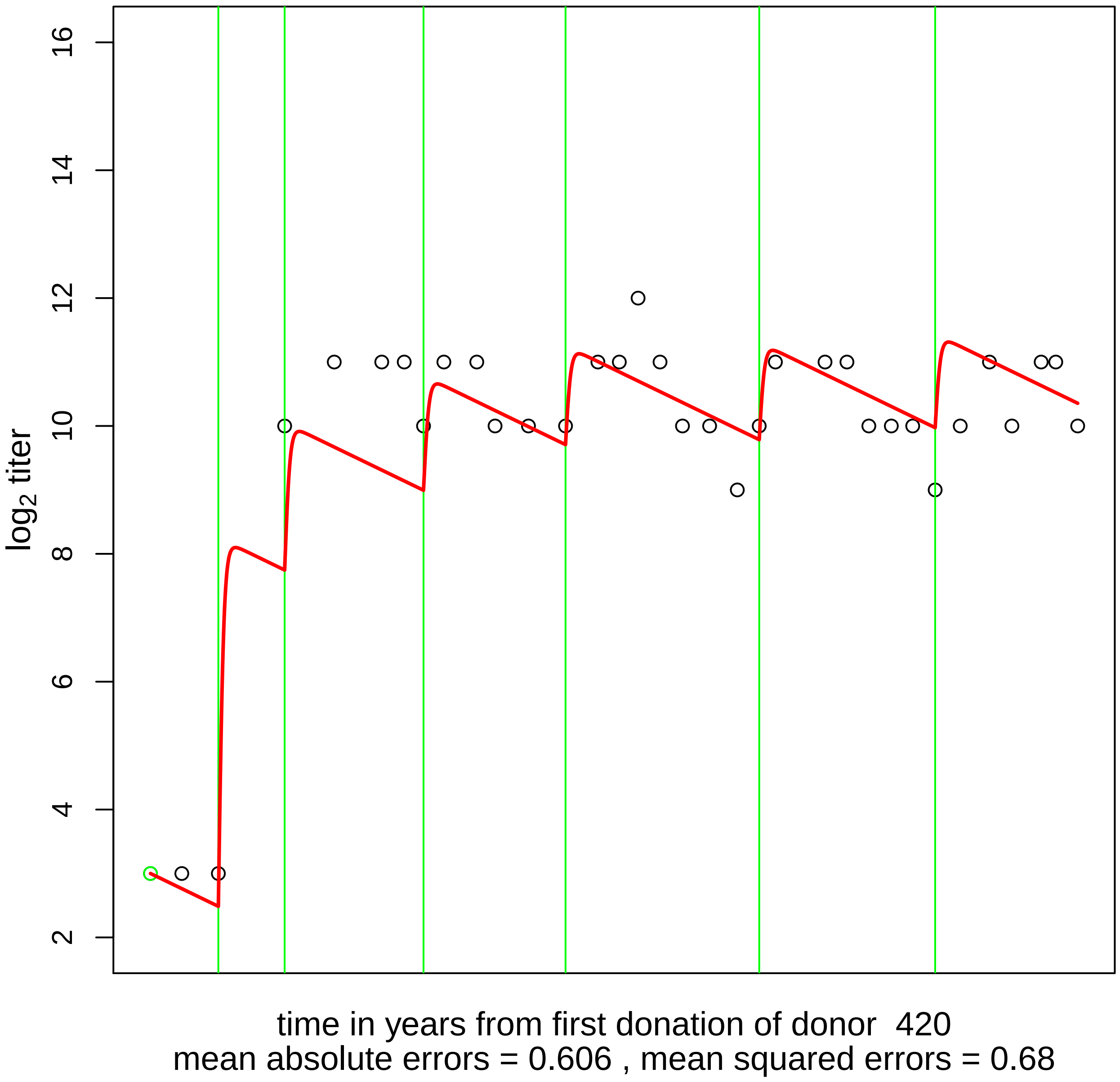

$\log_2$ titer
2
4
6
8
10
12
14
16
time in years from first donation of donor 420
mean absolute errors = 0.606 , mean squared errors = 0.68

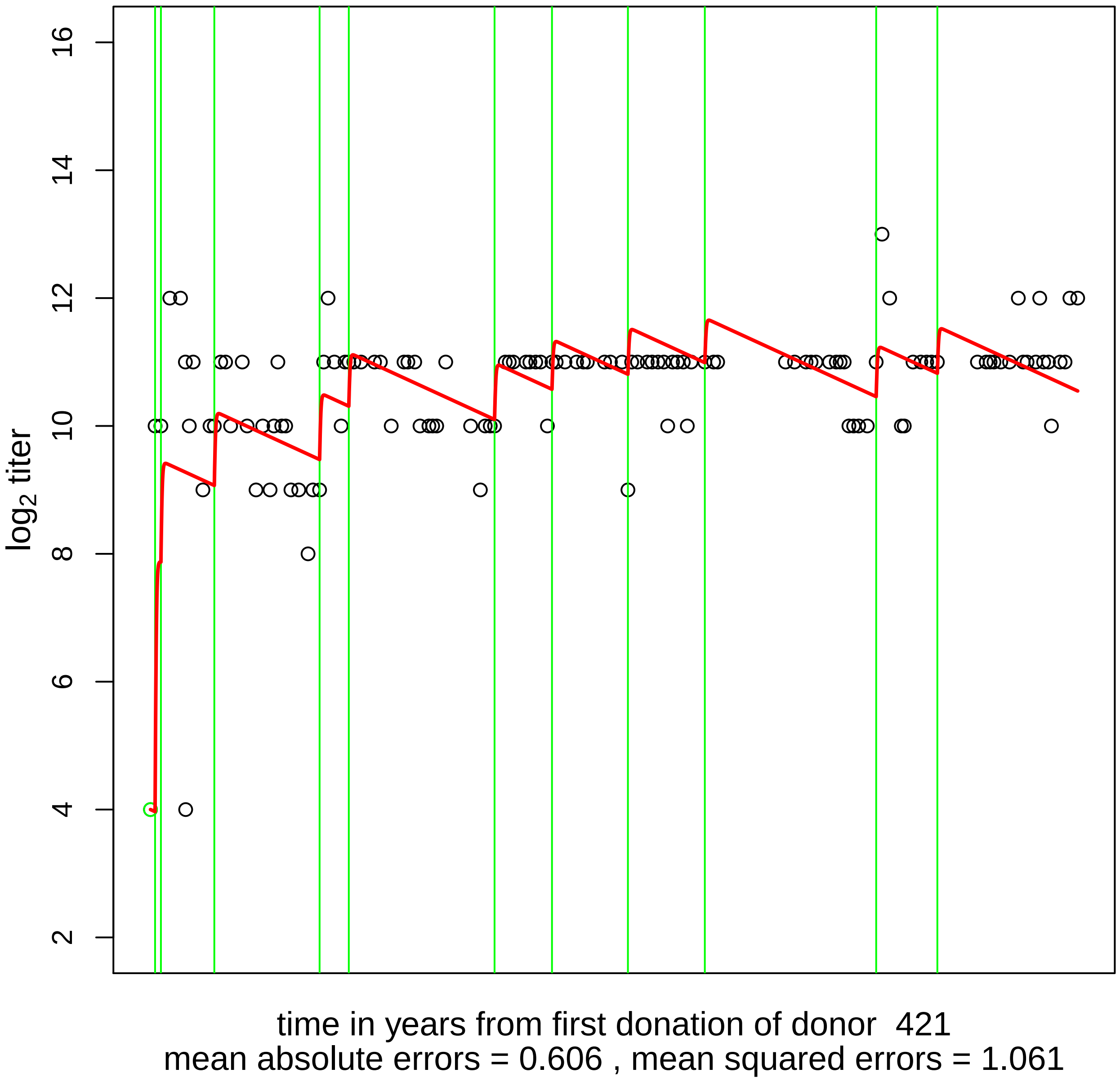

log2 titer
2
4
6
8
10
12
14
16
time in years from first donation of donor 421
mean absolute errors = 0.606 , mean squared errors = 1.061

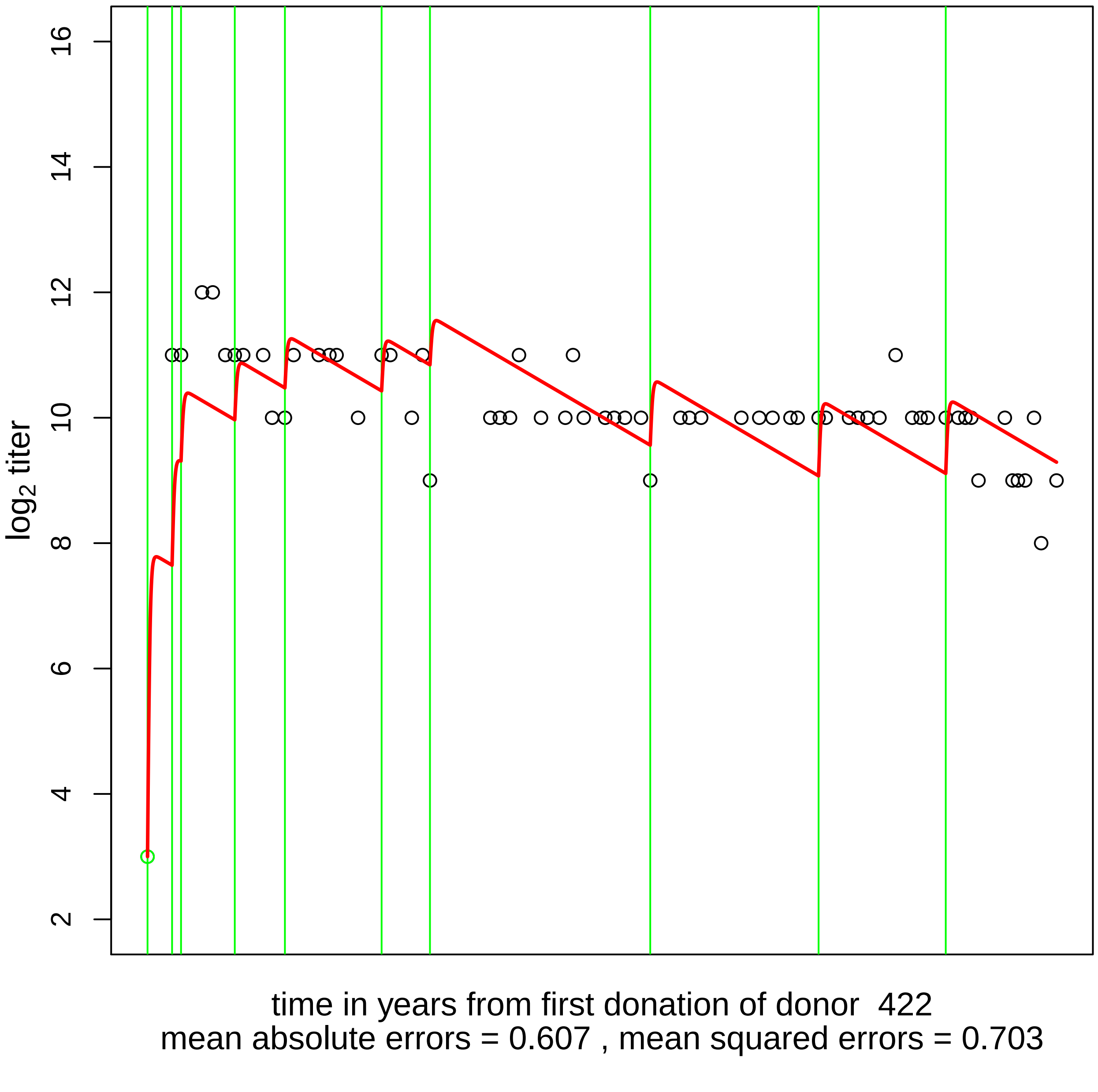
16
14
12
10
8
6
4
2
log2 titer
time in years from first donation of donor 422
mean absolute errors = 0.607 , mean squared errors = 0.703

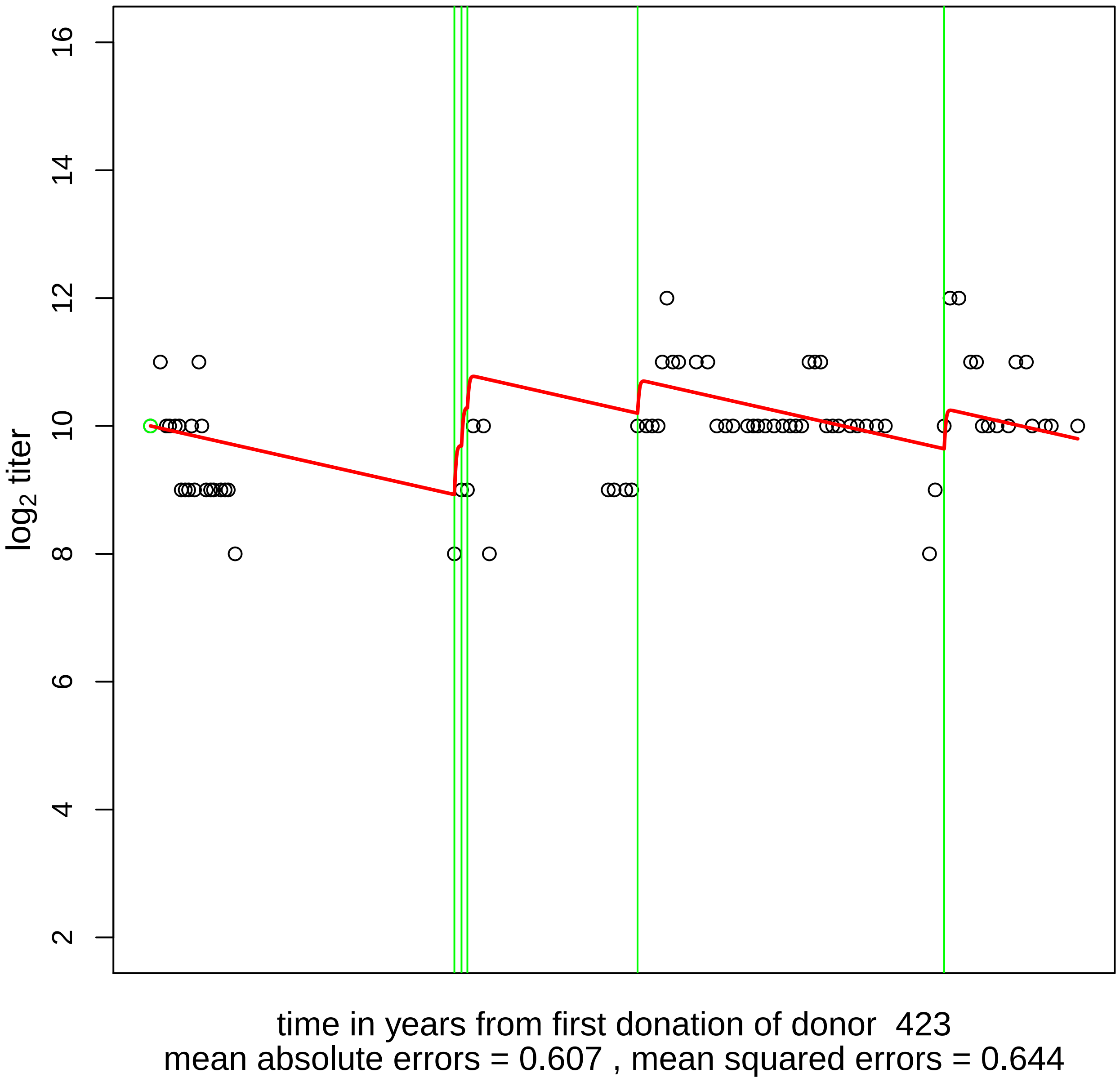

16
14
12
10
8
6
4
2
$\log_2$ titer
time in years from first donation of donor 423
mean absolute errors = 0.607 , mean squared errors = 0.644

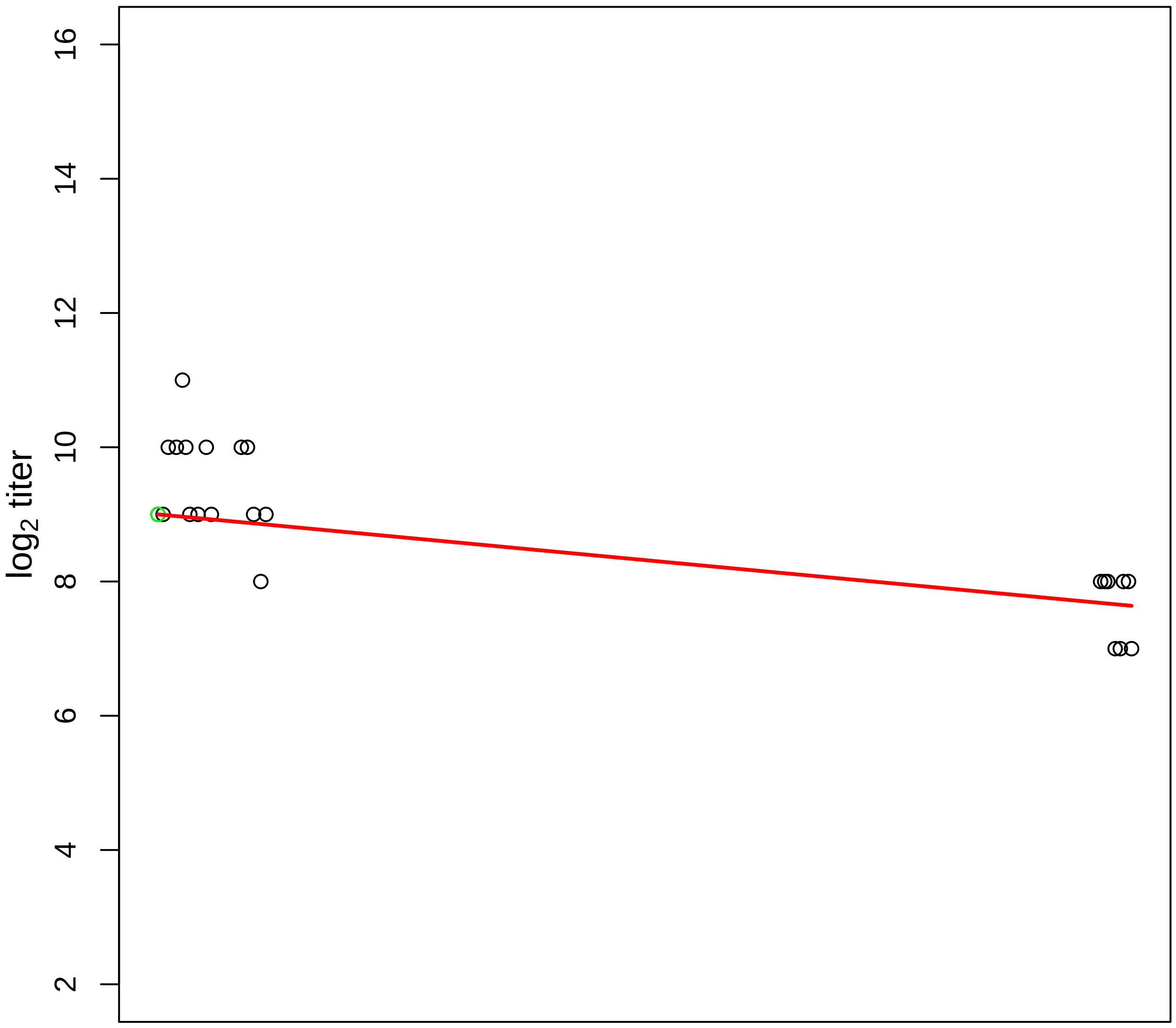

time in years from first donation of donor 424
mean absolute errors = 0.608 , mean squared errors = 0.617

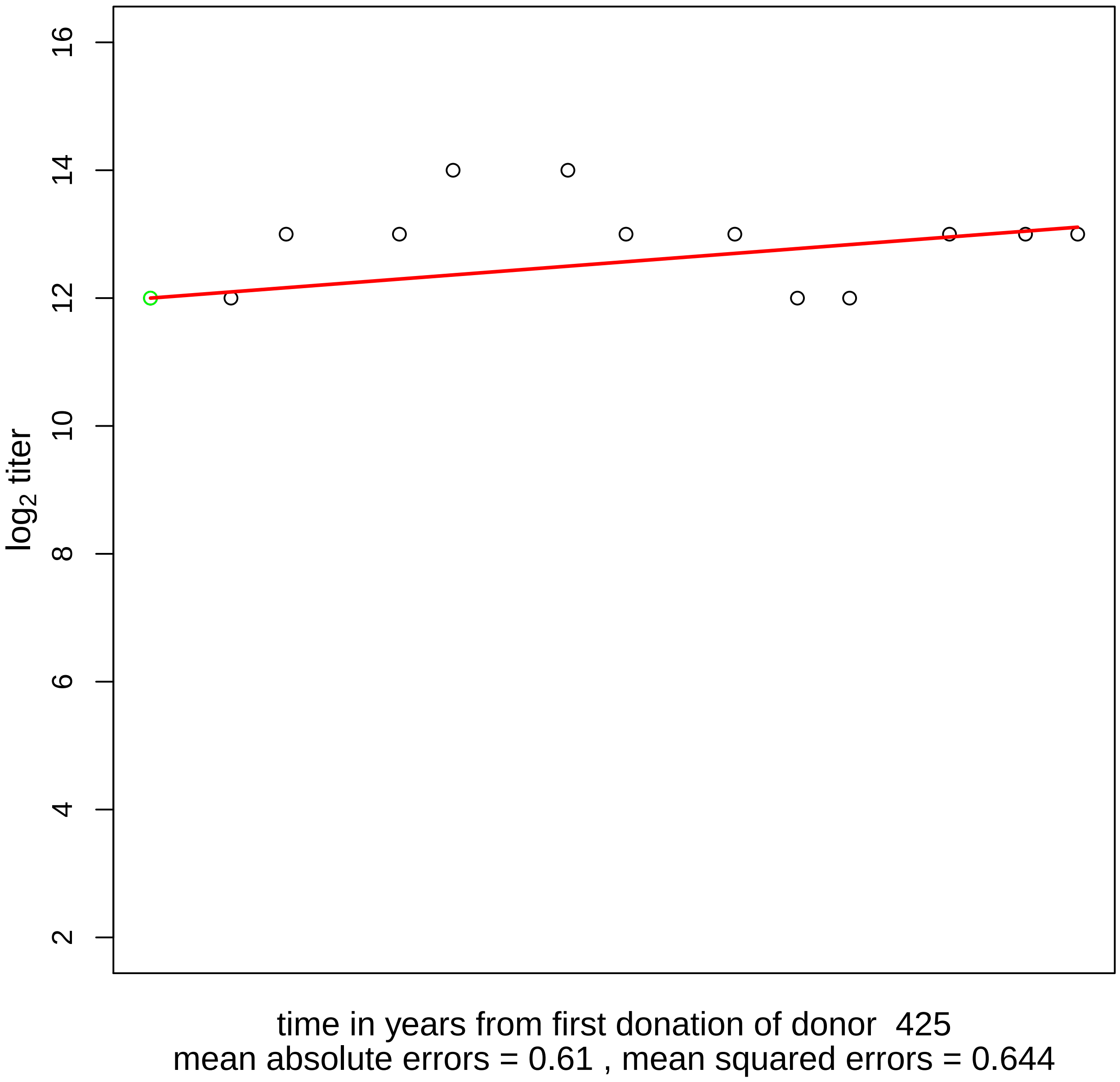

16
14
12
10
8
6
4
2
log2 titer
time in years from first donation of donor 425
mean absolute errors = 0.61 , mean squared errors = 0.644

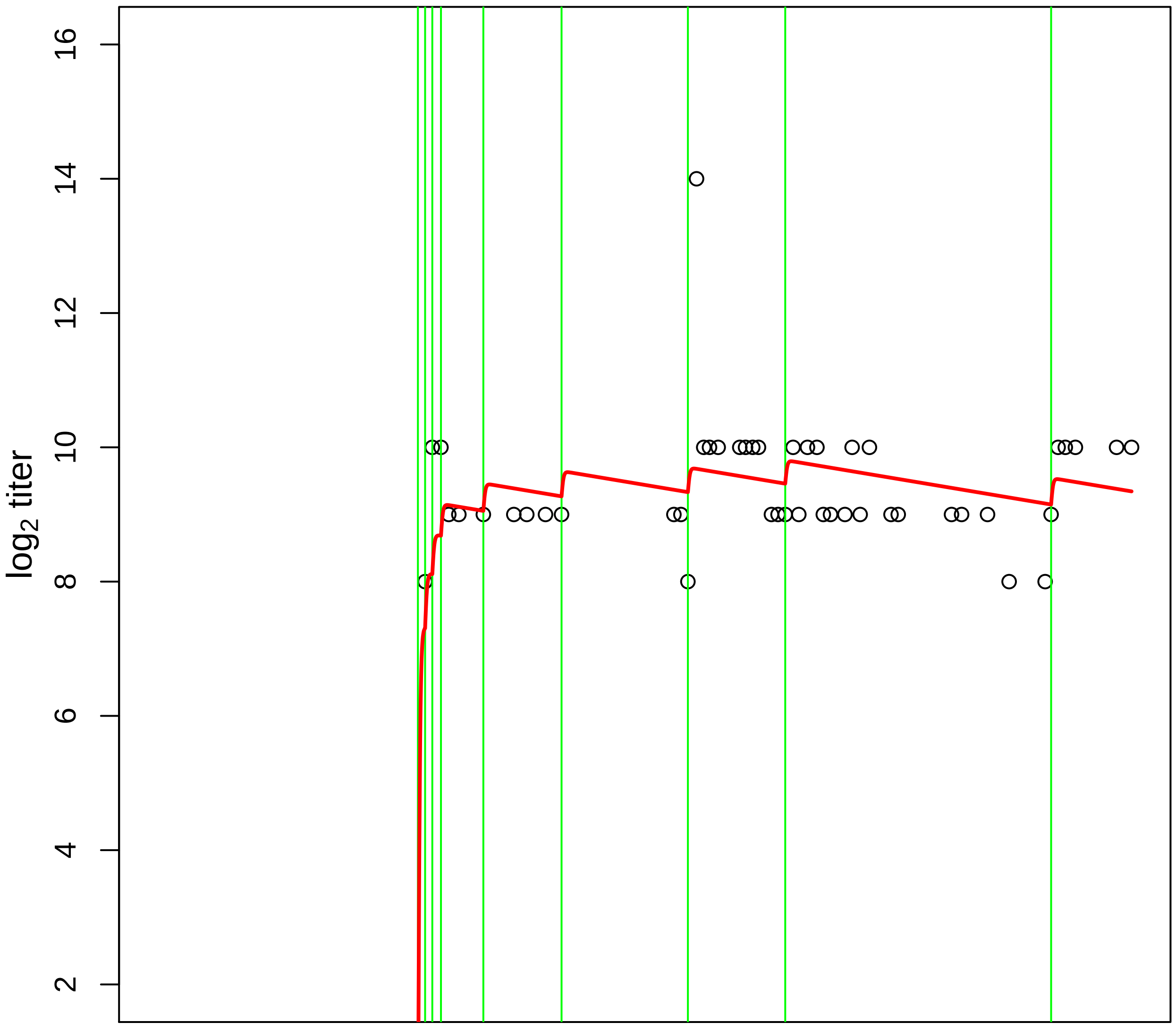

time in years from first donation of donor 426
mean absolute errors = 0.61 , mean squared errors = 0.783

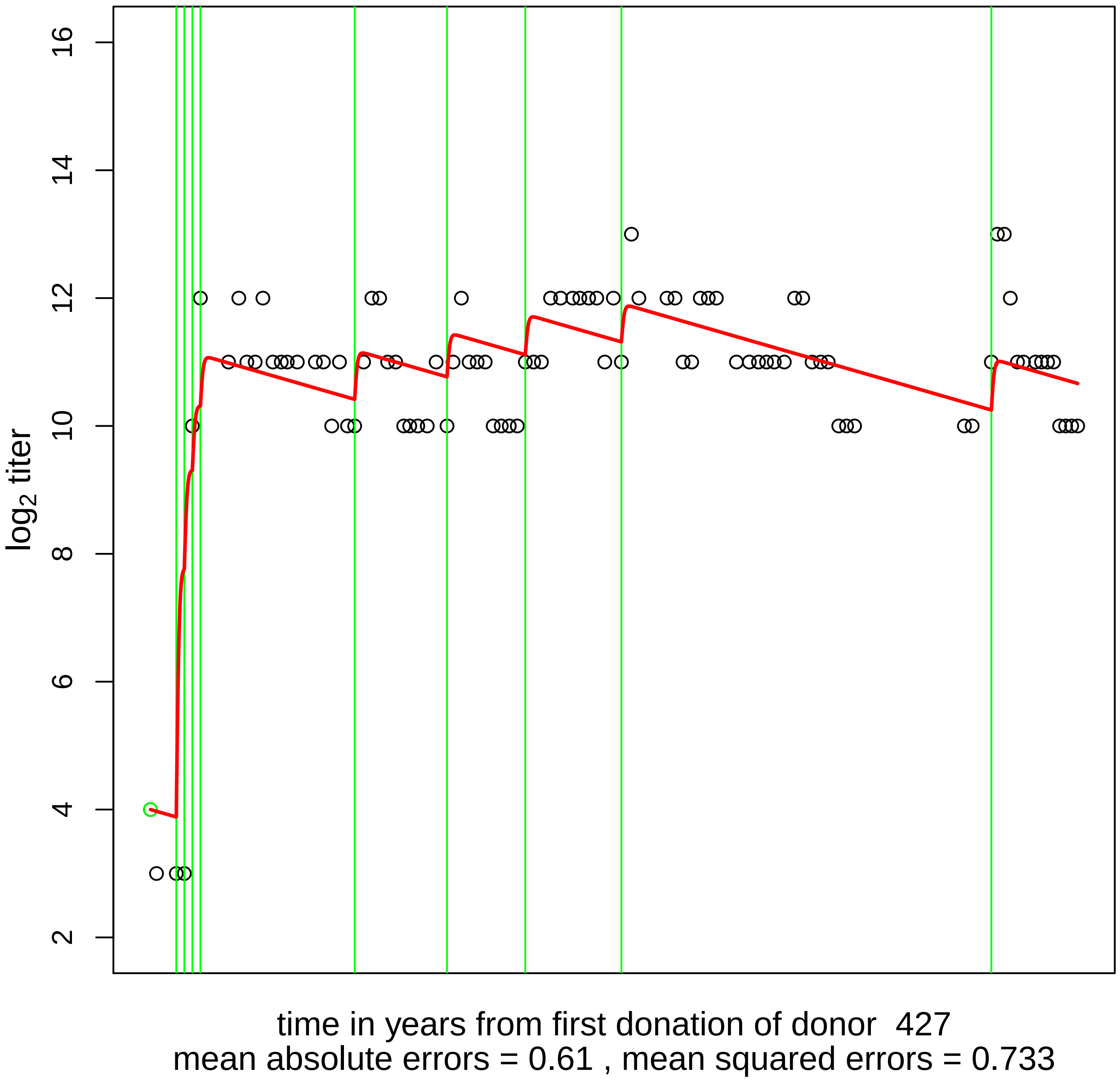

16
14
12
10
8
6
4
2
$\log_2$ titer
time in years from first donation of donor 427
mean absolute errors = 0.61 , mean squared errors = 0.733

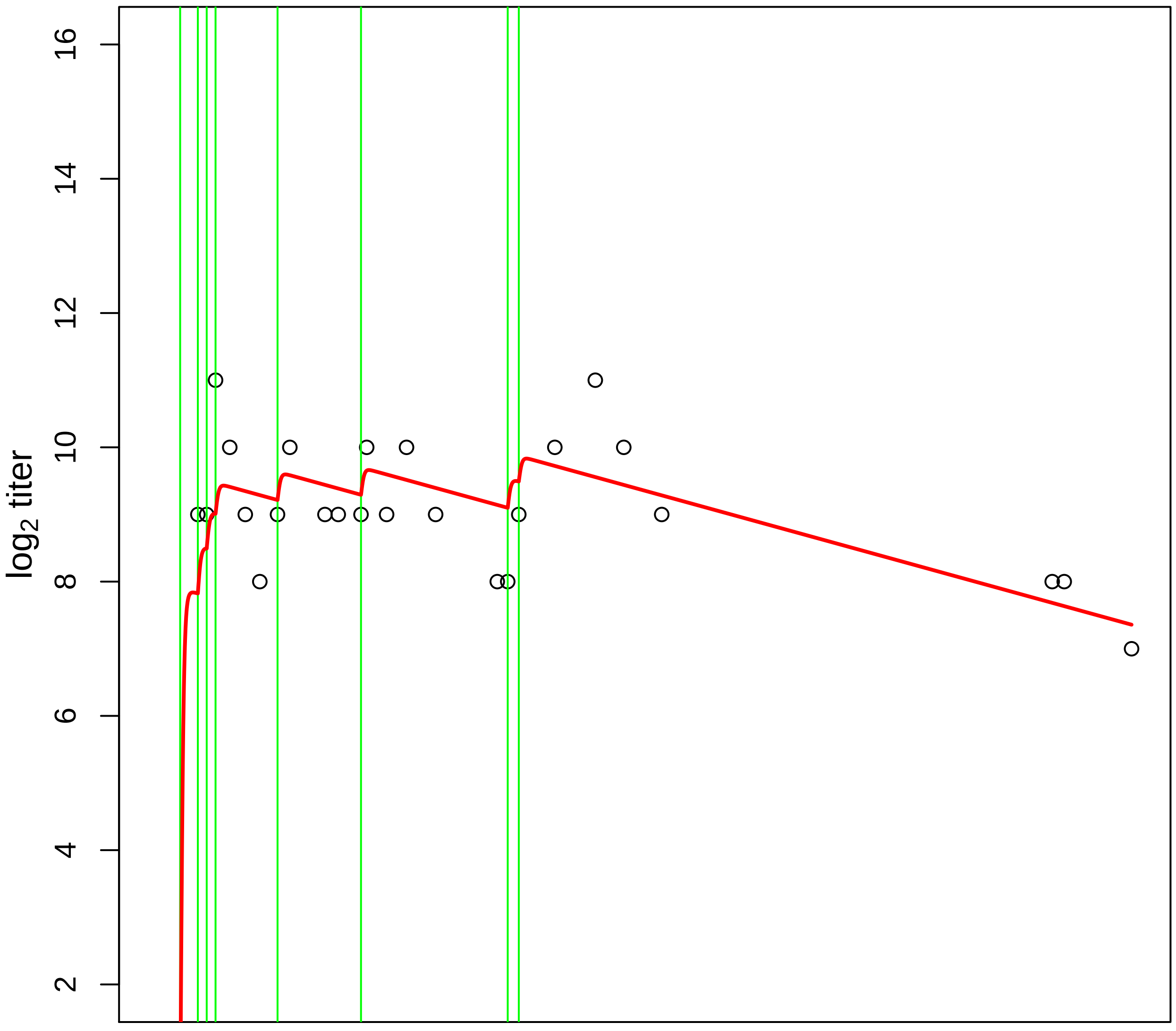

time in years from first donation of donor 428
mean absolute errors = 0.612 , mean squared errors = 0.574

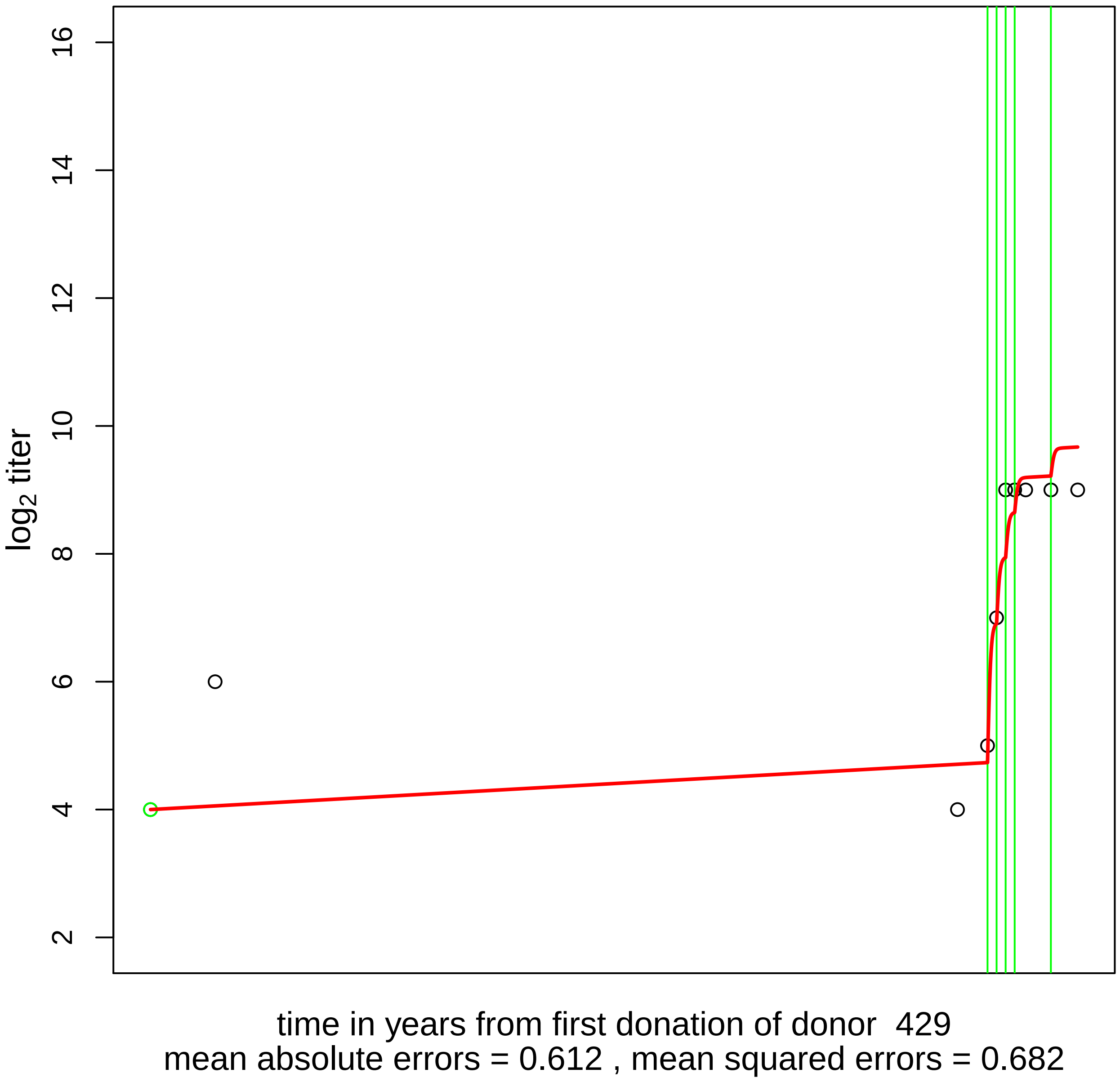

16
14
12
10
8
6
4
2
log2 titer
time in years from first donation of donor 429
mean absolute errors = 0.612 , mean squared errors = 0.682

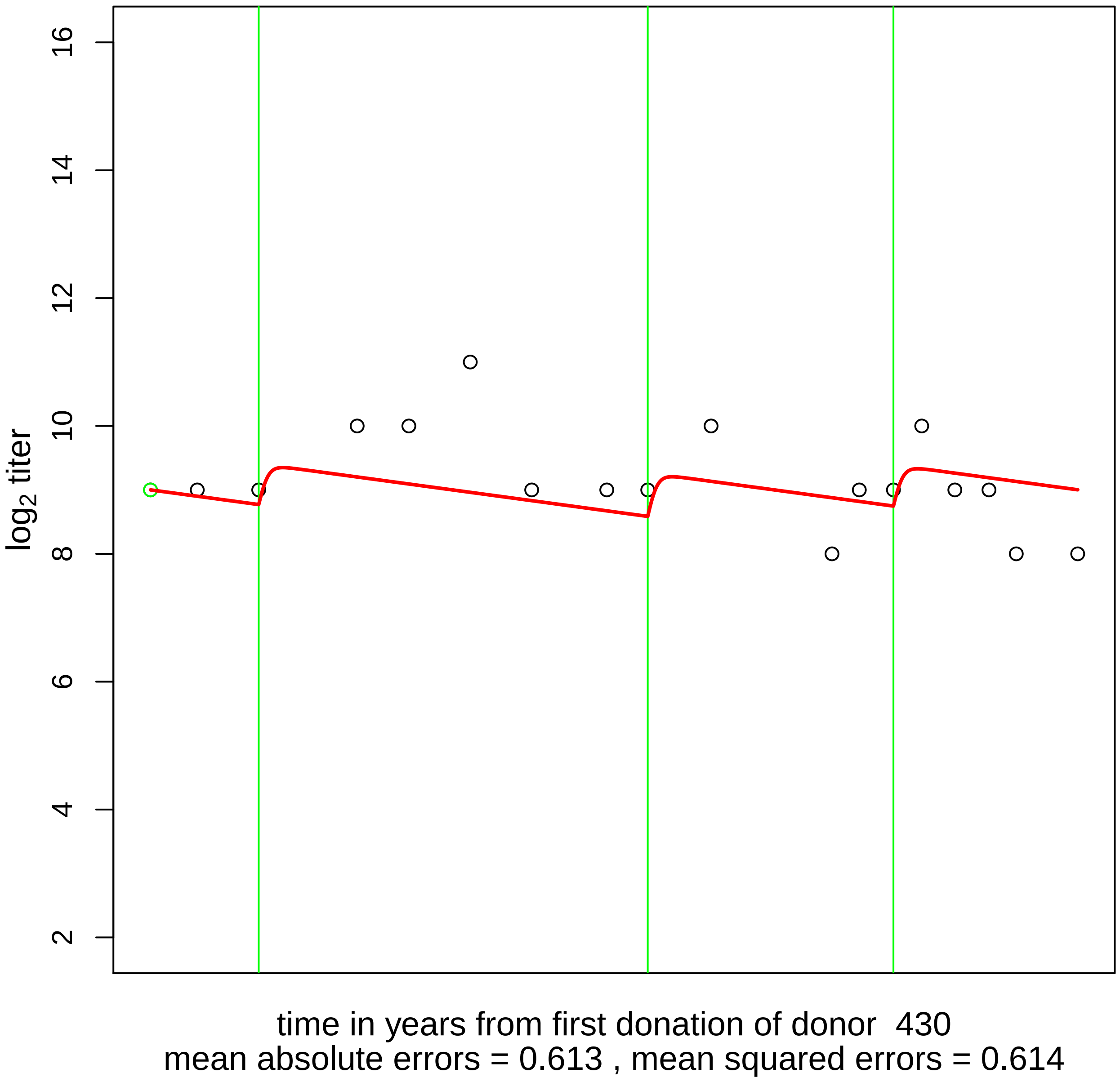

log2 titer
2
4
6
8
10
12
14
16
time in years from first donation of donor 430
mean absolute errors = 0.613 , mean squared errors = 0.614

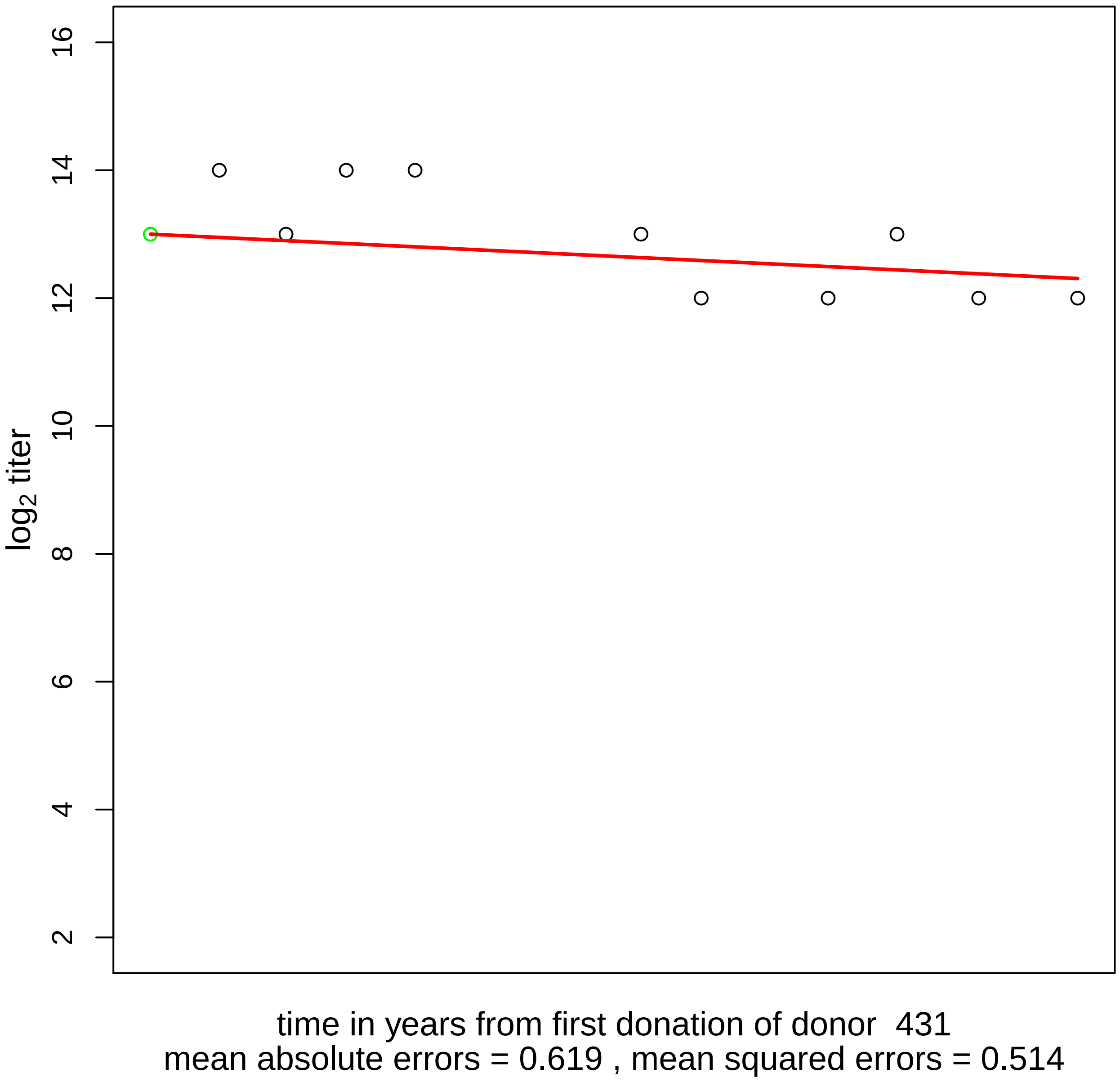

16
14
12
10
8
6
4
2
$\log_2$ titer
time in years from first donation of donor 431
mean absolute errors = 0.619 , mean squared errors = 0.514

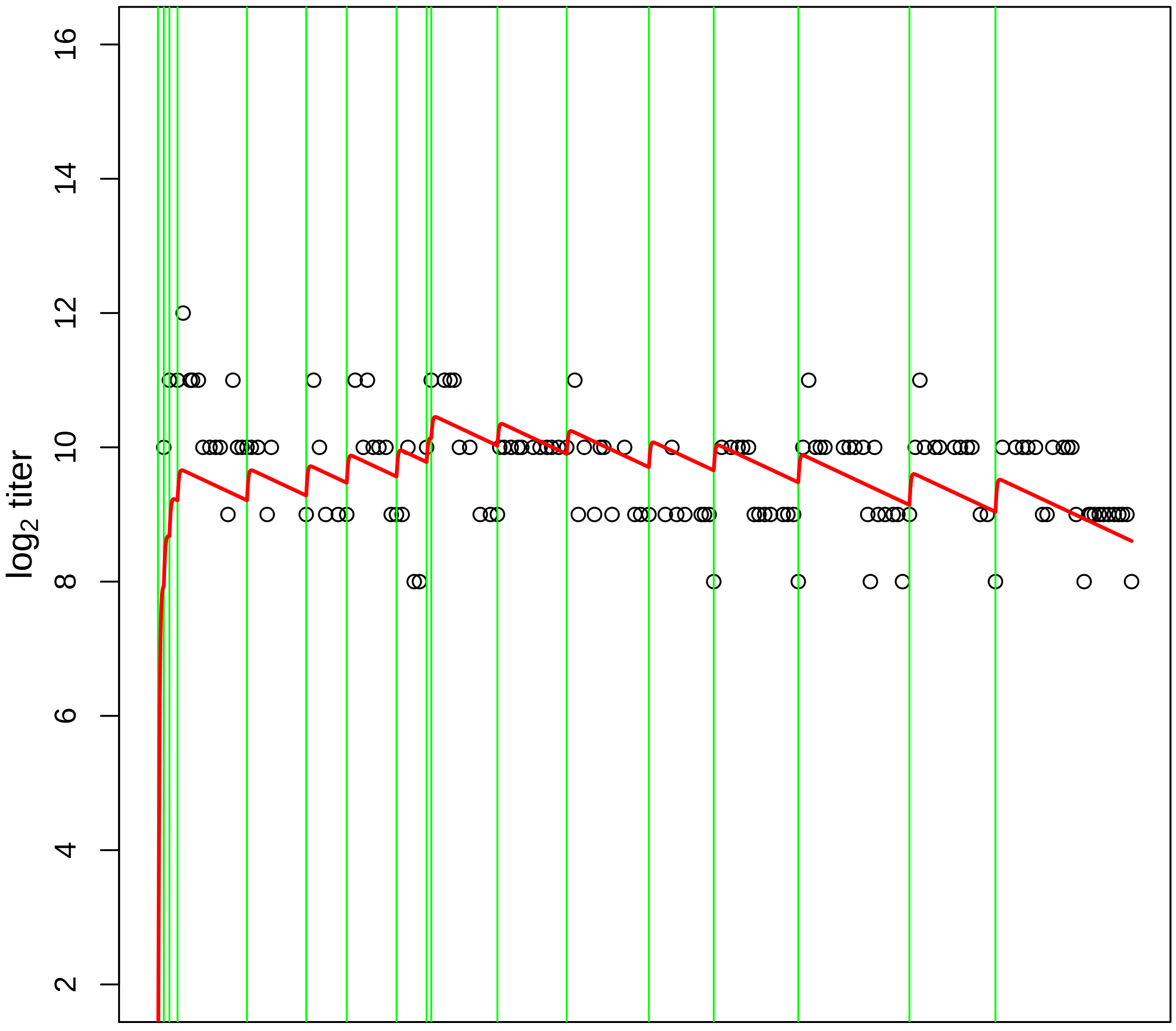

time in years from first donation of donor 432
mean absolute errors = 0.619 , mean squared errors = 0.621

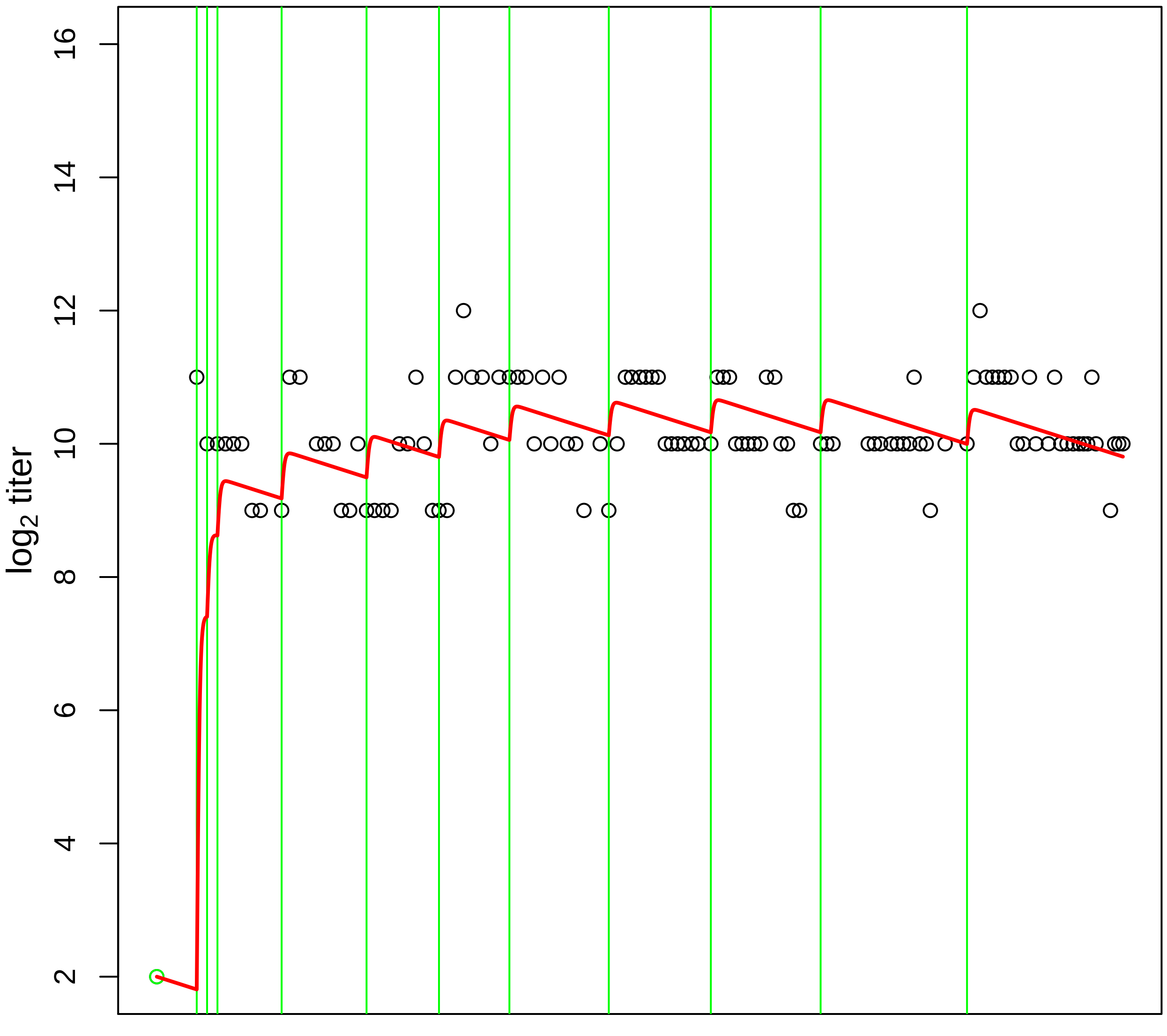

time in years from first donation of donor 433
mean absolute errors = 0.62 , mean squared errors = 1.196

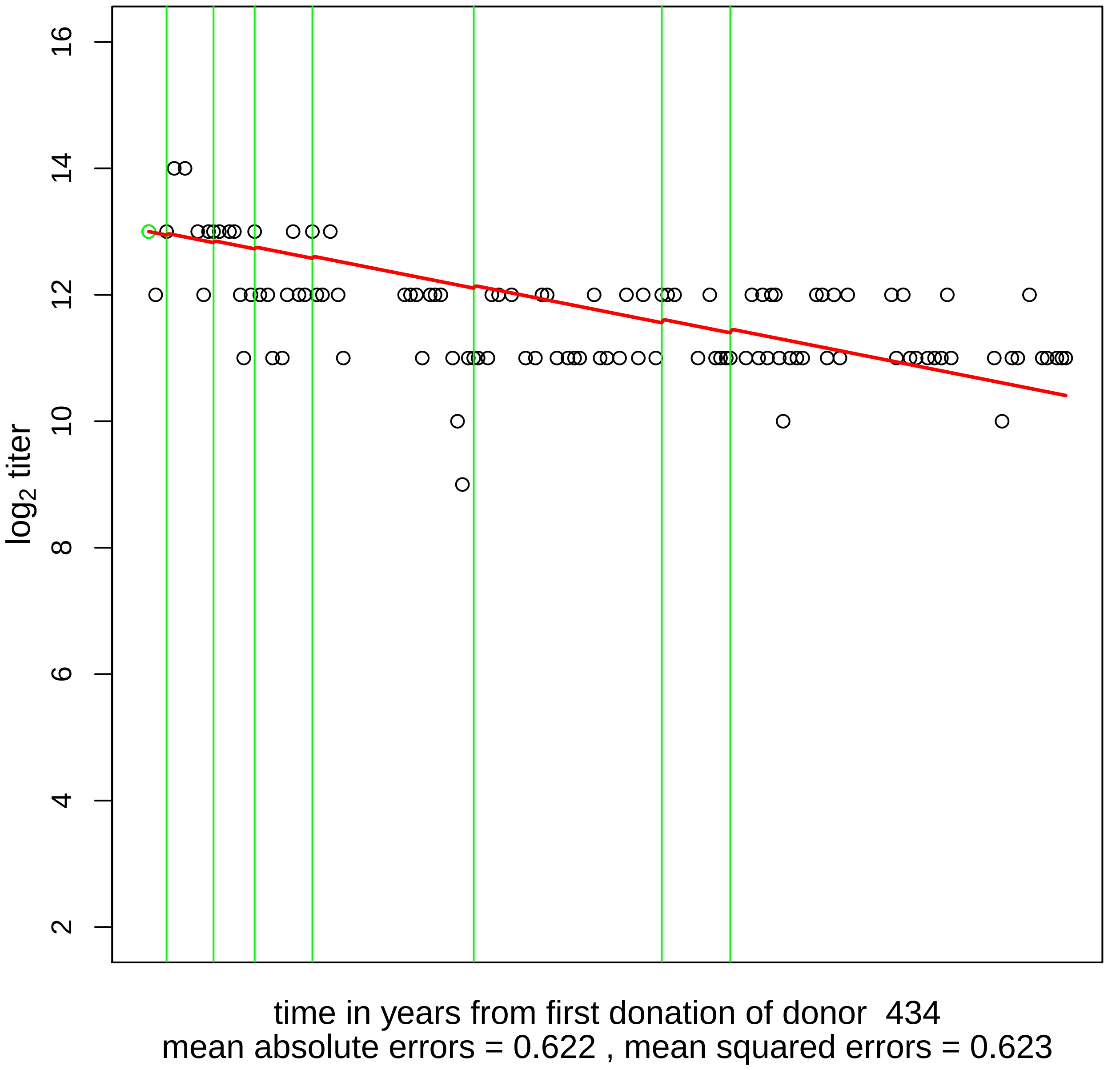

16
14
12
10
8
6
4
2
log2 titer
time in years from first donation of donor 434
mean absolute errors = 0.622 , mean squared errors = 0.623

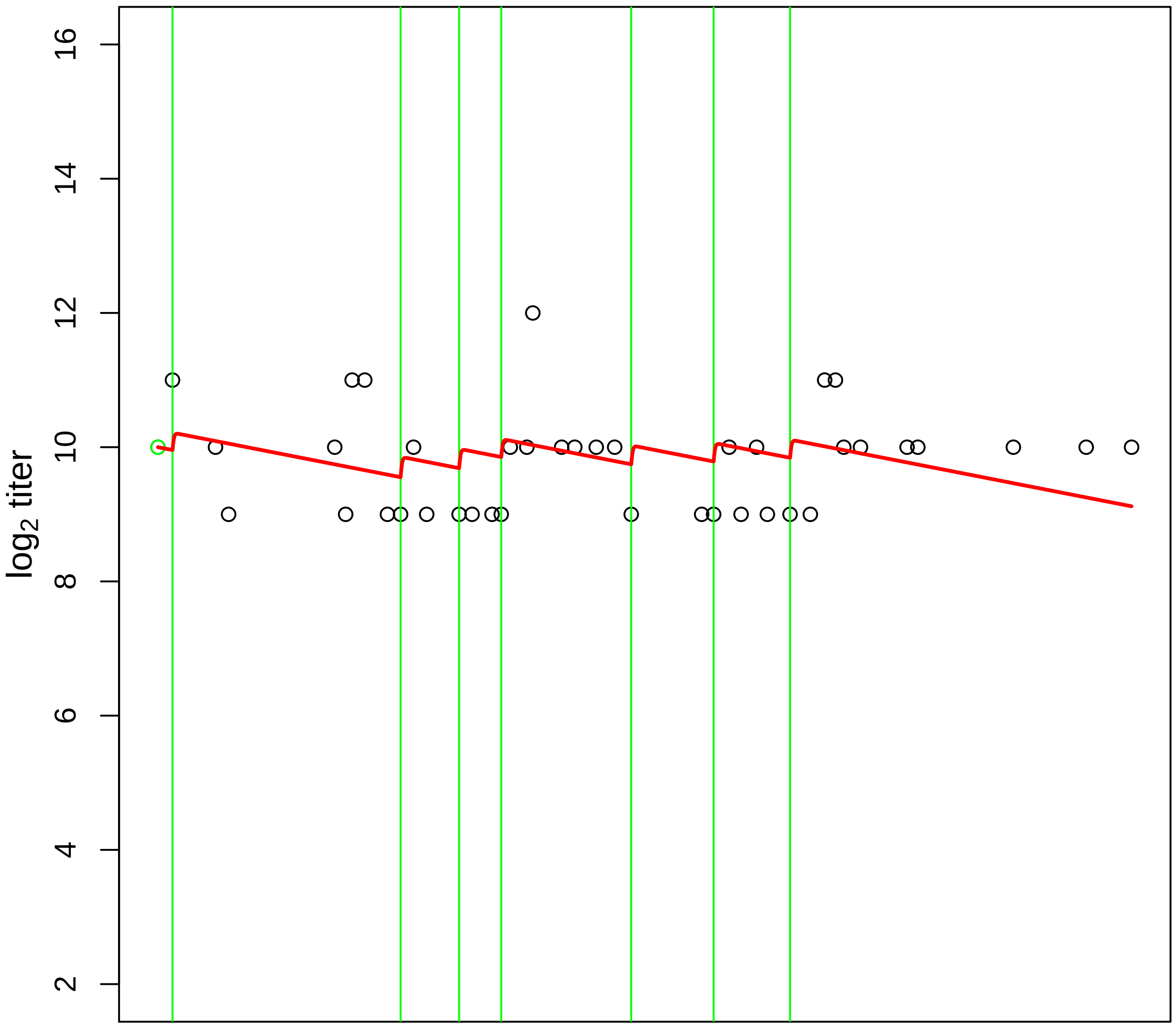

time in years from first donation of donor 435
mean absolute errors = 0.623 , mean squared errors = 0.591

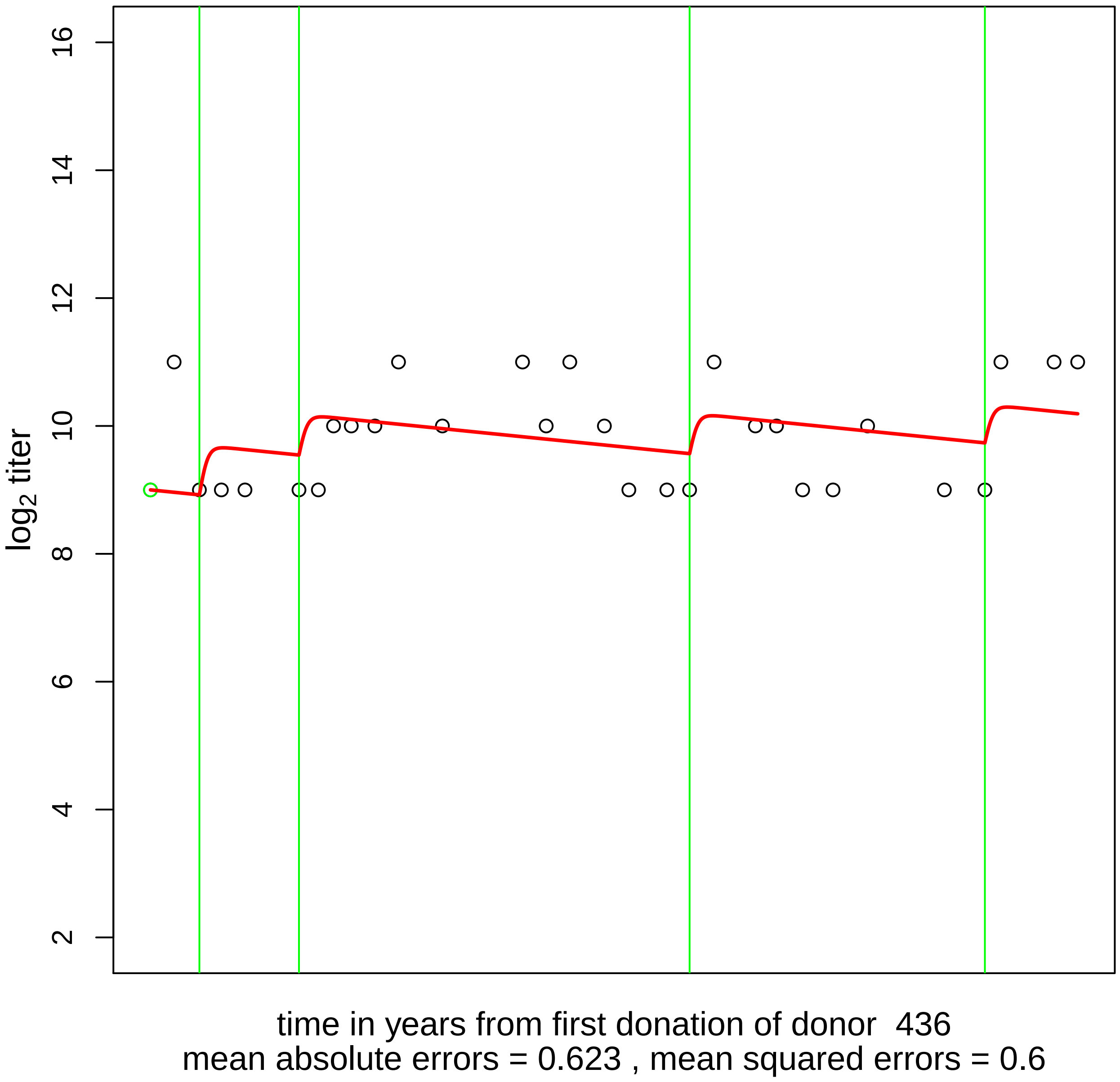

$\log_2$ titer
2
4
6
8
10
12
14
16
time in years from first donation of donor 436
mean absolute errors = 0.623 , mean squared errors = 0.6

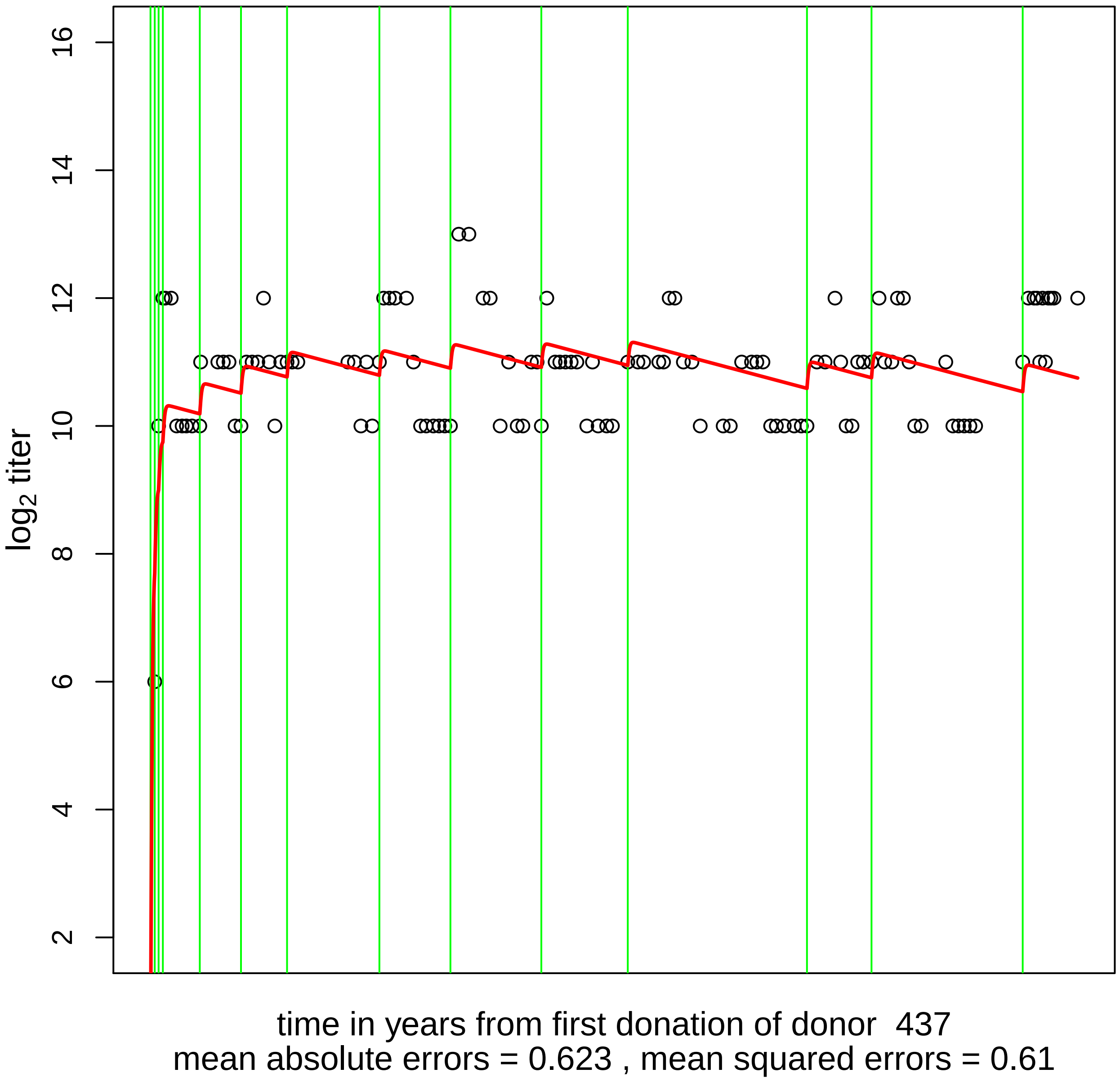

$\log_2$ titer
2
4
6
8
10
12
14
16
time in years from first donation of donor 437
mean absolute errors = 0.623 , mean squared errors = 0.61

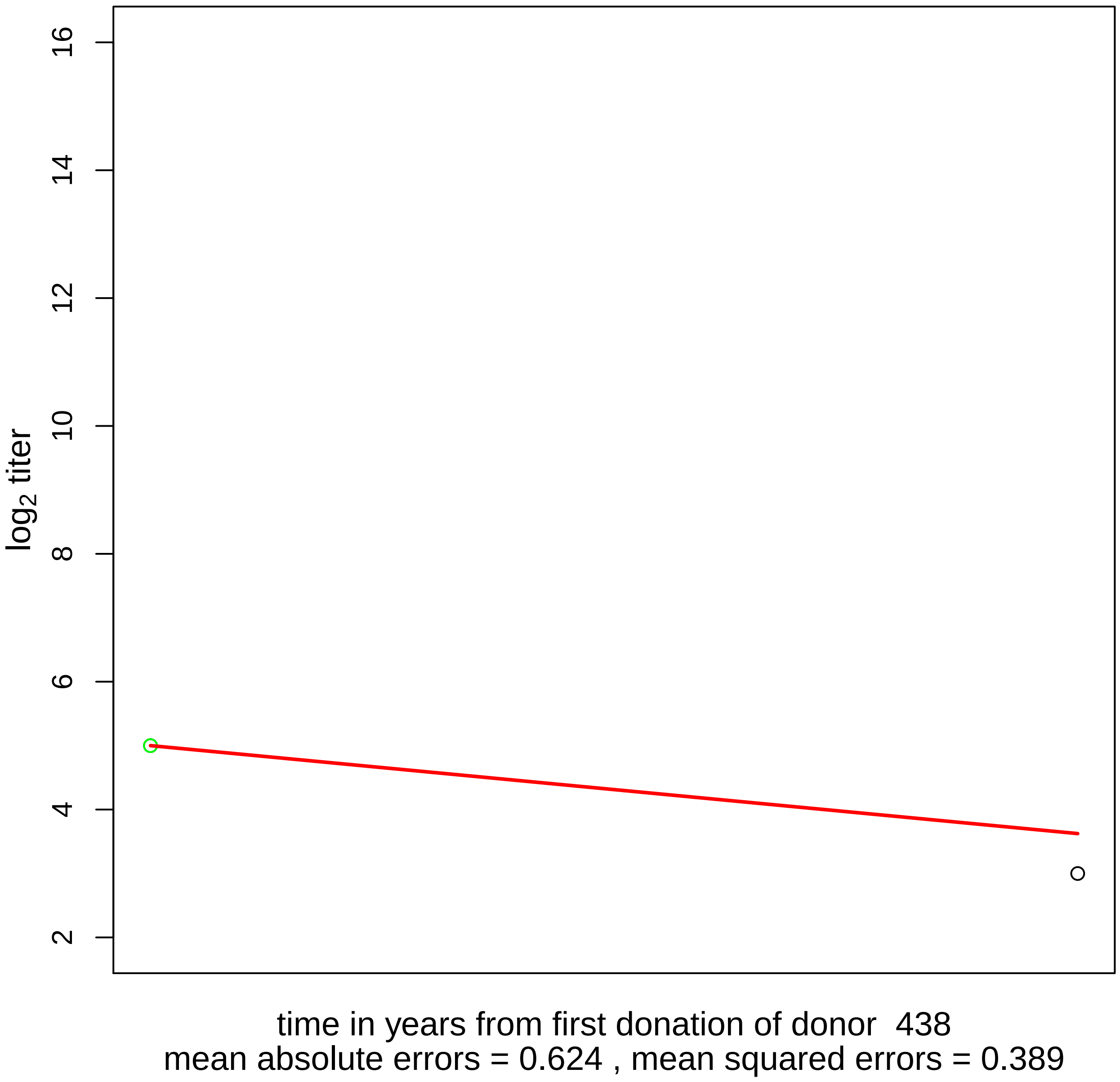

16
14
12
10
8
6
4
2
$\log_2$ titer
time in years from first donation of donor 438
mean absolute errors = 0.624 , mean squared errors = 0.389

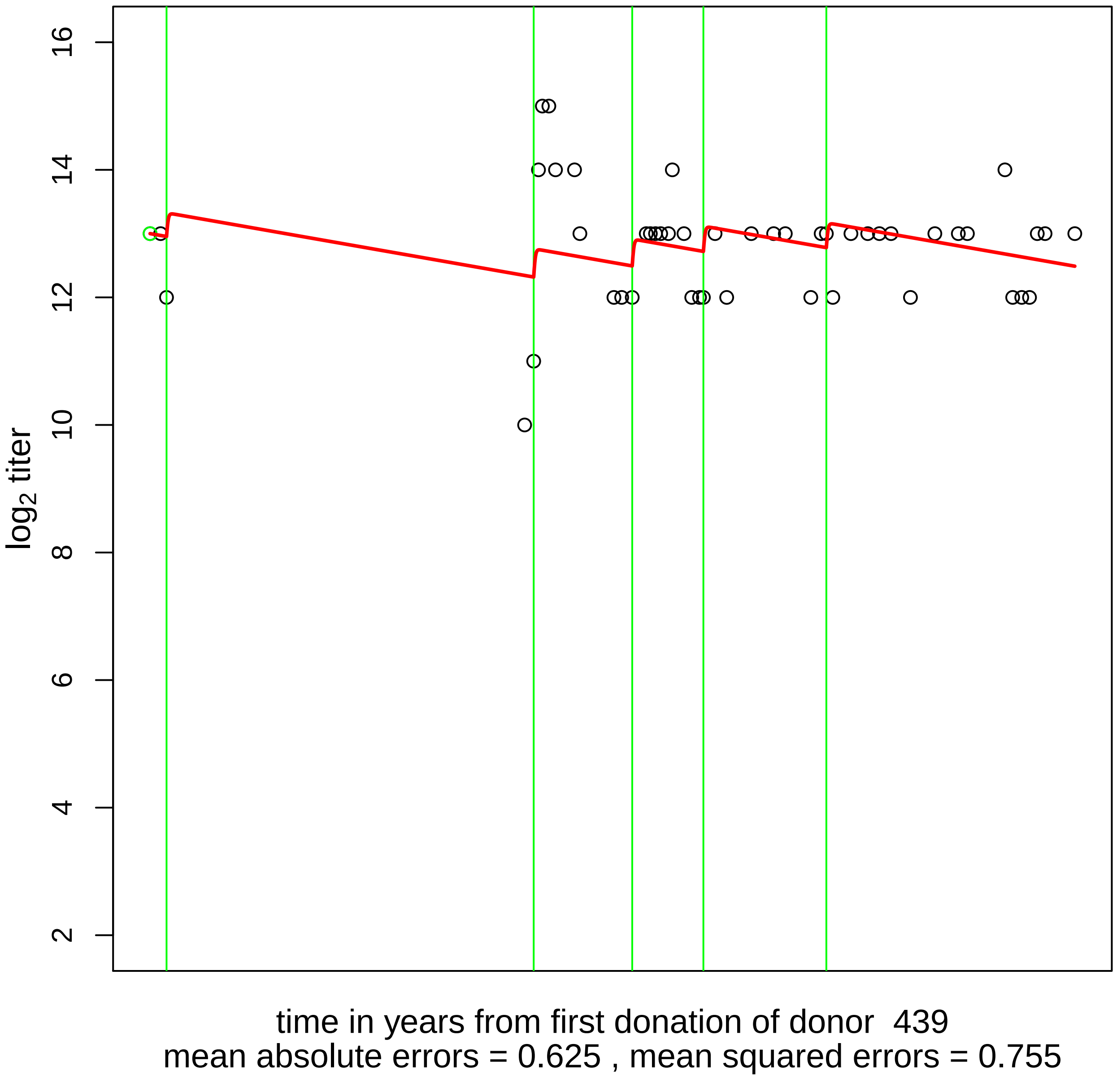
log2 titer
2
4
6
8
10
12
14
16
time in years from first donation of donor 439
mean absolute errors = 0.625 , mean squared errors = 0.755

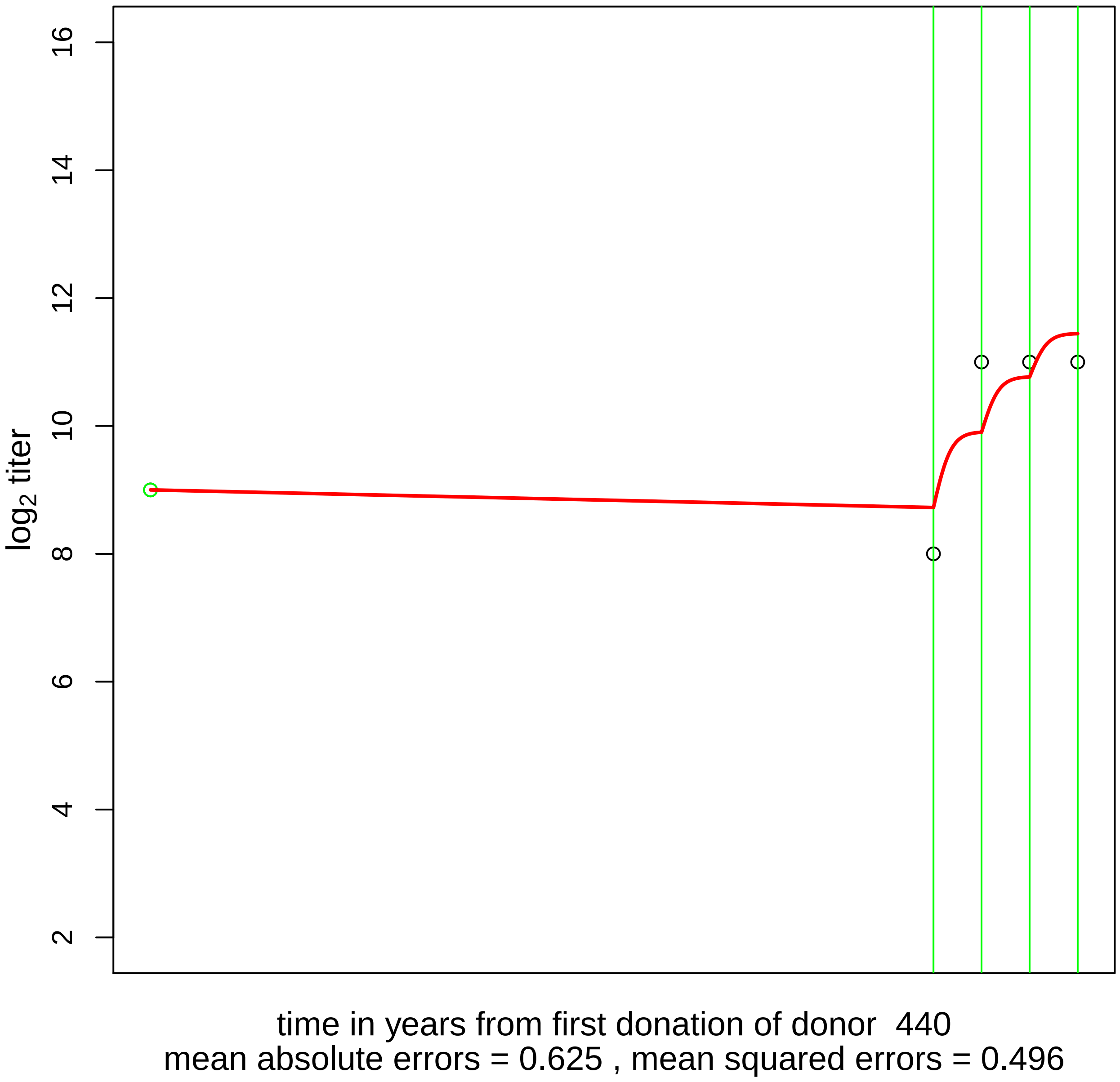

16
14
12
10
8
6
4
2
$\log_2$ titer
time in years from first donation of donor 440
mean absolute errors = 0.625 , mean squared errors = 0.496

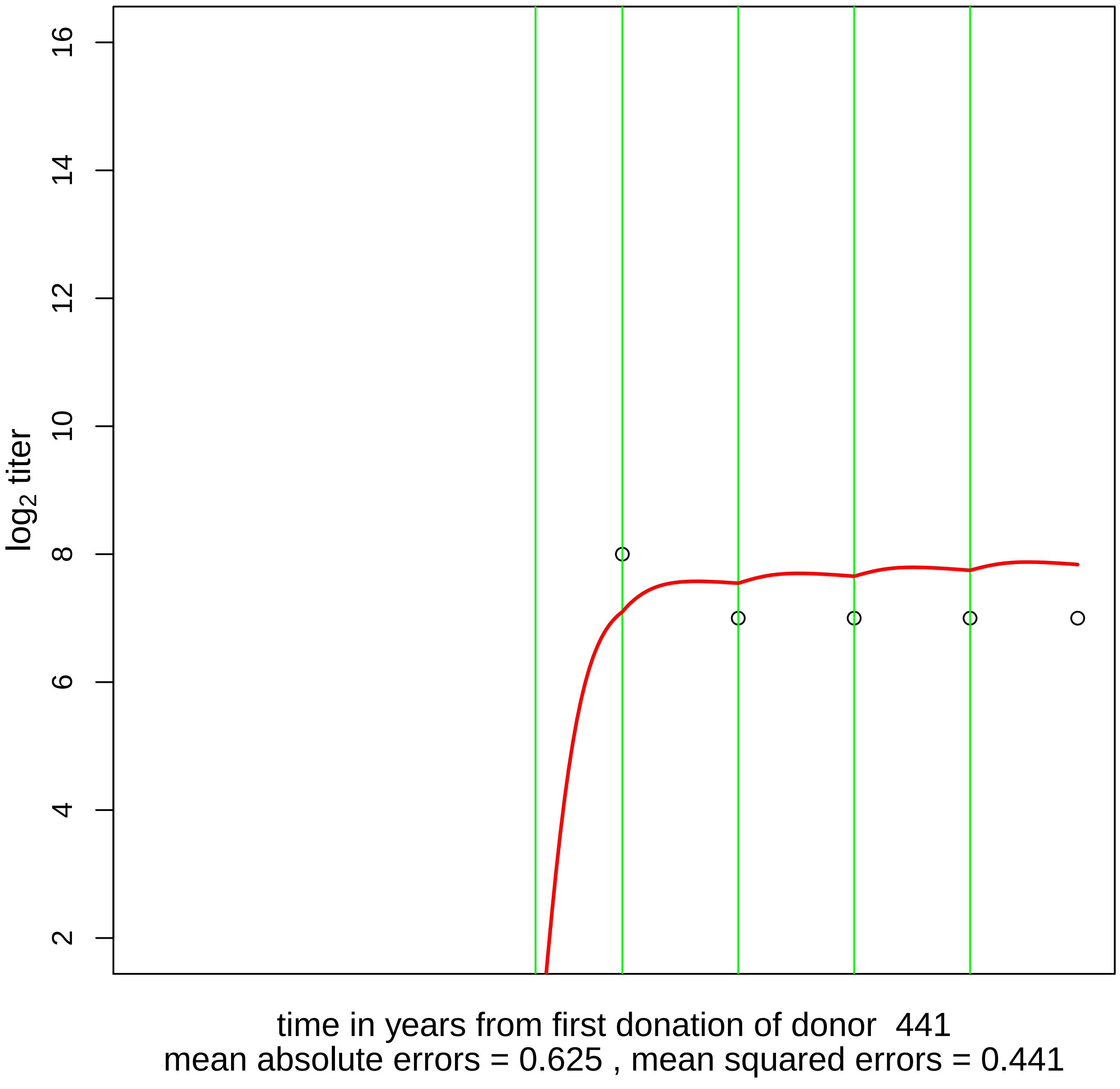

16
14
12
10
8
6
4
2
$\log_2$ titer
time in years from first donation of donor 441
mean absolute errors = 0.625 , mean squared errors = 0.441

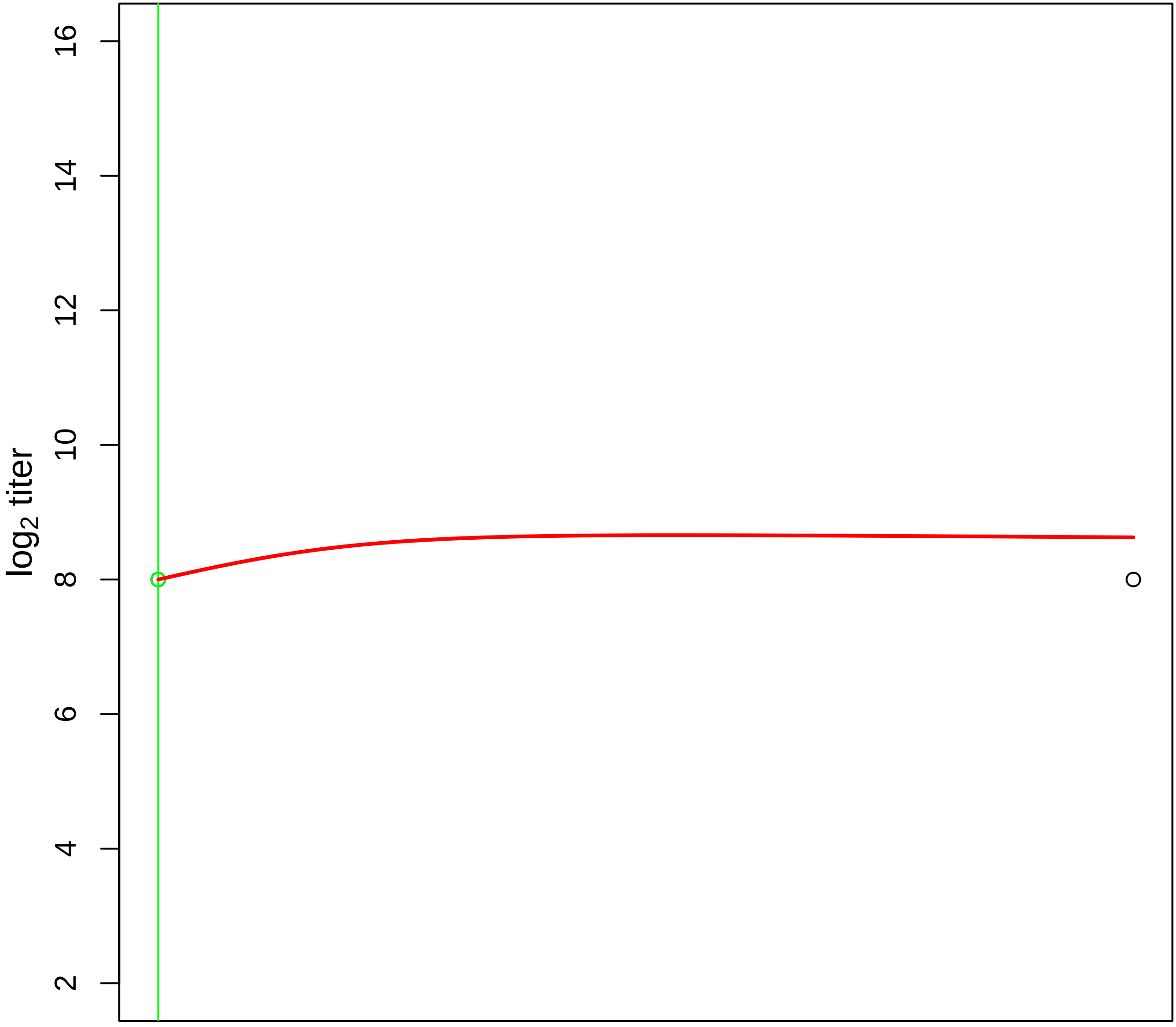

time in years from first donation of donor 442
mean absolute errors = 0.626 , mean squared errors = 0.391

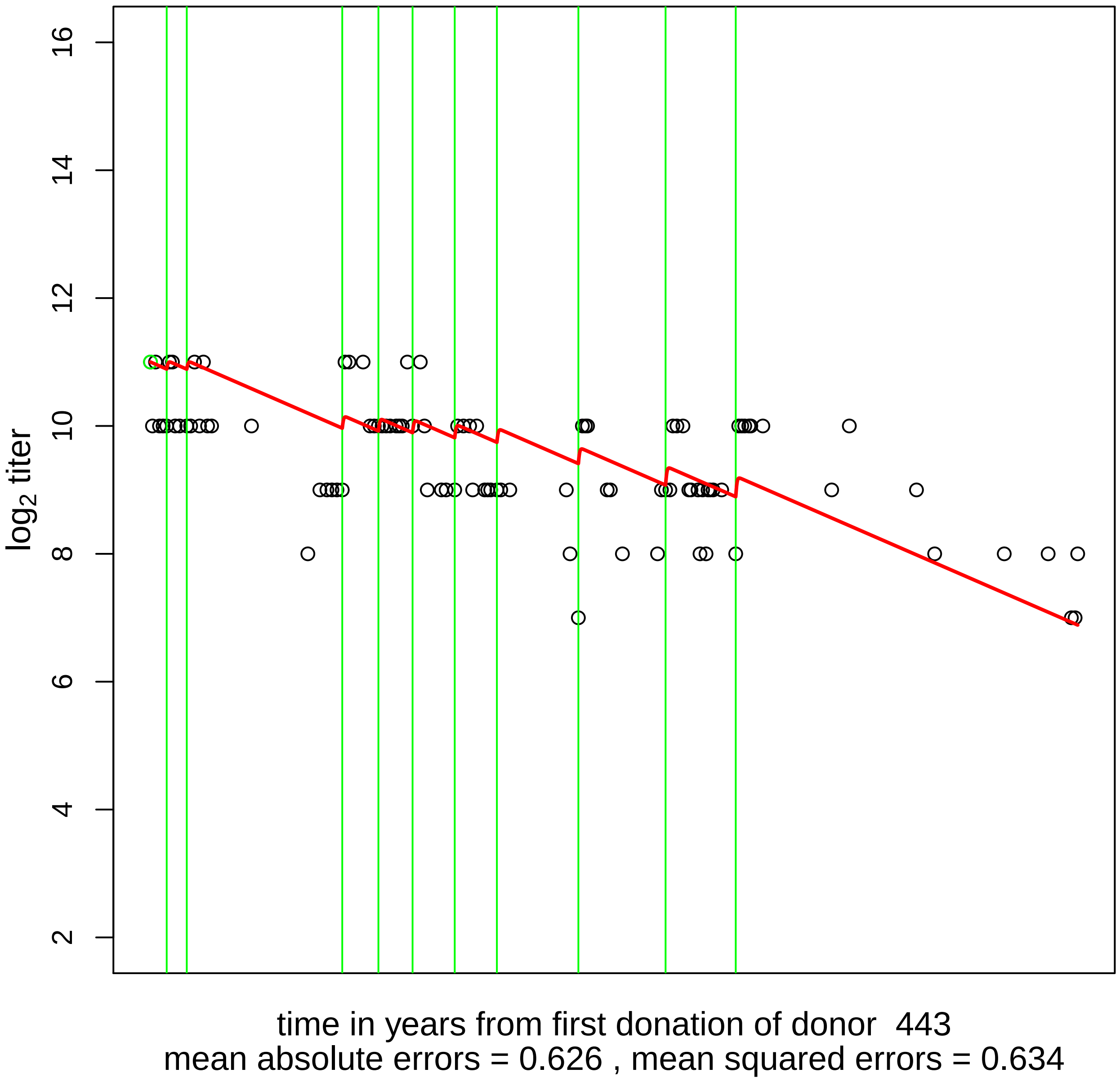

$\log_2$ titer
2
4
6
8
10
12
14
16
time in years from first donation of donor 443
mean absolute errors = 0.626 , mean squared errors = 0.634

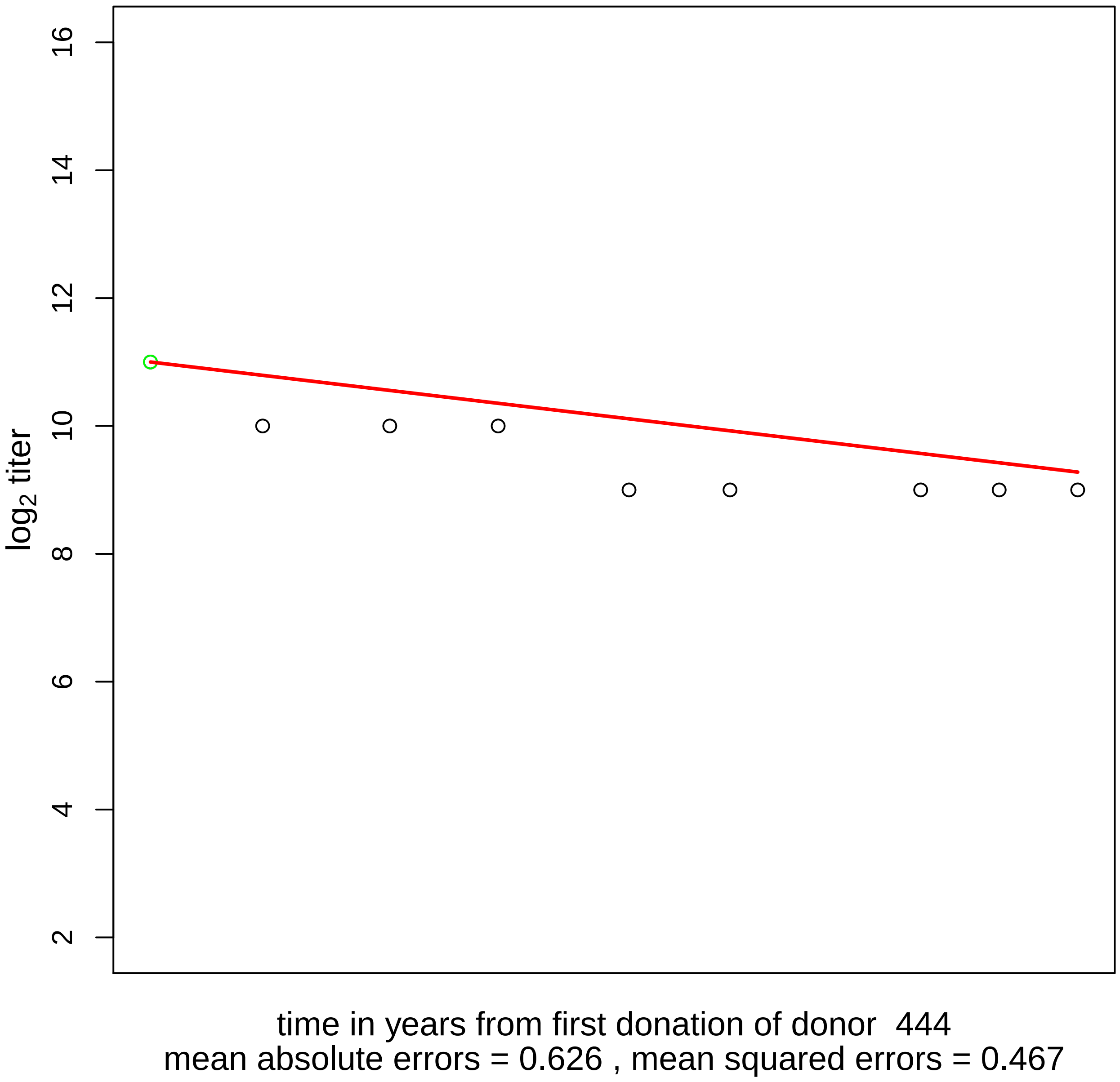
16
14
12
10
8
6
4
2
$\log_2$ titer
time in years from first donation of donor 444
mean absolute errors = 0.626 , mean squared errors = 0.467

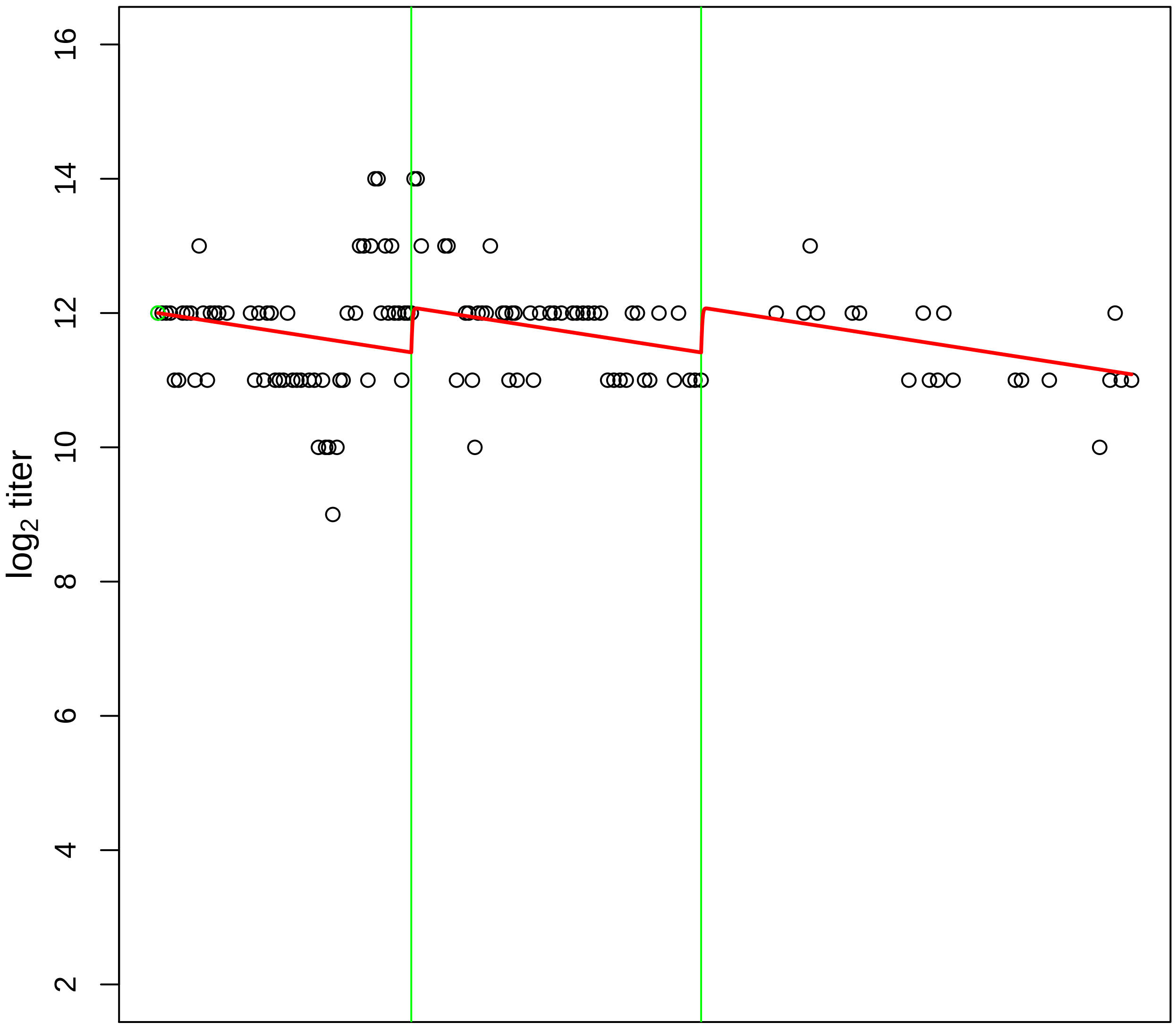

time in years from first donation of donor 445
mean absolute errors = 0.626 , mean squared errors = 0.689

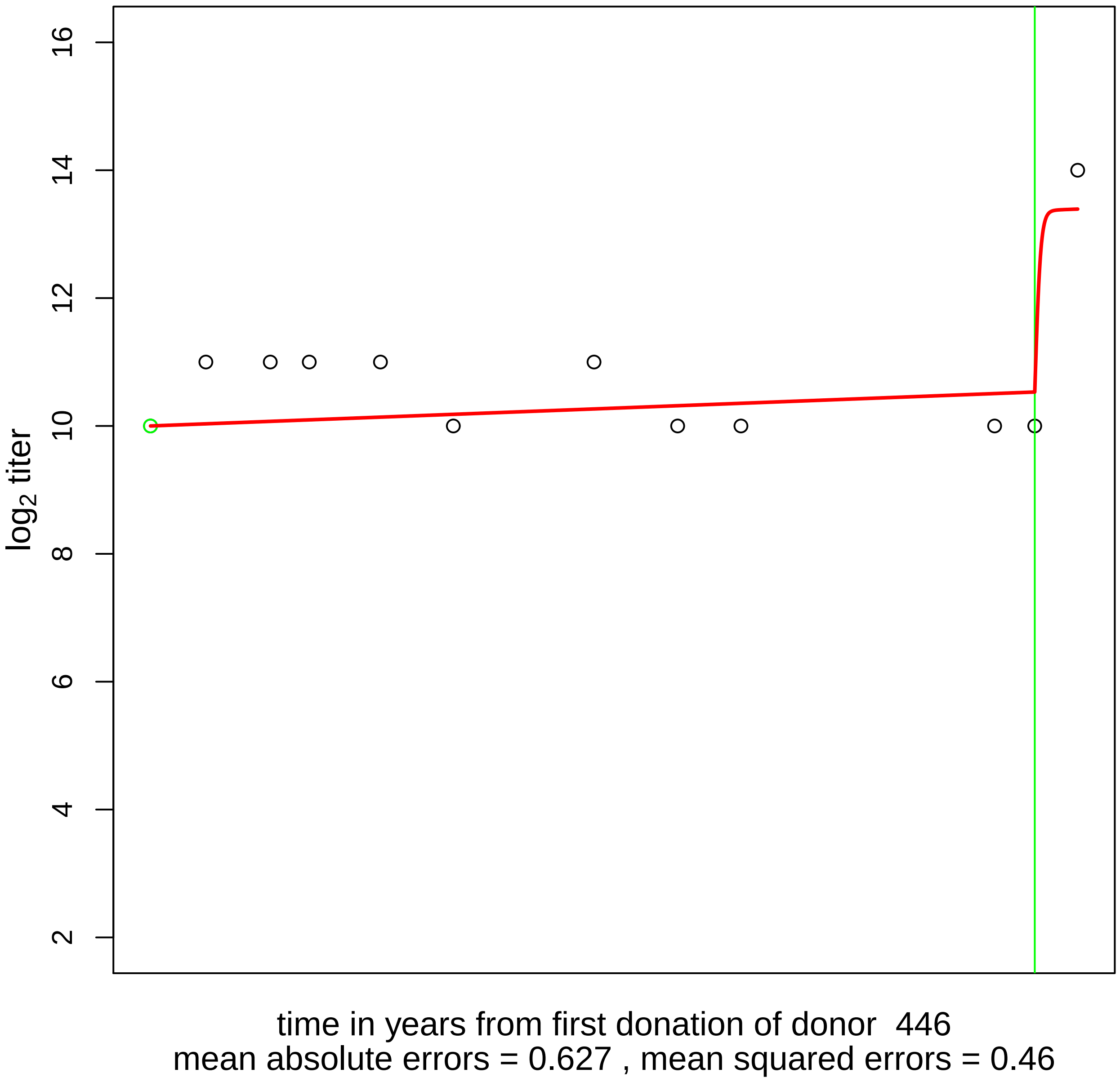
log2 titer
2
4
6
8
10
12
14
16
time in years from first donation of donor 446
mean absolute errors = 0.627 , mean squared errors = 0.46

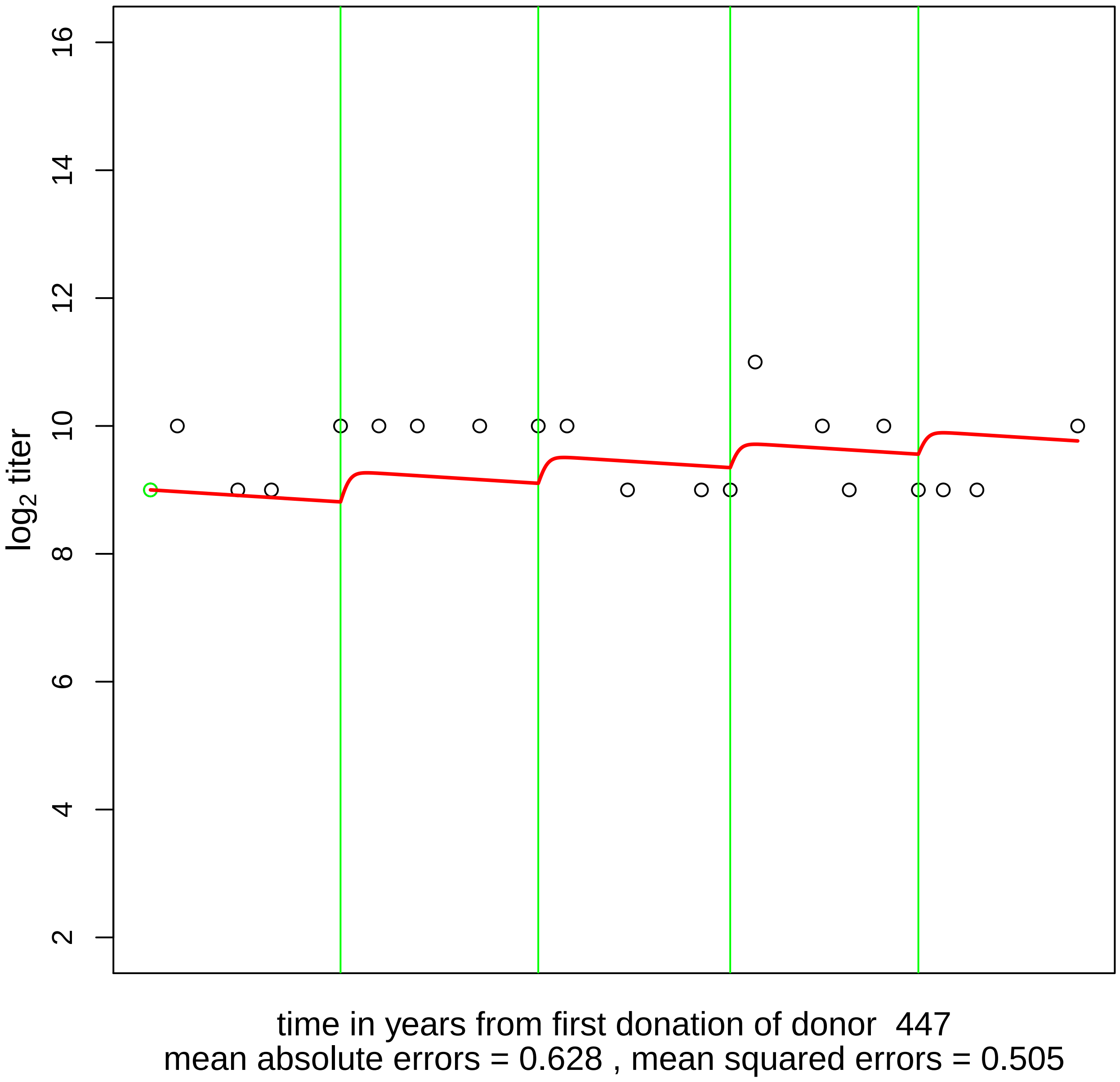

log2 titer
2
4
6
8
10
12
14
16
time in years from first donation of donor 447
mean absolute errors = 0.628 , mean squared errors = 0.505

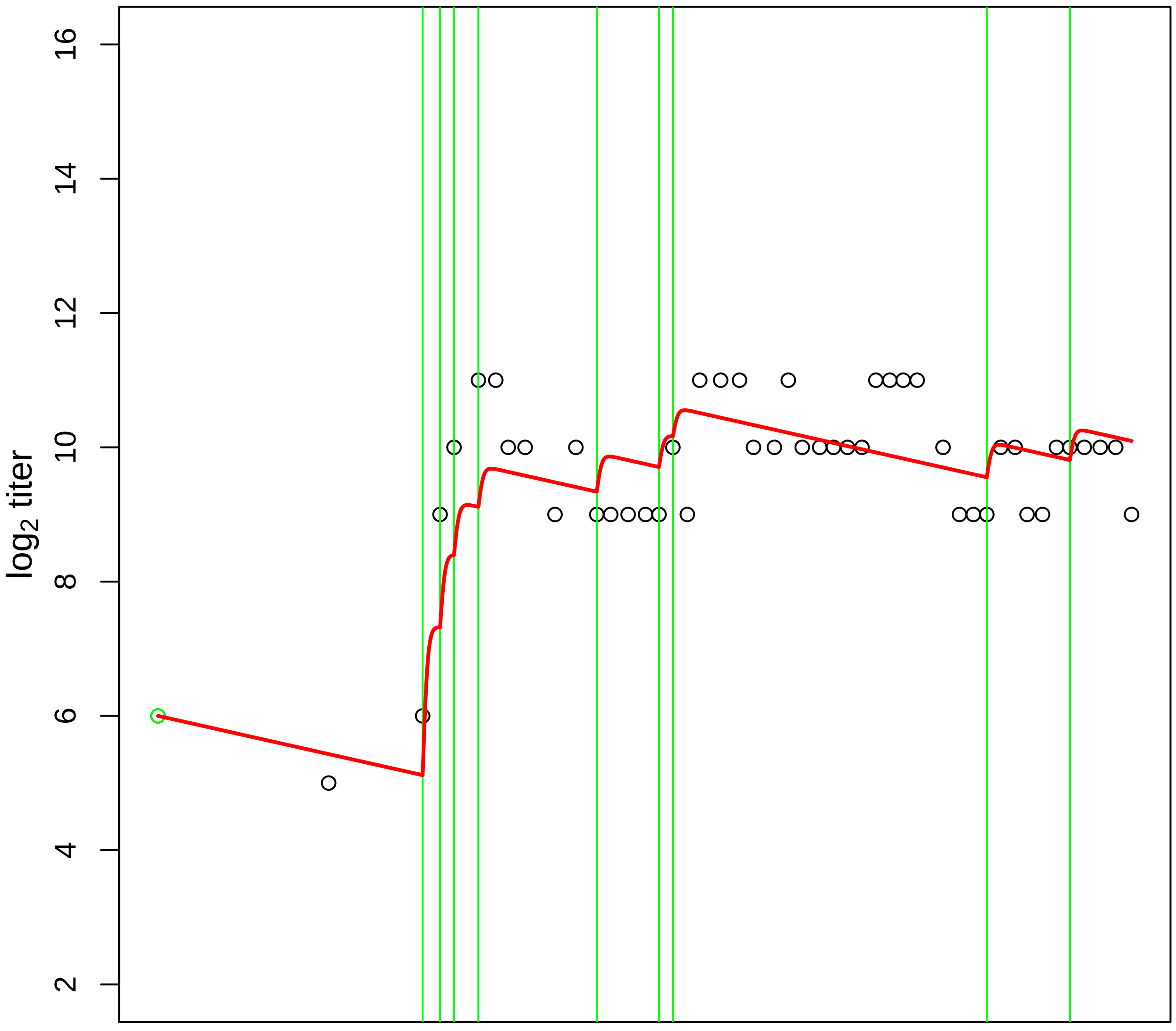

time in years from first donation of donor 448
mean absolute errors = 0.628 , mean squared errors = 0.628

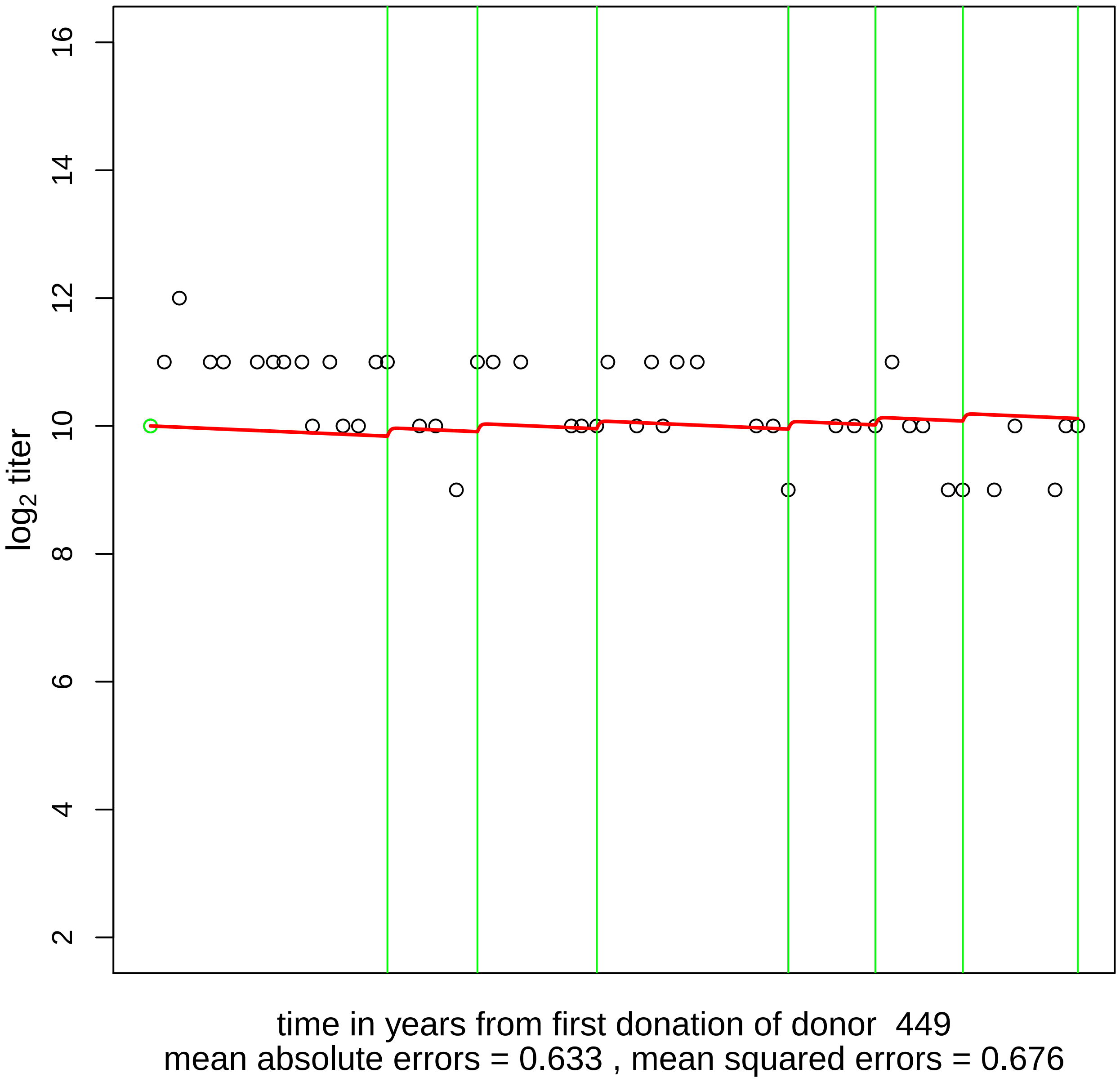

16
14
12
10
8
6
4
2
$\log_2$ titer
time in years from first donation of donor 449
mean absolute errors = 0.633 , mean squared errors = 0.676

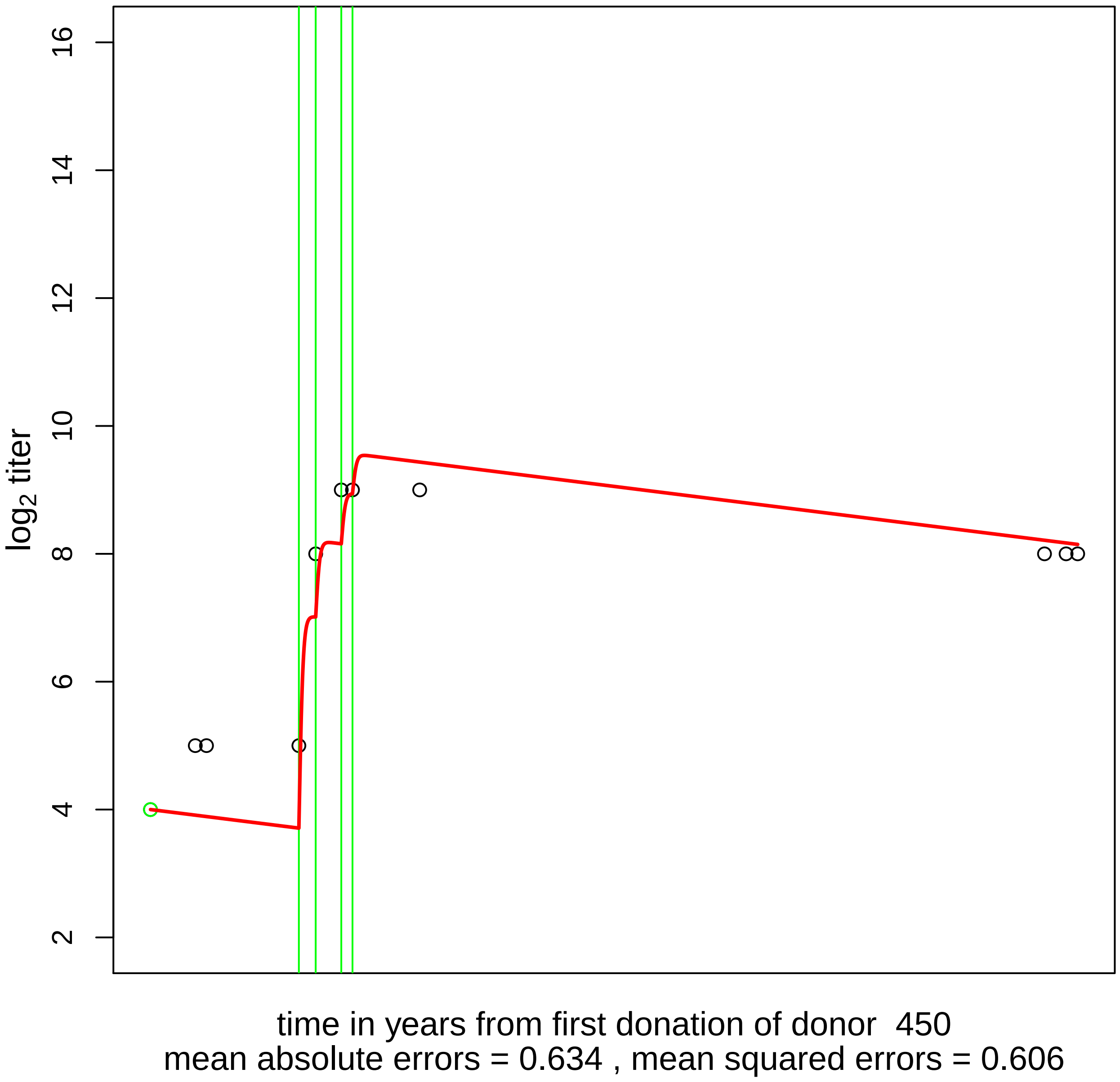

log2 titer
2
4
6
8
10
12
14
16
time in years from first donation of donor 450
mean absolute errors = 0.634 , mean squared errors = 0.606

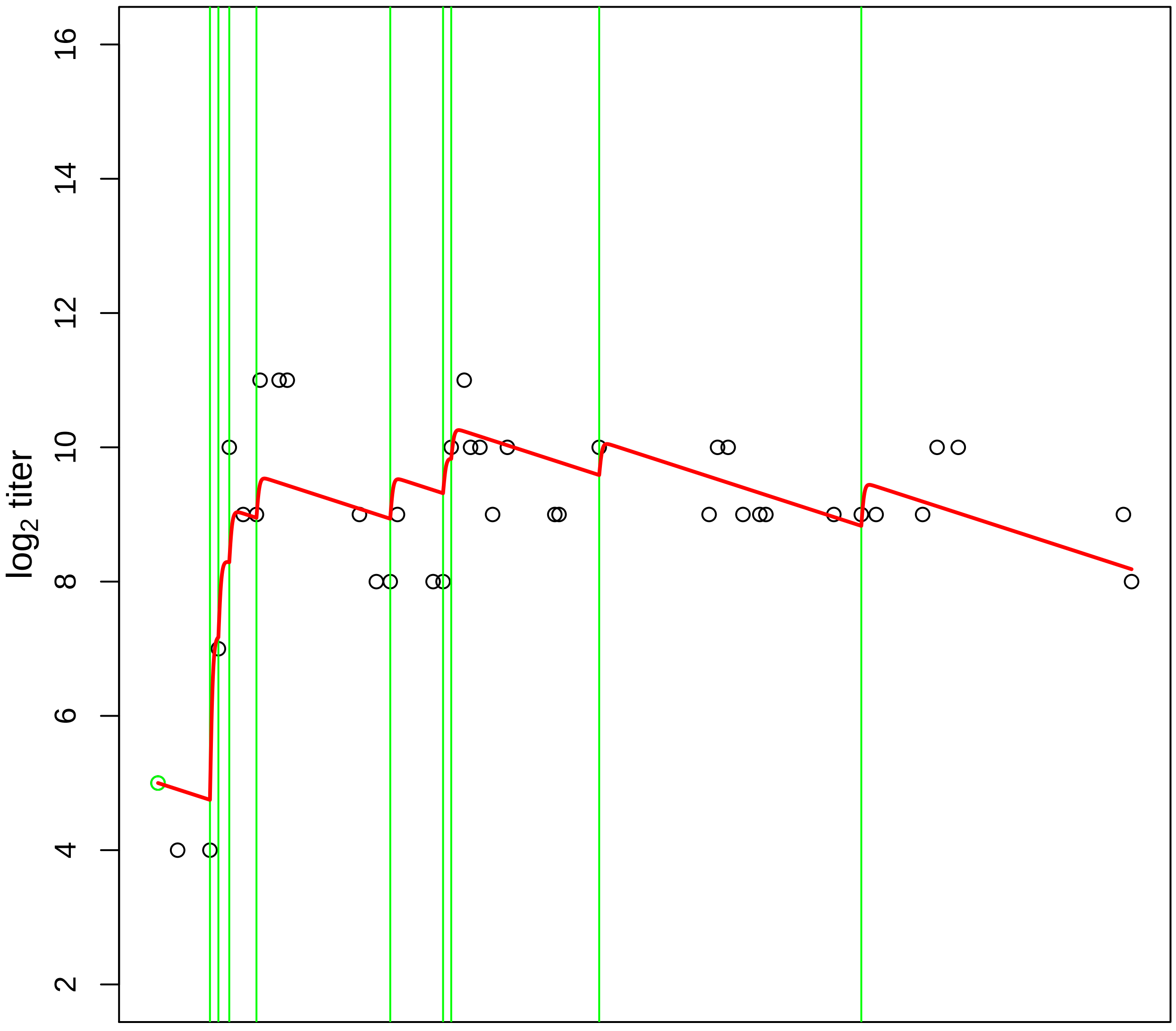

time in years from first donation of donor 451
mean absolute errors = 0.635 , mean squared errors = 0.642

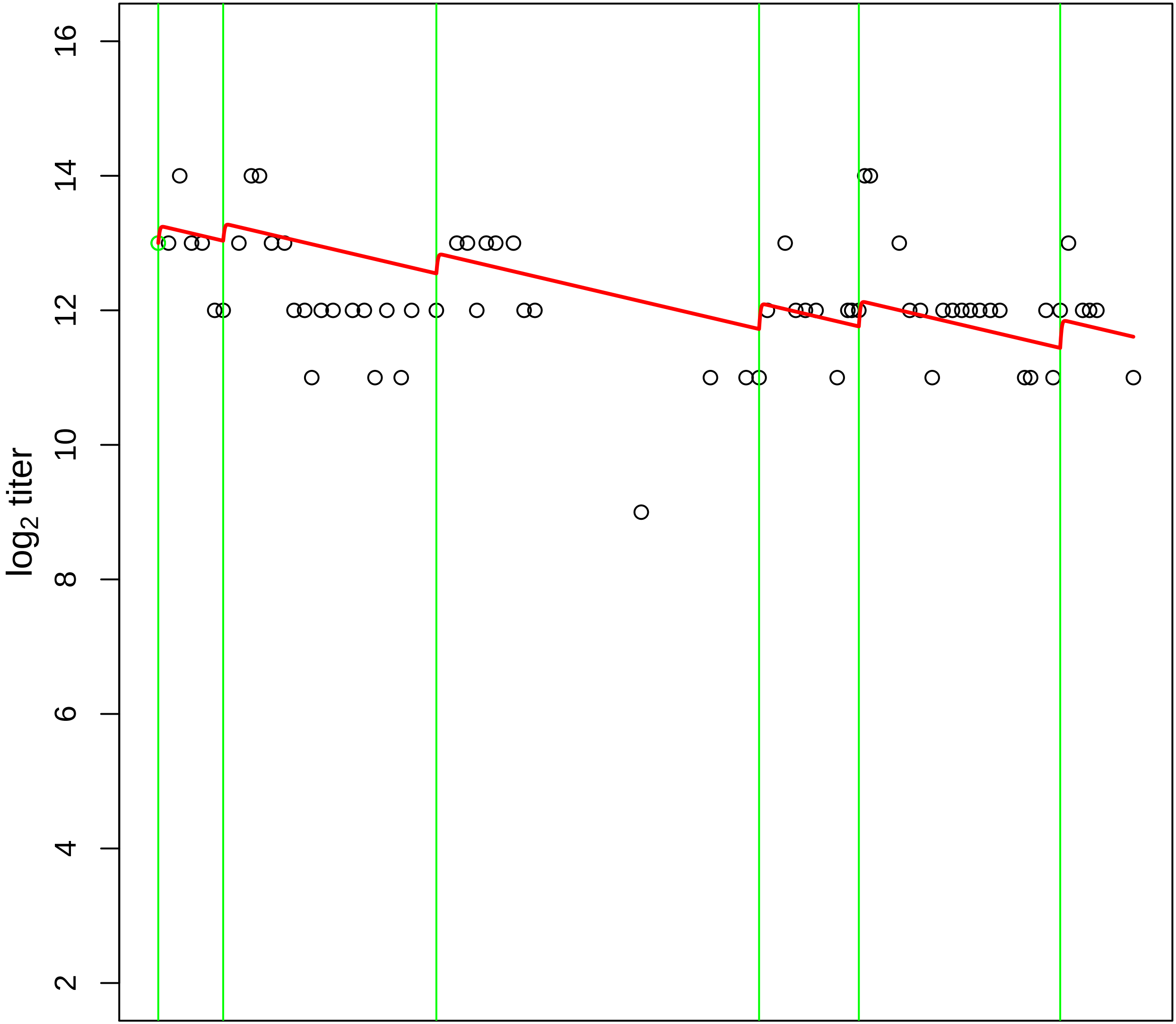

time in years from first donation of donor 452
mean absolute errors = 0.635 , mean squared errors = 0.723

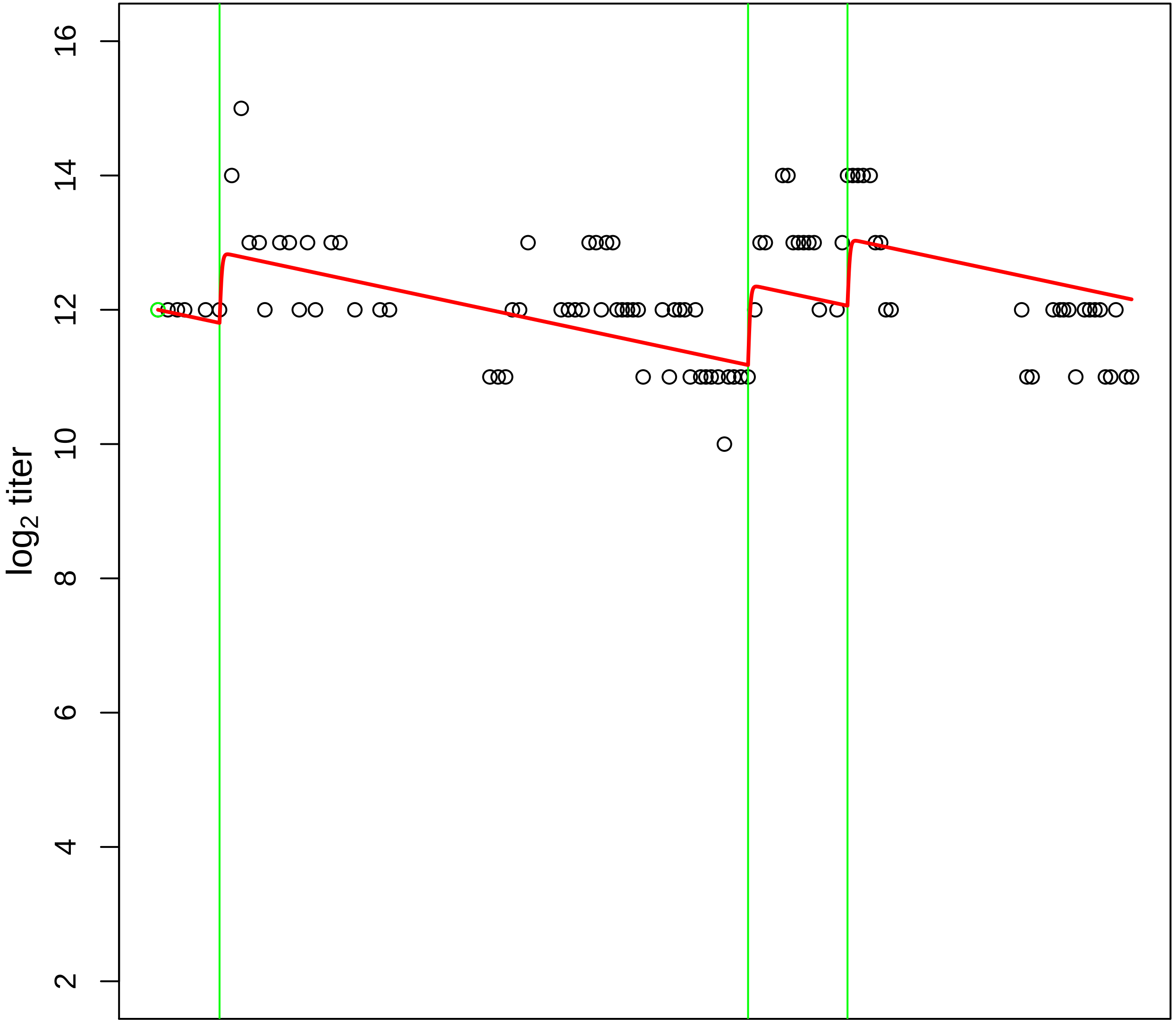

time in years from first donation of donor 453
mean absolute errors = 0.635 , mean squared errors = 0.626

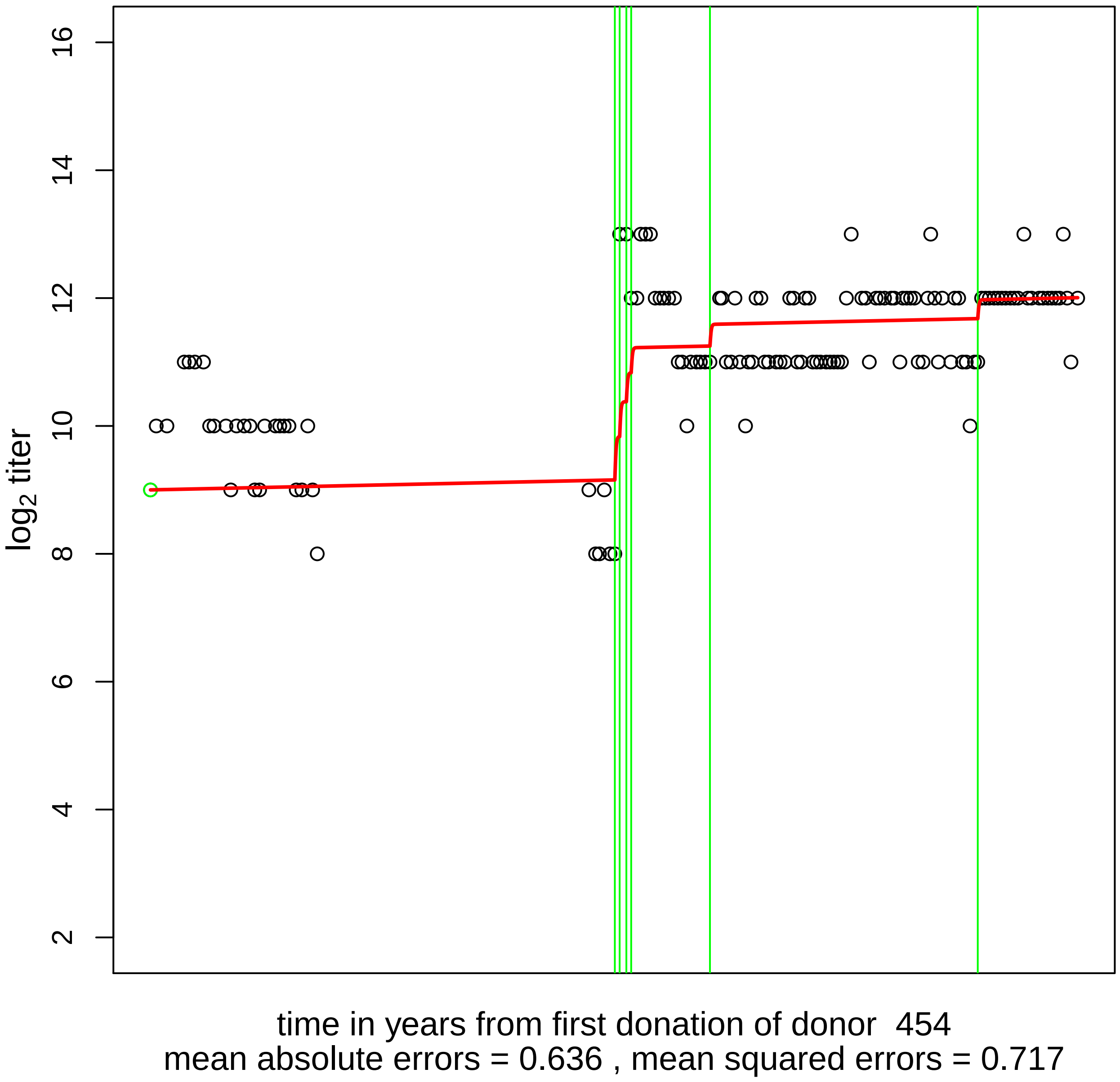

16
14
12
10
8
6
4
2
$\log_2$ titer
time in years from first donation of donor 454
mean absolute errors = 0.636 , mean squared errors = 0.717

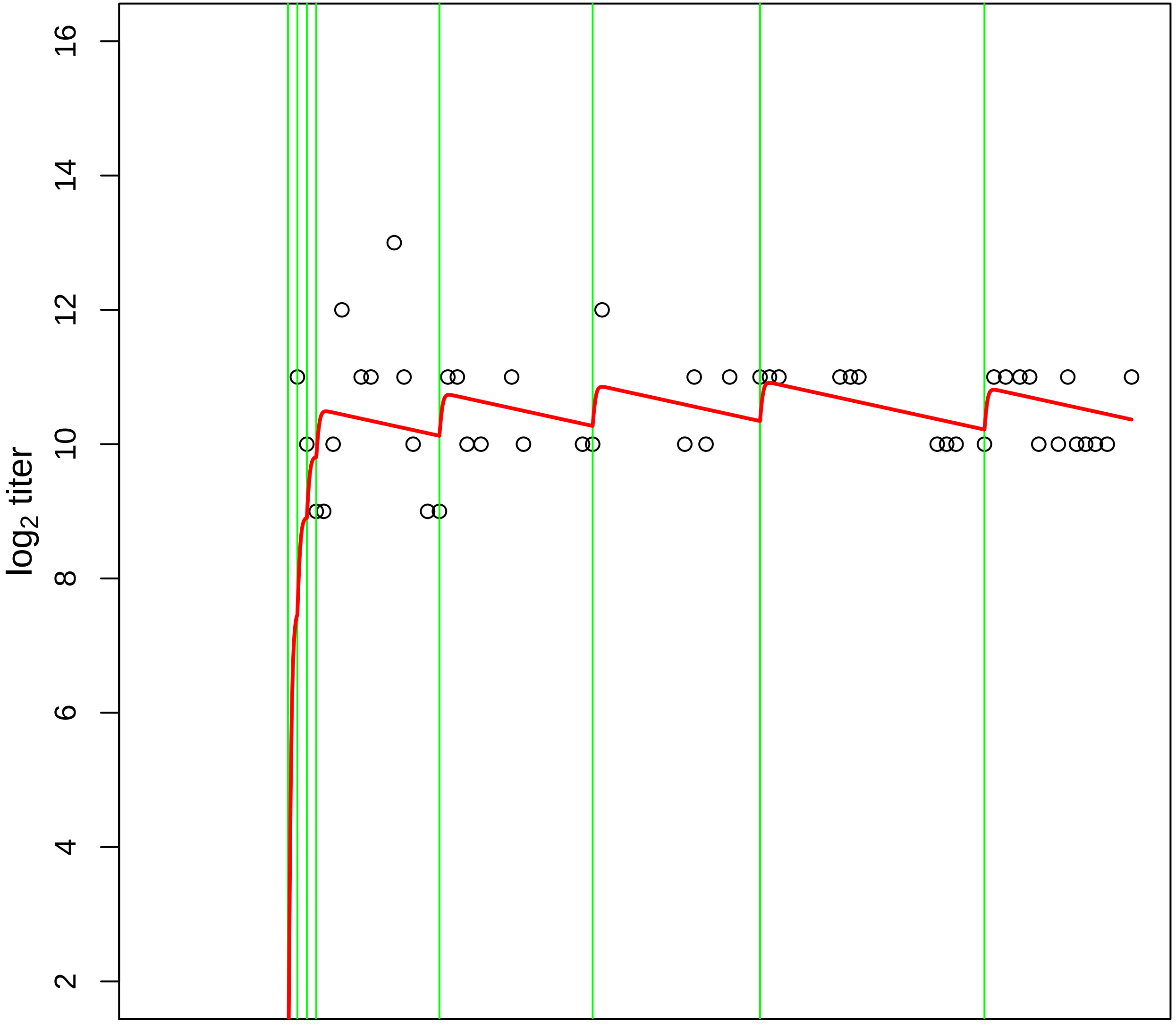

time in years from first donation of donor 455
mean absolute errors = 0.636 , mean squared errors = 0.777

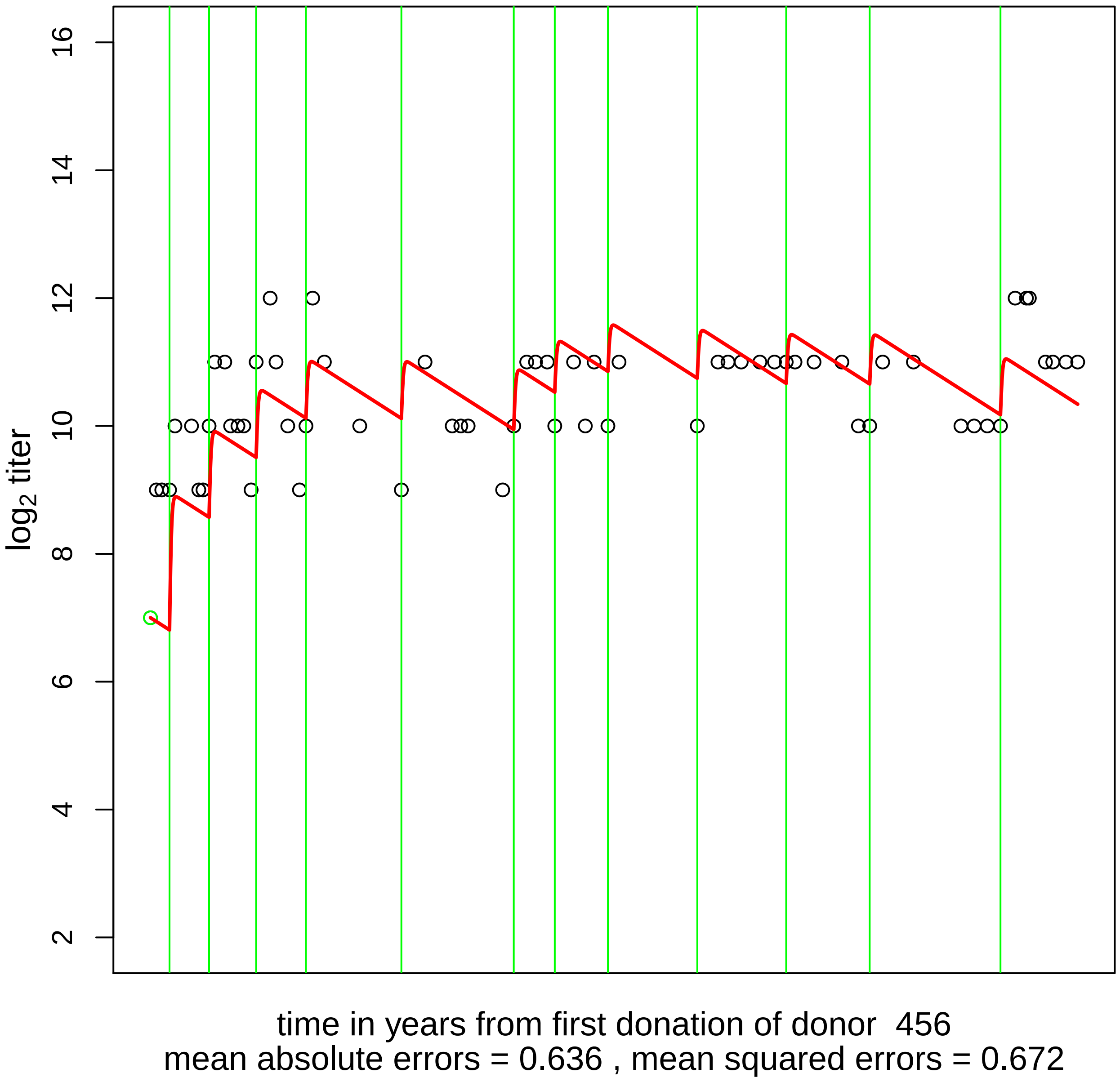

log2 titer
2
4
6
8
10
12
14
16
time in years from first donation of donor 456
mean absolute errors = 0.636 , mean squared errors = 0.672

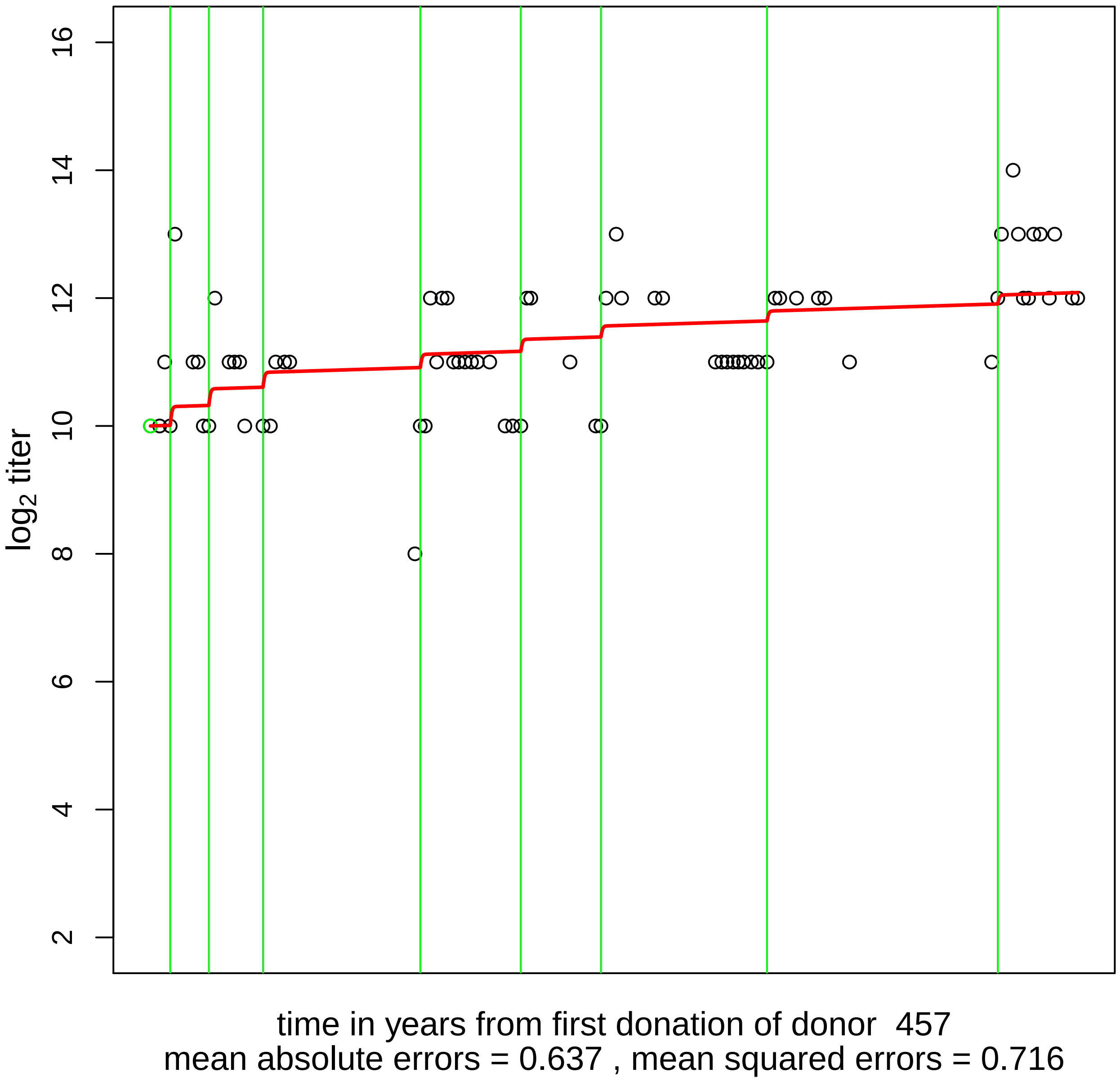

16
14
12
10
8
6
4
2
log2 titer
time in years from first donation of donor 457
mean absolute errors = 0.637 , mean squared errors = 0.716

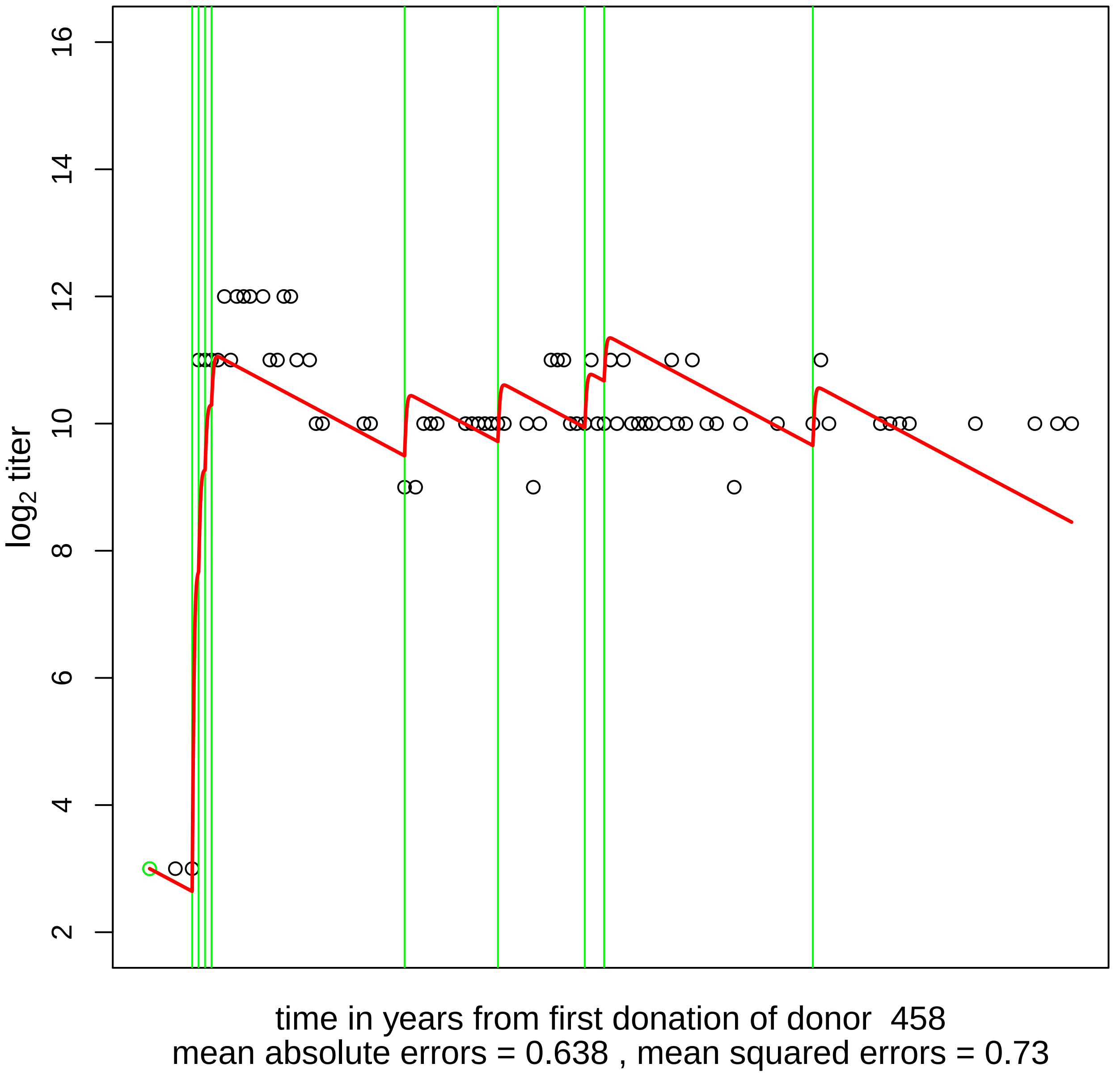

log2 titer
2
4
6
8
10
12
14
16
time in years from first donation of donor 458
mean absolute errors = 0.638 , mean squared errors = 0.73

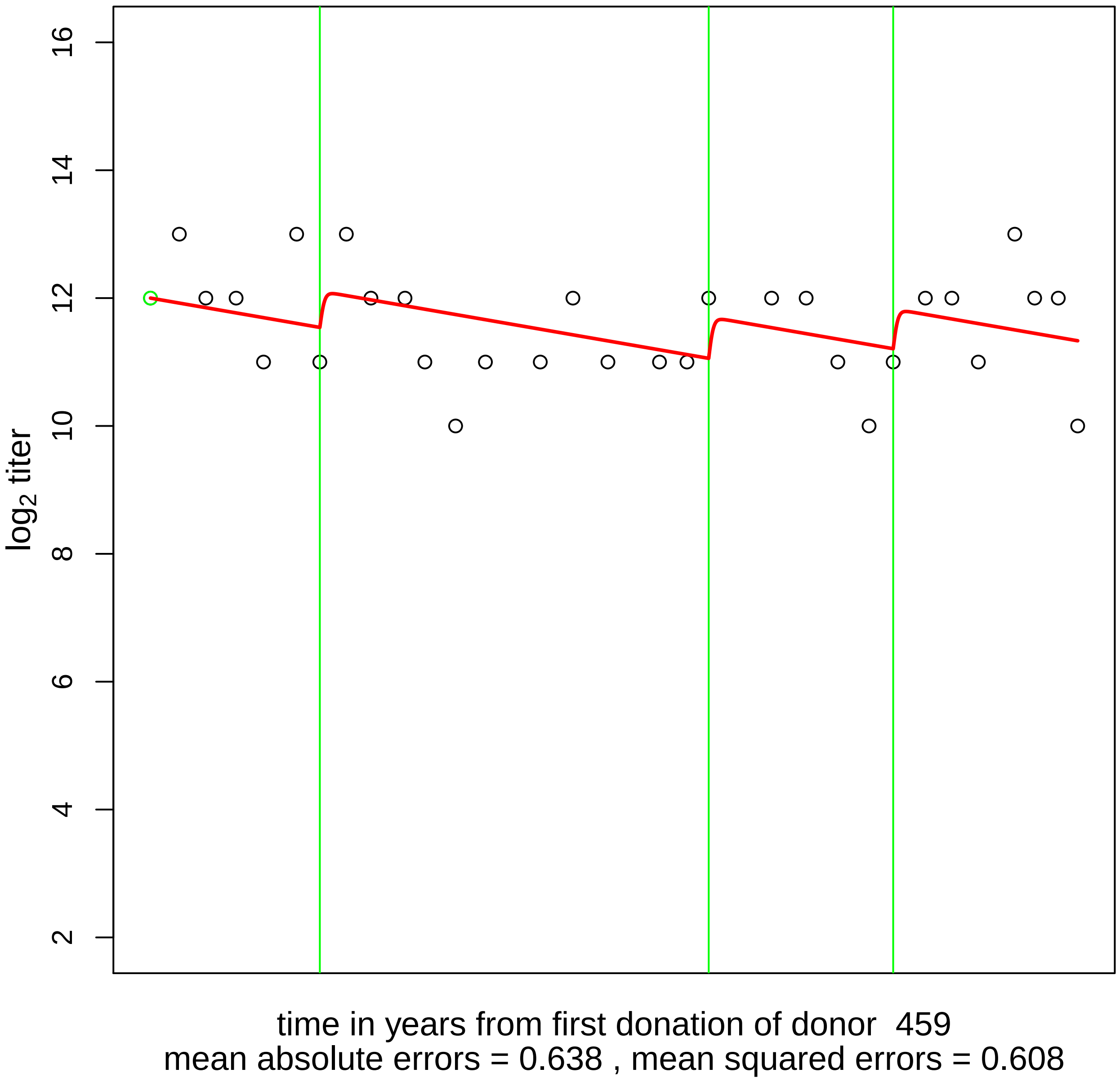

16
14
12
10
8
6
4
2
log2 titer
time in years from first donation of donor 459
mean absolute errors = 0.638 , mean squared errors = 0.608

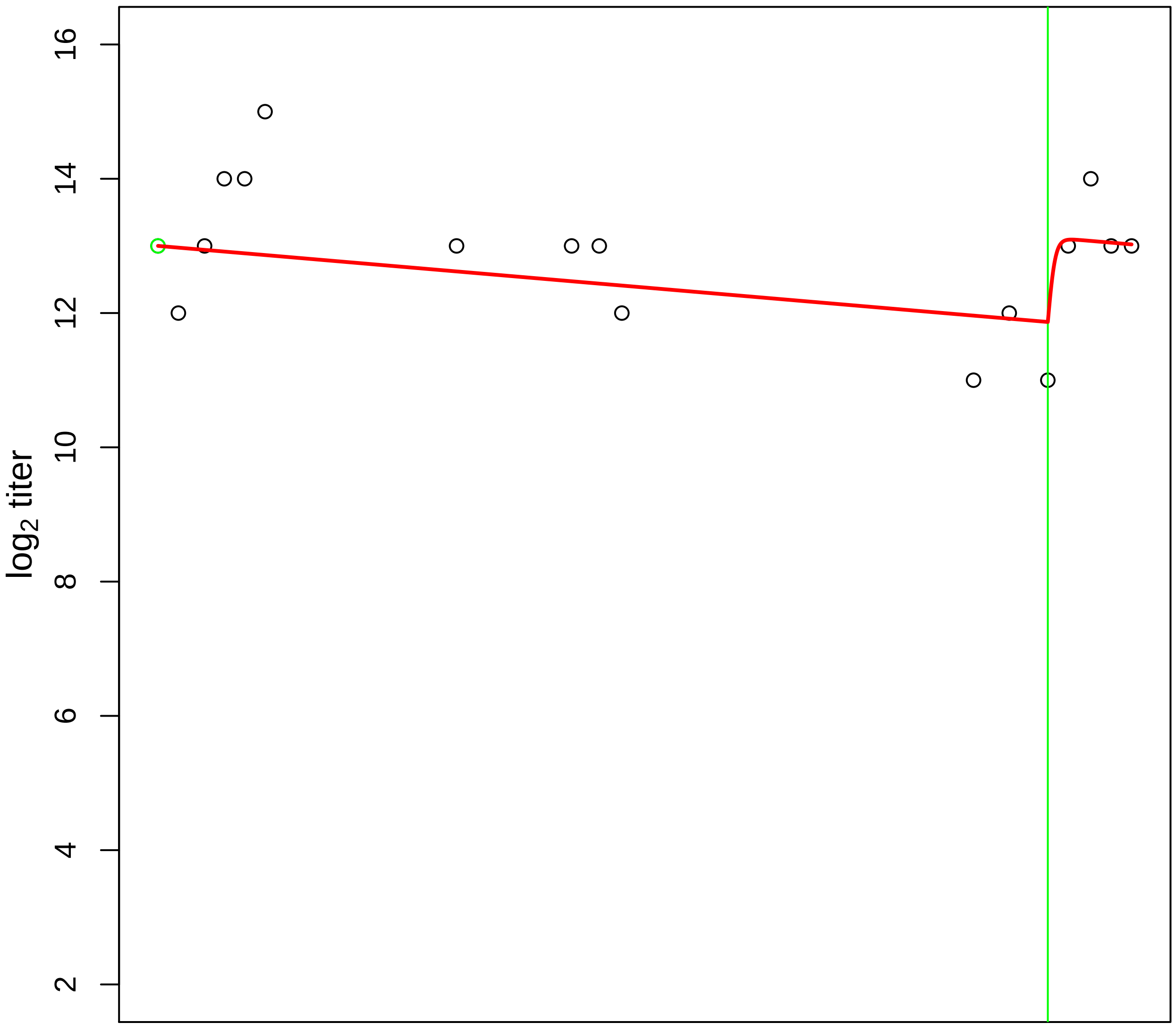

time in years from first donation of donor 460
mean absolute errors = 0.64 , mean squared errors = 0.711

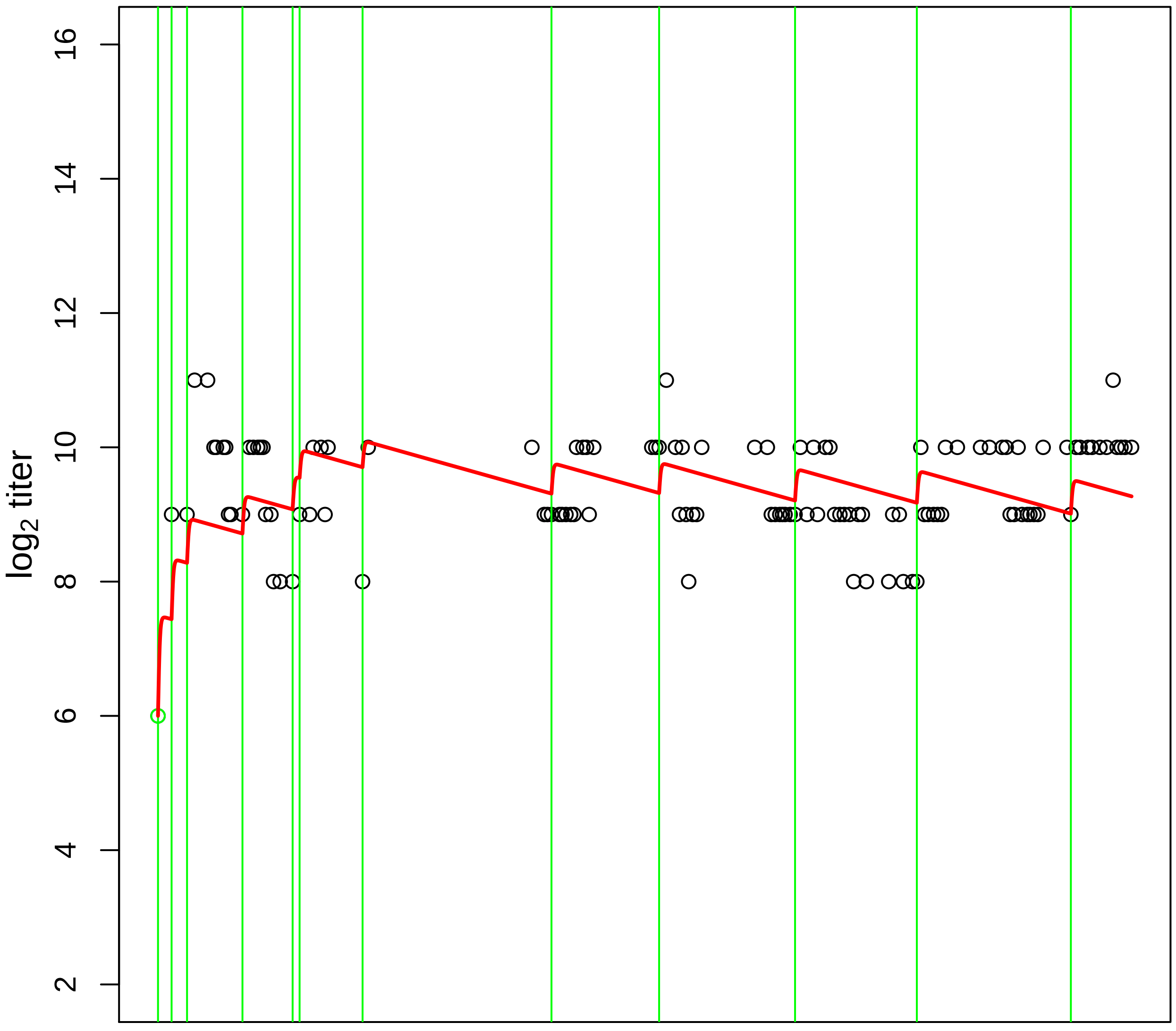

time in years from first donation of donor 461
mean absolute errors = 0.641 , mean squared errors = 0.582

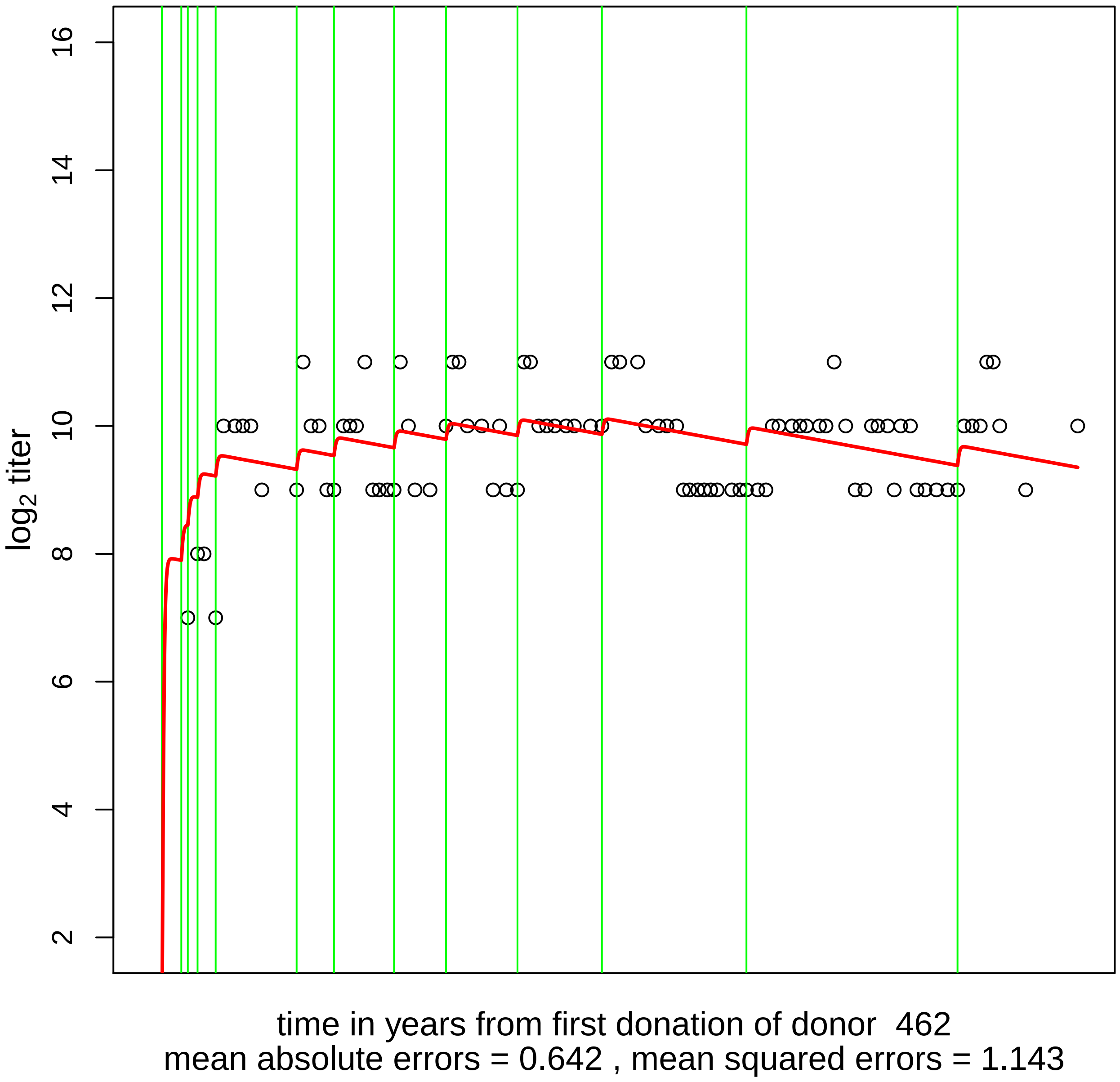

log2 titer
2
4
6
8
10
12
14
16
time in years from first donation of donor 462
mean absolute errors = 0.642 , mean squared errors = 1.143

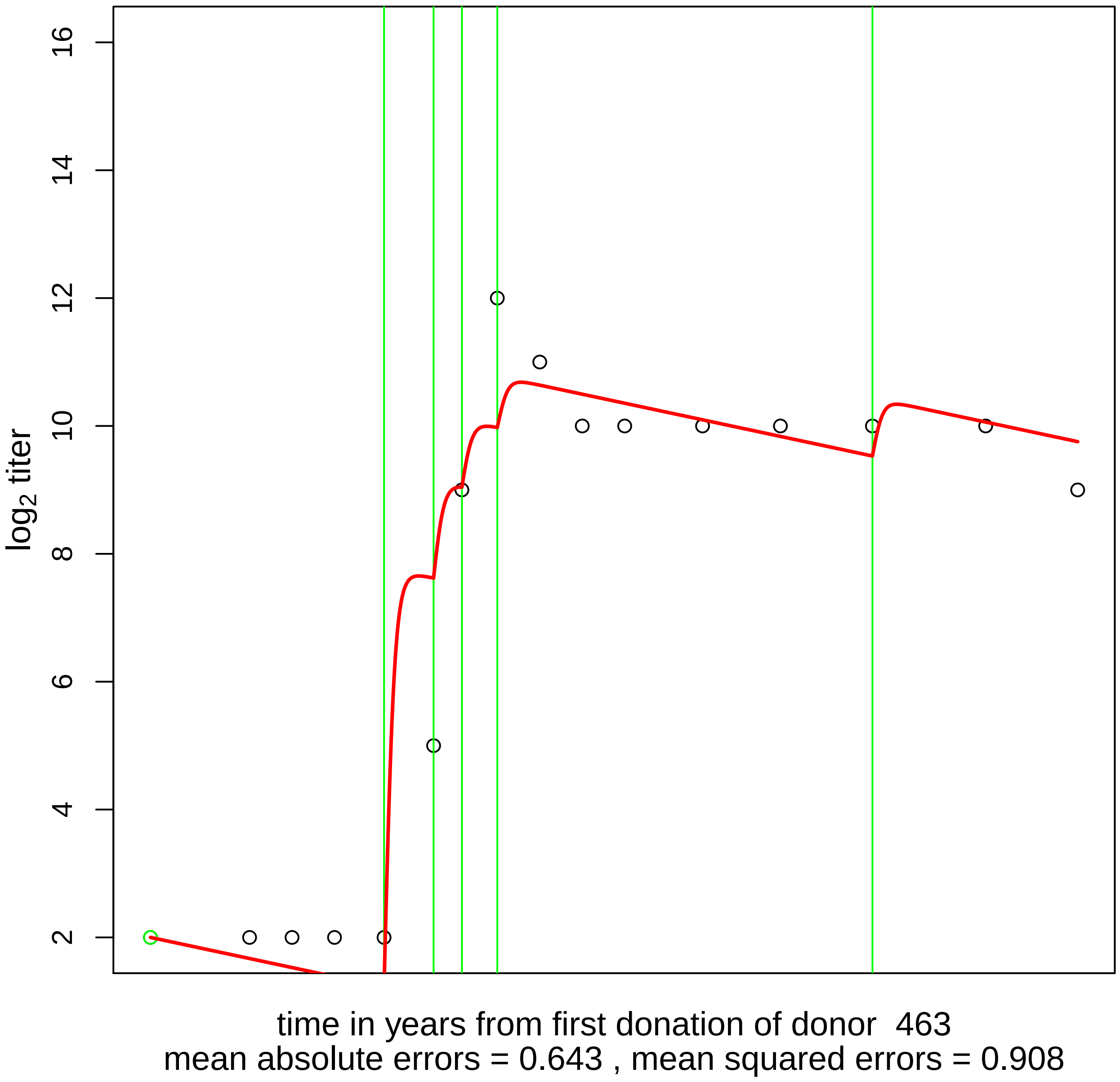
log2 titer
2
4
6
8
10
12
14
16
time in years from first donation of donor 463
mean absolute errors = 0.643 , mean squared errors = 0.908

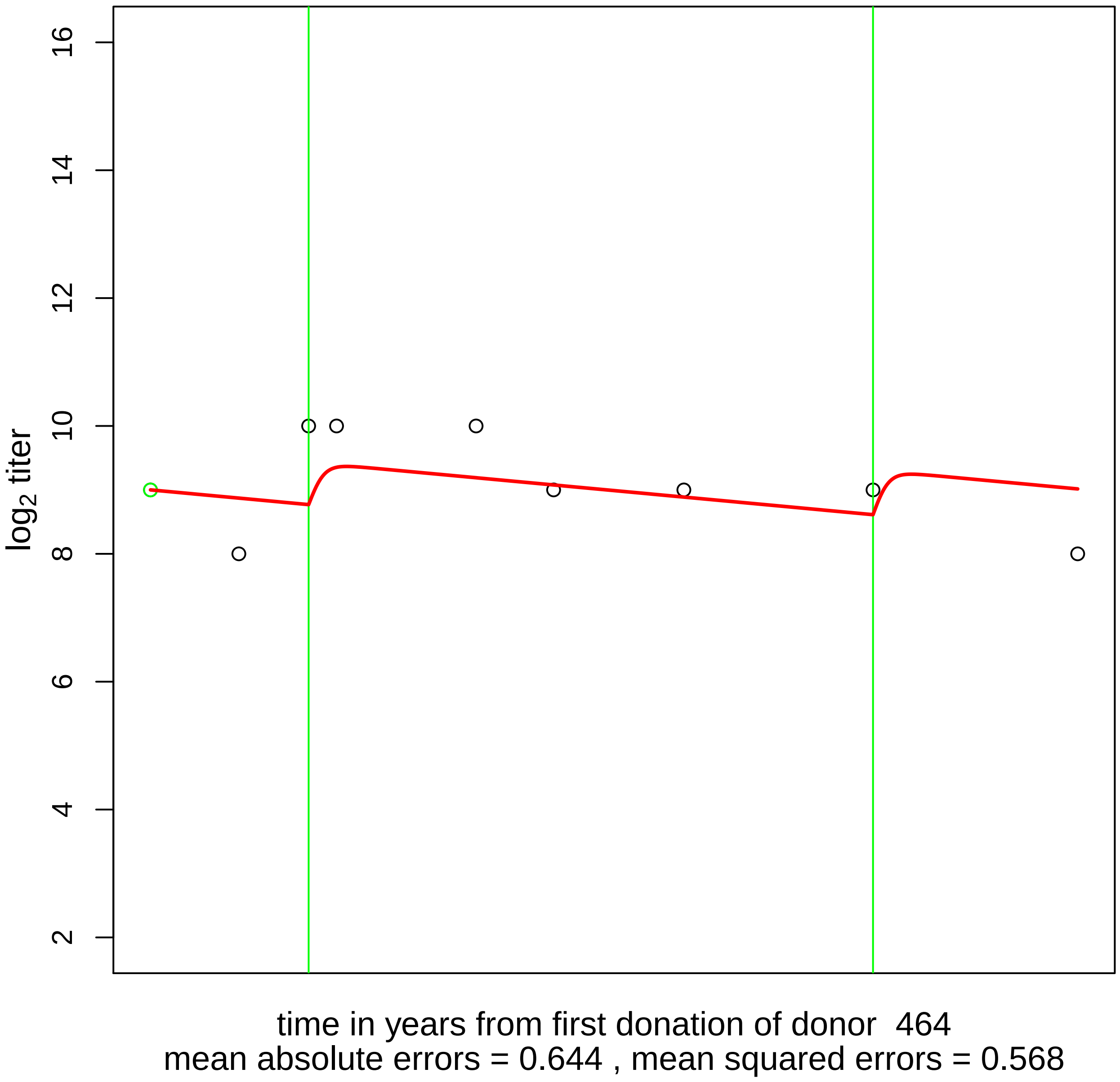

2
4
6
8
10
12
14
16
log2 titer
time in years from first donation of donor 464
mean absolute errors = 0.644 , mean squared errors = 0.568

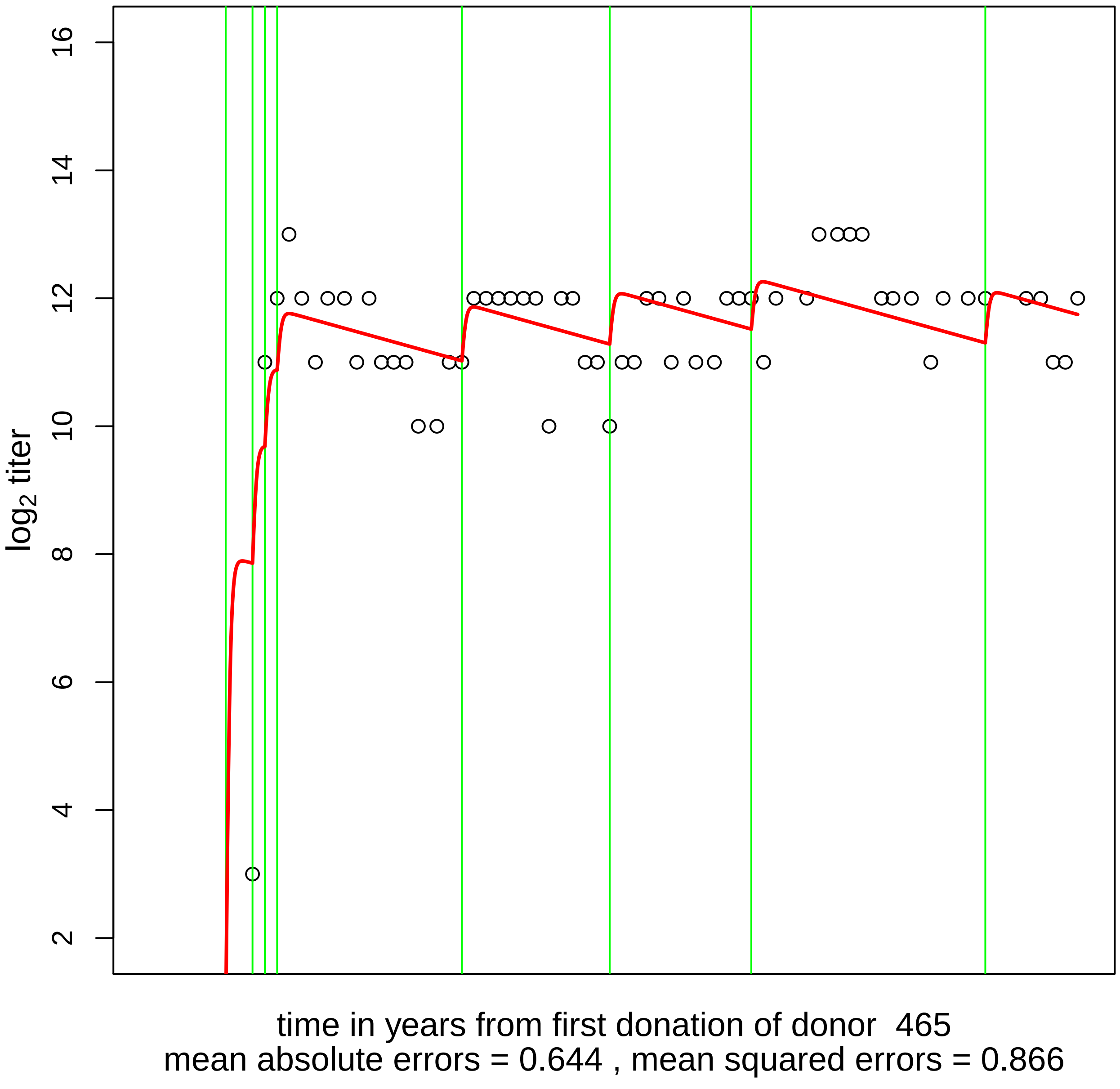

log2 titer
2
4
6
8
10
12
14
16
time in years from first donation of donor 465
mean absolute errors = 0.644 , mean squared errors = 0.866

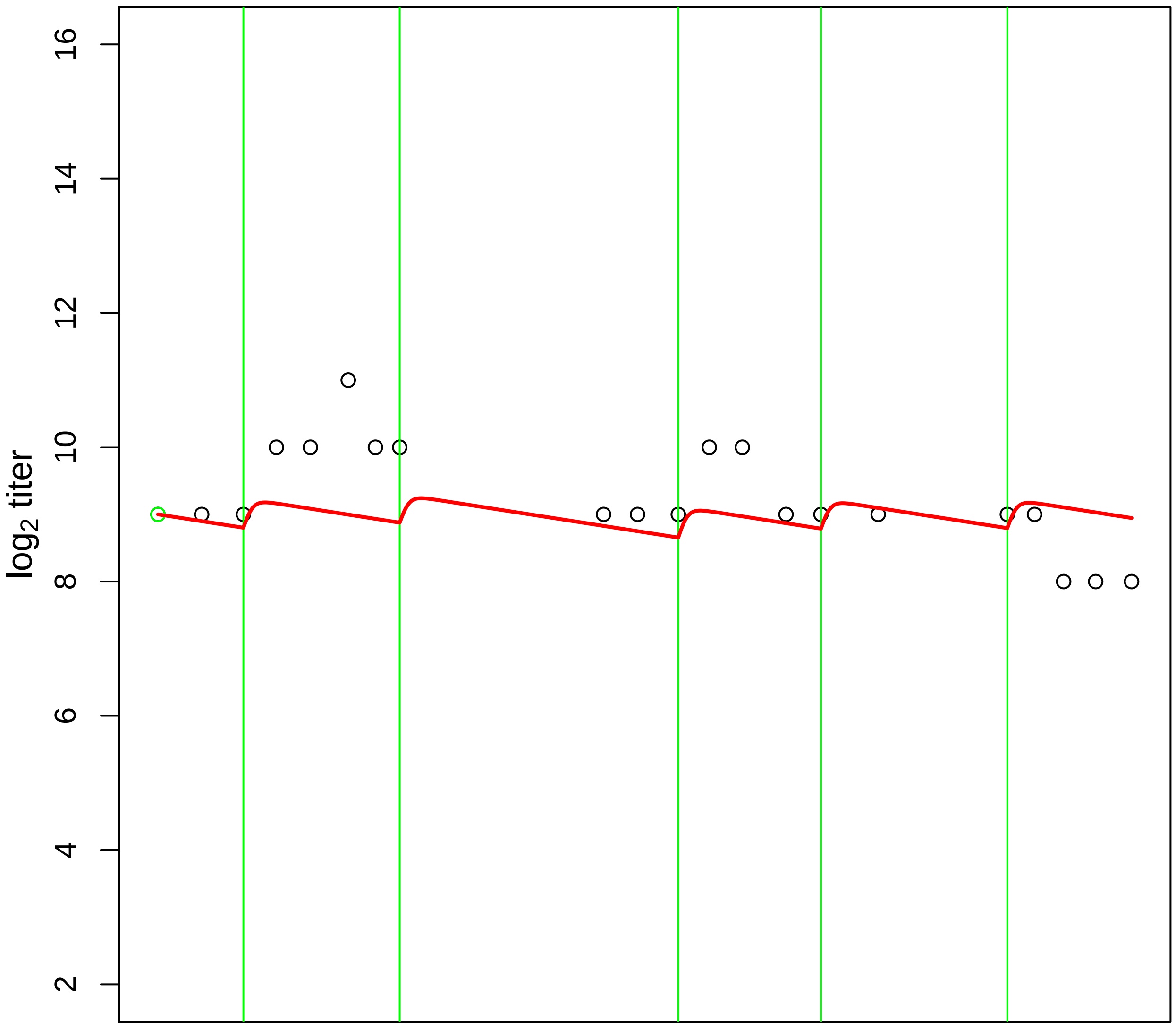

time in years from first donation of donor 466
mean absolute errors = 0.645 , mean squared errors = 0.675

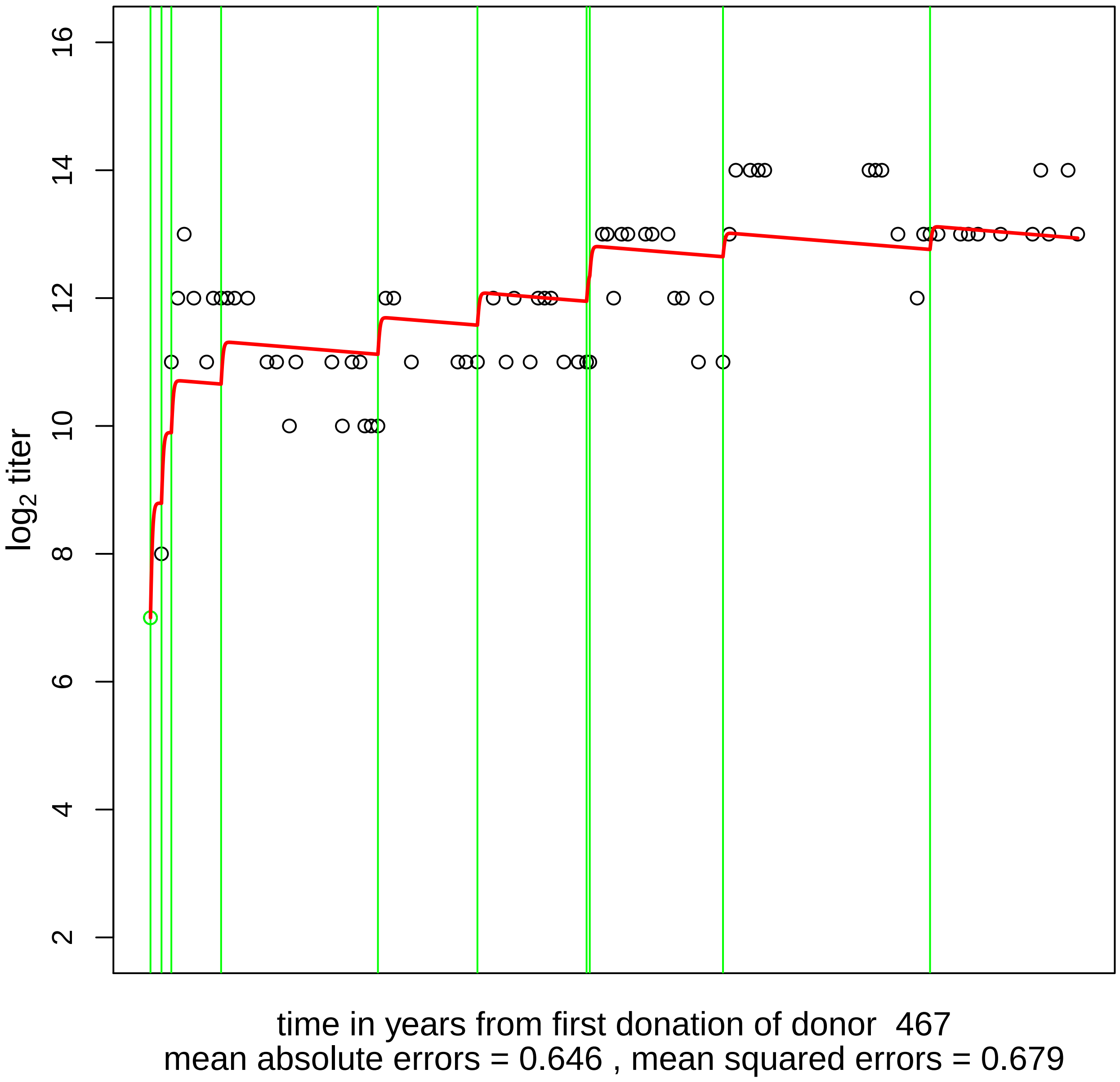

log2 titer
16
14
12
10
8
6
4
2
time in years from first donation of donor 467
mean absolute errors = 0.646 , mean squared errors = 0.679

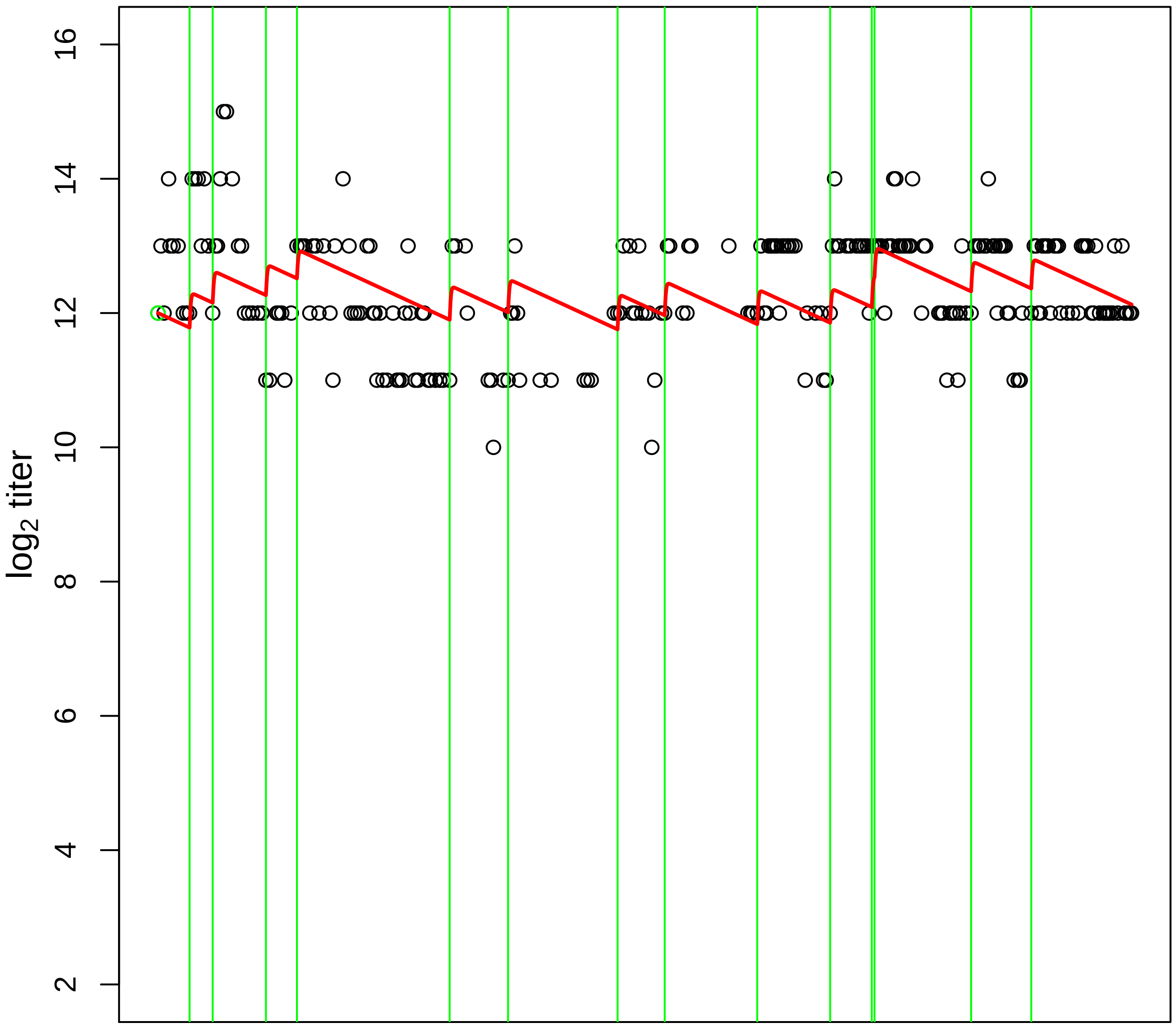

time in years from first donation of donor 468
mean absolute errors = 0.646 , mean squared errors = 0.644

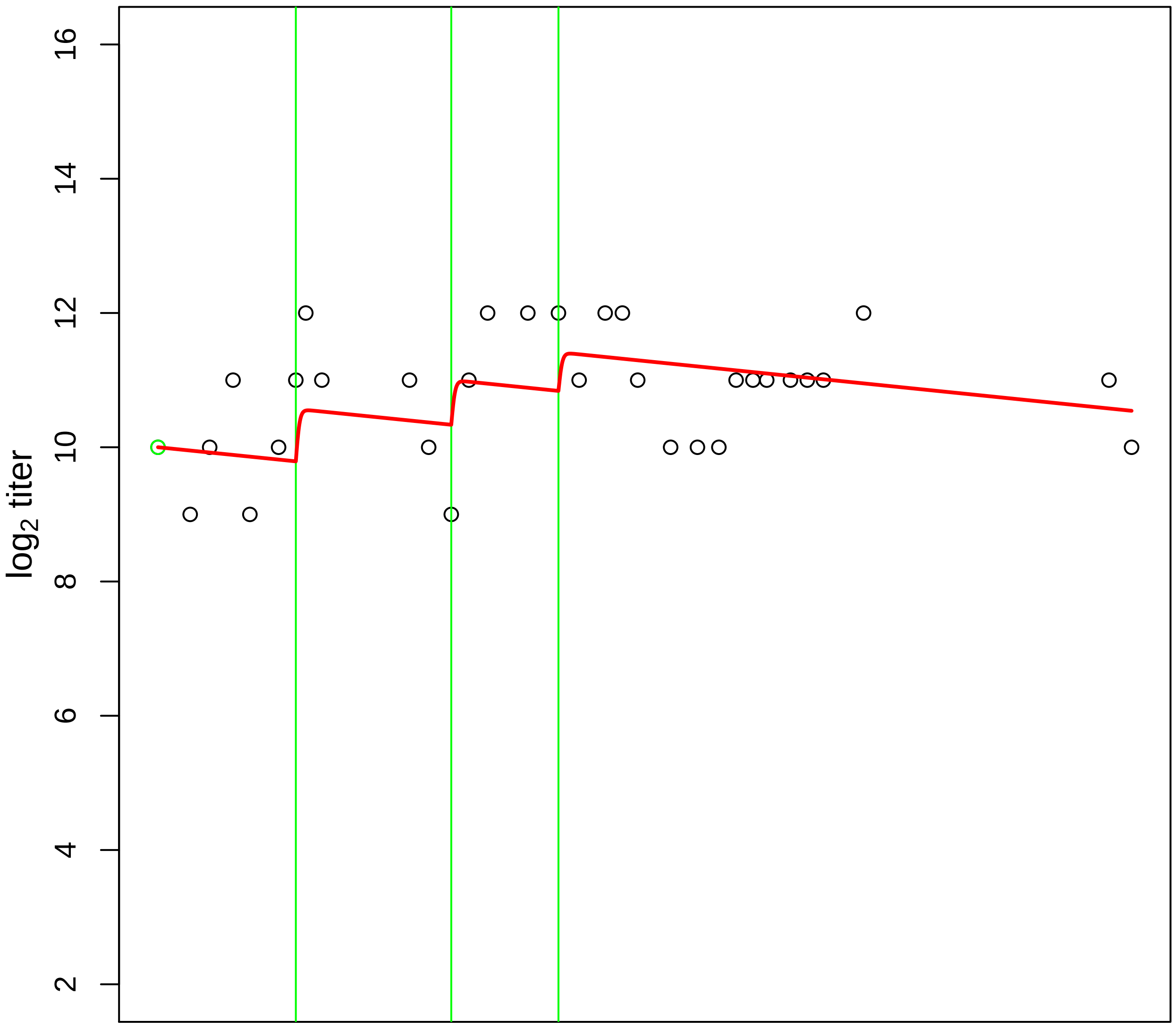

time in years from first donation of donor 469
mean absolute errors = 0.649 , mean squared errors = 0.639

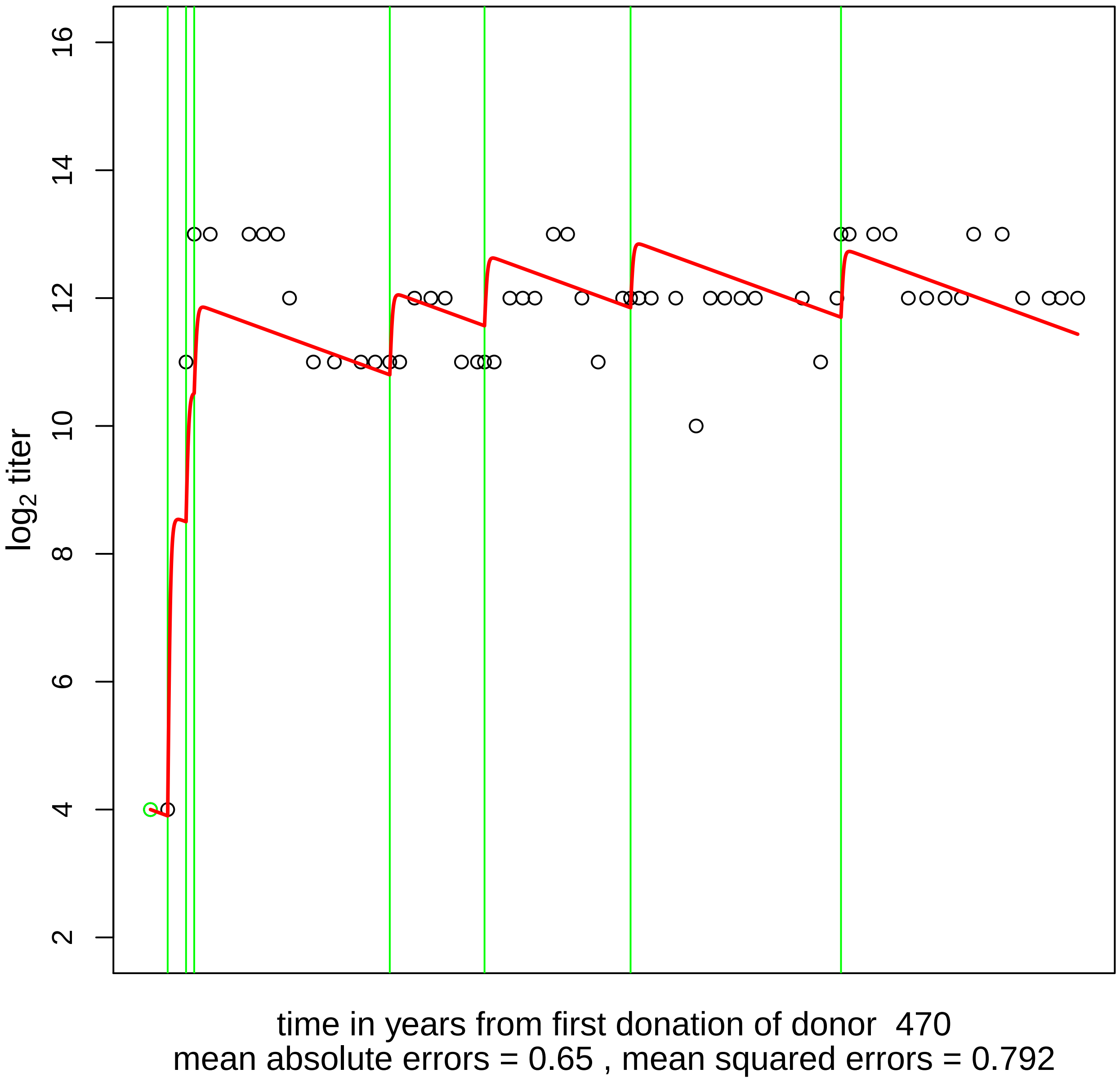

16
14
12
10
8
6
4
2
log2 titer
time in years from first donation of donor 470
mean absolute errors = 0.65 , mean squared errors = 0.792

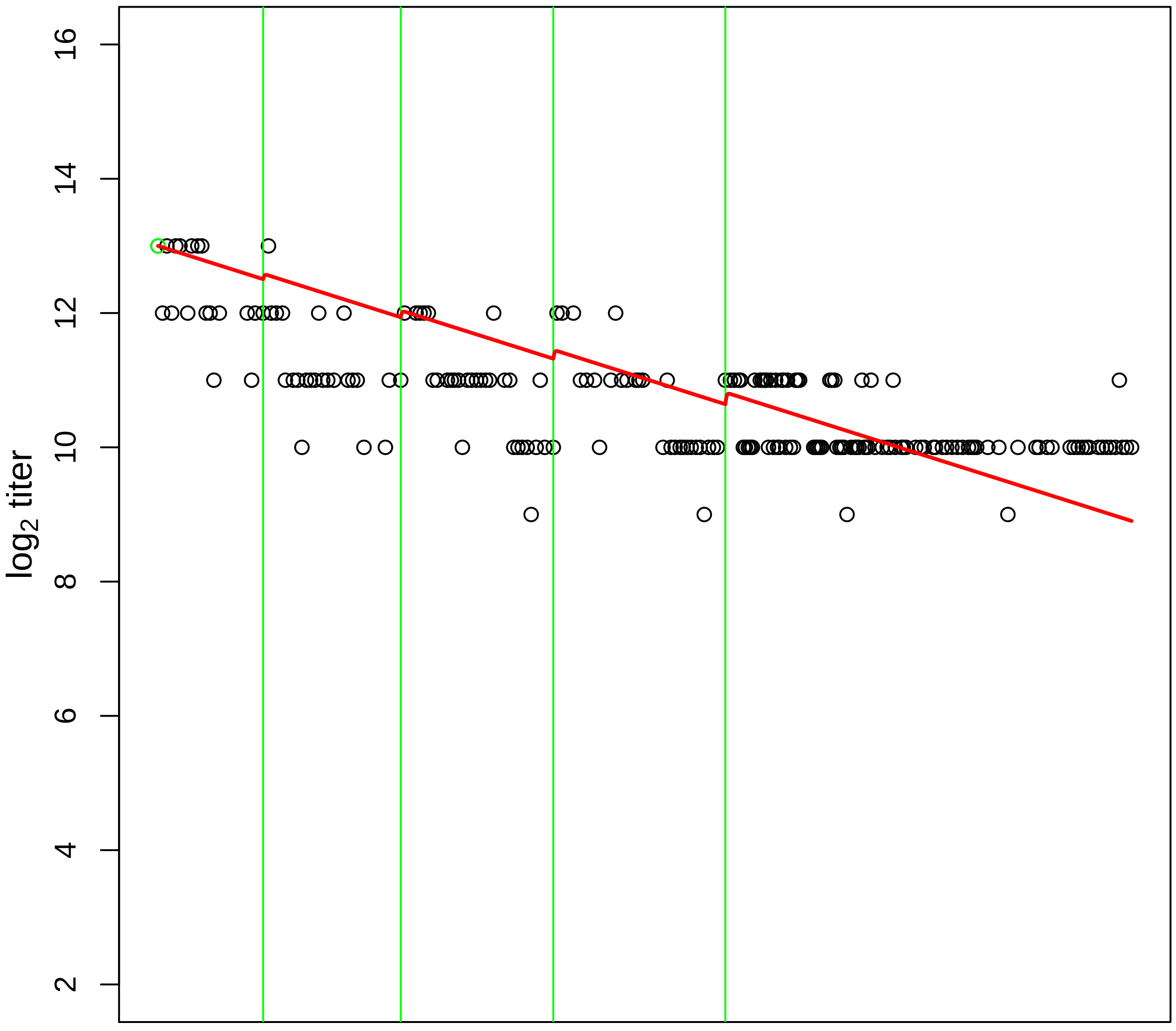

time in years from first donation of donor 471
mean absolute errors = 0.651 , mean squared errors = 0.657

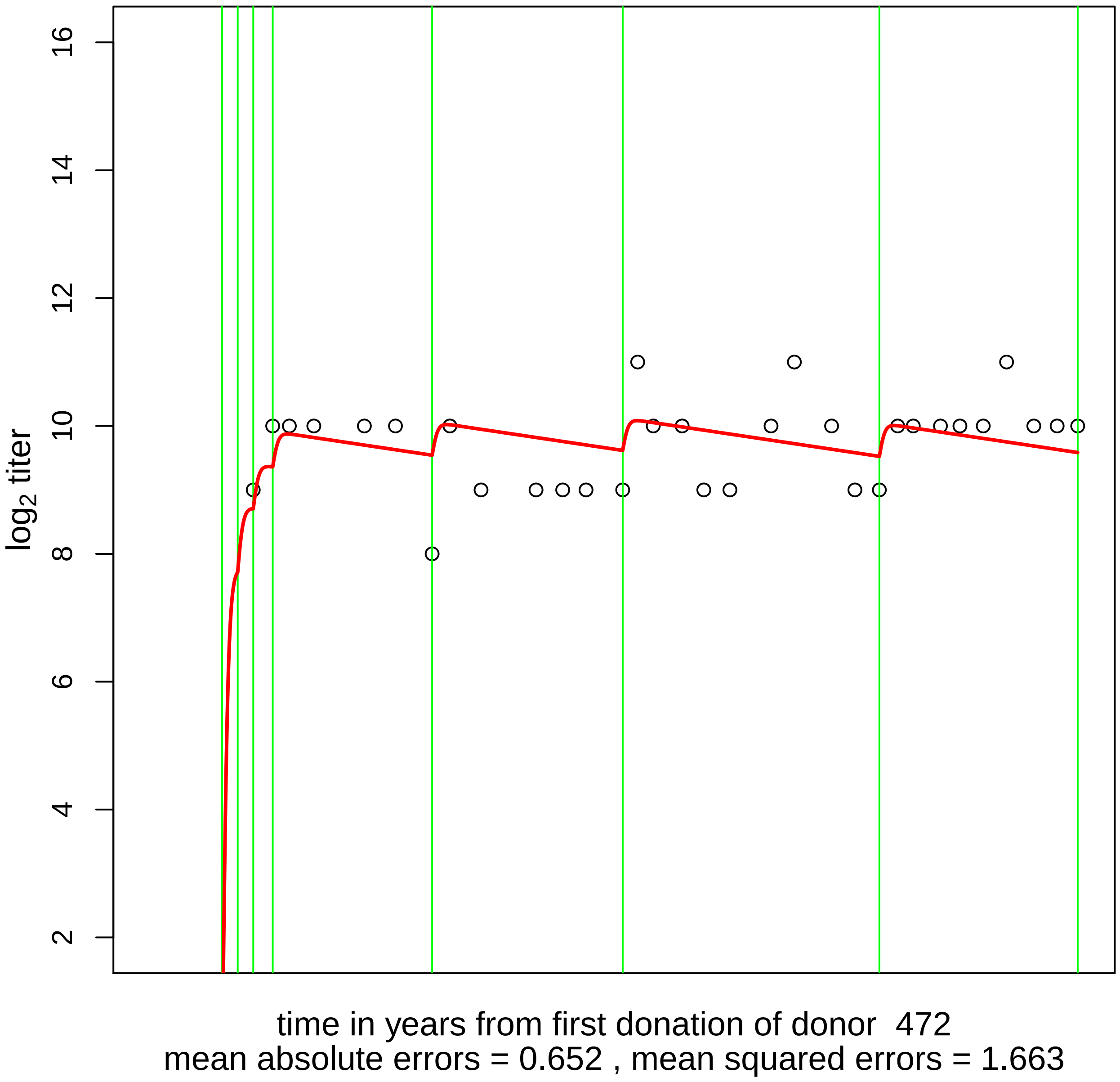

$\log_2$ titer
2
4
6
8
10
12
14
16
time in years from first donation of donor 472
mean absolute errors = 0.652 , mean squared errors = 1.663

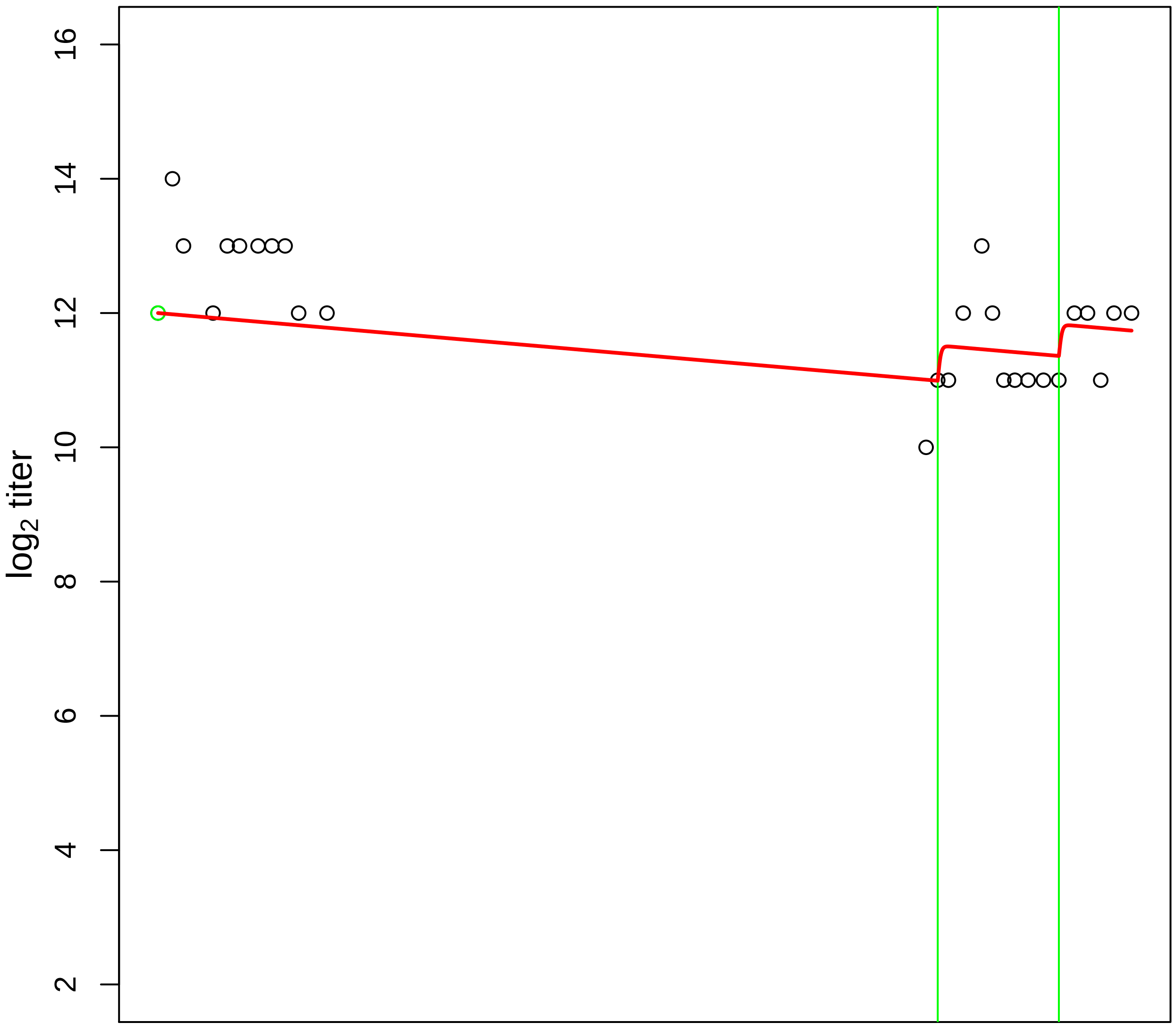

time in years from first donation of donor 473
mean absolute errors = 0.652 , mean squared errors = 0.669

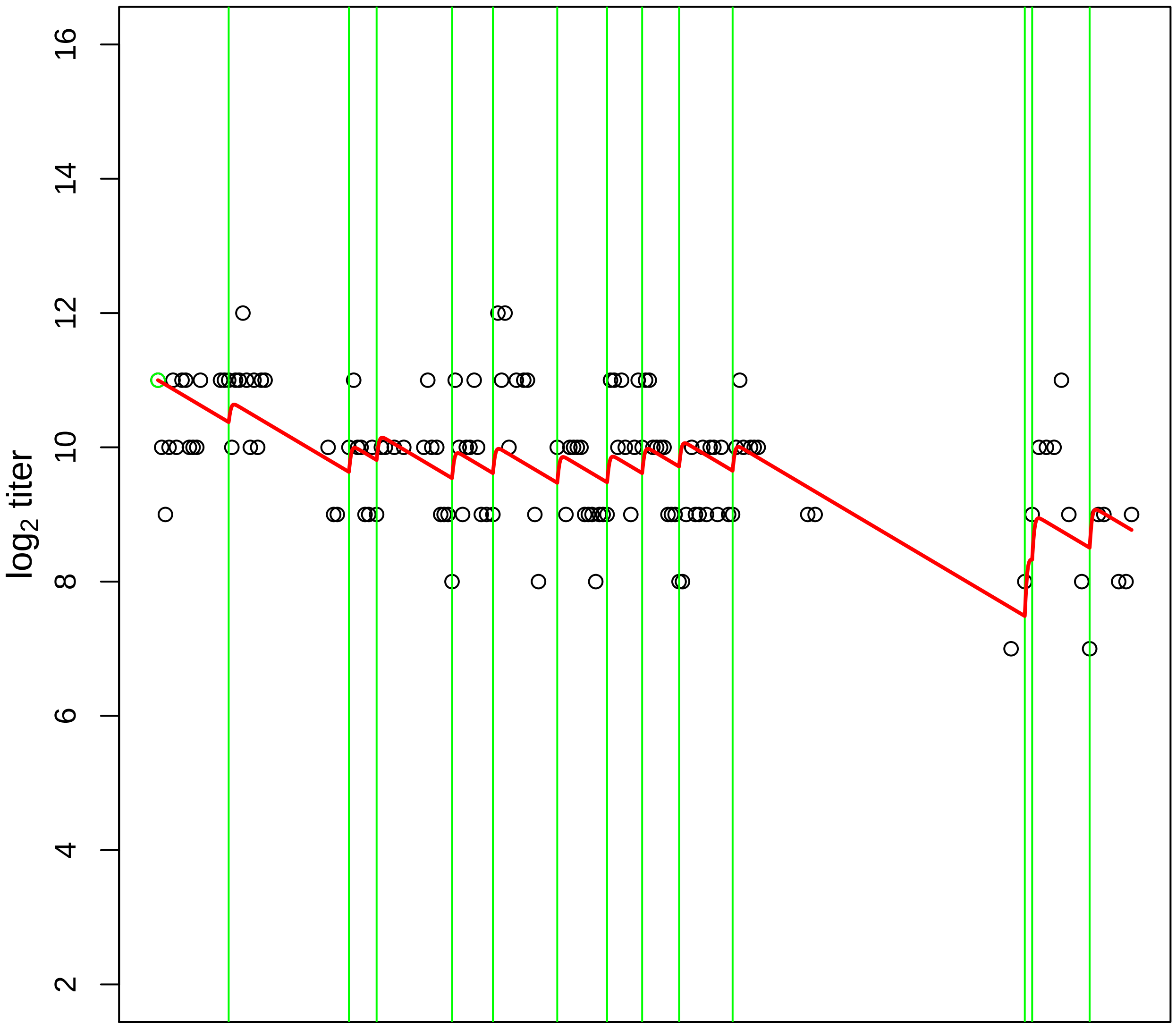

time in years from first donation of donor 474
mean absolute errors = 0.653 , mean squared errors = 0.67

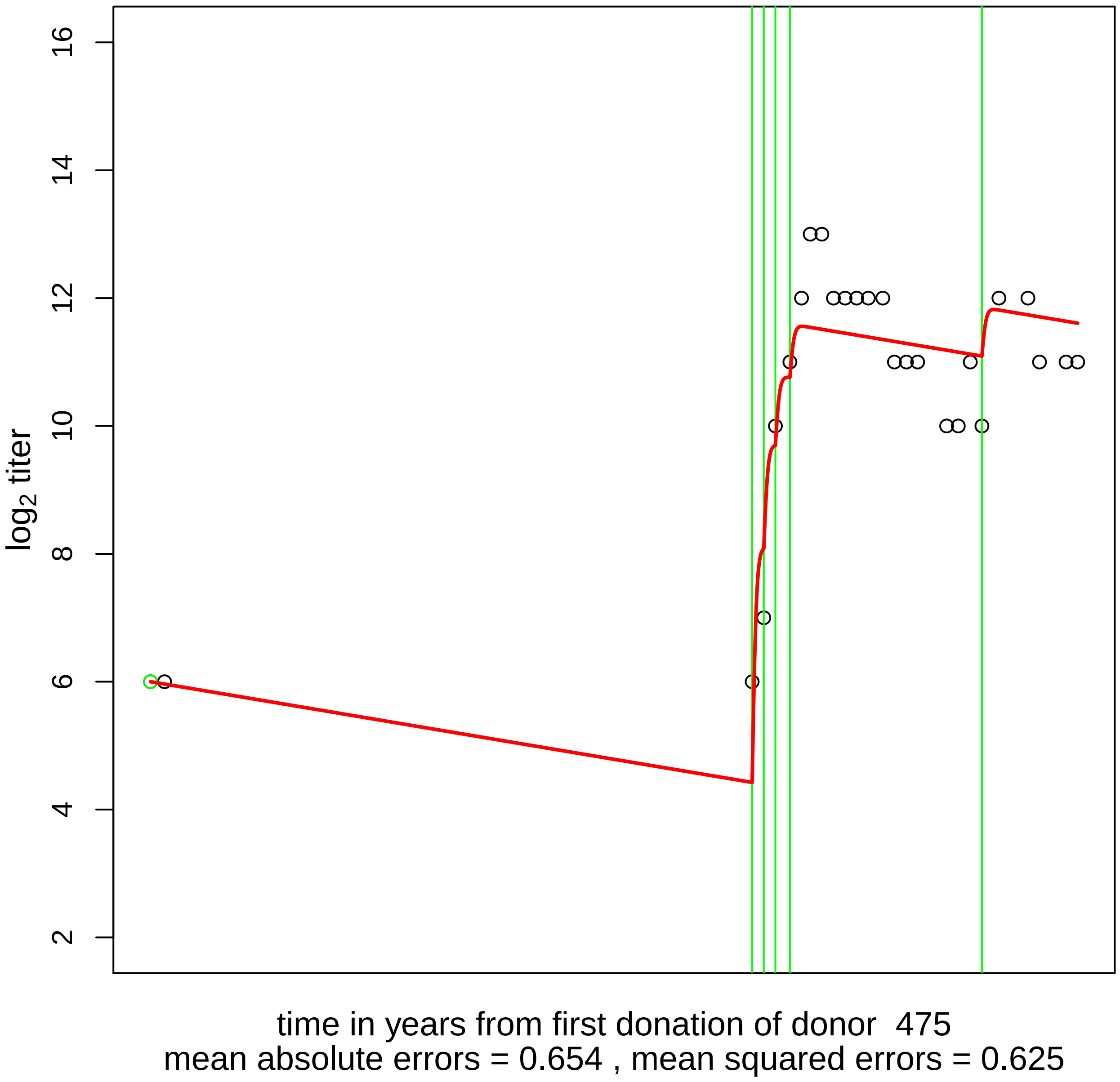

16
14
12
10
8
6
4
2
$\log_2$ titer
time in years from first donation of donor 475
mean absolute errors = 0.654 , mean squared errors = 0.625

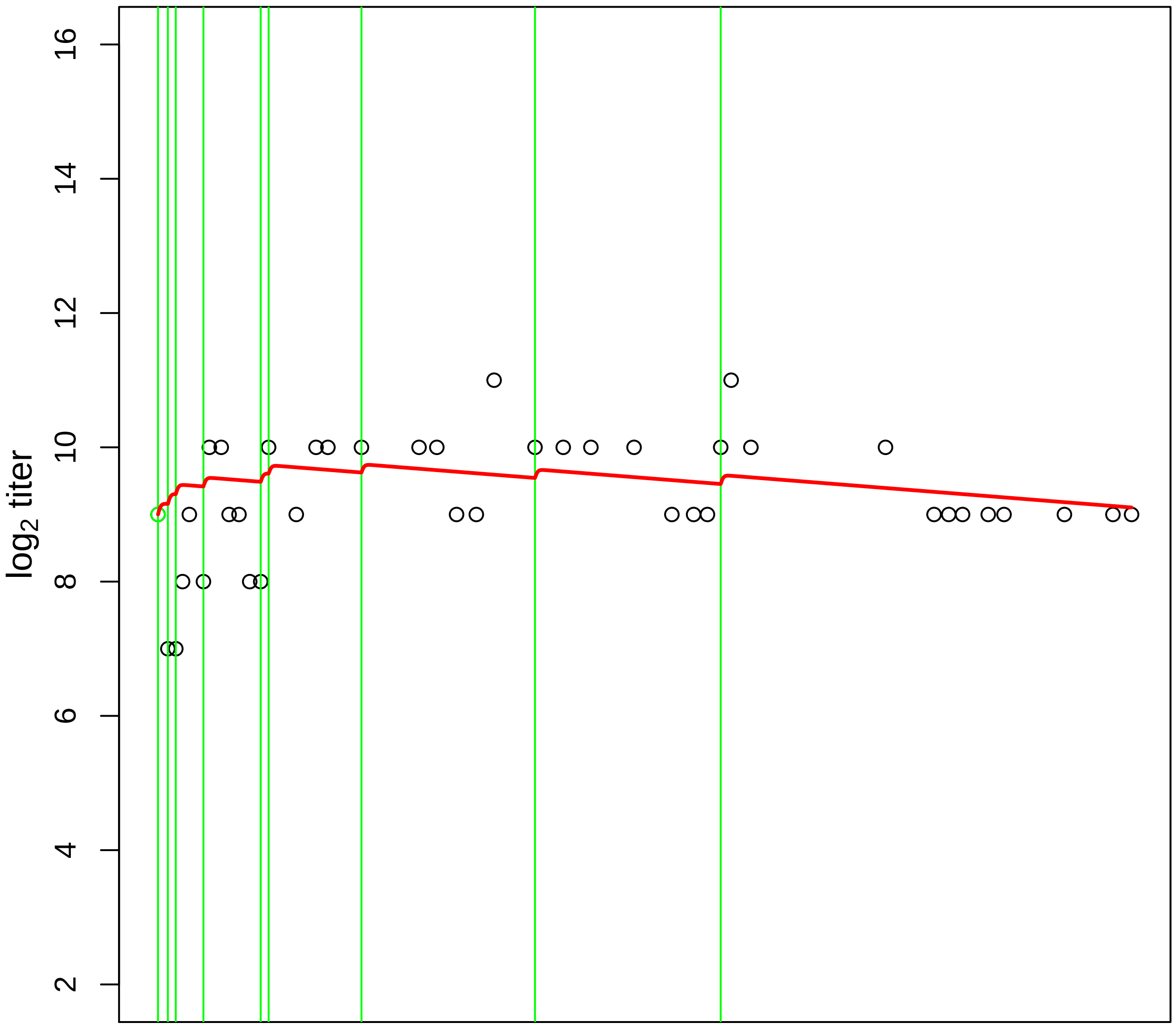

time in years from first donation of donor 476
mean absolute errors = 0.655 , mean squared errors = 0.711

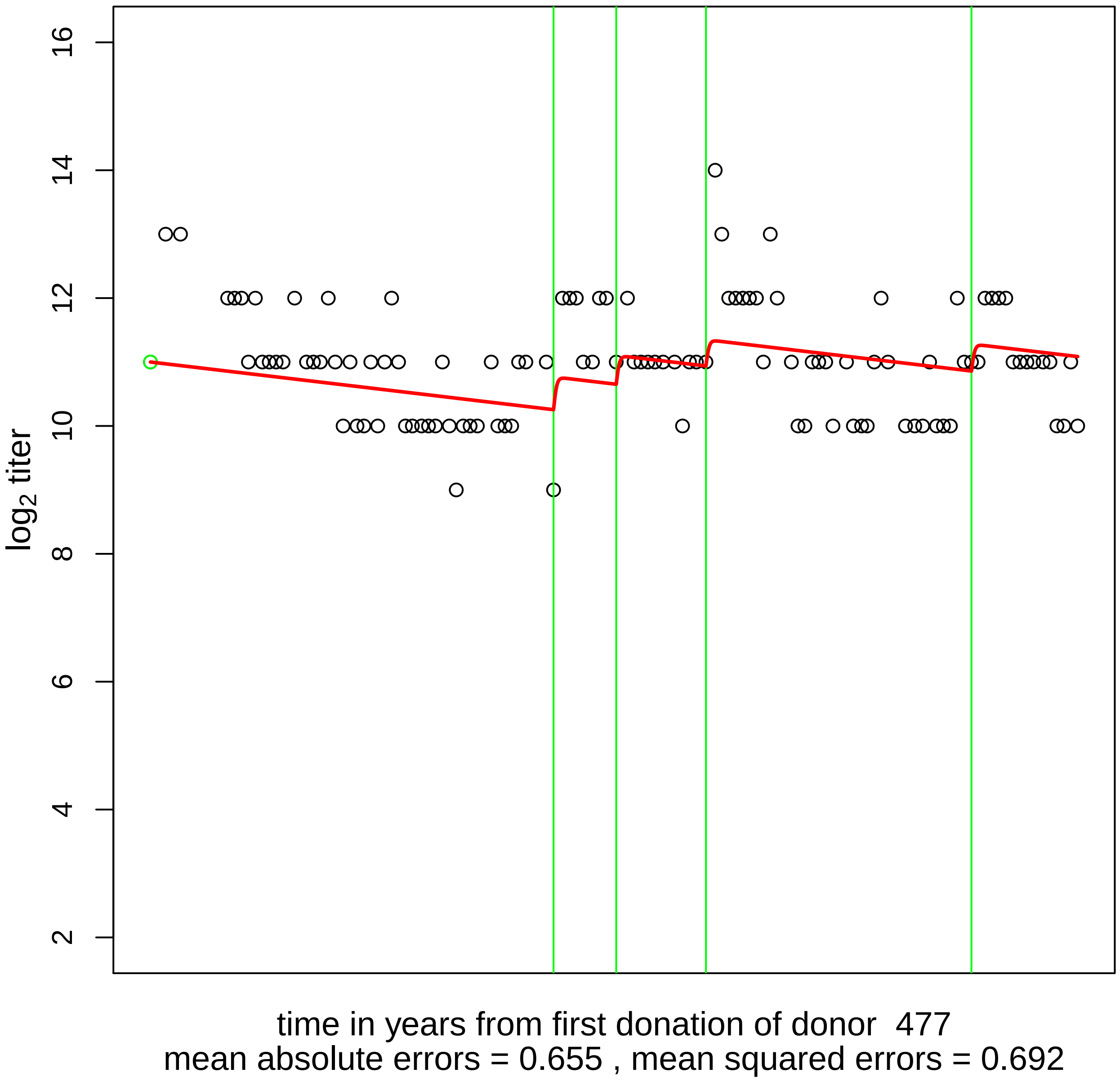

log2 titer
2
4
6
8
10
12
14
16
time in years from first donation of donor 477
mean absolute errors = 0.655 , mean squared errors = 0.692

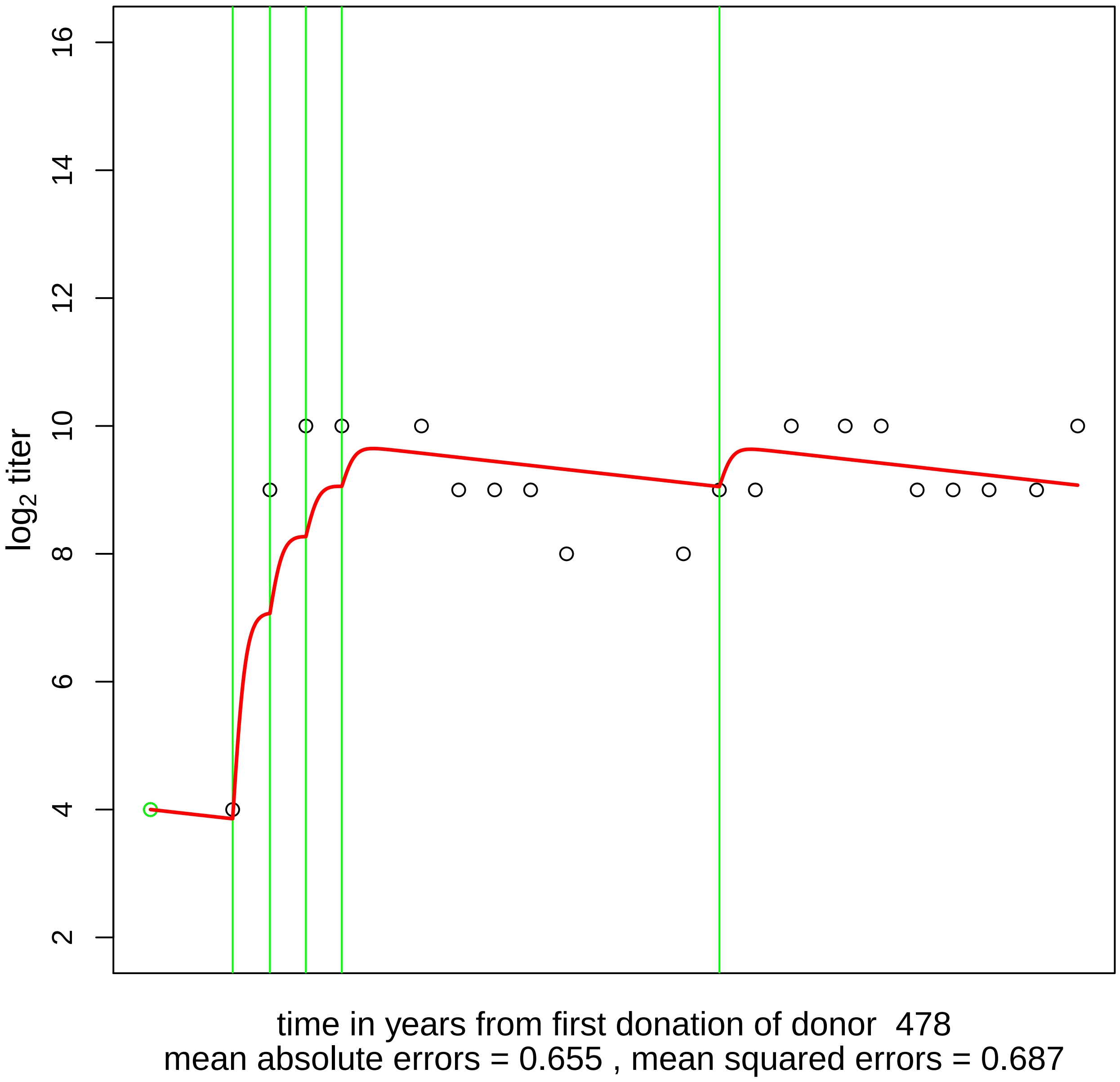

log₂ titer
2
4
6
8
10
12
14
16
time in years from first donation of donor 478
mean absolute errors = 0.655 , mean squared errors = 0.687

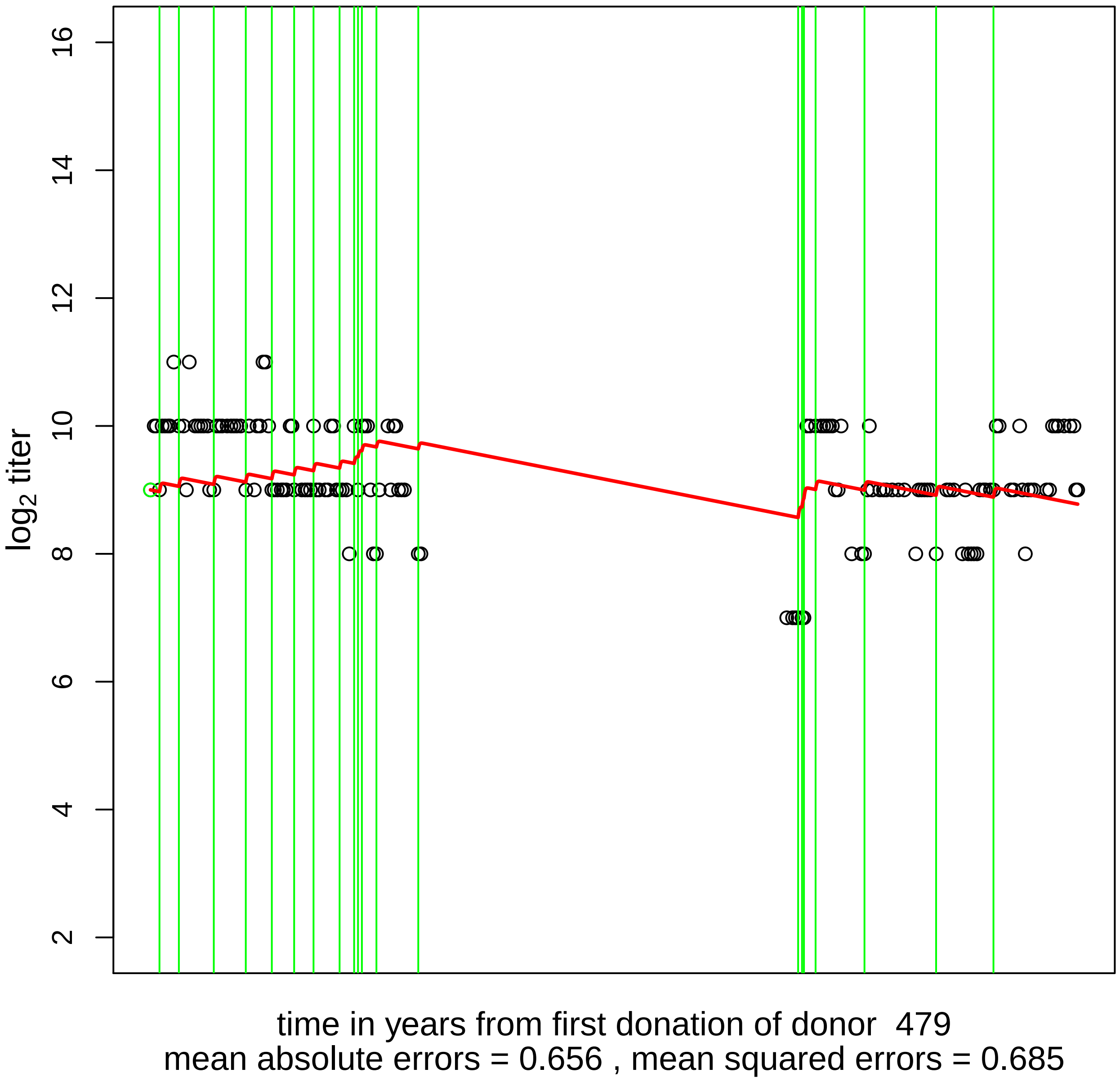

2
4
6
8
10
12
14
16
log2 titer
time in years from first donation of donor 479
mean absolute errors = 0.656 , mean squared errors = 0.685

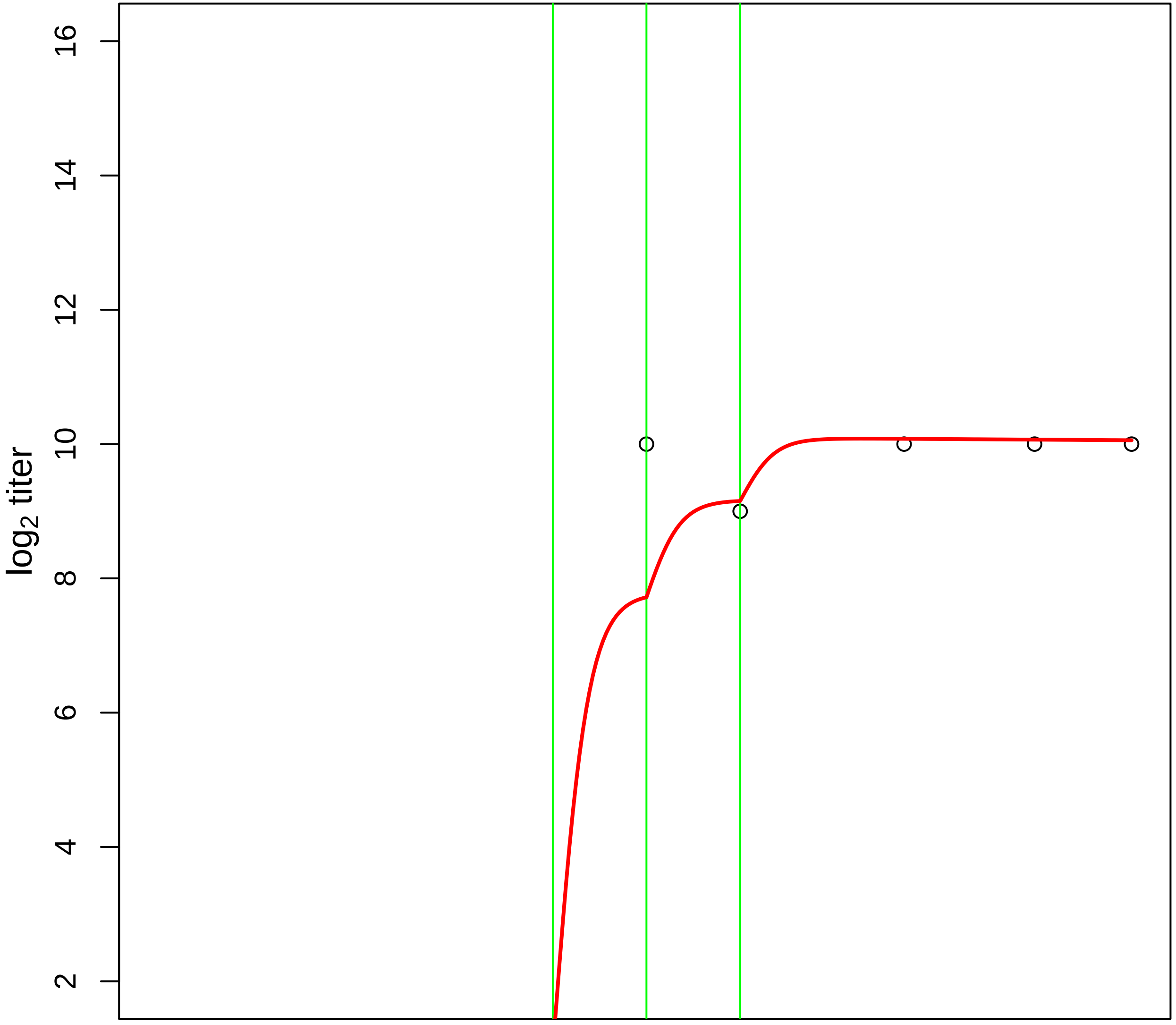

time in years from first donation of donor 480
mean absolute errors = 0.656 , mean squared errors = 1.023

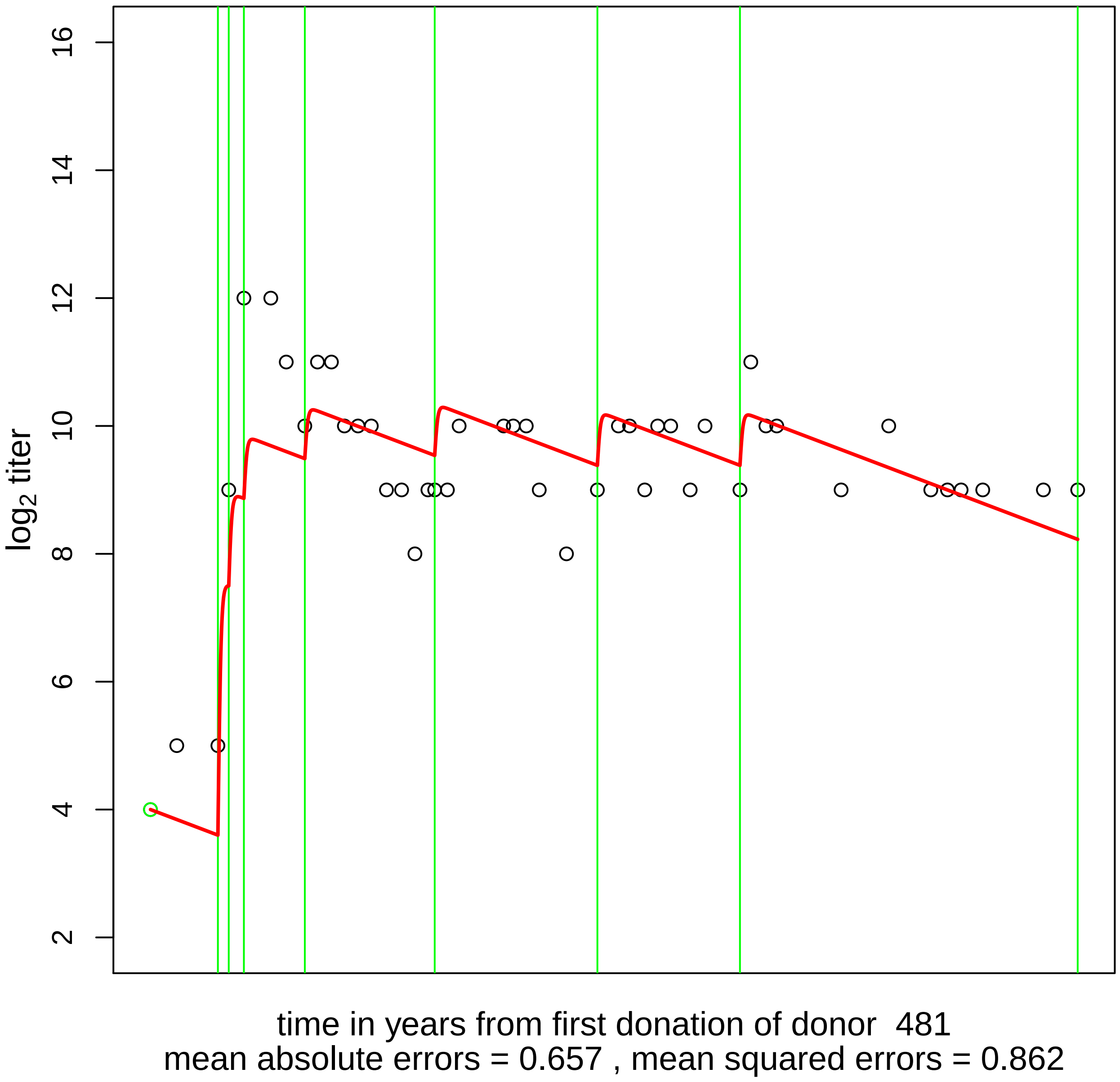

16
14
12
10
8
6
4
2
$\log_2$ titer
time in years from first donation of donor 481
mean absolute errors = 0.657 , mean squared errors = 0.862

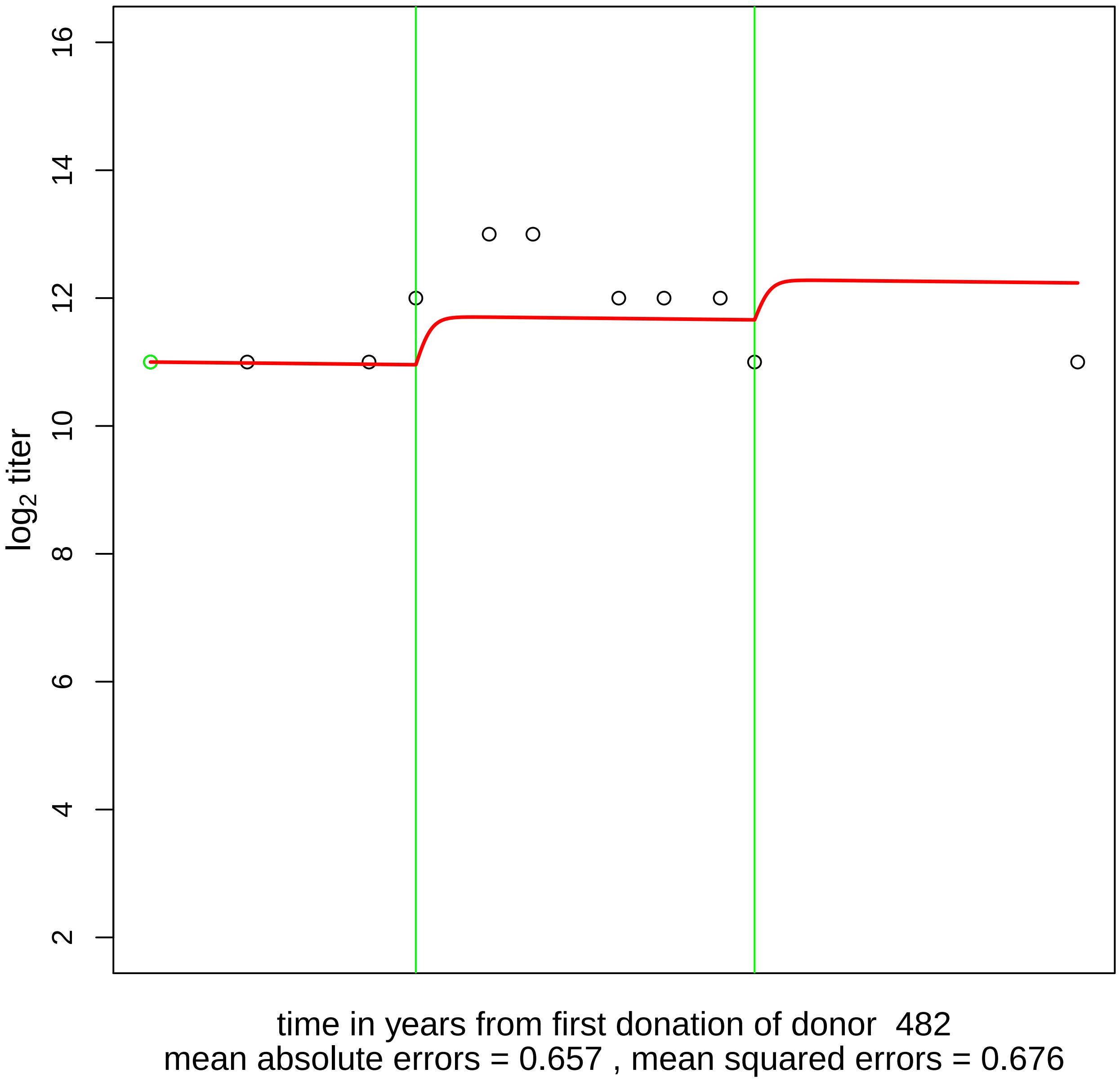

2
4
6
8
10
12
14
16
log2 titer
time in years from first donation of donor 482
mean absolute errors = 0.657 , mean squared errors = 0.676

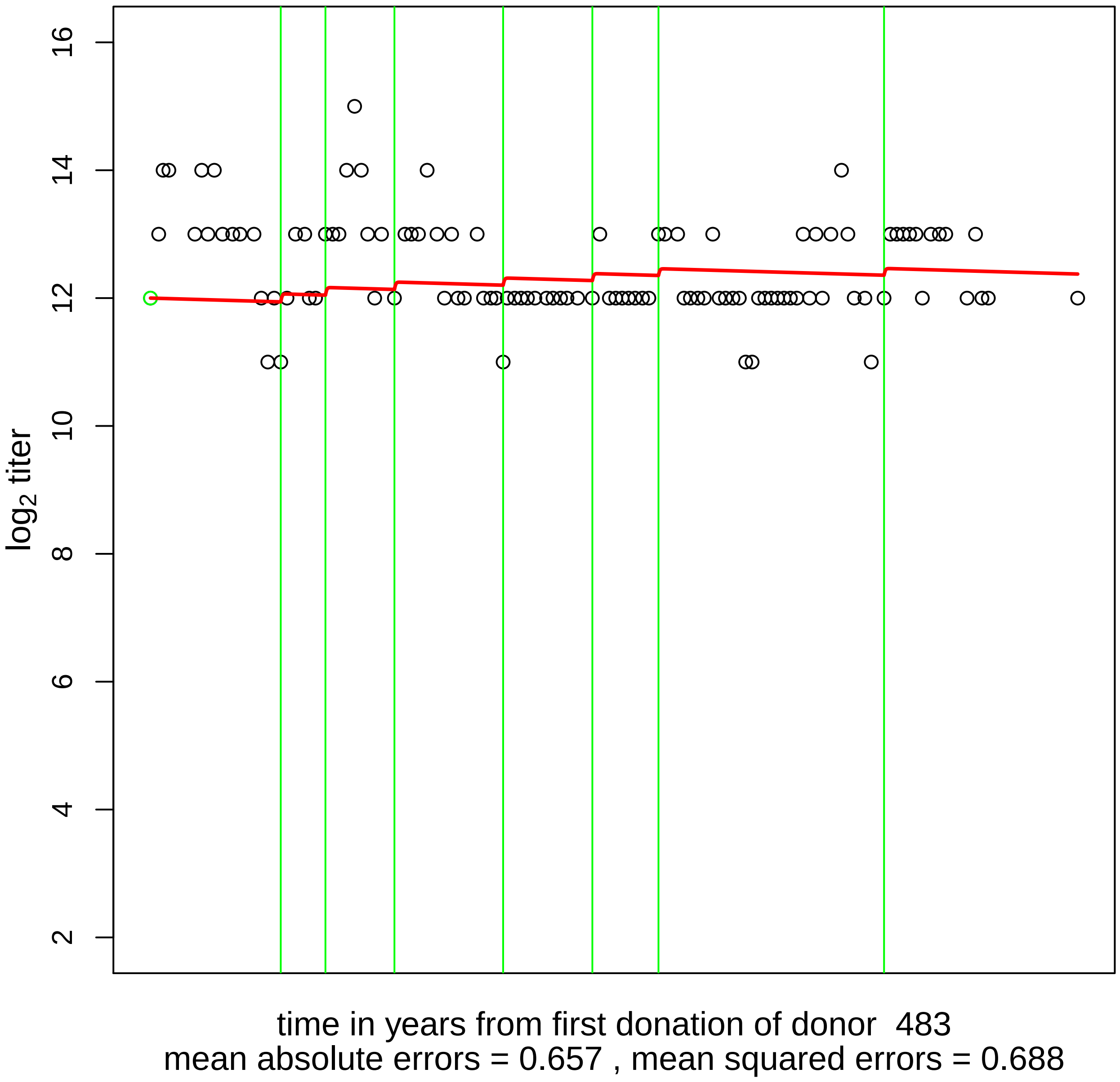

16
14
12
10
8
6
4
2
$\log_2$ titer
time in years from first donation of donor 483
mean absolute errors = 0.657 , mean squared errors = 0.688

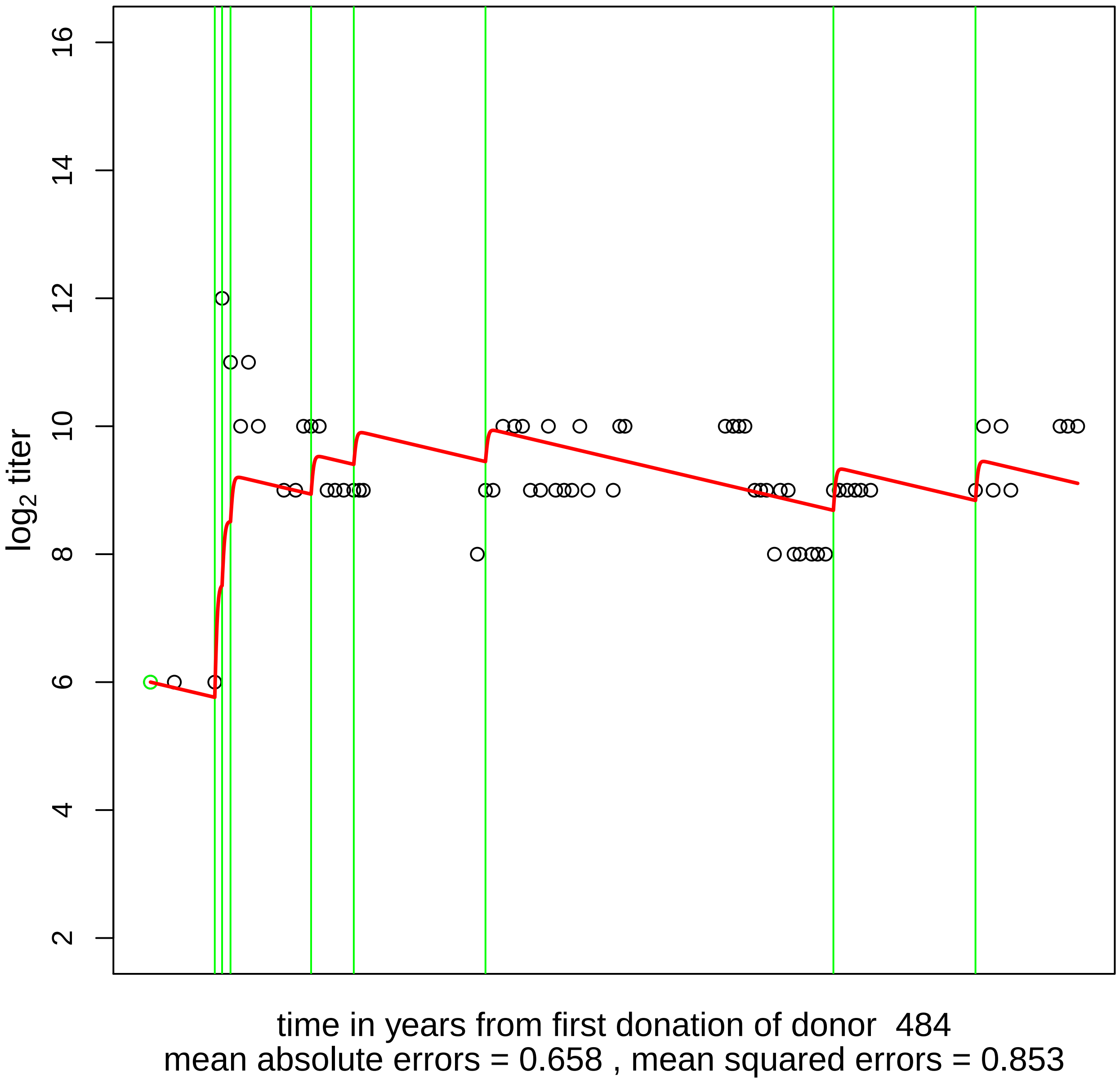

2
4
6
8
10
12
14
16
$\log_2$ titer
time in years from first donation of donor 484
mean absolute errors = 0.658 , mean squared errors = 0.853

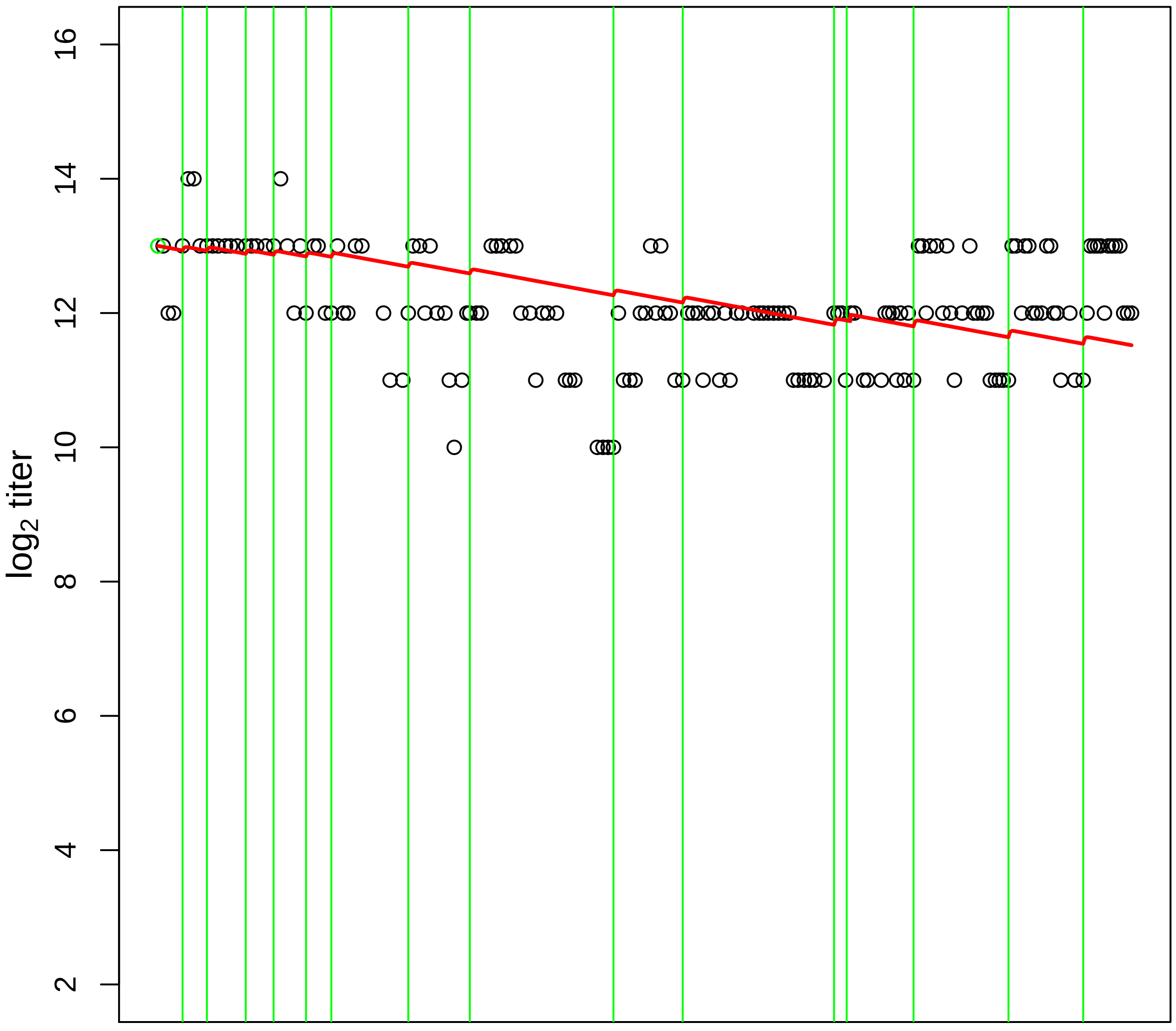

time in years from first donation of donor 485
mean absolute errors = 0.66 , mean squared errors = 0.745

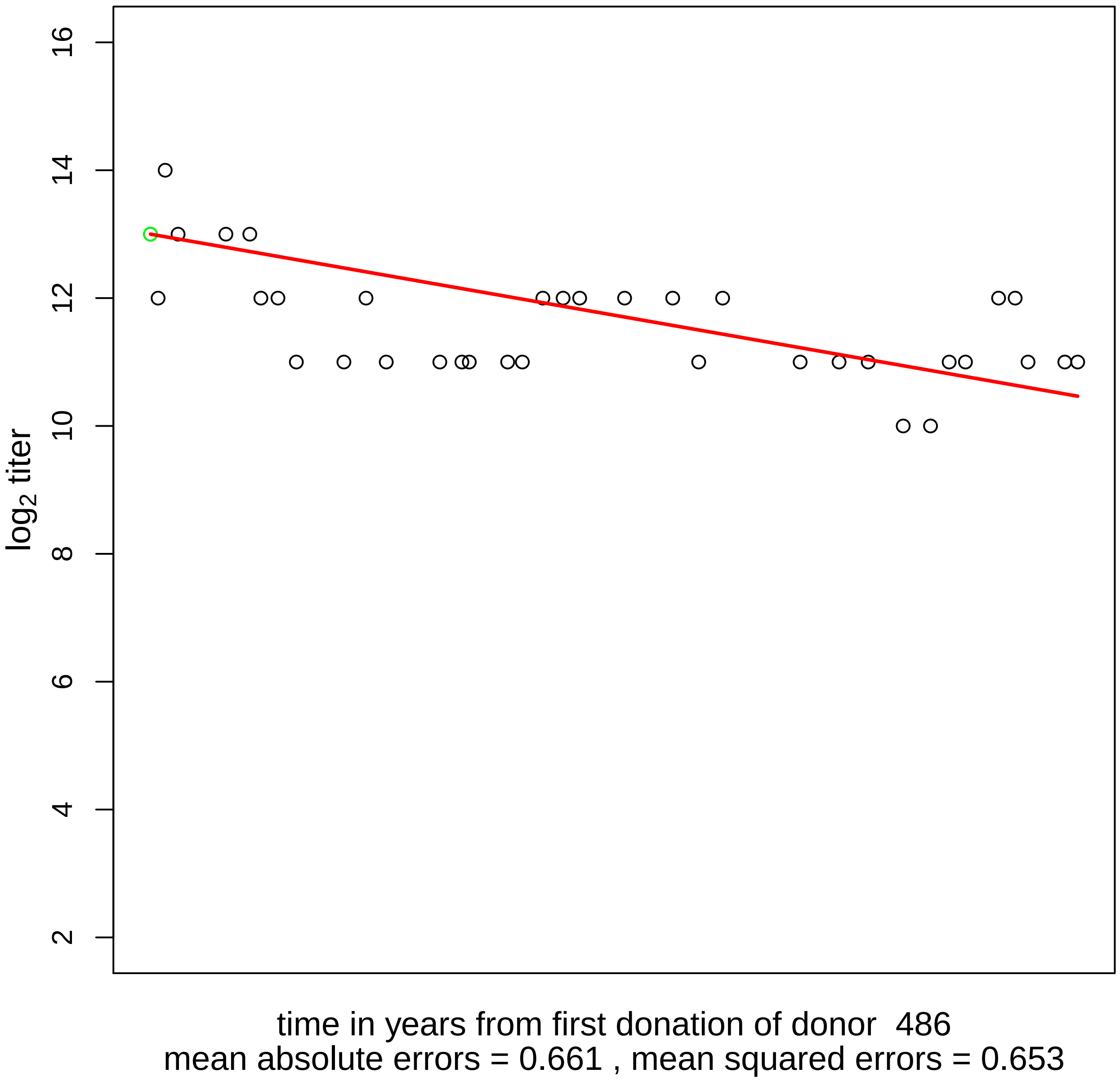

16
14
12
10
8
6
4
2
log2 titer
time in years from first donation of donor 486
mean absolute errors = 0.661 , mean squared errors = 0.653

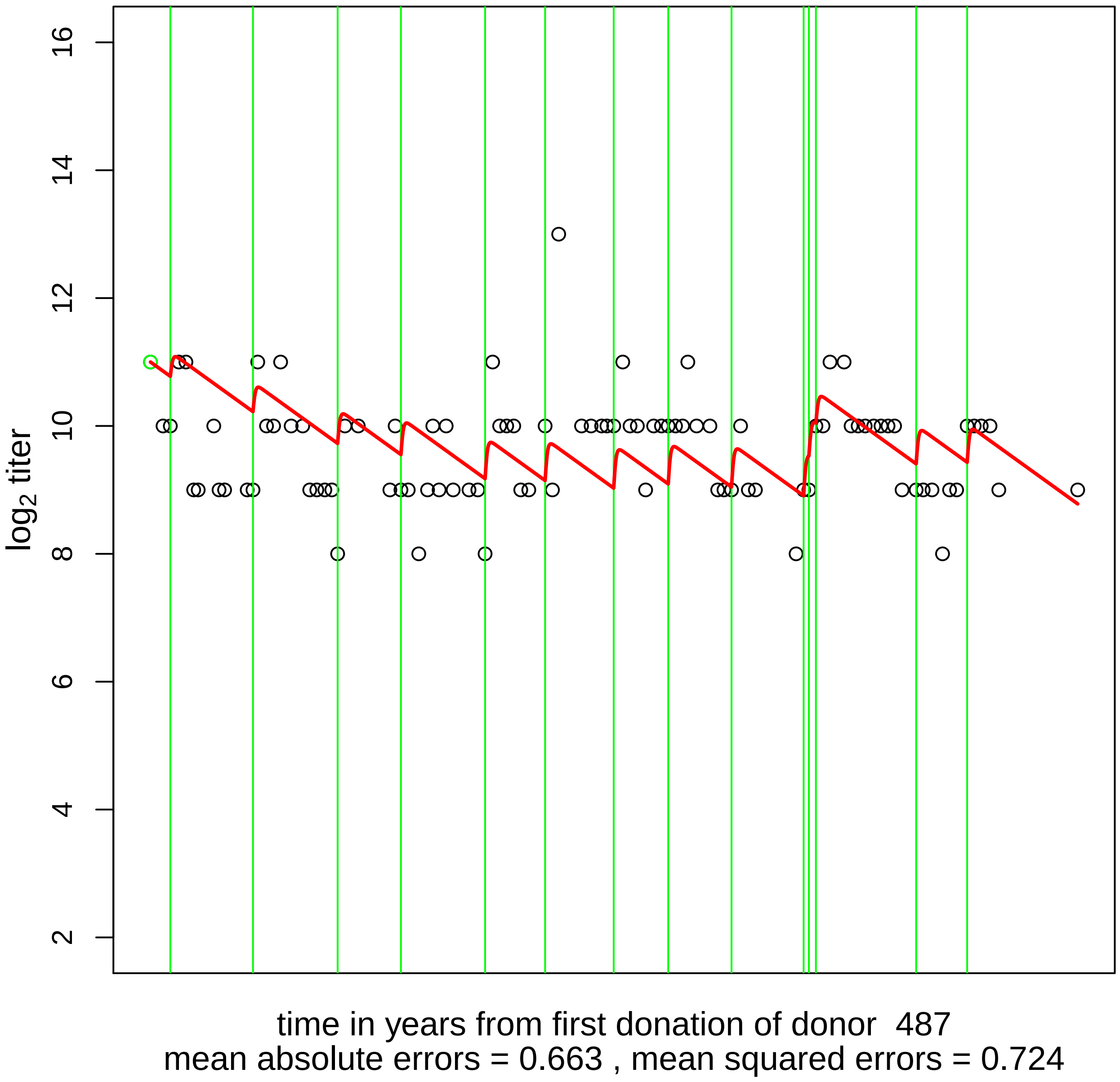

$\log_2$ titer
2
4
6
8
10
12
14
16
time in years from first donation of donor 487
mean absolute errors = 0.663 , mean squared errors = 0.724

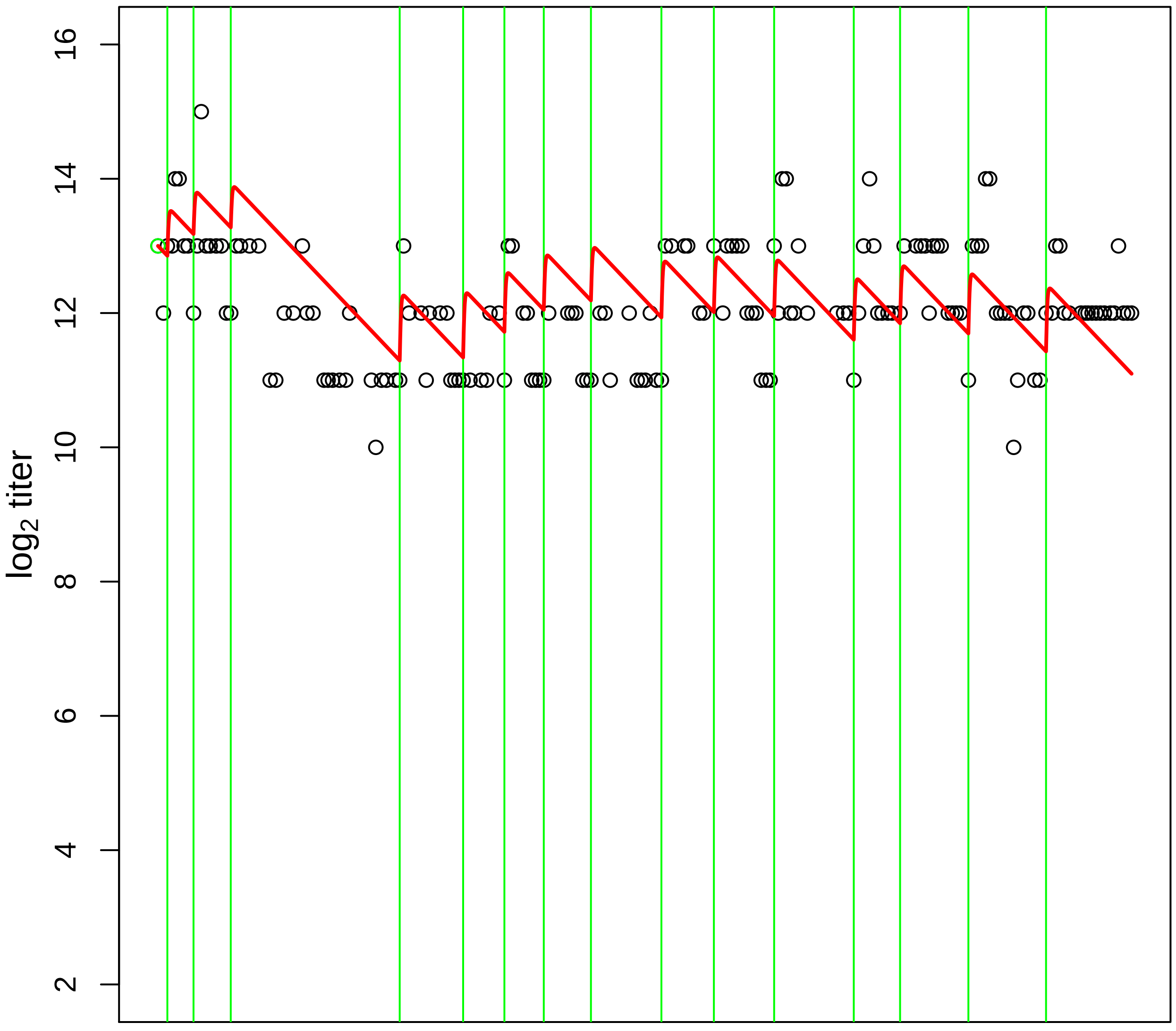

time in years from first donation of donor 488
mean absolute errors = 0.663 , mean squared errors = 0.658

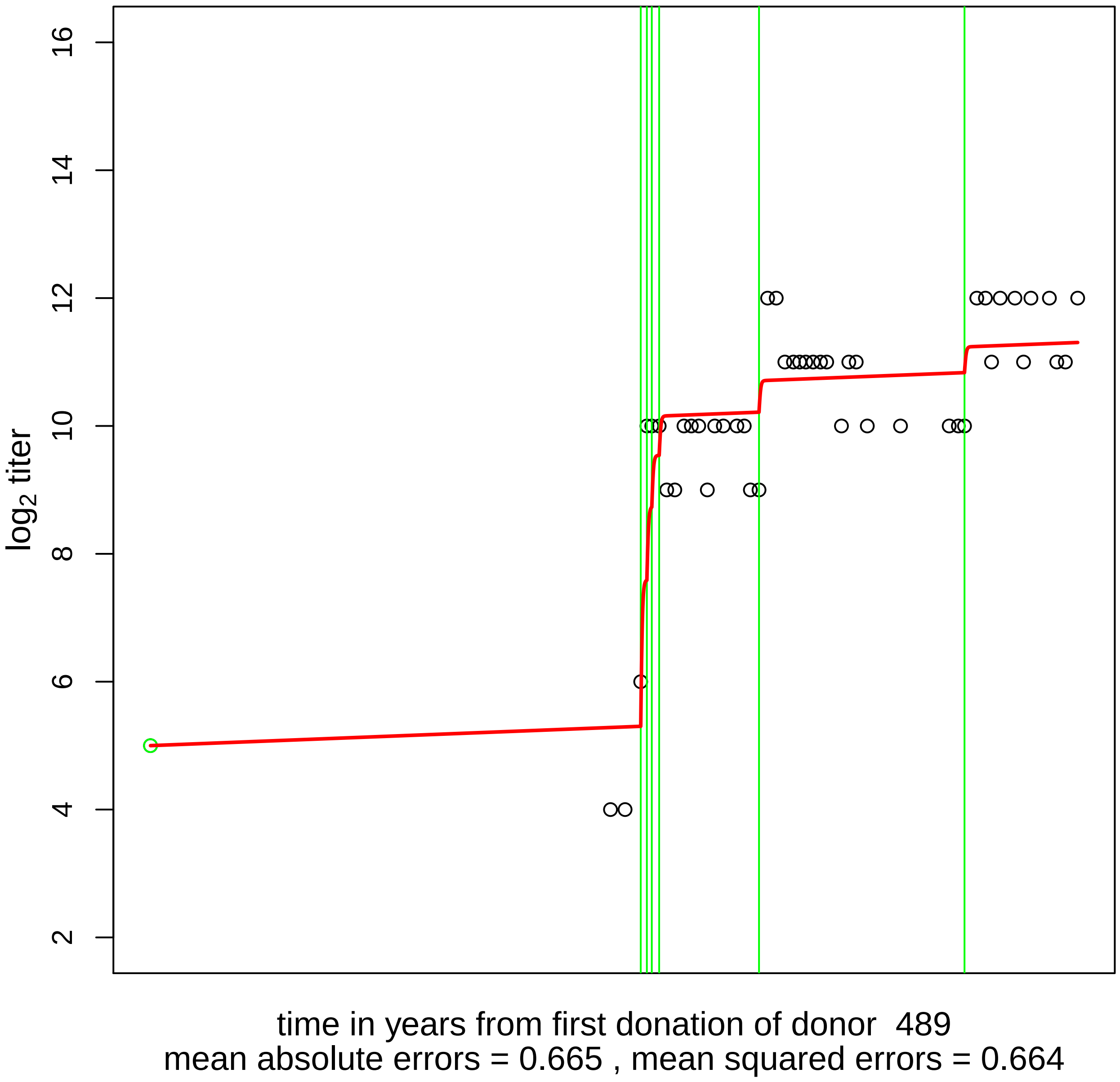

2
4
6
8
10
12
14
16
log2 titer
time in years from first donation of donor 489
mean absolute errors = 0.665 , mean squared errors = 0.664

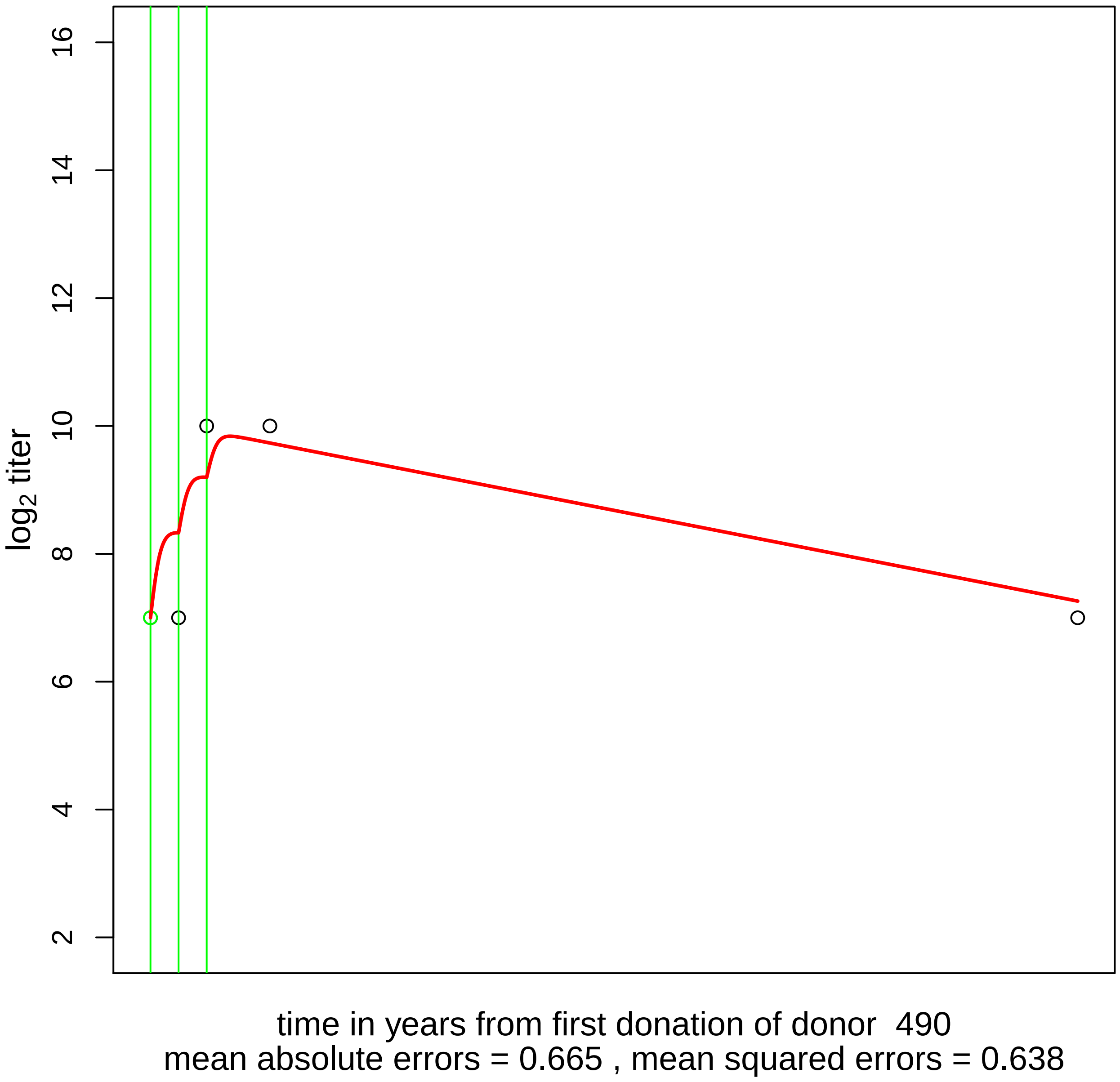

log2 titer
16
14
12
10
8
6
4
2
time in years from first donation of donor 490
mean absolute errors = 0.665 , mean squared errors = 0.638

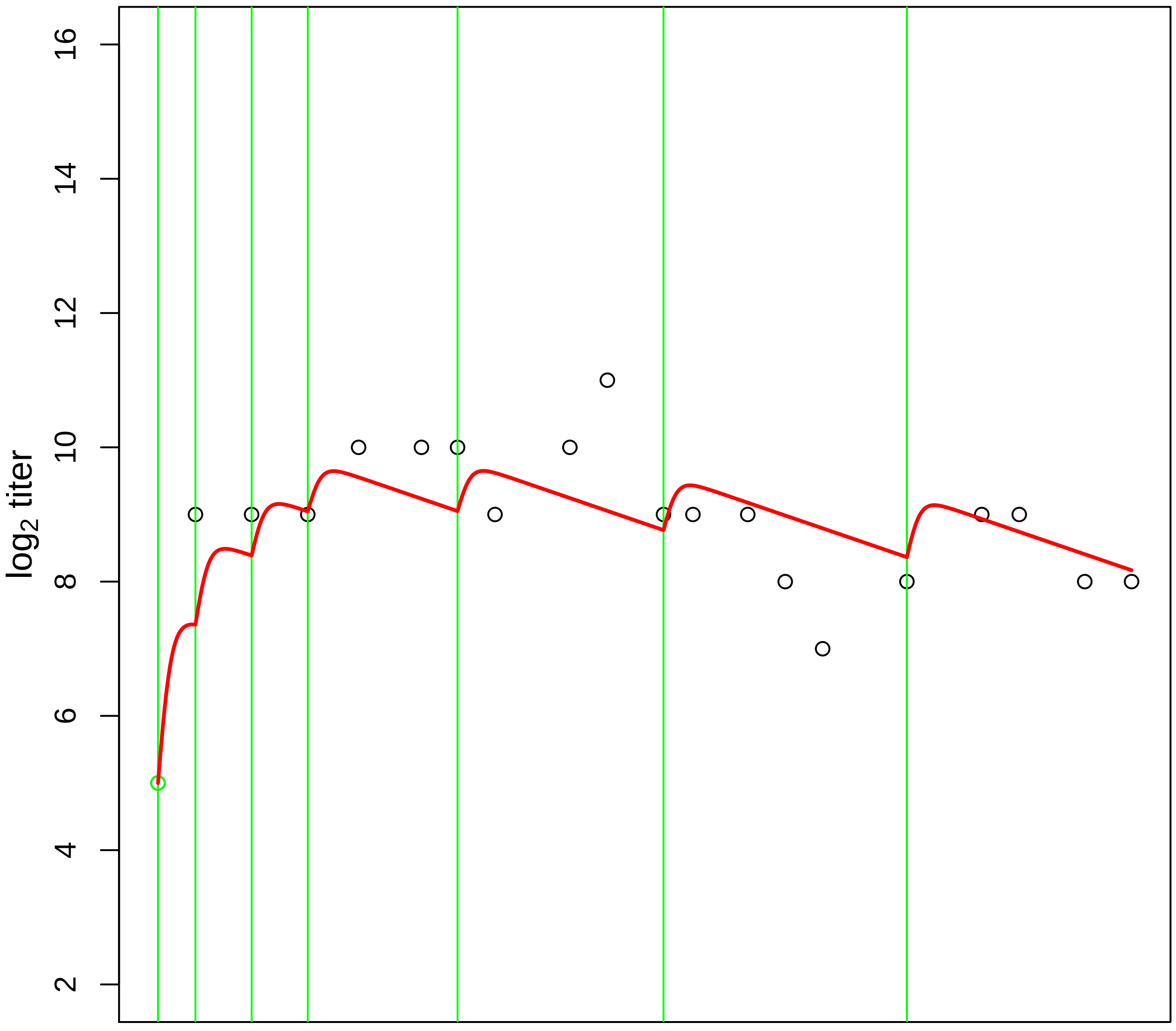

time in years from first donation of donor 491
mean absolute errors = 0.667 , mean squared errors = 0.756

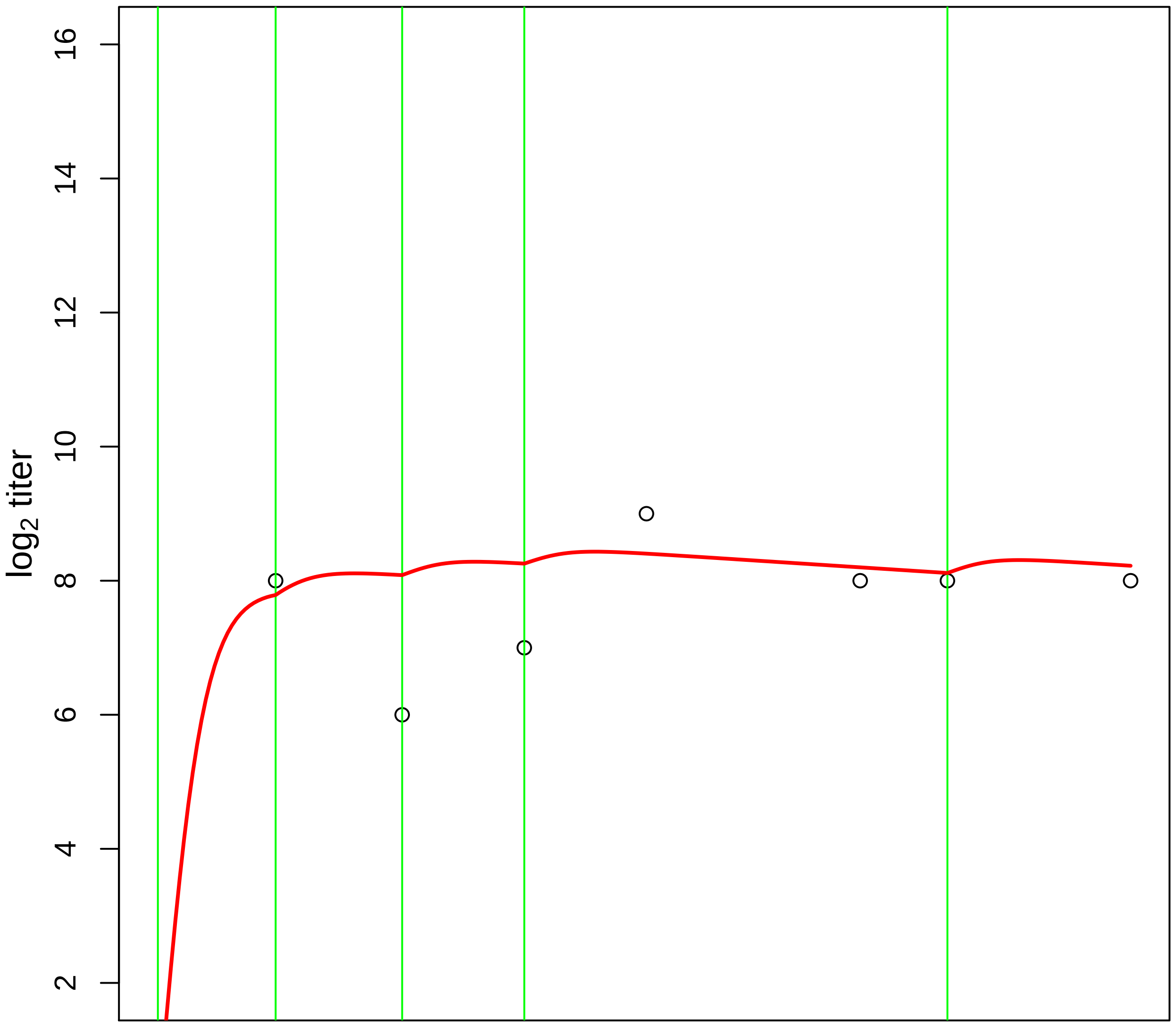

time in years from first donation of donor 492
mean absolute errors = 0.669 , mean squared errors = 0.917

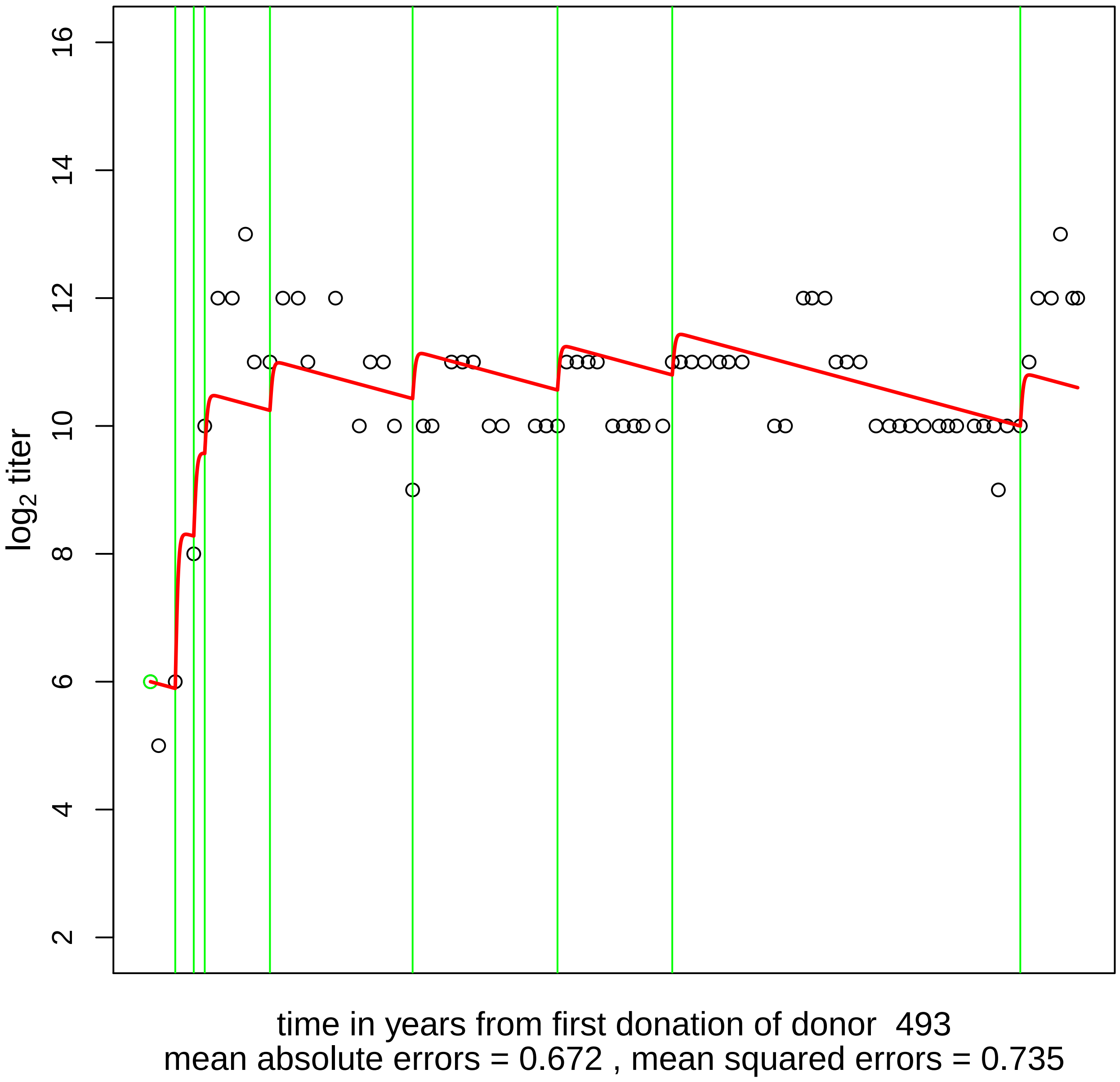

log2 titer
2
4
6
8
10
12
14
16
time in years from first donation of donor 493
mean absolute errors = 0.672 , mean squared errors = 0.735

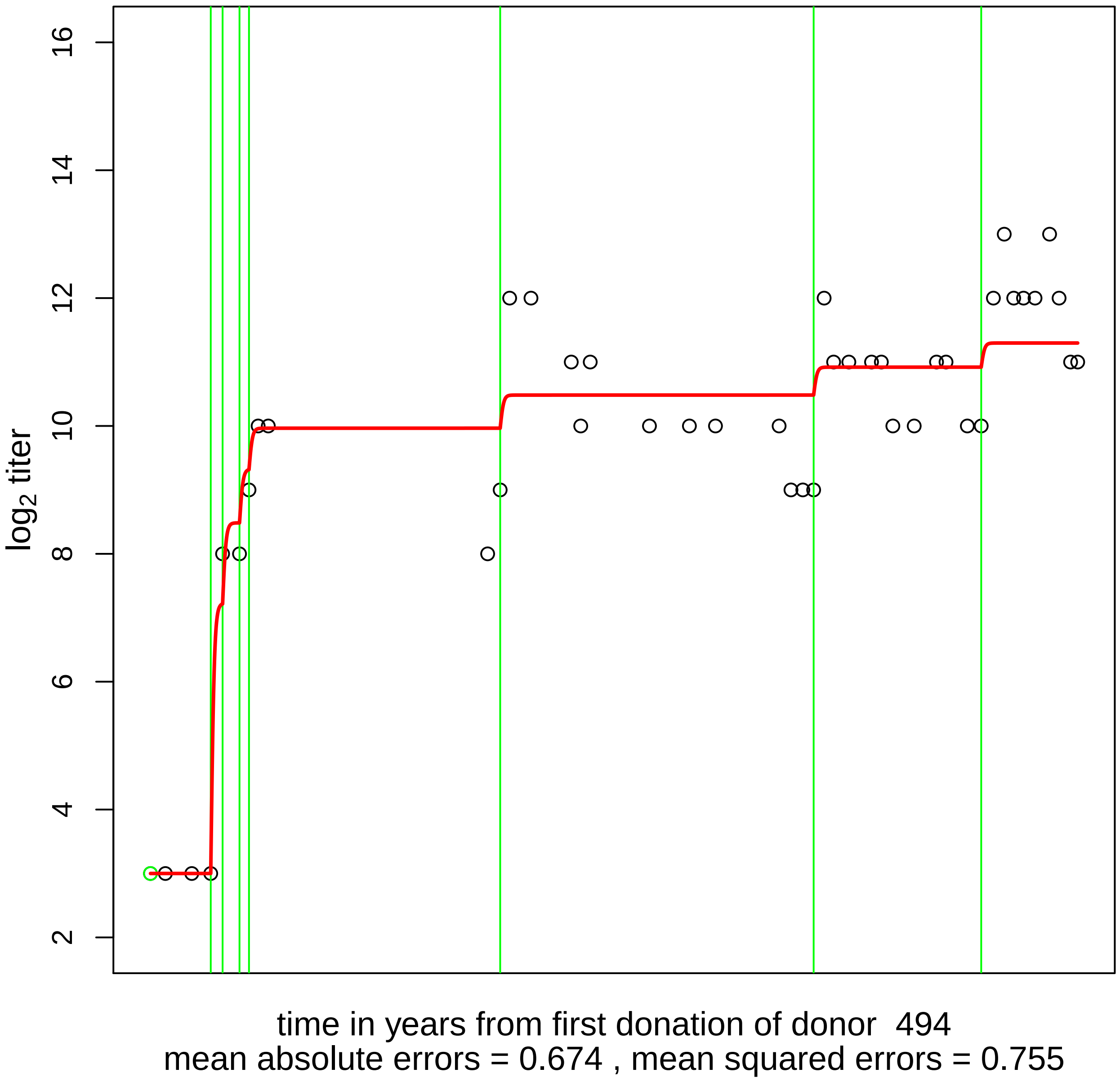

16
14
12
10
8
6
4
2
log2 titer
time in years from first donation of donor 494
mean absolute errors = 0.674 , mean squared errors = 0.755

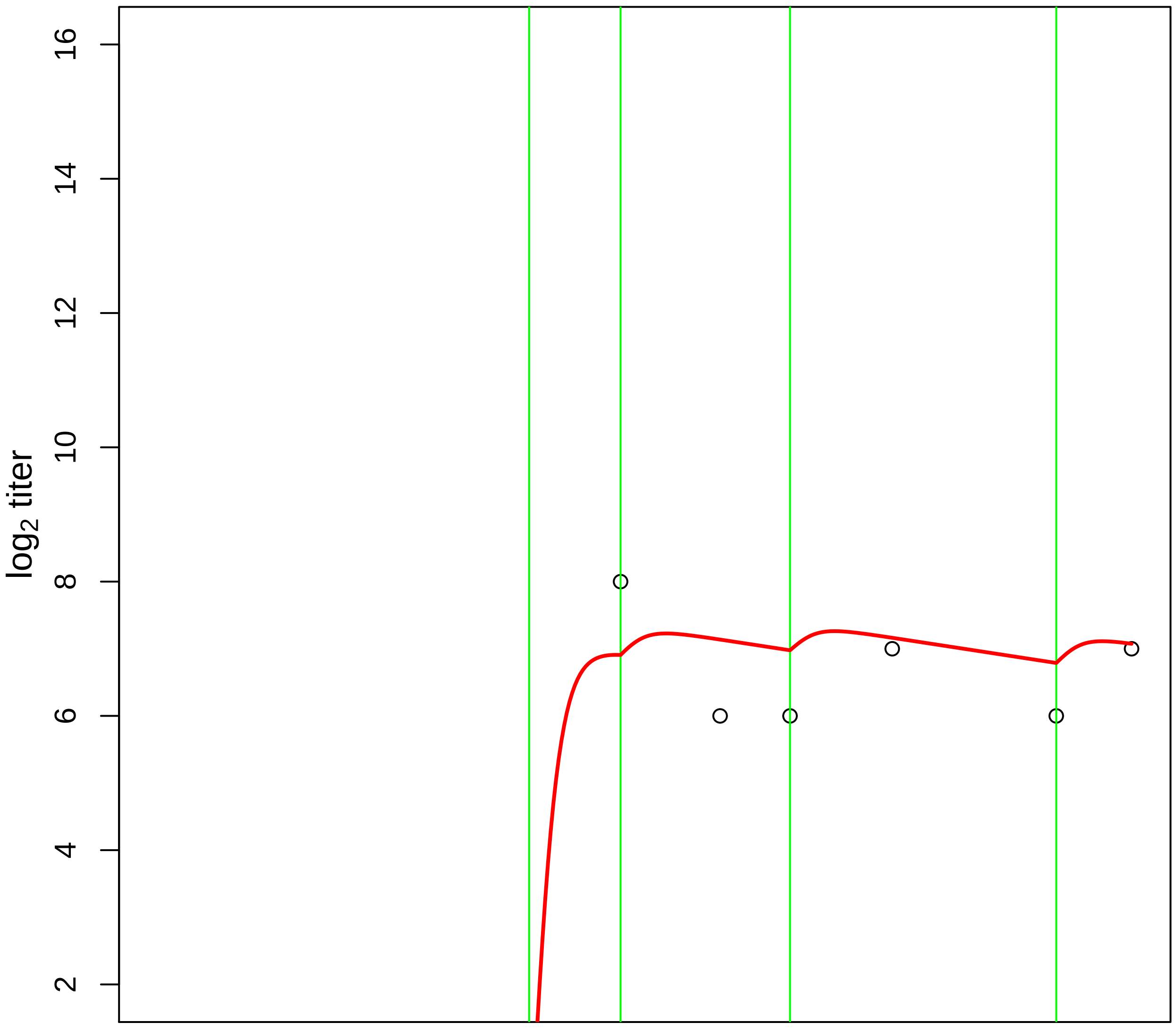

time in years from first donation of donor 495
mean absolute errors = 0.674 , mean squared errors = 0.614

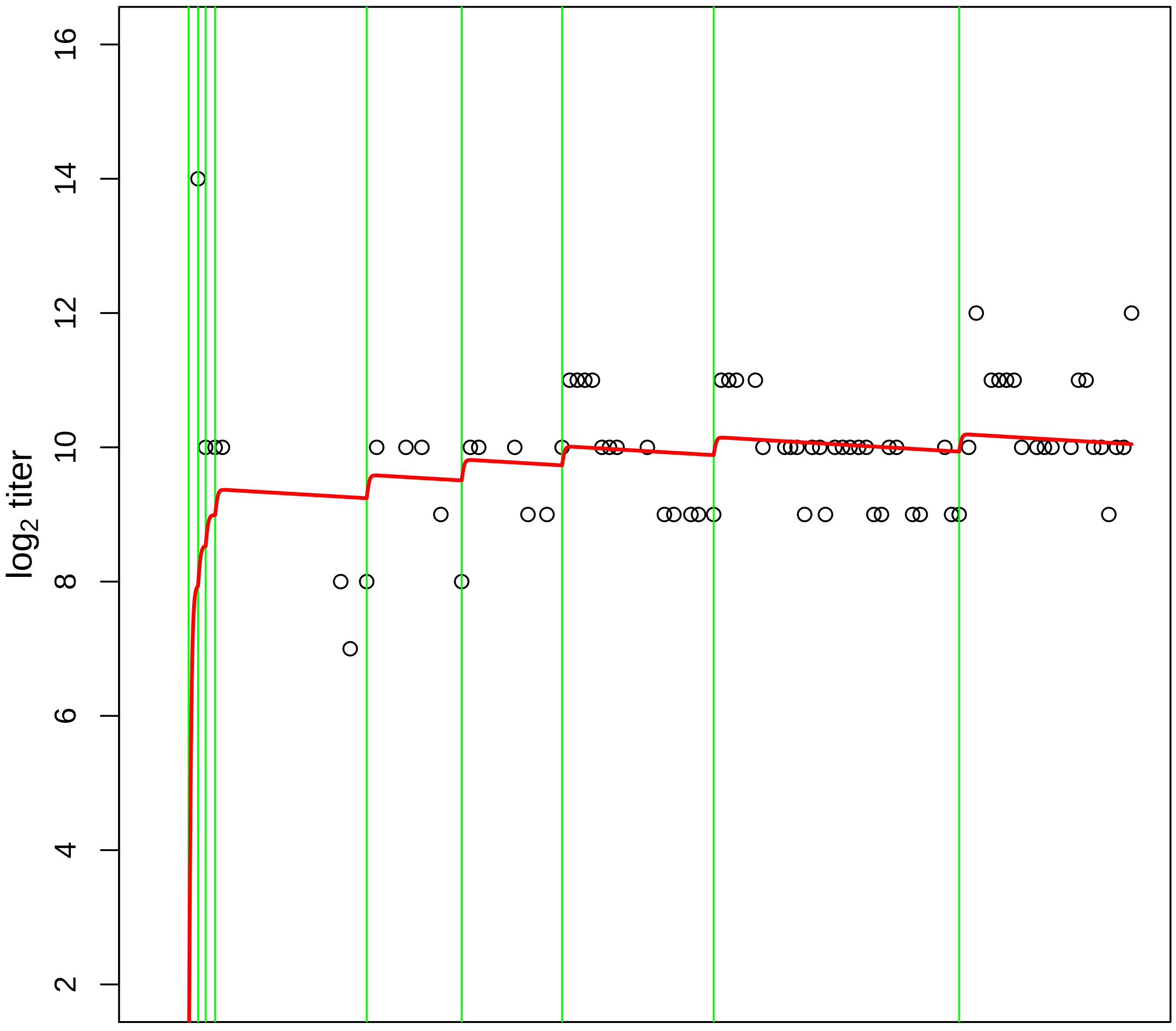

time in years from first donation of donor 496
mean absolute errors = 0.675 , mean squared errors = 1.104

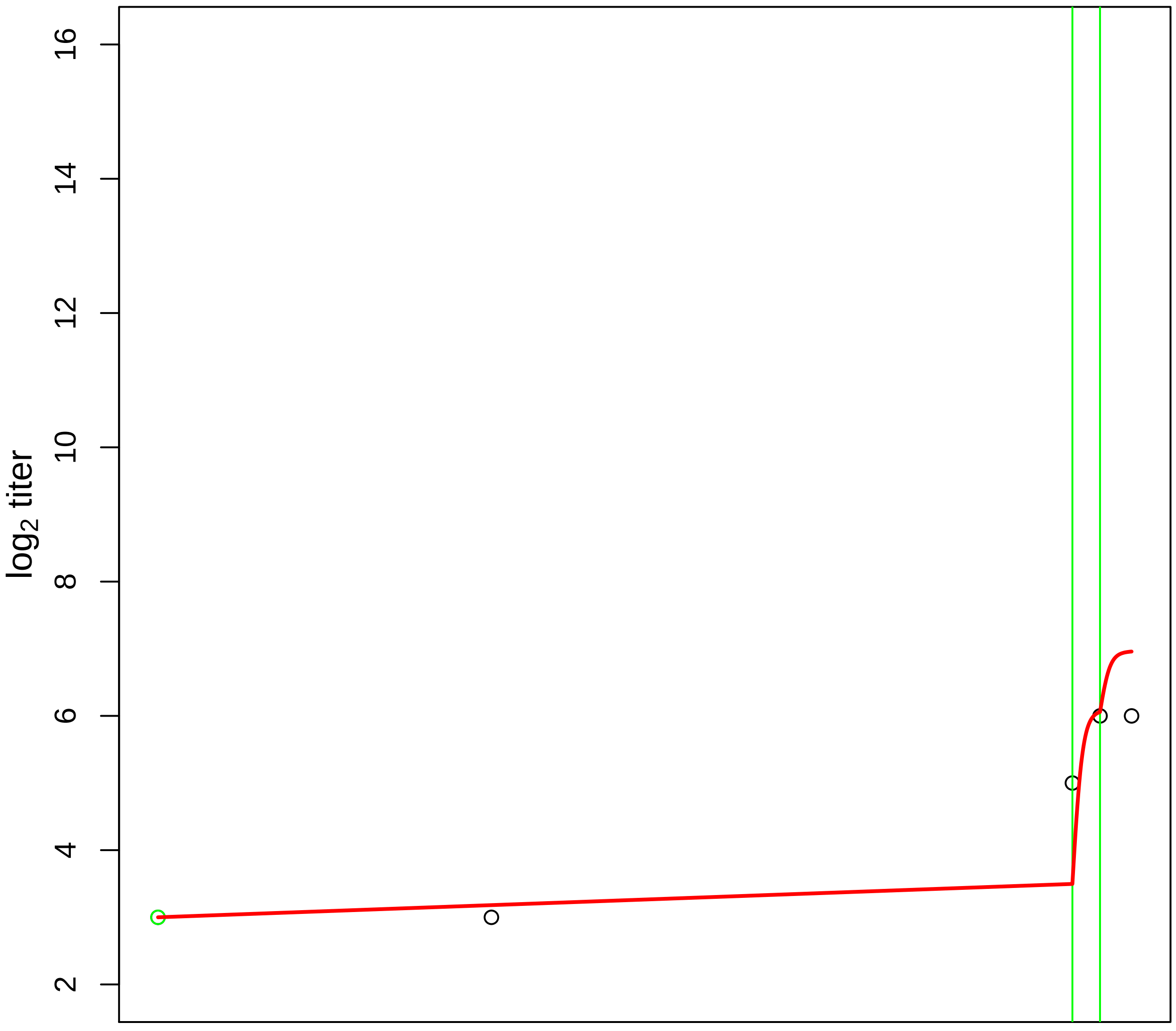

time in years from first donation of donor 497
mean absolute errors = 0.675 , mean squared errors = 0.803

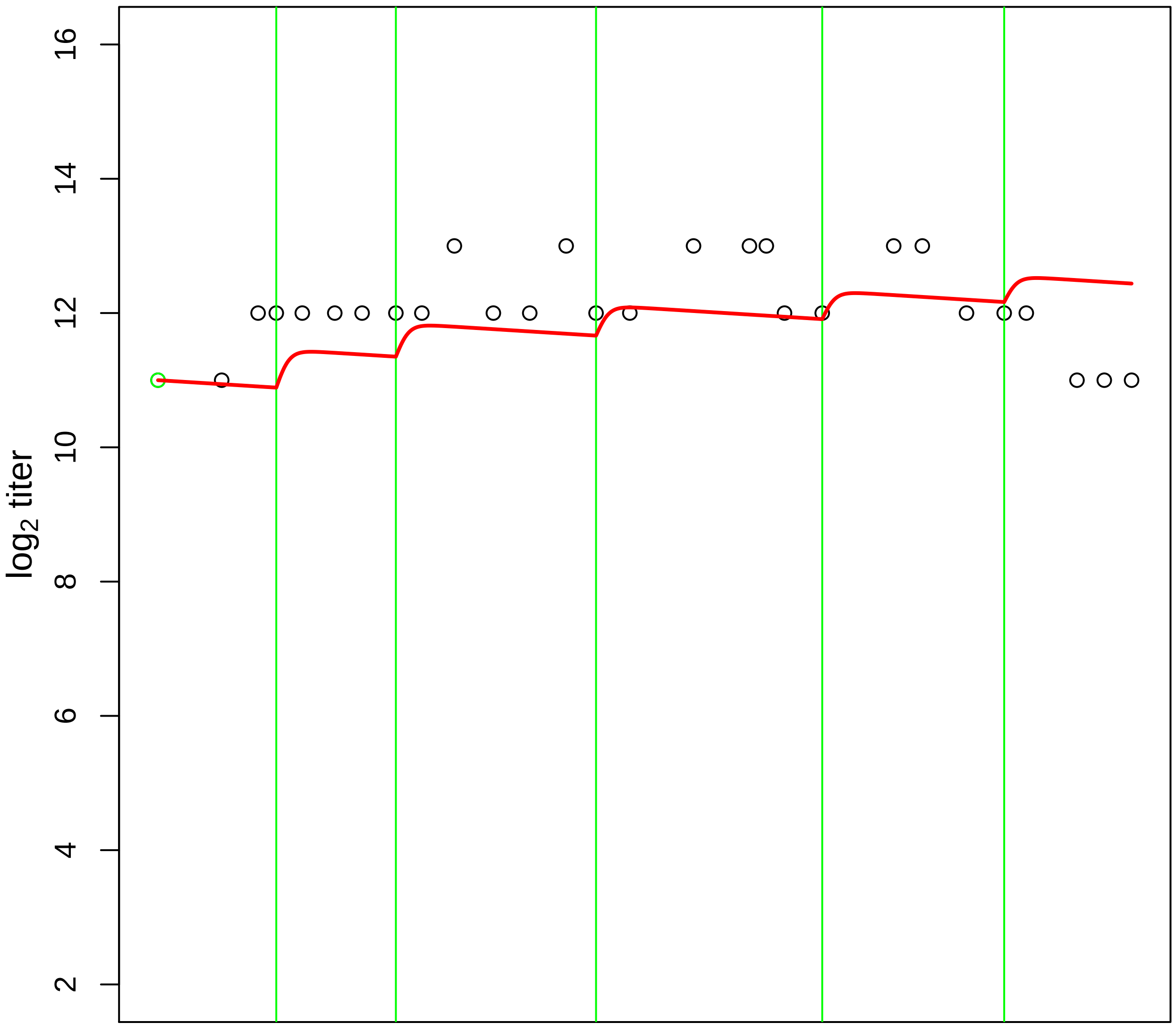

time in years from first donation of donor 498
mean absolute errors = 0.677 , mean squared errors = 0.679

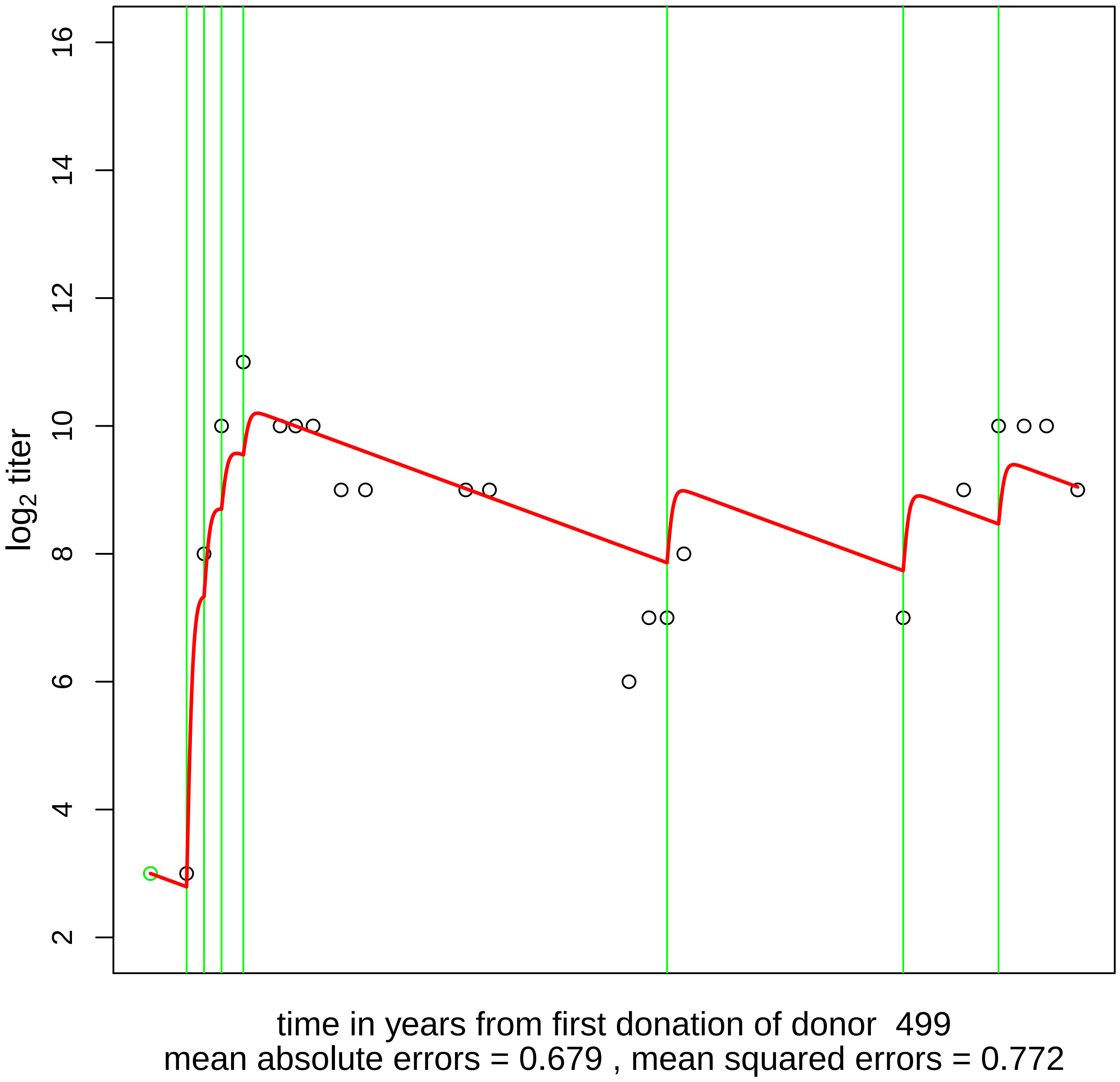

log2 titer
2
4
6
8
10
12
14
16
time in years from first donation of donor 499
mean absolute errors = 0.679 , mean squared errors = 0.772

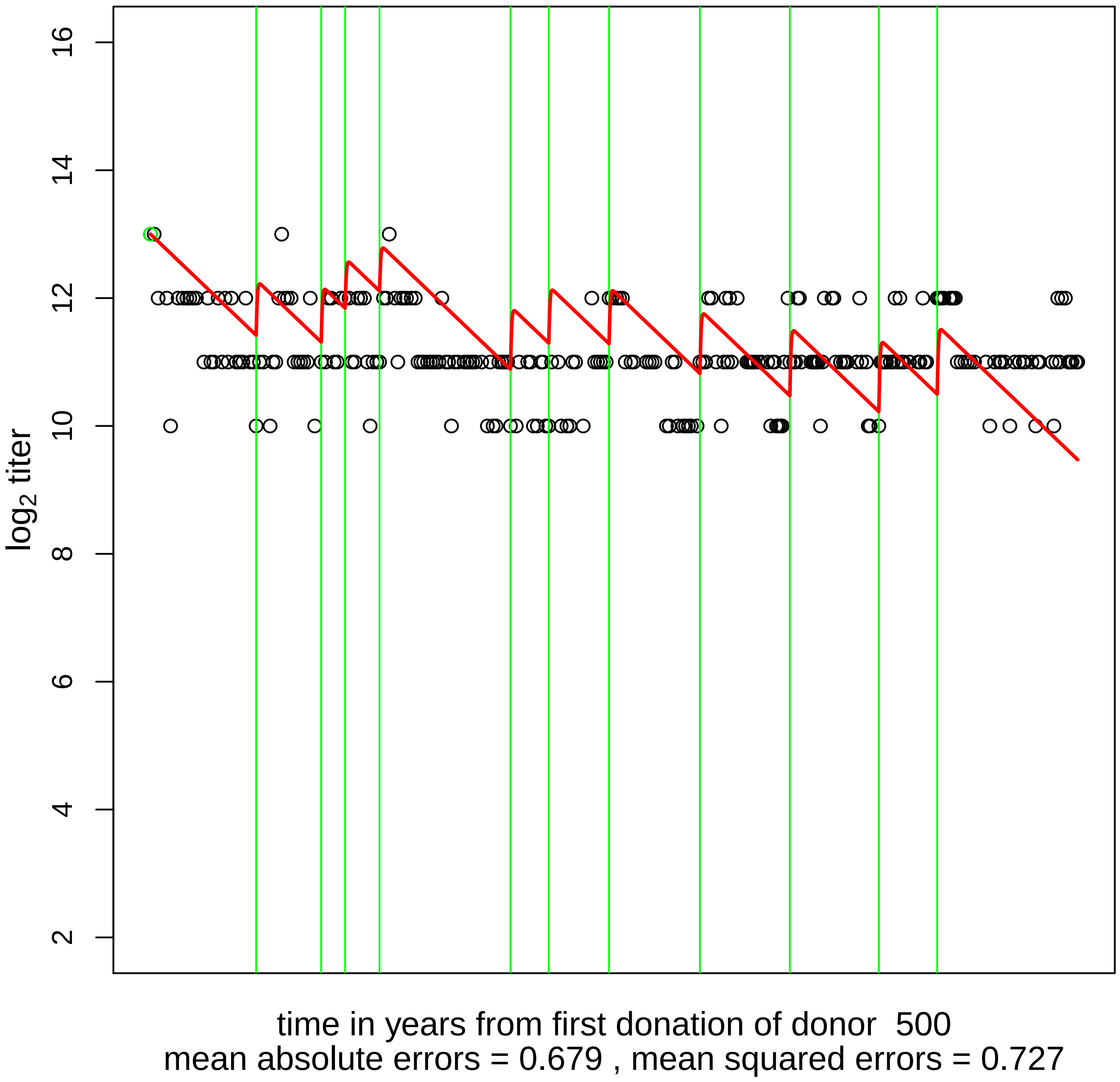

16
14
12
10
8
6
4
2
$\log_2$ titer
time in years from first donation of donor 500
mean absolute errors = 0.679 , mean squared errors = 0.727

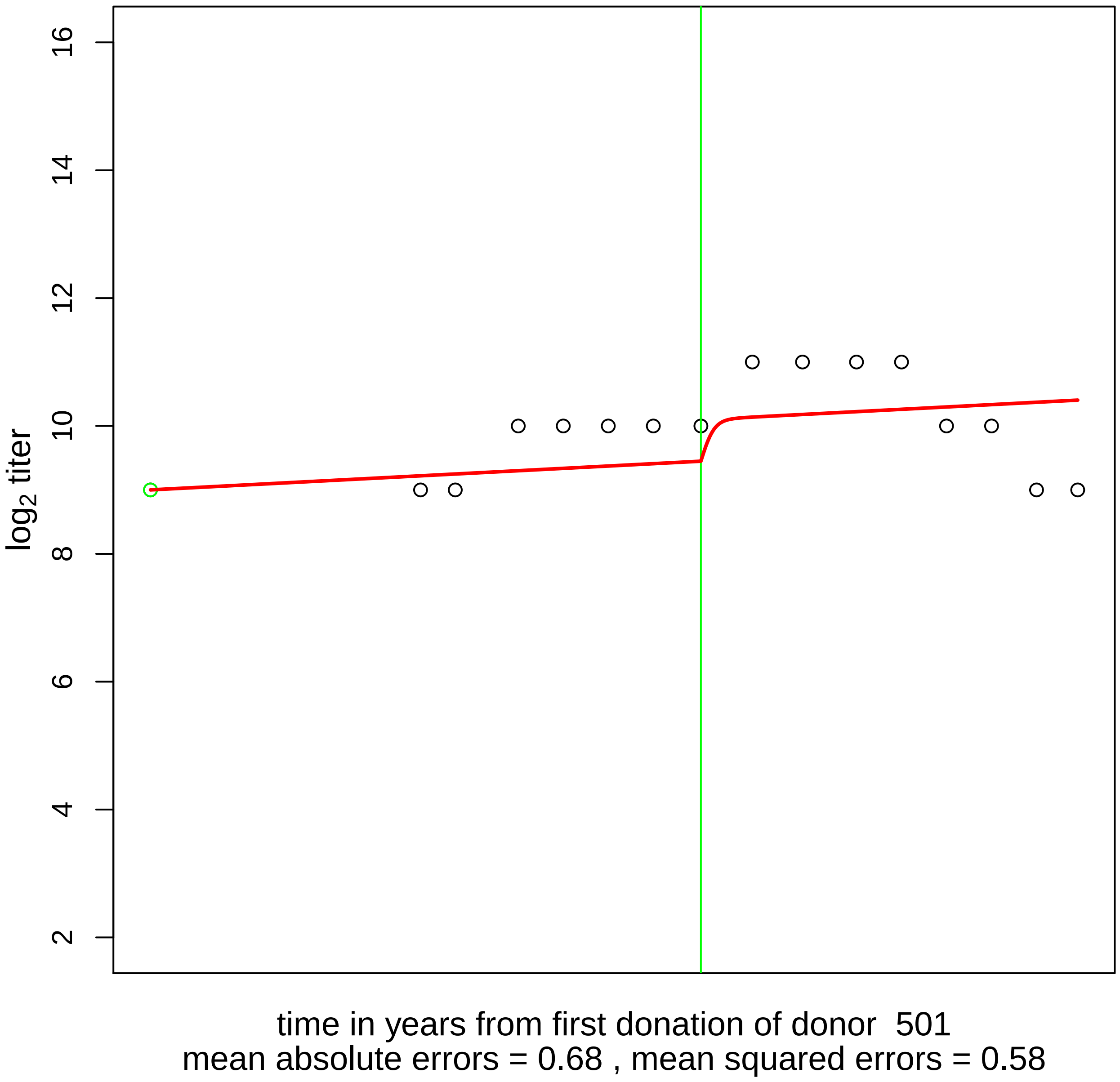

16
14
12
10
8
6
4
2
$\log_2$ titer
time in years from first donation of donor 501
mean absolute errors = 0.68 , mean squared errors = 0.58

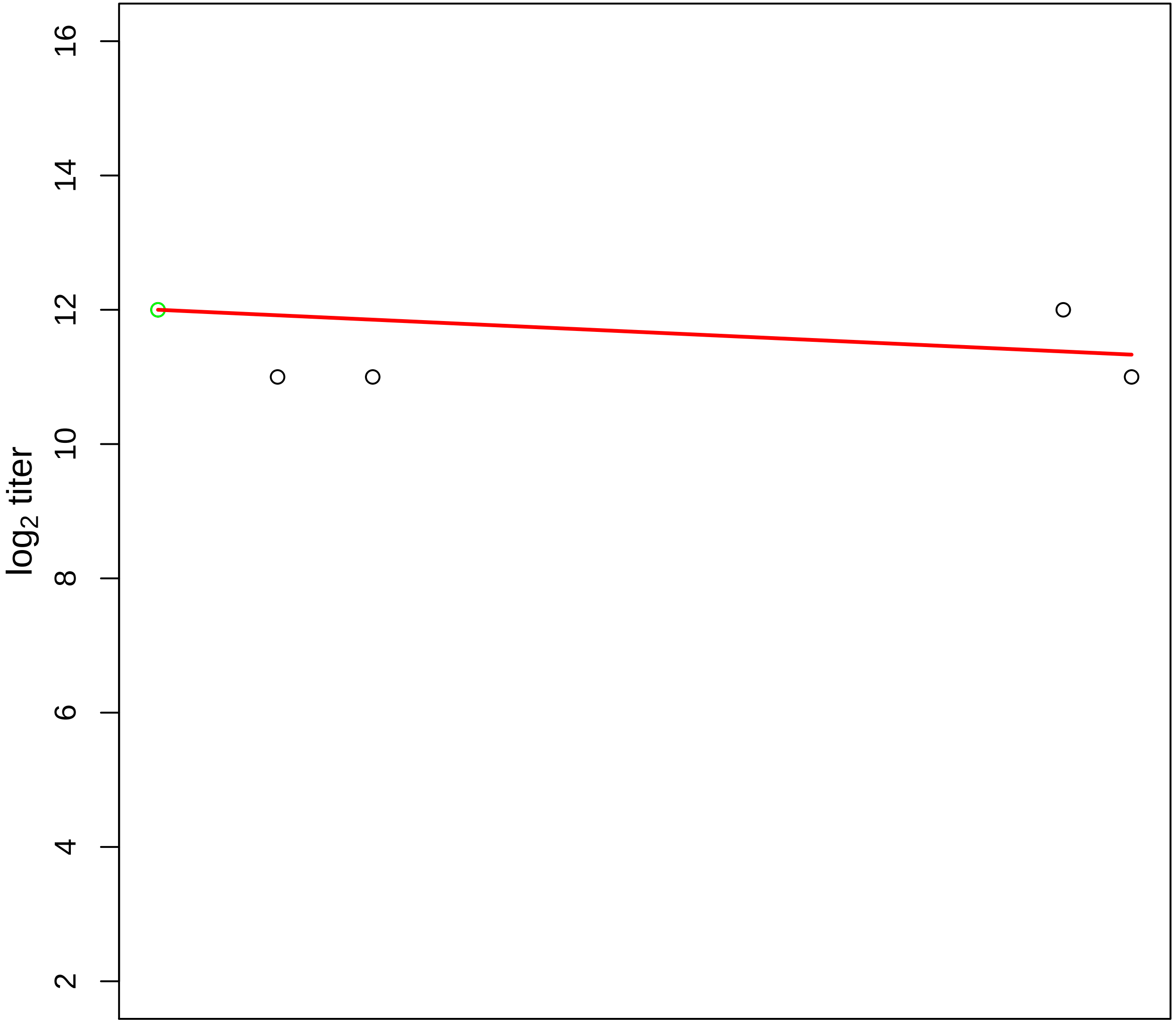

time in years from first donation of donor 502
mean absolute errors = 0.681 , mean squared errors = 0.516

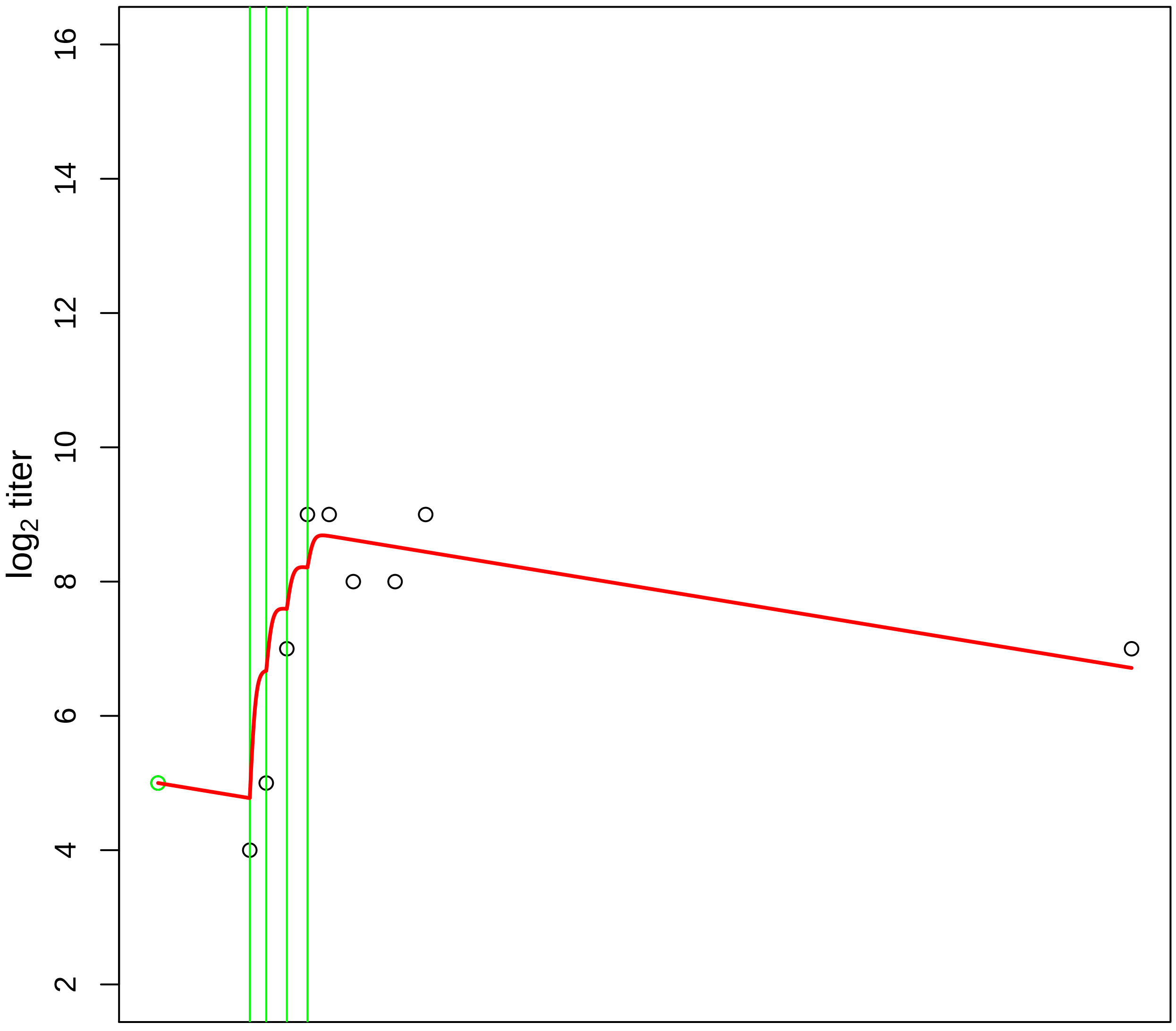

time in years from first donation of donor 503
mean absolute errors = 0.681 , mean squared errors = 0.613

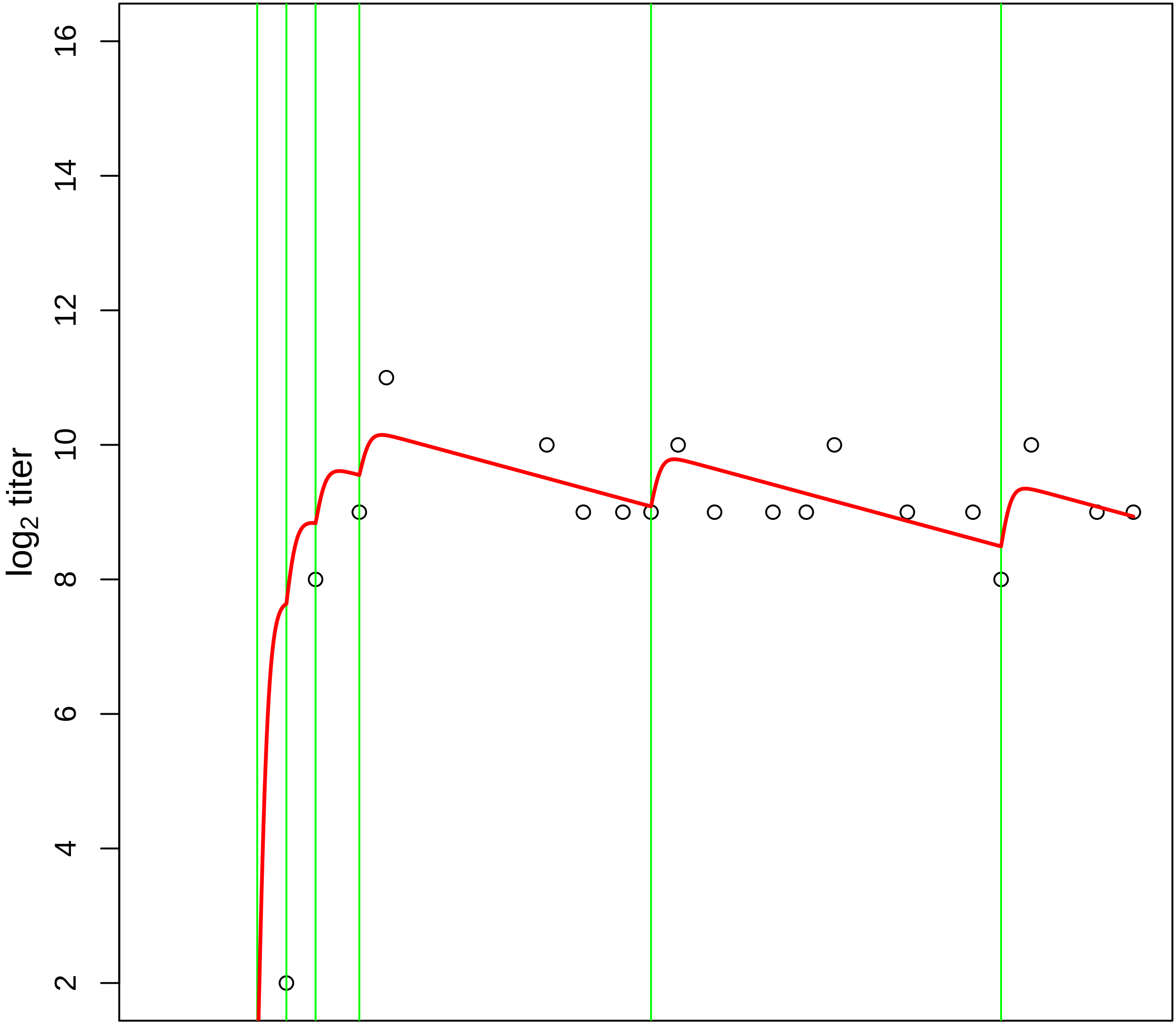

time in years from first donation of donor 504
mean absolute errors = 0.681 , mean squared errors = 1.818

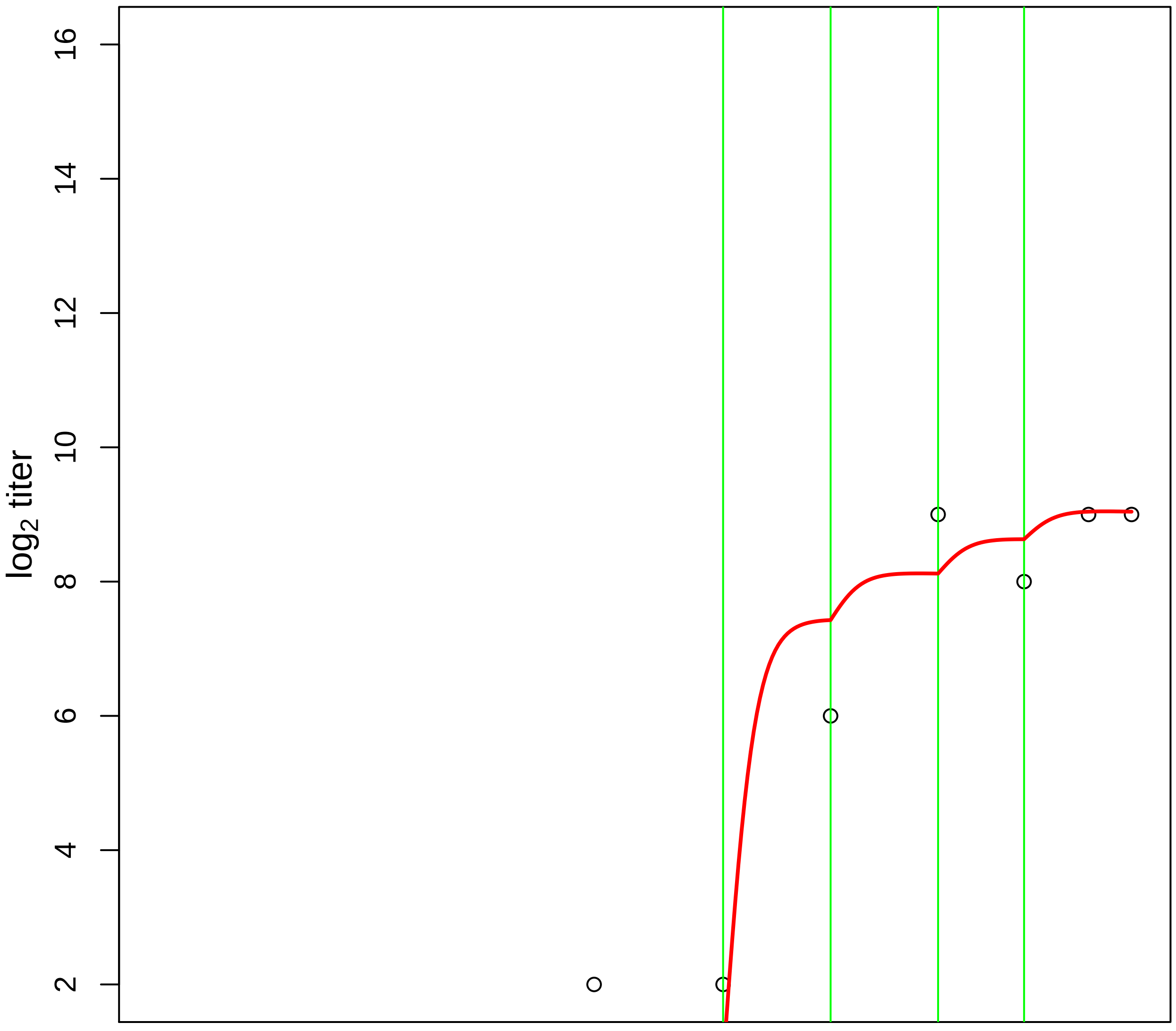

time in years from first donation of donor 505
mean absolute errors = 0.682 , mean squared errors = 0.746

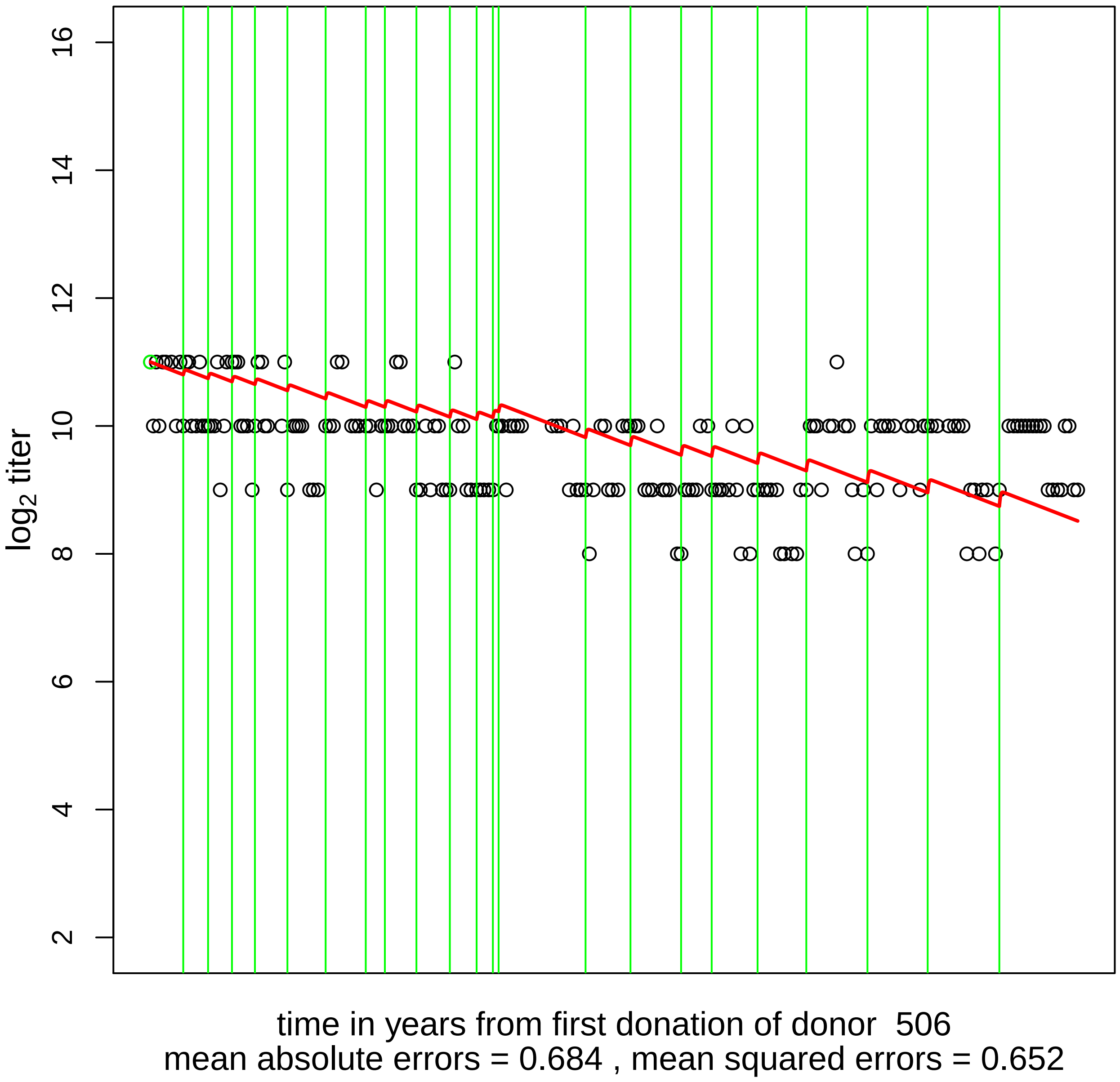

16
14
12
10
8
6
4
2
log2 titer
time in years from first donation of donor 506
mean absolute errors = 0.684 , mean squared errors = 0.652

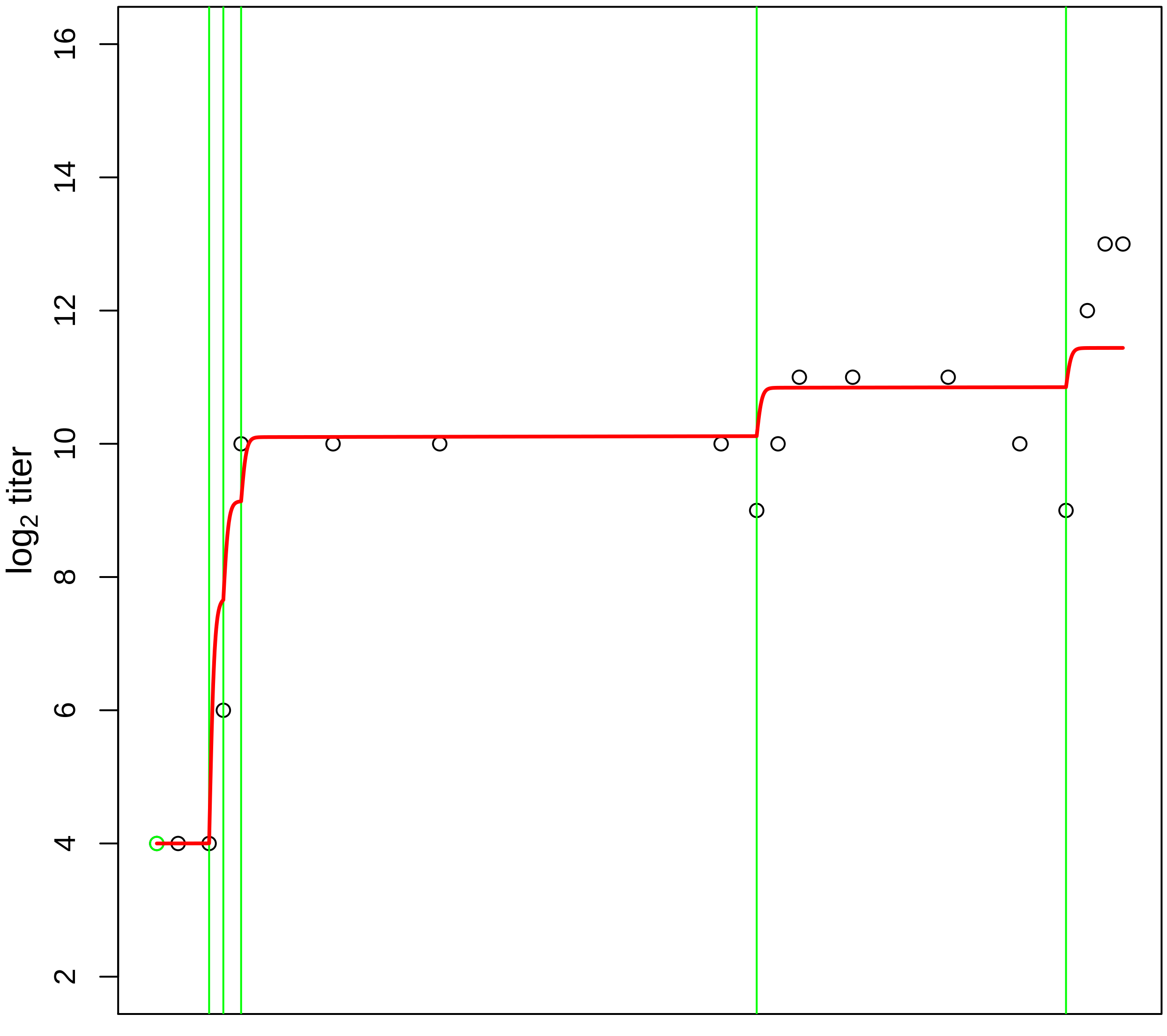

time in years from first donation of donor 507
mean absolute errors = 0.685 , mean squared errors = 0.875

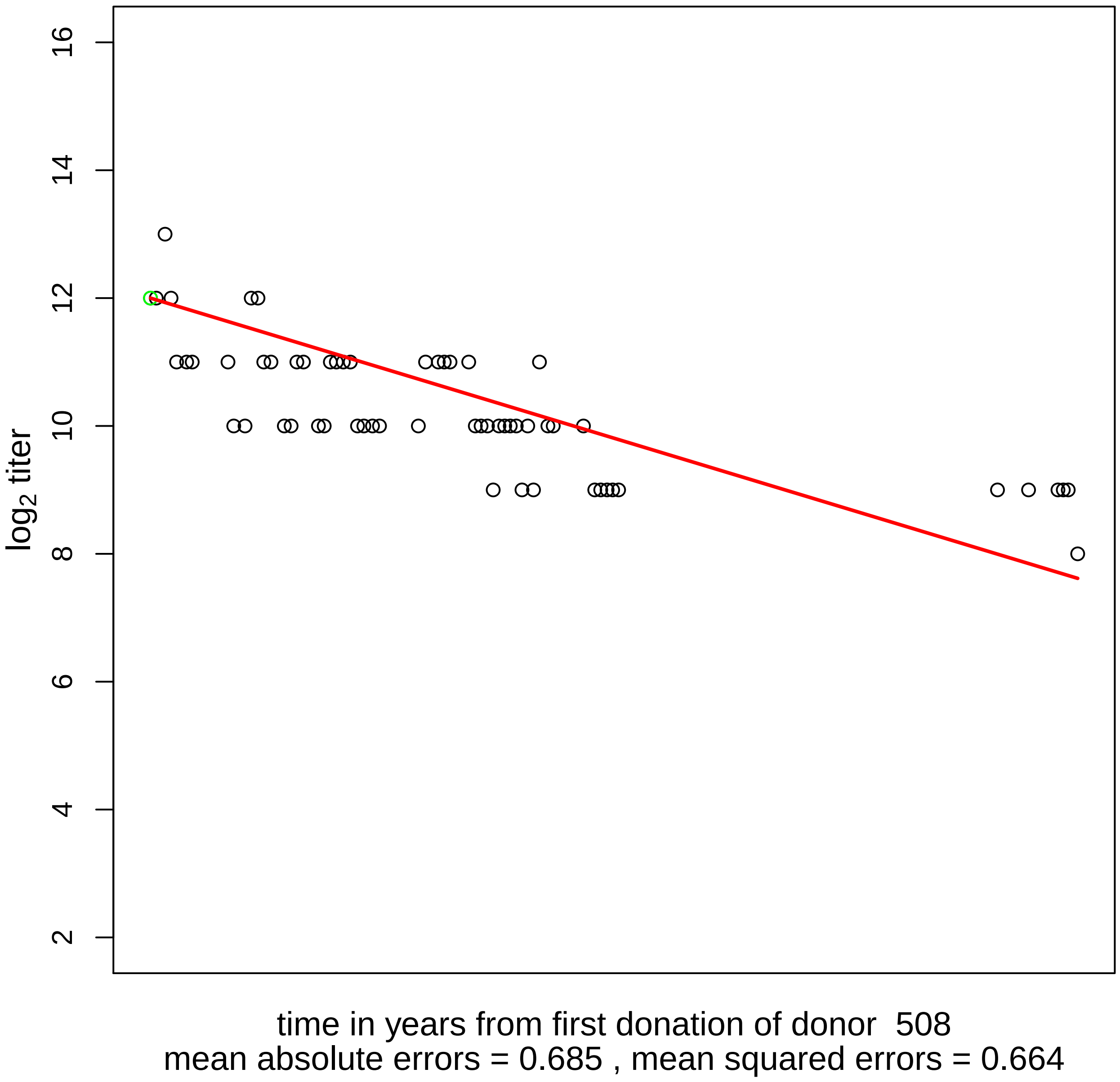

log2 titer
16
14
12
10
8
6
4
2
time in years from first donation of donor 508
mean absolute errors = 0.685 , mean squared errors = 0.664

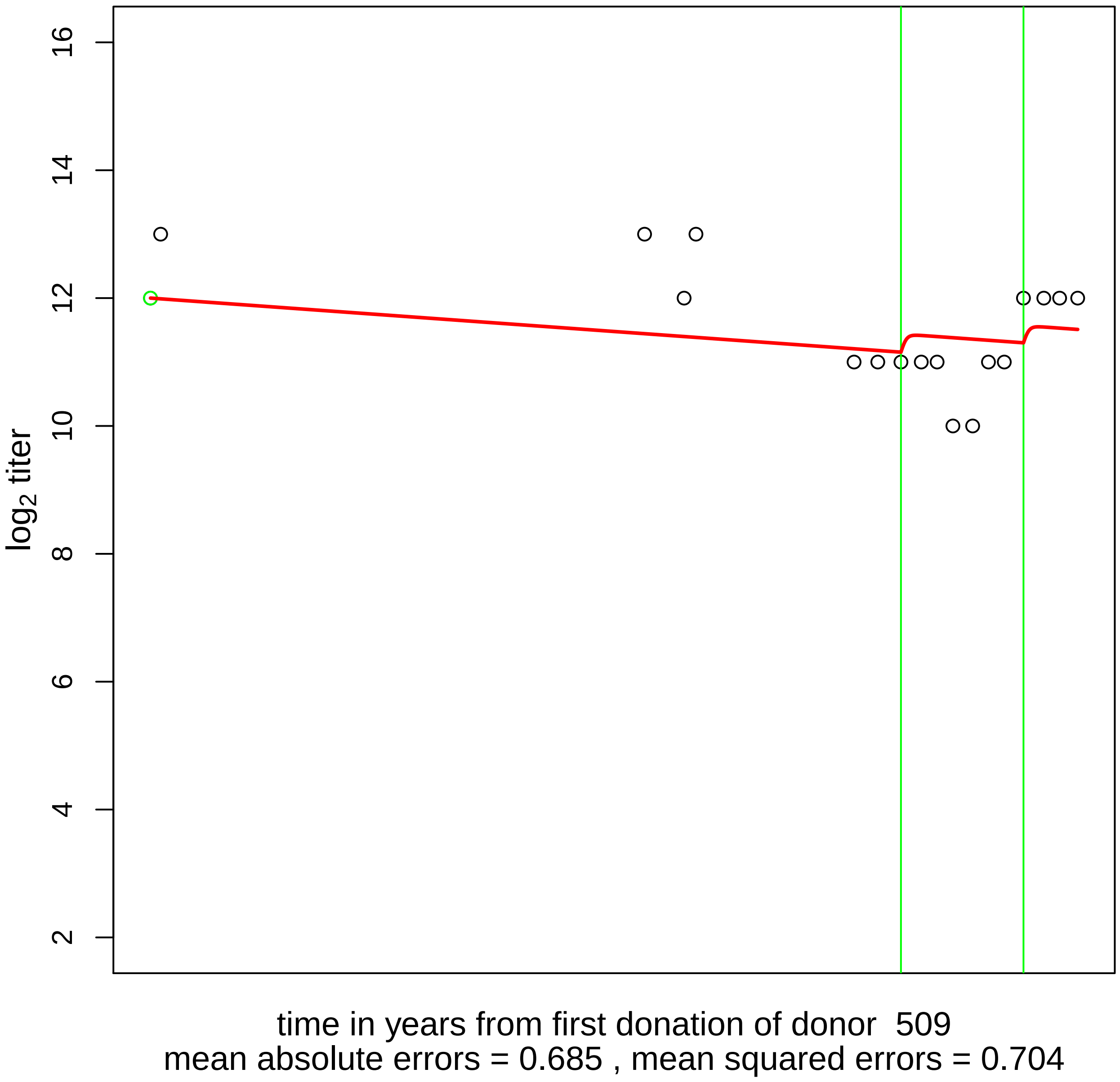

16
14
12
10
8
6
4
2
$\log_2$ titer
time in years from first donation of donor 509
mean absolute errors = 0.685 , mean squared errors = 0.704

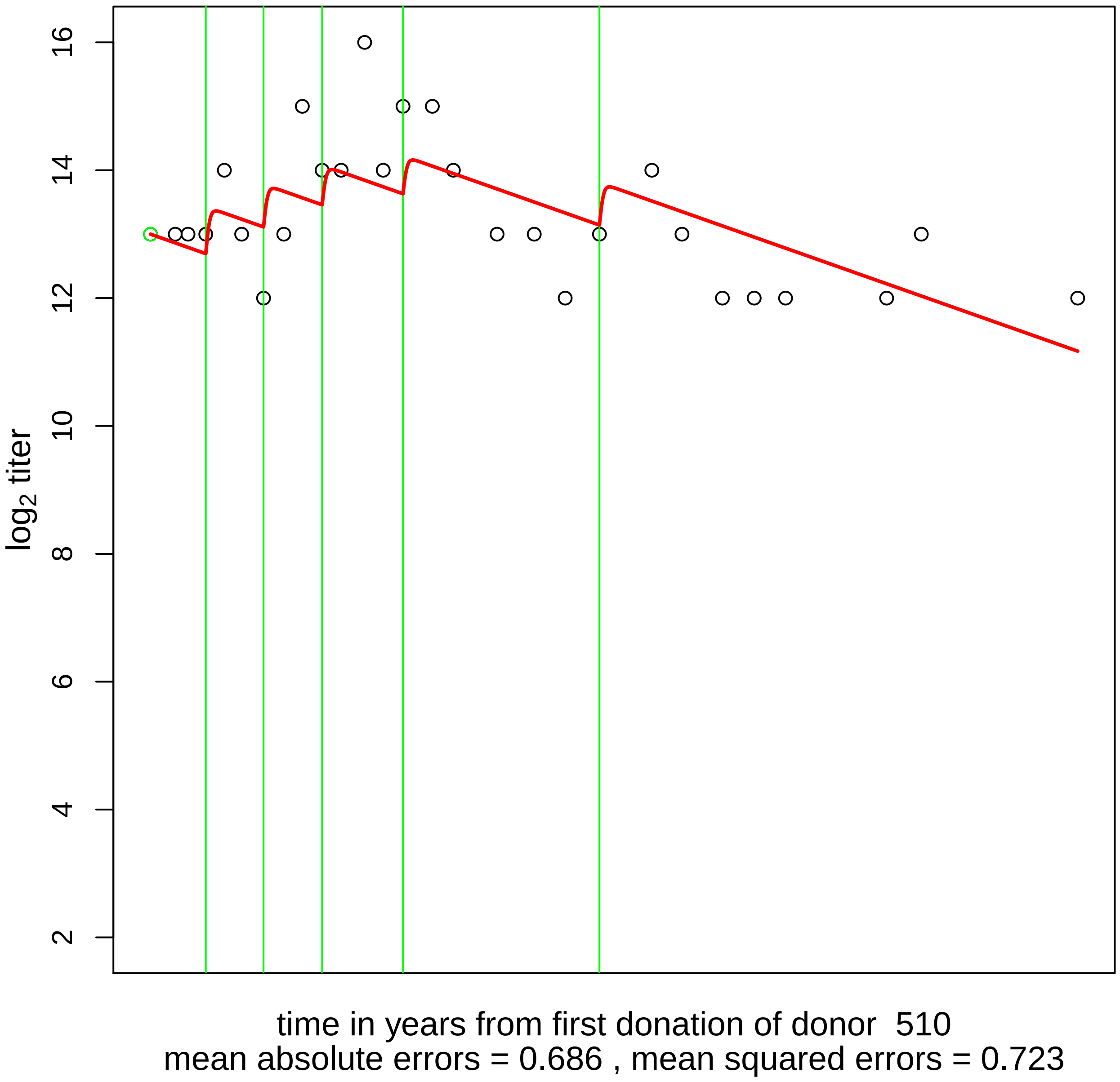

16
14
12
10
8
6
4
2
log2 titer
time in years from first donation of donor 510
mean absolute errors = 0.686 , mean squared errors = 0.723

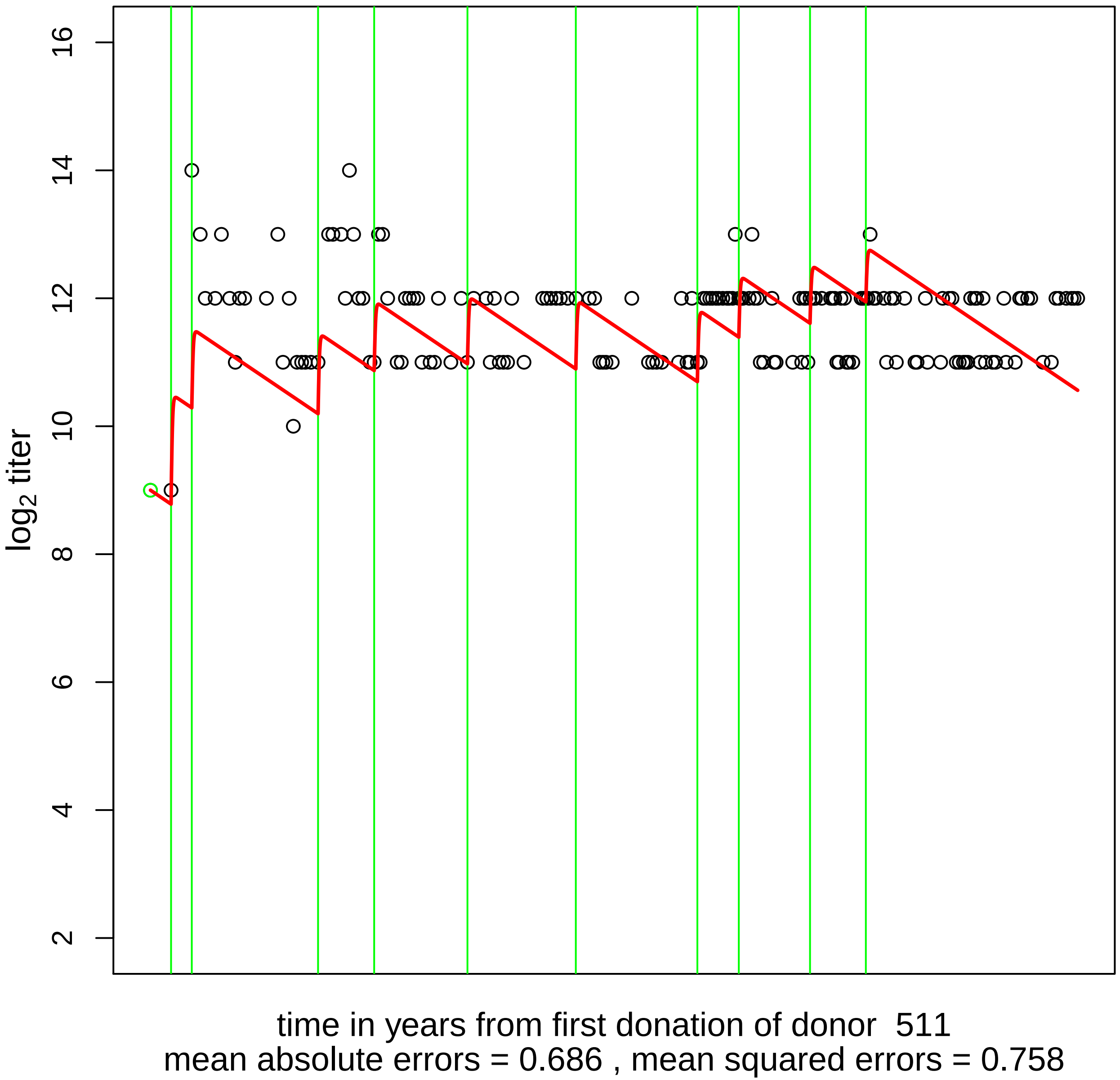

16
14
12
10
8
6
4
2
$\log_2$ titer
time in years from first donation of donor 511
mean absolute errors = 0.686 , mean squared errors = 0.758

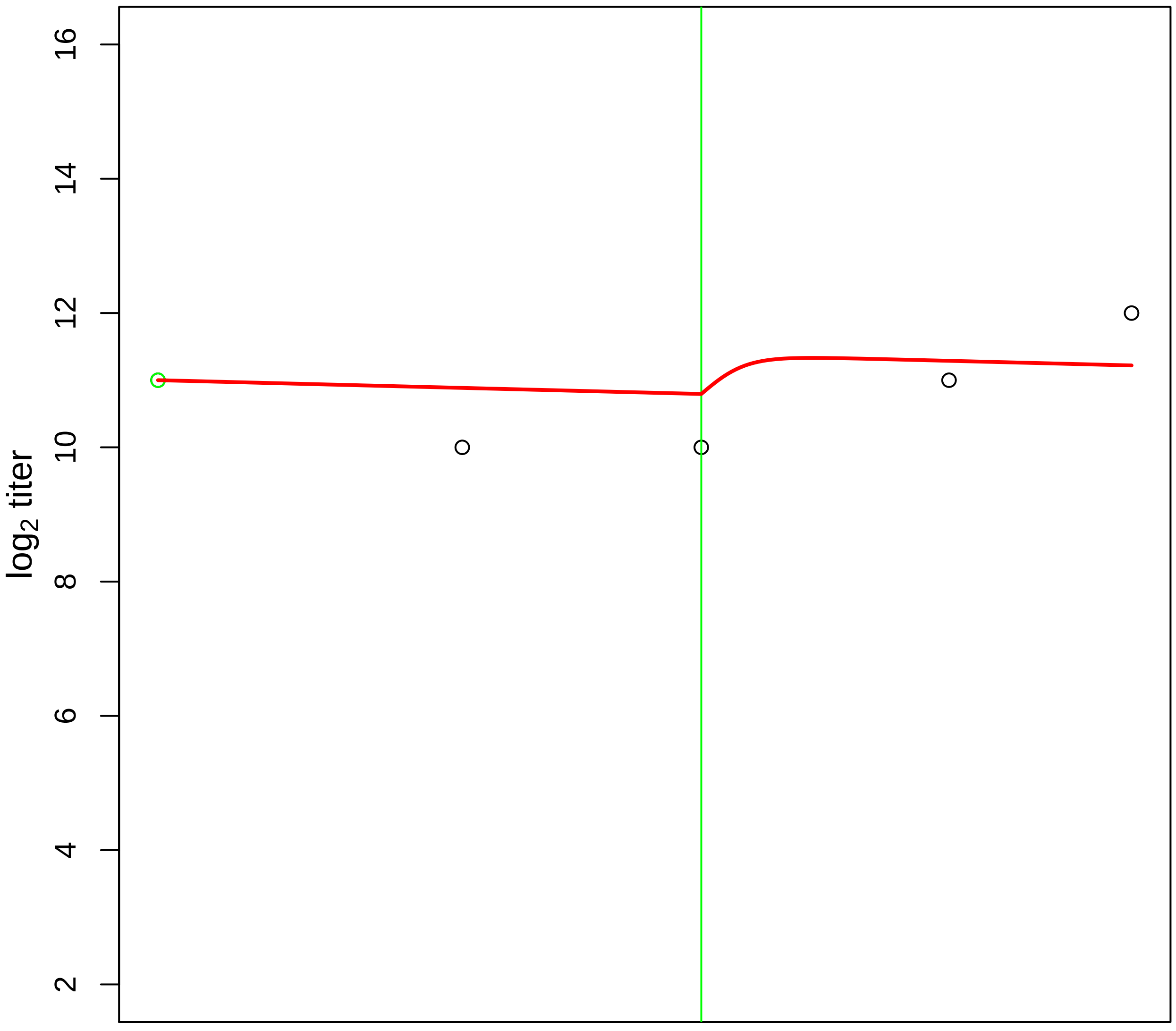

time in years from first donation of donor 512
mean absolute errors = 0.687 , mean squared errors = 0.527

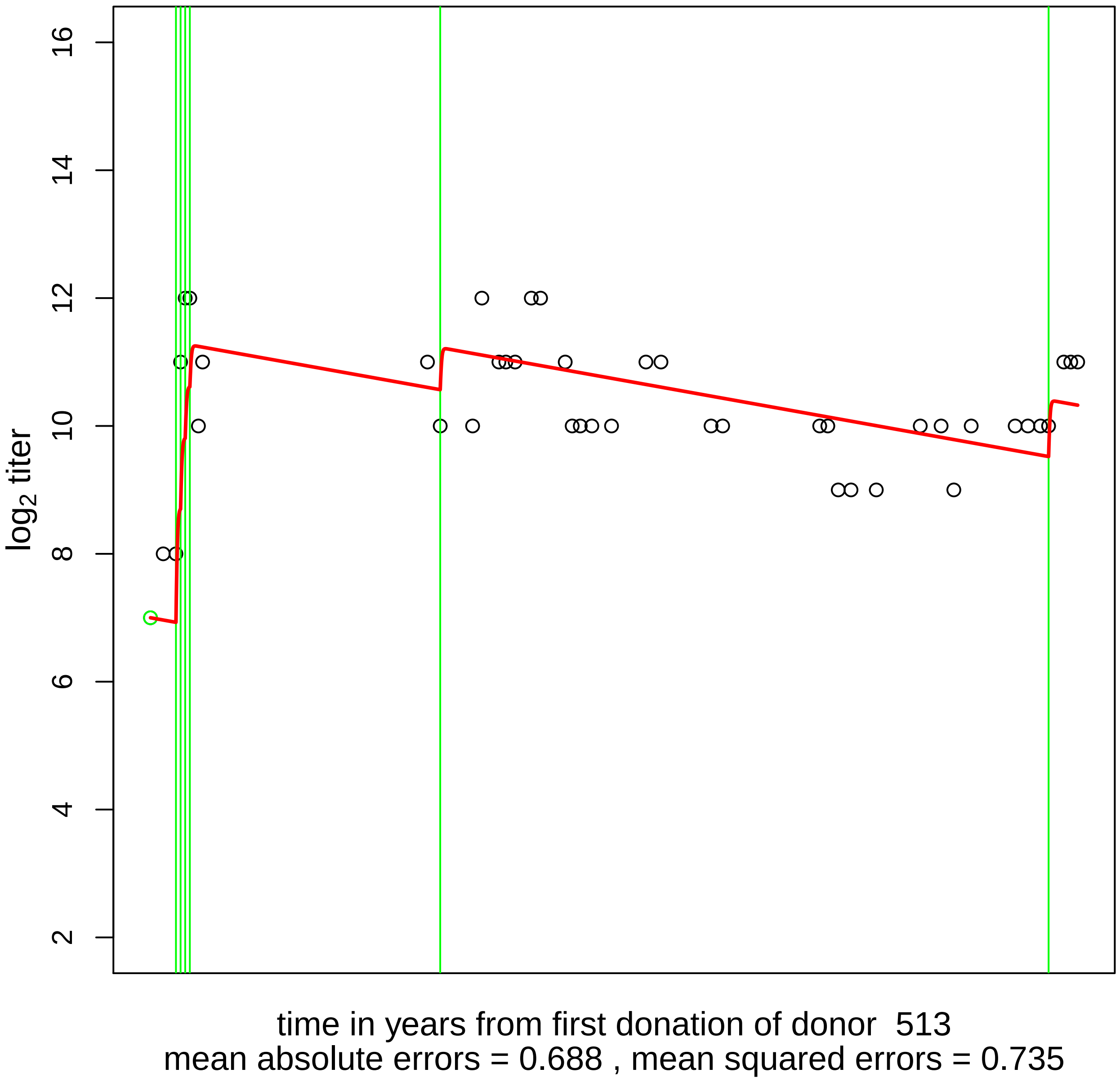

16
14
12
10
8
6
4
2
log2 titer
time in years from first donation of donor 513
mean absolute errors = 0.688 , mean squared errors = 0.735

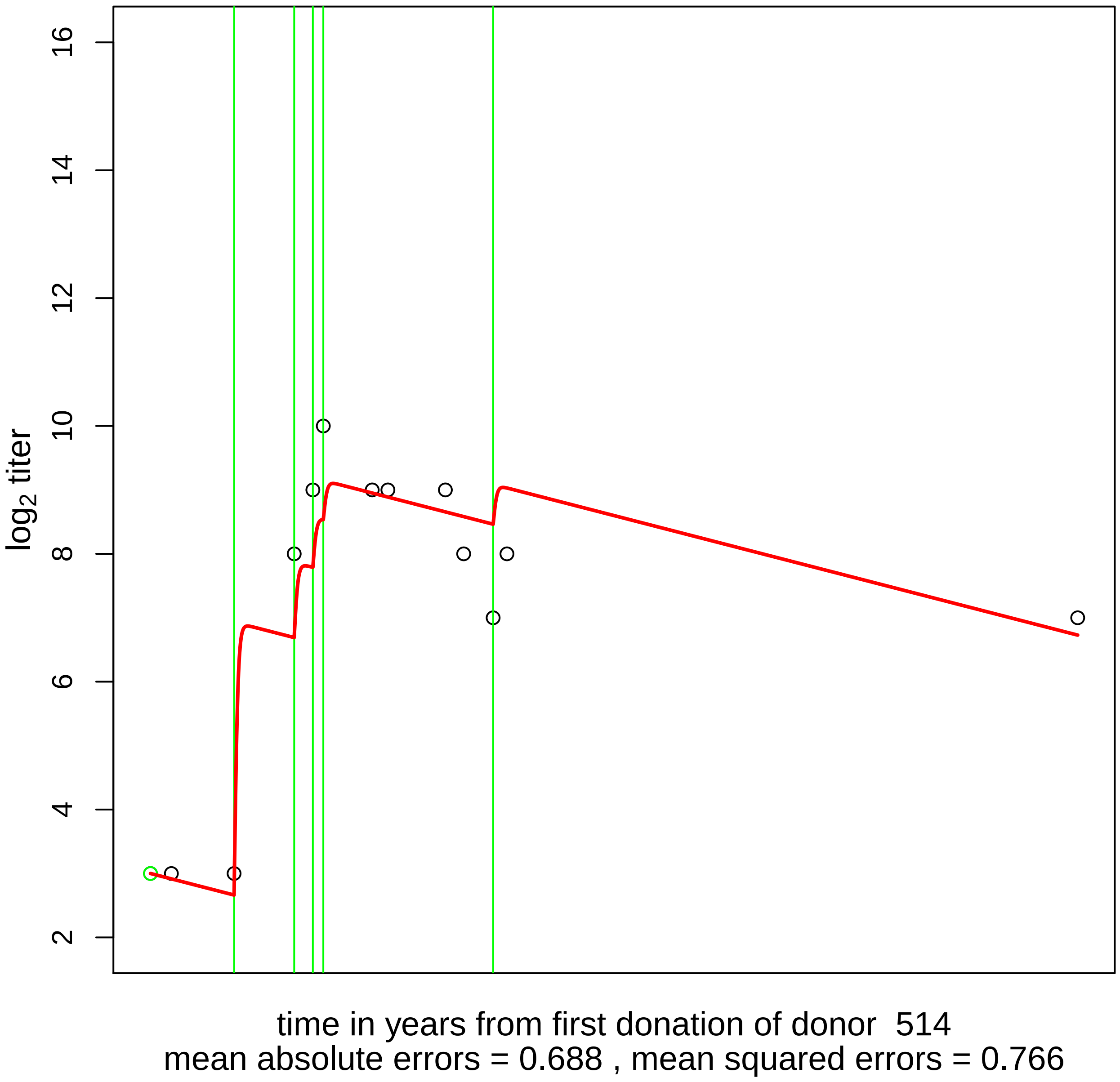
16
14
12
10
8
6
4
2
log2 titer
time in years from first donation of donor 514
mean absolute errors = 0.688 , mean squared errors = 0.766

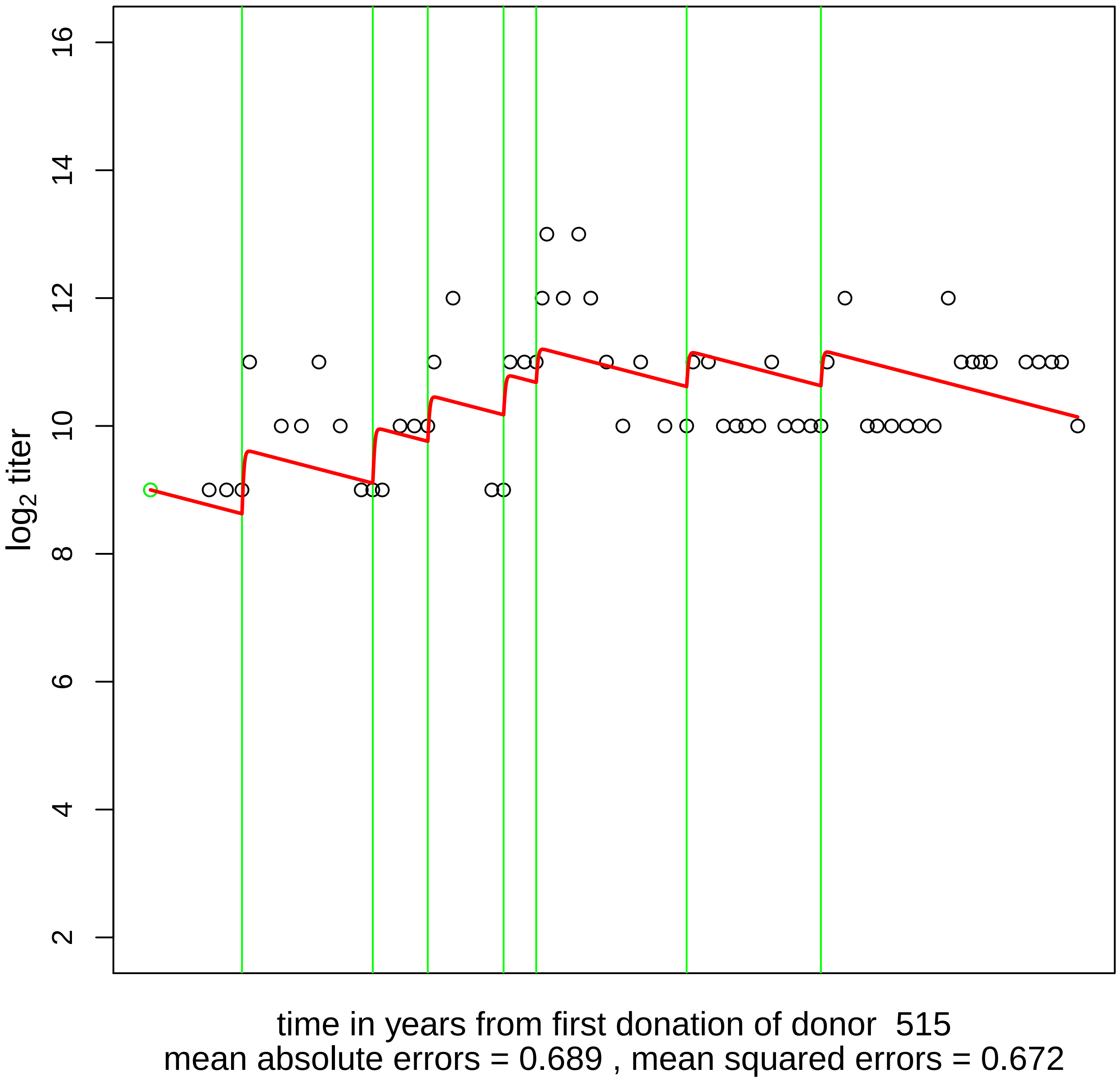

16
14
12
10
8
6
4
2
$\log_2$ titer
time in years from first donation of donor 515
mean absolute errors = 0.689 , mean squared errors = 0.672

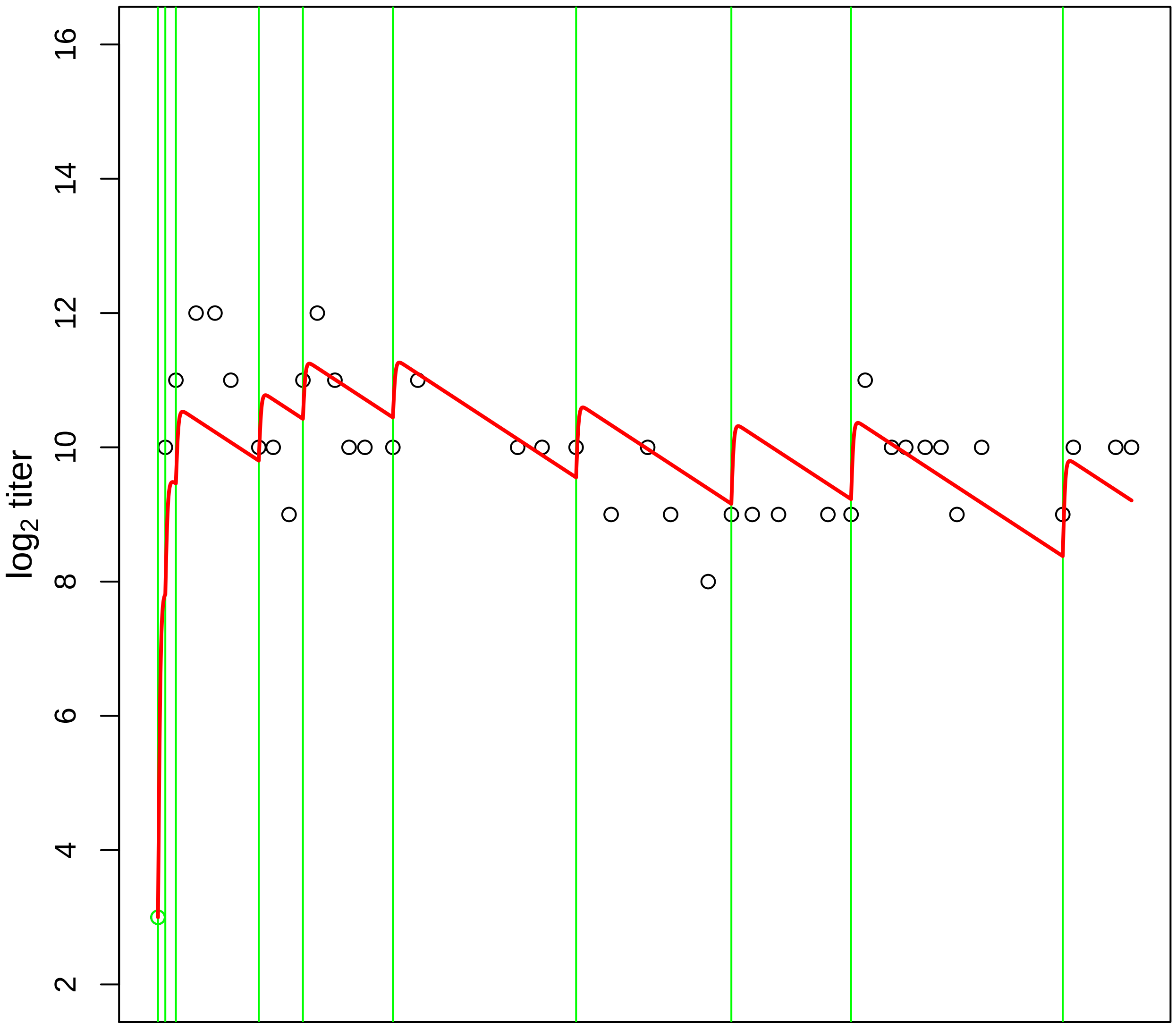

time in years from first donation of donor 516
mean absolute errors = 0.69 , mean squared errors = 0.771

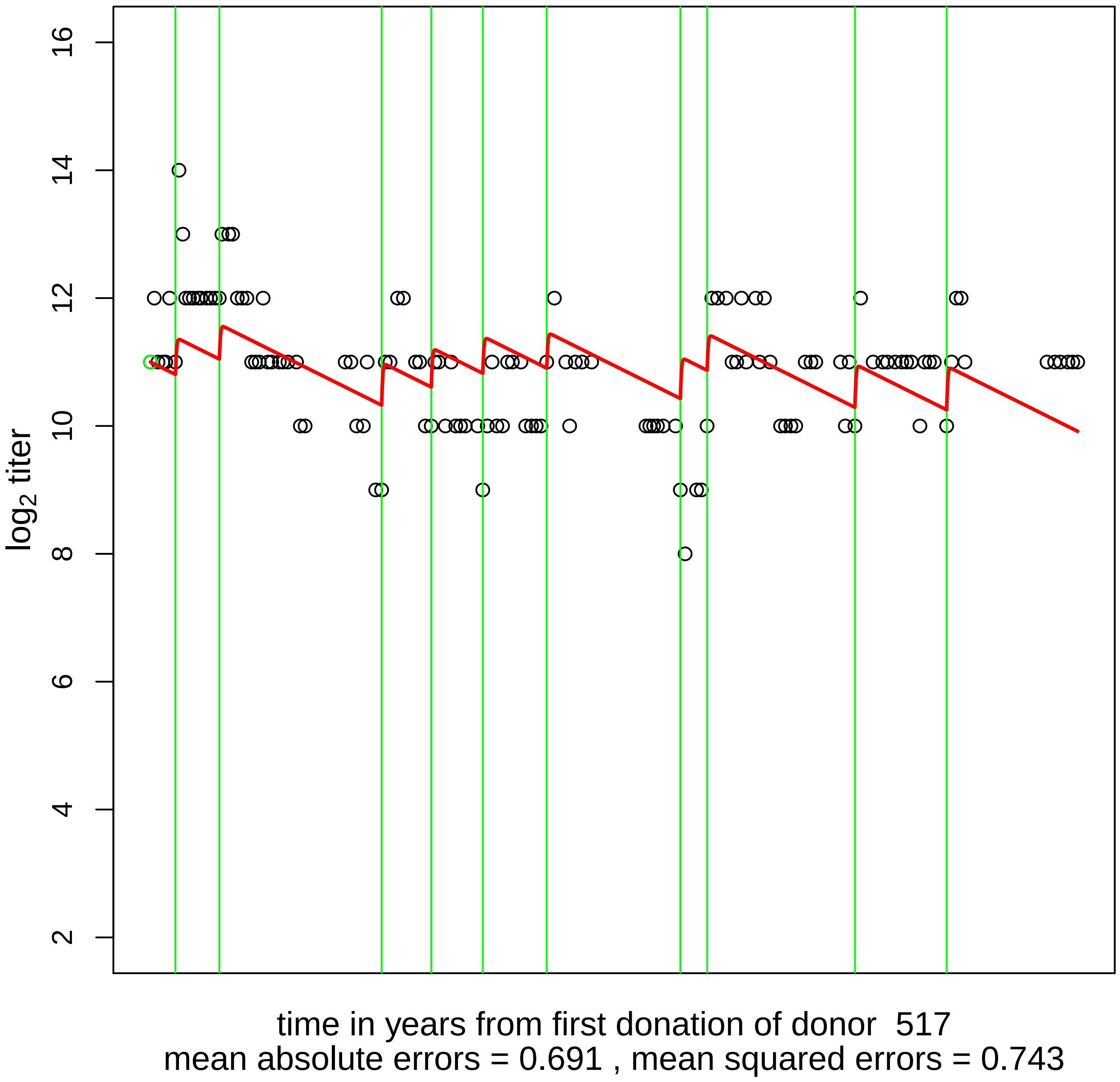

16
14
12
10
8
6
4
2
log2 titer
time in years from first donation of donor 517
mean absolute errors = 0.691 , mean squared errors = 0.743

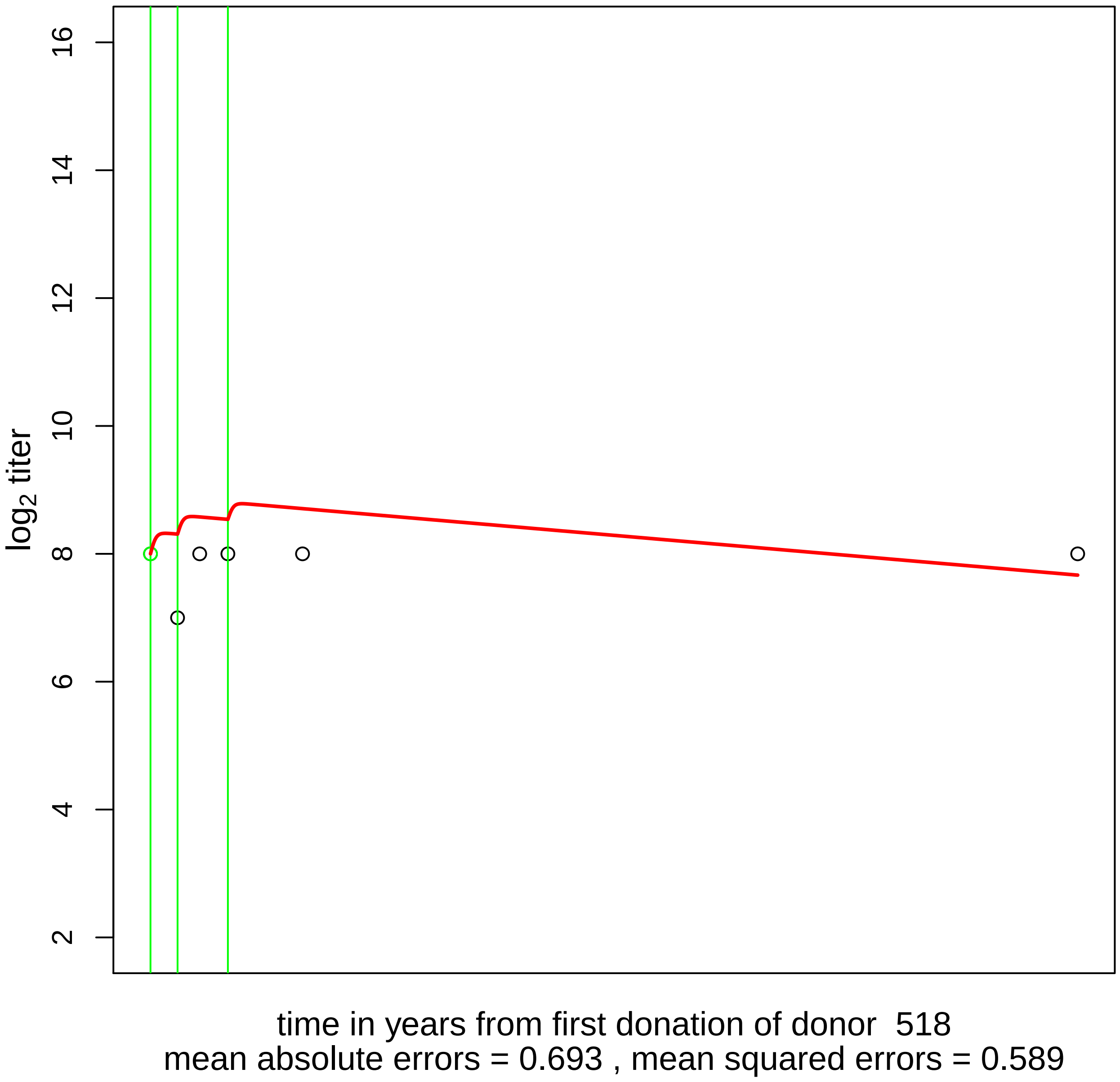

log2 titer
2
4
6
8
10
12
14
16
time in years from first donation of donor 518
mean absolute errors = 0.693 , mean squared errors = 0.589

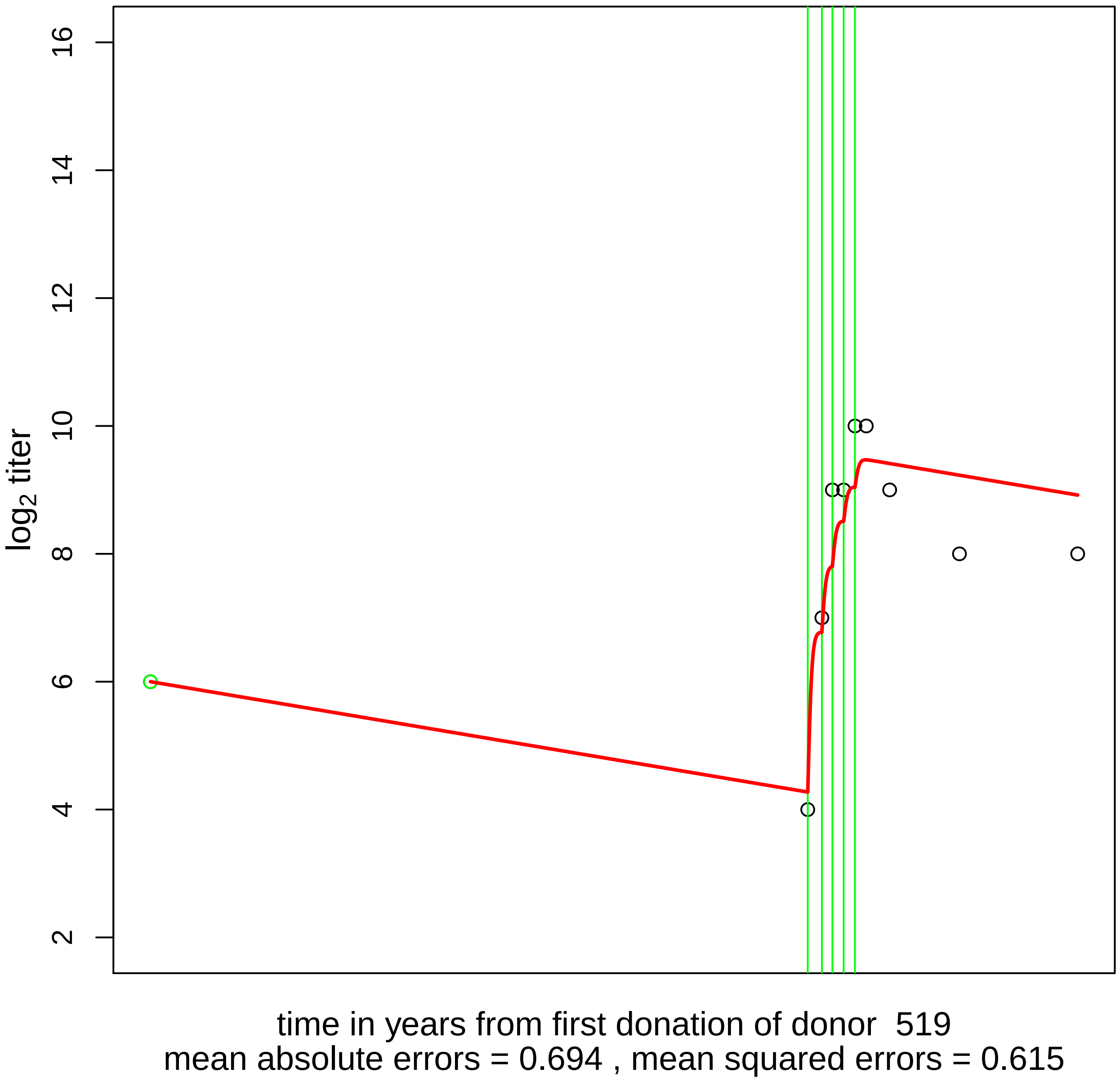

log2 titer
2
4
6
8
10
12
14
16
time in years from first donation of donor 519
mean absolute errors = 0.694 , mean squared errors = 0.615

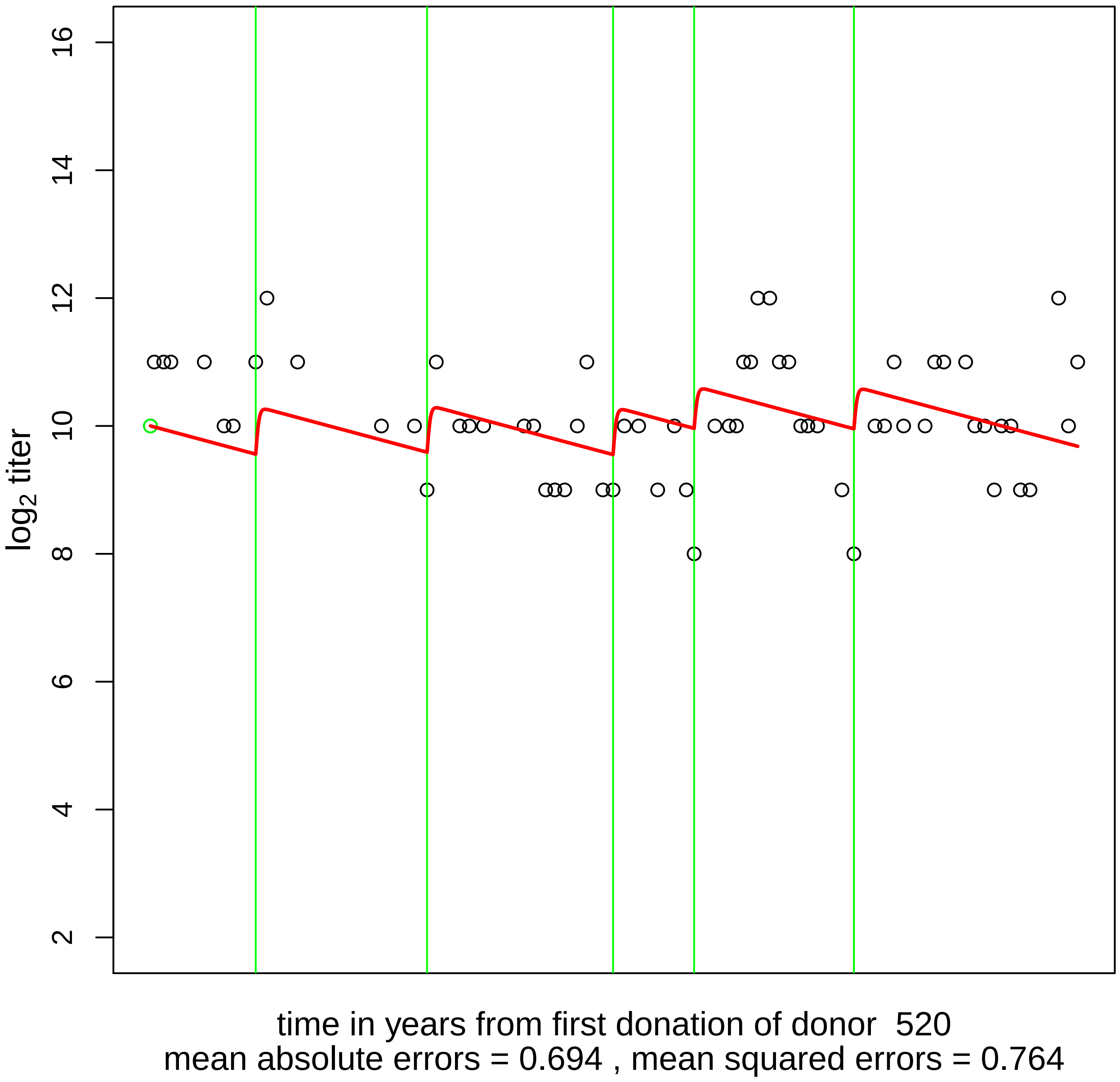

log2 titer
2
4
6
8
10
12
14
16
time in years from first donation of donor 520
mean absolute errors = 0.694 , mean squared errors = 0.764

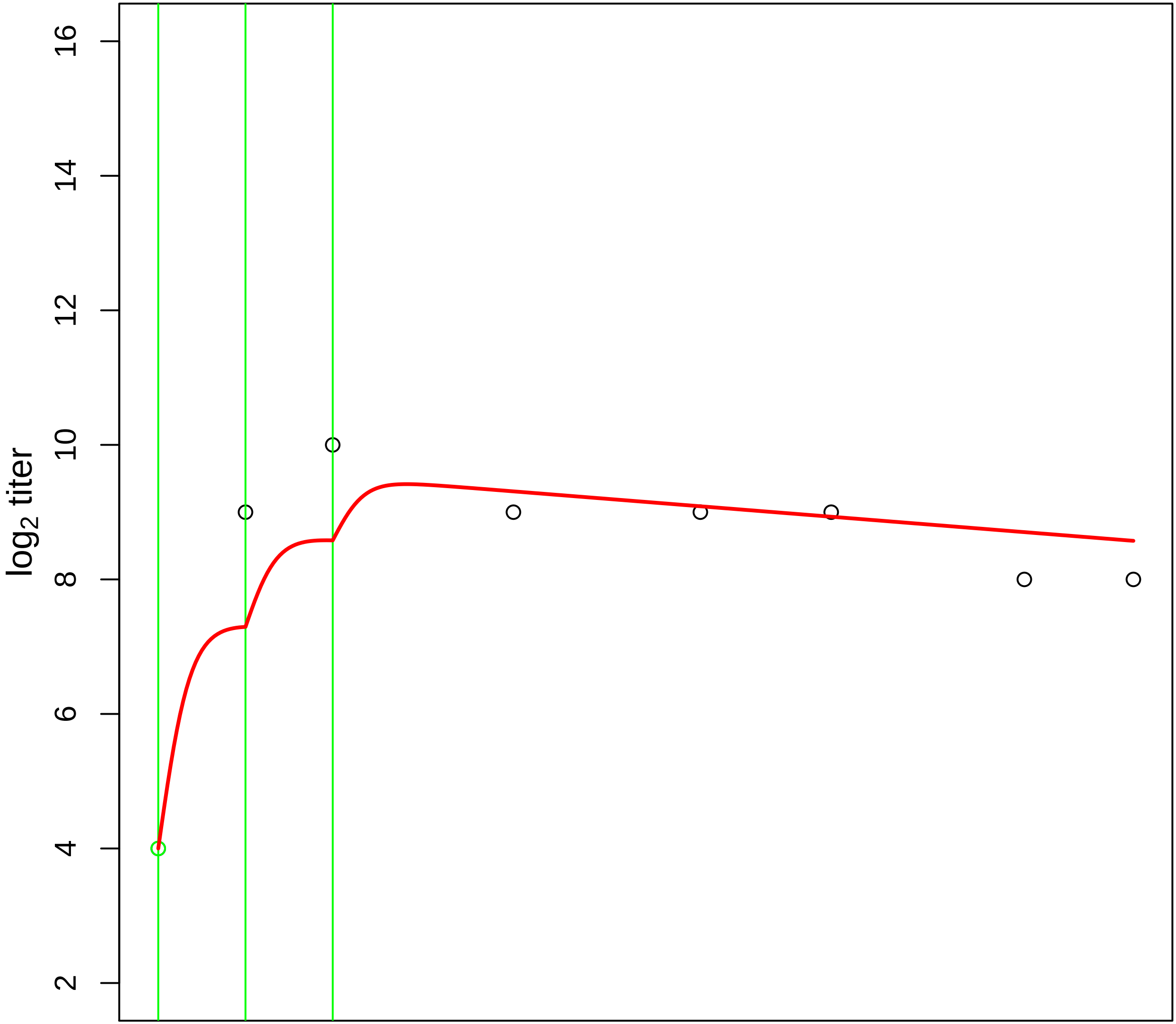

time in years from first donation of donor 521
mean absolute errors = 0.695 , mean squared errors = 0.837

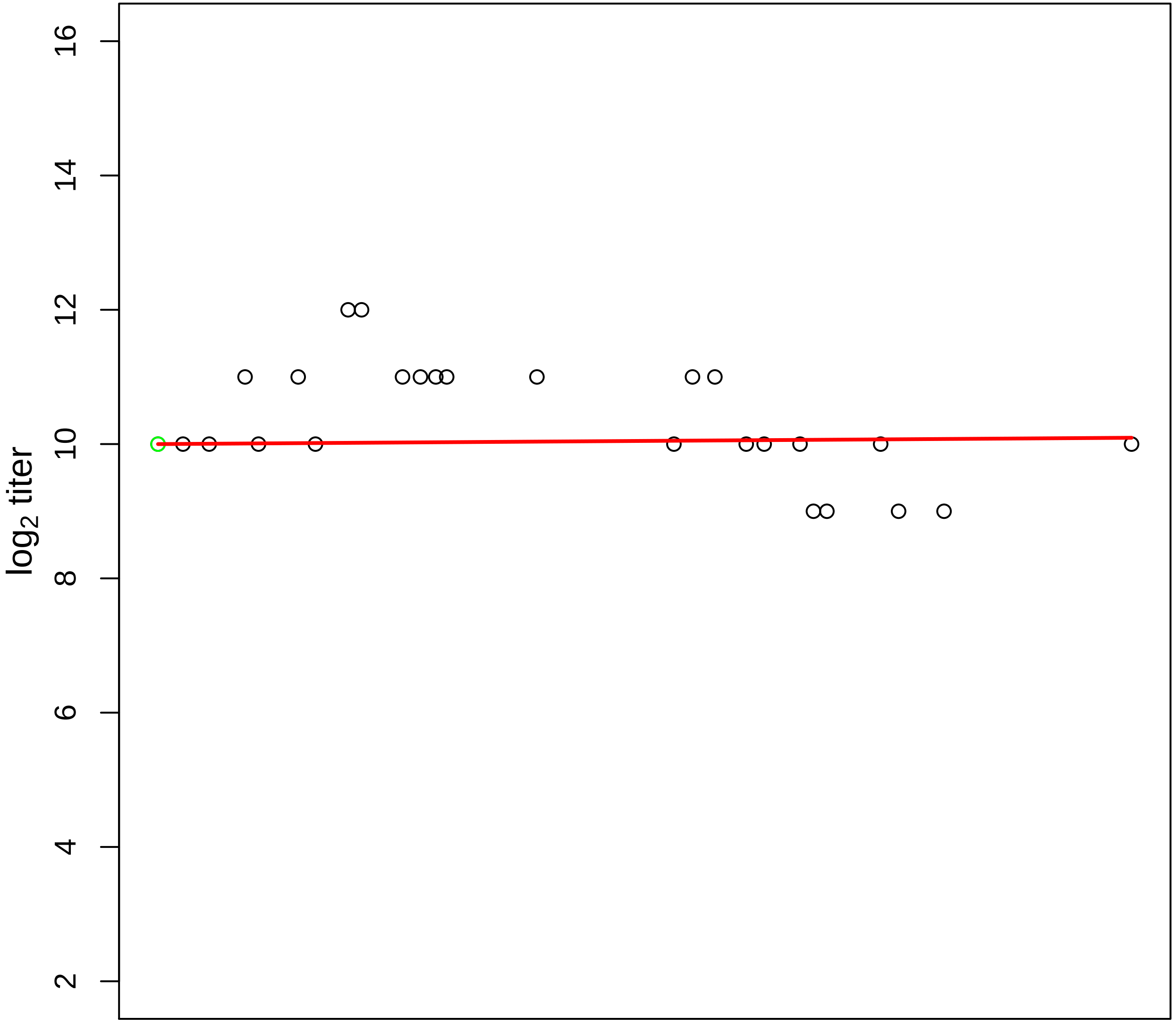

time in years from first donation of donor 522
mean absolute errors = 0.696 , mean squared errors = 0.837

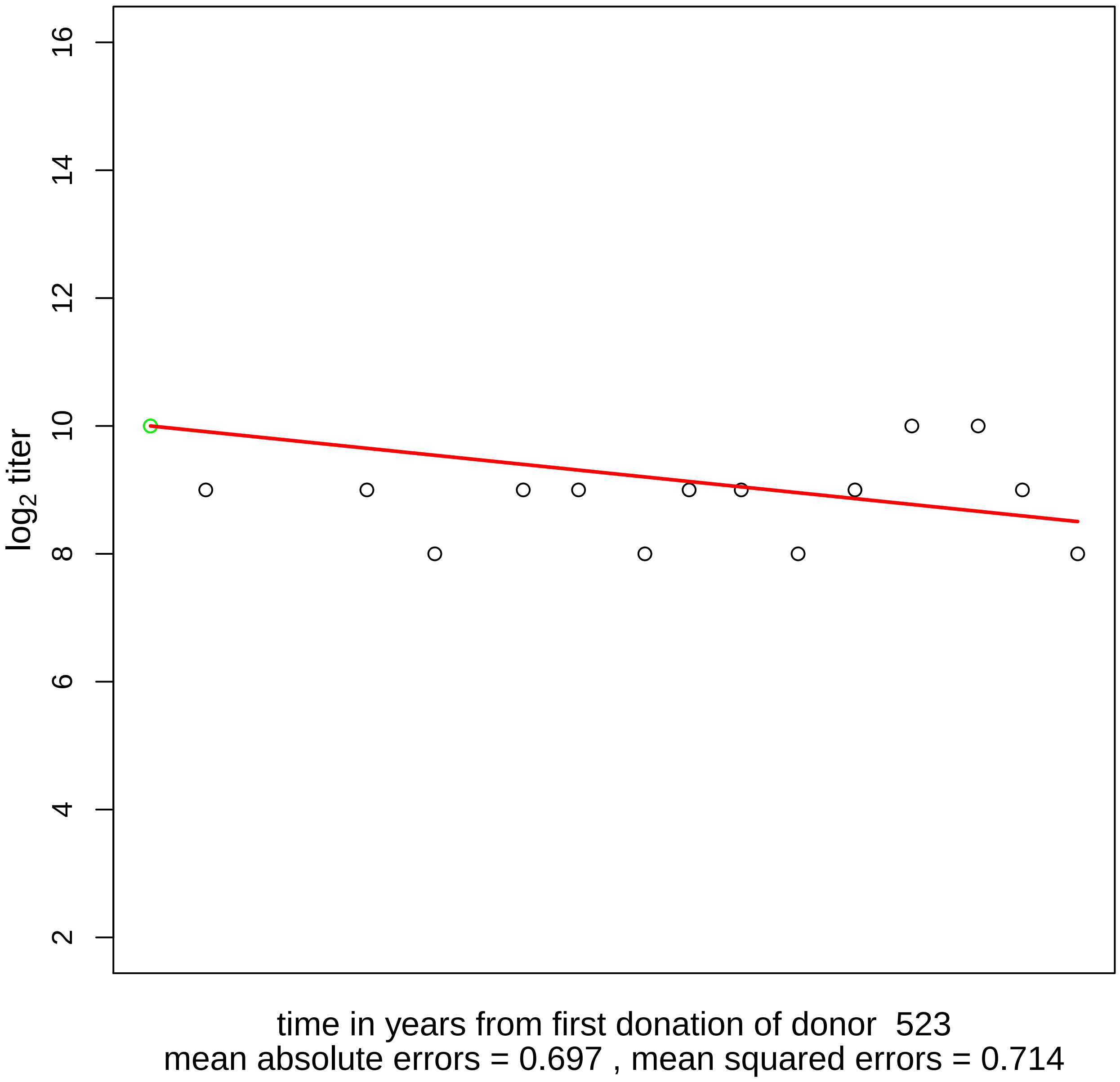

16
14
12
10
8
6
4
2
$\log_2$ titer
time in years from first donation of donor 523
mean absolute errors = 0.697 , mean squared errors = 0.714

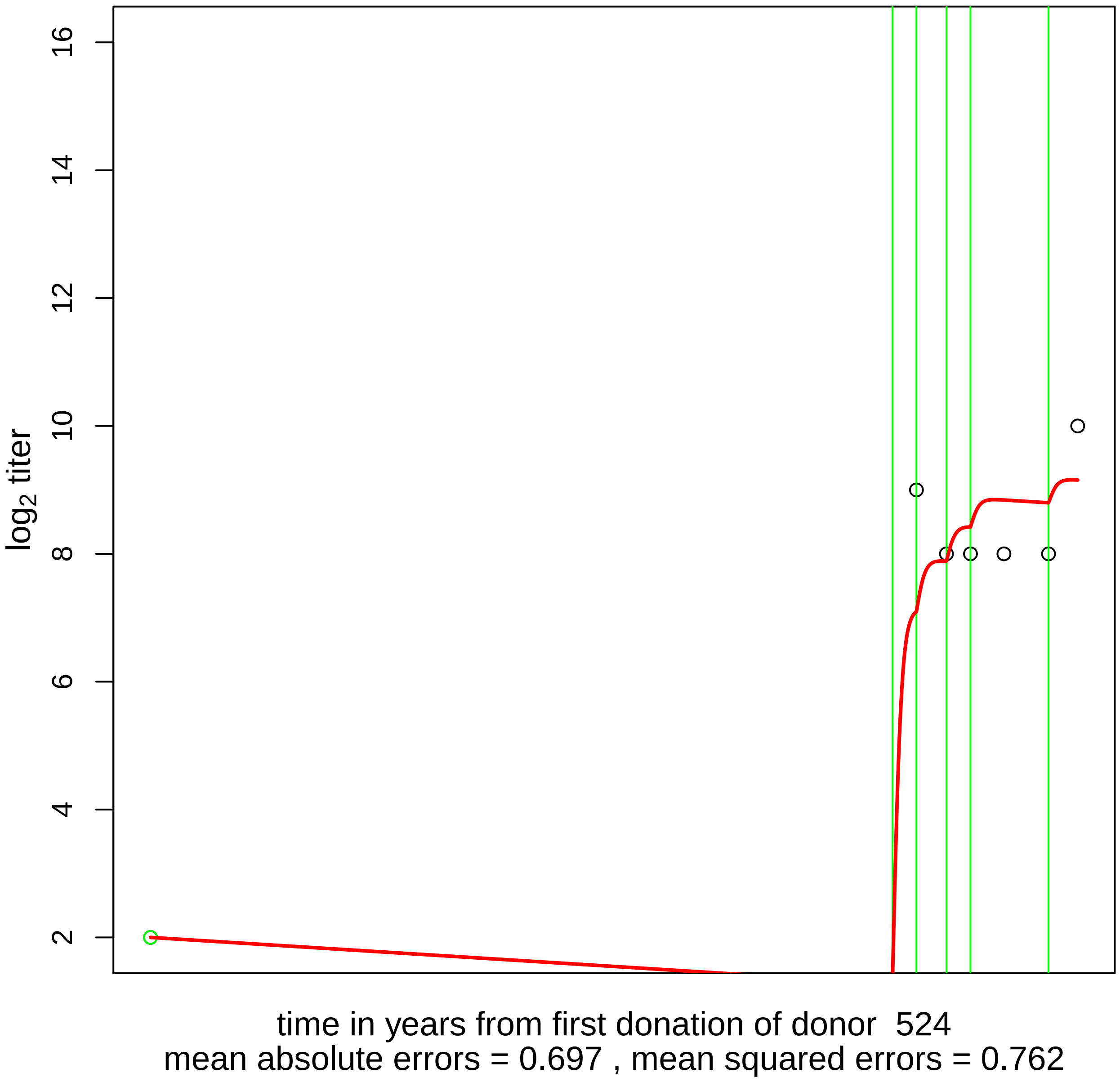

16
14
12
10
8
6
4
2
$\log_2$ titer
time in years from first donation of donor 524
mean absolute errors = 0.697 , mean squared errors = 0.762

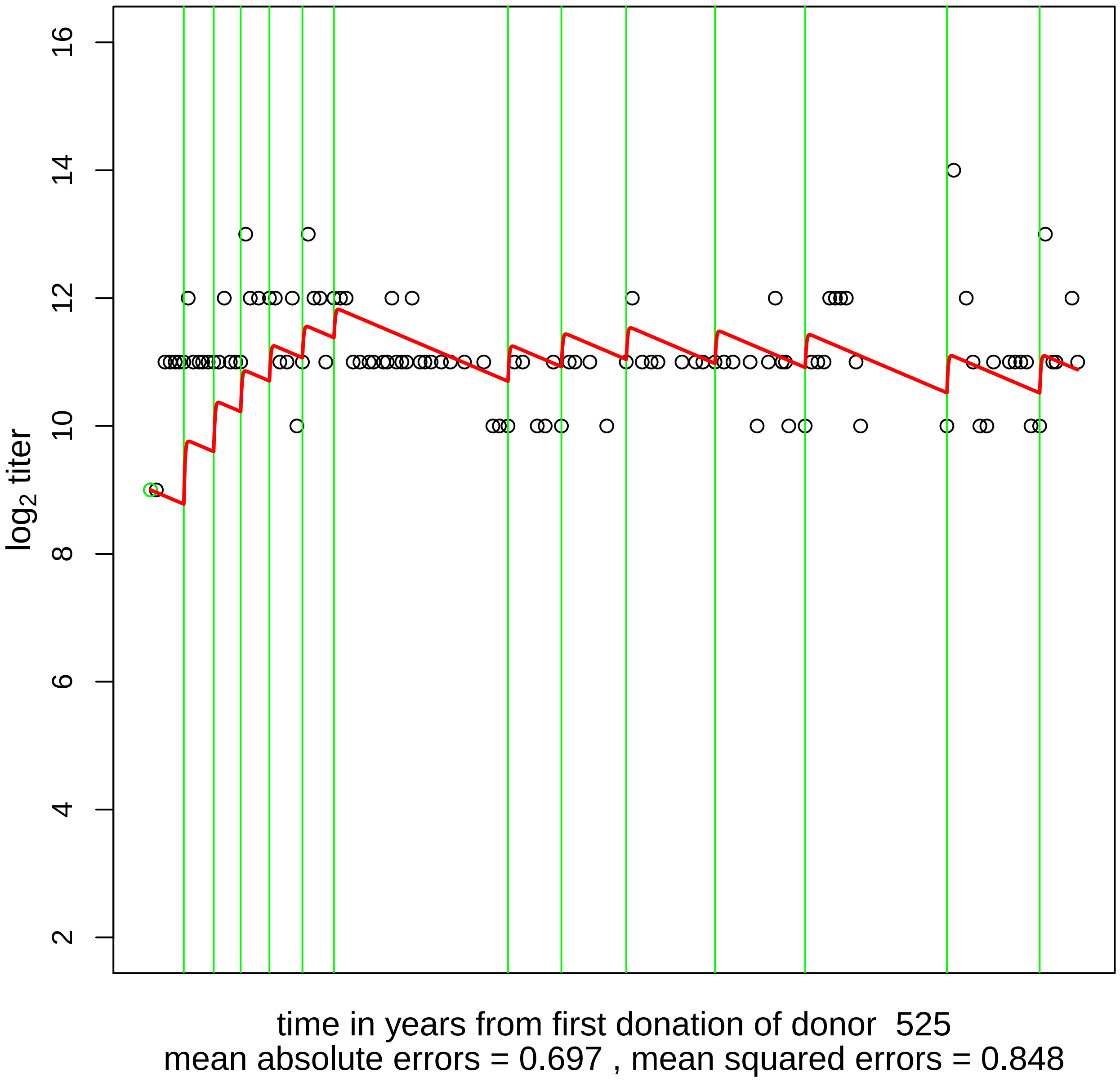

16
14
12
10
8
6
4
2
log2 titer
time in years from first donation of donor 525
mean absolute errors = 0.697 , mean squared errors = 0.848

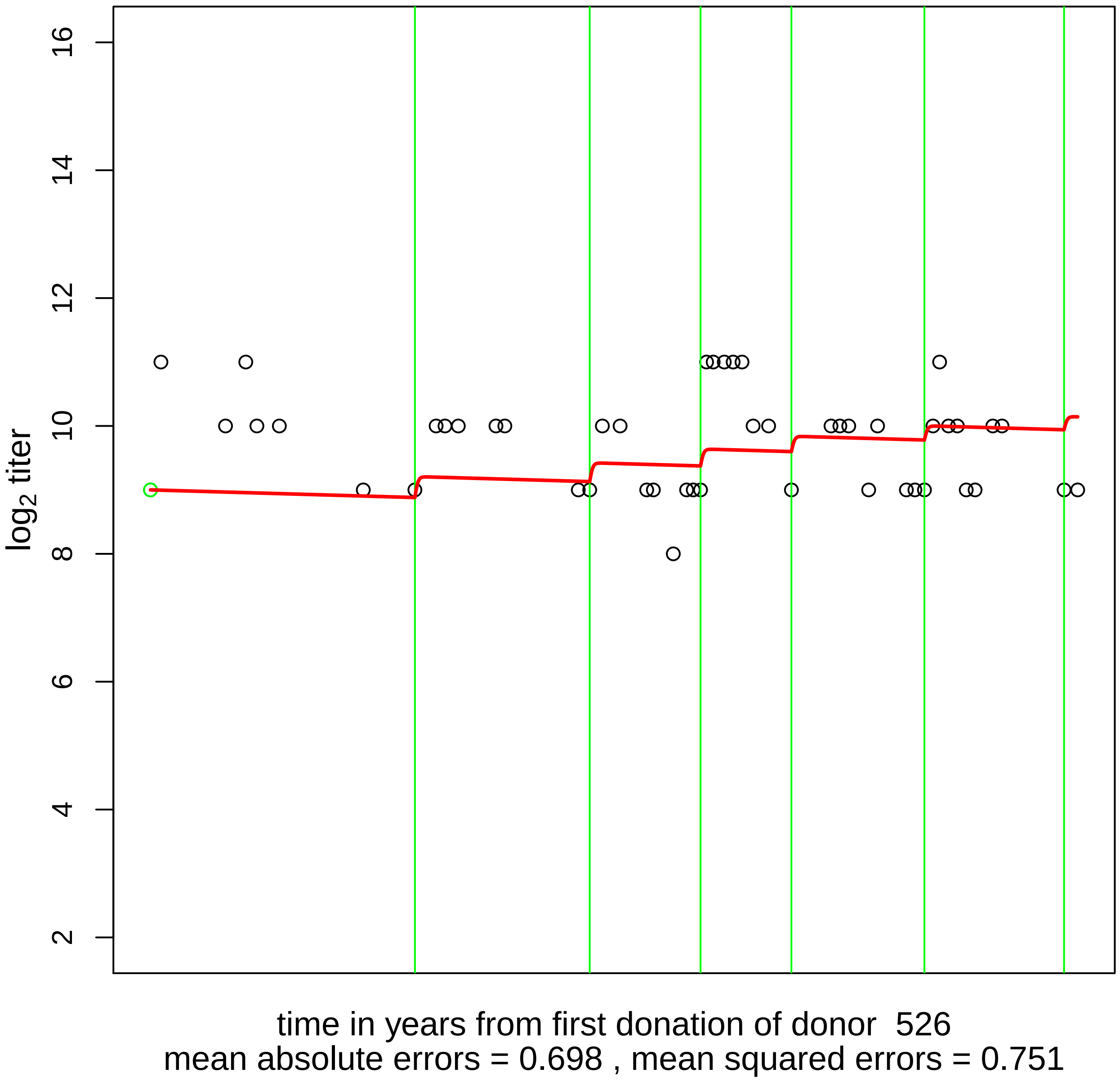

16
14
12
10
8
6
4
2
log2 titer
time in years from first donation of donor 526
mean absolute errors = 0.698 , mean squared errors = 0.751

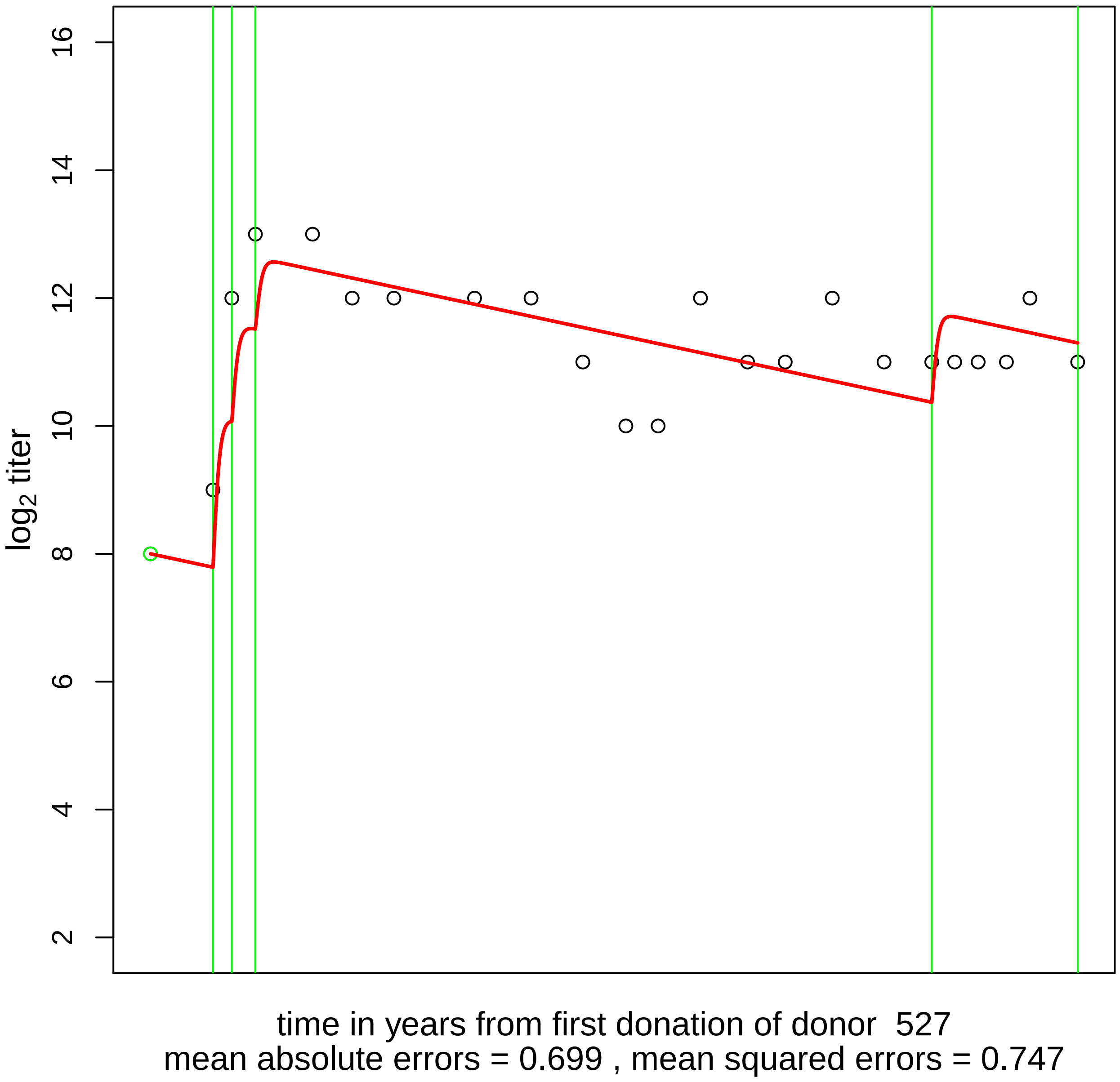

log2 titer
16
14
12
10
8
6
4
2
time in years from first donation of donor 527
mean absolute errors = 0.699 , mean squared errors = 0.747

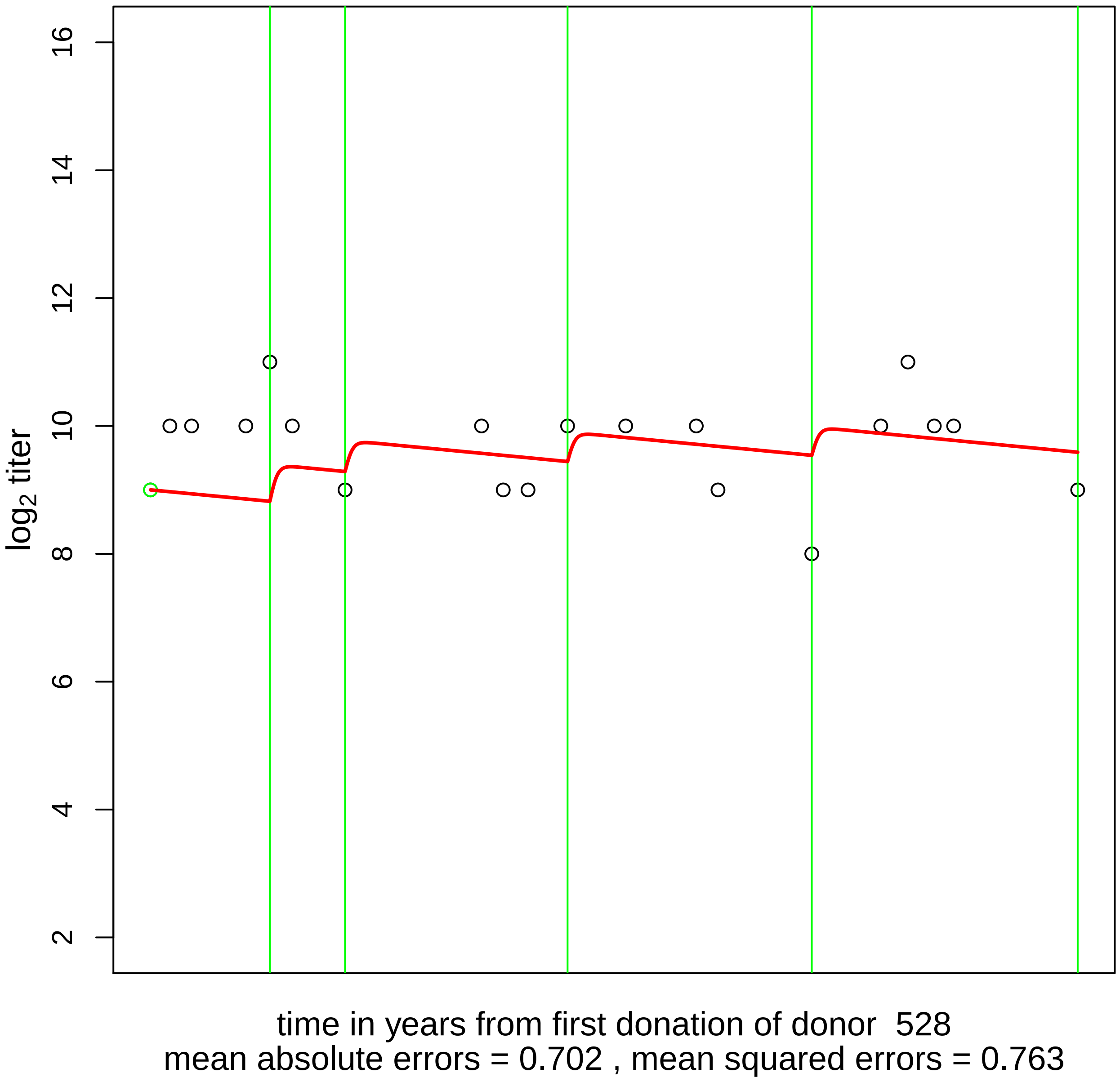

16
14
12
10
8
6
4
2
log2 titer
time in years from first donation of donor 528
mean absolute errors = 0.702 , mean squared errors = 0.763

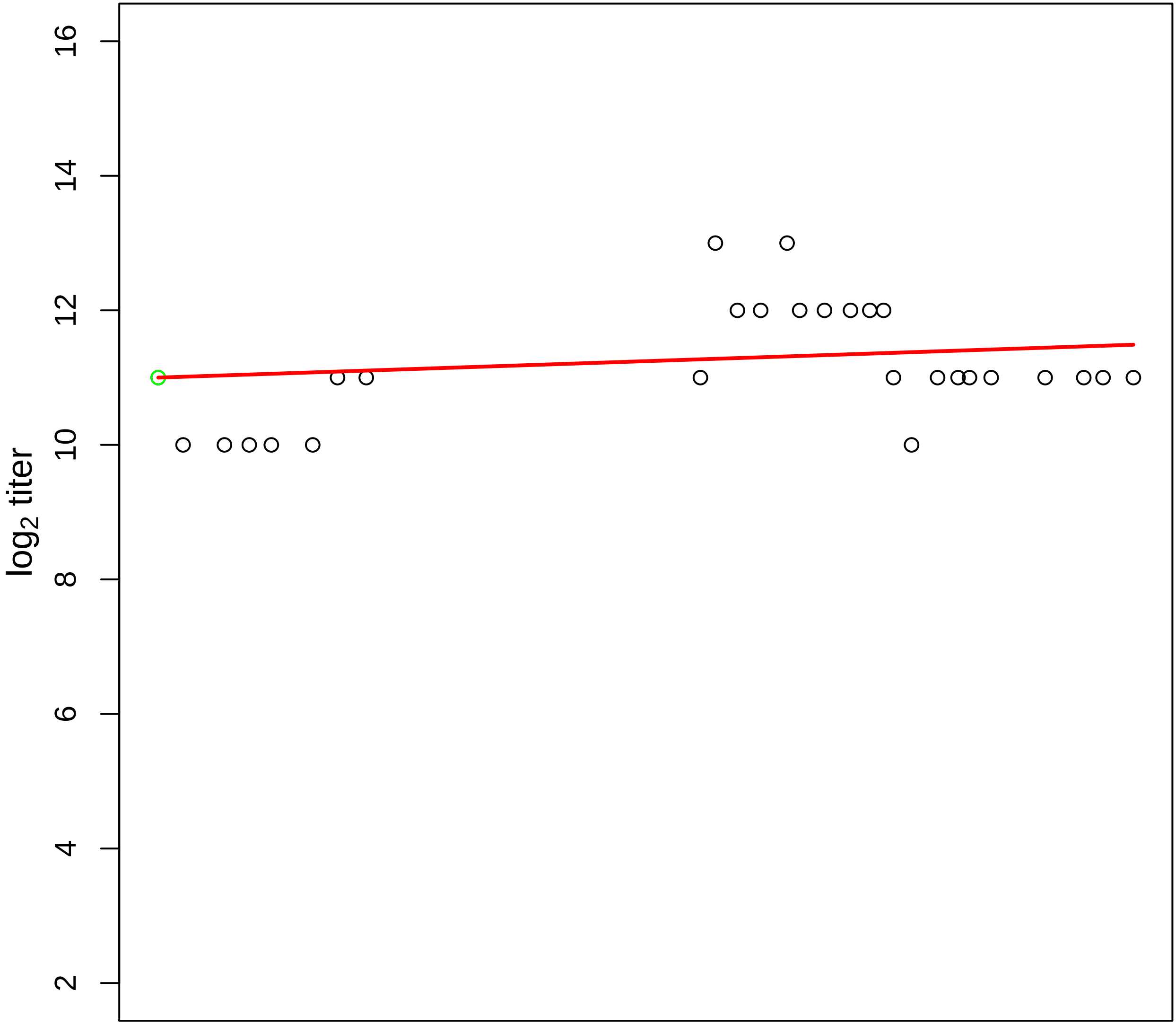

time in years from first donation of donor 529
mean absolute errors = 0.704 , mean squared errors = 0.669

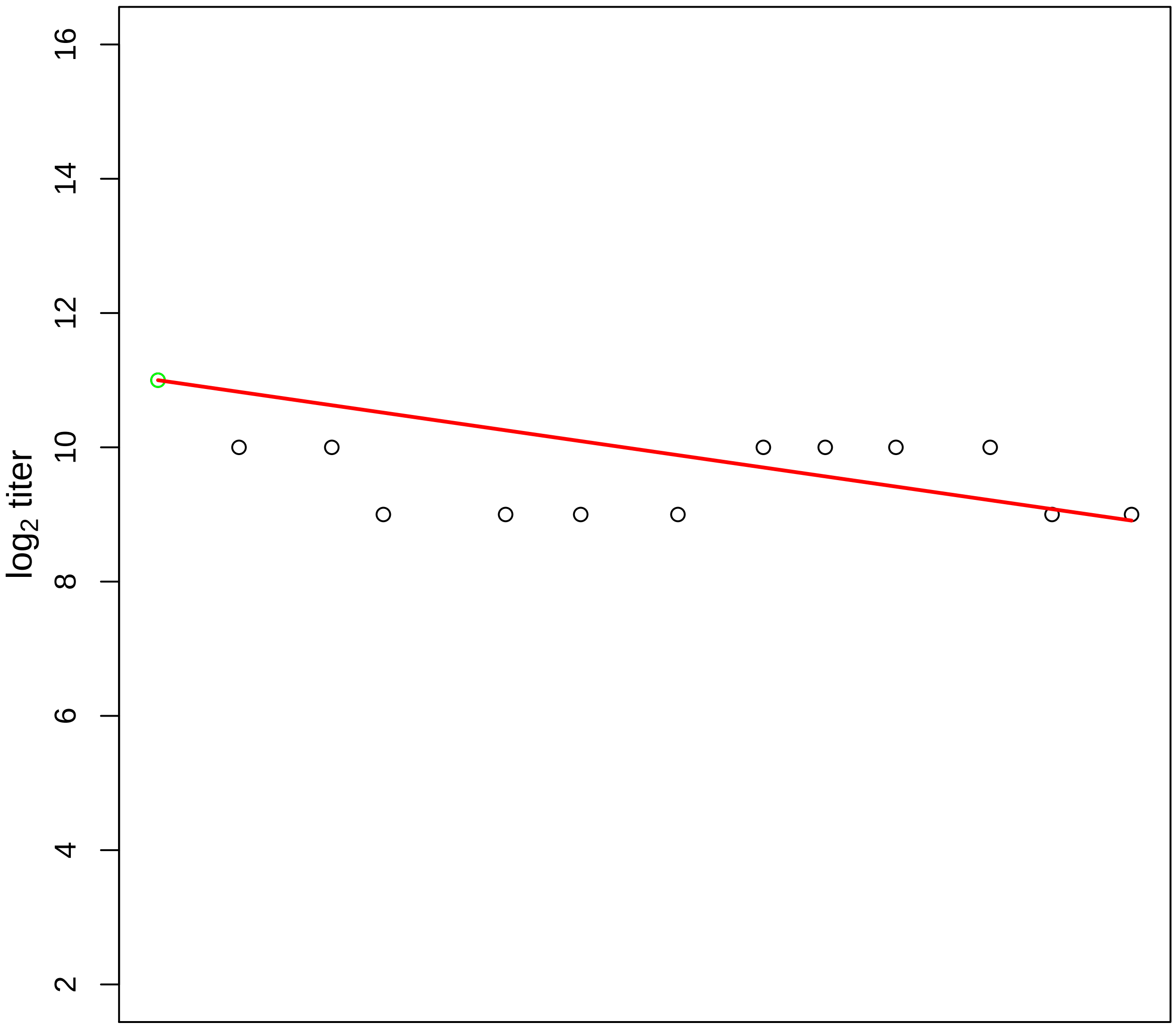

time in years from first donation of donor 530
mean absolute errors = 0.706 , mean squared errors = 0.681

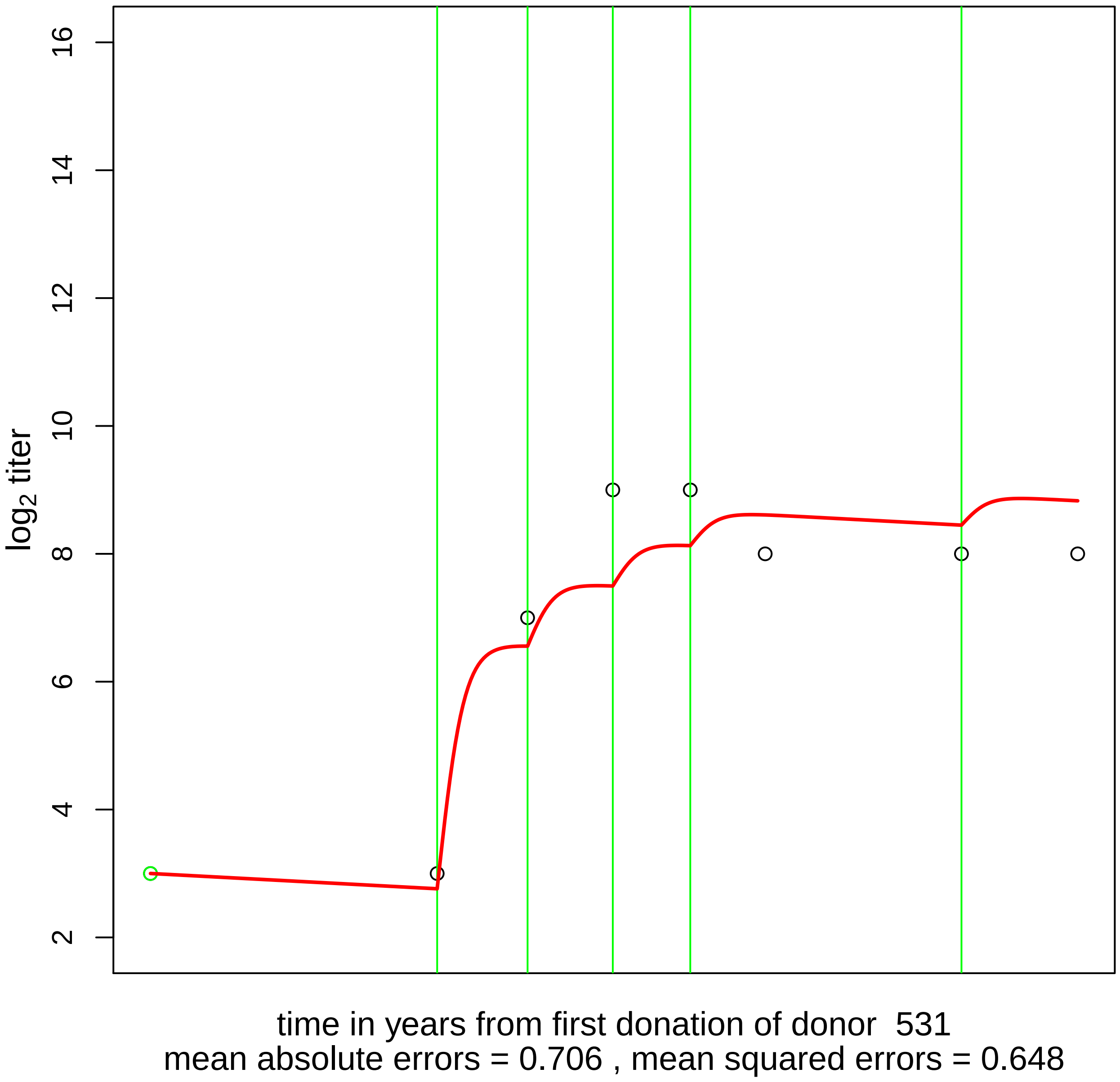

2
4
6
8
10
12
14
16
log2 titer
time in years from first donation of donor 531
mean absolute errors = 0.706 , mean squared errors = 0.648

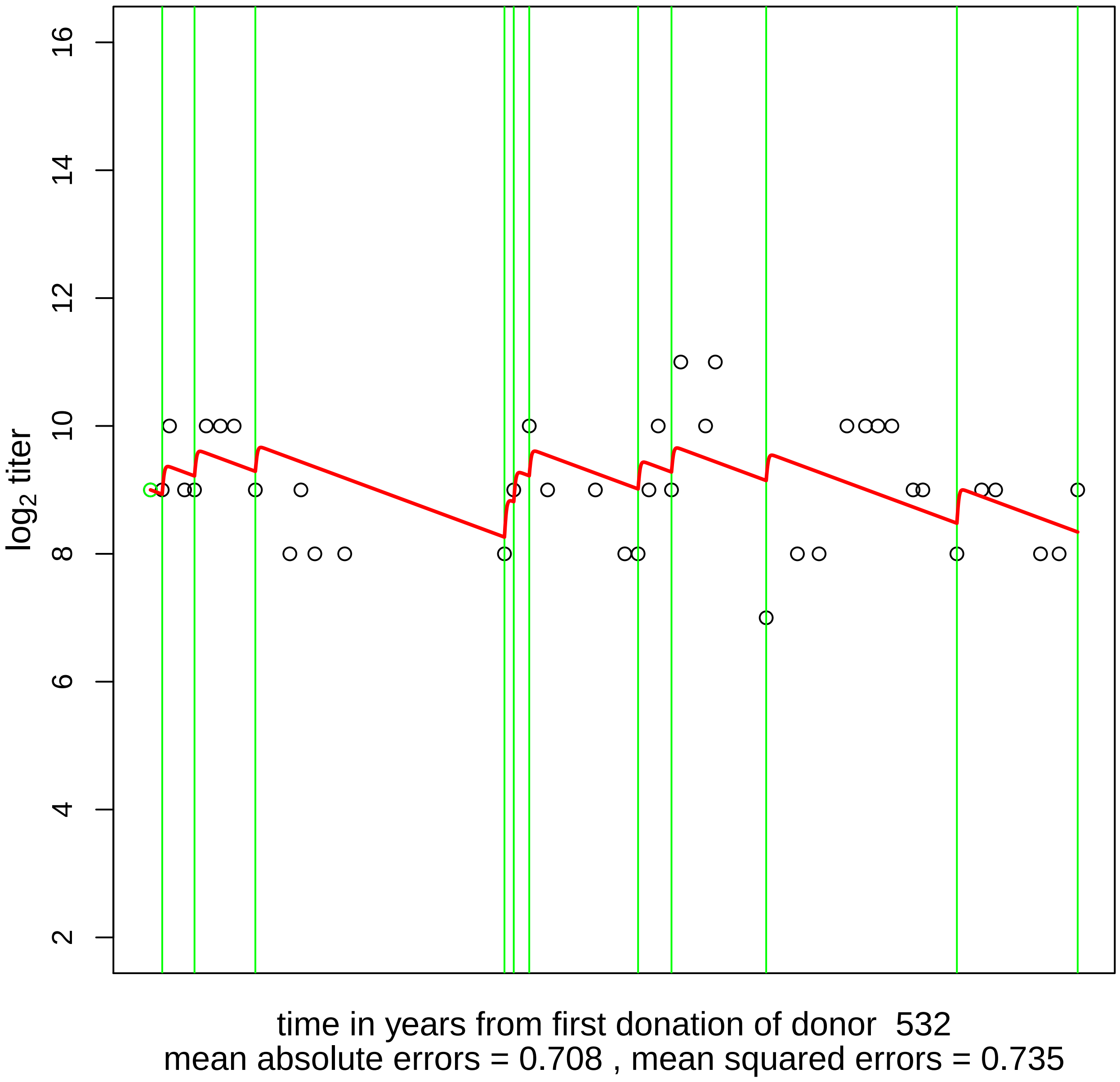

log2 titer
2
4
6
8
10
12
14
16
time in years from first donation of donor 532
mean absolute errors = 0.708 , mean squared errors = 0.735

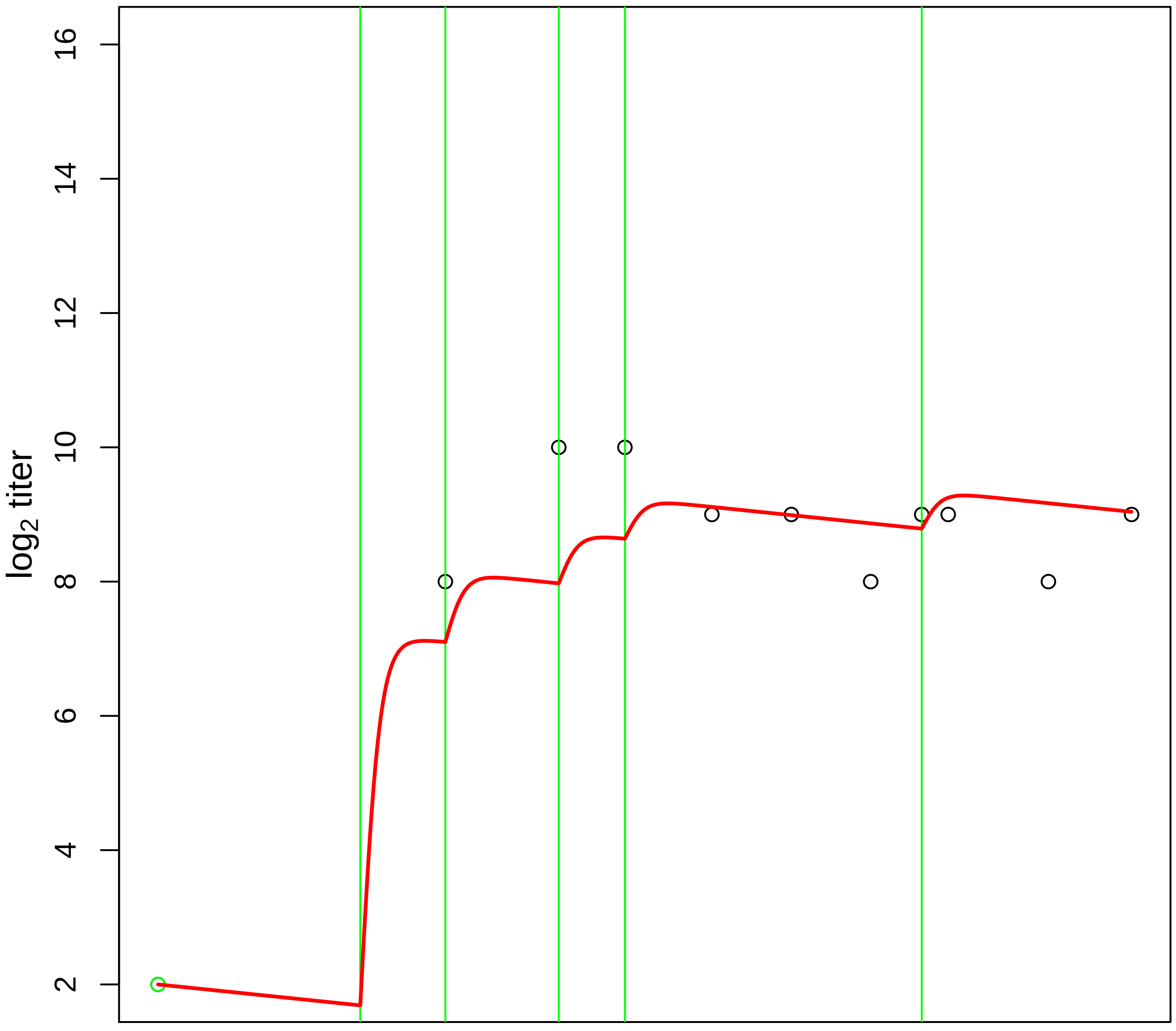

time in years from first donation of donor 533
mean absolute errors = 0.708 , mean squared errors = 0.852

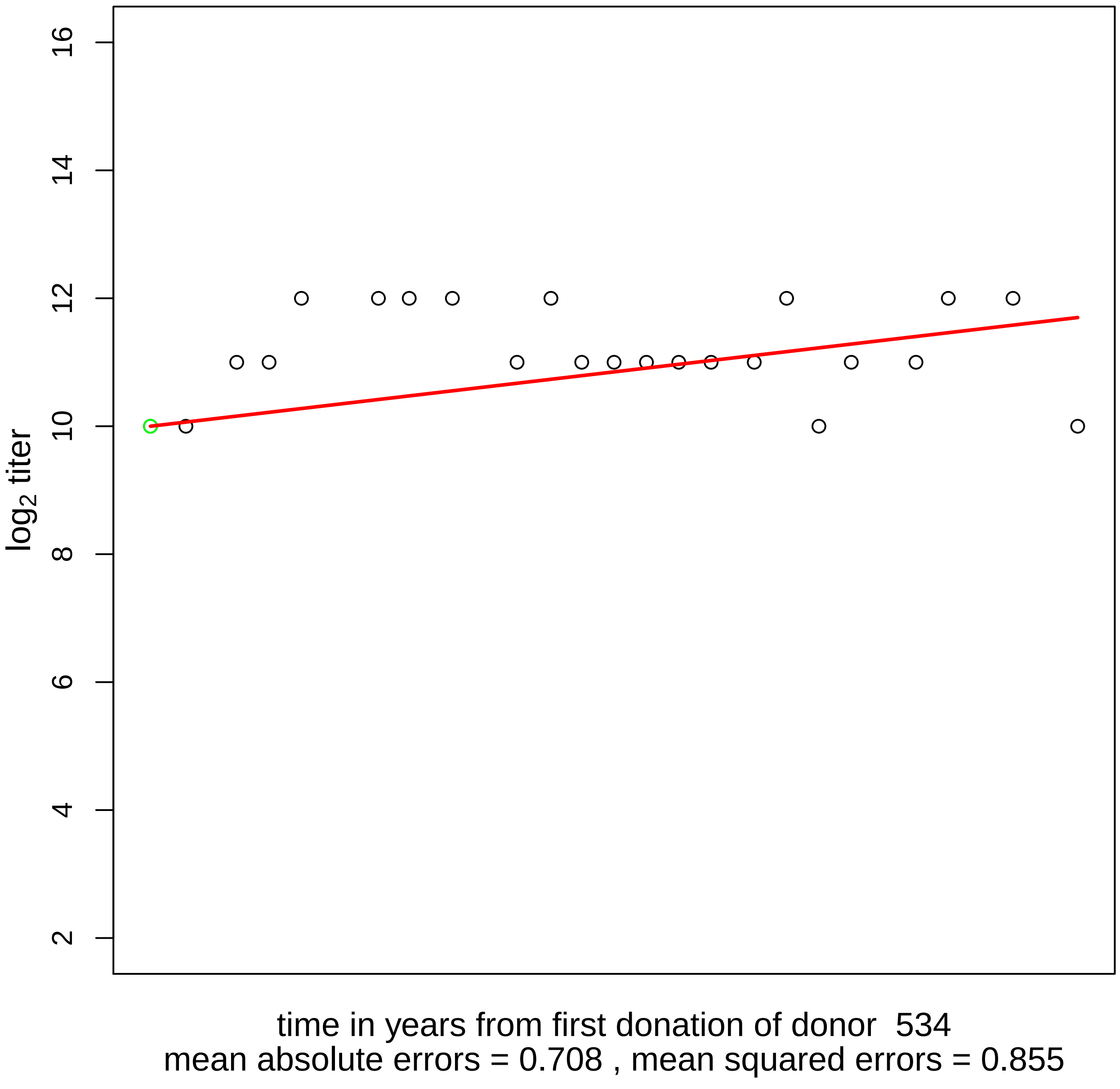

16
14
12
10
8
6
4
2
log2 titer
time in years from first donation of donor 534
mean absolute errors = 0.708 , mean squared errors = 0.855

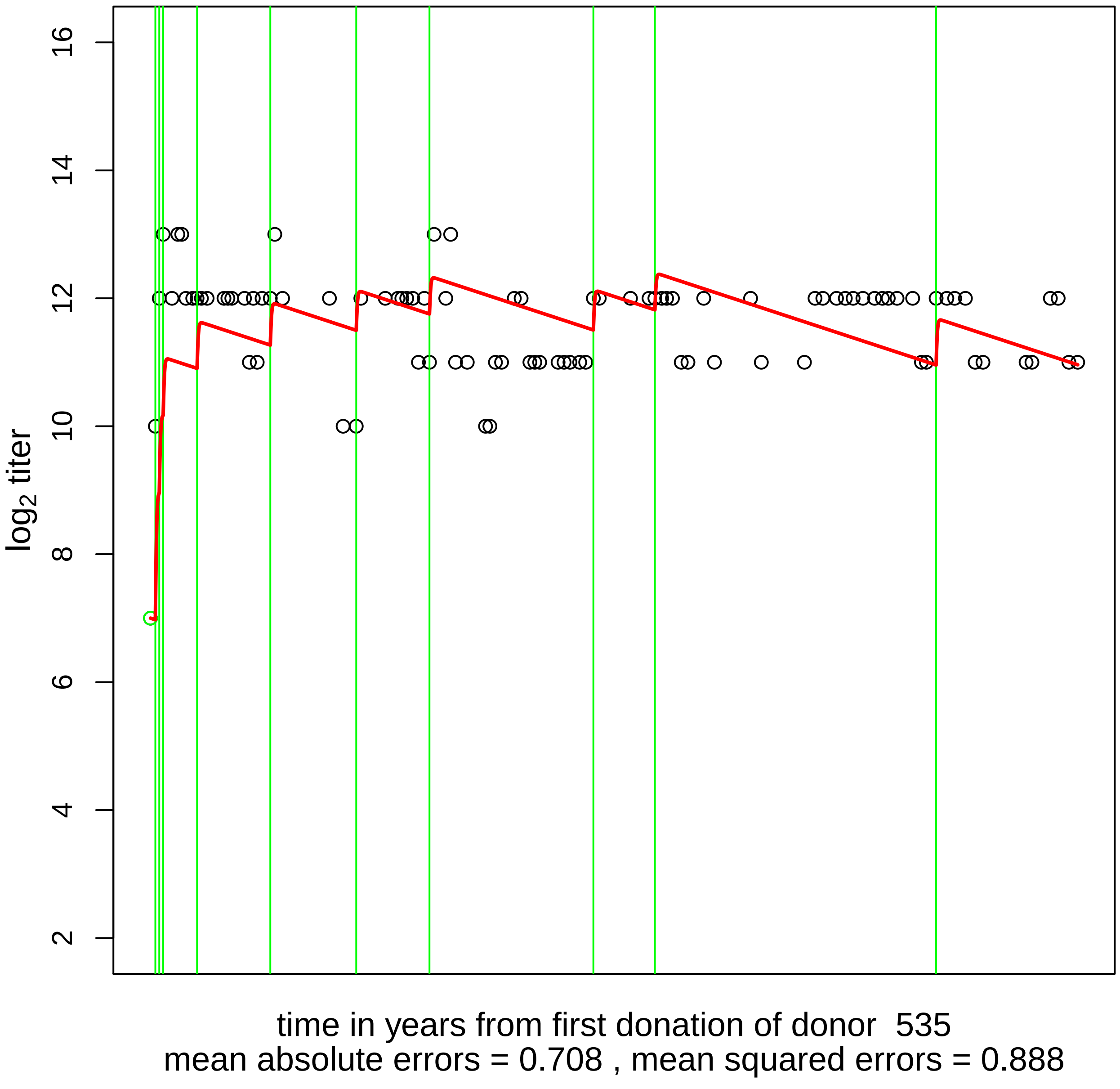

16
14
12
10
8
6
4
2
log2 titer
time in years from first donation of donor 535
mean absolute errors = 0.708 , mean squared errors = 0.888

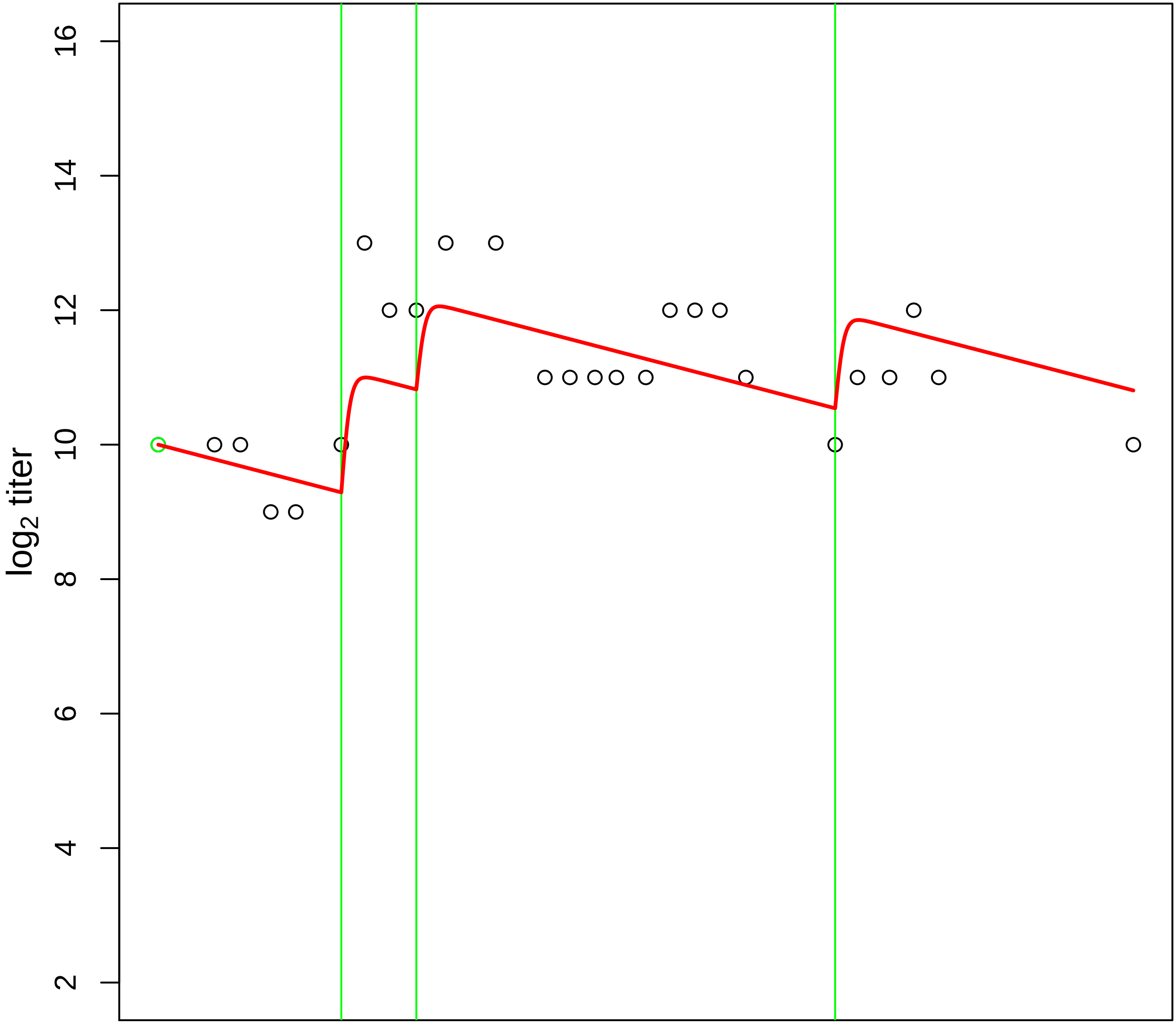

time in years from first donation of donor 536
mean absolute errors = 0.709 , mean squared errors = 0.656

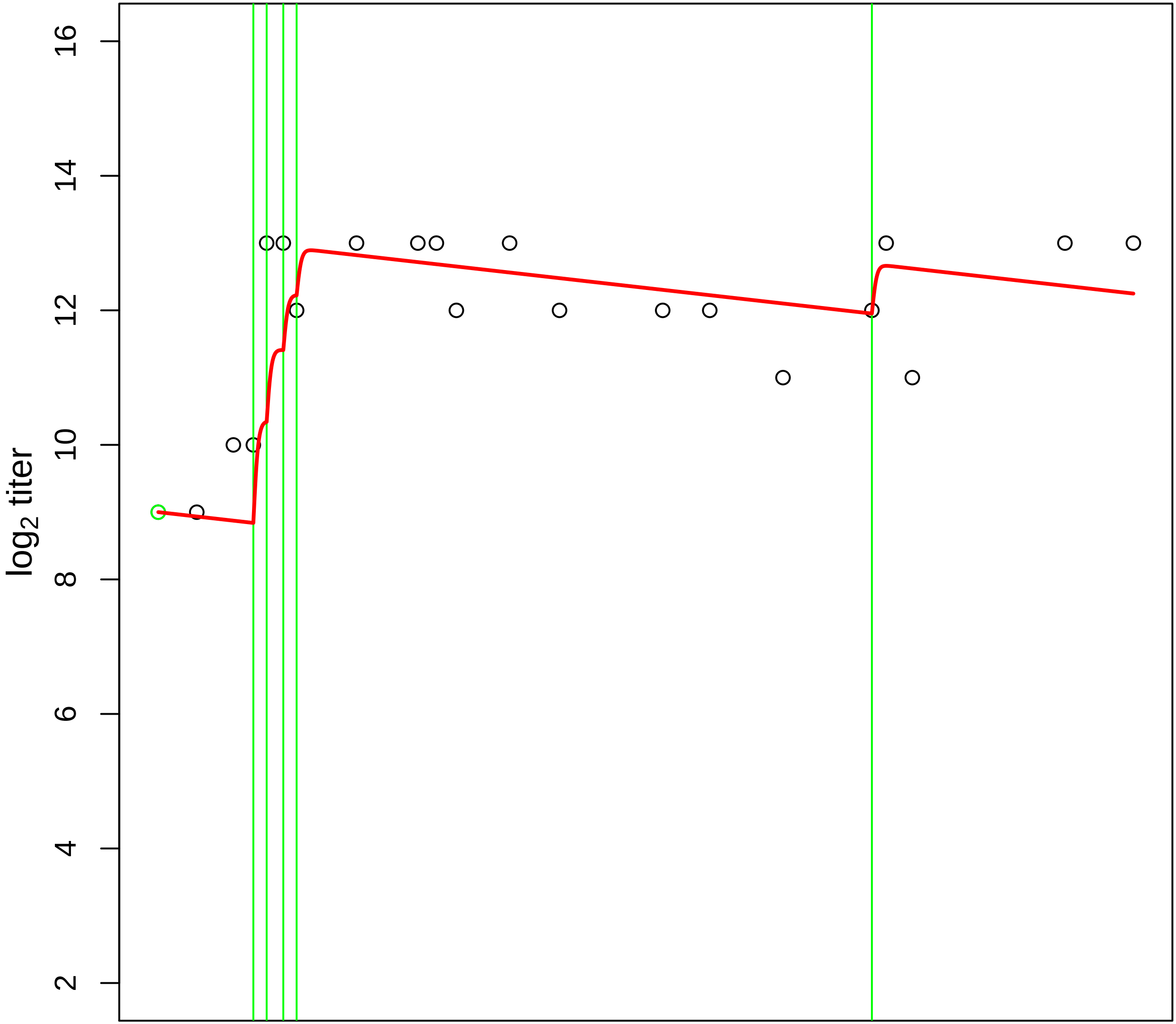

time in years from first donation of donor 537
mean absolute errors = 0.71 , mean squared errors = 0.92

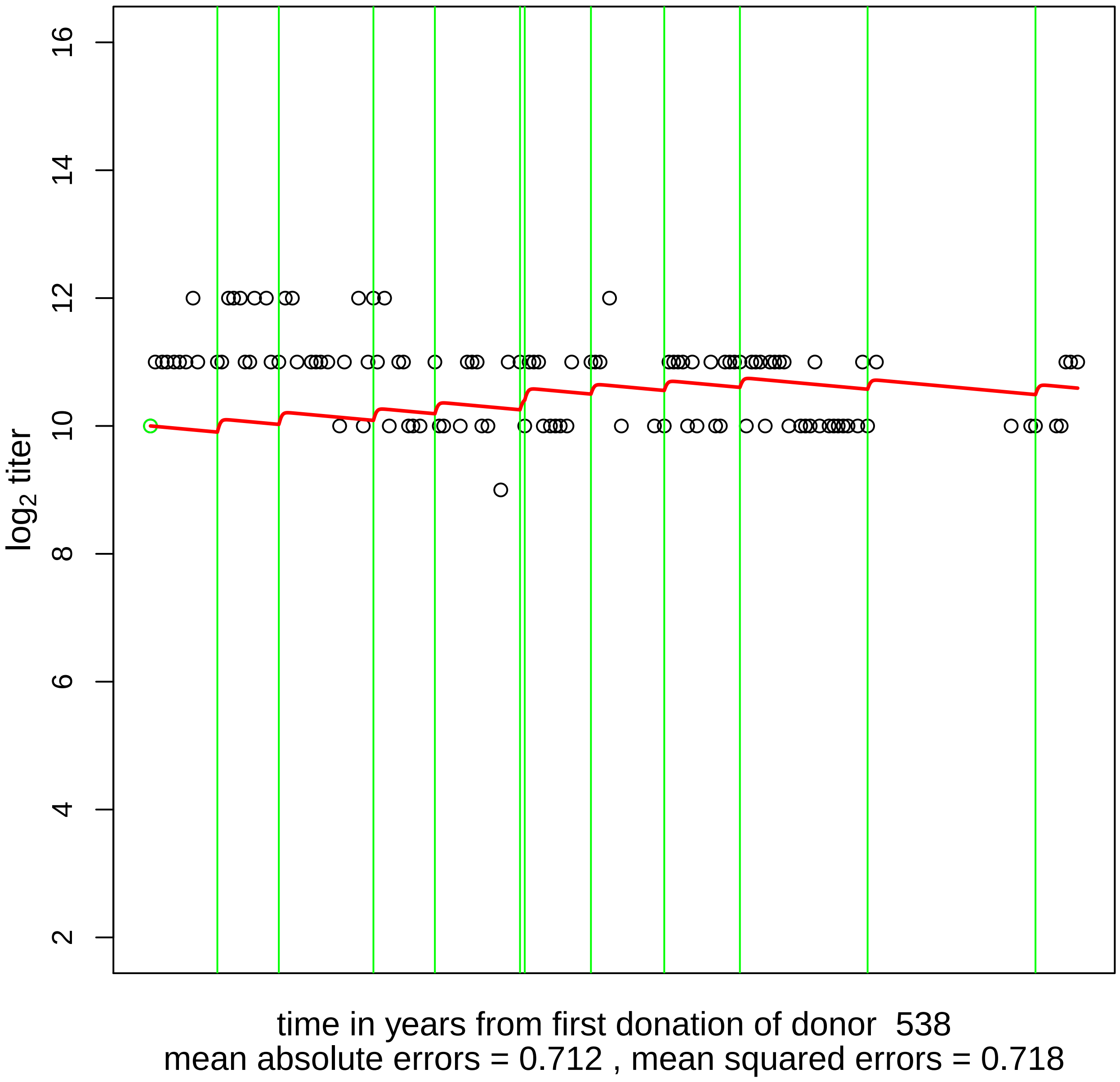

2
4
6
8
10
12
14
16
log2 titer
time in years from first donation of donor 538
mean absolute errors = 0.712 , mean squared errors = 0.718

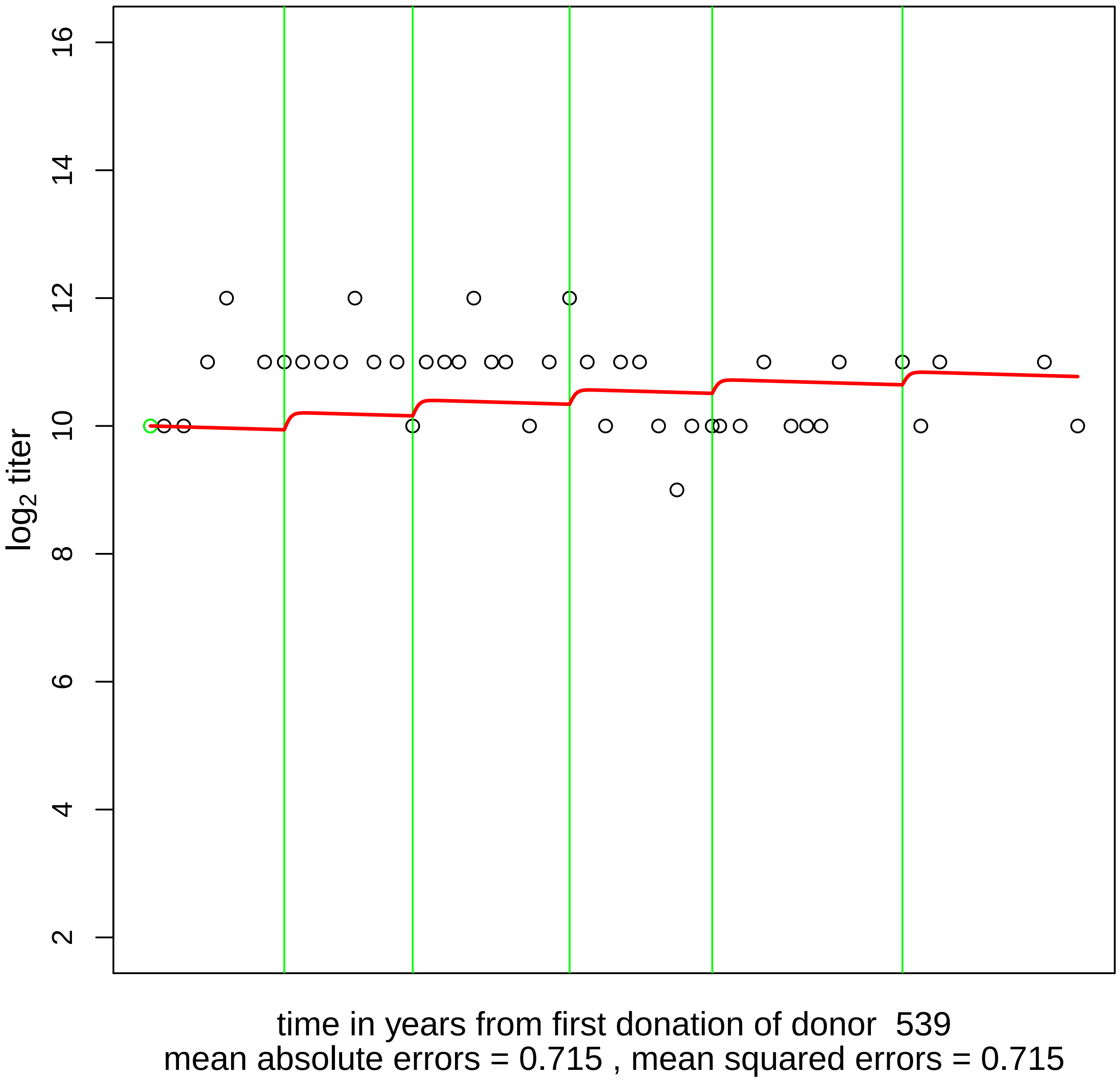

16
14
12
10
8
6
4
2
$\log_2$ titer
time in years from first donation of donor 539
mean absolute errors = 0.715 , mean squared errors = 0.715

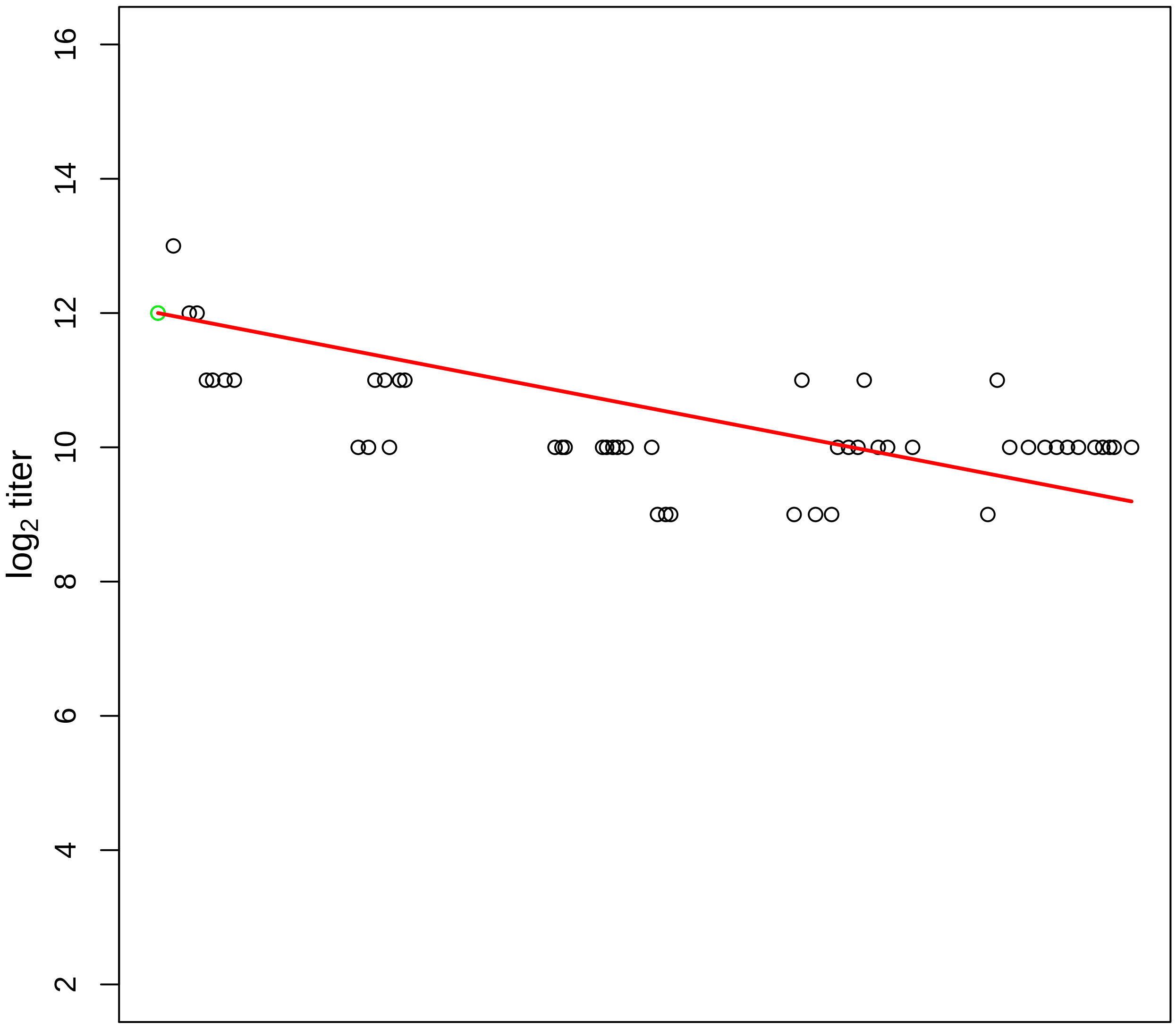

time in years from first donation of donor 540
mean absolute errors = 0.719 , mean squared errors = 0.692

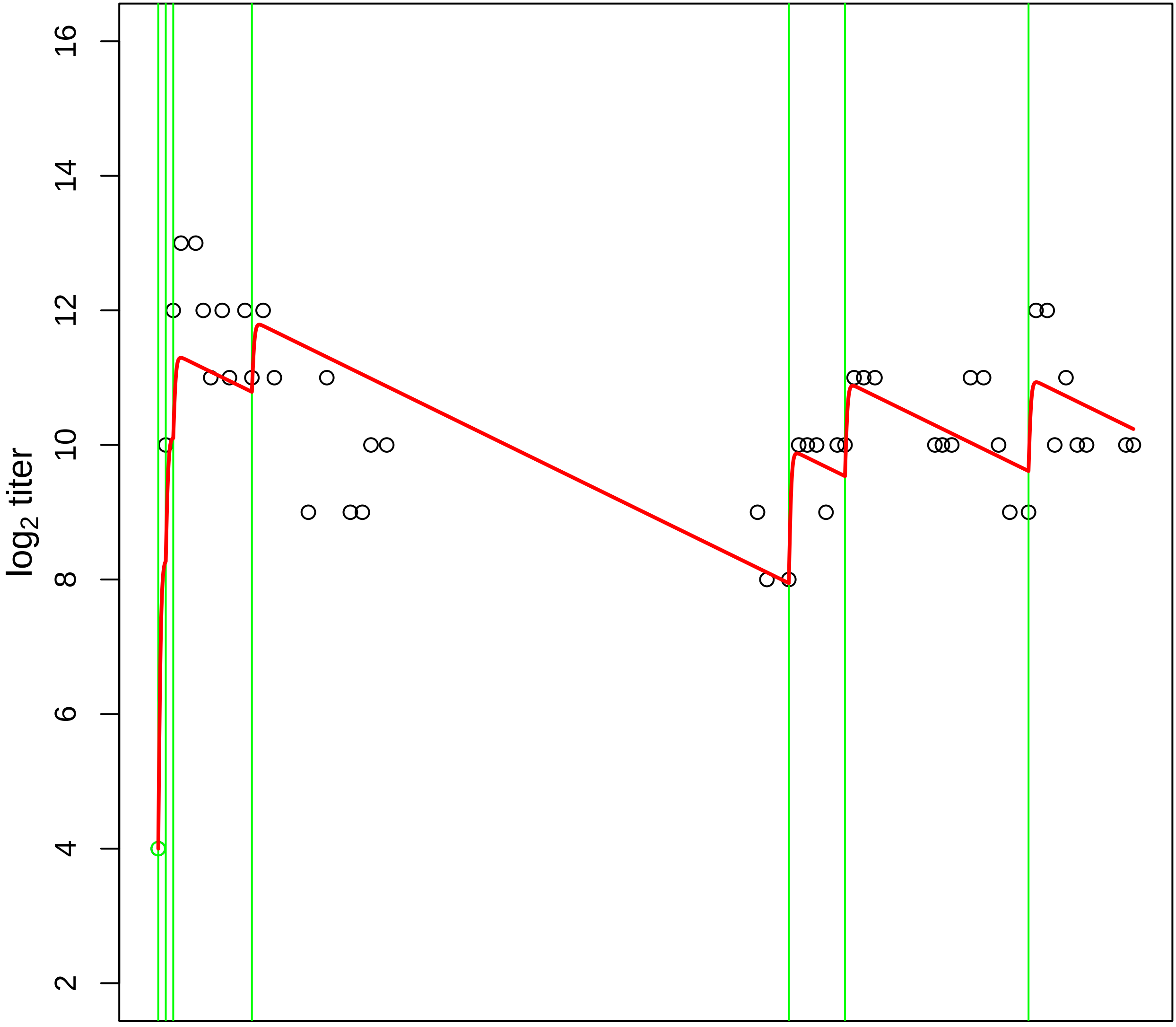

time in years from first donation of donor 541
mean absolute errors = 0.722 , mean squared errors = 0.913

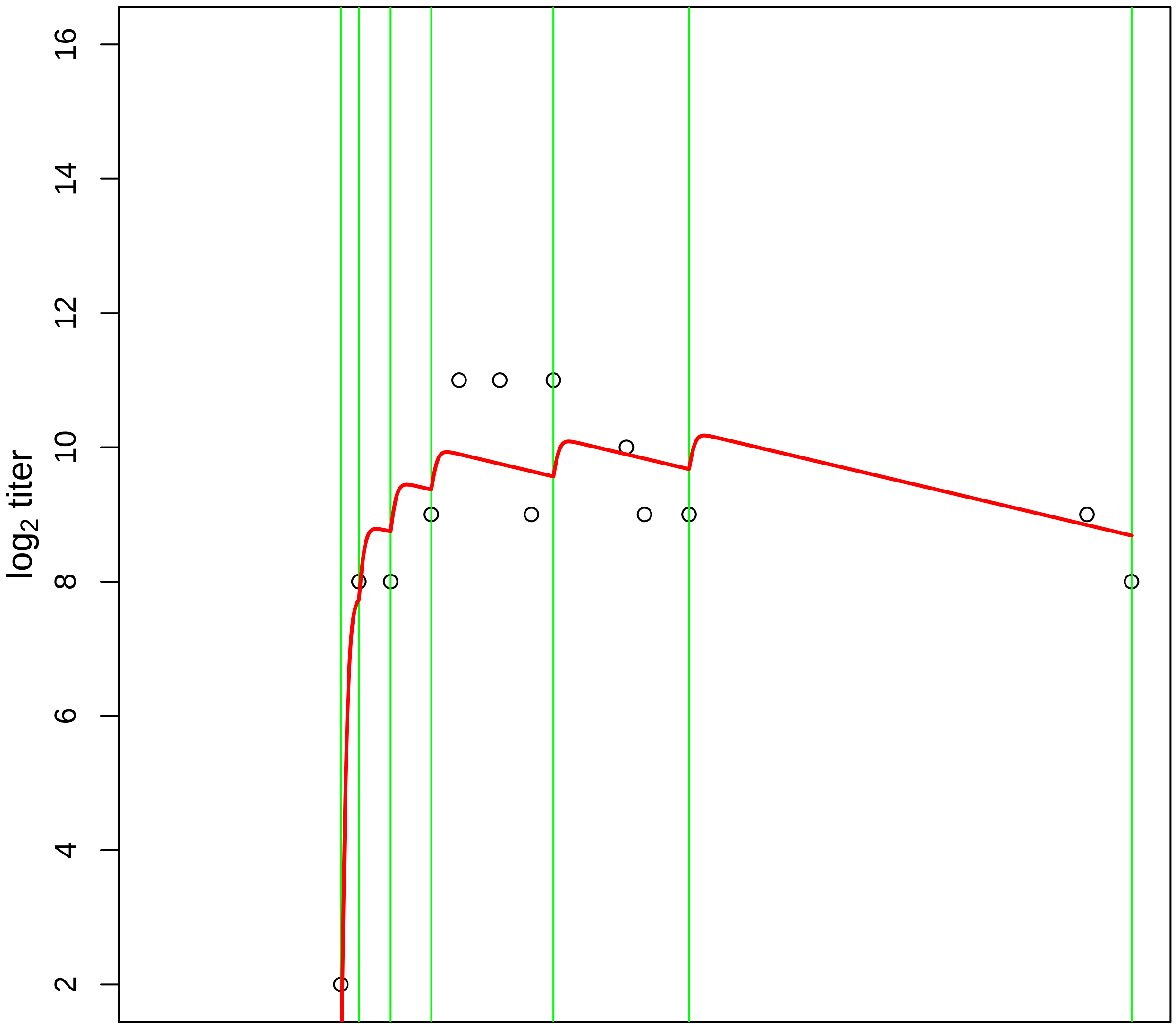

time in years from first donation of donor 542
mean absolute errors = 0.722 , mean squared errors = 0.743

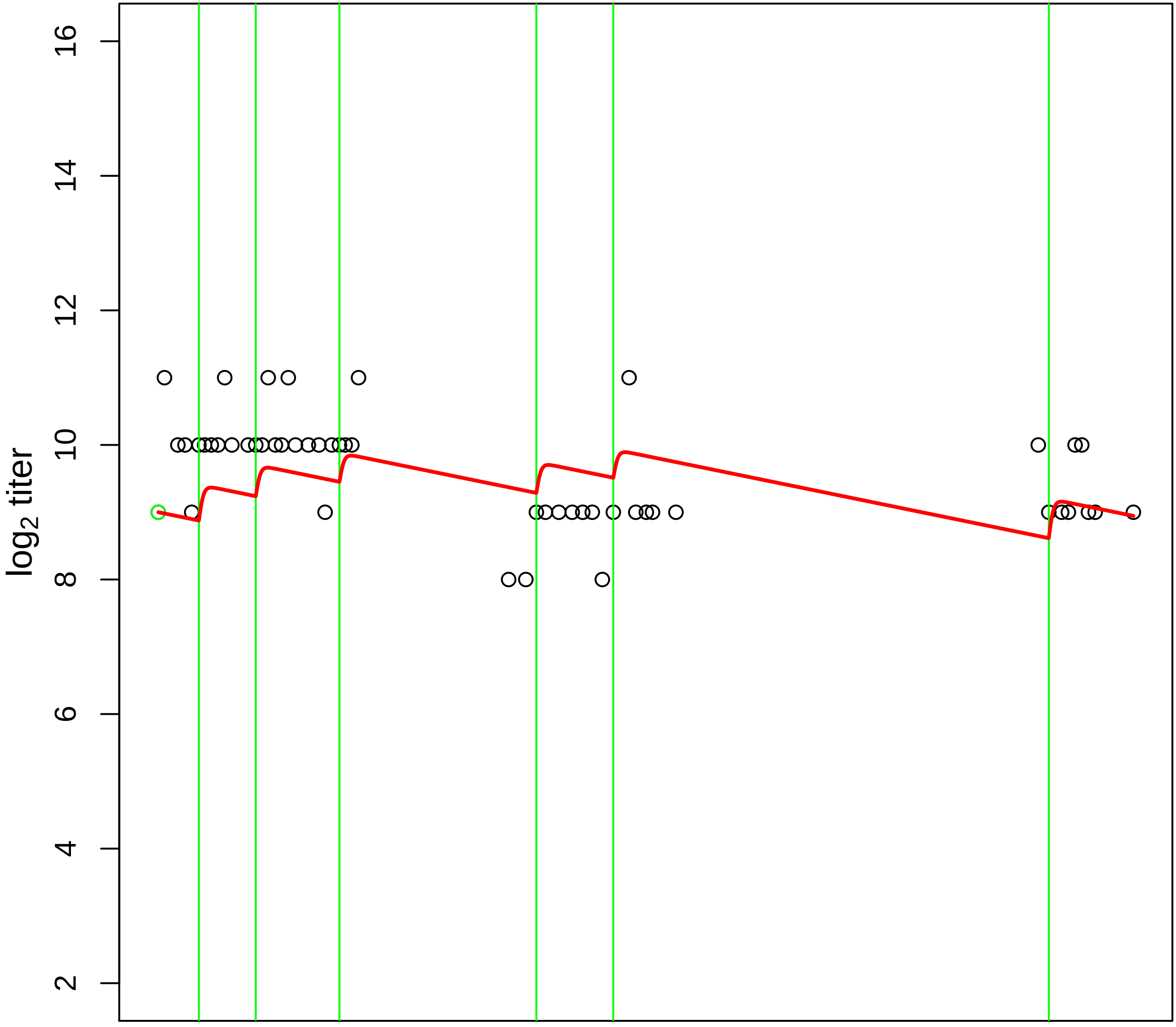

time in years from first donation of donor 543
mean absolute errors = 0.723 , mean squared errors = 0.726

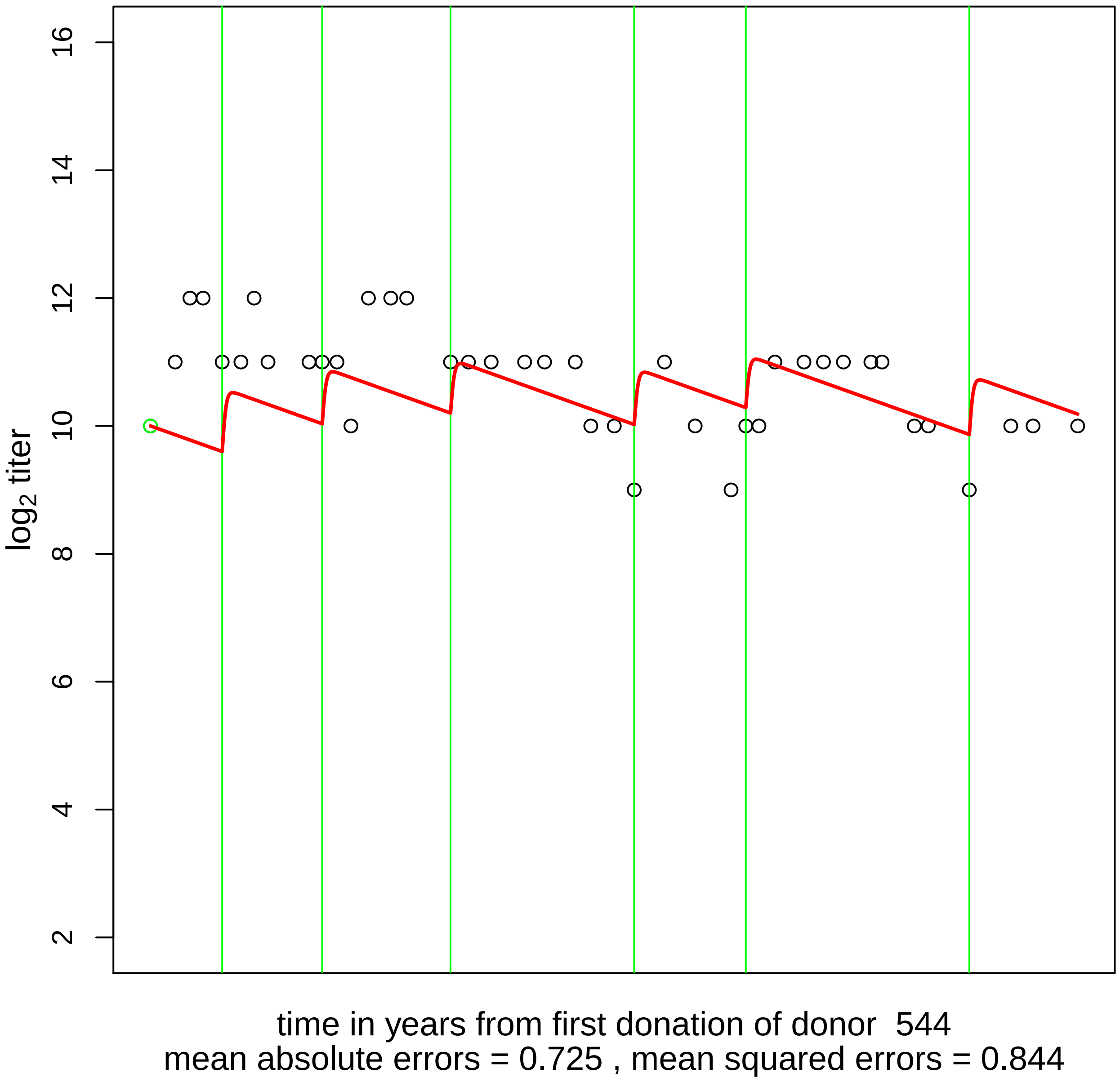

2
4
6
8
10
12
14
16
$\log_2$ titer
time in years from first donation of donor 544
mean absolute errors = 0.725 , mean squared errors = 0.844

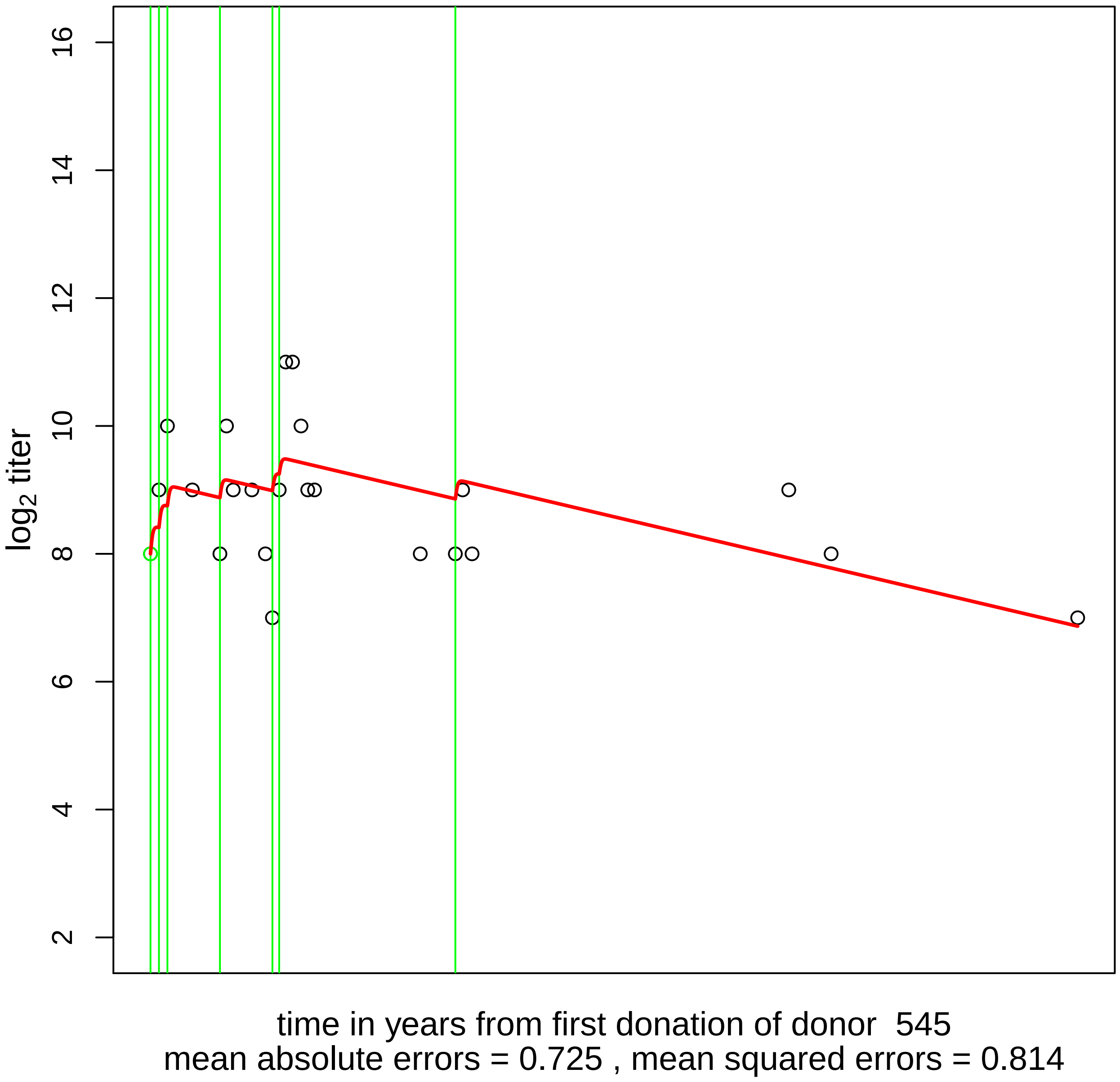

$\log_2$ titer
16
14
12
10
8
6
4
2
time in years from first donation of donor 545
mean absolute errors = 0.725 , mean squared errors = 0.814

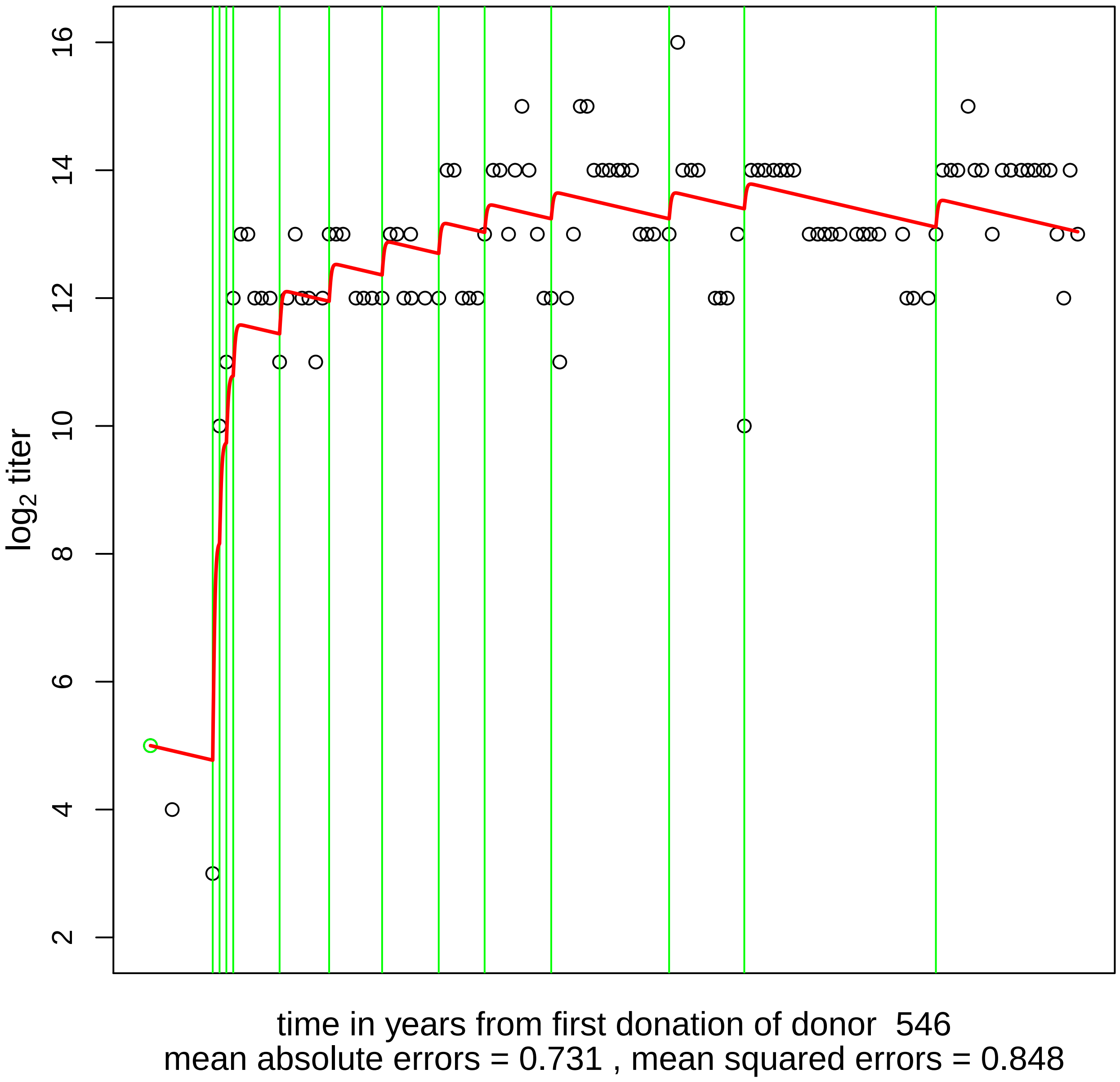

16
14
12
10
8
6
4
2
$\log_2$ titer
time in years from first donation of donor 546
mean absolute errors = 0.731 , mean squared errors = 0.848

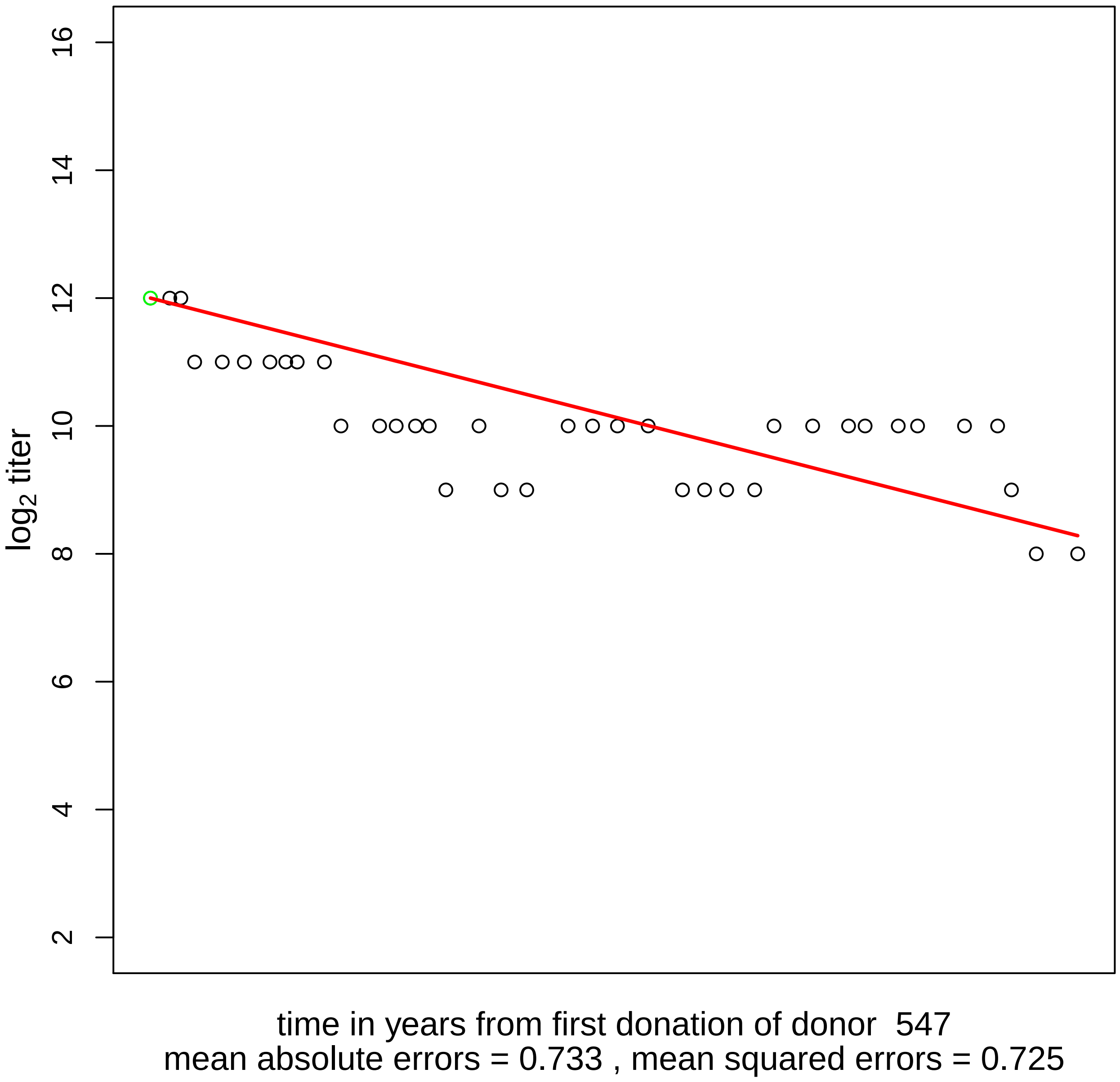
log2 titer
16
14
12
10
8
6
4
2
time in years from first donation of donor 547
mean absolute errors = 0.733 , mean squared errors = 0.725

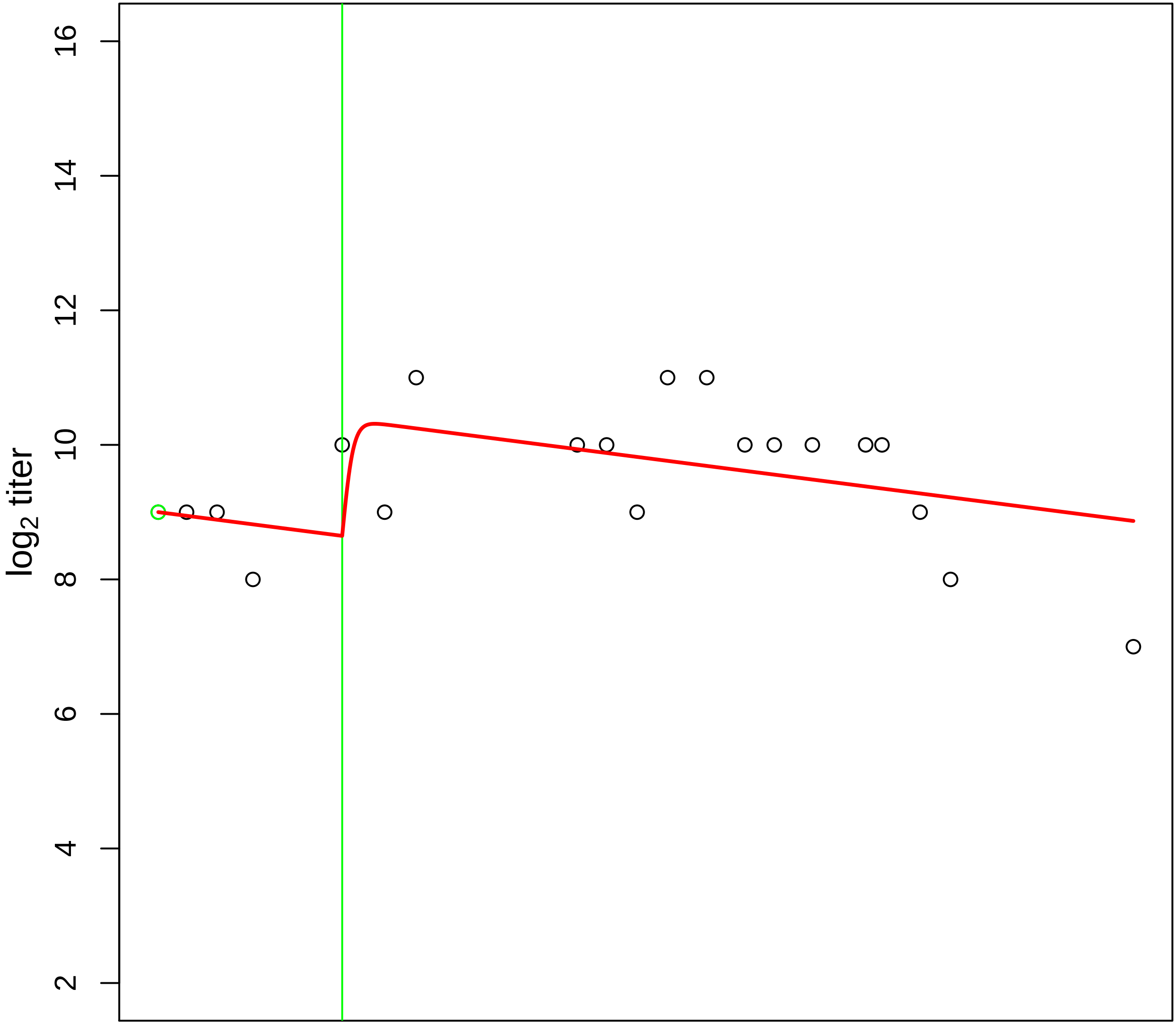

time in years from first donation of donor 548
mean absolute errors = 0.733 , mean squared errors = 0.8

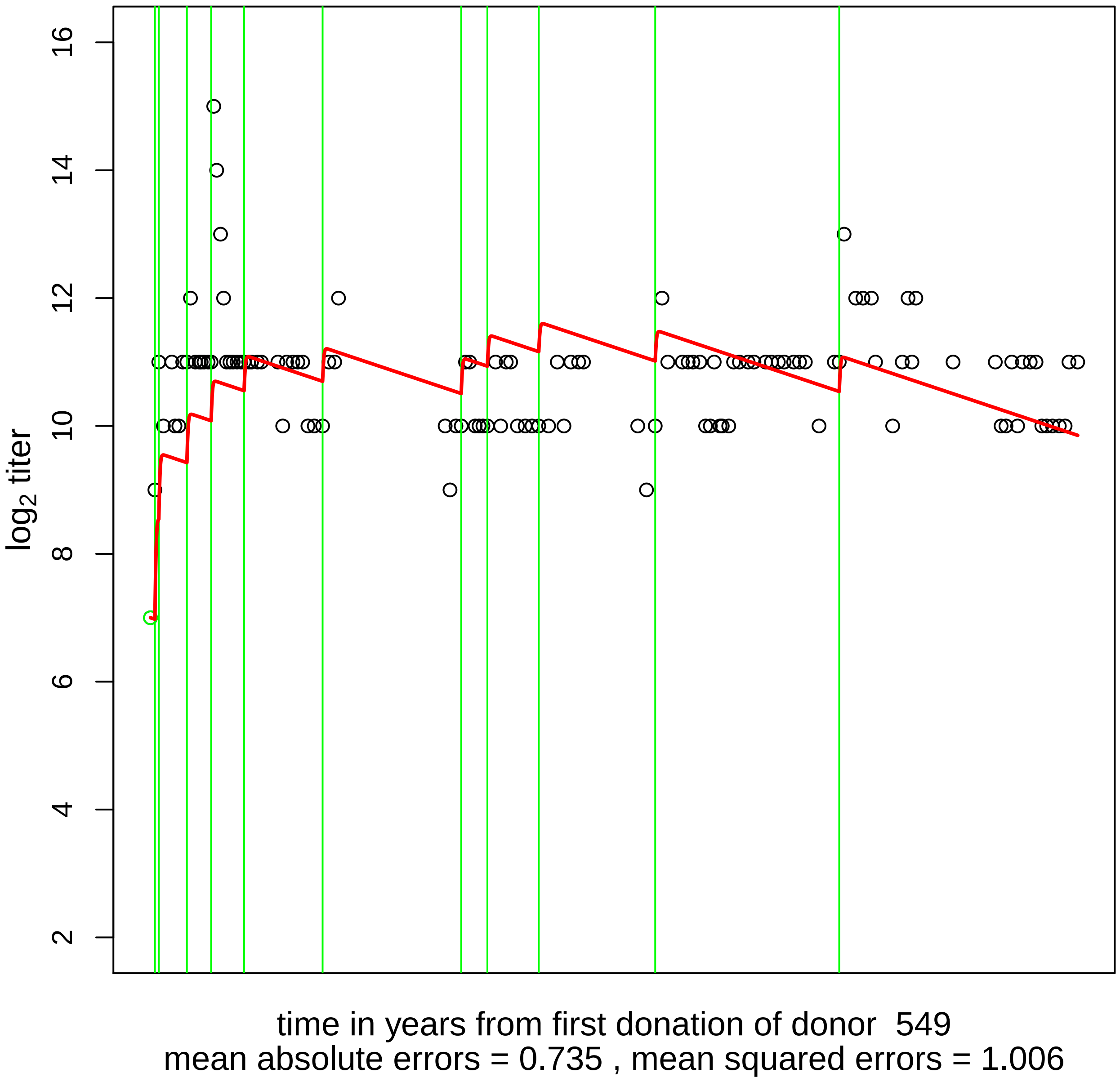

16
14
12
10
8
6
4
2
$\log_2$ titer
time in years from first donation of donor 549
mean absolute errors = 0.735 , mean squared errors = 1.006

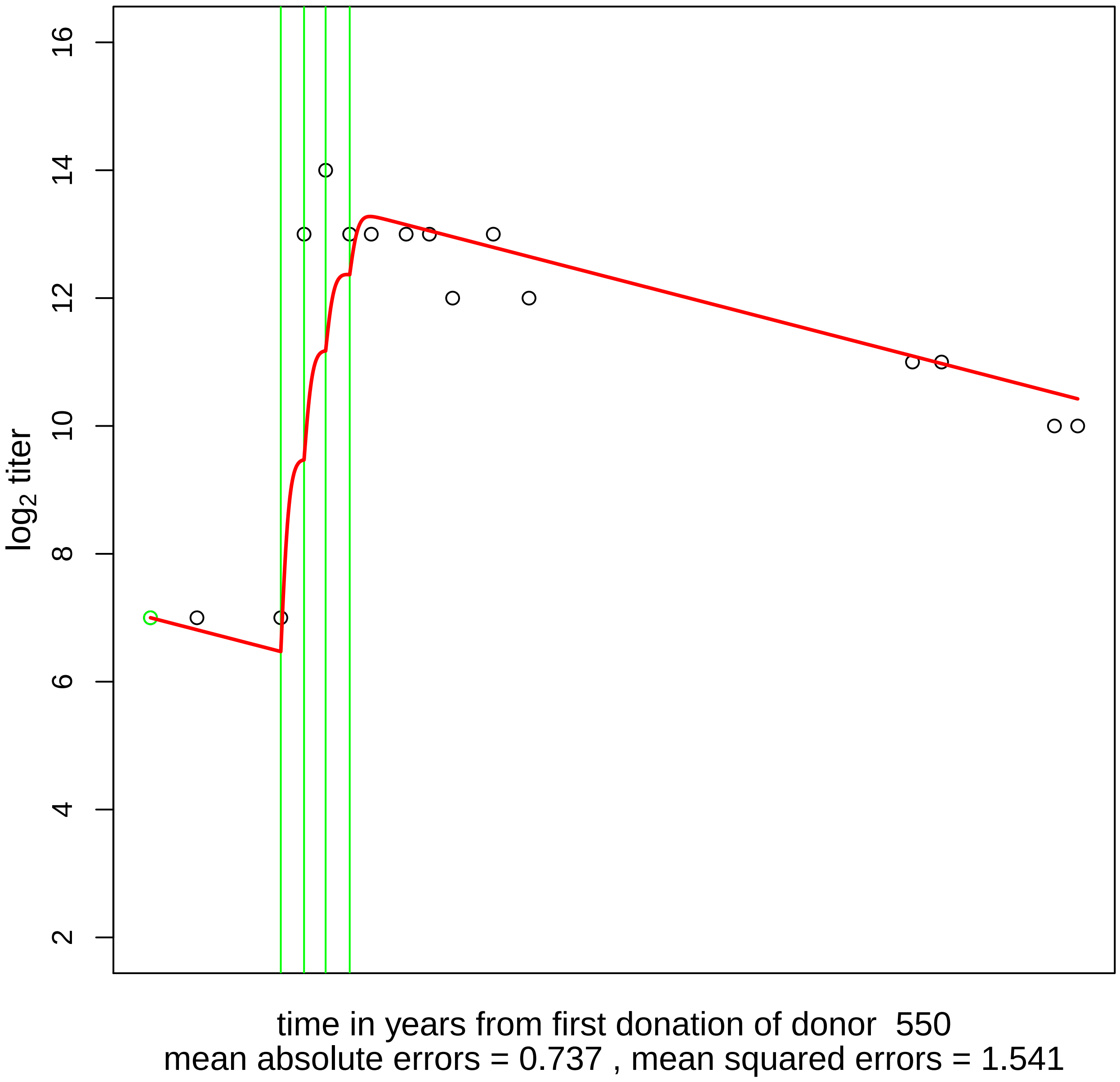

16
14
12
10
8
6
4
2
log2 titer
time in years from first donation of donor 550
mean absolute errors = 0.737 , mean squared errors = 1.541

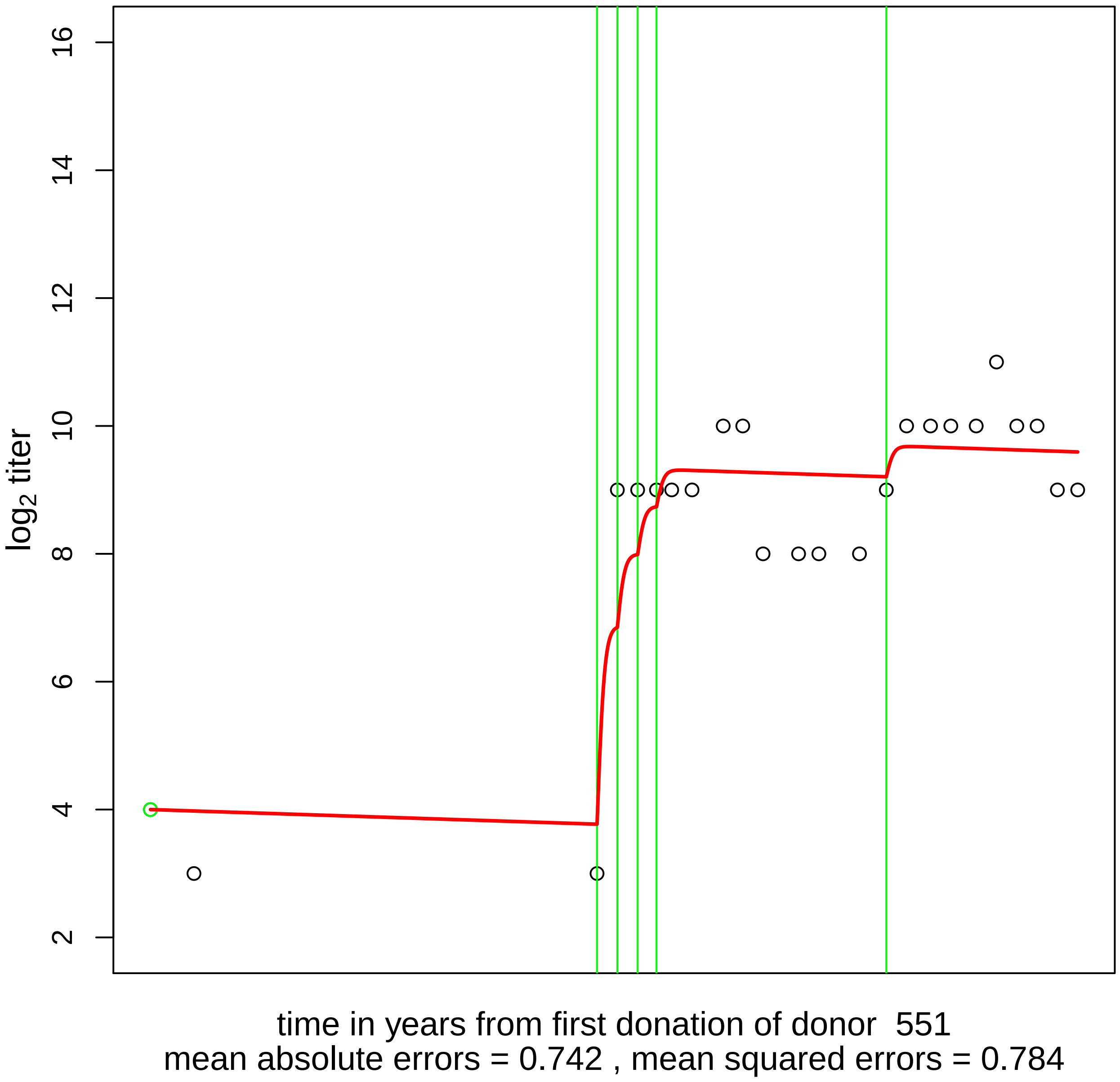

log2 titer
2
4
6
8
10
12
14
16
time in years from first donation of donor 551
mean absolute errors = 0.742 , mean squared errors = 0.784

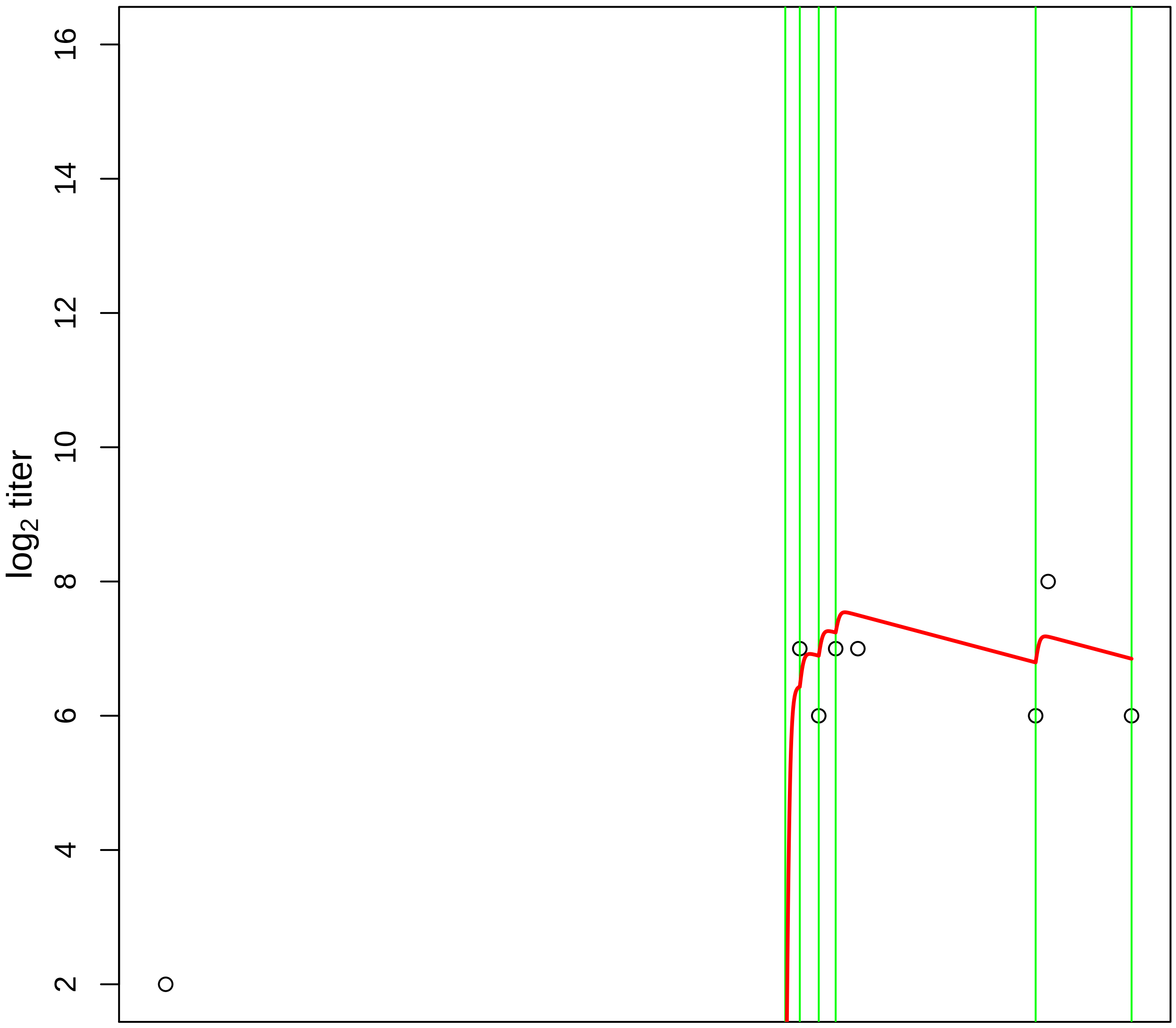

time in years from first donation of donor 552
mean absolute errors = 0.744 , mean squared errors = 0.613

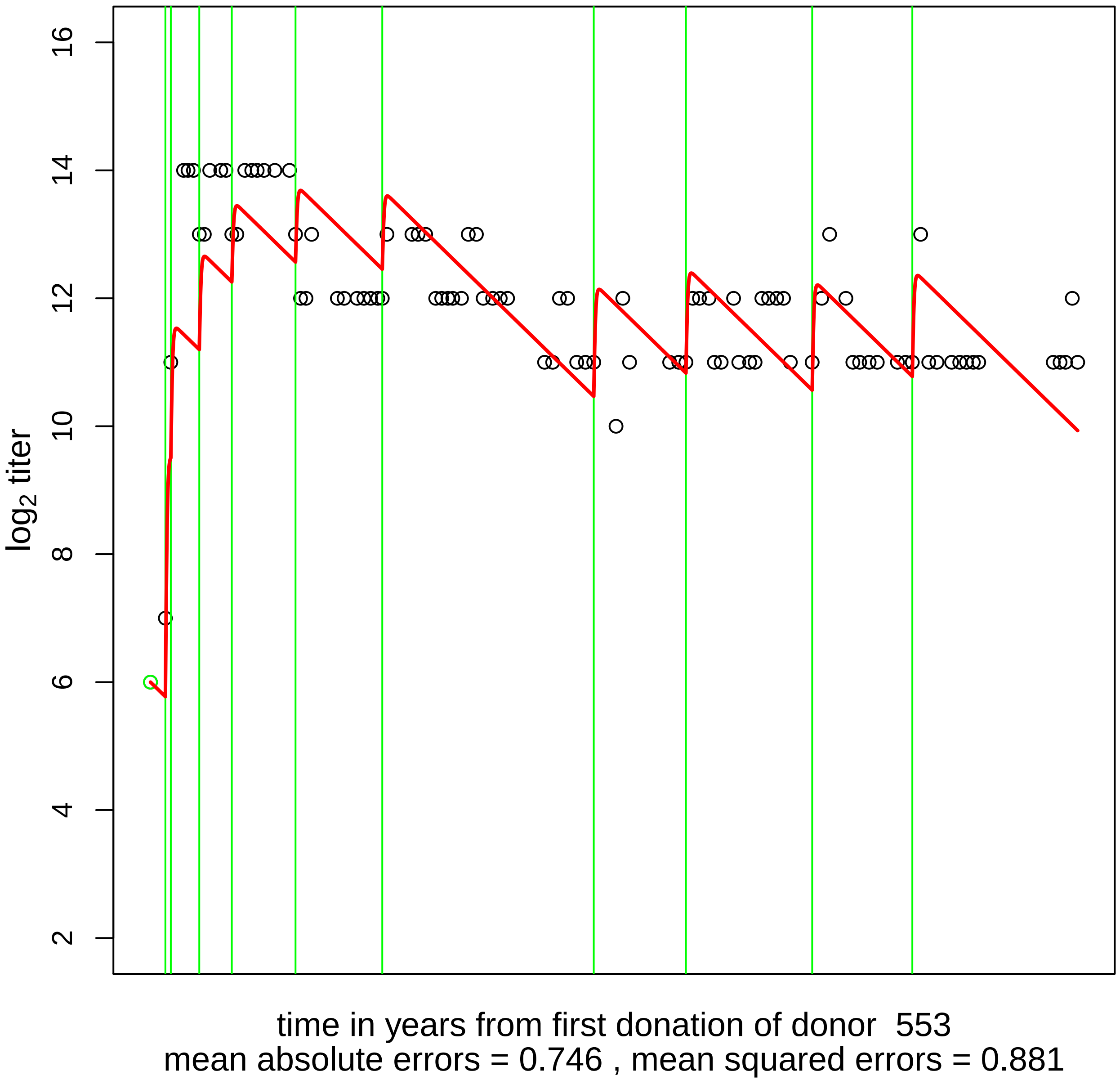

$\log_2$ titer
2
4
6
8
10
12
14
16
time in years from first donation of donor 553
mean absolute errors = 0.746 , mean squared errors = 0.881

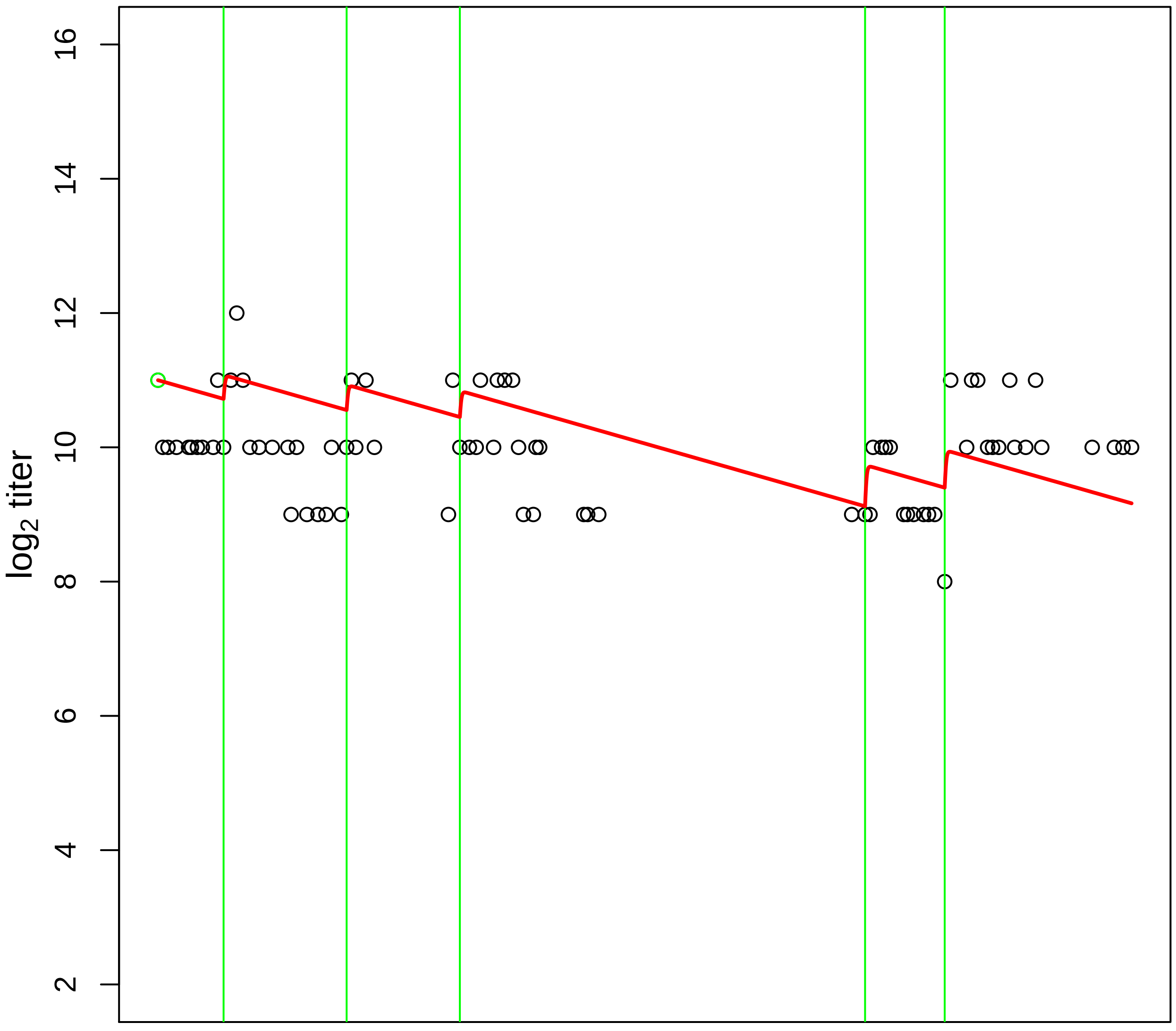

time in years from first donation of donor 554
mean absolute errors = 0.748 , mean squared errors = 0.761

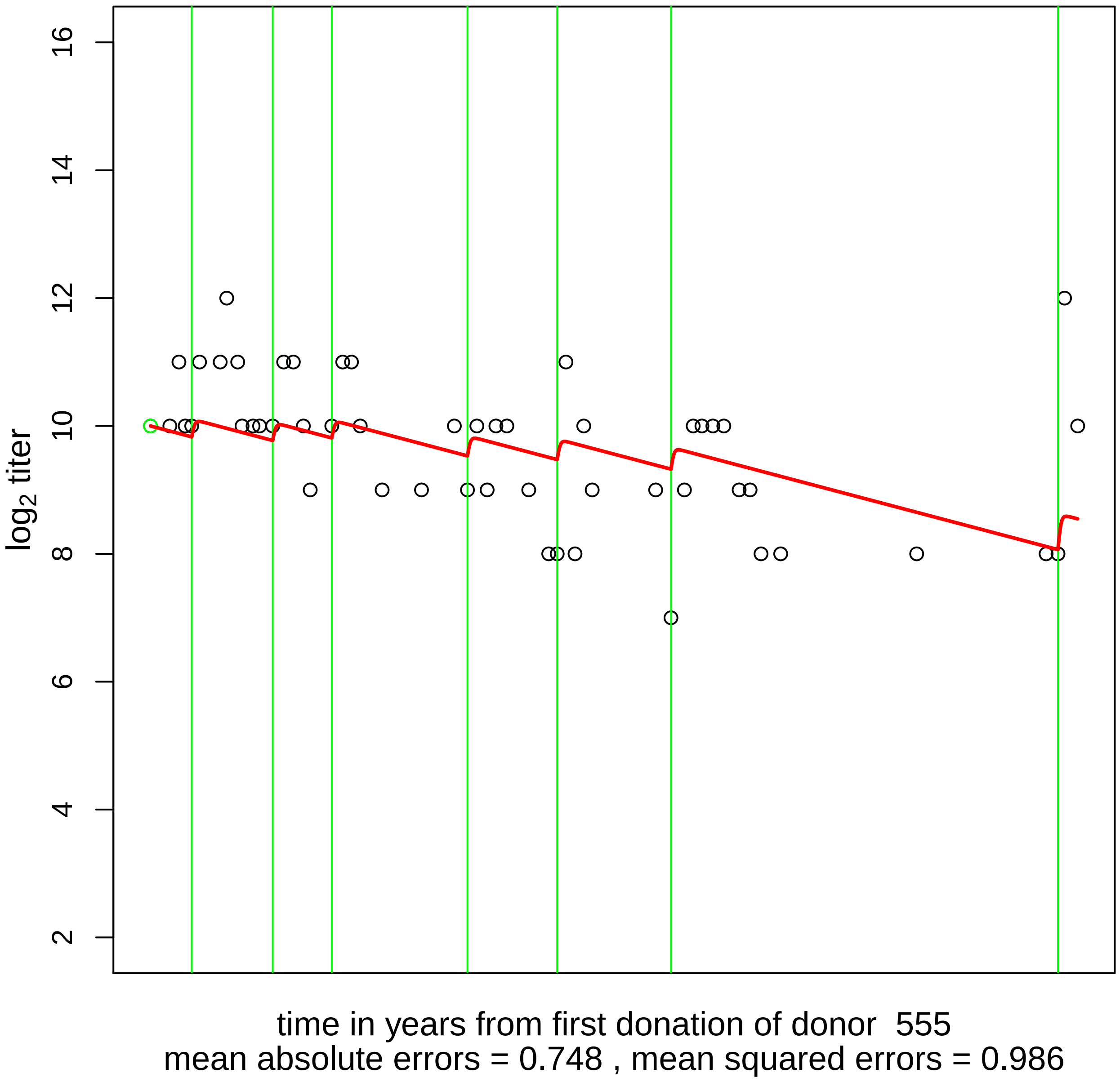

16
14
12
10
8
6
4
2
log2 titer
time in years from first donation of donor 555
mean absolute errors = 0.748 , mean squared errors = 0.986

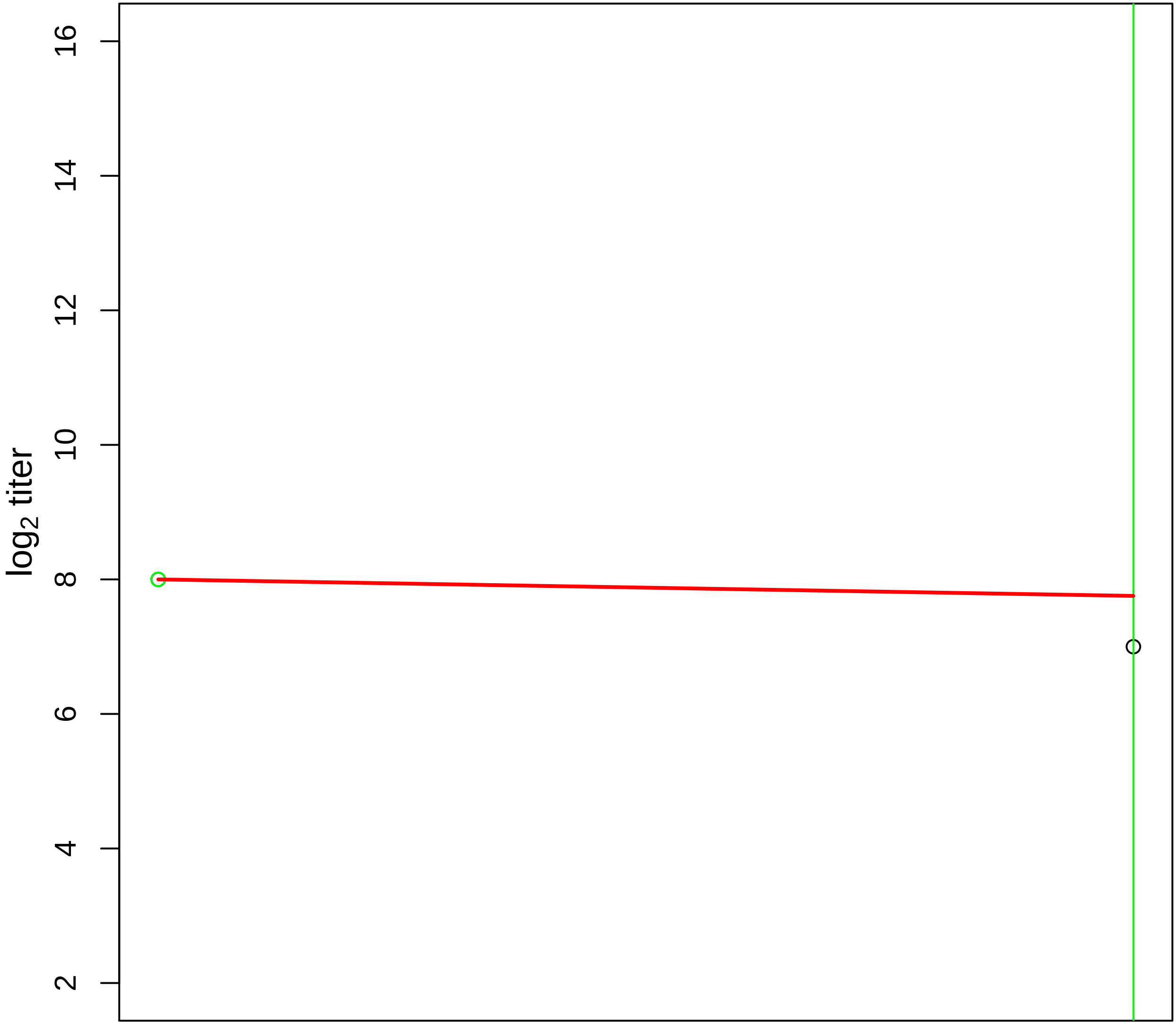

time in years from first donation of donor 556
mean absolute errors = 0.755 , mean squared errors = 0.57

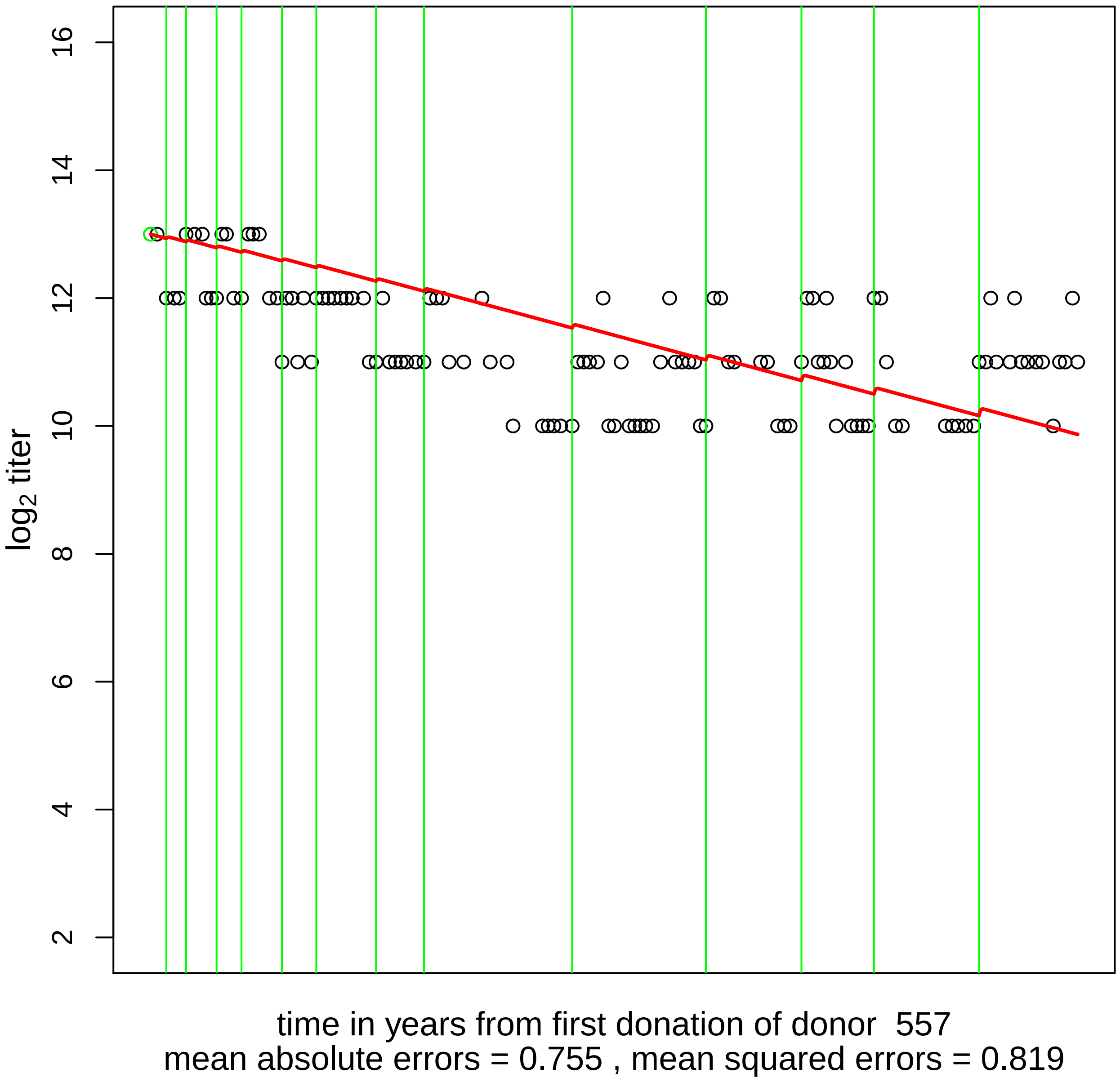

16
14
12
10
8
6
4
2
log2 titer
time in years from first donation of donor 557
mean absolute errors = 0.755 , mean squared errors = 0.819

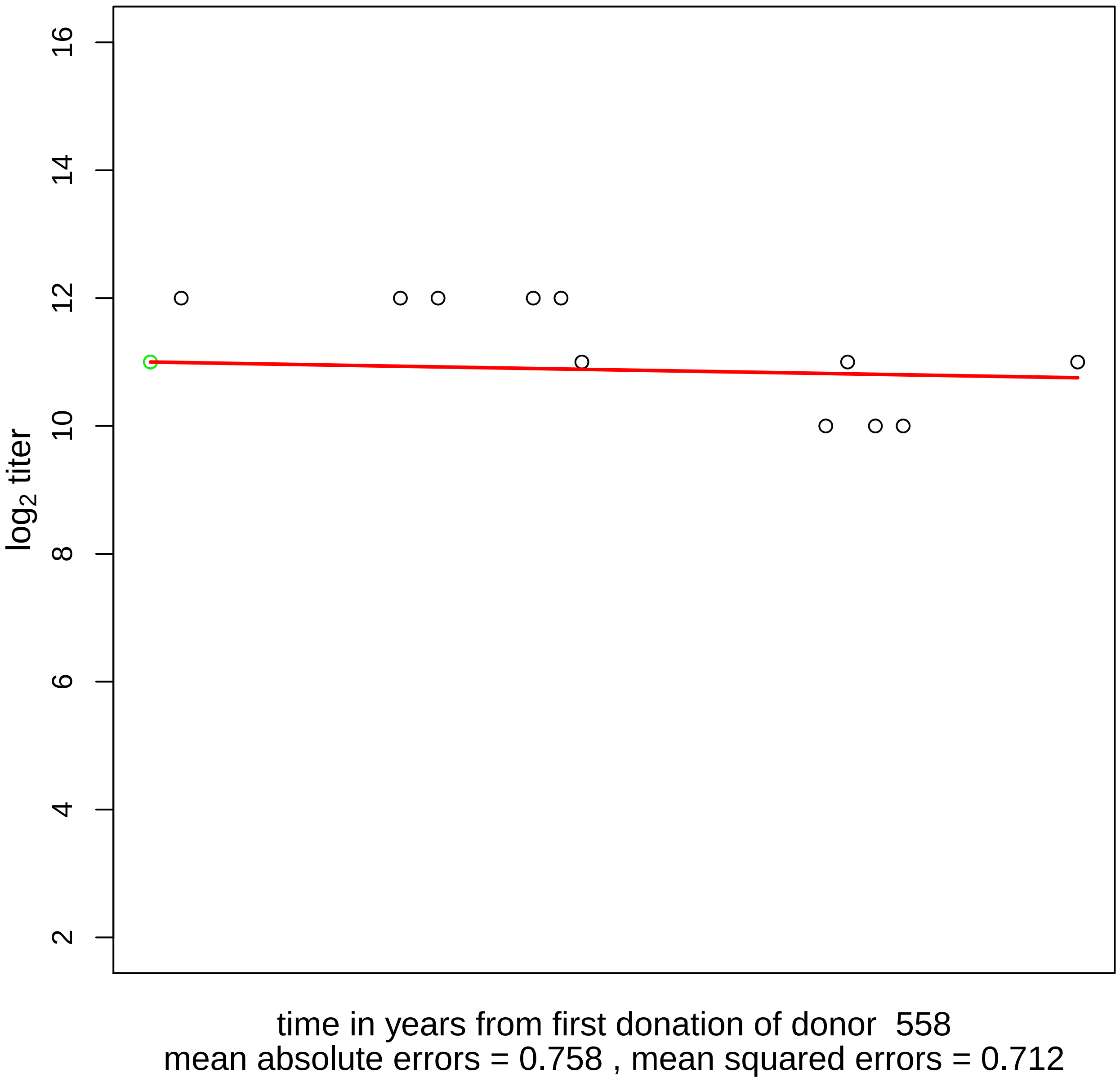

log2 titer
16
14
12
10
8
6
4
2
time in years from first donation of donor 558
mean absolute errors = 0.758 , mean squared errors = 0.712

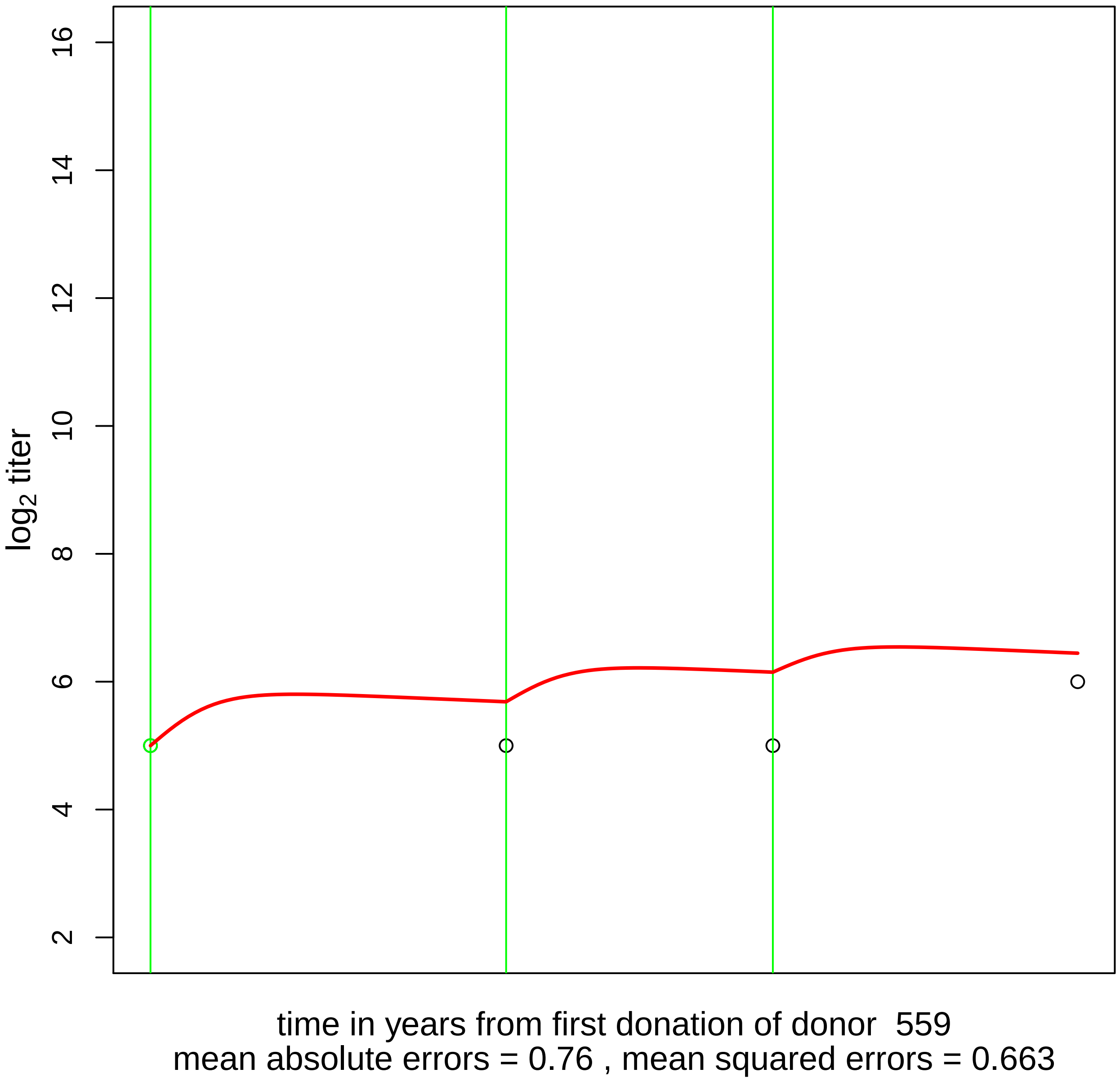
log2 titer
16
14
12
10
8
6
4
2
time in years from first donation of donor 559
mean absolute errors = 0.76 , mean squared errors = 0.663

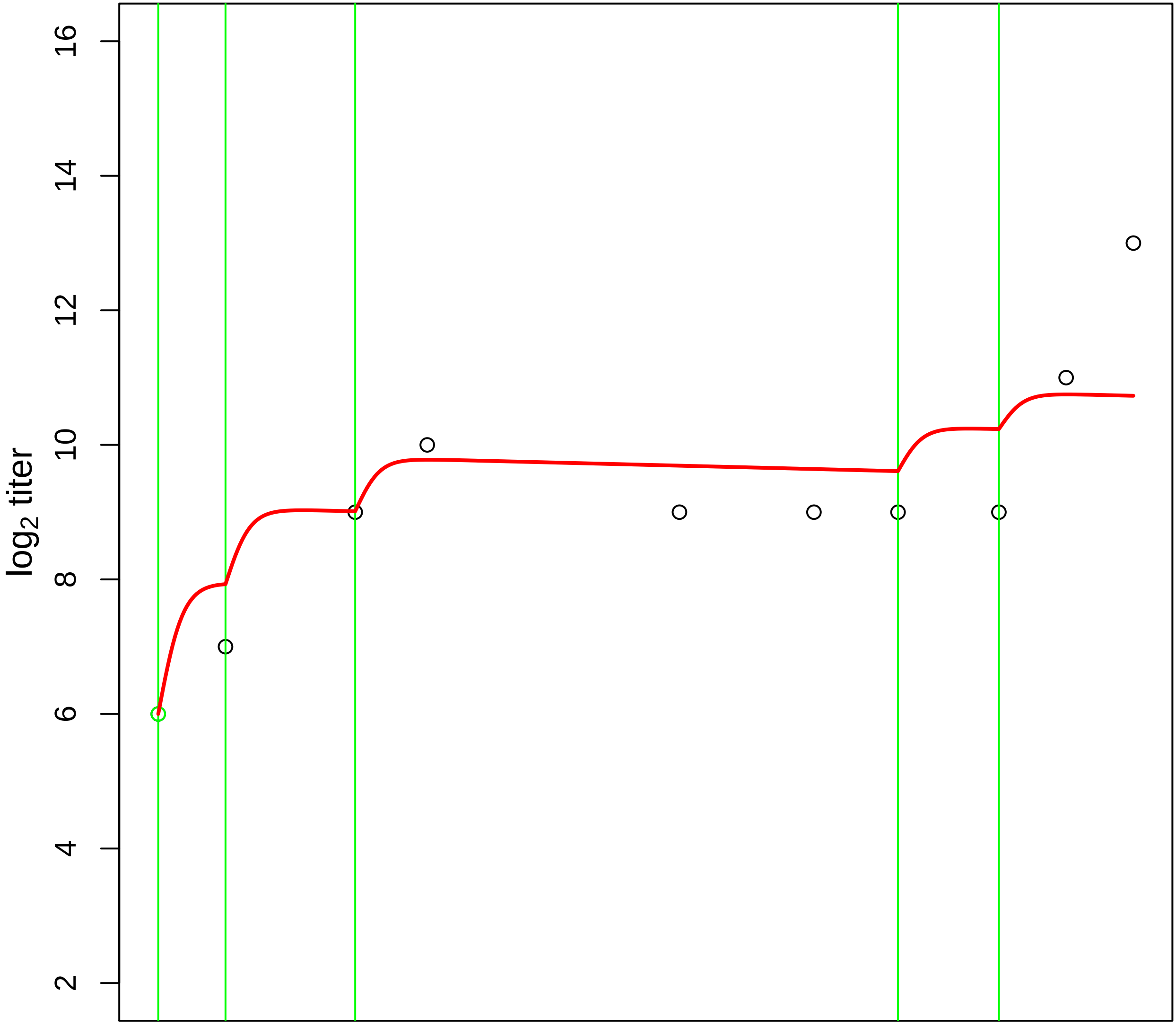

time in years from first donation of donor 560
mean absolute errors = 0.762 , mean squared errors = 0.99

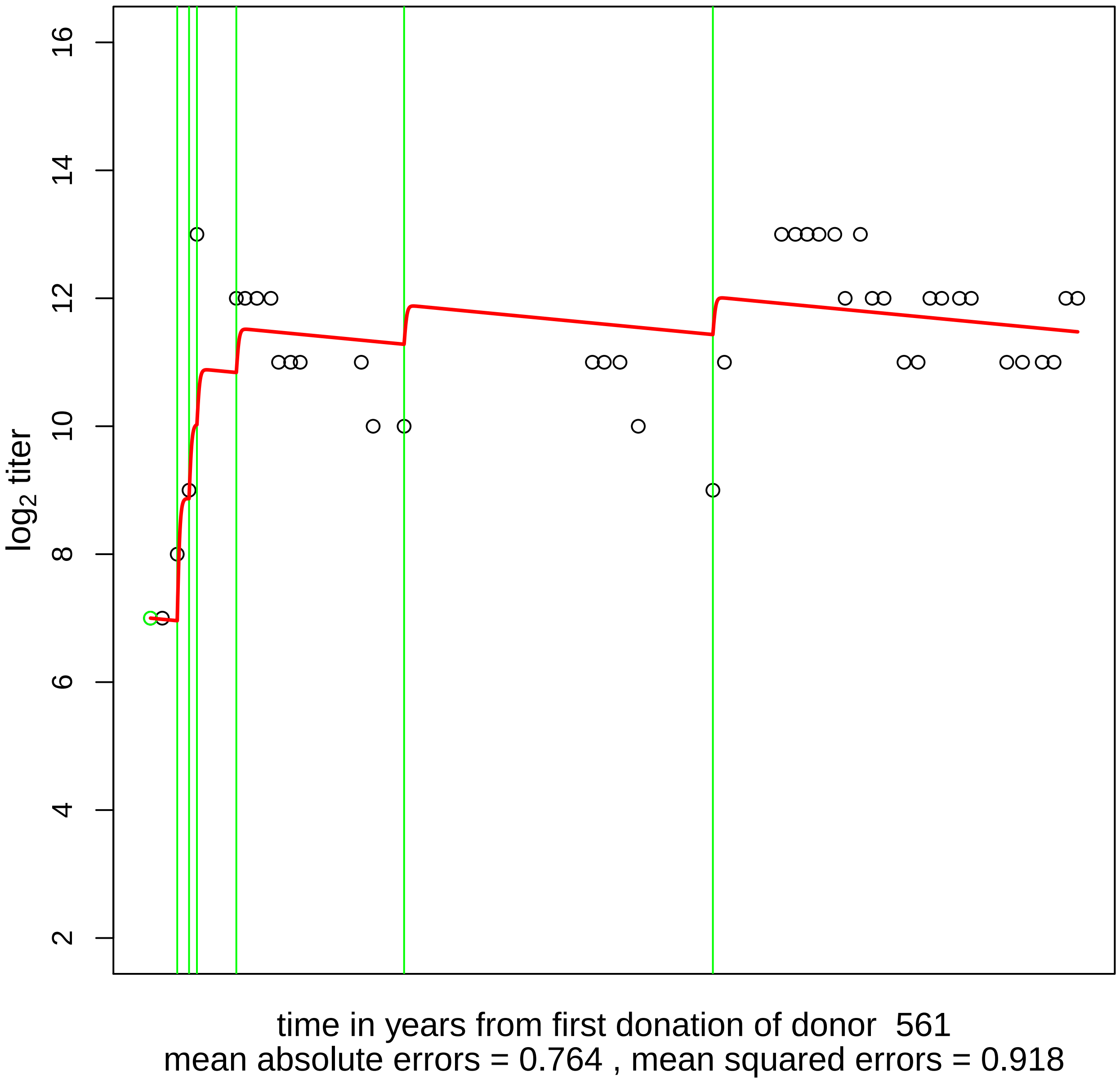

log2 titer
16
14
12
10
8
6
4
2
time in years from first donation of donor 561
mean absolute errors = 0.764 , mean squared errors = 0.918

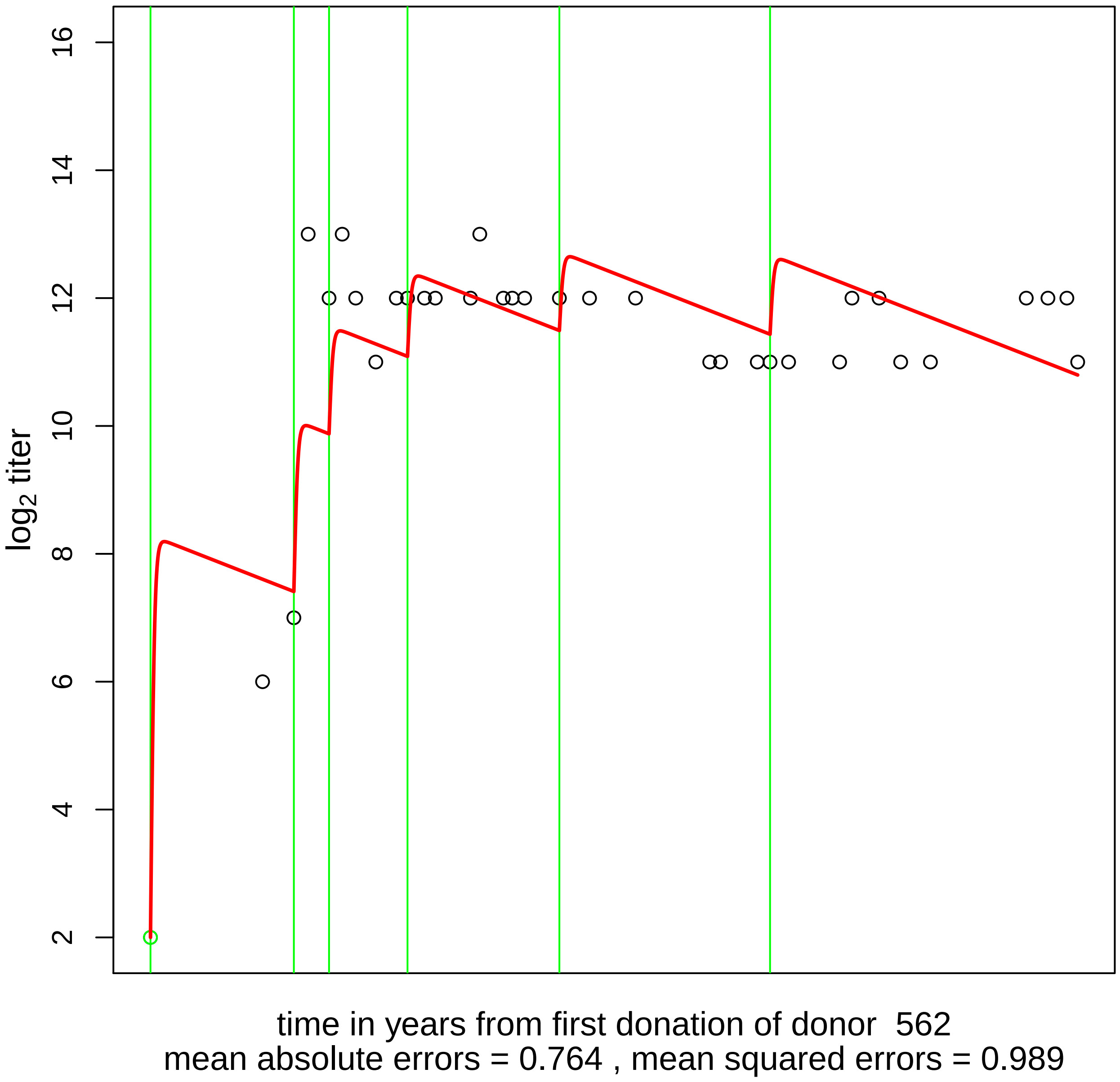

2
4
6
8
10
12
14
16
$\log_2$ titer
time in years from first donation of donor 562
mean absolute errors = 0.764 , mean squared errors = 0.989

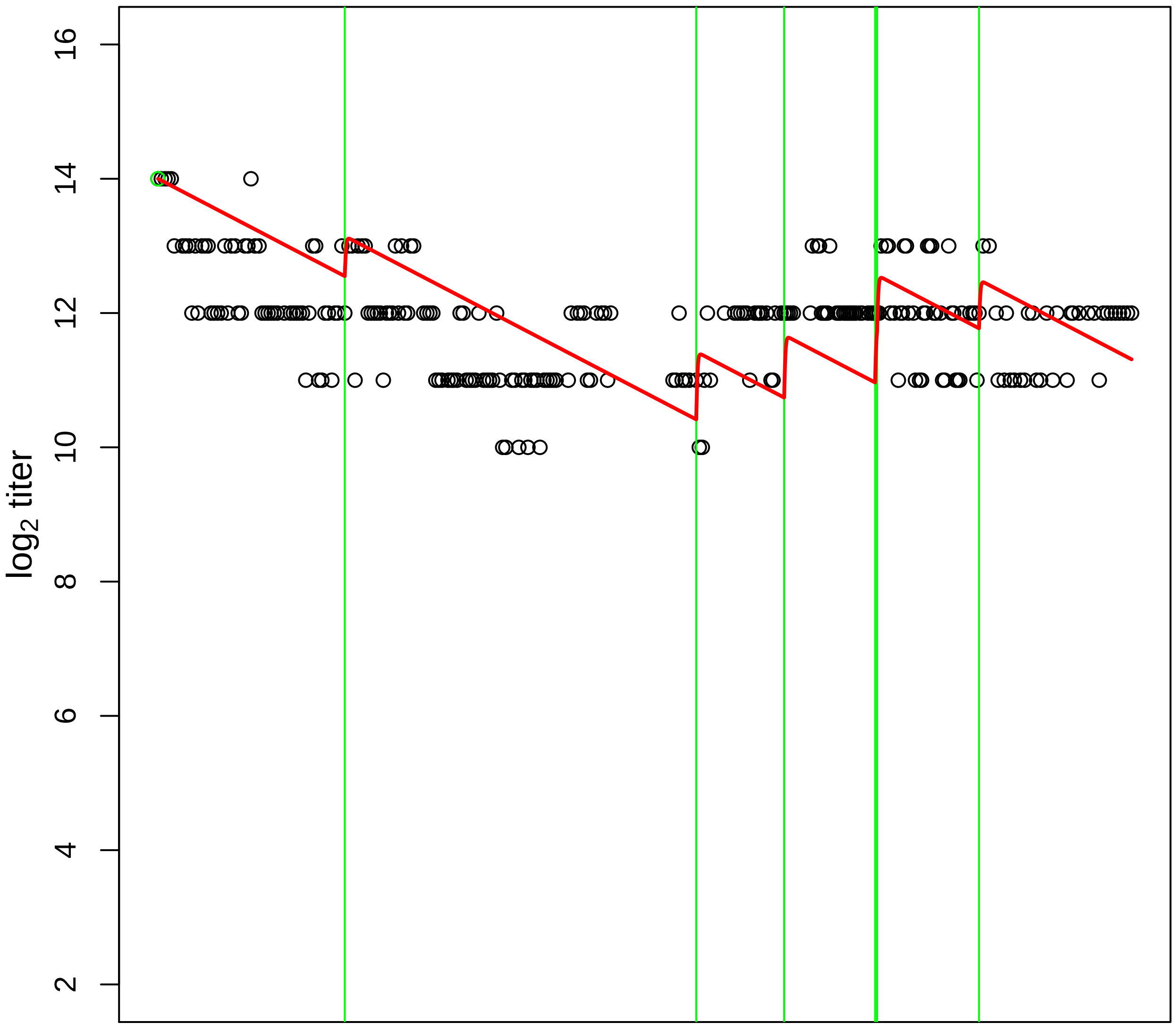

time in years from first donation of donor 563
mean absolute errors = 0.767 , mean squared errors = 0.799

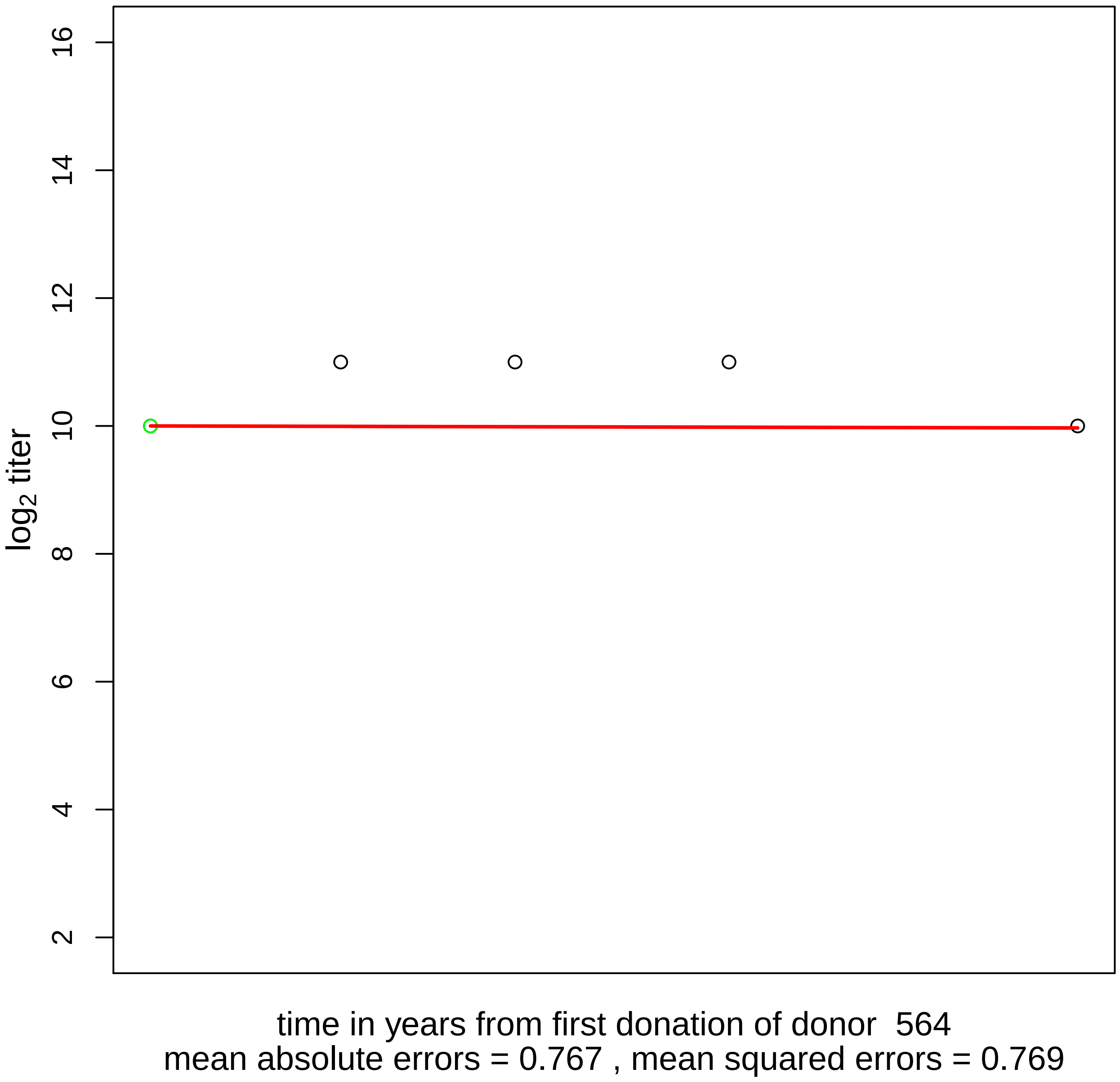

log2 titer
16
14
12
10
8
6
4
2
time in years from first donation of donor 564
mean absolute errors = 0.767 , mean squared errors = 0.769

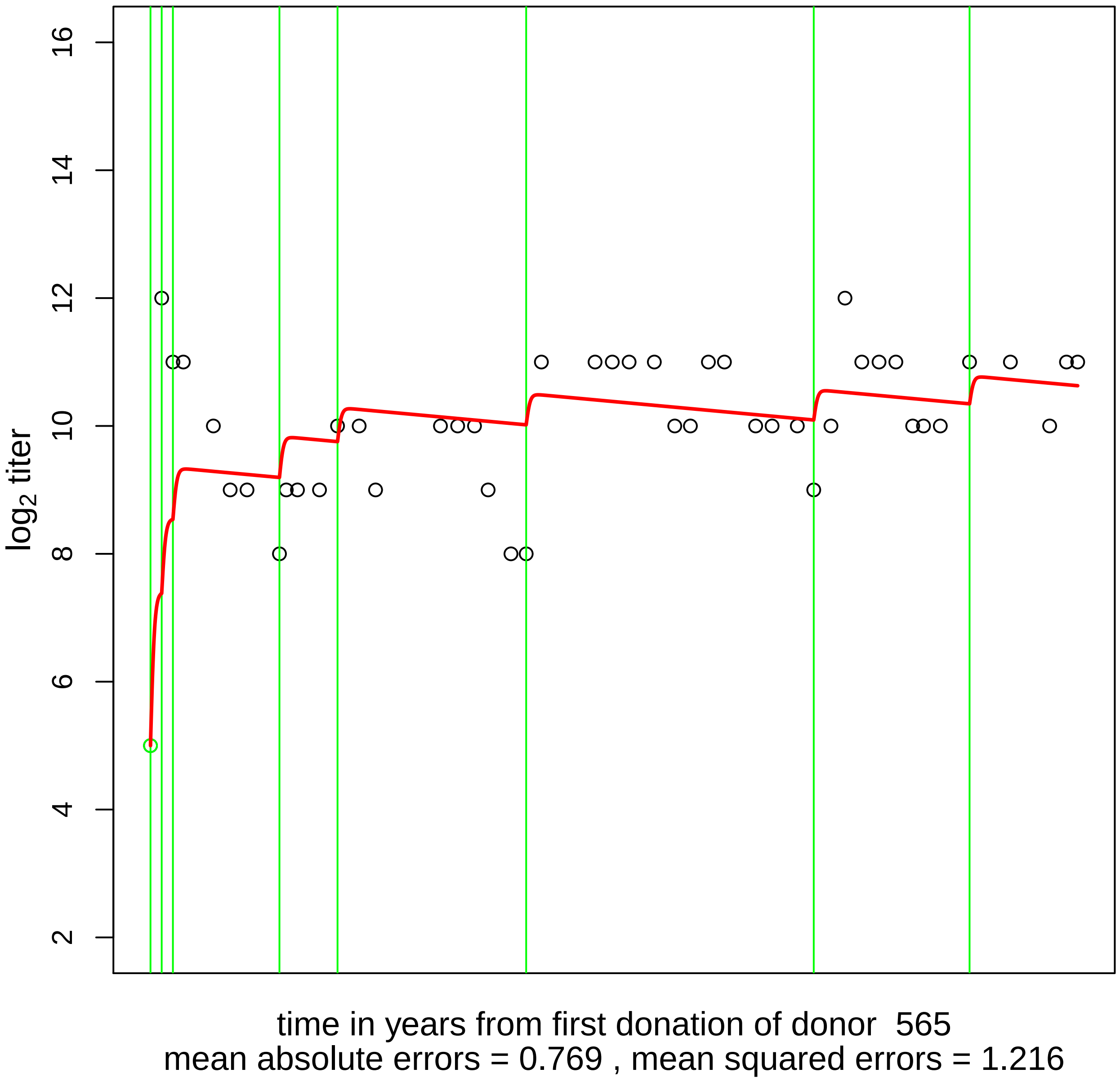

16
14
12
10
8
6
4
2
log2 titer
time in years from first donation of donor 565
mean absolute errors = 0.769 , mean squared errors = 1.216

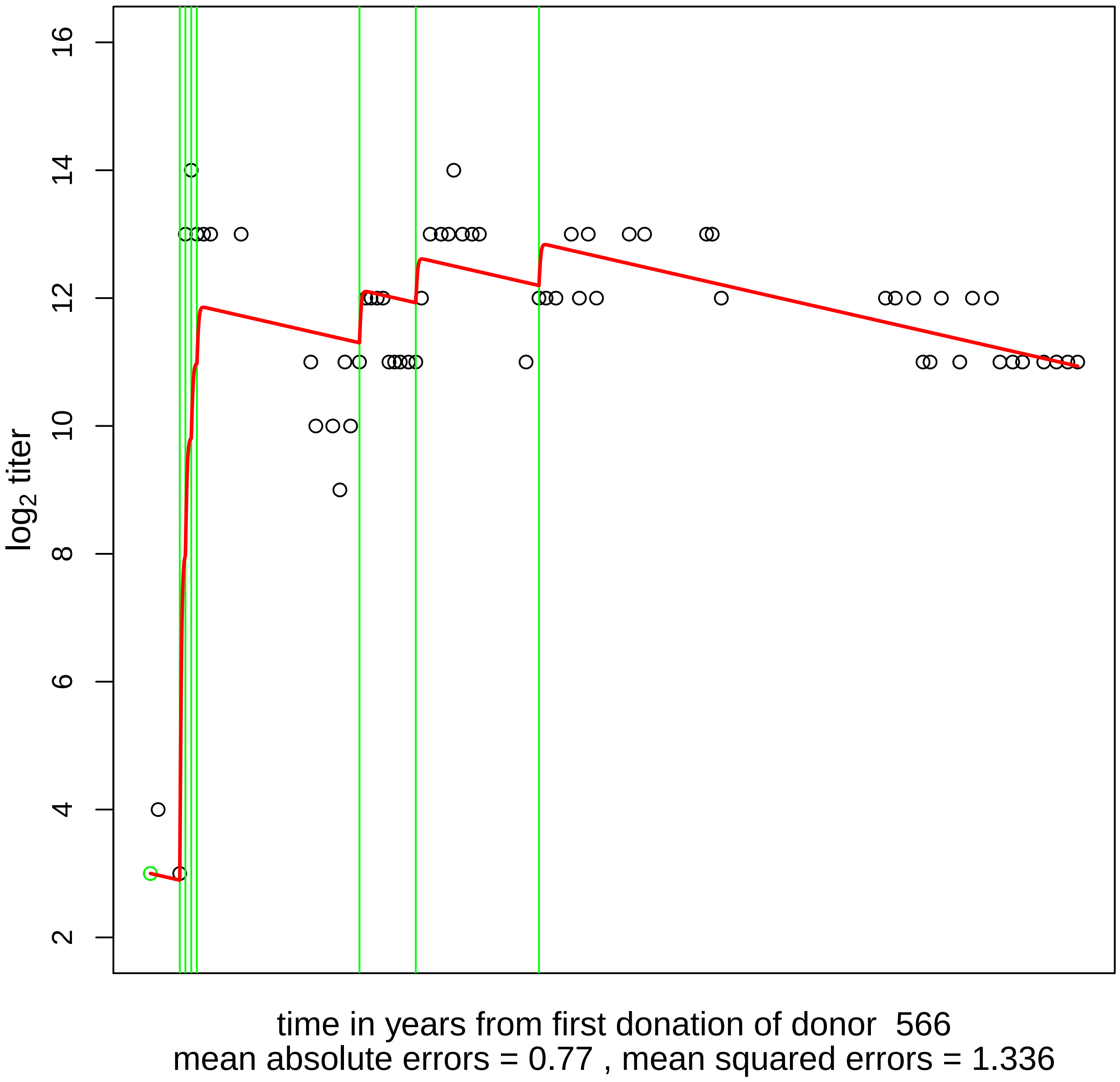

16
14
12
10
8
6
4
2
$\log_2$ titer
time in years from first donation of donor 566
mean absolute errors = 0.77 , mean squared errors = 1.336

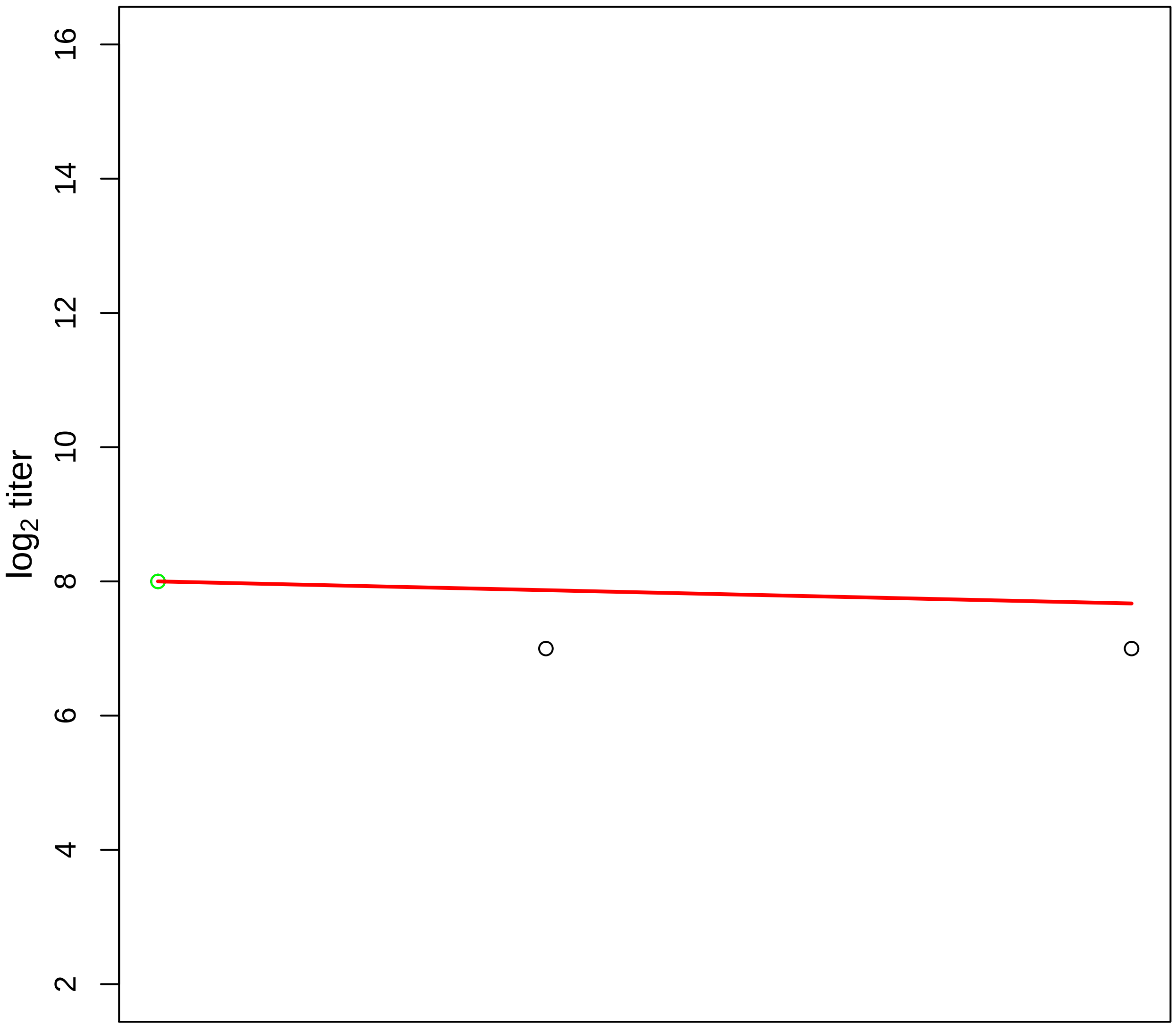

time in years from first donation of donor 567
mean absolute errors = 0.77 , mean squared errors = 0.603

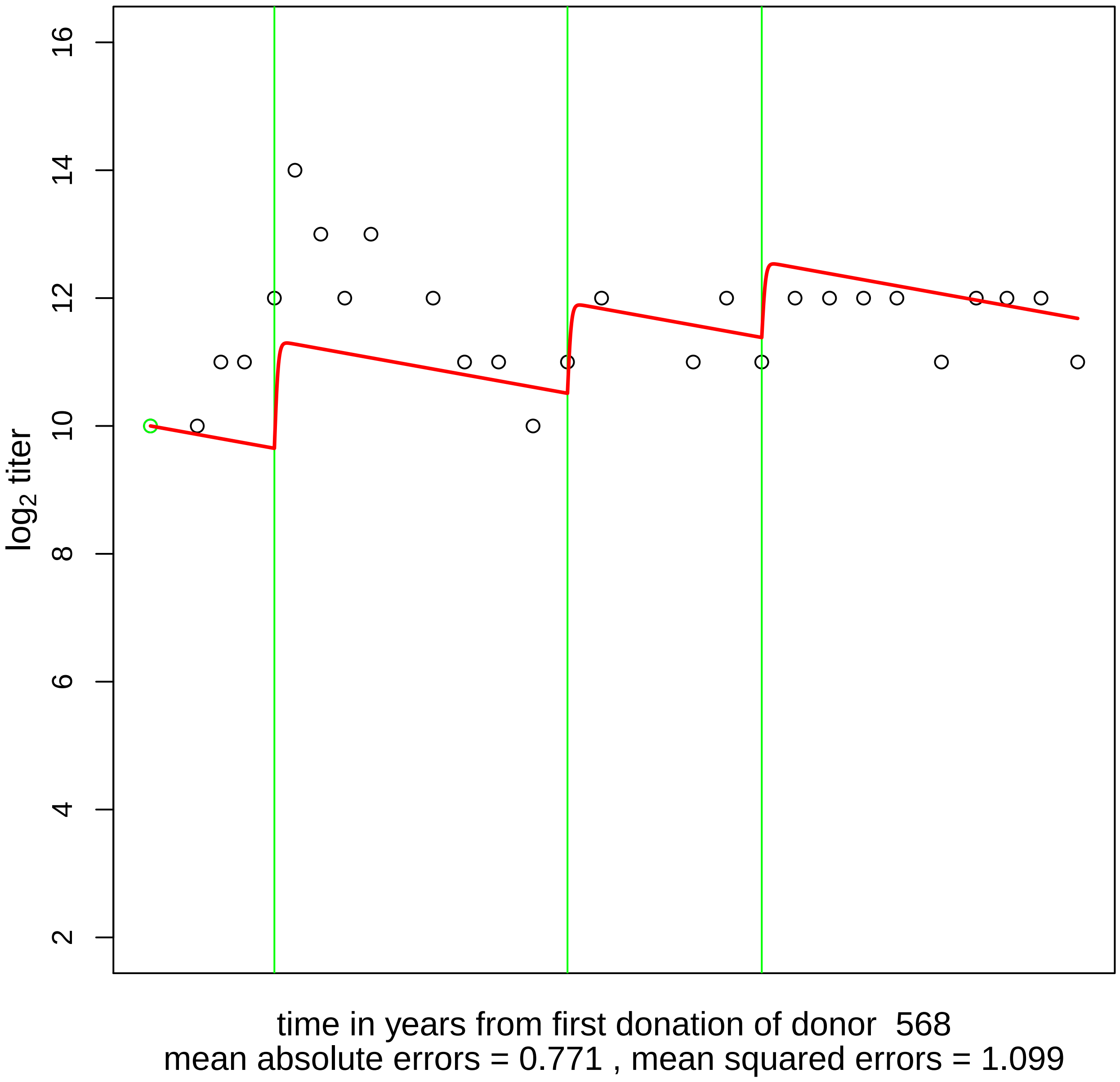

log2 titer
16
14
12
10
8
6
4
2
time in years from first donation of donor 568
mean absolute errors = 0.771 , mean squared errors = 1.099

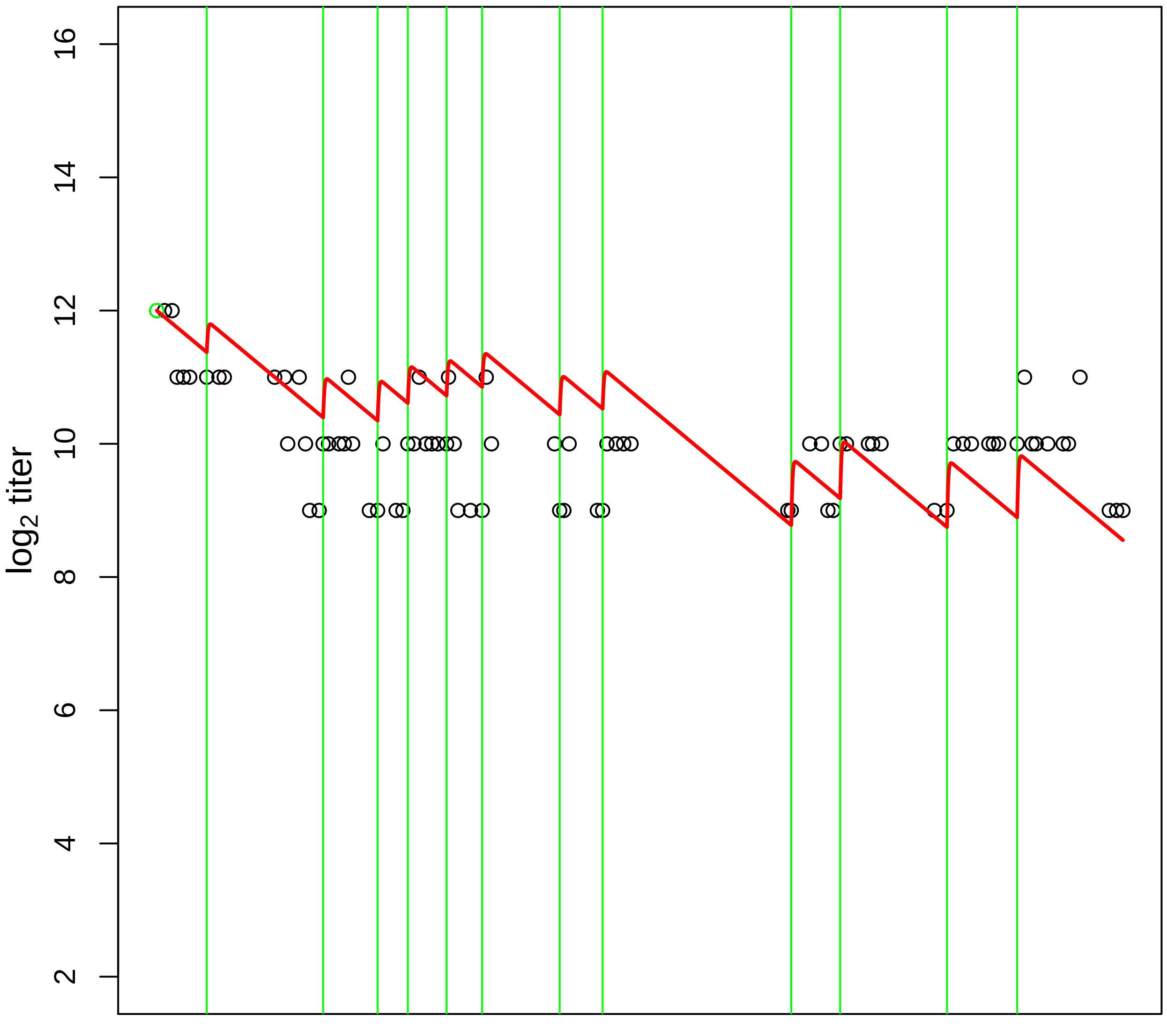

time in years from first donation of donor 569
mean absolute errors = 0.773 , mean squared errors = 0.875

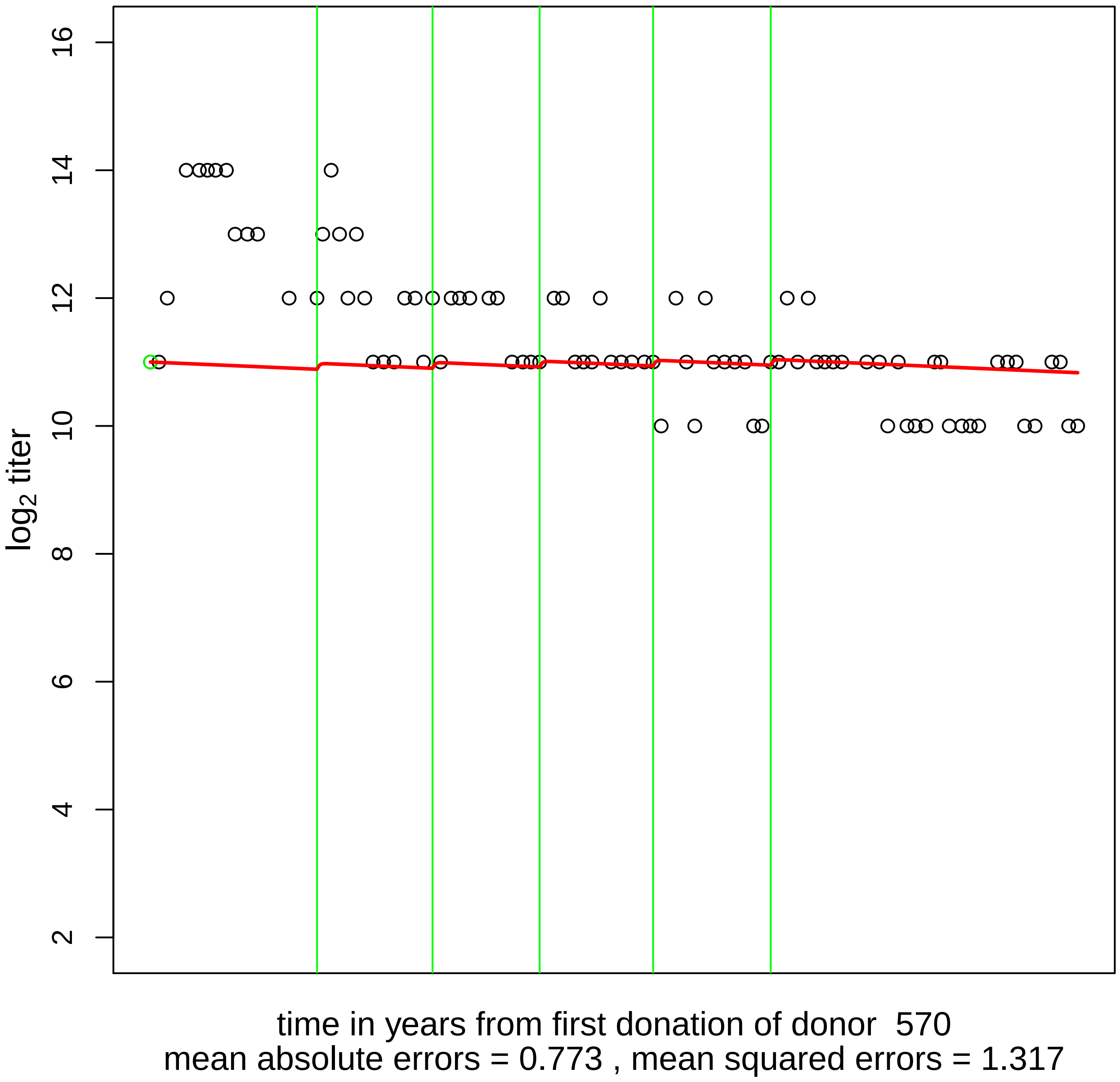

16
14
12
10
8
6
4
2
log2 titer
time in years from first donation of donor 570
mean absolute errors = 0.773 , mean squared errors = 1.317

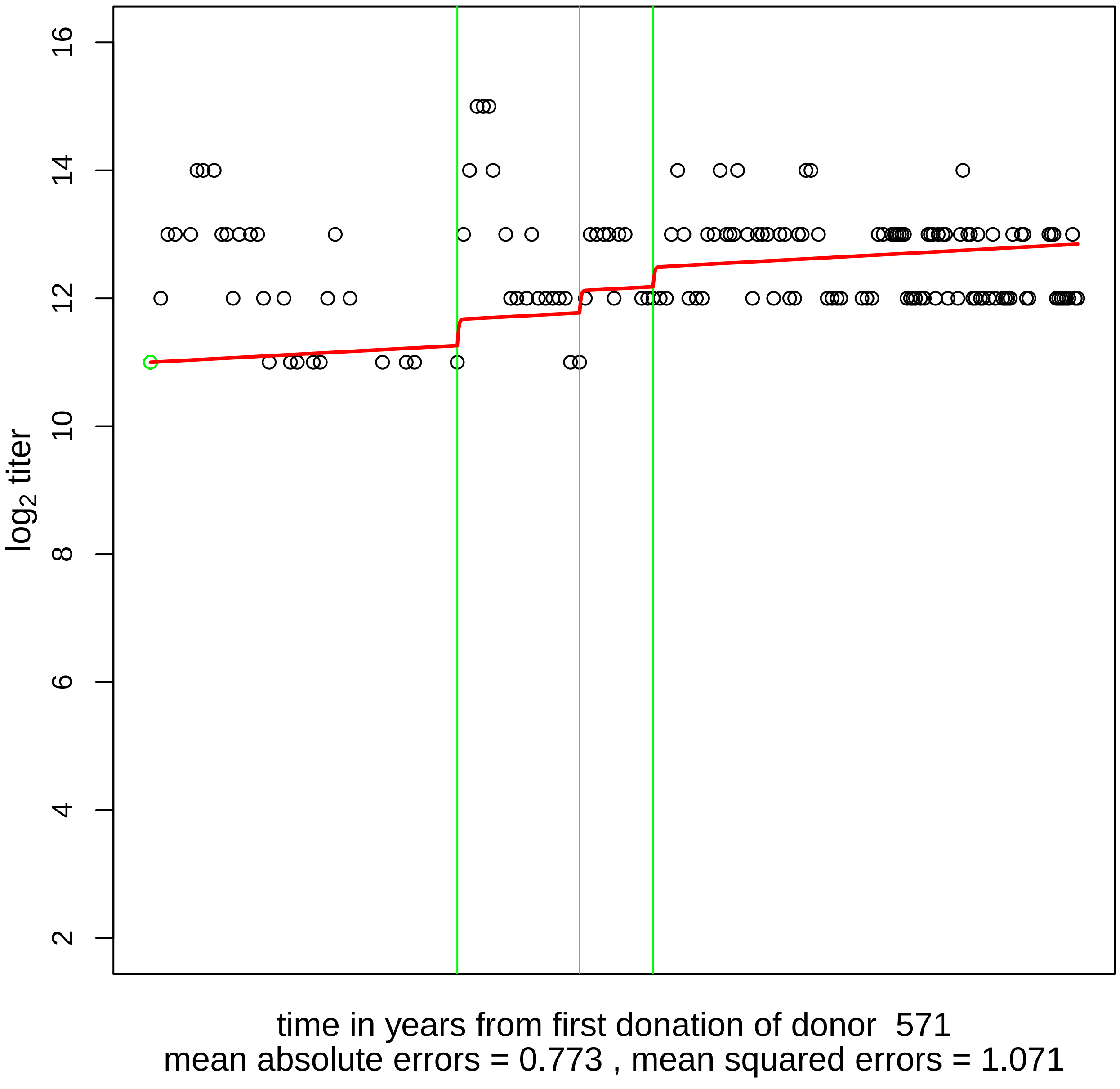

log2 titer
2
4
6
8
10
12
14
16
time in years from first donation of donor 571
mean absolute errors = 0.773 , mean squared errors = 1.071

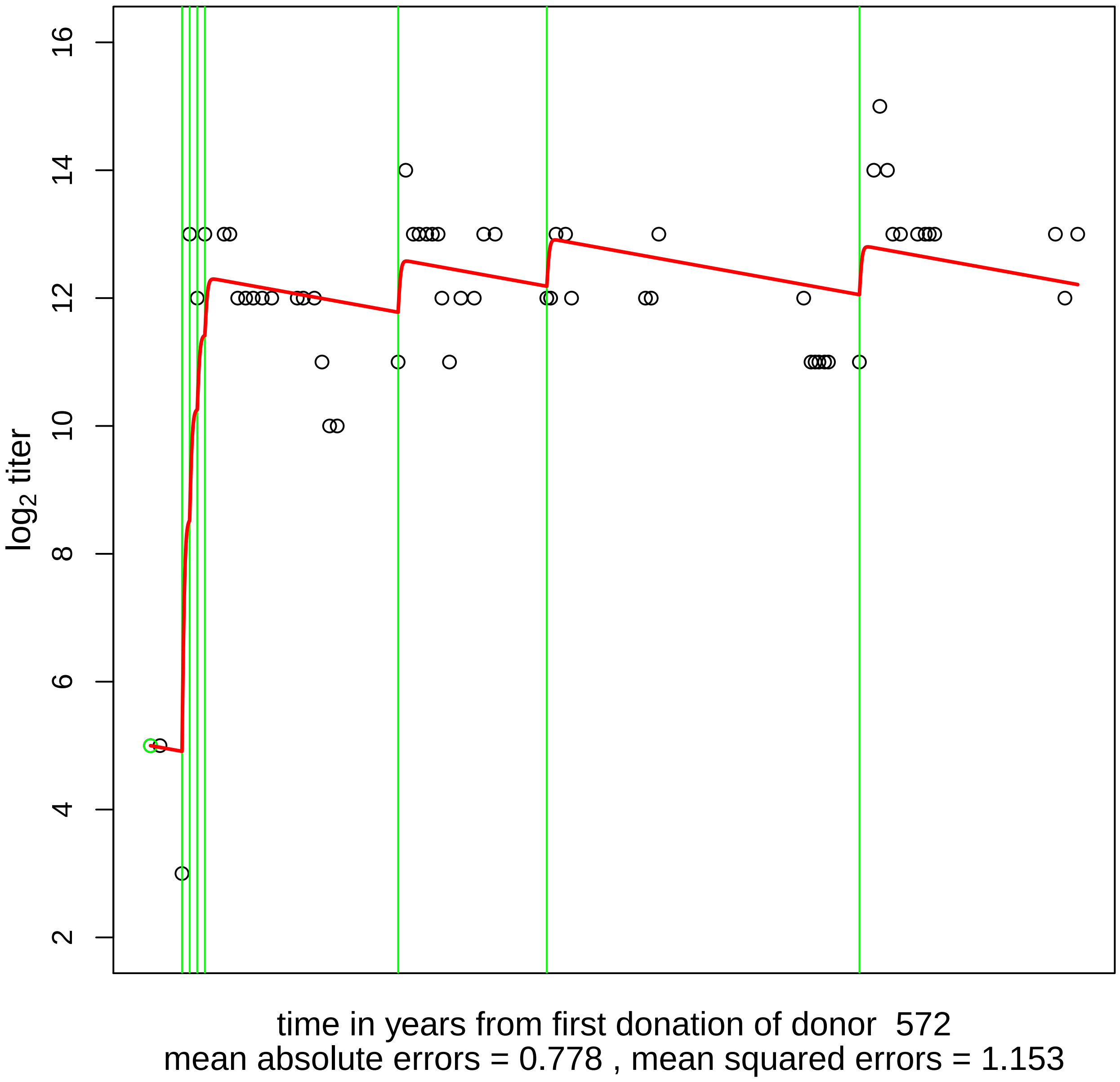

2
4
6
8
10
12
14
16
log2 titer
time in years from first donation of donor 572
mean absolute errors = 0.778 , mean squared errors = 1.153

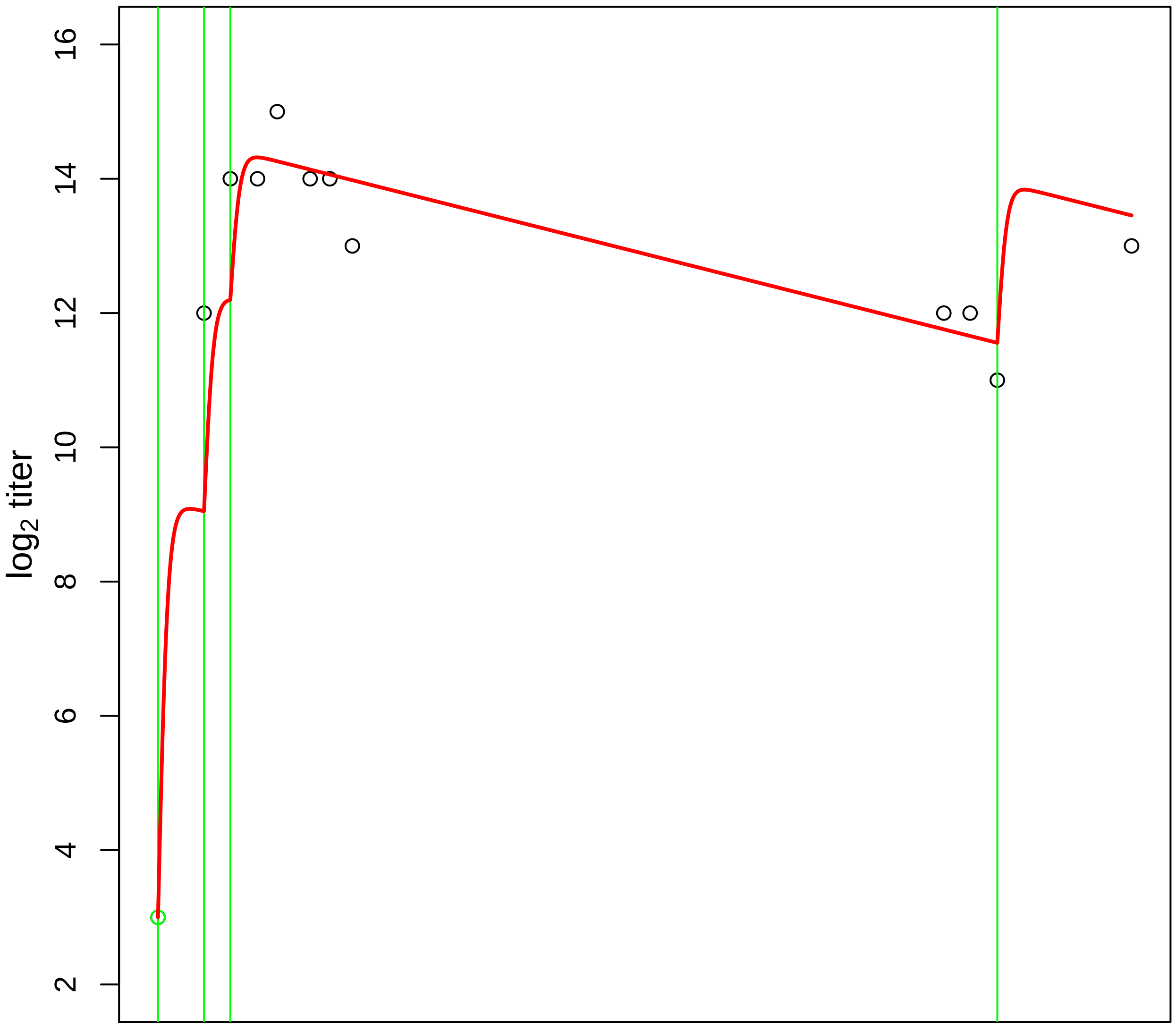

time in years from first donation of donor 573
mean absolute errors = 0.781 , mean squared errors = 1.3

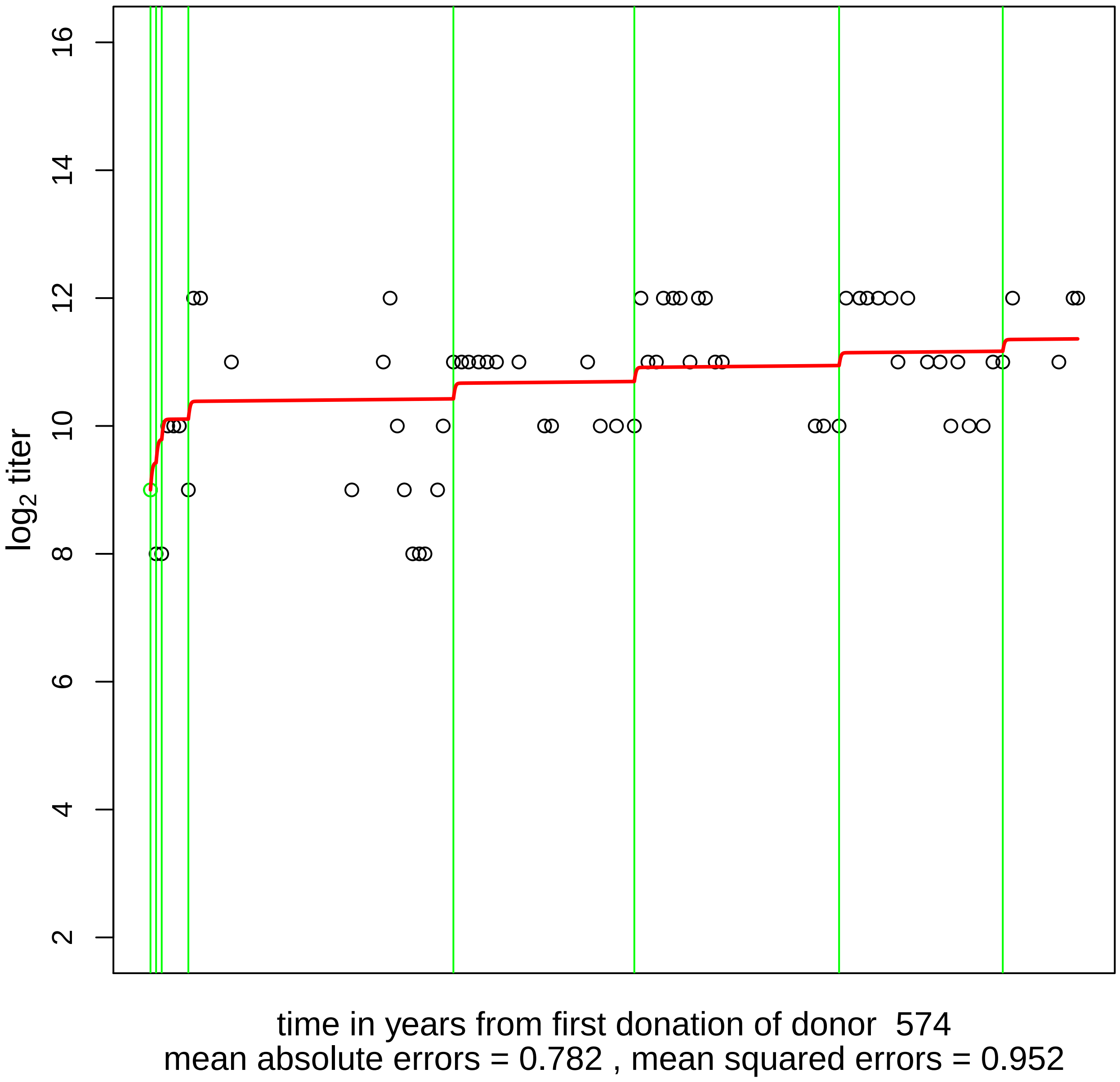

16
14
12
10
8
6
4
2
log2 titer
time in years from first donation of donor 574
mean absolute errors = 0.782 , mean squared errors = 0.952

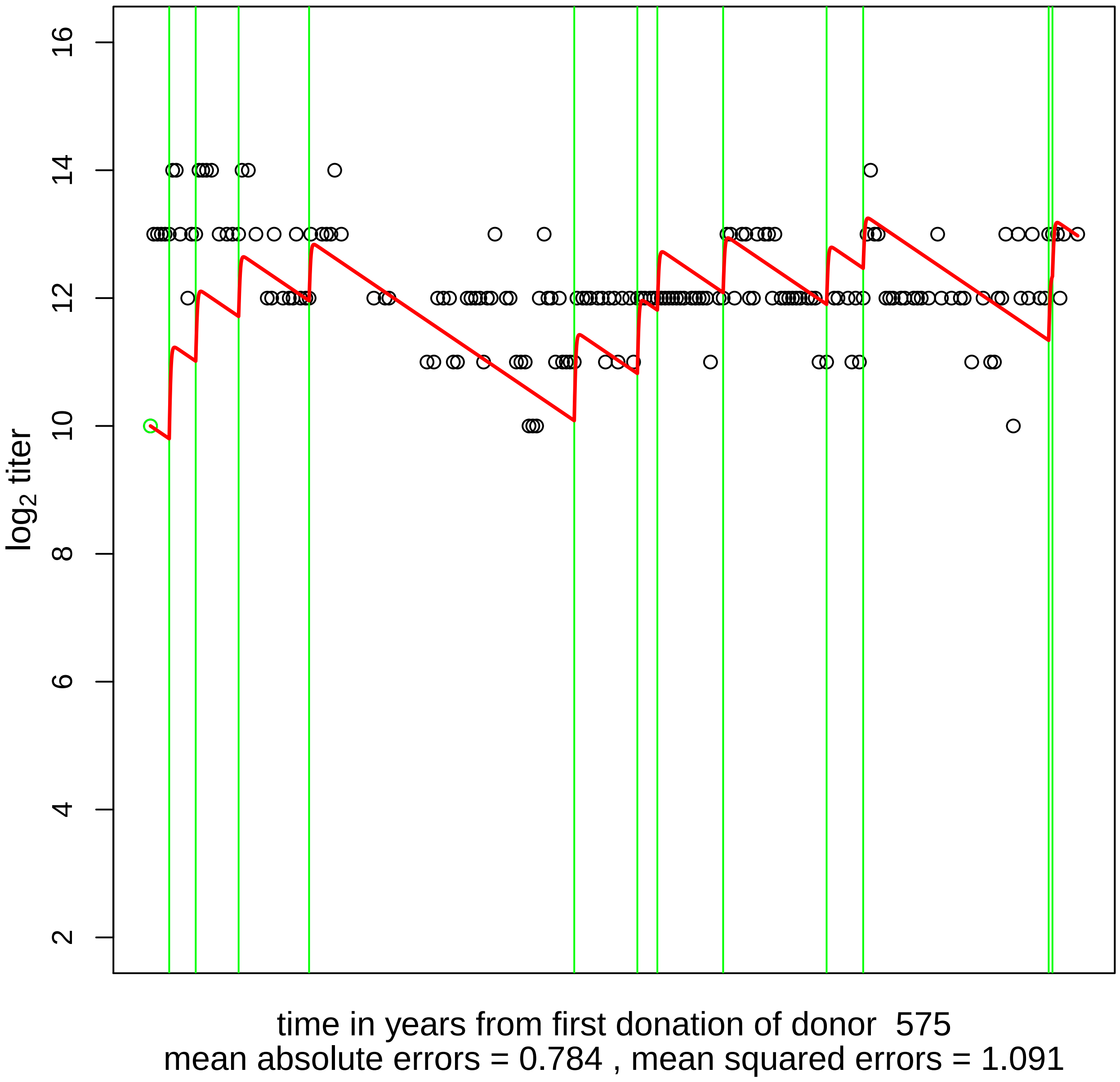

16
14
12
10
8
6
4
2
log2 titer
time in years from first donation of donor 575
mean absolute errors = 0.784 , mean squared errors = 1.091

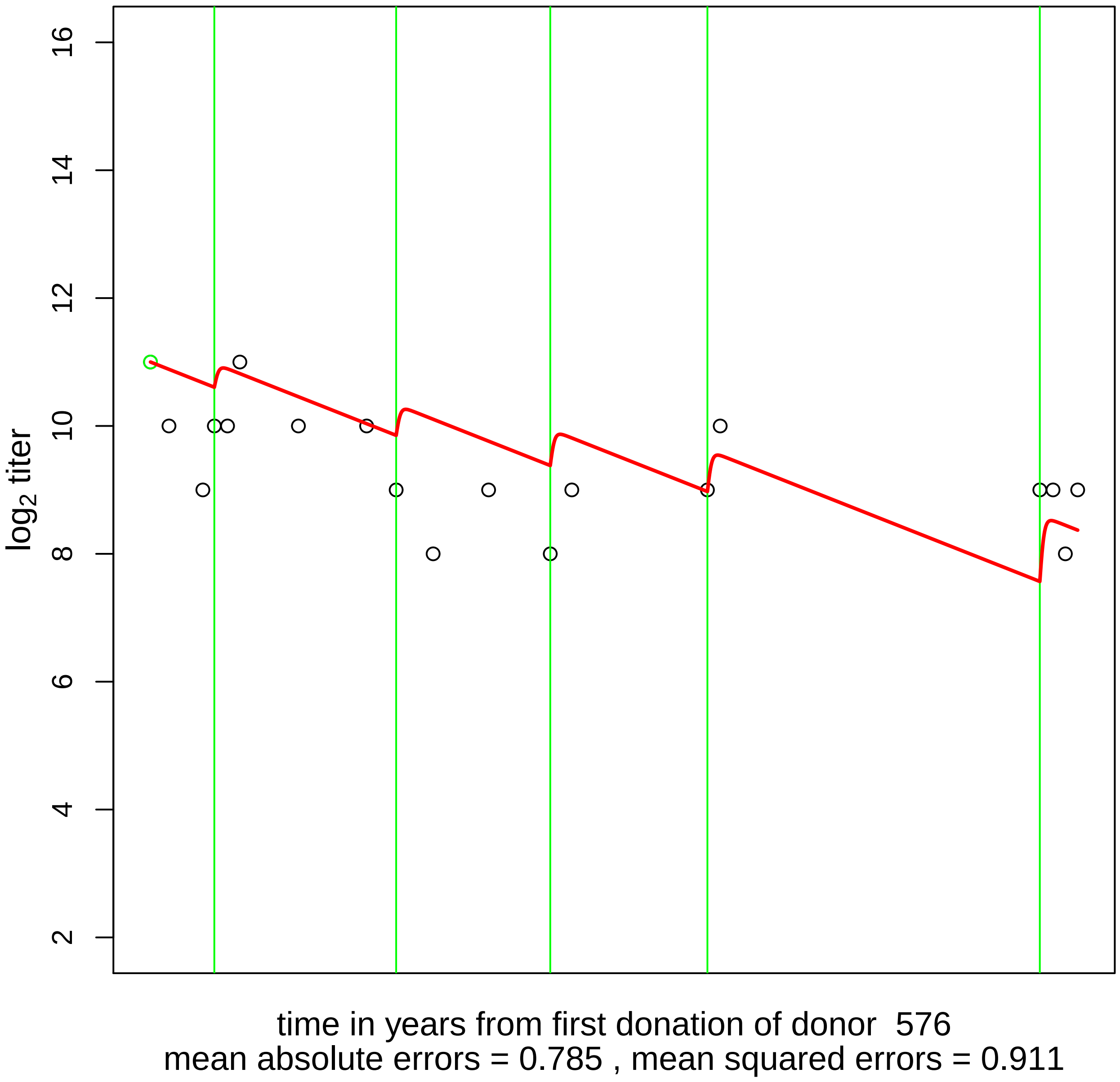
16
14
12
10
8
6
4
2
log2 titer
time in years from first donation of donor 576
mean absolute errors = 0.785 , mean squared errors = 0.911

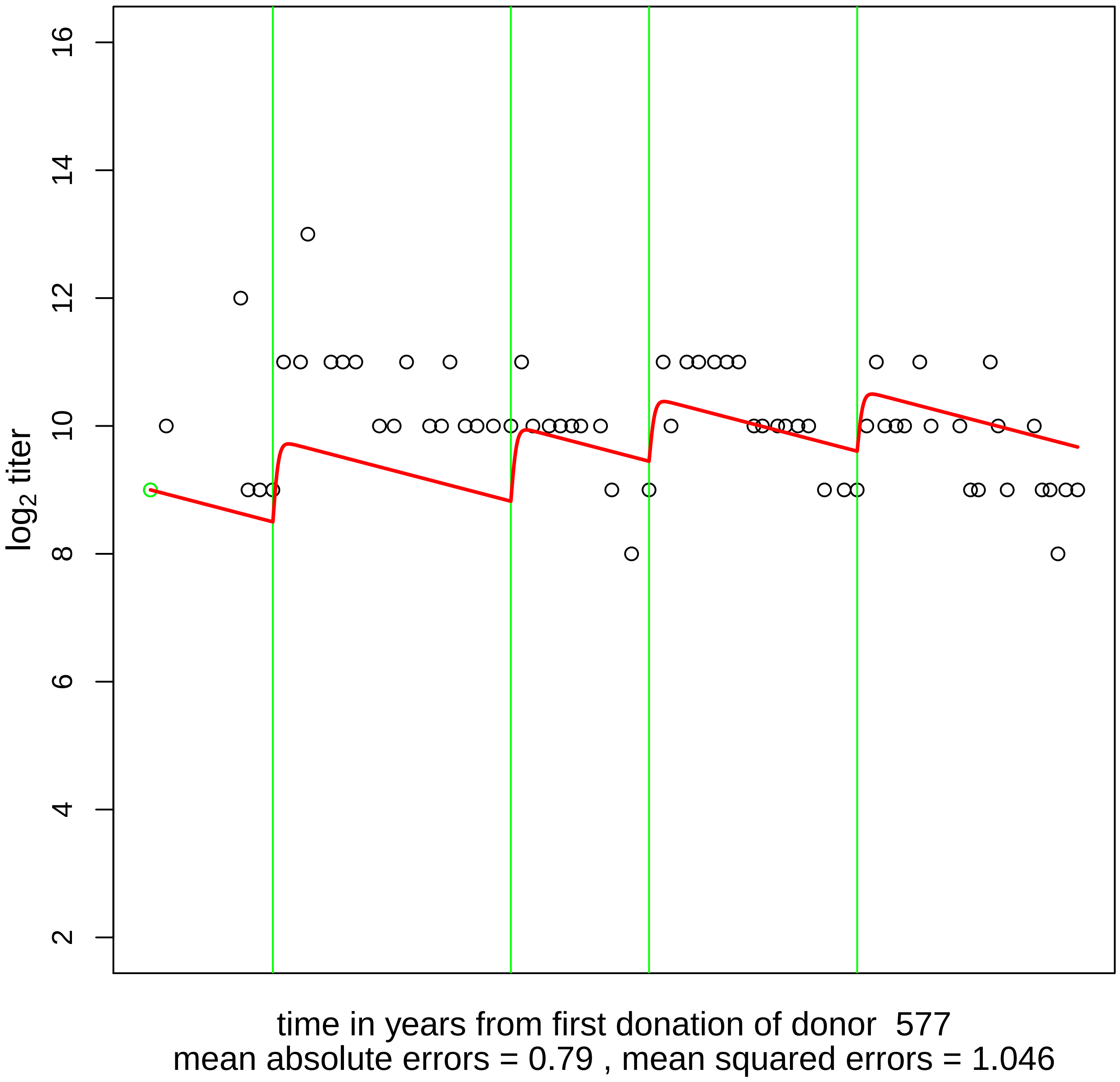
log2 titer
16
14
12
10
8
6
4
2
time in years from first donation of donor 577
mean absolute errors = 0.79 , mean squared errors = 1.046

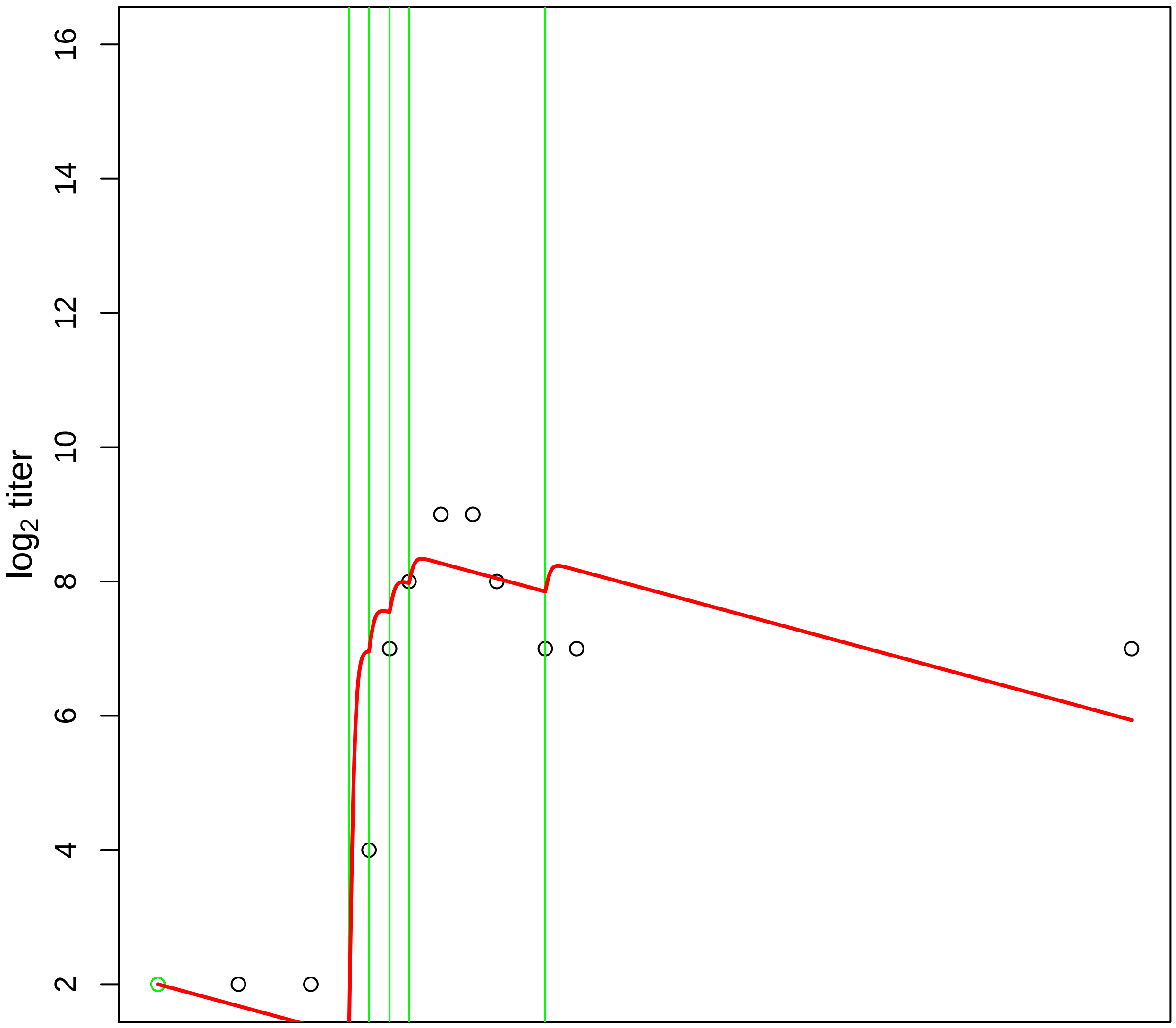

time in years from first donation of donor 578
mean absolute errors = 0.791 , mean squared errors = 1.14

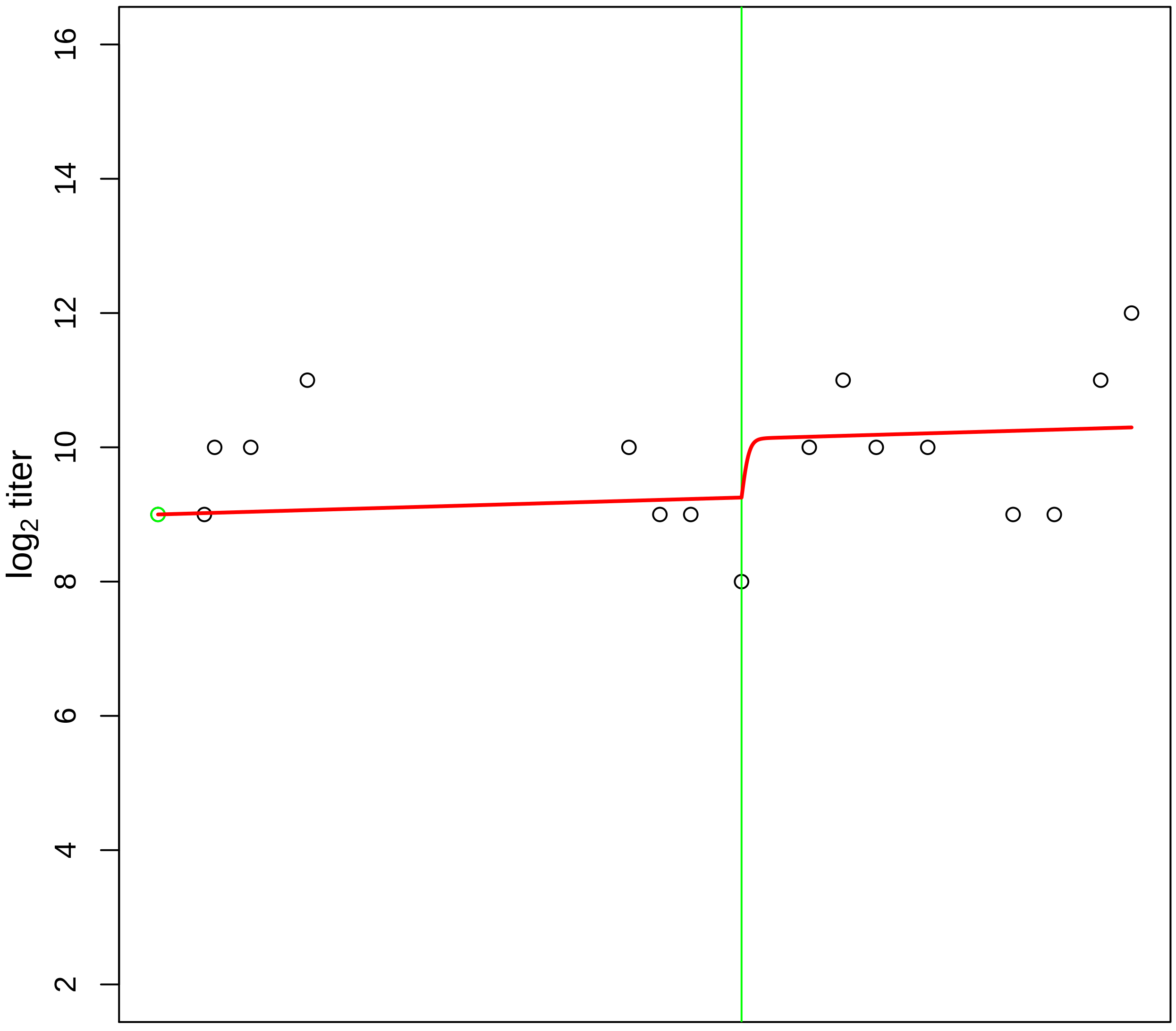

time in years from first donation of donor 579
mean absolute errors = 0.793 , mean squared errors = 0.955

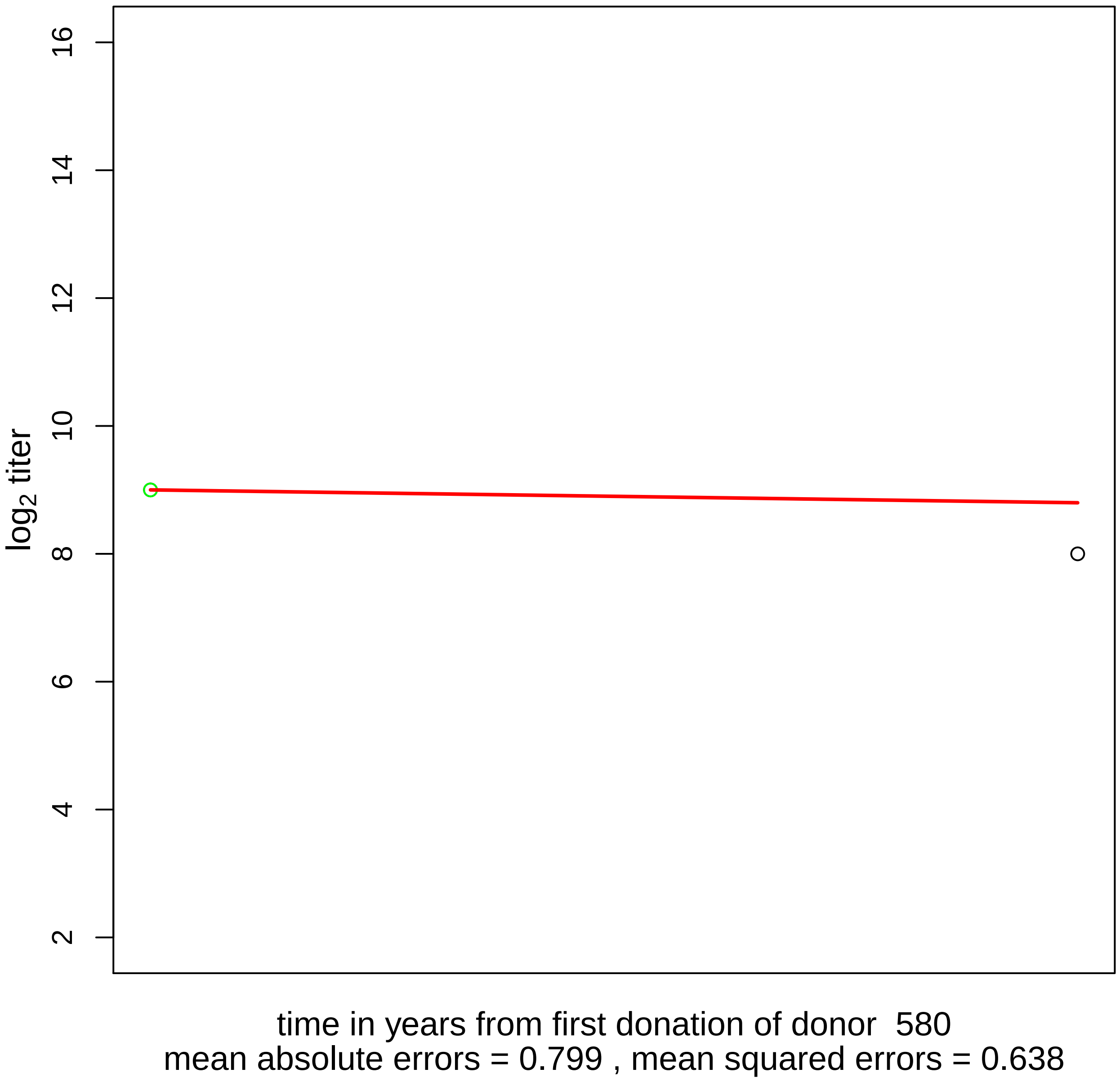

16
14
12
10
8
6
4
2
log2 titer
time in years from first donation of donor 580
mean absolute errors = 0.799 , mean squared errors = 0.638

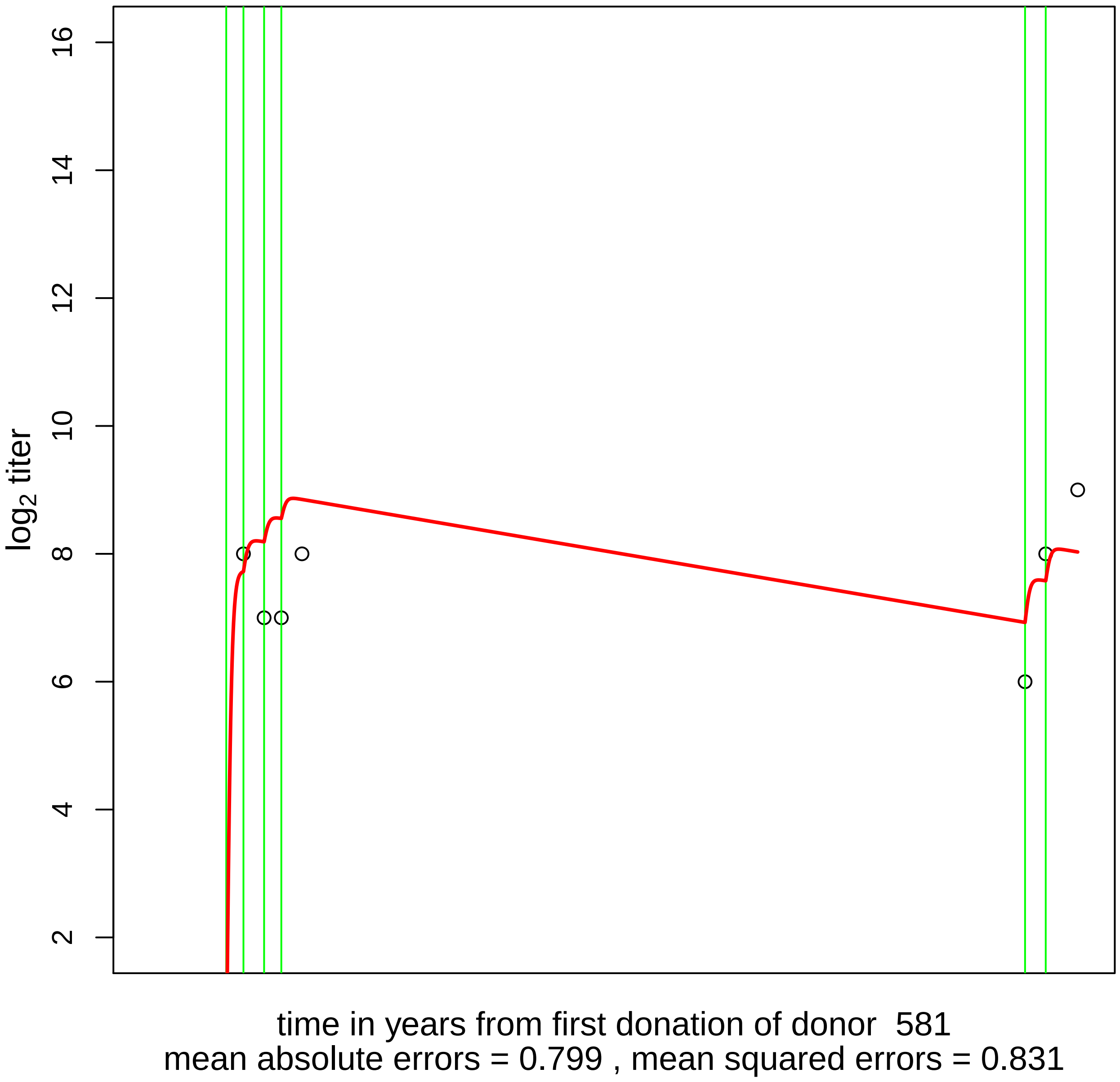

log2 titer
2
4
6
8
10
12
14
16
time in years from first donation of donor 581
mean absolute errors = 0.799 , mean squared errors = 0.831

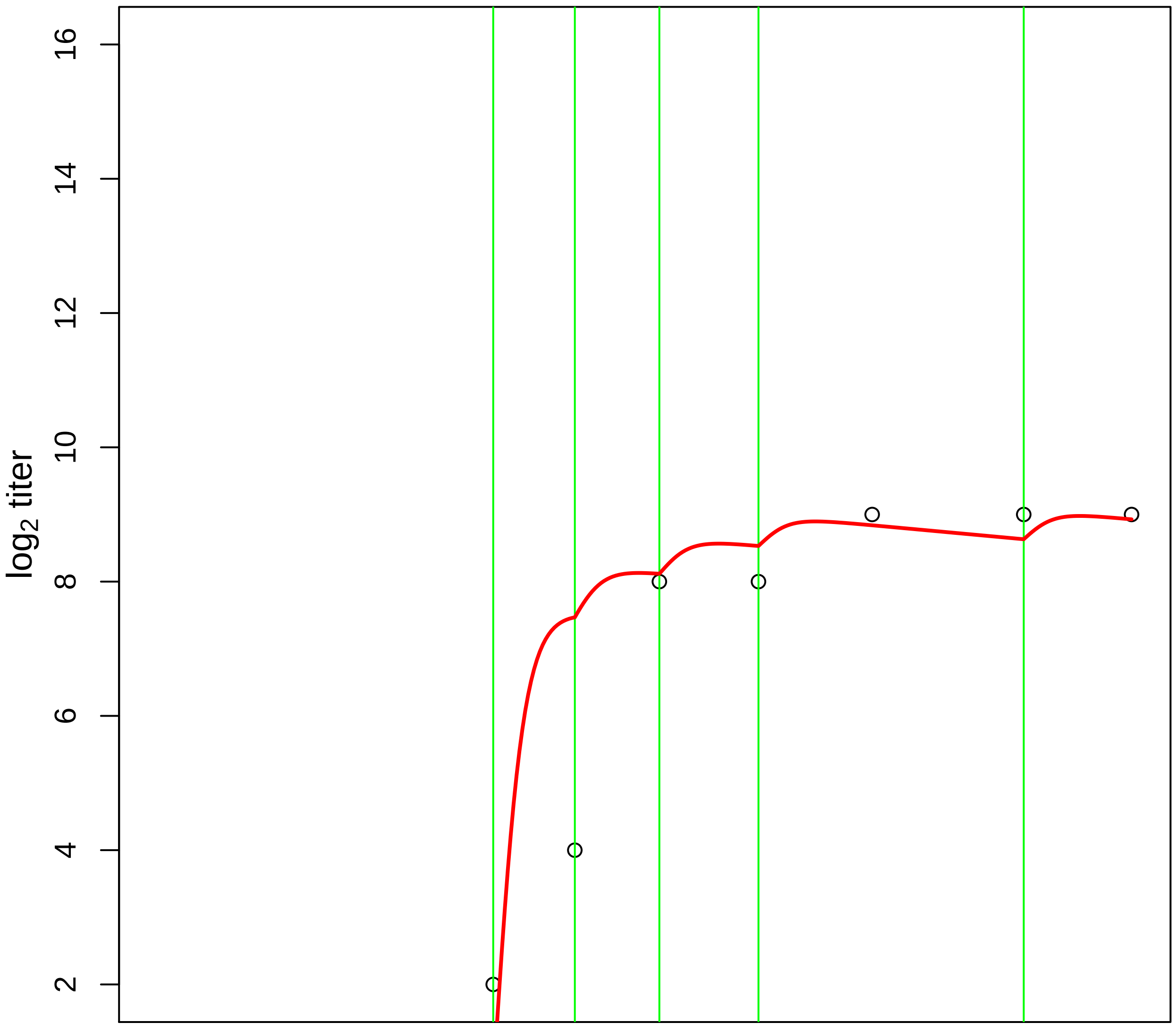

time in years from first donation of donor 582
mean absolute errors = 0.799 , mean squared errors = 1.833

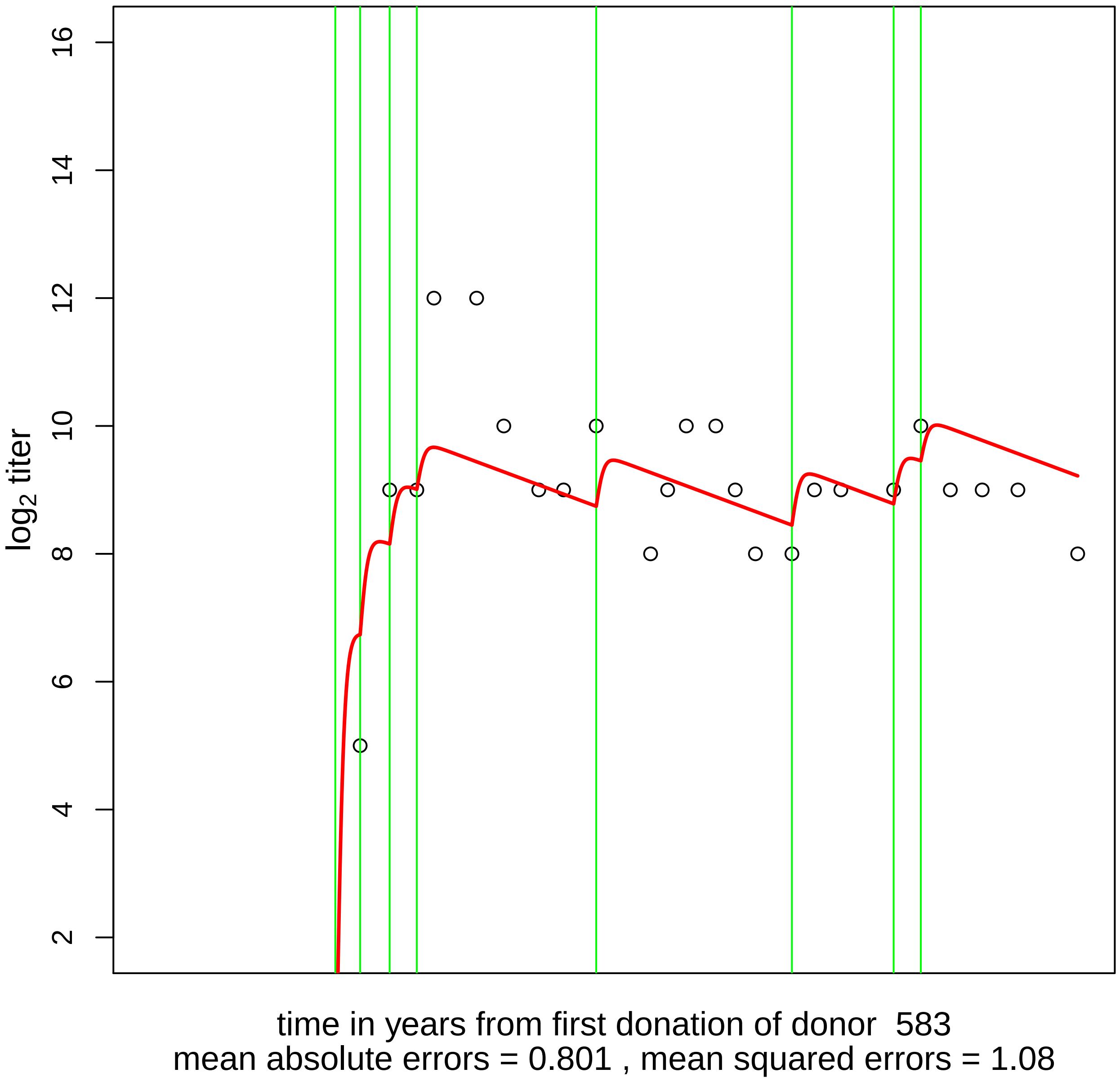

2
4
6
8
10
12
14
16
$\log_2$ titer
time in years from first donation of donor 583
mean absolute errors = 0.801 , mean squared errors = 1.08

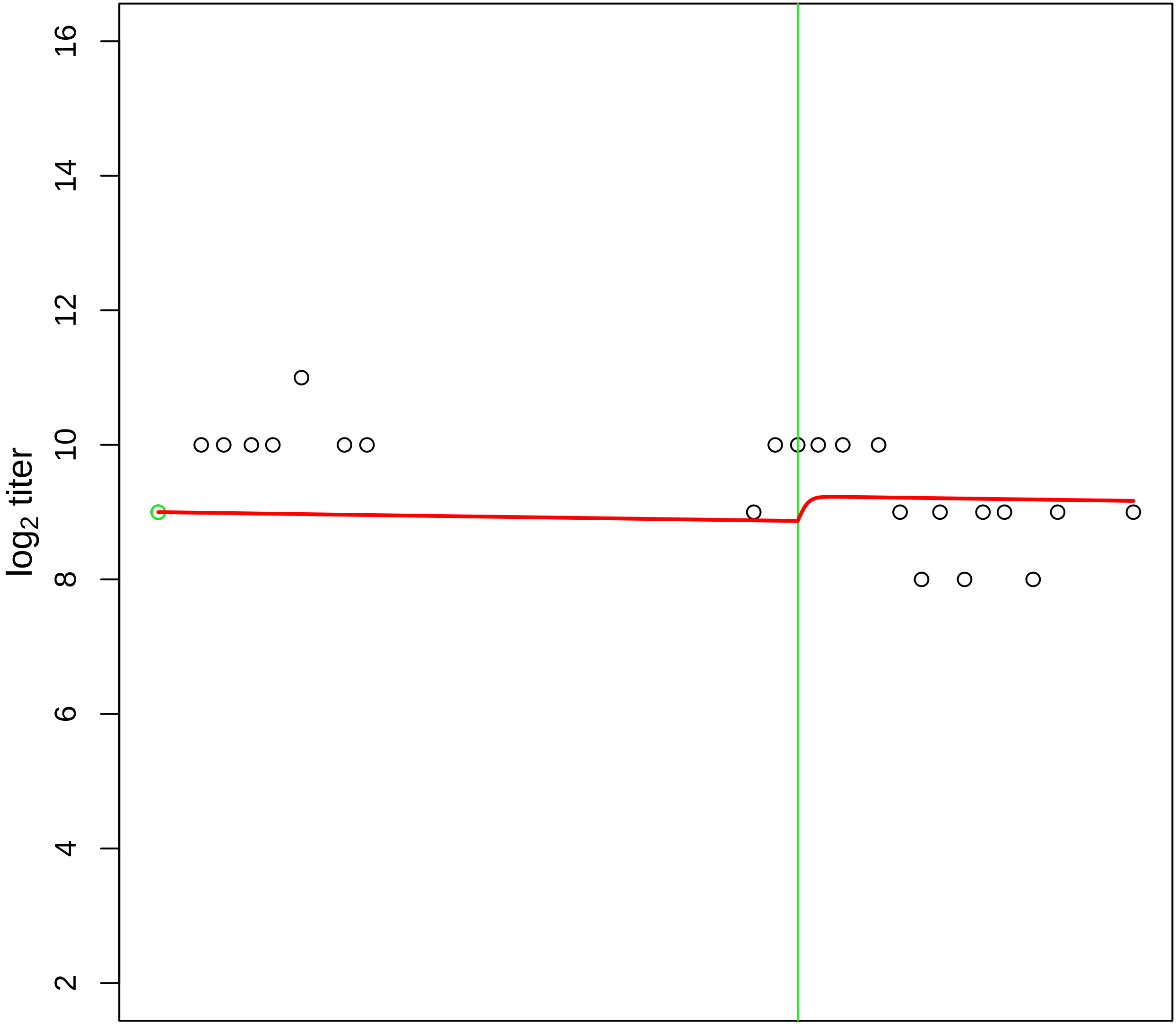

time in years from first donation of donor 584
mean absolute errors = 0.803 , mean squared errors = 0.879

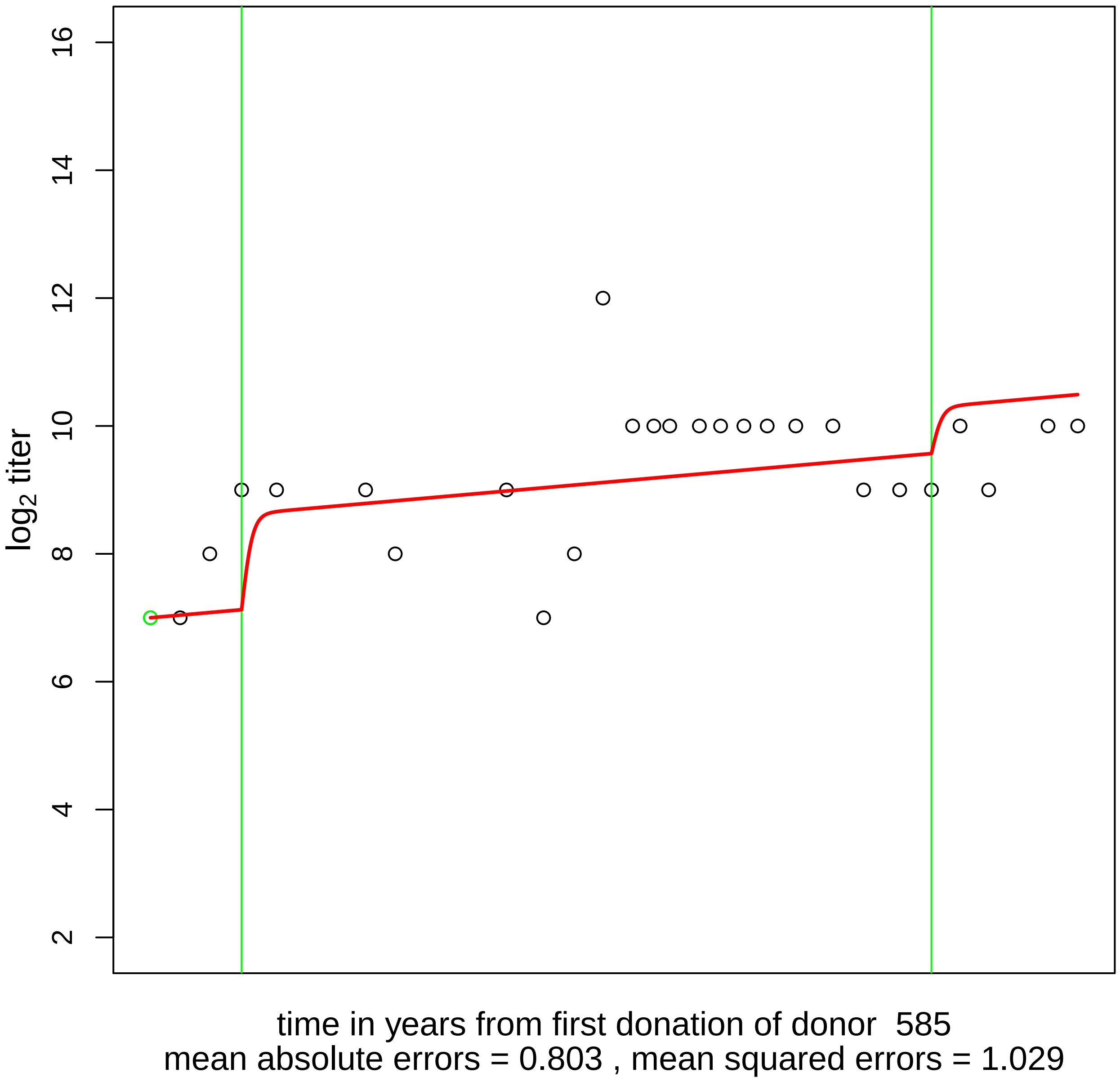

16
14
12
10
8
6
4
2
log2 titer
time in years from first donation of donor 585
mean absolute errors = 0.803 , mean squared errors = 1.029

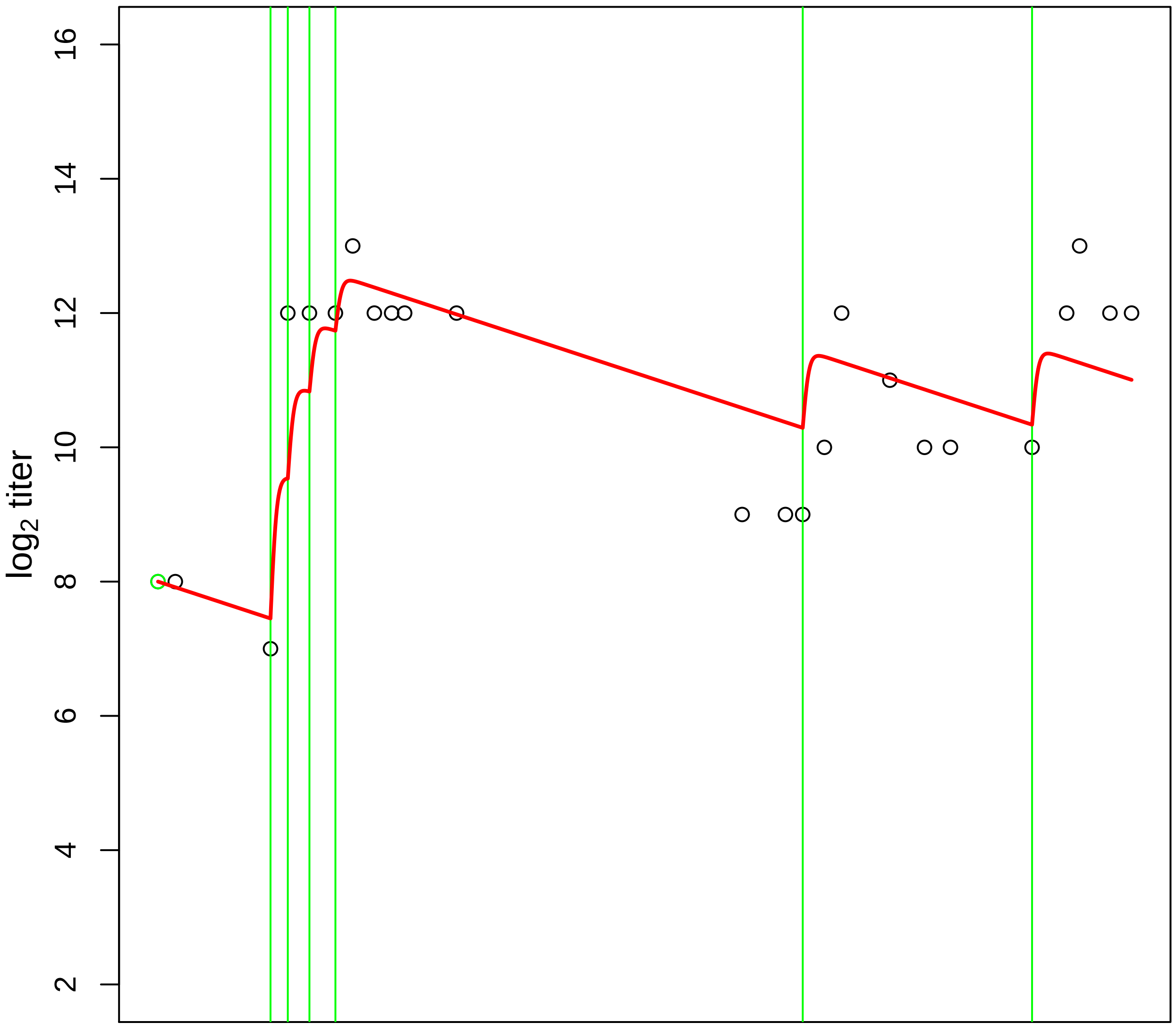

time in years from first donation of donor 586
mean absolute errors = 0.804 , mean squared errors = 1.016

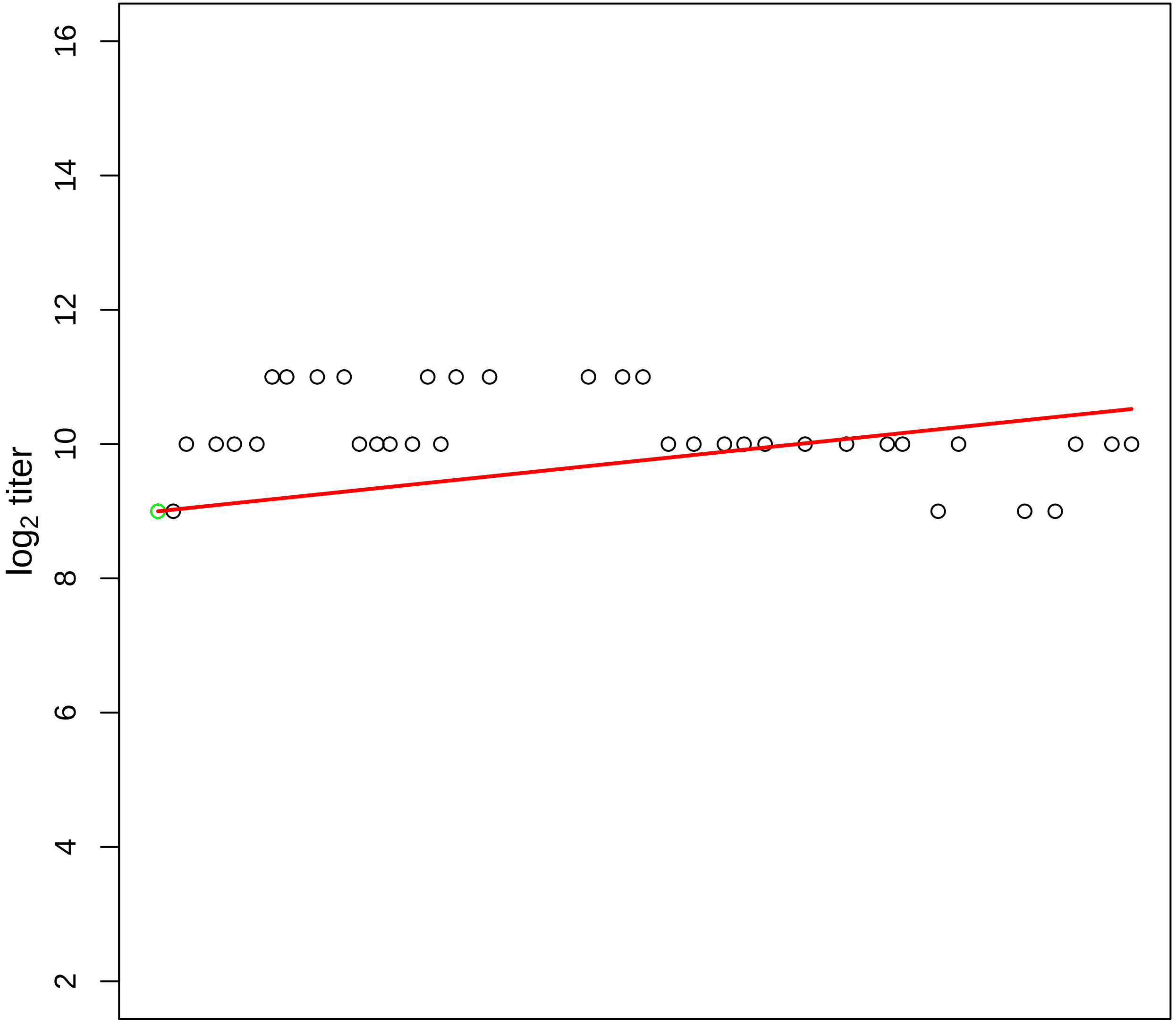

time in years from first donation of donor 587
mean absolute errors = 0.804 , mean squared errors = 0.998

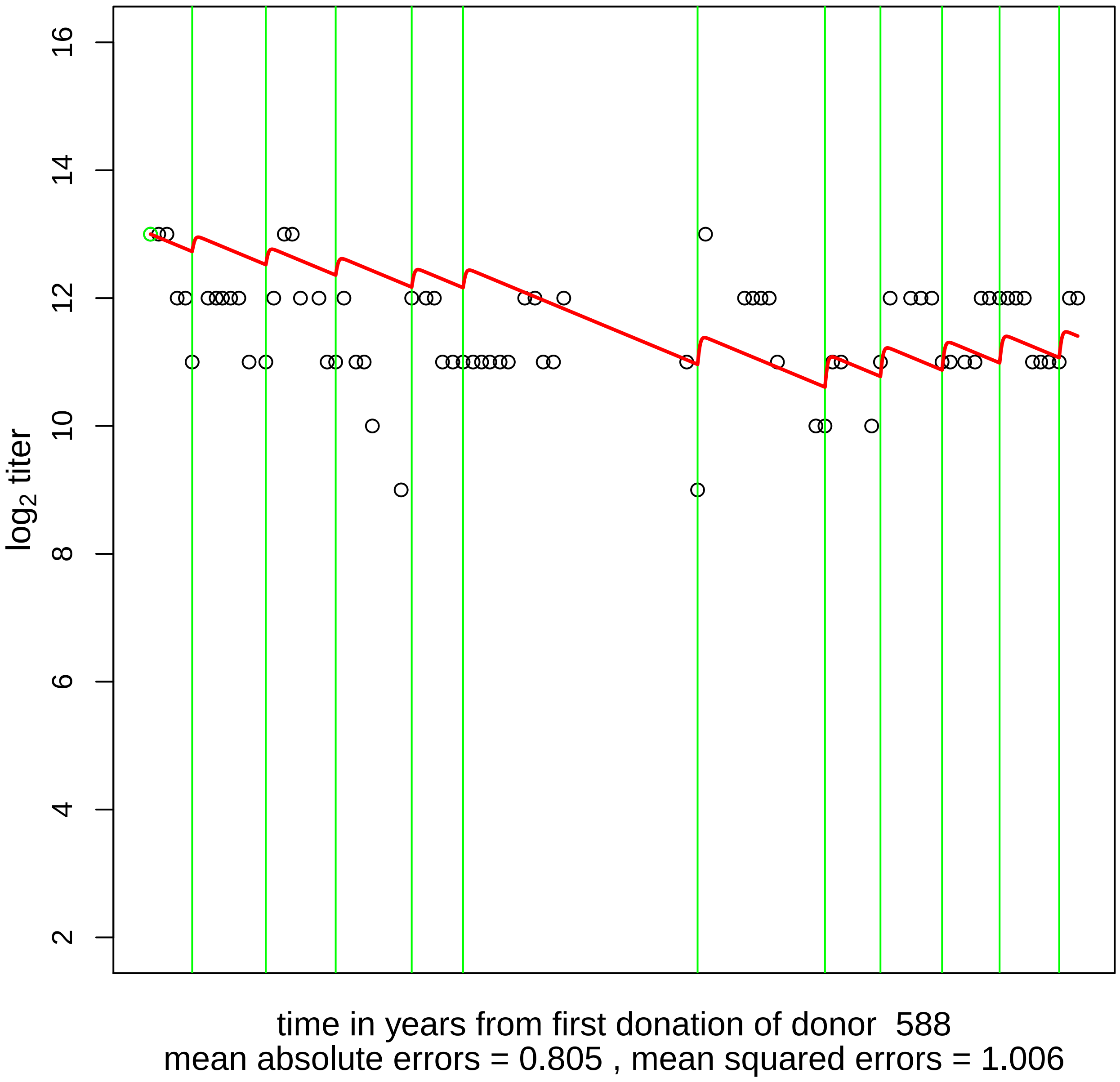
16
14
12
10
8
6
4
2
log2 titer
time in years from first donation of donor 588
mean absolute errors = 0.805 , mean squared errors = 1.006

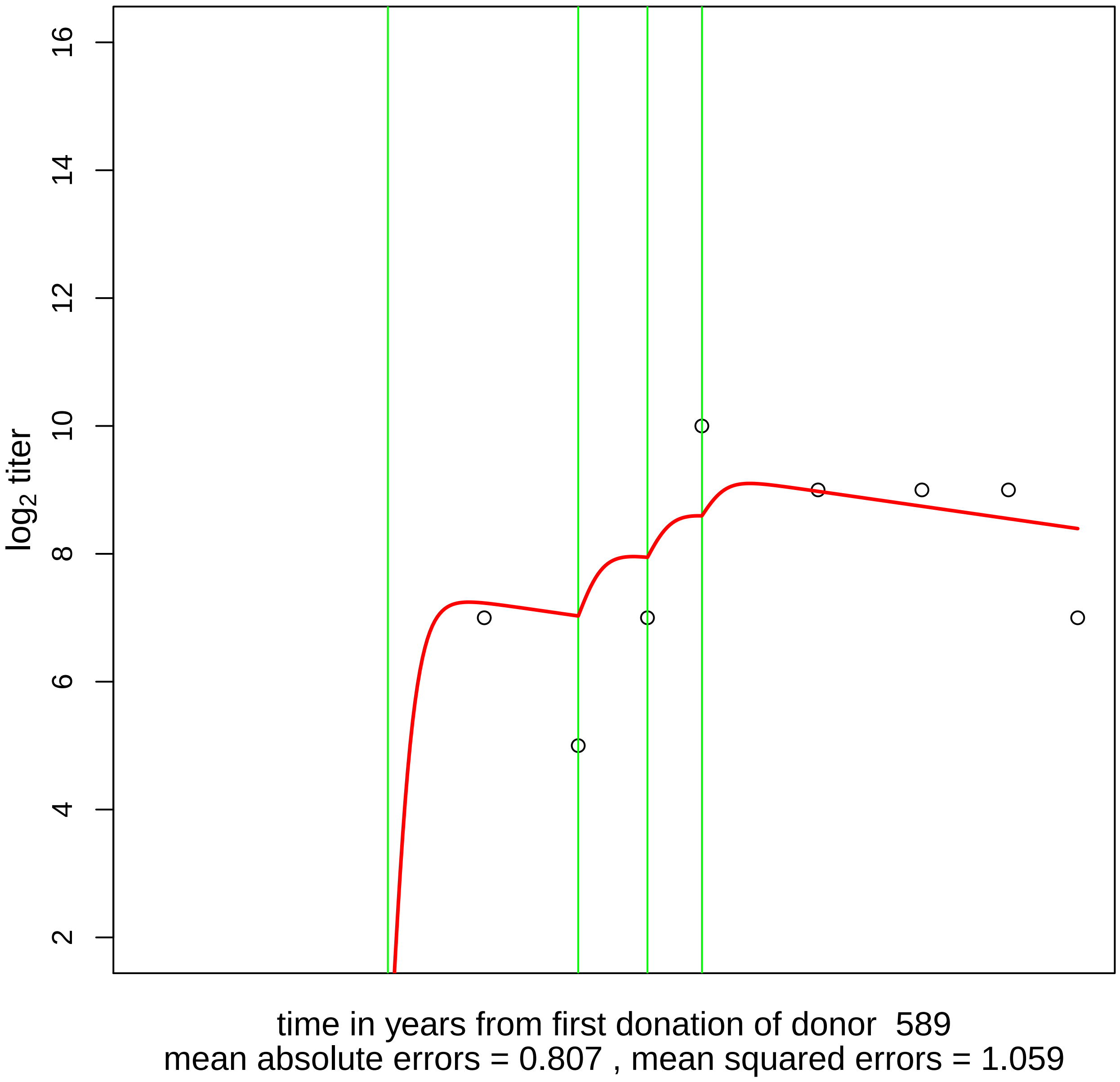

log2 titer
2
4
6
8
10
12
14
16
time in years from first donation of donor 589
mean absolute errors = 0.807 , mean squared errors = 1.059

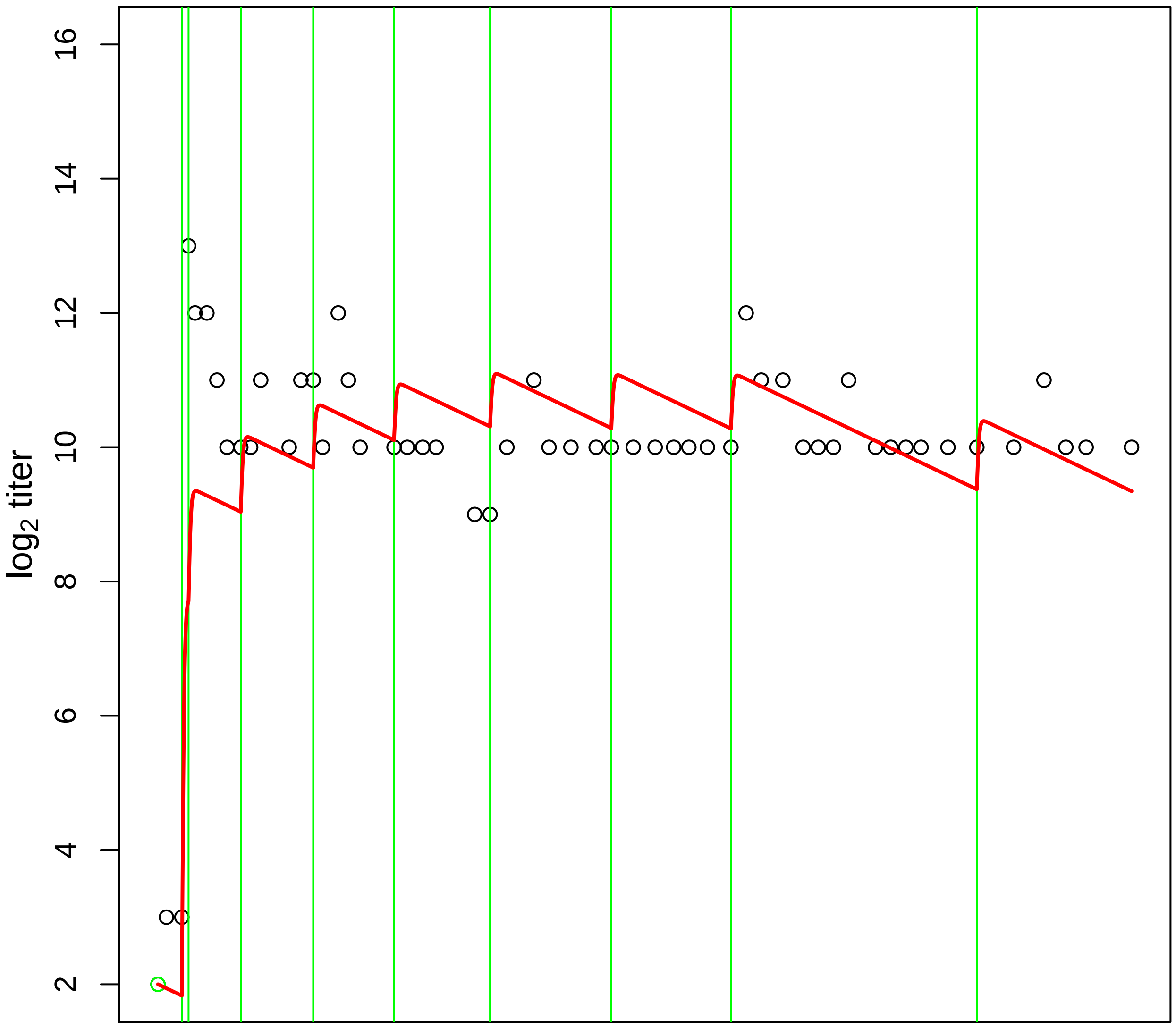

time in years from first donation of donor 590
mean absolute errors = 0.809 , mean squared errors = 1.361

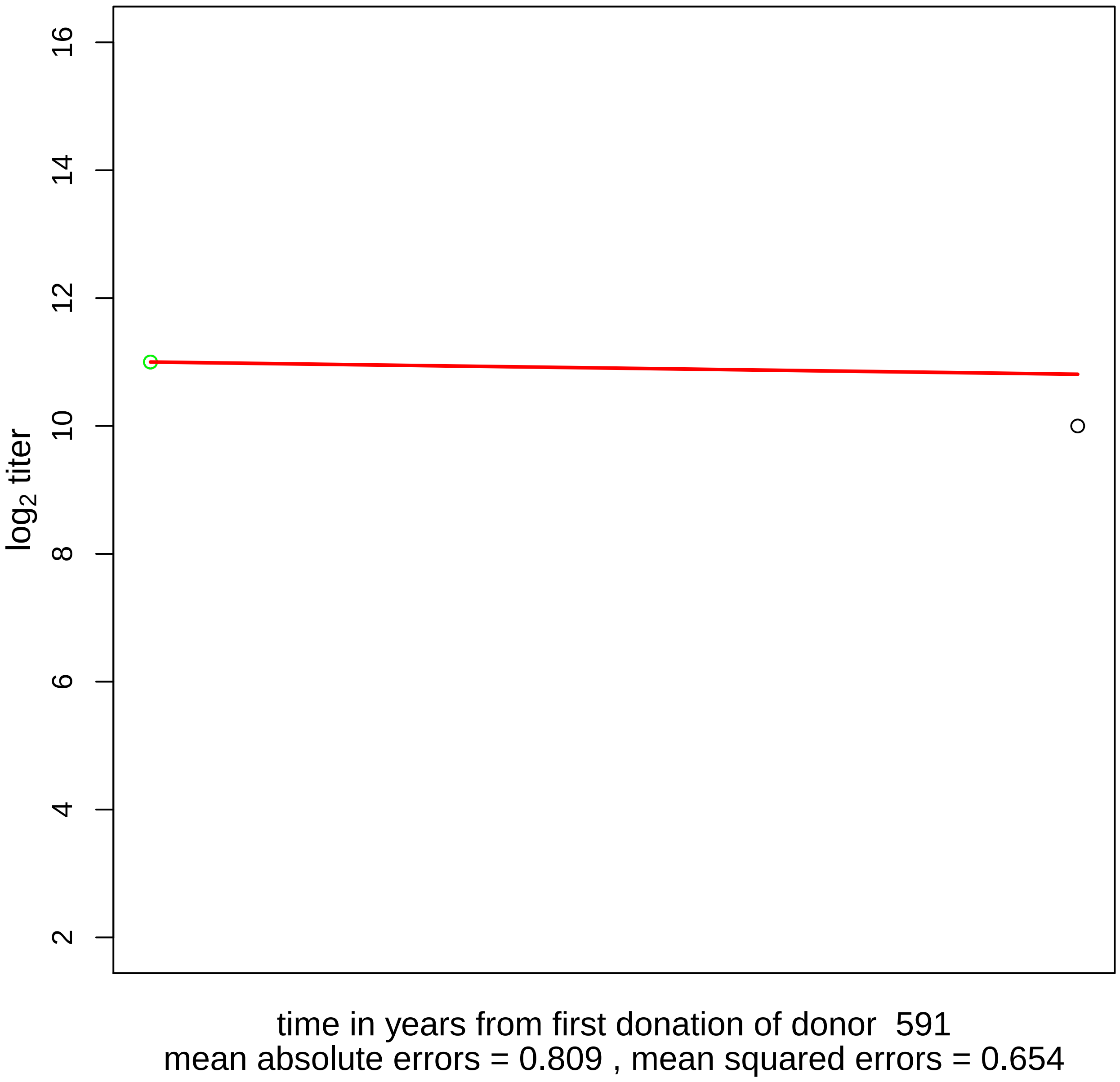

2
4
6
8
10
12
14
16
log2 titer
time in years from first donation of donor 591
mean absolute errors = 0.809 , mean squared errors = 0.654

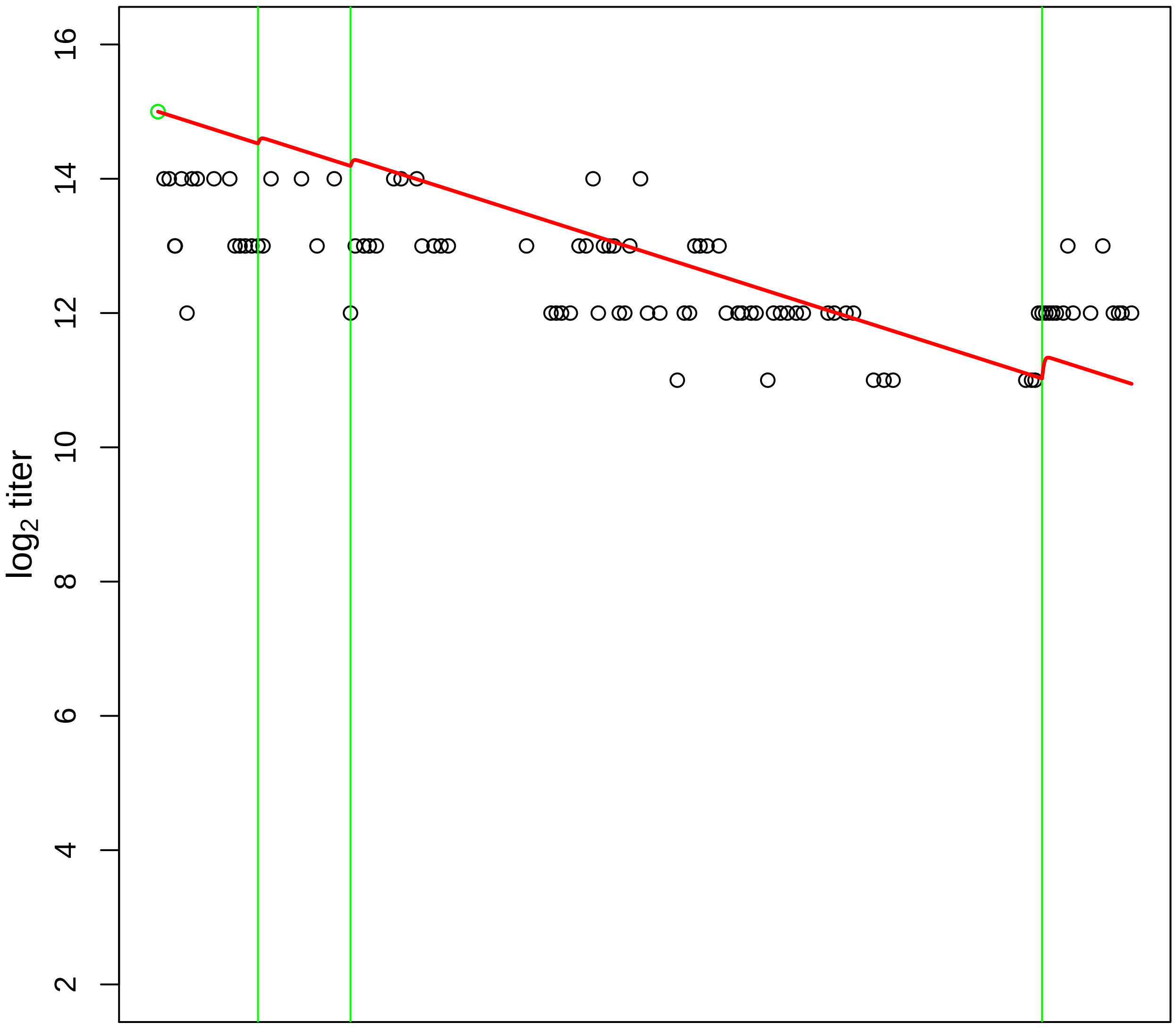

time in years from first donation of donor 592
mean absolute errors = 0.814 , mean squared errors = 0.99

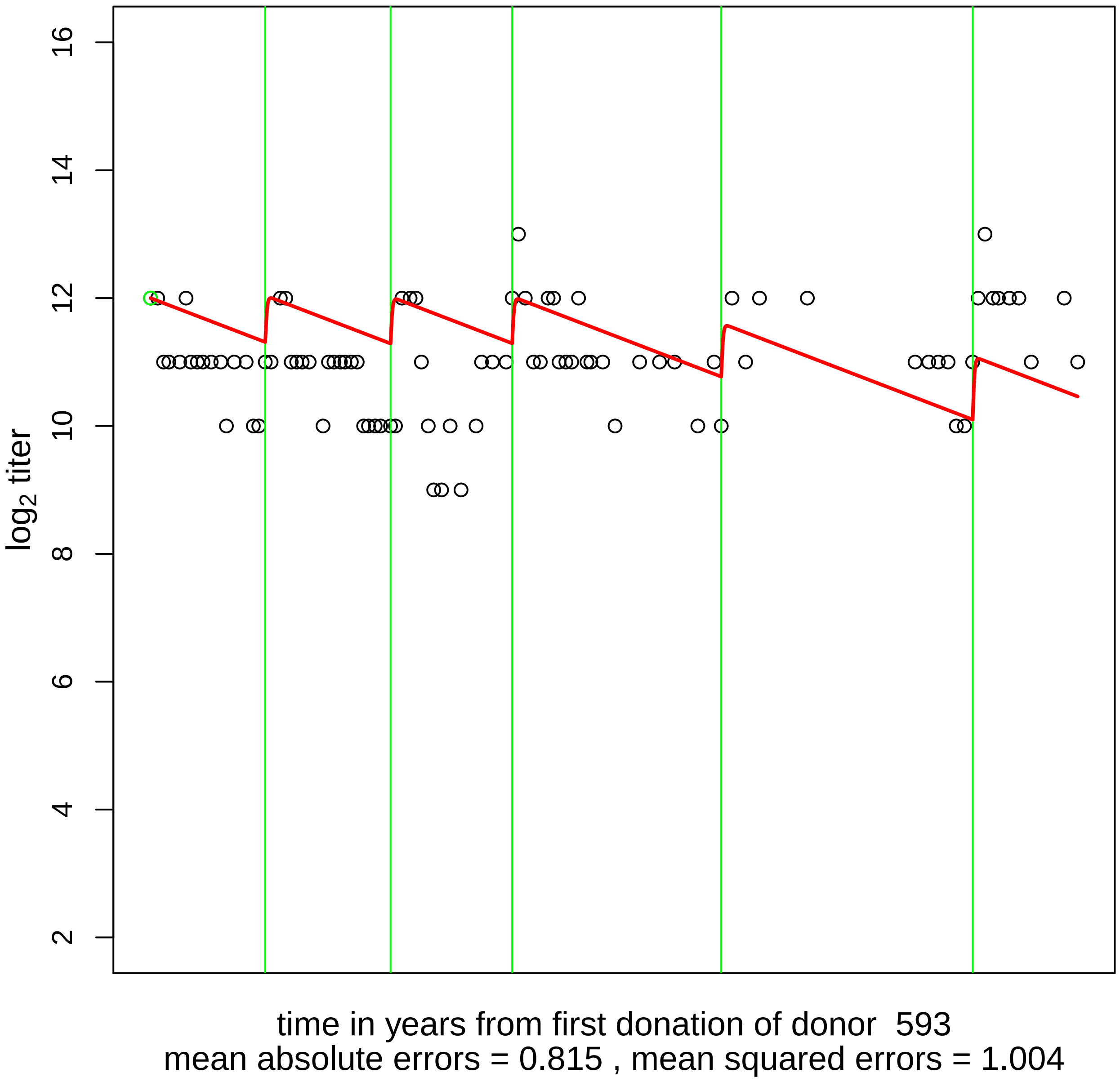

16
14
12
10
8
6
4
2
log2 titer
time in years from first donation of donor 593
mean absolute errors = 0.815 , mean squared errors = 1.004

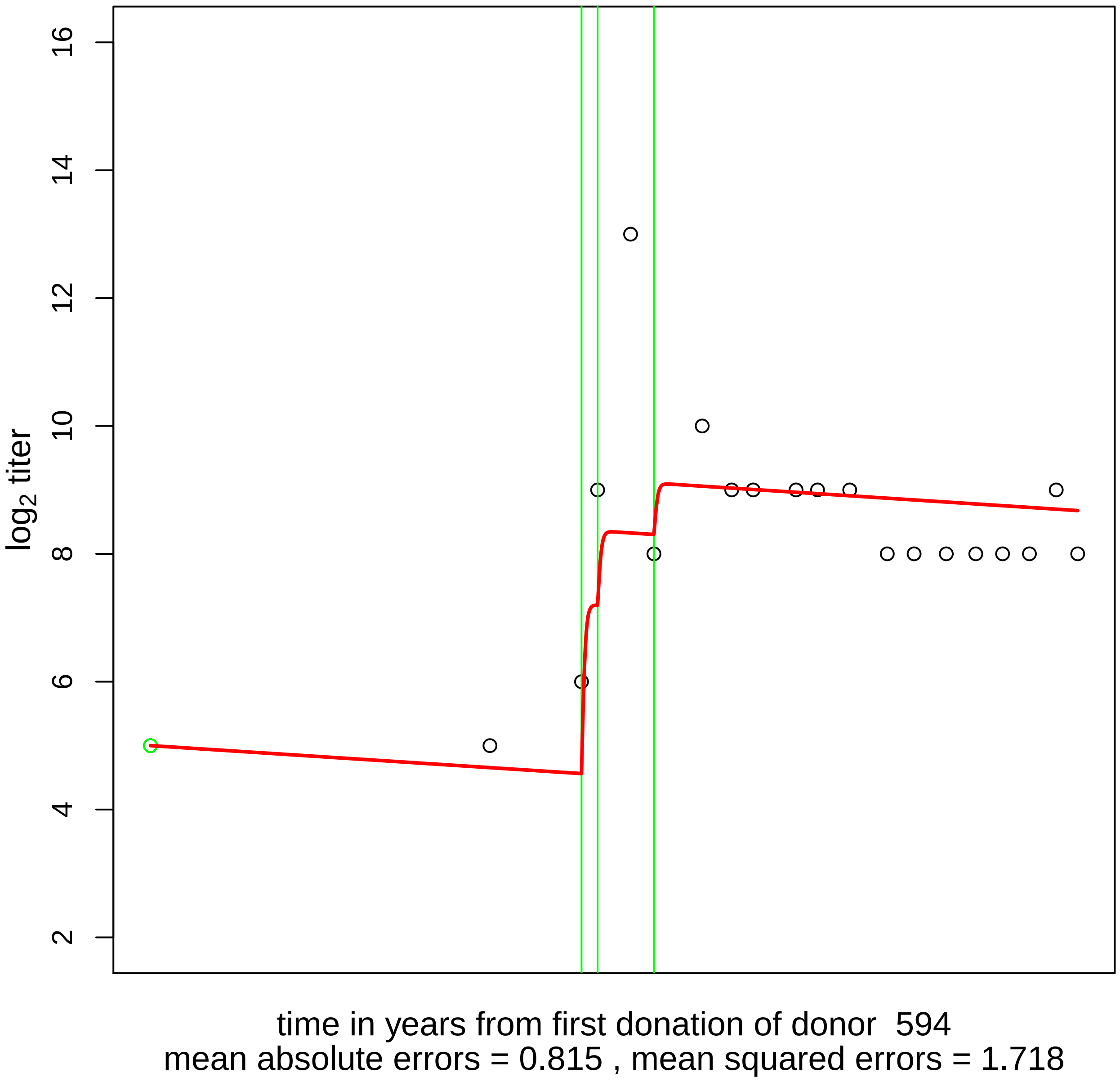

log2 titer
16
14
12
10
8
6
4
2
time in years from first donation of donor 594
mean absolute errors = 0.815 , mean squared errors = 1.718

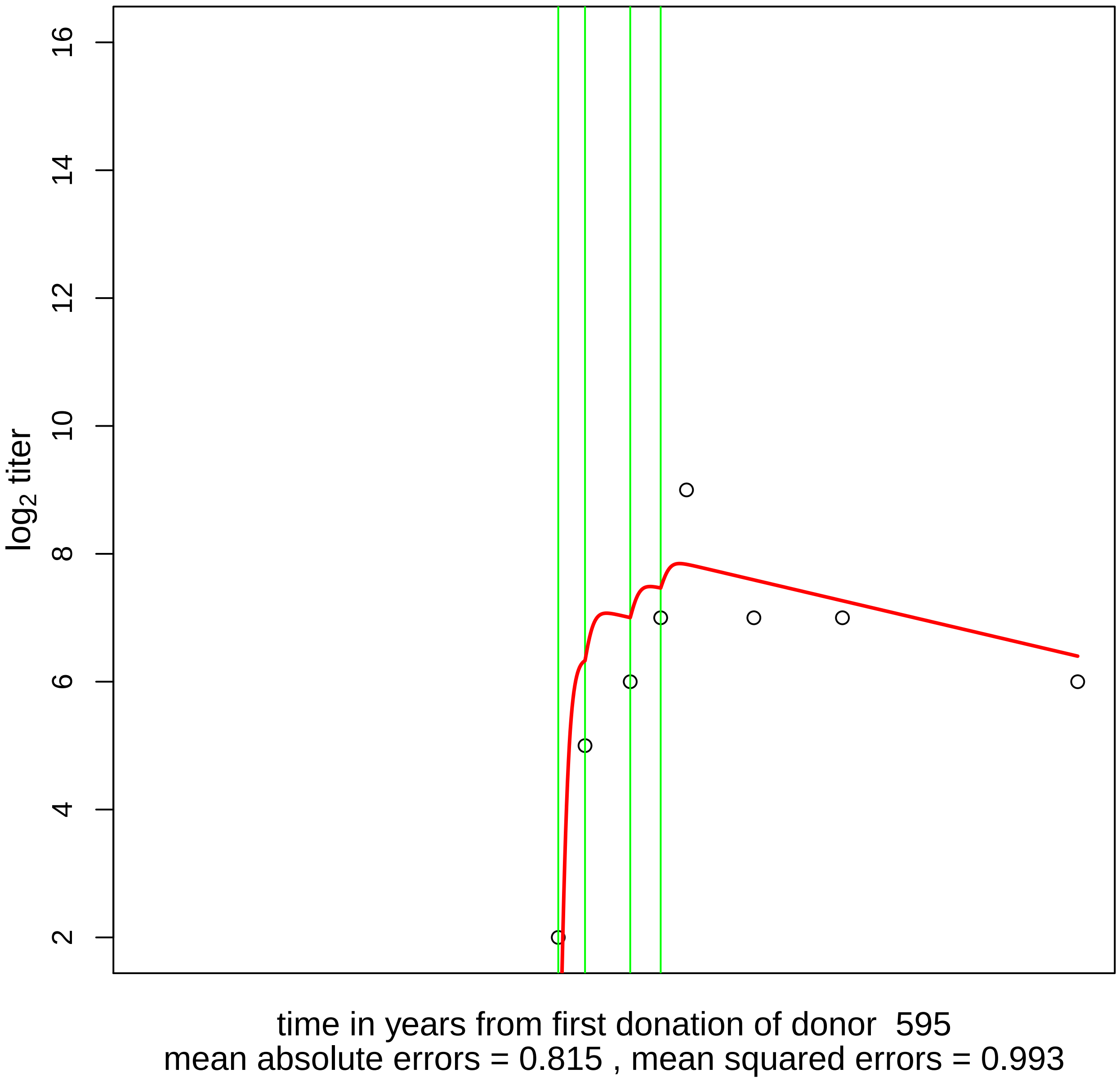

16
14
12
10
8
6
4
2
log2 titer
time in years from first donation of donor 595
mean absolute errors = 0.815 , mean squared errors = 0.993

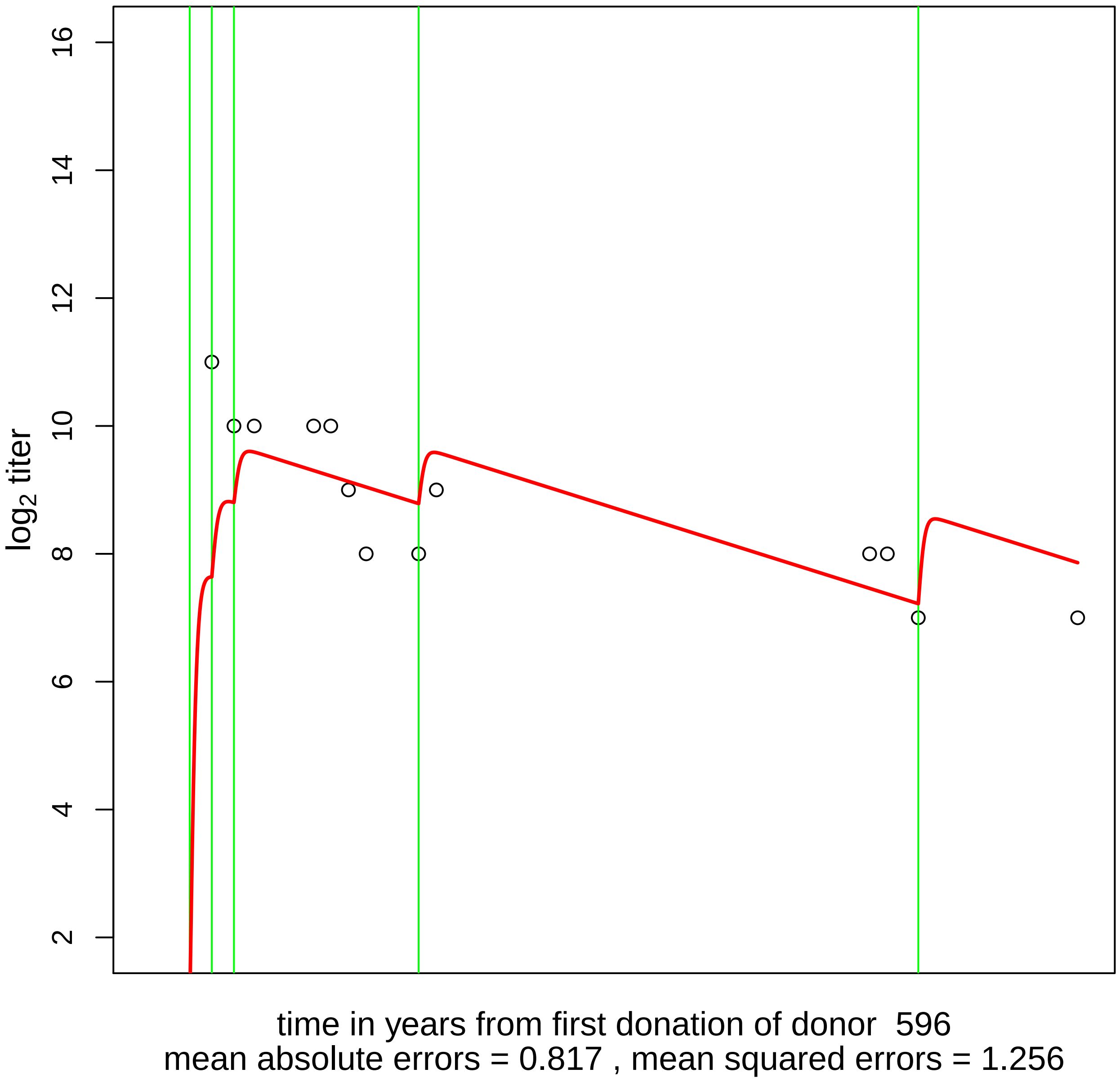
log2 titer
16
14
12
10
8
6
4
2
time in years from first donation of donor 596
mean absolute errors = 0.817 , mean squared errors = 1.256

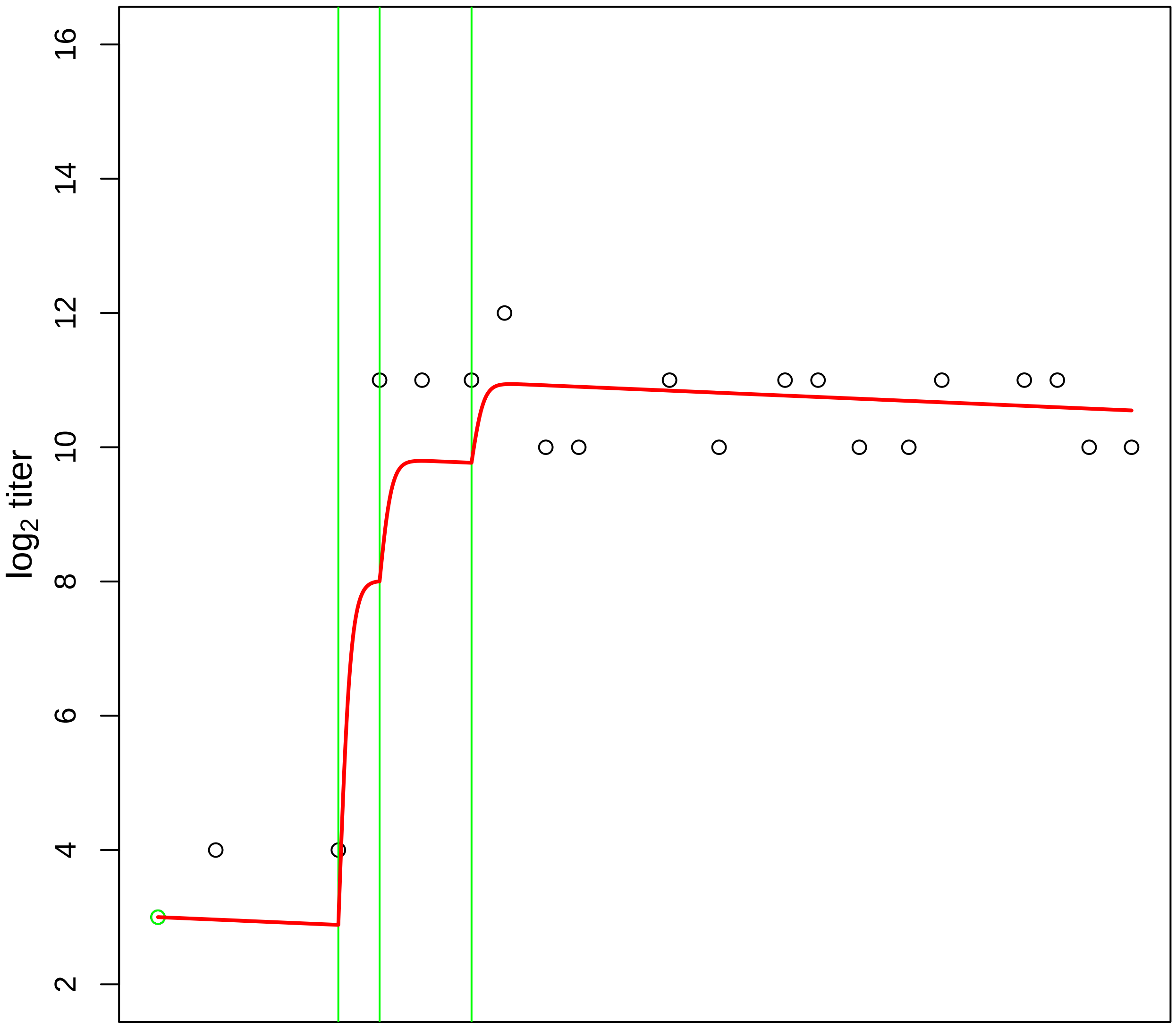

time in years from first donation of donor 597
mean absolute errors = 0.82 , mean squared errors = 1.048

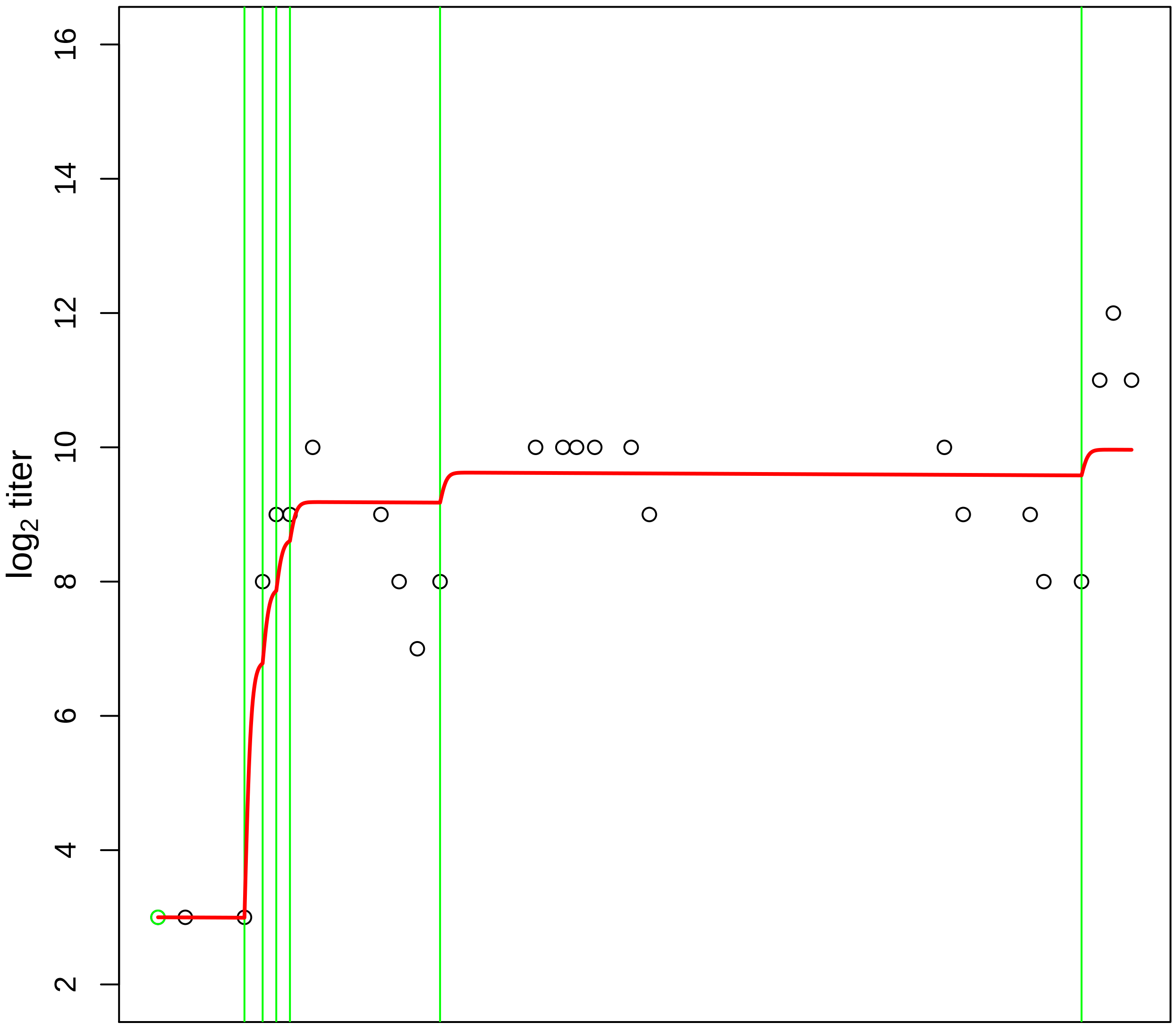

time in years from first donation of donor 598
mean absolute errors = 0.82 , mean squared errors = 1.018

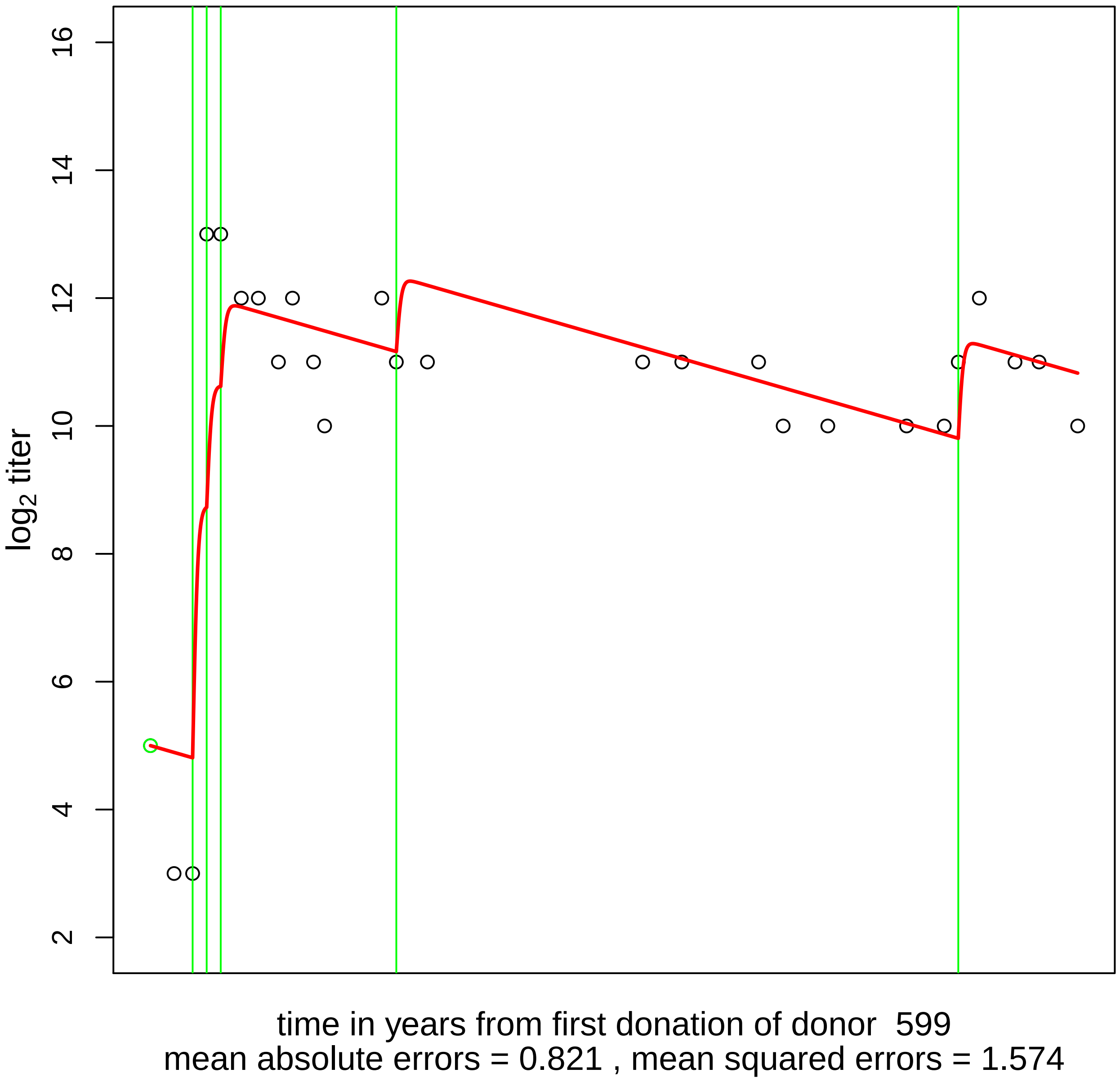

16
14
12
10
8
6
4
2
$\log_2$ titer
time in years from first donation of donor 599
mean absolute errors = 0.821 , mean squared errors = 1.574

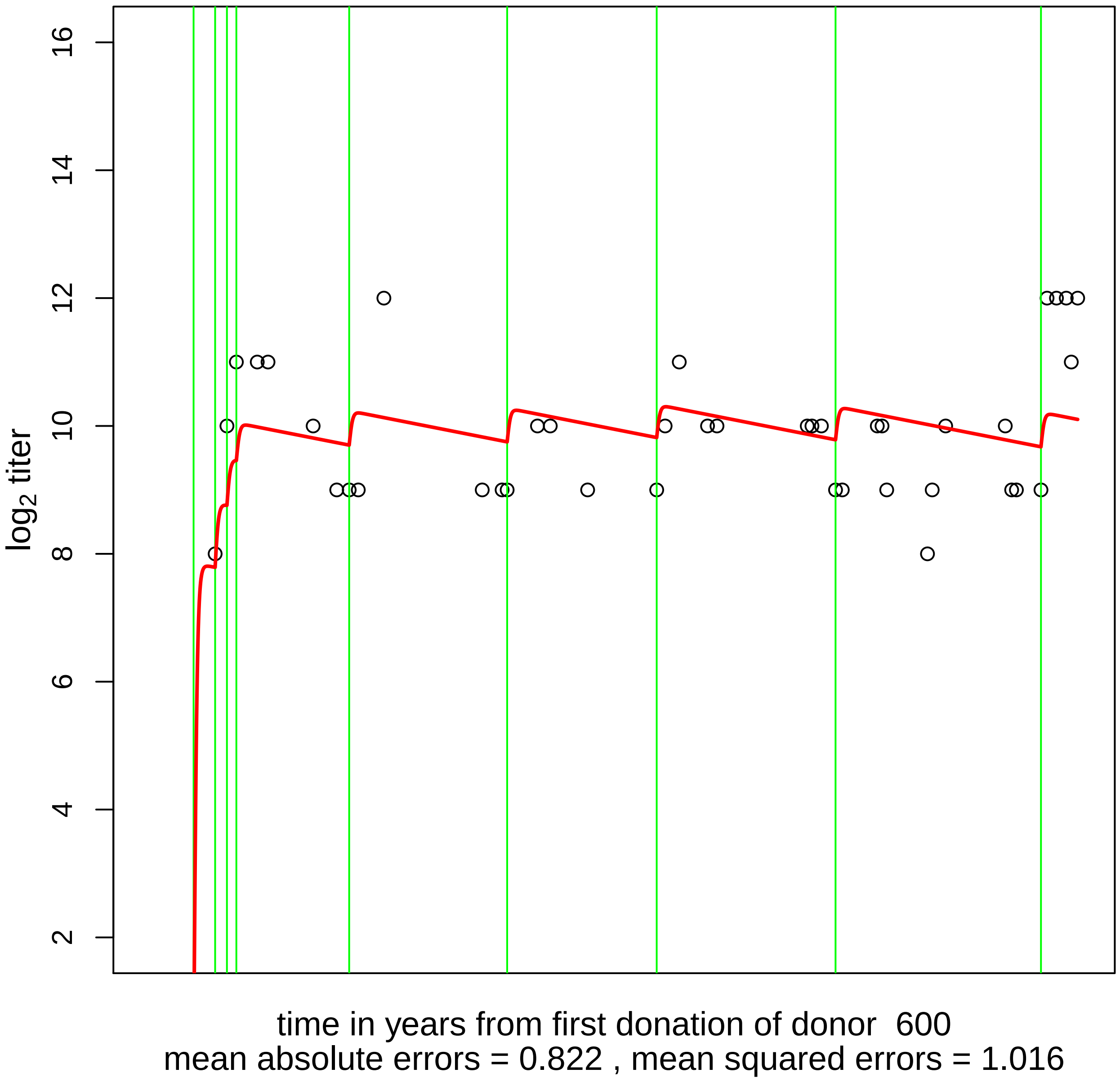

2
4
6
8
10
12
14
16
log2 titer
time in years from first donation of donor 600
mean absolute errors = 0.822 , mean squared errors = 1.016

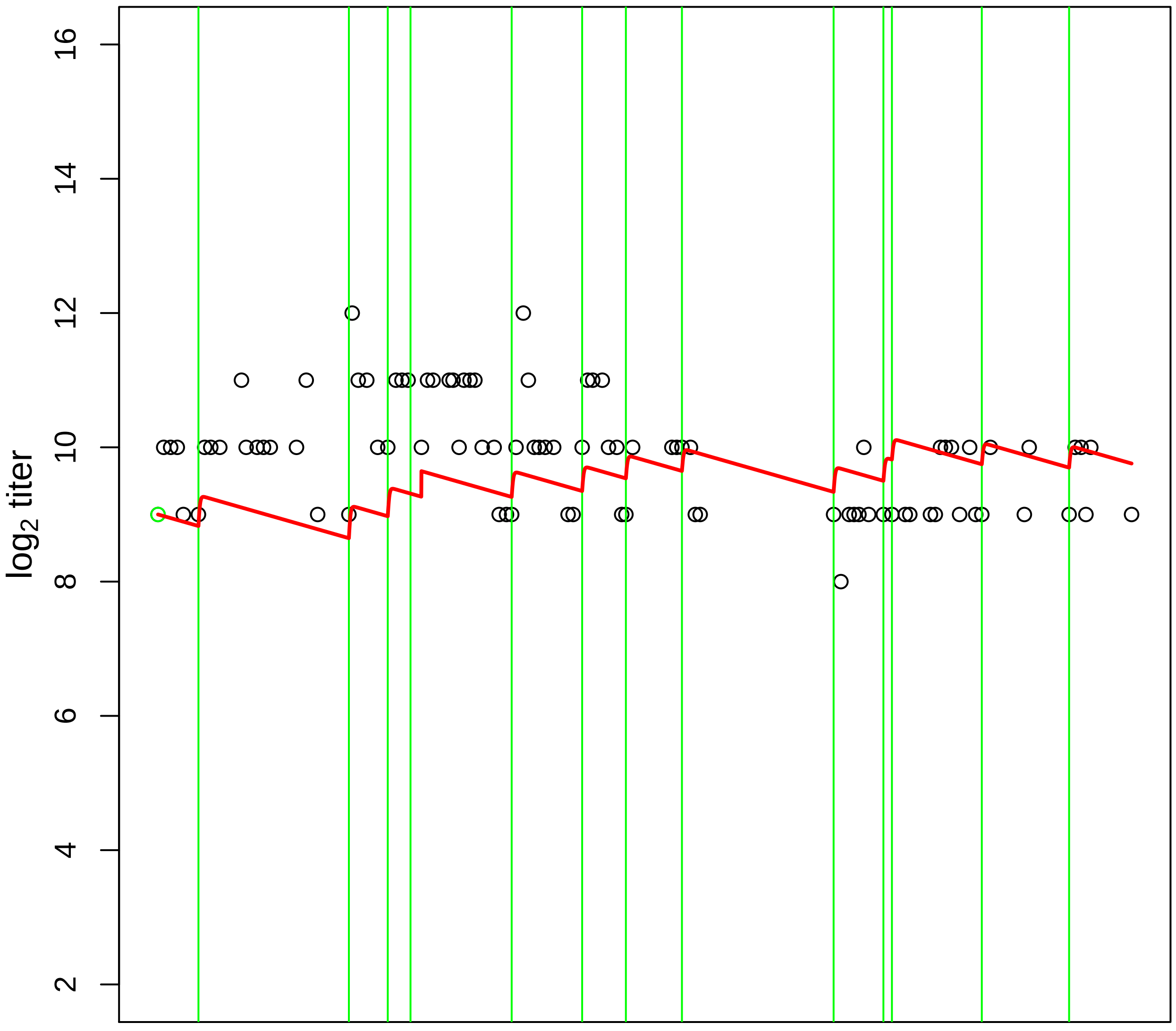

time in years from first donation of donor 601
mean absolute errors = 0.823 , mean squared errors = 1.021

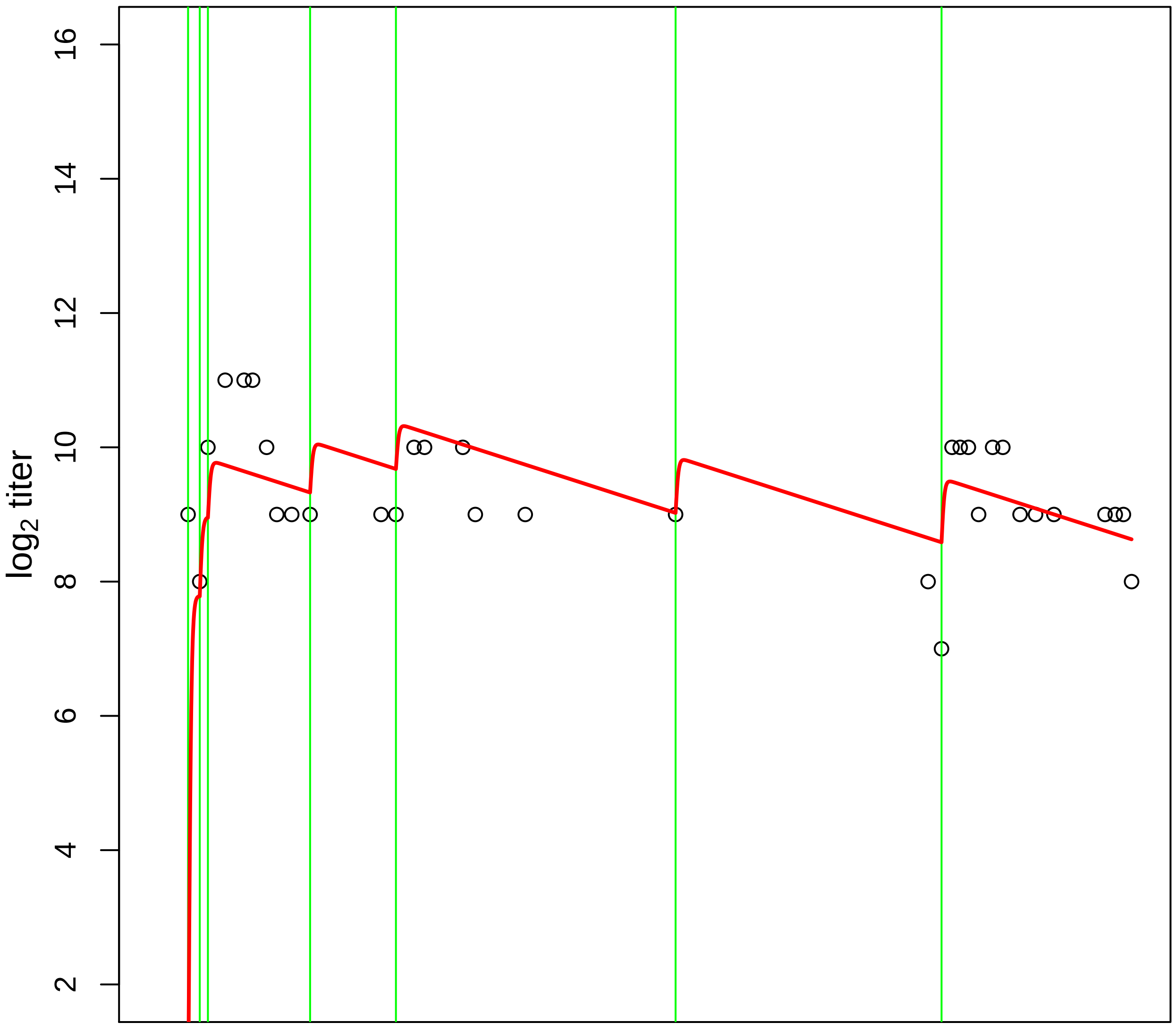

time in years from first donation of donor 602
mean absolute errors = 0.827 , mean squared errors = 3.009

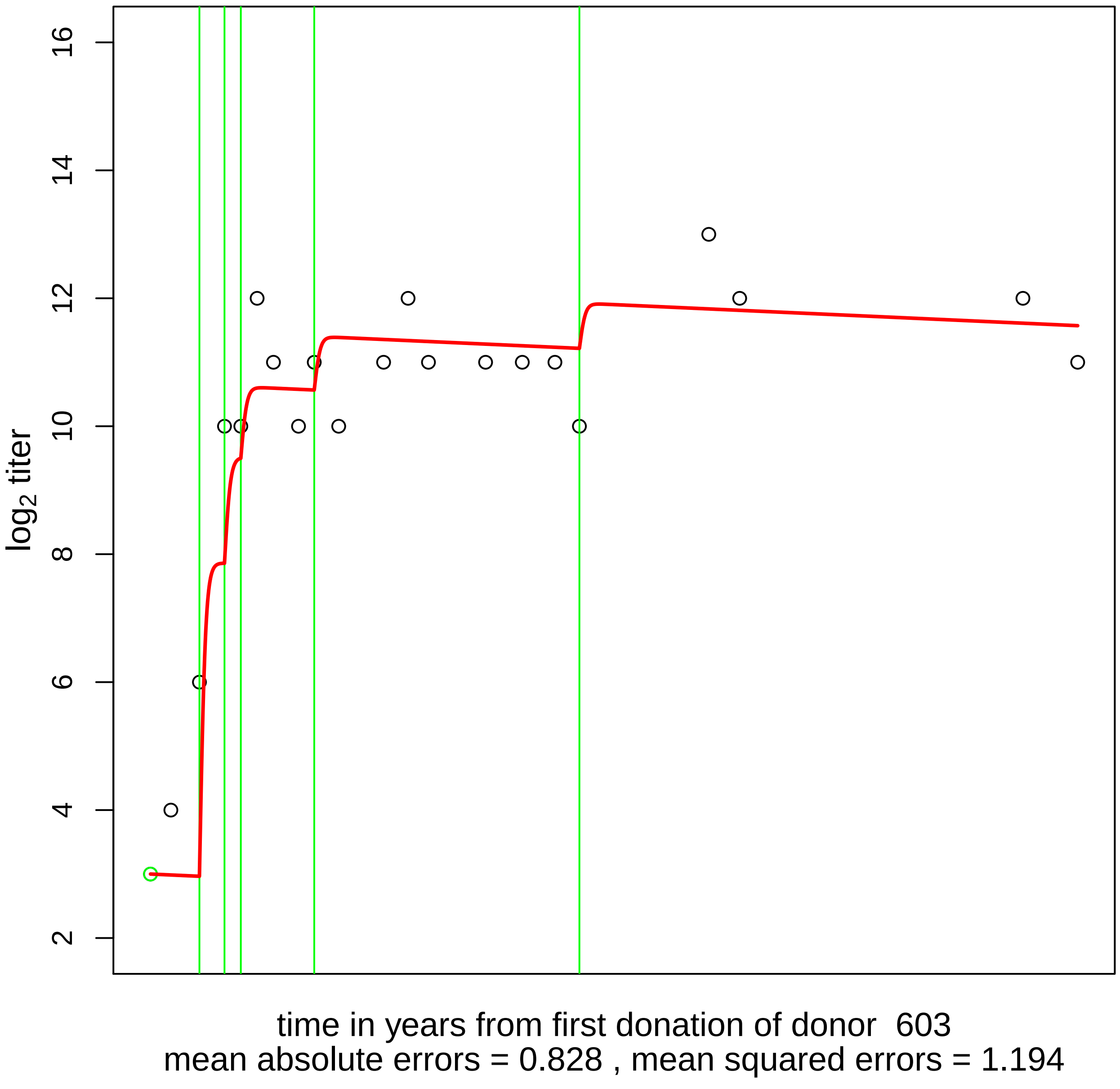

16
14
12
10
8
6
4
2
$\log_2$ titer
time in years from first donation of donor 603
mean absolute errors = 0.828 , mean squared errors = 1.194

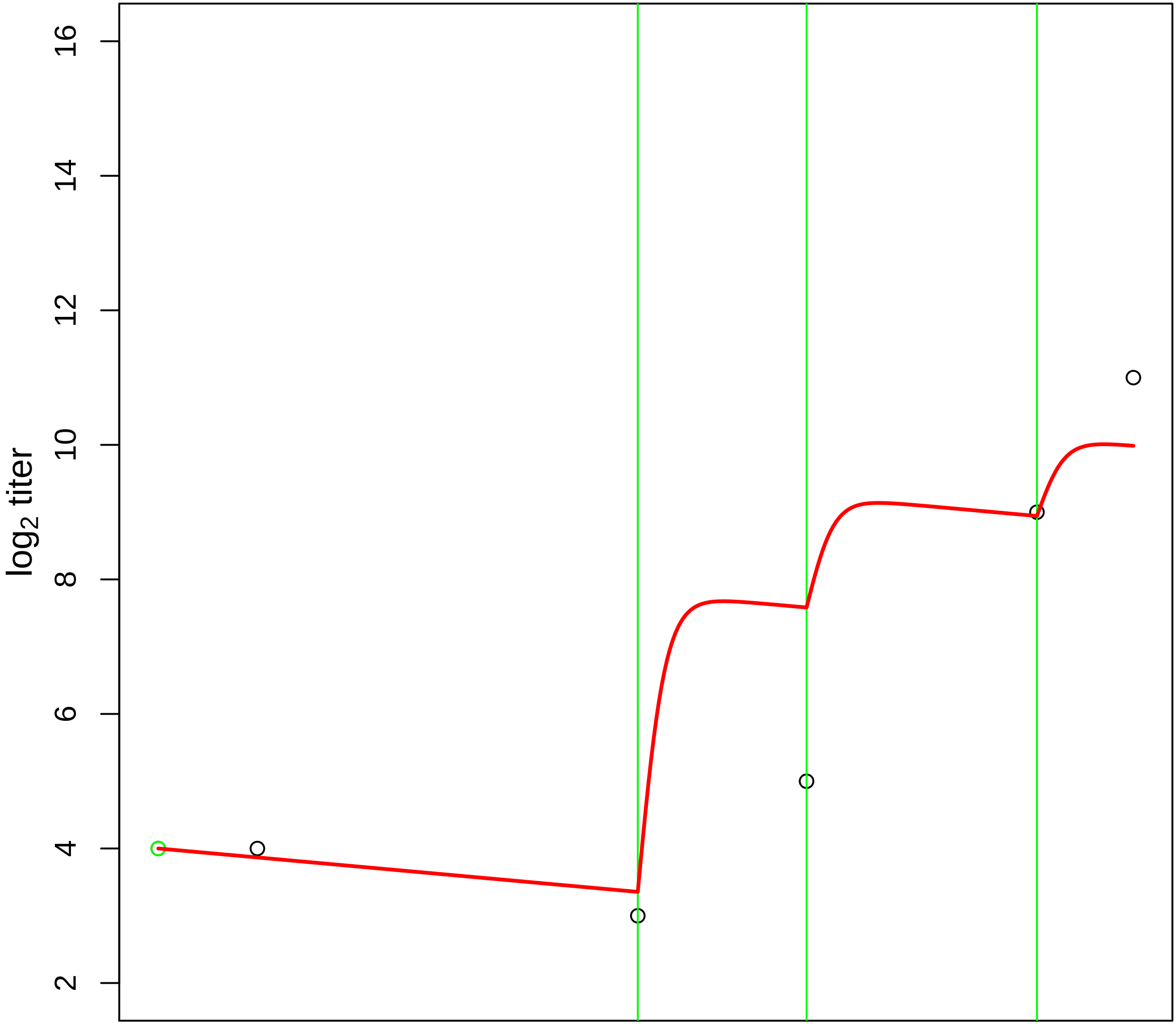

time in years from first donation of donor 604
mean absolute errors = 0.828 , mean squared errors = 1.569

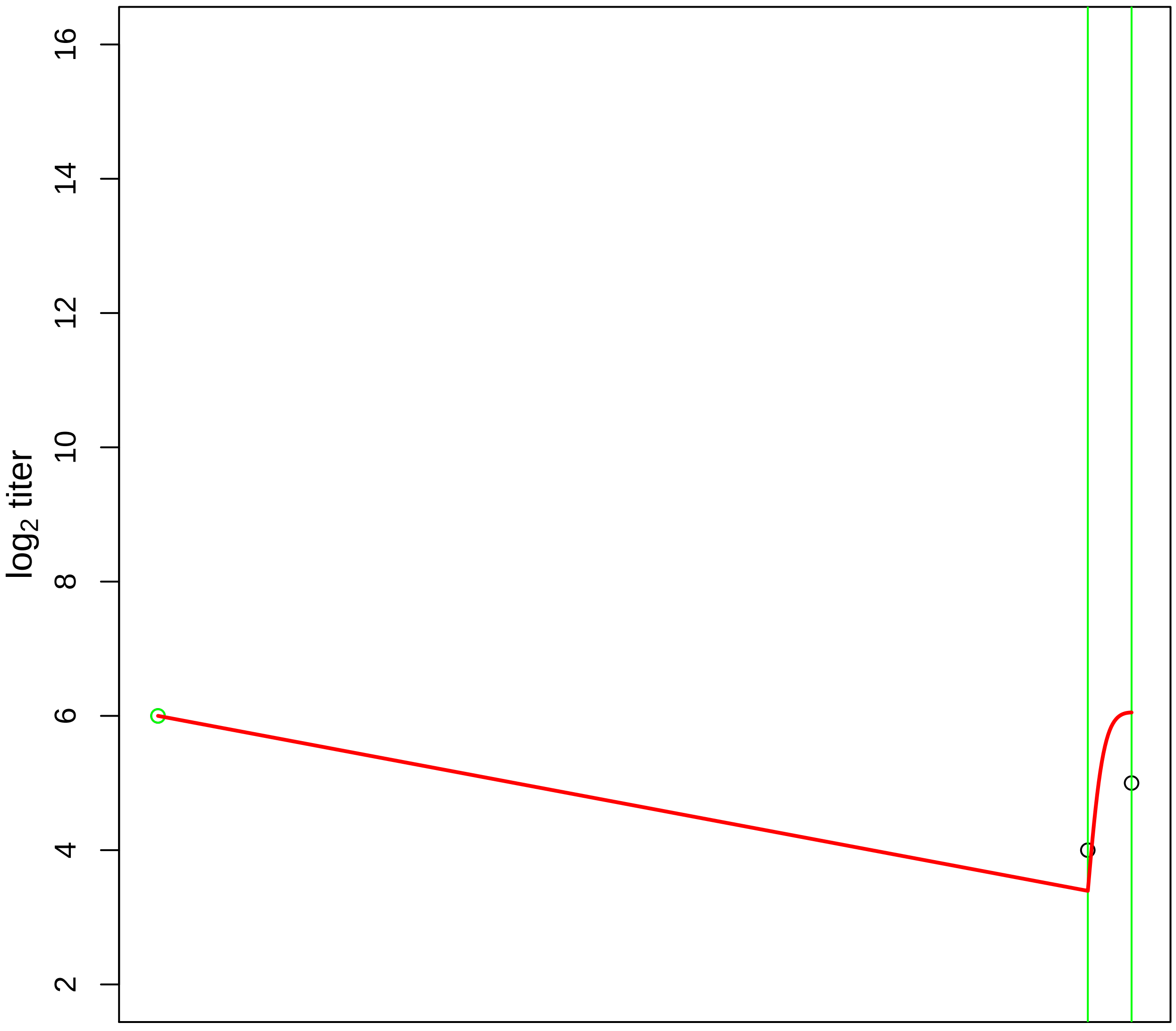

time in years from first donation of donor 605
mean absolute errors = 0.829 , mean squared errors = 0.736

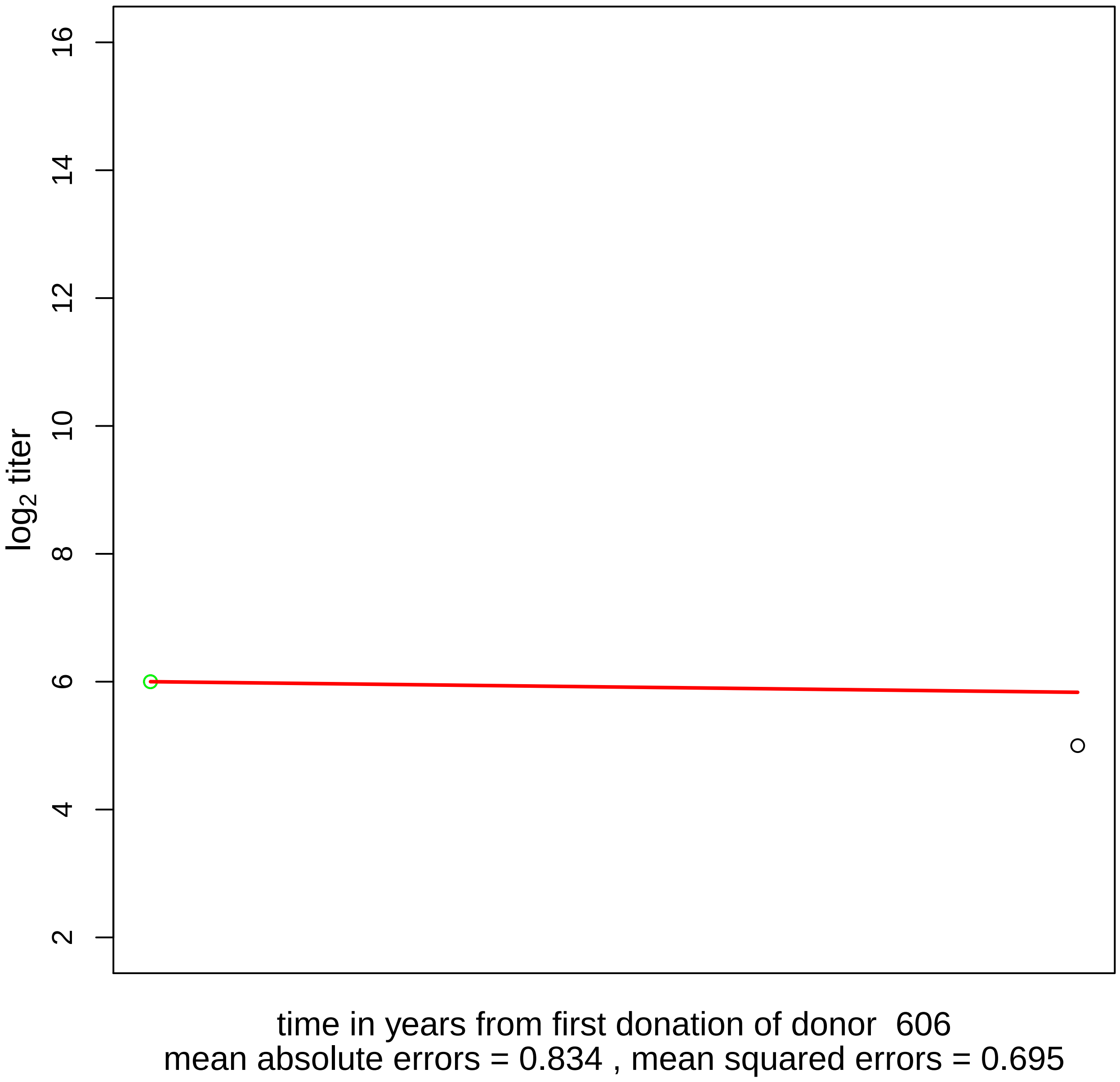

16
14
12
10
8
6
4
2
log2 titer
time in years from first donation of donor 606
mean absolute errors = 0.834 , mean squared errors = 0.695

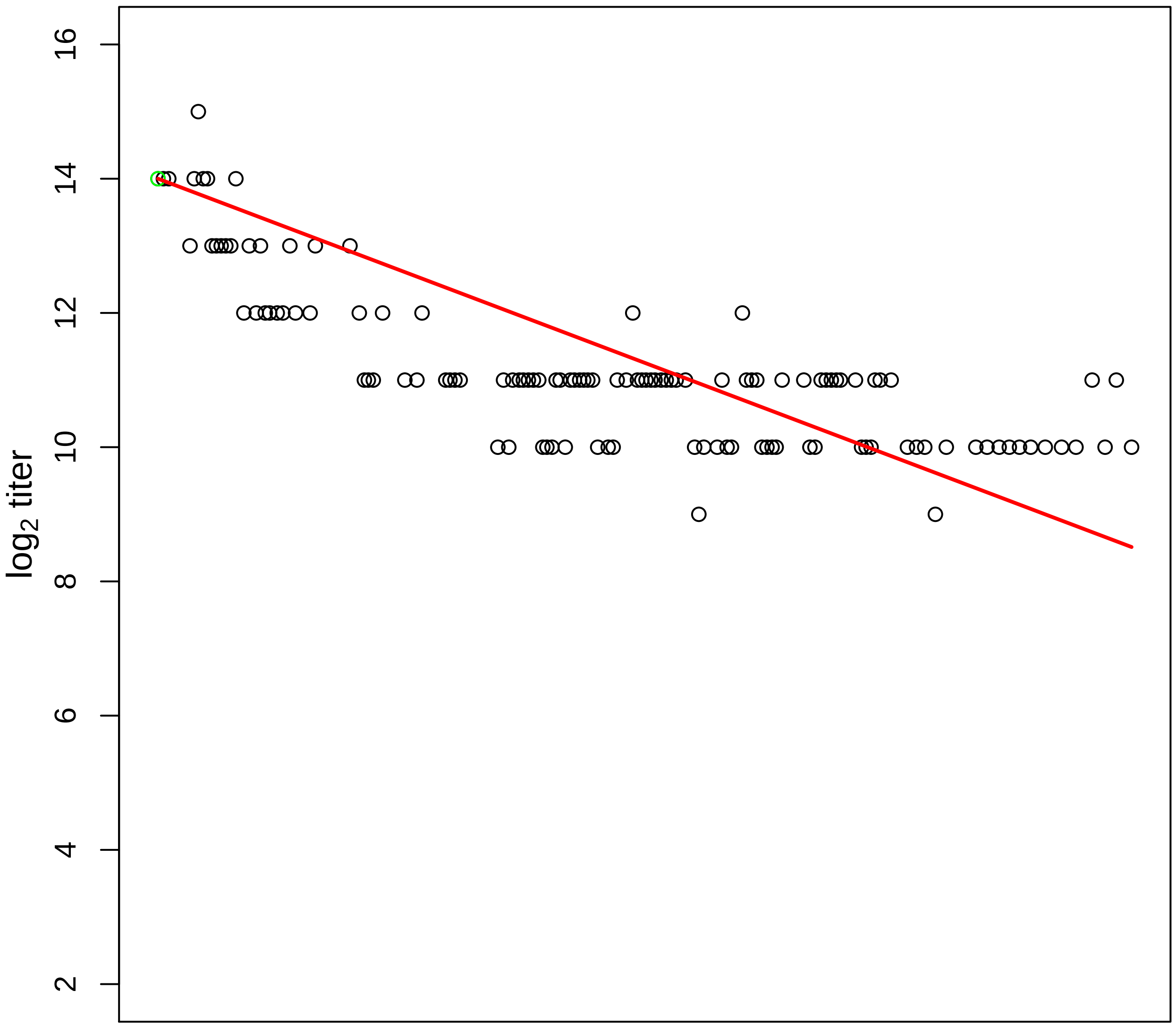

time in years from first donation of donor 607
mean absolute errors = 0.835 , mean squared errors = 0.998

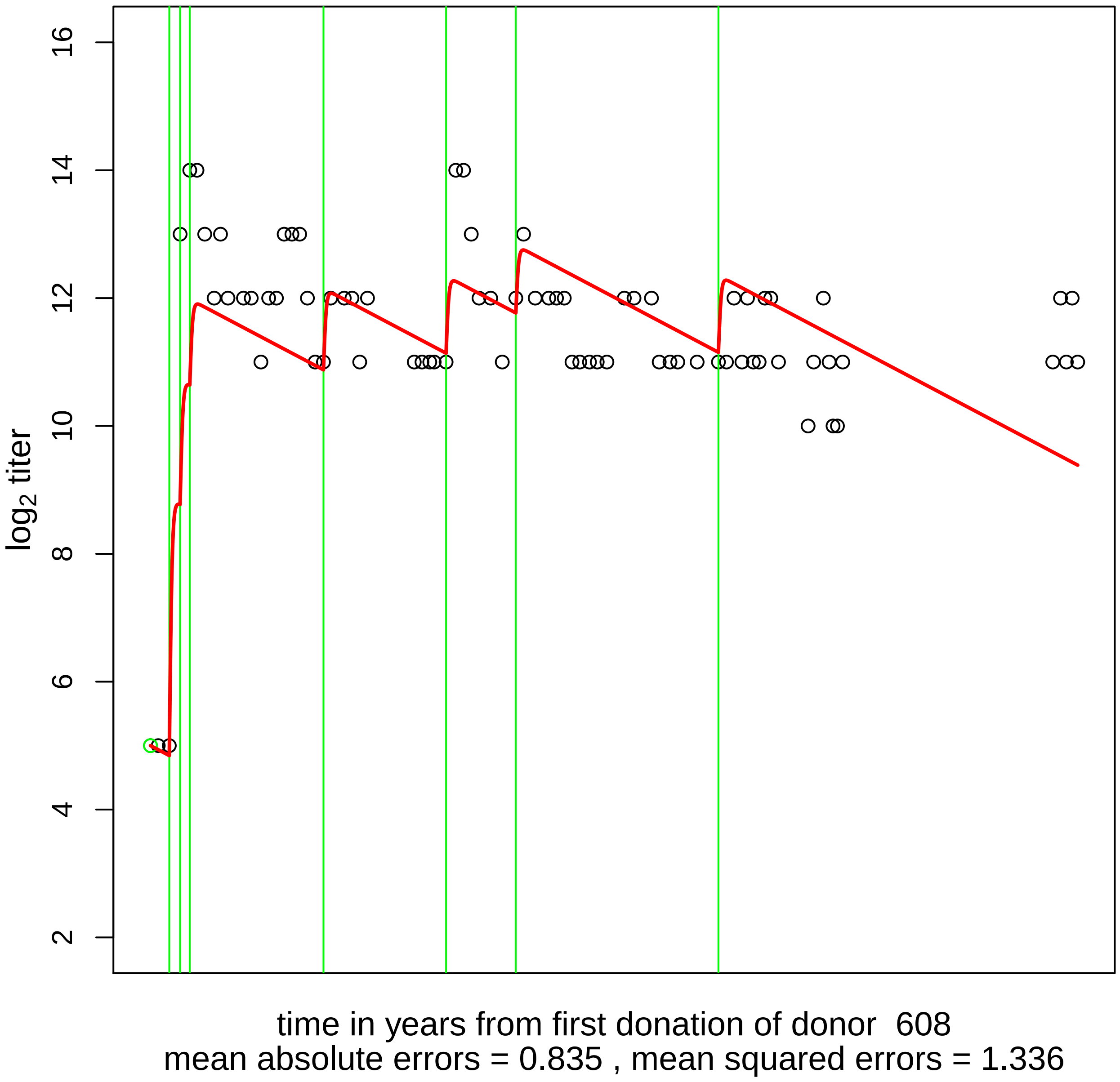

log2 titer
2
4
6
8
10
12
14
16
time in years from first donation of donor 608
mean absolute errors = 0.835 , mean squared errors = 1.336

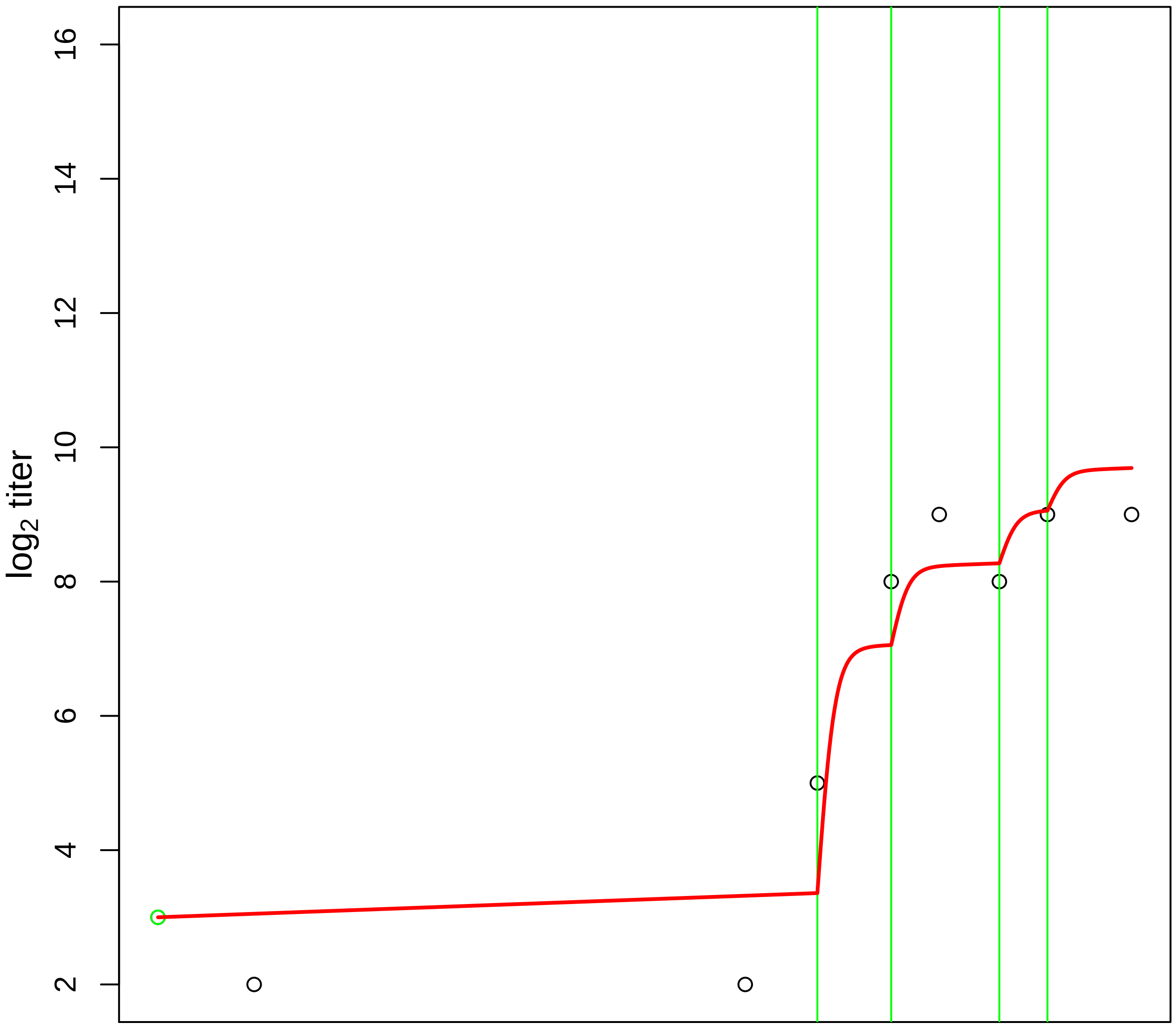

time in years from first donation of donor 609
mean absolute errors = 0.844 , mean squared errors = 0.948

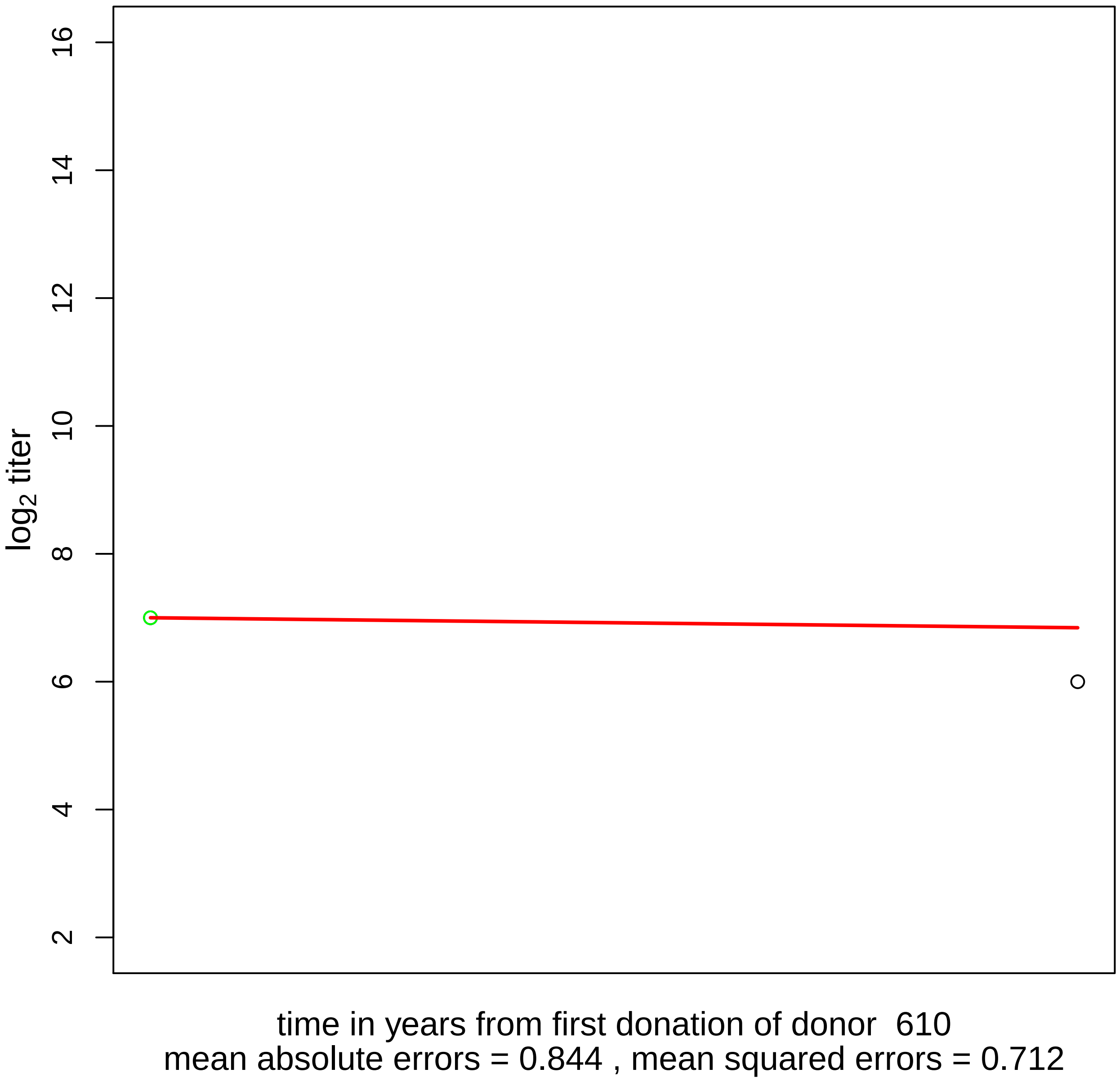

log2 titer
2
4
6
8
10
12
14
16
time in years from first donation of donor 610
mean absolute errors = 0.844 , mean squared errors = 0.712

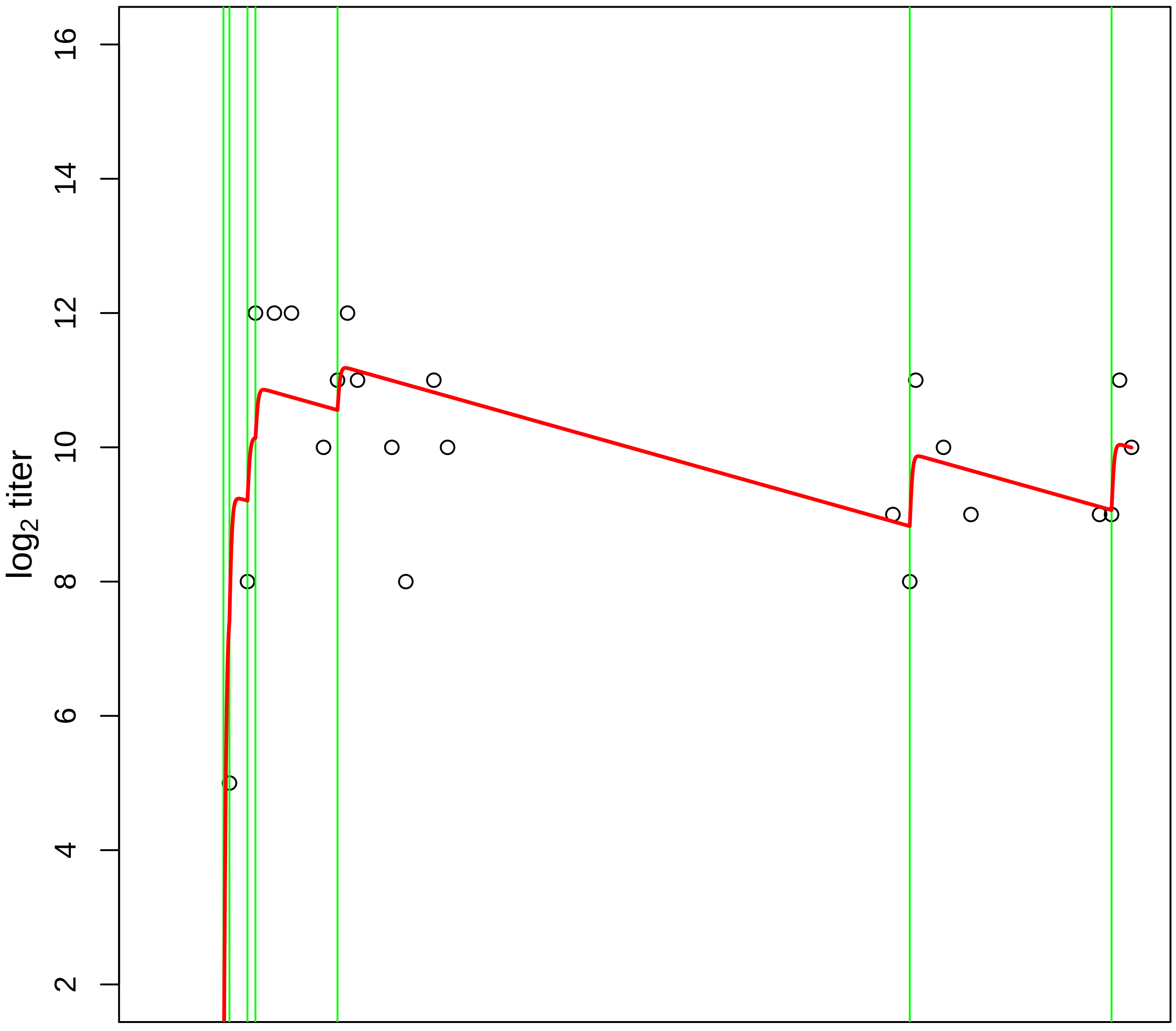

time in years from first donation of donor 611
mean absolute errors = 0.844 , mean squared errors = 1.261

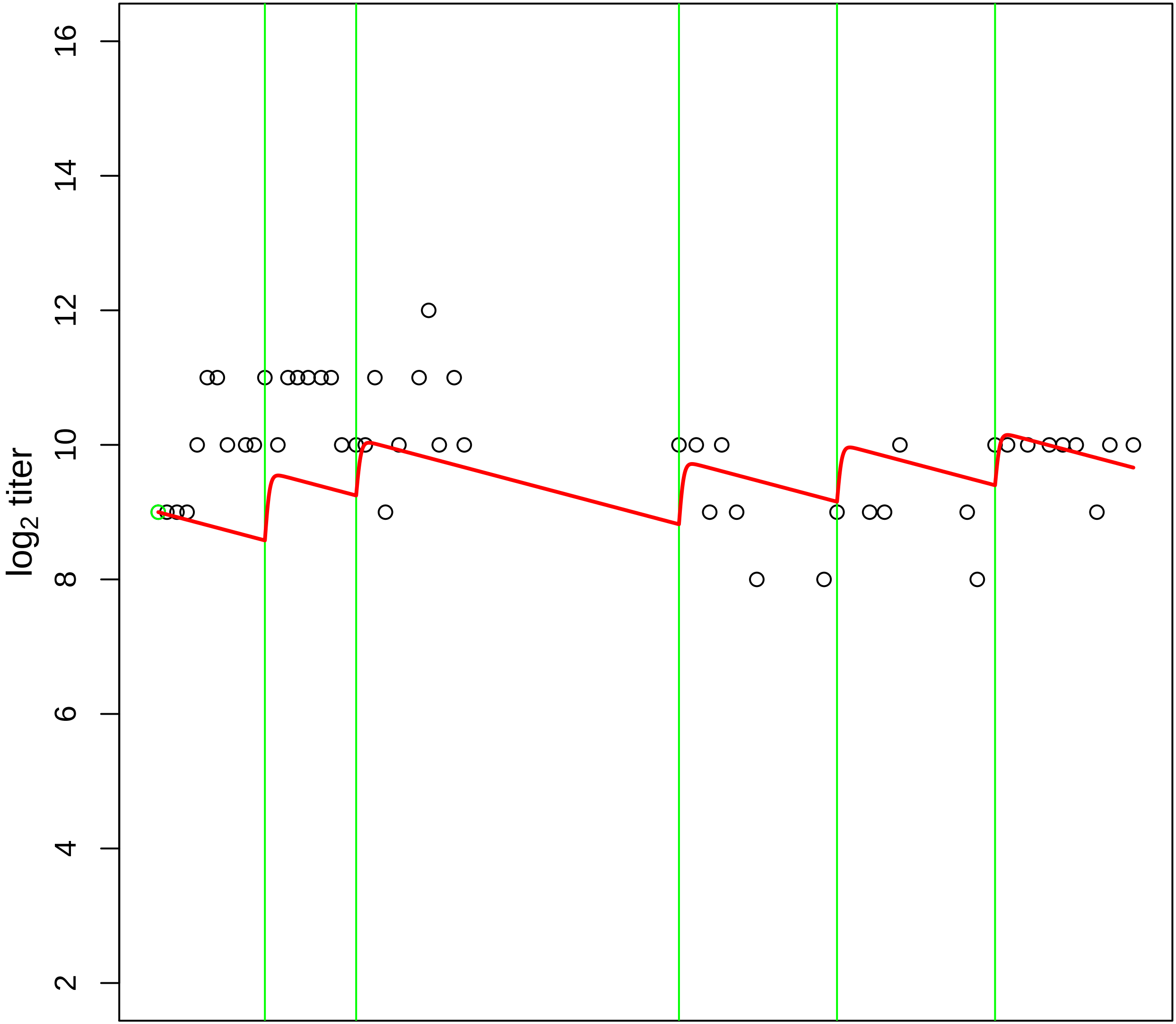

time in years from first donation of donor 612
mean absolute errors = 0.846 , mean squared errors = 1.163

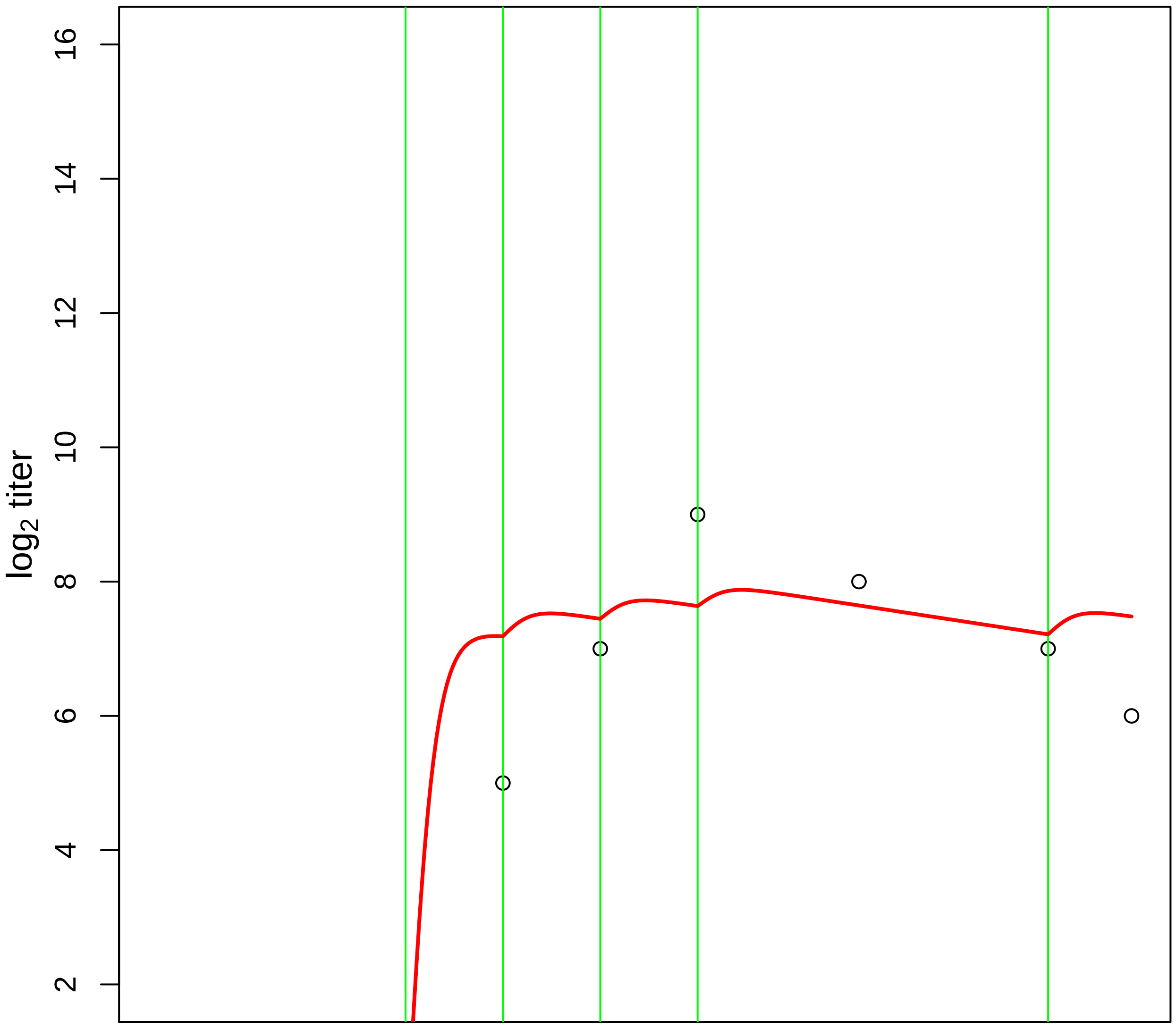

time in years from first donation of donor 613
mean absolute errors = 0.848 , mean squared errors = 1.193

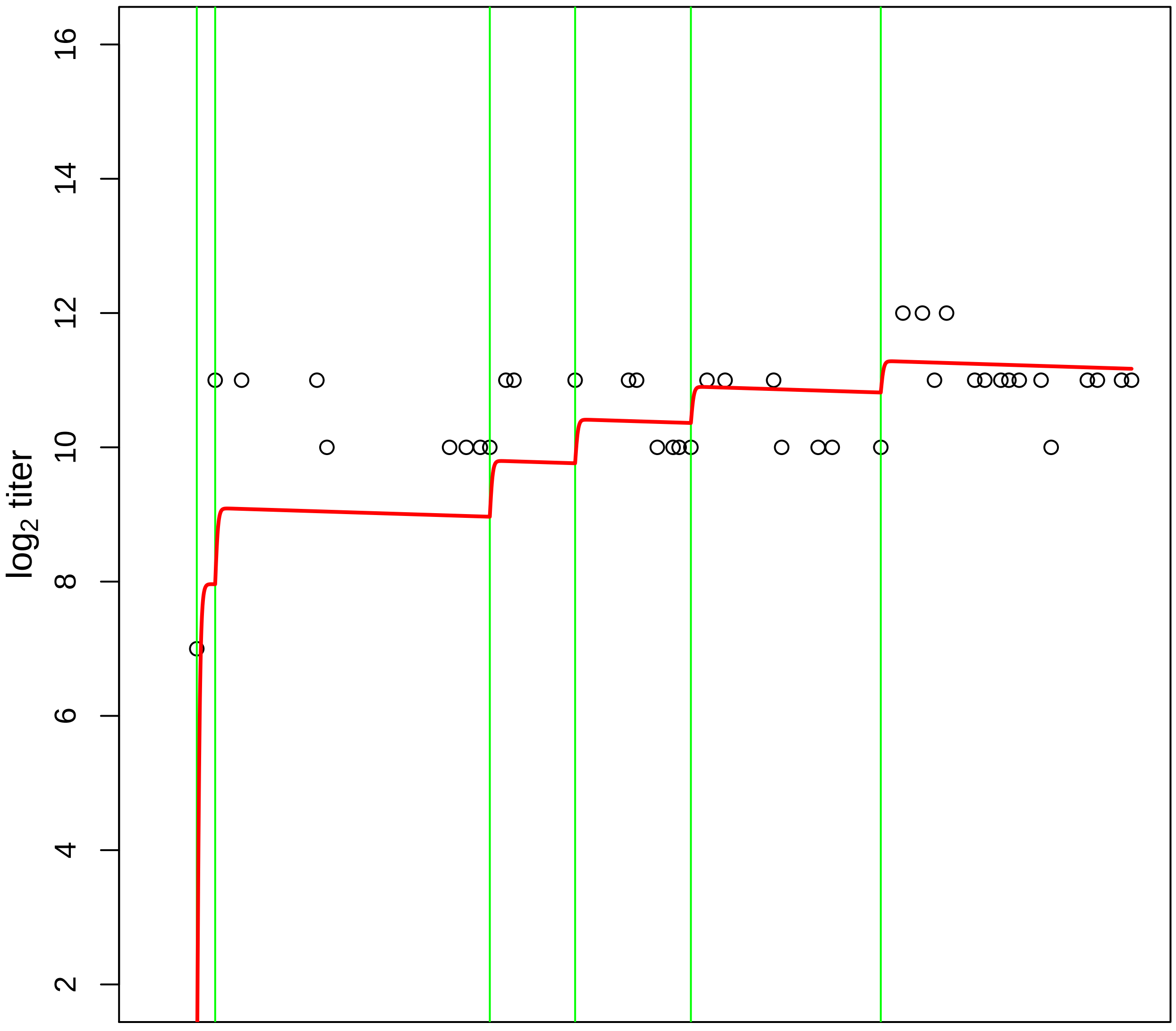

time in years from first donation of donor 614
mean absolute errors = 0.849 , mean squared errors = 2.032

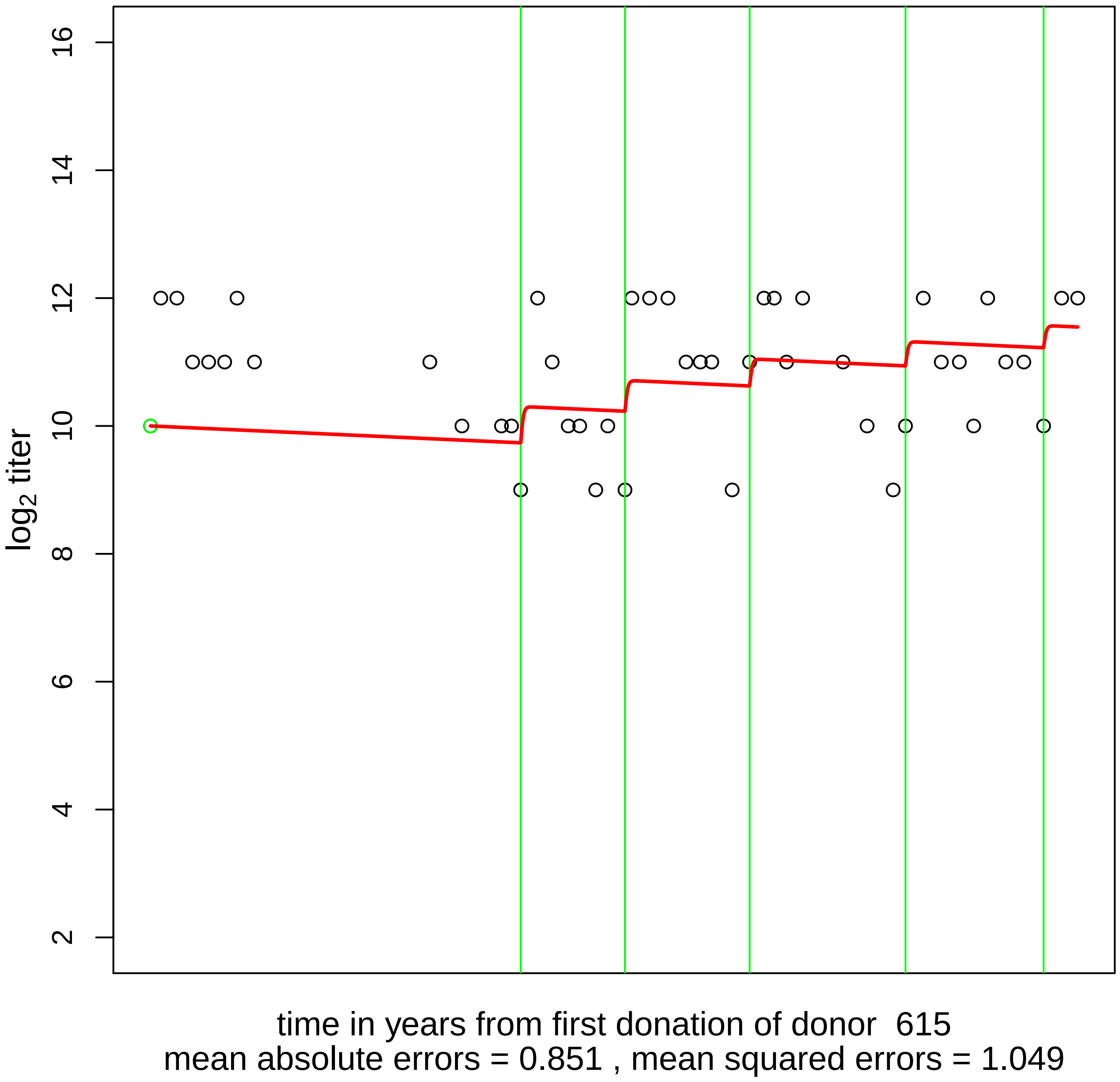
16
14
12
10
8
6
4
2
$\log_2$ titer
time in years from first donation of donor 615
mean absolute errors = 0.851 , mean squared errors = 1.049

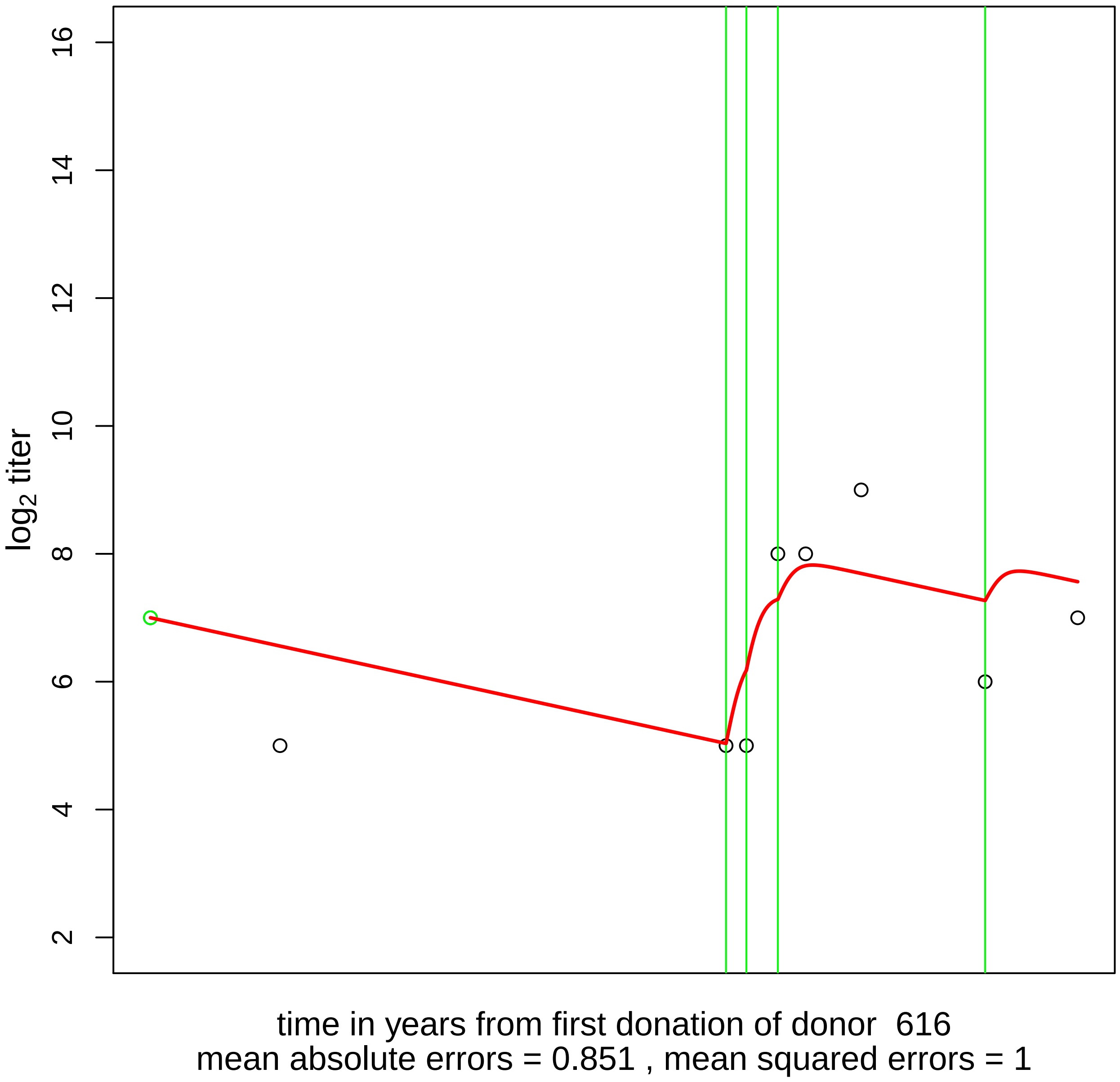

log2 titer
2
4
6
8
10
12
14
16
time in years from first donation of donor 616
mean absolute errors = 0.851 , mean squared errors = 1

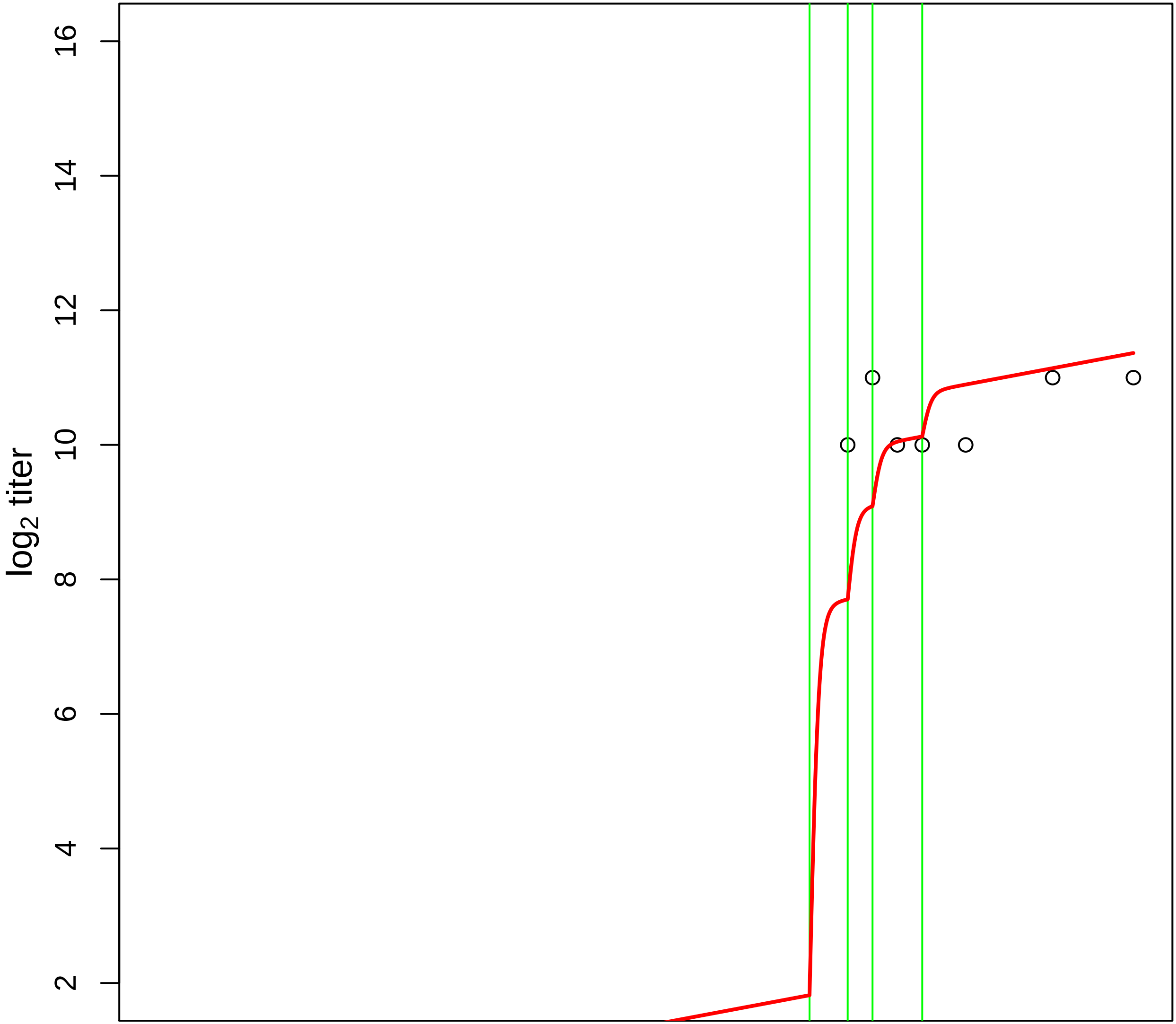

time in years from first donation of donor 617
mean absolute errors = 0.852 , mean squared errors = 1.469

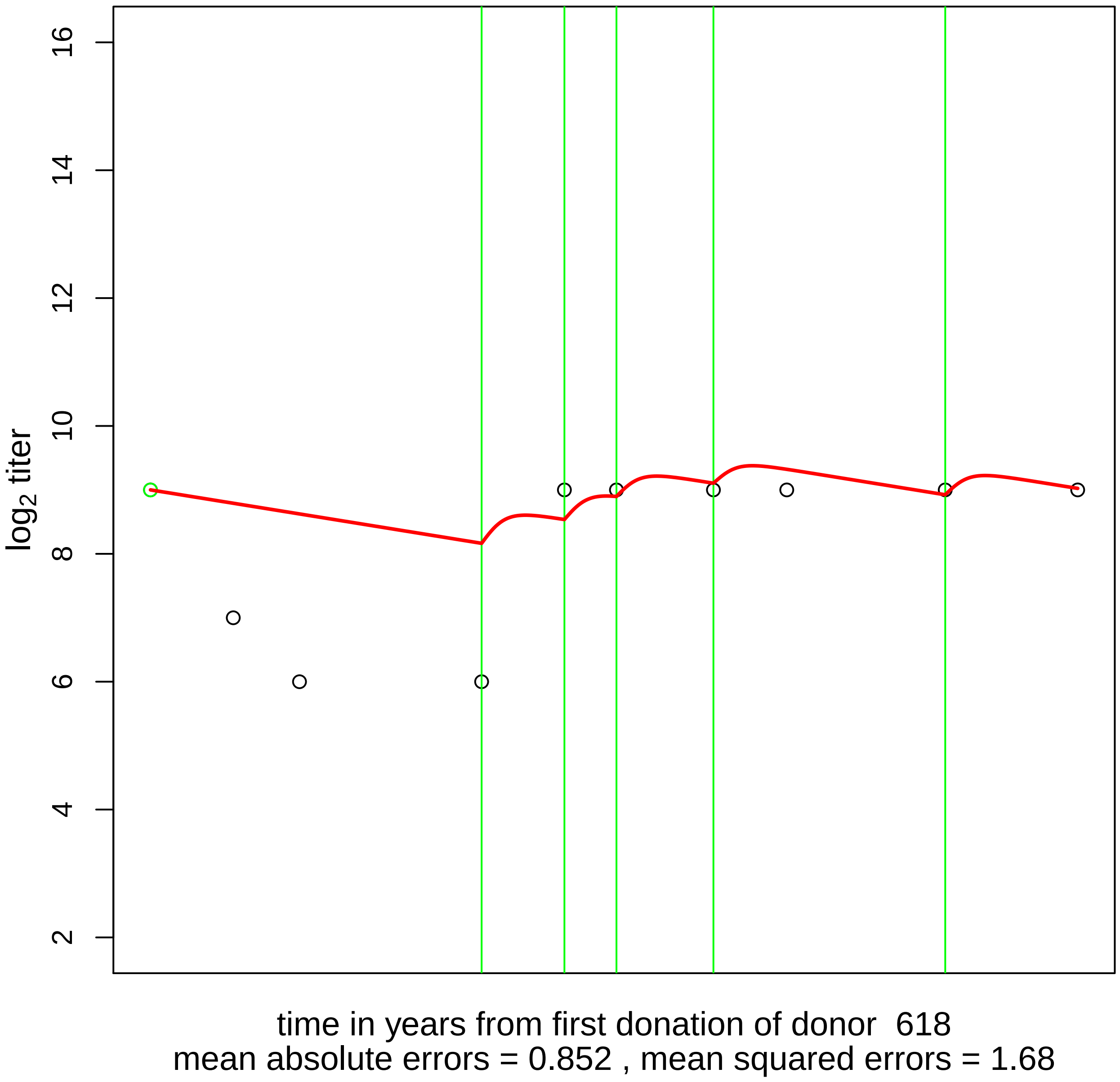

16
14
12
10
8
6
4
2
log2 titer
time in years from first donation of donor 618
mean absolute errors = 0.852 , mean squared errors = 1.68

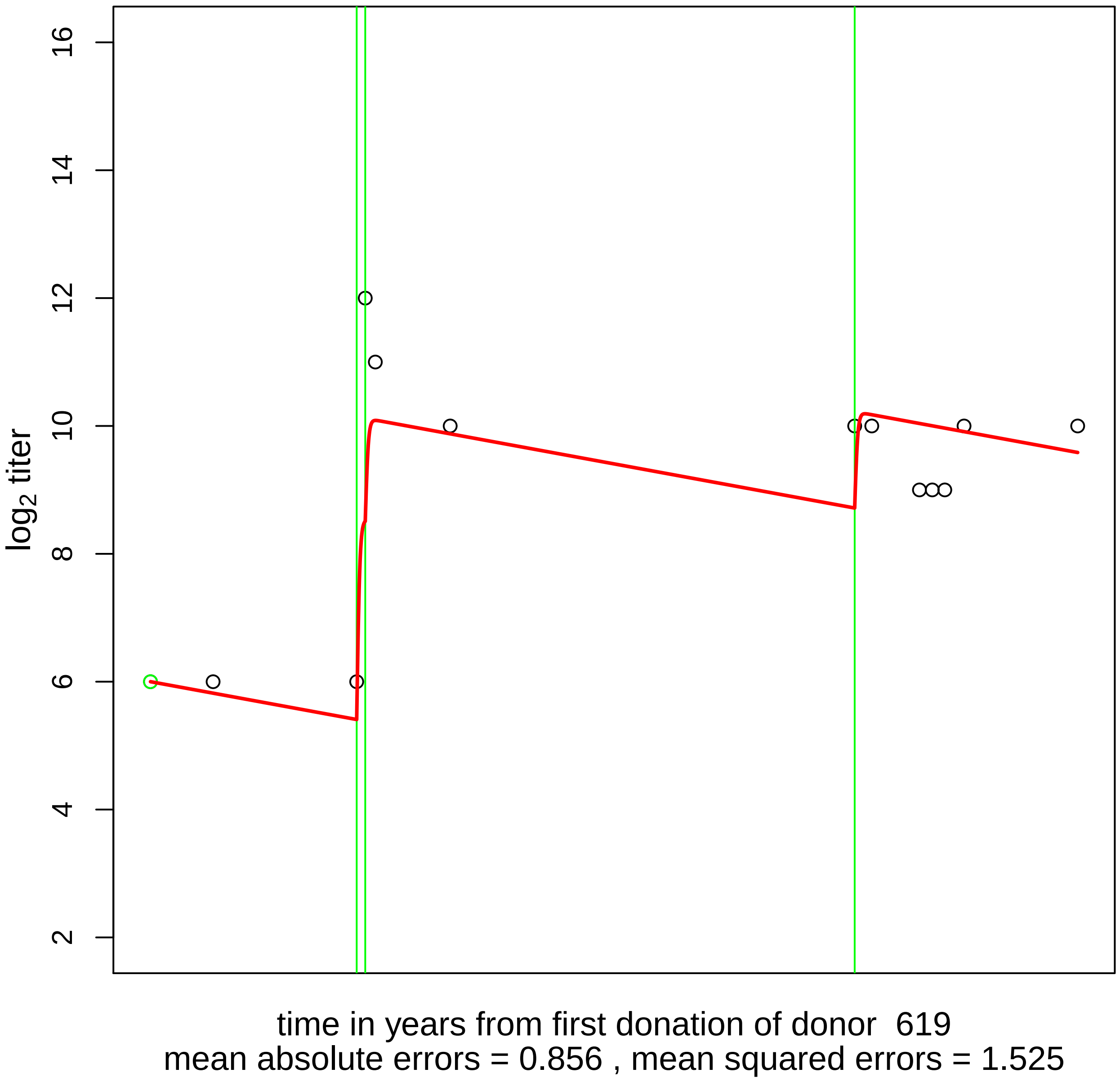

log2 titer
2
4
6
8
10
12
14
16
time in years from first donation of donor 619
mean absolute errors = 0.856 , mean squared errors = 1.525

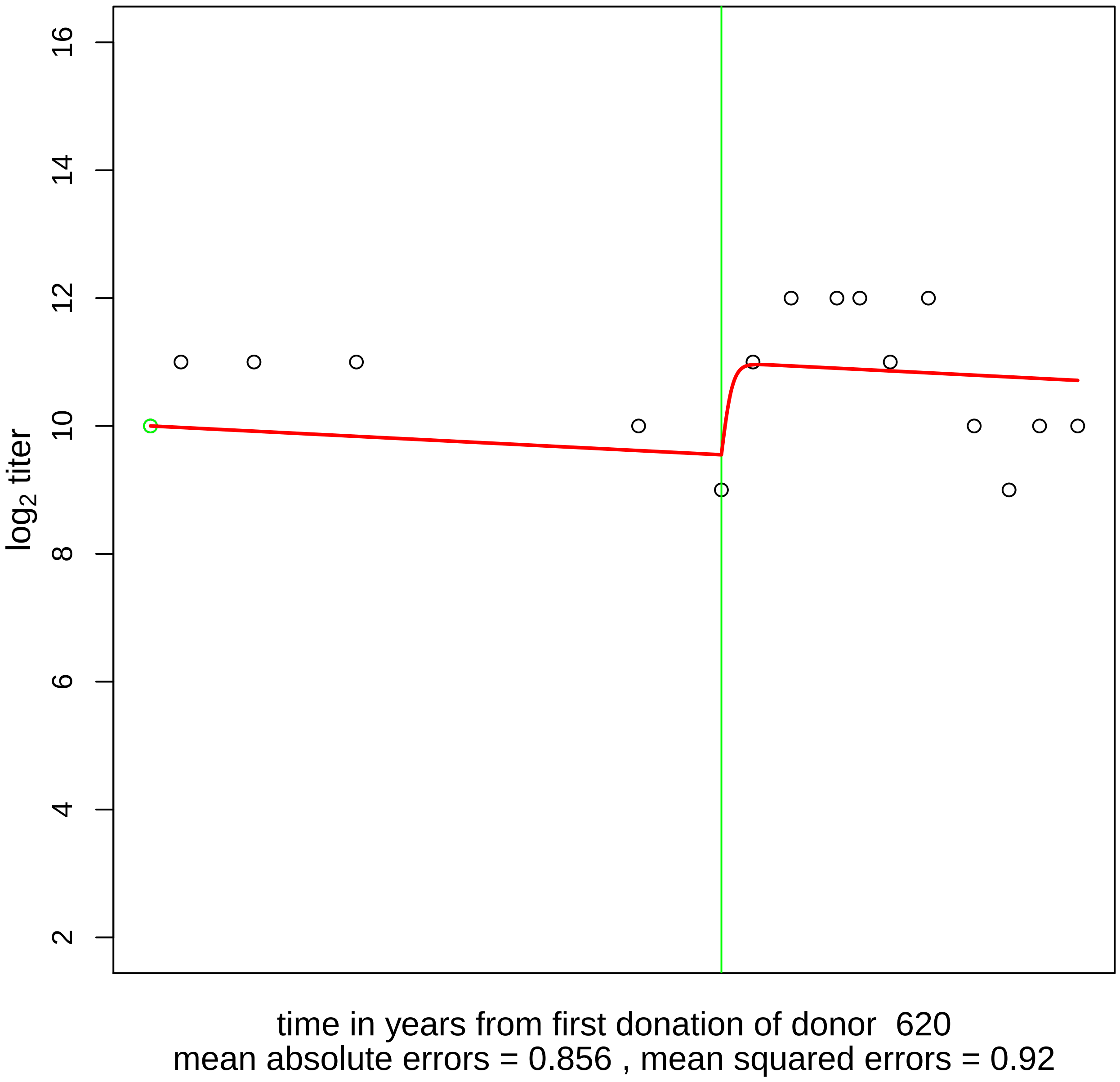

16
14
12
10
8
6
4
2
log2 titer
time in years from first donation of donor 620
mean absolute errors = 0.856 , mean squared errors = 0.92

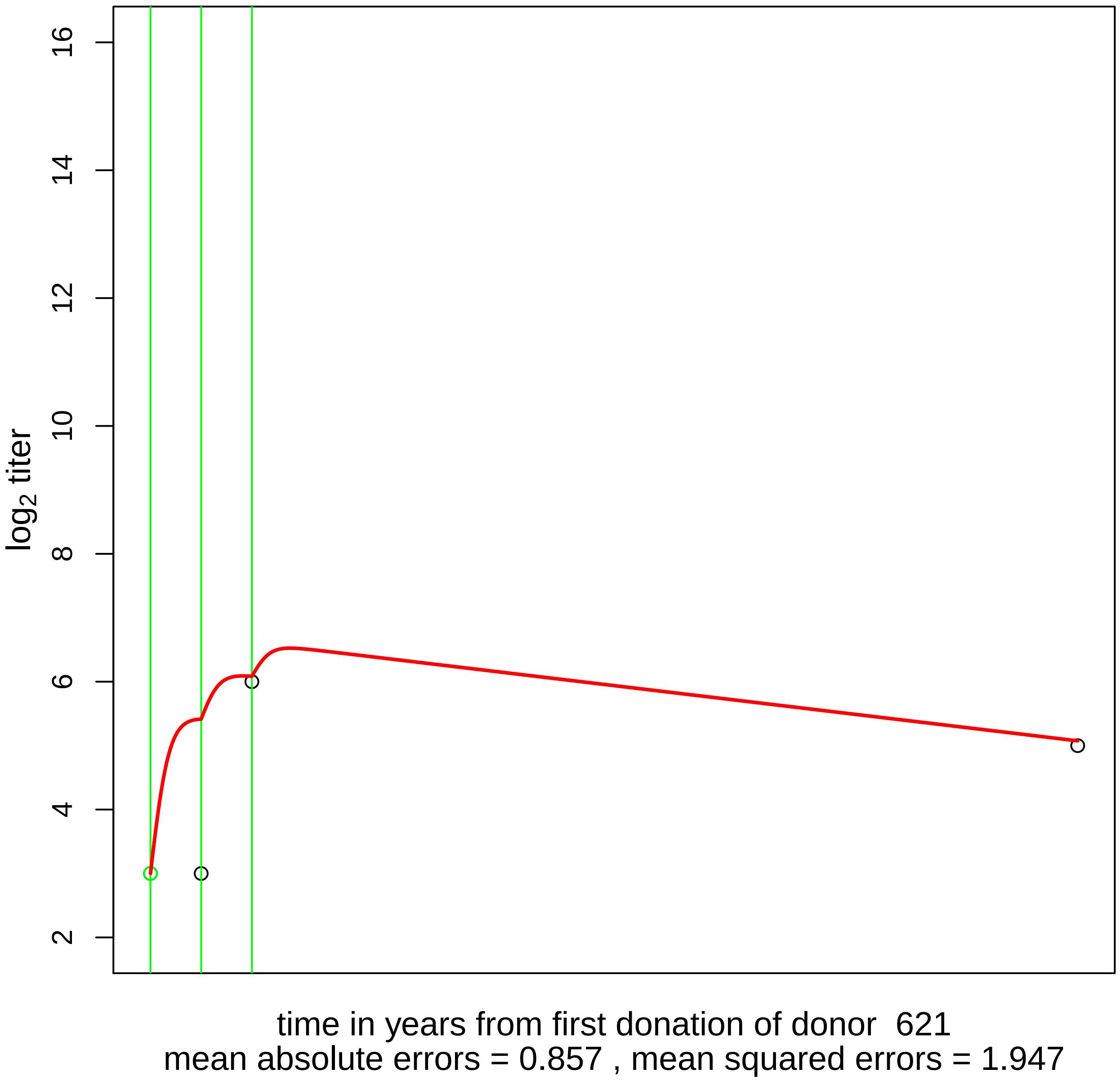

16
14
12
10
8
6
4
2
log2 titer
time in years from first donation of donor 621
mean absolute errors = 0.857 , mean squared errors = 1.947

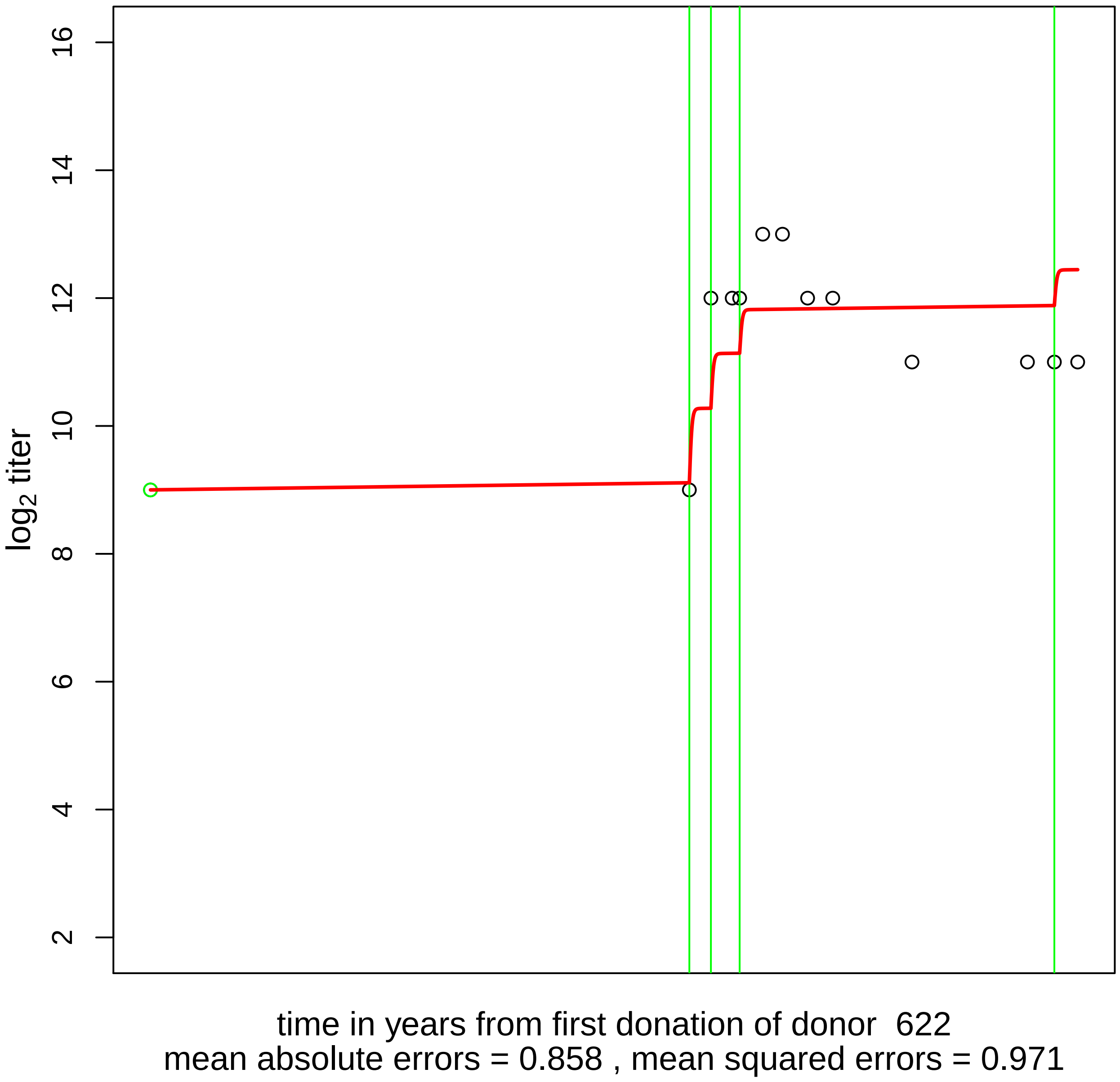
log2 titer
16
14
12
10
8
6
4
2
time in years from first donation of donor 622
mean absolute errors = 0.858 , mean squared errors = 0.971

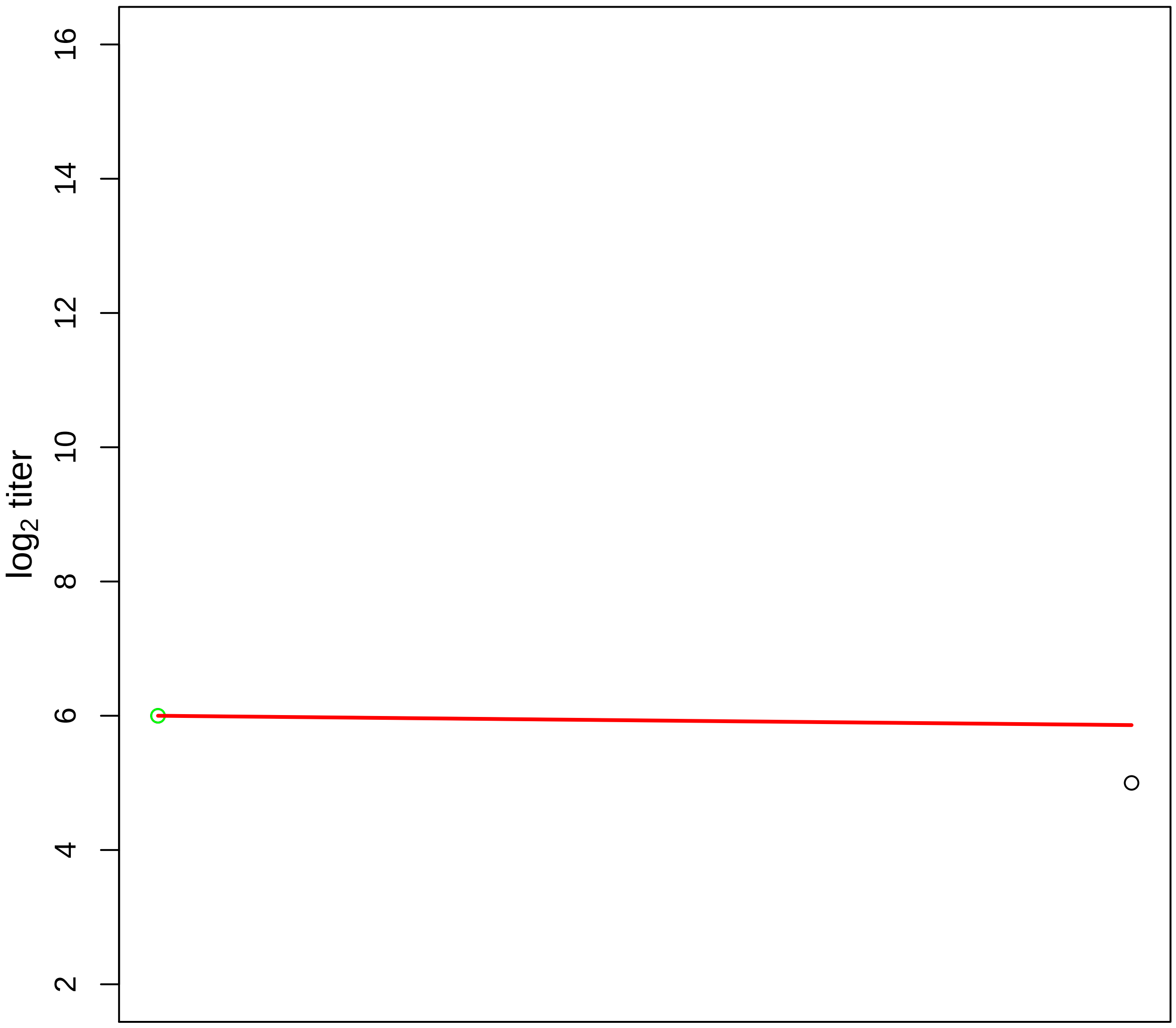

time in years from first donation of donor 623
mean absolute errors = 0.861 , mean squared errors = 0.741

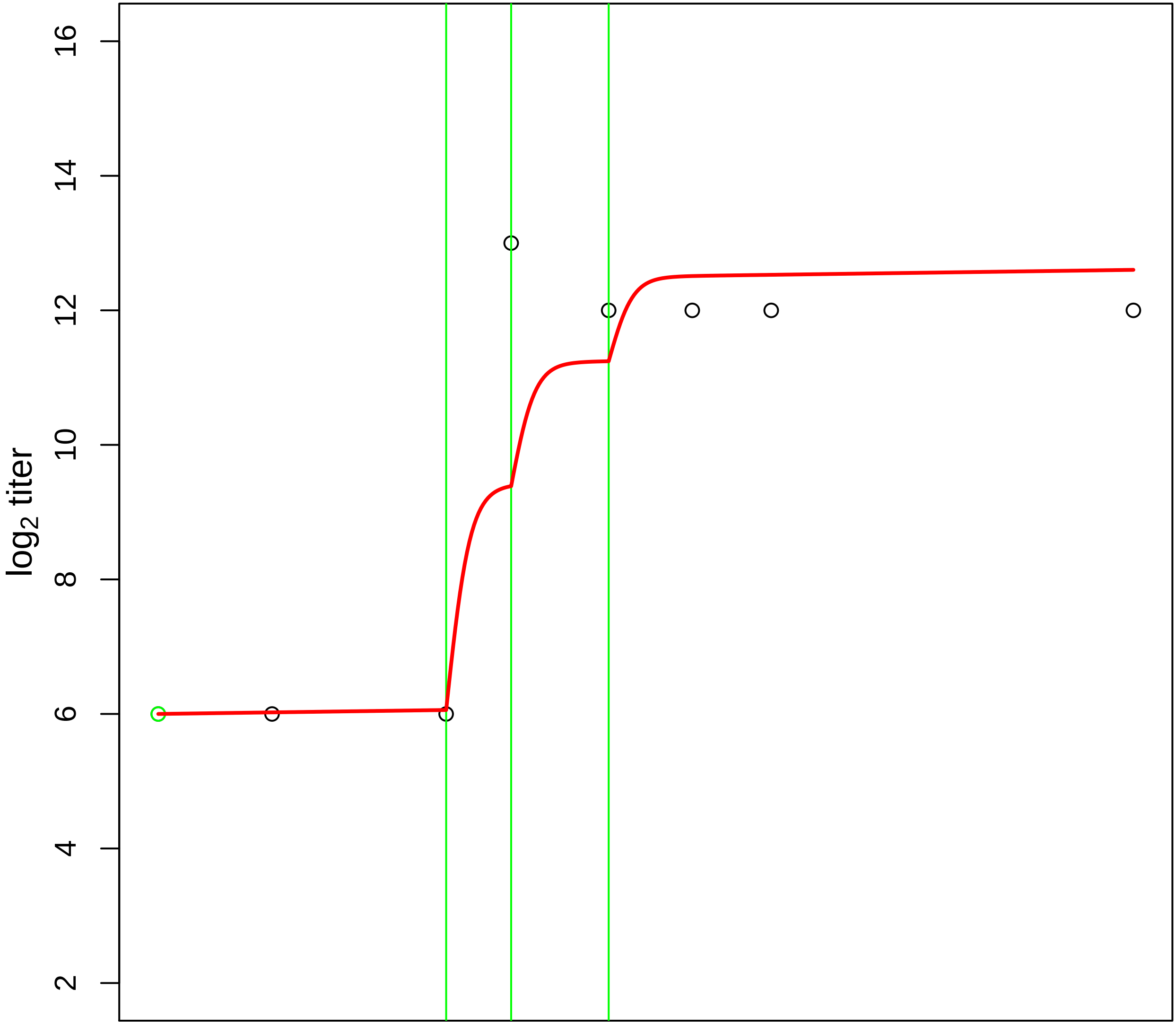

time in years from first donation of donor 624
mean absolute errors = 0.87 , mean squared errors = 2.077

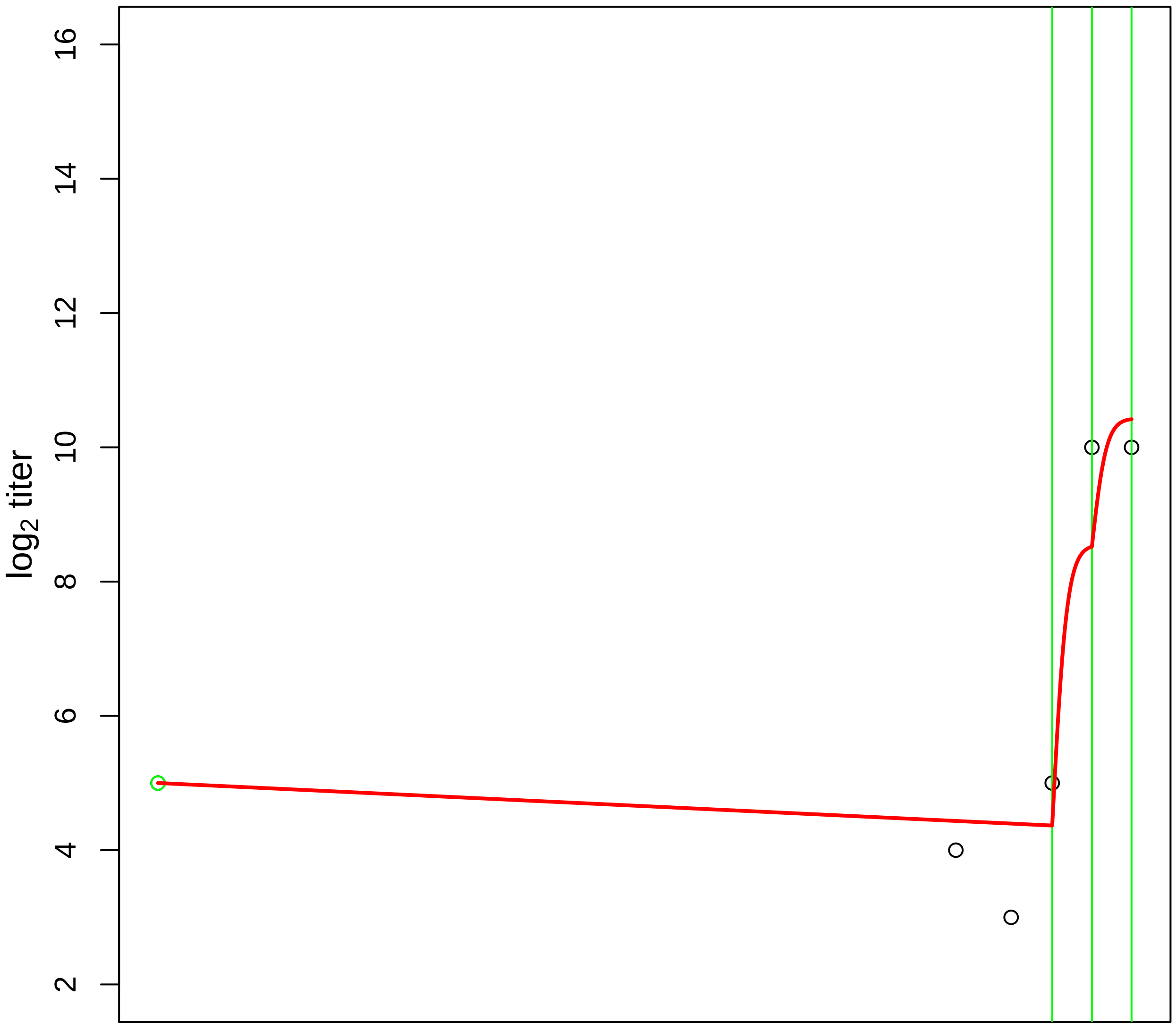

time in years from first donation of donor 625
mean absolute errors = 0.872 , mean squared errors = 0.981

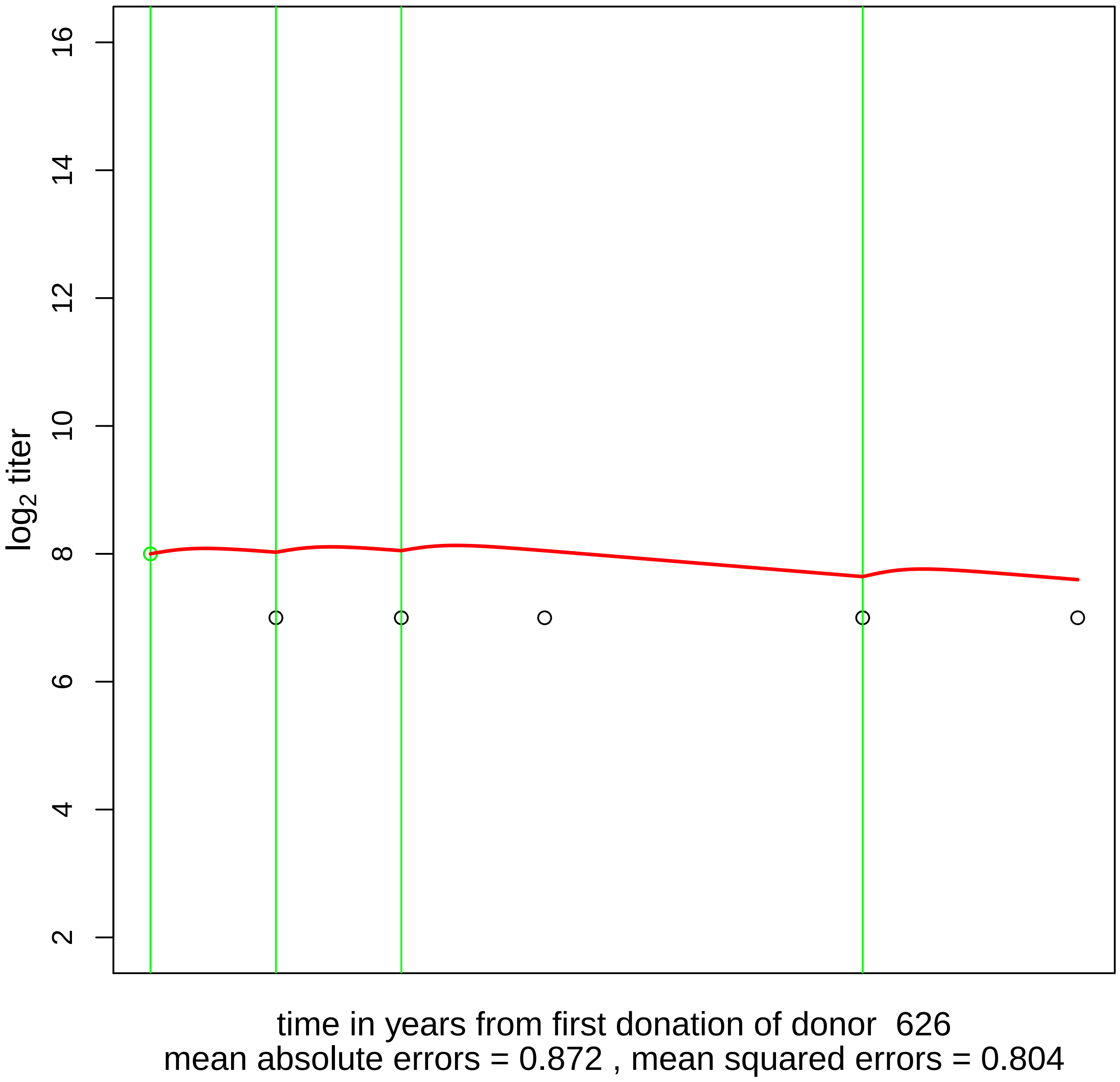

16
14
12
10
8
6
4
2
$\log_2$ titer
time in years from first donation of donor 626
mean absolute errors = 0.872 , mean squared errors = 0.804

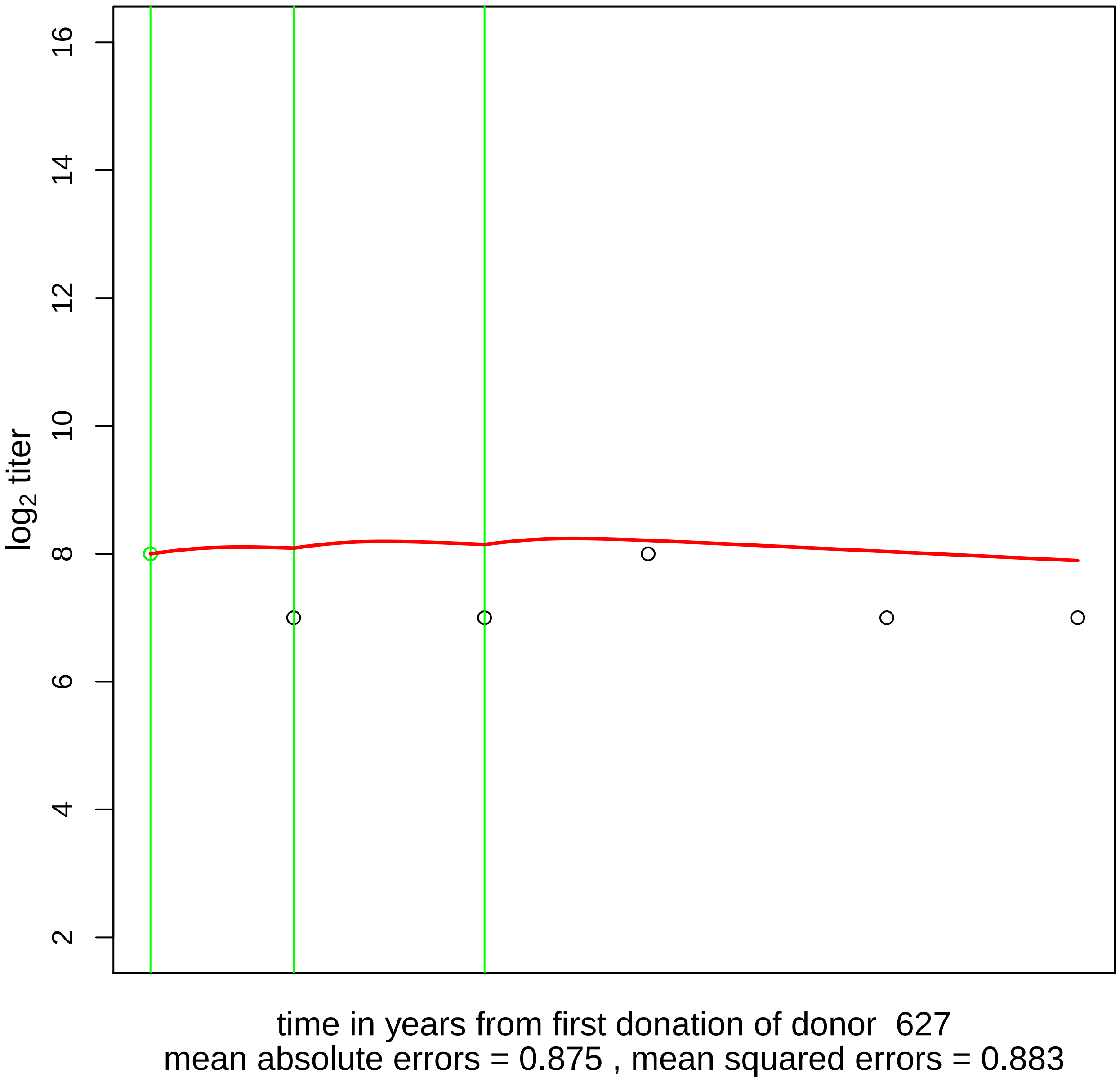

$\log_2$ titer
16
14
12
10
8
6
4
2
time in years from first donation of donor 627
mean absolute errors = 0.875 , mean squared errors = 0.883

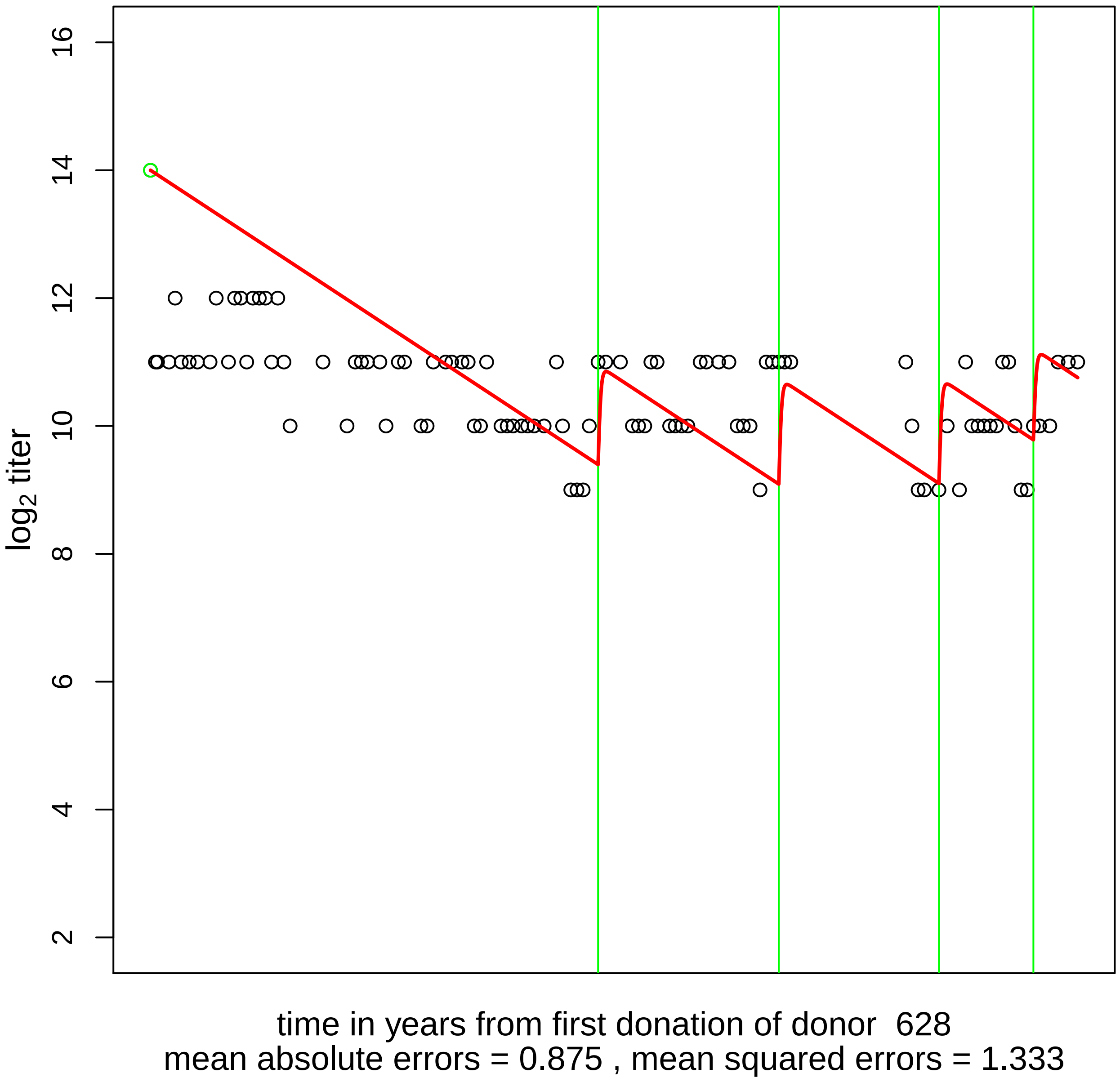

16
14
12
10
8
6
4
2
log2 titer
time in years from first donation of donor 628
mean absolute errors = 0.875 , mean squared errors = 1.333

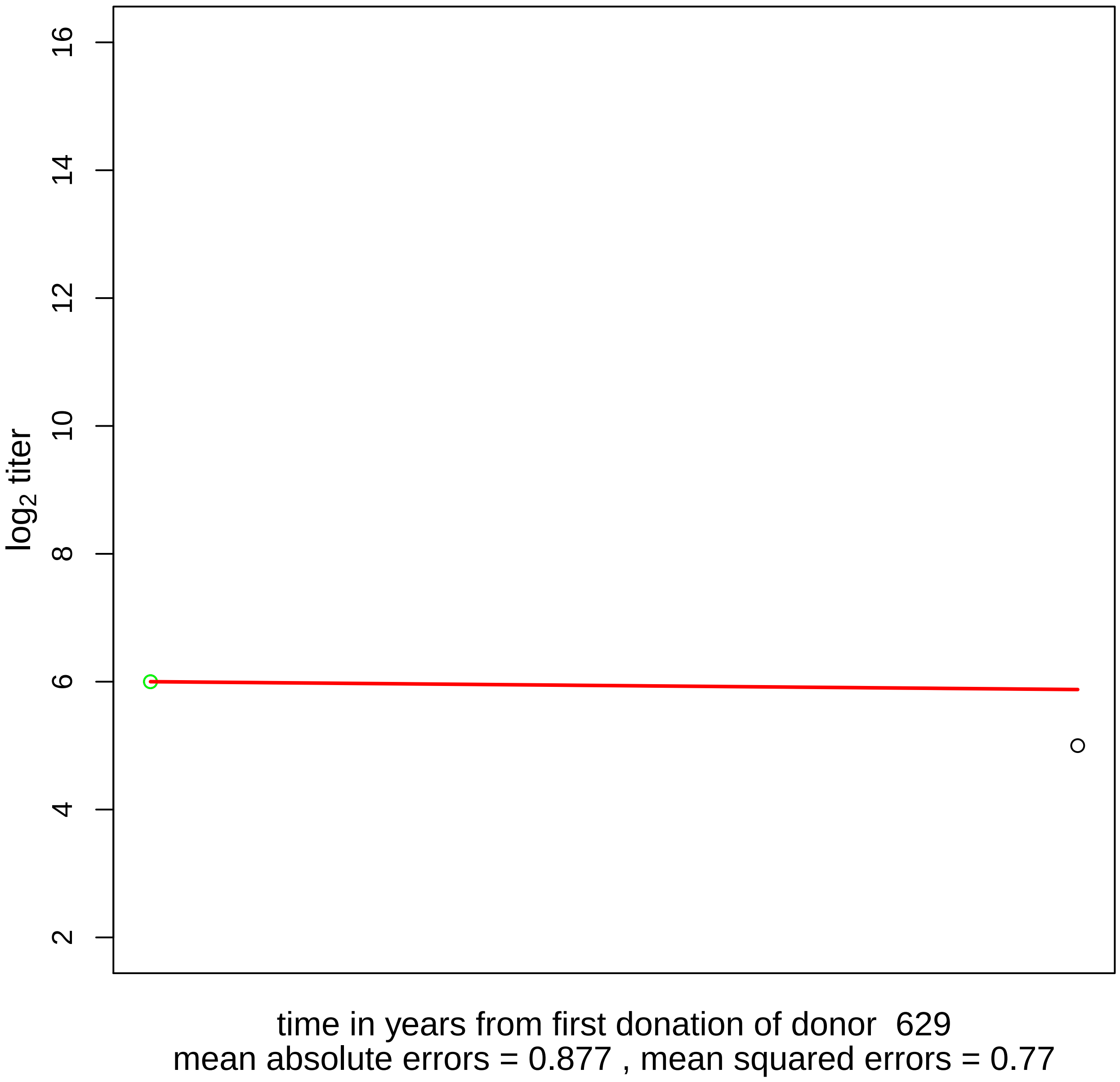

16
14
12
10
8
6
4
2
log2 titer
time in years from first donation of donor 629
mean absolute errors = 0.877 , mean squared errors = 0.77

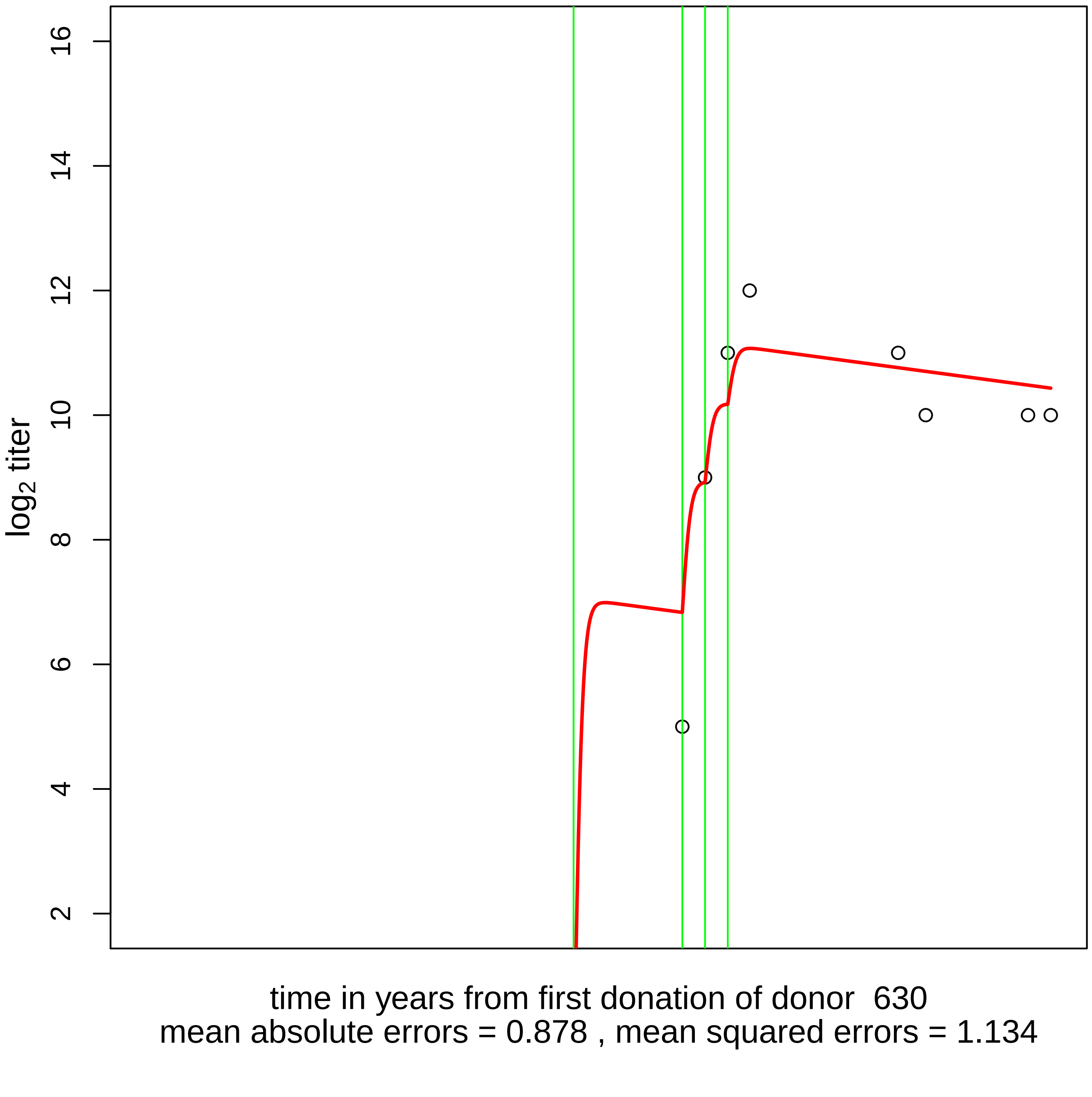
16
14
12
10
8
6
4
2
$\log_2$ titer
time in years from first donation of donor 630
mean absolute errors = 0.878 , mean squared errors = 1.134

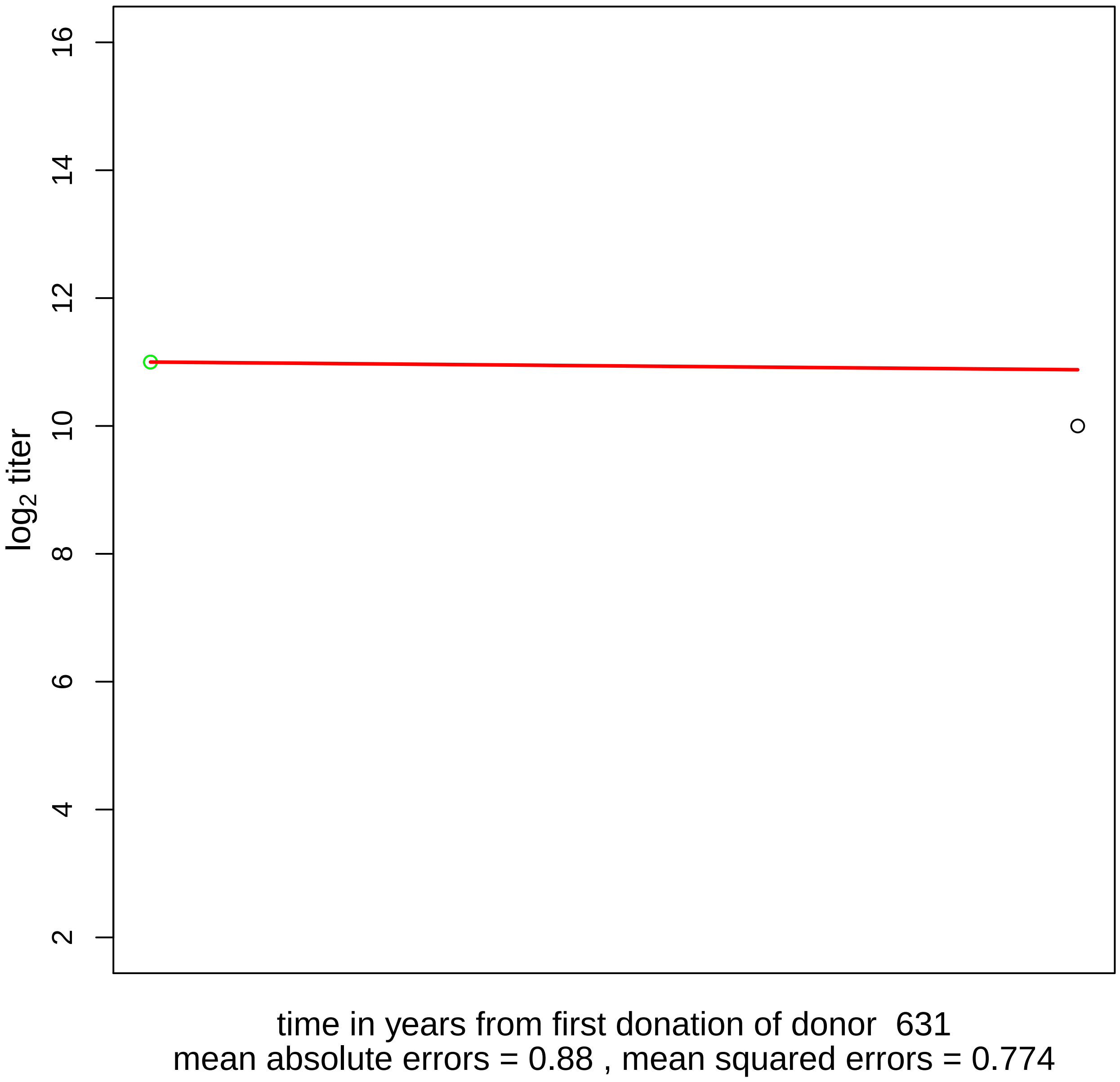
log2 titer
16
14
12
10
8
6
4
2
time in years from first donation of donor 631
mean absolute errors = 0.88 , mean squared errors = 0.774

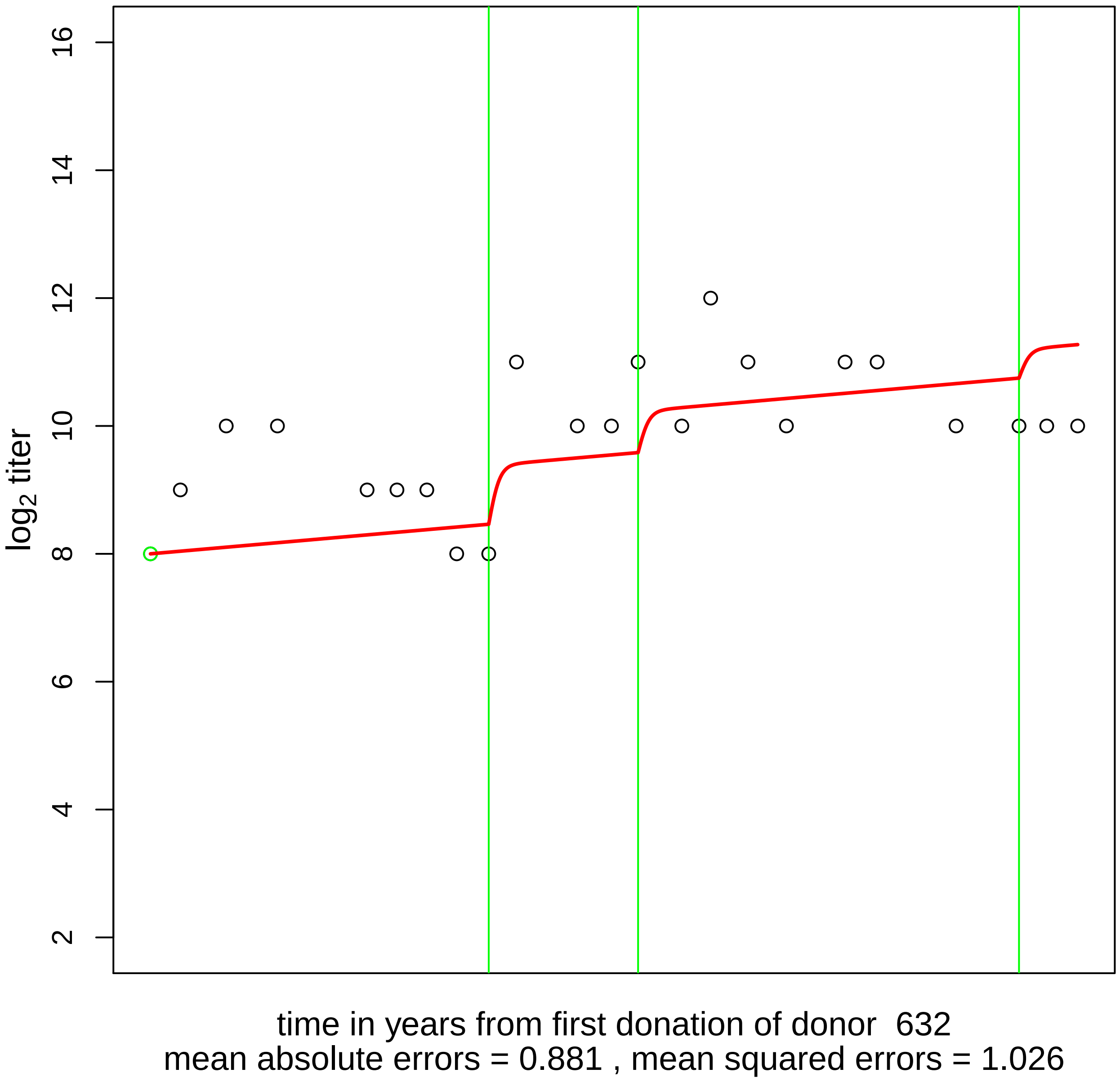

log$_2$ titer
16
14
12
10
8
6
4
2
time in years from first donation of donor 632
mean absolute errors = 0.881 , mean squared errors = 1.026

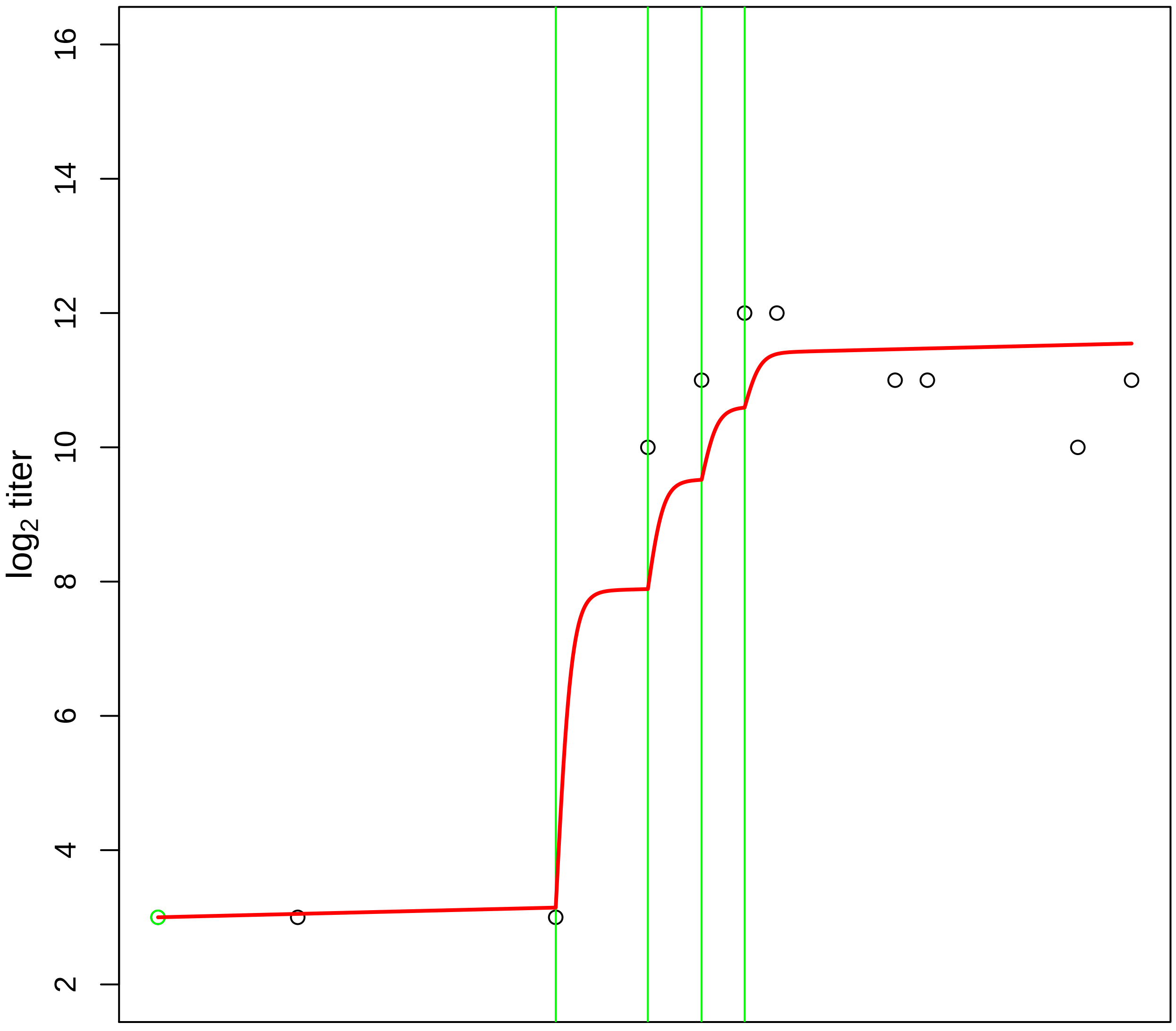

time in years from first donation of donor 633
mean absolute errors = 0.881 , mean squared errors = 1.21

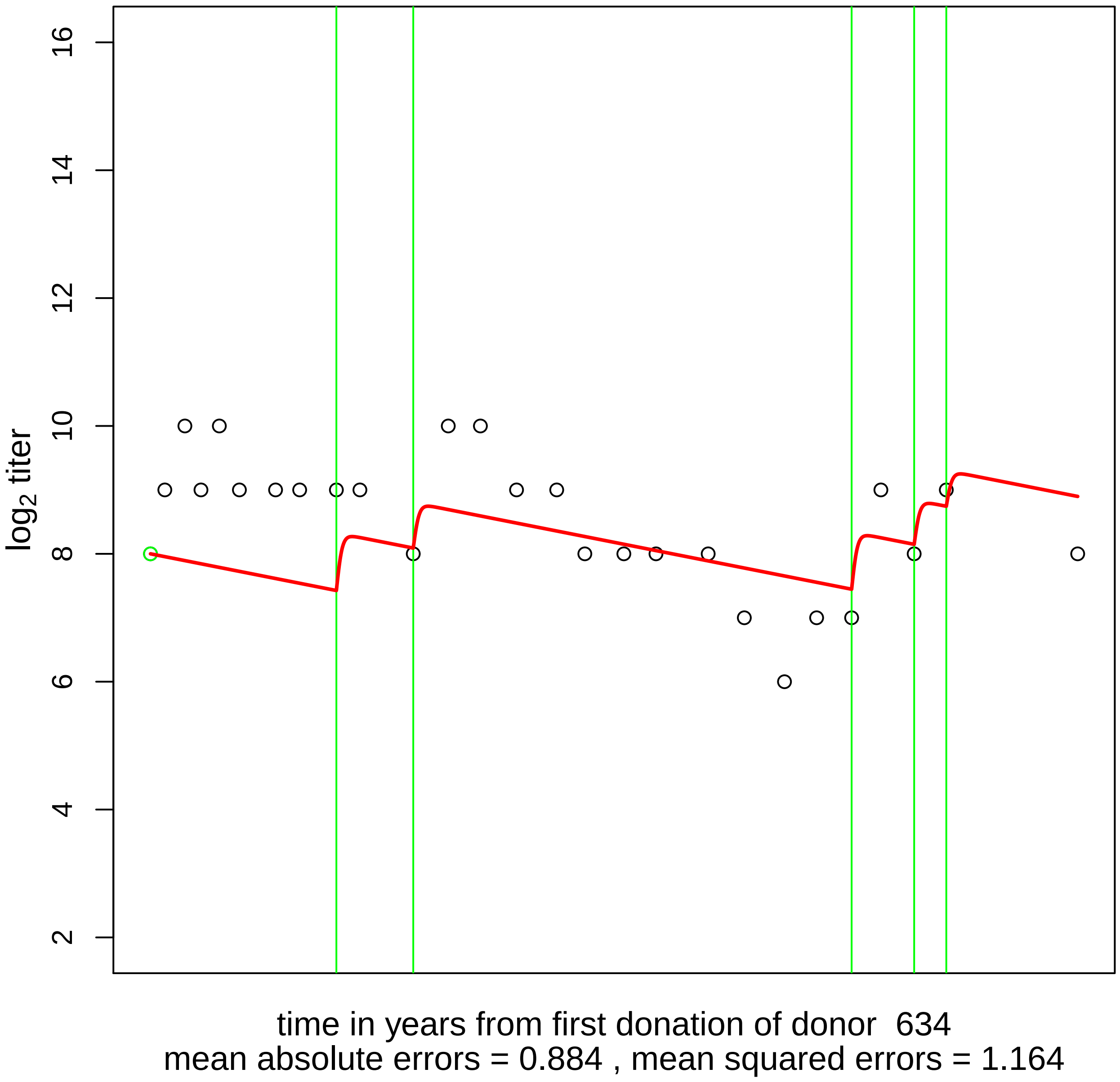

2
4
6
8
10
12
14
16
$\log_2$ titer
time in years from first donation of donor 634
mean absolute errors = 0.884 , mean squared errors = 1.164

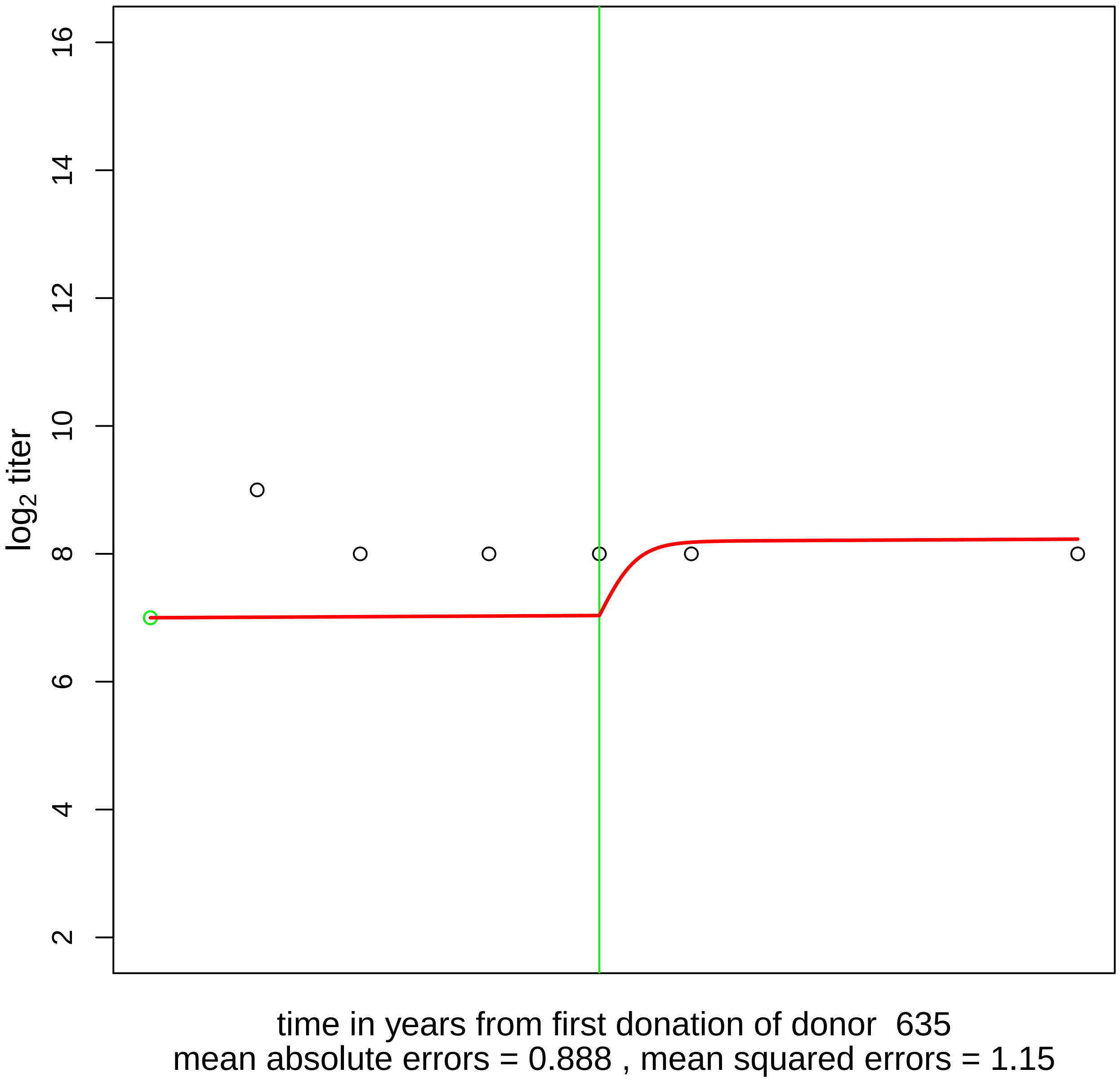

log2 titer
2
4
6
8
10
12
14
16
time in years from first donation of donor 635
mean absolute errors = 0.888 , mean squared errors = 1.15

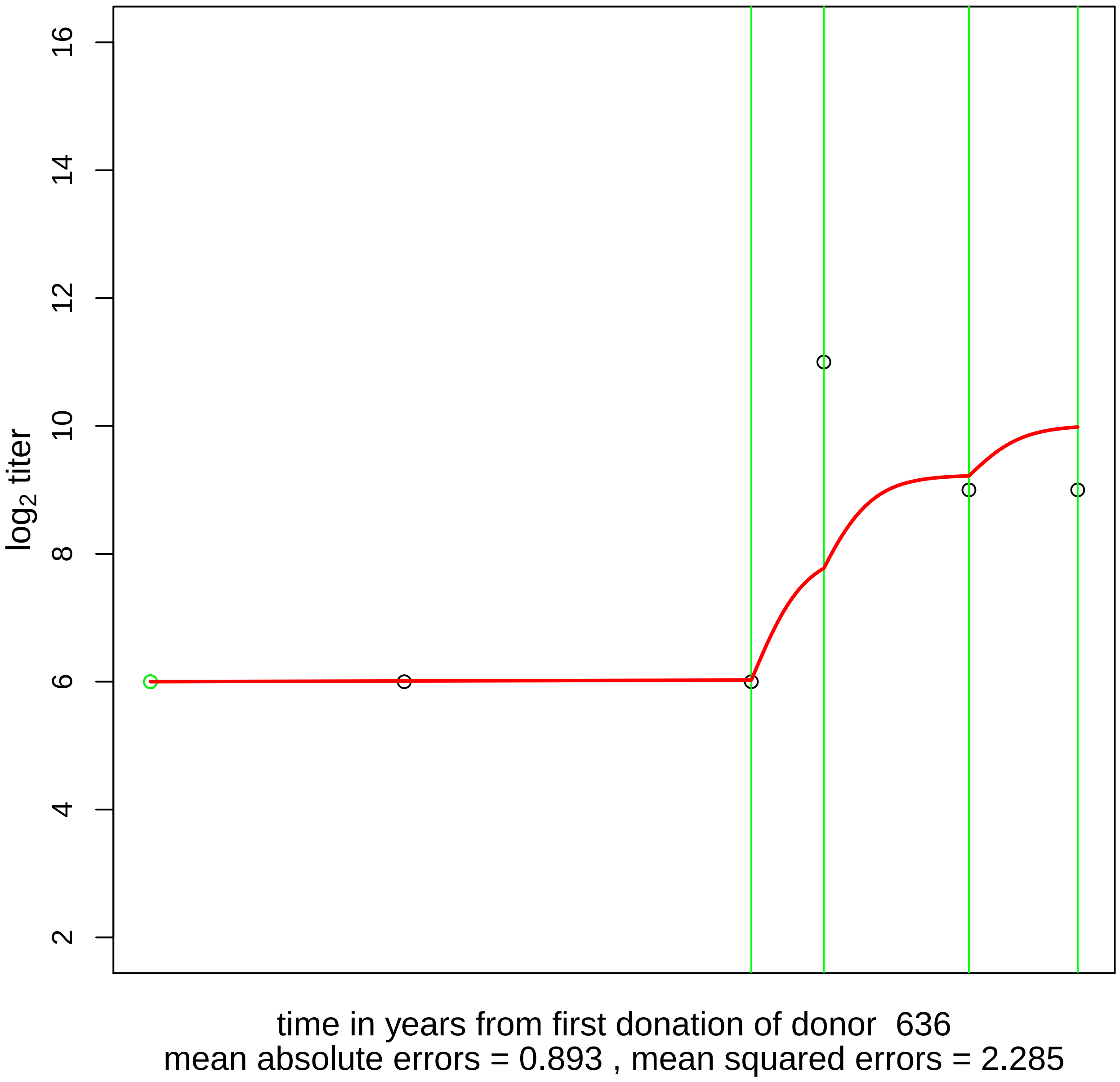
log2 titer
2
4
6
8
10
12
14
16
time in years from first donation of donor 636
mean absolute errors = 0.893 , mean squared errors = 2.285

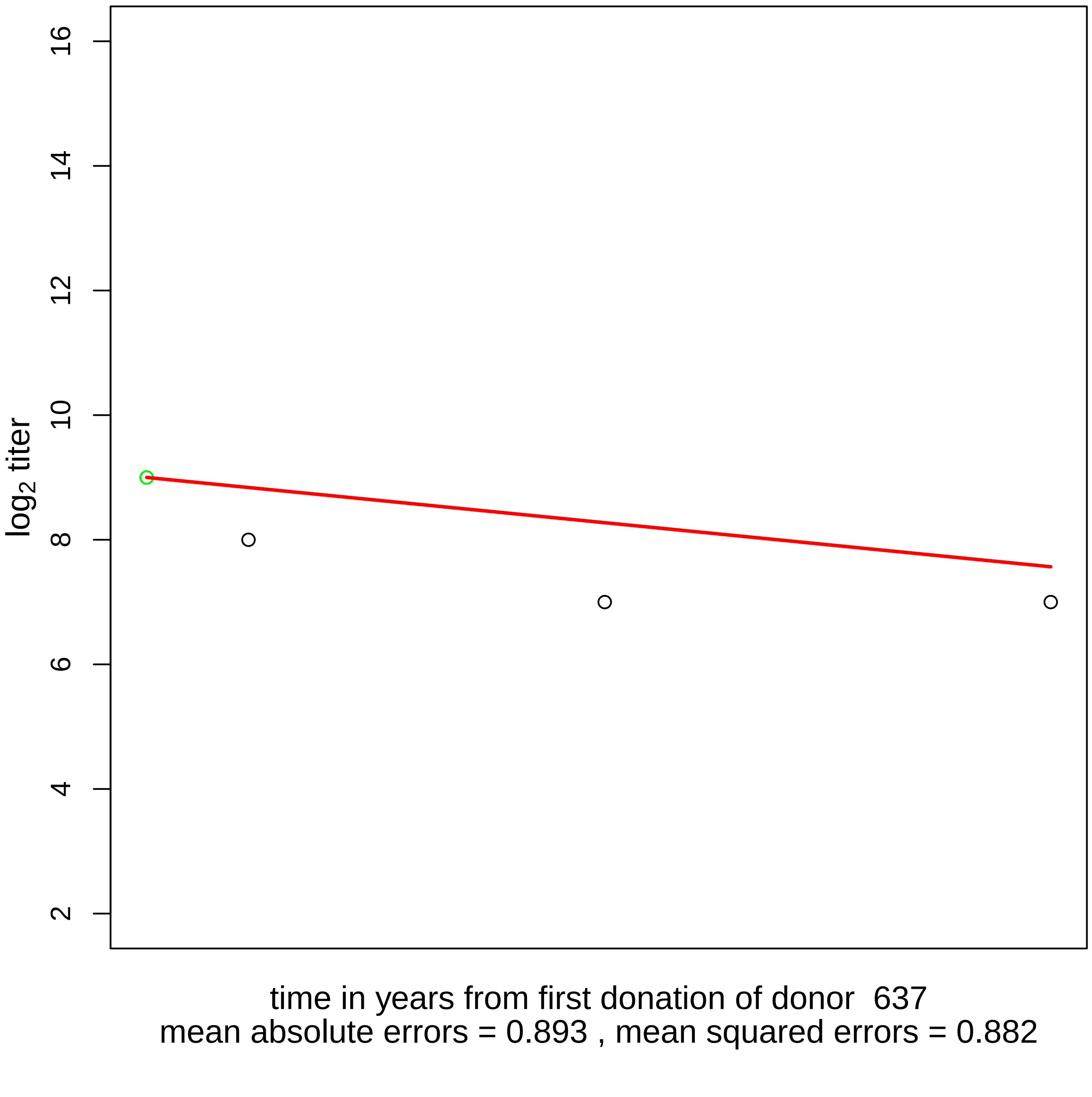

16
14
12
10
8
6
4
2
log2 titer
time in years from first donation of donor 637
mean absolute errors = 0.893 , mean squared errors = 0.882

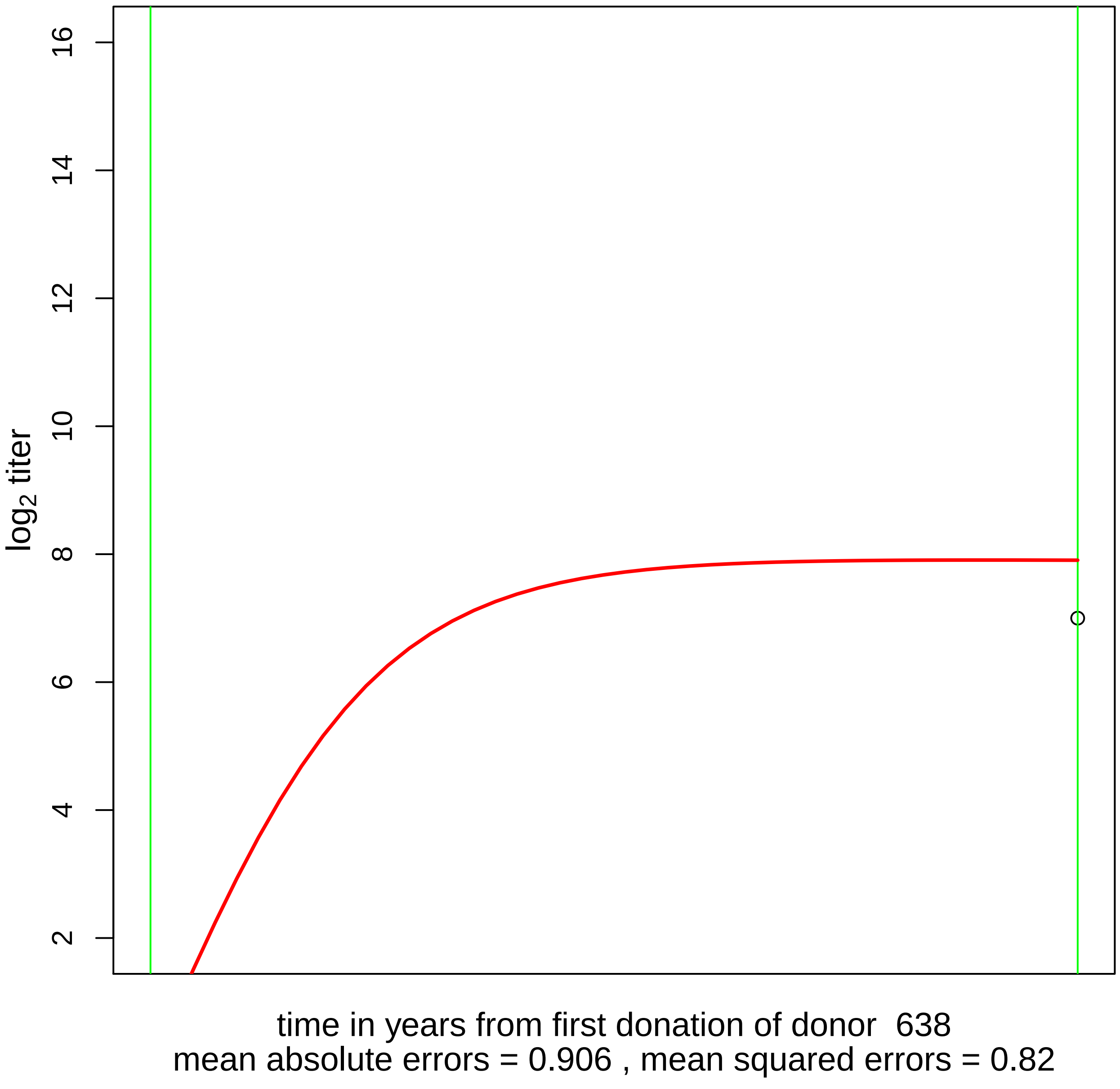

log2 titer
16
14
12
10
8
6
4
2
time in years from first donation of donor 638
mean absolute errors = 0.906 , mean squared errors = 0.82

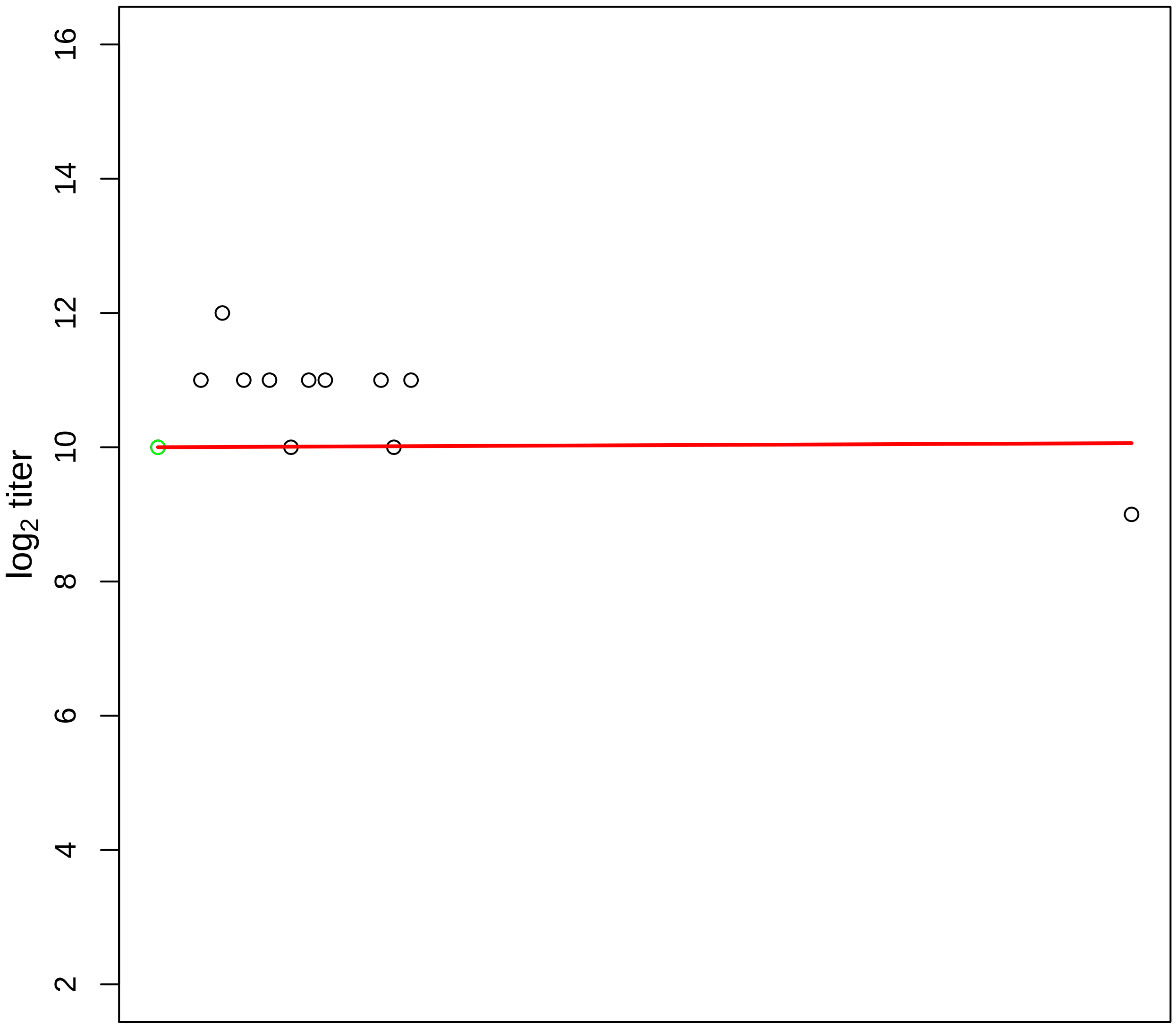

time in years from first donation of donor 639
mean absolute errors = 0.91 , mean squared errors = 1.089

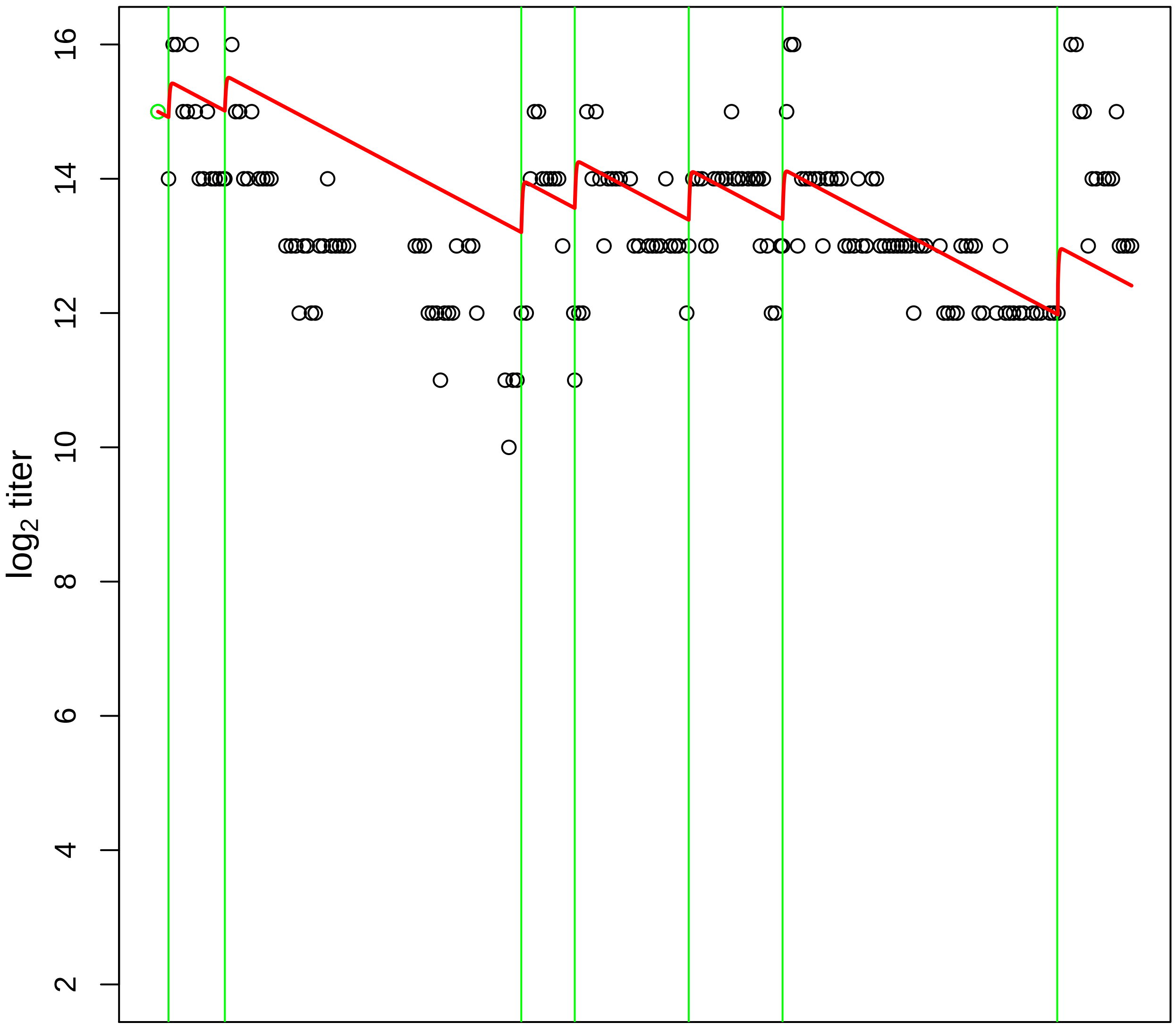

time in years from first donation of donor 640
mean absolute errors = 0.911 , mean squared errors = 1.547

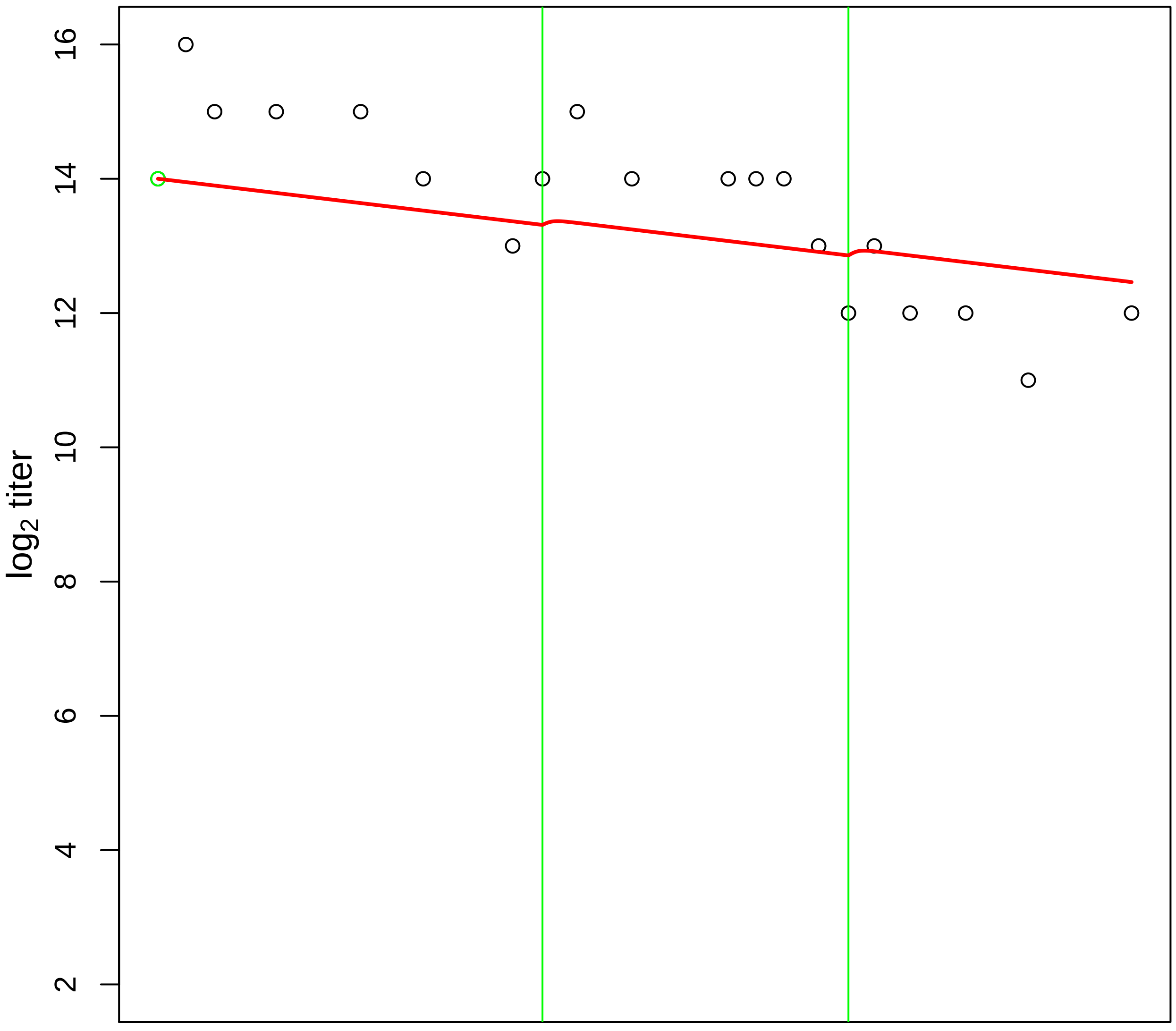

time in years from first donation of donor 641
mean absolute errors = 0.913 , mean squared errors = 1.091

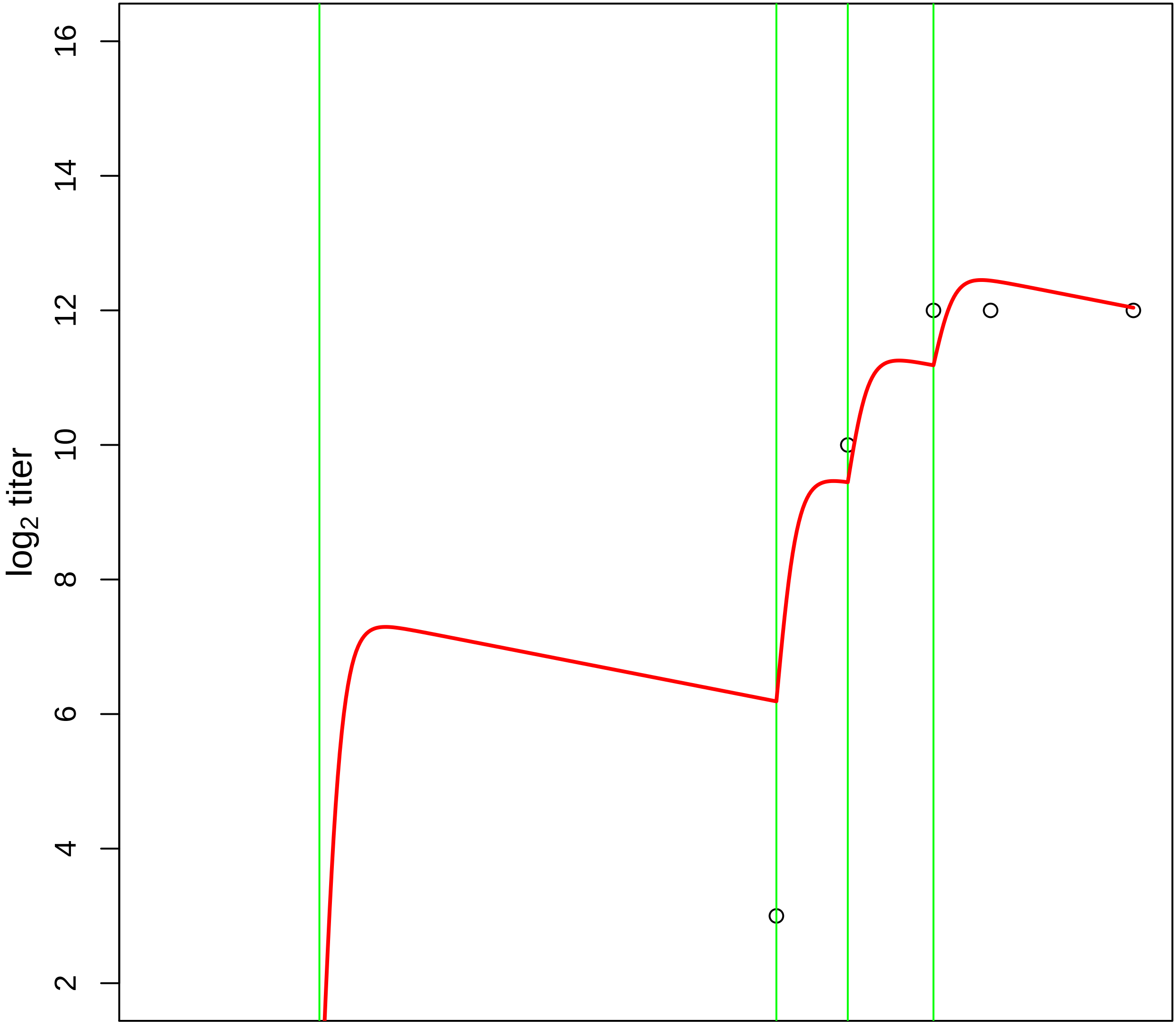

time in years from first donation of donor 642
mean absolute errors = 0.918 , mean squared errors = 1.926

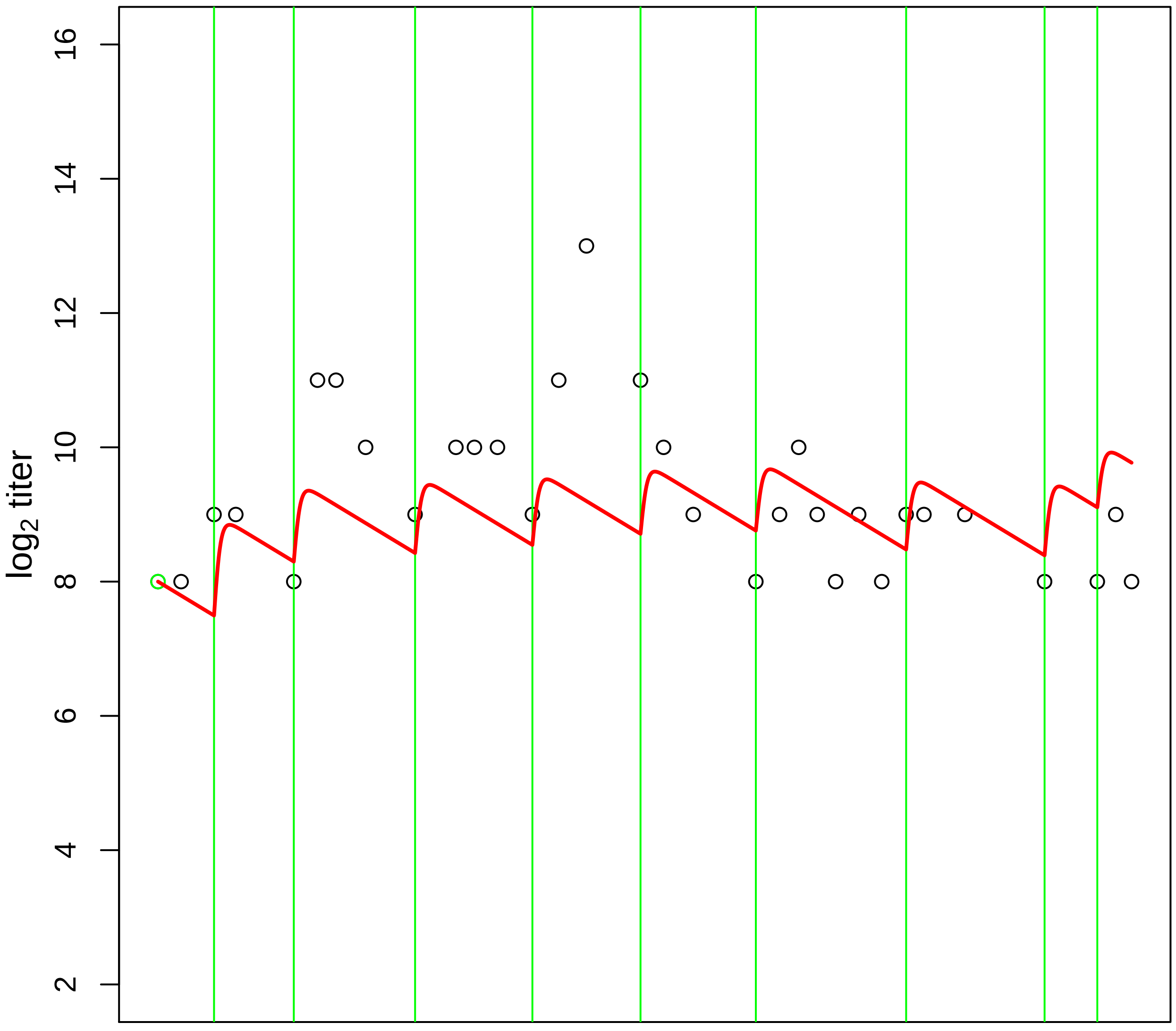

time in years from first donation of donor 643
mean absolute errors = 0.92 , mean squared errors = 1.439

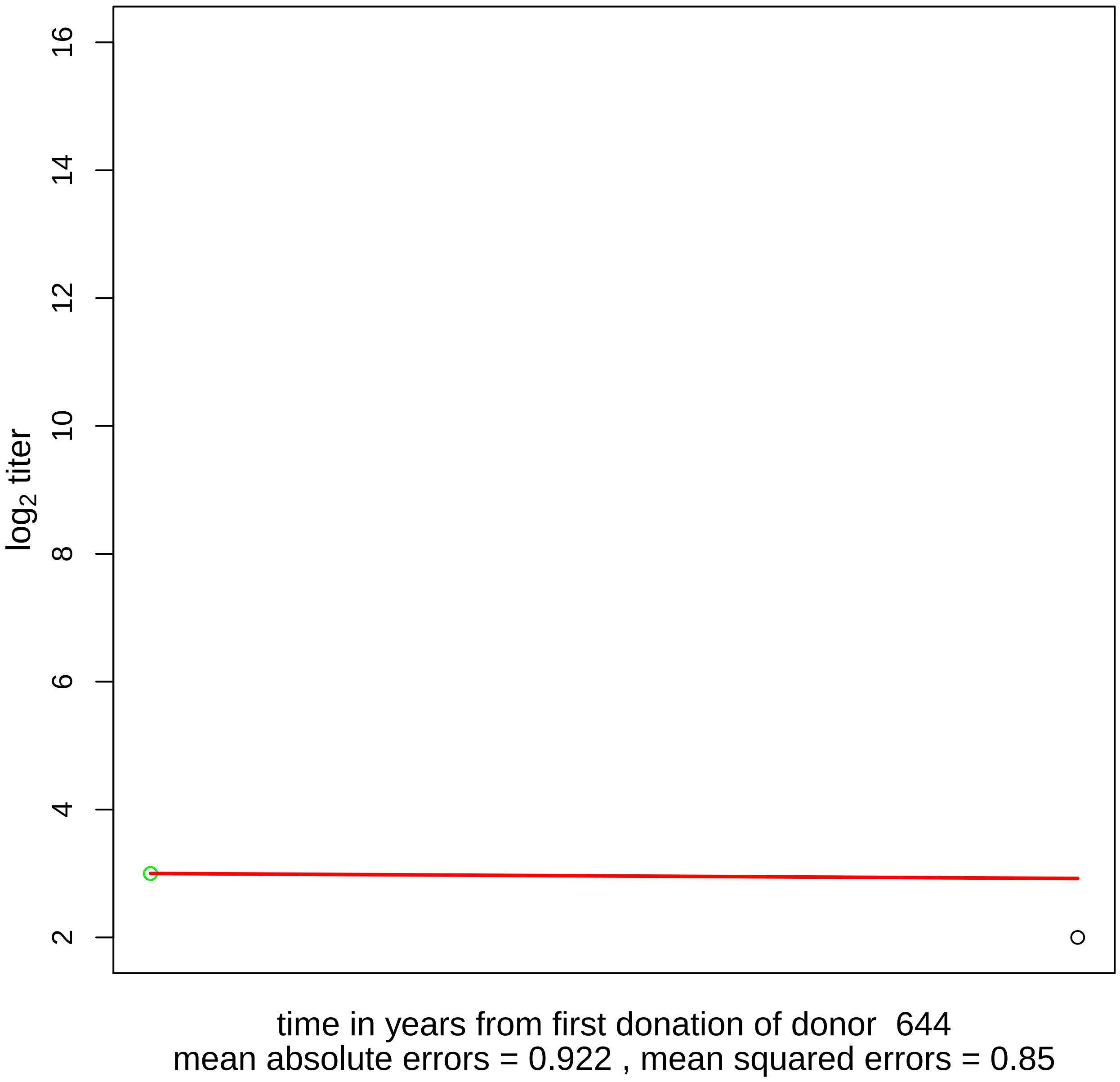

2
4
6
8
10
12
14
16
$\log_2$ titer
time in years from first donation of donor 644
mean absolute errors = 0.922 , mean squared errors = 0.85

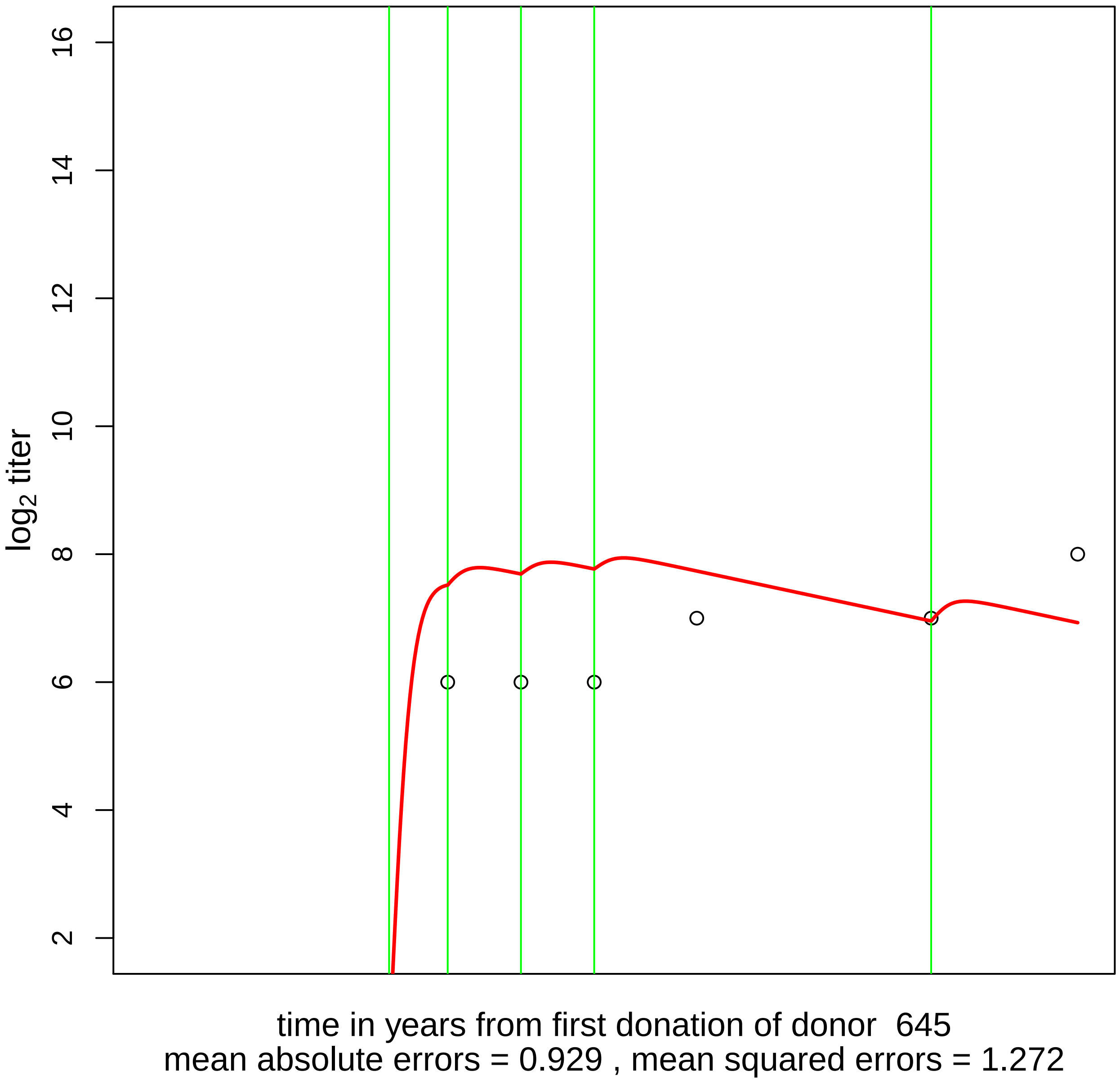

log2 titer
16
14
12
10
8
6
4
2
time in years from first donation of donor 645
mean absolute errors = 0.929 , mean squared errors = 1.272

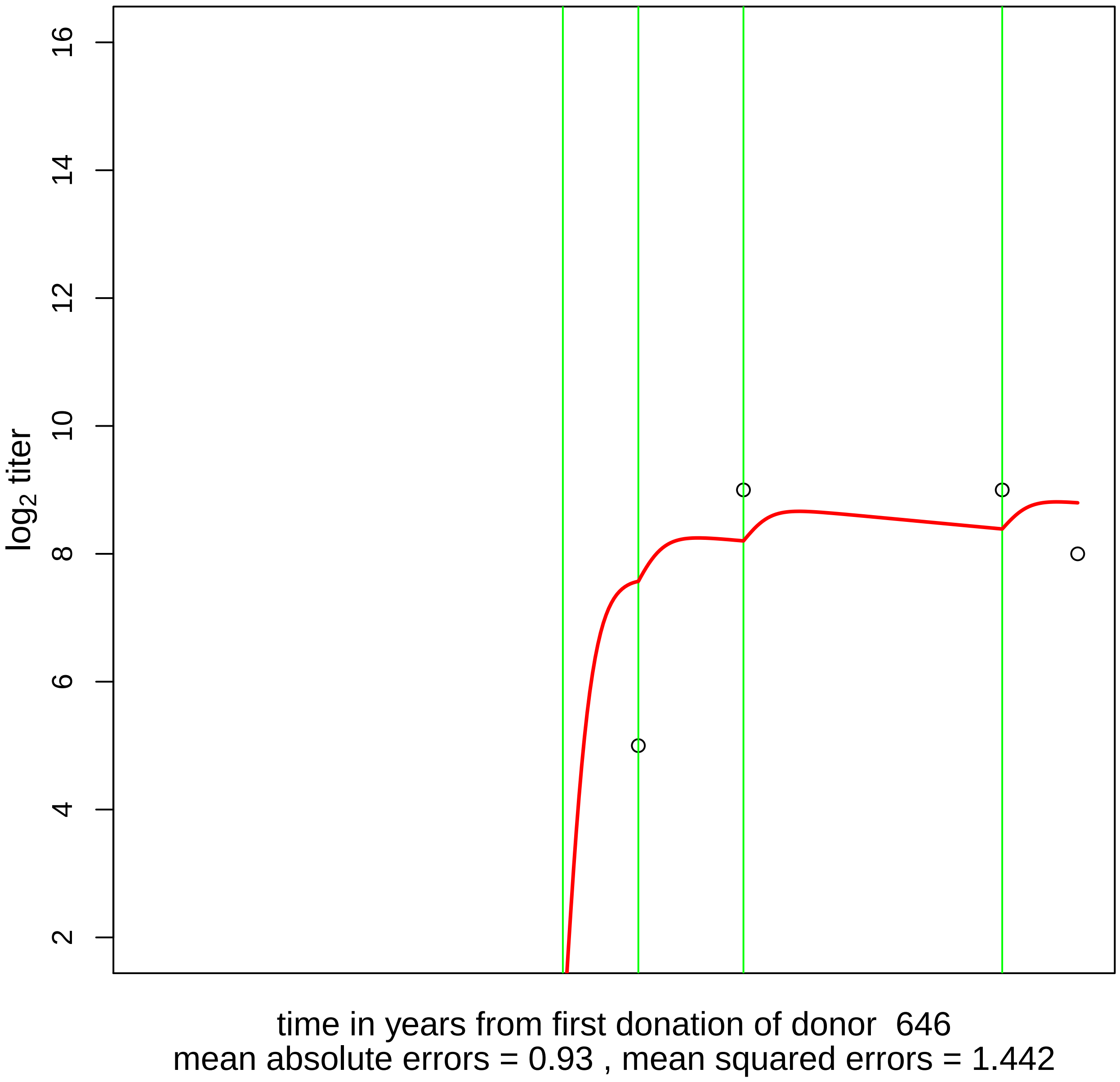
16
14
12
10
8
6
4
2
log2 titer
time in years from first donation of donor 646
mean absolute errors = 0.93 , mean squared errors = 1.442

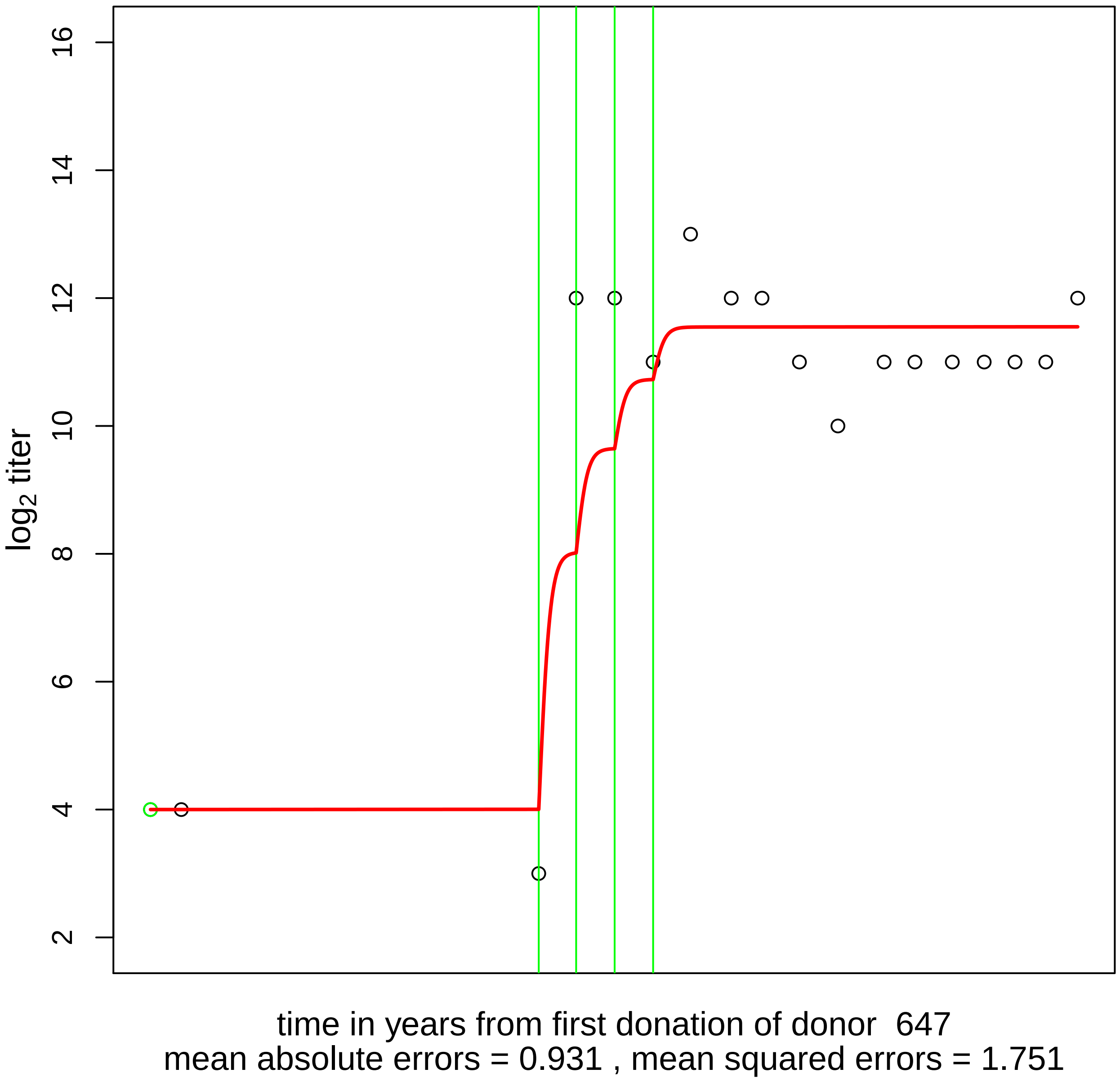

16
14
12
10
8
6
4
2
log2 titer
time in years from first donation of donor 647
mean absolute errors = 0.931 , mean squared errors = 1.751

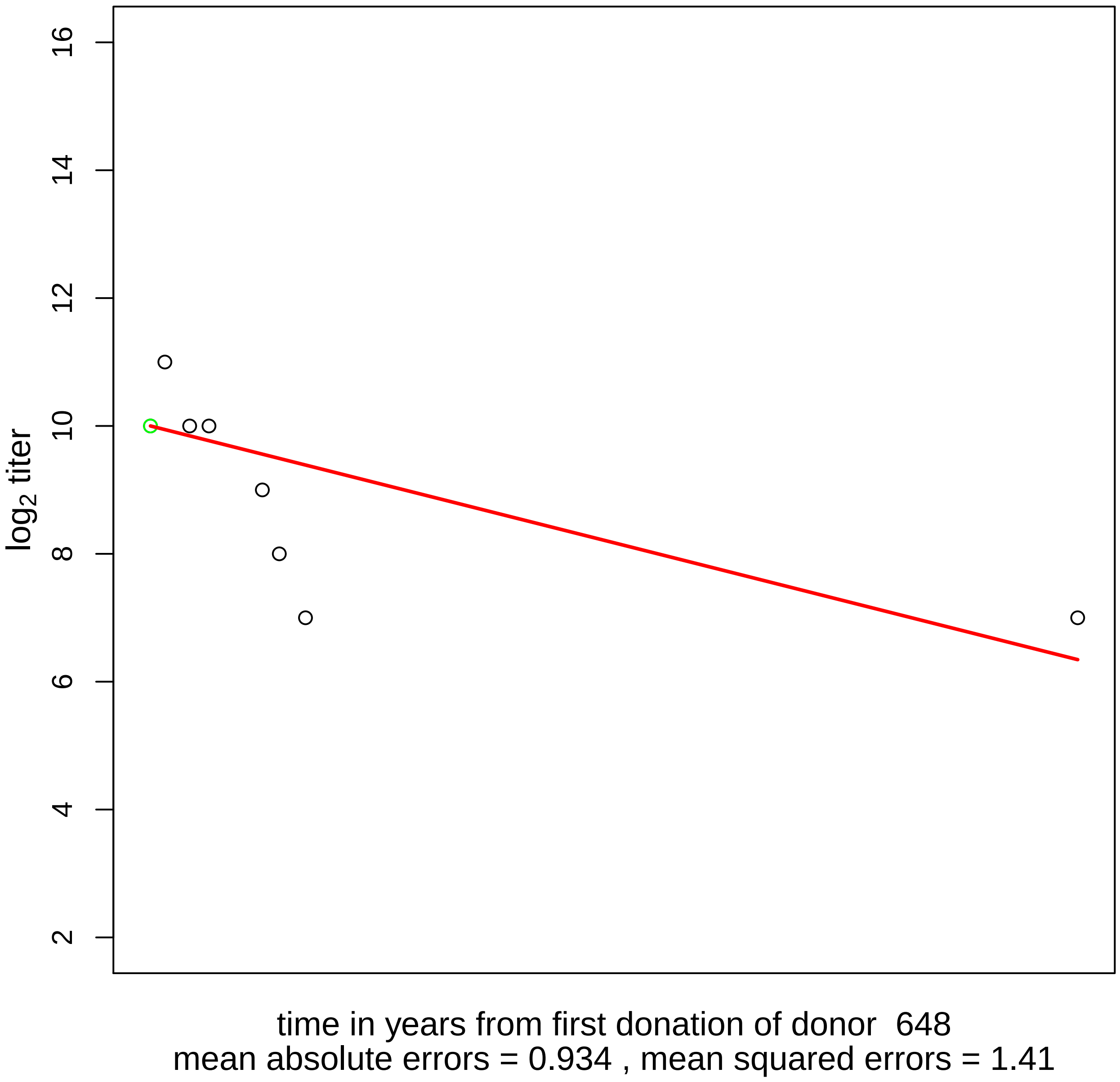

16
14
12
10
8
6
4
2
log2 titer
time in years from first donation of donor 648
mean absolute errors = 0.934 , mean squared errors = 1.41

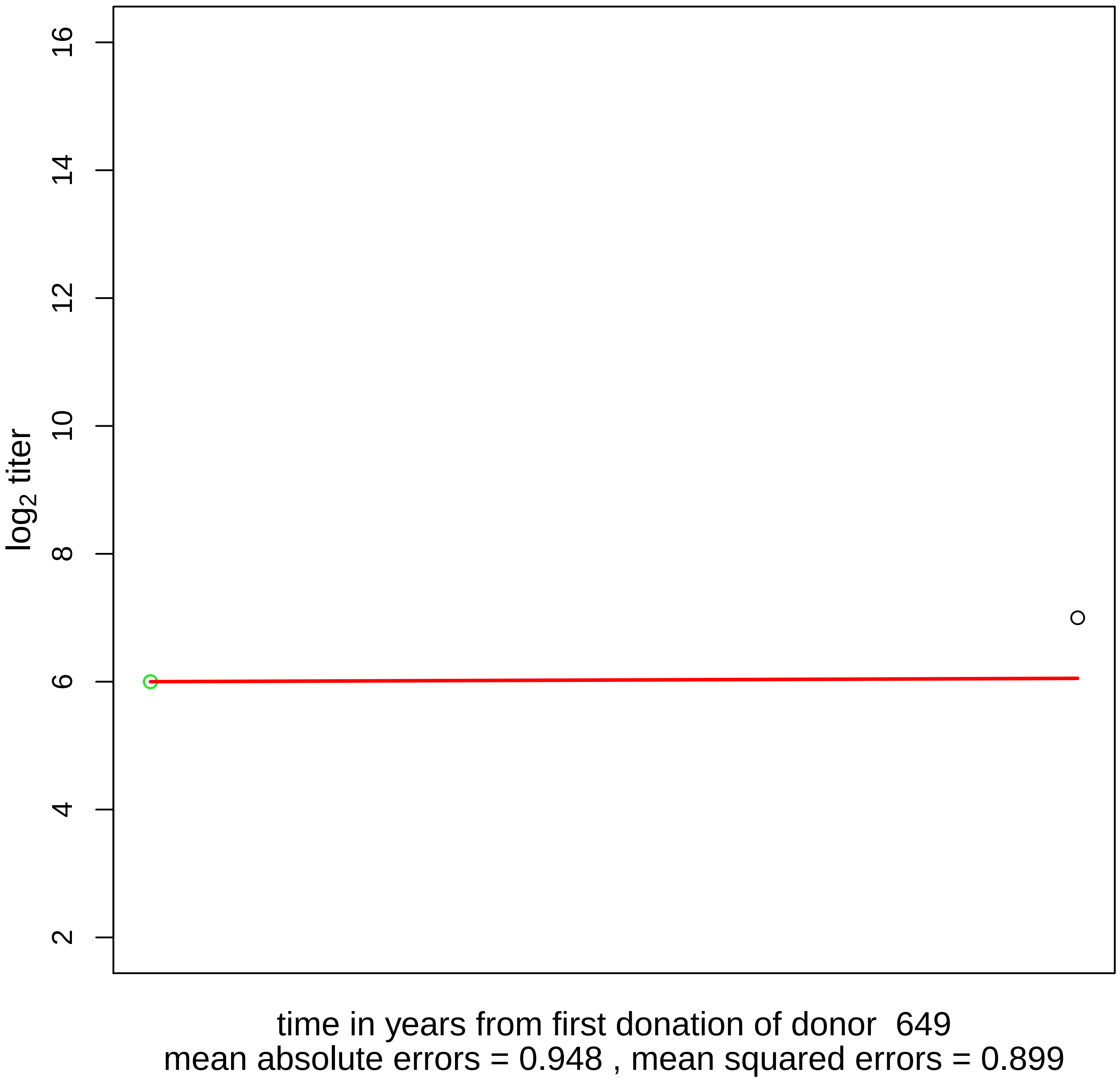
log2 titer
16
14
12
10
8
6
4
2
time in years from first donation of donor 649
mean absolute errors = 0.948 , mean squared errors = 0.899

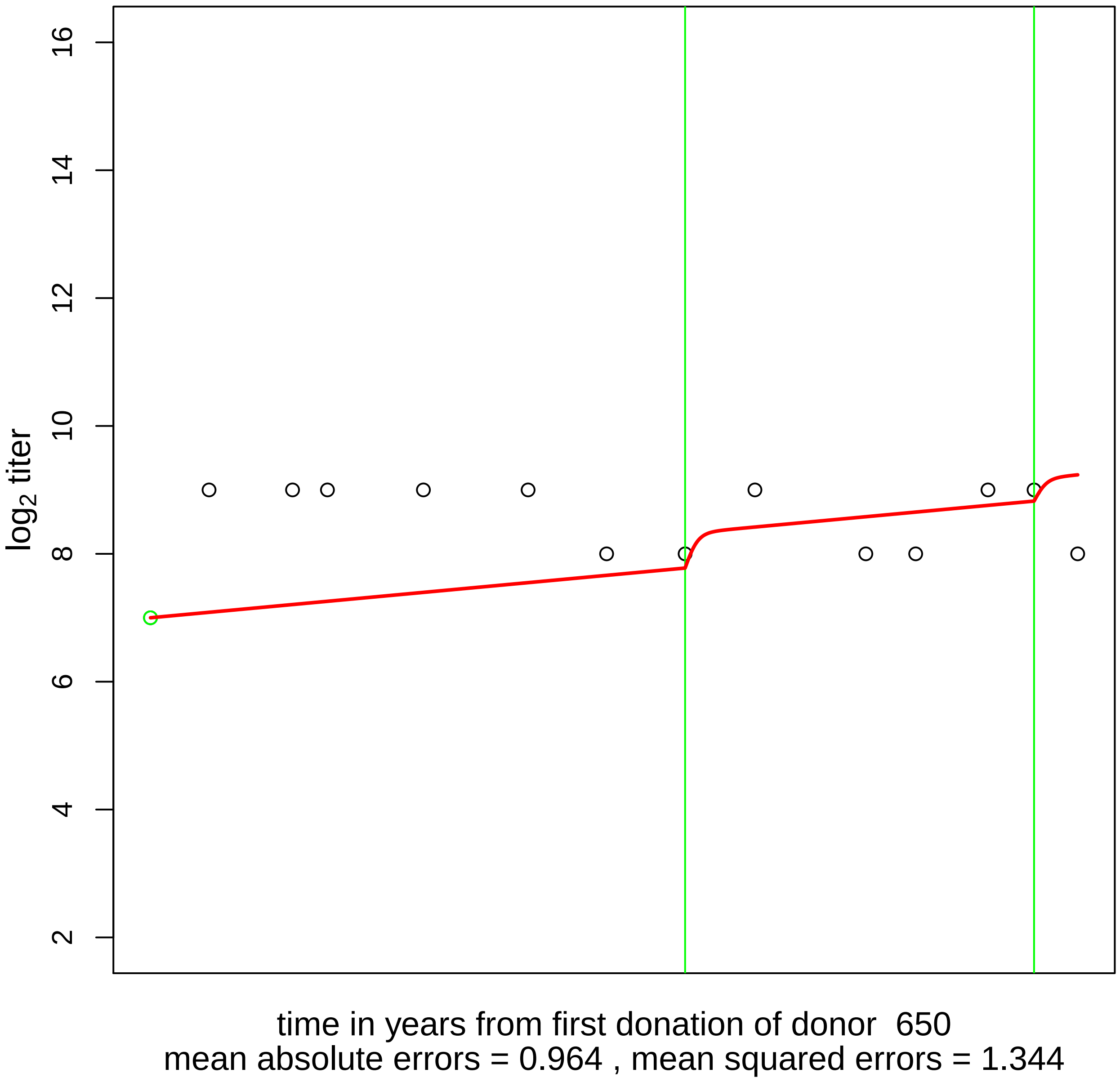

16
14
12
10
8
6
4
2
$\log_2$ titer
time in years from first donation of donor 650
mean absolute errors = 0.964 , mean squared errors = 1.344

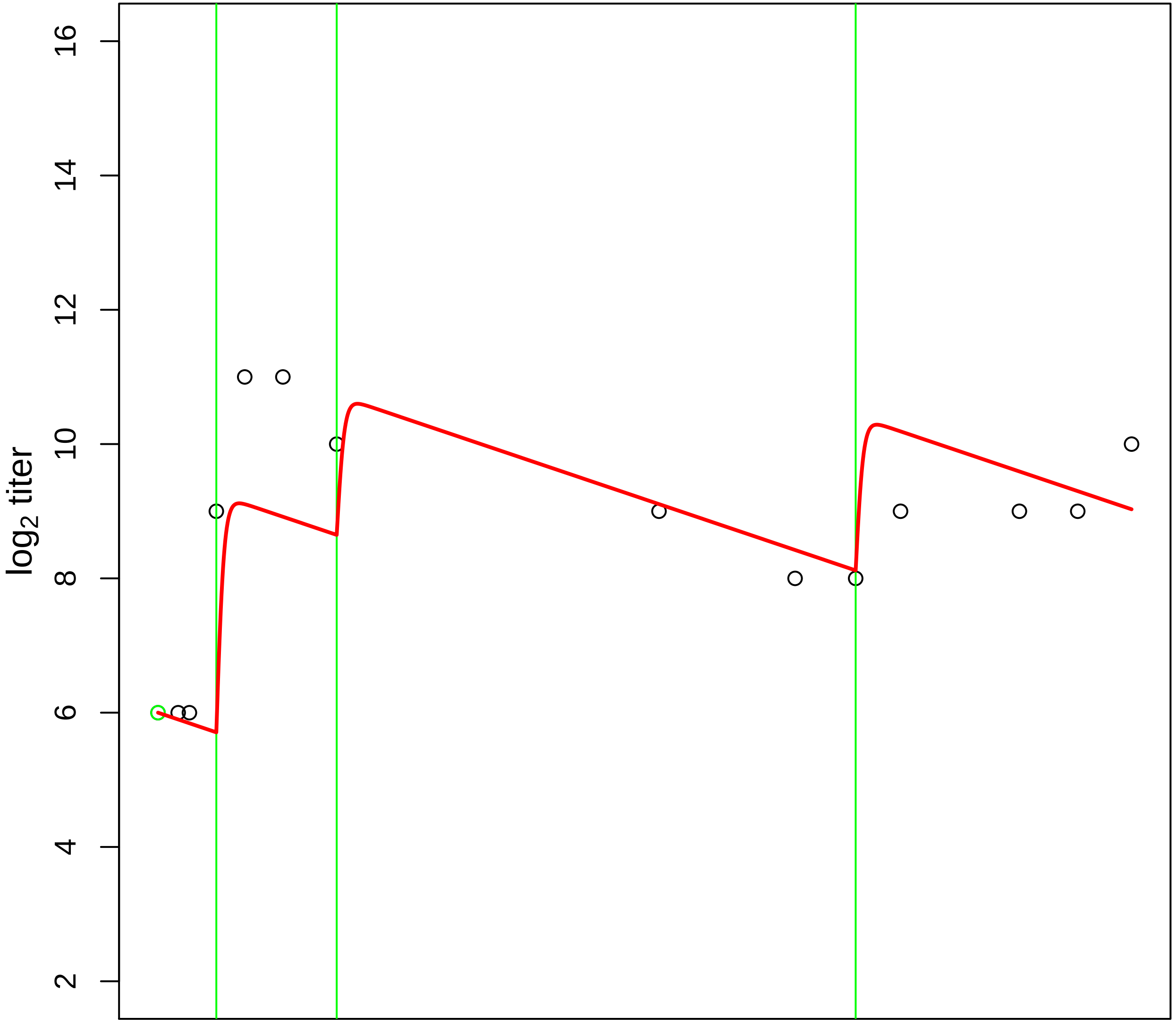

time in years from first donation of donor 651
mean absolute errors = 0.968 , mean squared errors = 1.818

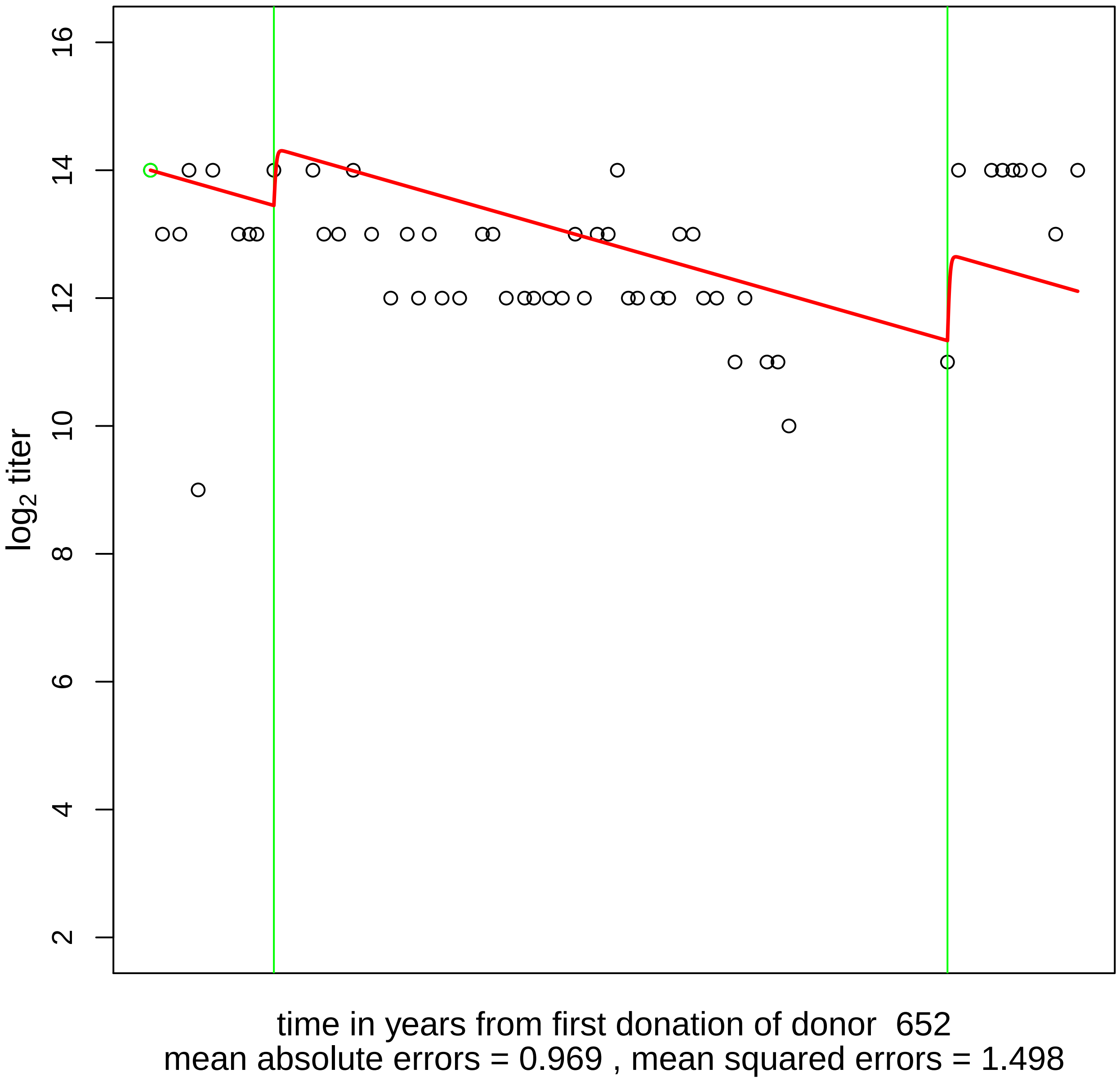
16
14
12
10
8
6
4
2
log2 titer
time in years from first donation of donor 652
mean absolute errors = 0.969 , mean squared errors = 1.498

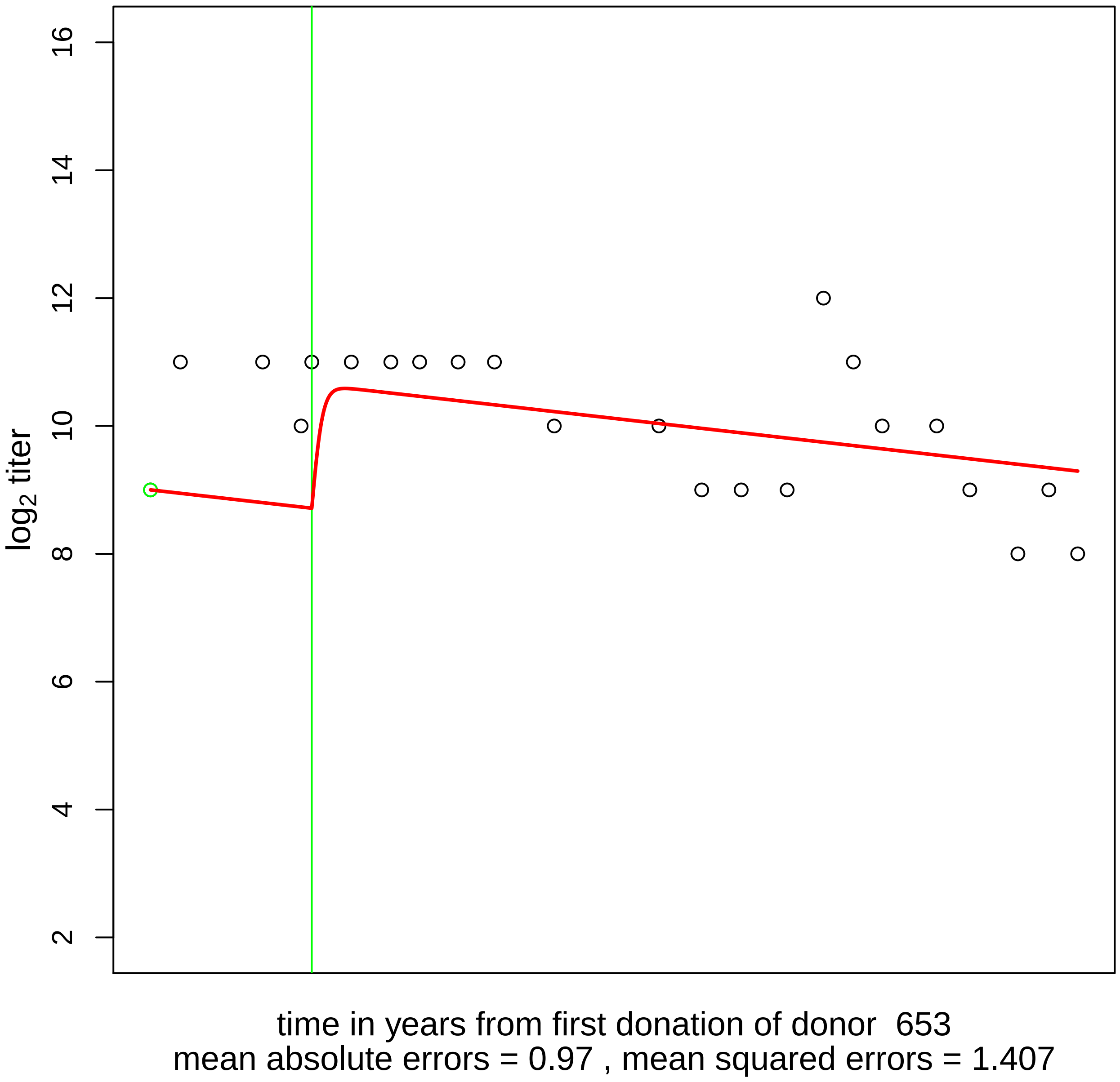
log2 titer
2
4
6
8
10
12
14
16
time in years from first donation of donor 653
mean absolute errors = 0.97 , mean squared errors = 1.407

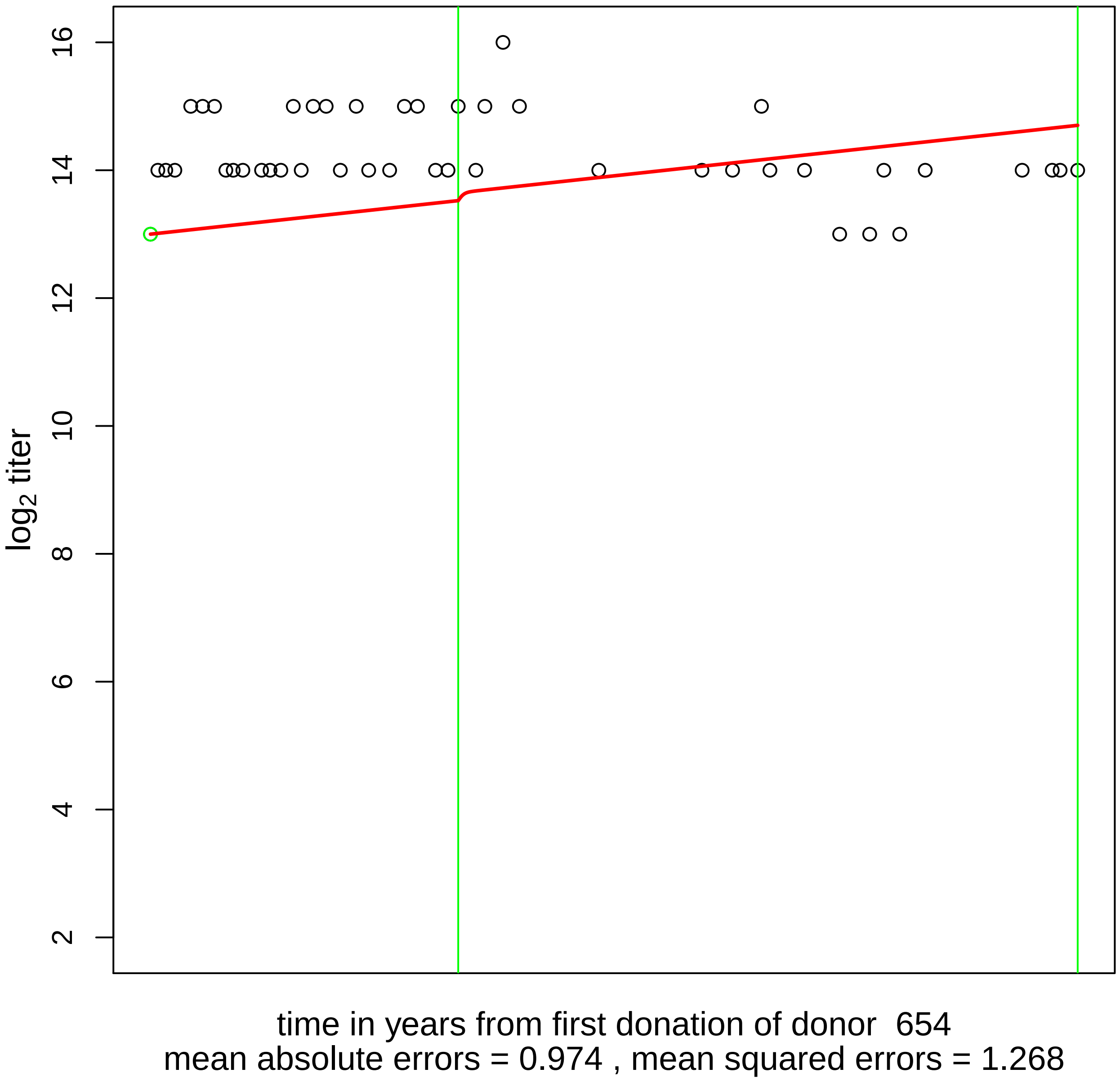
16
14
12
10
8
6
4
2
$\log_2$ titer
time in years from first donation of donor 654
mean absolute errors = 0.974 , mean squared errors = 1.268

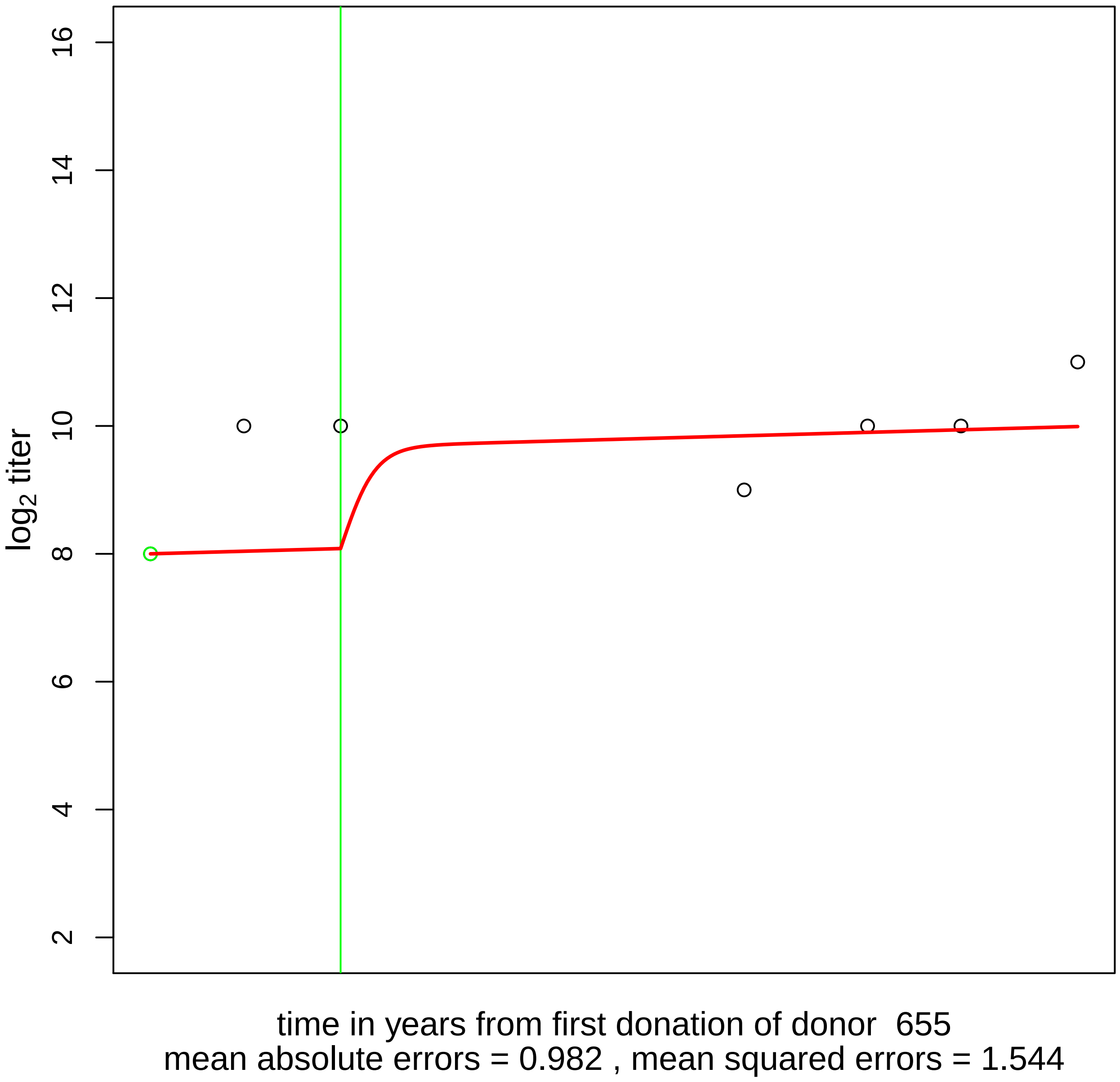

16
14
12
10
8
6
4
2
log2 titer
time in years from first donation of donor 655
mean absolute errors = 0.982 , mean squared errors = 1.544

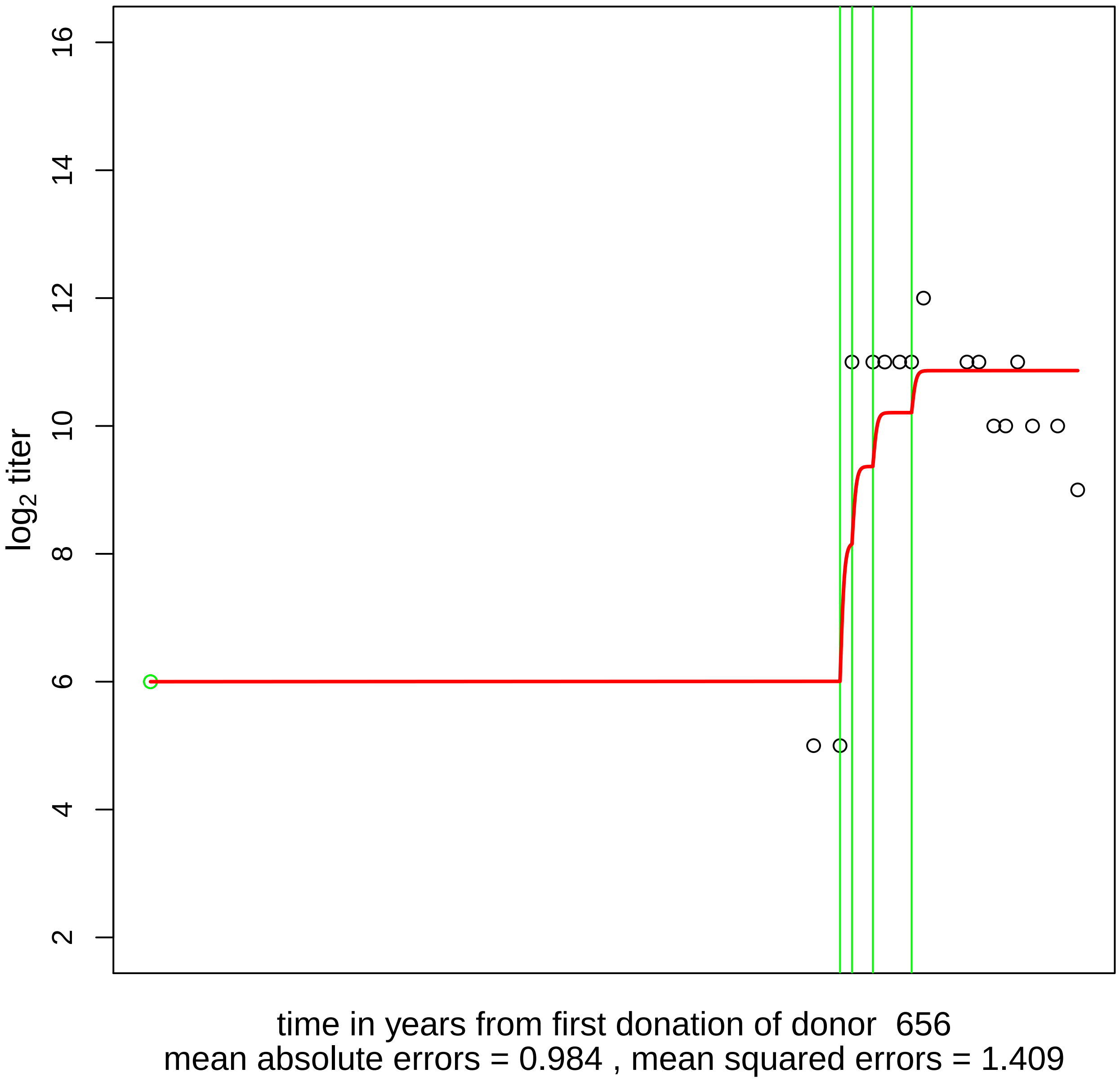

2
4
6
8
10
12
14
16
$\log_2$ titer
time in years from first donation of donor 656
mean absolute errors = 0.984 , mean squared errors = 1.409

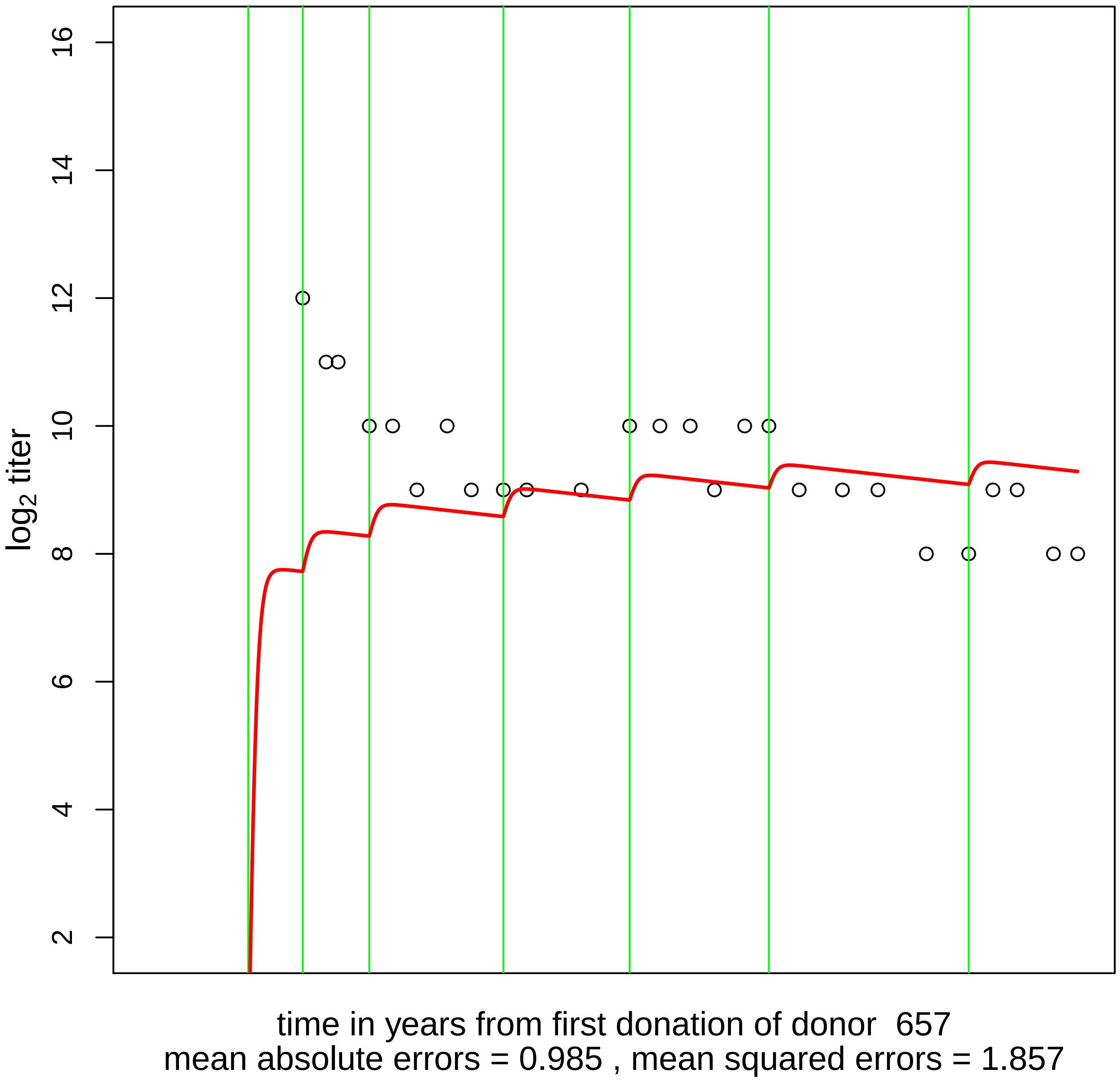

log2 titer
2
4
6
8
10
12
14
16
time in years from first donation of donor 657
mean absolute errors = 0.985 , mean squared errors = 1.857

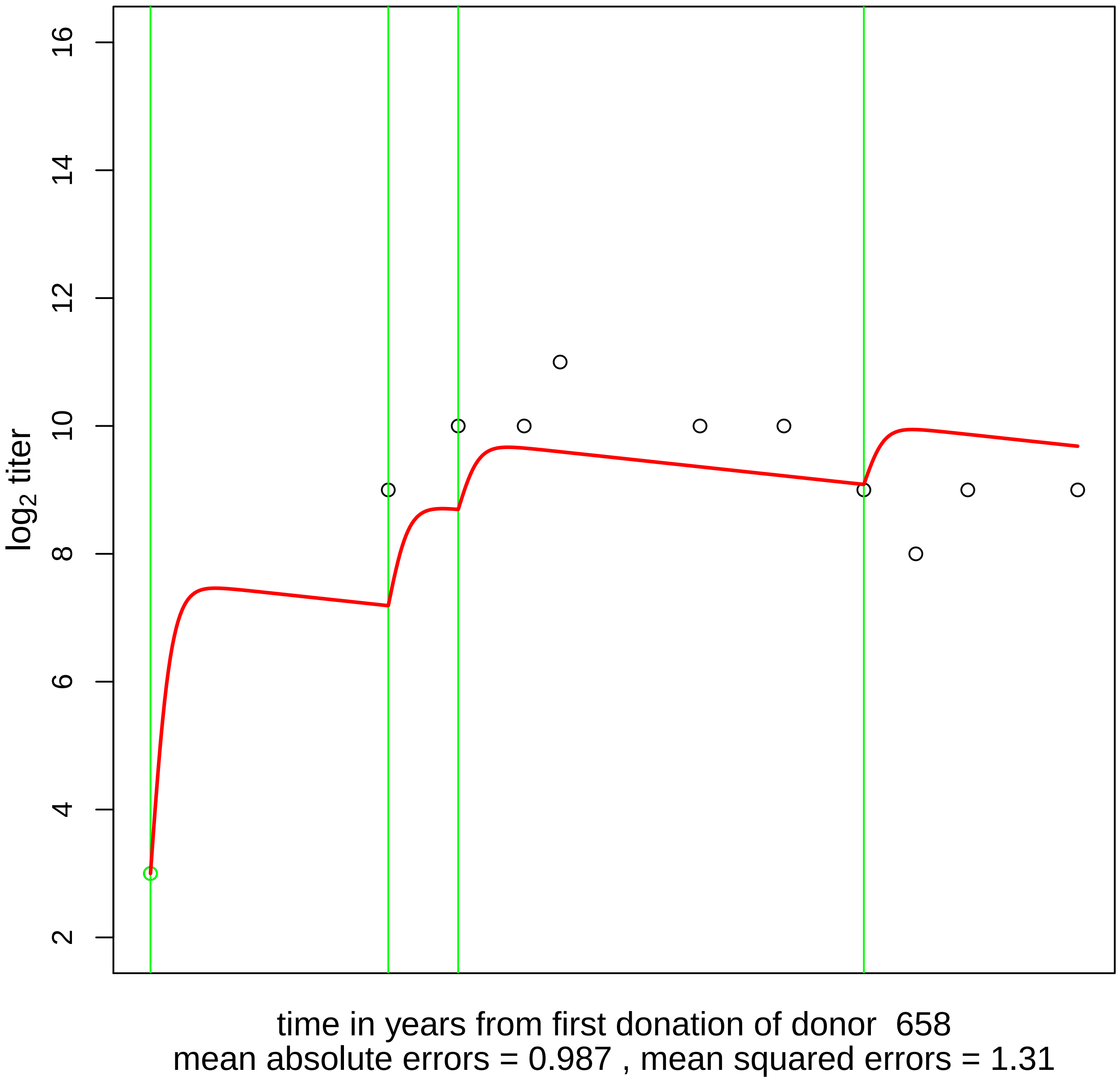

16
14
12
10
8
6
4
2
log2 titer
time in years from first donation of donor 658
mean absolute errors = 0.987 , mean squared errors = 1.31

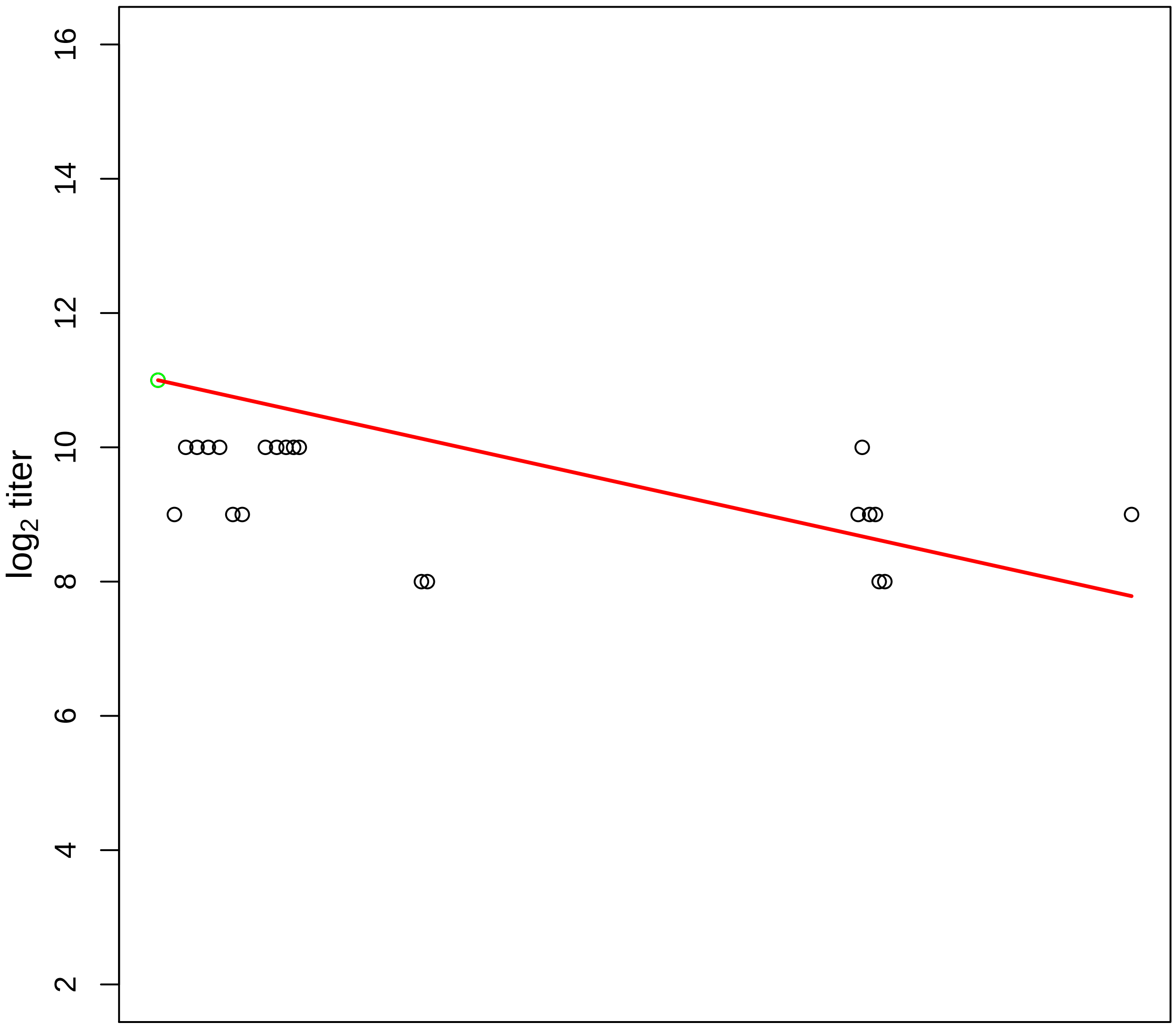

time in years from first donation of donor 659
mean absolute errors = 0.989 , mean squared errors = 1.322

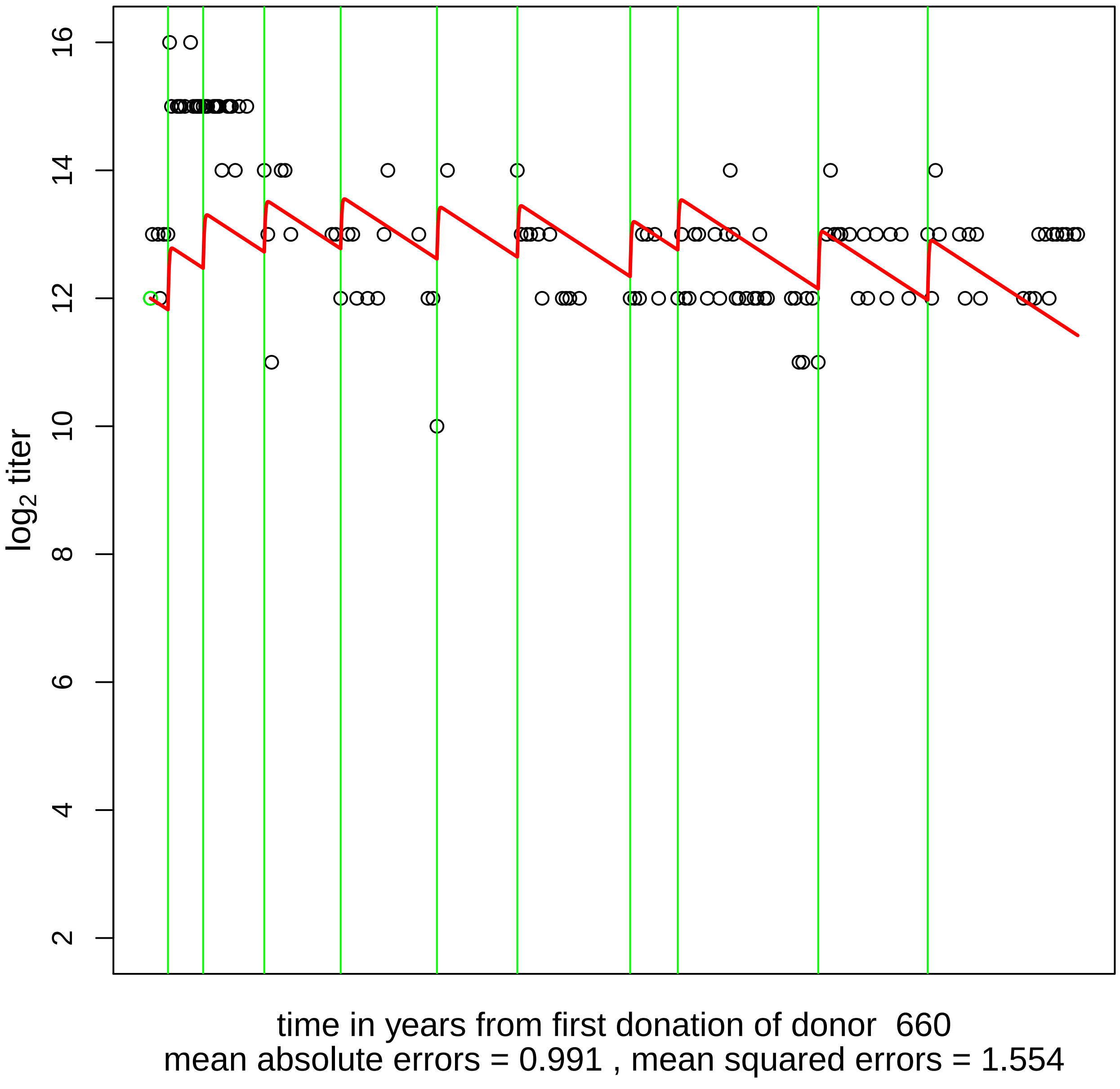

16
14
12
10
8
6
4
2
$\log_2$ titer
time in years from first donation of donor 660
mean absolute errors = 0.991 , mean squared errors = 1.554

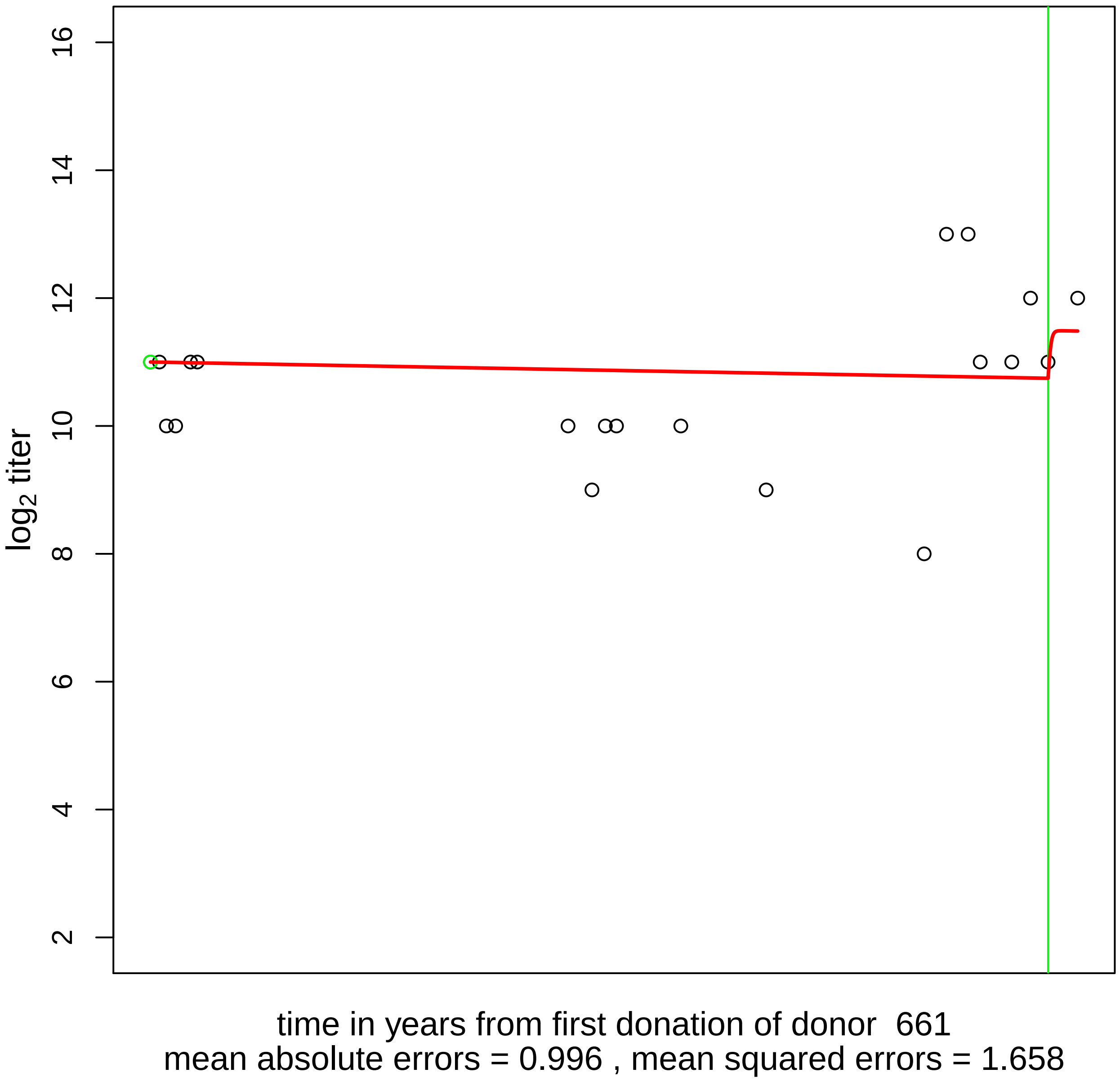

16
14
12
10
8
6
4
2
log2 titer
time in years from first donation of donor 661
mean absolute errors = 0.996 , mean squared errors = 1.658

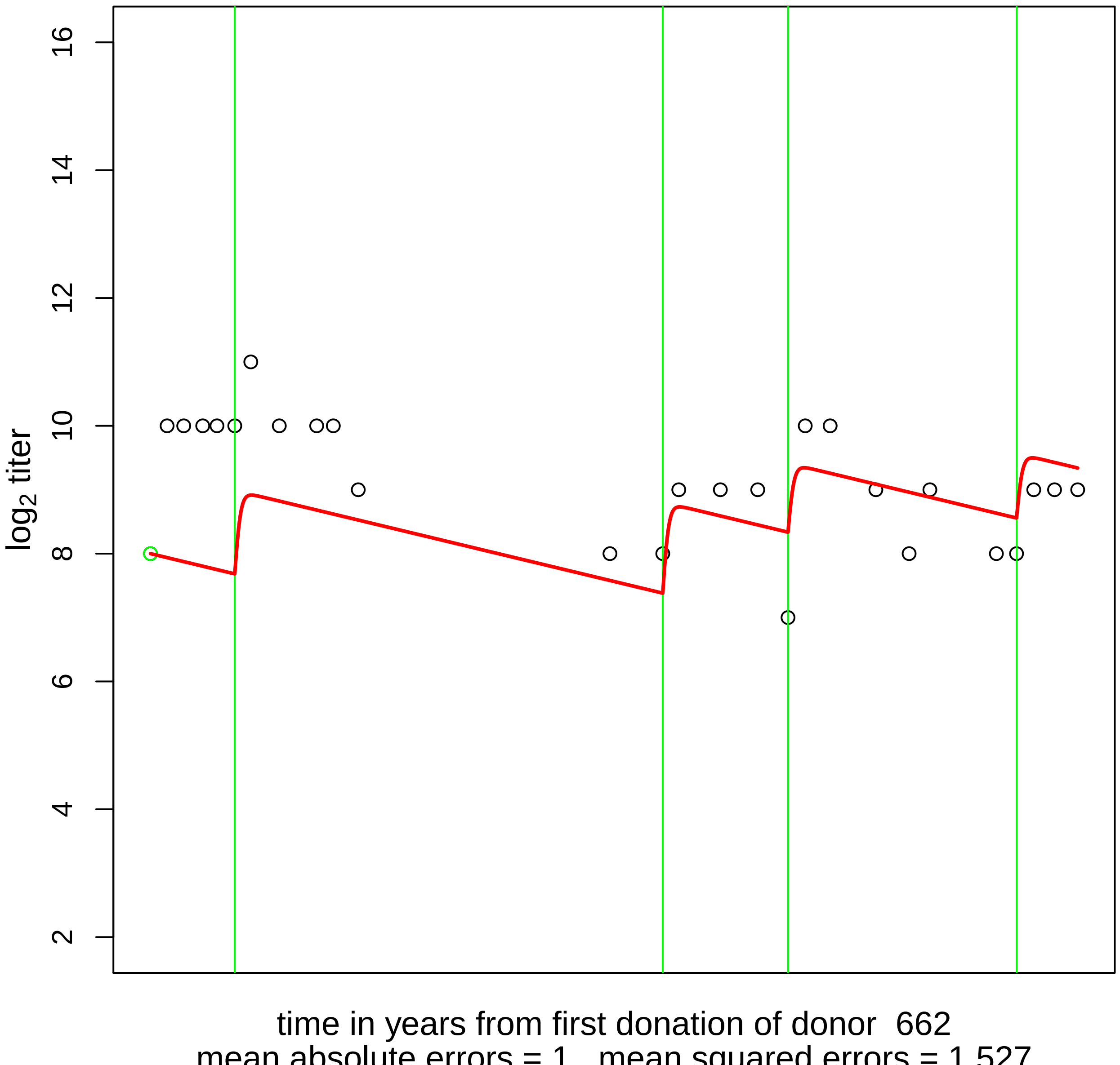

log2 titer
2
4
6
8
10
12
14
16
time in years from first donation of donor 662
mean absolute errors = 1 , mean squared errors = 1.527

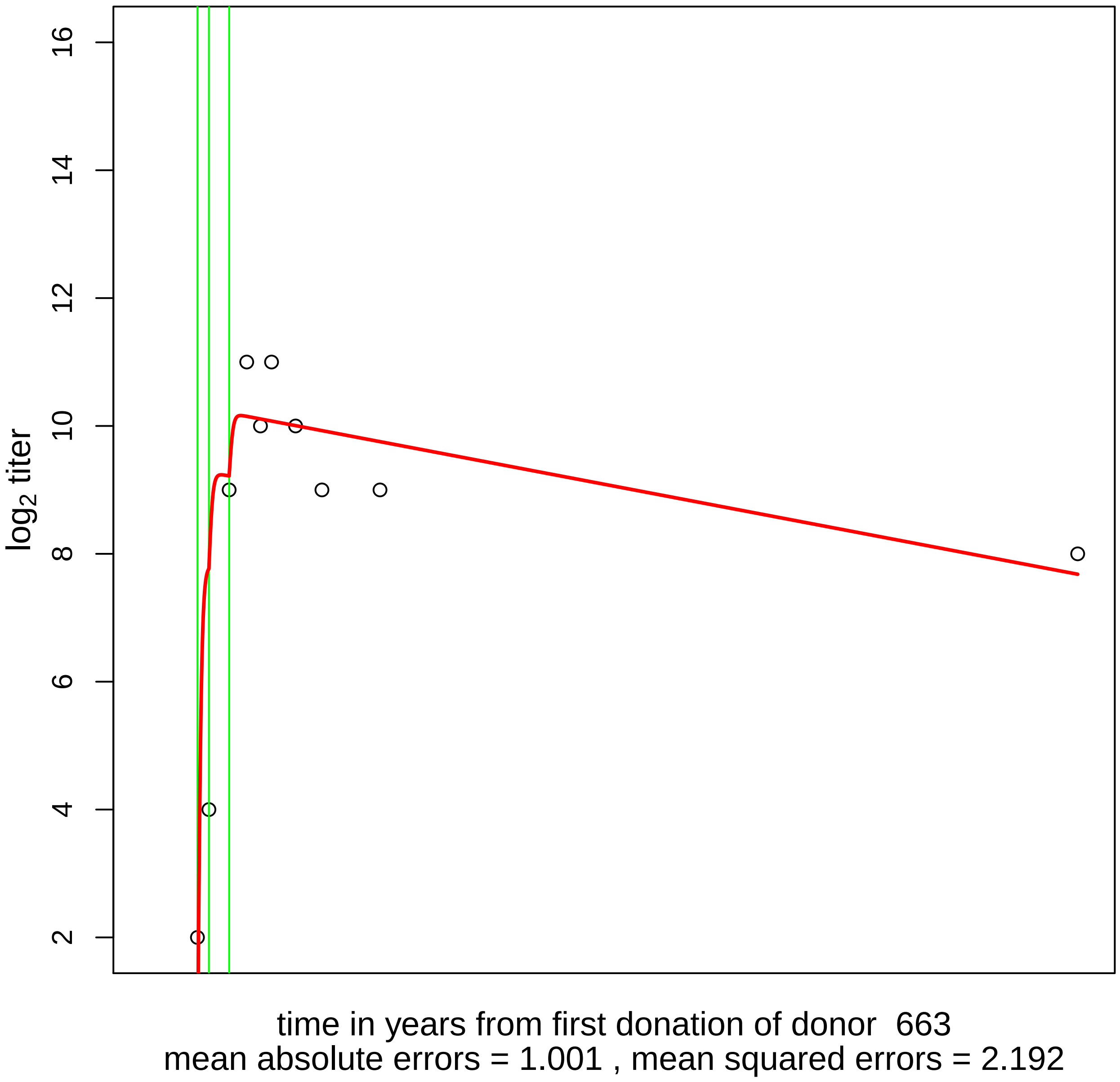
16
14
12
10
8
6
4
2
log2 titer
time in years from first donation of donor 663
mean absolute errors = 1.001 , mean squared errors = 2.192

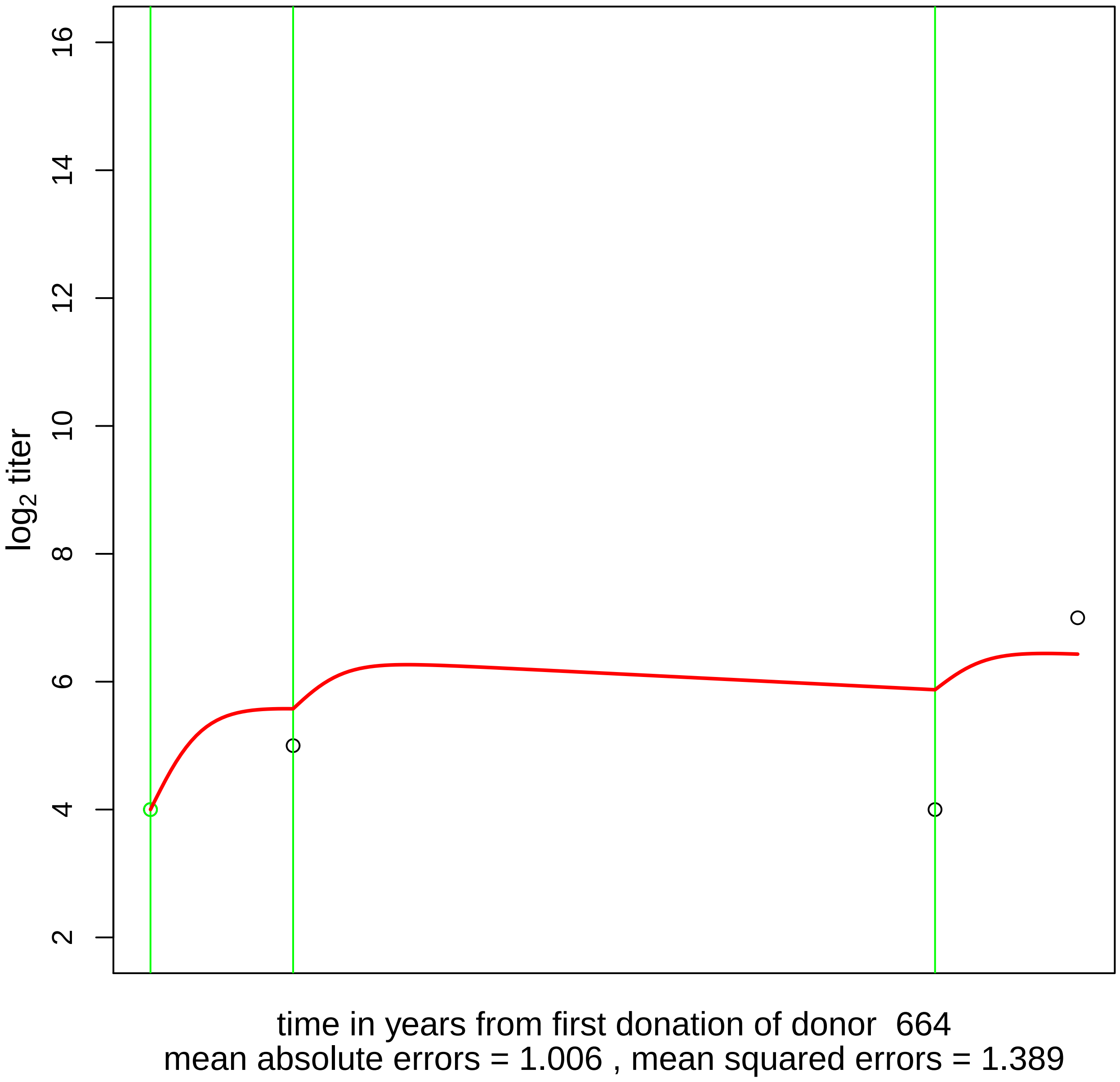

16
14
12
10
8
6
4
2
log2 titer
time in years from first donation of donor 664
mean absolute errors = 1.006 , mean squared errors = 1.389

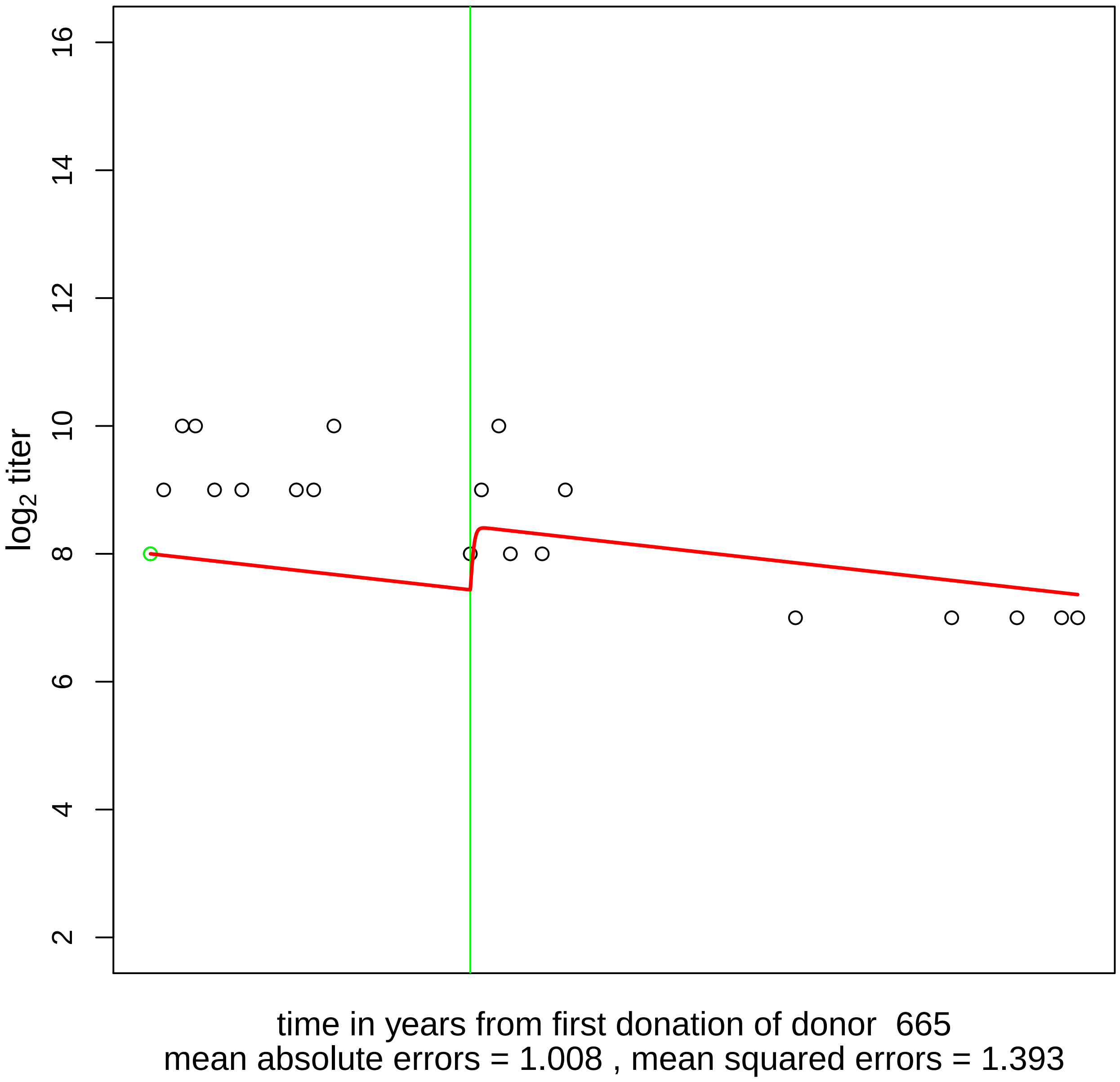

log2 titer
16
14
12
10
8
6
4
2
time in years from first donation of donor 665
mean absolute errors = 1.008 , mean squared errors = 1.393

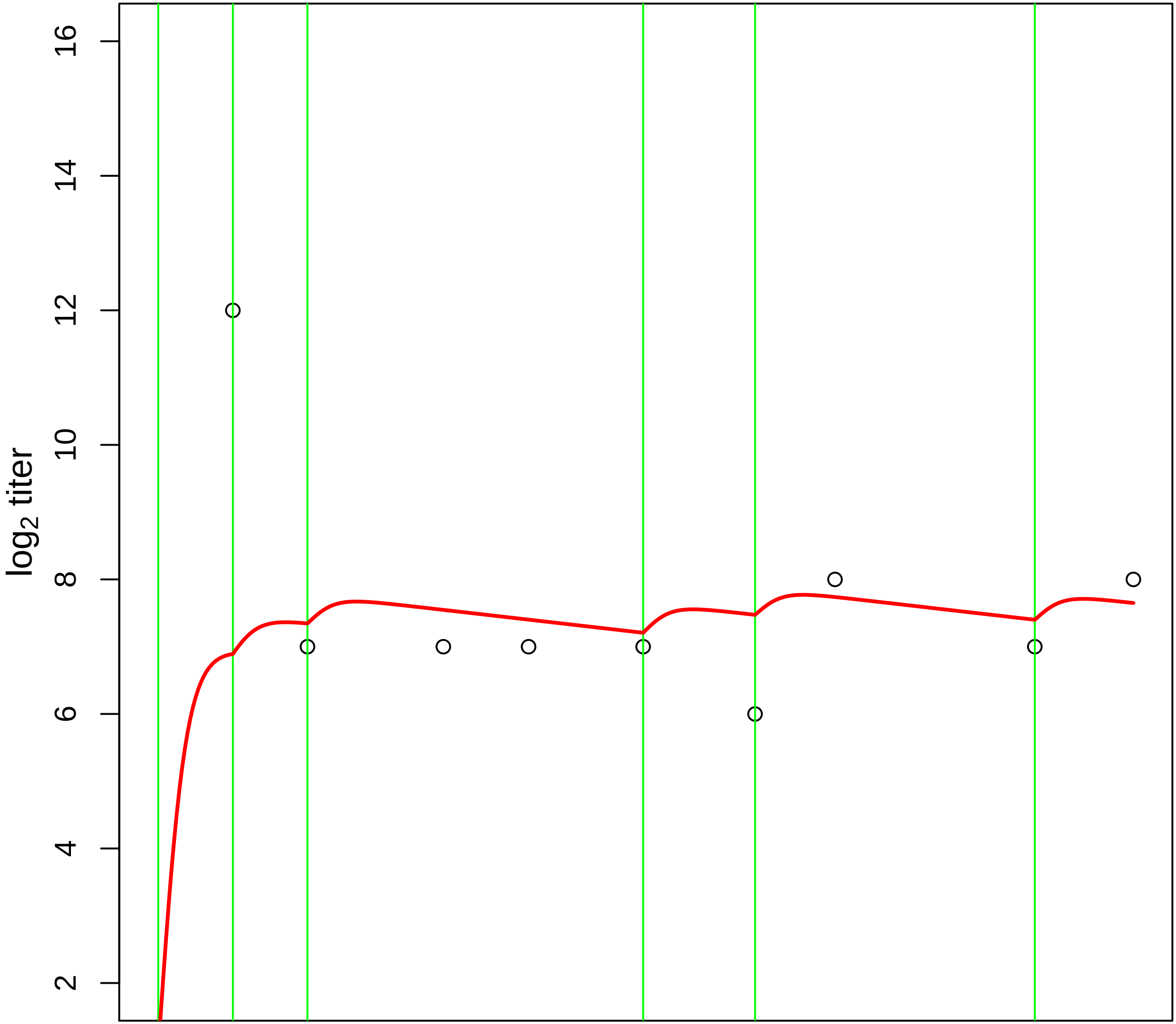

time in years from first donation of donor 666
mean absolute errors = 1.011 , mean squared errors = 3.25

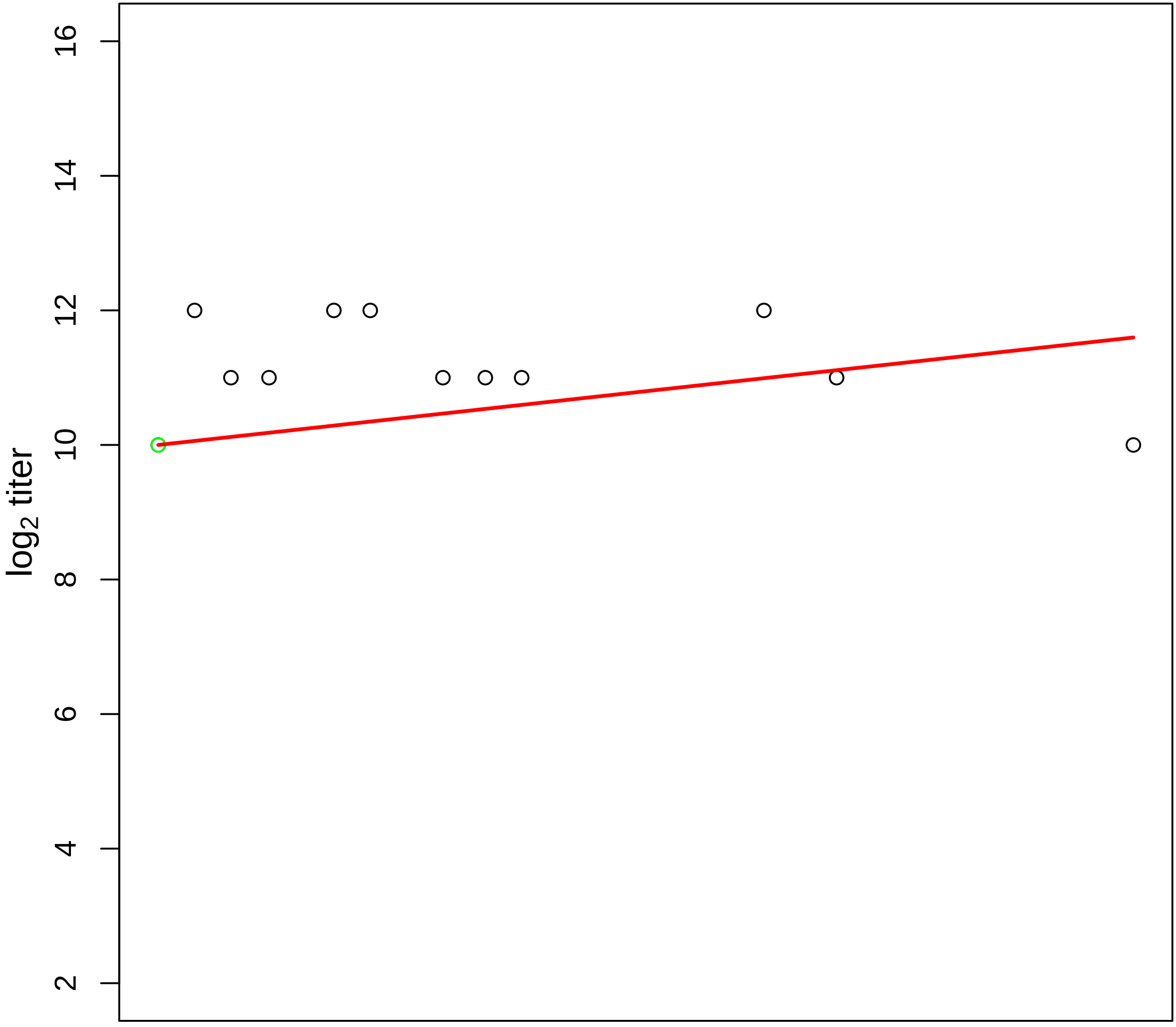

time in years from first donation of donor 667
mean absolute errors = 1.011 , mean squared errors = 1.374

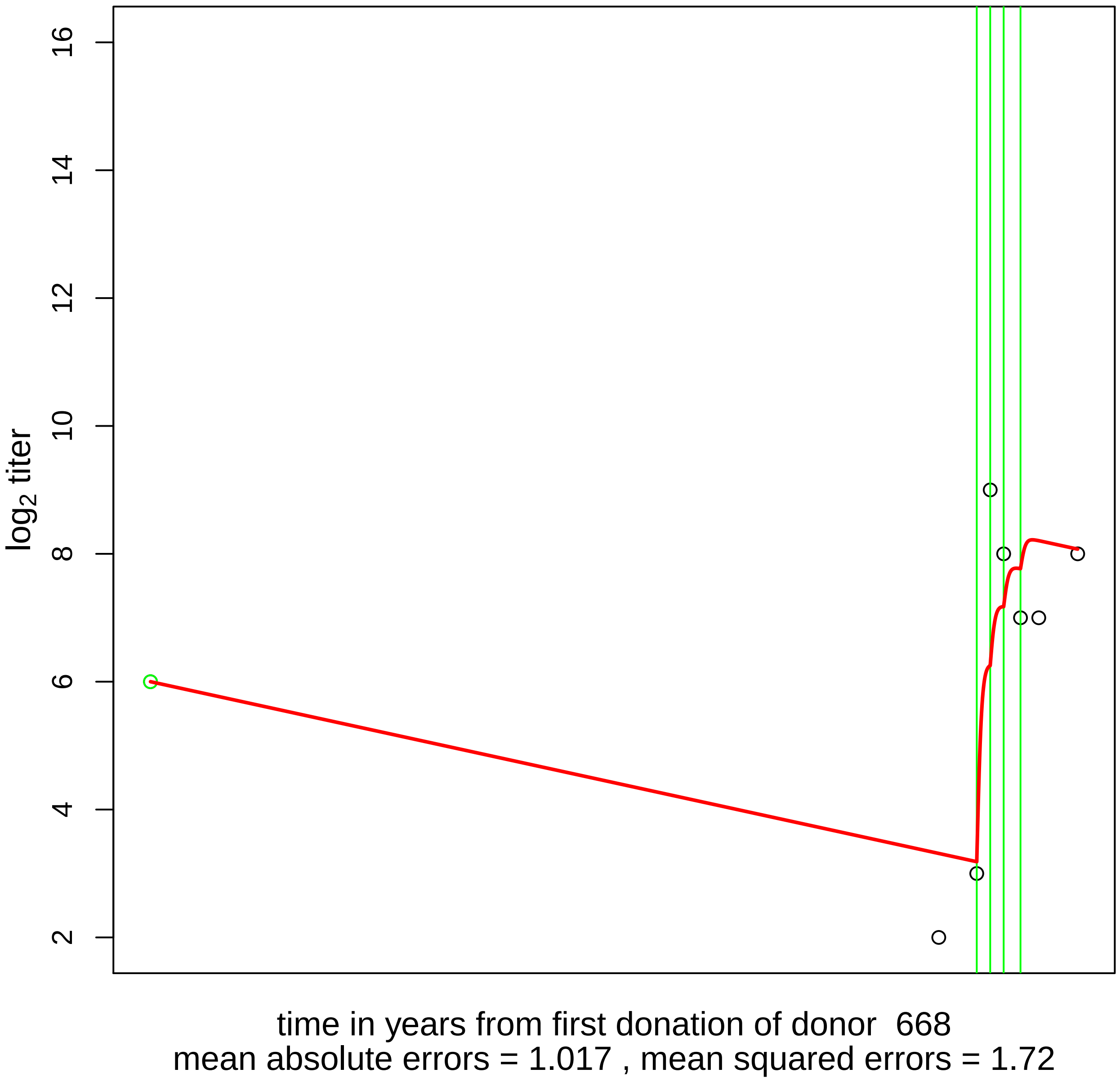

16
14
12
10
8
6
4
2
log2 titer
time in years from first donation of donor 668
mean absolute errors = 1.017 , mean squared errors = 1.72

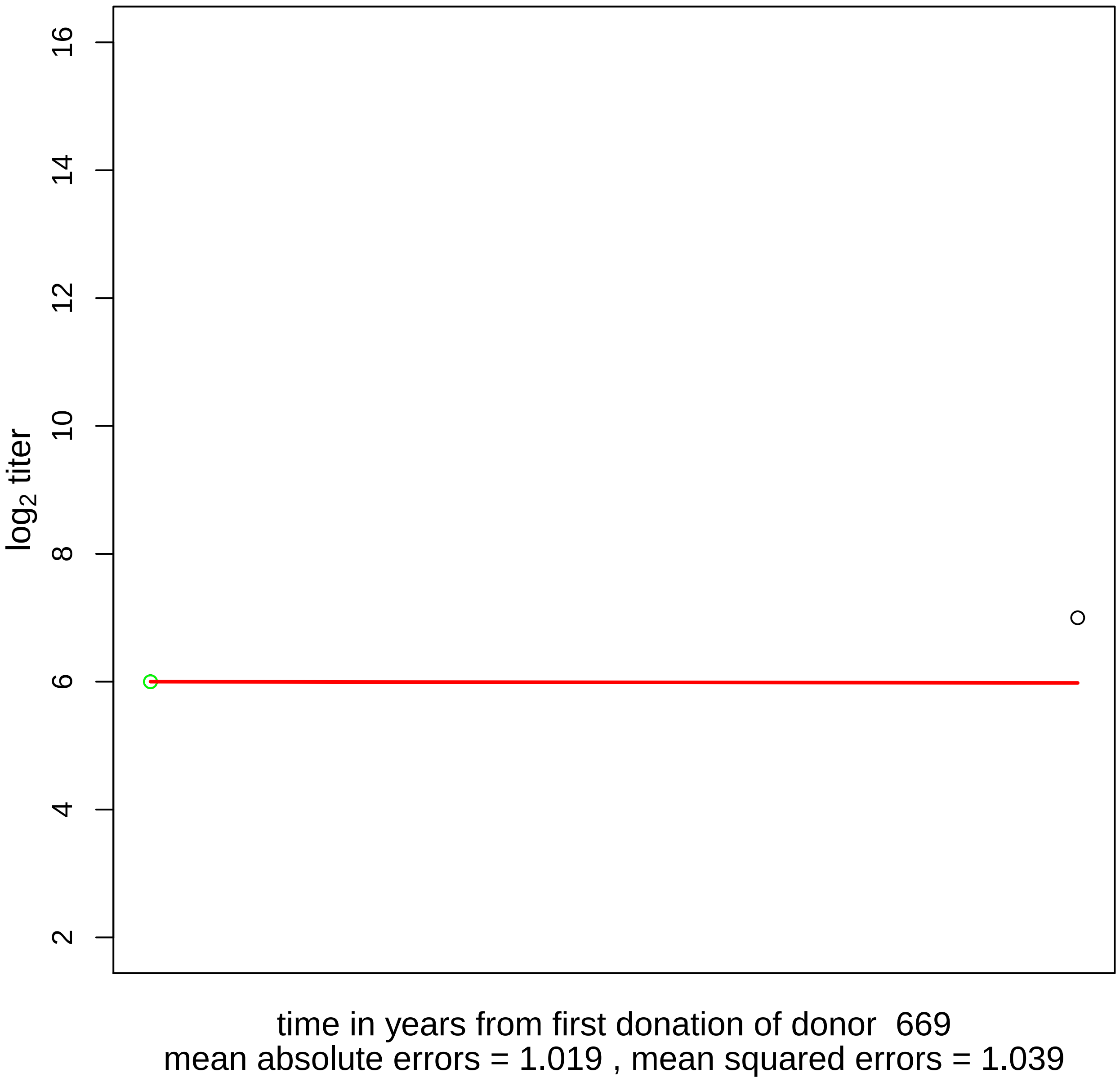

16
14
12
10
8
6
4
2
log2 titer
time in years from first donation of donor 669
mean absolute errors = 1.019 , mean squared errors = 1.039

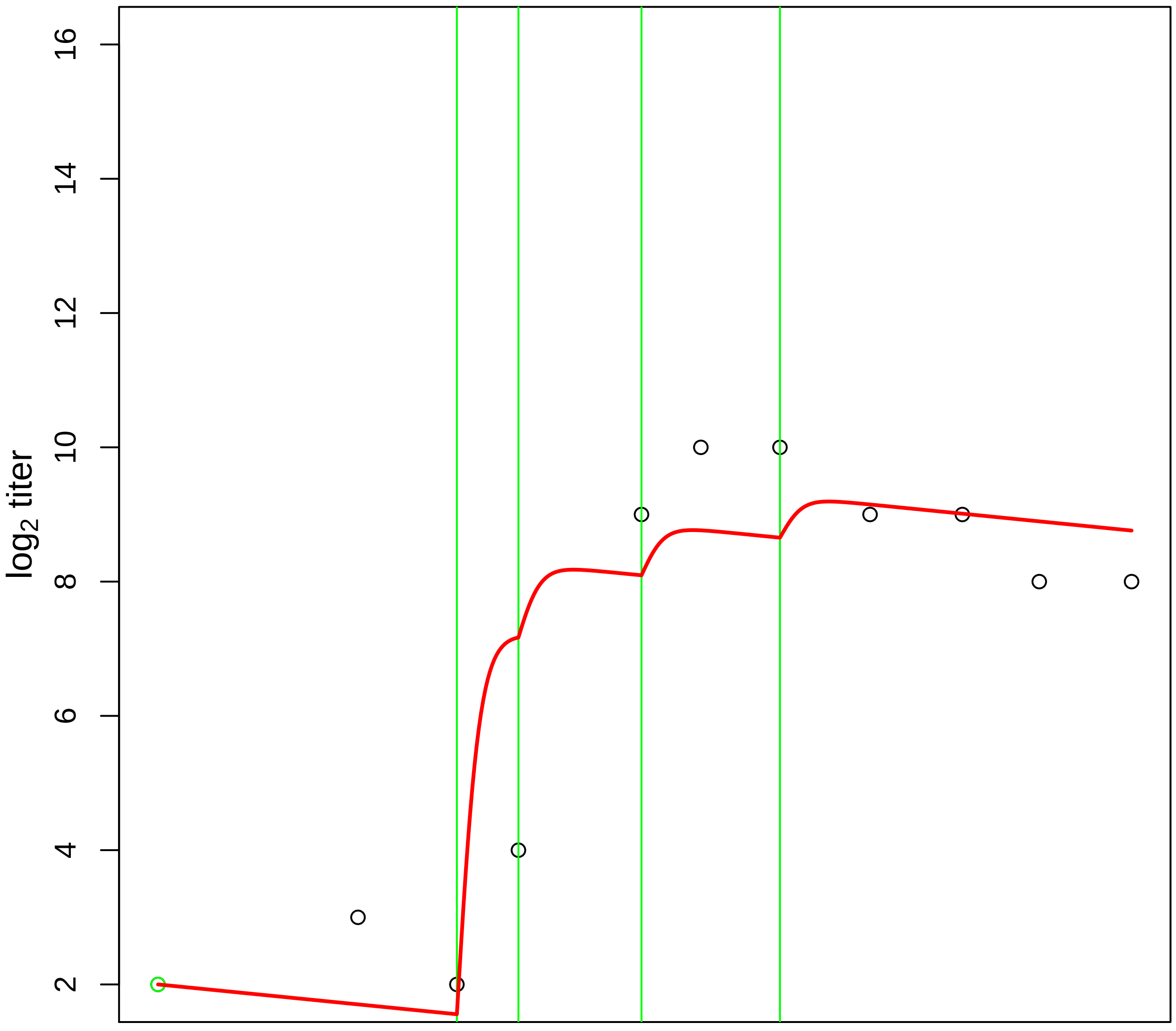

time in years from first donation of donor 670
mean absolute errors = 1.021 , mean squared errors = 1.748

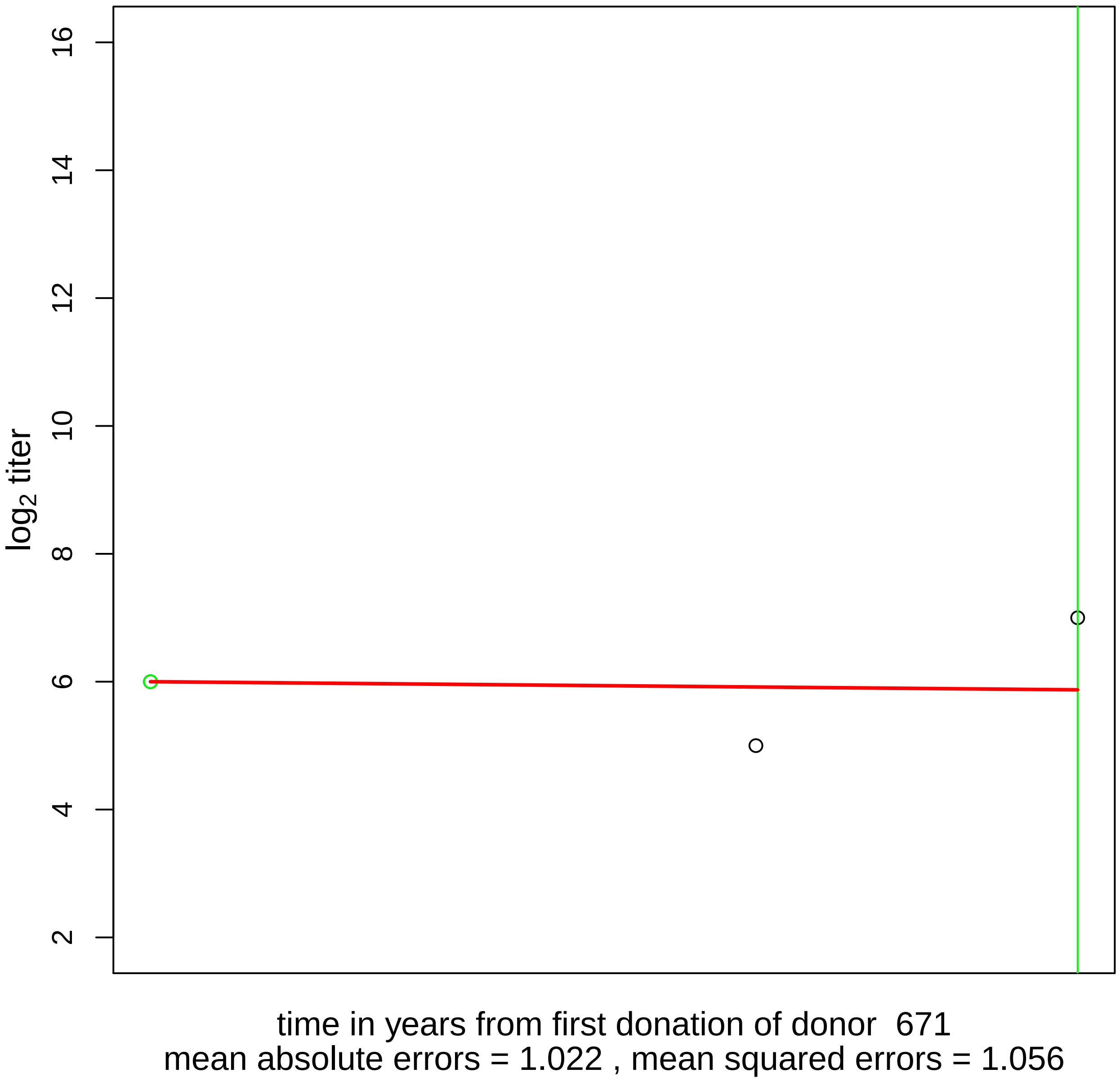

log2 titer
16
14
12
10
8
6
4
2
time in years from first donation of donor 671
mean absolute errors = 1.022 , mean squared errors = 1.056

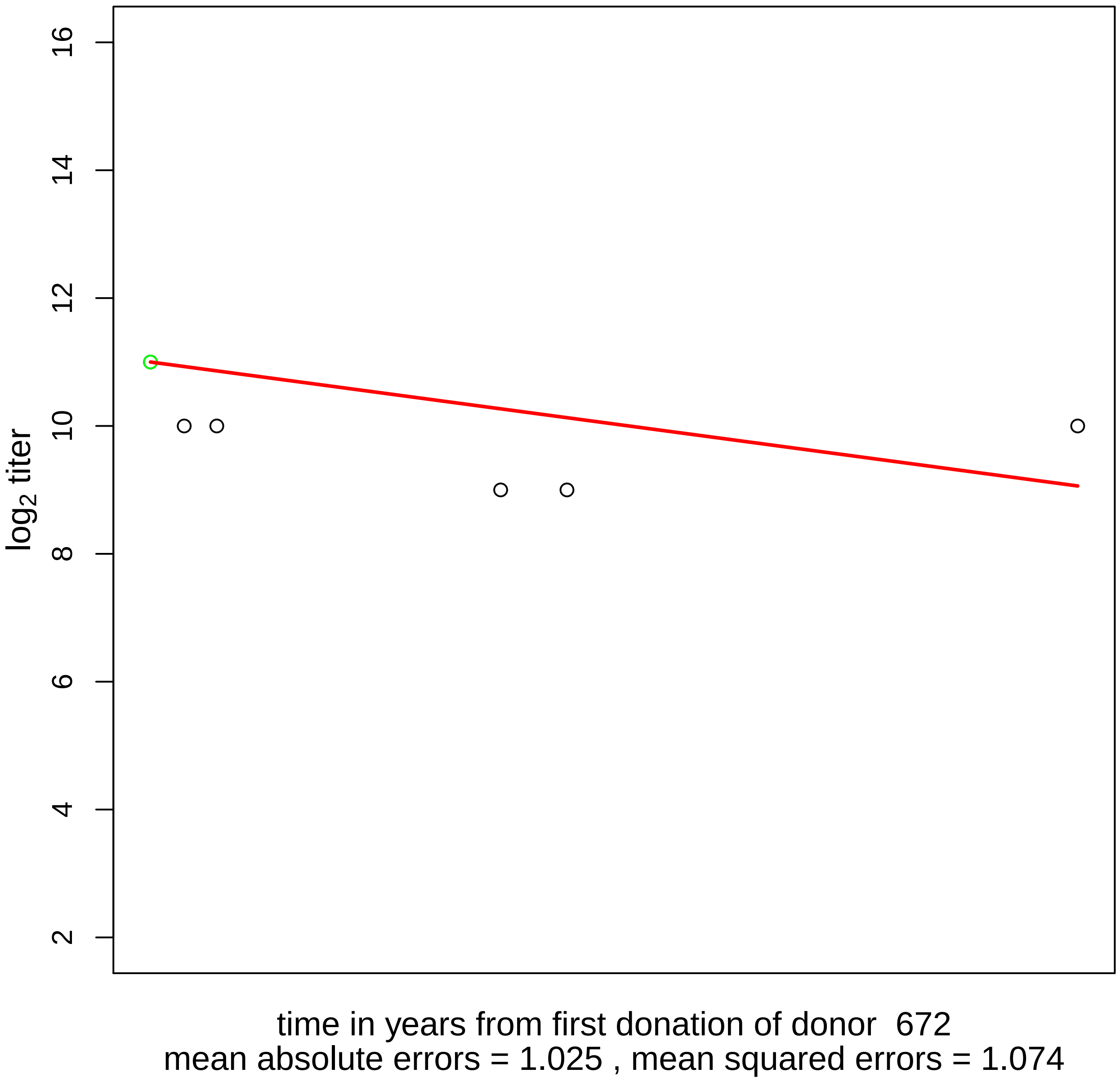

2
4
6
8
10
12
14
16
log2 titer
time in years from first donation of donor 672
mean absolute errors = 1.025 , mean squared errors = 1.074

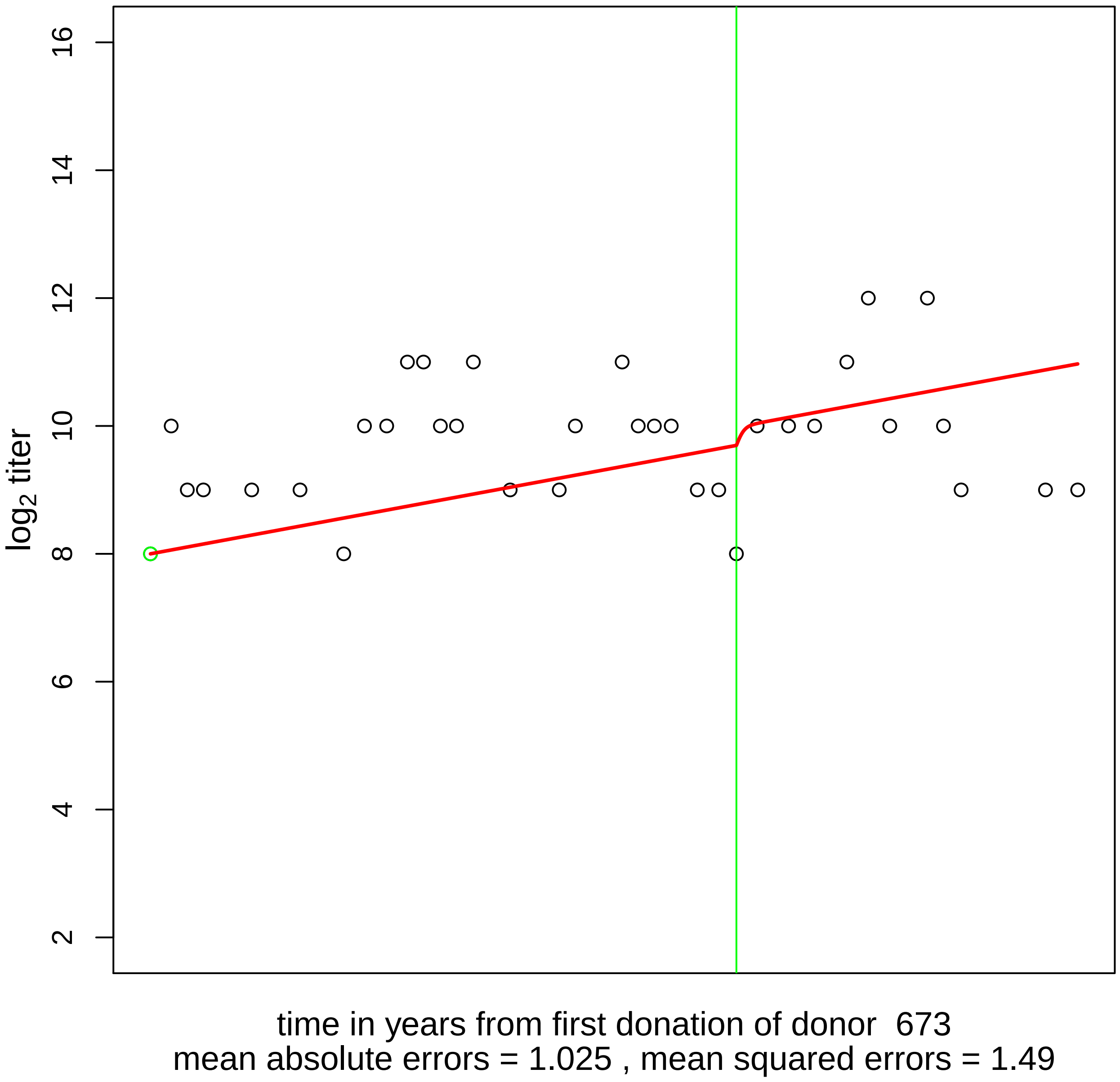

log2 titer
2
4
6
8
10
12
14
16
time in years from first donation of donor 673
mean absolute errors = 1.025 , mean squared errors = 1.49

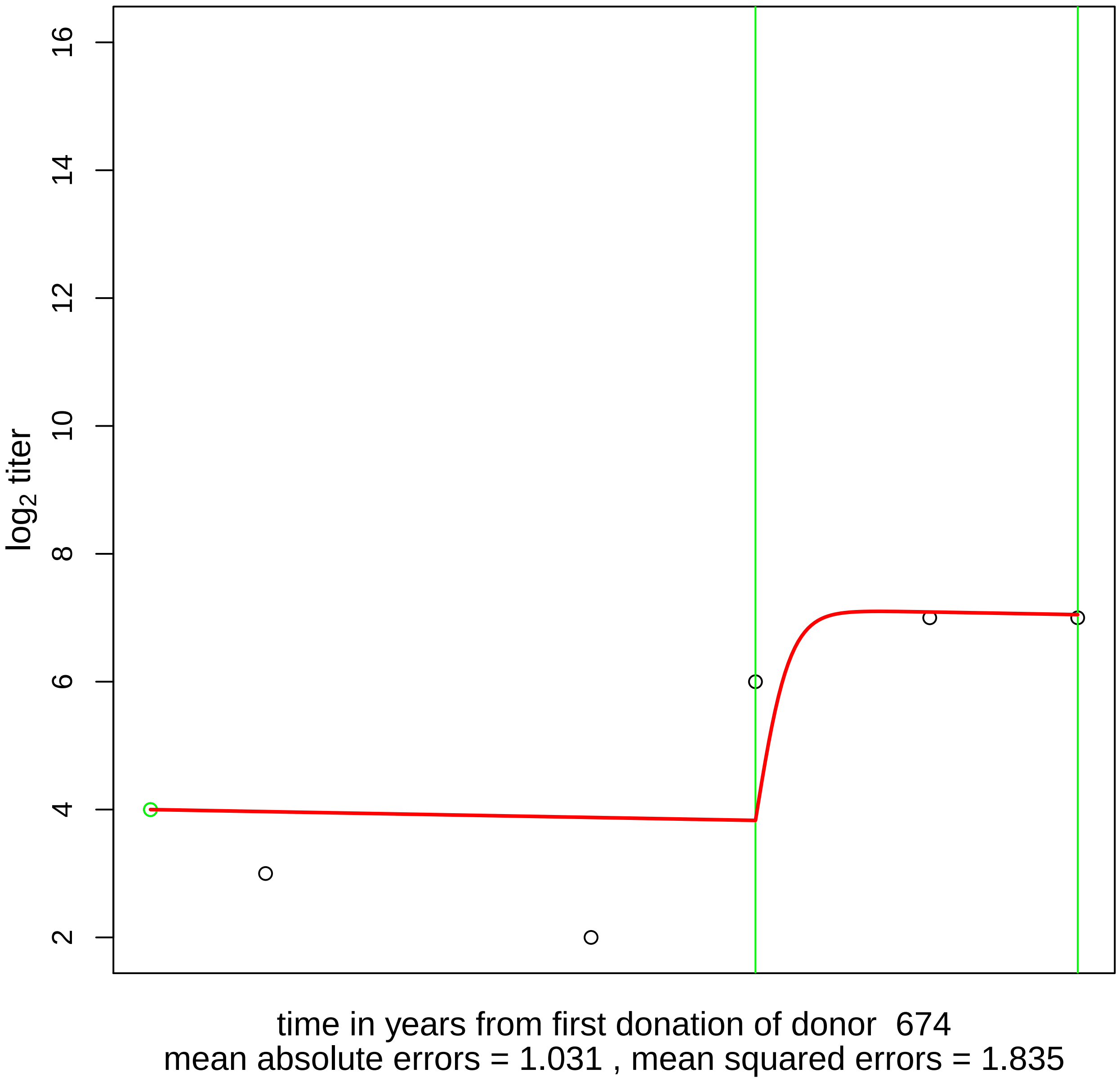
log2 titer
2
4
6
8
10
12
14
16
time in years from first donation of donor 674
mean absolute errors = 1.031 , mean squared errors = 1.835

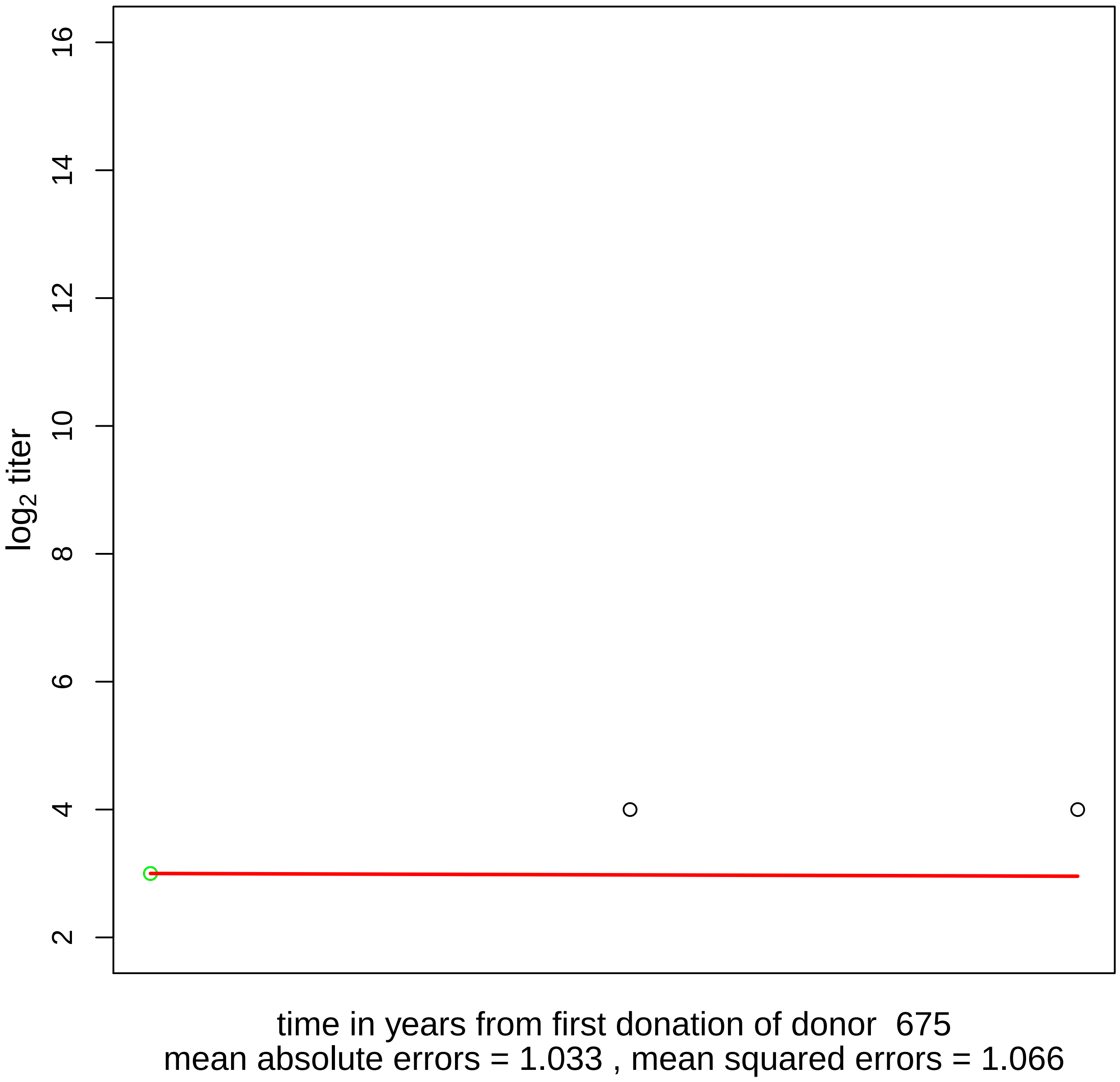

16
14
12
10
8
6
4
2
log2 titer
time in years from first donation of donor 675
mean absolute errors = 1.033 , mean squared errors = 1.066

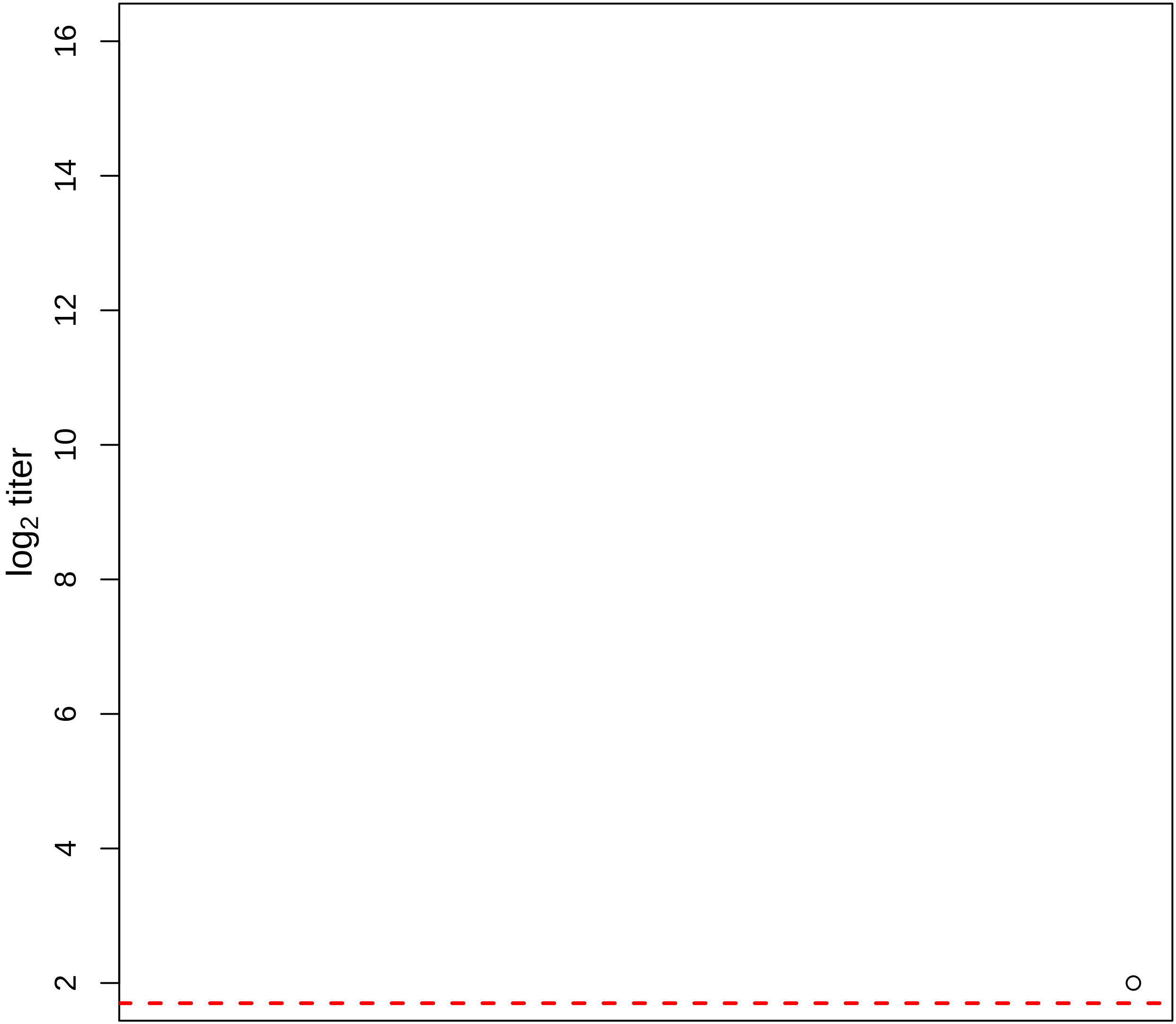

time in years from first donation of donor 676
mean absolute errors = 1.036 , mean squared errors = 1.074

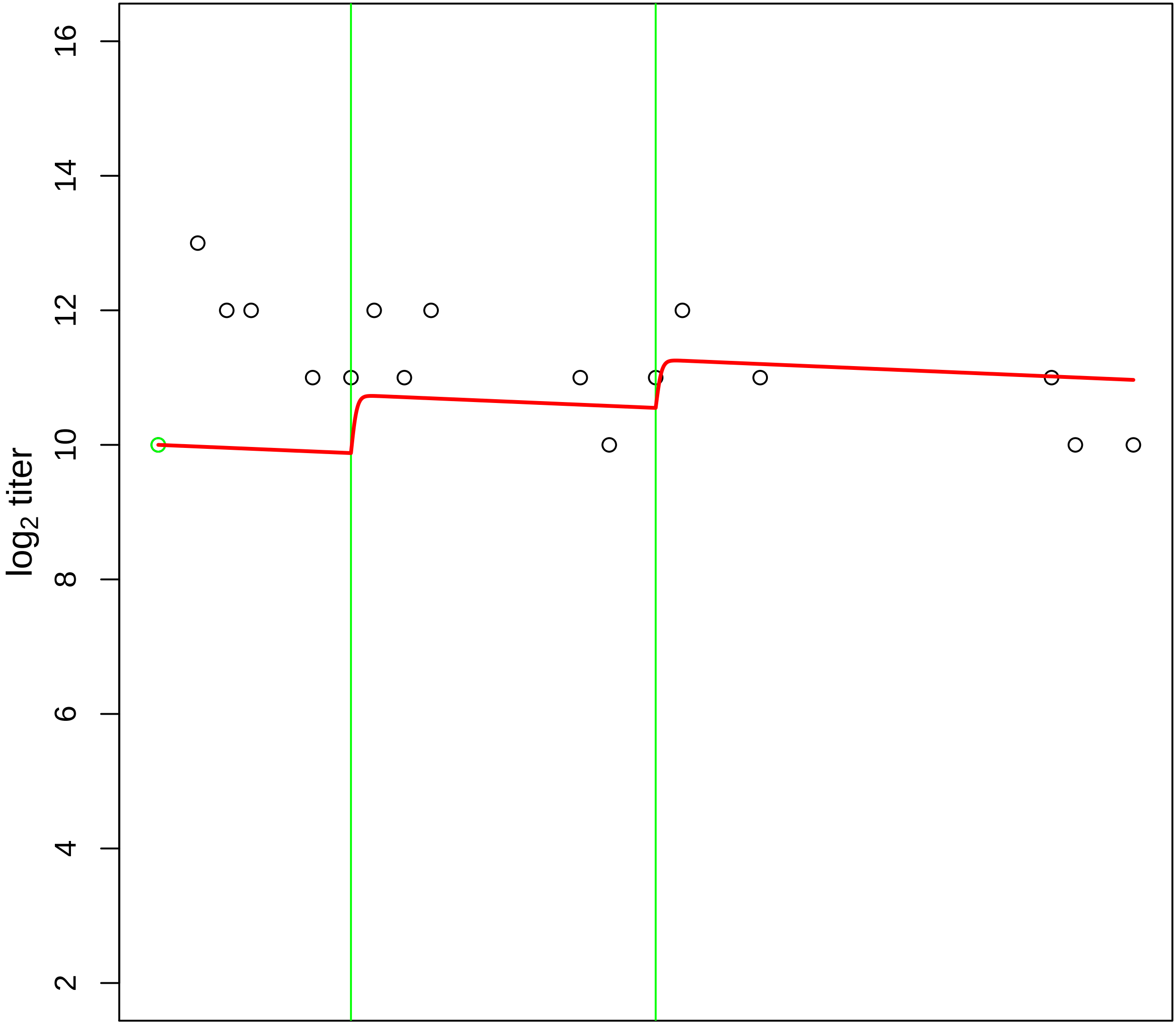

time in years from first donation of donor 677
mean absolute errors = 1.037 , mean squared errors = 1.668

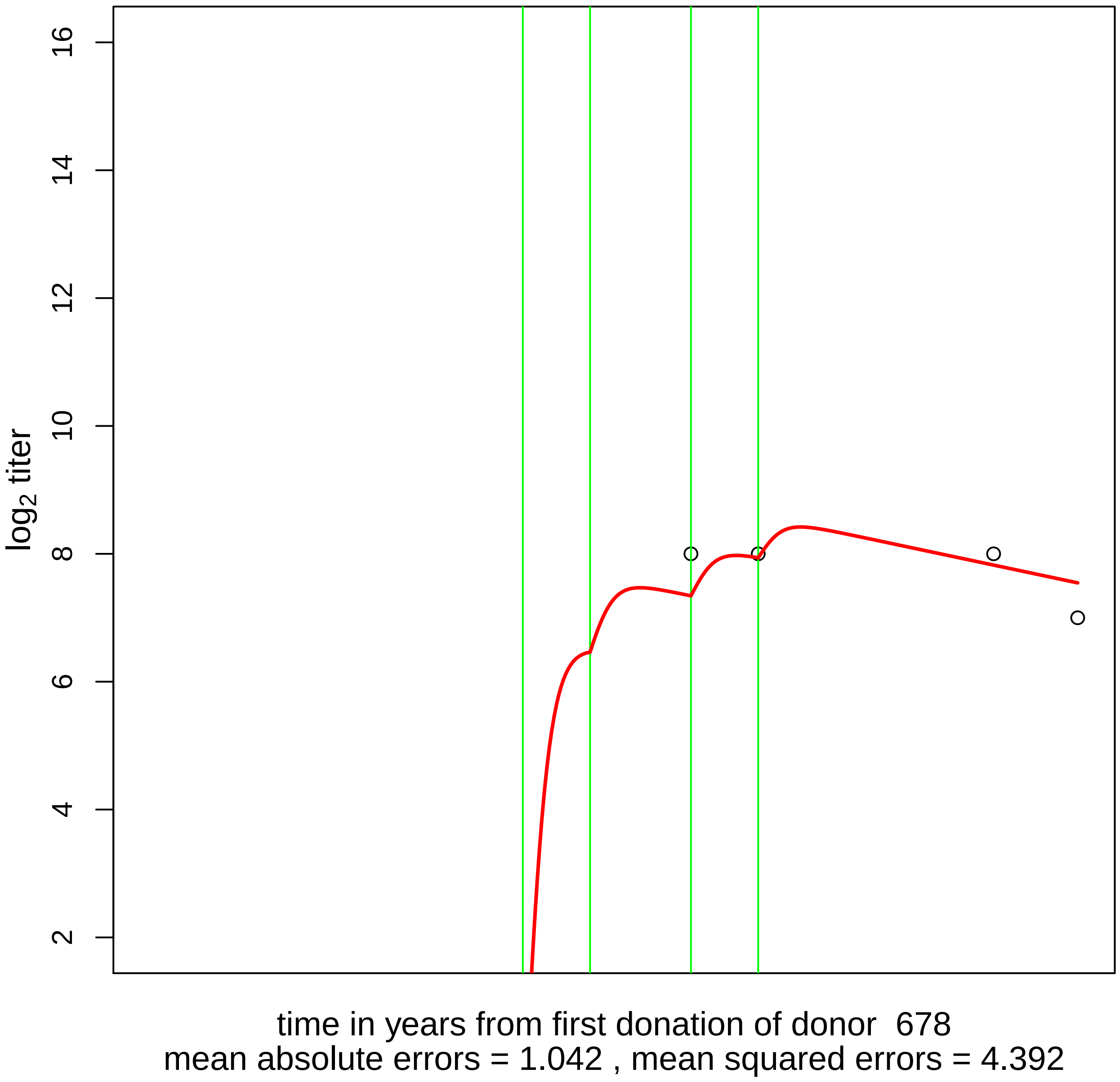

log2 titer
2
4
6
8
10
12
14
16
time in years from first donation of donor 678
mean absolute errors = 1.042 , mean squared errors = 4.392

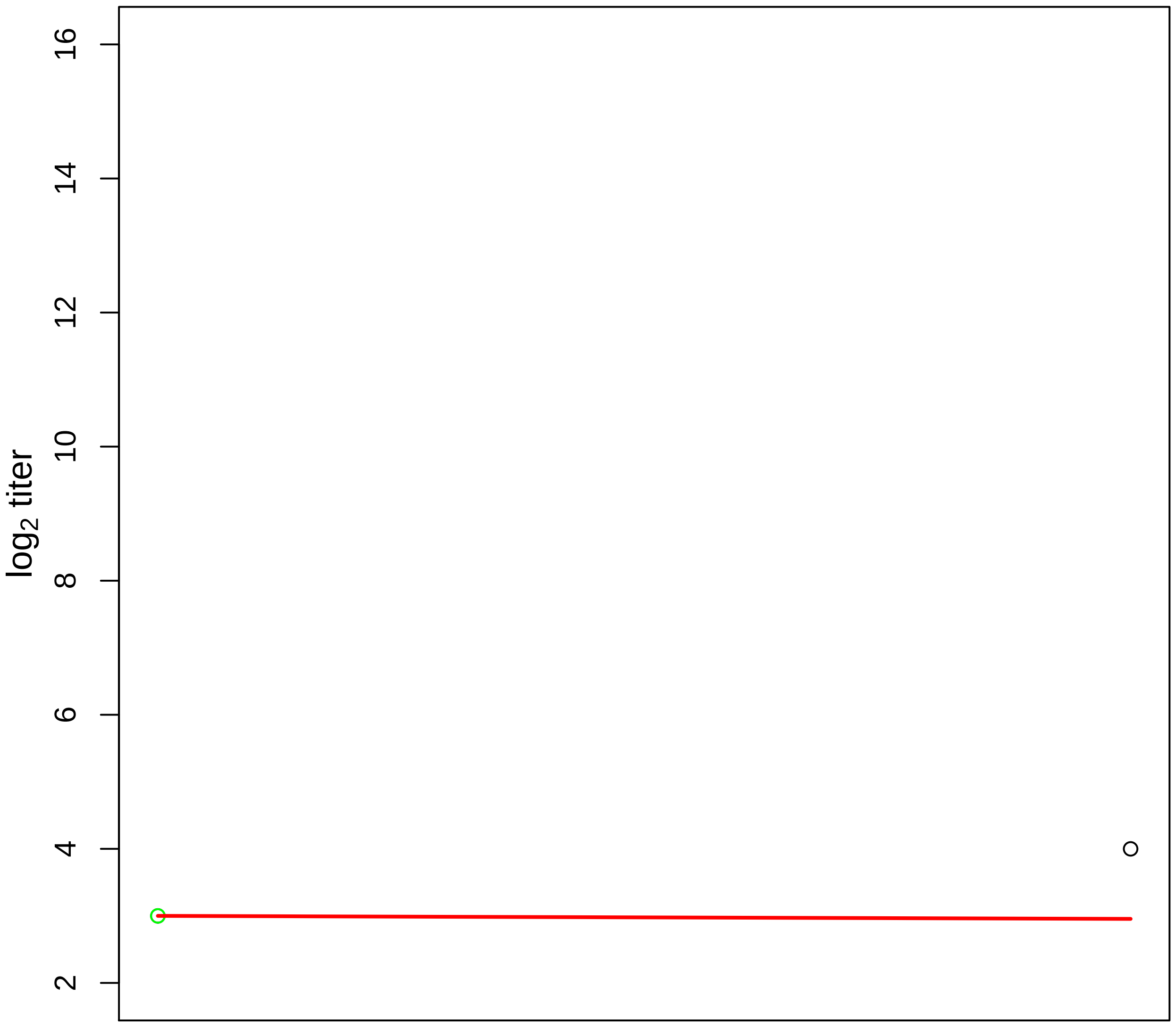

time in years from first donation of donor 679
mean absolute errors = 1.045 , mean squared errors = 1.092

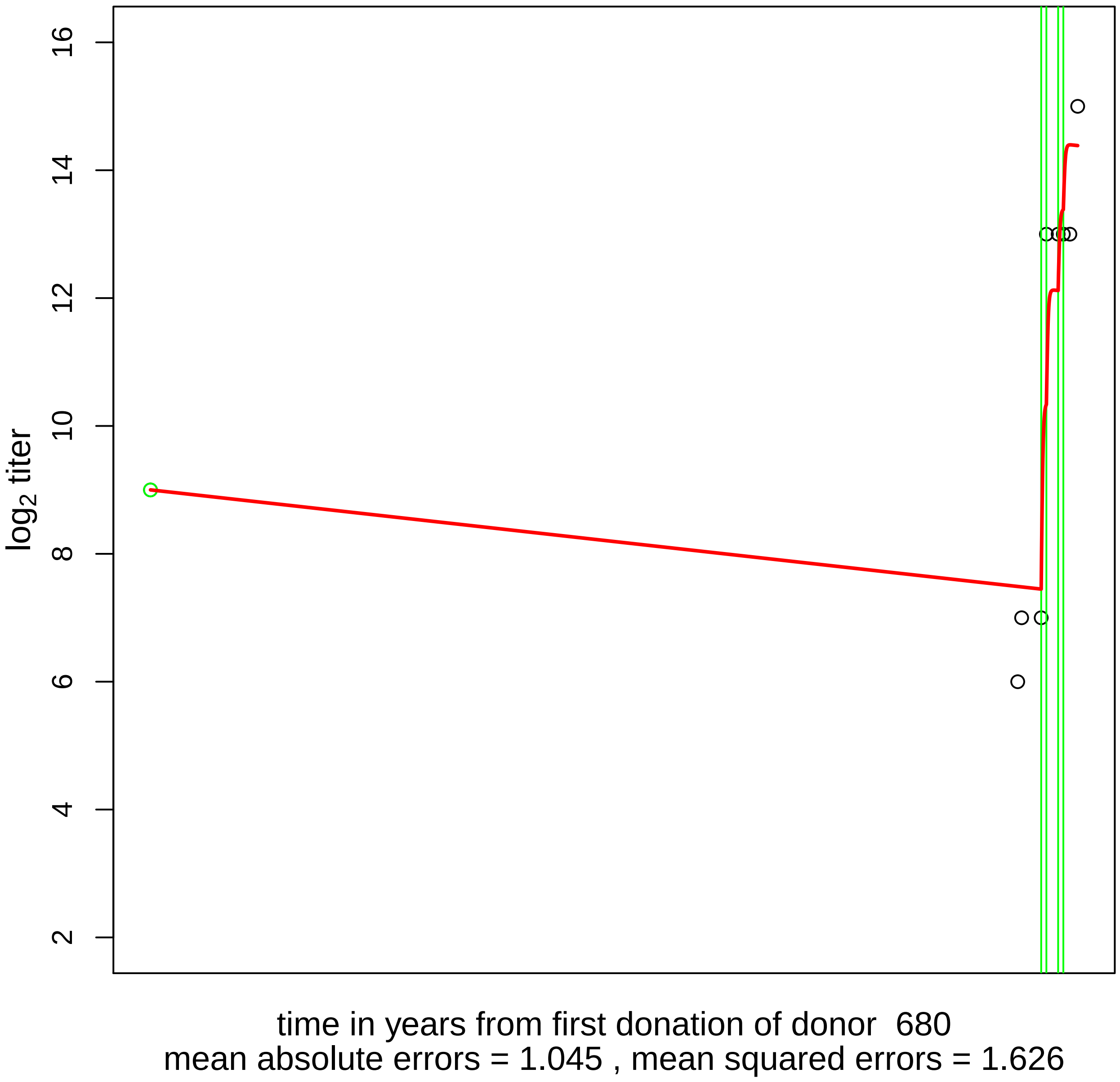

16
14
12
10
8
6
4
2
log2 titer
time in years from first donation of donor 680
mean absolute errors = 1.045 , mean squared errors = 1.626

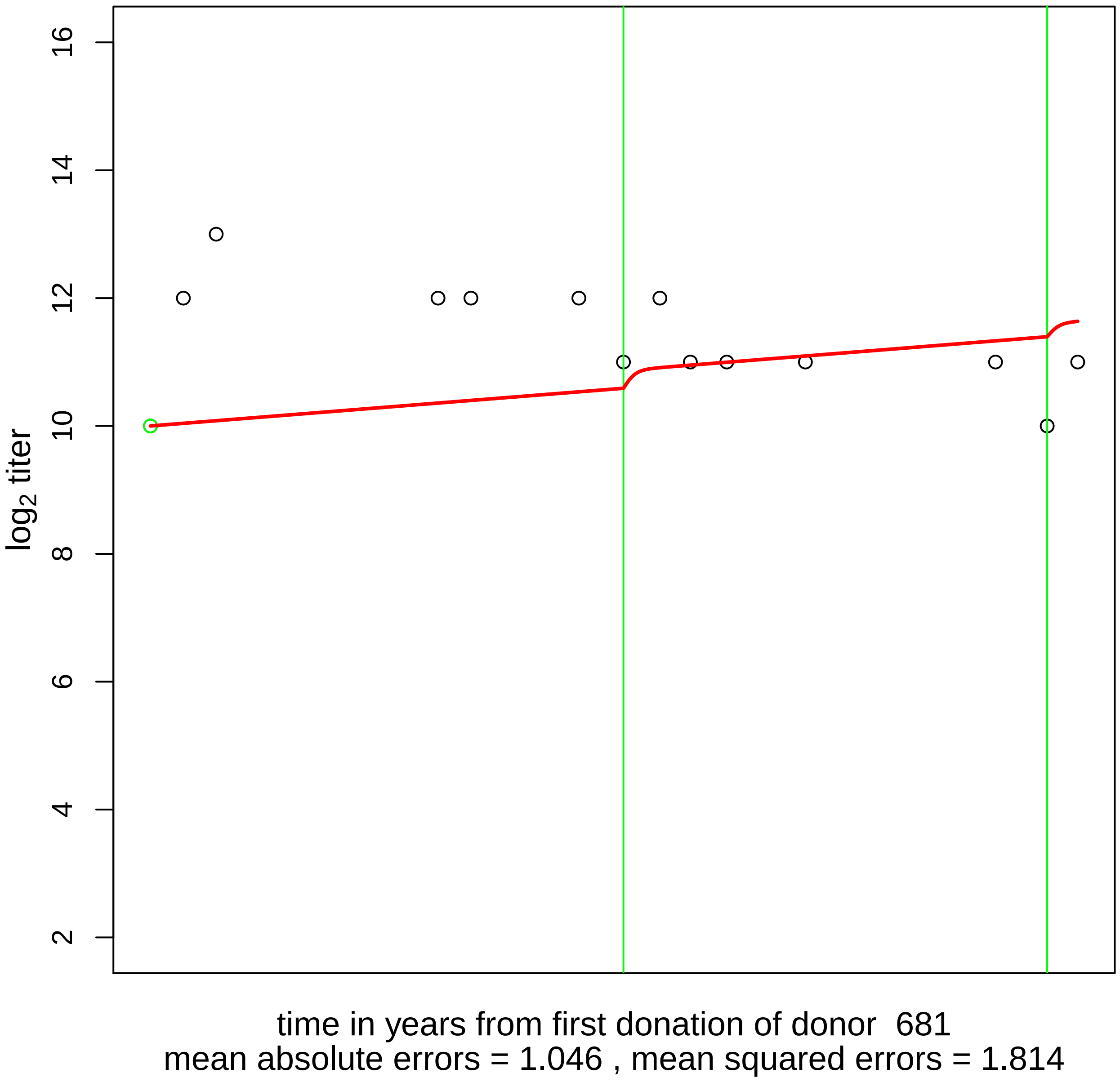

16
14
12
10
8
6
4
2
log2 titer
time in years from first donation of donor 681
mean absolute errors = 1.046 , mean squared errors = 1.814

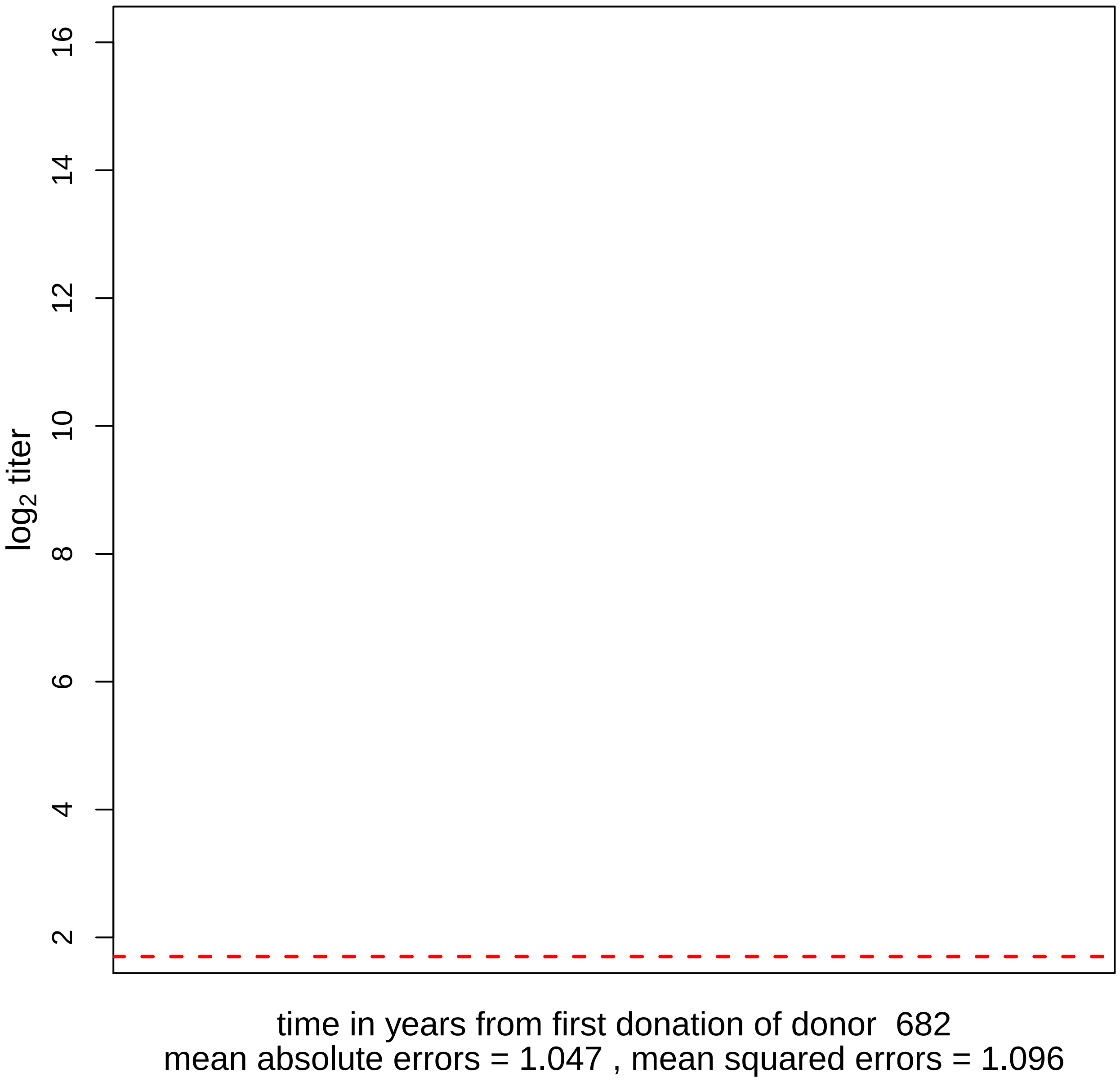

log2 titer
16
14
12
10
8
6
4
2
time in years from first donation of donor 682
mean absolute errors = 1.047 , mean squared errors = 1.096

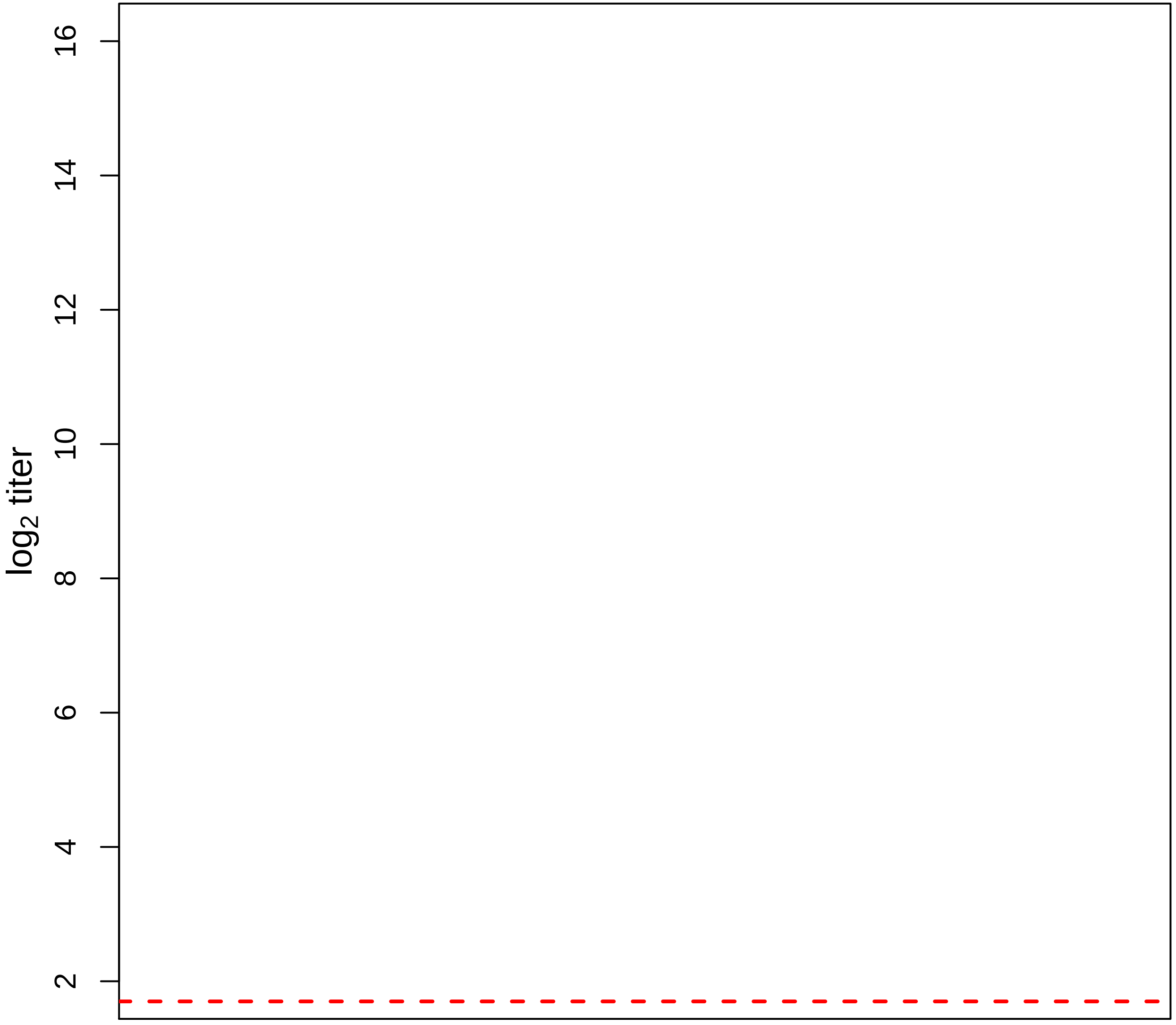

time in years from first donation of donor 683
mean absolute errors = 1.047 , mean squared errors = 1.097

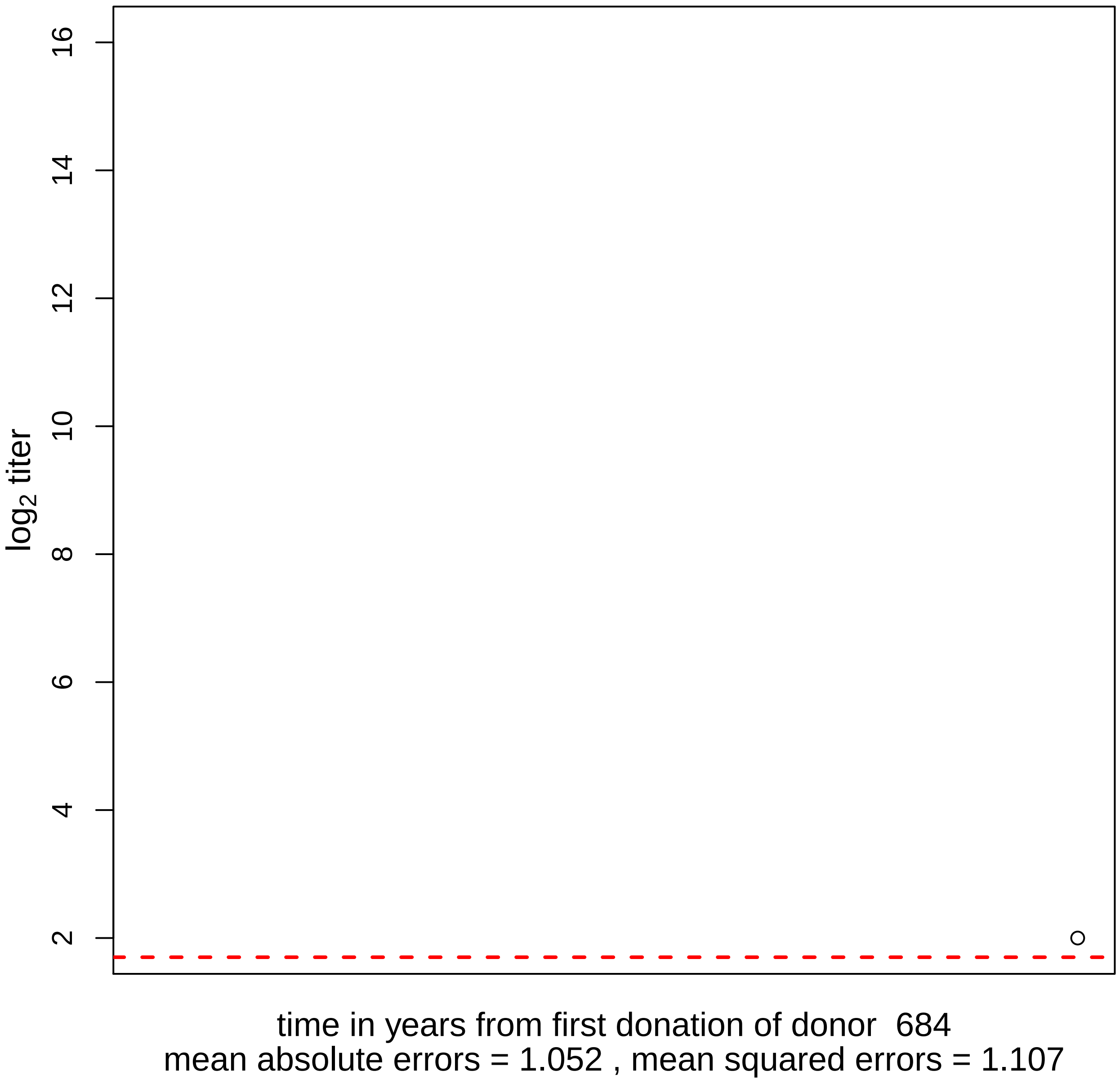

16
14
12
10
8
6
4
2
log2 titer
time in years from first donation of donor 684
mean absolute errors = 1.052 , mean squared errors = 1.107

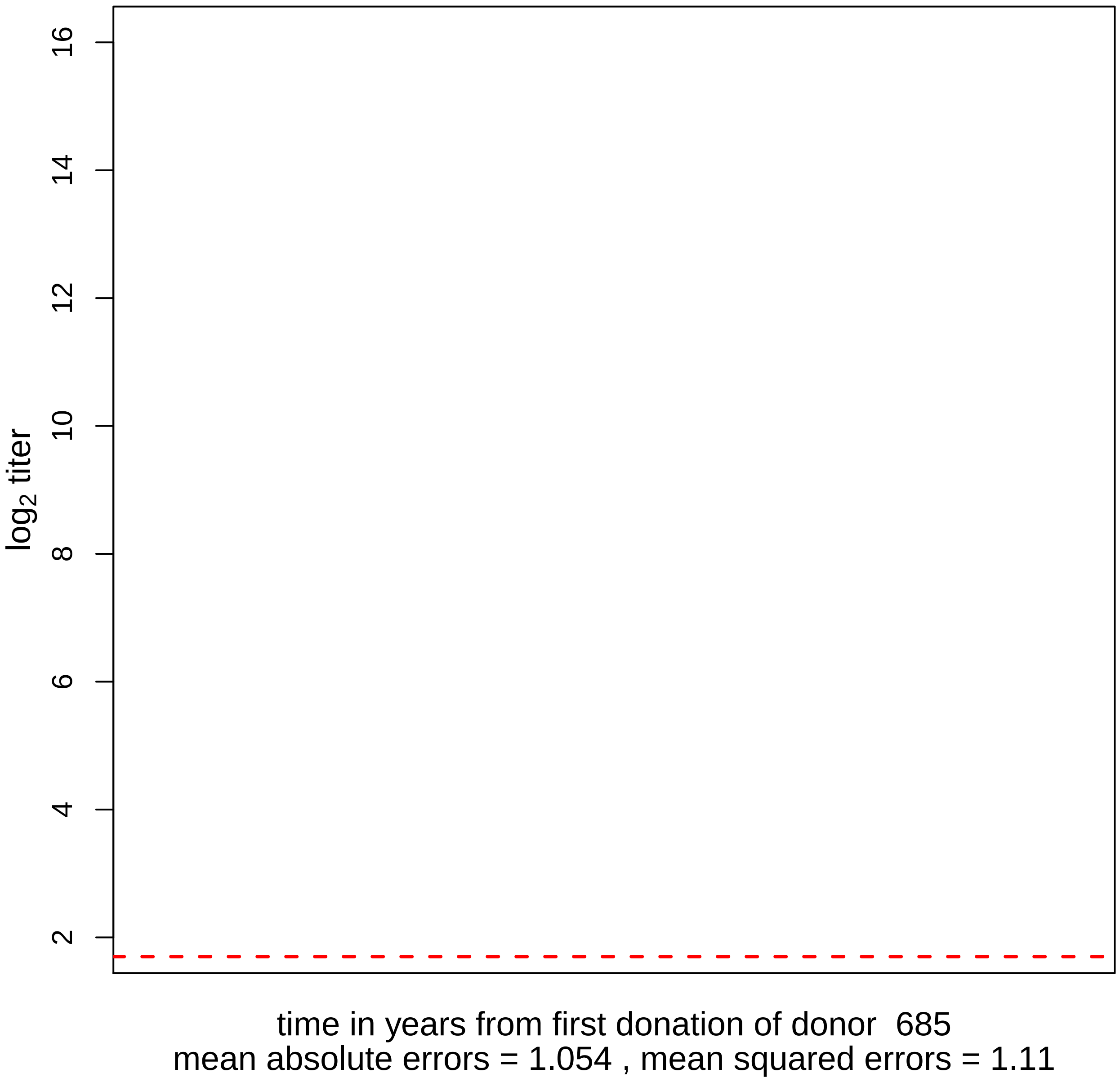

16
14
12
10
8
6
4
2
log2 titer
time in years from first donation of donor 685
mean absolute errors = 1.054 , mean squared errors = 1.11

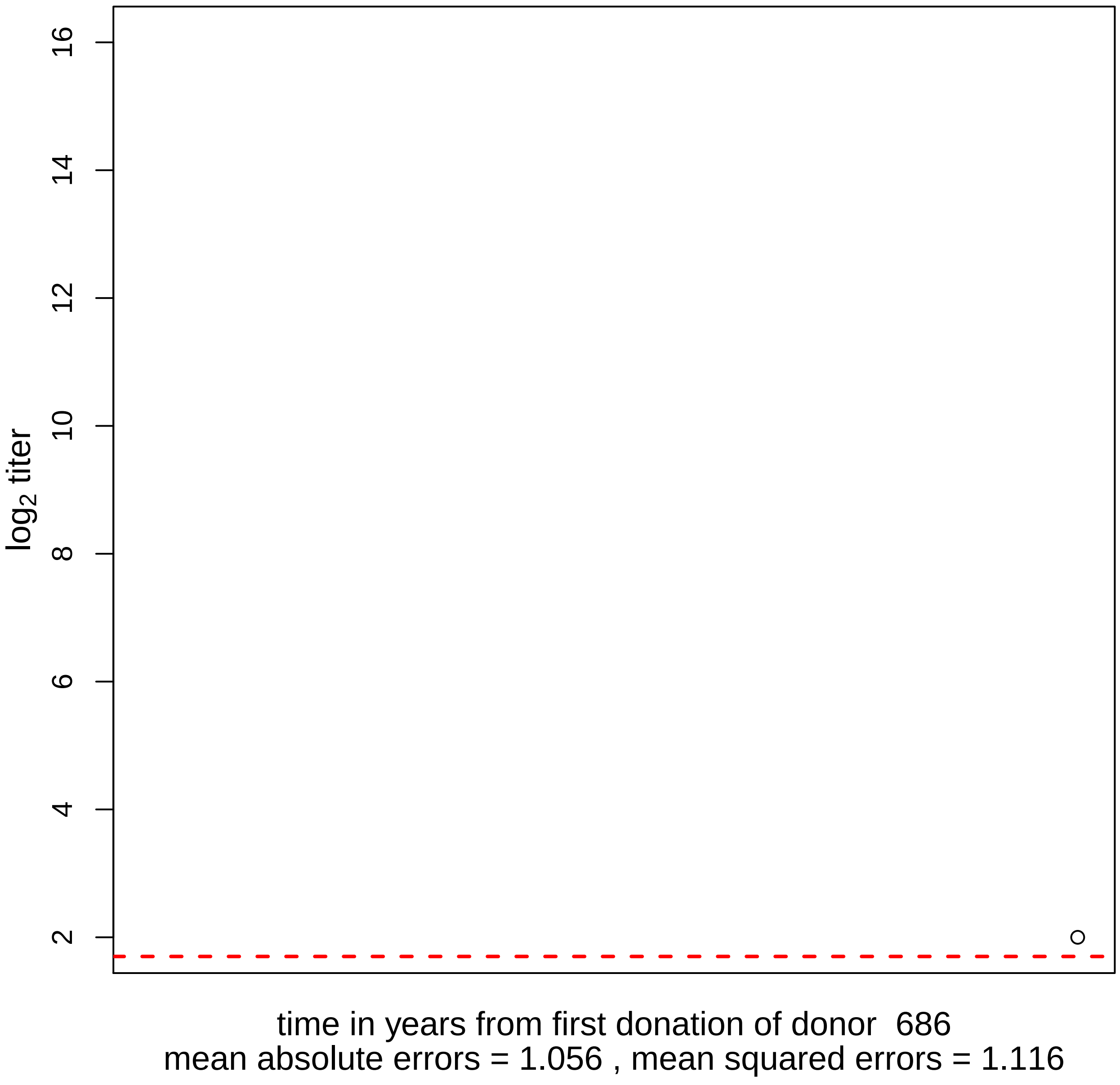

log2 titer
16
14
12
10
8
6
4
2
time in years from first donation of donor 686
mean absolute errors = 1.056 , mean squared errors = 1.116

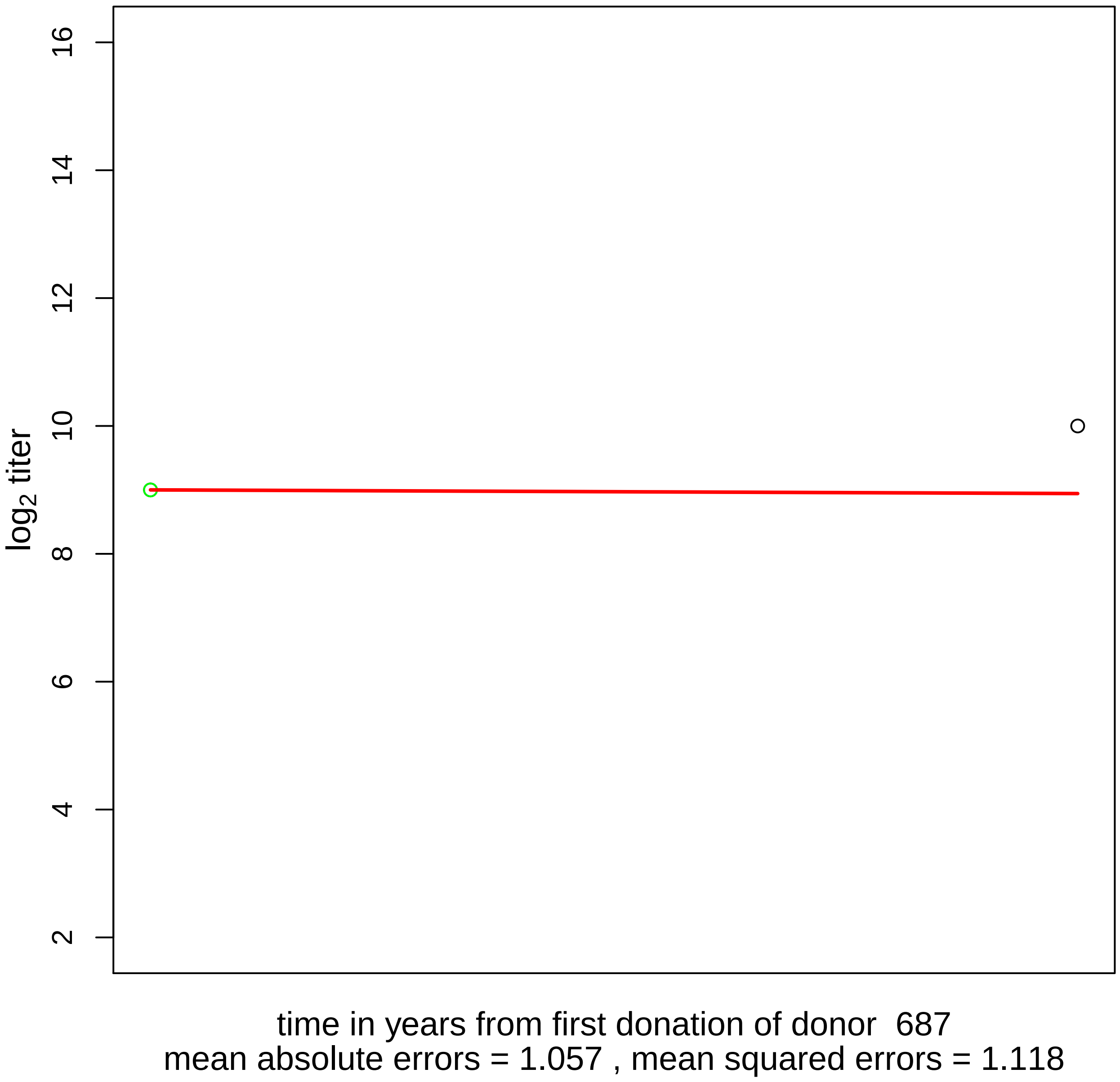
16
14
12
10
8
6
4
2
log2 titer
time in years from first donation of donor 687
mean absolute errors = 1.057 , mean squared errors = 1.118

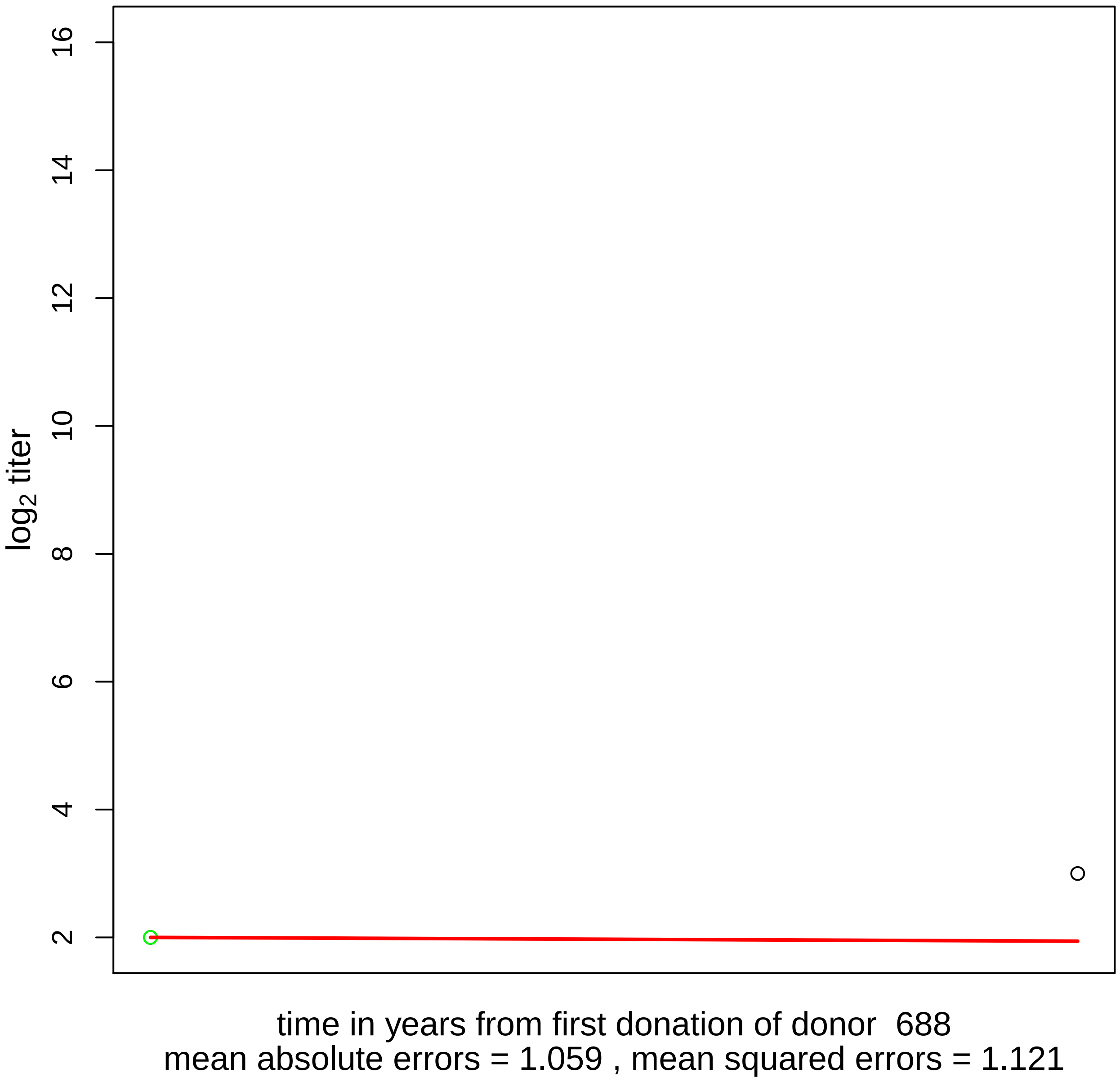
2
4
6
8
10
12
14
16
$\log_2$ titer
time in years from first donation of donor 688
mean absolute errors = 1.059 , mean squared errors = 1.121

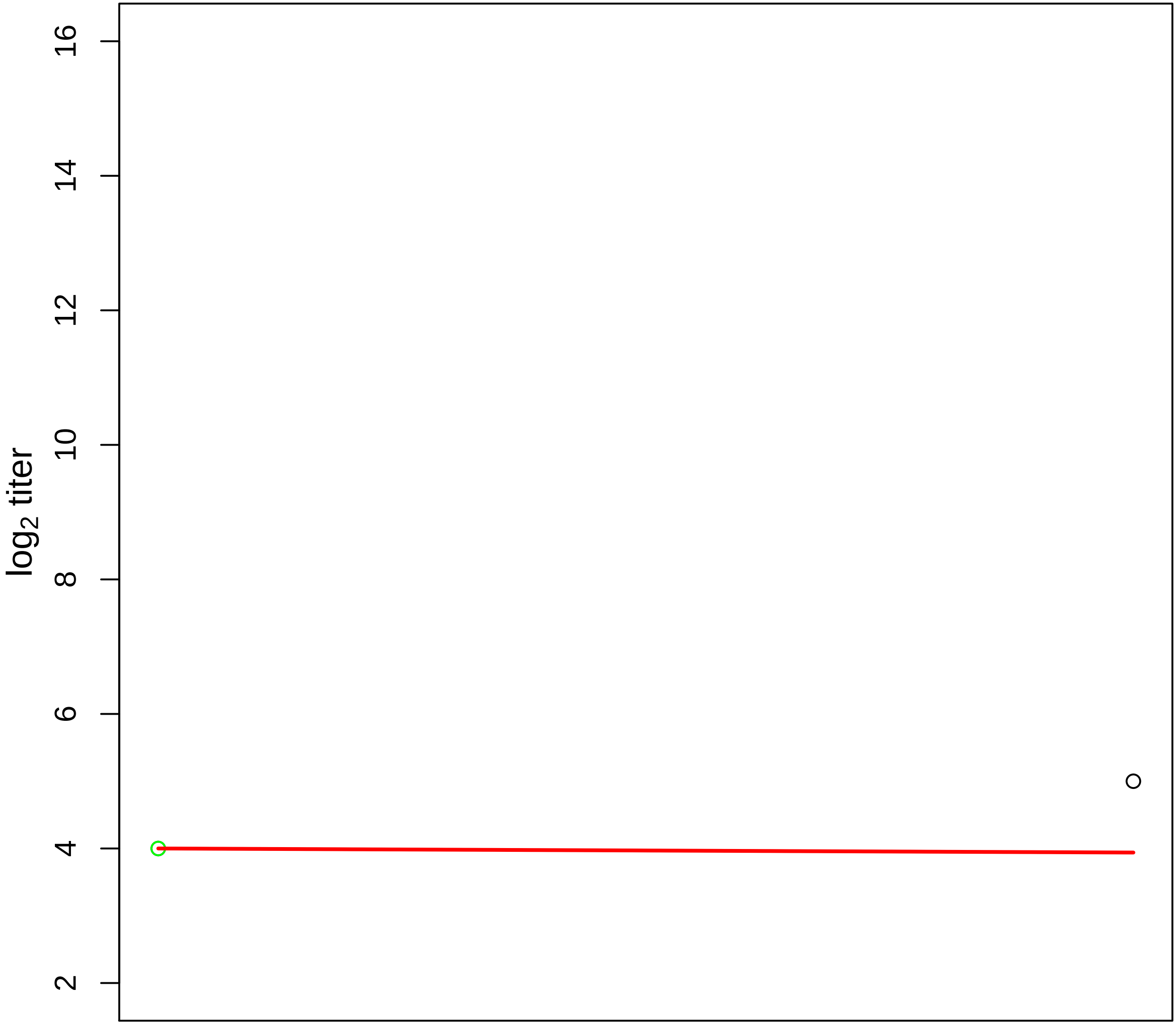

time in years from first donation of donor 689
mean absolute errors = 1.06 , mean squared errors = 1.124

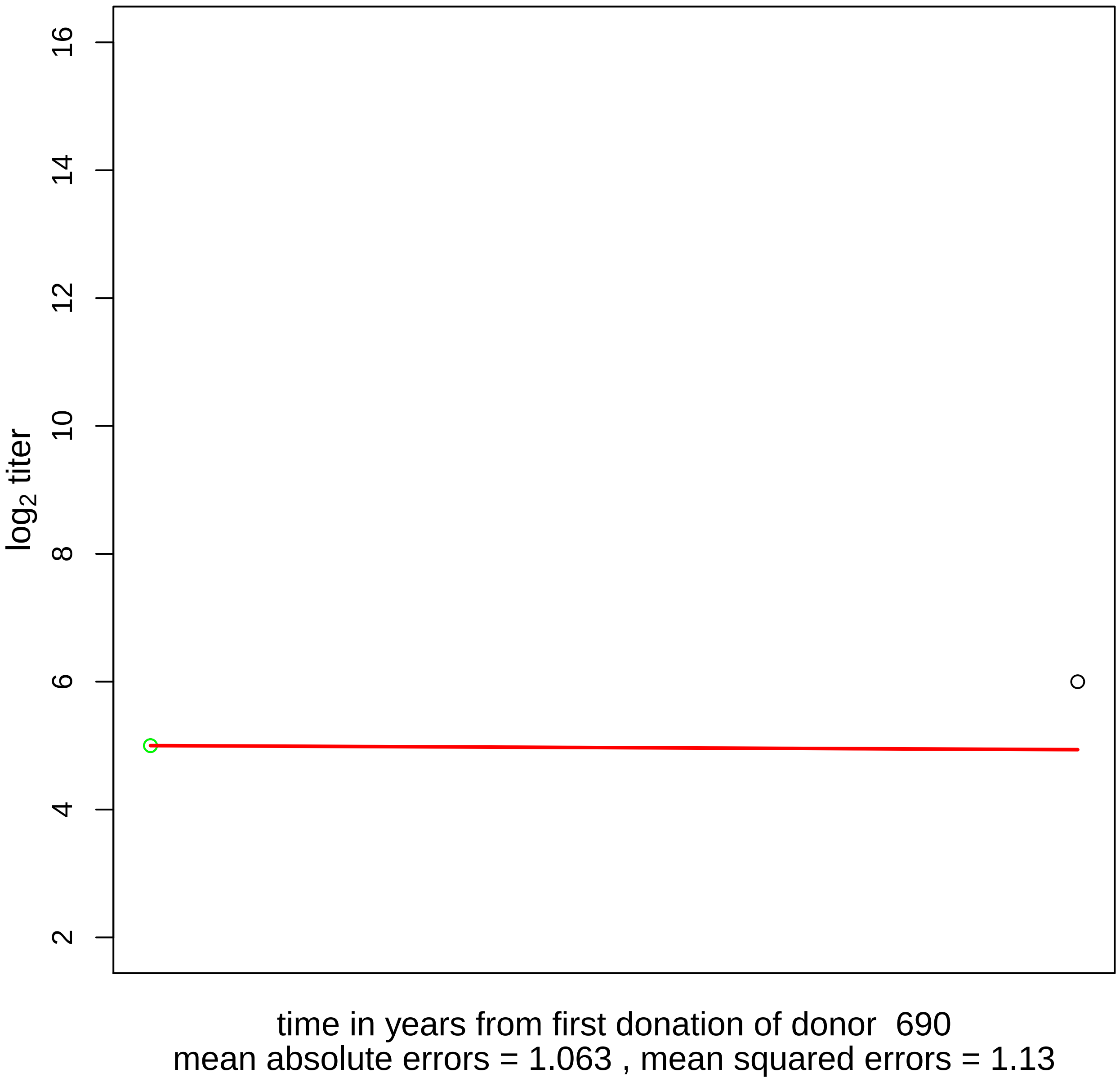

log2 titer
2
4
6
8
10
12
14
16
time in years from first donation of donor 690
mean absolute errors = 1.063 , mean squared errors = 1.13

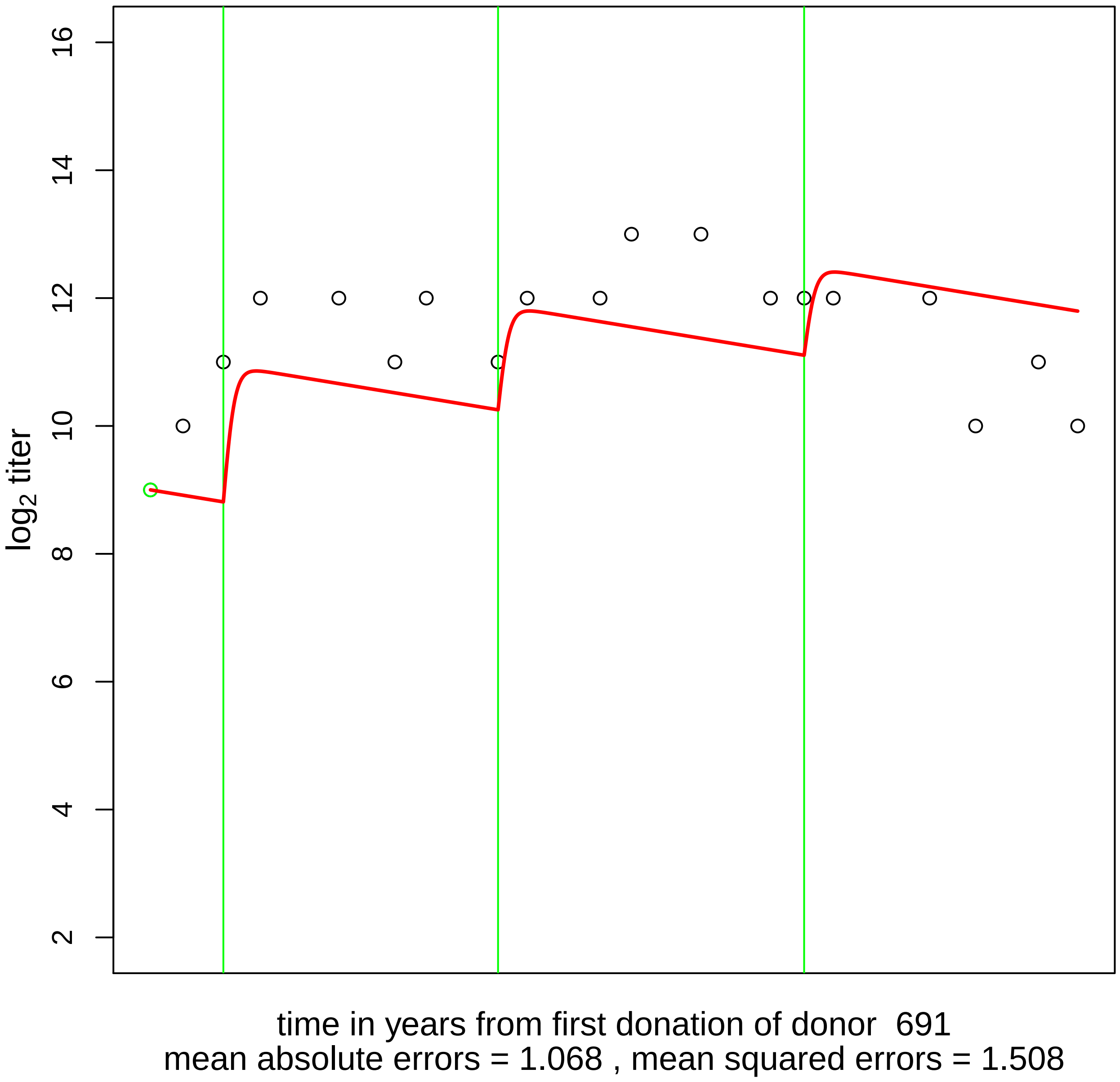

$\log_2$ titer
2
4
6
8
10
12
14
16
time in years from first donation of donor 691
mean absolute errors = 1.068 , mean squared errors = 1.508

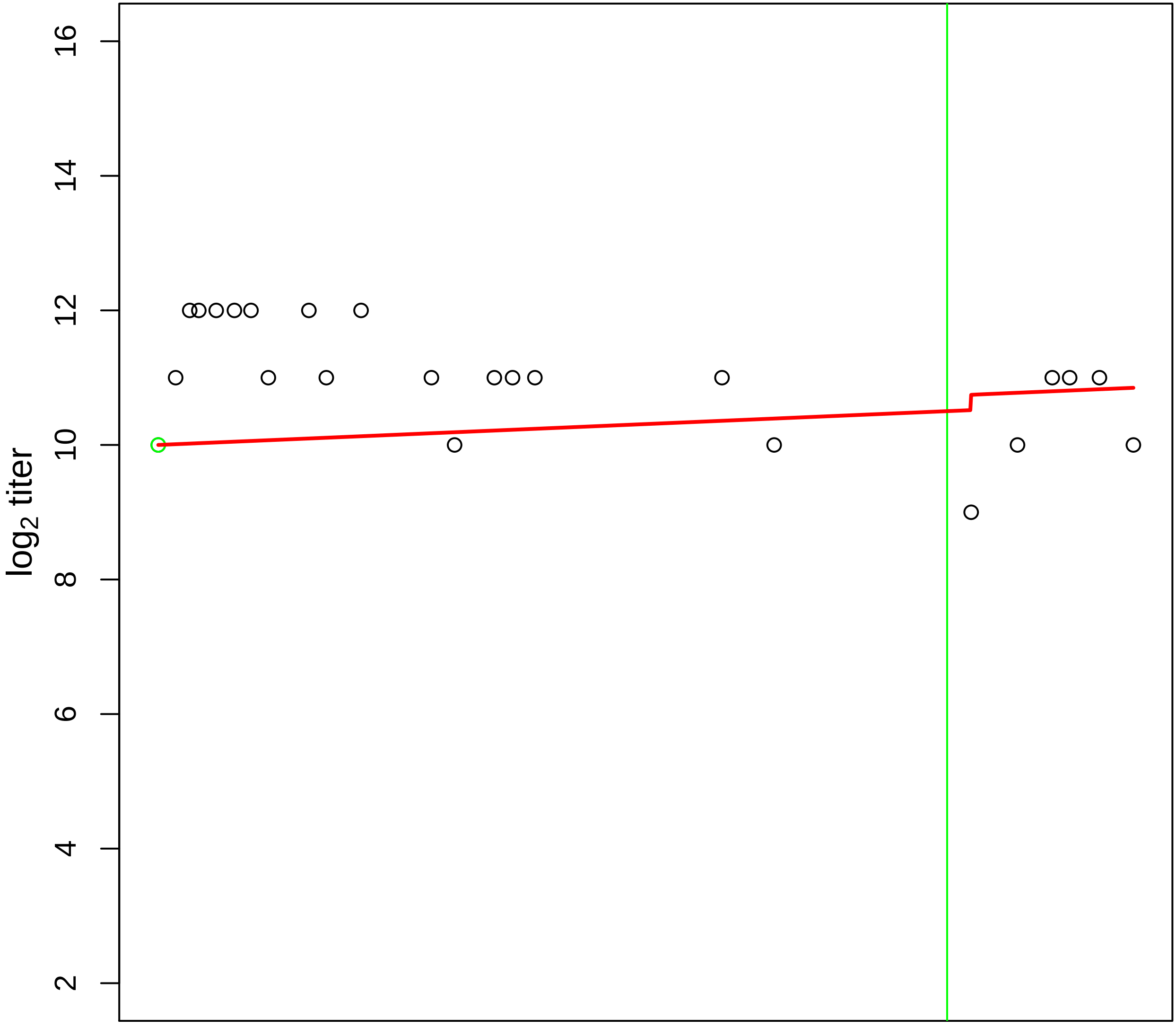

time in years from first donation of donor 692
mean absolute errors = 1.074 , mean squared errors = 1.59

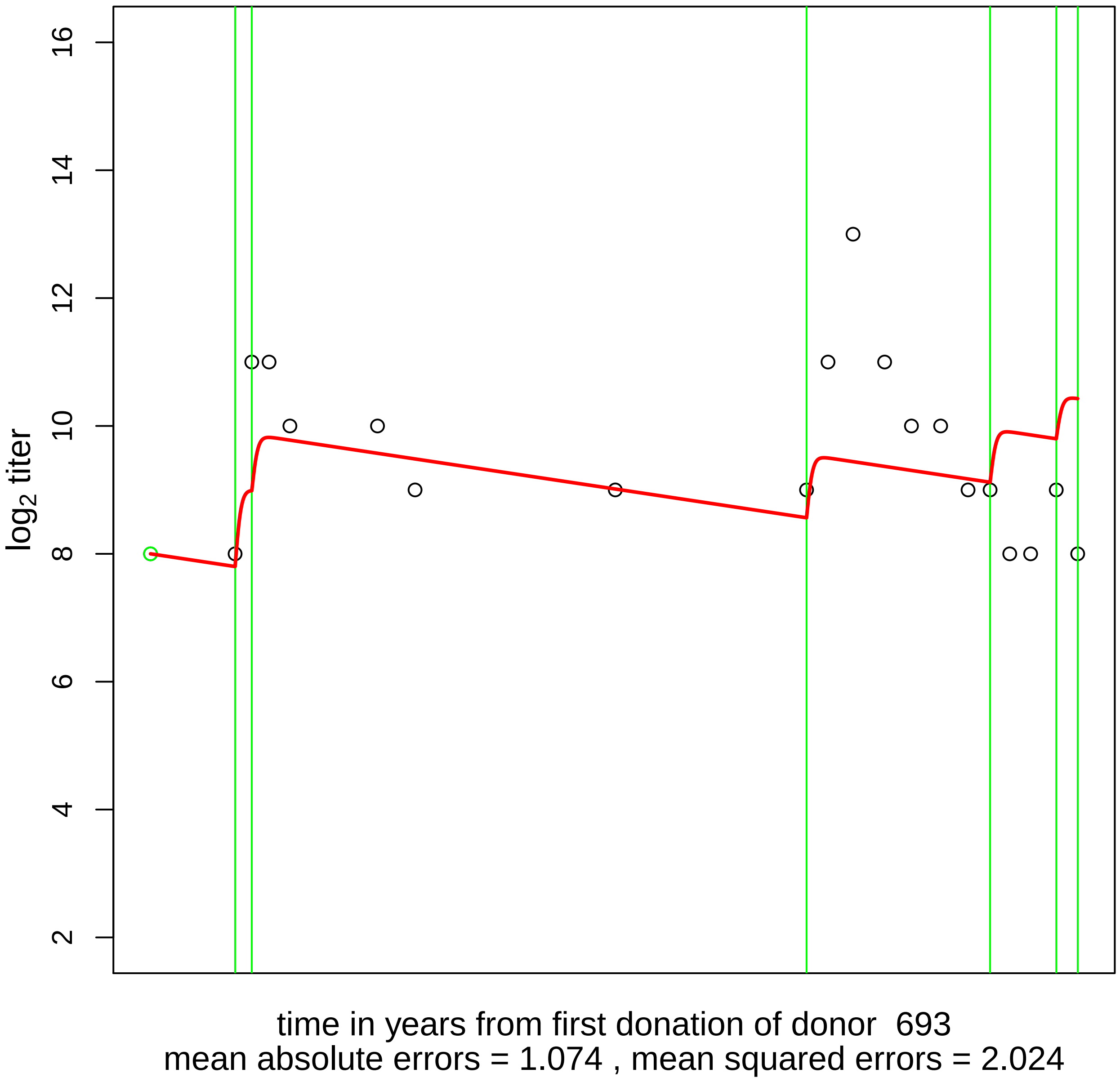

16
14
12
10
8
6
4
2
$\log_2$ titer
time in years from first donation of donor 693
mean absolute errors = 1.074 , mean squared errors = 2.024

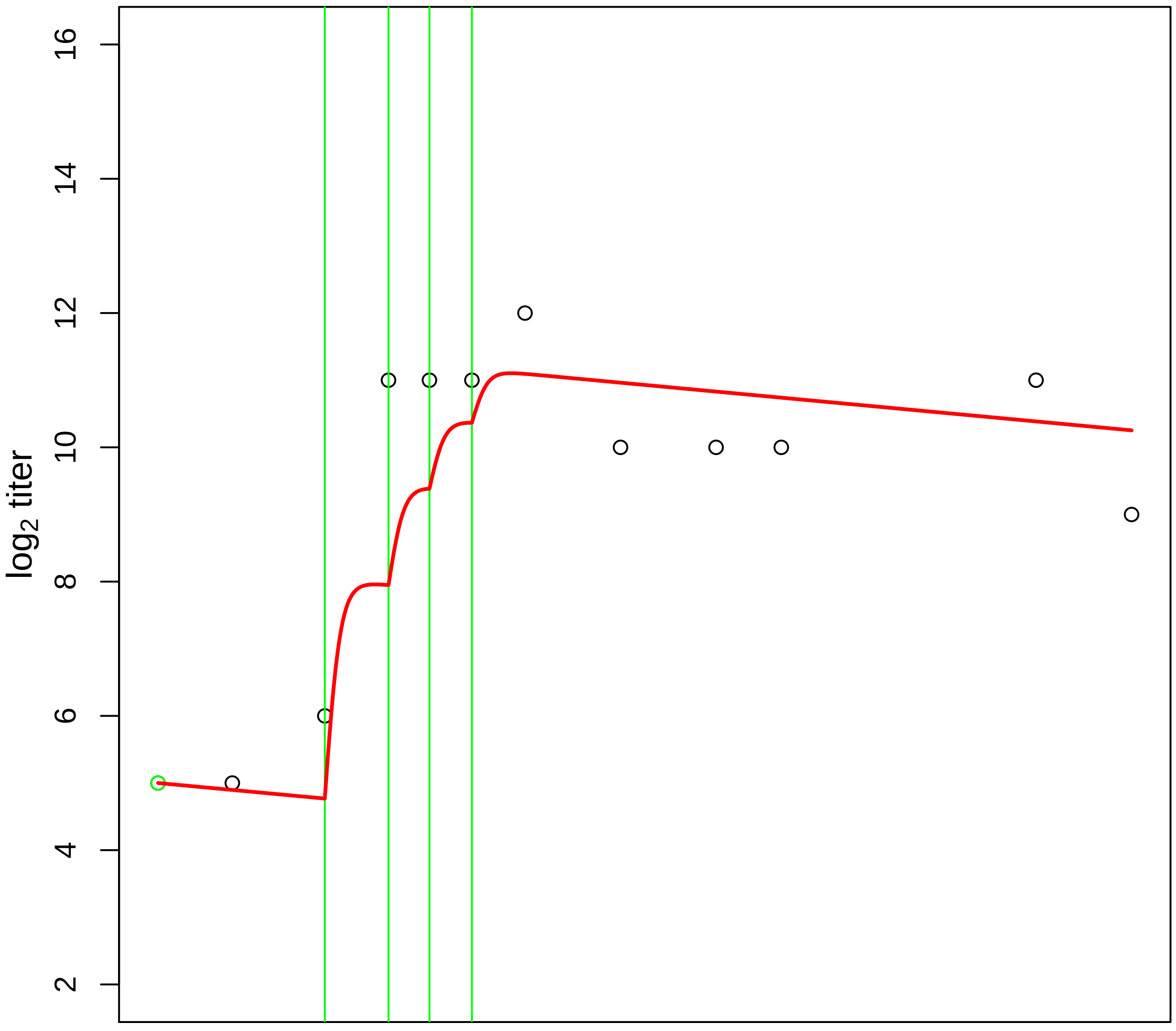

time in years from first donation of donor 694
mean absolute errors = 1.086 , mean squared errors = 1.708

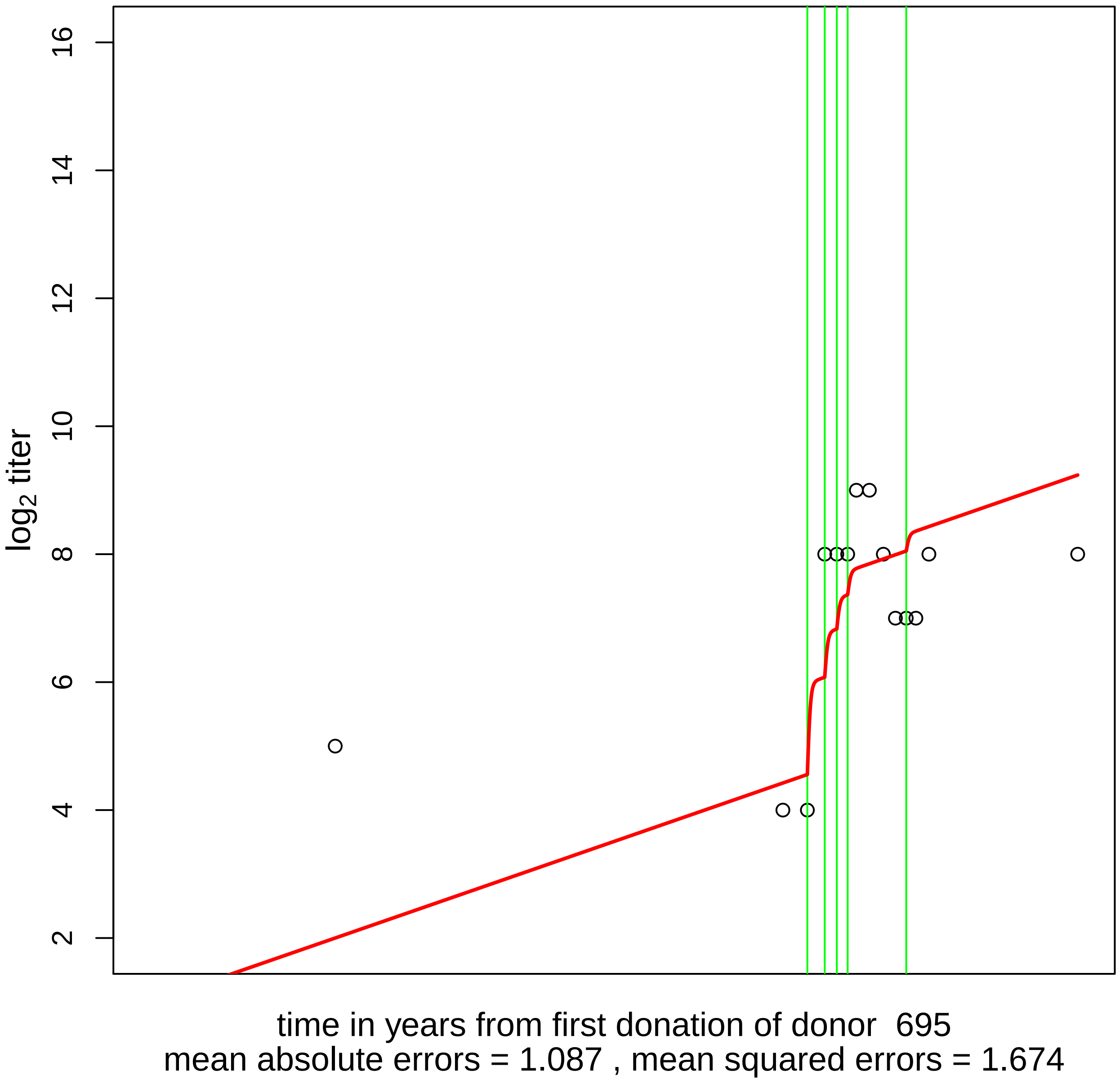

16
14
12
10
8
6
4
2
log2 titer
time in years from first donation of donor 695
mean absolute errors = 1.087 , mean squared errors = 1.674

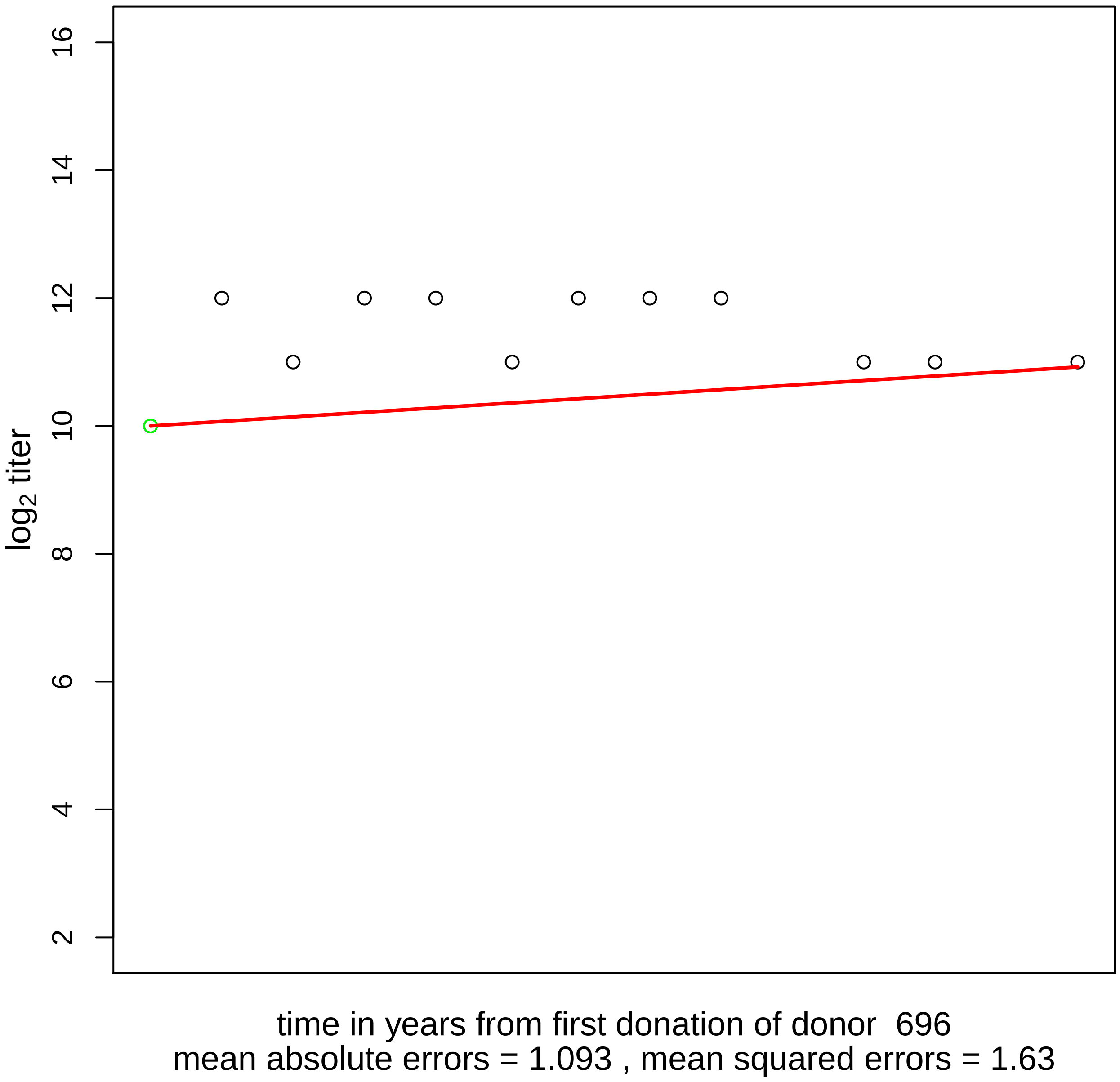

16
14
12
10
8
6
4
2
log2 titer
time in years from first donation of donor 696
mean absolute errors = 1.093 , mean squared errors = 1.63

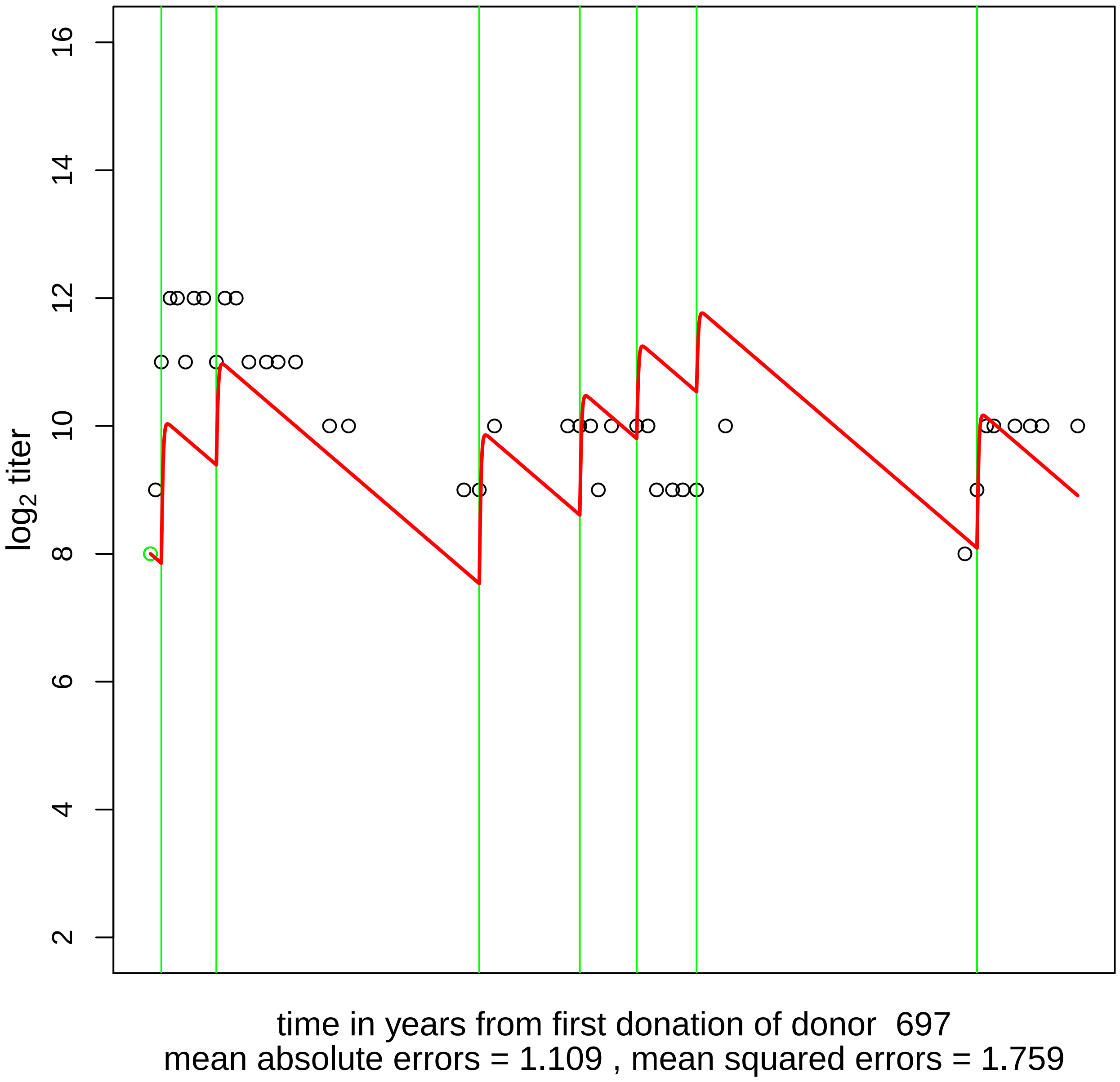

log2 titer
2
4
6
8
10
12
14
16
time in years from first donation of donor 697
mean absolute errors = 1.109 , mean squared errors = 1.759

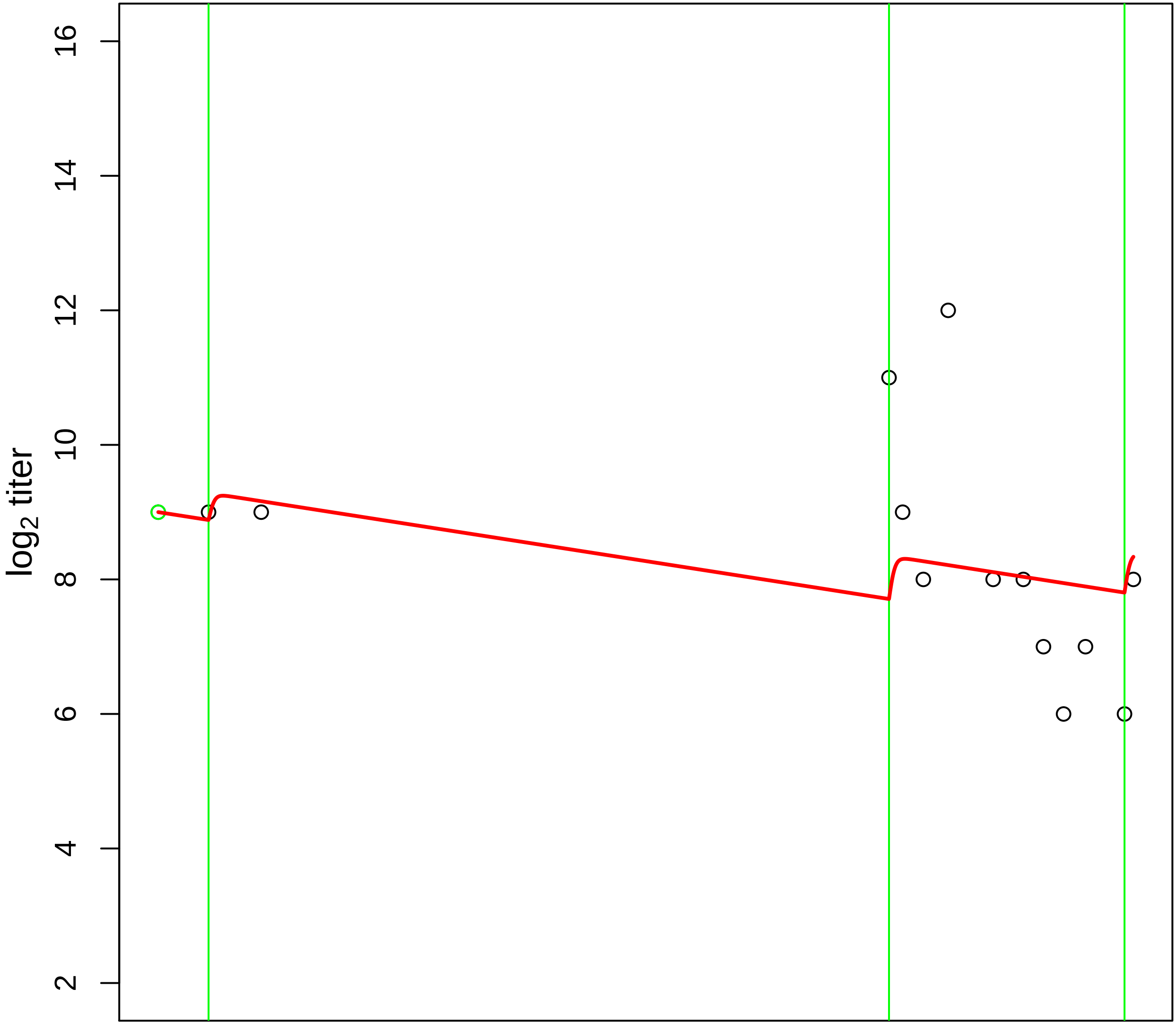

time in years from first donation of donor 698
mean absolute errors = 1.111 , mean squared errors = 2.671

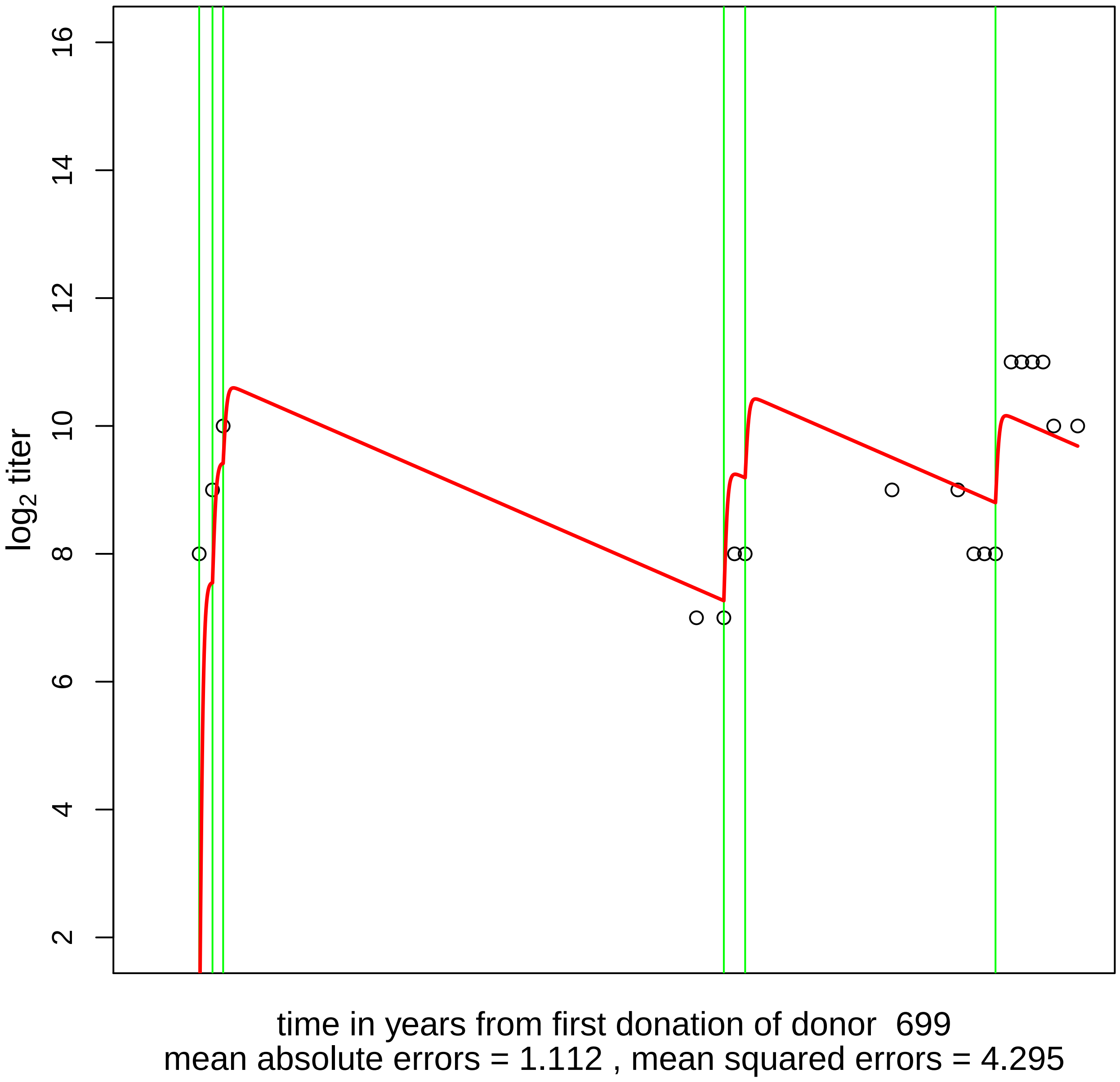
16
14
12
10
8
6
4
2
$\log_2$ titer
time in years from first donation of donor 699
mean absolute errors = 1.112 , mean squared errors = 4.295

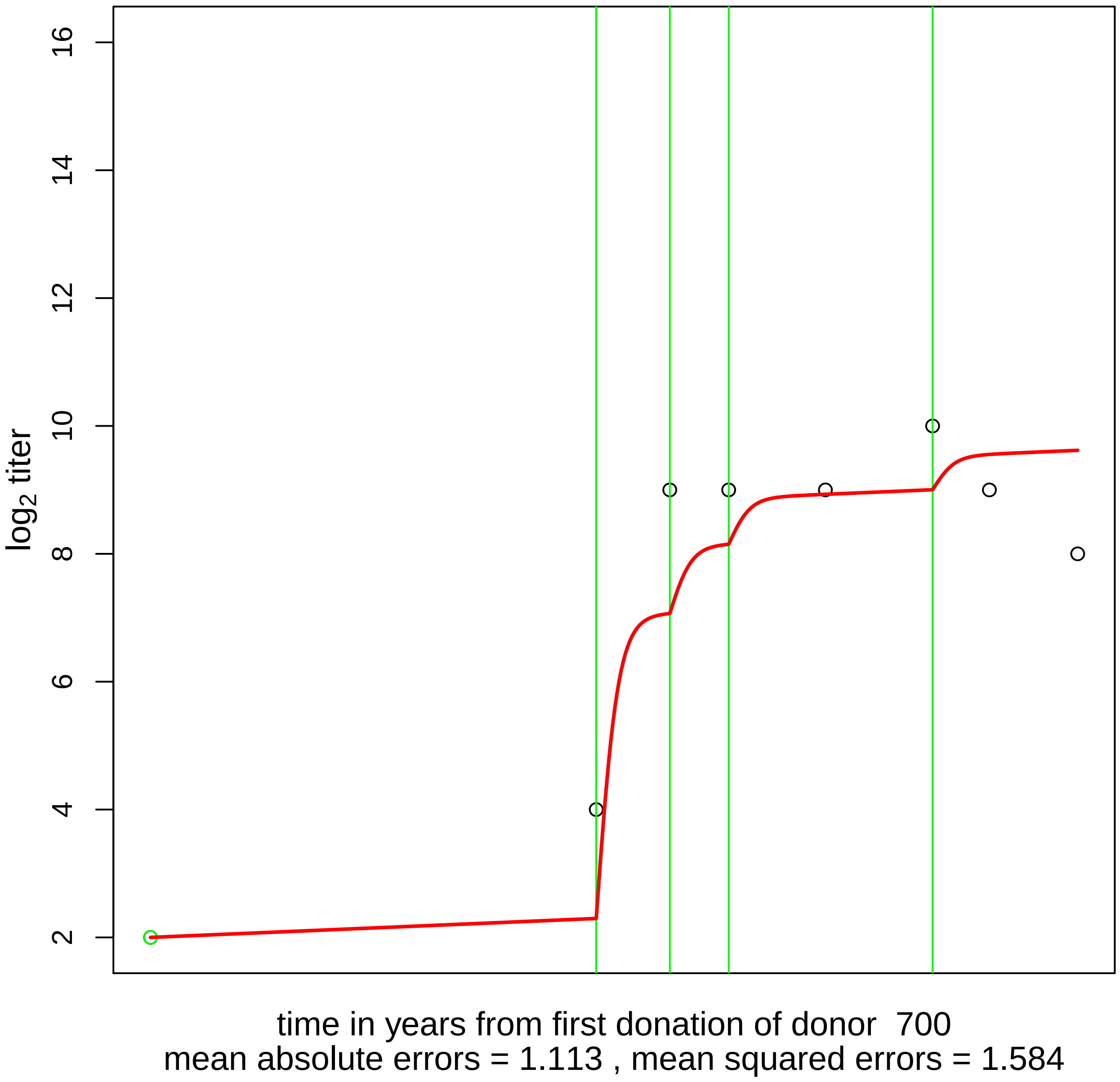

16
14
12
10
8
6
4
2
$\log_2$ titer
time in years from first donation of donor 700
mean absolute errors = 1.113 , mean squared errors = 1.584

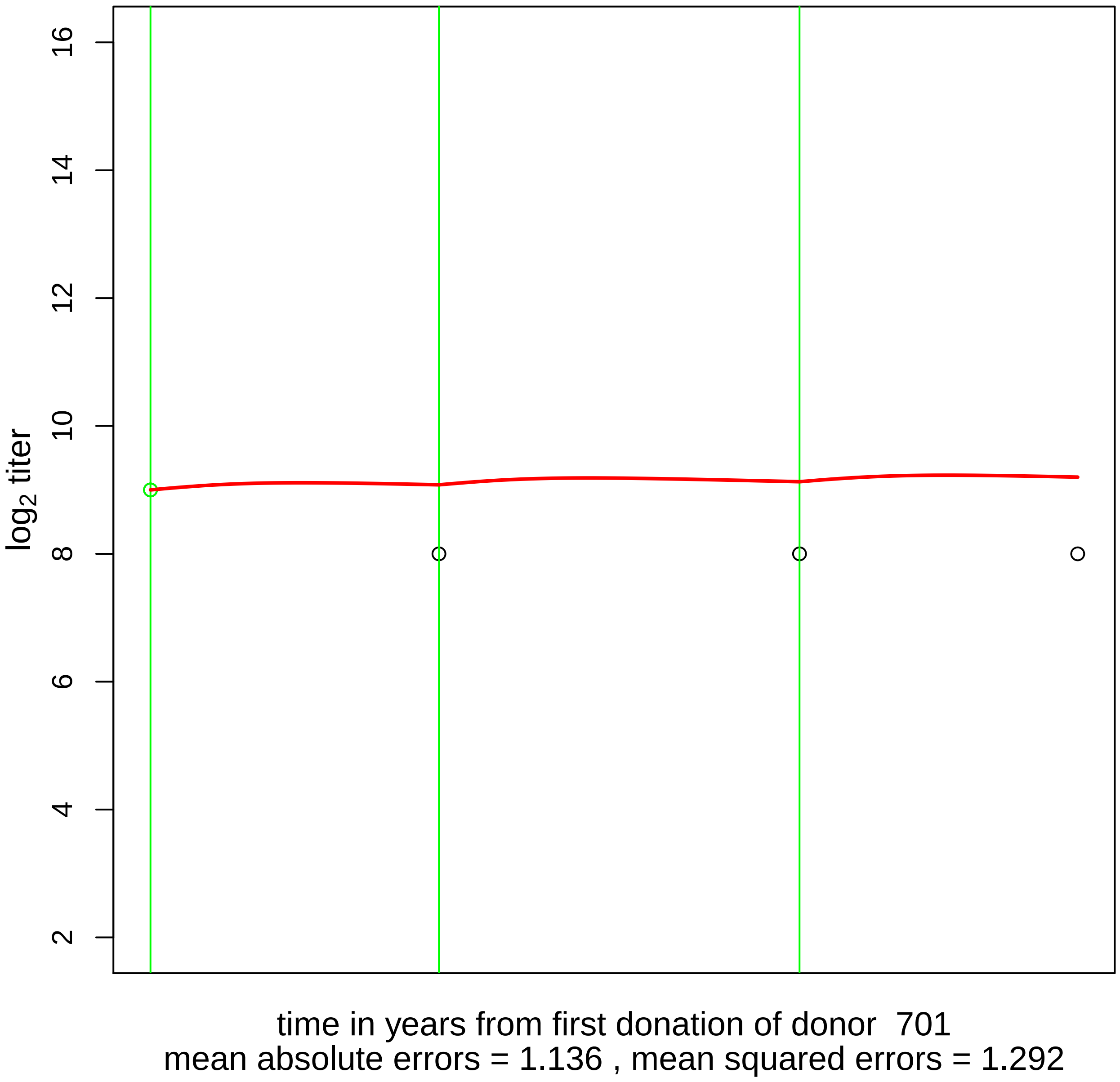

2
4
6
8
10
12
14
16
log2 titer
time in years from first donation of donor 701
mean absolute errors = 1.136 , mean squared errors = 1.292

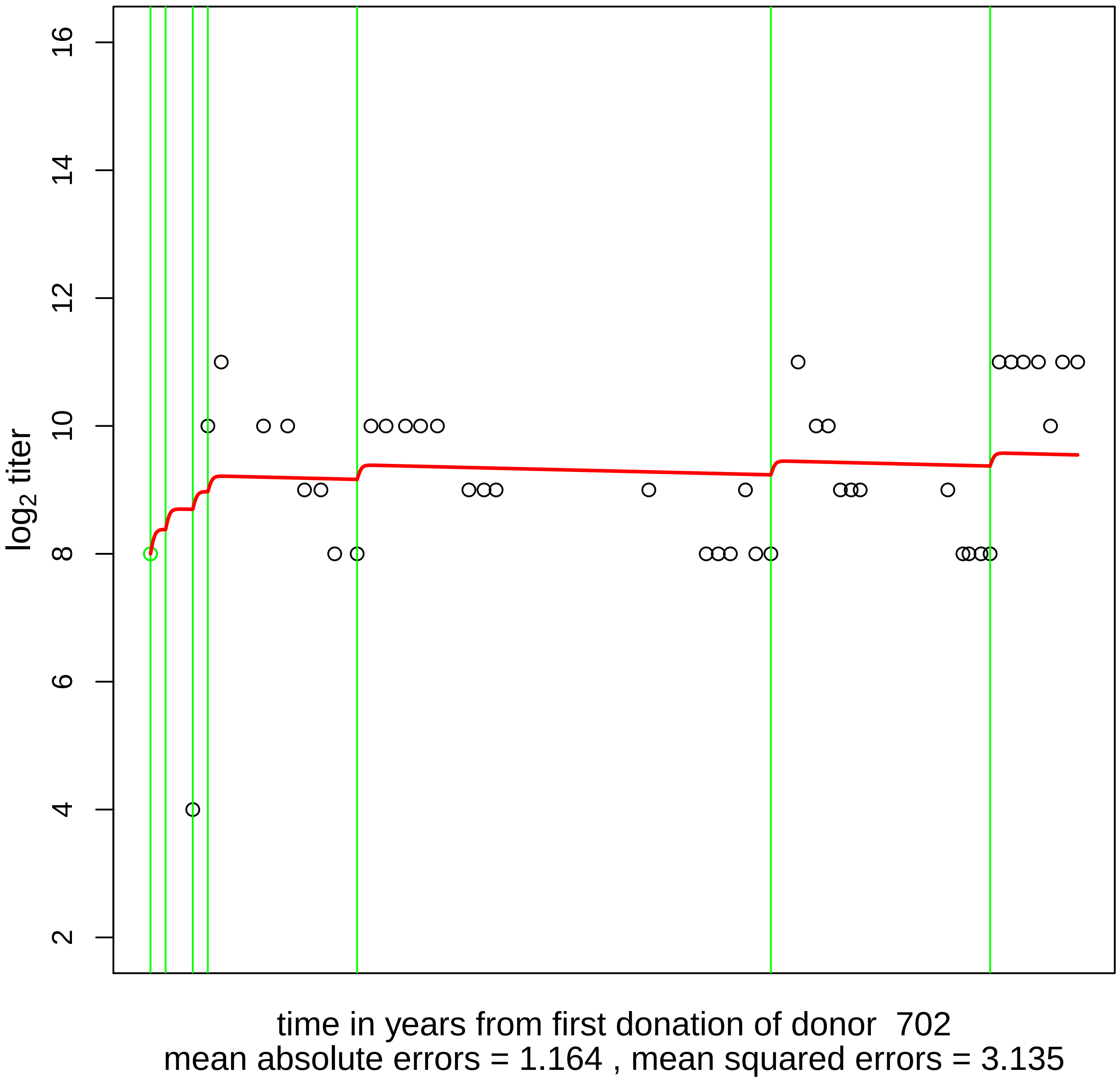

16
14
12
10
8
6
4
2
log2 titer
time in years from first donation of donor 702
mean absolute errors = 1.164 , mean squared errors = 3.135

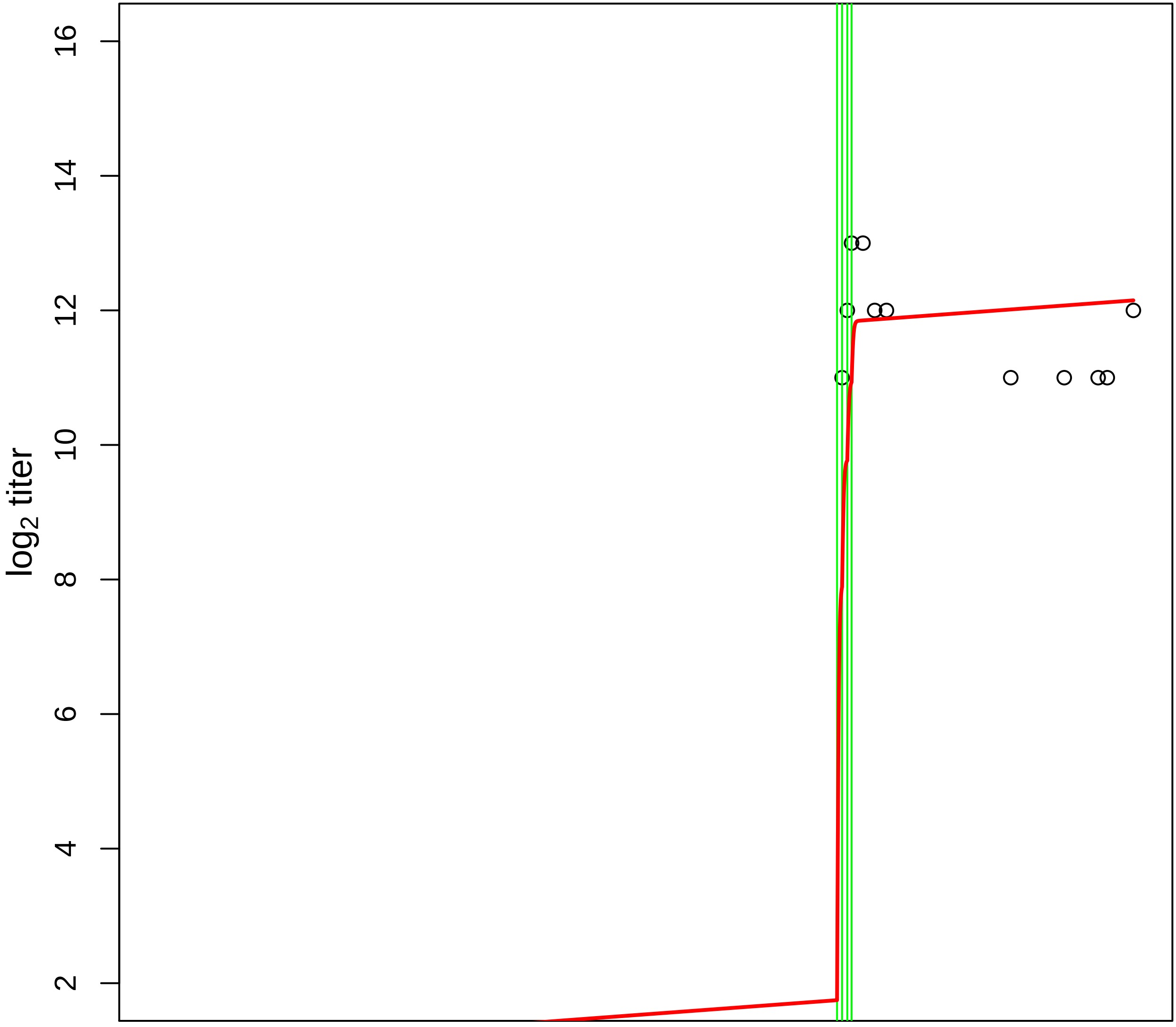

time in years from first donation of donor 703
mean absolute errors = 1.168 , mean squared errors = 2.065

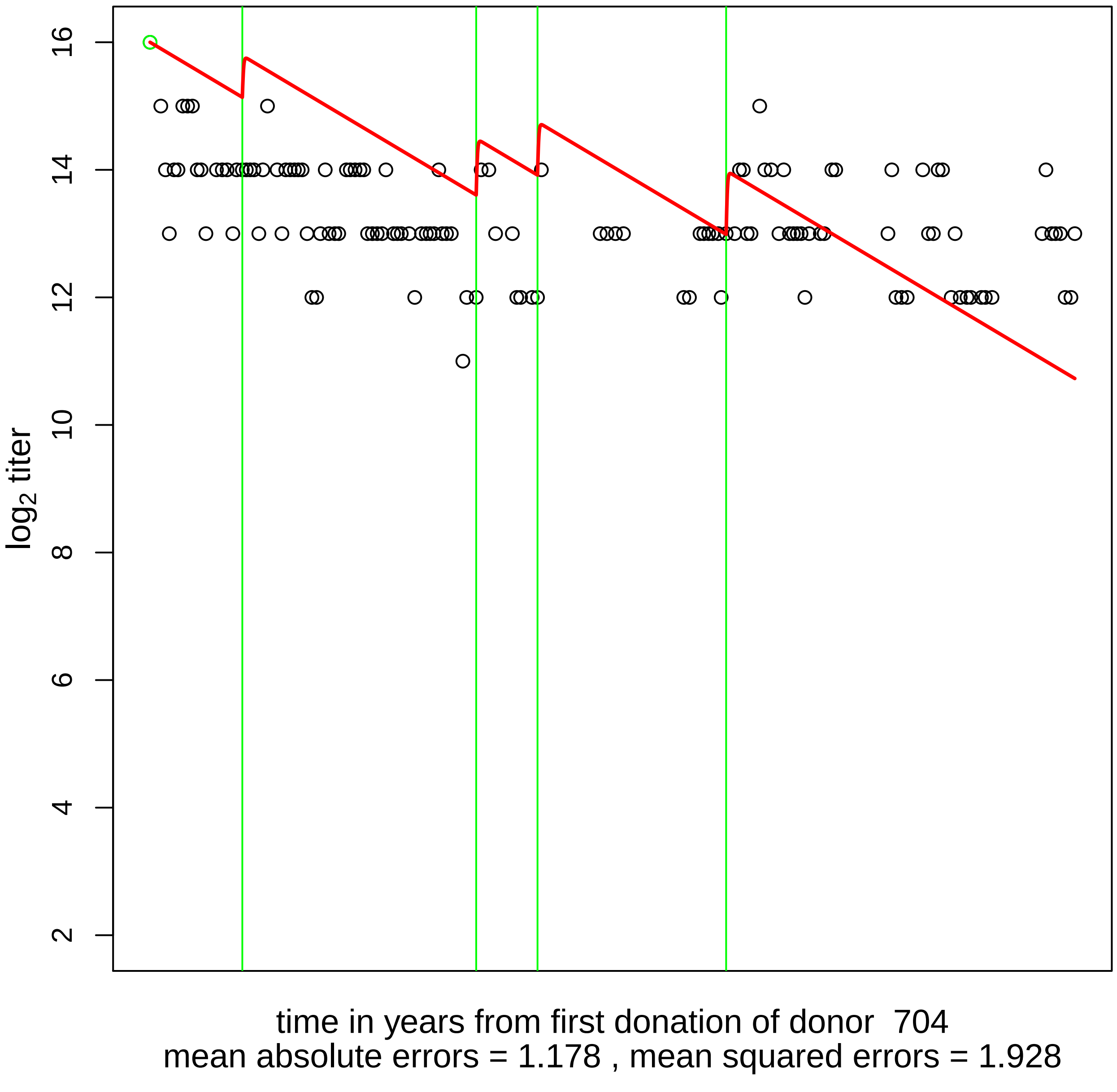

$\log_2$ titer
16
14
12
10
8
6
4
2
time in years from first donation of donor 704
mean absolute errors = 1.178 , mean squared errors = 1.928

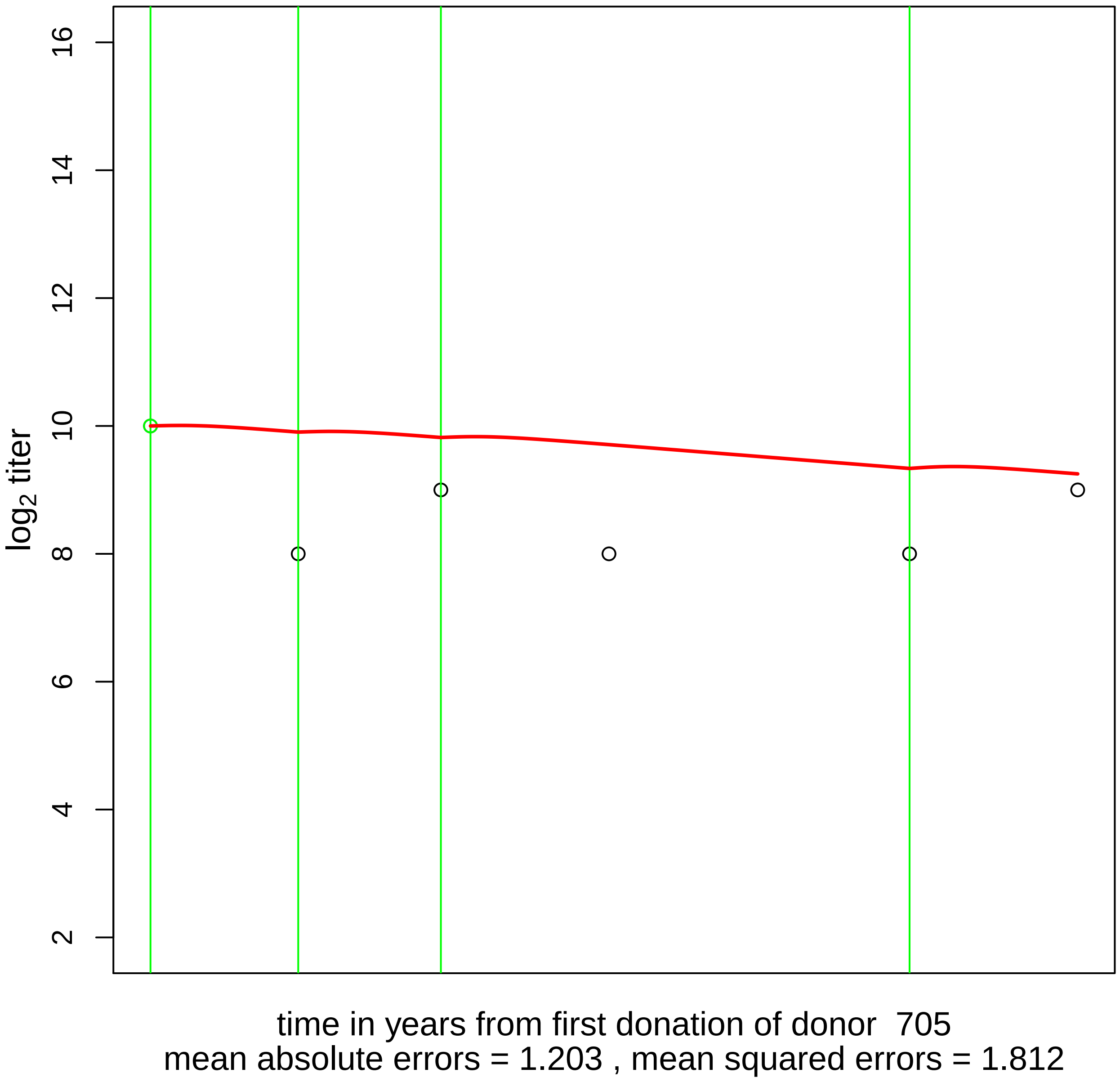

16
14
12
10
8
6
4
2
log2 titer
time in years from first donation of donor 705
mean absolute errors = 1.203 , mean squared errors = 1.812

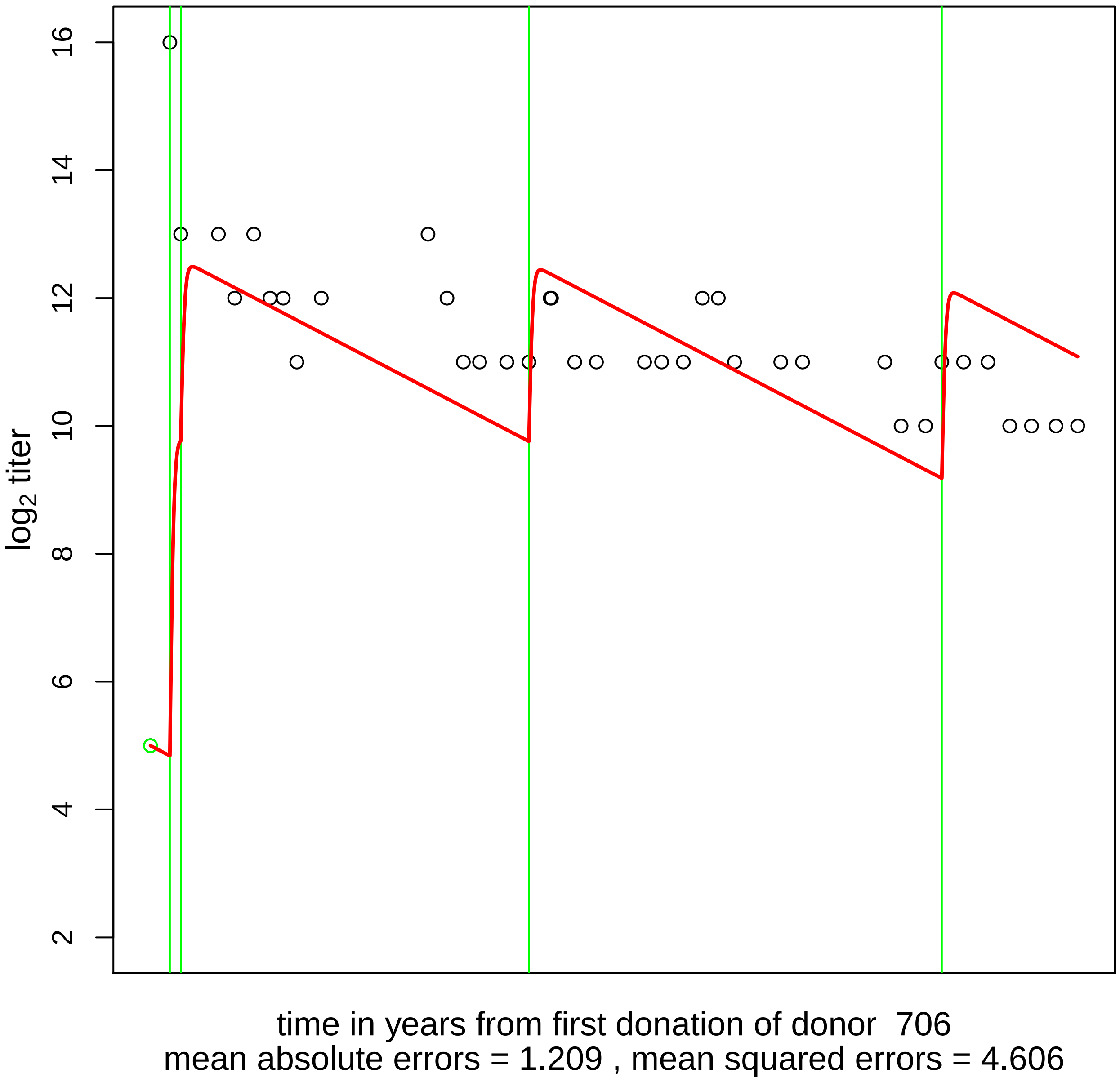

16
14
12
10
8
6
4
2
log2 titer
time in years from first donation of donor 706
mean absolute errors = 1.209 , mean squared errors = 4.606

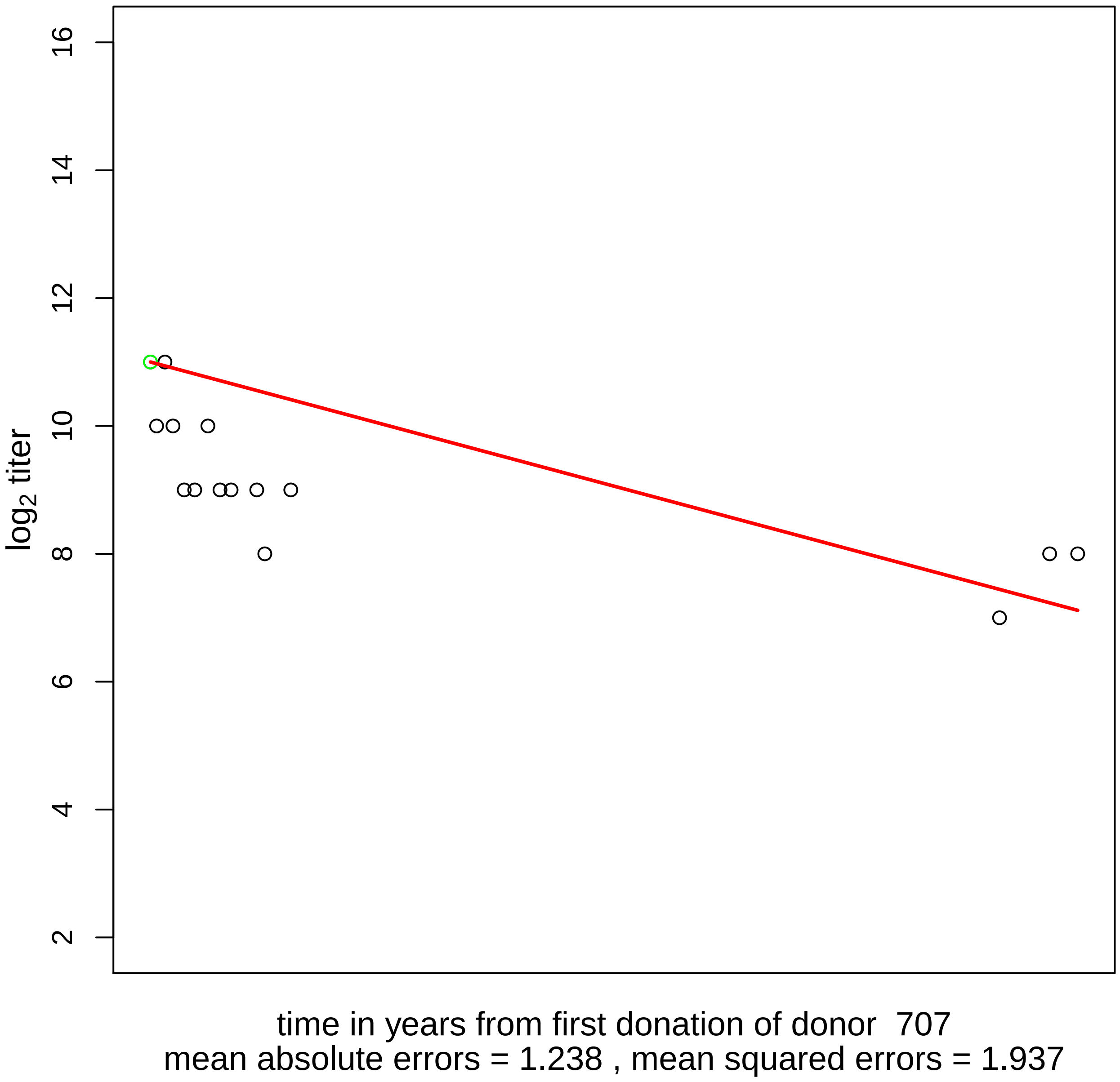

16
14
12
10
8
6
4
2
log2 titer
time in years from first donation of donor 707
mean absolute errors = 1.238 , mean squared errors = 1.937

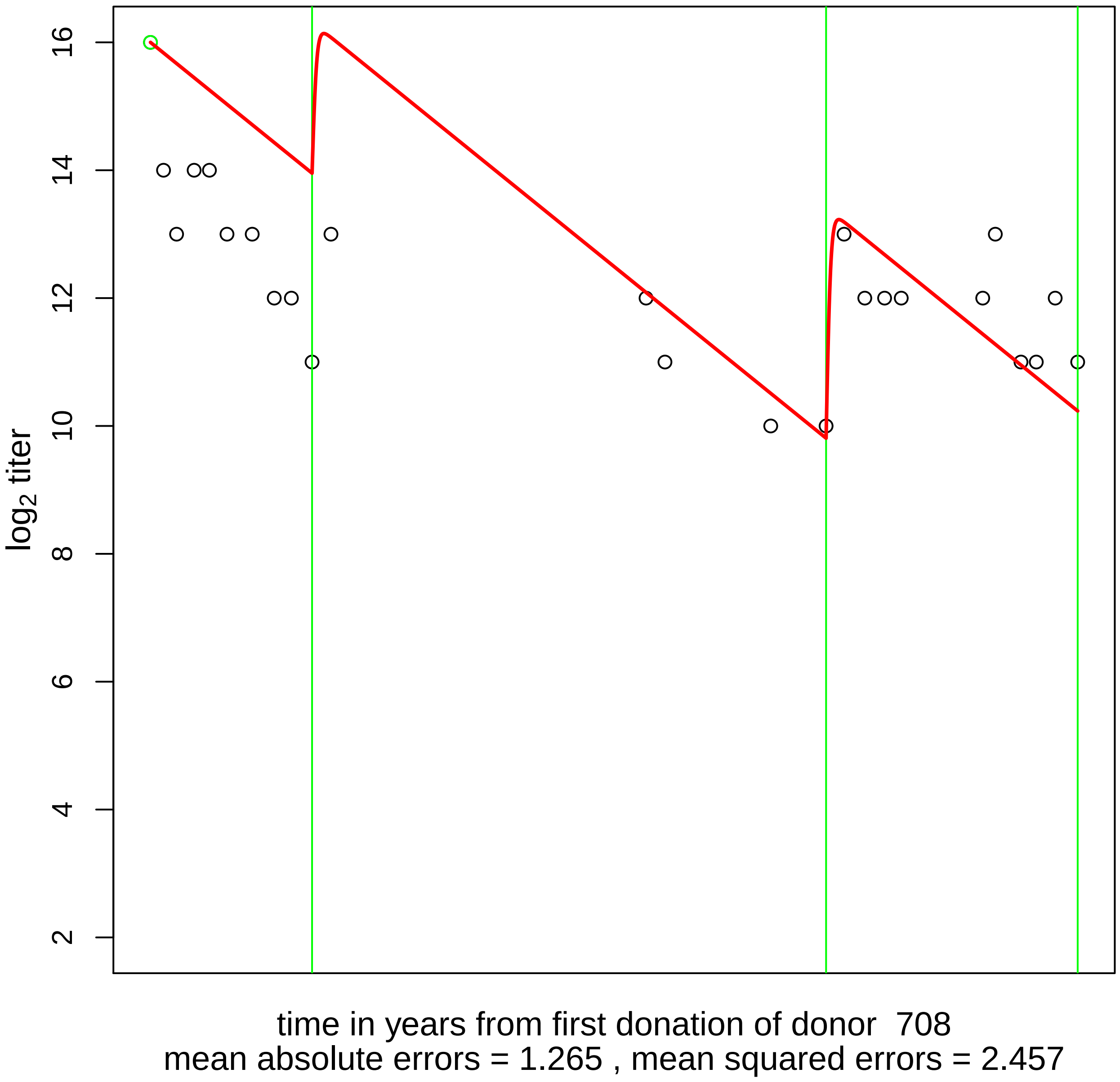

16
14
12
10
8
6
4
2
$\log_2$ titer
time in years from first donation of donor 708
mean absolute errors = 1.265 , mean squared errors = 2.457

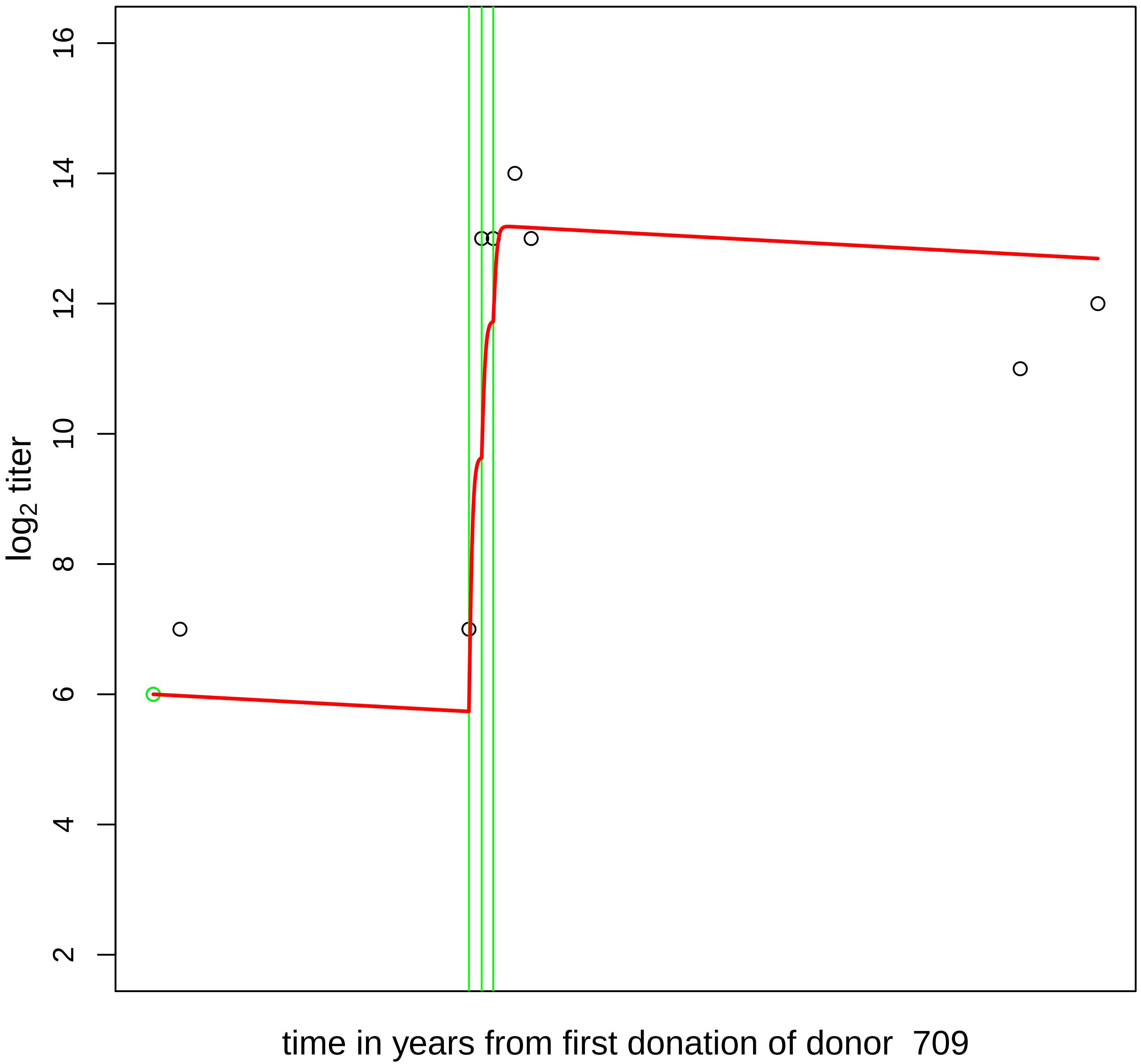

mean absolute errors = 1.296 , mean squared errors = 2.486

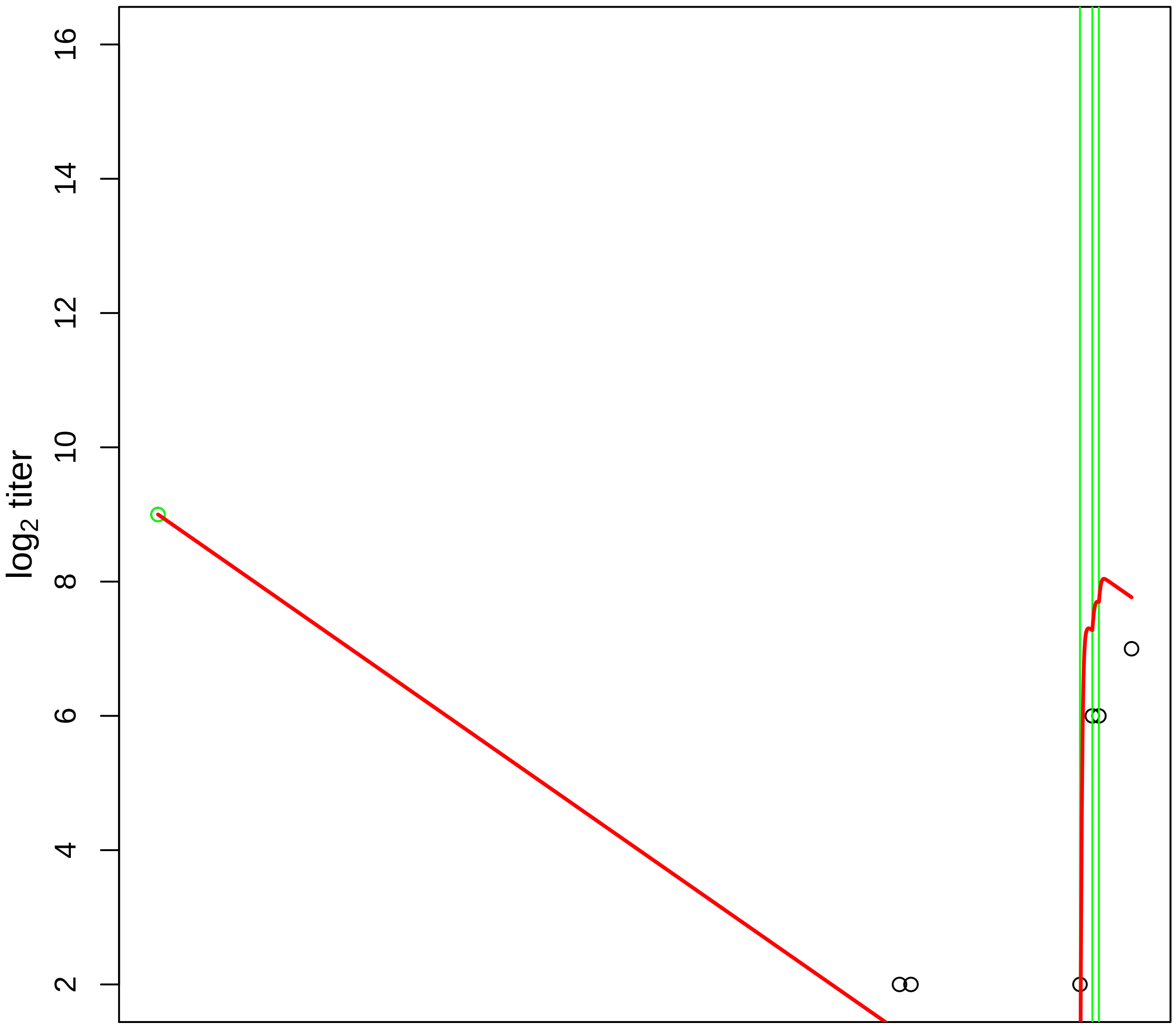

time in years from first donation of donor 710
mean absolute errors = 1.309 , mean squared errors = 2.157

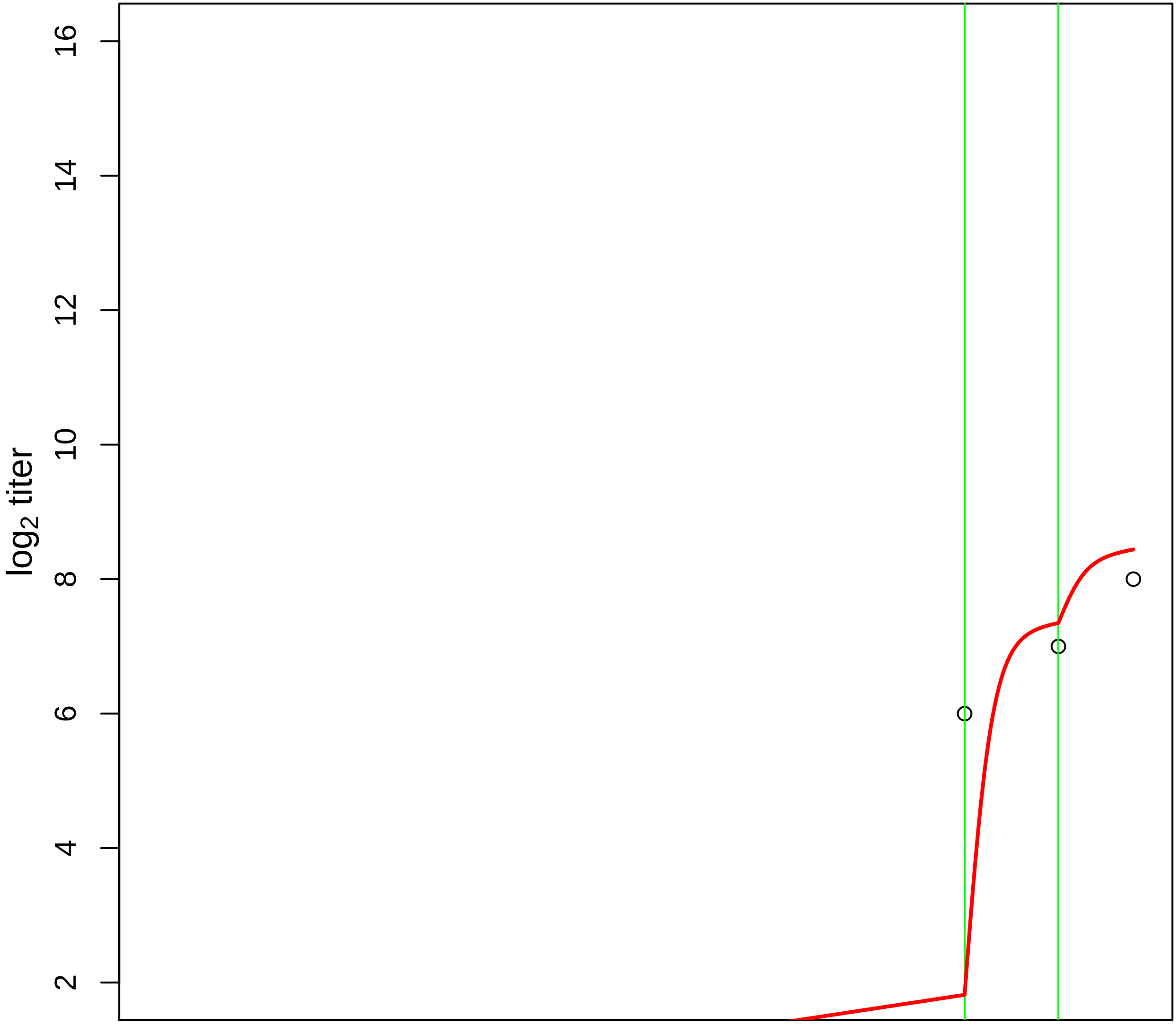

time in years from first donation of donor 711
mean absolute errors = 1.317 , mean squared errors = 4.475

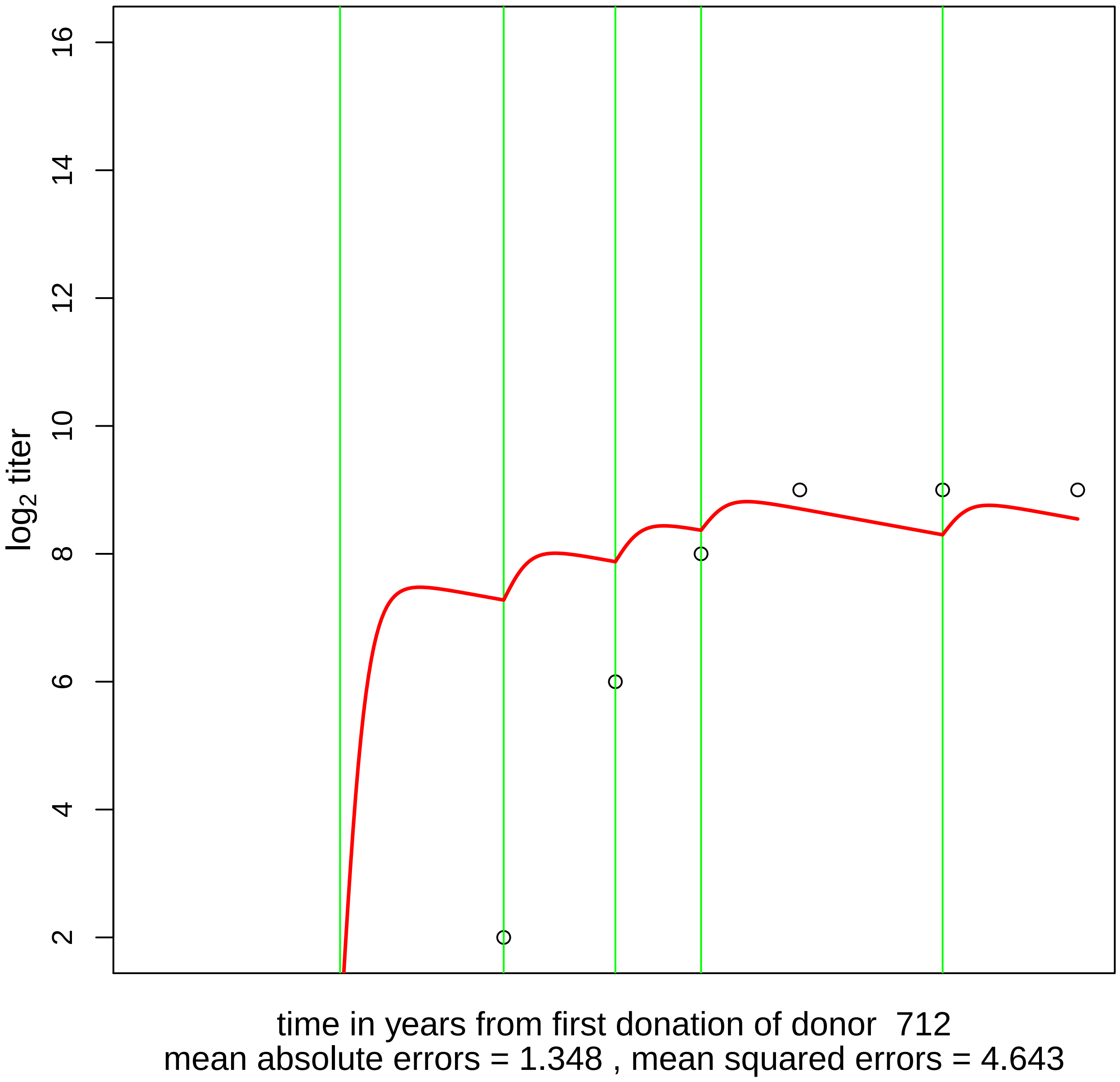
16
14
12
10
8
6
4
2
log2 titer
time in years from first donation of donor 712
mean absolute errors = 1.348 , mean squared errors = 4.643

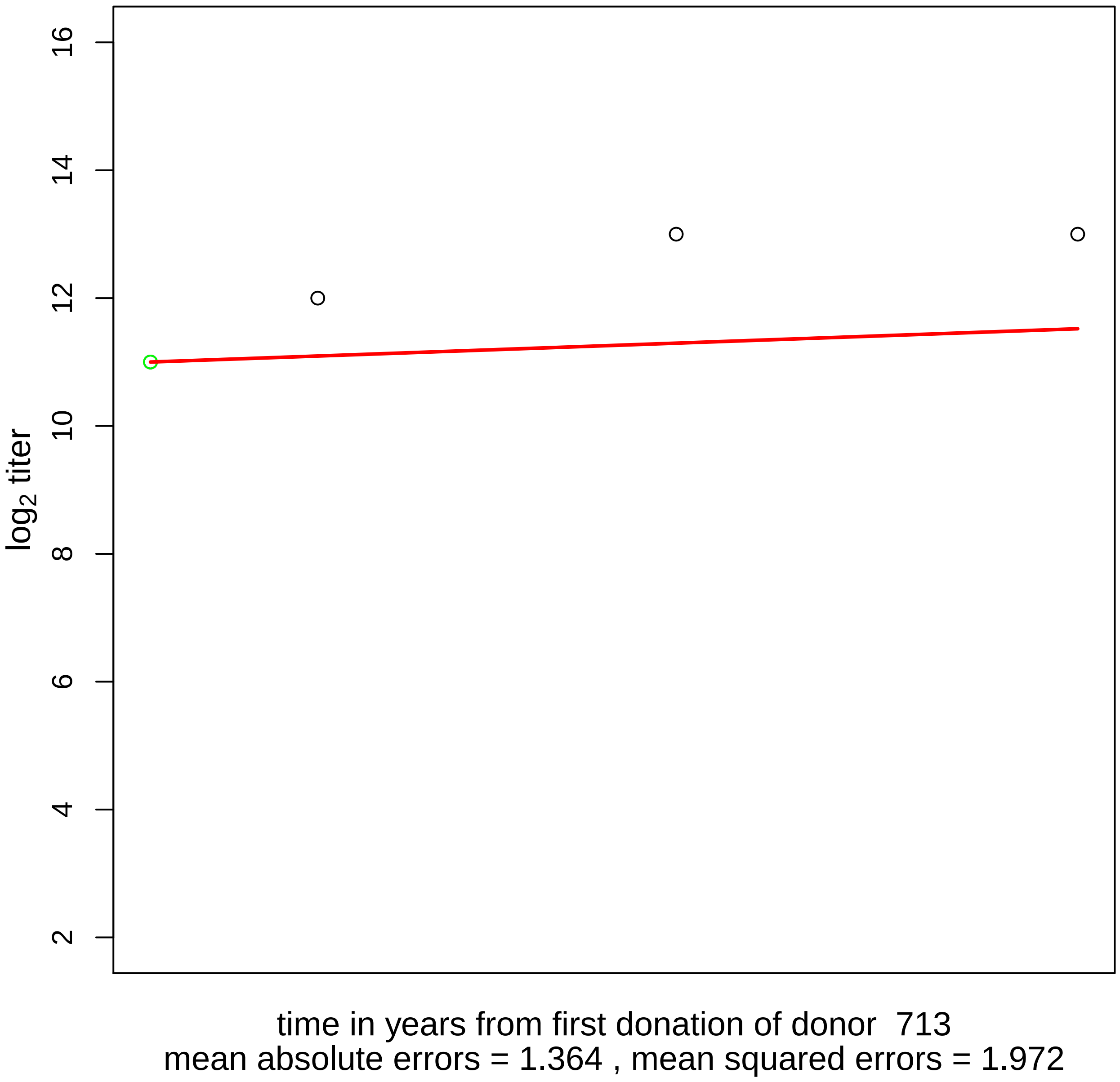

log2 titer
2
4
6
8
10
12
14
16
time in years from first donation of donor 713
mean absolute errors = 1.364 , mean squared errors = 1.972

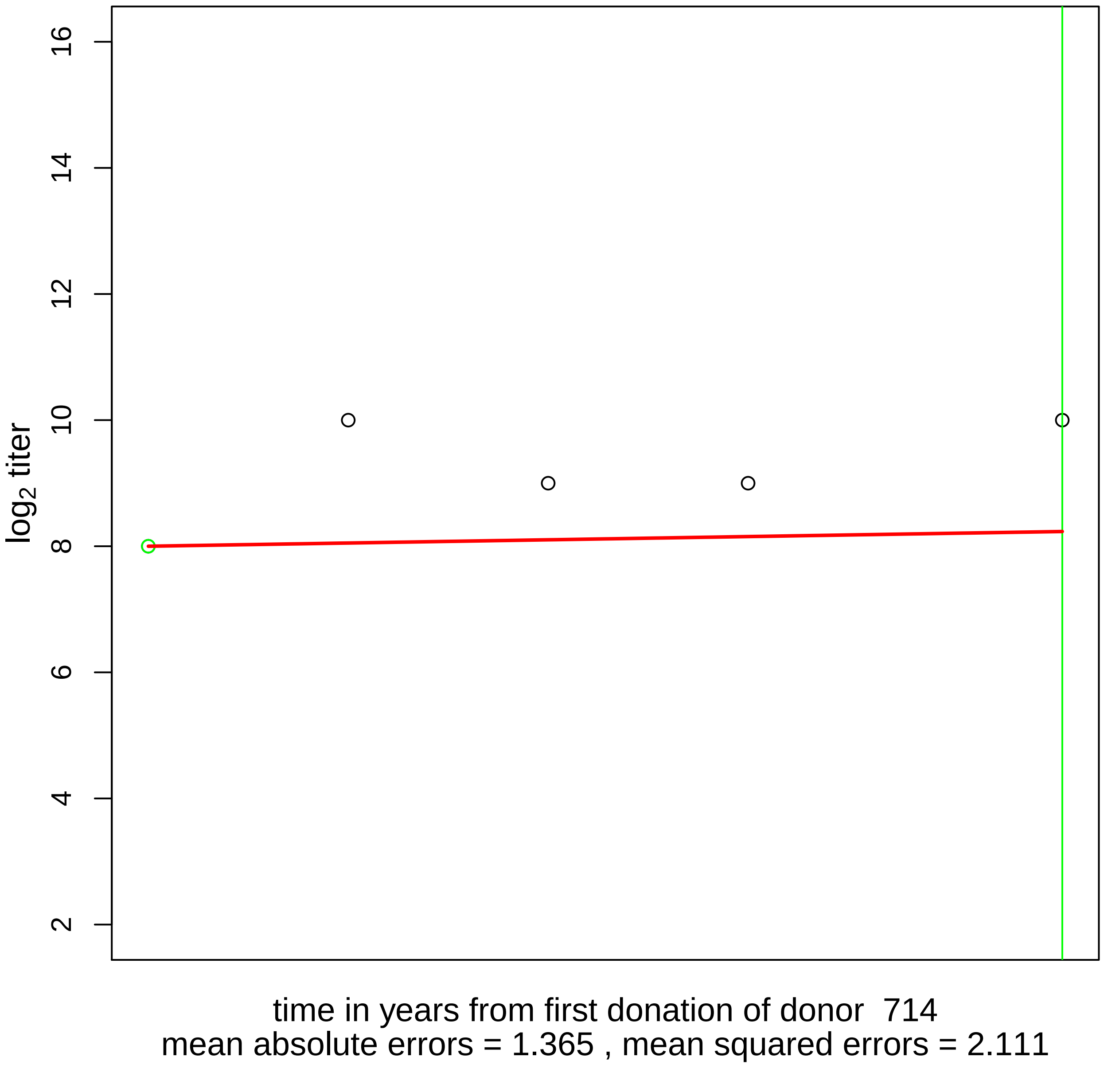

16
14
12
10
8
6
4
2
$\log_2$ titer
time in years from first donation of donor 714
mean absolute errors = 1.365 , mean squared errors = 2.111

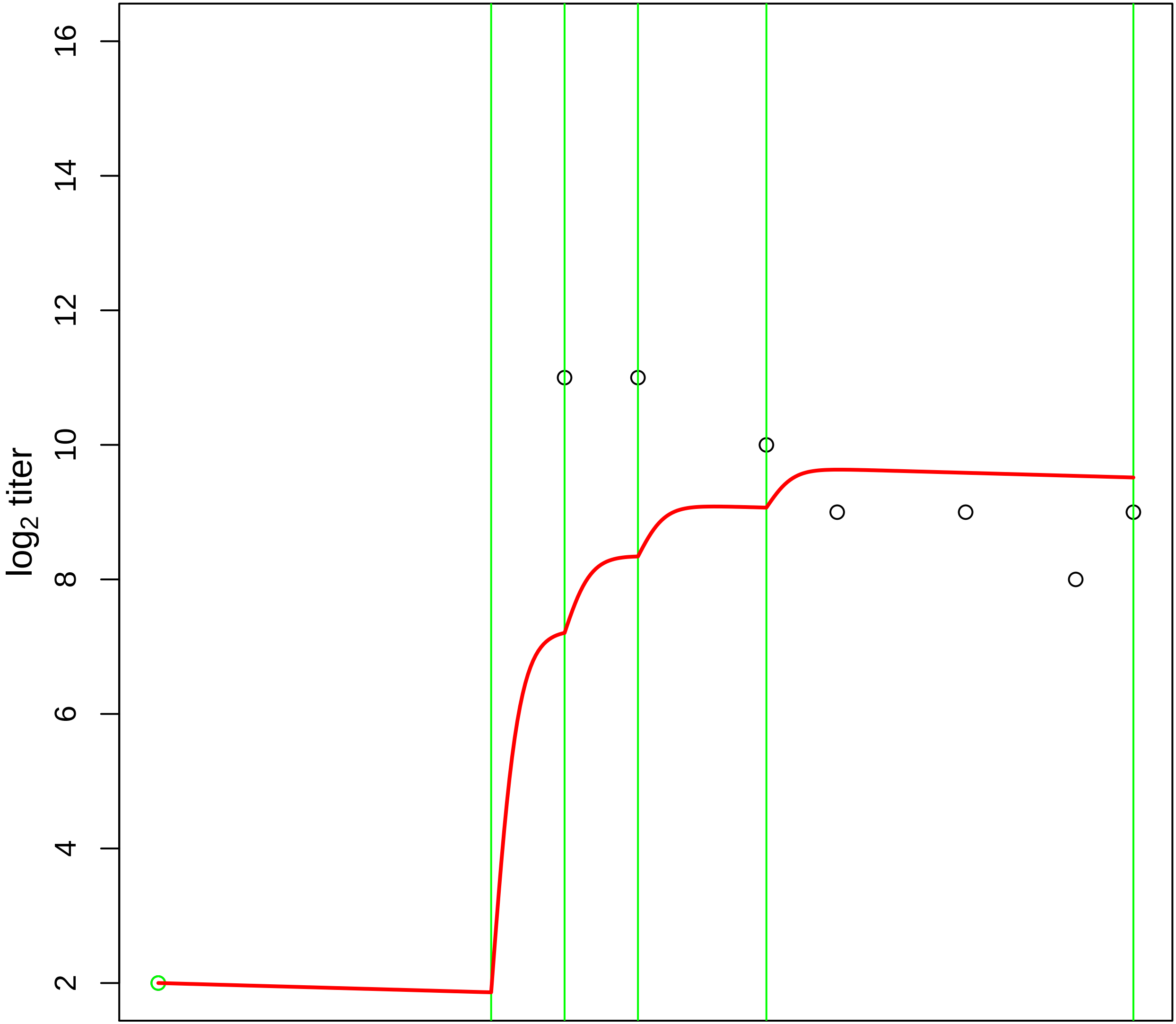

time in years from first donation of donor 715
mean absolute errors = 1.385 , mean squared errors = 3.04

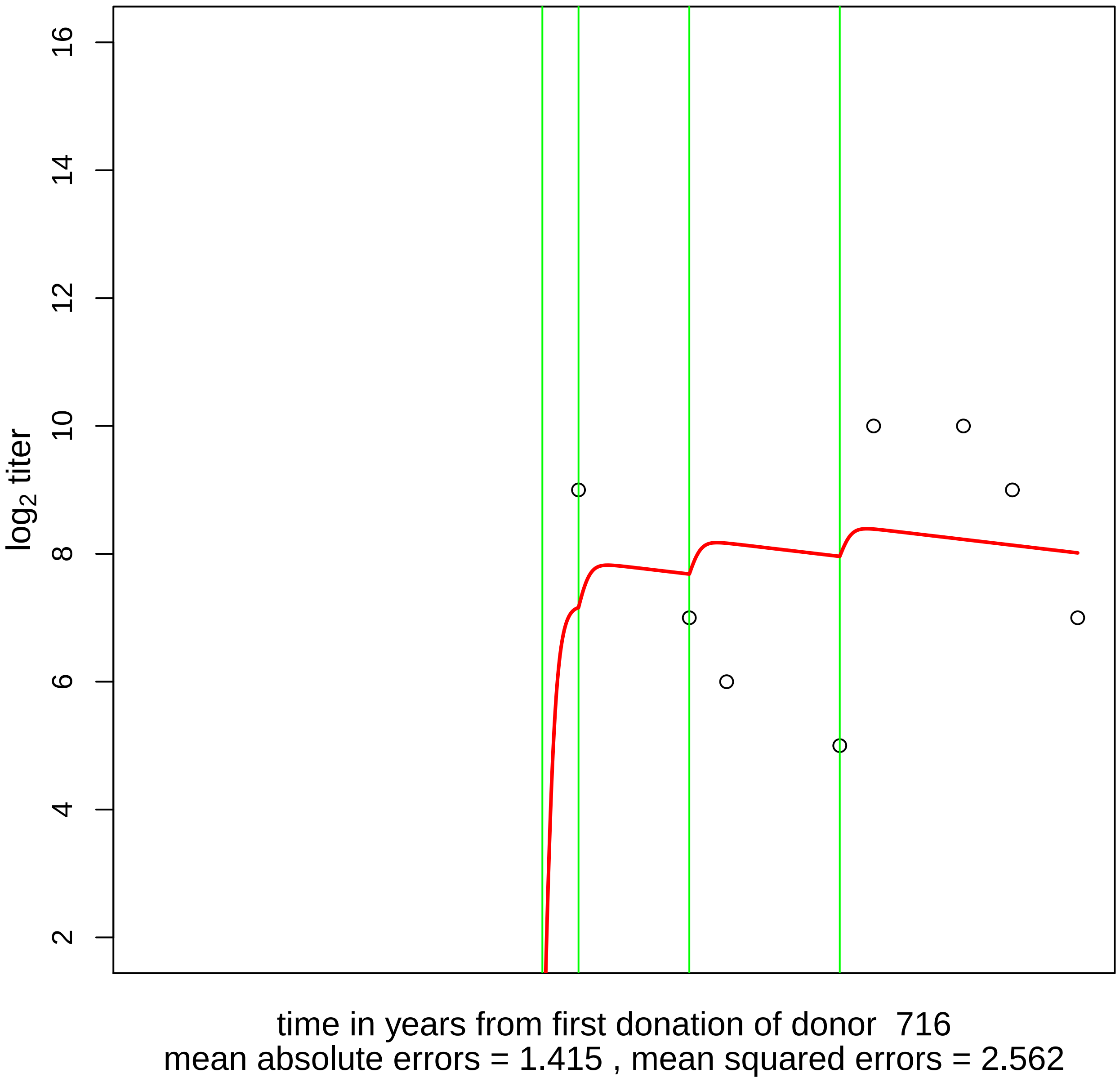

16
14
12
10
8
6
4
2
$\log_2$ titer
time in years from first donation of donor 716
mean absolute errors = 1.415 , mean squared errors = 2.562

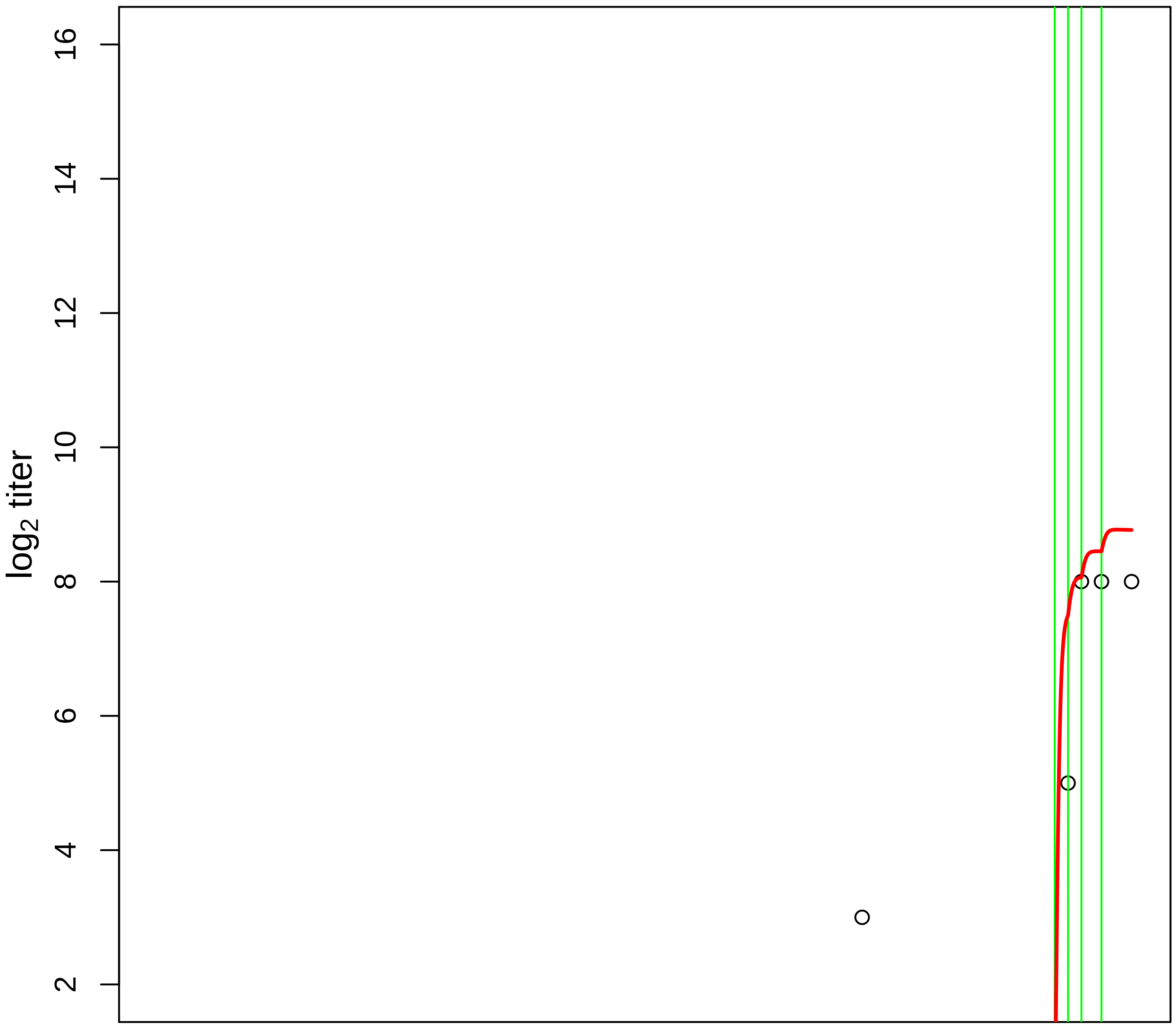

time in years from first donation of donor 717
mean absolute errors = 1.42 , mean squared errors = 3.347

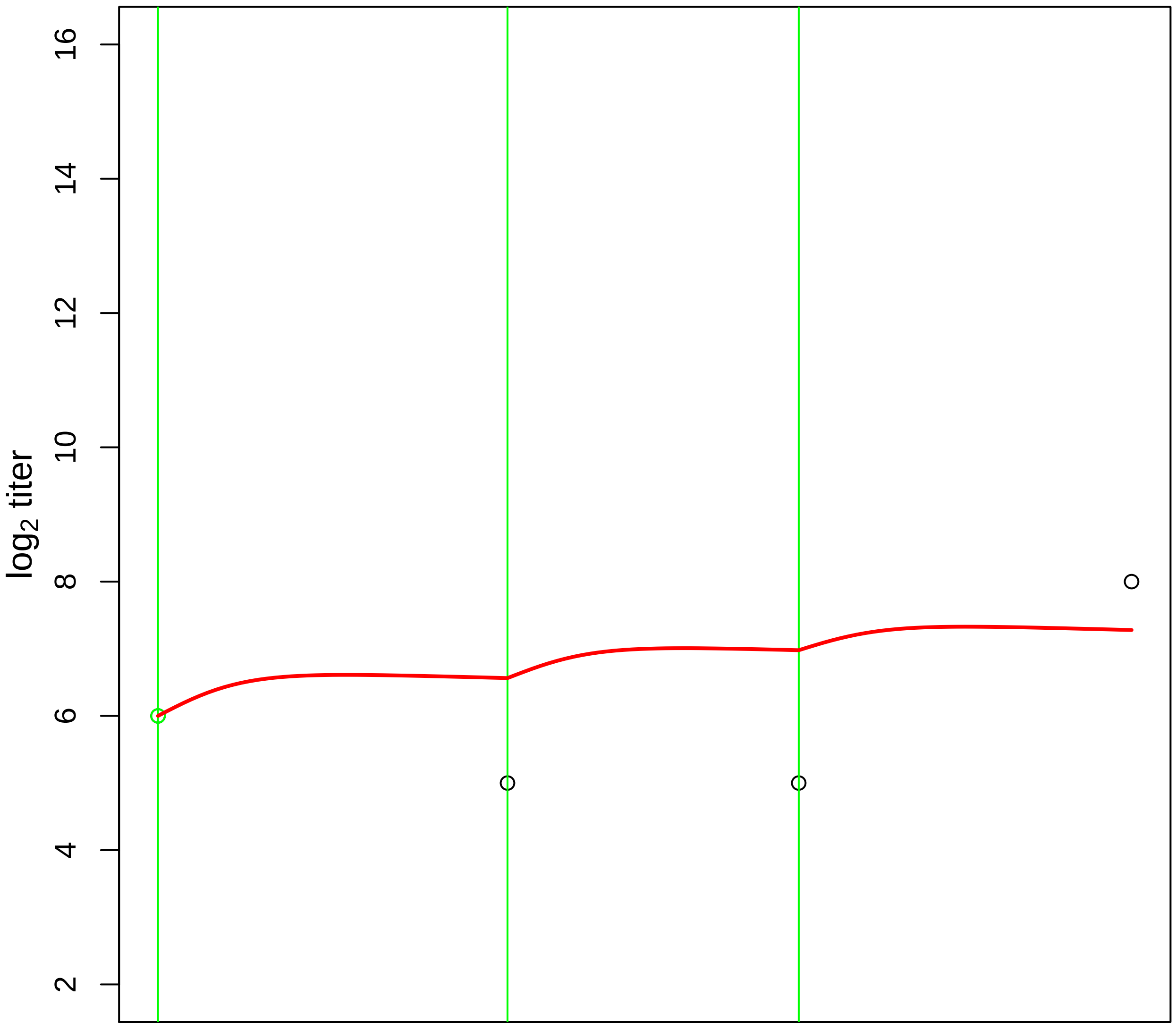

time in years from first donation of donor 718
mean absolute errors = 1.421 , mean squared errors = 2.292

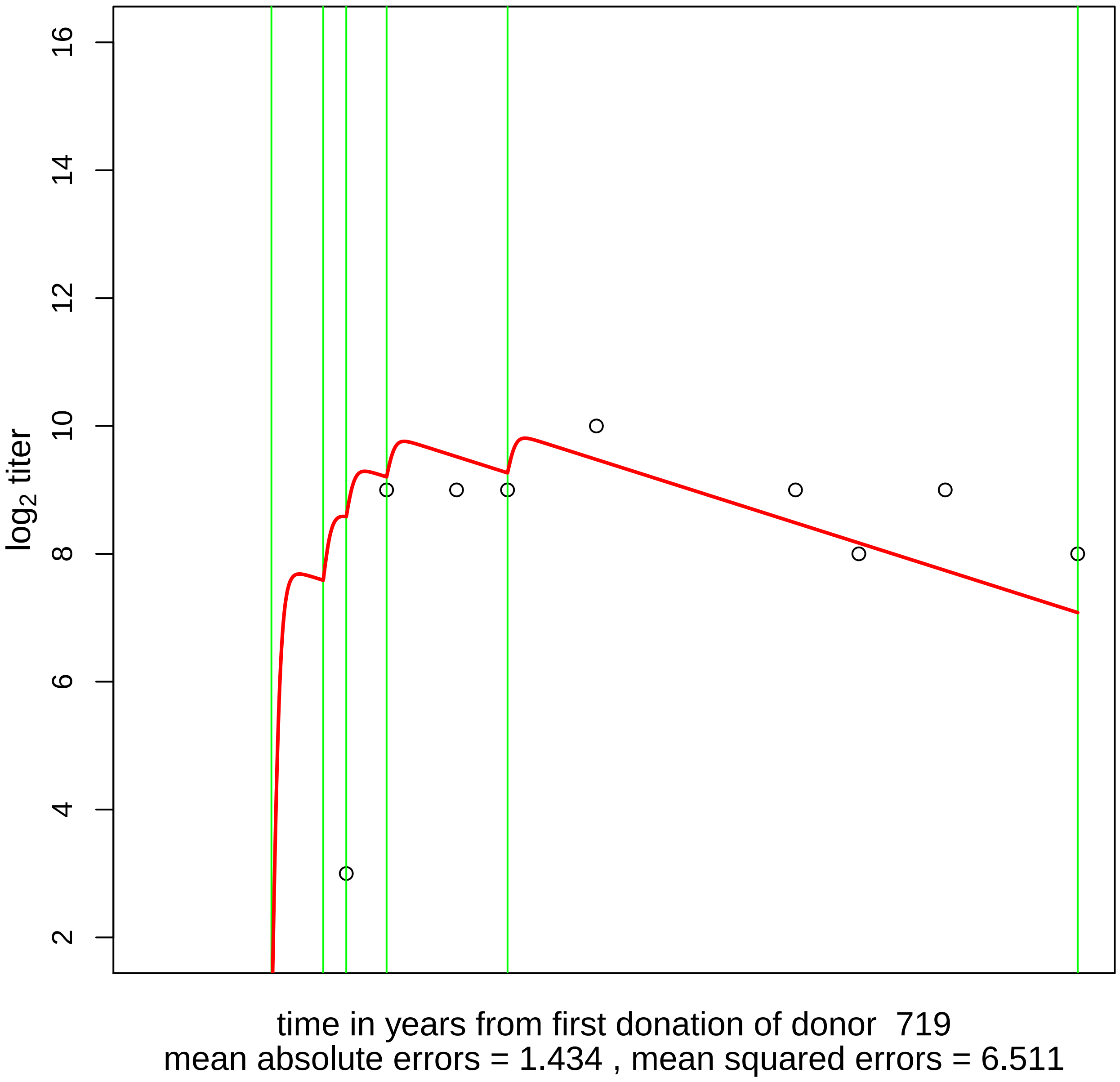

16
14
12
10
8
6
4
2
$\log_2$ titer
time in years from first donation of donor 719
mean absolute errors = 1.434 , mean squared errors = 6.511

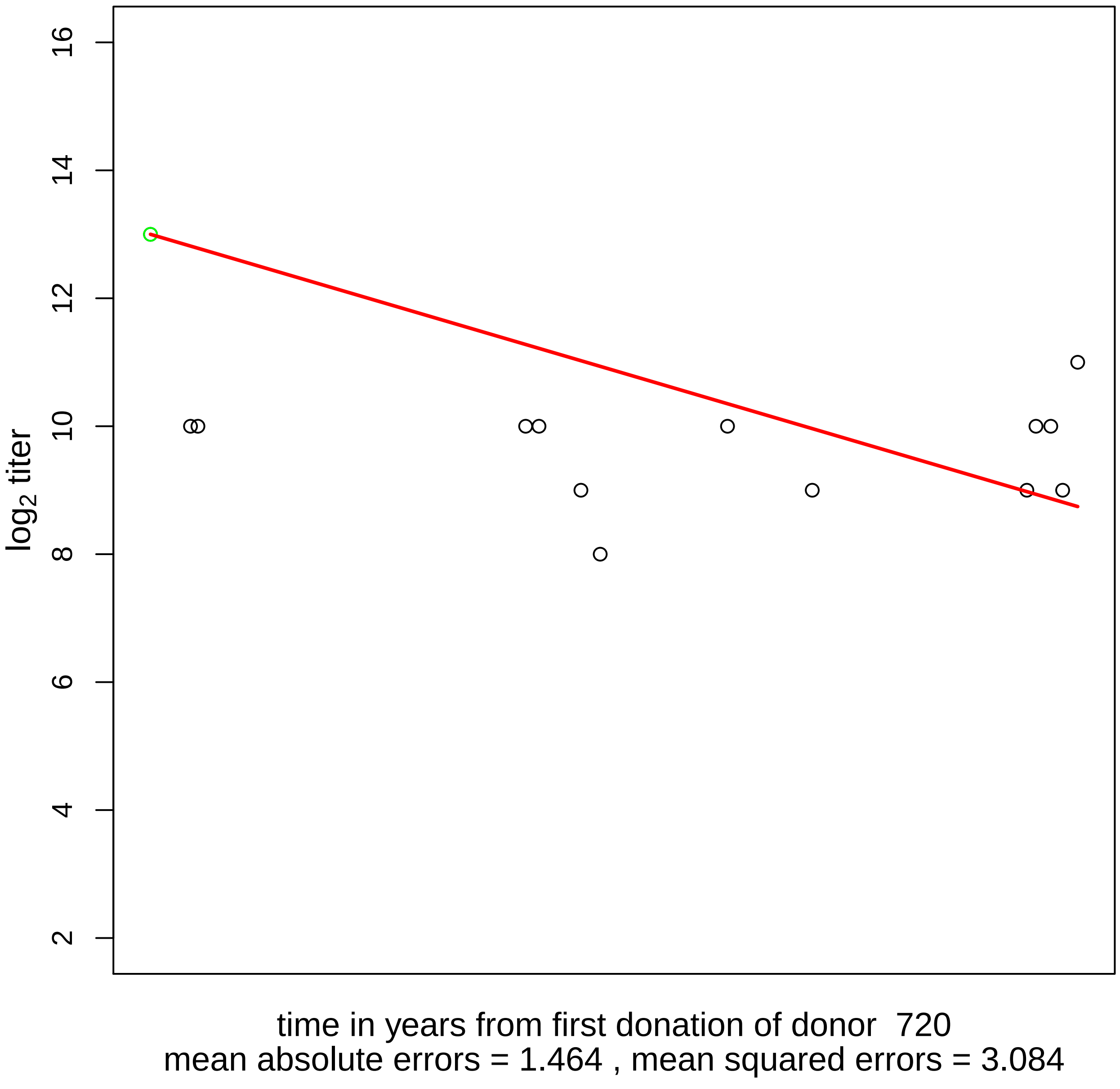

log2 titer
2
4
6
8
10
12
14
16
time in years from first donation of donor 720
mean absolute errors = 1.464 , mean squared errors = 3.084

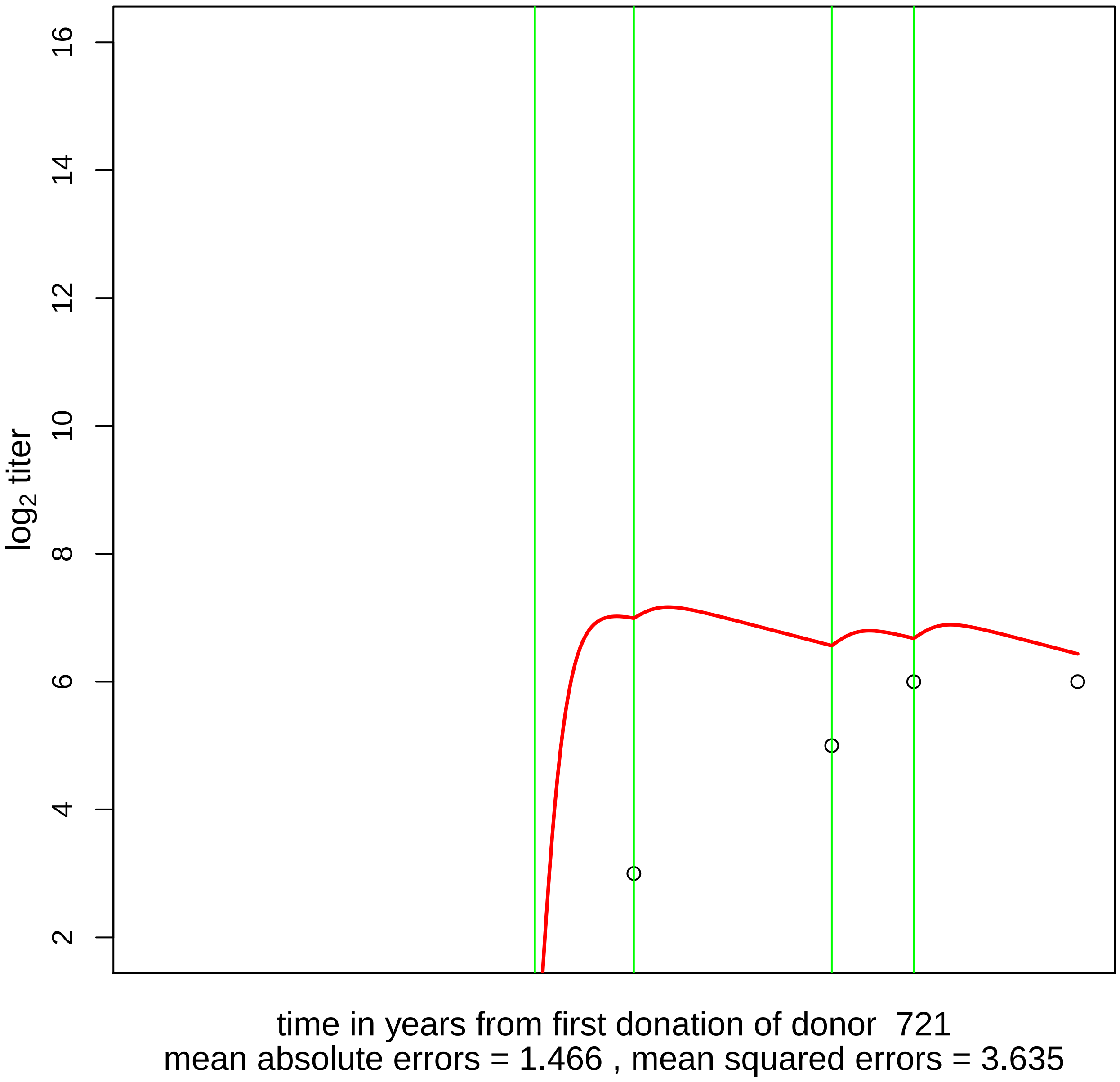

16
14
12
10
8
6
4
2
log2 titer
time in years from first donation of donor 721
mean absolute errors = 1.466 , mean squared errors = 3.635

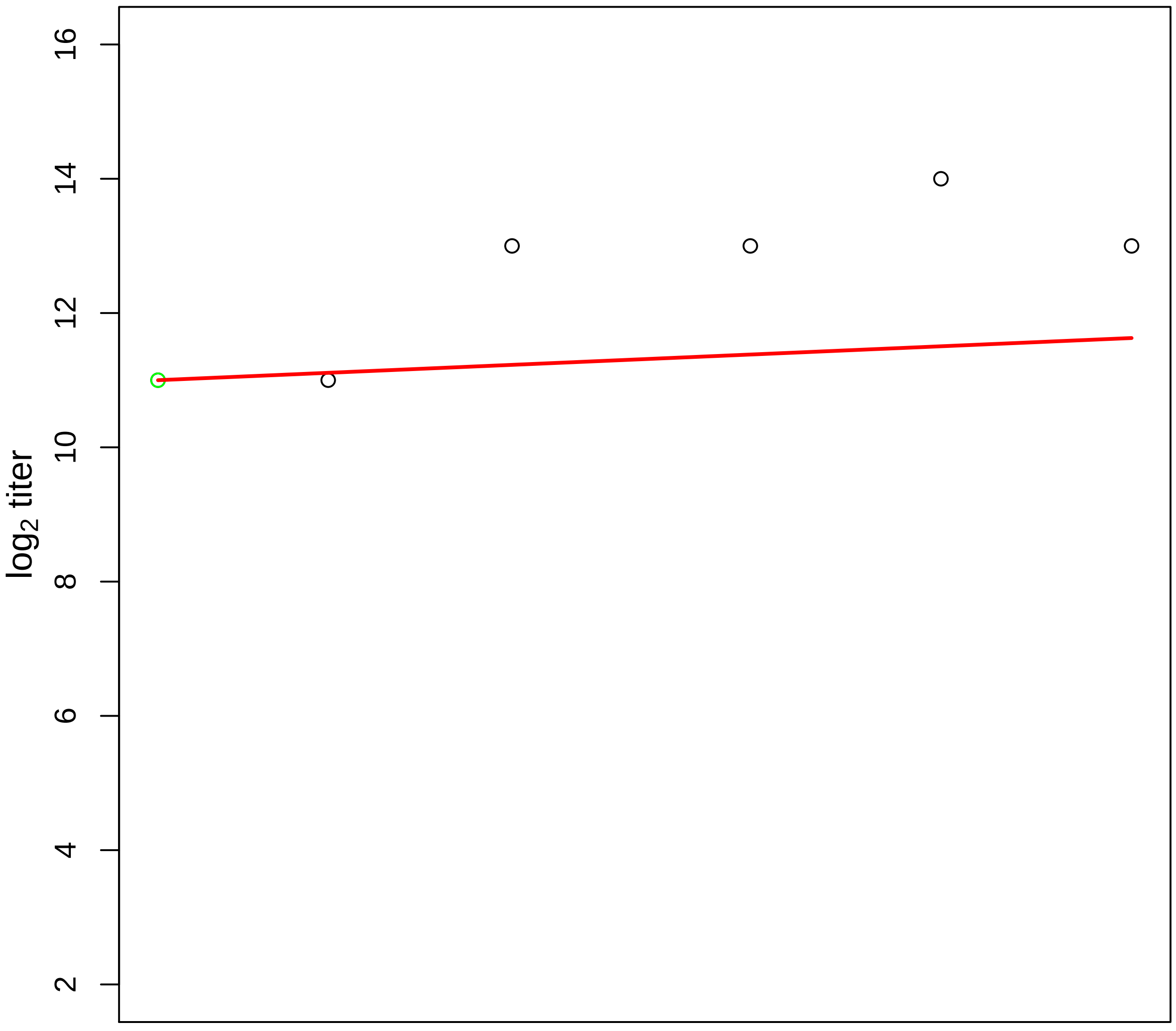

time in years from first donation of donor 722
mean absolute errors = 1.473 , mean squared errors = 2.776

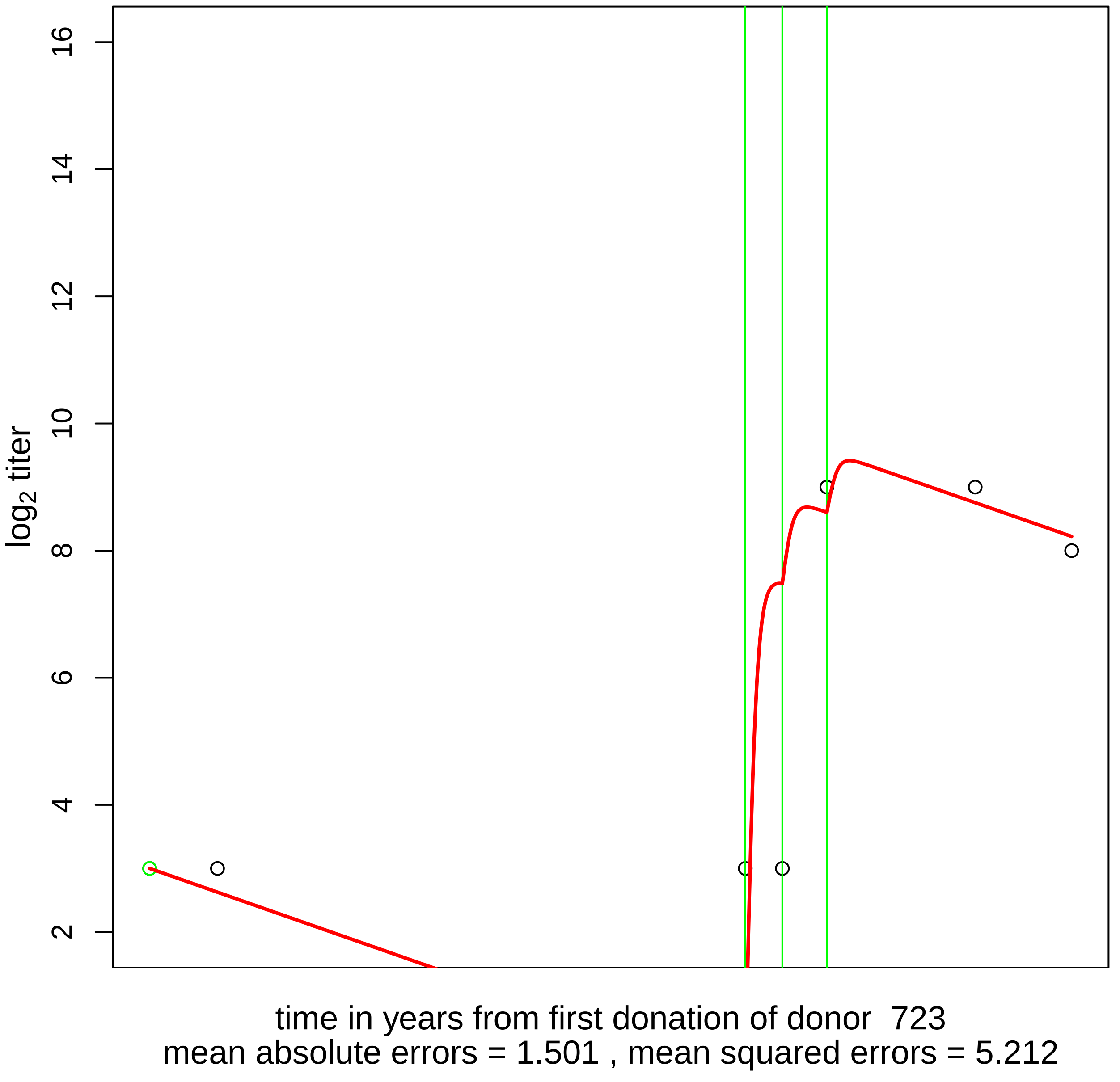

16
14
12
10
8
6
4
2
$\log_2$ titer
time in years from first donation of donor 723
mean absolute errors = 1.501 , mean squared errors = 5.212

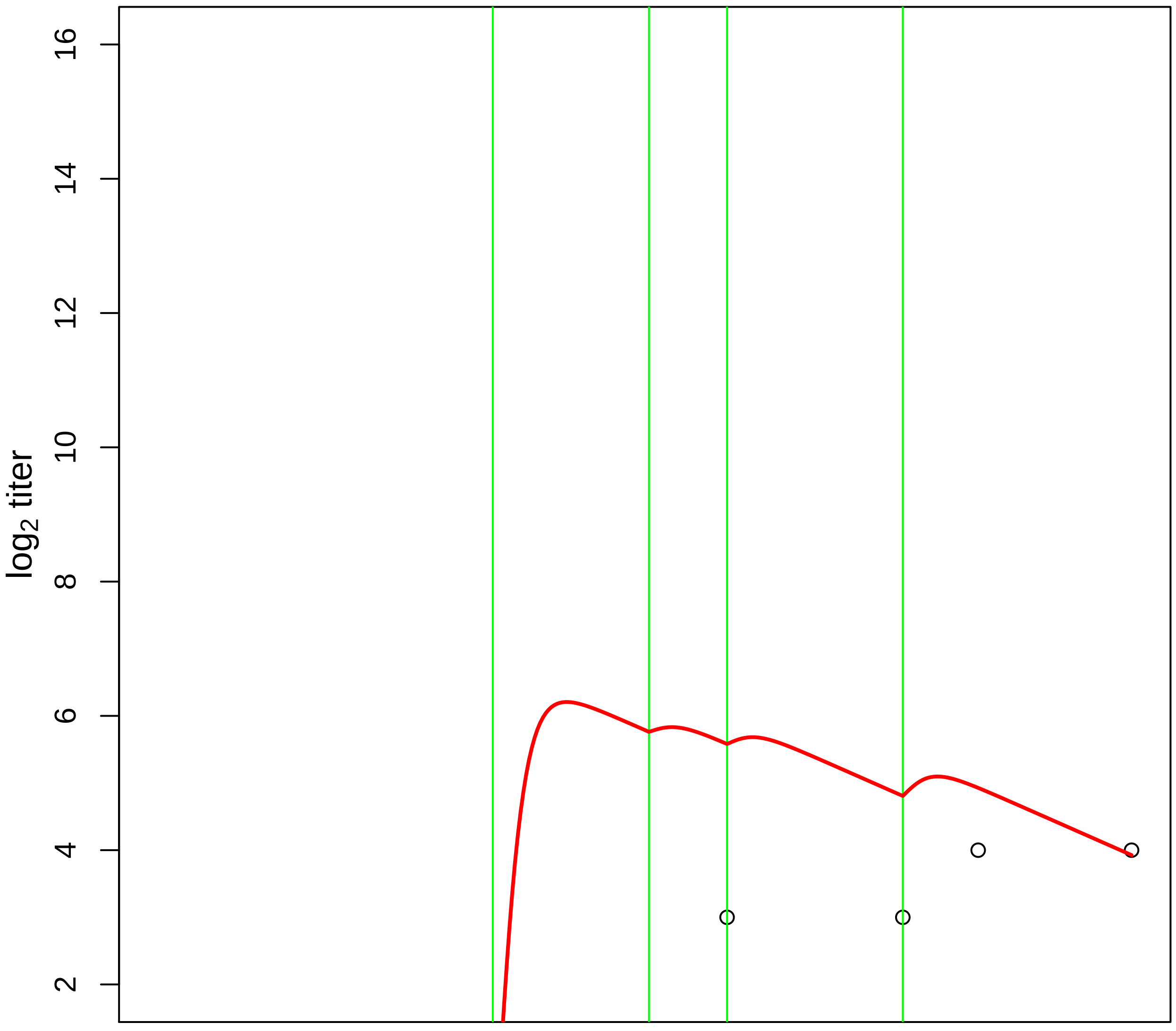

time in years from first donation of donor 724
mean absolute errors = 1.557 , mean squared errors = 4.861

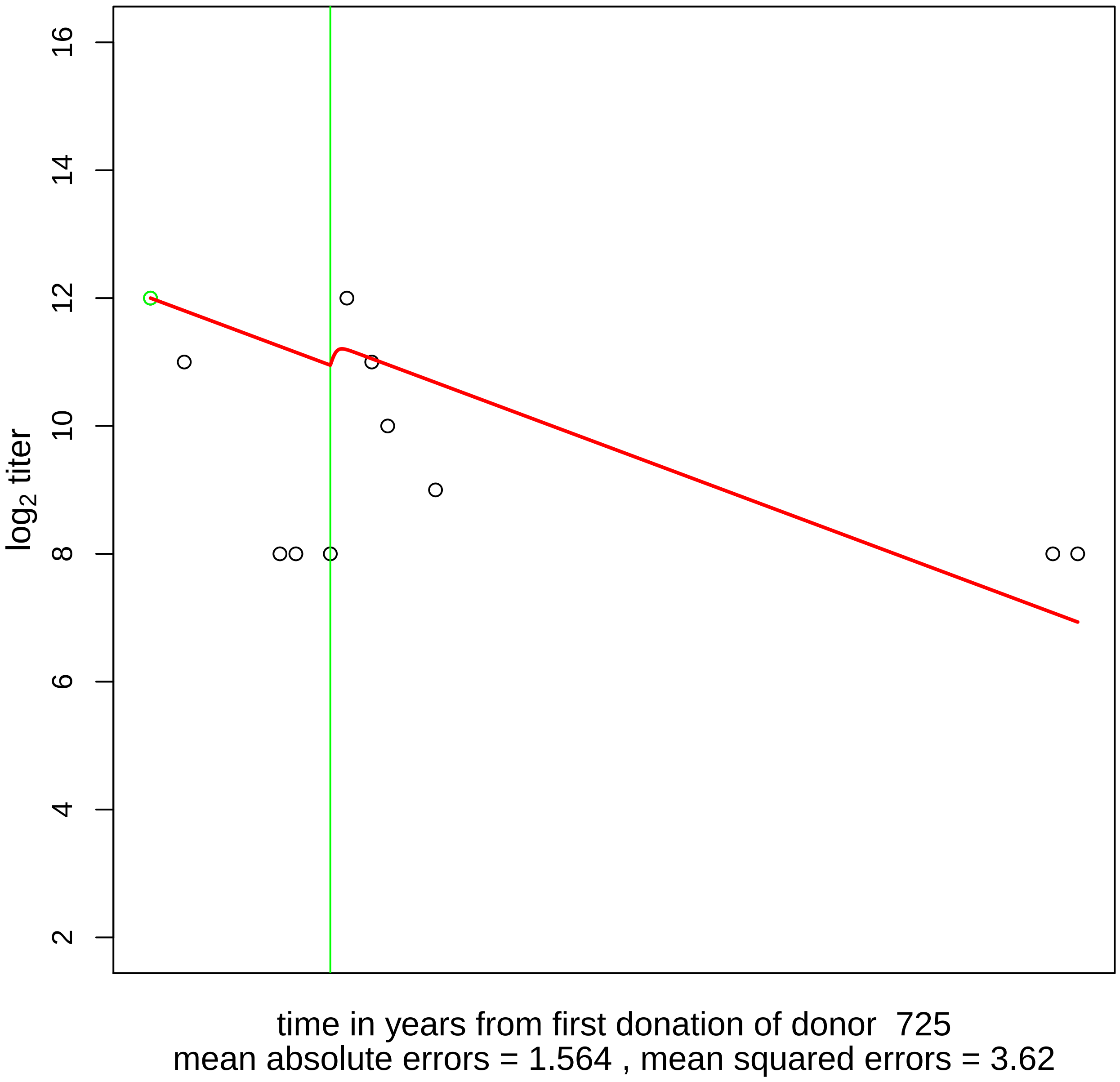

16
14
12
10
8
6
4
2
log2 titer
time in years from first donation of donor 725
mean absolute errors = 1.564 , mean squared errors = 3.62

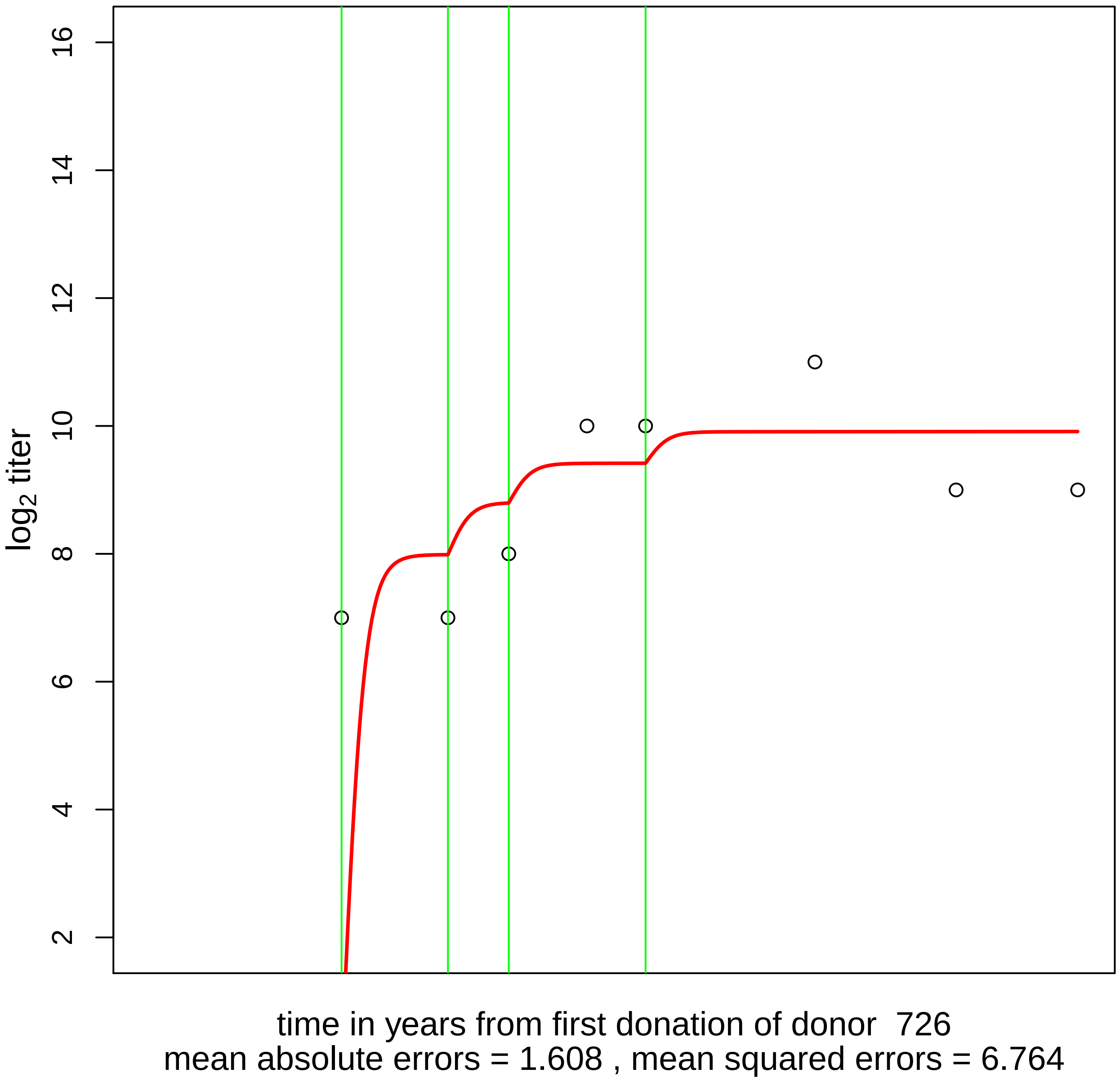

log2 titer
2
4
6
8
10
12
14
16
time in years from first donation of donor 726
mean absolute errors = 1.608 , mean squared errors = 6.764

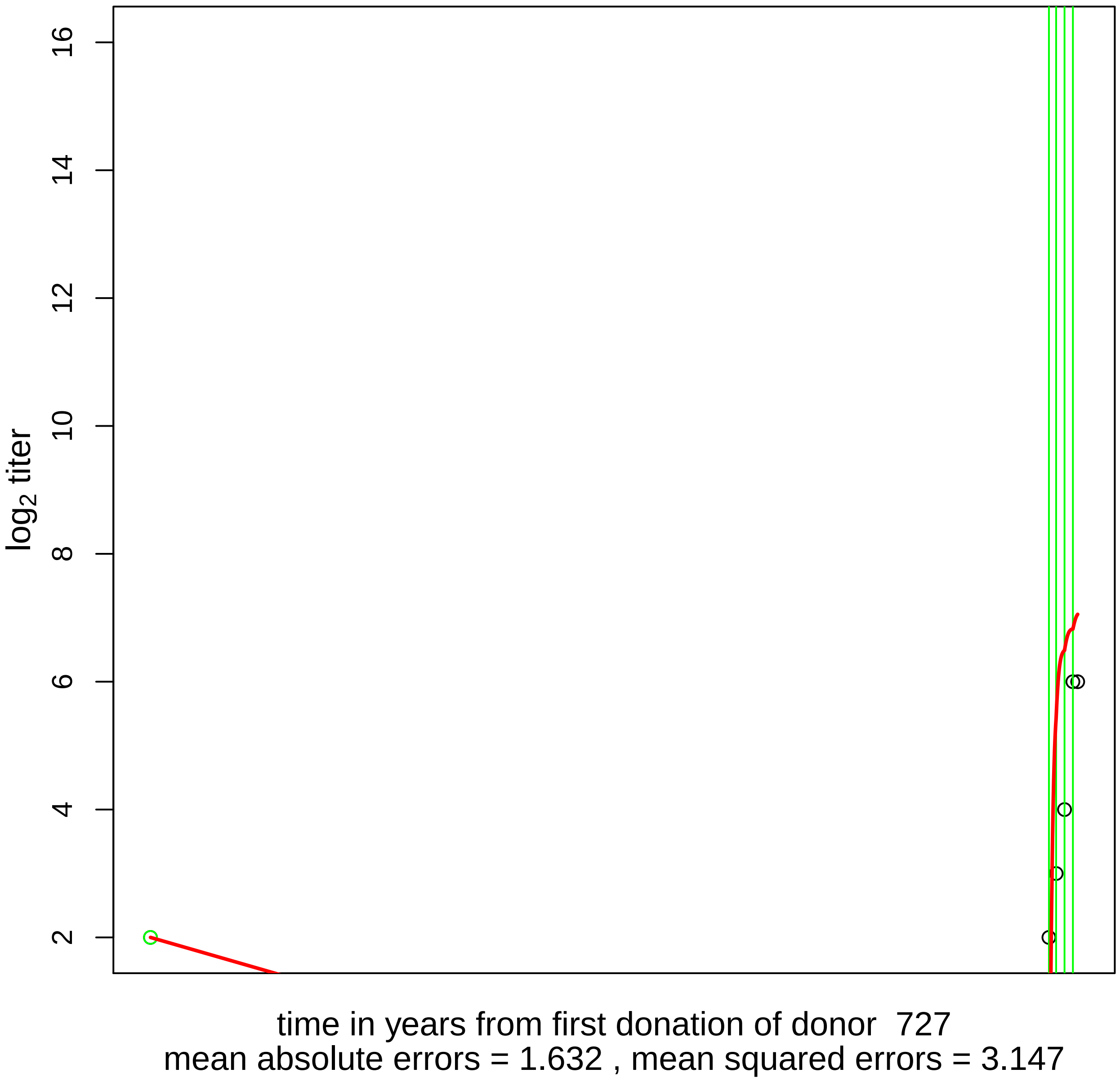

16
14
12
10
8
6
4
2
log2 titer
time in years from first donation of donor 727
mean absolute errors = 1.632 , mean squared errors = 3.147

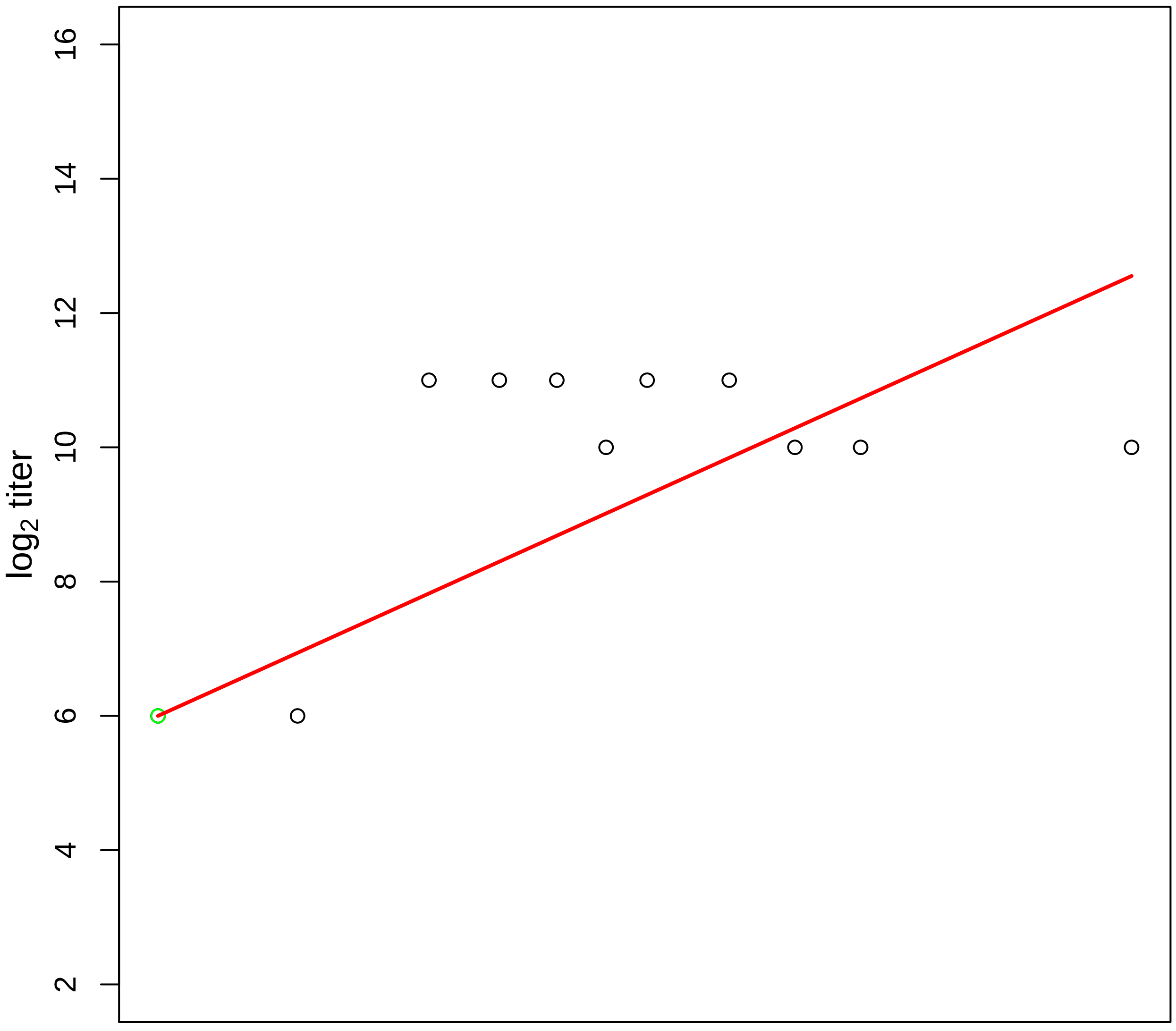

time in years from first donation of donor 728
mean absolute errors = 1.655 , mean squared errors = 3.599

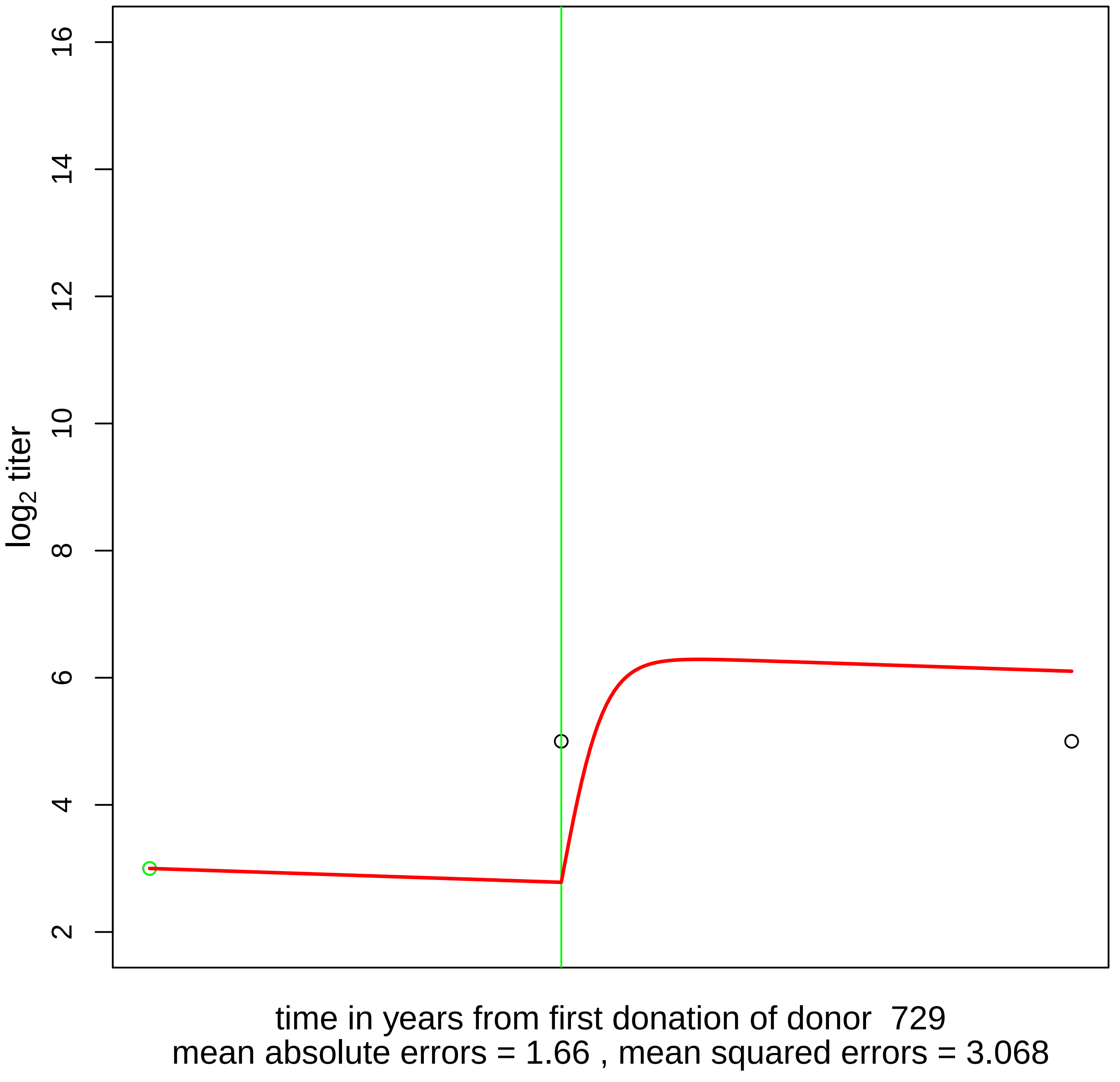

log2 titer
2
4
6
8
10
12
14
16
time in years from first donation of donor 729
mean absolute errors = 1.66 , mean squared errors = 3.068

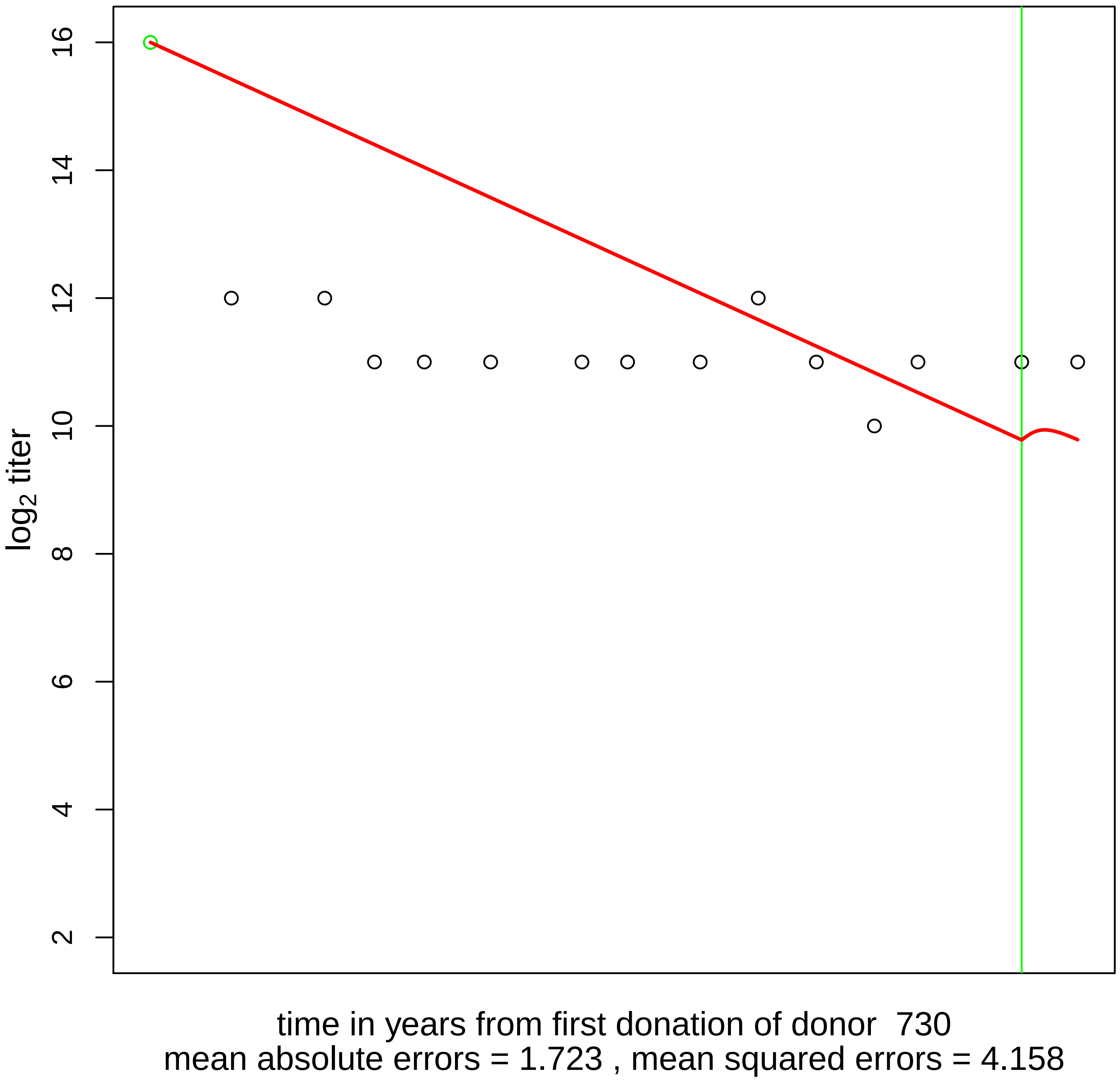
16
14
12
10
8
6
4
2
log2 titer
time in years from first donation of donor 730
mean absolute errors = 1.723 , mean squared errors = 4.158

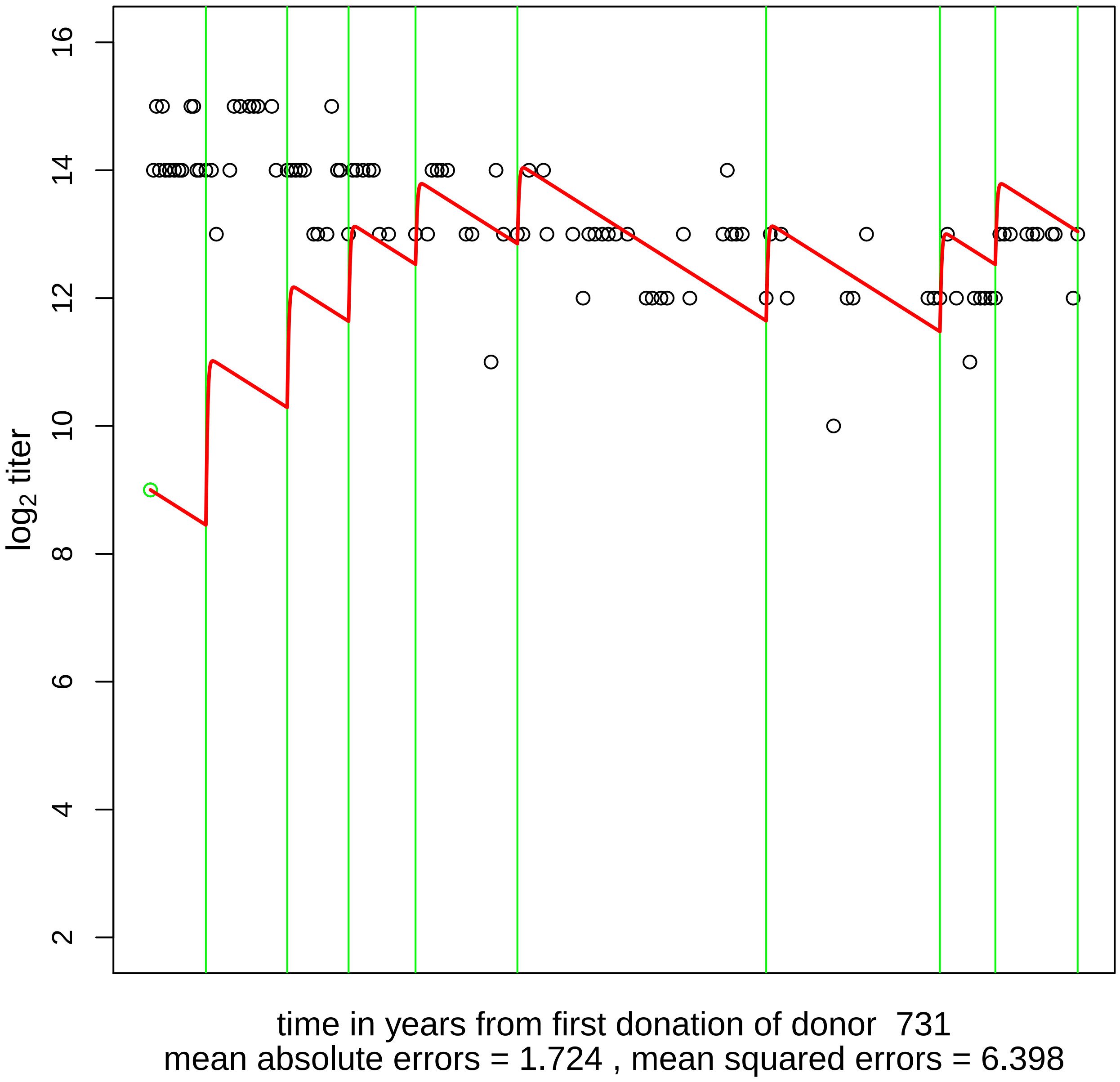

16
14
12
10
8
6
4
2
$\log_2$ titer
time in years from first donation of donor 731
mean absolute errors = 1.724 , mean squared errors = 6.398

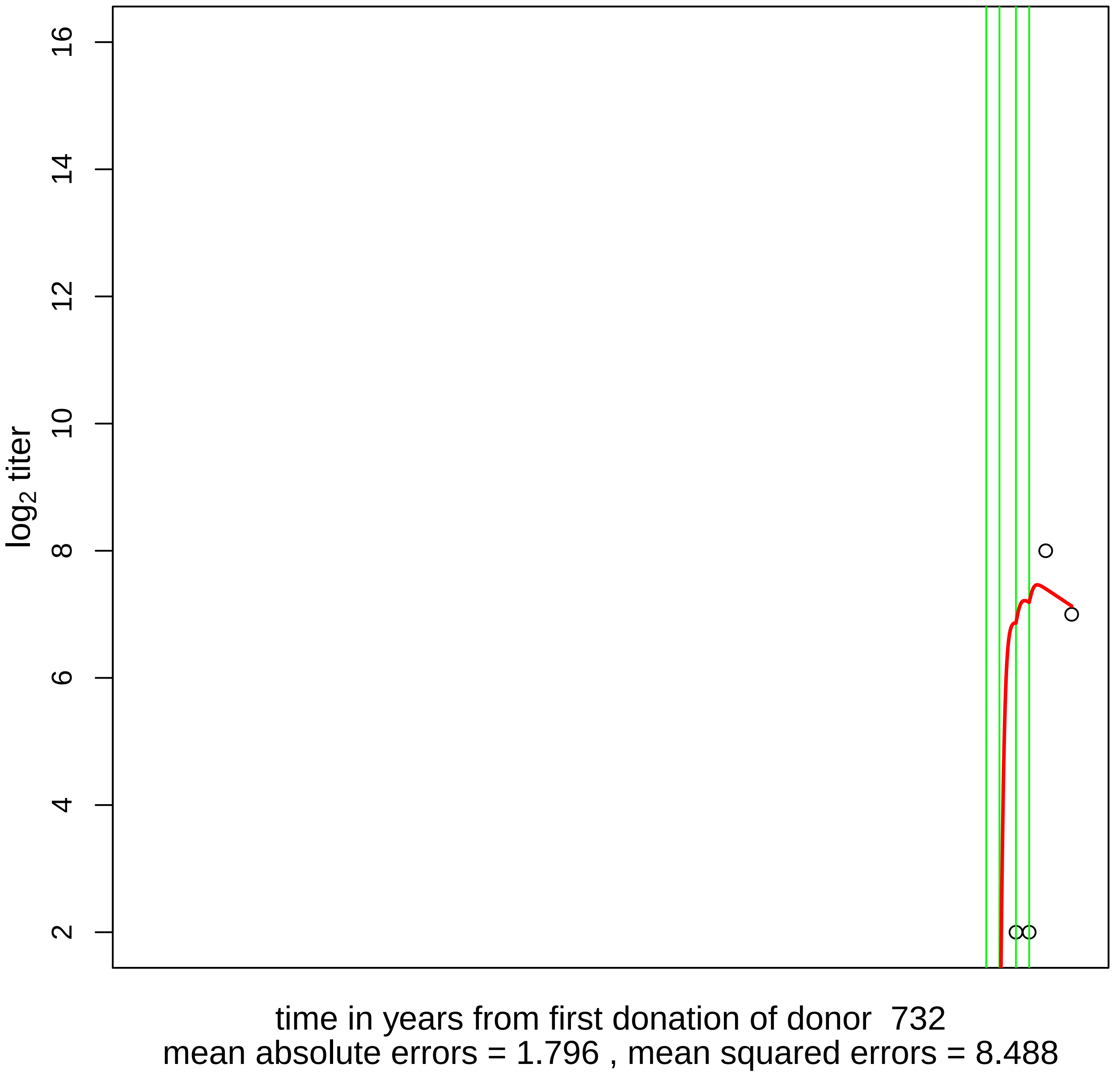
log2 titer
2
4
6
8
10
12
14
16
time in years from first donation of donor 732
mean absolute errors = 1.796 , mean squared errors = 8.488

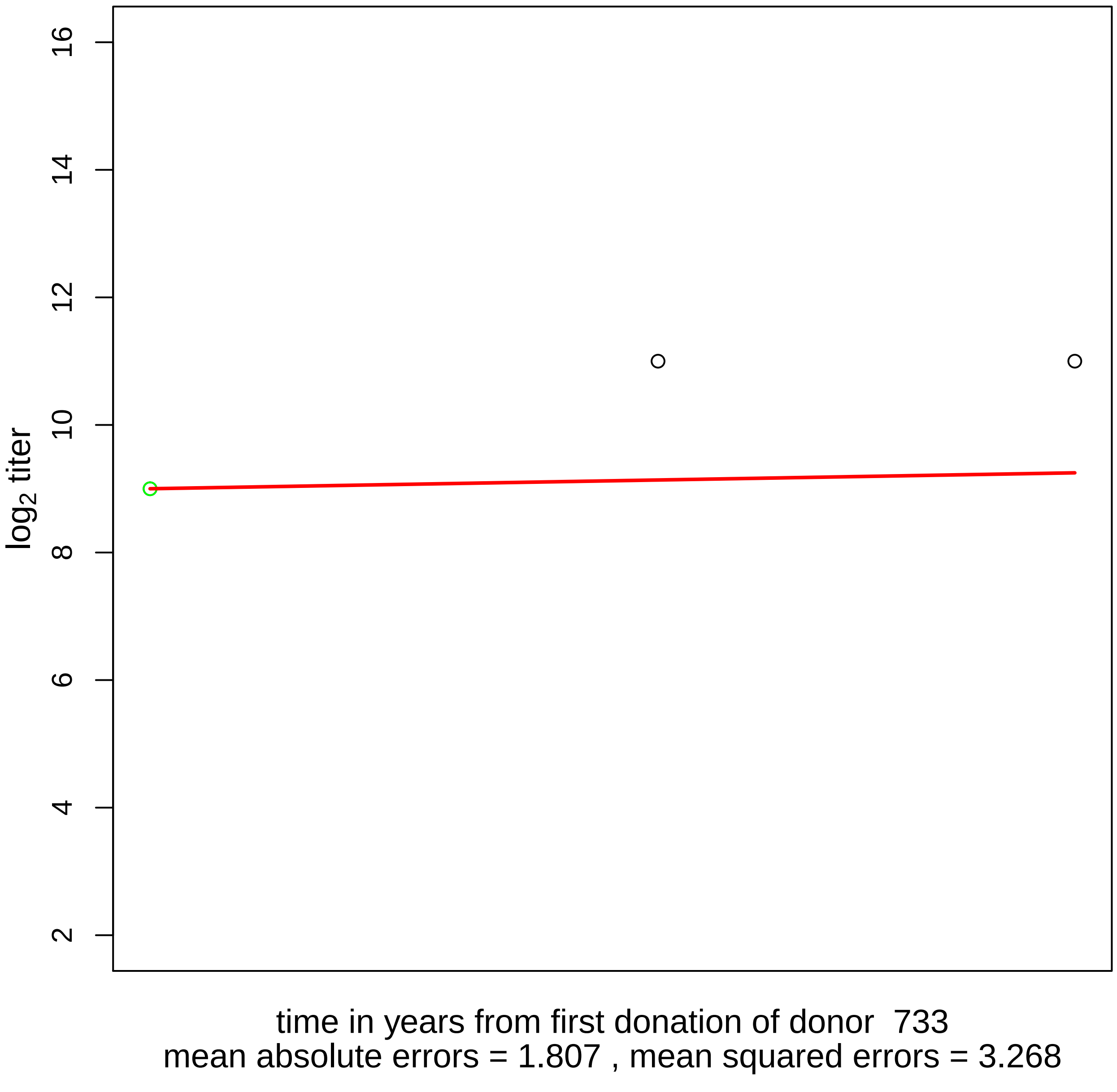

log2 titer
16
14
12
10
8
6
4
2
time in years from first donation of donor 733
mean absolute errors = 1.807 , mean squared errors = 3.268

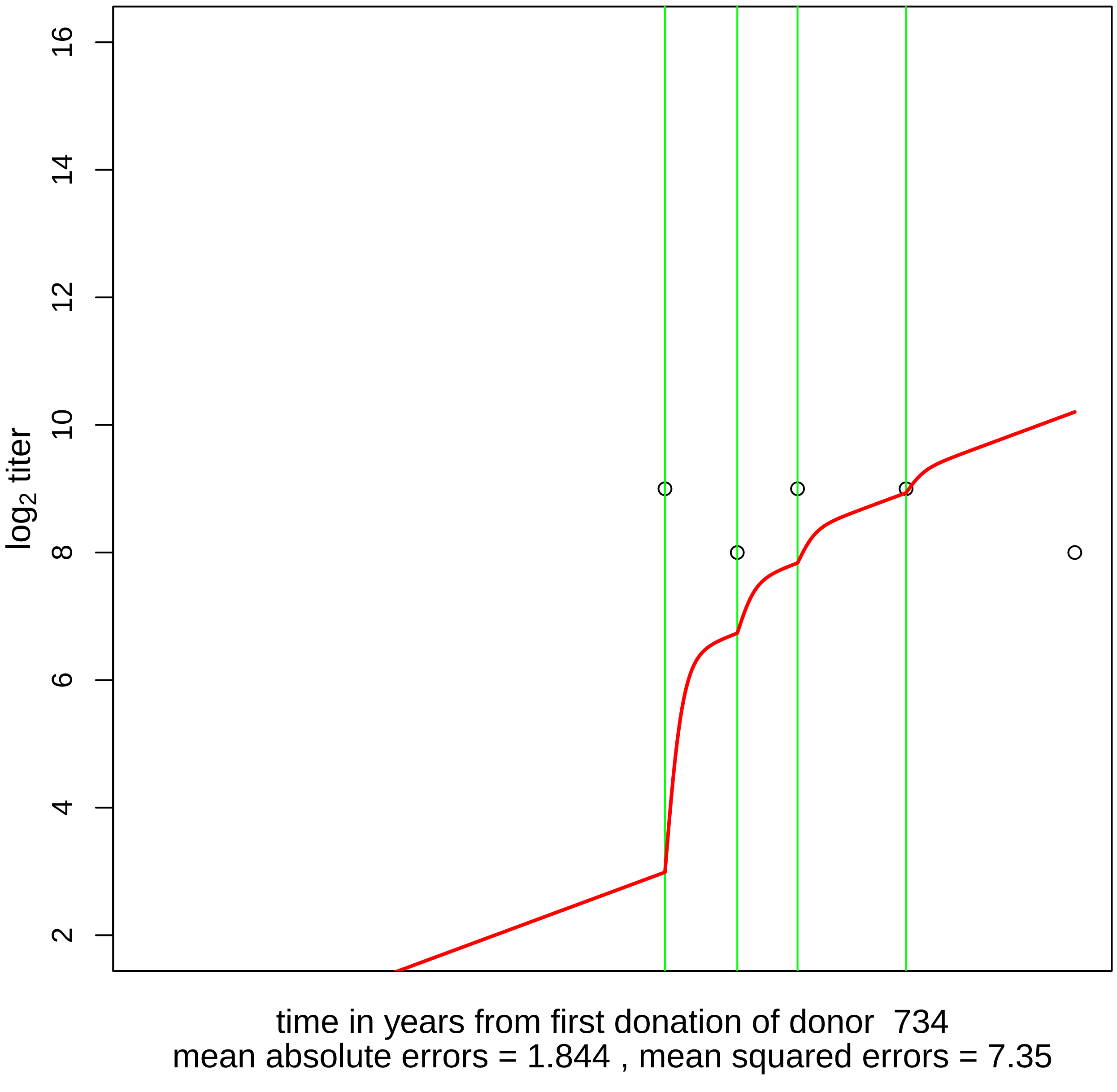

16
14
12
10
8
6
4
2
log2 titer
time in years from first donation of donor 734
mean absolute errors = 1.844 , mean squared errors = 7.35

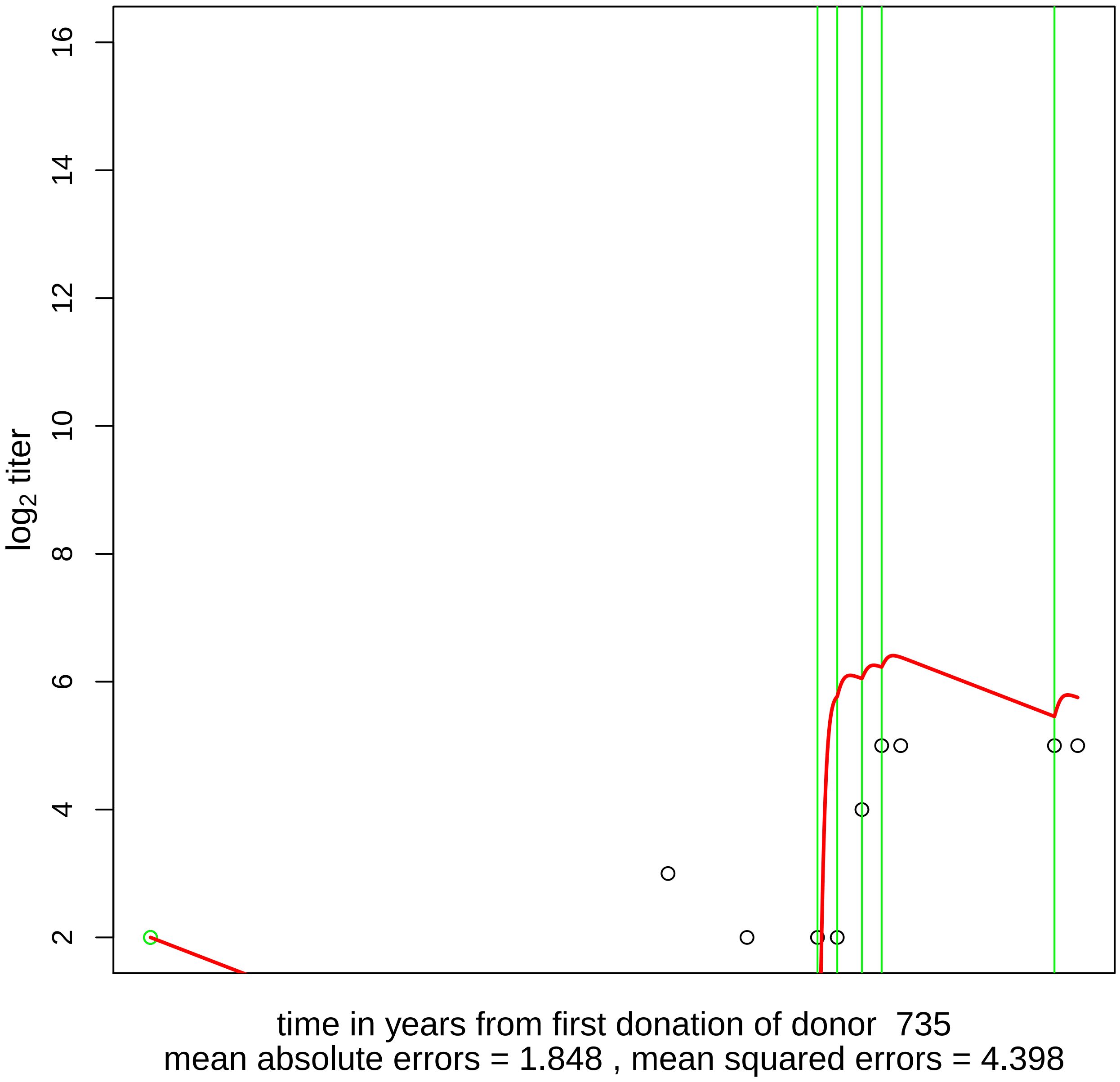

log2 titer
2
4
6
8
10
12
14
16
time in years from first donation of donor 735
mean absolute errors = 1.848 , mean squared errors = 4.398

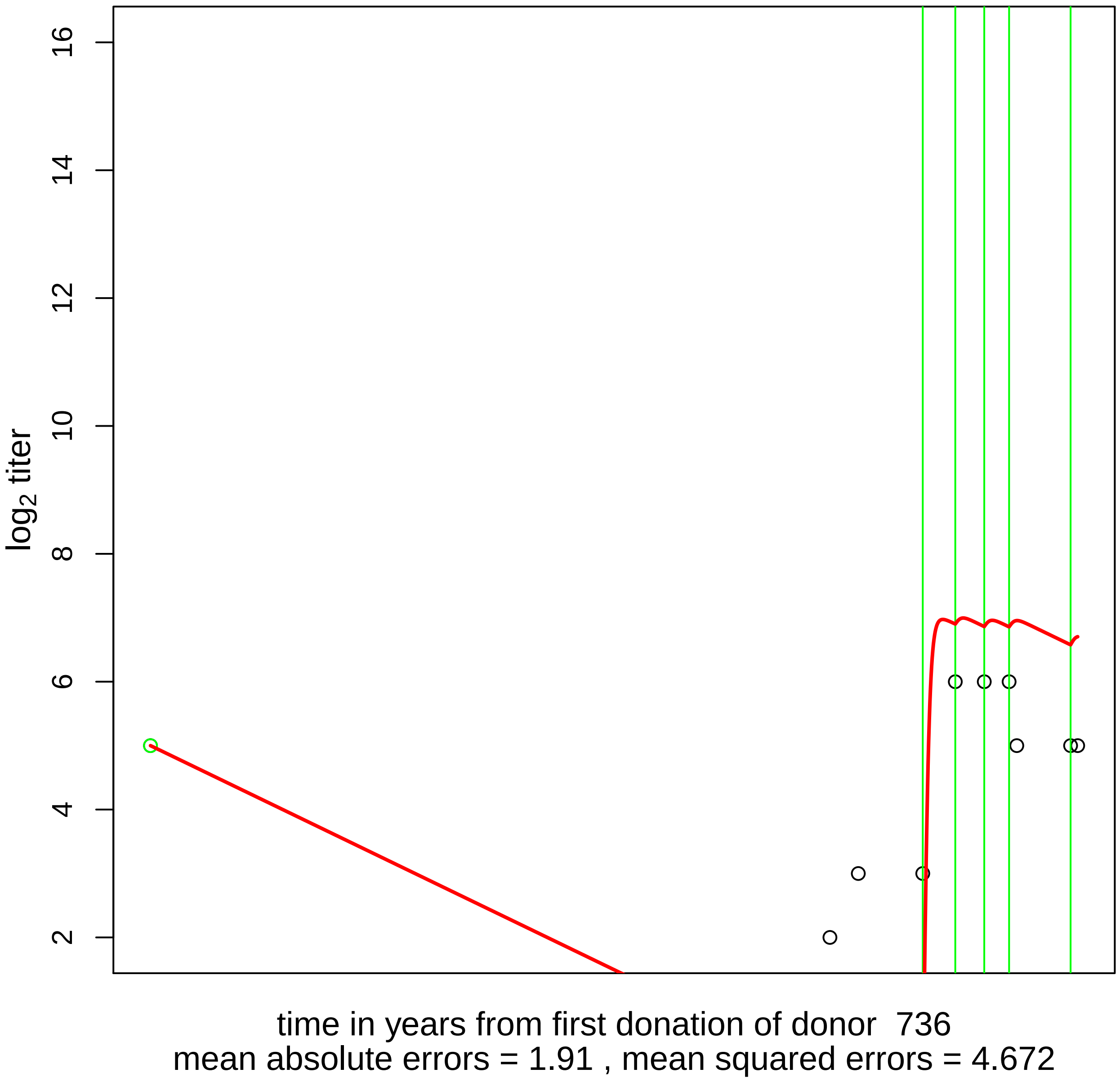

log2 titer
16
14
12
10
8
6
4
2
time in years from first donation of donor 736
mean absolute errors = 1.91 , mean squared errors = 4.672

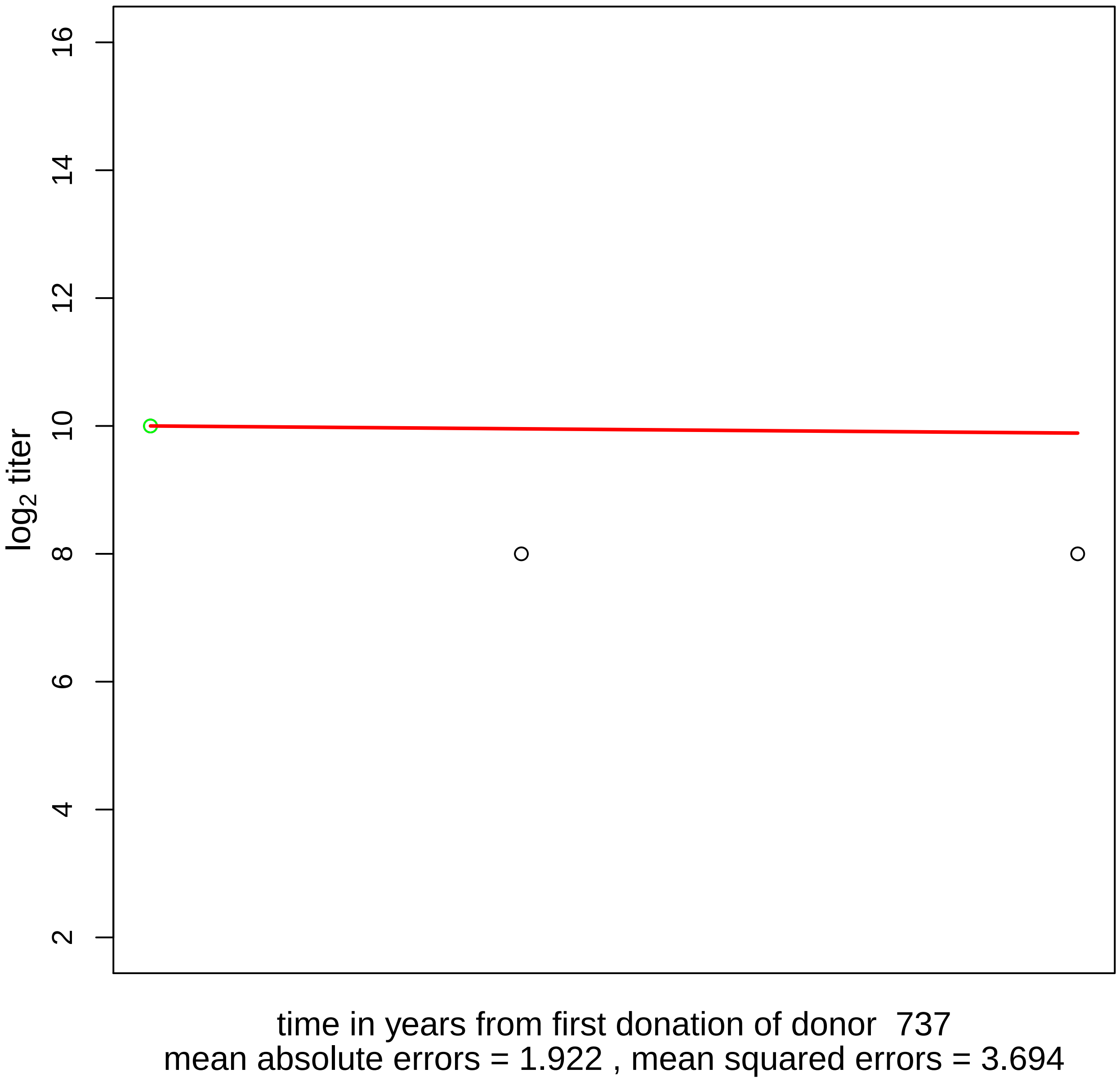

16
14
12
10
8
6
4
2
$\log_2$ titer
time in years from first donation of donor 737
mean absolute errors = 1.922 , mean squared errors = 3.694

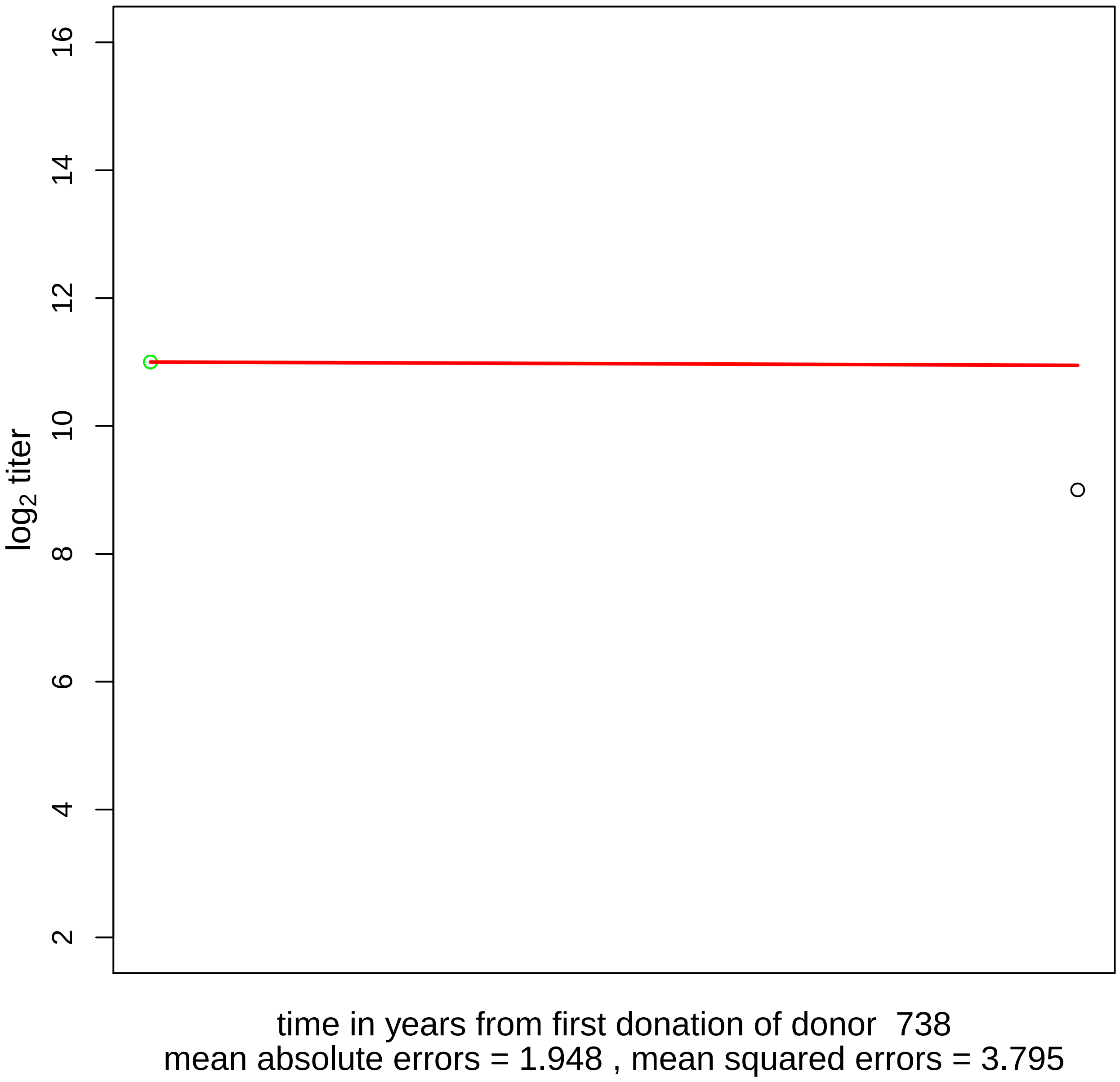

log2 titer
16
14
12
10
8
6
4
2
time in years from first donation of donor 738
mean absolute errors = 1.948 , mean squared errors = 3.795

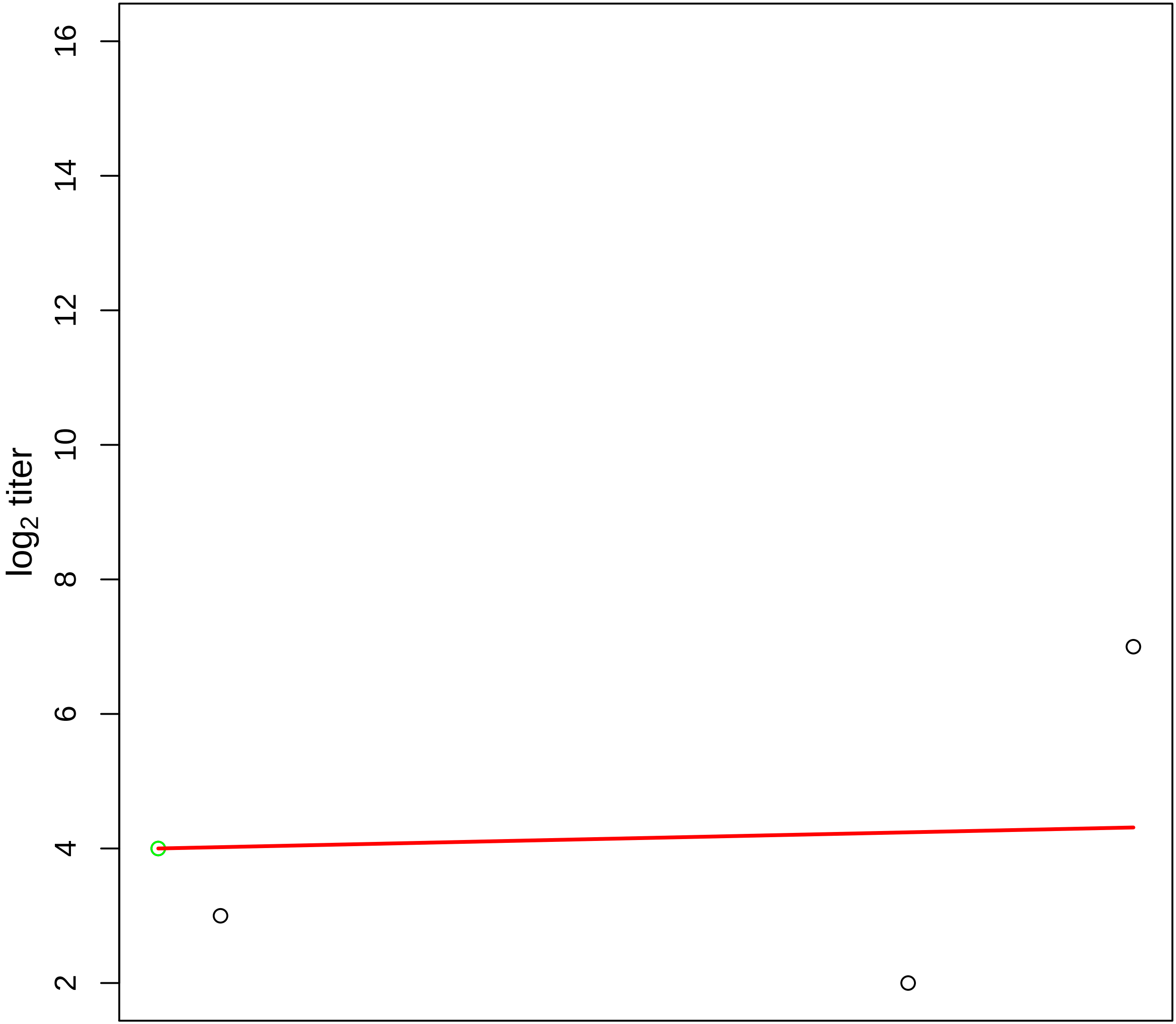

time in years from first donation of donor 739
mean absolute errors = 1.983 , mean squared errors = 4.427

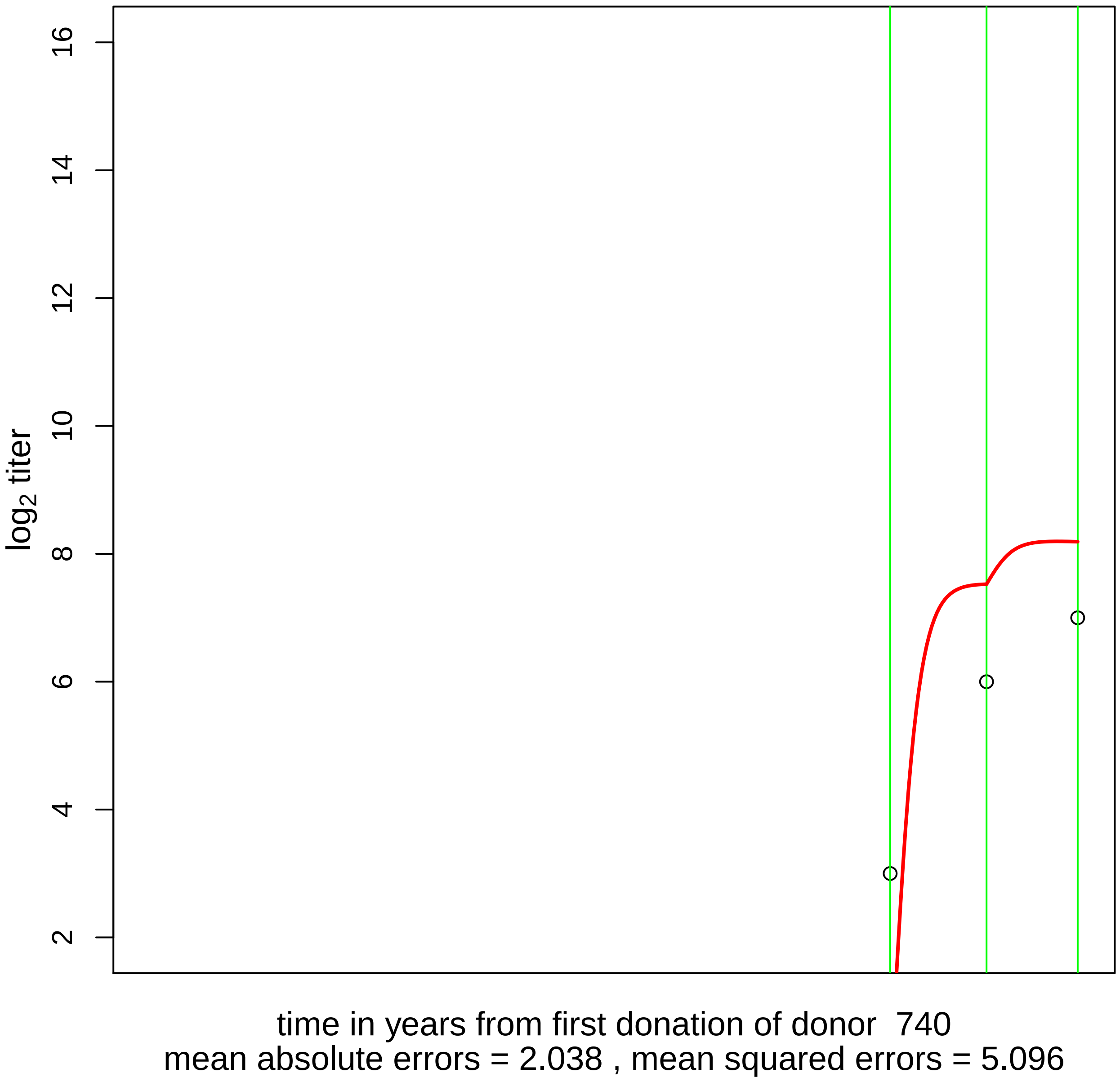
log2 titer
2
4
6
8
10
12
14
16
time in years from first donation of donor 740
mean absolute errors = 2.038 , mean squared errors = 5.096

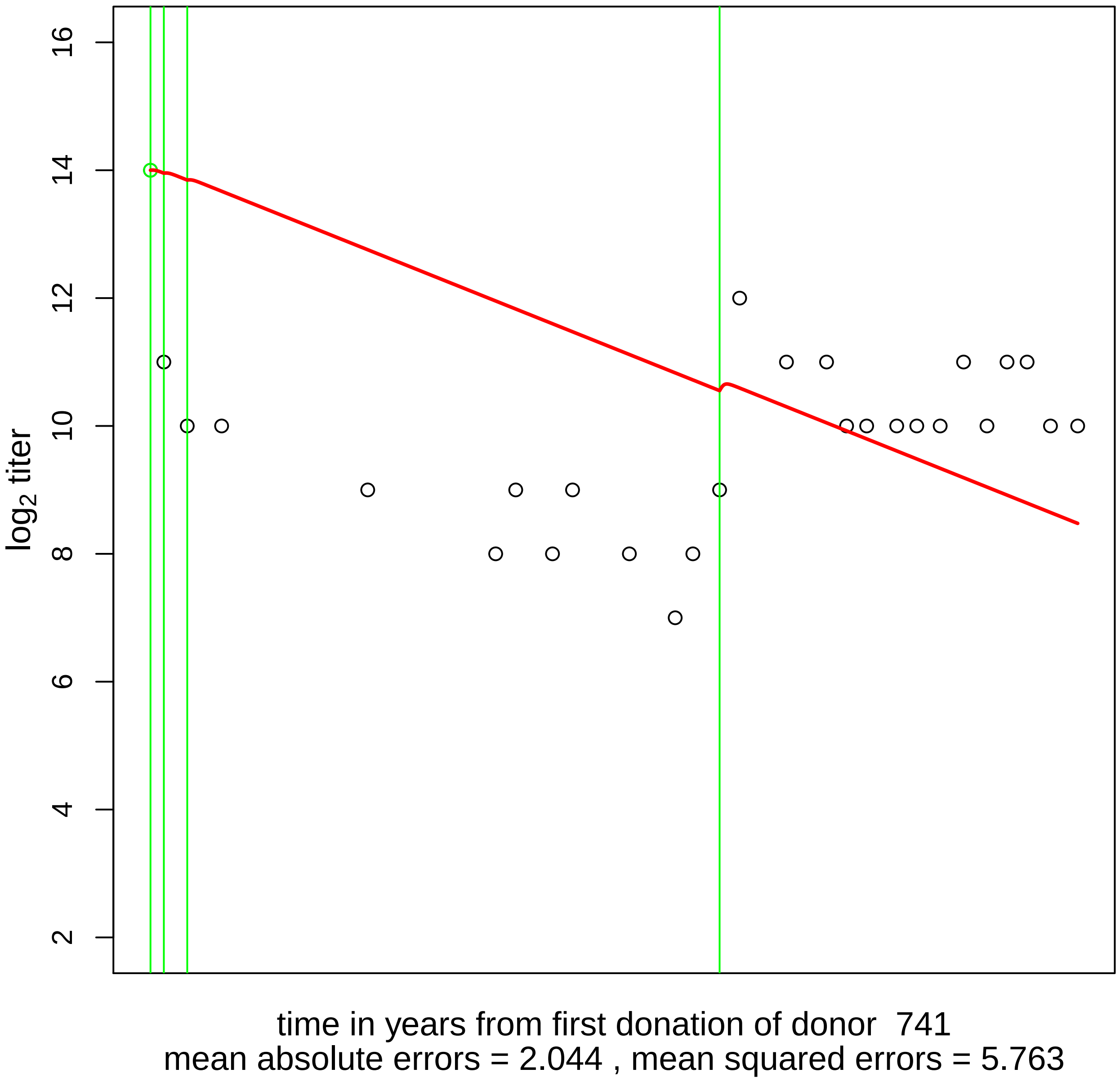

16
14
12
10
8
6
4
2
$\log_2$ titer
time in years from first donation of donor 741
mean absolute errors = 2.044 , mean squared errors = 5.763

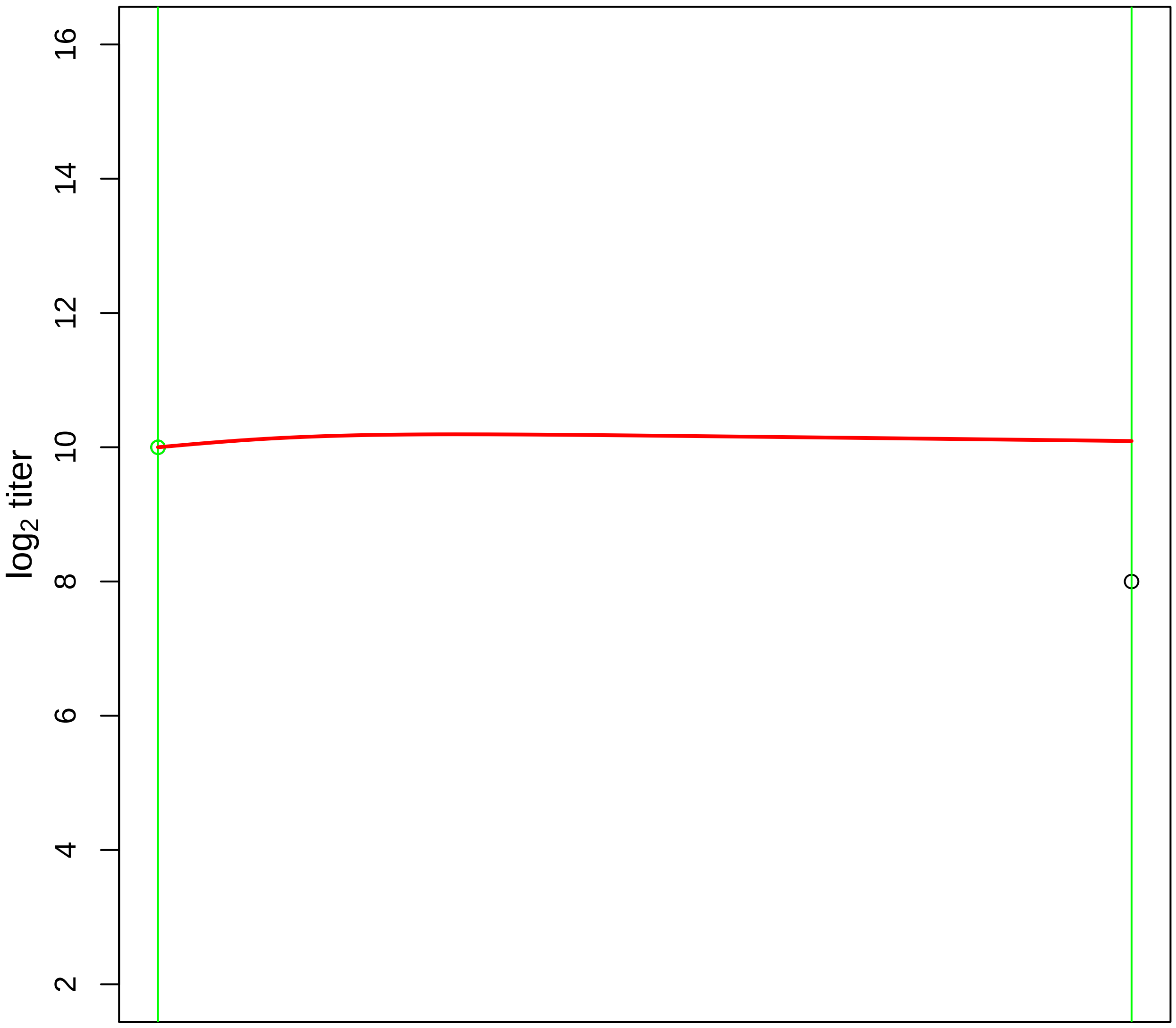

time in years from first donation of donor 742
mean absolute errors = 2.093 , mean squared errors = 4.379

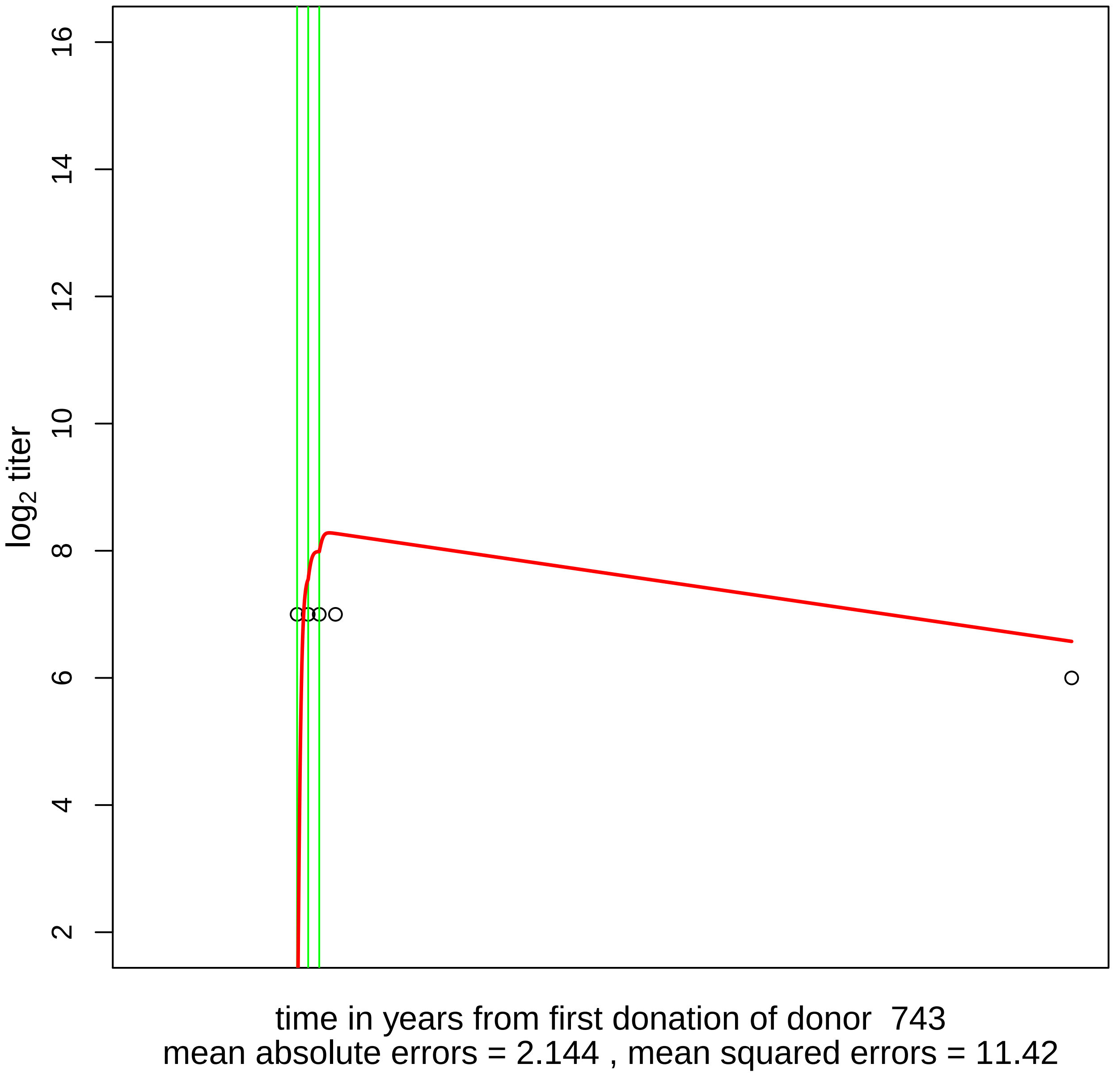
log2 titer
16
14
12
10
8
6
4
2
time in years from first donation of donor 743
mean absolute errors = 2.144 , mean squared errors = 11.42

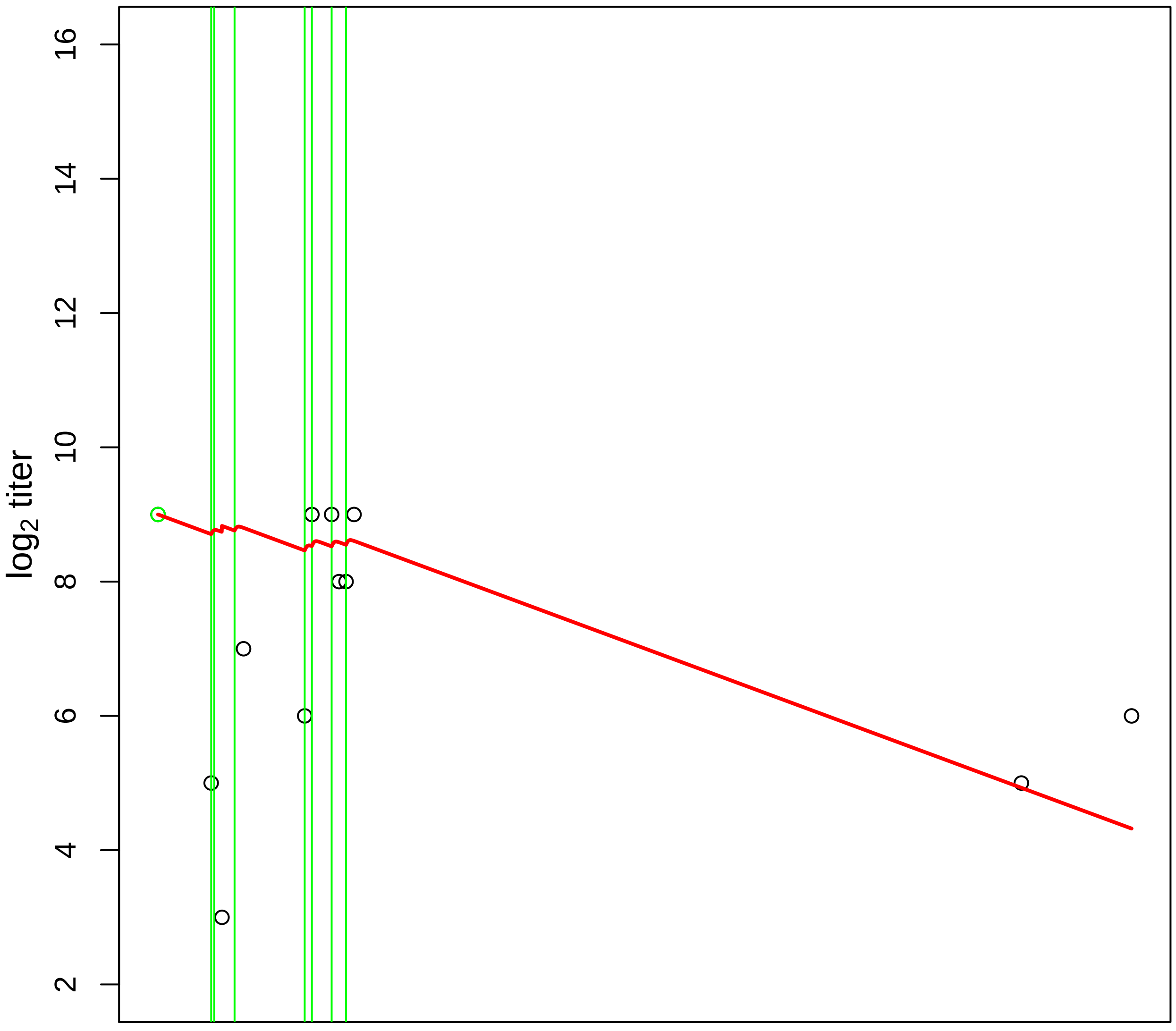

time in years from first donation of donor 744
mean absolute errors = 2.149 , mean squared errors = 10.11

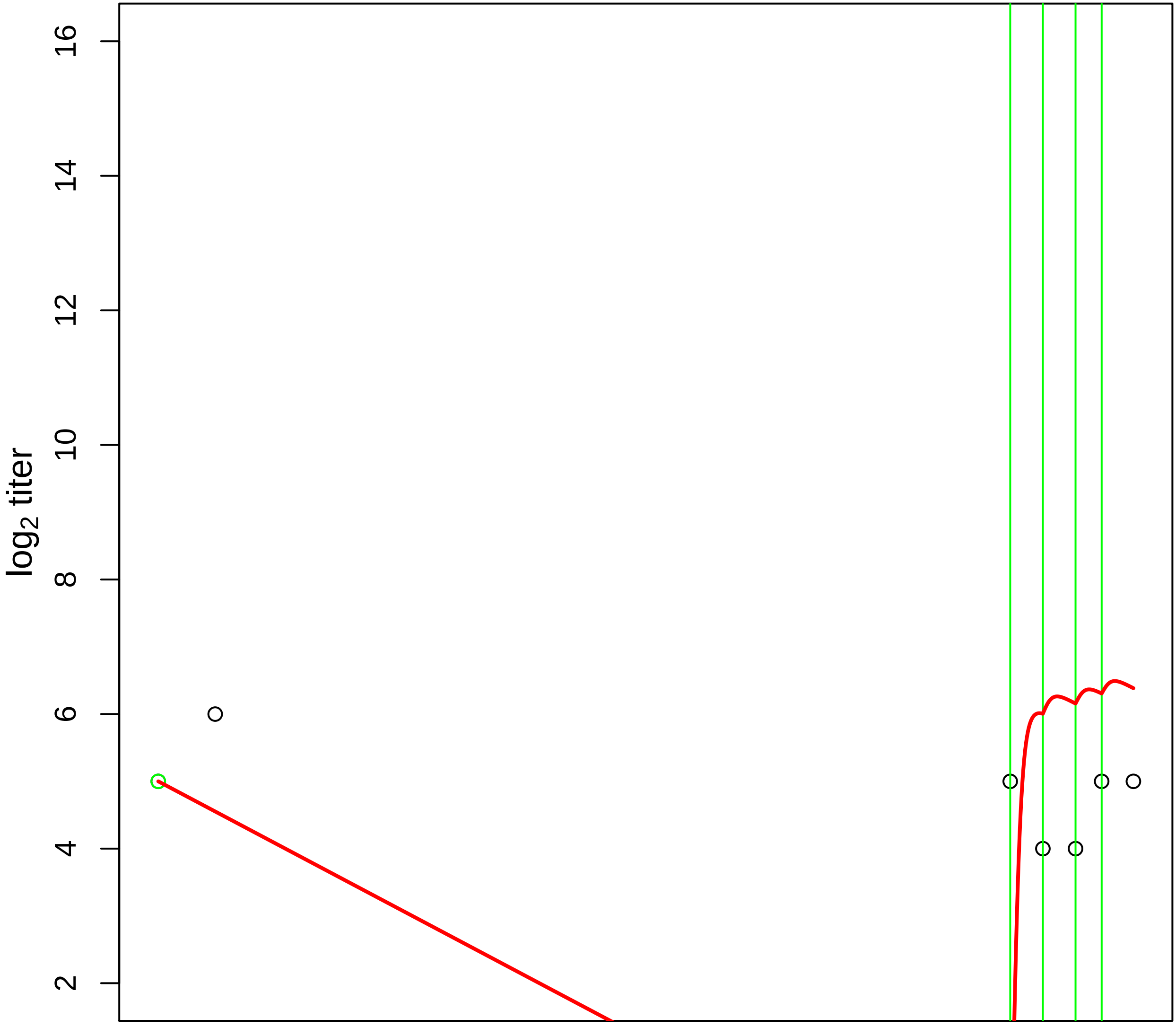

time in years from first donation of donor 745
mean absolute errors = 2.216 , mean squared errors = 6.564

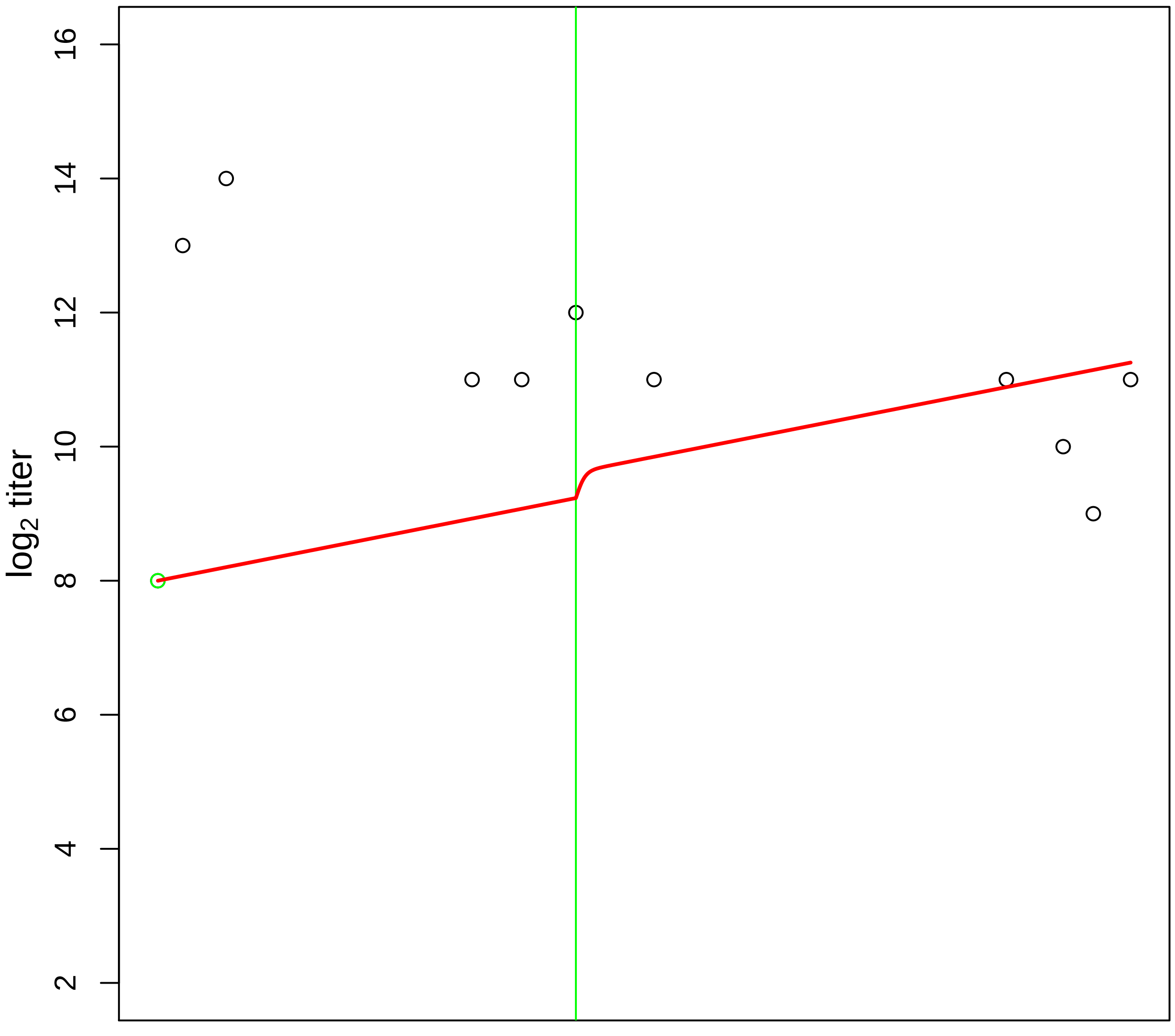

time in years from first donation of donor 746
mean absolute errors = 2.221 , mean squared errors = 8.068

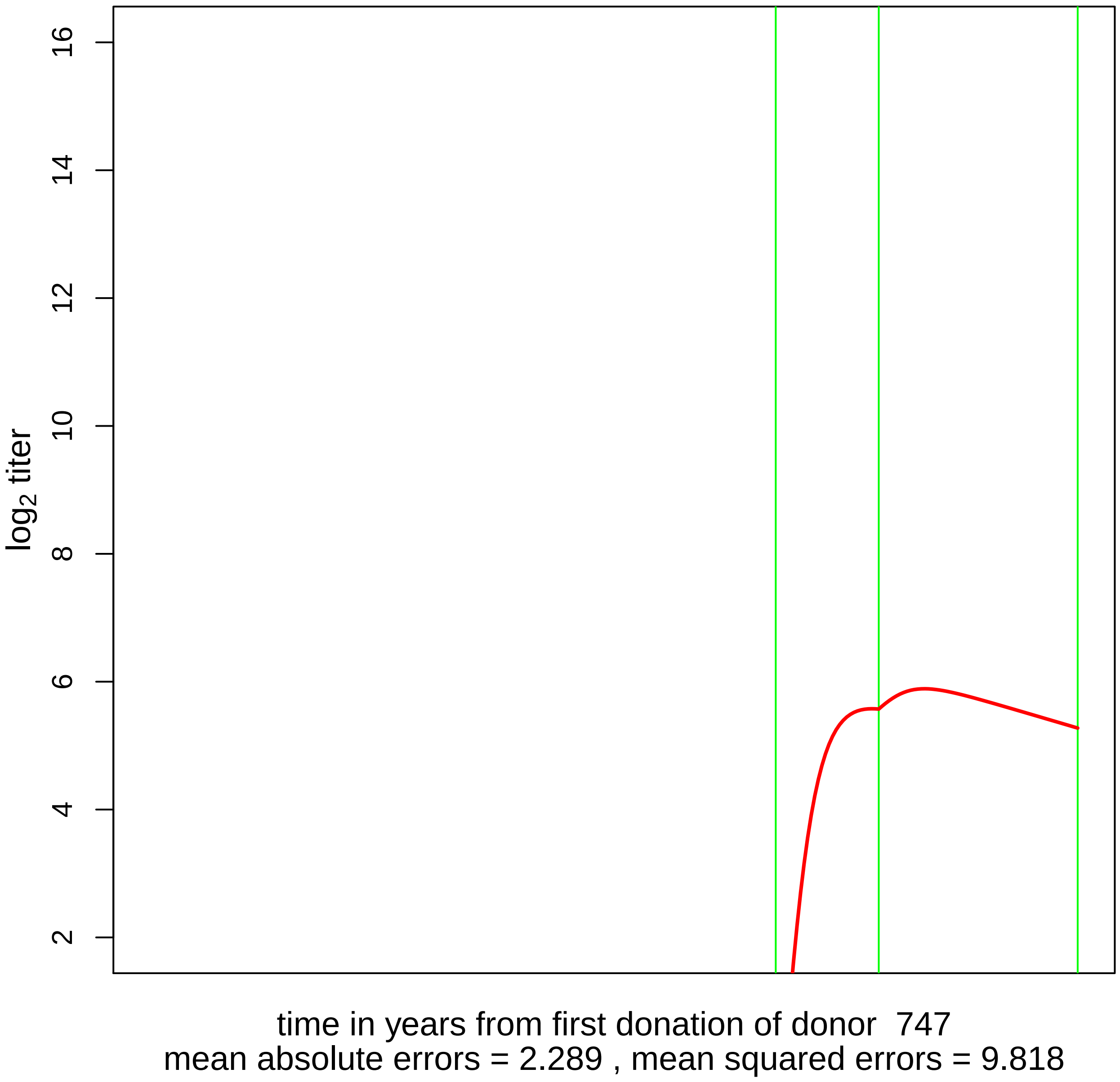

log2 titer
16
14
12
10
8
6
4
2
time in years from first donation of donor 747
mean absolute errors = 2.289 , mean squared errors = 9.818

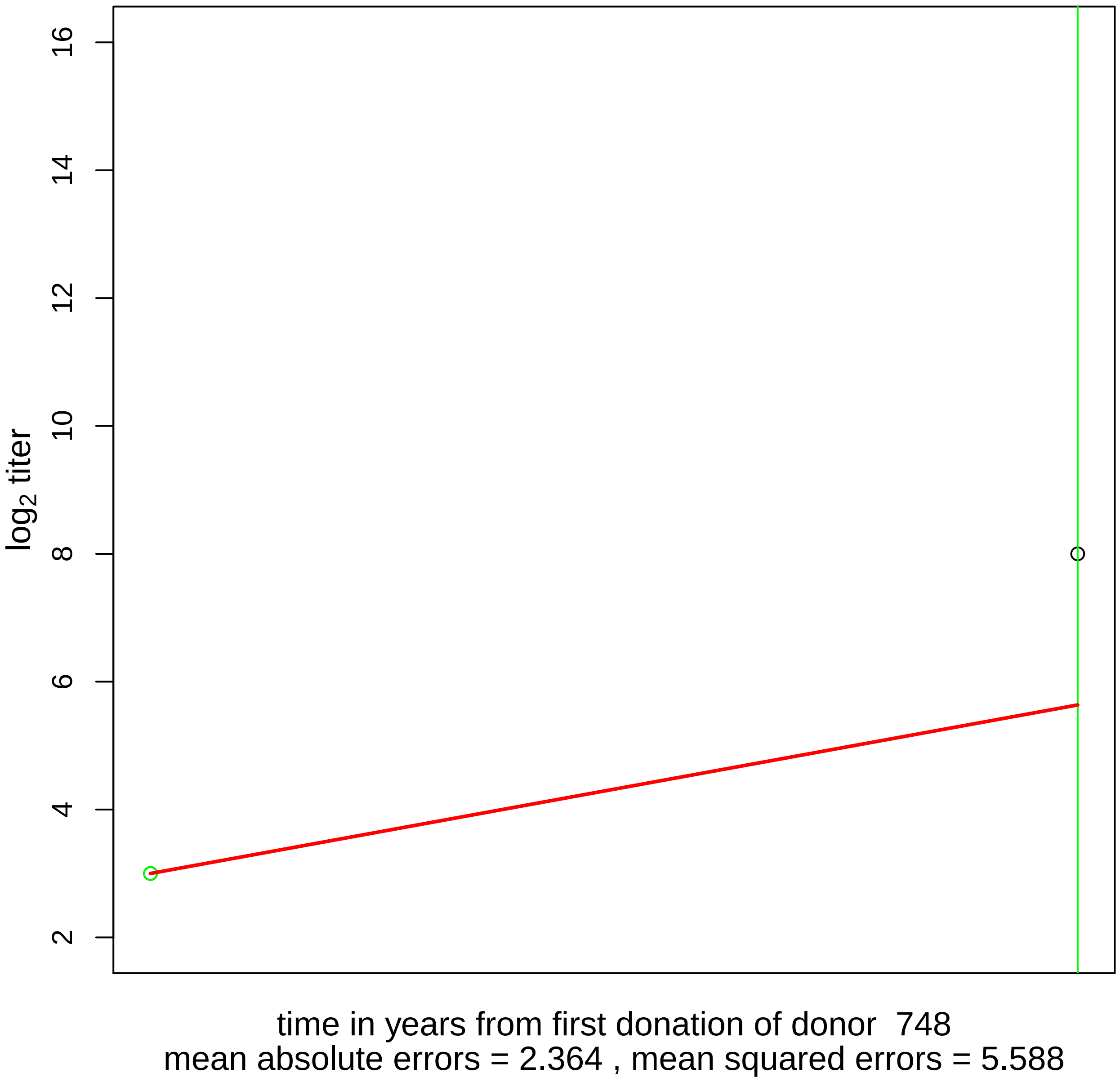
16
14
12
10
8
6
4
2
log2 titer
time in years from first donation of donor 748
mean absolute errors = 2.364 , mean squared errors = 5.588

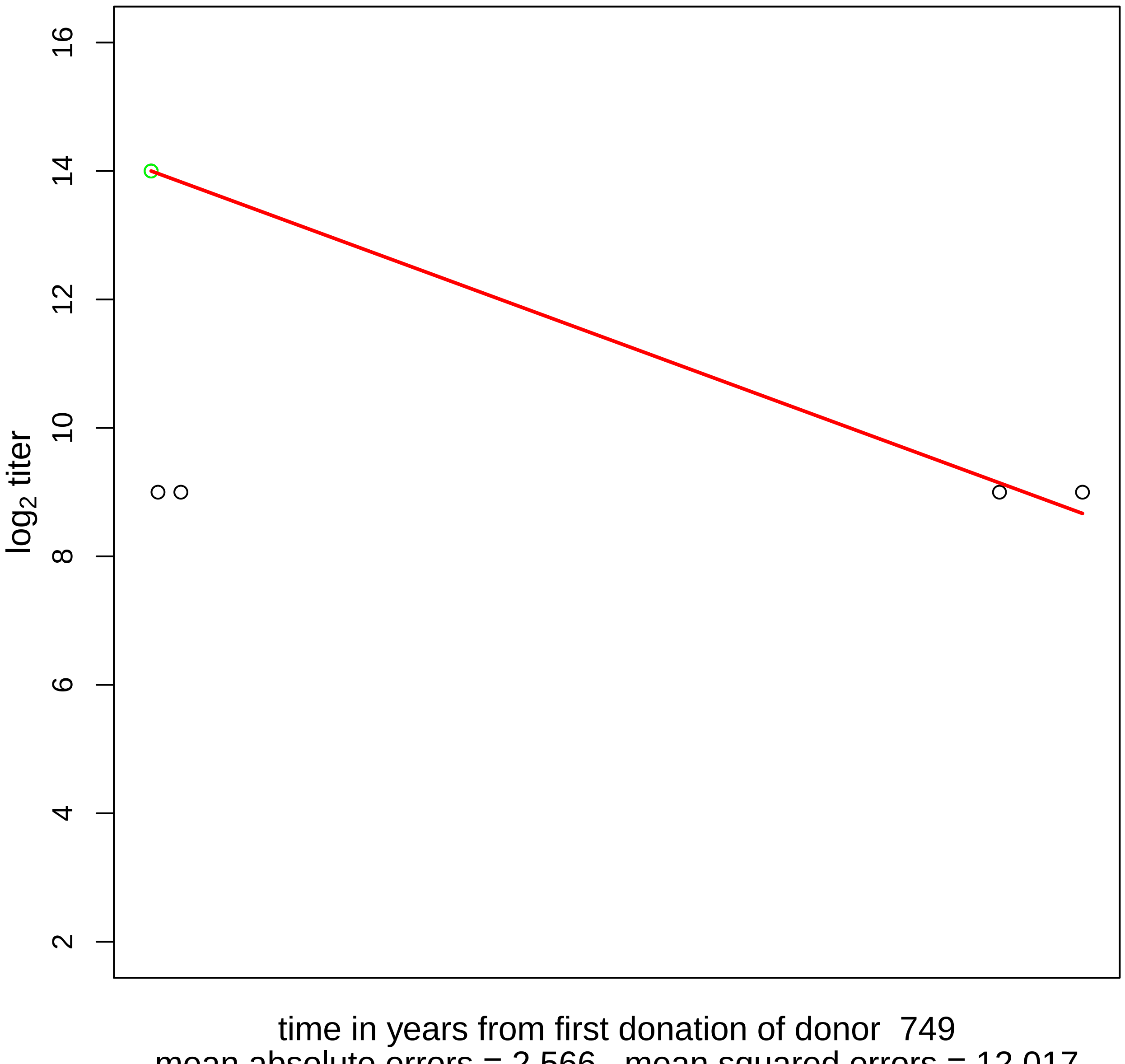

16
14
12
10
8
6
4
2
$\log_2$ titer
time in years from first donation of donor 749
mean absolute errors = 2.566 , mean squared errors = 12.017

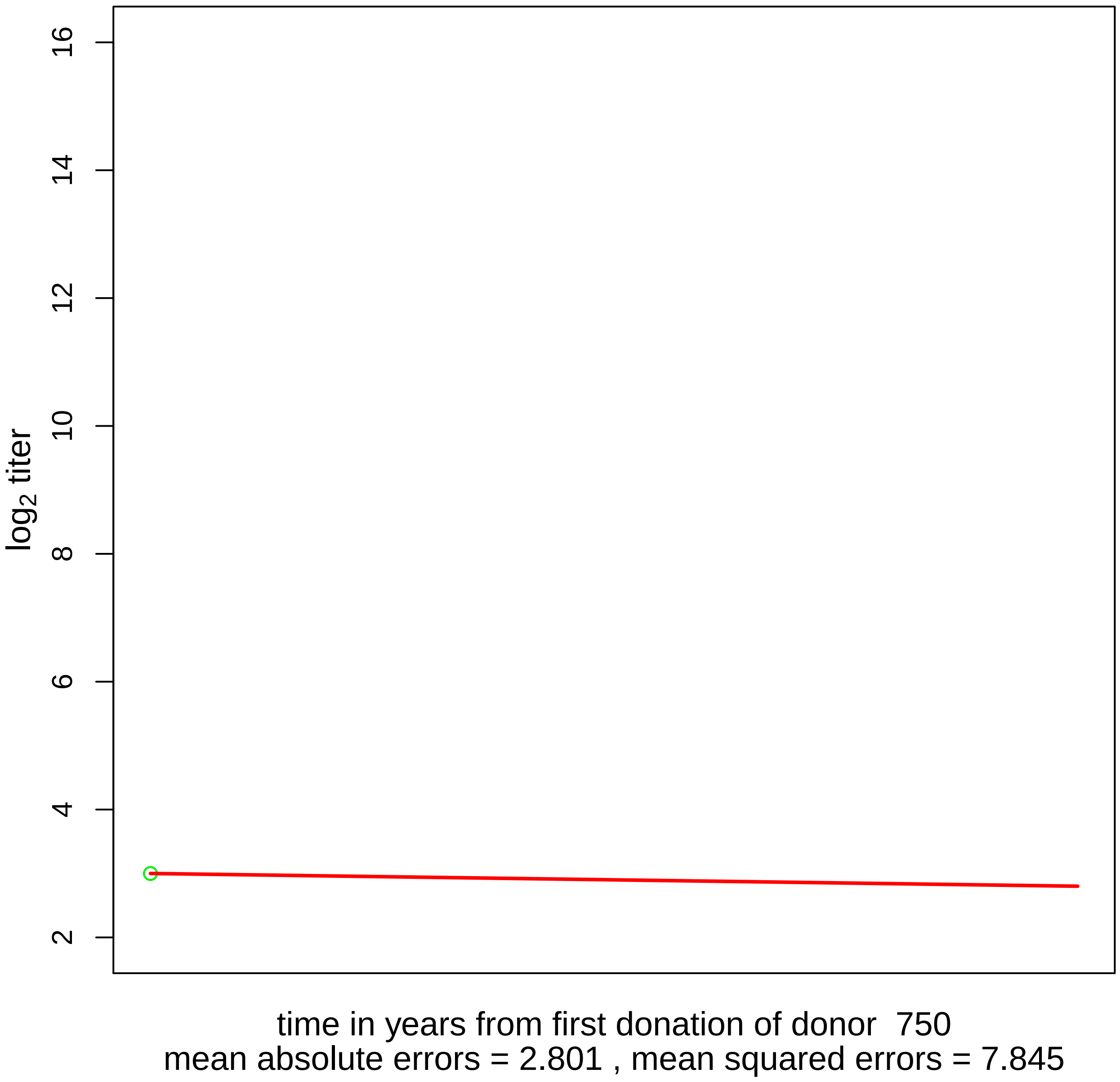

log2 titer
16
14
12
10
8
6
4
2
time in years from first donation of donor 750
mean absolute errors = 2.801 , mean squared errors = 7.845

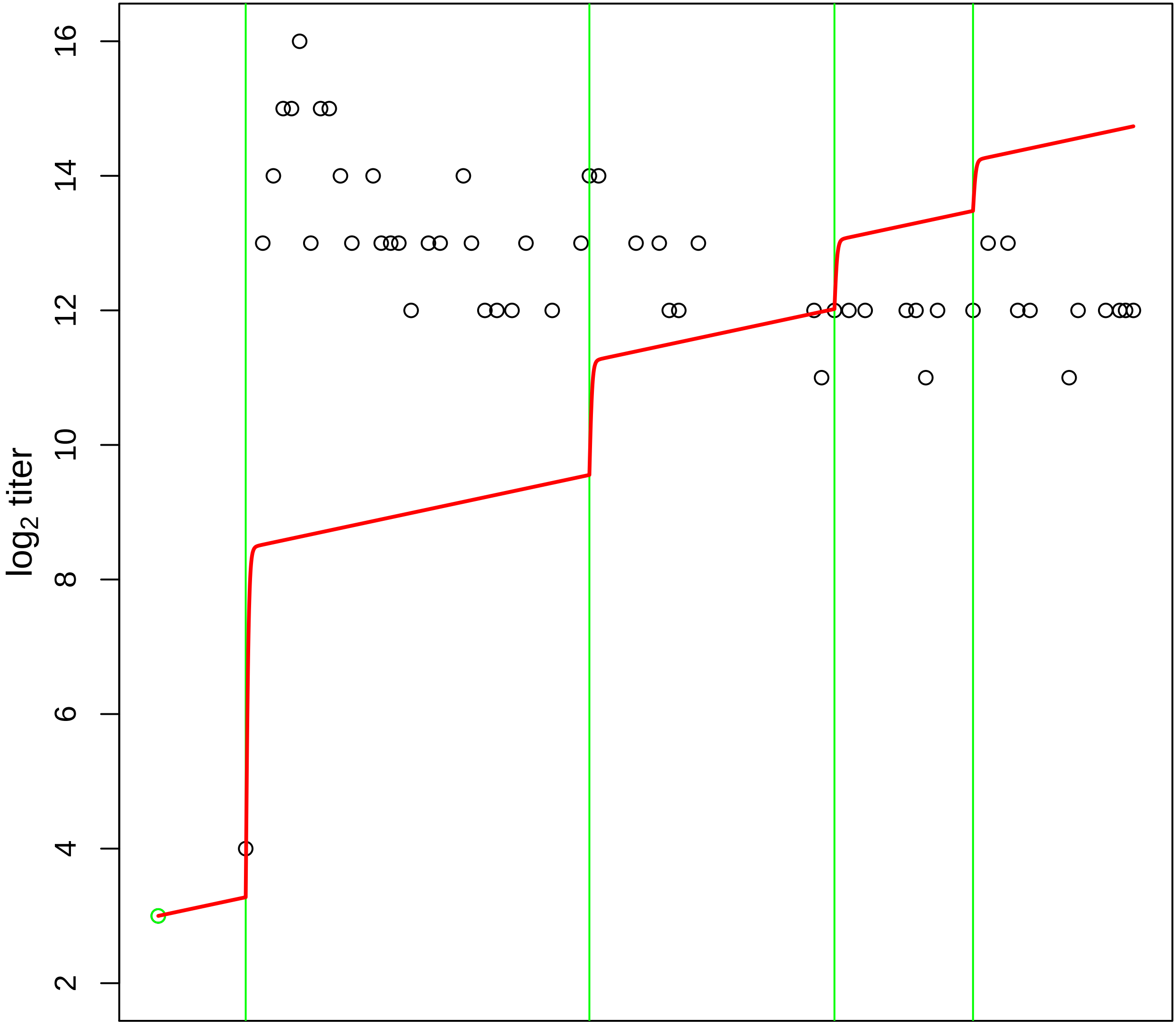

time in years from first donation of donor 751
mean absolute errors = 3.018 , mean squared errors = 12.302

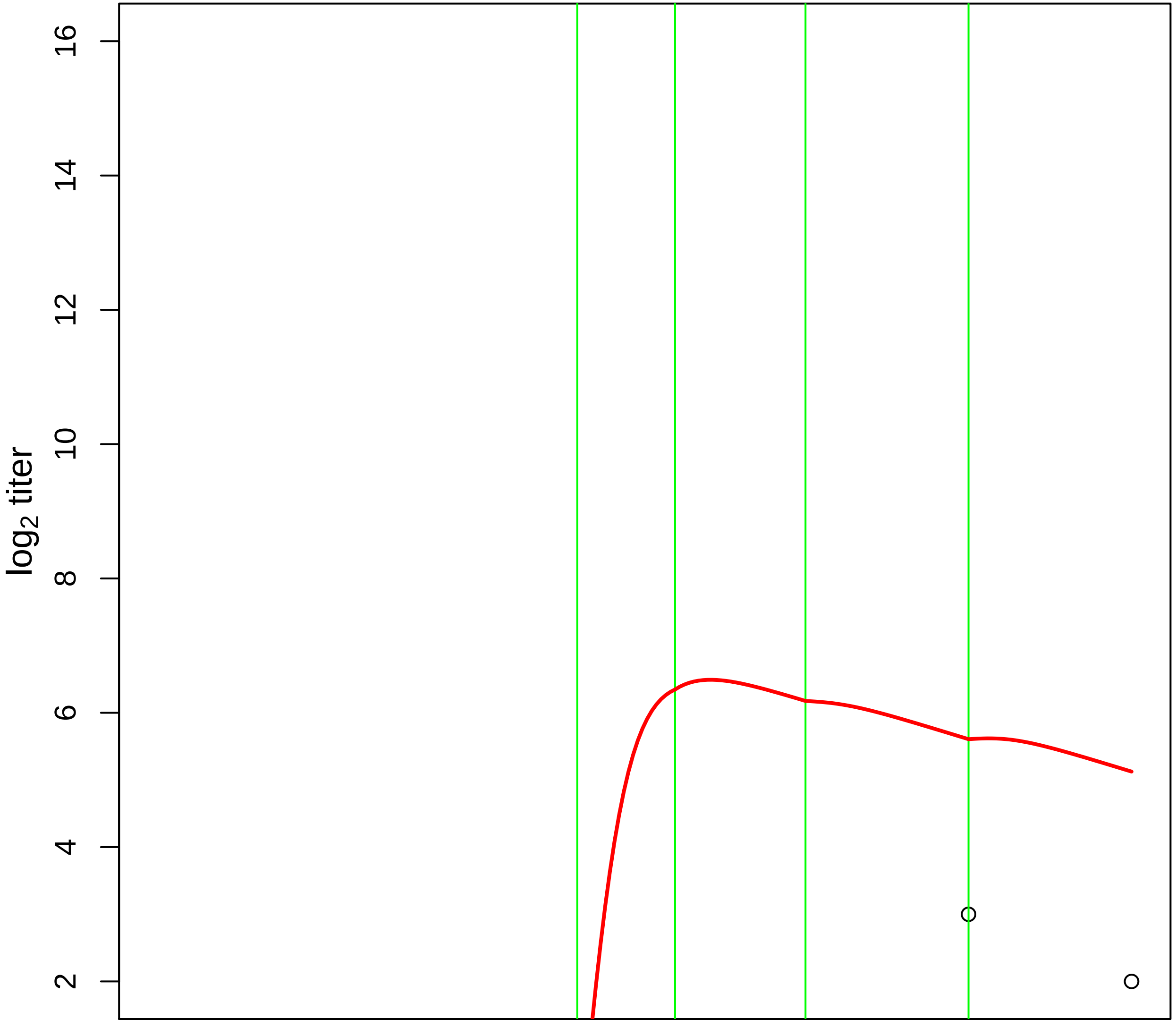

time in years from first donation of donor 752
mean absolute errors = 3.103 , mean squared errors = 12.637

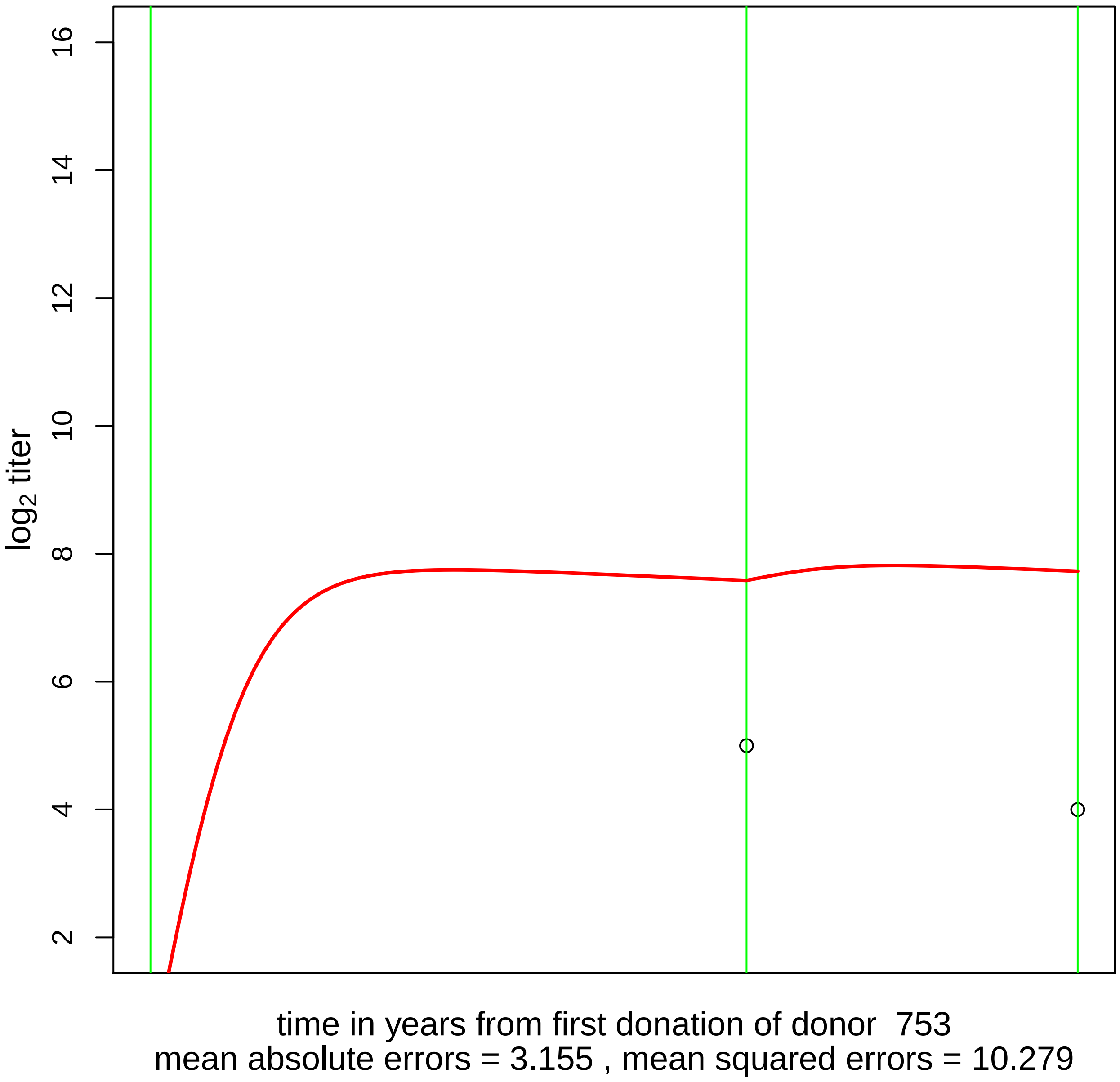

log2 titer
2
4
6
8
10
12
14
16
time in years from first donation of donor 753
mean absolute errors = 3.155 , mean squared errors = 10.279

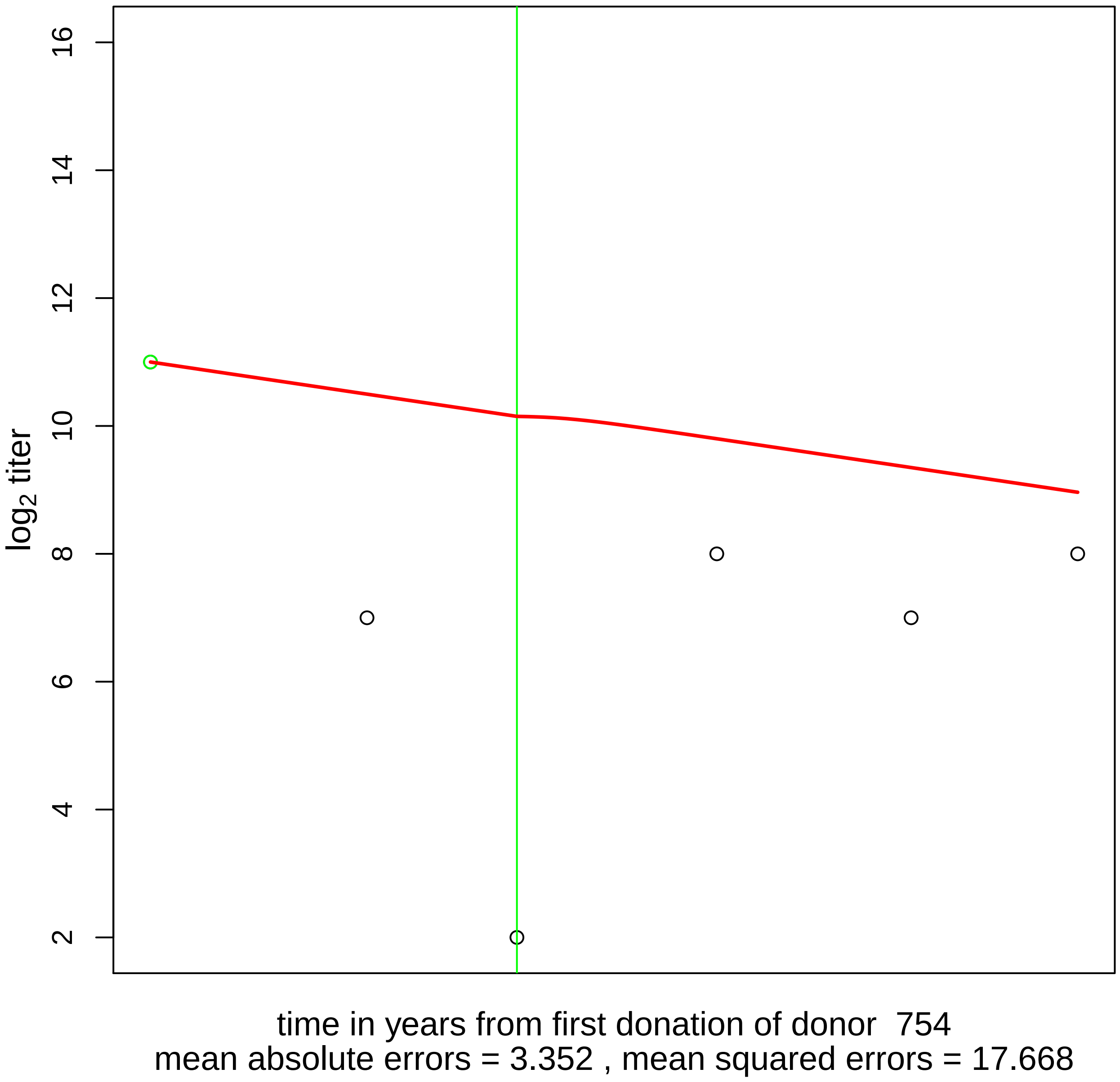

log2 titer
16
14
12
10
8
6
4
2
time in years from first donation of donor 754
mean absolute errors = 3.352 , mean squared errors = 17.668

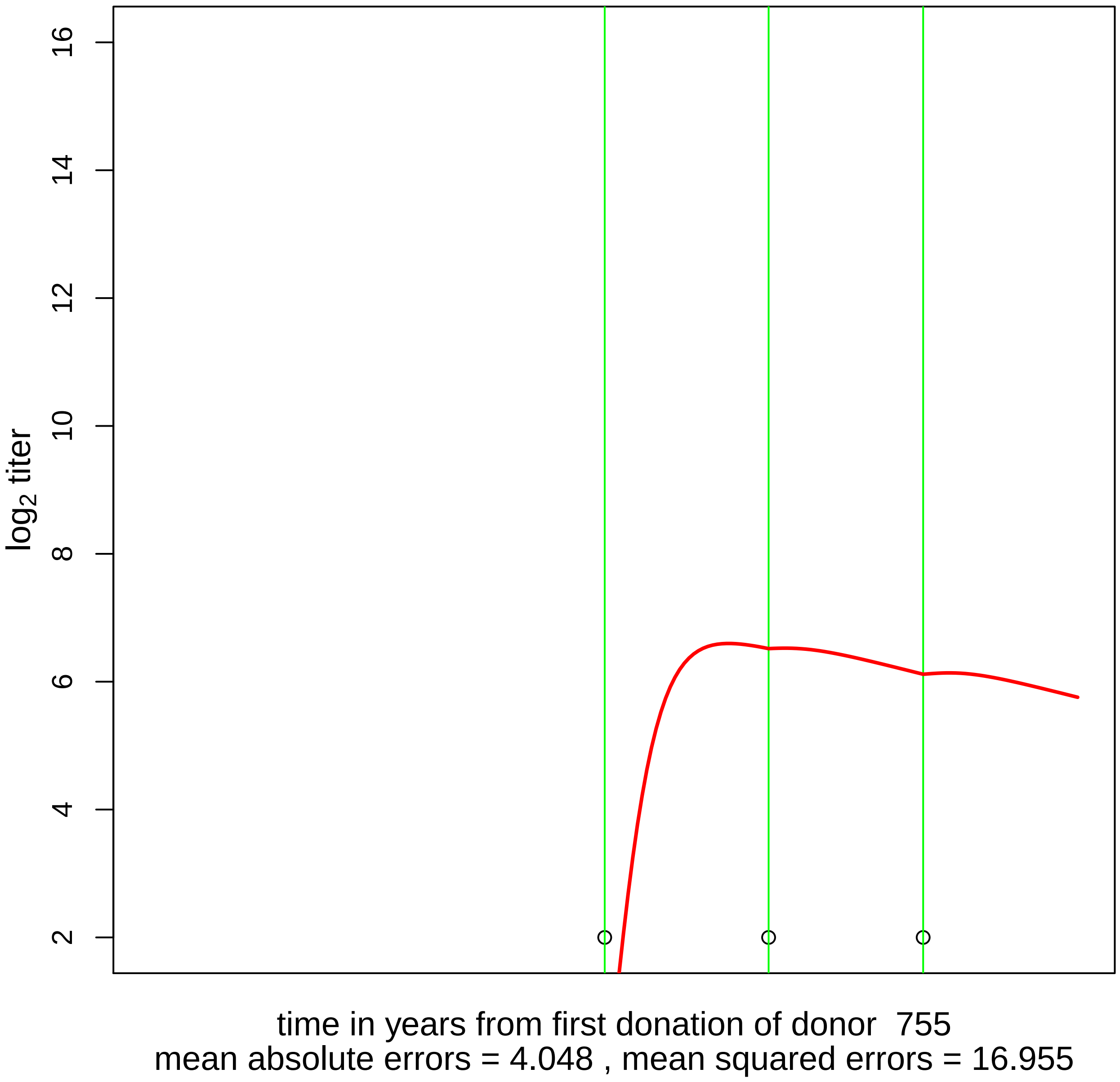

log2 titer
16
14
12
10
8
6
4
2
time in years from first donation of donor 755
mean absolute errors = 4.048 , mean squared errors = 16.955
